# Supplementary material for: Bilingual translations of intensifiers in Dong-A Ilbo’s news about China: A corpus-based discourse analysis approach
Source: PLoS One. 2024 Feb 6;19(2):e0292603. doi: 10.1371/journal.pone.0292603 (PMC10846699; doi:10.1371/journal.pone.0292603)
Supplement: S1 File — (ZIP) [file pone.0292603.s001.zip › corpus data/subcorpus of korean source text.docx]

# 美의 中반도체 규제후 중국내 韓점유율 5.5%P↓

미국의 대중국 반도체 규제 이후 중국 수입 반도체 시장에서 한국산이 차지하는 비중이 줄어든 것으로 나타났다.

　전국경제인연합회는 2018년 대비 2021년 중국 수입 반도체 시장의 국가별 점유율 변화를 분석한 결과 한국은 5.5%포인트가 낮아졌다고 25일 밝혔다. 제재 당사자인 미국(0.3%포인트 하락)보다 더 영향을 받은 것이다. 대만(4.4%포인트), 일본(1.8%포인트), 아세안 6개국(0.4%포인트)의 점유율은 미국의 규제 전보다 더 증가했다.

　미국 상무부는 2019년 4월∼2020년 9월 네 차례에 걸쳐 중국 화웨이, SMIC를 상대로 미국의 반도체 소프트웨어·장비를 활용해 생산한 반도체의 공급을 거래제한명단에 올리는 등의 방식으로 규제했다.

　미국 제재 전인 2018년과 비교했을 때 지난해 중국의 전체 반도체 수입 규모는 37.2%나 늘었다. 한국산 수입도 6.5% 늘긴 했으나 치열하게 경쟁 중인 대만산(57.4%), 일본산(34.8%) 등과 비교했을 땐 증가 폭이 작다.

　중국의 한국 반도체 수입 비중이 줄어든 결정적 원인은 미국 규제의 영향으로 한국 기업들의 화웨이 공급이 중단됐기 때문이다. 제재가 시작된 2019년 기준 삼성전자와 SK하이닉스의 전체 매출에서 화웨이가 차지하는 비중은 각각 3.2%(약 7조3700억 원)와 11.4%(약 3조 원)였다.

# 中, 우크라전쟁속 핵무장 박차…사막에 ICBM 격납고

중국 지도부가 미국에 맞선다는 명분으로 핵무장에 박차를 가하고 있다고 미국 월스트리트저널(WSJ)이 보도했다. 우크라이나 전쟁에서 나온 블라디미르 푸틴 러시아 대통령의 핵 위협이 효과를 발휘했다는 판단을 한 것으로 풀이된다.

　9일 WSJ는 중국 지도부 사정을 잘 아는 소식통을 인용해 “중국은 우크라이나 전쟁 전부터 핵전력 증강을 추진 중이었고 이번 전쟁을 통해 확신을 얻게 됐다”면서 “미국이 전쟁에 직접 개입을 자제하는 이유에 대해 러시아가 보유한 핵무기 때문이라는 결론을 내린 것으로 보인다”고 전했다. 이어 “중국은 대만과 군사적 충돌이 발생했을 때 미국이 핵무기를 사용할 수 있다는 우려를 내세워 이에 맞대응하기 위한 핵무기를 늘리고 있다”고 덧붙였다. 중국과 대만 간 충돌이 발생할 경우 우크라이나 전쟁에서처럼 미국의 직접 개입을 막기 위해 핵무기를 증강하고 있다는 것이다.

　위성사진을 분석한 전문가들에 따르면 중국은 서부 사막 지역인 간쑤성 위먼(玉門) 인근에 있는 신형 대륙간탄도미사일(ICBM) 둥펑(DF)-41 격납고 의심 시설 100여 곳 건설의 마무리 작업을 서두르고 있다. 1월 촬영된 위성사진에서는 격납고를 가리고 있던 임시 장막이 모두 제거됐다. 이는 정보 노출이 우려되는 민감한 작업이 끝났다는 것을 의미한다. 핵탄두 탑재가 가능한 둥펑-41의 최대 사거리는 미국 본토 전역을 타깃으로 할 수 있는 1만5000km인 것으로 알려졌다. 중국 당국은 이 격납고에 대해 함구하고 있다.

　미국 전문가들은 현재 중국이 핵탄두 수백 개를 보유했을 것으로 추정하고 있다. 2020년대 말 1000여 개까지 늘어날 것이라는 관측이 나온다.

봉쇄 장기화에 흉흉한 상하이… 항구 폐쇄설도 번져중국 경제 수도 상하이의 신종 코로나바이러스 감염증(코로나19)으로 인한 봉쇄가 길어지면서 시민들의 공포가 커지고 있다. 상하이 항구의 선적·하역 대기 선박도 봉쇄 이후 300척 이상 급증해 물류 장애가 더 심해질 것이라고 CNN이 보도했다. 상하이 봉쇄가 장기화하면서 지난달 28일 생산을 중단한 전기차 업체 테슬라의 공장 폐쇄도 계속될 것으로 보인다. 2019년 말 가동을 시작한 이래 최장기간 중단이다.

　4일 중국 국가위생건강위원회에 따르면 전날 하루 중국 전역의 코로나19 확진자는 1만3137명으로 역대 최다인 2020년 2월 12일 1만5152명에 근접했다. 이날 상하이 신규 확진자는 9006명이었다. 당초 시 당국이 밝힌 봉쇄 시한은 4일까지였지만 봉쇄는 계속되고 있다.

　상하이 시민의 불안 심리가 커지면서 관련 소문도 확산되고 있다. 3일 웨이보(중국판 트위터)에서는 상하이 한 병원에서 어린아이가 코로나19 치료를 못 받아 숨졌다는 소식과 동영상이 빠르게 퍼졌다. 이 동영상에서는 병상에 누운 아이에게 의료인이 다가가는 모습이 보이고 화면 밖에서 부모인 듯한 사람들의 고함이 들린다.

　상하이시 위생건강위원회는 이날 밤 이례적으로 성명을 발표하고 “해당 영상은 상하이 푸단대병원에서 고열로 의식을 잃은 아이를 응급처치 하려던 장면”이라고 공식 해명했다. 그러면서 “아이는 의식을 회복했고 부모도 의료진에게 사과했다”고 전했다. 상하이에 채소 등을 공급하는 업체가 채소를 쌓아뒀다가 폐기했다거나, 세계 최대 물류항인 상하이항이 곧 폐쇄된다는 소문도 퍼지고 있다. 이에 대해 상하이시 당국은 사실이 아니라고 해명하고 있지만 정부에 대한 불신이 누적된 시민들은 믿지 않는 분위기다. 상하이시는 “봉쇄는 없다”고 공식 발표한 다음 날인 지난달 28일 전격적으로 봉쇄를 시작하는 등 이미 시민들의 신뢰를 잃은 상태다.

# 北 도발에 대응할 열쇠 쥔 中, 두둔과 감싸기 더는 안 된다

윤석열 대통령 당선인이 어제 시진핑(習近平) 중국 국가주석과 첫 전화 통화를 했다. 북한의 ‘괴물 ICBM’ 발사 다음날 이뤄진 통화다. 윤 당선인은 한반도 안보 상황과 북한의 도발에 대한 대응방안을 놓고 시 주석과 협의했다. 북한은 이날 ICBM이 ‘화성-17’형임을 확인하며 ‘믿음직한 핵전쟁 억제수단’으로 완성됐다고 주장했다.

　　‘괴물 ICBM’ 발사로 레드라인을 깬 북한은 핵 위협 수위를 노골적으로 높이고 있다. 김정은 북한 국무위원장은 “용감히 쏘라”고 적은 친필 명령서를 하달했고, 평양 순안비행장을 찾아 발사 과정을 지켜봤다. 북한 매체는 이를 보도하면서 ‘핵’ 단어를 13번 사용했다. 핵실험을 비롯한 추가 도발을 언제라도 이어나갈 태세다.

　북한의 망동을 막아야 할 중국의 대응은 실망스러웠던 게 사실이다. 중국은 그제 북한의 ICBM 발사에 “유관 각국이 대화, 협상의 올바른 방향을 견지하기 바란다”고 했다. 규탄은커녕 주변국에 책임을 떠넘기는 듯한 뉘앙스다. 중국은 유엔 안보리에서 마땅히 내야 할 목소리도 내지 않고 있다. 중국이 신냉전 기류에 편승해 북한의 도발 여지를 열어줬다는 비판을 피하기 어렵다.

　중국이 대북 문제를 놓고 당장 윤 당선인과 협력할 가능성은 크지 않다. 중국은 ‘당당한 외교’를 천명한 윤 당선인의 대중 정책에 불편한 기색을 내비쳐 왔다. 그렇다고 중국이 북한의 핵 도발까지 마냥 방조하고 있을 여유는 없다. 북한의 핵기술 고도화는 3기 집권을 앞둔 시 주석에게 정치적 부담이자 안보 위협이다. 주변국의 핵 확산을 부추겨 지역 불안정을 가속화하는 요인이다.

　중국은 지금이라도 북한이 도발을 중단하고 대화에 나서도록 압박에 나서야 한다. 우선 국제사회의 대북 제재에 어깃장만 놓는 태도부터 바꿔야 한다. 유엔 안보리 결의 2397호는 북한의 ICBM 발사시 자동으로 추가 제재를 부과하는 ‘트리거 조항’을 담고 있지만, 이 또한 결의안이 통과돼야 가능하다. 중국의 동참은 국제사회의 일원으로서 마땅히 져야 할 의무다. 새 정부 또한 출범 직후 중국과 조속한 실무 협의를 할 수 있도록 준비해야 한다. 북 핵 저지는 한중 양국이 국익이 걸린 공통분모이자 향후 관계를 좌우할 핵심 현안이다.

# 조선 개항 이끈 중국 팽창주의 속내는

1882년 6월 조선 군사들이 궁궐에 난입한 직후 일본과 청은 즉각 출병에 나선다. 일본이 군함과 300명의 병력을 제물포로 보낸 데 이어 청이 광둥(廣東) 주둔군을 중심으로 3000명의 병력을 남양만에 상륙시킨다. 일촉즉발의 위기에 양국 간 교섭이 시작되고, 청군은 군란의 배후에 있던 대원군을 톈진(天津)으로 납치하기에 이른다.

　미국 캘리포니아 데이비스대(UC데이비스) 교수로 동아시아 근대사를 연구한 저자는 이 책에서 19세기 후반 한중일 3국 간 국제관계가 근대 세계질서로 편입되는 과정을 추적했다. 저자는 특히 중국 중심의 조공 체제가 와해된 역사적 사건으로 임오군란을 바라보고 있다. 당시 청은 군란을 진압한 후에도 한반도에 군대를 계속 주둔시키며 조선 내정에 깊이 간여했다. 이는 유교 질서에 따라 종주국으로서 의례적 권한만 행사할 뿐, 조공국 내정에 간섭하지 않는 조공 체제 전통과 어긋나는 행태였다.

　중국이 이처럼 팽창주의로 기운 건 당시 일본, 러시아의 동아시아 침투가 직접적인 원인이었다. 북중국은 물론 수도 베이징과도 멀지 않은 한반도는 자국 안보에 있어 핵심 완충국이었다는 것이다.

　흥미로운 건 아편전쟁 이후 서구 열강과 굴욕적 외교 조약을 맺은 청이 조선에 열강과의 조약을 통한 개항을 요구한 점이다. 이는 서구 열강들을 끌어들여 일본, 러시아를 견제하려는 이른바 변형된 이이제이(以夷制夷) 전략이었다. 그런데 이것은 조선이 대등한 주권국 간의 외교 행위를 근간으로 하는 ‘근대 세계질서’에 편입되는 걸 의미했다. 다시 말해 이 책 제목이 암시하듯 종주국을 정점으로 한 동아시아 세계질서가 사라지는 종막(終幕)이었던 셈이다.

　최근 베이징 겨울올림픽 판정 논란으로 반중 정서가 팽배한 가운데 중국을 어떻게 볼 것이냐가 화두가 되고 있다. 6·25전쟁의 분수령이 된 마오쩌둥의 참전 결정 이전에 19세기 청의 팽창주의가 한반도를 둘러싼 동아시아 세계질서를 바꾼 한 축이었음은 의미하는 바가 적지 않다.

# 1억명 하루 식수를 인공눈으로…베이징 ‘反환경 올림픽’ 논란

중국이 2022년 베이징 겨울올림픽의 모든 설상 경기를 인공 눈으로 치르기로 하면서 환경에 악영향을 미칠 수 있다는 우려가 제기되고 있다. 이번 대회 인공 눈의 양은 1억 명이 하루에 마시는 물의 양과 맞먹어 경기장 인근 주민들의 물 부족 사태가 벌어질 것이란 전망도 나온다. 중국은 베이징 올림픽 개회식에서 작은 성화를 선보이며 ‘친환경 올림픽’이라고 강조했지만 이와 대조되는 현상이 벌어지고 있는 것이다.

　미국 CNN은 이번 대회가 겨울올림픽을 열기에 적합하지 않은 기후에서 진행되고 있고, 지구온난화로 인한 기온 상승까지 겹쳐 인공 눈 제조에 더 많은 전력과 물이 소모될 것이라고 5일 보도했다. 국제올림픽위원회(IOC)에 따르면 이번 올림픽에서 인공눈을 만들기 위해 약 4900만 갤런(약 1억8548억 L)이 소모될 것으로 예측된다. 이는 약 1억 명이 하루에 마시는 물의 양과 비슷하다.

　야외 종목이 열리는 지역 중 상당수가 올겨울 극심한 가뭄 탓에 강설량이 부족해 인공눈에 대한 의존도가 커졌다는 분석도 나온다. 야외 종목이 진행되는 장자커우(張家口) 지역은 평소에도 연평균 강설량이 200mm에 불과하다. 1인당 사용 가능한 물의 양이 중국 전체 평균의 5분의 1도 안 되는 건조한 지역이다. 앞서 블룸버그는 이 지역의 스키장을 채우기 위해 200m³의 물이 필요하지만 53m³밖에 확보되지 못했다고 보도하기도 했다.

　중국은 ‘친환경 올림픽’을 실현했다며 자화자찬하는 분위기다. 4일 개회식에서 올림픽 사상 가장 작은 성화를 선보인 장이머우 총감독은 “연료가 대량으로 쓰이는 대형 성화 대신 중국 정부의 환경친화적 아이디어를 전달한 것”이라고 했다.

# 안보리 무력화한 中, 지금 北 고삐 안 잡으면 되레 당할 것

북한의 중거리탄도미사일(IRBM) 도발에 대응하기 위한 유엔 안정보장이사회 회의가 4일 열렸지만 어떤 결과물로 내놓지 못한 채 종료됐다. 북한의 잇단 미사일 도발에 올해 들어 세 번째 열린 안보리 회의였지만 이번에도 안보리 이사국 과반의 공동대응 요구에 거부권을 쥔 중국과 러시아의 반대에 부딪쳤기 때문이다. 중국 측은 북한의 도발을 규탄하기는커녕 오히려 미국을 향해 “북한의 우려사항을 수용하는 정책과 행동을 보이라”고 주장했다.

　이번 안보리 회의에선 사거리 5000km의 IRBM 도발을 논의한 만큼 이전 두 차례 단거리미사일 때와는 다를 것이라는 기대도 없지 않았다. 2017년 북한의 무더기 핵·미사일 도발 때 안보리는 중거리급 도발에도 북한 기관과 단체를 대북제재 명단에 추가하는 등 적극 대응했다. 하지만 북한이 다시 4년여 만에 가장 높은 수위의 도발을 벌였는데도 안보리 차원의 성명 한 장도 나오지 않았다. 중국 측이 이번엔 언론성명 초안을 본국에 보내 검토한다지만, 이 역시 흐지부지될 가능성이 적지 않다.

　안보리 대응이 무산된 뒤 미국을 포함한 9개국은 공동성명을 내고 “안보리의 침묵은 북한을 더욱 대담하게 만들어 결의 위반을 당연시하고 국제평화를 계속 위협할 것”이라고 했다. 안보리 이사국이 아닌 일본도 참여했지만 한국은 빠진 이 성명은 한낱 우려에 그치지 않는다. 북한은 벌써 핵실험과 대륙간탄도미사일(ICBM) 도발까지 협박하고 있다. 이 모든 게 중국이 감싸주고, 러시아가 거들고, 한국이 뒷짐 지고 있기에 벌어지는 일이다.

　세계 평화와 안전의 보루라는 유엔 안보리가 무력화된 데는 미·중 간 패권경쟁과 미·러 간 군사대치 같은 국제적 대결 정세와도 무관치 않다. 북한도 이런 신(新)냉전 기류에 편승해 한껏 도발을 벌이고 있다. 하지만 지금 북한의 핵 질주에 제동을 걸지 못하면 결국 중국에 큰 골칫거리가 될 것이다. 시진핑 주석이 굵직한 국제행사를 열 때마다 북한이 핵·미사일 도발로 잔칫상에 재를 뿌렸던 때가 불과 몇 년 전이다.

# 베이징 올림픽 성화 14년만에 다시 타올라

14년 만에 중국 베이징에서 다시 올림픽의 불꽃이 타오른다. 4일 오후 9시 열리는 개회식을 시작으로 2022 베이징 겨울올림픽이 17일간의 열전에 돌입한다. 베이징은 올림픽 역사상 최초로 여름, 겨울 대회를 모두 개최하는 도시다.

　개회식이 열리는 중국 베이징 국가체육장은 2008년 베이징 여름올림픽 당시 개·폐회식, 육상, 남자축구 결승전 등을 치른 곳이다. 독특한 디자인으로 ‘냐오차오(鳥巢·새 둥지)’로도 불린다. 이번에는 개·폐회식 외에 따로 경기가 열리지는 않는다. 총감독 역시 2008년에도 개·폐회식 총감독을 했던 장이머우(張藝謀) 감독이 맡는다. 이번 개회식에는 약 100분간 3000여 명의 공연자가 출연한다. 이 중 95%가량은 10대다. 4시간 동안 열린 2008년 개회식 때는 1만5000여 명이 출연했다. 신종 코로나바이러스 감염증(코로나19) 확산과 추운 날씨 등을 감안해 규모를 줄인 것. 게다가 미국을 비롯한 일부 서방 국가가 외교적 보이콧 의사를 드러내면서 외빈도 줄었다. 개최국인 중국의 시진핑 국가주석, 러시아의 블라디미르 푸틴 대통령 등의 참석이 예정돼 있다.

　개회식의 꽃인 최종 점화자, 점화 방식 등은 비밀에 부쳐져 있다. 2008년에는 중국 체조 영웅 리닝(59)이 와이어를 달고 경기장 지붕 안쪽 벽을 타고 달려가 성화대에 불을 붙였다. 장 감독은 “창의적인 성화 점화 방법으로 사람들을 놀라게 하겠다”고 공언한 상태다. 성화 연료로 수소를 써온 만큼 친환경 점화 방식에 대한 기대의 목소리도 나온다. 한편 최종 점화자 후보로는 역대 겨울올림픽에서 6개의 메달을 따낸 쇼트트랙 대표 왕멍(37), 2008년 대회 3관왕을 차지했던 체조 대표 저우카이(34) 등이 거론된다.

# 中대륙 못 도는 성화…베이징 인근 사흘만 달려

2022 베이징 겨울올림픽 개회에 앞서 중국 전역을 돌아다녀야 할 성화(사진)가 신종 코로나바이러스 감염증(코로나19) 확산 우려로 한껏 움츠러들었다.

　지난해 10월 20일 중국 베이징에 도착한 올림픽 성화는 4일 현재 베이징 올림픽타워에 보관돼 타고 있다. 다음 달 2일부터 3일 동안 주자 1200명이 경기가 열리는 장소인 베이징 중심부부터 베이징 교외인 옌칭구와 허베이성 장자커우만을 달린다. 왕복 거리로 따져도 300km 안팎이다. 2008년 베이징 여름올림픽 당시에는 주자 2만1880명이 5개 대륙 세계 19개 도시를 포함해 13만7000km를 누볐다.

　최근 올림픽과 비교해도 봉송 규모가 크게 줄었다. 도쿄 여름올림픽에서 성화는 지난해 3월 25일 후쿠시마를 시작으로 121일간 1만 명의 주자와 함께 일본 열도 2000km를 돌아 도쿄로 돌아왔다. 2018 평창 겨울올림픽 당시에는 2017년 11월 1일부터 101일 동안 7500명의 주자가 17개 시도와 강원도 시군 전체를 돌아 2018km를 달렸다.

# 홍콩 反中매체 시티즌뉴스 폐간선언

홍콩의 반중(反中) 온라인 매체 시티즌뉴스가 2일 폐간했다. 날로 거세지는 당국의 언론 탄압으로 규모가 큰 언론사조차 최근 속속 폐간을 택하자 더 버티는 것이 불가능하다는 판단을 한 것으로 풀이된다. 지난해 6월 당시 홍콩 최대 일간지인 핑궈일보, 지난해 12월 29일 유명 온라인 매체 리창신문에 이어 약 반년 사이에 세 곳의 반중 언론이 문을 닫으면서 홍콩의 언론 자유가 사실상 사라졌다는 우려가 나온다.

　시티즌뉴스는 2일 페이스북에서 “위기의 시기에 배에 탄 모든 이의 안전을 우선 보장해야 한다. 무거운 마음으로 폐간을 발표한다”고 이유를 밝혔다. 저널리즘의 정신을 계승해 대중에 봉사하고 싶었지만 최근 2년간 홍콩 사회의 변화와 언론 환경의 악화로 시티즌뉴스라는 작은 배가 강한 바람과 파도에 부딪혔다고도 설명했다.

　설립자 겸 편집국장인 크리스 융은 3일 기자회견을 열고 “폐간 결정은 짧은 기간 안에 이뤄졌다. 우리가 위험에 노출될 수도 있다는 점을 배제할 수 없었다”고 밝혔다. 특히 결정적인 폐간 계기는 불과 4일 전 폐간한 리창신문의 선택이었다고도 했다. 핑궈일보와 리창신문은 모두 폐간 직전 전현직 간부가 줄줄이 체포되고 회사 자산까지 동결되자 폐간을 택했다. 구성원에게 이런 일을 겪게 할 수 없었다는 뜻으로 해석된다.

　시티즌뉴스는 2017년 1월 1일 창간한 직원 40명 규모의 소규모 온라인 매체다. 자유, 개방성, 다양성, 포용성 등을 추구하며 2019년 범죄인 인도법(송환법) 반대 및 2020년 홍콩 국가보안법 반대 시위에서 민주 진영의 목소리를 충실히 전했다.

# G2 암울한 새해 전망…中, 인도에 밀리고 美, 코로나에 치이고

세계 패권국 지위를 놓고 대립하고 있는 미국과 중국이 올해 나란히 힘든 한 해를 겪을 것이라고 주요 외신이 일제히 보도했다. 중국은 신종 코로나바이러스 감염증(코로나19)에 따른 강경 봉쇄 정책, 부동산 부실, 전력난 등의 여파로 경제 성장이 대폭 둔화될 위험이 있다고 영국 텔레그래프가 지난해 12월 31일 보도했다. 미국 역시 코로나19 확산세, 인플레이션, 공급망 위기와 물류대란, 국제사회에서의 지도력 저하 등의 문제에 직면했다. 집권 2년 차를 맞은 조 바이든 미 행정부 또한 ‘지뢰밭’에 직면했다고 미 정치매체 더힐이 1일 평가했다.

○　中 성장률, 46년 만에 美에 뒤질 듯

　일본 투자은행 노무라증권은 올해 중국 경제가 4.3% 성장할 것으로 전망했다. 미국 성장률 전망치는 이보다 0.3%포인트 높은 4.6%로 제시했다. 세계은행 통계에 따르면 미국이 중국보다 높은 성장률을 기록하는 것은 1976년 이후 46년 만에 처음이다.

　중국의 경제 성장률은 1991년부터 2018년까지 약 30년간 연 6% 이하로 내려간 적이 없다. 노무라증권은 확진자가 단 1명만 나와도 도시 전체를 봉쇄하는 중국 특유의 ‘제로(0) 코로나’ 정책이 장기화할수록 이에 따른 경제적 악영향이 클 것으로 내다봤다.

　텔레그래프는 ‘떠오르는 거인’ 인도는 중국보다 약 2배 높은 8.5% 성장이 예상된다며 인도 경제가 중국을 제치고 오랫동안 고성장을 구가할 것으로 내다봤다. 프랑스 악사자산운용 역시 ‘세계의 공장’으로 불리는 중국의 생산 능력이 심각한 타격을 받을 것으로 내다봤다. 중국은 한국의 최대 수출국일뿐 아니라 중국 현지에 생산 공장을 둔 한국 기업도 많기에 한국 경제 또한 악영향이 불가피할 것으로 보인다.

　대만을 둘러싼 미국과의 갈등 격화 또한 우려를 낳고 있다. 시진핑(習近平) 중국 국가주석은 12월 31일 관영 중국중앙(CC)TV 생중계로 발표한 신년사에서 “조국의 완전한 통일은 양안(중국과 대만) 동포의 공통된 염원”이라며 대만을 압박했다. 이날 그는 지난해 7월 열린 중국 공산당 100주년 기념식 사진들을 배경으로 놓고 신년사를 발표했다.

　중국 군용기 또한 새해 첫날인 1일 오전 8시경 대만 방공식별구역(ADIZ)에 진입했다. 이에 차이잉원(蔡英文) 대만 총통 또한 같은 날 페이스북 생중계 연설에서 “중국이 상황을 오판하지 말고 군사적 모험주의가 내부에서 확장되는 걸 막도록 일깨워줘야 한다”고 맞섰다.

○　집권 2년 차 바이든 ‘지뢰밭길’ 암울

　최근 코로나19 일일 신규 확진자가 연일 사상 최고치를 경신하고 있는 미국에서는 새해 첫날에도 항공 대란이 이어지는 등 교통, 행정 기능이 타격을 받고 있다. 항공편 추적 사이트 플라이트어웨어에 따르면 코로나19 감염으로 인한 조종사 부족, 폭설 등으로 1일 총 2655편의 운항이 취소됐다.

　더힐은 이날 바이든 대통령이 코로나19와 오미크론 변이 확산, 전염병 대유행이 의료 및 금융체계에 미치는 여파, 인플레이션 등을 해결해야 한다고 지적했다. 최근 그의 국정수행 지지율은 30∼40%대로 취임 후 최저 수준을 기록하고 있다. 집권 민주당의 선거전략가 조엘 페인은 “바이든은 코로나19 덕분에 대통령이 됐지만 이제는 코로나19 때문에 힘든 처지에 놓였다. 그의 운은 코로나19와 함께 간다”고 했다.

　국내외 위험 요인도 많다. 바이든 대통령은 지난해 1월 도널드 트럼프 당시 대통령의 지지자가 트럼프의 대선 패배에 불복하며 의회에 난입한 사건이 발생한 지 1년이 되는 6일 대국민 연설을 하겠다고 예고했다. 그러자 트럼프 전 대통령 또한 같은 날 ‘맞불 회견’을 열겠다고 밝혔다. 전현직 대통령의 충돌이 미국의 분열과 양극화를 심화시키고 있다는 우려가 높다.

　러시아의 우크라이나 침공 위협이 높아지고 있다는 지적이 나오는 가운데 바이든 대통령은 미 동부시간 2일 볼로디미르 젤렌스키 우크라이나 대통령과 통화해 우크라이나 영토 보전에 대한 의지를 재확인할 예정이라고 백악관이 밝혔다. 바이든 대통령은 지난해 12월 30일 블라디미르 푸틴 러시아 대통령에게 “러시아가 우크라이나를 침공하면 가혹하게 제재할 것”이라고 경고했다.

# 中, 한미 가까워질수록 보복할 것…한국이 치러야할 불가피한 대가”

“한국이 미국에 가까워질수록 중국은 한국에 보복할 것이다. 안타깝지만 이건 한국이 치러야 하는 피할 수 없는 대가다.”

　국제정치학계 분야의 대표적 석학인 존 미어샤이머 미국 시카고대 석좌교수(75·사진)는 최근 동아일보와의 신년 인터뷰에서 미중 갈등에 대해 “중국이 더 강력해질수록 한국의 안보 위협은 커질 것”이라며 “누가 (한국의) 대통령이 되든 한국과 미국이 어떤 관계를 맺을지가 가장 중요한 문제”라고 강조했다. 시진핑(習近平) 중국 국가주석의 3연임으로 장기집권 체제를 다진 중국이 미국을 넘어서는 패권국이 되려고 시도하는 과정에서 한국을 향한 압박이 더욱 거세질 것이라는 얘기다. 가장 영향력 있는 현실주의 이론가인 미어샤이머 교수는 미중 패권 경쟁을 가장 정확하게 예측한 인물로 꼽힌다.

　미어샤이머 교수는 “세계는 ‘2차 냉전’에 돌입하고 있다”며 “중국은 곧 미국과 동등한 힘을 갖게 되고, 앞으로 30년간 경제 성장을 이어간다면 미국을 제치고 가장 강력한 국가가 될 것”이라고 내다봤다. 그는 또 “미중이 15년 이내에 대만을 두고 전쟁을 벌일 가능성이 높다고 본다”고 전망했다.

　특히 미어샤이머 교수는 한국의 이른바 ‘안미경중(안보는 미국, 경제는 중국)’ 외교에 대해 “한국이 한미 동맹에 전념하지 않는 것은 ‘어리석음의 극치(height of foolish)’가 될 것”이라고 강조했다. 사드(THAAD·고고도미사일방어체계) 사태에서 볼 수 있듯이 중국의 위협이 커질수록 한국은 미국과 안보 협력을 통한 생존을 선택할 수밖에 없다는 것. 그는 “한국과 일본이 긴밀히 협력하면 중국의 위협에 더 잘 대응할 수 있을 것”이라고 조언했다.

# 정의용 “베이징올림픽 계기 남북관계 개선 어려워져”

정의용 외교부 장관이 “베이징 올림픽을 남북관계 개선의 계기로 삼길 희망했지만 현재로선 그런 기대가 사실상 어려워지고 있다“고 밝혔다.

　정 장관은 29일 서울 외교부 청사에서 열린 기자간담회에서 내년 2월 베이징 겨울올림픽에서 남북 정상회담 가능성 등을 묻는 질문에 이같이 말했다. 신종 코로나바이러스 감염증(코로나19) 여파로 북한의 올림픽 참석이 불투명한 가운데 미국도 외교적 보이콧을 선언한 상황에서 올림픽을 종전선언 등을 위한 발판으로 삼기 힘들다고 인정한 것. 다만 정부 고위관계자는 “정 장관의 발언은 ‘올림픽’을 평화 프로세스에 활용하기 어려워졌다는 취지”라며 “오히려 올림픽 전이라도 남북관계에서 큰 진전이 있을 수 있는 것”이라고 전했다.

　정 장관은 베이징 올림픽 외교적 보이콧 관련해선 “검토하지 않고 있다”며 기존 입장을 확인했다. 문재인 대통령의 참석 가능성이 열려 있느냐는 질문에는 “현 단계에서 공유할 내용이 없다”고 말을 아꼈다. 청와대 내부에선 현재로선 문 대통령의 올림픽 참석이 어렵지 않겠느냐는 기류가 강한 것으로 알려졌다.

　정 장관은 한미가 조율 중인 종전선언 문안에 대해선 “사실상 합의가 돼 있는 상태”라고 밝혔다. “조율이 거의 끝난 상태”라던 기존 입장보다 한발 나아간 것. 또 “북한과 (종전선언 관련) 협의를 어떻게 진전시켜야 할지 검토하고 있다”며 “북한의 구체적인 반응이 있기를 기대하고 있다”고도 했다.

　정 장관은 일본군 위안부 문제 관련해선 “원죄가 어디 있는지 여러분이 잘 아시지 않느냐”며 과거사 문제로 인한 한일 관계 경색에 대한 책임을 일본 정부에 돌렸다. 그러면서 “일본이 끝까지 우리가 2015년 (위안부) 합의를 그대로 지켜야 한다는 입장을 아주 완강하게 고수하고 있어 (한일 관계가) 전혀 진전을 이루지 못하고 있다”고도 했다. 최근 일본이 조선인 강제노역 현장인 사도(佐渡) 광산을 세계문화유산에 등재하려는 움직임과 관련해선 “깊이 우려하고 깊은 유감을 표한다”고 비판했다.

　정 장관은 우리 정부가 북한, 중국의 인권 문제에만 눈 감고 있다는 지적에 대해서는 “북한, 중국과는 특수한 관계에 있고 우리 안보와 직결돼 협력할 부분이 많기 때문”이라고 설명했다.

# 삼성전자 “中시안 공장, 코로나 확산에 탄력운영”

신종 코로나바이러스 감염증(코로나19) 확산으로 중국 산시성 시안(西安)이 사실상 봉쇄된 가운데 삼성전자의 현지 반도체 생산이 일부 차질을 겪고 있다.

　삼성전자는 29일 자사 뉴스룸을 통해 “중국 시안 반도체 사업장은 코로나19 확산세가 지속됨에 따라 생산라인의 탄력적 조정을 진행 중”이라고 밝혔다. 탄력적 조정은 반도체 생산이 평시보다 적은 수준으로 낮춰진다는 걸 뜻한다. 삼성전자는 “임직원의 안전과 건강을 최우선으로 고려해야 한다는 회사의 경영 방침에 따른 것”이라고 덧붙였다.

　산시성 성도이자 인구 1300만 명의 대도시인 시안은 이달 9∼22일 코로나19 신규 확진자가 206명 발생하면서 주민 외출이 전면 금지됐다. 중국 당국이 확진자가 1명이라도 생길 경우 해당 지역 전체를 봉쇄하고 주민 모두를 격리하는 정책을 시행하고 있기 때문이다. 삼성전자는 그동안 중국 정부의 특별조치를 받아 공장을 정상 가동해 왔다. 하지만 지역 봉쇄가 길어지면서 임직원 출퇴근은 물론 물류까지 차질이 빚어지자 생산량 조절에 나섰다.

　시안 반도체 공장은 삼성전자의 유일한 해외 메모리반도체 공장으로 2014년부터 낸드플래시를 생산하고 있다. 낸드플래시는 모바일 기기의 데이터 저장소뿐 아니라 서버, PC 등에서 데이터 저장에 필요한 솔리드스테이트드라이브(SSD) 제조에 쓰인다. 삼성전자의 낸드플래시 세계 시장 점유율은 40%가량인데, 삼성전자 전체 생산량 중 약 40%가 시안 공장에서 만들어지고 있다.

　한편 시안에 있는 삼성SDI의 전기차용 배터리 공장은 현재 정상 가동 중이다. 삼성SDI는 코로나19 확산 정도와 중국 정부 조치에 따라 향후 공장 운영 방향을 결정할 계획이다.

# 美 내년1분기 성장률 전망 5.2% → 2.2%

　신종 코로나바이러스 감염증(코로나19) 새 변이인 오미크론이 급속도로 퍼지면서 미국과 중국 등 세계 경제에 충격이 현실화될 조짐이다.

　27일 월스트리트저널(WSJ)에 따르면 각국의 경제 전문가들은 내년도 미국과 전 세계의 경제 전망을 하향 조정하고 있다. 무디스애널리틱스의 마크 잰디 수석이코노미스트는 내년 1분기(1∼3월) 미국의 국내총생산(GDP) 증가율을 기존의 5.2%(연율)에서 2.2%로 낮췄다. 그는 최근 항공대란에 따른 여행 감소와 스포츠 경기, 브로드웨이 공연 중단 등을 거론하며 “올여름 델타 변이 확산 때와 매우 비슷한 현상이 벌어지고 있다”고 진단했다. 경제 연구기관인 판테온 매크로이코노믹스도 내년 1분기 미국의 성장률 전망치를 종전의 5%에서 3%로 낮췄다.

　최근 세계은행(WB) 역시 중국의 내년 성장률 전망치를 5.4%에서 5.1%로 낮췄다. 오미크론 변이가 봉쇄 조치의 장기화로 이어지면서 경제 활동이 내년에도 계속 위축될 것으로 예상했다. 독일의 중앙은행인 분데스방크도 독일의 내년 성장률을 6월에 전망한 5.2%에서 4.2%로 하향 조정했다.

# 리그도 연기한 NHL, 베이징 겨울올림픽 안간다

2022 베이징 겨울올림픽 흥행에 ‘빨간불’이 켜졌다. 겨울올림픽의 꽃이자 최고 흥행 종목으로 꼽히는 아이스하키에 북미아이스하키리그(NHL) 선수들이 불참하기 때문이다.

　미국 스포츠전문매체 ESPN은 22일 NHL 노사가 베이징 올림픽에 참가하지 않기로 합의했다고 보도했다. NHL의 올림픽 참가의 발목을 잡은 것은 신종 코로나바이러스 감염증(코로나19)의 확산 때문이다.

　NHL은 코로나19 오미크론 변이 확산으로 2021∼2022시즌 정규시즌 50경기를 연기했다. 크리스마스 연휴에만 사흘간 쉬려고 하다가 이틀을 더해 22일부터 26일까지 리그를 중단하기로 했다. ESPN은 “NHL은 내년 1월 10일까지 올림픽 불참을 결정하면 벌금 등의 페널티를 피할 수 있다”며 “조만간 불참과 관련한 공식 발표를 할 예정”이라고 전했다.

　NHL은 1998년 나가노부터 2014년 소치까지 모든 올림픽에 나섰다. 하지만 2018년 평창 올림픽은 경제적으로 이득이 없고, 빠듯한 리그 일정 등을 이유로 불참했다. NHL은 13억 인구를 지닌 거대 중국 시장을 고려해 베이징 올림픽은 참가하겠다고 했지만 결국 두 올림픽을 건너뛰고 2026년 밀라노-코르티나담페초 올림픽에 참가하게 됐다.

　베이징 올림픽은 해외 관중을 받지 않고 자국민에게만 입장권을 판매하기로 했다. NHL 불참에 따른 티켓 판매 격감 등의 문제는 없겠지만 대회 권위 추락과 시청률 저하 등은 불가피할 것으로 전망된다.

### “화이자-모더나 이외 백신, 오미크론 예방효과 거의 없어”

신종 코로나바이러스 감염증(코로나19) 백신 중 메신저리보핵산(mRNA) 방식인 화이자와 모더나를 제외한 나머지 백신들은 오미크론 변이 감염 예방 효과가 거의 없다고 미국 뉴욕타임스(NYT)가 19일(현지 시간) 보도했다. mRNA 방식이 아닌 다른 백신에 의존해 온 저소득 국가들을 중심으로 계속 감염이 늘고 변이가 출현할 것이라는 우려가 나온다. 오미크론 변이가 코로나19 항체 치료제도 무력화한다는 연구 결과도 나왔다.

　NYT는 “화이자, 모더나 백신만이 오미크론 변이로부터의 감염을 막을 수 있다는 연구 결과가 속속 발표되고 있다”고 보도했다. 영국 보건안전청(HSA)은 백신 접종자들을 분석한 결과 “아스트라제네카 백신을 맞은 지 6개월이 지나면 오미크론 감염 예방 효과가 0%대로 떨어진다”고 12일 밝혔다. 1회 접종 방식인 얀센 백신은 아프리카에서 수요가 급증하고 있지만 “오미크론 감염 예방 효과는 거의 무시해도 될 정도”라고 NYT는 지적했다. 전 세계 백신 중 절반을 차지하는 중국 백신(시노팜, 시노백)에 대해선 “오미크론 감염을 거의 막지 못한다. 중국, 멕시코, 브라질 등이 주로 이 백신을 접종했다”고 전했다. 아프리카와 중남미 국가에서 주로 맞은 러시아의 스푸트니크 백신도 오미크론 예방 효과가 매우 낮다고 보도했다.

　미국 존스홉킨스대 블룸버그공중보건대학원의 톨버트 니엔스와 연구원은 “부유한 선진국들은 첨단기술이 사용된 mRNA 백신 기술을 공유하지 않았고, 저소득 국가들은 다른 백신에 의존해야 했다. 그 결과 이들 국가에서 변이가 계속 나타나 코로나19 대유행을 연장시킬 것”이라고 지적했다.

　20일 AP통신에 따르면 미국 제약사 리제네론과 일라이릴리는 미국 식품의약국(FDA)이 긴급사용을 승인해 미국 병원에서 가장 많이 사용 중인 자사의 항체 치료제가 오미크론에 듣지 않는 것으로 나타났다고 밝혔다. 두 회사의 항체 치료제는 코로나19 환자가 중증으로 치닫는 것을 막는 데 쓰이고 있다. 두 제약사는 새로운 항체 치료제를 신속하게 개발하겠다고 밝혔지만 AP통신은 “적어도 몇 달은 걸릴 것이다. 의료진은 새 위기에 봉착하게 될 것”이라고 했다.

# 文“베이징올림픽 외교 보이콧 검토 안해”

호주를 국빈 방문 중인 문재인 대통령이 13일 내년 2월 베이징 겨울올림픽 외교적 보이콧과 관련해 “한국 정부는 보이콧을 검토하고 있지 않다”고 밝혔다. 중국과 첨예하게 맞서고 있는 미국을 시작으로 호주 영국 등이 보이콧 행렬에 동참하고 있지만 문 대통령은 일단 선을 긋고 나선 것.

　문 대통령은 이날 호주 캔버라에서 스콧 모리슨 호주 총리와의 정상회담 뒤 가진 공동기자회견에서 “미국을 비롯한 어느 나라로부터도 (보이콧) 참가 권유를 받은 적이 없다”며 이같이 말했다. 이어 “경제적인 측면, 한반도의 평화와 안정, 북한의 비핵화를 위해 중국의 건설적인 노력이 요구된다”며 “한국은 미국과의 굳건한 동맹을 기반으로 삼으면서 중국과도 조화로운 관계를 유지해 나갈 수 있도록 노력해 나가고 있다”고 했다. 미중 모두 종전선언의 중요한 관련국인 만큼 전략적 모호성 기조를 이어가며 균형외교를 펼치겠다는 의미로 풀이된다.

　이를 두고 미국 블룸버그통신은 ‘한국이 베이징 올림픽 보이콧을 놓고 미국과 결별했다(Breaks With US)’고 보도하며 “대북 화해를 핵심 목표로 삼은 문 대통령이 남은 임기 내 진전을 이루려면 중국의 도움이 필요할 것”이라고 해석했다.

# 정부, 세계 무역 15% 차지 CPTPP 가입 본격 추진

정부가 세계 무역의 15%를 차지하는 ‘포괄적·점진적 환태평양경제동반자협정(CPTPP)’ 가입 추진을 공식화했다. 중국에 대한 무역 의존도를 낮추고 교역을 다변화할 수 있는 기대가 나오지만 농산물 수입이 늘어날 것을 우려하는 농수산업계의 반발도 예상된다.

　13일 홍남기 경제부총리 겸 기획재정부 장관은 대외경제관계장관회의에서 “교역과 투자 확대를 통한 경제적, 전략적 가치 등을 고려해 CPTPP 가입을 본격 추진한다”며 “다양한 이해 관계자 등과 사회적 논의를 바탕으로 절차를 개시한다”고 밝혔다. 정부가 2013년 CPTPP의 전신인 환태평양경제동반자협정(TPP)을 검토한 지 약 8년 만이다.

　CPTPP는 미국이 TPP를 탈퇴한 후 일본, 호주, 멕시코 등 11개국이 2018년 출범시킨 다자간 자유무역협정(FTA)이다. 일본이 의장국을 맡고 있고 올해 9월 중국과 대만이 가입을 신청했다.

　CPTPP의 관세 철폐율은 최대 96% 수준으로 시장 개방도가 높다. 세계 무역의 15%를 차지하는 CPTPP에 가입하면 중국에 대한 무역 의존도가 낮아지고 수출시장이 다변화될 것으로 전망된다.

　올해 1월 한국개발연구원(KDI)은 “CPTPP 가입은 중국 의존도를 낮추고 통상 지형을 확대하는 데 매우 효과적”이라고 밝혔다. 미중 무역갈등이 지속되는 상황에서 안정적인 글로벌 공급망에 편입될 수 있다는 점도 장점이다. 특히 한국과 FTA를 체결하지 않은 멕시코와 처음으로 FTA를 체결하는 효과도 기대할 수 있다.

　반면 농수산업계는 농산물 수입이 늘어날 것을 우려하고 있다. CPTPP 참여국 중 호주, 칠레, 캐나다 등 농업 강국이 많기 때문이다. 이날 한국종합농업단체협의회는 성명을 내고 “상대적으로 가격 경쟁력이 높은 수입 농산물의 증가는 장기적으로 농업 생산기반 붕괴로 이어질 수 있다”고 반발했다.

　실제 CPTPP에 가입하려면 공청회와 국회 보고 절차, 회원국과의 세부 협상이 필요해 2∼3년이 걸릴 것으로 보인다. 정부가 가입 검토를 시작한 지 8년 만에 공식 가입 절차에 들어가 ‘뒷북 가입’으로 협상력만 떨어졌다는 지적도 나온다. 최종 가입을 위해 일본 등 참여국의 만장일치 동의가 필요하기 때문이다. 최원목 이화여대 법학전문대학원 교수는 “중국보다 가입이 한발 늦어져 한국의 협상력이 떨어질 수밖에 없다”며 “주도국인 일본은 강제징용 피해자에 대한 일본 기업의 배상 판결 등을 협상에 활용할 수 있다”고 분석했다.

# 美 “베이징올림픽 외교적 보이콧”…中 “결연히 반격”

미국이 내년 2월 열리는 베이징 겨울올림픽에 대한 외교적 보이콧(diplomatic boycott)을 6일(현지 시간) 선언했다. 중국 당국의 신장(新疆)위구르 지역에 대한 인권탄압을 ‘제노사이드’(집단 학살)로 규정하면서 외교제재에 나선 것이다. 중국의 공개적인 보복 경고에도 뉴질랜드 등이 보이콧 동참을 선언한 가운데 종전선언을 추진하고 있는 문재인 정부는 일단 올림픽 외교사절단 파견에 무게를 싣고 있다.

　젠 사키 미국 백악관 대변인은 이날 “조 바이든 행정부는 베이징 겨울올림픽과 패럴림픽에 외교 및 공식 대표단을 파견하지 않기로 했다”고 밝혔다. 선수단은 파견하되 개·폐회식에 정부 고위급이나 정치권 인사들로 구성된 공식 사절단은 보내지 않는다는 의미다. 사키 대변인은 “중국 신장에서 제노사이드와 인권 유린이 계속되고 있는 상황”이라며 “우리는 올림픽 경기의 팡파르에 동참할 수 없다”고 했다.

　바이든 행정부는 다른 동맹국의 보이콧 참여도 사실상 공개 요청했다. 네드 프라이스 국무부 대변인은 “더 많은 국가로부터 (보이콧) 소식을 듣게 될 것”이라고 했다. 이런 가운데 뉴질랜드가 7일 “베이징 올림픽에 정부 고위 대표단을 보내지 않기로 했다”고 밝혔다. 영국, 호주, 캐나다 등을 중심으로 보이콧 선언이 이어질 것으로 보인다.

　중국은 “단호한 반격 조치를 취하겠다”며 강하게 반발했다. 류펑위(劉鵬宇) 주미 중국대사관 대변인은 7일 “성공적인 올림픽 개최에 아무런 영향을 미치지 못할 정치적 조작”이라고 비판했다. 이에 앞서 자오리젠(趙立堅) 중국 외교부 대변인은 6일 정례 브리핑에서 “만약 미국이 독단적으로 행동한다면 중국은 반드시 반격하는 조치를 결연하게 취할 것”이라고 했다.

　미국은 9, 10일 한국 등 110여 개국을 초청해 화상으로 여는 민주주의 정상회의에서도 보이콧 동참을 요청할 것으로 보이는 가운데 청와대는 일단 올림픽 불참 가능성에 거리를 두고 있다. 최영삼 외교부 대변인은 7일 정례 브리핑에서 “미국 측은 외교 경로를 통해 이번 결정을 우리 측에 미리 알렸다. 다만 보이콧 동참을 요구해온 바는 없다”고 밝혔다. 그러면서 “우리 정부는 베이징 올림픽의 성공적 개최를 지지해 왔다. 올림픽이 세계 평화와 번영 및 남북관계에 기여하기를 희망한다”고 했다.

# 홍콩, ‘보안법’ 1년만에 5500명 학교 떠나

지난 1년 동안 홍콩에서 중고등학교를 그만둔 학생과 교사가 5000명을 넘어선 것으로 드러났다. 학교를 그만둔 학생 10명 가운데 6명은 아예 홍콩을 떠나겠다고 밝혔다. 반중 활동을 한 홍콩 시민을 최대 무기징역에 처할 수 있도록 한 홍콩 국가보안법 시행 등으로 홍콩의 사회 환경이 보안법 시행 전과 크게 달라진 여파 때문이라는 분석이 나오고 있다.

　2일 홍콩 매체 HK01 등에 따르면 전날 홍콩 중고교 교장들의 모임인 홍콩중학교장회는 140개 중고교를 대상으로 조사한 결과 2020∼2021학년도 1년 동안 학생 4460명과 교사 987명이 학교를 그만둔 것으로 나타났다고 밝혔다. 학교당 평균 32명의 학생과 7명의 교사가 그만둔 셈이다. 한 해 전 조사에서 각각 학생 2700명과 교사 498명이 그만둔 것보다 대폭 늘었다.

　특히 학교를 그만둔 학생 가운데 2643명(약 59.2%)은 홍콩을 떠나 다른 나라로 가겠다고 밝혔다. 중학교장회는 “학교를 그만두고 다른 나라로 이민을 선택하는 교사도 7배 이상 증가했다”며 “지난 1년 동안 학생과 교사의 이탈이 상당히 심각한 상황이라는 것은 명백한 사실”이라고 우려했다.

　이런 사태의 이유로 보안법 시행으로 홍콩의 전반적인 사회 환경이 억압적으로 변하고 교육 정책과 커리큘럼 또한 친중 일색으로 바뀌면서 학생과 교사 모두 실망했기 때문이라는 분석이 나온다. 신종 코로나바이러스 감염증(코로나19) 확산을 막기 위해 중국과 홍콩 간 왕래가 제한되면서 중국 본토 학생들이 홍콩 학교로 등하교할 수 없는 것도 영향을 미친 것으로 풀이된다. 중학교장회는 앞서 7월에도 당국에 “많은 학생과 교사들이 해외로 떠나고 있다”며 대책 마련을 요청했다.

# 中도 저출산-고령화… “4년뒤 ‘성인용 기저귀 〉 유아용’ 역전”

중국에서 2025년부터 성인용 기저귀 판매량이 유아용 판매량을 추월할 것이라고 영국 파이낸셜타임스(FT)가 보도했다. 중국이 겪고 있는 심각한 저출산 문제와 고령화 현상이 반영된 결과로 보인다.

　FT는 최근 중국의 유아용 기저귀 판매 수요가 점차 둔화되고 있는 반면 요실금을 앓는 고령층, 노인 요양시설 등을 위한 성인용 수요는 늘고 있다고 지난달 29일 보도했다. 중국 기업 관계자들은 2025년이면 연간 판매량에서 성인용 기저귀가 유아용을 앞설 것이라고 FT에 말했다.

　FT에 따르면 중국 1위 기저귀 판매사 유니참은 유아용보다 성인용에 마케팅 비용을 더 쓰고 있다. 중국 후베이성에 있는 기저귀 생산 공장 관계자는 “유아용 생산 라인을 성인용으로 바꿨다”고 했다. FT는 중국 기저귀 시장이 변곡점을 향해 가고 있다고 분석했다.

　홍콩계 증권사 CLSA의 분석에 따르면 지난해 중국의 기저귀 시장은 890억 달러(약 105조7943억 원)였는데 그중 성인용 시장은 10억 달러에 못 미쳤다. 하지만 성인용 기저귀 시장은 2040년이면 300억 달러(약 35조7300억 원) 규모로 성장할 것으로 전망됐다.

　이는 중국 인구 구조의 변화 때문이다. 세계은행 통계와 중국 인구조사에 따르면 중국 여성 1인당 평균 출산율은 1961년 5.9명에서 지난해 1.3명으로 줄었다. 1952년 조사 이래 최저치다. 프랑스 투자은행 나티시스는 지난해 중국의 고령층은 인구의 10%였지만 2050년이 되기 전에 25%로 늘어날 것이라고 전망했다.

　FT는 “기저귀 판매량의 변화는 인구통계학적 변화와 사회구조의 전환을 의미한다”고 전했다. 앞서 고령화를 겪은 일본도 10년 전부터 성인용 기저귀 판매량이 유아용을 추월하는 현상이 벌어졌다.

# 서훈 내일 1박 2일 방중…양제츠와 종전선언 논의

서훈 청와대 국가안보실장이 2일부터 1박 2일 일정으로 중국을 방문한다. 미국과의 종전선언 논의가 마무리 단계에 접어든 가운데 서 실장은 이번 방중에서 종전선언과 관련해 중국 측에 협조를 구하고 북한을 대화 테이블로 이끌기 위한 협력 방안을 모색할 것으로 보인다.

　30일 외교 소식통에 따르면 서 실장은 2, 3일 중국을 방문해 양제츠(楊潔지) 중국 공산당 외교담당 정치국원과 회동한다. 두 사람이 만나는 것은 지난해 8월 이후 1년 3개월여 만이다. 청와대는 “한중 양국은 고위급이 다양한 계기를 통해 전략적 소통을 유지하고 있고, 그런 맥락 속에서 서 실장 방중을 중국 측과 협의 중에 있다”고 밝혔다.

　서 실장은 이번 방중에서 종전선언에 대한 중국의 협조를 당부하고 내년 2월 베이징 겨울올림픽에 대한 의견을 교환할 것으로 보인다. 미국이 베이징 올림픽에서 외교적 보이콧을 시사했고, 신종 코로나바이러스 감염증(코로나19) 상황이 지속되는 등 올림픽 흥행 실패를 우려하는 중국은 우리 정부의 협조를 구하는 것으로 알려졌다. 청와대 관계자는 “베이징 올림픽이 남북관계 개선을 위한 전기가 되고 동북아 세계평화에 기여하는 계기가 되길 바란다는 입장에는 변함이 없다”면서도 “(외교적 보이콧 등에 대해) 아직 정부가 특별한 입장을 가질 상황은 아니다”라고 했다.

　이번 방중에서 서 실장은 시진핑(習近平) 중국 국가주석의 방한 문제도 논의할 것으로 보인다. 다만 코로나19 상황이 이어지고 있는 만큼 청와대는 화상 정상회담 방식도 고려하고 있는 것으로 알려졌다.

# 고향과 집

고향은 우리의 그리움이 향하는 곳이다. 그것은 자크 데리다에 따르면 “선영들이 묻혀 있는 땅” 혹은 “모든 여행과 모든 거리를 거기에서부터 가늠하는 부동의 장소”다. 낯선 땅에 살던 사람들이 죽을 때 고향에 묻히기를 바라는 것은 그래서다. 그런데 지난주에 제1회 부천디아스포라문학상 수상을 위해 한국을 찾은 미국 작가 하 진은 고향을 그렇게 인식하지 않는다. 그에 따르면 고향은 인간이 뿌리를 내리고 살아가는 곳이다. 두고 떠나온 곳이 아니라 어딘가에서 다시 만드는 유동적인 것.

　그의 삶을 생각하면 맞는 말이다. 미국에서 유학 중이던 그는 1989년 톈안먼 대학살의 실상을 텔레비전으로 지켜보다가 망명을 택했다. 그리고 중국을 배경으로 하는 작품들을 영어로 써서 전미도서상을 수상할 정도로 유명 작가가 되었다. 중국 정부는 그를 배반자로 낙인찍고 몇십 년 동안 입국을 허용하지 않았다. 그러나 그는 배반의 주체가 자신이 아니라 무고한 젊은이들을 학살한 국가라고 생각했다. 중국은 보호해줘야 하는 “자식들을 잡아먹은 어미”였다. 그에게 중국이 고향이 아니게 된 이유다.

　그러나 그의 심리적 현실은 다르다. 그는 미국이 집이라고 말하지만 중국으로 거듭 돌아간다. 중국인이나 중국인 이민자를 소설에 매번 등장시키는 것도 심리적, 은유적인 의미에서 보면 귀향이나 다를 바 없기 때문이다. 그가 정체성이 이미 확립된 서른 살 무렵에 고향을 떠났기에 더욱 그러한지 모른다. 그의 말과 다르게 미국이 진짜 고향, 진짜 집이 되는 것은 거의 불가능해 보인다.

　그는 한국에 왔음에도 몇 시간이면 갈 수 있는 고향에 가지 못하고 그의 집이 있는 미국으로 쓸쓸히 돌아갔다. 하기야 지난 36년을 그렇게 살았다. 부모가 세상을 떠났을 때도 고향에 가서 애도조차 할 수 없었다. 가혹한 형벌이었다. 그러한 실존이 그를 디아스포라 작가로 만들었다. 그가 쓴 시와 소설 주변에 상처가 아른거리는 이유다.

# 美中 반도체 전쟁에 낀 하이닉스, 초격차 확대로 극복해야

네덜란드산 첨단 장비를 도입해 중국 장쑤성 우시 공장을 개선하려는 SK하이닉스의 계획이 미국 정부의 반대로 무산될 위기에 처했다고 로이터통신이 어제 보도했다. “SK하이닉스가 미국과 중국의 지정학적 분쟁에서 다음 차례 희생양이 될 수 있다”고도 했다. 미중 경제패권 전쟁의 한복판에 놓인 우리 반도체 기업들이 가장 우려하던 사태가 현실로 나타날 가능성이 커진 것이다.

　SK하이닉스의 3분기 세계 D램 반도체 시장 점유율은 27%로 삼성전자(44%)에 이은 2위다. 정부로부터 각종 보조금과 세제지원을 받으며 추격하는 미국 유럽연합 중국 경쟁업체들을 따돌리기 위해서는 첨단 기술, 장비에 대한 공격적 투자가 필수적이다. 특히 우시 공장은 이 회사 D램 제품 절반이 생산되는 중요 시설이어서 기업 경쟁력에 악영향이 미칠 수 있다.

　조 바이든 정부는 미국 및 동맹국 기술이 사용된 첨단 반도체 장비가 중국으로 들어가는 걸 절대 용납하지 않을 태세다. 중국의 군사력 강화에 악용될 수 있다는 게 표면적 이유지만 양과 질 모든 면에서 제조업 최강국에 올라서겠다는 ‘중국제조 2025’ 계획 등을 견제하는 게 주목적이다. 미국 정부는 지난주 중국 현지 실리콘웨이퍼 생산량을 늘리려던 자국 반도체업체 인텔의 계획까지 포기시켰을 정도로 일체의 예외를 인정하지 않는 분위기다.

　문제는 중국이 한국산 반도체 40%를 사가는 최대 고객이란 점이다. 홍콩을 통한 우회수출을 합하면 비중이 60%가 넘는다는 분석도 있다. 미국의 제동 때문에 중국 공장에 대한 시설투자를 제대로 못하면 중국은 미국 대신 애먼 한국 기업을 표적삼아 보복에 나설 가능성이 있다. ‘싸드 사태’ 때 그랬듯 전혀 다른 분야의 한국기업에까지 불똥이 튈 수도 있다.

　이런 불편한 상황을 주도적으로 해결하는 방법은 어떤 갈등이 불거져도 중국 기업들이 한국산 반도체를 사지 않을 수 없도록 기술, 품질 초격차를 더 벌리는 길뿐이다. 그런데도 SK하이닉스의 투자가 예정된 용인 반도체 클러스터는 주민 설득, 인허가가 지연돼 당초 계획보다 착공이 1년 이상 늦어지고 있다. 정부와 정치권은 반도체 산업 육성을 위한 특별법에 더 과감한 지원책과 규제완화 방안을 담아 서둘러 통과시켜야 한다.

# 美 간 최종건 “현실적으로 中과 파트너십 필요”

최종건 외교부 1차관이 15일(현지 시간) 미국 워싱턴에서 한미 관계를 주제로 열린 전략포럼에서 “중국은 전략적 파트너이며 현실적으로 베이징과의 파트너십이 필요하다”며 한중 관계의 중요성을 강조했다. 이에 미국 행정부 전직 고위당국자들은 “한미 동맹이 장기적으로 약화하고 미국의 정책결정 과정에서 한국이 간과될 위험성이 있다”는 우려를 제기했다.

　최 차관은 이날 워싱턴의 싱크탱크 전략국제문제연구소(CSIS)와 한국국제교류재단(KF)이 공동 주최한 한미전략포럼 기조연설에서 우선 한미동맹의 중요성을 역설했다. “한미 두 나라는 21세기의 동맹이 어떤 것인지를 전 세계에 보여주고 있다”며 한미 동맹이 전통적인 안보뿐 아니라 경제, 문화 분야에서도 파트너십을 진전시키고 있다고 평가했다.

　그러나 그는 이어진 질의응답에서 중국에 대한 한국의 입장을 묻는 질문에 “그들은 전략적 파트너”라며 “다른 국내정책과 마찬가지로 외교정책 또한 한국인, 한국 중산층의 필요와 이해관계에 맞는 것이어야 한다”고 말했다. 이어 “중국과의 교역 규모는 미국 및 일본을 합친 것보다 크고 그 시장에서 오는 큰 수익의 혜택을 즐기는 것은 우리 국민들”이라고 설명했다. 또 공급망 문제를 거론하며 “중국에서 오는 여러 품목에 대한 의존도는 우리 문제만이 아니라 모두의 문제”라고 지적했다. 북한 문제에 있어서도 “현실적으로 베이징과 파트너십이 필요하다”며 “우리가 좋든 싫든 간에 그것이 우리 정책의 현실”이라고 했다. 한국이 지리적으로 중국에 가장 가까운 국가임을 상기시키며 “우리는 중국과 좋은 관계를 형성하려고 노력하고 있다”고 했다.

　최 차관의 질의응답이 끝난 뒤 같은 자리에 패널로 참석한 랜들 슈라이버 전 미국 국방부 아시아태평양 담당 차관보는 “어느 한쪽은 뭔가를 중요하고 핵심적인 도전으로 보는데 다른 한쪽은 이를 받아들이지 않는 동맹관계를 가질 수는 없다”며 “(한국이) 그런 식으로 표류한다면 (한미)동맹이 점차 약화할 수 있다는 점에서 위험할 수 있다”고 했다.

# 中요소수 1만8700t 풀기로 일단 숨통

중국 정부가 한국 기업들과 계약한 요소수 물량 1만8700t에 대한 수출 절차를 정상적으로 진행하겠다고 우리 정부에 통보했다. 국내 소요량의 두세 달 치에 해당하는 물량이다. 이 중 차량용 요소 1만여 t은 이르면 이달 말에서 내달 초 사이 국내에 들어올 것으로 예상된다. 일단 요소수 부족 사태에 대한 급한 불은 껐지만 중국이 요소수에 대한 수출 전 검사 제도를 중단하지는 않은 만큼 중국산 요소수 수입이 지속적으로 원활하게 이뤄질지는 미지수다. 외교부는 10일 “중국산 요소 수입 절차의 조속한 진행을 위해 다양한 채널로 중국 측과 소통한 결과 우리 기업들이 이미 계약한 물량(1만8700t)에 대한 수출 절차가 진행될 것임을 확인했다”고 밝혔다. 수출 전 검사 신청부터 검사 완료까지 약 2주가 걸리는 것을 감안하면 이달 말에서 내달 초까지 계약 물량이 국내에 들어올 수 있다는 것. 또 1만8700t 중 7100t은 수출 전 검사를 신청한 상황이고, 이 중 A사가 수입하는 차량용 요소수 300t은 검사가 완료돼 다음 주 한국으로 들어온다고 외교부가 밝혔다.

　다만 중국이 사실상 요소수 수출에 제동을 건 만큼 앞으로 수급이 이전 수준으로 완전히 회복되기는 어려울 것으로 관측된다. 외교부 당국자는 “단기적으로 (수출 절차가) 정상화된 것이라고 이해하면 된다”고 말했다.

# “中헝다 사태 리스크 국제 경제 위협 우려”

미국 중앙은행인 연방준비제도(Fed·연준)가 파산 위기에 몰린 중국의 대형 부동산 회사 헝다(恒大) 사태의 파장이 미국 등 글로벌 경제로 확산될 수 있다고 8일(현지 시간) 경고했다. 올 9월만 해도 제롬 파월 연준 의장이 “헝다 문제는 중국에 국한된 것”이라며 글로벌 위기로의 확산 가능성을 일축한 것에 비하면 미국이 이 사태를 바라보는 시각이 달라진 것으로 풀이된다.

　연준은 이날 발간한 반기 금융안정보고서에서 “중국의 부동산 문제가 미국 금융 시스템에 리스크를 주고 있다”면서 이같이 짚었다. 연준은 보고서에서 “중국 경제와 금융 시스템의 규모, 전 세계와 무역 연계도 등을 감안했을 때, 중국 금융의 불안은 위기 심리의 악화를 통해 글로벌 금융시장에 압박을 주고 글로벌 경제 성장을 위협하며 미국에도 영향을 줄 수 있다”고 분석했다.

　연준은 또 “중국은 기업과 지방정부의 부채가 여전히 크고 금융 부문의 레버리지(차입금을 통한 투자)가 높으며 부동산 가치도 지나치게 오른 상태”라며 “이런 환경에서 중국이 레버리지가 높은 기관에 대한 규제에 집중할 경우, 헝다 사태에서 보듯이 부동산 섹터 등 부채가 높은 기업들에 위기를 초래할 가능성이 있다”고 했다. 헝다그룹은 그간 차입금을 바탕으로 공격적으로 사업을 확장해 왔지만 최근 당국이 부동산 규제에 나서면서 유동성 위기에 처한 상태다. 연준은 이어 “위기가 금융회사에 전이되고 부동산 가격이 갑작스러운 조정을 겪거나 투자자들의 위험 감수 성향이 줄어들 경우 중국의 금융 시스템이 큰 압력을 받을 수 있다”고 덧붙였다.

# 내년 1월 RCEP 발효… 日 “韓中과 첫 FTA 기대”

내년 1월부터 중국, 일본 등 10개국 간에 역내포괄적경제동반자협정(RCEP)이 발효되면서 일본에서 경제 활성화에 대한 기대감이 나오고 있다. RCEP 발효로 일본 국내총생산(GDP)이 2.7% 상승할 것이라는 전망도 나왔다. 한국도 RCEP에 서명했지만 아직 국회 비준이 끝나지 않아 내년 1월 발효국에는 포함되지 않았다.

　일본 외무성은 “호주와 뉴질랜드가 2일 RCEP 협정을 비준하면서 발효 조건이 충족됐다”며 “내년 1월 1일부터 일본, 중국, 호주, 뉴질랜드, 싱가포르, 베트남, 태국, 브루나이, 캄보디아, 라오스 등 10개국에서 RCEP가 발효된다”고 3일 발표했다. 우리나라를 비롯해 인도네시아, 말레이시아, 미얀마, 필리핀 등 5개국은 아직 비준서를 기탁하지 않았다.

　RCEP는 가맹국 간에 상호 관세를 내려 역내 통상을 활성화하는 다국 간 자유무역협정(FTA)이다. 15개 서명국이 모두 비준을 마치게 되면 RCEP는 미국이 빠진 채 11개국이 참여 중인 포괄적·점진적 환태평양경제동반자협정(CPTPP)보다 규모가 큰 세계 최대 FTA가 된다. 전체 무역 규모는 2019년 기준 5조6000억 달러(약 6600조 원·전 세계의 31.9%), GDP는 26조 달러(30.8%), 인구는 22억7000만 명(29.7%)이다. RCEP는 한중일 세 나라가 참여하고 향후 발전 가능성이 큰 동남아국가연합(ASEAN)이 포함된 FTA라는 점에서 주목을 받고 있다.

　일본 정부 대변인인 마쓰노 히로카즈(松野博一) 관방장관은 4일 기자회견에서 “세계의 성장 센터 지역들과 우리나라의 연결로 지금보다 더 경제성장에 기여할 것”이라며 “(일본이) 주도적인 역할을 해 나가겠다”고 했다.

　니혼게이자이신문은 “일본이 한국, 중국과 맺은 첫 FTA가 움직이기 시작한다”며 “일본 GDP를 약 2.7% 끌어올리는 효과가 전망된다”고 분석했다. 요미우리신문은 “참가국은 경제 회복에 대한 기대감이 커지는 한편으로 아시아태평양 지역의 통상 분야에서 주도권을 잡으려는 중국에 대한 경계감도 높아지고 있다”고 전했다.

　인도도 RCEP 참여를 타진했으나 최종 서명에서 빠졌다. 중국과의 무역에서 적자에 시달려 온 인도는 값싼 중국 제품의 공세가 거세질 것을 우려해 참여하지 않기로 했다.

# 미군 2인자 “中군사력 증강 충격, 이대로면 美 추월”

미군 서열 1위인 마크 밀리 합참의장이 중국의 극초음속 미사일 발사를 확인하며 이에 대한 우려를 나타낸 데 이어 존 하이튼 합참차장도 중국의 군사력 증강 속도를 두고 “충격적(stunning)”이라고 했다. CNN 등에 따르면 하이튼 합참차장은 21일(현지 시간) “중국이 움직이는 속도는 충격적이다. 이 속도와 궤적대로라면 중국은 러시아와 미국을 따라잡게 될 것”이라고 말했다. 또 “이는 게임(의 판도)을 바꾸게 된다는 점에서 미국뿐 아니라 우리 동맹국의 문제이기도 하다”고 강조했다.

　그의 설명에 따르면 최근 5년간 중국이 수백 회의 극초음속 미사일 시험발사를 한 데 비해 미국은 9회밖에 하지 않았다. 이런 큰 차이를 두고 그는 “좋지 않다”고 지적했다. 미국의 발사 실패에 대해선 “학습 과정으로 봐야 한다”며 “위험을 감수하고 실패에서 배워야 더 빨리 전진하는 것”이라고 했다. 하이튼 합참차장은 그러면서 김정은 북한 국무위원장이 과거의 실패한 시험발사를 통해 개발에 속도를 내는 법을 배웠다며 북한을 사례로 들었다. 김정은은 과학자와 엔지니어들이 실패했을 때도 아버지(김정일)와 달리 이들을 처형하지 않기로 결정했고 오히려 격려하며 실패에서 배우기도 했다는 것이다. 그는 “결국 그렇게 해서 세계 경제 순위 118위 국가인 북한이 대륙간탄도미사일(ICBM) 역량을 확보했다”고 덧붙였다.

# 공급난 美中 가격 줄인상…한국도 수입물가 빨간불

세계적으로 확산하고 있는 공급 대란의 여파로 미국과 중국을 비롯한 글로벌 경제의 인플레이션 압력이 커지고 있다. 극심한 구인난과 공급망 위기에 처한 미국 기업들은 비용 증가를 견디다 못해 제품 가격을 줄줄이 인상하고 있다. 최근 전력난과 원자재 가격 급등의 직격탄을 맞은 ‘세계의 공장’ 중국도 물가가 크게 뛰었다. 중국의 인플레이션이 전 세계로 전이될 위험이 커진 가운데 한국 역시 수입물가에 비상이 걸렸다. 공급망 위기가 경기 회복을 저해함에 따라 미국의 3분기(7∼9월) 경제성장률은 앞선 분기의 반 토막 수준에 불과할 것이란 전망도 나오고 있다.

　27일(현지 시간) 뉴욕타임스(NYT)와 로이터통신 등에 따르면 이날 3분기 실적을 발표한 미국의 주요 기업들이 일제히 제품 가격 인상을 예고하고 나섰다. 원자재와 인건비 상승에 따른 비용 증가분을 소비자에게 결국 전가하고 있는 것이다.

　패스트푸드 체인 맥도날드는 인건비와 원자재 가격 상승분을 반영하기 위해 올해 제품 가격을 6%가량 인상한다고 밝혔다. 맥도날드는 구인난으로 인해 올해 인건비가 벌써 10% 이상 올랐고 음식 재료와 기타 자재 값도 최대 4% 증가하는 등 비용 압박을 받고 있는 것으로 알려졌다. 물가 상승에 대응해 제품 가격을 이미 1.5% 올린 식품기업 크래프트하인즈는 내년에도 이런 가격 정책 기조를 이어가겠다고 밝혀 추가적인 가격 인상 가능성을 내비쳤다. 코카콜라 역시 이날 실적을 발표하면서 “인건비와 물류비용이 높게 유지된다면 필요에 따라 가격을 올릴 수 있다”고 발표했다. 글로벌 제조기업 3M도 “폴리프로필렌 등 원자재 비용과 인건비 상승에 직면하고 있다”면서 “인플레이션과 공급망 압력에 대응하기 위해 제품 가격을 인상할 것”이라고 했다.

　기업들의 이 같은 가격 인상 행렬은 최근 이례적인 인력난과 공급망 위기가 주요 원인이다. 미국에서는 월별 구인 건수가 계속 1000만 명을 넘을 정도로 일손이 많이 필요한 상황이지만 정작 일할 사람이 부족해 기업들이 앞다퉈 임금을 올리고 있는 실정이다. 또 항만과 육상 물류에 병목 현상이 생기면서 기업들은 단가가 비싼 항공 화물에 대한 의존도가 높아졌고 아예 자체 화물선을 띄우는 곳도 등장하고 있다

# 베이징올림픽, 방역 위해 중국 본토 관중만 입장

중국이 내년 2월 베이징 겨울올림픽을 최소 규모로 치르겠다고 밝혔다. 불필요한 활동과 절차를 줄이고 올림픽에 투입되는 인력도 대폭 감축할 방침이다. 신종 코로나바이러스 감염증(코로나19) 확산을 막기 위한 조치다.

　26일 관영 신화통신 등 중국 매체들에 따르면 베이징 겨울올림픽 조직위원회는 전날 올림픽 방역 수칙이 담긴 방역수첩(매뉴얼)을 공개했다. 1차로 공개된 이번 방역 매뉴얼은 선수와 대회 관계자들에게 적용되며 앞으로 한두 차례 더 보완될 수 있다.

　매뉴얼에 따르면 이번 올림픽을 위해 마련한 전용 교통편과 숙소, 부대시설, 경기장과 훈련장 등 모든 공간은 하나로 연결돼 운영된다. 이 공간들은 마치 거품(버블)을 덮어씌운 것처럼 다른 외부 공간과는 완전히 격리된 폐쇄 구역이 된다. 대회 참가자들은 제한된 공간만 오갈 수 있으며 매일 코로나19 검사를 받아야 한다. 코로나19 백신 접종을 완료하지 못한 대회 참가자는 중국 도착 후 21일간 베이징에서 격리해야 한다.

　올림픽 투입 인력을 최소화할 방침이어서 선수단 편의를 위해 활동했던 자원봉사자들도 사라질 것으로 보인다. 무관중으로 치러진 2020 도쿄 올림픽과 달리 관중을 입장시킬 계획이지만 규모를 최소화할 방침이며 중국 본토 밖에서 온 관중은 수용하지 않는다.

　베이징 올림픽을 앞두고 미국과 유럽연합(EU)의 집단 보이콧 움직임도 지속되고 있다. 하지만 전문가들은 보이콧이 실제로 이뤄질 가능성이 높지 않다고 보고 있다. 김흥규 아주대 국제학부 정치외교학과 교수는 “미국이 최근 중국에 유화적인 모습을 보이고 있다”며 “중국과의 불화는 미국 여론에 악영향을 미칠 수 있다. 내년 중간선거를 앞둔 조 바이든 정부가 그런 위험을 감당하려 하진 않을 것”이라고 분석했다.

# 일본인 90% “中 싫어”…중국인 66% “日 싫어”

일본인 10명 중 9명은 중국에 대한 인상을 “좋지 않다”고 답한 것으로 나타났다. 일본에 대한 인상을 “좋지 않다”고 답한 중국인도 66.1%다. 중일 양국은 내년 9월 국교정상화 50주년을 앞두고 있지만 올해 양국 모두 상대에 대한 감정이 전년보다 악화됐다.

　21일 마이니치신문에 따르면 일본의 비영리단체 겐론NPO와 중국 국제출판집단은 8월 21일∼9월 25일 18세 이상 남녀 1000명(일본), 1547명(중국)을 대상으로 상대국에 대한 인식을 조사했다. 일본인 가운데 중국에 부정적인 인상을 가진 사람은 지난해보다 1.2%포인트 늘어난 90.9%였다. 2016년 조사에서 91.6%로 조사를 시작한 이후 가장 높았는데 5년 만에 다시 90%를 넘었다.

　중국인 가운데 일본에 부정적인 인상을 가진 사람은 지난해보다 13.2%포인트 급증한 66.1%였다. 대일(對日) 부정적 인상은 센카쿠열도(중국명 댜오위다오) 갈등으로 양국이 충돌했던 2013년 90.1%로 최고를 찍은 뒤 꾸준히 줄었지만 1년 만에 크게 늘어났다. 구도 야스시(工藤泰志) 겐론NPO 대표는 20일 기자회견에서 “상호 군사적인 위협만 논의되고 양국 국민의 불안이 방치되고 있다”고 분석했다.

　중일 국민이 갖고 있는 상대국에 대한 부정적 인식은 한일 국민보다 높았다. 겐론NPO가 한국 동아시아연구원(EAI)과 실시한 한일 국민 상호인식 조사 결과에 따르면 한국인이 갖고 있는 일본에 대한 부정적 인상은 지난해 71.6%에서 올해 63.2%로 줄었다. 일본인이 가진 한국에 대한 부정적 인상은 같은 기간 46.3%에서 48.8%로 소폭 증가하는 데 그쳤다. 2015년 이후 일본인의 반중 감정은 반한 감정보다 2배 가까이 높은 상태가 지속되고 있다.

# “中, 8월 극초음속 미사일 시험발사… 美 놀라게 했다”

중국이 8월 핵무기를 탑재할 수 있는 극초음속 미사일을 비밀리에 시험발사한 것으로 드러났다고 영국 파이낸셜타임스(FT)가 소식통을 인용해 16일 보도했다.

　FT에 따르면 중국의 극초음속활공체(HGV)는 목표물에서 약 32km 거리에 떨어졌다. FT는 “중국 극초음속 무기의 진전을 보여준 이 테스트는 미국 정보기관을 놀라게 했다”고 전했다. 중국이 극초음속 미사일을 보유한 것은 이미 알려졌지만 이번 시험발사로 미국이 알고 있는 것보다 중국의 극초음속 무기 관련 기술이 발전했다는 사실이 입증되었기 때문이다. 중국 핵무기 정책 전문가인 테일러 프레이블 매사추세츠공대(MIT) 교수는 FT 인터뷰에서 “핵탄두를 탑재한 극초음속 미사일이 완성되면 미국의 미사일방어(MD) 체계를 무용지물로 만들 것”이라고 말했다.

　극초음속 미사일은 음속의 5배에서 최대 20배 이상까지 속도를 내는 미사일로 전쟁의 판도를 바꿀 수 있는 ‘게임 체인저’로 불리는 첨단 무기다. 탄도미사일은 우주로 높이 올라 포물선을 그리며 날아가지만 극초음속 미사일은 대기 중에서 낮은 궤도로 날아가다가 목표물을 빠르게 타격한다. 순항미사일과 탄도미사일을 겨냥한 각국의 MD 체계를 무력화시킬 것으로 평가되고 있다.

　전 세계 어느 곳이든 1∼2시간 안에 타격할 수 있고 미사일방어망을 뚫을 수 있어 미국, 중국, 러시아 등 군사 강대국들은 극초음속 미사일 개발에 앞장서고 있다. 북한도 지난달 28일 극초음속 미사일 ‘화성-8형’ 시험발사를 진행하며 경쟁에 가세했다. 중국은 2019년 10월 건국 70주년 열병식에서 극초음속 미사일 ‘둥펑-17’을 처음 공개한 바 있다. 같은 해 말 러시아는 ‘아방가르드’를 실전 배치했으며 지난해에는 신형 극초음속 순항미사일인 ‘지르콘’ 시험발사에 성공했다.

　미국은 최근 극초음속 미사일 개발을 강도 높게 추진해 왔으나 현재까지는 중국, 러시아와의 경쟁에서 밀리고 있다는 평가를 받는다. 미국은 4월 B-52H 전략폭격기가 극초음속 미사일인 ‘AGM-183A ARRW’를 발사하는 시험을 했지만 성공하지 못했다.

# 시진핑, 떠나는 메르켈에 이례적 웃음… 한시 인용 덕담

# 시진핑(習近平) 중국 국가주석이 2005년부터 16년간 집권했으며 곧 퇴임을 앞둔 앙겔라 메르켈 독일 총리와 13일 화상회담을 가진 후 메르켈을 ‘라오펑유(老朋友·오랜 친구)’라고 부르며 극진히 예우했다. 메르켈 총리가 재임 중 중국을 12차례나 방문했고 미중 갈등에서도 일방적으로 미국 편만 들지 않았다는 점을 높이 평가한 것으로 풀이된다. 메르켈 총리는 주독 미군 방위비 분담금 증액 압박 등을 가하는 도널드 트럼프 전 미 행정부와 상당한 마찰을 빚었다. 　14일 공산당 기관지 런민일보는 전일 시 주석과 메르켈 총리가 화상회담을 가지면서 활짝 웃고 있는 사진을 1면에 게재했다. 시 주석은 공개석상에서 거의 웃지 않는 것으로 유명하지만 이날 이례적으로 시종일관 미소를 지었다. 런민일보는 “메르켈이 재임하는 동안 중국과 독일 관계는 물론이고 중국과 유럽연합(EU)의 관계도 매우 돈독해졌다”고 평했다. 특히 시 주석이 “중국인은 정(情)과 의(義)를 중시하고 라오펑유를 잊지 않는다. 중국의 대문은 언제라도 당신을 향해 활짝 열려 있다”고 강조했다고 덧붙였다. 　중국 외교부 또한 홈페이지를 통해 시 주석이 이번 회담에서 ‘사람과 사람이 서로 아는 것이 제일 중요하고, 서로 알려면 상대의 마음을 알아야 한다(人之相識 貴在相知, 人之相知 貴在知心)’는 맹자의 구절을 인용했다고 밝혔다. 또 메르켈의 재임 중 중국과 독일은 ‘제로섬’ 게임을 피하고 상호 이익을 얻을 수 있음을 증명했다고 치하했다. 　관영 영자지 글로벌타임스도 메르켈이 주요국 지도자 중 중국을 가장 많이 방문했으며 실용적인 대중국 정책을 펼쳤다고 호평했다. 2005년 11월 취임한 메르켈은 신종 코로나바이러스 감염증(코로나19) 발생 전인 2019년 9월까지 총 12차례 중국을 방문했다. 같은 기간 시 주석은 세 차례 독일을 찾았다. 두 사람은 코로나19 사태 후 전화와 화상회담으로 접촉을 이어갔다. 특히 올해에만 다섯 번 교류했다. 유가 80달러… 공급대란에 高물가 ‘쓰나미’까지 밀려오나

공급망 위기와 원자재 값 폭등이 겹치면서 세계 경제가 혼돈 속으로 빠져들고 있다. 부품이 없어 공장이 멈추고, 배럴당 80달러까지 오른 유가는 글로벌 경기 회복의 발목을 잡고 있다. 당장 미국 중국 등 거대 경제권의 경기가 둔화될 조짐을 보이고 있다. 이는 수출로 지탱하던 한국 경제에 직격탄이 될 수 있다. 유가로 인한 물가 상승까지 겹치면 내수 위축도 불가피한 상황이다.

　11일(현지 시간) 미국 서부텍사스산원유(WTI) 가격은 7년 만에 배럴 당 80달러를 돌파했다. 코로나 회복세와 난방용 수요가 겹친 탓이다. 이 때문에 핀란드 노르디아뱅크는 내년 미국 성장률 예상치를 3.5%에서 1.5%로 낮췄다. 유가 폭등 탓에 물가가 오르고 소비가 둔화될 수밖에 없다는 뜻이다. 이런 흐름은 글로벌 경제와 한국 수출에 악영향을 줄 수밖에 없다.

　공급망 위기도 악화되고 있다. 원자재와 중간재를 가리지 않고 병목 현상을 빚는데다 물류난까지 벌어지고 있다. 소비자가 제품을 제 때 공급받지 못하면서 물가가 가파르게 오르고 있다. 물가가 오르면 기업들이 비싼 값에 팔려고 생산을 늘리는 게 상식이다. 하지만 생산 자체가 어려운 상황이어서 자칫 경기침체 속에 물가만 오르는 스태그플레이션 우려도 나오고 있다.

　한국 소비자물가는 9월까지 6개월 연속 2%대 상승률을 나타내며 당초 관리 목표인 1.8%를 넘어섰다. 물가를 잡으려면 금리 인상 등 긴축에 나서야하지만 경기 회복에 찬물을 끼얹을 수 있다. 한국은행이 어제 기준금리를 동결한 것도 이런 고민의 결과다.

　기업들은 원자재와 부품 공급을 위해 비상 체제를 가동하고 있다. 정부도 수출 호조에 취해 안일하게 대응할 때가 아니다. 공급망에 차질이 없도록 기업과 공동 대응에 나서고, 유가 상승에 따른 전기요금 영향도 면밀히 따져봐야 한다. 물가와 경기를 감안한 세심한 금리 정책도 쉽지 않은 과제이다. 상황을 지켜만 보기에는 글로벌 환경이 너무 긴박하다.

# 대규모 전력난에… 중, 호주산 석탄 다시 수입 ‘백기’

미중 갈등 속에서 미국 편에 선 호주에 보복하기 위해 중국 당국이 호주산 석탄의 수입을 막았지만 석탄 부족 사태가 심각해지자 중국 수입업자들이 호주산 석탄을 하역하기 시작했다고 영국 파이낸셜타임스(FT)가 4일 보도했다. 석탄 부족으로 발전소 운영이 중단되고 대규모 전력난으로 이어지자 사면초가 상황에 몰린 중국이 호주에 굴복했다는 분석이 나온다.

　FT에 따르면 지난달 말부터 중국 주요 항구에서는 바다에 대기 중이었던 호주 화물선에서 석탄을 하역하는 작업이 이뤄지고 있다. 국제 선박중개회사 브래마 ACM의 닉 리스틱 화물책임자는 석탄 45만 t이 하역됐다고 전했다. 에너지컨설팅업체 케이플러도 지난달 선박 5척에서 호주산 석탄 38만3000t이 하역됐다고 FT에 밝혔다. 현지 무역업자들은 중국 당국이 “통관을 허락한다”는 신호를 보낸 것으로 받아들이고 있다.

　지난해 중국은 국영 에너지 기업과 제철소에 “호주산 석탄 수입을 중단하라”고 명령했다. 세계 최대 석탄 수출국인 호주에 대한 무역 보복이었다. 이 조치로 호주는 약 39억 달러(약 4조6352억 원)의 손실을 입었다.

　호주산 석탄 수입이 금지되고 시진핑 중국 국가주석의 ‘친환경 저탄소’ 정책이 겹치면서 중국에서는 석탄 부족 사태가 벌어졌다. 이는 중국 동북부의 전력난으로 이어져 일부 지역에서는 공장 가동이 중단되고 가정용 전기 공급도 제한되고 있다. 지린성 등 중국 각 지방정부는 인도네시아, 러시아, 몽골, 카자흐스탄 등에서 석탄을 확보하기 위해 안간힘을 쓰고 있지만 세계적으로 석탄 수요가 늘면서 가격도 폭등해 수입하기 어려운 상황이다.

# ‘파산 위기’ 中헝다 사태 여파 스웨덴까지 번져

약 355조 원에 달하는 천문학적 부채로 파산 위기에 직면한 중국 부동산회사 헝다그룹 사태의 후폭풍이 북유럽 스웨덴으로도 번졌다.

　2일 블룸버그 등에 따르면 헝다 계열사 헝다뉴에너지자동차(헝다자동차)와 합작해 전기차를 개발하고 있는 스웨덴 자동차회사 ‘내셔널일렉트릭비이클스웨덴(NEVS)’은 최근 공장 직원 670명 중 절반에 가까운 300명을 해고했다.

　내셔널일렉트릭 관계자는 “헝다자동차로부터 자금 조달이 이뤄지지 않아 감원은 물론 전기차 개발도 중단됐다”며 “공장을 사실상 폐쇄했다”고 밝혔다. 스테판 틸크 최고경영자(CEO) 역시 “헝다가 아닌 새로운 합작사와 투자자를 찾고 있다”고 밝혔다.

　내년 전기차 양산을 준비했던 헝다자동차는 최근 중국 내 공장 설비업자에게도 대금을 지급하지 못했다. 일부 직원의 월급도 밀렸고 연구소 연구원들을 위한 무료 식사 제공도 모두 중단했다.

　헝다그룹이 지난달 말 자회사가 보유 중인 주식을 팔아 약 1조8300억 원의 자금을 확보했지만 올해 안에 갚아야 할 이자만 7500억 원에 달하는 등 위기가 계속되고 있다. 특히 주력 사업인 부동산은 중국 정부의 규제로 침체기에 접어들었고, 유망했던 전기차 사업마저 차질이 불가피해져 헝다그룹의 자력 회생은 사실상 불가능하다는 전망도 나온다.

　헝다그룹은 지난달 29일 달러화 채권 이자 약 559억 원을 채권자들에게 지급하지 못했다. 앞서 같은 달 23일에도 달러화 채권 이자 약 993억 원을 지급하지 않고 30일 유예시켰다.

# 정의용 “中 공세적 외교는 당연”…韓외교수장, 美서 ‘中두둔’ 논란

정의용 외교부 장관이 22일(현지 시간) 미국과 한국, 일본, 호주 등 동맹국들을 중국에 맞서는 하나의 연대로 묶는 것을 두고 “냉전시대 사고”라고 규정했다. 미국에서 비판받는 중국의 ‘공세적(assertive) 외교’에 대해서는 “중국으로서는 당연한 일”이라고 했다. 조 바이든 미국 행정부의 중국 견제 정책이 본격화하고 있는 시점에 한국의 외교수장이 미국에서 중국을 두둔하는 듯한 발언을 내놓은 것이어서 논란이 되고 있다.

　유엔총회 참석차 미국을 방문 중인 정 장관은 이날 뉴욕의 싱크탱크 미국외교협회(CFR) 초청 대담에서 ‘최근 몇 년간 중국이 점점 더 공세적으로 돼 가고 있다고 보느냐’는 대담 진행자의 질문에 “그것은 당연한 일(only natural)”이라며 “중국은 경제적으로 더 강해지고 있고 지금은 20년 전의 중국이 아니다”라고 답변했다. 그는 “중국이 가진 것을 외교정책에 반영하기를 바라는 것은 자연스러운 일”이라며 “이것을 공세적이라고 부를 수 있는지 모르겠다”고 했다. “중국은 국제사회 일원으로 자신들의 목소리가 반영되기를 바라는 것”이라며 “우리는 그들이 우리에게 하려는 이야기에 귀 기울여야 한다”고도 했다.

　진행자 파리드 자카리아 CNN 앵커가 인도태평양 지역의 외교 지형과 관련한 설명을 하면서 미국, 한국, 일본, 호주를 중국에 맞서는 하나의 블록으로 구분하려 하자 정 장관은 “그것은 중국 사람들이 말하듯이 냉전시대 사고(the mentality of Cold War)”라고 했다. 정 장관은 미국이 인도태평양 지역에서 중국 견제를 위한 주요 연합체로 삼고 있는 ‘쿼드(Quad)’ 가입에 대한 질문에는 “가입해야 할 긴급한 필요성을 느끼지 않는다”고 했고, 미중 두 나라를 두고서는 “어느 한쪽을 선택해야 한다고 생각하지 않는다”고 답변했다.

# 9•11테러 20년, 美中‘힘의 오만’ 접고 경쟁•협력해야

9·11테러 20주년을 맞은 11일, 미국 곳곳에선 희생자들을 기리는 추모식이 열렸다. 2977명의 목숨을 앗아간 테러가 발생한 지 20년이라는 상징성에다 그 테러가 유발한 아프가니스탄 전쟁의 지난달 말 종료는 추모 분위기를 한층 엄숙하게 만들었다. 곳곳엔 ‘절대 잊지 않겠다(Never Forget)’는 문구가 내걸렸고, 전·현직 대통령 등 미국 지도자들은 한결같이 테러에 맞선 국민의 통합과 단결을 주문했다. 세계 정상들도 국제적 연대를 강조했다.

　9·11테러는 21세기 벽두 자유주의 국제질서에 엄청난 공포와 분노를 불러오면서 유일 초강대국 미국의 파워를 시험대에 올린 사건이었다. 10년 전 냉전 승리와 함께 일극(一極)의 질서를 이끌던 미국은 즉각 ‘테러와의 전쟁’을 선포하면서 아프간 전쟁, 그리고 이라크 침공까지 그 파워를 유감없이 발휘했다. 하지만 아프간과 이라크는 미국의 ‘수렁’이었다. 지난달 아프간에서 쫓겨나듯 철수하던 미군의 초라한 모습은 이를 상징적으로 보여줬다.

　미국은 이제 그 수렁에서 벗어나 중국과의 본격적인 패권경쟁에 온전히 집중하려 한다. 조 바이든 행정부는 취임 직후부터 이전 행정부보다 훨씬 정교하고 치밀한 전략 아래 중국을 견제·포위하겠다는 뜻을 숨기지 않았다. 물론 그것은 중국의 거센 도전을 막아내지 않고선 미국의 지위도 위태롭다는 위기의식의 산물이다. 이런 미국에 중국은 극도의 경계심을 나타내면서도 대결에는 대결로 맞서겠다는 결연한 태도를 보여왔다.

　바이든 대통령이 10일 시진핑 중국 주석과 7개월 만에 가진 통화에서 상호 대화와 소통을 통해 국제적 책임을 다하자는 데 원론적으로 공감한 것은 그나마 다행이다. 비록 입장차가 여전하지만 미중 정상의 9·11테러 20년 전날의 통화는 상징성이 크다. 테러와의 전쟁 20년이 됐지만 그 전쟁은 한층 더 어려워졌다. 미중이 협력해야 할 분야는 테러만이 아니다. 기후변화는 물론 북핵 등 비확산 문제 등 산적해 있다. 국제정치에서 치열한 경쟁은 불가피하다. 하지만 강대국 간 대결, 힘의 정치가 지배하면 스스로는 물론 국제사회에 재앙을 가져올 뿐이다. 경쟁하면서 협력해야 한다.

# 中 베이징대 교수, 시진핑 ‘공동부유’ 정면비판

중국 베이징대의 한 경제학과 교수가 최근 시진핑(習近平) 중국 국가주석이 주창하고 있는 ‘공동부유(共同富裕)’에 정면으로 반기를 들었다. 정부의 과도한 개입으로 ‘공동부유’가 ‘공동빈곤’이 될 수 있다는 것이다. 시 주석 집권 이후 반대 세력에 대한 대대적인 사정 작업과 감시·규제 확대로 정부 정책에 반대하는 목소리가 거의 사라진 가운데 나온 주장이어서 이목이 집중되고 있다.

　홍콩 사우스차이나모닝포스트(SCMP)는 4일 장웨이잉(張維迎·62·사진) 베이징대 경제학과 교수가 최근 공익성 민간학문기구인 ‘경제 50인 논단(CE50)’ 홈페이지에 올린 글을 통해 “시장의 힘에 대한 신뢰를 잃고 정부 개입에 자주 의존하면 공동빈곤으로 이어질 것”이라고 비판했다고 보도했다. 이어 그는 “기업가들이 부를 창출할 동기가 없다면 정부가 빈곤층에 줄 돈이 없어져 상류가 말라버린 강처럼 될 것”이라며 “계획경제는 빈곤층에 더 많은 복지를 제공하려 했지만 결과적으로 더 많은 빈곤층이 생겼다. 시장 지향적 개혁을 앞당기는 것만이 보다 공정한 사회를 만들 수 있는 유일한 길”이라고 강조했다.

　장 교수는 중국 시안에서 대학을 졸업하고 영국 옥스퍼드대에서 경제학 석·박사 학위를 받은 뒤 1994년부터 베이징대 교수로 재직 중이다. 2008년에는 베이징대 주요 싱크탱크인 국가발전연구원을 설립했다. 장 교수는 2018년 10월에도 “중국의 지난 40년 고성장은 시장화, 기업가 정신, 서구 300년의 기술 축적으로 이룬 것이지 이른바 ‘중국모델’ 때문은 아니다”라고 주장해 파문을 일으키기도 했다.

　현재 CE50 홈페이지에 게재됐던 장 교수의 글은 내려진 상태며 장 교수의 개인 위챗(중국판 카카오톡) 계정에서도 삭제된 상태다. 위챗에서 해당 글을 전송하는 것도 안 되고 있다.

# 바이든, 아프간 철군 다음날 “이제 中-러 등 새 위협 대처해야”

조 바이든 미국 대통령이 아프가니스탄 전쟁 종식을 선언하며 이제는 중국, 러시아 등 미국이 직면한 21세기의 위협에 대처할 때라고 강조했다.

　바이든 대통령은 미국이 아프간 철군을 완료한 지 하루 만인 지난달 31일(현지 시간) 백악관 대국민 연설에서 “세상이 바뀌고 있다”며 “우리는 중국과 심각한 경쟁 중이고, 러시아의 도전을 다루고 있으며, 사이버 공격과 핵 확산에 대응해야 한다”고 말했다. “미국이 또 다른 10년을 아프간의 수렁에 빠지는 것을 중국과 러시아만큼 좋아할 나라는 없을 것”이라며 “우리는 2021년을 위한 새로운 도전에 맞설 역량을 보여줘야 한다”고도 했다.

　바이든 대통령은 중동이 아닌 중국이라는 미국의 최대 위협에 집중할 필요성을 역설하며 “지난 20년간의 외교정책 페이지를 넘길 때”라고 강조했다. 연설에서 바이든 대통령은 미국이 ‘세계의 경찰’ 역할을 벗어던지고 국익을 바탕으로 현재와 미래의 외교안보 위협 대응에 집중하겠다는 외교정책 방향을 선명하게 드러냈다. ‘핵 확산’을 언급한 부분은 중국과 북한 등의 핵 위협이 커지는 상황을 감안한 것으로 보인다.

　이날 백악관은 북한의 영변 핵시설 재가동 움직임과 관련해 “(대북 접촉의) 문을 열어두고 있다”며 북한과의 대화 필요성을 재차 언급했다. 젠 사키 백악관 대변인은 브리핑에서 북한의 현재 핵 프로그램에 대한 평가 요청과 함께 ‘김정은과 접촉하려는 새로운 시도가 있었느냐’는 질문을 받고 “언제, 어디서나 전제조건 없이 만나겠다는 우리의 제안은 유지되고 있다”고 답변했다. 방미 중인 노규덕 한반도평화교섭본부장은 이날 워싱턴 특파원들과의 간담회에서 “한미는 양국이 공동으로 추진할 수 있는 대북 인도적 분야 협의를 하는 등 북한에 관여할 다양한 방안을 협의 중”이라며 “북한이 호응한다면 언제든 추진하도록 만반의 준비를 한다는 게 양국의 공통된 입장”이라고 했다.

# 中 허난성 물폭탄에 최소 33명 사망…전세계 아이폰 절반 공급 공장도 타격

중국 중부 허난성 성도(省都) 정저우에 60년 만의 기록적인 폭우가 내린 가운데 22일 현재 최소 33명이 사망하고 25만6000명이 대피했다. 300만 명이 넘는 이재민이 발생하는 등 최악의 홍수 사태에 대만의 차이잉원(蔡英文) 총통이 이례적으로 위로 메시지를 발표했다.

　22일 중국 허난성 당국에 따르면 이날 오전까지 확인된 사망자는 최소 33명으로 전날 25명보다 8명 증가했다. 지하철 침수로 정저우에서만 12명이 사망한 것을 포함해 각 지역에서 산사태 등으로 매몰됐던 사람들이 확인되면서 사망자가 늘었다. 현재까지 발생한 이재민은 300만4000명이고, 25만6000명이 긴급 대피했다. 허난성은 “직접적 경제적 손실만 12억2000만 위안(약 2239억6500만 원)”이라고 밝혔다. 허난성은 면적이 한국의 1.6배이며 인구는 1억 명 정도다.

　중국의 비 피해가 심각해지자 그동안 중국과 첨예한 대립각을 세워온 대만의 차이 총통은 자신의 명의로 된 위로 메시지를 발표했다. 이날 대만 중앙통신사에 따르면 차이 총통은 “불행히 숨진 사람과 그 가족들에게 애도를 표하고 재해 지역이 조기에 정상적인 생활로 돌아오기를 바란다”고 전했다.

　정저우에는 애플 아이폰을 위탁생산하는 대만 폭스콘 공장이 있어 아이폰 공급 차질도 예상된다. 폭스콘은 정저우에서 3개 공장을 운영하고 있으며 약 35만 명의 인력이 90개 생산 라인에 투입돼 있다. 이곳에서 생산되는 아이폰은 전 세계 아이폰 물량의 절반 이상을 차지한다. 월스트리트저널(WSJ)은 폭스콘 직원들을 인용해 “20일 오후 폭스콘 공장 세 곳 모두 수 시간 동안 전기가 끊기고 일시 정전됐다”고 보도했다. 한 직원은 공장 내 물이 허벅지까지 차오르면서 사람들이 생산 설비와 재고품들을 부랴부랴 옮겼다고 WSJ에 전했다.

# 文 만난 셔먼 “中과 대북정책 심도있게 논의할 것”

한국을 방문 중인 미국 국무부 ‘넘버2’인 웬디 셔먼 부장관이 22일 문재인 대통령과 만나 “중국을 방문해 대북정책과 관련한 심도 있는 논의를 할 것”이라고 밝혔다. 미중은 최근 경제 이슈와 별개로 북한 문제 등 외교 현안 등에만 초점을 맞춘 정례 대화 채널을 다시 가동하기 시작한 것으로 알려져 북-미 대화 재개를 둘러싸고 미중이 어떤 협의를 할지 주목된다. 셔먼 부장관은 한국에 이어 25, 26일 중국을 방문해 왕이 중국 외교부장과 회담한다.　

　문 대통령은 이날 청와대에서 셔먼 부장관을 접견하고 “앞으로 북-미 대화 재개를 위해 적극 노력해 달라”고 당부했다. 이에 셔먼 부장관은 “북한이 미국의 대화 제의에 조기 호응해 오기를 기대한다”며 “대북정책과 관련해 긴밀히 조율된 노력을 함께해 나가길 바란다”고 답했다.

　특히 셔먼 부장관은 “K팝 스타인 방탄소년단의 ‘퍼미션 투 댄스(Permission to Dance)’가 전 세계적으로 인기인데, 한국과 미국은 함께 호흡을 맞추었기 때문에 퍼미션(허가)이 필요 없다”고 말해 눈길을 끌었다. 공고한 한미동맹이 중요하다고 강조한 것으로 풀이된다.

　외교 소식통에 따르면 미중은 도널드 트럼프 미 행정부 때 사실상 단절됐던 정례적 외교 채널을 최근 재가동했다. 소식통은 “이 채널에선 미중 양국이 협력 필요성을 제기한 북한 문제 등 외교 사안 중심의 협의가 이뤄질 것으로 보인다”며 “미중이 경제와 외교를 별개로 보는 투트랙 접근에 나선 것”이라고 전했다.

# “中, 선양수용소 수감된 탈북자 50여명 北 보내”

중국 정부가 랴오닝성 선양 수용소에 수감 중이던 탈북자 50여 명을 14일 북한으로 보냈다고 자유아시아방송(RFA)이 16일 보도했다. 중국은 이들을 4월부터 북송하려 했는데 신종 코로나바이러스 감염증(코로나19) 환자 유입을 우려한 북한이 몇 차례 거부해 늦어진 것으로 알려졌다. 북송된 이들은 북한에서 극형을 면하기 어려울 것으로 보인다고 RFA는 전했다.

　중국 당국은 선양 수용소에 1, 2년가량 수감돼 있던 탈북자들을 14일 단둥 국경 세관을 거쳐 북한으로 보냈다. 보도에 따르면 버스 2대가 탈북자들을 나눠 실었고 공안 수십 명이 오전 일찍부터 세관 주변에서 경계를 서며 사람들이 북송 장면을 촬영하지 못하게 한 것으로 전해졌다.

　북송된 50여 명 중에는 북한군 병사와 공군 파일럿 출신도 있었다. 30대 탈북 여성은 중국인 남성과 결혼해 12세 아들을 뒀고, 중국에서 상당히 많은 돈을 벌었던 것으로 알려졌다. 한 소식통은 “이 여성은 두 번째 북송되는 것이어서 생사를 가늠할 길이 없다. 남편이 아내를 구하기 위해 뇌물을 쓰려 했지만 통하지 않았다”고 전했다. RFA에 따르면 선양 수용소에는 이날 북송된 50여 명 외에도 탈북자들이 더 남아 있다.

　코로나19 방역 등을 위해 닫혀 있던 단둥 세관이 이날 하루 개통하면서 그동안 북한에 머물고 있던 화교와 북한 무역대표부 관계자 등 98명도 중국으로 이동했다.

# ‘치사율 80%’ 원숭이B바이러스, 中서 첫 사망자

원숭이로부터 매우 드물게 옮는 바이러스 감염증 환자가 중국과 미국에서 잇따라 발생했다.

　17일 중국 관영 매체 글로벌타임스에 따르면 베이징의 영장류 연구기관에서 일하던 53세 수의사가 ‘원숭이 B 바이러스’에 감염돼 치료받다가 5월 27일 사망했다. 이 수의사는 올해 3월 죽은 원숭이 2마리를 해부하면서 이 바이러스에 감염됐고, 그로부터 한 달 뒤 메스꺼움과 구토 증세를 보인 것으로 조사됐다. 중국에서 사람이 이 바이러스에 감염된 것은 처음이라고 글로벌타임스는 전했다. 이 수의사와 접촉한 사람들 중 추가 감염자는 없다고 한다.

　원숭이 B 바이러스는 헤르페스 바이러스의 일종으로 감염된 원숭이에게 물리거나 긁힐 경우, 또는 원숭이의 분비물이 사람의 눈 점막 같은 곳에 튈 경우 전염될 수 있다. 사람에게서 사람으로도 감염될 수 있는 바이러스로 치사율이 70∼80%에 이른다.

　미국 텍사스주 북부 댈러스에서는 희소 감염병인 원숭이두창(Monkeypox) 바이러스 환자가 발생했다고 16일 미국 워싱턴포스트(WP)가 보도했다. 댈러스카운티 보건당국은 최근 아프리카 나이지리아를 방문한 댈러스 주민이 이 바이러스에 감염된 사실을 확인했다고 이날 밝혔다.

　이 환자는 이달 8일 나이지리아 라고스에서 미국 델타항공 비행기를 타고 애틀랜타를 경유해 9일 댈러스에 도착했다. 미 질병통제예방센터(CDC)가 비행기에서 환자와 접촉한 사람들을 파악하고 연락을 취하고 있다. 댈러스카운티 당국은 “신종 코로나바이러스 감염증(코로나19) 예방을 위해 마스크 착용이 요구됐기에, 이 바이러스가 비행기나 공항에서 비말을 통해 다른 사람들에게 확산했을 위험은 낮다”고 밝혔다.

　원숭이두창 바이러스는 우두 바이러스와 비슷한 종류로 1958년 처음 발견됐다. 사람이 감염되면 독감과 비슷한 증상을 보이면서 림프샘이 붓다가, 얼굴과 몸에 넓게 발진이 나타난다. 감염자는 100명 중 1명꼴로 사망한다고 CDC는 밝혔다. 인간 감염 사례는 1970년 콩고민주공화국에서 최초로 확인됐다. 미국에서는 2003년 아프리카 가나에서 수입된 원숭이에게서 감염이 시작돼 47명의 감염자가 나온 바 있다.

# 北과 밀착하는 中 “美, 수십년간 北위협 반성해야”

중국이 미국을 향해 “수십 년 동안 북한에 가한 위협과 압박을 반성해야 한다”며 이례적으로 강도 높게 비판했다.

　4일 중국 외교부에 따르면 왕이(王毅) 중국 외교 담당 국무위원 겸 외교부장(사진)은 전날 칭화대에서 열린 제9차 세계평화포럼에 참석해 “한반도 핵 문제는 최근 30년 동안 질질 끌면서 우여곡절을 반복했다”며 이처럼 주장했다. 그는 “(북한과) 대화와 협상을 통한 평화적 해결이 기본 원칙이고, 한반도 비핵화와 평화체제 구축을 병행하는 게 올바른 길”이라고 강조했다.

　왕 부장은 “한반도의 일은 중국 문 앞의 일”이라며 “중국은 한반도의 안정을 위해 일관되게 건설적인 역할을 할 것”이라고 말했다. 성 김 미국 국무부 특별대표가 북-미 대화 재개 가능성을 타진하기 위해 한국을 방문하는 등 최근 미국의 움직임에 대해선 “한반도 평화와 안정에 도움이 되는 모든 언행을 지지한다”고 밝혔다.

　그는 이날 연설에서 신장위구르와 홍콩의 인권 문제 등에 대한 서방의 비판에 대해 ‘중국 내정에 간섭하지 말라’는 입장을 다시 밝혔다. 대만에 대해서는 ‘분할할 수 없는 중국 영토의 일부’라고 전제한 뒤 “조국의 평화통일을 추진하는 것은 중국 정부가 견지해온 방침으로, 미국 일부 세력이 대만 독립 세력을 지원하는 것은 매우 잘못되고 위험한 것”이라고 경고했다. 왕 부장은 일본 정부의 후쿠시마 원전 오염수 해양 방류 결정에 대해 “일본 정부는 국제사회의 합리적인 관심을 충분히 경청하고 관련 국가와 국제기구의 협상 전에 함부로 태평양에 오염수를 방류해서는 안 된다”고 강조했다.

　칭화대가 운영하는 ‘세계평화포럼 사무국’이 주최하는 세계평화포럼은 2012년에 발족됐다. 중국에서 비정부기구가 주최하는 유일한 국제안보 관련 글로벌 포럼이다.

# 北 ‘코로나 국경봉쇄’ 1년만에 中과 교역 재개

북한이 북-중 접경지역을 중심으로 최근 중국과 제한적으로 물자 교류를 재개했다. 생필품 부족으로 불만이 쌓인 주민들을 달래기 위해 공식적으로는 국경 폐쇄 상태를 유지한 채 ‘비공식적인’ 교역에 나선 것으로 보인다. 북한은 그동안 신종 코로나바이러스 감염증(코로나19) 확산에 대한 공포로 1년 넘게 국경을 봉쇄해 왔다. 일각에선 이러한 교역 재개가 11일 북-중 우호협력조약 60주년을 앞두고 양국이 공조 체제를 조이는 신호라는 해석도 나온다.

　2일 정부 소식통에 따르면 북-중 무역 거점 도시인 중국 랴오닝(遼寧)성 단둥(丹東)시 등을 중심으로 지난달 말 일부 물자가 오간 것으로 전해졌다. 소식통에 따르면 교류는 트럭 등을 이용해 육로로 매우 제한적인 수준에서 이뤄졌다고 한다.

# 시진핑 “中 괴롭히면 머리 깨져 피 흘리게 될것”

시진핑(習近平·사진) 중국 국가주석이 1일 중국공산당 100주년을 맞아 “중화민족이 괴롭힘을 당하는 시대는 끝났다. 외부 세력이 우리를 괴롭히면 14억 인민의 피와 살로 만든 강철 만리장성에 머리가 깨져 피가 흐를 것”이라고 밝혔다. 그는 “대만과의 완전한 통일을 이끌어내는 것이 새 의무”라며 미국 등 서방이 대만, 홍콩 문제 등에 개입하면 강경 대응할 뜻을 천명했다. 1월 출범 후 내내 중국을 거세게 압박해온 조 바이든 미 행정부에 일종의 선전포고를 했다는 관측이 제기된다.

　시 주석은 이날 베이징 톈안먼(天安門)광장의 창당 100주년 경축대회에서 “중화민족은 세계에서 가장 위대한 민족으로 5000년이란 유구한 문명과 역사를 가지고 인류문명 발전에 불멸의 공헌을 했다”며 “누구도 주권과 영토를 보전하려는 중국의 굳은 결심과 확고한 의지, 강한 능력을 과소평가하면 안 된다”고 말했다. 그는 마오쩌둥(毛澤東)이 1949년 중화인민공화국 건국을 선포한 톈안먼 망루 위 연단에 마오와 똑같은 회색 중산복을 입고 등장해 ‘사회주의 현대화 강국 전면 건설’이란 제2의 100년 목표를 제시했다. 첫 번째 100년 목표였던 ‘샤오캉(小康·모든 국민이 편안하고 풍족한 생활을 누림)’ 사회를 실현한 만큼 이제 국제사회에서 패권국 위치를 강화하는 데 힘쓰겠다는 **뜻을 밝혔다.**

# 공산당 창당 100주년 앞둔 베이징 ‘반계엄 상태’

중국이 다음 달 1일 공산당 창당 100주년 기념일을 앞두고 사실상 반(半)계엄 상태에 돌입했다고 대만 쯔유(自由)시보가 23일 보도했다. 100주년을 기념하는 불꽃 축제와 각종 문화 공연을 성대하게 준비하면서도 인력, 물자 이동 등을 철저히 차단해 공산당과 시진핑 국가주석의 장기 집권에 대한 불만을 막겠다는 의도로 풀이된다.

　중국 당국은 21일부터 수도 베이징으로 들어가는 모든 택배에 대해 2단계 전수조사를 실시하고 있다. 우선 발신지 택배회사에서 베이징으로 보내는 모든 택배를 엑스레이 검사기로 검사한 후 보안검색 완료 표시를 붙인다. 이후 택배가 도착한 베이징 현지에서 다시 검사를 진행한다. 당국은 다음 달 1일까지 2단계 전수조사를 하겠다고 밝혔다.

　이로 인한 배달 지체를 우려하는 목소리도 높다. 온라인 쇼핑과 택배가 발달한 중국은 경제가 발달한 남부 광둥성, 상하이, 선전 등에서 생산된 물건이 택배를 통해 베이징으로 많이 들어온다. 과거에는 2, 3일이면 충분했지만 2단계 전수조사가 실시된 후 1주일 이상 걸릴 것이란 지적이 나온다. 일부 소비자들은 “온라인 주문을 통해 베이징 외곽에 있는 음식점에서 시내로 음식 배달을 시키는 것도 사실상 차단됐다”고 불만을 토로했다.

　베이징 공안당국은 지난주부터 시내 임대주택 등을 대상으로 가택 방문조사를 하고 있다. 각 파출소에서 파견한 검사원들이 직접 집을 방문해 당초 신고된 거주자와 실제 거주자가 동일한지 확인하고 있다. 특히 베이징에 거주하는 외국인에 대한 조사를 강화하는 것으로 알려졌다.

　이 외에도 베이징 등 9개 주요 대도시에서는 드론을 포함한 모형 항공기, 연, 풍선 등 모든 비행물체를 띄우는 것 또한 금지됐다.

　　‘중국의 암행어사’ 기관으로 불리는 공산당 중앙기율검사위원회는 최근 웹사이트를 통해 1930년대 공산당을 배반했던 사람들의 처참한 말로를 소개했다. 기율검사위원회는 “당을 배반하지 않겠다는 것이 맹세로만 끝나서는 안 된다”며 공산당에 대한 절대 충성을 압박했다. 혹시라도 발생할 수 있는 반(反)공산당 행위를 아예 뿌리 뽑겠다는 의지로 해석된다.

# 바이든, CIA 등에 “코로나 中실험실 기원 재조사하라”

신종 코로나바이러스 감염증(코로나19)의 기원에 대한 논란이 계속되는 가운데 조 바이든 미국 대통령이 미 정보당국의 판단이 엇갈린 상황이라며 추가 조사를 지시했다.

　바이든 대통령은 26일 성명에서 올 3월 코로나19가 동물과 인간의 접촉으로 시작됐는지, 실험실 사고로 발생했는지 등 기원을 분석하라고 중앙정보국(CIA) 등 정보기관에 지시했다고 밝혔다.

　바이든 대통령은 최근 이와 관련한 보고를 받은 결과 정보당국이 분명한 결론에 이르지 못했다고 설명했다. 정보기관 중 두 곳은 동물 기원설에, 한 곳은 실험실 유출설에 무게를 두고 있으나 모두 낮거나 중간 정도의 신뢰도만 있다고 밝혔다. 바이든 대통령은 분명한 결론에 가까워질 수 있도록 분석을 더 해 90일 이내에 다시 보고할 것을 정보당국에 지시했다.

　미국이 결론이 나지 않은 정보당국의 활동을 공개한 것은 이례적이다. 블룸버그는 미국이 중국 실험실 유출의 가능성을 배제하지 않고 있음을 보여주는 것이라고 분석했다. 바이든 대통령은 성명에서 “중국이 완전하고 투명하며 증거에 기반한 국제적 조사에 협력할 수 있도록 미국은 같은 생각을 가진 전 세계 동맹들과 함께하겠다”며 중국을 압박했다.

　중국은 이에 대해 미국이 코로나19 기원을 정치화시킨다며 반발했다. 주미 중국대사관은 바이든 대통령의 성명은 언급하지 않은 채 “모종의 정치 세력이 코로나19 대유행에 대항해야 하는 긴급한 필요는 무시하고 비난 게임에 몰두하고 있다”고 27일 홈페이지를 통해 비난했다.

　세계보건기구(WHO)는 코로나19가 중국 우한바이러스연구소에서 유출됐을 가능성이 낮다는 보고서를 내놨지만 최근 월스트리트저널이 우한바이러스연구소가 유출지일 수 있다는 의혹을 보도하면서 발원지를 둘러싼 논란이 커지고 있다.

# 中대사 “한국, 대만 언급 없었다면 좋았을것”

싱하이밍 주한 중국대사(사진)가 26일 한미 정상회담 공동성명에 대만해협과 남중국해 관련 내용이 “아예 없었다면 좋았을 것”이라고 말했다. 우리 정부가 대만 관련 언급이 “매우 원론적인 내용”이라며 진화에 나선 상황에서 대만 문제 거론 자체를 문제 삼고 나선 것. 싱 대사는 “중국과 미국이 화해하기 위해 한국을 비롯한 중간 나라들이 좋은 역할을 하면 좋겠다”면서 한국에 미중 간 균형외교를 요구하고 나섰다.　

　싱 대사는 이날 MBC 시사프로그램에 출연해 한미 성명에 “중국”이라는 표현이 빠진 데 대해 “(한국이) 많이 노력한다고 평가한다”면서도 “중국 단어가 나오지 않았지만 (중국을) 겨냥한 부분이 있는 것 같다”며 이같이 말했다. 그는 “한중이 수교할 때 이미 대만이 중국의 일부분이라고 명확히 인정했다. 남중국해 문제도 우리는 주변국과 협력해서 해결하면 된다고 생각한다”고 했다.

　또 쿼드(미국 중국 일본 인도 4자 협의체) 언급을 겨냥해 “하나의 나라, 몇 개의 나라가 만드는 질서에 대해 우리는 좀 다르게 생각한다”면서 “미국은 자꾸 그룹을 만들어 중국을 포위하려는 경향이 있는데 (한국이) 우리 입장을 많이 고려해주면 대단히 고맙겠다”고 했다. 한국의 쿼드 참여는 안 된다고 주장한 것.

　한미가 정상회담을 통해 안보뿐 아니라 경제·첨단기술 분야로 동맹을 확대한 데 대해서도 싱 대사는 경계심을 드러냈다. 그는 “중국은 미국처럼 (자국의) 기술을 다른 나라에 안 주는 식으로 통제한 적이 한 번도 없다”며 “(중국이) 10년 안에 22조 달러를 해외로부터 수입할 텐데 한국이 국익을 판단해 이런(중국) 시장을 활용해 경제 발전을 하는 게 맞다고 생각한다”고 했다.

　다만 이날 싱 대사의 발언은 한미 정상회담 이후 나온 중국의 반발 수위에서 더 나아가지 않았다. 정부가 “불장난하지 말라”는 중국의 불만에 “한중은 특수관계”로 진화에 나서고 있는 점을 고려해 일단 우리 정부의 행보를 지켜보기로 한 것으로 보인다. 특히 한국마저 등을 돌릴 경우 동맹국을 규합한 미국의 중국 견제를 막아내기 힘들다는 판단이 작용한 것으로 알려졌다. 싱 대사는 시진핑 주석 방한 계획에 대해서는 “현 상태에서는 확실하게 말할 게 없다”고 했다.

# 두달만 뛰면 되는 中택한 김연경, 다음 목표는 美? 유럽?

‘배구 여제’ 김연경(34·사진)의 중국행은 ‘예측 가능한 미래’였다. 그러나 그 다음 행선지가 어디가 될지는 여전히 예측 불가능한 상태다.

　20일 김연경의 에이전시 업무를 맡고 있는 라이언엣에 따르면 김연경은 상하이 연고 중국 리그 팀 광밍유베이와 계약 협상 마무리 단계를 밟고 있다. 예상 계약 기간은 한 시즌이다.

　배구 이적 시장에 밝은 관계자는 “베이징자동차에서도 김연경에게 관심을 보였다. 그러나 김연경이 2017∼2018 시즌 몸담았던 친정 팀을 더 선호한 것으로 알고 있다”며 “김연경도 이제 30대 중반으로 중국 리그는 일정이 짧아 체력 부담이 작고 다음 무대를 준비하기가 쉽다”고 말했다.

　김연경은 4년 전 중국 리그로 진출할 때도 “경기 수가 많으면 체력적으로 부담스럽다”는 뜻을 밝혔다. 광밍유베이는 지난 시즌 12경기밖에 치르지 않았다. 반면 김연경은 지난해 제천·MG새마을금고컵 대회부터 V리그 챔피언결정전까지 총 41경기를 뛰었다. 게다가 이재영-이다영 쌍둥이 자매가 학교 폭력 사태로 전력에서 이탈하면서 심리적 부담까지 안은 채 경기에 나서야 했다.

　아직 2021∼2022시즌 중국 리그 일정은 나오지 않은 상태지만 새 시즌 역시 길어도 두 달 안에 모든 일정이 마무리될 가능성이 높다. 김연경은 중국 리그 일정을 소화한 뒤 내년 1월 초에 다시 유럽 진출을 시도할 수 있다. 올해 프로 리그가 막을 올린 미국 무대 진출도 가능하다.

　중국 리그가 끝난 뒤 흥국생명으로 돌아오는 것도 전혀 불가능한 일만은 아니다. 김연경은 해외 리그 팀과 계약할 때는 자유계약선수(FA) 신분이지만 국내에서는 흥국생명에서 다음 시즌 일정의 40%(14경기) 이상을 소화해야 FA 자격을 얻는다.

# 홍콩, 대만공관 일방폐쇄… 교류 끊나

# 홍콩이 대만에서 외교 공관 역할을 해 왔던 경제무역문화판사처를 일방적으로 폐쇄했다. 이번 결정은 중국과 대만 관계가 악화하고 있는 가운데 나온 것이어서 중국 정부의 영향을 받은 홍콩이 사실상 대만과 교류 중단을 염두에 둔 것 아니냐는 분석도 나온다. 　19일 홍콩 사우스차이나모닝포스트(SCMP) 등에 따르면 홍콩 정부는 전날 인터넷 홈페이지를 통해 주(駐)대만 경제무역문화판사처 운영을 잠정 중단한다고 밝혔다. 중단 사유와 운영 재개 시점 등에 대해서는 아무런 설명이 없었다. 이에 대해 대만의 대중국 담당 부처인 대륙위원회는 “홍콩 정부가 일방적 결정을 내렸다”며 “깊은 유감을 표명한다”고 밝혔다. 　주대만 경제무역문화판사처는 홍콩과 대만 사이의 경제·무역 교류를 촉진하기 위해 2011년 문을 열었다. 대만도 홍콩에 같은 역할을 하는 경제문화판사처를 두고 있다. 이들 기관은 공식적인 정부 기구는 아니지만 교민 보호 등 실질적인 영사관 역할을 수행해 왔다. 　홍콩과 대만이 이런 상주 기구를 운영하는 것과 달리 중국과 대만은 상대 지역에 상주 기구가 아예 없다. 홍콩이 중국과 다른 정책을 펼 수 있었던 것은 시진핑(習近平) 중국 국가주석 집권 이전까지는 일국양제(一國兩制·한 국가 두 체제) 원칙이 비교적 잘 지켜졌기 때문이다. 하지만 2013년 시 주석 집권 이후 ‘양제’보다 ‘일국’ 원칙이 더 강조되면서 상황이 달라지기 시작했다. 특히 지난해에는 홍콩 국가보안법이 도입됐고 올해는 홍콩 선거제까지 개편되면서 홍콩의 중국화 현상이 뚜렷해지고 있다.　 　‘中, 달 이어 화성도 터치다운…‘우주굴기’ 전세계 과시

중국이 화성 탐사용 무인이동 로봇(로버)을 화성 표면에 성공적으로 착륙시키면서 미국과 옛 소련에 이어 세 번째로 화성 표면에 탐사선을 착륙시킨 국가가 됐다. 화성 표면 탐사를 위해 이동하는 로버를 보낸 것은 미국에 이어 두 번째다.

　중국 신화통신에 따르면 화성 주변 궤도를 돌던 중국 탐사선 톈원(天問) 1호에서 분리된 화성 탐사 로버 ‘주룽(祝融)’이 15일 오전 8시 18분(한국 시간) 화성 북반구 유토피아 평원 남쪽에 성공적으로 내려앉았다. 시진핑 국가주석은 축전을 보내 “중국의 행성 탐사 장정에 중요한 한 걸음”이라며 “지구와 달 사이에서 이제는 행성 간 탐사로의 도약을 이뤄냈다”고 밝혔다.

　지난해 7월 발사된 톈원 1호는 2월 10일 화성 궤도에 안착한 뒤 3개월 가까이 궤도를 돌며 착륙의 기회를 엿봤다. 톈원은 ‘하늘에 묻는다’라는 뜻으로 중국 전국시대 초나라 시인 굴원의 시에서 따왔다. 주룽은 중국 고대 신화에 나오는 불의 신 이름이다.

　이날 착륙에 성공한 주룽은 크기가 가로 2.6m, 세로 3m, 높이 1.85m, 무게 240kg인 바퀴 6개짜리 이동형 로봇이다. 약 90 화성일(1 화성일은 지구로 따지면 24시간 37분) 동안 탐사를 하도록 설계됐다. 주룽에는 화성 탐사 로버 최초로 지하 100m까지 탐사할 수 있는 레이더 장비가 장착됐다. 중국은 이번 탐사에서 화성 표면에서 물과 얼음의 흔적을 찾고 토양과 암석 성분을 분석할 예정이다. 중국은 두 번의 도전 끝에 화성 탐사에 성공했다. 2011년 러시아와 함께 화성 탐사를 시도했지만 실패한 바 있다. 시 주석은 “용감한 도전이 중국을 행성 탐사 분야에서 선진국 반열에 오르게 했다”고 치하했다.

중국은 최근 수년 새 화성 탐사와 달 탐사, 독자 우주정거장 건설 추진 등 ‘우주굴기’에 속도를 내며 미국을 위협하는 우주 강국으로 떠올랐다. 2019년 달 뒷면에 인류 최초로 탐사선 ‘창어 4호’를 착륙시킨 데 이어 지난해 12월 또 다른 탐사선 창어 5호를 달에 보내 월면토를 채취해 지구로 돌아왔다.

　지난달에는 중국 독자 우주정거장 ‘톈허(天和)’를 구성할 첫 구조물을 자국 발사체 ‘창정 5B호’에 실어 우주로 보냈다. 2024년 임무를 종료하는 국제우주정거장(ISS)이 폐쇄되면 톈허는 유일한 우주정거장이 된다.

　2016년엔 미국에 앞서 세계 첫 양자통신위성 ‘모쯔(墨子)호’를 쏘아 올려 2400km 거리에서 무선 양자암호통신을 시현하는 데 성공했다.

　미국은 중국의 우주 진출을 경계하고 있다. 이달 9일 창정 5B호가 통제력을 잃고 지구에 낙하하며 잔해 일부가 인도양에 떨어진 것과 관련해 미국은 “중국이 우주 파편에 관해 책임감 있는 국제기준을 충족하지 못하고 있다”며 빌 넬슨 미국 항공우주국(NASA) 국장 명의의 비판 성명을 내기도 했다. 외신들은 이러한 갈등 이면에 우주 패권을 둔 경쟁의식이 있다고 해설했다

# 中 22t 우주로켓 잔해 주말 지구 낙하 가능성

중국의 로켓 ‘창정(長征)5B호’의 잔해가 주말경 지구 대기권에 진입할 것으로 보인다. 한미 군 당국은 추락 예측지에 한반도가 포함되지는 않지만 유사시를 대비해 상호 공조 체계를 강화하기로 했다.

　공군 우주정보상황실은 7일 미 우주사령부 연합우주작전센터와 화상회의를 갖고 8, 9일경 지구 대기권에 진입할 것으로 예상되는 로켓 잔해 경로에 대한 감시정보를 지속 공유하기로 했다. 창정5B호 크기를 고려하면 대기권에서도 잔해가 소멸되지 않고 추락할 것으로 예측된다. 일단 한미 군 당국은 잔해가 대서양에 추락할 것으로 보고 있으나, 대기권 진입 시 잔해가 본궤도를 이탈할 가능성도 배제하지 않는 것으로 알려졌다.

　중국은 지난달 29일 독자 우주정거장 구축을 위해 발사한 핵심 모듈인 ‘톈허(天和)’를 정상궤도에 안착시켰지만, 이를 실어 나른 창정5B호 로켓이 통제 불능 상태에 빠져 22.5t에 달하는 잔해가 280km 상공에서 매일 1∼2km가량 지구로 낙하하는 중이다.

　군 당국은 일단 미 우주사령부의 감시자산을 활용한 잔해 궤도 정보를 공유 받기로 했다. 이와 함께 잔해가 한반도 상공으로 향하는 최악의 상황을 고려해 전력화를 진행 중인 ‘전자광학위성감시체계’나 ‘고출력레이저위성추적체계’ 등을 활용할 방침인 것으로 알려졌다.

# 블링컨 “中 점점 더 공격적 행동…세계 지배국 되려 해”

토니 블링컨 미국 국무장관은 2일(현지 시간) “중국이 점점 더 억압적이고 공격적으로 행동하고 있다”고 비판했다. 미국의 대중국 정책 핵심은 이런 중국에 맞서 규칙에 기반한 국제사회의 질서를 지키는 것이라며 동맹들과의 협력을 재차 강조했다.

　블링컨 장관은 이날 CBS방송 시사 프로그램 ‘60분’과의 인터뷰에서 “우리는 중국이 국내에서 더 억압적으로, 해외에서도 더 공격적으로 행동하는 것을 봐왔다”고 했다. 중국은 규칙에 기초를 둔 국제질서에 도전하거나 이를 약화시킬 군사적, 경제적, 외교적 역량을 갖춘 나라라는 것이다. ‘중국의 목표가 무엇인 것 같으냐’는 질문에는 “중국은 자신들이 전 세계를 지배하는 국가가 될 수 있고, 돼야 한다고 믿는 것 같다”고 답변했다. 다만 그는 진행자가 ‘미국이 중국과 군사적 대치 상황으로 나아가고 있느냐’고 묻자 “그런 상황에 도달하거나 그런 방향으로 가는 것조차 미국과 중국 모두의 이익에 심하게 반하는 일”이라고 선을 그었다.

　블링컨 장관은 “조 바이든 대통령이 지식재산권 탈취를 포함해 중국이 취해 온 행동들에 대해 실제로 우려하고 있다”고도 전했다. 신장위구르족에 대한 중국 당국의 인권침해를 ‘집단학살(genocide)’이라고 부르며 중국의 인권 문제도 직접 겨냥했다. 그는 “우리는 중국을 다루지 않아도 되는 여유가 없다”며 “적대적인 부분이든, 경쟁적이거나 협력적인 부분이든 (중국과의) 관계는 정말로 복잡한 측면들이 있다”고 했다.

　그는 진행자가 중국의 국내총생산(GDP)이 2028년에 미국을 추월하게 될 것이라는 전망을 언급하자 “부국을 만드는 핵심은 인적 자원, 또 그 인적 자원의 잠재력을 극대화하는 국가의 능력에 달려 있다”며 “그런 점에서 우리는 (중국보다) 훨씬 더 좋은 위치에 있다”고 말했다. 또 같은 가치관을 공유하는 동맹 및 파트너 국가들과 함께 협력할 것이라는 점도 재확인했다.

# “신의주역 가림막 철거…” 北中국경 봉쇄 해제 징후

북-중 교역이 이르면 이달 말 재개될 것으로 알려진 가운데 북한 신의주역에서 열차 가림막이 철거되는 등 북-중 간 국경 봉쇄 해제 징후가 속속 포착되고 있다.

　24일 미국의 소리(VOA)에 따르면 위성사진 분석 결과 지난해 10월 북한 신의주역 일대에 설치됐던 열차 가림막이 지난달 31일 돌연 철거됐다. 민간 위성사진업체 맥사테크놀로지의 지난달 16일자 위성사진에서는 신의주역에 설치된 약 400m 길이의 터널 형태 가림막이 확인됐다. 하지만 이달 사진에서는 이 가림막이 사라진 상태였다. 가림막은 장기간 세워둔 열차를 눈이나 비 등으로부터 보호하는 목적으로 설치됐던 것으로 추정된다.

　북한 신의주와 마주하고 있는 북-중 무역 거점 도시인 중국 랴오닝(遼寧)성 단둥(丹東)시와 인근 북-중 접경지 역에는 북한으로 보낼 대규모 물자가 대기 중인 것으로 알려졌다. 단둥시 기차역에선 평양 외곽 지역인 ‘서포’ 지명이 적힌 화물열차가 물자를 실은 채 세워져 있는 모습이 목격됐다.

　선박을 통한 북-중 간 교역은 지난달 재개됐다. 중국 해관총서에 따르면 지난달 북한은 중국으로부터 비료 등 1297만 달러 상당의 물자를 수입했다. 북-중 관계에 밝은 대북 소식통은 25일 “지난달 중국에서 북한으로 들어간 물자 대부분은 선박을 통해 들어갔다”고 말했다. 이르면 27일부터 대북 지원용 비료를 실은 열차가 신의주로 들어갈 것으로 알려졌다.

# 美 ‘대북제재 완전한 이행’ 밝힌 날, 中은 한국에 ‘제재 완화 노력’ 촉구

한미일 3국 안보실장들이 “유엔 안전보장이사회 대북 제재 결의의 완전한 이행”을 강조했다고 미국이 밝힌 날 중국은 한국에 “북한의 합리적 안보 우려 해결을 위해 노력해야 한다”고 요구했다. 중국은 ‘북한의 합리적 안보 우려’를 북한 체제 보장과 대북 제재 완화 등을 가리키는 말로 써 왔다.

　미 백악관은 2일(현지 시간) 메릴랜드 아나폴리스 해군사관학교에서 열린 한미일 3국 안보실장 회의 뒤 낸 공동 언론성명에서 “3국 안보실장들은 북한을 비롯해 국제사회가 유엔 안보리 결의를 완전히 이행하는 것이 긴요하다는 데 동의했다”며 “핵 확산 방지와 한반도에서 (대북) 억지 강화 및 평화·안정 유지를 위해 협력해 나가기로 했다”고 강조했다. “북한의 핵 및 탄도미사일 프로그램에 대한 우려를 공유하고, 비핵화를 위한 한미일 3자 간 조율된 협력을 통해 이 문제들을 해결하려는 의지를 재확인했다”고도 밝혔다. 대북 억지를 강조하면서 북한과 중국에 대북 제재 준수를 압박한 것.

　서훈 청와대 국가안보실장은 회의 뒤 기자들에게 “한미일은 북핵 문제의 시급성과 외교적 해결의 필요성에 대해 공감했고 북-미 협상의 조기 재개를 위한 노력이 계속돼야 한다는 데 뜻을 같이했다”고 했다. 하지만 한미일 3국 조율을 통해 백악관이 밝힌 성명에 북-미 협상의 조속한 재개는 포함되지 않았다.

　반면 왕이(王毅) 중국 외교부장은 3일 중국 푸젠(福建)성 샤먼(廈門)에서 열린 한중 외교장관 회담에서 정의용 외교부 장관에게 “한반도 평화와 안정을 수호하고 북한의 합리적 안보 우려를 확실히 해결해야 한다”며 “각 측이 이를 위해 적극적으로 노력해야 한다”고 했다. 　왕 부장은 또 “중국은 한국과 5세대(5G) 이동통신, 반도체 집적회로 등 분야의 협력을 중점적으로 강화하고 질 높은 협력 파트너가 되기를 원한다”고도 했다. 반도체를 중국 견제를 위한 국가안보 이슈로 다루기 시작한 미국은 한미일 안보실장 회의에서 3국의 반도체 공급망 유지를 주요하게 논의한 것으로 알려졌다.

# 현대건설기계, 中진출 최대 2500억원 수주

현대건설기계가 중국에서 역대 최대 규모의 수주를 했다.

　현대건설기계는 최근 중국 고객사들로부터 건설장비 2200여 대를 수주했다고 29일 밝혔다. 현대건설기계가 중국에서 사업을 벌인 이후 가장 큰 규모의 수주다. 5.5∼85t급 굴착기와 휠로더 등으로 금액은 2500억 원에 달한다. 지난해 현대건설기계의 중국 수주 규모가 7800여 대였던 걸 감안하면 지난해 연간 실적의 30%에 달한다. 현대건설기계는 이번에 수주한 장비들을 5월까지 공급할 예정이다.

　최근 중국 시장은 지난해 신종 코로나바이러스 감염증(코로나19)으로 인한 경기침체를 극복하기 위해 중국 정부 차원의 대대적인 사회간접자본(SOC) 투자가 이뤄지고 있다. 현대건설기계는 중국 건설장비 시장이 호황기에 들어선 걸로 판단하고 이달 초 대형 굴착기, 수륙양용 굴착기, 파일해머, 니퍼 등 신제품 13종을 중국 시장에 선보였다.

　철광석 가격이 1t당 170달러(약 20만 원)에 육박하는 등 원자재 가격이 강세를 띠면서 중국을 비롯한 신흥시장에서의 건설장비 수요 증가도 기대된다. 현대건설기계는 올해 중국의 건설장비 수요가 지난해보다 8% 늘어난 31만5000여 대에 이를 것으로 보고 있다.

# 중국, 자국 백신 접종 외국인 비자 발급 간소화 하기로

중국 정부가 중국산 코로나19 백신을 맞은 뒤 홍콩을 통해 입국하는 외국인에 대해 핵산검사 증명서 제출을 면제하는 등 비자 발급 절차를 간소화하기로 했다.　

　14일 중국 관영 글로벌타임스에 따르면 중국 외교부 홍콩 주재 사무소는 12일 홈페이지를 통해 중국 백신을 2회 접종하거나 비자 신청 14일 전에 1회 맞은 사람은 중국 비자를 신청할 때 별도의 핵산검사 증명서와 건강 및 여행기록 증명서를 제출하지 않아도 된다고 밝혔다.

　중국은 현재 외국인이 중국에 입국할 때 72시간 이내 코로나19 핵산검사 음성 증명서 등을 제출하도록 하고 있다. 장례식 참석이나 친척 방문 등 인도적 목적으로 비자를 신청하는 경우도 중국산 백신을 맞았다면 간소화 대상으로 분류해 비자를 발급하기로 했다. 이 조치는 15일부터 시행된다.

　글로벌타임스는 “중국산 백신 접종자에 대해 핵산검사 음성 증명서 제출을 생략하도록 한 것은 중국산 백신에 대한 자신감을 보여주는 것”이라고 설명했지만 중국산 백신 접종자에 한해서만 비자 발급을 간소화하는 이번 조치는 중국산 백신의 보급을 확대하기 위한 것이란 분석이 많다. 중국 입국을 위한 수요가 상당하다는 점에서 이들의 중국산 백신 접종을 늘리기 위한 조치라는 것이다.

　다만 중국 내에서도 중국산 백신에 대한 불신으로 백신 접종 인구가 2월 말 현재 전체 인구의 3% 안팎에 그쳐 이번 조치의 실효성에 의문을 제기하는 시각도 있다.

# 바이든 정부 첫 안보지침 “동맹과 단합해 중에 대응”

조 바이든 미국 행정부가 3일(현지 시간) 중국을 집중 겨냥하는 내용이 담긴 ‘잠정적 안보전략 지침’을 발표했다. 바이든 행정부 취임 이후 40여 일 만에 나온 것으로 향후 미국의 외교안보 정책의 방향과 청사진을 담고 있다.

　앤서니 블링컨 국무장관은 이날 백악관의 지침 발표에 앞서 “중국은 21세기의 가장 큰 지정학적 시험”이라며 “중국은 안정적이고 개방된 국제시스템에 도전할 경제적, 외교적, 군사적, 기술적 힘을 가진 유일한 국가”라고 했다. 또 “중국은 우리가 원하는 방식으로 전 세계가 작동하도록 하는 모든 규범과 가치에 도전이 되고 있다”고 밝혔다.

　블링컨 장관은 “우리가 빠져나간 자리를 중국이 채웠다”며 동맹 및 파트너 국가들과의 협력 강화로 이에 대응하겠다고 강조했다. 동맹들을 ‘힘을 배가시키는 미국의 독특한 자산’이라고 부른 뒤 “우리의 단합된 무게는 중국이 이를 무시하지 못하게 만든다”고 역설했다.

# 시진핑 “회색코뿔소 - 블랙스완에 대비하라” 黨에 지시

시진핑(習近平) 중국 국가주석이 ‘회색코뿔소’와 ‘블랙스완’을 언급하며 중국이 직면한 위험을 강조했다.

　중국 관영 중국중앙(CC)TV에 따르면 시 주석은 지난달 28일 공산당 중앙정치국 집단학습에서 “각종 위험과 도전을 잘 예측해야 하며 각종 회색코뿔소와 블랙스완 사건에 잘 대비해야 한다”고 말했다. 회색코뿔소는 예상할 수 있지만 간과하기 쉬운 위험을, 블랙스완은 발생할 확률은 낮지만 한 번 일어나면 큰 충격을 주는 위험을 의미한다.

　시 주석은 또 “세계적으로 100년간 전례 없는 대변화가 일어나는 가운데 복잡한 국제 정세의 영향을 깊이 인식하고 중국의 발전에 유리한 환경을 조성해야 한다”고 주문했다. 이 같은 발언은 미국 조 바이든 행정부의 출범 이후에도 양국 대결 구도는 계속될 것이라는 전망이 제기되는 가운데 나왔다. 도널드 트럼프 행정부 때 악화된 미중 갈등은 현재 무역을 넘어 외교·안보 분야까지 확대된 상태다. 지난달 28일 미국의 유력 정치전문 매체 폴리티코에는 “중국 공산당 지도부의 균열을 통해 시 주석 교체를 도모해야 한다”는 내용의 전직 미국 고위당국자 익명 기고문까지 실렸다.

　시 주석은 위기를 강조할 때마다 회색코뿔소와 블랙스완을 언급하고 있다. 신종 코로나바이러스 감염증(코로나19)이 확산하던 지난해 2월엔 인도네시아 대통령, 말레이시아 총리 등과 전화통화를 하면서 “방역에 있어 회색코뿔소나 블랙스완을 어떻게 막을 수 있을지는 전 세계의 고민”이라고 했다. 또 중국이 28년 만에 가장 낮은 경제성장률을 발표하던 2019년 1월에도 “국제 정세가 예측하기 어렵고 주변 환경은 복잡하고 민감하다. 블랙스완을 고도로 경계하고 회색코뿔소도 예방해야 한다”고 강조했다. 시 주석은 특히 회색코뿔소나 블랙스완 같은 위험을 막는 일을 전쟁에 비유하기도 했다.

　시 주석은 이번 정치국 학습에서 올해는 14차 5개년 계획의 첫해이자 공산당 창당 100년을 맞는 해로 경제사회 발전을 보장하는 것이 매우 중요하다고도 했다.

# 시진핑 ‘파카’ 입었더니… 관련 의류회사 주가 10% 껑충

시진핑(習近平) 중국 국가주석이 18일 2022 베이징 겨울올림픽 공식 후원사인 안타(安踏)그룹 산하기업의 방수코트(파카)를 입고 등장하자 하루 뒤 홍콩 증시에 상장된 안타그룹 주가가 약 10% 상승했다.

　홍콩 사우스차이나모닝포스트(SCMP)에 따르면 시 주석은 18일 올림픽 준비 상황을 점검하기 위해 베이징 외곽의 겨울올림픽 시설을 방문하면서 짙은 푸른색의 ‘아크테릭스’ 파카를 입었다. 원래 핀란드기업 아메르스포츠에 속한 브랜드였는데 2019년 ‘중국의 나이키’로 불리는 안타그룹에 인수됐다. 아크테릭스 제품은 온라인 매장에서 보통 700∼2000달러(약 80만∼220만 원)에 판매된다. 시 주석이 입은 제품의 정확한 가격은 알려지지 않았다.

　안타는 중국 스포츠용품 시장에서 미국 나이키(시장 점유율 23%), 독일 아디다스(20%)에 이어 점유율 15%로 3위를 차지하고 있다. 시 주석은 2017년에도 안타 재킷을 입고 등장했다. 2018년 평창 겨울올림픽에 등장한 중국 대표팀도 안타 제품을 유니폼으로 착용했다. 시 주석이 자국 의류회사를 지원하기 위해 일부러 이 모습을 공개했다는 분석이 제기된다. 실제 19일 안타그룹 주가는 10% 가까이 상승했다.

# 미국은 왜 중국의 일대일로 경계하나

　‘아시아인들은 생각할 수 있는가(Can Asians Think)?’

　이 도발적인 문장은 1993년 키쇼어 마부바니가 쓴 책의 제목이다. 싱가포르 리콴유공공정책대학원장인 마부바니는 당시 책을 통해 세계의 흐름이 변하고 있으며, 아시아가 서양에 가르칠 것이 더 많다고 경고했다. 아시아인이 생각할 수 있느냐는 식민주의적 사고가 아닌, 아시아인이 어떤 생각을 하는지를 연구할 시점이라면서 말이다.

　20여 년이 지나 달라진 아시아의 위상은 경제 수치로도 입증되고 있다. 지난해 11월 한중일과 호주 등 15개국이 서명한 ‘역내포괄적경제동반자협정(RCEP)’은 인구 22억 명, 국내총생산(GDP) 규모 총 26조2000억 달러로 ‘세계 최대의 자유무역협정(FTA)’이라고 불렸다. 저자는 앞으로 세계 지형도는 ‘아메리카 퍼스트’가 아닌 ‘아시아 퍼스트’가 될 것이라 단언한다. 그러면서 잘 알려지지 않은 아시아의 여러 단면을 자세히 소개한다.

　첫 출발은 고대 아시아 문명이다. 그리스 문명 등 서구 중심으로 쓰인 세계사의 그늘에 가려진 인도와 서아시아, 동아시아 문명을 간략하게 정리한다. 그 뒤 아시아의 주요 정치, 경제, 사회, 문화에 관한 기본적인 지식과 사건을 제시한다. 호주와 러시아는 왜 일찍부터 ‘아시아화’에 뛰어들었는지, 미국은 왜 중국의 일대일로 프로젝트를 경계하는지 등 시의성 있는 주제도 다룬다.

　　‘아시아의 관점에서 지난 20년은 조지 W 부시의 무능력, 버락 오바마의 무성의, 도널드 트럼프의 예측 불가능성의 시대’라거나, ‘서양의 오해와 달리 아시아는 중국 중심을 향해 움직이고 있지 않다’는 관점이 흥미롭다. 동아시아를 넘어 인도와 동남아시아 등 광활한 아시아의 현주소를 훑어볼 수 있다는 것도 장점이다. 다만 아시아에 무지한 서구인을 독자로 설정하고 있다는 한계도 느껴진다.

　인도에서 태어난 저자는 미국과 유럽에서 국제 관계 전문가로 활동하고 현재는 싱가포르에 정착했다. 원제는 ‘The Future is Asian’.

# “달 표면 샘플 44년만에 가져왔다” 中들썩

　“14억 중국 국민 전체가 들썩이고 있다.”

　중국 무인 달 탐사선 ‘창어(嫦娥) 5호’가 달 표면 샘플을 채취해 17일 새벽 무사히 귀환했다. 달 샘플 채취는 1976년 옛 소련의 ‘루나 24’ 로봇 탐사 이후 44년 만. 중국 주요 매체들은 일제히 “중국이 우주 강국의 대열에 들어섰다”며 자축하고 나섰다.

　중국 관영 신화통신은 이날 “중국이 달 샘플 채취에 성공하면서 미국과 옛 소련 이후 세 번째 달 탐사에 성공한 국가가 됐다”면서 “세 번째이긴 하지만 창어 5호는 기존 탐사와는 다른 점들이 많아 ‘세계 최초’라는 타이틀이 많다”고 강조했다.　

　신화통신에 따르면 창어 5호는 지금까지 인류가 가본 적 없는 용암 평원인 ‘폭풍우의 바다’에 착륙했다. 이 지역 암석과 토양은 다른 지역에 비해 비교적 최근에 생성돼 달의 화산 활동 연구에 도움이 될 것이라는 기대가 있다. 중국 관영 글로벌타임스는 “지난해 1월 창어 4호가 인류 최초로 달 뒷면에 착륙한 데 이어 창어 5호 역시 달의 새로운 지역을 처음으로 탐사하면서 ‘최초’ 타이틀을 이어가게 됐다”고 전했다.

　채취한 샘플 무게가 2kg에 이르는 것도 새로운 기록이다. 옛 소련의 루나 24가 채취한 330g의 약 6배에 달하는 양이다. 창어 5호가 다량의 샘플을 채취할 수 있었던 것은 지구로 돌아올 때 이용할 연료를 크게 줄였기 때문이다. 과거 미국과 옛 소련의 경우 달착륙선이 자체 보유한 연료를 이용해 지구로 귀환했다. 하지만 창어 5호는 달 궤도에서 귀환선과 도킹하는 방식을 이용했기 때문에 상대적으로 적은 연료만으로도 귀환이 가능했다.

　신화통신은 “달 궤도에서 창어 5호와 귀환선의 도킹 작업은 최고의 우주 기술”이라면서 “고속으로 이동하는 우주선 간 안전한 도킹을 위해 연구진이 661번 연습을 진행했다”고 전했다.

　중국은 올해 7월 화성탐사선 ‘톈원(天問) 1호’를 쏘아 올렸고, 이번에 창어 5호가 임무를 마치고 귀환하는 등 ‘우주 굴기’ 계획을 착착 실행하고 있다. 중국은 지난해 27차례 로켓 발사를 통해 66기의 비행체를 우주로 보냈고, 올해에도 40기 이상의 로켓을 우주로 쏘아 올렸다. 내년에도 최소 80기 이상의 로켓을 우주로 발사할 계획이다.　

　시진핑(習近平) 중국 국가주석은 이날 창어 5호의 무사 귀환을 자축하며 프로젝트에 참여한 연구진에게 당·정·군 수장의 명의로 축전을 보냈다. 시 주석은 “이번 프로젝트를 통해 중국의 항공우주 기술에 큰 진전을 이뤘다”고 말했다.

# 中관영언론 “김치-파오차이 완전히 다른 음식”

최근 중국 매체가 ‘김치 종주국’ 논란을 촉발시켜 비판을 받은 가운데 중국 관영 언론이 “김치와 파오차이(泡菜)는 완전히 다른 음식”이란 입장을 내놨다. 이번 논란을 ‘단순 번역 오류’라고 설명하면서 한발 물러서는 모양새다.

　중국 관영 영자지 글로벌타임스는 9일(현지 시간) “김치와 파오차이를 둘러싼 논란은 번역 오류로 인한 ‘시시한 소동’에 불과하다”며 두 음식의 차이점을 설명했다. “두 음식 모두 중국어로 ‘파오차이’라 불리지만 만드는 방식과 재료는 전혀 다르다”라면서 “발효 음식인 김치는 한국요리를 대표하는 반면 파오차이는 쓰촨성의 절임 채소에서 유래했다”고 전했다. 이 신문은 기사에서 김치를 ‘Kimchi’라고 명확히 표기했는데, 기존의 중국 매체들은 김치라는 표현 자체를 쓰지 않고 파오차이로 통칭해왔다.

　글로벌타임스는 또 서경덕 성신여대 교수의 요구로 중국 포털 바이두 백과사전이 8일 ‘한국 김치는 중국에서 유래했다’는 부분을 삭제했다며 “중국 전문가들은 이를(김치 기원 논란) ‘시시한 소동’이라고 보고 있다”고 설명하기도 했다. 이어 “바이두 이용자라면 누구나 등록·편집·수정할 수 있는 (백과사전) 시스템 때문에 벌어진 해프닝”이라고 주장했다. 그러면서도 “단순 번역 오류를 한국의 김치문화 옹호자들이 ‘(중국이) 우리 문화를 훔치려 한다’고 비판하면서 불화가 시작됐다”며 한국 측에 논란의 책임을 떠넘겼다.

　이번 김치 논란을 촉발시켰던 중국 관영 환추시보도 이날 “김치(Kimchi)는 파오차이와는 다른 음식”이라고 전했다.

　김치 종주국을 둘러싼 논란은 지난달 29일 환추시보가 중국이 절임 채소인 파오차이 제조법을 국제표준화기구(ISO)에 등록한 것을 두고 “중국의 파오차이 산업이 국제 시장에서 기준이 됐다”며 “사실 한국이 파오차이 종주국이라는 주장은 이미 유명무실하다”고 주장하면서 시작됐다. 한국의 김치와 중국의 파오차이는 엄연히 다른 음식임에도 양국이 김치의 표준규격을 놓고 신경전을 벌이는 듯한 오해를 부른 것. 이후 논란이 커졌지만 중국은 별다른 입장을 내놓지 않다가 9일 만에 ’번역 실수‘라고 한 것.

　조정은 세계김치연구소 전략기획본부장은 “이번 상황은 일단락됐지만 중국의 ‘김치공정’은 늘 대비해야 한다”며 “한국을 대표하는 음식 문화유산인 김치를 지켜내기 위해선 전략적 접근이 필요하다”고 강조했다.

# “北, 中에 올해 4000억원대 석탄 밀수출"

유엔 제재로 석탄 수출이 금지된 북한이 올 1∼9월 중국에 최대 4억1000만 달러(약 4452억 원)어치의 석탄을 수출한 것으로 분석됐다. 북한 선박은 인공기를 버젓이 달고 운항하기도 해 대북 제재에 구멍이 크다는 지적이 나온다.

　월스트리트저널(WSJ)은 미 국무부 관료 인터뷰와 국무부가 제공한 위성사진을 토대로 “북한 선적 선박들이 지난 1년 동안 중국 닝보-저우산 지역으로 수백 차례 석탄을 직접 실어 날랐다”고 7일(현지 시간) 보도했다. 구체적으로 8월 12일 촬영된 위성사진에는 인공기를 단 석탄 운반선 4척이 중국 닝보-저우산항 인근 해역에 중국 선박과 함께 정박한 것이 포착돼 석탄 불법 환적을 한 것으로 분석됐다.

　북한은 올해 들어 9월까지 410만 t의 석탄을 수출한 것으로 미 정부는 파악하고 있다. 1t당 80∼100달러에 팔렸다고 가정할 때 수출액은 3억3000만∼4억1000만 달러(약 3583억∼4452억 원)에 이른다. 미국을 비롯한 국제사회는 북한의 불법 환적에 대한 공동 감시 태세에 나선 상황이지만 북한 선박은 인공기까지 달고 운항했다고 WSJ는 전했다. 그동안 외국 국적 선박을 동원하거나 선박의 명칭을 바꾸고, 선박위치식별장치(AIS)를 끄는 등 회피 수법을 써왔지만 이젠 대놓고 불법 환적에 나서고 있는 것.

　미국 측은 중국이 유엔 제재를 무시하기 때문에 벌어지는 일이라고 비판한다. 미 국무부 고위 관리는 “북한에서 중국으로 (석탄을) 직접 운송하는 건 2017년 유엔 안보리의 제재 채택 이후 처음 목격하는 큰 변화”라며 “중국과 북한이 더 이상 제재 감시를 피하기 위해 위장하려 애쓰지 않고 있다”고 말했다.

# 美의회, 中견제 구상에 국방예산 2조원 책정

미국 의회가 2021회계연도 국방예산안을 담은 국방수권법(NDAA) 법안에 ‘태평양억지구상(Pacific Deterrence Initiative)’ 항목을 신설하고 22억 달러(약 2조3800억 원)를 배정했다. 사실상 중국을 겨냥한 국방예산을 신설하면서 내년 1월 조 바이든 행정부 출범 이후에도 미국의 대중 강경정책이 이어질 것임을 예고했다.

　6일(현지 시간) 워싱턴포스트(WP)에 따르면 상하원이 내놓은 NDAA 법안 중 인도태평양 지역에 관한 부분에 태평양억지구상 항목이 추가됐다. 법안에는 국방장관이 역내 미군 주둔 병력의 현대화 및 강화 방안이 담긴 계획을 수립하고, 인도태평양사령부와 협의를 거쳐 이 내용이 담긴 보고서를 2021년 2월 15일까지 의회에 제출하도록 돼 있다. 법안은 “이 구상은 인도태평양 지역에서 미국의 억지력과 국방력, 준비태세를 강화하고 역내 동맹 및 파트너 국가들을 확신시키기 위해 우선시되는 활동들을 수행하기 위한 것”이라고 명시했다.

　이 법안이 시행되면 미국이 인도태평양에서의 억지력을 높이기 위해 주한미군을 감축하지 않고 유지할 가능성이 높다. 한편으로는 미국이 한국을 향해 반중(反中) 연합전선에 참여하라는 압박이 심해질 수 있다는 분석도 나온다.

# 中, 4년만에 한국게임 서비스 허가...한한령 풀리나 촉각

중국 정부가 약 4년 만에 처음으로 한국 게임에 ‘판호(版號·중국 내 게임 서비스 허가)’를 내줬다. 한국 게임에 대한 ‘한한령(限韓令·한류 제한령)’이 풀리는 신호탄이라는 기대와 일회성에 그칠 것이라는 부정적 전망이 동시에 나온다.

　3일 게임사 컴투스에 따르면 2일 중국 국가신문출판서는 홈페이지에 컴투스의 모바일 역할수행게임(RPG)인 ‘서머너즈 워: 천공의 아레나’에 대해 판호를 발급했다고 공지했다. 2014년 6월 전 세계에 선보인 서머너즈 워는 컴투스의 해외 매출 중 약 80%를 차지하는 인기 게임이다.

　컴투스는 2016년 말 중국 시장에 서머너즈 워를 선보이기 위해 판호 발급을 신청했다. 하지만 2017년 사드(THAAD·고고도미사일방어체계) 배치를 놓고 한중 간 갈등이 확대되면서 중국은 비공식적으로 한국산 게임 등 문화 콘텐츠에 대한 유통을 제한해왔다. 특히 게임의 경우 2017년 초를 마지막으로 판호를 발급해주지 않았다. 컴투스 관계자는 “오래전에 신청했던 건인 만큼 전혀 예상하지 못했고, 사전 연락도 없었다”고 말했다.

　중국 정부의 깜짝 판호 발급에 국내 게임업계에서는 세계 최대 게임 시장 중 하나인 중국 시장의 문이 다시 열리는 것 아니냐는 기대가 나오고 있다. 중국 시장 진출을 앞두고 있는 넥슨의 모바일게임 던전앤파이터를 비롯해 과거 중국에서 인기를 얻었던 지식재산권(IP)을 활용한 게임들의 수출이 조만간 이루어질 수 있다는 것이다. 이 같은 기대감에 3일 국내 주식시장에서 컴투스가 전날 대비 6.19% 상승 마감한 것을 비롯해 위메이드(5.75%), 펄어비스(14.11%), 넷마블(3.59%), 엔씨소프트(2.21%) 등 게임사들의 주가가 강세를 보였다.

　다만 이번 조치로 중국 시장이 다시 열렸다고 판단하기는 이르다는 신중한 반응도 나오고 있다. 중국이 한한령을 전면 철폐했다고 확신하기 위해서는 추가 판호 발급이 이어져야 한다는 것이다. 위정현 한국게임학회장은 “중국이 판호를 줄줄이 내줄 것이라고 기대하기는 어렵다”며 “다만 이번 판호 발급을 지렛대로 삼아 정부와 업계 등이 치열한 외교적, 전략적 노력을 해야 한다”고 지적했다.

# BBC “중국 김치가 세계 표준?...중 언론 오보”

중국이 절임 채소인 파오차이 제조법을 국제표준화기구(ISO)에 등록한 것을 놓고 중국 관영매체가 김치 국제표준을 제정한 것처럼 보도한 것에 대해 영국 BBC가 ‘오보(false report)’라며 중국의 주장을 지적했다.

　BBC는 지난달 30일(현지 시간) ‘김치, 한중 문화 갈등을 발효하다’는 제목의 기사에서 “중국이 한국 전통 음식인 김치 제조법을 국제적으로 인정받았다는 오보에 한국이 반박하고 나섰다”며 “(김치 논란은) 한국과 중국 간 가장 최근에 벌어진 문화 분쟁”이라고 전했다.

　앞서 지난달 29일 중국 관영매체 환추시보는 파오차이 국제표준을 제정했다고 보도하면서 “중국의 파오차이 산업이 국제 시장에서 기준이 됐다”면서 “사실 한국이 ‘파오차이 종주국’이라는 주장은 이미 유명무실하다”고 주장했다. 파오차이와 김치를 같은 음식인 것처럼 표현하고, 파오차이를 국제표준으로 등록한 것에 한국 김치까지 포함되는 것처럼 선전한 것이다.

　이에 한국 농림축산식품부는 즉각 2001년 유엔 국제식량농업기구 산하 국제식품규격위원회(CODEX)의 규격에 따라 김치는 국제규격으로 설정됐으며, 파오차이는 김치로 해석할 수 없다고 반박한 바 있다.

　BBC도 농식품부의 주장을 인용하면서 김치의 특성과 김장문화를 소개했다. “매운 염장 음식인 김치는 중국에서 파오차이라는 이름으로 공급되고 있기는 하지만 같은 이름의 중국 고유 음식이 있다”며 “ISO 문서에는 이번 식품 규격이 ‘김치에는 적용되지 않는다’고 적시돼 있는데도 일부 중국 언론은 이와 다르게 보도했다”고 지적했다.

　이어 “김치는 채소를 소금에 절인 뒤 양념과 발효된 해산물을 넣고 항아리에 보관한다”며 “매년 김치를 만드는 김장은 유네스코 인류무형문화유산에 등재돼 있다”고 소개했다. 또 “한국은 김치 수요가 많아 중국에서 수입하지만 한국은 중국의 엄격한 규제로 수출길이 사실상 막혔다”고 덧붙였다.

　BBC는 최근 이어진 한국과 중국 간 문화 갈등 사례도 소개했다. 10월 방탄소년단(BTS)이 6·25전쟁 70주년을 맞아 한미 관계 발전에 기여한 공로로 밴플리트상을 받으며 “(한미) 양국이 함께 겪었던 고난의 역사”라는 표현을 쓰자 중국 누리꾼들이 “중국의 희생을 무시했다”며 집단으로 반발했다. 11월에는 중국 배우 쉬카이(許凱)가 중국 소셜미디어 웨이보에 ‘한복은 중국 의상’이라는 취지의 글을 올려 논란이 일었다.

# 왕이가 띄운 ‘한중일FTA’, 아직은 외교•경제 리스크 크다

최근 한국과 일본을 순방한 왕이 중국 외교부장이 ‘한중일 자유무역협정(FTA)’ 추진을 주장했다. 왕 부장은 강경화 외교부 장관과의 회담에서 “한중일FTA를 적극 추진해보자”고 했고 일본 외무상과의 회담에서도 이를 강조했다. 중국 환추시보는 “한중일 FTA는 동북아 지역협력 제도의 부족함을 보충하는데 효과적”이라며 분위기 띄우기에 나섰다. 한중일FTA는 기대 효과와 함께 경제적 외교적 리스크도 큰 만큼 신중하게 접근해야 한다.

　한중일 FTA는 2013년부터 협상이 시작됐으나 핵심 분야에 대한 이견과 외교 갈등으로 협상이 부진했다. 세계 경제의 24%를 차지하는 세 나라가 상품과 서비스 장벽을 없애면 경제성장을 촉진하는 효과가 생길 수 있으나 그만큼 위험도 크다. 세계적인 제조업 강국인 3국은 반도체 자동차 철강 등 핵심 수출 산업에서 겹치는 부분이 많다. 한중 FTA가 2015년 발효됐는데 그 이상의 개방은 우리 산업에 타격을 줄 우려가 있다. 한국과 일본도 자국 산업 보호를 위해 서로 개방을 꺼리는 분야가 많다. 한중일 FTA가 그동안 여러 차례 논의됐으나 더 이상 진전되지 못한 것도 이 때문이다.　

　정치 외교적인 측면에서의 함의도 빼놓을 수 없다. 미국의 정권 교체를 앞두고 중국이 지역협력과 경제통상 문제를 꺼내드는 것은 동북아지역에서 미국의 영향력을 견제하려는 의도가 깔려 있다. 그동안 한중일FTA 협상은 한일이 과거사 문제로 갈등하고 중국이 한국의 고고도미사일방어체계(THAAD)를 트집 잡아 중단되는 등 정치 외교적 영향을 크게 받았다.

　미국은 조 바이든 대통령 당선인이 ‘민주주의 정상회의’ 개최를 추진하면서 중국 견제에 나섰다. 바이든 당선인은 대선 캠페인 과정에서 “세계 민주 국가들이 모여 민주주의 체제를 강화하고, (민주주의에) 역행하는 국가들에 맞서자”고 했는데 최근 본격화 움직임을 보이고 있다. 미중 양쪽에서 끌어당기는 힘이 커지면서 한국의 ‘전략적 모호성’은 또다시 시험대에 올라섰다. 미중의 패권 경쟁에서 등터지는 새우가 되지 않으려면 될지, 전략적 활용으로 국익을 높일 지는 정부 하기에 달렸다.

# 귀국 한국인 직원 감염… SK하이닉스 中공장 가동중단

SK하이닉스 중국 충칭(重慶) 공장에 파견을 나갔던 한국 직원이 신종 코로나바이러스 감염증(코로나19) 확진 판정을 받아 중국 현지 공장 가동이 중단됐다. 중국 충칭시 정부는 해당 공장에 근무하는 전 직원 2700여 명의 코로나19 전수 조사를 명령했다.

　29일 중국 관영 신화통신 및 재계에 따르면 SK하이닉스 직원 A 씨는 약 1년 6개월 동안의 중국 근무를 마치고 인천공항으로 돌아와 받은 코로나19 검사에서 28일 양성 판정을 받았다. A 씨가 확진 판정을 받은 직후 충칭시는 A 씨가 근무했던 SK하이닉스 공장에 대한 역학 조사를 진행하고, 현장 통제 및 소독 등 긴급 조치를 시행하는 것으로 알려졌다.

　재계 관계자는 “충칭시 및 SK하이닉스 등은 A 씨가 특별한 증상이 없었던 무증상 감염자로 추정하고 있다”라며 “충칭시는 A 씨가 묵었던 호텔 역시 운영을 중단하고 25일부터 이 호텔에 묵었던 투숙객들을 대상으로 핵산 검사를 진행하고 있다”고 말했다.

　가동이 중단된 SK하이닉스 충칭 공장은 반도체 패키징(포장) 등 후공정 작업을 하는 곳이다. 일반적으로 반도체 생산 공장은 잠시라도 전력 공급이 끊기면 생산 라인을 복구하기까지 수일이 걸리고, 피해액도 크다. 반면 이번에 가동이 중단된 패키징 공장은 시 정부의 방역 조치가 완료되면 곧바로 정상 가동이 가능할 것으로 보인다. 하지만 현재로서는 가동 재개 시점이 불투명해 공장 가동 중단 장기화 시 생산 차질을 피하긴 어려울 것으로 전망된다.　

　SK하이닉스 측은 이날 “중국 정부에 적극 협조해 빠른 시일 안에 조업이 재개될 수 있도록 최선을 다하겠다”고 밝혔다.

# “이소룡, 여전히 우리 가슴속에 있다” 탄생 80주년 행사 잇따라

홍콩의 전설적 액션스타 이소룡(리샤오룽·李小龍·1940∼1973·사진)의 탄생 80주년을 맞아 중화권에서 그를 기리는 행사가 잇따르고 있다.

　28일 중국 관영 영자지 글로벌타임스에 따르면 이소룡 부친의 고향이자 이소룡이 유년 시절 잠시 거주한 광둥성 포산에서는 1940년 11월생인 이소룡을 기려 이달 초부터 ‘이소룡 탄생 80주년’ 행사가 열리고 있다. 닮은 사람 찾기 콘테스트, 이소룡의 삶을 회고하는 온·오프라인 행사 등이 잇따른다. 포산 당국은 이런 행사를 통해 중국 무술이 아닌 전 세계의 무술문화 중심지로 발돋움하겠다는 속내를 드러내고 있다.

　홍콩 우체국 또한 ‘세계 무술 속 이소룡의 유산’을 주제로 특별우표 발행에 나섰다. 홍콩 센트럴 지역을 관통하는 일부 트램에도 이소룡의 탄생 80주년을 기념하는 광고가 전면에 실렸다. 이 트램은 내년 1월까지 운영될 예정이다.

　웨이보 등 중국 소셜미디어에서도 여러 누리꾼이 이소룡 동영상과 사진을 공유하며 그의 탄신일을 기렸다. 글로벌타임스는 “이소룡은 많은 사람에게 쿵푸스타 이상이었다”면서 “중국인에 대한 서구의 고정 관념을 깼고 여전히 많은 사람의 가슴 속에 있다”고 평가했다.　딸 섀넌 리(李香凝·51)는 부친의 영어 이름 ‘브루스 리’를 딴 웹사이트 ‘브루스닷컴’에 글을 올려 “아버지는 세상을 떠났지만 그의 삶은 계속해서 우리에게 새로운 영감을 주고 있다”고 회고했다.

　이소룡은 미국 샌프란시스코에서 태어나 생후 3개월 때 홍콩으로 이주했다. 성년이 된 후 23편의 영화에 출연하며 1970, 80년대 전 세계 남성들에게 대중문화의 아이콘으로 떠올랐다. 불과 33세에 요절한 점도 세계적 팬덤 현상을 부채질했다.

# 미국 편에 서지 말라 압박하며 한한령은 풀지 않는 중오만

왕이 중국 외교부장이 어제 2박3일의 방한을 마치고 귀국했다. 왕 부장은 짧은 일정에도 한국 대통령부터 국회의장, 여권 실세까지 두루 만났다. 한중 외교장관회담에 25분이나 지각하는 결례도 범했다. 그의 방한을 계기로 양국 간엔 폭넓은 협력 방안을 논의됐다지만 중국의 한한령(限韓令·한류 금지령) 해제 같은 핵심 현안에서는 아무런 진전이 없었다.

　왕 부장 방한 목적은 미국의 정권 교체기를 맞아 주변국 분위기 탐색과 외교적 관리를 위한 것 그 이상도 이하도 아니었던 듯하다. 중국 측은 양국 간 합의라며 10개 항을 열거했지만 거기에 핵심 의제는 없었다. 왕 부장은 시진핑 주석 방한에 코로나19 완전 통제라는 조건을 내세웠다. 사드(THAAD·고고도미사일방어체계) 문제는 “적절히 처리해야 한다”며 철수를 압박했고, 그 보복 조치인 한한령 해제 요청에는 “지속적 소통을 희망한다”고만 했다.

　중국은 그간 미국 트럼프 행정부의 전방위 공세에 시달려왔다. 조 바이든 시대에도 그런 미-중 긴장은 계속될 것이라는 관측이 지배적이다. 중국으로선 미국의 동맹인 한국·일본이 반중(反中)전선에 서지 않도록 주변국 외교에 부쩍 공을 들이고 있다. 이번에도 새삼 한중일 경제통합을 강조하며, 미국의 중국 정보기술·기업 퇴출에 맞선 ‘글로벌 데이터안보 이니셔티브’에 동참해줄 것을 요청했다.

　바이든 시대의 미중 관계는 아직 예단하기 어렵다. 트럼프 대통령은 예외지만 선거 때 중국을 맹비난했던 미국 대통령들은 당선 후엔 모두 중국과의 통상 확대에 치중했다. 규범과 가치를 앞세운 바이든식 국제주의는 트럼프 시절의 무역전쟁 같은 거친 방식이 아닌, 치밀한 제도적 포위망으로 중국을 옥죌 가능성이 높다. ‘민주주의 정상회의’ 같은 가치연대 구상은 한국을 더는 피하기 어려운 선택의 기로에 서게 만들 수 있다.

　왕 부장은 “이 세계에 미국만 있는 게 아니다”고 했다. 한국을 향해 미국 편에 서지 말라는 압박일 것이다. 동아시아에도 중국만 있는 게 아니다. 자국의 거대시장 접근을 제멋대로 막고 풀며 치졸한 외교를 계속하는 한 중국은 어디에도 설 자리가 없을 것이다. 중국에서 누구든 방한할 때마다 ‘황제 칙사처럼 구느냐’는 얘기가 왜 나오는지 중국은 알아야 한다.

# 한국 온 中왕이 “세계에 미국만 있는 게 아니다”

방한 중인 왕이(王毅) 중국 국무위원 겸 외교부장이 26일 “세계에 미국만 있는 게 아니다. 190여 국가가 있고 모두 독립 자주 국가다. 한국과 중국도 그렇다”고 밝혔다. 한중 협력이 미국의 영향을 받아서는 안 된다는 점을 강조한 것으로 풀이된다. 시진핑(習近平) 중국 국가주석의 방한에 대해서는 신종 코로나바이러스 감염증(코로나19)이 “완전히 통제돼야 한다”고 밝혀 사실상 한국 정부가 추진해 온 연내 방한이 무산됐음을 시사했다.

　왕 부장은 이날 오전 외교부 청사에서 강경화 외교부 장관과 회담을 마친 뒤 기자들과 만나 ‘이번 방한이 한국 정부와 여권 인사들에게 미국 편에 서지 말라는 미중 경쟁 차원인가’라는 질문에 이같이 말하면서 “한중은 이웃 국가이고 친척처럼 자주 오가야 한다”고 말했다. 왕 부장은 “우리(한중)는 전략적 협력 동반자 관계이니 전방위로 조율하고 협력해야 한다”고도 했다. 그는 강 장관과의 회담 모두 발언에서도 “국제, 지역 문제에 대해 전략적 소통을 할 생각이 있다”고 밝혔다. 강 장관에게 한국에 배치된 사드(THAAD·고고도미사일방어체계)에 대한 우려도 표시했다고 외교부 당국자가 전했다.

　왕 부장은 ‘시 주석의 연내 방한 가능성이 얼마나 되느냐’는 질문에는 취재진을 가리키며 “다들 마스크를 쓰고 있다”며 “중요한 것은 (코로나19를) 완전히 통제하는 것”이라고 말했다. 한국에서 코로나19가 재확산되고 있어 시 주석 방한이 어렵다고 밝힌 것이다.

# 다시 찾아온 중국발 미세먼지 재앙... ‘저자세’ 외교로는 해결 요원하다

코로나19 사태 와중에 일시 진정되는 듯 했던 미세먼지가 다시 기승을 부리고 있다. 그제 서울에 올 가을 들어 첫 초미세먼지주의보가 발령됐으며 수도권과 충청권 등 중서부 지역은 12일부터 나흘 연속으로 초미세먼지 농도가 ‘나쁨’ 수준을 이어갔다. 충남지역은 14, 16일 이틀간 비상저감조치가 발령됐으며, 어제도 광주전남과 부산경남을 제외한 전국 대부분이 ‘나쁨’ 수준으로 공기가 나빴다.

　우리나라 미세먼지는 고농도 시 최대 80%가 중국에서 유입될 정도로 중국의 영향이 압도적이다. 최근 특히 심해진 것도 편서풍을 타고 중국에서 날아온 대기오염물질이 국내에서 대기정체로 쌓였기 때문이라는 게 환경당국의 분석이다. 코로나19 사태로 떨어졌던 중국의 공장가동률이 100% 가까이 회복된 데다 겨울철 난방이 시작되면서 중국에서 유입되는 오염물질의 총량이 다시 늘어난 탓이다. 올 들어 9월까지 중국 전역의 미세먼지 농도가 평균 11.8% 감소하는 등 중국 대기질이 일부 개선됐다고는 하지만 코로나19 사태로 인한 착시현상이었을 뿐 중국발 미세먼지 재앙은 여전히 한국인의 건강과 일상을 망가뜨리고 있다.

　지난해 봄 한반도를 덮친 최악의 미세먼지 사태를 겪으며 중국발 미세먼지 해결이 국가적인 당면 과제로 떠오르자 문재인 정부도 뭔가 근본적인 대책을 강구하는 듯 싶었다. 하지만 그후 2년 가까이 지나도록 뭐하나 진전을 이룬게 없다. 양국 환경장관회의와 실무자 협의를 1년에 한두 번 열어 대응책을 논의하고 있다지만 여기서 나오는 대책이라는게 정보교류나 학술연구 수준을 넘지 못하고 있다.

　문재인 대통령은 지난해 미세먼지 사태로 민심이 들끓자 반기문 전 UN사무총장을 위원장으로 하는 범국가적 대책기구인 국가기후환경회의를 출범시켰지만 그후엔 별다른 진전이 없다. 중국은 여전히 “서울 미세먼지는 현지에서 배출된 것”이라며 자국의 책임을 인정하지 않는데도 우리 정부는 공식 항의조차 못한채 미온적으로 임하고 있다.

　물론 중국의 산업·환경정책이 획기적으로 바뀌지 않는한 중국발 대기오염물질의 내습을 근본적으로 차단할 방책은 없을 것이다. 하지만 중국이 동부 연안 공장들의 오염물질 배출 기준을 대폭 강화하고 배출총량 저감을 위한 과감한 투자에 나선다면 현재 한국을 덮치고 있는 미세먼지의 상당량이 줄어들 것이다. 미세먼지 사태는 국민들이 숨을 쉴 수 있냐 없냐는 생존의 문제인 만큼 저자세 대응을 버리고 중국에 근본적인 미세먼지 저감 대책 마련을 강력하게 촉구해야 한다.

# 文대통령, 中주도 RCEP 가입 서명

문재인 대통령이 15일 세계 최대 규모의 다자 자유무역협정(FTA)인 역내포괄적경제동반자협정(RCEP)에 최종 서명했다.

　문 대통령은 이날 청와대에서 화상으로 열린 ‘제4차 RCEP 정상회의’에서 RCEP에 서명하며 “코로나(신종 코로나바이러스 감염증)의 도전과 보호무역 확산, 다자체제의 위기 앞에서 젊고 역동적인 아세안이 중심이 돼 세계 최대 규모의 자유무역협정을 체결하게 됐다. 우리는 자유무역 가치 수호를 행동으로 옮겼다”고 말했다. RCEP는 한중일과 아세안 10개국, 호주, 뉴질랜드 등 15개국이 참여하는 세계 최대 규모의 FTA이다. 무역 규모, 국내총생산(GDP), 인구 측면에서 전 세계 약 30%를 차지한다. 가맹국 사이에서 관세 문턱을 낮추고 체계적인 무역·투자 시스템을 확립해 교역을 활성화하자는 것이 기본 취지다.

　일각에선 사실상 중국이 주도하는 RCEP 가입을 계기로 미중 갈등 속 한국 정부의 외교적 셈법이 더욱 복잡해질 것이라는 관측이 나온다. 버락 오바마 전 미 대통령이 중국의 팽창을 막기 위해 2010년부터 환태평양경제동반자협정(TPP)을 추진하자 중국은 이 포위망을 뚫기 위해 2012년부터 RCEP 구축에 나서며 한국 참여를 종용해 왔다. TPP는 2017년 다자 체제를 거부한 트럼프 대통령이 탈퇴를 선언하며 좌초됐지만 2018년 일본 호주가 주축이 돼 CPTPP로 이름을 바꿔 발효됐다. 조 바이든 미 대통령 당선인은 후보 시절부터 CPTPP 복귀를 시사해 왔다. 이에 청와대 관계자는 “중국은 다른 나라와 마찬가지로 RCEP에 참여한 15개국 중 하나”라면서도 “필요하다고 느끼면 우리도 CPTPP에 들어갈 수 있다”고 했다.

# 블랙핑크 판다 맨손 터치 논란

맹목적 애국주의를 앞세워 K팝 그룹 방탄소년단(BTS)을 공격했던 중국 누리꾼과 관영매체가 이번에는 걸그룹 블랙핑크에 대한 공격에 나섰다. 이달 3일 중국 희귀동물 판다와 접촉한 동영상을 자체 유튜브 등에 공개한 블랙핑크가 자신들이 원하는 수준만큼 판다를 귀중하게 다루지 않았다고 주장했다.

　관영 영자지 글로벌타임스는 6일 “블랙핑크 멤버들이 한국 에버랜드에 있는 생후 약 3개월의 새끼 판다 ‘푸바오’, 2016년 한국에 온 ‘화니’를 접촉할 때 짙은 화장을 했다. 때때로 장갑과 마스크도 끼지 않았다”고 주장했다. 이어 “새끼 판다는 면역력이 약해 짙은 화장을 하거나 방역 장비를 착용하지 않으면 위험할 수 있다”며 “판다는 중국의 ‘국보’이고 해외에서 태어나더라도 일정한 시기가 되면 중국으로 돌아와야 하므로 중국의 소유”라고 덧붙였다.

　일부 누리꾼은 웨이보에 “한국에 있는 판다를 회수하자”는 글을 올렸다. 전 세계 동물원의 판다는 모두 중국이 임대해 주는 형태다.

　중국야생동물보호협회는 5일 성명에서 “한국 아이돌 멤버가 국보 판다를 장갑도 끼지 않은 채 만진 사태를 묵과할 수 없다”고 주장했다. 에버랜드와 블랙핑크 측에 각각 판다에 대한 불법 접촉 행위를 즉시 멈추고, 해당 장면이 나온 동영상을 삭제하라고 공식 항의했다고도 덧붙였다.

　같은 날 에버랜드는 블랙핑크의 영상 예고편을 인스타그램에 게재하며 “본 촬영은 담당 수의사와 사육사의 감독하에 철저한 소독과 방역 후 진행됐다”고 공지했다. 중국이 반발하자 이 영상을 삭제했다.

# 현대차 “2025년까지 중에 수소트럭 3000대 보급”

현대자동차가 중국에서 수소전기트럭 보급에 나서며 중국 수소경제 사업 확대에 속도를 낸다.

　현대차는 중국 수도 베이징이 있는 ‘징진지(京津冀)’ 지역과 경제 도시 상하이가 있는 ‘창장강(長江·양쯔강) 삼각주’ 지역의 기업들과 수소경제 생태계 구축을 위한 업무협약(MOU)을 연이어 체결했다고 4일 밝혔다.

　우선 현대차는 지난달 27일 상하이전력고분유한공사, 상하이순화신에너지시스템유한공사, 상하이융화전과융자리스유한공사 등 창장강 삼각주 지역 내 주요 에너지 및 금융회사와 MOU를 맺었다. 수소충전소 및 수소생산설비 구축, 수소전기차 운영을 위한 금융 서비스를 추진해 2025년까지 이 지역에 현대차의 수소전기트럭 3000대 이상을 보급하는 게 목표다.

　이어 4일에는 중국강연집단 안타이과기고분유한공사 및 허강집단 허베이철강공업기술복무유한공사와 MOU를 체결해 징진지 지역에서 수소충전소 구축과 수소전기트럭 시범운행, 2025년까지 수소전기트럭 1000여 대 보급 추진에 뜻을 모았다. 이인철 현대차 상용사업본부장(부사장)은 “중국 시장에 수소 차량 판매뿐만 아니라, 수소차 리스, 충전소 운영 등 수소 생태계 전반에 걸친 사업 생태계를 구축할 계획”이라고 말했다. 현대차는 스위스에서도 초기 수소전기트럭 구매 비용에 부담을 느끼는 물류업체들의 부담을 덜고자 사용료를 받고 차를 대여하는 형태로 수소트럭 보급에 나선 바 있다.

　중국은 수소전기차에 쓰이는 수소의 원료인 ‘부생수소’(석유화학 공정의 부산물)가 풍부하고, 지속적인 산업 기반 확충으로 트럭 수요가 꾸준한 시장으로 꼽힌다, 수소전기트럭 시장 성장 잠재력이 충분한 것이다. 중앙정부는 물론 지방정부 차원에서도 수소전기차 보급 확대, 수소 생산과 충전에 필요한 기반 구축에 속도를 내고 있기도 하다.

　2030년까지 수소전기차 100만 대 보급을 추진 중인 정부 목표에 발맞춰 지난달 현대차도 2030년까지 중국에 수소전기트럭 2만7000대 이상을 수출하겠다는 목표를 제시했다. 같은 기간 북미와 유럽으로의 각 1만2000대, 2만5000대 수출 목표를 능가한다.

# 두산인프라, 中서 굴착기 생산 20만대 돌파

두산인프라코어가 중국 내 굴착기 누적생산 20만 대를 돌파했다고 3일 밝혔다. 1994년 10월 중국 진출 이후 26년 만에 달성한 기록이다.

　중국 옌타이에 공장을 가지고 있는 두산인프라코어는 2001년 누적생산 5000대를 넘어섰다. 당시 중국 시장은 일본의 건설기계 업체들이 선점하고 있었지만, 공격적인 마케팅과 최신 제품 출시로 2000년대 이후 중국 내 해외 건설기계 업체 부문 점유율 1위 기업으로 부상했다. 2000년대 중반부터는 연간 생산 1만 대를 넘겼고, 2010년에는 연간 2만 대 생산을 초과하며 성장해갔다.

　이번 20만 대 누적생산 기록은 중국에 진출한 해외 건설기계 회사 중 최초다. 두산인프라코어는 중국 현지 기업을 제외한 굴착기 시장에서 3분기(7∼9월) 점유율 22.8%를 기록했다. 미국의 건설기계 업체 캐터필라와 함께 이 시장의 점유율 1, 2위를 다투고 있다.

　두산인프라코어는 지난해 중국에서 건설기계 1만5000여 대를 판매했다. 올해는 신종 코로나바이러스 감염증(코로나19)으로 인한 시장 침체에도 불구하고 3분기까지 1만4348대를 팔아 지난해 실적과 맞먹는 성과를 거두고 있다. 두산인프라코어는 중국 굴착기 시장의 성장세에 맞춰 신제품과 특수장비들을 계속 출시할 계획이다. 또한 중국 내 지역사회 발전을 위해 낙후지역 청소년 학업 지원과 교육환경 개선 사회공헌 활동도 추진하고 있다.

# BTS “한국전쟁, 한미 고난의 역사”에 中 생트집

세계적 케이팝 그룹 방탄소년단(BTS)이 수상소감에서 6·25전쟁을 언급했다가 중국에서 거센 비판을 받고 있다. 6·25전쟁과 관련해 ‘양국(한국 미국)’만 언급한 점을 두고 ‘중국을 무시했다’며 발끈한 것이다.

　BTS는 7일(현지 시간) 미국 비영리단체 코리아소사이어티가 주는 ‘밴플리트상’을 받았다. 이 상은 6·25전쟁에 참전한 제임스 밴플리트 미 8군 사령관을 기리기 위한 것으로 1995년부터 매년 한미관계 증진에 기여한 개인이나 단체에 수여된다. 김대중 전 대통령, 이건희 삼성그룹 회장, 대한상공회의소 등도 수상한 바 있다.

　이 자리에서 BTS의 리더 RM(본명 김남준)은 수상 소감으로 “올해는 한국전쟁 70주년으로 우리는 양국(한미)이 함께 겪었던 고난의 역사와 많은 남성과 여성의 희생을 영원히 기억해야 한다”라고 말했다. 이 소감이 뒤늦게 중국에 알려지면서 중국 언론과 누리꾼들이 발끈한 것이다.

　12일 중국 관영 환추시보는 “수상 소감 중 ‘양국이 겪었던 고난의 역사’라는 부분에 중국 누리꾼들이 분노하고 있다”고 보도했다. 신랑왕(新浪網) 텅쉰왕(騰訊網) 등 유명 뉴스 포털사이트에서는 관련 댓글에 “한국전쟁 당시 중국 군인들의 고귀한 희생을 무시한 것”이라면서 “BTS의 수상 소감은 미국의 침략과 아시아에 대한 간섭을 무시하는 발언”이라는 의견까지 등장했다.

　중국은 6·25전쟁을 ‘항미원조(抗美援朝·미국에 맞서 북한을 도움) 전쟁’이라고 부르고 있다. 특히 미중 갈등이 고조되는 와중에 올해 참전 70주년을 맞아 민족주의 애국주의 영웅주의 등의 의미를 담은 ‘항미원조 정신’을 강조하고 있다. BTS에 대한 중국 누리꾼들의 과격한 반응도 이 연장선에서 나온 것으로 분석되고 있다.

# 트럼프 “中 의존 영원히 끝낼것” 또 공격

미국과 중국의 경제 갈등이 재점화하고 있다. 도널드 트럼프 미국 대통령은 중국이 미국과의 무역에서 얻은 돈을 군비 강화에 쓰고 있다고 비난하면서 “중국에 대한 의존을 끝내겠다”고 강조했다. 이에 중국 왕이(王毅) 외교담당 국무위원 겸 외교부장은 자국 정보기술(IT) 기업들을 겨냥한 미국의 압박을 즉각 비난하면서 맞섰고 중국 관영매체는 중국이 보유한 미국 국채 매각 가능성을 언급하며 보복을 경고했다.

　트럼프 대통령은 7일(현지 시간) 노동절 휴일을 맞아 진행된 언론 브리핑에서 “지금까지 중국만큼 우리(미국)를 뜯어먹은 나라는 없었다”면서 “중국은 우리가 준 돈을 군사력 강화에 쓰고 있다”고 말했다. 그는 이어 “내가 우리 군사력을 강화해서 망정이지, 안 그랬으면 중국에 추월당했을 것”이라며 “그것이 디커플링(탈동조화)이든, 우리가 계속 해온 막대한 관세든 간에 우리는 중국에 대한 의존을 영원히 끝낼 것”이라고 말했다.

　최근 트럼프 행정부는 화웨이와 틱톡, 위챗 등 중국의 IT 기업들을 국가안보 위협으로 규정하면서 강력한 규제에 나서고 있다. 특히 미국은 중국의 대표적인 반도체 기업 SMIC를 거래제한 기업 리스트에 올리는 방안까지 검토하고 나섰다. 금융 분야에서는 미국의 회계 기준을 지키지 않는 중국 기업들을 미 증시에서 퇴출시키겠다는 방안을 발표하는 등 파상 공세를 이어가고 있다.

　이에 중국은 8일 왕 부장 주도로 ‘글로벌 데이터 안보’에 관한 이니셔티브를 발표하며 미국에 대한 견제에 나섰다. 막연히 ‘안보 위협’이라고 비난할 게 아니라 구체적인 규칙과 표준을 제정해 따져 보자는 것이다. 왕 부장은 “중국 정부는 중국 기업에 대해 다른 나라 법을 위반하면서 국외 데이터를 제공하라고 하지 않을 것”이라며 “일부 국가가 안전을 핑계로 선두 기업을 공격하는 것은 노골적인 횡포”라며 미국 정부를 정조준했다.

　또 중국 관영 글로벌타임스는 최근 “중국이 미국 국채를 상당 부분 매각할 수 있다”고 보도했다. 현재 중국 정부가 보유한 미 국채는 1조 달러(약 1200조 원) 이상인데, 이를 8000억 달러까지 점진적으로 낮출 수 있다는 것이다. 그러면서 양국의 군사적 충돌 같은 극단적인 상황에서는 모든 보유 국채를 내다팔 수도 있다고도 덧붙였다. 미국 국채의 최대 보유국인 중국이 미 국채를 대량으로 팔면 달러화 가치가 폭락하고 금리가 급등하는 등 국제 금융시장이 마비되는 상황이 발생할 수 있다.

　중국과의 경제 관계를 끊어버리겠다는 미국의 엄포에 대응해서도 자구책을 마련하고 있다. 글로벌타임스는 6일 ‘중국은 미국의 디커플링 시도를 상쇄할 방안을 찾고 있다’는 제목의 칼럼에서 “중국은 자국에 적대적인 나라에는 등을 돌리고 현재 추진 중인 일대일로(一帶一路) 정책에 따라 유럽 및 아프리카 아시아 나라들과 긴밀한 경제적 파트너십을 형성할 것”이라며 “트럼프 행정부가 미중 간 디커플링에 나선 만큼 중국은 스스로 자신의 운명을 개척해야 한다”고 보도했다.

# “中어선, 동해 북한 수역서 오징어 5200억원어치 잡아들였다”

중국의 암흑선단이 유엔 제재를 받고 있는 북한의 동해로 몰래 들어가 약 2년간 불법 조업으로 5200억 원어치가 넘는 오징어를 남획했다는 인공위성 분석 결과가 공개됐다. 암흑선단은 선박의 위치를 송출하지 않거나 공개된 모니터링 시스템에 나타나지 않는 무허가 불법 선박이다. 한국인 데이터 과학자와 국제 비정부기구가 주도한 국제 연구가 밝힌 결과다. 중국의 불법 조업 선단 때문에 영세한 북한 어민이 더 위험한 먼바다로 밀려났다는 사실도 확인됐다.

　비영리 민간연구단체 ‘글로벌어업감시’와 한국해양수산개발원, 일본수산연구교육기구, 미국 캘리포니아대는 2017, 2018년 북한 동해에서 중국 어선들이 이 같은 세계 최대 규모의 불법 조업을 벌였다는 인공위성 정밀 분석 결과를 국제학술지 ‘사이언스 어드밴시스’에 22일 공개했다.

○중국 암흑선단 추적하는 국제 공조

　중국 정부는 수년째 자국 어민의 남획으로 각국 정부와 환경단체의 비난을 듣고 있다. 그럼에도 중국은 아무런 조치를 취하지 않고 있다. 해양 보호단체인 오세아나, 비영리 위성정보 분석단체인 스카이트루스, 구글은 급기야 2016년부터 인공위성을 동원해 세계 바다를 운항하는 어선 3만5000척을 추적하는 ‘글로벌어업감시’ 프로젝트를 벌이고 있다. 인공위성과 선박 정보를 이용해 남획을 일삼는 대형 어선을 추적하겠다는 의도다.

　박재윤 글로벌어업감시 수석데이터과학자를 포함한 연구팀은 2017, 2018년 북한의 배타적 경제수역에 진입한 오징어잡이 선박을 집중 감시했다. 이들 선박 가운데 상당수는 중국 앞바다에서 활동하던 암흑선단이 남해를 거쳐 동해로 진출한 것으로 추정된다. 하지만 이들을 지속적으로 추적 감시할 방법이 마땅히 없는 실정이다. 박 수석과학자는 e메일 인터뷰에서 “한반도 동해 북측 수역은 암흑선단 활동이 심각하지만 인접국 간 협력이 이뤄지지 않아 불법 어로 활동이 제대로 감시되지 않고 있다”며 “인공지능(AI)과 여러 위성 데이터를 바탕으로 암흑선단의 조업을 종합적으로 밝혀낼 곳으로 동해를 선택했다”고 말했다.

　연구팀은 네 가지 위성 관측 기술을 조합해 어떤 환경에서도 불법 어선을 추적 감시할 수 있는 기술을 개발했다. 먼저 미국의 위성영상 서비스 기업 플래닛랩스가 보유한 군집위성을 이용해 두 척의 배가 그물로 어류를 포획하는 쌍끌이 어선을 찾아 AI를 이용해 식별했다.

　여기에 구름이 낀 날에도 어선을 찾고 추적할 수 있는 위성 레이더(SAR) 3기를 동원해 선박 크기와 위치, 이동 경로를 추적했다. 마지막으로 선박 이름과 속력 등 정보를 자동으로 수집, 추적해 충돌을 감시하는 선박자동식별시스템(AIS)을 통해 선박의 공식적인 움직임을 추적했다. 추적 결과 연구팀은 2017년 796척, 2018년 588척의 쌍끌이 어선을 찾아냈다.

　연구팀은 대부분의 오징어잡이 어선이 밤에는 불을 켜고 오징어를 유인해 잡는다는 점에 착안해 고감도 적외선감지기(VIIRS)를 장착한 위성을 동원해 이를 추적하는 데 성공했다. 이런 방식으로 2017년에는 108척, 2018년에는 130척의 오징어잡이 선박을 찾아냈다.

　공동연구팀이 2년간 수집한 위성 정보를 분석해 찾아낸 중국의 불법 선박은 1600척이 넘는다. 잡아들인 오징어는 16만4000t으로, 금액으로 환산하면 4억4000만 달러(약 5263억 원)어치에 해당하는 것으로 추정된다. 공식적으로 가장 많은 오징어 어획량을 올린 일본과 한국의 전체 어획량을 더한 것과 맞먹는 양이다. 박 수석과학자는 “이런 규모의 불법 선단은 중국 전체 원양어선의 3분의 1에 달하는 규모”라며 “한 국가의 상업 선단이 다른 나라 수역에서 저지른 불법 조업 사례 중 가장 큰 규모”라고 말했다.

○영세한 북한 어민은 먼바다로 밀려나

　연구팀은 선체 길이가 10∼20m에 불과하고 전구 몇 개만 달고 조업하는 작고 영세한 북한 어선들이 러시아 연안에서 오징어를 잡고 있는 상황을 포착했다. 2018년에만 이런 활동은 3000회 이상 포착됐다. 이정삼 한국해양수산개발원 연구위원은 “길이가 50m에 첨단 장비로 무장한 중국 쌍끌이 어선과의 경쟁에 밀려 북한 어민들이 인근 러시아 해안까지 가게 된 것으로 보인다”며 “이들이 타고 있는 소형 목선은 작고 열악해 이처럼 먼바다로 나가는 데 적합하지 않고 위험하다”고 말했다.

　실제로 최근 북한 어선 수백 척이 러시아나 일본 해안을 표류하고 일부 어민들이 숨진 채로 발견되고 있는 것도 중국 어선들의 북한 수역 진출과 무관하지 않다는 게 연구팀의 분석이다. 2018년 러시아 해역에서 북한 어선의 어로 활동이 2015년에 비해 약 6배 늘어났다는 사실도 이번에 드러나 해가 갈수록 중국 암흑선단의 횡포가 극심해지고 있는 것으로 나타났다.　

　박 수석과학자는 “중국의 대규모 상업 어선단 때문에 영세 어민이 피해를 받는 사례는 라이베리아 등 서아프리카에서도 발생하고 있다”며 “위성 데이터와 AI를 이용해 국가 어업감시기구에 기술을 지원하면 지속 가능하고 공정한 어로 활동을 제공할 수 있다”고 말했다.

　중국 암흑선단의 불법 남획으로 동해의 어류 자원이 고갈되고 해양 생태계가 파괴되고 있는 것도 문제다. 2003년 이후 한국과 일본의 오징어 어획량은 각각 80%와 82% 줄어든 상태로, 배후에는 중국의 불법 조업이 있는 것으로 추정된다. 박 수석과학자는 “오징어와 같이 국가 간 경계선을 넘나드는 어종을 관리하려면 정보 공유가 중요하다”며 “역내 국가들이 데이터와 과학적 접근을 바탕으로 지역 어업을 협력적으로 관리할 수 있는 메커니즘을 만들길 기대한다”고 말했다.

# ‘휴스턴 中총영사관 폐쇄’ 갈등 고조

미국이 텍사스주 휴스턴 주재 중국 총영사관에 폐쇄 조치를 내린 것과 관련해 도널드 트럼프 대통령이 “중국 공관을 추가로 닫는 것은 언제든지 가능하다”고 말했다. 중국의 강력한 반발과 전 세계의 우려에도 물러서지 않고 오히려 추가 조치 가능성을 언급하면서 대중 압박 수위를 최고조로 끌어올린 것이다.

　트럼프 대통령은 22일(현지 시간) 백악관 브리핑에서 이렇게 밝히며 “우리가 폐쇄한 곳(휴스턴 주재 중국 총영사관)에서 불이 났다고 생각했고 모두가 ‘불이야’라고 했지만 내 생각에 그들은 서류와 문서를 태운 것 같다”고 말했다. 중국 총영사관이 미국 내 불법 활동과 관련된 기록을 없애려 했을 것이라는 취지의 발언이다.

　스티븐 비건 국무부 부장관은 이날 상원 외교위원회가 미국의 대중 정책을 주제로 개최한 청문회에서 이번 조치가 트럼프 대통령의 지시에 따른 것이었다고 확인했다. 비건 부장관은 “중국의 미국 기술 탈취와 지식재산권 침해 등 현안마다 이어진 분쟁이 이런 조치를 내리게 된 배경”이라며 중국을 조목조목 비판했다.

　주미 중국대사관은 성명을 내고 “미국의 주장은 근거가 전혀 없는 견강부회”라고 비판했다. 차이웨이(蔡偉) 휴스턴 주재 중국 총영사도 ABC방송과의 인터뷰에서 “미국의 결정에 큰 충격을 받았다”며 “미국 일부 정치인은 입만 열면 거짓말하는 수작을 집어치워라”라고 원색적으로 비난했다.

# 美-中홍콩갈등 폭발 ‘헥시트’ 문이 열린다

미국이 지난달 29일(현지 시간) 홍콩에 대해 국방물자 수출 중단 및 첨단 기술의 수출 규제에 나섰다. 중국의 홍콩 국가보안법 강행 처리에 대응하기 위해 홍콩의 특별지위를 박탈하는 작업에 본격 착수한 것이다. 한동안 물밑으로 가라앉았던 미중 간 갈등이 다시 격화되고, 홍콩의 앞날은 격랑에 빠지게 됐다.

　로이터통신 등에 따르면 윌버 로스 미 상무장관은 이날 성명에서 “수출 허가 예외 등 홍콩에 특혜를 주는 미 상무부의 규정이 중단됐다”며 “다른 (특혜) 조치를 폐지할지는 검토 중”이라고 밝혔다. 홍콩이 중국에 반환됐던 상징적인 날(1997년 7월 1일)을 코앞에 두고 내놓은 조치다. 마이크 폼페이오 국무장관도 이날 성명에서 “국방물자 수출을 중단하고 (군과 민간의) 이중 용도 첨단기술 규제를 중국과 마찬가지로 홍콩에 적용하는 절차를 시작할 것”이라고 했다. 그는 “이제는 더 이상 통제 품목의 수출에 대해 홍콩과 중국 본토를 분리할 수 없다”고 덧붙였다.

　미국이 이번 조치를 시작으로 홍콩에 적용되던 관세 특혜 철폐 등을 포함한 특별지위의 전면 박탈에 나설 경우 글로벌 금융자본과 인력이 대거 홍콩에서 빠져나가는 ‘헥시트’(홍콩+엑시트)가 현실화될 것이라는 우려가 나온다. 캐리 람 홍콩 행정장관은 “미국의 어떠한 제재도 두렵지 않다”고 반발했다.

　중국은 미국의 전방위 압박에도 불구하고 이날 홍콩보안법 제정을 완료했다. 홍콩 사우스차이나모닝포스트(SCMP)에 따르면 중국 전국인민대표대회(전국인대) 상무위원회는 참석자 162명 전원의 찬성으로 홍콩보안법을 상정 15분 만에 전격 통과시켰다. 홍콩 정부는 홍콩의 실질적인 헌법인 기본법 부칙에 이 법을 즉시 삽입해 홍콩 주권 반환일인 7월 1일부터 시행할 것으로 보인다.

　국가 전복, 테러, 외국 세력과 결탁 등의 행위를 금지하는 홍콩보안법을 어기면 최대 종신형에 처해진다. 반중(反中) 인사 재판에는 홍콩 행정장관이 특정 판사를 지명할 수 있도록 했다. 홍콩보안법이 통과되면서 홍콩의 대표적 민주화 인사인 조슈아 웡 홍콩 데모시스토당 비서장(24)과 반중 성향 일간지 핑궈(빈果)일보 사주 지미 라이 회장(72)이 곧 체포될 것이라는 관측이 나온다.

# 코로나19가 왜 美-中대립의 속도를 높이나

신종 코로나바이러스 감염증(코로나19)은 인류가 직면한 매우 보기 드문 생물 안보 재난이다. 중국과 미국 양국에 공통의 위협이며 미중이 협력해야만 전 세계인들과 함께 코로나19에 승리할 수 있다. 하지만 코로나19 이후 미중 관계가 계속 악화되고 양국 관계가 ‘신냉전’에서 불과 한 발짝밖에 떨어져 있지 않아 보인다.

　코로나19는 미중 관계를 더욱 악화시키고 있다. 도널드 트럼프 미국 대통령 집권 이후 미국의 ‘대중국 정책 발언 시스템’에 역사적인 후퇴가 나타났다. 트럼프 정부는 강하게 중국을 ‘악마화’해 왔다. 이런 ‘중국 악마화’는 트럼프 정부와 극우 공화당 세력이 보여준, 중국인들로서는 이해하기 힘든 이른바 ‘(중국에 당한) 피해자 콤플렉스’를 대표한다. 이 콤플렉스는 3년여 동안 트럼프 정부 대중국 정책의 기본 기조가 됐다. 코로나19가 원래 미중 협력의 기회였음에도 유감스럽게도 트럼프 정부는 이른바 미국 이익 우선 정책을 더욱 강력하게 진행하면서 중국에 대해 높은 대립 정서를 표출했다. 이는 중국을 압박하는 주요한 수단이 됐다.

　우선 미국 내 코로나19의 심각한 상황은 트럼프 정부와 미국 공화당 우익 정치세력들이 보여온 반중(反中) 피해자 콤플렉스를 더욱 히스테릭하게 변화시켰다. 트럼프 정부는 코로나19를, 중국을 더욱 압박하고 정치 경제적으로 중국과의 관계를 청산하는 기회로 본다.

　두 번째로 코로나19는 미국의 중국에 대한 우려를 격화했다. 트럼프 정부가 추진하는 디커플링(관계 단절) 방향의 중국 정책을 강화시켰다. 미국인은 코로나19가 중국에 큰 기회의 창을 열었다고 여긴다. 중국이 전 세계에 대한 영향력을 더욱 강하게 추구하고 미국의 세계 리더 지위를 밀어내려 한다고 여긴다. 중국에 대한 과학기술 전쟁, 무역 전쟁, 언론 전쟁, 심지어 앞으로 금융 전쟁까지 강화해 산업망, 공급망, 가치망을 중국에서 빼내는 것은 중국의 굴기를 억제하는 것이고, 미국의 지속적인 패권 우위를 유지하는 것으로 생각한다.

　세 번째 코로나19는 트럼프 정부가 자유자재로 사용하는 중국 압박의 정치적 도구가 됐다. 중국을 압박하면 미국이 코로나19 초기에 보여준 무능하고 효과가 낮은 대응에 대한 미국인들의 원망을 중국으로 돌릴 수 있다. 이뿐 아니라 트럼프가 ‘당신들은 중국인을 증오해야 한다. 중국인이 오늘날 이런 두려운 국면을 만들었다’고 말할 수 있게 됐다. 이는 이미 트럼프 대통령의 대선 전략의 중요한 부분이 됐다. 미국에서 중국 문제는 이미 트럼프 대통령에 의해 완전히 정치화됐다. 마지막으로 코로나19는 미국 사회의 반중(反中), 혐중(嫌中), 중국에 대한 공포 정서를 높였고 트럼프의 중국 압박 정책은 미국 내에서 더 많은 지지를 얻게 됐다.

　오늘날 미국 국민의 70%가 ‘중국이 코로나19 확산에 대해 책임을 져야 한다’고 여긴다. 코로나19는 미국 내 아시아계 주민에 대한 인종주의 차별과 배척을 격화시켰다. 다수의 미국 정치 엘리트와 국민들이 중국 정책을 보는 견해는 코로나19의 영향을 받아 1950년대와 비슷한 ‘신(新)매카시즘(정치적 반대자를 공산주의자로 매도하는 태도)’으로 돌아가기 시작했다. 이성적이고 온화한 중국 정책의 목소리는 계속 밀려나고 있다.

　미중 관계는 양국 모두 진지하고 이성적인 정책과 책략, 반성이 필요한 시점에 도달했다. 국제 체계에서 강대국의 흥망성쇠는 종종 피할 수 없는 강대국 간 격렬한 권력 경쟁과 전략적 대립을 가져온다. 하지만 21세기의 오늘날 미중의 어떤 ‘신냉전’의 앞날도 세계 안정과 평화와 번영을 해칠 것이다. 더욱이 동북아 지역 경제 발전에 재난과 같은 충격을 가져올 것이다. 최근 미국의 지미 카터, 빌 클린턴, 조지 W 부시, 버락 오바마 전 대통령이 함께 목소리를 내 트럼프의 대내외 정책을 호되게 비판했다. 그리고 미국 정책의 ‘재난적 실패’의 근원을 반성하라고 요구했다. 이와 마찬가지로 중국 정부 역시 코로나19 사태 과정에서 중국이 보여준 수많은 문제 가운데 정치 경제 개혁을 촉진하고 이미지 개선을 가속화하는 미래의 길을 찾아야 할 필요가 있다. 이렇게 할 때만 중국과 미국 양국이 계속해서 세계 다수 국가의 이해와 존중을 얻을 것이다.

# 中외교부 “한반도 안정 희망”... 北에 자제 촉구

북한이 16일 오후 개성 남북공동연락사무소를 폭파한 것에 대해 주요 외신들은 일제히 속보로 보도하며 “한반도 내 긴장이 높아지고 있다”고 전했다. 중국 외교부는 “한반도의 평화와 안정을 바란다”며 북측에 자제를 촉구했다.

　AP통신과 CNN 방송, 뉴욕타임스(NYT), 아사히신문 등은 이날 오후 통일부의 발표를 인용해 북한이 연락사무소 청사를 폭파했다는 사실을 전하면서 이번 폭파는 13일 김여정 북한 노동당 제1부부장이 한국 정부가 탈북단체의 대북 전단 살포를 막지 못한 것을 비난하며 예고했던 것이라고 덧붙였다.

　AP통신은 일부 전문가를 인용해 한국이 미국 주도의 대북 제재로 경협을 재개할 수 없는 것에 대해 북한이 답답함을 토로하고 있다고 분석했다. NYT는 “최근 남북 간의 화해 무드를 끝내겠다고 위협해온 북한이 남한에 대한 불만을 극적인 방법으로 표시했다”고 전했다. 워싱턴포스트(WP)는 북한이 최근 몇 주간 한국에 점점 더 날카로운 어조로 비판해왔다면서 연락사무소 파괴가 갈등을 급격히 증폭시킬 것이라고 전망했다. 아사히신문은 연락사무소가 문재인 정부에는 대북 정책의 성과를 상징하는 것이었다며 큰 타격이 될 수밖에 없을 것이라고 내다봤다.

　자오리젠(趙立堅) 중국 외교부 대변인은 이날 정례 브리핑에서 북한의 연락사무소 폭파에 관련한 질문을 받고 “북한과 한국은 같은 민족”이라며 “중국은 이웃 국가로서 한반도의 평화와 안정 유지를 일관되게 희망한다”고 밝혔다. 스가 요시히데(菅義偉) 일본 관방장관은 “계속해서 미국, 한국 등과 함께 긴밀히 협력하면서 필요한 정보의 수집, 분석을 실시하고 정세를 주시하는 한편으로 경계, 감시에 전력을 기울이고 있는 중”이라고 밝혔다.

# “베이징 집단감염 원인 유럽 수입연어 가능성”

중국 베이징(北京) 남부 신파디(新發地) 농수산물 도매시장에서 시작된 신종 코로나바이러스 감염증(코로나19) 재확산이 베이징 이외 지역으로 확산되고 있다. 중국 정부는 유럽에서 수입한 연어 등에 코로나19가 묻어서 유입됐을 가능성을 제기하고 나섰다.　

　15일 중국 국가위생건강위원회에 따르면 14일 하루 동안 베이징에서 신규 확진자가 36명 발생했다. 11일 신파디 시장발 첫 환자가 발생한 이후 나흘 동안 베이징에서 총 79명의 확진자가 나왔고, 베이징 16개 구 가운데 8개에서 환자가 확인됐다.

　베이징시는 14일 시민 7만6499명을 대상으로 코로나19 검사를 진행하는 등 대대적으로 검사를 하고 있어 신규 확진자 수가 빠르게 늘어날 것으로 예상된다. 쑨춘란(孫春蘭) 국무원 부총리는 14일 “베이징 코로나19 확산 위험이 매우 크다”고 우려했다.

　베이징 외에 허베이(河北)성의 바오딩(保定)시에서 신파디 시장 상인의 일가족 3명이 확진 판정을 받았고, 쓰촨(四川)성에서도 신파디 시장과 관련된 의심 환자 1명이 확인됐다.

　중국은 유럽에서 수입된 수산물이나 육류에 바이러스가 묻어 중국으로 들어왔을 가능성을 제기했다. 베이징 질병예방통제센터 양펑(楊鵬) 주임은 이날 관영 중국중앙(CC)TV에서 “바이러스가 어떻게 왔는지 불확실하다”면서도 “유전자 서열 분석을 통해 (신파디 시장에서 발견된 코로나19) 바이러스가 유럽 쪽에서 온 것임을 확인했다”고 밝혔다.

　이어 “해외 코로나19 상황이 아직 심각해 (해외에서) 육류와 수산물을 처리하는 과정에서 (감염자의) 바이러스에 오염돼 수입됐을 가능성이 있다”고 말했다. 중국 전문가들은 코로나19 바이러스의 생존 능력이 저온에서 극대화되기 때문에 냉동 냉장 유통 과정에서 바이러스가 유입됐을 가능성이 충분하다고 주장한다.

　중국 소셜네트워크서비스(SNS)에는 “또 해외 유입(에 책임을 돌리나)”이라는 비판과 “해외 (수산물, 육류) 가공 직원들이 마스크를 쓰지 않는 걸 봤다”는 주장이 동시에 나왔다. “후베이(湖北)성 우한(武漢)에서 퍼졌던 바이러스 역시 유입된 것”이라는 음모론까지 다시 고개를 들었다.

# 美 “中보복땐 한국 위해 뭐든 할 준비 돼”

키스 크라크 미국 국무부 경제담당 차관은 11일(현지 시간) 한국이 반중(反中) 경제블록구상인 ‘경제번영네트워크(EPN)’나 화웨이 제재 등에 동참해 중국의 보복 조치에 직면할 경우 “미국은 한국을 돕기 위해 무엇이든 할 준비가 돼 있다”고 밝혔다. 또 “전 세계가 중국의 위협과 보복에 맞서기 위해 일어서야 한다”며 동맹 및 파트너 국가들에 미국의 강경한 대중정책 동참과 연대를 요구했다.

　크라크 차관은 이날 인도, 브라질 등 5개 국가 주요 언론사들과 진행한 전화 간담회에서 미국의 대중 경제제재 및 정책 구상에 대해 설명하며 이렇게 밝혔다. 한국 언론사 중에서는 동아일보가 유일하게 간담회에 참여했다.

　그는 미국이 우방들에 ‘미국의 대중정책에 동참해 달라’고 요구한 것과 관련해 “중국이나 미국 중 한쪽을 선택하라는 게 아니다”며 “선택은 누구에게나 열려 있지만 결국 어느 쪽을 신뢰할 것이냐의 문제”라고 설명했다.

　크라크 차관은 “한국은 전 세계의 경제적, 기술적 파워하우스이자 미국뿐 아니라 전 세계적으로 큰 무역 파트너”라며 한국과의 경제협력을 강조했다. 특히 삼성전자에 대해선 “세계 3대 5세대(5G) 관련 기업 중 하나이며 가장 발달한 반도체 생산업체”라고 높이 평가했다.

# 美 “中에 함께 맞서자“... EPN 참여 원칙과 실익 면밀 검토해야

키스 크라크 미국 국무부 경제차관은 11일 중국에 맞서는 새로운 경제블록 구상인 경제번영네트워크(EPN)에 한국이 참여해줄 것을 요청하며 중국의 보복 조치에 직면할 경우 “미국은 한국을 돕기 위해 무엇이든 할 것”이라고 말했다. 그러면서 “중국·미국 중 선택하라는 게 아니다. 선택은 누구에게나 열려 있지만 결국 어느 쪽을 신뢰하느냐의 문제”라고 했다.

　크라크 차관의 발언은 그간 미국이 추진해온 EPN 구상과 화웨이 제재 등 중국 견제 정책에 한국이 적극 참여해야 한다는 요청이다. EPN은 중국을 배제하고 미국 주도의 새로운 글로벌 공급사슬(GSC)을 구축하겠다는 구상이다. 중국의 일대일로(一帶一路) 세력권 확장에 맞선 미국의 안보전략이 인도태평양전략이라면, EPN은 경제 차원의 중국 견제전략이다. 여기에 미국의 동맹으로서 한국이 참여해야 한다는 본격적인 압박인 것이다.

　미국은 중국식 국가주의적 자본주의에 맞서 민주주의와 인권, 투명성, 지적재산권 보호 같은 가치를 공유하는 자유주의적 자본주의 국가 간 연대의 필요성을 강조한다. 하지만 문제는 EPN 구상은 아직 전혀 익지 않은 과일로 보인다는 점이다. 중국 배제 이외에는 어떤 구속력을 가진 형태가 될지, 그 협력 내용은 무엇인지 제대로 구체화된 게 없다. 오직 ‘세계의 공장’이 된 중국을 배제하겠다는 미국의 전략적 판단만 두드러질 뿐이다.

　참가국들에 대한 중국의 보복 우려에 대해 미국은 “뭐든 돕겠다”고 한다. 미국이 나선다면 중국 보복에 맞선 충분한 대항력과 보상도 가능할 것이다. 하지만 EPN 참여는 중국의 보복에 따른 직접적인 피해는 물론 산업구조 전반의 변화를 요구하는 사안이다. 특히 우리에겐 중국의 사드(THAAD·고고도미사일방어체계) 보복 당시 제3자연하던 미국의 태도가 여전히 아픈 기억으로 남아 있다.

　미국이 내건 대로 자유와 민주의 가치를 공유하는 국가연대에 동맹국인 한국이 빠질 이유는 없다. EPN을 현재 중국 의존도가 높은 수출과 생산을 다변화할 수 있는 기회로 만들 수도 있다. 그러나 우리의 최대 교역국이자 세계 최대 시장에 완전히 등을 돌리는 것은 가능하지 않고 미국도 그렇게까지 요구하진 못할 것이다. 범정부, 나아가 산업계 의견을 모으면서 참여의 원칙과 수준, 실익, 국제적 동향을 면밀히 따져가며 단단히 준비해야 한다.

# 한국 조선 수주 점유율, 中과 격차 좁혀

앞서 가던 중국의 조선 수주 점유율이 주춤하며 추격하던 한국과의 격차가 대폭 좁혀졌다. 중국이 자국 물량을 쏟아 부으면서 인위적으로 수주량을 끌어올리던 시도가 한계에 다다랐기 때문이다.

　9일 영국 조선 해운 시황 분석기관인 클라크슨리서치에 따르면 지난달 전 세계 선박 발주량은 총 57만 CGT(표준화물선 환산 톤수)로 141만 CGT를 기록한 전월보다 40% 정도 감소했다. 국가별로는 지난달 중국이 27만 CGT(13척, 47%)를 수주했고, 한국은 23만 CGT(8척, 40%), 일본은 5만 CGT(2척, 9%)를 수주했다.

　올해 수주 실적은 중국이 세계 1위를 달리고 있다. 그러나 한국과 중국의 점유율 격차는 점차 줄어들고 있다. 1∼5월 국가별 누적 수주 실적은 중국 288만 GCT, 한국 90만 CGT, 일본 49만 CGT다. 4월 한국과 중국의 월별 수주 점유율은 55%포인트까지 차이가 났지만 지난달 월별 수주 점유율 차이는 7%포인트까지 줄었다. 이는 중국의 자국 발주 물량이 대폭 감소했기 때문이다. 지난달 수주량의 경우 한국은 4월과 비슷한 수준을 유지했지만 중국은 전월 대비 73% 급감했다. 특히 지난달 중국의 수주량 중 85%는 자국 발주 물량이었지만 한국은 전부 유럽과 아시아 국가 선주들로부터 수주한 물량이다.

　이에 업계에서는 꾸준한 수주를 하고 있는 한국이 하반기(7∼12월)에 중국의 수주량을 제칠 것으로 보고 있다. 한국은 최근 카타르와 대규모 액화천연가스(LNG) 운반선 건조 슬롯 계약을 체결했고, 러시아와 모잠비크에서도 한국이 강점을 가진 대형 LNG 발주 프로젝트가 예정돼 있기 때문이다.

# 무역협회 “美中 홍콩갈등 격화땐 韓수출 타격”

홍콩보안법 제정을 둘러싼 미국과 중국의 갈등이 심각해지면 홍콩을 중계무역 기지로 활용하던 우리 수출에도 타격이 불가피하다는 전망이 나왔다.

　29일 무역협회 국제무역통상연구원에 따르면 홍콩은 우리 기업들이 중국으로 재수출을 하기 위해 이용하는 중계무역의 요충지다. 홍콩은 중국 본토로의 접근성이 좋고 부가가치세 환급, 낮은 법인세, 각종 비과세 등의 세제 혜택, 뛰어난 무역 인프라를 갖추고 있기 때문이다. 이에 한국→홍콩→중국으로의 물류 이동이 활발한 상태다. 지난해에만 한국에서 홍콩으로 간 수출의 90% 이상이 다시 중국으로 들어갔을 정도다. 특히 미국은 1992년부터 홍콩에 대해 비자 발급과 투자 유치, 법 집행 등에서 특별무역지위를 부여해 대우를 해왔다. 이는 홍콩이 아시아의 대표 금융 물류 요충지로 성장하는 데 중요한 역할을 했다.

　그러나 미국의 대(對)홍콩 제재가 강화되면 각종 혜택이 사라지는 것은 물론이고 외국계 자본의 대거 이탈도 예상된다. 금융과 물류 허브로서의 각종 이점이 사라지기 때문이다. 무역협회는 “이런 사태가 발생하면 중국으로 직접 수출을 할 수밖에 없어 각종 물류비가 증가하고 중국 직수출을 위한 항공편 확보 등에 차질이 생길 수 있다”고 전망했다.

　무역협회 관계자는 “홍콩은 우리나라의 네 번째 수요 수출 국가로 중계무역 기지로서 가치가 높았는데 홍콩의 금융, 서비스, 물류 기능이 약화되면 수출에 타격을 받을 수밖에 없다”고 말했다.

# 미중 갈등 속 中위안화 가치 급락... 유탄 피할 길 없는 한국경제

중국 위안화 환율이 달러당 7위안을 넘는 이른바 ‘포치(破七)’가 일어났다. 중국의 중앙은행인 런민(人民)은행이 25일 달러대비 위안화 기준환율을 전날에 비해 0.38%오른 7.1209위안으로 고시한 것이다. 달러당 7위안은 심리적 저항선으로 여겨져 지난해 8월 미중 무역전쟁이 격화될 당시 ‘포치’가 발생하자 미국은 즉각 중국을 환율조작국으로 지정한 바 있다.

　중국이 위안화 환율을 올린 것은 무역전쟁에 이어 최근 홍콩보안법, 코로나19 발원지 갈등을 둘러싼 미국의 파상 공격에 대한 반격조치로 해석될 여지가 있다. 미국이 압박을 가해오면 중국은 위안화 환율을 높여, 즉 자국의 화폐가치를 낮춰 수출경쟁력을 높이는 방식으로 대응할 것이라는 관측은 늘 제기돼 오던 것이다.

　물론 중국이 1000조 원에 이르는 대규모 경기부양을 위해 통화량을 늘리고 재정적자를 확대한다는 방침을 밝힘에 따라 위안화 가치가 자연스럽게 떨어진 측면도 있다. 양국 관계가 우호적일 때 서로 양해될 수도 있겠지만 거의 극단으로 치닫는 미중 갈등의 양상을 보건대 미국은 수출 확대가 절실한 중국이 적극적으로 평가절하를 유도했다고 볼 가능성이 없지 않다.　

　작년 8월 환율조작국 지정은 올해 1월 15일 1차 미중 무역합의가 이뤄지면서 해제됐다. 이번 ‘포치’를 계기로 미국이 재차 중국에 대해 환율조작국 지정 카드를 꺼내들 경우 어렵게 체결한 무역합의는 휴지조각이 되고 미중 갈등이 환율이슈라는 민감한 국면으로 접어들 우려가 있다.

미중 환율전쟁의 유탄을 맞을 수 있는 대표적인 국가가 한국이다. 대외의존도가 높으며 특히 미중에 치우친 무역구조 때문에 불안한 환율은 우리 기업과 정부 모두에 상당한 애로요인이다. 당장은 위안화와의 동조현상에 따른 원화 약세로 수출경쟁력에 도움이 되겠지만 국내 자본시장에서 달러가 빠져 나가 금융시장이 흔들릴 수도 있다.

　그렇다고 해도 당장 뾰족한 대처방안도 있을 수 없다. 정부는 외환시장을 면밀히 모니터링하면서 국제적으로 용인되는 범위 내에서는 적극적으로 개입하는 노력은 게을리 하지 않아야한다. 이런 어려움이 닥칠 때를 대비해서라도 평소에 기업의 대외 경쟁력과 재정건전성 확보를 통해 외부로부터의 파고(波高)를 막아낼 방파제를 튼튼하게 구축해야 한다.

# ‘기업인 신속통로’ 이용 삼성전자-SK이노 등 중에 인력 550여명 파견

삼성전자 등 국내 주요 기업들이 21, 22일 이틀 동안 ‘한중 기업인 신속통로(입국 절차 간소화)’ 제도를 통해 550여명의 인력을 중국에 파견했다. 신종 코로나바이러스 감염증(코로나19) 확산으로 지연됐던 중국 내 공장 증설 등의 프로젝트가 빠르게 재개될 것으로 보인다.

　삼성전자는 22일 중국 시안(西安) 반도체 제2공장 증설을 위해 필요한 본사·협력업체 기술진 300여 명을 전세기 편으로 파견했다. 이 비행기에는 시안에서 배터리 공장을 운영하고 있는 삼성SDI의 인력 30여 명도 함께 탑승했다. 이재용 삼성전자 부회장이 코로나19 사태 이후 첫 해외 출장지로 시안을 다녀온 뒤 3일 만에 이뤄진 조치다.

　기업인 신속통로는 기업인 등에 한해 출국 전후 각각 코로나19 검사를 받고 음성 판정이 나오면 현지에서 14일 의무격리를 면제해주는 조치다. 삼성전자가 이 제도로 대규모 인력을 해외 현장에 파견한 것은 이번이 처음이다. 4월 시안에 200여 명의 인력을 파견할 때는 이 제도가 생기기 전이라 ‘특별 입국’을 요청해 격리 조치 없이 인력을 파견했다.

　시안 공장은 삼성전자의 유일한 해외 메모리 반도체 생산 기지로 총 150억 달러(약 18조4500억 원) 규모의 투자가 진행되고 있다.

　기아자동차도 이날 전세기 편으로 중국 옌청(鹽城) 공장에 인력 100여 명을 파견했다. SK이노베이션은 앞서 21일 옌청 배터리 공장 건설 현장에서 근무할 인력 120여 명을 전세기로 보냈다.

# 백악관 “中은 약탈경제” 新냉전 선포

도널드 트럼프 미국 행정부가 중국을 상대로 한 미국의 향후 전략 및 정책 방향을 담은 보고서를 공개했다. 중국과의 협력이 아닌 공개 압박, 사실상의 중국 봉쇄 등 ‘경쟁적 접근(competitive approach)’을 하겠다는 점을 분명히 해 사실상 ‘신(新)냉전’을 선언했다는 평가가 나온다.

　워싱턴포스트(WP)는 백악관이 20일(현지 시간) 국방부 초안을 바탕으로 작성한 ‘미국의 대중국 전략 보고서’를 의회에 제출했다고 보도했다. 백악관이 홈페이지에 공개한 16장 분량의 보고서는 “중국의 근본적인 경제 개혁 및 정치적 개방에 대한 기대는 실패로 끝났다. 중국은 생명과 자유, 행복추구권에 대한 미국의 기본적인 신념을 흔드는 정책을 추진하고 있다”고 진단했다. 이어 “이제 중국에 대해 경쟁적 접근을 할 것”이라고 선언했다.

　보고서는 “대중 외교가 헛된 시도임이 확인되면 미국은 중국의 행동에 상응하는 비용을 지렛대로 사용해 미국의 이익을 보호하는 데 나서고 중국 정부에 대한 공개적인 압박을 확대할 것”이라고 밝혔다. 중국의 악의적 행동, 투자, 의도 등을 언급하며 ‘악의적(malign)’이란 형용사를 8차례나 사용했다. ‘약탈적(predatory) 경제’란 표현도 등장했다.

　보고서는 또 ‘전략핵무기 3축체계(Nuclear Triad)’의 현대화로 힘을 통한 평화를 유지할 뜻을 강조했다. 극초음속 미사일체계, 사이버·우주 기반 무기의 실전 배치 등을 앞당기겠다는 계획도 담았다.

　중국의 위협에 맞서기 위한 방안으로는 역내 동맹 및 파트너들과의 관계 강화를 언급했다. 문재인 정부의 ‘신남방 정책’을 미국이 협력해야 할 역내 동맹국 정책으로도 꼽았다. 브루스 베넷 랜드연구소 선임연구원은 미국의소리(VOA) 방송에 “이번 보고서는 사실상 미국 정부가 중국에 대해 신냉전을 선포한 성격이 짙다”고 평가했다.

　또 중국 정부가 홍콩 국가보안법 제정에 나선 것에 대해 미국이 강력하게 반발해 미중 갈등의 새로운 뇌관으로 떠올랐다. 트럼프 대통령은 “만약 그것(홍콩 국가보안법 제정)이 일어난다면 우리는 그 문제를 매우 강하게 다룰 것”이라고 경고했다.

# 美中전방위 패권전쟁 개전...한생존전략 정립하라

미국과 중국이 코로나19 사태를 계기로 정치 외교안보 경제 전반에 이르는 대결을 본격화하고 있다. 미 백악관은 21일(현지시각) 의회에 제출한 보고서에서 ‘중국의 경제 정치 군사적 힘 확대가 미국의 이익과 전 세계 국가의 주권을 훼손했다’면서 ‘지난 20년간 미국의 대중(對中) 정책에 대한 근본적 재검토가 필요하다’고 했다. 미중 갈등이 코로나19에 대한 책임론과 경제전쟁을 넘어 장기적인 패권전쟁으로 나아가고 있다.

　세계 1, 2위 경제대국인 미국과 중국은 트럼프 정부 출범 이후 노골적인 무역분쟁을 일으켜왔는데 최근엔 코로나19 사태의 책임을 놓고 “악랄한 독재정권” “완전히 미쳤다”면서 정면충돌했다. 중국이 전국인민대표대회에서 ‘홍콩 국가보안법’을 제정하겠다고 하자 미국이 강경 대응 방침을 밝히는 등 전선(戰線)은 점점 늘고 있다.

　미국은 한국의 외교안보 동맹이지만 경제 분야에서는 대중 수출이 1위, 대미 수출이 2위다. 한국은 그동안 미중 사이에서 전략적 모호성을 유지해왔으나 점점 양자택일의 압력이 강해지고 있다. 미국은 중국을 빼고 안보상 믿을 수 있는 나라들끼리 글로벌 공급망을 새로 짜자는 ’경제번영네트워크(EPN)＇을 들고 나와 한국의 참여를 압박하고 있다. 중국 화웨이에 연간 10조 원 이상의 반도체를 수출하는 한국 기업들에게도 수출 중단 압력을 넣고 있다. 안보는 미국, 경제는 중국에 기대는 안미경중(安美經中) 구조를 가진 한국에는 크나큰 시련이다. 사드(THAADㆍ고고도미사일방어체계) 사태로 경제에 큰 타격을 입었던 악몽을 떠올릴 수밖에 없다.

　그러나 한국 정부가 미중 신(新)냉전 시대를 맞아 제대로 대비하고 있는지 의문이다. 포스트 코로나 시대 글로벌 외교 안보 경제에 대한 총론 없이 남북관계와 시진핑 중국 국가 주석의 방한 등에만 관심을 쏟고 있는 것이 아닌지 걱정스럽다. 북한의 천안함 폭침에 대응한 5·24조치에 대해 한국 정부가 사실상 폐기를 선언하자 당장 미국이 “비핵화 진전과 보조를 맞춰야 한다＂고 제동을 거는 등 한미간에 미묘한 냉기류마저 감돈다.

　외교부는 지난해 미중 갈등 대응을 위해 외교전략조정회의를 출범시켰지만 올해 들어 단 한차례도 열지 않았다. 오히려 문재인 대통령은 취임 3주년 기자회견에서 북-미대화만 바라보지 말고 남북간에 할 수 있는 일을 해야 한다고 강조했다. 중국에 대한 경제의존도가 높은 상황에서 미국의 EPN 압박을 어떻게 풀어나갈지, 시 주석의 연내 방한이 추가적인 긴장요인으로 작용하는 것은 아닌지 정부의 외교 경제 전략을 총체적으로 재점검해야 한다.

# 美, 양회 앞둔 中에 “또라이” “악랄한 독재정권” 원색비난

중국의 최대 정치행사인 양회(兩會)를 앞두고 도널드 트럼프 미국 행정부가 중국에 대한 비난 수위를 최고조로 끌어올렸다. 트럼프 대통령은 중국 정부의 입장 발표에 ‘또라이’ ‘얼간이’라며 막말을 퍼부었고 마이크 폼페이오 국무장관은 “악랄한 독재정권”이라고 중국 정부를 정조준했다.

　트럼프 대통령은 20일 트위터에 “중국의 어떤 또라이(wacko)가 방금 수십만 명을 죽인 바이러스에 대해 중국을 제외한 모든 이들을 비난하는 성명을 발표했다”며 “이 얼간이(dope)에게 이러한 전 세계적 대규모 살상을 저지른 것이 다름 아닌 중국의 무능이라는 것을 설명 좀 하라”고 적었다. 누구를 겨냥한 것인지는 구체적으로 언급하지 않았지만 신종 코로나바이러스 감염증(코로나19)과 관련한 중국 외교부 등 주요 기관의 대변인일 가능성이 높다.

　폼페이오 장관은 이날 오전 기자회견에서 “중국은 1949년부터 악랄하고 권위주의적인 공산 정권에 의해 지배돼 왔다”며 “중국은 이데올로기적으로도, 정치적으로도 자유국가에 적대적”이라고 주장했다. 이어 “이 전염병으로 9만 명에 이르는 미국인이 숨졌고, 3월 이후 3600만 명이 일자리를 잃었다. 중국 공산당의 (대응) 실패로 전 세계적으로 최대 9조 달러의 피해를 입었다”고 비판했다.

　폼페이오 장관은 이날 1억6200만 달러 규모의 해외 코로나19 피해 지원책을 발표했다. 그는 미 국제개발처(USAID)와 함께 100억 달러의 지원을 약속한 것 외에 추가로 이를 지원한다는 점을 강조하면서 “중국이 내놓은 20억 달러는 세계에 끼친 (피해)비용에 비하면 쥐꼬리(paltry) 수준이다. 그거라도 약속한 대로 이행하기를 기대한다”고 꼬집었다.

　그는 이 자리에서 차이잉원(蔡英文) 대만 총통의 재취임을 거듭 축하했다. 홍콩과 관련해서는 “중국으로부터 높은 수준의 자율성을 갖고 있는지에 대한 판단을 아직 내리지 않았고 현재 일어나는 일을 예의 주시하고 있다”고 밝혔다. 반도체 위탁생산(파운드리) 업체인 대만 TSMC의 미국 투자와 상무부의 화웨이 수출 제재안 발표 등도 언급했다.

　이런 트럼프 행정부의 거친 ‘중국 때리기’는 국내 정치용이라는 분석이 나온다. 공화당은 이미 중국 공격을 연말 대선은 물론이고 전국 주요 주지사 및 의원 선거의 핵심 전략으로 삼는 지침을 정했다. 공화당 캠페인 전략팀이 지난달 당에 배포한 메모에는 이와 함께 선거 경쟁자들을 친(親)중국파 혹은 중국에 대해 유약한 이미지로 공격하라는 등의 내용이 담겼다.

　거듭된 공격에 중국도 발끈했다. 중국 관영 영어방송 CGTN은 트위터에 ‘폼페이오 신뢰도 테스트’라는 영상(사진)을 올려 중국을 공격하는 폼페이오 장관을 우스꽝스럽게 묘사했다. 21일 런민일보(人民日報)에 따르면 시진핑 주석은 셰이크 하시나 방글라데시 총리와 통화에서 “중국은 전염병 퇴치를 위한 국제 협력에 방해하는 행위에 반대한다”며 에둘러 미국의 공세를 비판했다.

# 트럼프 “WHO, 한달내 중서 독립 증명해야...아니면 탈퇴할수도”

신종 코로나바이러스 감염증(코로나19) 발원지를 놓고 중국과 치열하게 대립하고 있는 도널드 트럼프 미국 대통령이 세계보건기구(WHO)의 친중 성향을 지적하며 ‘30일 안에 개선이 없으면 자금 지원을 영구 중단하거나 탈퇴할 수 있다’는 최후통첩을 날렸다.

　트럼프 대통령은 18일 테워드로스 아드하놈 거브러여수스 WHO 사무총장에게 보낸 4쪽짜리 서한에서 “30일 안에 중국으로부터의 독립성을 증명하는 개선안을 내놓지 않으면 자금 지원을 영원히 중단할 수 있음을 알린다. 우리의 회원 자격도 재고하겠다”고 주장했다. WHO가 앞으로 나아갈 유일한 길은 중국으로부터의 독립을 보여주느냐에 달렸다며 “미국 이익에 부합하지 않는 조직에 납세자의 돈을 지원할 수 없다”고도 했다.

　그는 취재진 앞에서도 WHO를 중국의 꼭두각시(puppet)라고 강력히 비판했다. 미국이 WHO에 연 4억5000만 달러의 분담금을 내는 반면 중국은 3800만 달러만 낸다는 점도 문제 삼았다.

　앨릭스 에이자 미 보건장관은 이날 사상 최초로 화상으로 열린 세계보건총회(WHA)에서“한 회원국이 투명성 의무를 조롱해 전 세계에 엄청난 희생을 초래했다. 다시는 이런 일이 일어나면 안 된다”며 중국을 정면 비판했다. 마이크 폼페이오 국무장관은 중국의 반대로 대만의 WHA 참가 시도가 무산된 것을 비판하는 성명을 발표했다. 폼페이오 장관은 “WHO 사무총장은 대만을 WHA에 참가시킬 모든 법적 권한이 있음에도 중국의 압력 때문에 하지 않았다. 사무총장의 독립성 결여가 WHO의 신뢰와 효율성을 훼손시켰다”고 지적했다.

　존 울리엇 미 백악관 국가안보회의(NSC) 대변인은 시진핑(習近平) 중국 국가주석이 WHA 화상 회의에서 “코로나19 피해국에 20억 달러를 지원하겠다”고 밝힌 것을 비난했다. 그는 “점점 더 많은 나라가 중국의 책임을 묻는 것에서 주의를 분산하려는 시도”라고 지적했다.

　WHA는 19일 회의에서 코로나19 독립 조사 진행에 대한 결의안을 표결한다. 194개 WHO 회원국 중 3분의 2인 129개국의 지지를 얻으면 통과된다. 이미 호주 영국 프랑스 러시아 등 122개국이 지지를 표명해 통과 가능성이 높다는 관측이 제기된다.

　중국도 반격에 나섰다. 관영 환추(環球)시보는 19일 “이 조사에 미국도 포함해야 한다. 중국은 조사를 두려워하지 않는다”고 맞섰다. 상무부 역시 “향후 5년간 호주산 보리에 반덤핑 관세를 부과하겠다”고 밝혔다. 호주산 보리와 쇠고기의 최대 수입국인 중국은 12일에도 일부 호주산 쇠고기의 수입을 금지했다. 호주가 미국 편에 서서 코로나19 조사를 촉구해 왔다는 이유로 경제 보복에 나섰다는 분석이 나온다. 발끈한 호주 역시 세계무역기구(WTO)에 제소할 의사를 밝혔다.

　미중 갈등의 한복판에 있는 중국 최대 통신장비업체 화웨이는 18일 성명에서 “미국의 화웨이 제재는 자의적이고 치명적이며 결국 미국의 이익도 해칠 것”이라고 주장했다.

　양국 갈등이 미 주식시장으로도 옮겨붙는 모습이다. 로이터통신은 미 2위 증권거래소 나스닥이 중국 기업의 기업공개(IPO) 자격 및 회계감사 등을 강화하는 규제를 조만간 공개할 것이라고 전했다. 중국 금융당국 역시 중국 기업에 영국 런던 증시 상장을 독려하고 있다고 덧붙였다.

# 美구축함, 상하이 인근 해상까지 접근

미국과 중국 간에 경제뿐 아니라 군사 측면에서도 긴장감이 높아지고 있다. 미 군함들이 이례적으로 중국 근해까지 진출했고, 양국이 최신 무기 개발 경쟁에도 나서고 있다.　　

　홍콩 사우스차이나모닝포스트(SCMP)는 16일 베이징대 소속 연구기관인 남중국해전략태세감지계획의 발표를 인용해 미 해군 알레이버크급 구축함인 라파엘 페랄타함(DDG-115)이 최근 상하이(上海)에서 115해리(약 213km) 떨어진 해상까지 접근했다고 보도했다. 미 해군 태평양함대도 전날 트위터에 날짜를 특정하지 않은 ‘라파엘 페랄타함이 이번 주에 동중국해를 항해했다’고 공개했다.

　라파엘 페랄타함의 이번 항해는 중국 인민해방군이 14일부터 황해 보하이만에서 실사격 훈련을 하는 가운데 이뤄졌다. 항공모함이 참여할 것으로 예상되는 중국군의 훈련은 두 달 반 동안 계속된다.

　앞서 지난달 17일 다른 미 해군 구축함인 매케임벨함(DDG-85)도 산둥(山東)성 웨이하이(威海)에서 불과 42해리 떨어진 해상까지 접근한 바 있다. 한 달 새 두 번이나 미 군함이 중국 연안에 바짝 다가선 것이다.

　미국은 지속적으로 군사력 강화를 진행하고 있다. 도널드 트럼프 미국 대통령은 15일(현지 시간) 우주군기(旗) 공개 행사에 참석해 “우리는 지금까지 본 적이 없는 놀라운 군사 장비를 개발 중”이며 “이 기막힌 미사일은 지금 우리가 보유한 것보다 17배 빠르다고 들었다”고 과시했다. 이어 “우리의 적국들이 있기 때문에 해야만 하는 일”이라고 덧붙였다. 중국과 러시아가 극초음속 무기 개발을 추진하는 시점에서 트럼프 대통령이 이런 언급을 한 것은 중국과 러시아를 겨냥한 것으로 보인다고 CNN은 전했다.

　미 국방부는 이에 대한 언론의 질의에 구체적인 답변을 내놓지 않았다. 국방부 대변인인 로버트 카버 공군 중령은 CNN에 “극초음속 무기의 실전 배치는 기술 연구 및 공학에 있어 최우선 사항”이라며 “미국은 극초음속 무기 시스템의 개발을 위한 탄탄한 프로그램을 확보하고 있다”고 강조했다.

# “미친 짓”까지 터져나온 美-中갈등

　미중 갈등이 선을 넘고 있다. “중국과 모든 관계를 끊을 수도 있다”는 도널드 트럼프 미국 대통령의 발언에 중국 언론은 “정신이상자”라고 비난했다. 미국이 관계를 끊으면 대만을 공격하자는 주장까지 나왔다. 미국은 미국 증시에 상장된 중국 기업을 정조준했다.

　중국 런민(人民)일보 자매지 환추(環球)시보는 15일 사설에서 전날 트럼프 대통령의 발언을 “미친 짓”이라고 비판하면서 “중국은 과학기술 및 이와 관련 있는 경제, 인문·사회과학 등 미중 관계의 핵심 분야에서 실질적인 ‘관계 단절’에 대한 준비를 해야 한다”고 주장했다.

　중국 글로벌타임스는 트럼프 대통령을 향해 “궁지에 몰린 짐승 같다” “미친 것처럼 보인다”고 원색적으로 비판했다. 진찬룽(金燦榮) 런민(人民)대 국제관계학원 부원장은 “미국이 일방적으로 관계를 끊으면 중국은 즉각 대만을 (무력)통일할 수 있다. 중국은 미중 관계를 유지하기 위해 대만 문제를 해결하지 않았다”고 말했다.

　트럼프 대통령은 전날 폭스비즈니스 인터뷰에서 미국 증시에 상장하려는 중국 기업들은 미국 회계기준을 준수하도록 의무화하는 방안을 “매우 강하게” 살펴보고 있다고 밝혔다. 뉴욕증권거래소(NYSE)와 나스닥에 상장됐지만 미국 기업에 적용되는 일반회계 기준(GAAP)을 따르지 않는 중국 기업에 대한 제재를 검토할 수 있다는 뜻으로 풀이된다. 반도체, 의약품 등의 생산·공급시설을 미국에 유치해 공급망에서 중국을 제외하려는 움직임도 진행되고 있다.

　대만과 남중국해에서도 미중 간 군사 긴장이 높아져 우발적 충돌 위험이 커지고 있다. 미국은 최근 남중국해에 잇달아 군함과 전략폭격기를 보내는 등 중국에 대한 군사적 압박을 높이고 있다고 CNN이 보도했다.

# 美中갈등 속 시진핑 방한에 매달려선 우리 외교 길을 잃는다

시진핑 중국 국가주석이 그제 문재인 대통령과의 통화에서 “금년 중 방한하는 데 대한 굳은 의지는 변하지 않았다”고 말했다고 청와대가 전했다. 이에 문 대통령은 한중관계에서 시 주석 방한이 무엇보다 중요하다고 했다. 이번 통화는 시 주석의 요청으로 이뤄졌다고 한다.

　양국 정상의 통화는 미중 갈등이 다시 격화되는 미묘한 시기에 이뤄졌다. 무엇보다 중국은 한국을 자기 편으로 끌어들이려는 의도가 역력하다. 한국도 사드(THAAD·고고도미사일방어체계) 갈등을 마무리하기 위해 시 주석의 조속한 방한을 원하고 있다. 그런데 정작 중국 관영매체은 시 주석 방한과 관련한 내용은 전하지 않은 채 양국이 코로나19 공동방역을 두고 ‘비바람 속에서도 한 배를 타고 있다(風雨同舟)’고만 했다.

　코로나19 발원지를 둘러싼 미중 대립은 모든 분야에서 확전을 예고하고 있다. 도널드 트럼프 미국 대통령은 코로나19 중국 책임론을 거론하며 관세 부과는 물론이고 중국에 편중된 글로벌 공급망을 바꾸겠다며 동맹국들의 협조를 요청했다. 중국과의 무역전쟁을 넘어 국제적 무역질서의 재편까지 예고한 셈이다. 이런 갈등 국면에서 한국이 어느 한쪽 편을 들다간 무역 보복을 당하거나 외교안보 공조의 틀이 흔들리는 위기에 빠질 수 있다.

　미중은 벌써부터 글로벌 편 가르기에도 나섰다. 미국은 한국을 비롯한 주요 동맹국 외교장관들을 모아 코로나19 대응을 위한 국제협력에서 중국을 견제하고 미국의 리더십을 따르라는 메시지를 보냈다. 중국도 전면 대응을 시작했다. 중국은 미국 편을 들어 국제조사를 요구한 호주에 대해 쇠고기 수입금지로 보복에 나섰다. 지난해 미중이 화웨이 제품 사용을 두고 국제사회에 선택을 강요하던 일이 보다 큰 폭으로 되풀이되고 있는 셈이다.

　연말 미국 대선 때까지 계속될 이번 미중 갈등은 과거와는 다른 차원의 파장을 낳을 수 있다. 미중 갈등이 무역전쟁에만 집중된다면 우리 경제의 체질 강화로 버틸 수 있겠지만, 이를 뛰어넘는 국제질서 전반의 변동을 낳는다면 선택지가 많지 않다. 양쪽의 러브콜은 우리의 헤엄칠 공간을 넓히는 대신 잘못 운신했다간 설자리를 잃게 되는 양날의 칼과도 같다.

　동맹을 돈으로만 판단하는 트럼프 대통령의 무리한 방위비분담금 요구로 동맹 피로감이 생긴 것도 현실이다. 우리의 선택은 더욱 어려워졌다. 이런 때일수록 지혜로운 외교가 필요하다. 우선 한미동맹을 근간으로 한 우리 외교정책의 좌표를 분명히 하고 그 토대 위에서 한중협력의 구체적 수준을 정해야 한다. 그래야 미중 양국이 막연한 기대감으로 우리를 끌어들이기 위해 압박하는 대신 실제로 협력이 가능한 분야를 두고 협의에 나설 것이다.

# NYT “中, 해커 동원해 美코로나 백신자료 노려”

전 세계가 신종 코로나바이러스 감염증(코로나19) 치료제 개발에 몰두하고 있는 가운데 중국 등 세계 10여 개국이 정부 차원에서 해커를 동원해 백신 관련 정보 쟁탈전을 벌이고 있다고 10일 뉴욕타임스(NYT)가 전했다.

　미국 연방수사국(FBI)과 국토안보부는 조만간 “중국이 미국의 코로나19 치료제 및 백신 개발에 관한 연구 자료를 훔치려 하고 있다. 미국의 관련 지식재산권과 공중보건 자료를 불법적 수단을 통해 획득하려 한다”는 경고문을 발표하기로 했다. 특히 중국이 정보 요원이 아닌 유학생, 교수, 연구원 등을 동원해 미 주요 대학과 민간 연구소의 코로나19 정보를 빼내려 하고 있다고 지적했다.

　미국은 중국의 해외 인재 유치 프로젝트 ‘천인계획(千人計劃)’이 미국의 첨단 기술을 훔치려는 계획의 일환이라고 의심해 왔다. FBI는 조만간 주요 대학을 방문해 보안 강화를 촉구할 것으로 알려졌다. 나노기술의 세계적 석학인 찰스 리버 미 하버드대 교수(61) 역시 1월 ‘천인계획’에 참여한 사실을 고의로 숨긴 혐의로 체포됐다. 그는 2012∼2017년 코로나19 진원지로 꼽히는 후베이(湖北)성 우한이공대에서 수십억 원을 지원받았음에도 “참여 요청을 받은 적이 없다”고 거짓 진술을 했다.

　도널드 트럼프 행정부는 줄곧 코로나19의 중국 유래설을 주장하며 중국과 맞서고 있다. 중국 역시 거세게 반발하고 있어 해킹 논란이 가뜩이나 나쁜 미중 갈등을 더 악화시킬 것이라는 우려도 제기된다.

　NYT는 한국, 이란, 베트남 등도 해커를 동원해 타국의 코로나19 정보 수집에 몰두하고 있다고 전했다. 특히 “한국 해커들이 세계보건기구(WHO)는 물론이고 북한, 일본, 미국 정부 관계자들의 이메일을 해킹해 정보를 수집하려 했다. 미국의 동맹국조차 미국의 통계를 의심하고 있음을 보여준다”고 보도했다.

# 美-中코로나 갈등으로 다시 무역전쟁 전운...수출처 다변화 등 전략짜야

코스피 주가 지수가 4일 다시 1900선이 무너졌다. 그동안 소강상태를 보이던 미·중 무역갈등이 재개될지 모른다는 불안감으로 외국인 투자자들이 국내 주식시장에서 1조 원어치나 팔고 나갔기 때문이다. 코로나19가 다른 나라에 비해 조기에 진정되고 있고, 경제활동도 조만간 본격적으로 재개될 것이라는 기대감을 가져온 한국경제에 찬물을 끼얹는 것이다.

　미중 무역전쟁 재발 가능성에 불을 붙인 것은 코로나19 확산에 대한 중국 책임론을 거론하며 1조 달러(약 1200조원)의 관세를 추가로 부과할 수 있다고 시사한 도널드 트럼프 미국 대통령의 발언이다.　

　한국의 수출은 이미 올 4월 실적이 작년 4월에 비해 23%나 급감했고 무역수지는 2012년 1월 이후 99개월 만에 처음 적자를 나타냈다. 트럼프 대통령이 11월 대선을 앞두고 유리한 여론형성을 위해 중국 때리기를 본격화할 경우 코로나 사태로 이미 수출시장과 글로벌 공급망에서 큰 타격을 입은 우리로서는 또 하나의 초대형 악재가 아닐 수 없다.

　다소 희망적인 부분은 코로나 방역 과정에서 한국의 전반적인 국격이 높아졌고 경제적 저력 또한 세계시장에서 높이 평가받고 있다는 점이다. 예컨대 앞으로 비대면 활동이 늘 것이라는 기대로 삼성전자와 SK하이닉스 2개 회사가 세계 시장에서 70% 이상을 차지하는 메모리반도체 D램의 4월 평균 가격이 39개월 만에 가장 큰 폭으로 올랐다. 반도체와 함께 또 다른 주력 수출상품 가운데 하나인 자동차분야에서도 현대차가 베트남 시장에서 일본 도요타를 제치고 판매 1위를 차지했다는 소식도 들린다.

　그렇다고 해도 우리 앞에는 불안요소가 더 많다. 세계 경기 침체는 이제 시작일 뿐이고 미중 무역갈등은 적어도 미 대선이 끝나는 11월까지는 이어질 가능성이 높다. 그럴수록 우리가 자체적으로 할 수 있고 반드시 해야 할 일은 해야 한다. 당장은 적극적 소비촉진을 통해 내수 경기를 살리고 각종 규제를 풀어 기업의 사기를 북돋우는 작업이다. 길게는 보호무역 색채가 더욱 강해질 포스트 코로나시대의 수출환경에 맞춰 내수 비중을 높이고 수출 시장을 미국과 중국 중심에서 동남아 인도 유럽 등으로 다각화하는 노력에 박차를 가해야한다.

# ‘코로나 우한硏발원설’ 목청 키우는 美...트럼프 “中끔찍한 실수”

신종 코로나바이러스 감염증(코로나19)의 발원지를 둘러싼 미국과 중국 간의 갈등이 폭발 직전으로 치닫고 있다. 도널드 트럼프 미 대통령과 마이크 폼페이오 국무장관이 자극적 표현으로 중국 책임론을 제기하며 조사를 압박하자 중국은 ‘정치 쇼’라고 거칠게 반발했다.

　CNN 등에 따르면 트럼프 대통령은 3일 ‘중국 연구소에서 바이러스가 나왔느냐’는 취재진의 질문을 받고 “중국이 끔찍한 실수를 저질렀다고 생각한다. 그들은 이를 덮으려 했지만 불을 끄지 못했고 실수를 인정하지 않는다”고 주장했다. 이어 “우한 연구소에서 무슨 일이 있었는지에 대한 보고를 받을 것이며 그것이 결정적일 것”이라고 강조했다. 그는 지난달 30일에도 “바이러스가 중국 우한 연구소에서 유래했다는 증거를 봤다”며 관세 보복 등을 거론했다.

　같은 날 폼페이오 장관은 ABC방송 인터뷰에서 “코로나19가 우한의 연구소에서 나왔다는 것을 입증할 ‘엄청난 증거(enormous evidence)’가 있다”며 “중국 연구소의 실패로 세계가 바이러스에 노출된 것은 이번이 처음이 아니다”라고 지적했다.

　그는 중국이 의도적으로 바이러스를 퍼뜨렸는지, 우발적 사고였는지를 묻는 질문에 “알아야 할 것이 많다. 의문을 풀기 위해서라도 현지 조사가 필요하다. 그곳에 가야 한다”며 중국 측을 압박했다. 대통령의 최측근인 집권 공화당의 린지 그레이엄 상원의원 역시 “중국이 우한 연구소 조사에 협조할 때까지 제재를 가하는 법안을 마련했다”고 밝혔다.

　미국 내에서는 코로나19 중국 발원설을 두고 우한 연구소가 생물학적 무기로 사용할 목적으로 바이러스를 만들었다는 설과 우한 연구소에서 사고로 우연히 유출됐다는 설이 나온다. 정치 매체 액시오스는 “생물학 무기설은 가능성이 낮고 사고설은 개연성이 있지만 직접 증거가 나오지 않았다”고 전했다. 트럼프 대통령은 이날 중국의 ‘실수’라고 표현했고, 폼페이오 장관 역시 코로나19가 인공적으로 만들어진 것이 아니라는 것에 대해 “이를 의심할 이유가 없다”며 동의했다. 그럼에도 우발적 사고라면 어떤 식으로 유출됐는지를 알기 위해서라도 우한 연구소 조사가 불가피하다는 뜻을 강조한 것으로 해석된다.

　3일 AP통신에 따르면 미 국토안보부는 “중국 지도부가 의료물품 및 장비 비축을 위해 1월 초부터 의도적으로 코로나19의 심각성을 은폐했다”는 4장짜리 내부 보고서를 작성했다. 중국이 코로나19의 위험성을 세계보건기구(WHO)에 보고하는 것을 일부러 늦추면서 해외로부터 의료장비를 수입했고, 그 결과 올해 초 중국의 마스크 및 보호장갑 수입량이 급증했다는 것이다.

　양측은 대만의 WHO 회의 참석을 두고도 대립하고 있다. WHO 최고 의결기관인 세계보건총회(WHA)는 18일 화상회의를 개최한다. 미 국무부와 주유엔 미국대표부는 2일 트위터에 “대만의 WHO 가입을 지지한다”는 해시태그를 올렸다. 미국의 주대만 대사관 역할을 하는 미국재대만협회(AIT) 역시 이날부터 매일 대만의 WHA 참여를 지지하는 글을 페이스북에 올릴 것이라고 밝혔다.　

　중국은 “미국이 코로나19 이슈를 정치화하고 있다”고 강하게 반발했다. 주제네바 중국대표부는 “이는 (대만이 중국의 일부라는) 하나의 중국 원칙을 위반한 것이고 대만 독립 세력에게 잘못된 신호를 줄 수 있다”고 비난했다.

# 트럼프 “코로나 중서 발생 증거봤다” 보복관세 시사

도널드 트럼프 미국 대통령이 “신종 코로나바이러스 감염증(코로나19)이 중국 우한(武漢) 바이러스연구소에서 발원했다는 증거를 봤다”고 주장하며 대중 관세를 검토하고 있다고 밝혔다. 그가 재선을 위해 강력하고 구체적인 칼을 빼들었다는 분석이 제기된다. 올해 1월 1단계 무역합의를 통해 어렵사리 출구를 찾은 양국 무역전쟁이 다시 전면전으로 비화할 가능성도 배제할 수 없다.

　트럼프 대통령은 지난달 30일(현지 시간) 백악관 기자회견에서 ‘우한연구소 유래설에 대한 증거를 봤느냐’는 취재진 질문에 “그렇다. 나는 봤다”고 두 차례 반복해서 답했다. 그는 “중국이 확산을 막지 못했거나 확산되도록 내버려뒀다”면서도 구체적인 증거는 밝히지 않았다. 그는 관련 조사가 진행 중이라며 “머지않은 장래에 답을 얻을 것이다. 그 결과가 중국에 대해 어떻게 느끼는지를 결정할 것”이라고 설명했다.

　그는 ‘중국을 응징하기 위해 채무 이행 중단을 검토하고 있느냐’는 질문에 “이 같은 일을 할 수 있지만 단지 관세를 부과함으로써 더 많은 돈을 얻을 것”이라고 말했다.

　워싱턴포스트(WP)는 미국이 중국을 대상으로 ‘주권국은 타국 법정의 피고가 될 수 없다’는 국제법의 ‘주권 면제’ 조항을 박탈하는 방안을 논의하고 있다고 전했다. 중국을 미 법정에 세워 손해배상을 받아내겠다는 의도다. CNN 역시 미국이 경제 제재, 채무상환 거부, 새 무역정책 등을 검토하고 있다고 보도했다.

　미 17개 정보기관을 관장하는 국가정보국(DNI)은 이날 “정보기관들은 코로나바이러스가 사람이 만들거나 유전적으로 변형된 것이 아니라는 과학적 합의에 동의한다. 발병이 우한연구소의 사고 결과인지, 감염된 동물과의 접촉으로 시작됐는지 판단하기 위해 조사하겠다”는 성명을 냈다. 사실상 ‘우한 발원설’을 지지했다는 관측이 나온다.

　수차례 ‘백신 개발에 최소 18개월이 걸린다’고 언급한 앤서니 파우치 알레르기전염병연구소(NIAID) 소장은 “내년 1월까지 수억 개의 백신 공급이 가능할 것”이라며 태도를 바꿨다. 역시 대선과 무관하지 않다는 분석이 나온다. 뉴욕타임스(NYT)는 “파우치 소장에 불만을 가진 대통령이 앨릭스 에이자 보건장관에게 직접 연내 개발을 지시했다. 백신이 질병 및 사망을 야기해도 책임을 묻지 않는 식으로 개발을 앞당길 것”이라고 전했다.

　트럼프 행정부의 총공세는 코로나19에 따른 인명 피해 및 경제침체 장기화가 11월 대선에 악영향을 끼칠 것이란 우려 때문으로 풀이된다. 반대파의 화살을 중국으로 돌리고 지지층을 결집시키기 위해 중국을 겨냥했다는 의미다. 트럼프 대통령은 지난달 29일 “중국이 나의 승리를 저지하기 위해 뭐든 할 것”이라고 주장했다.

　중국은 격렬히 반발했다. 우첸(吳謙) 국방부 대변인은 지난달 30일 기자회견에서 “미 정치인이 책임을 회피하고 중국을 비난하는 것은 이기적이고 무책임하다”고 주장했다. 전날 러위청(樂玉成) 외교부 부부장도 NBC 인터뷰에서 “중국에 근거 없는 혐의를 뒤집어씌우지 말라. 중국에 배상금을 요구할 법적 근거가 없는데도 터무니없는 주장을 한다”며 “황당한 정치적 웃음거리”라고 일축했다.

# 트럼프 “中에 코로나 배상책임 물을것...진지하게 조사 진행중”

도널드 트럼프 미국 대통령이 “중국에 신종 코로나바이러스 감염증(코로나19) 확산에 대한 거액의 배상 책임을 묻겠다”고 밝혀 미중 공방이 가열되고 있다.

　폴리티코 등에 따르면 트럼프 대통령은 27일 워싱턴 백악관 기자회견에서 미국이 독일처럼 중국에 배상을 청구할 수 있느냐는 질문에 “금액을 결정하지 못했지만 미국은 독일보다 더 많은 배상금을 요구할 수 있다”며 “코로나19로 인한 피해가 상당하다. 이 피해는 미국에만 국한되는 게 아니라 전 세계적”이라고 답했다. 최근 독일 최대 일간지 빌트는 중국에 코로나19로 인한 경제적 피해 배상금 1490억 유로(약 198조 원)를 청구해야 한다고 주장했다.

　　‘살균제 인체 주입’ 발언 논란으로 지난주 후반 코로나19 일일 정례 기자회견에 등장하지 않았던 트럼프 대통령은 이날 발언의 대부분을 중국 공격에 할애했다. 그는 “중국이 코로나19 확산에 대한 책임을 져야 한다. 우리는 매우 진지하게 조사를 진행하고 있다”고 거듭 강조했다.

　대중 강경파로 유명한 피터 나바로 백악관 무역제조업 정책국장도 폭스뉴스에 출연해 ‘중국이 미국에 저질 코로나19 검사장비를 수출했고 폭리를 취했다’고 비난했다. 나바로 국장은 “이동 통제를 해제하고 미국인이 일터로 복귀하려면 코로나19 진단 검사를 늘려야 한다. 그런데 중국 장비로 검사하면 잘못된 결과가 나온다”며 “중국산 장비가 경제 정상화에 지장을 줄 것”이라고 지적했다.

　국립보건원(NIH)은 24일 중국과 손잡고 코로나19 연구를 수행하던 비영리 연구소 ‘에코헬스얼라이언스’에 대한 자금 지원을 중단했다. 이 연구소는 박쥐가 사람에게 어떻게 코로나바이러스를 전파하는지를 연구해왔다. 미국 정부는 2015년부터 현재까지 370만 달러(약 46억 원)를 지원했지만 올해 보조금 중 아직 쓰이지 않은 약 37만 달러의 사용을 중지하라고 통보했다.

　폴리티코는 이 연구소가 중국 우한바이러스연구소와 손잡은 것이 트럼프 행정부의 심기를 거스른 것으로 보인다고 추측했다. 미국에서는 우한바이러스연구소가 코로나바이러스를 고의로 만들어내거나 유출했다는 주장이 제기되고 있다. 피터 다스작 에코헬스얼라이언스 이사장은 “우한바이러스연구소와 협력했지만 돈을 주고받은 적은 없다”며 억울함을 호소했다.

　트럼프 행정부는 최근 대만과의 협력도 부쩍 강화하고 있다. 앨릭스 에이자 미 보건장관은 이날 천스중(陳時中) 대만 위생부장과 통화했다. 코로나19 백신 및 치료제 개발 등에서 양국 협력을 강화하기 위한 목적이다. 블룸버그통신은 “두 장관이 30분간 대화를 나눴다. 대만에 대한 미국의 강력한 지지를 보여준다”고 평가했다.

　대만 정부는 미국이 대만의 세계보건기구(WHO) 가입 및 국제보건 현안의 참여 기회를 늘려줄 것이라는 기대를 드러내고 있다. 중국 정부가 주장하는 ‘하나의 중국’ 원칙 때문에 대만은 WHO의 정식 회원국이 되지 못하고 ‘옵서버’ 지위만 갖고 있다. 대만은 친중 성향으로 유명한 테워드로스 거브러여수스 WHO 사무총장이 중국의 지지를 받아 2017년 7월 취임한 후 “WHO로부터 노골적인 홀대를 받고 있다. 코로나19 발발 초기에도 제대로 된 정보를 공유받지 못했다”는 불만을 제기해왔다.

# 하얼빈 71명 집단감염에... 中, 다시 ‘準봉쇄조치’

중국이 신종 코로나바이러스 감염증(코로나19) 재확산을 막기 위해 인구 1085만 명인 헤이룽장(黑龍江)성 성도 하얼빈(哈爾濱)시에 대해 준(準)봉쇄 조치를 취했다. 하얼빈시에서는 최근 해외에서 돌아온 감염자 1명이 71명을 연쇄·집단 감염시키는 사태가 발생했다.

　하얼빈시 정부는 22일 “시내 모든 주거단지와 농촌마을의 출입구를 통제해 외부인과 외부인 차량의 진입을 금지한다”고 밝혔다. 결혼식과 장례식도 금지했다. 공연, 경기, 포럼, 전시 등 대형 행사도 중단시켰다.

　중국은 그동안 “코로나19 통제에 성공했다”며 이동 통제 조치를 완화해 왔다. 하지만 해외 유입에 이은 집단 감염이 다시 발생하면서 2차 유행 가능성이 우려되자 화들짝 놀란 중국 당국이 다시 강경 조치를 꺼내 든 것이다.

　하얼빈시의 연쇄·집단 감염은 지난달 미국에서 홍콩과 베이징을 거쳐 돌아온 유학생 한(韓·여·22)모 씨로부터 시작됐다. 가족과 이웃들이 감염됐고, 이들 중 일부가 방문한 하얼빈 시내 대형 병원 2곳에서 집단 감염이 발생했다. 이로 인해 하얼빈시뿐 아니라 랴오닝(遼寧)성 푸순(撫順)시와 네이멍구(內蒙古)자치구에서도 환자가 각각 1명씩 발생했다. 하얼빈시 정부는 병원 내 집단 감염 당시 이곳에 있었던 4106명의 감염 여부를 조사하고 있다.

　특히 한 씨는 이달 초 상하이(上海)에도 다녀온 것으로 알려졌다. 중국 내 코로나 재유행 범위가 확산될 수 있다는 우려가 나왔다.

# 거세지는 ‘中코로나 책임론’... 메르켈 “中투명하게 공개해야”

신종 코로나바이러스 감염증(코로나19) 확산에 대한 중국의 책임론이 갈수록 거세지고 있다. 미국에 이어 유럽 각국 정상들도 코로나19 사태의 축소 의혹을 명확히 밝히라고 촉구했다.

　로이터통신 등에 따르면 앙겔라 메르켈 독일 총리는 20일 “중국 정부는 코로나바이러스 기원과 초기 확산을 투명하게 공개해야 한다. 그래야 세계 모두가 교훈을 얻어 상황이 나아질 것”이라고 밝혔다. 독일에서는 최근 대표 일간지인 빌트 등 언론을 통해 ‘코로나 중국 책임론’이 확산되기도 했다.

　에마뉘엘 마크롱 프랑스 대통령은 앞서 17일 파이낸셜타임스(FT)와의 인터뷰에서 “중국이 (코로나19 사태에) 더 잘 대응했다는 건 순진한 생각”이라며 “우리가 알 수 없는 일들이 벌어지고 있다”고 지적했다. 도미닉 라브 영국 외교장관도 16일 기자회견에서 “중국에서 어떻게 바이러스가 퍼졌는지 철저한 분석이 필요하다”고 밝혔다.

　유럽은 미국과 달리 코로나19 사태와 관련해 중국 비판을 자제해 왔다. 2017년 도널드 트럼프 대통령 취임 이후 미국과의 관계가 벌어지면서 중국에 대한 의존도가 커진 탓이다. 유럽의 기조가 바뀐 데 대해 미국 외교전문매체 포린폴리시는 “중국이 발표한 코로나 관련 데이터들은 신뢰를 잃었다. 이를 토대로 세운 각국의 코로나 대책도 잘못 만들어졌다”고 설명했다. 중국은 코로나 발병 시기와 누적 사망자 숫자 등을 명확히 밝히지 않아 불신을 키운 바 있다.

　코로나가 자국에서 발병했음에도 자국 체제를 선전하고 다른 국가를 깎아내린 점도 반감을 샀다. 최근 주프랑스 중국 대사는 홈페이지에 “서구의 코로나 대응은 느림보”라는 글을 올려 프랑스 정부의 항의를 받았다. 인도적 명분을 내세워 의료물품을 지원해 의도적으로 유럽연합(EU)의 분열을 노린다는 지적도 있다. 일각에서는 서구사회에 대한 가짜뉴스를 퍼뜨린다는 의혹도 나온다.

　자국 내 여론 환기를 위해 중국으로 화살을 돌리고 있다는 분석도 나온다. 미국을 비롯해 프랑스 독일 영국 등의 코로나 사망자만 수만 명에 달할 정도로 피해가 크다. FT는 “서방 정치인들은 베이징을 공격해 자신들의 실패를 피하면서 비판 대상을 분산시키고 있다”고 전했다.

　중국에 대한 비판이 전 세계로 확산되자 중국을 옹호해 온 세계보건기구(WHO)는 난처한 입장에 빠졌다. 테워드로스 아드하놈 거브러여수스 WHO 사무총장은 20일 스위스 제네바 본부 언론 브리핑에서 “우리는 미국에 (정보를) 감춘 것이 아무것도 없다”고 강조했다.

# 中1분기―6.8% 사상첫 마이너스 성장

신종 코로나바이러스 감염증(코로나19) 사태 충격으로 중국의 1분기(1∼3월) 경제성장률이 통계 작성 이후 처음으로 마이너스 성장을 기록했다. 중국 정부는 고강도 경기 부양 조치를 예고했다.

　중국 국가통계국 마오성융(毛盛勇) 대변인은 17일 기자회견에서 “1분기 국내총생산(GDP) 총액이 20조6504억 위안(약 3554조 원)으로 지난해 같은 기간에 비해 6.8% 감소했다”고 밝혔다. 중국이 분기별 경제성장률을 발표하기 시작한 1992년 이후 28년 만의 첫 마이너스 성장이자 최저치다. 지난해 4분기 경제성장률인 6%에 비해서는 12.8%포인트 급락한 것이기도 하다.

　중국의 연간 경제성장률이 마이너스를 기록한 것은 문화대혁명으로 중국 경제가 붕괴 직전까지 갔던 1976년(―1.6%)이 마지막이었다. 올해 전체 경제성장률이 마이너스를 기록한다면 44년 만이다.

　마 대변인은 “1분기 2차산업(제조업) 생산이 9.6%, 3차산업(서비스업) 생산이 5.2% 감소했다”고 말했다. 1분기 산업생산은 지난해 같은 기간에 비해 8.4% 하락했고, 소비 수요를 보여주는 소매 판매는 19%나 하락했다.

　중국 외교부는 21일 오후 “국내에 있는 사람들은 해외여행을 하지 말라. 해외에 있는 사람은 다른 나라로 이동하지 말라”고 발표했다. 상황이 심상치 않자 다음 달 1일 노동절 연휴를 앞두고 중국인들의 국경 간 이동 금지 조치를 발표한 것으로 해석된다

# 中우한 코로나 사망자 축소 의혹 사실로

중국이 신종 코로나바이러스 감염증(코로나19) 사망자 수를 축소해 왔다는 의혹이 사실로 드러났다. 중국 신화(新華)통신에 따르면 17일 후베이(湖北)성 우한(武漢)시 정부는 16일까지 우한시의 누적 사망자 수가 기존 공식 발표보다 1290명이 많은 3869명이라고 밝혔다. 우한시의 누적 확진자 수는 기존보다 325명 많은 5만333명이라고 공개했다.

　중국 보건 당국인 국가위생건강위원회(위건위)가 같은 날 2시간 전 발표한 16일까지의 우한시 누적 사망자 수는 2579명이었다. 기존 공식 누적 사망자의 절반에 해당하는 숫자가 지금까지 집계에 포함되지 않은 셈이 된다.

　우한시 당국은 시 위건위와 질병예방통제센터, 공안(경찰) 등 여러 부서가 병원, 양로원, 장례 정보 시스템 등을 조사했다고 밝혔다. 우한시 당국은 △코로나19 사태 초기 환자 급증으로 환자들이 입원하지 못한 채 집에서 사망했고 △병원 과부하로 의료진의 지연 보고, 보고 누락, 오보(誤報) 현상이 나타났으며 △사망자 정보가 부정확해 중복되거나 잘못 보고 됐기 때문이라고 해명했다. 하지만 당국이 책임을 회피한 채 사망자 수 누락의 책임을 일선 의료진에게 떠넘긴 것 아니냐는 비판이 나왔다.

　중국의 코로나19 통계는 그동안 오락가락 집계와 은폐 의혹으로 불신이 컸다. 중국 매체 차이신(財新)은 최근 공식 사망자 수보다 최소 2배 많은 유골함이 운반됐다고 의혹을 제기했다.

# 中기습 입국금지에 기업인 등 대혼란

중국이 신종 코로나바이러스 감염증(코로나19) 방역을 이유로 사실상 국경을 봉쇄하는 초강경 조치를 발표했다. 중국 현지에서 생산 공장 및 판매법인을 운영하고 있는 국내 기업뿐 아니라 현재 중국 밖에서 머물고 있던 한국 교민 및 유학생들도 중국 입국이 사실상 불가능해지면서 큰 혼란을 피할 수 없게 됐다.

　이날 중국 외교부와 국가이민관리국은 28일 0시를 기준으로 중국 체류 비자와 거류허가증을 가진 외국인도 중국 입국을 금지하기로 했다. 중국은 경제무역, 과학기술 활동 및 긴급한 인도주의 사유가 있으면 현지 중국 대사관, 영사관에 예외적으로 비자를 신청할 수 있다고 밝혔다.

　하지만 주중한국대사관은 “중국 측에 어떤 경우에 예외 조치로 비자를 신청할 수 있는지 구체적인 설명을 요구했지만 중국 측이 분명한 답을 하지 않았다”고 전했다.

　중국 입국 금지 조치는 시진핑(習近平) 중국 국가주석을 포함한 주요 20개국(G20) 정상들이 화상 정상회의를 끝낸 직후 공동성명을 통해 “국제무역을 촉진하고 국가 간 이동과 무역에 불필요한 장애를 유발하지 않는 방식으로 대응하기 위해 협력할 것”이라고 밝힌 지 불과 약 1시간여 만에 발표됐다.

　중국의 기습적 입국금지 조치에 한국 정부도 적잖게 당황하는 분위기다. 외교 고위당국자는 27일 기자들과 만나 “어제(26일) 갑작스럽게 입국금지를 통보 받았다. 사전에 통보를 받지 못한 것에 대해 유감스럽게 생각한다”고 말했다.

# 美확진자 8만5000명...中넘어 세계 최다

26일(현지 시간) 미국의 신종 코로나바이러스 감염증(코로나19) 확진자 수가 8만5000명을 넘어서며 세계 최대 감염국이 됐다. 1월 21일 미국에서 첫 환자가 발생한 후 65일 만이다.

　글로벌 통계 사이트 월드오미터 등에 따르면 이날 미국 확진자 수는 전일 대비 1만6939명 증가한 8만5520명으로 중국(8만1340명)을 앞질렀다. 사망자는 261명 증가한 1297명이다. 이달 19일 1만 명을 돌파한 지 1주일 만에 8만 명을 넘어서는 등 증가 속도가 가파르다.

　미국 최대 도시 뉴욕이 속한 뉴욕주 환자가 3만7258명에 달한다. 데버라 벅스 백악관 코로나19 책임자는 “미 전체 감염자의 55%가 뉴욕과 인근 뉴저지주에서 발생했다. 시카고와 디트로이트의 환자 급증세도 예사롭지 않다”고 밝혔다. 의료진 및 의료용품 부족도 심각해 이날 앤드루 쿠오모 뉴욕주지사는 산소호흡기 1개를 환자 2명이 함께 사용하는 방안을 승인했다.

　블룸버그통신은 도널드 트럼프 행정부의 안이한 현실 인식, 허술한 초동대처가 현 상황을 야기했다며 “코로나19는 트럼프의 베트남전”이라고 질타했다. 하지만 트럼프 대통령은 이날 기자회견에서도 “다음 달 12일 부활절 전 코로나19 확산 방지를 위한 사회적 거리 두기 조치를 풀겠다”는 기존 입장을 고수해 비판이 일고 있다.

　이탈리아(8만589명)도 감염자 증가 속도가 빨라 조만간 중국을 앞지를 것으로 전망된다. 일본 감염자는 하루 만에 역대 최고치인 96명 증가해 2099명(다이아몬드 프린세스호 포함)으로 늘었다.

# 세계화 최대 수혜자 중국에 닥친 2차 위기

최근 중국 소셜네트워크서비스(SNS) 웨이보에서는 이탈리아에서 유학 중인 중국인 야오야오(瑤瑤·가명) 씨의 귀국기가 화제다. 이탈리아의 신종 코로나바이러스 감염증(코로나19) 상황이 심각해지자 그는 고향인 광둥(廣東)성 선전으로 돌아가기로 결심했다.

　아부다비, 베이징(北京)을 거쳐 선전에 도착할 때까지 28시간을 야오야오 씨는 아무것도 먹지도 마시지도 않고 마스크를 한 번도 벗지 않은 채 버텼다. 야오야오 씨의 웃지 못할 사연에 중국 누리꾼들은 “교과서식 귀국”이라며 박수를 쳤다. 하지만 그처럼 “중국이 가장 안전하다”며 유럽과 미국을 ‘탈출’하는 중국인이 크게 늘자 이들로 인한 코로나19 역유입도 증가세다. 중국 내 코로나19 신규 감염자 수가 ‘0’이라고 선전하던 중국 당국이 화들짝 놀랐다.

　이에 중국 당국은 입국 통제 조치를 전면화했다. 외국발 베이징행 항공기들이 베이징에 바로 착륙하지 못하게 하는 제한 조치도 시작했다. 중국 상황이 심각할 때 중국에 문을 걸어 잠근 세계 각국을 중국이 비판했던 것과는 상반된다. 하늘길이 통제되자 중국 항공업체들의 타격이 현실화됐다. 지난달 에어차이나 등 중국 대표 항공사들의 승객 수가 지난해 같은 기간에 비해 80% 이상 급감했다.

　코로나19로 세계 각국이 국경을 닫거나 통제하자 글로벌 공급망이 타격을 입기 시작했다. 코로나19 확산세가 멈춘 중국은 공장을 다시 가동한다. 하지만 세계 공급망 파괴로 원재료가 부족해 실제로는 상당수 공장이 제대로 운영되지 못한다. 중국 화난(華南) 지역 미국 상공회의소의 최근 조사에 응한 중국 남부지역 기업 237곳 가운데 32%가 원재료 공급 부족에 직면했다.

　코로나19로 해외 각국에서 소비가 줄어든 것도 중국 경제에는 악영향을 주고 있다. 중국의 제조업 중심지 항저우(杭州)의 한 자동차 부품 관련 기업은 “지난달부터 공장을 가동했지만 중국 내 수요 감소뿐 아니라 한국 일본에 대한 수출도 지장을 받아 평상시 주문의 30%만 들어온다”고 미국 워싱턴포스트에 밝혔다. 미국 코어사이트리서치에 따르면 미국 신발 제조업체인 스티브매든은 제품 73%를, 미국 전자제품 기업 베스트바이는 60%를 중국에서 생산한다. 코로나19로 미국 내 수요가 감소하면 중국 내 공장 생산도 줄어 중국 경제도 피해를 입는다는 것이다.

　중국 정부는 ‘코로나19 퇴치전에서의 승리’를 선언하려고 하지만 이제 글로벌 공급망 파괴와 소비 급감이 중국 경제에 미칠 타격을 걱정해야 하는 처지가 됐다. 세계화의 가장 큰 수혜자인 중국은 세계화 단절의 가장 큰 피해자가 될 수 있기 때문이다. 코로나19의 대규모 확산이라는 1차 위기 이후 중국이 직면한 이 2차 위기는 1차 때와 달리 중국 정부 혼자 해결할 수 없다.

　그런데 생산·소비·무역의 상호의존도가 가장 큰 세계 1, 2위 경제대국 미국과 중국이 코로나19 대처와 발원지 문제로 책임 회피와 소모적 갈등을 되풀이하고 있다. 미중이 이런 식으로 계속 싸우면 다른 나라들이 글로벌 경제 위기에 열심히 대처하려고 해도 효과가 떨어질 것이라는 우려가 크다.

# 싱하이밍 주한 中대사 “한국 기업인 격리 면제할수도”

싱하이밍(邢海明) 주한 중국대사(사진)가 신종 코로나바이러스 감염증(코로나19)과 관련해 “중국은 상대국에서 근무하는 외국 국민들의 필수적인 왕래를 보장하며 중국을 방문하는 한국 국민들에게 맞춤형 편리를 제공할 것”이라고 밝혔다. 정부가 코로나19 음성 확인서나 건강증명서를 지참한 기업인에 대해 예외적으로 입국을 허용하도록 중국과 협의 중인 가운데 중국 고위 당국자가 이에 대해 긍정적인 입장을 시사한 것은 처음이다.

　싱 대사는 17일 주한 중국대사관에서 진행된 동아일보와의 단독 인터뷰에서 한국 기업인에게 입국 시 격리 조치를 면제하는 방안에 대해 “한국에서 검사하고 (중국에) 내려서 한 번 더 검사해 발열 증상이 없으면 그렇게 되지 않을까 한다”며 이같이 말했다. 중국은 17일 현재 24개 지방정부(성·시·자치구)에서 한국발 입국자들을 14일간 격리하고 있다. 싱 대사는 “한국이 한중 코로나19 대응 방역협력체제 1차 화상회의에서 건강확인증으로 문제가 없다는 것을 입증한다면 입국을 허용하는 원칙을 제언했고, 중국 정부는 이를 진지하게 검토하고 있다”고 전했다.

　시진핑(習近平) 중국 국가주석의 방한과 관련해 싱 대사는 “시 주석은 이미 문재인 대통령의 방한 요청을 수용했기 때문에 반드시 한국을 방문할 것”이라면서도 “시기는 결정된 상황이 아니다”라고 했다. 또 방한 시기가 방일과 연계돼 있느냐고 묻자 “그렇지 않다”고 말했다.

# 시진핑 “韓中은 같은 배 탄 우호국...힘닿는한 돕겠다”

시진핑(習近平) 중국 국가주석이 신종 코로나바이러스 감염증(코로나19)과 관련해 문재인 대통령에게 감사와 위로를 담은 전문을 보냈다.

　14일 관영 중국중앙(CC)TV에 따르면 시 주석은 “중한은 같은 배를 탄 우호 국가”라며 “한국 정부와 사회 각계각층은 그동안 잇달아 중국의 방역 상황에 안부를 묻고 많은 도움을 줬다”고 했다. 지난달 20일 한중 정상 통화 당시 문 대통령이 “중국의 어려움이 우리의 어려움”이라며 코로나19 극복을 위한 양국 공조를 강조한 데 이어 시 주석이 전문을 통해 이를 재차 언급한 것이다.

　시 주석은 “감염병에는 국경이 없고 세계 각국은 동고동락하는 운명 공동체”라며 “중국 정부와 인민은 한국이 현재 겪고 있는 어려움에 공감하며 힘닿는 데까지 (한국을) 돕겠다”고 강조했다. 이어 “나는 중한 관계 발전을 매우 중시하고, 문 대통령과 함께 노력해 양국의 전략적 협력 동반자 관계를 더 높은 수준으로 끌어올리길 바란다”고 덧붙였다.

　앞서 양국은 13일 ‘한중 합동 코로나19 방역 체계’를 출범시키고 외교부와 방역당국 등 관계부처가 참석한 가운데 ‘한중 코로나19 대응 방역 협력 대화(국장급)’ 화상회의를 개최했다.

# 中외교부 “日의 입국제한 이해”...문제제기 안해

일본이 한국과 함께 중국에 대해서도 사실상 입국 금지 조치를 취했지만 중국의 반응은 한국 정부와 전혀 다르다. 정부와 언론매체 모두 “이해할 수 있다”며 문제 제기를 하지 않았다. 최근 가까워지고 있는 중일 관계를 상징적으로 보여주는 장면이라는 분석이 나온다.

　중국 외교부 자오리젠(趙立堅) 대변인은 5일 일본의 입국 제한 조치에 대해 “중국이든 일본이든 자국과 외국 시민의 건강과 안전, 지역과 세계 공공 보건 안전을 위해 과학적이고 전문적인 조치를 취하는 데 대해 여러분 모두 이해할 수 있을 것”이라고 밝혔다. 그는 “양국은 외교 경로로 긴밀히 소통을 유지하고 있다”고 밝혀 일본이 중국에 미리 조치를 알려왔음을 시사했다.

　배타적 성향의 환추(環球)시보도 아베 신조(安倍晋三) 일본 총리가 발표한 한국, 중국발 승객 14일 격리 소식을 전한 소셜네트워크서비스(SNS) 공식 계정 기사의 제목을 “이해할 수 있다”고 달았다.

　중국 SNS 웨이보에는 비자 효력 취소에 따른 우려와 문의가 잇따랐지만 일본의 조치를 노골적으로 비난하는 글은 찾기 어려웠다. 한 중국 누리꾼은 관련 기사에 “중국이 비상시기에 먼저 저렇게 했다. 일본의 조치는 비난할 일이 아니다. 한국도 저렇게 할 것을 제안한다”는 댓글을 달았다.

　중국 정부와 매체의 반응은 신종 코로나바이러스 감염증(코로나19)의 역유입을 막는다는 명분으로 이미 중국 여러 지역에서 한국과 일본발 승객에 대해 14일 강제 격리 조치를 시행하는 것을 정당화하기 위한 측면이 있다.

　이와 함께 2018년 10월 아베 총리가 중국을 방문해 ‘새로운 중일관계’를 선언한 이후 양국 관계가 꾸준히 개선돼온 흐름과도 무관치 않다는 분석이 나온다. 중국은 미중 갈등 속에서 일본을 끌어들이려 했고, 일본 역시 미국의 동맹 홀대 속에서 경제·안보를 위해 중국과의 관계 개선이 필요한 상황이다. 실제 코로나19가 중국에서 본격적으로 확산되기 시작한 1월 일본은 중국에 가장 먼저 마스크 등 방역 물품 지원 의사를 밝혔다. 당시 중국 정부는 “어려울 때 친구가 진짜 친구”라며 일본에 대한 감사를 표했다.

# “시진핑 방한 변함 없지만 코로나 극복 안되면 영향”

신종 코로나바이러스 감염증(코로나19) 사태 장기화로 시진핑(習近平·사진) 중국 국가주석의 상반기 방한 계획이 영향을 받을 수 있다는 기류가 외교 당국에서 확산되고 있다.

　외교 고위당국자는 3일 기자들과 만나 “일본 언론에 4월 중으로 추진되던 시 주석의 방문 계획이 연기된다는 보도가 많이 나오고 있다”며 “(코로나19) 사태가 빨리 극복되지 않는다면 (시 주석 방한 일정에도) 영향이 있을 수도 있지 않을까 생각해 보게 된다”고 말했다. 코로나19 사태로 인해 4월로 추진하던 시 주석 방일 일정을 가을로 미루는 것을 중일이 조율하고 있다는 외신 기사를 거론한 것이지만, 정부 고위 당국자가 시 주석 방한도 코로나19의 영향을 받을 수 있다고 언급한 것은 처음이다. 다만 이 고위 당국자는 “기존의 협의 틀 속에서 변함없이 (상반기 방한) 일정을 추진하고 있다”고 덧붙였다.

　올해로 수교 30주년을 맞은 러시아와의 외교 일정도 이른 시일 내 구체화하기는 어려워졌다는 평가가 나온다. 정부는 세르게이 라브로프 러시아 외교장관의 방한을 이르면 이달 중으로 추진하려 했으나 코로나19 여파로 어려워진 것으로 전해졌다. 이외에도 낮은 급에서의 외교 일정 다수는 이미 연기 및 취소되고 있는 것으로 알려졌다.

　외교 당국이 미국의 한국인 입국금지 등의 가능성에 촉각을 곤두세운 가운데 미국은 당분간 ‘검사 강화’ 수준에서 대처에 나선다는 입장을 밝혔다. 마이크 펜스 미 부통령은 2일(현지 시간) 기자회견에서 “이탈리아와 한국에서 오는 모든 직항편에 대해 공항에서 100% (발열) 검사가 이뤄질 것”이라고 말했다. 다만 도널드 트럼프 미 대통령은 같은 날 ‘여행 제한 강화를 검토하느냐’는 취재진의 질문에 “(코로나19가) 더 많이 발병하고 있는 특정 국가들에 대해 그렇게 할 것”이라고 답해 추가 대응 가능성을 시사했다.

　일본 외무성은 2일 경북 경산시, 영천시, 칠곡군, 의성군, 성주군, 군위군 등 6개 지역의 감염증 위험정보를 기존 ‘레벨1’에서 ‘레벨3’으로 올렸다. 레벨3은 방문 중지를 권고하는 수준으로 4단계 중 두 번째로 심각한 단계다.

　3일 오후 기준 한국인의 입국을 금지하거나 격리 등에 나선 국가는 총 89개국인 것으로 집계됐다. 유엔 회원국(193개국) 절반에 달하는 46% 정도다. 해외에서 자가 및 지정시설에 격리 조치를 당하고 있는 한국인은 3일 오전 기준 1200명을 넘는다고 외교부는 밝혔다. 중국에서 960명, 베트남에서 270명가량이 격리 중이며, 러시아 키르기스스탄 카타르 등지에 각각 10여 명이 격리 중인 것으로 알려졌다.

# 시진핑 “코로나19 발원지 찾아라” 지시

시진핑(習近平) 중국 국가주석이 신종 코로나바이러스 감염증(코로나19)의 발원지를 밝혀내라고 중국 과학자들에게 지시했다. 최근 중국 관영매체와 당국 관계자들을 중심으로 중국이 발원지가 아닐 수 있다는 주장이 제기되는 가운데 나온 지시여서 배경이 주목된다.

　2일 관영 중국중앙(CC)TV에 따르면 시진핑은 이날 코로나19 관련 연구를 진행 중인 베이징(北京)의 군사의학연구원과 칭화(淸華)대 의학원을 시찰하면서 “인공지능(AI)과 빅데이터 등 새로운 기술을 활용해 (코로나19의) 근원을 추적하라. (코로나19) 병원(病源)이 어디에서 왔는지, 어디로 가는지 명확히 밝히고 검사의 정확도와 효율을 높이라”고 주문했다.

　일부 전문가들은 “코로나19의 발원지가 여러 곳일 수 있고, 발원 동물(숙주) 역시 여러 종일 수 있다”는 의견을 냈다.

　중국 환추(環球)시보는 이달 초 “미국이 발원지일 수 있다”는 주장까지 펼쳐 코로나19가 세계로 확산되자 중국이 책임론에서 벗어나려는 의도라는 해석이 나온다.

# 中최상급 보건당국 “에어로졸 전파 가능성 존재”

중국의 보건당국 중 가장 상급기관이 에어로졸(대기 중에 떠도는 미세한 입자)에 의한 신종 코로나바이러스 감염증(코로나19) 전파 가능성을 처음으로 공식 인정했다.

　중국 국가위생건강위원회(위건위)는 19일 발표한 ‘코로나19 진료 방안(제6판)’에서 전파 경로에 관해 “상대적으로 밀폐된 환경에서 장시간 고농도의 에어로졸에 노출되면 에어로졸 전파 가능성이 존재한다”는 대목을 추가했다고 밝혔다.

　위건위는 4일 발표한 진료 방안(제5판)에서는 “에어로졸 전파 경로는 아직 명확하지 않다”고 밝혔다. 하지만 이후 전문가와 일부 지방정부가 에어로졸 감염 가능성을 제기했다. 8일에는 상하이(上海)시 정부가 “주요 전파 경로는 비말(침방울)을 통한 직접 전파, 에어로졸 전파, 접촉 전파로 확정할 수 있다”고 밝혔다. 하지만 다음 날인 9일 위건위가 “에어로졸 전파를 보여주는 증거가 없다”고 반박하면서 논란이 불거졌다.

　비말은 상대적으로 무거워 통상 2m 범위를 벗어나지 못하는 반면 에어로졸은 가벼워 멀리 퍼진다. 사무실 등 밀폐된 실내 공간에 남아 있는 에어로졸을 통한 집단 감염이 일어날 수도 있다.

　위건위는 또 후베이성에만 적용하던 ‘폐렴 증상이 있는 임상 진단 환자’ 분류 대목을 삭제한다고 밝혔다. 후베이성이 13일 이 항목으로 분류되는 환자를 확진 환자에 포함하면서 하루 사이에 후베이성의 환자가 1만4840명이나 늘어났다. 하지만 새로 나온 진료 방안을 적용하면 후베이성도 임상 진단 환자를 확진 환자에 포함시킬 필요가 없어져 통계상 후베이성의 환자 규모를 줄이려는 의도가 아니냐는 지적이 나온다.

# 환자 9배 폭증...중 신뢰잃은 ‘코로나 통계’

중국이 신종 코로나바이러스 감염증(코로나19) 확진 환자 판정에 대한 기준을 바꾸면서 후베이(湖北)성의 감염자와 사망자 수가 폭증했다. 코로나19 발생지인 우한(武漢) 등 후베이성의 코로나19 확산세가 알려진 것보다 훨씬 심각하고 그동안 실상을 은폐·축소해온 것 아니냐는 의혹이 제기됐다.

　후베이성 위생건강위원회는 12일 하루 사이 확진 환자는 1만4840명, 사망자는 242명 늘었다고 13일 발표했다. 11일에 비해 확진 환자 수는 약 9배, 사망자 수는 약 2.6배 증가한 것이다. 매일 오전 공식 집계를 발표해왔던 국가위생건강위원회는 이날 오후까지도 통계를 발표하지 않았다.

　후베이성은 “환자들이 제때 확진 판정을 받아 치료 성공률을 높이기 위해 임상(치료) 진단을 확진 판정 기준으로 추가했다”고 해명했다. “그동안 핵산 검사를 통해 확진 판정을 내렸지만 이제 의료진의 판단 및 컴퓨터단층촬영(CT) 영상을 통해 확진 판정을 내릴 수 있도록 기준을 바꾸면서 확진자가 대폭 늘었다”는 설명이다. 후베이성에 따르면 이런 ‘임상 진단’에 따른 확진 환자와 사망자는 각각 이날 증가 환자의 약 90%(1만332명)와 약 56%(135명)를 차지했다.

　후베이성은 “전국 다른 성(省)이 공표한 확진 판정 기준에 맞추기 위해 13일부터 ‘임상 진단’ 환자를 확진 환자에 포함시켜 발표하기로 했다”고 밝혔다. 다른 성에서 이미 적용해 왔던 확진 판정 기준을 이제야 적용했다고 밝힌 것이다. 특히 이 확진 분류의 근거인 중국 국가위생건강위원회 ‘코로나19 진단 방안’(제5판)은 일주일여 전인 4일에 이미 나온 것으로 확인됐다. 홍콩 사우스차이나모닝포스트(SCMP)는 이날 “우한 등에서 코로나19로 의심되는 폐렴 환자와 사망자가 나와도 과부하에 걸린 의료진이 일반 폐렴으로 분류해 왔다”고 지적했다.　

　또 일본 후생노동성은 13일 크루즈선 ‘다이아몬드 프린세스’호에서 신규 감염자가 44명(승객 43명, 승무원 1명) 나왔다고 밝혔다. 이로써 크루즈선에서만 확진 환자가 218명 나왔고, 일본 내 감염자 수는 총 247명으로 늘었다.

　한국에서는 이날 추가 감염자가 확인되지 않았지만 보건당국과 의료계는 중국의 코로나19 확진 환자와 사망자 급증에 긴장하는 모습이다. 질병관리본부는 중국 보건당국에 환자 수 급증에 대한 확인을 요청했다고 13일 밝혔다.

# 中환자 증가폭 매일 신기록...홍콩서도 첫 사망자

중국에서 신종 코로나바이러스 감염증(신종 코로나)으로 인한 사망자가 400명을 넘어섰다. 홍콩에서는 신종 코로나로 인한 첫 사망자가 나왔다. 중국 본토 외에서 사망자가 발생한 것은 필리핀에 이어 두 번째다.

　4일 중국 내 확진 환자 수는 전날보다 3136명 증가한 총 2만438명에 달했다. 하루 환자 증가 수가 처음으로 3000명을 넘어섰다. 사망자는 모두 426명으로 전날보다 65명이나 늘었다. 하루에 발생한 확진 환자와 사망자 수 모두 최고 증가치를 보여 갈수록 증가치가 높아지는 추세다. 중국 정부가 공식적으로 밝힌 중증 환자가 2788명에 달하는 데다 신종 코로나 발생지인 후베이(湖北)성 우한(武漢)시에서는 5%가 넘는 사망률을 보이고 있어 앞으로도 사망자 증가세가 이어질 것이라는 우려가 나왔다.

　또 홍콩 밍(明)보에 따르면 우한을 방문한 적이 있는 39세 남성이 지난달 31일 확진 판정 뒤 치료를 받다가 이날 사망했다. 홍콩은 전날 선전(深(수,천))만 검문소 등 2곳을 제외하고 중국 본토와 연결되는 모든 검문소를 폐쇄하겠다고 발표했으나 홍콩 내에서 중국 본토와의 접경을 완전 봉쇄하라는 요구가 거세지고 있다.

# 中대사 “입국제한, WHO 근거 따라야”

“한국이 취한 (중국인 일부 입국 제한) 조치에 대해 제가 많이 평가하지 않겠다.”

　싱하이밍(邢海明) 주한 중국대사는 4일 주한 중국대사관에서 가진 기자회견에서 이날부터 시행된 정부의 후베이(湖北)성 방문 외국인 입국 제한 방침에 대한 중국의 입장을 묻는 질문에 이같이 답했다. 신종 코로나바이러스 감염증(신종 코로나) 확산으로 한국 내 중국 비판 여론이 감지되는 상황에서 적극적인 반대 의사를 표하기보다 비교적 낮은 수위로 불쾌감을 표한 것이다.

　싱 대사는 이날 세계보건기구(WHO) 입장을 인용하는 방식으로 한국이 강경한 입국제한 조치를 취하지 않길 바란다는 뜻을 밝혔다. 그는 “(WHO는) 이번 전염병이 국제여행 등을 불필요하게 방해할 이유는 없다고 했다”며 “세계적이고 과학적인 것은 WHO 근거인 만큼 (입국 제한 등은) WHO 근거에 따르면 되지 않을까 한다”고 말했다. 그는 또한 “(한중이) 과학적인 태도로 역지사지하며 (전염병에) 대응해 나가자”고도 덧붙였다.

　한국이 대규모 인도적 지원을 결정한 것에 대해선 “전염병과의 투쟁에 큰 힘을 실어줘서 깊은 감사를 표하며, 이 따뜻한 선물을 영원히 잊지 않을 것”이라고 말했다. 중국에 500만 달러 지원을 약속한 외교부는 전날 화물기를 빌려 민간에서 제공한 마스크 300만 장 등 구호물품을 실어 중국 우한에 보냈다.

　한국의 출입국 정책 강화를 두고 중국이 껄끄럽다는 속내를 내비치는 가운데, 정부는 여전히 추가 조치를 배제할 수 없다는 입장을 견지하고 있다. 김인철 외교부 대변인은 4일 정례 브리핑에서 “여행경보 (상향 등) 조정 문제는 계속 검토해 나가고 있다”고 말했다.

# WHO “전례없는 확산” 국제비상사태 선포

세계보건기구(WHO)가 신종 코로나바이러스 감염증(우한 폐렴)에 대해 ‘국제적 공중보건 비상사태(PHEIC)’를 선포했다. 테워드로스 아드하놈 거브러여수스 WHO 사무총장은 지난달 30일(현지 시간) 우한 폐렴에 대해 “지난 몇 주 동안 이전에 알지 못했던 병원체의 출현을 목격했고 전례가 없는 발병으로 확대했다”고 말했다. 다만 비상사태 선포는 “중국 내 발생 때문이 아니라 다른 나라에서 일어나는 일 때문”이라며 “(중국과) 교역과 여행을 방해하는 조치를 할 이유가 없다”고 주장했다.

　중국 내 우한 폐렴 확진 환자와 사망자 수 모두 1일 최대치로 증가하면서 확산 속도가 빨라지고 있다. 중국 내 확진 환자는 9692명으로 전날보다 1866명 늘었고, 사망자는 213명으로 43명 증가했다. 전 세계 확진 환자 수는 9831명으로 2003년 사스(SARS·중증급성호흡기증후군) 전 세계 환자 수 8098명을 넘어섰다. 　중국에서는 우한 폐렴의 ‘공기 감염’ 가능성까지 제기됐다. 중국질병예방통제센터(CDC)의 우쭌유(吳尊友) 수석전문가는 관영 중국중앙(CC)TV 인터뷰에서 “우한 폐렴은 사스와 많이 다르고 유행성감기(독감)의 전파 패턴과 훨씬 비슷하다”며 “우한 폐렴 감염자가 (특정한) 폐쇄공간에서 공기 중에 남긴 비말(飛沫·분비물)이나 에어로졸(미세한 고체 입자나 물방울)을 다른 사람이 흡입해 감염원 없는 감염을 일으킨다는 것을 조사 과정에서 발견했다”고 말했다.

# 中서 무증상자發 집단전염...3차감염 사례도

중국에서 ‘3차 감염’을 비롯해 신종 코로나바이러스 감염증(우한 폐렴) 집단 전염 사례가 잇따르면서 통제 가능한 범위를 넘어선 것 아니냐는 우려가 커지고 있다.

　29일 중국 허난(河南)성 안양(安陽)시 위생건강위원회에 따르면 후베이(湖北)성 우한(武漢)에 갔다가 안양 집에 돌아온 루(魯)모 씨에 의해 아버지(45)와 고모 2명이 전염됐다(2차 감염). 이어 루 씨 아버지에게서 루 씨 어머니 저우(周·42)모 씨와 루 씨의 또 다른 고모가 다시 한번 감염(3차 감염)됐다. 루 씨는 확진 환자임에도 10일 우한에서 돌아온 뒤 잠복기(최장 14일)를 지났는데도 증상이 나타나지 않아 무(無)증상 환자에 의한 집단 감염이 현실화됐다. 또 안후이(安徽)성 허페이(合肥)시에서는 동창 모임에 참석한 20대 6명이 집단으로 감염돼 28일 확진 판정을 받았다. 안후이성 황산(黃山)시에서도 가족 6명이 잇따라 감염됐다.　

　28일까지 중국 본토의 우한 폐렴 확진 환자 누계는 6018명으로 2003년 사스(SARS·중증급성호흡기증후군) 유행 당시 중국 본토 최종 확진 환자 5327명을 넘어섰다. 우한 폐렴 사망자는 132명으로 전날보다 26명 늘었다. 중동에서는 처음으로 아랍에미리트에서 우한 폐렴 확진 환자가 발생했다.

　크리스티안 린드마이어 세계보건기구(WHO) 대변인은 28일(현지 시간) “감염자가 어느 정도 증상이 나타나야 우한 폐렴을 전파할 수 있는지는 단정할 수 없다”며 무증상 감염 가능성을 언급했다. 하지만 박혜경 질병관리본부 중앙방역대책본부 총괄팀장은 “WHO 문건에는 그런 문구가 없고 무증상자로 인한 감염 근거는 없다고 돼 있다”고 말했다. 29일 국내에서 추가 확진 환자는 발생하지 않았다.

# 시진핑을 흔든 ‘우한의 기침’...성장률-리더십 위기

　‘신종 코로나바이러스 감염증(우한 폐렴)’ 사태로 시진핑(習近平) 중국 국가주석의 지도력이 적잖은 타격을 입고 있다. 중국 정부는 위기관리 능력에 문제점을 드러냈고, 경제에도 그림자가 드리울 것으로 보인다. 미 뉴욕타임스(NYT)는 “시 주석이 수년 만에 가장 심각한 정치적 위기를 맞았다”고 지적했다.

○　‘시진핑 리더십’에 비판 고조

　시 주석은 지난해 12월 30일 우한시 당국이 우한 폐렴 발병 사실을 밝힌 뒤 25일이 지난 이달 25일에야 처음으로 우한 폐렴 대응을 공식적으로 언급했다.

　정부의 미온적인 대응에 불만이 높아지자 27일 리커창(李克强) 총리는 시 주석의 지시로 폐렴 진원지인 후베이(湖北)성 우한(武漢)을 찾았다. 그는 병원과 마트 등을 방문해 의료진과 주민들을 격려했다. 임시 격리병동 건설 현장에서는 노동자들에게 “어려움이 있으면 말하라. 해결해 주겠다”고 했다. 현장에 모인 사람들은 “(어려움이) 없습니다!”라고 외쳤다. 하지만 현지 매체에 따르면 하루 전까지 이들은 마스크와 의료용 장갑 등 물자 부족에 대한 어려움을 토로했다. 소셜미디어 웨이보에는 “각지에 도움을 구하더니 갑자기 말을 바꿨네?” “참 중국적인 답”이라는 조소가 이어졌다.

　정부의 보고 및 의사결정 체계에도 상당한 문제가 있음이 드러났다. 저우셴왕(周先旺) 우한시장은 관영 중국중앙(CC)TV에 “우리도 정보 공개에 대한 불만이 있다. 지방정부인 우리는 관련 정보와 권한을 얻은 다음에야 정보를 공개할 수 있었다”고 불만을 표했다.

　중국 의료체계의 취약점도 드러났다. NYT에 따르면 우한 주민인 샤오시빙 씨(51)는 발열과 호흡 곤란을 겪었지만 우한시 병원 병상 부족으로 병원을 전전하다가 보름 이상 지난 26일에야 입원했다. 아내 펑시우 씨는 “(환자를) 이리저리 공 차듯 한다”고 불만을 토로했다. 마궈창(馬國强) 후베이성 부서기 겸 우한시 서기는 26일 밤 기자회견에서 우한시 병동이 극도의 과부하 상태임을 인정했다. 그는 “최근 며칠간 매일 1만5000명의 발열 환자가 병원들에 몰려 길게 줄을 서는 문제가 발생했다”고 밝혔다. 중국은 다음 달까지 우한시에 병상 1000개, 1300개 규모의 임시 격리 병동 2곳을 완공할 계획이다.

○　“경제성장률 4% 기록할 수도”

　우한 폐렴 확산에 따른 노동 일수 감소, 관광 위축 등이 장기화하면 정부의 목표치인 성장률 6% 사수가 어려워질 가능성이 있다. 홍콩 사우스차이나모닝포스트(SCMP)는 미국의 중국 연구기관 플리넘을 인용해 “중국의 1분기 성장률이 4%대를 기록할 수 있다”고 전망했다.

　중국은 춘제 연휴를 다음 달 2일까지로 사흘 연장했으나 우한 폐렴이 폭증하면서 더 연장할 가능성이 크다. 금융 도시 상하이(上海)는 기업들에 다음 달 9일까지 업무를 재개하지 말라고 통보했다. 로이터통신은 상하이시의 이번 조치로 인해 해당 지역에 공장을 소유하고 있거나 현지 합작사를 운영하고 있는 테슬라, 제너럴모터스(GM), 폭스바겐이 피해를 입을 것이라고 전했다.

　제조업 중심지인 쑤저우(蘇州)도 다음 달 8일 정오까지 업무를 중단하라고 기업들에 요구했다. 정보기술(IT) 기업 텐센트와 중국 최대 전자상거래 기업 알리바바 등은 직원들에게 14일간 재택근무를 하라고 통지한 것으로 알려졌다. 자금성(紫禁城)과 만리장성, 상하이 디즈니랜드 등 주요 관광지도 잠정 폐쇄됐다. 춘제 시즌이 피크인 영화 개봉도 잇따라 연기됐다. 중국 농구리그(CBA)도 무기한 중단됐다. 베이징(北京)은 춘제 연휴가 끝나더라도 후베이성 등 지방에서 돌아온 시민들에게 14일간 집에서 자가 격리할 것을 권고했다.

　일부 대기업은 의료진을 지원하기 위한 펀드 조성에 나섰다. 바이두(百度)는 27일 우한 폐렴 치료를 위한 연구개발 등을 지원하기 위해 3억 위안(약 506억 원)에 달하는 기금을 조성하겠다고 밝혔다. 음식 배달 앱 메이탄도 의료진을 지원하기 위한 2억 위안(약 337억 원) 규모의 기금을 조성한다. 이와 함께 우한 시내 의료진에 매일 1000회의 무료 테이크아웃 식사 제공권과 후베이성 내 의료진에 30만 대의 공용 자전거 무료 이용권을 약속했다.

# 우한 폐렴 경보

신종 코로나바이러스인 우한 폐렴이 확산 일로다. 지난해 12월 31일 중국 후베이(湖北)성 성도(省都)인 우한(武漢)에서 발생한 지 한 달도 되지 않아 미국 1명을 포함해 8개국에서 확진 환자 581명이 발생했다. 이 중 사망자는 17명으로 모두 중국인이다. 한국도 인천공항에서 감염자로 의심돼 격리된 중국인 1명이 확진 판정을 받았다. 교통 요충지로 ‘중국의 배꼽’이라 불리는 인구 1100만 도시 우한은 23일 오전 10시부터 도시로 들어가고 나가는 모든 대중교통 운행이 중단됐다. 중국 역사상 성도 봉쇄는 처음이다.

　▷우한 폐렴의 숙주는 박쥐나 뱀으로 알려져 있다. 사스(SARS·중증급성호흡기증후군)는 박쥐와 사향고양이, 메르스(MERS·중동호흡기증후군)는 낙타였다. 우한 폐렴 발원지인 수산물시장에서는 온갖 야생동물이 불법으로 거래돼 왔는데 두 번째 사망자도 수산물시장 가게 주인이었다. 잠복기는 짧게는 2, 3일, 길면 10∼12일이다. 증세는 감기나 독감과 비슷하고 치료제나 백신은 없다. 감염자의 침이나 콧물로 전파되므로 사람이 많은 곳에 갈 땐 마스크를 써야 한다. 마스크에 바이러스가 묻을 수 있으므로 한 번 쓰고는 버리는 것이 좋다.

　▷홍콩 전문가들은 우한 폐렴이 2003년 사스 때처럼 대유행할 조짐이 있다고 경고한다. 전염병 확산은 동물에서 인간→인간 간 전염→환자 가족과 의료진에 전염→대규모 발병 단계로 진행되는데 우한 폐렴은 마지막 단계 진입을 코앞에 두고 있다는 것이다. 중국의 정보 통제와 뒷북 대응이 바이러스 확산을 가속화했다는 비판이 나온다. 우한은 봉쇄됐지만 이미 수백만 우한 시민이 빠져나간 것으로 추정된다. 뉴욕타임스는 중국이 언론과 시민사회를 통제하고 정부가 정보를 독점하다 사태를 키웠다고 지적했다.

　▷우한 봉쇄 소식에 중국 주가지수가 폭락했다. 세계 경제가 입을 피해 규모도 2003년 사스 때를 능가할 것으로 보인다. 세계 경제에서 중국이 차지하는 비중이 2003년 8.7%에서 올해는 20%로 커질 것으로 전망되기 때문이다. 회복 기미를 보이던 한국 경제도 연초부터 악재를 만났다. 5년 전 메르스 때처럼 소비가 얼어붙어 성장률 반등에 지장을 줄 우려가 있다.

　▷오늘 시작되는 설 연휴가 1차 고비다. 이동이 많고 사람이 모여 감염 우려가 높지만 대다수 병원은 문을 닫는다. 중국인 관광객 14만 명도 몰려들 것으로 예상된다. 정부 당국은 24시간 비상방역체계를 가동하고, 개인은 관련 정보에 귀를 열어둔 채 손 소독과 마스크 쓰기를 비롯한 전염병 예방행동수칙을 따라야 한다.

# 中, 폐렴 발원지 우한시 뒤늦게 전면봉쇄

　신종 코로나바이러스로 인해 발생한 ‘우한 폐렴’ 확진자와 사망자가 크게 늘자 중국 당국이 뒤늦게 바이러스 발원지인 후베이성(湖北)성 우한(武漢)시를 긴급 봉쇄했다. 인구 1100만 명의 우한시는 후베이성의 성도(省都)다. 성도급 도시를 봉쇄한 것은 사상 처음이다.

　우한시 당국은 23일 오전 2시(현지 시간) 긴급 성명에서 “이날 오전 10시부터 버스와 지하철, 페리, 시외 장거리 버스 운행을 중단한다”며 “시민들은 특수한 상황이 아니면 우한을 떠나지 말라. 항공편과 열차편도 임시 중단하고 재개 시점은 추후 통지하겠다”고 밝혔다.

　중국 중부에 위치한 우한은 주변 9개 성(省)과 연결된 교통의 요충지이기도 하다. 전염병 대유행(팬데믹) 조짐이 보이자 다급해진 중국 당국이 초유의 조치를 내린 것으로 풀이된다.

　하지만 중국 내 우한 폐렴 확진 환자는 이날 600명으로 집계돼 전날보다 56명 늘었다. 중국 31개 성, 시(市) 가운데 27개에서 확진 또는 의심 환자가 발생했다. 우한 폐렴 영향권에 들지 않은 지역은 서부의 간쑤(甘肅)성, 칭하이(靑海)성, 신장(新疆)위구르자치구, 티베트자치구 등 4곳만 남았다. 중남미의 멕시코 브라질 콜롬비아, 북미의 캐나다에서도 의심 환자가 발생하는 등 전 세계적으로 우한 폐렴 확산 우려가 커지고 있다.

　이에 따라 외교부는 이날 중국 우한에 여행경보 2단계(여행 자제)를 발령했다. 우한 폐렴과 관련해 정부가 여행경보를 내린 것은 처음이다. 현재 2단계 여행경보 지역은 미국과 무력 갈등을 빚고 있는 이란, 대규모 시위가 이어졌던 홍콩 등이다. 외교부는 후베이성 전역엔 여행경보 1단계(여행 유의)를 발령했다. 외교부는 “이 지역(우한 등)을 여행할 예정인 국민들은 여행 필요성을 신중히 검토해 주시고, 체류 중인 국민은 신변 안전에 특별히 유의해 달라”고 당부했다. 질병관리본부는 신속한 현지 상황 파악 등을 위해 이날 중국 베이징(北京) 한국대사관에 역학조사관을 파견했다.

# 美로 번진 우한폐렴, 대유행 조짐

미국에서 신종 코로나바이러스로 인한 ‘우한 폐렴’ 첫 확진 환자가 발생하고, 중국에서 환자 폭증세가 이어지면서 전 세계적인 대유행 조짐을 보이고 있다.

　21일(현지 시간) 로이터통신에 따르면 미국 질병통제예방센터(CDC)는 최근 바이러스 발생지인 중국 후베이(湖北)성 우한(武漢)으로 여행을 다녀온 30대 남성이 우한 폐렴 확진 판정을 받았다고 밝혔다. 미국 워싱턴주 시애틀 인근에 거주하는 이 남성은 15일 귀국한 뒤 치료를 받고 있다. 아시아 외 대륙에서 확진 환자가 나온 것은 처음이다.

　중국 질병예방통제센터는 22일 우한 폐렴 확진 환자가 440명(대만 포함)으로 늘었다고 밝혔다. 하루 만에 130여 명이 증가한 것이다. 13개 중국 성(省), 시(市)에서 확진 환자가 발생했다. 의심 환자까지 합치면 21개 성, 시가 영향권에 들어 중국 31개 성, 시의 68%에 달했다. 이날 마카오에서도 처음으로 확진 환자가 발생했다. 중국 내 사망자도 6명에서 9명으로 늘어났다. 당국이 관찰 중인 밀접 접촉자가 1394명에 달해 환자 급증세가 이어질 것으로 보인다.

　중국 관영 신화통신은 “바이러스의 변이 가능성이 있어 전염 상황이 더욱 확산될 위험이 있다”는 국가위생건강위원회 전문가팀의 지적을 전했다. 이들은 “지역사회 전파도 있다”고 밝혔다. 일부 지역에서 집단 감염 사례가 있음을 인정한 것이다. 전문가팀의 중난산(鍾南山) 팀장은 “슈퍼 전파자 출현을 막아야 한다”고 경고했다. 홍콩 사우스차이나모닝포스트(SCMP)는 2003년 사스(SARS·중증급성호흡기증후군) 때와 같은 전면적 확산 단계에 진입할 것이라고 전망했다.

　중국 14억 인구 중 4억5000만 명 이상이 이동하는 춘제(중국의 설·25일)가 다가와 대유행의 분수령이 될 것으로 보인다. 사스 사태급 대응을 천명한 중국 당국은 우한으로 가거나 우한을 떠나지 말라는 우한 여행 자제 권고령을 내렸다.

　국내에서도 우한 폐렴과 유사한 증상을 보이는 ‘유증상자’ 4명이 추가로 발생했다. 이로써 국내 우한 폐렴 유증상자는 19일 확진 판정을 받은 중국인 여성 A 씨(35)를 포함해 총 16명으로 늘었다. 새로 추가된 유증상자 중 3명에는 A 씨와 함께 우한에서 인천행 비행기를 탄 승객 및 공항 관계자가 포함된 것으로 알려졌다.

　문재인 대통령은 22일 상황을 보고받은 뒤 “검역 및 예방 조치에 만전을 기함과 동시에 경제에 미치는 영향도 종합 점검하라”고 지시했다고 한정우 청와대 부대변인이 전했다. 문 대통령은 전날 정부세종청사에서 열렸던 국무회의에서도 “방역에 각별히 힘써 달라”는 취지의 지시를 한 것으로 알려졌다.

# 춘절 앞둔 中, ‘사스 공포’ 재현되나

　중국 내에서 신종 코로나바이러스로 인한 ‘우한 폐렴’ 환자 수가 폭발적으로 늘어나면서 ‘중국의 방역체계가 뚫린 것 아니냐’는 우려가 커지고 있다. 중국 누리꾼들은 당국의 은폐, 늑장 대응 의혹까지 제기했다. 2002∼2003년 사스(SARS·중증급성호흡기증후군) 발생 초기 중국 당국이 전염 사실을 은폐하는 바람에 초기 대응에 실패해 중국과 홍콩에서만 648명이 목숨을 잃은 전례가 되풀이되는 것 아니냐는 목소리도 나온다.

　우한 폐렴은 전염 규모와 범위 모두 걷잡을 수 없이 커지는 양상이다. 발원지인 후베이(湖北)성 우한(武漢)에서 18, 19일 이틀 동안 확진 환자가 136명 늘면서 기존 환자의 3배 이상으로 증가했다. 또 베이징(北京) 남부 다싱(大興)구와 광둥(廣東)성 선전(深(수,천))시에서 각각 확진 환자 2명, 1명이 발생했고 선전시에서 8명, 저장(浙江)성에서 5명의 의심 환자가 나와 중국 전역으로 확산될 조짐을 보이고 있다. ‘전염력이 약하고 사람 간 전염 가능성이 낮다’고 강조해온 중국 당국의 발표와는 정면으로 배치되는 것이다.

　다싱구에는 지난해 운영을 시작한 베이징신공항이 있다. 베이징시 당국은 확진 환자가 우한에 여행을 다녀왔다고 밝혔지만 언제 어떤 경로로 전염됐는지 공개하지 않았다. 상하이(上海)시 당국은 “일부 의심 환자에 대해 전염 방지 조치를 취했다”고 밝혔지만 의심 환자 수는 공개하지 않았다.

　선전시 당국은 확진 환자 1명이 우한에 친척을 만나러 다녀온 66세 남성이라고 밝혔다. 하지만 인도 통신사인 PTI는 선전시 국제학교 교사인 인도인 프리티 마헤슈와리 씨(45·여)가 확진 판정을 받아 선전시 병원에서 격리 치료 중이라고 보도했다. 이 보도가 사실이라면 중국 당국이 인도인 환자 발생 사실을 숨긴 셈이 된다. 더욱이 이 여성은 우한에 간 적이 없다고 PTI는 전했다.

　그동안 지방 당국 차원에서만 대응해오던 중국은 19일에야 국가위생건강위원회가 예방 대책을 발표하고 중국 전역에 실무대응팀을 파견했다. 중국 당국은 “바이러스 전염의 원천을 찾지 못했고 전파 경로도 완전히 파악하지 못했다”고 밝혔다. 한국 정부 당국자는 “중국 당국이 새로운 조사 방법을 적용했더니 우한에서 확진 환자 수가 크게 늘었다고 전해왔다”고 말했다. 특히 중국은 춘제(春節·중국의 설)를 전후해 연인원 30억 명이 대이동할 것으로 예상돼 걷잡을 수 없는 대유행으로 악화할 가능성을 배제할 수 없다.

　중국 누리꾼들의 불신도 확산하고 있다. 한 누리꾼은 중국 소셜미디어 웨이보에 “전염력이 약하다더니 이렇게 급증했나. 또 속이고 숨기는가”라고 썼다. “애국 바이러스라는 말은 더 이상 안 나오겠네”라고 비꼬는 글도 보였다.

# 중국몽 위협하는 새로운 세대의 등장

지난해 12월과 이달 대만 대선을 취재하기 위해 타이베이를 방문했다. 현장에서 만난 20대 대만 젊은이들은 자신을 “톈란두(天然獨)”라고 묘사했다. 어릴 때부터 대만이 독립된 주권을 가진 국가라고 여기며 자랐다는 뜻이다. 대학 4학년인 청(程·22)모 씨는 “그래서 우리는 중국이 제기한 일국양제(一國兩制·1국가 2체제) 통일 방안을 받아들일 수 없다”고 말했다.

　현지에서 만난 대만의 2030세대는 자신을 중국인이 아닌 대만인이라고 여겼다. 이들의 표심이 반중(反中) 성향 차이잉원(蔡英文) 총통 재선의 원동력이었다. 1980년대에 태어난 젊은이들을 기성세대와 달라지게 한 건 대만의 민주화였다.

　1980년대 민주화운동을 거쳐 1996년 총통직선제가 도입되기 전 대만은 중국에서 건너온 외성인(外省人)이 주축이 된 국민당이 장기 집권한 권위주의 사회였다. 1970년대 이전에 태어난 ‘구세대’는 중국과 통일해야 한다는 ‘톈란퉁(天然統)’ ‘당란퉁(當然統)’이 많다. 반면 어릴 때부터 민주화를 경험한 젊은이들은 대만인으로서의 정체성이 강하다. 자신이 중국인이냐 대만인이냐, 중국과 통일해야 하느냐 독립해야 하느냐 혼란을 겪는 40대 이상 대만인들과 다르다. 대만에서 만난 전문가들은 “젊은이들은 과거엔 취업 등 자신의 문제에만 관심을 가졌다. 하지만 자신들의 미래를 중국과 통일을 원하는 은퇴한 구세대에 맡겨서는 안 된다는 위기의식이 투표 열기로 이어졌다”고 주장했다.

　대만 대선이 차이 총통의 압승으로 끝나자 대만 언론들은 “이들이 이제 톈란두에서 톈란타이(天然臺)로 변하고 있다”고 분석했다. 이들은 “이미 대만은 주권 국가이기 때문에 굳이 독립을 추구할 필요가 없고 현재의 대만 그 자체로 현상을 유지하기를 원한다”는 것이다. 대만 통일에 필요하다면 무력도 배제하지 않겠다는 시진핑(習近平) 중국 국가주석과는 대척점에 서 있는 셈이다.

　지난해 홍콩 시위 현장에서도 톈란두와 거의 똑같은 새로운 밀레니얼 세대의 출현을 목격했다. 홍콩중문대 학생 찬모 씨(21·여)는 기자에게 “홍콩이 중국에 반환된 1990년대에 태어난 우리는 100% 홍콩에 속한 첫 세대”라고 말했다. 영국의 식민지가 아니면서 “중국과도 다른 체제를 보장받은 자유로운 사회 홍콩에서 교육 받으며 자란 우리는 중국인이 아닌 홍콩인”이라는 얘기였다.　

　　‘중국 건국 100주년인 2049년까지 세계 강국으로 우뚝 서겠다’는 중국몽(夢)에는 일국양제의 성공도 포함된다. 홍콩과 마카오의 일국양제를 발판으로 대만도 통일하겠다는 구상이다. 하지만 기존 세대와 완전히 다른 가치관을 가진 대만의 톈란두와 홍콩의 밀레니얼 세대가 새로운 변수로 떠올랐음을 세계가 목격했다. 현장에서 이들이 중국인 정체성을 거부하는 것이 일시적 현상이 아니라는 점을 확인할 수 있었다. 이들은 시간이 흐를수록 대만과 홍콩의 주축 세대가 될 것이다.

　대만과 홍콩에서 만난 전문가들은 “중국이 새로운 세대의 등장이라는 변화를 직시하지 못한 채 이를 중국에 대한 대항과 도전으로만 보고 강경 대응해 역효과가 났다는 것이 대만과 홍콩의 공통점”이라고 지적했다. 어떤 정치세력이든 민심의 변화를 읽지 못하면 위기를 겪을 수밖에 없다는 점을 새삼 일깨워준다.

# 美-中1단계 무역 합의... 중국 수출 비상 걸린 한국

미국과 중국이 2년 가까이 끌었던 무역전쟁에서 1단계 합의를 하며 휴전에 들어갔다. 세계 1, 2위 경제 대국들이 벌이는 싸움에 위축되고 불안했던 글로벌 경제는 일단 짙은 먹구름이 가셨다. 그러나 중국 화웨이 제재를 비롯한 민감한 갈등은 2단계로 넘겨진 데다 1단계 합의 이행 과정이 순조롭지 못할 경우 언제든 관세 전쟁이 재개될 수 있어 불확실성은 여전하다. 무엇보다 중국이 막대한 양의 미국 제품을 추가 수입하기로 함으로써 한국의 대중(對中) 수출에 큰 타격이 우려된다.

　미중은 15일 중국이 미국산 제품을 2년 간 2000억 달러(약 232조 원) 어치 추가 구매하는데 합의했다. 대신 미국은 중국산 제품에 부과하던 15% 관세를 7.5%로 줄이고, 작년 12월 부과 예정이었던 다른 제품들에 대한 추가 관세는 부과하지 않기로 했다. 합의안에는 중국의 지식재산권 보호와 기술이전 강요 금지, 인위적인 위안화 절하 중단 등에 대한 약속도 담겼다.

　중국이 미국에서 추가 수입하기로 한 2000억 달러는 한국의 연간 총 수출액의 33%에 이르는 금액이다. 농산물 뿐 아니라 공산품 에너지 서비스 등 다양한 제품들이 포함된다. 중국의 내수량이 크게 늘지 않는다면 그만큼 다른 나라에서 수입하던 양이 줄어들 것이다. 국제통화기금(IMF)은 지난해 말 ‘미중 무역협정의 부작용’이란 보고서에서 미중 협상이 타결되면 한국 일본 유럽연합(EU) 등 다른 나라들이 타격을 입을 것이라는 분석을 내놨다. 중국의 수입액이 기존과 같을 때 한국은 최대 460억 달러(약 53조 원), 국내총생산(GDP)의 3% 가량 수출이 줄어들 것이라고 봤다.

　한국은 미중 무역갈등의 중단에 안도할 틈도 없이 대중 수출에 직격탄을 맞을 가능성이 높아졌다. 강대국들끼리의 ‘짬짜미 협정’으로 글로벌 무역체계가 인위적으로 재편됨에 따라 향후 경제에 미칠 영향의 불확실성도 커졌다. 한국은 대중 수출이 전체의 27%나 차지한다. 수출을 다변화해 대중 무역 의존도를 줄이고 미중 무역의 틈새시장을 찾는 일이 급해졌다.

# 中, 美전역 사정권 SLBM 시험발사...러-이란과 합동훈련도

미국이 이란에 대해 전례 없이 강력한 제재를 지속하는 가운데 중국 러시아가 이란과 함께 인도양 북부와 오만해에서 사상 처음으로 해상 연합 군사훈련에 나선다. 중국은 미국 전역을 타격할 수 있는 대륙간탄도미사일(ICBM)급 핵탄두 탑재 가능 잠수함발사탄도미사일(SLBM)도 시험 발사해 무력시위를 벌였다. 중국, 러시아가 손잡고 미국과 맞서는 모습이 지속되고 있어 주목된다.

　25일 AP통신과 이란 메르통신 등에 따르면 세 나라 해군이 참가하는 이번 훈련은 ‘해양 안보벨트’라는 이름으로 27∼30일 진행된다. 이 지역은 걸프해의 입구이며 세계 최대 원유 수송 해역인 호르무즈 해협과도 가깝다. 이란군은 “이번 훈련은 중동 지역의 국제 교역 안보 강화가 목적이다. 이란, 러시아, 중국이 안보 경험을 교환하고, 테러와 해적 행위에 맞서는 게 가장 중요한 목표”라고 밝혔다.

　하지만 이번 훈련은 미국의 중거리핵전력조약(INF) 탈퇴 및 경제 제재로 어려움을 겪는 이란을 지원하기 위한 것으로 중국과 러시아가 중동 지역에서 미국에 대항하는 움직임으로 보는 시각이 많다.

　미국은 이란에 대한 군사적 압박을 높이기 위해 우방국들과 걸프 해역에서 활동하는 해군 군사 연합체인 ‘호르무즈 호위연합’을 결성했고, 이에 대해 중국과 러시아는 ‘중동 지역의 안정을 위협한다’며 반발해왔다. 한국도 미국의 요청에 따라 호르무즈 해협에 파병할 가능성이 있다는 점에서 주목된다.

　미국은 예멘 후티 반군이 자신들의 행위라고 밝힌 올해 9월 사우디아라비아 국영 석유기업 아람코의 석유 생산시설과 유전에 대한 무인기(드론) 및 미사일 공격에도 이란이 직접 개입했다며 이란에 대한 압박 강도를 높이고 있다. 미국은 아람코 피격 사태 후 사우디에 미군을 증파했고, 미사일방어(MD) 시스템도 추가 설치했다.

　이란은 이번 훈련을 중국, 러시아와의 군사 협력을 확대하는 계기로 활용할 의지를 내보였다. 이란 파르스통신에 따르면 이란 해군의 호세인 한자디 소장은 “이번 훈련은 중국 러시아 해군과의 광범위한 협력의 한 부분이며, 여기에는 잠수함과 구축함 생산도 포함된다”고 말했다. 파키스탄처럼 반미 성향이 강한 나라가 향후 중국 러시아 이란의 합동 군사훈련에 참여할 수 있다는 전망도 나온다.

　중국은 22일 보하이(渤海)에서 미국 전역을 사거리로 하는 ICBM급 SLBM인 ‘쥐랑(巨浪)-3’을 서쪽 방향으로 시험 발사했다. 중국 관영 관차저왕(觀察者網)은 쥐랑-3 발사 사실을 직접 언급하지 않으면서도 “베이징(北京)에서 (미사일 궤적인) 이상한 구름들이 목격됐다”며 “중국 정부가 20∼27일 보하이에서 군사 임무를 이유로 항행을 금지했다”고 보도했다.

　쥐랑-3은 중국의 094형 전략 핵추진 잠수함에서 발사된 것으로 알려졌다. 사거리가 1만 km에 달해 핵탄두를 탑재하고 미국 본토를 타격할 수 있는 것으로 평가된다.

# 美안팎 퍼지는 차이나 배싱…복잡한 글로벌 외교 방정식

워싱턴의 한 중견 로펌에서 근무하는 P 변호사에겐 요즘 중국인 의뢰인이 부쩍 늘었다. 미국 내 중국 과학자들이 미국 정부로부터 감시를 당하고, 석연치 않은 이유로 활동을 제한당하고 있다는 것. 법적 대응을 해주는 그는 “중국 과학자들한테 미국 정보요원들이 거의 일대일로 붙어서 밀착 감시하는 분위기”라며 “미중 관계가 나빠지니 일은 많아져서 좋다”고 했다.

　　‘설마 민간 분야 학자들까지 그렇게 치밀하게 관리할까…’ 궁금했지만 정색하는 P 변호사의 표정을 보니 틀린 말은 아닌 것 같았다. 화웨이 사례에서 보듯 중국의 기술패권을 견제하려는 도널드 트럼프 행정부의 움직임은 이미 전방위로 확장하는 기류다. 미국의 ‘중국 때리기(China bashing)’가 워싱턴 외교안보 분야의 분명한 흐름이라는 점도 부인할 수 없다.

　중국을 사실상 ‘적대국가’로 겨냥한 백악관과 국무부 고위 당국자들의 발언 강도는 점점 강해지고 있다. 최근 홍콩 시위와 관련해 중국의 인권 상황을 문제 삼는 내용이 많아졌다. 마이크 폼페이오 국무장관은 지난달 독일 베를린장벽 30주년을 기념해 진행한 연설에서조차 “중국 공산당이 국민을 억압하고 있다”며 중국을 비판했다. 옛 동독에 이어 중국까지 공산주의 문제점을 지적하는 방식이었지만 ‘1989년의 교훈: 자유와 우리의 미래’라는 제목의 연설 취지와는 연관성이 낮아 보였다.

　올해 70주년을 맞은 북대서양조약기구(NATO·나토)가 이달 초 정상회의 성명에서 중국을 겨냥한 것도 예사롭지 않다. 중국의 아프리카 공략과 사이버 활동, 군사력 증강 등이 유럽에도 새로운 도전이 될 수 있다는 논리지만 지구 맞은편의 중국을 타깃으로 삼고 나서는 것은 다소 뜬금없다. 나토의 큰손 회원국인 미국의 그림자가 어른거린다. 미국의 공격적인 대중 전략이 수출되는 느낌이랄까.

　중국 연구에 대한 강도를 높이는 싱크탱크도 많아졌다. 전략국제문제연구소(CSIS)는 분야별로 중국을 연구하는 팀만 4개로 만들었다. 이달 들어서만 ‘중국의 부상과 글로벌 질서’ ‘중국의 인권 문제와 미국의 대응’ ‘중국의 정치적, 종교적 인권 문제’ 등 중국을 주제로 한 세미나가 잇따라 진행됐다. 막상 현장에 가보면 중국인은 거의 없고 현지 학계 및 언론 인사와 홍콩, 대만, 한국 같은 외신기자들로 북적인다. 중국인은 견제 분위기가 워낙 강해서인지 얼굴을 내밀기도 어려운 눈치다.

　미국이 12일 중국과의 1단계 무역합의를 타결하면서 미중 사이 긴장감이 다소 누그러지는 듯하다. 그러나 지식재산권 등 훨씬 까다로운 내용을 다룰 2단계 협상이 남아 있다. 잠시 동결일 뿐 미중 관계가 좋아질 것이라고 기대하는 기류를 찾기 어렵다.

　중국이 미국 외교안보 정책의 변수가 아닌 상수가 된 건 이미 오래전이다. 이런 충돌 속에서 베이징의 반발은 더 거세지고 이로 인한 동북아 정세는 더 출렁이고 있다. 내년에는 ‘새로운 길’을 예고한 북한 문제까지 포함되면 미중 사이에서 풀어야 할 외교 함수도 더 복잡해진다. 이젠 중국 자체는 물론이고 미국의 대중 정책과 전략에 대한 더 많은 분석과 연구까지 필요한 시간이 다가오고 있다.

# 中, 27년만에 최저성장 침체 그림자 짙어진다

중국의 3분기(7∼9월) 경제성장률이 27년 만에 최저인 6.0%에 그쳤다. 한국 기업이 수출과 직접투자를 가장 많이 하는 중국 시장이 쪼그라들면서 한국 경제의 성장 발판이 흔들리고 있다는 분석이 나온다.

　중국 국가통계국은 18일 “3분기 국내총생산(GDP)이 24조6865억 위안(약 4119조 원)으로 지난해 같은 기간보다 6.0% 증가했다”고 밝혔다. 이 같은 성장률은 분기별 성장률 통계를 집계하기 시작한 1992년 이후 27년 만에 가장 낮은 것으로 당초 시장 전망치보다 0.1%포인트 하락한 것이다. 중국은 2015년 2분기 7.0% 성장세를 나타낸 뒤 4년 동안 6%대 성장에 머물다가 6% 선이 무너질 상황에 몰렸다. 지난해 1분기부터는 분기 성장률이 매번 하락세를 보이고 있는 점을 감안하면 4분기 성장률은 5%대로 떨어질 가능성이 있다는 전망이 많다.

　3분기 중국 성장률이 시장 예상보다 낮아진 것은 미중 무역분쟁과 아프리카돼지열병(ASF)으로 수출과 내수 기반이 동시에 약해졌기 때문이다. 미중 무역 갈등 여파로 중국의 9월 수출은 지난해 같은 달보다 3.2% 줄었다. 제조업의 활력 정도를 보여주는 생산자물가지수(PPI)는 올 7∼9월 3개월 연속 감소해 디플레이션 우려까지 나오고 있다. 여기에 ASF로 지난달 돼지고기 가격이 70% 올라 지난달 소비자물가가 3% 상승했다. 그 여파로 소비가 감소하면서 성장률을 떨어뜨리는 효과가 나타났다. 영국 파이낸셜타임스(FT)는 “지방정부가 도로 다리 등 인프라 건설을 통해 성장률 목표를 달성해 왔지만 이런 사업도 말라가고 있다”고 했다.

　중국 경제가 부진에 빠짐에 따라 제조업과 금융 부문에서 밀접하게 연관돼 있는 한국 경제가 타격을 받을 수 있다는 우려가 적지 않다. 한국경제연구원 등 민간 연구소들은 중국 성장률이 1%포인트 내려가면 한국 성장률은 0.5%포인트 하락할 수 있다고 보고 있다. 주원 현대경제연구원 경제연구실장은 “세계 경제 침체가 장기화되고 중국 정부의 각종 부양책도 먹히지 않으면서 성장 둔화가 내년까지 이어질 수 있고 한국 경제가 부정적 영향을 받을 수 있다”고 말했다.

# 홍콩시위대-중국군 첫 대치...부대 접근하자 “후폭풍 책임져야”

홍콩 반중 시위대와 홍콩 주둔 중국 인민해방군이 6일 밤 잠시 대치했다. 6월부터 넉 달째 이어지고 있는 이번 시위에서 양측의 첫 직접 대치여서 긴장감이 고조됐다.

　이날 시위대 수백 명은 카오룽 지역의 인민해방군 부대 근처에서 레이저 불빛을 부대 막사 건물에 비췄다. 중국군은 즉각 막사 옥상에서 노란 깃발을 들어 시위대에 경고 신호를 보냈다. 깃발에는 중국 본토에서 사용하는 푸퉁화(普通話)와 영어로 “당신은 법을 어기고 있다. 기소될 수 있다”는 문구가 적혔다. 중국군은 홍콩에서 쓰는 광둥(廣東)어로 “이후 발생하는 후과는 모두 자신이 책임져야 한다”는 육성 경고도 했다.

　중국군은 이 과정에서 카메라로 시위대를 촬영하며 이들의 동태를 면밀히 감시했다. 다만 시위대가 곧 부대 주변을 떠나면서 더 이상의 충돌은 일어나지 않았다. 사우스차이나모닝포스트(SCMP)는 “중국군이 유례없는 움직임으로 경고했다”고 전했다. 중국군이 시위대에 발포하거나 유혈 진압에 나서면 시위 사태가 걷잡을 수 없는 방향으로 흘러갈 수도 있다는 우려가 나온다. 친중파 세력들은 시위대의 이번 행동이 서방의 개입을 이끌어내려는 고의적 도발이 아니냐고 주장하고 있다.

　5일부터 시작된 복면금지법 시행에 따른 대립도 점점 격화되고 있다. 홍콩 경찰은 7일 복면금지법 위반 혐의로 18세 대학생 및 38세 여성을 처음으로 기소했다. 이들은 5일 새벽 마스크를 쓰고 시위를 벌이다 체포됐다. 교육 당국은 중·고등학교 교장들에게 “8일부터 마스크를 쓰고 등교하는 학생, 수업을 거부하는 학생, 인간 띠 시위를 벌이거나 구호를 외치는 학생들의 명단을 제출하라”고 지시했다. 경찰은 6일 대학의 허락 없이 홍콩중문대와 침례대 안으로 진입해 시위대를 체포했고, 마스크를 착용한 시민들을 무차별적으로 체포하고 있는 것으로 알려졌다.

　6일 삼서이보 지역에서는 60대 운전기사가 모는 택시 1대가 시위대를 향해 돌진해 2명이 차에 깔려 중상을 입었다. 이후 시위대들이 기사를 끌어내 구타했고, 기사가 얼굴에 피를 흘리며 의식을 잃은 모습도 목격됐다. 한 방송사 기자는 시위대가 던진 화염병을 맞아 얼굴에 화상을 입기도 했다.

　홍콩 지하철은 7일 오전에도 전체 지하철역(94곳) 중 39곳만 운행했다. 이날 오후 6시부터는 내부 수리를 이유로 전체 노선을 폐쇄해 유령 도시를 방불케 했다. 대형 쇼핑몰은 문을 닫았고 주요 마트들도 영업시간을 단축했다. 마트에 생필품을 사려는 사람들이 장사진을 이뤄 물건이 동나는 광경도 목격됐다. 일부 시민들은 “전쟁 같은 분위기”라고 전했다.

　한편 미국프로농구(NBA)의 유명 구단 휴스턴 로키츠의 대릴 모레이 단장은 시위대를 지지했다가 로키츠를 후원하던 중국 기업들이 스폰서 중단을 선언해 곤욕을 치렀다. 그는 6일 트위터에 “복잡한 사건에 대해 한쪽 편만 들었다”고 썼다.

# 애국주의 들끓는 건국 70년… 中내부에서도 책임 목소리

중국 건국 70주년 기념 열병식이 오전 10시(현지 시간)부터 베이징 중심 톈안먼(天安門)일대에서 시작된 1일. 기자를 비롯한 외신 기자들은 열병식 시작 5시간 반 전인 오전 4시 반경 1차 보안검사를 거친 뒤 베이징 서부 미디어센터에 모였다. 컴컴한 새벽, 교통통제로 텅 빈 도로를 달려 오전 6시경 도착한 톈안먼광장 인근 첸먼(前門). 열병식 참관 장소인 톈안먼광장 맨 앞으로 향하기 위해 2차 보안검사를 통과해야 했다.

　런민(人民)일보 신화통신 등 중국 관영매체 기자들은 전날인 지난달 30일 오후 11시 미디어센터에 집합했다. 이들이 톈안먼 일대에 도착한 시간은 1일 오전 1시 50분경이었다. 열병식 참가 병사들은 이미 톈안먼을 지나는 창안(長安)대로에 집결했다. 하룻밤을 꼬박 거리에서 지냈지만 표정은 매우 밝았다.

　열병식 시작 전 현장에서 만난 중국인들도 벅찬 감정을 감추지 못했다. 3만여 관중은 각 지역에서 선발돼 열병식 참관 기회를 얻어 새벽부터 현장에 도착했다. 톈안먼 광장에서 “강한 조국의 군대”를 직접 보는 것은 중국인들의 꿈이었다.

　“어떤 세력도 우리 위대한 조국의 지위를 흔들 수 없다”고 선포한 시진핑(習近平) 중국 국가주석의 연설에 중국인들은 환호했다. 시 주석이 “70년 전 중국의 건국으로부터 근대 이후 중국의 길고 긴 가난함과 약함, 괴롭힘을 당한 비참한 운명을 철저히 바꿨다”고 말한 것이 의미심장했다. ‘강해진 조국’에 대한 중국인들의 환호는 이제 미국은 물론이고 세계 어떤 나라도 중국을 건드릴 수 없다는 자부심으로 보였다. 그 자부심은 때로 외부 세계에 대한 호전적인 언어로 드러난다.

　지금 중국 전역은 ‘인민의 단결’이 가장 중요하다는 애국주의로 들끓고 있다. 국경절 연휴(1∼7일)에 개봉한 애국주의 영화 ‘나와 내 조국’ 등의 흥행 바람이 몰아치고 있다. ‘중화민족의 위대한 부흥’이라는 민족주의가 애국주의를 떠받친다. 애국주의 자체는 탓할 일이 아니다. 하지만 ‘강대국이 져야 할 무거운 책임’도 함께 깨달아 가고 있는지 중국 내부에서도 우려가 나온다.

　베이징대 국제관계학원 부원장을 지낸 왕이저우(王逸舟) 베이징대 교수는 인터뷰에서 “민족주의를 잘못 다루면 쇼비니즘(맹목적 애국주의)으로 변할 수 있다”고 경고했다. “특히 한 국가가 괴롭힘을 당한 역사가 있거나 주권 분쟁이 있으면, 민족주의는 상상하지 못한 (방식으로) 남을 해치는 결과를 낳을 수 있다”고 지적했다. “좋은 외교는 반드시 좋은 내치, 좋은 사회의 기초가 있어야 한다”고 일갈한 그가 주장하는 ‘인(仁)의 사회’로 가는 조건에는 “인민들이 더 개방적이고 호전적인 정서가 없는 사회” 등이 포함되어 있다.

　그는 다른 국가들이 중국의 굴기(굴起)에 왜 우려하고 위협을 느끼는지 중국이 제대로 인식하지 못하는 것을 걱정했다. 중국의 굴기가 갈림길에 놓였다고 한 그는 “높은 산 정상으로 갈수록 풍경이 아름답지만 고산 증세가 심해진다”고 했다. 지금은 과거 중국에는 없었던 문제, 즉 세계 속에서 더 큰 책임과 의무라는 문제를 마주하고 있다는 것인데, 애국주의만 지나치게 강조하다 눈을 가리는 게 아닌지 다시 돌아보게 하는 대목이다.

# 시진핑 “어떤 힘도 중국 흔들지 못해”

중국이 1일 건국 70주년을 맞아 베이징 톈안먼(天安門)광장에서 무기 전시장을 방불케 하는 대규모 열병식을 열어 국력을 과시했다. 시진핑(習近平) 국가주석은 미국을 겨냥해 “어떤 힘도 중국을 흔들어 놓을 수 없다”고 강조했다. 홍콩에는 일국양제를, 대만에는 평화통일을 언급하며 애국, 단결, 민족주의를 거듭 외쳤다.

　이날 열병식은 건국 70주년을 자축하는 70번의 예포 발사와 오성홍기 게양식으로 시작했다. 인민복 차림의 시 주석은 양 옆에 장쩌민(江澤民), 후진타오(胡錦濤) 전 주석을 대동한 채 “지난 70년 동안 중국은 눈부신 성장을 이뤄냈다. 어떠한 힘도 우리의 지위를 흔들 수 없다. 중국 인민과 중화민족의 전진을 막을 어떠한 세력도 있을 수 없다”고 말했다. 미중 무역전쟁, 홍콩 반중 시위, 경제 둔화 등 내우외환에도 불구하고 미국의 압박에 굴하지 않겠다는 뜻으로 풀이된다.

　그는 홍콩과 대만을 겨냥해 “평화통일, 일국양제의 원칙을 준수하며 홍콩과 마카오의 장기적 번영과 안정을 유지해야 한다”고 했다. 또 “중국의 내일은 훨씬 나아질 것”이라며 중화민족의 위대한 부흥을 뜻하는 ‘중국몽’ 실현을 위해 단합하자고 주문했다. 연설 말미에는 “중화인민공화국이여, 위대한 중국 공산당이여, 위대한 중국인들이여 영원하라”고 외쳤다.

　시 주석은 톈안먼광장 앞 창안제(長安街)에서 미리 도열해 있던 59개 제대, 군사 1만5000명의 사열을 받았다. 최첨단 무기 전시장을 방불케 한 열병식은 그 자체로 미국을 향한 메시지였다. 가장 이목을 끈 무기는 미국 수도 워싱턴을 타격할 수 있는 차세대 대륙간탄도미사일(ICBM) ‘둥펑-41’. 이날 최초 공개된 이 미사일은 사거리가 1만4000km여서 전 세계가 사정권이다. 최고 10개의 핵탄두를 장착할 수 있고 공격 목표의 오차 범위도 100m에 불과한 것으로 알려졌다.

　극초음속 활강 기술을 사용해 미국의 미사일방어체계(MD)를 뚫을 수 있는 것으로 알려진 ‘둥펑-17’, 항공모함 킬러로 불리는 극초음속 미사일 ‘둥펑-100’ 등도 선보였다. 미국 F-35에 맞먹는 신형 스텔스 전투기 ‘J-20’, 미 군용헬기 블랙호크에 필적하는 ‘Z-20’도 가세했다. 중국 언론은 이날 열병식에 동원된 무기 중 40%가 최초로 공개됐다고 전했다. 관영 글로벌타임스는 “어떤 위협에도 대응할 전략 핵무기를 갖고 있다는 뜻을 알렸다”고 했다. 미국뿐 아니라 한국, 일본 등 주변국에 대한 위협 효과도 있다는 분석도 나온다.

　이날 건국 70주년을 맞아 각국 지도자들도 축전을 보냈다. 중국은 블라디미르 푸틴 러시아 대통령, 도널드 트럼프 미국 대통령, 김정은 북한 국무위원장의 순서로 축전을 소개했다.

　김 위원장은 축전에서 “(북한은) 나라의 안정과 핵심이익을 수호하고 지속적인 발전을 이룩하기 위한 중국의 투쟁을 전적으로 지지한다”며 “사회주의를 고수하고 빛내기 위한 한길에서 언제나 (중국과) 함께 있을 것”이라고 밝혔다고 조선중앙통신이 1일 보도했다. 이어 ‘북-중 간 여러 차례의 상봉(정상회담)에서 이룩된 중요한 합의정신’을 언급하며 “새 시대의 요구와 두 나라 인민의 공동의 염원에 맞게 (양국 관계가) 날로 활력 있게 발전할 것이라고 굳게 믿는다”고 강조했다.

# 中, 위안화 환율 또 올려… 美는 中가구에 징벌관세

중국 중앙은행 런민(人民)은행이 9일 위안화 고시 환율을 또 올렸다. 위안화 고시 환율은 전날 2008년 5월 이후 11년 만에 ‘포치(破七·달러당 7위안이 넘는 것)’를 돌파했고 이날 추가 상승했다.

　런민은행은 이날 오전 달러 대비 위안화 중간 환율을 7.0136위안으로 고시했다. 전일 고시 환율은 7.0039위안보다 0.14% 상승(위안화 가치 하락)했다. 위안화 고시 환율은 지난달 31일 이후 7거래일 연속 상승했다.

　중국이 미국의 거센 반발에도 위안화 약세를 관세 및 환율전쟁 ‘무기’로 사용한다는 지적이 나오면서 미국도 중국에 대한 ‘맞불’ 제재에 나섰다. 미 상무부는 8일(현지 시간) 중국산 목제 가구에 고율의 상계관세를 물리기로 결정했다고 AP통신 등이 전했다. 상계관세는 교역 상대국 정부가 수출 경쟁력을 높이기 위해 보조금을 지급하는 상품에 적용하는 징벌적 세금이다. 중국이 지난해 미국에 수출한 나무 찬장과 화장대의 규모는 44억117만 달러(약 5조3000억 원)에 달한다.

　상무부는 이날 중국산 찬장과 화장대에 대한 상계관세 조사 결과, 중국 기업이 최저 10.97%에서 최고 229.24%의 국가 보조금을 받은 것으로 판정했다고 밝혔다. 상무부는 해당 기업으로부터 판정된 불공정 보조금 지급액만큼 현금을 징수하겠다고도 덧붙였다. 이 조치는 내년 1월 30일 미 무역위원회(ITC)의 최종 판정을 거쳐 집행된다.

　미국은 이날 중국 최대 통신장비업체 화웨이에 대한 수출규제 완화도 보류했다고 블룸버그 등이 전했다. 윌버 로스 미국 상무장관은 지난달 30일 “화웨이와 거래하게 해 달라”는 미 기업의 요청에 “다음 주까지 응답할 수 있다”고 긍정적 반응을 보였다. 하지만 양국의 대립이 격화되자 이를 무기한 연기한 것으로 풀이된다. 상무부는 5월 이란과의 거래 정황을 이유로 화웨이와 계열사를 거래제한 블랙리스트에 올렸다. 이후 화웨이와 거래를 원하는 미 기업은 반드시 정부의 사전 승인을 받아야 한다.

　한편 금융시장에서는 ‘위안화 인상’의 다음 마지노선을 달러당 7.2∼7.3위안으로 전망한다고 9일 니혼게이자이신문이 보도했다. 다음 달 1일부터 3000억 달러 규모의 중국산 제품에 대해 10%의 추가 관세를 부과하겠다고 밝힌 도널드 트럼프 미 행정부의 발표를 추산한 결과다. 3000억 달러는 중국의 전체 대미 수출의 약 60%이며, 여기에 10%의 관세를 부과하면 대미 수출 전체에 미치는 영향은 6%가 된다. 7.0136위안인 현 위안화 환율에 6%의 인상을 적용하면 달러당 7.3위안이 된다고 니혼게이자이는 분석했다. 일각에서는 위안화 환율이 7.3위안까지 상승하면 중국 기업의 달러화 부채가 급증해 위안화 투매 및 가치 추가 하락이 일어날 수도 있다고 우려하고 있다.

# 사드 처음 명시한 中국방백서… “긴장 고조” 美비난

중국 국방부가 24일(현지 시간) 발표한 2019 국방백서에서 ‘사드(THAAD·고고도미사일방어체계)’를 거론하며 한미 양국에 날을 세웠다. 백서는 “미국이 한국에 사드를 배치하면서 아시아태평양지역의 전략적 균형과 안보 이익을 엄중하게 훼손했다”고 주장했다.

　중국 국방부는 이날 ‘신시대 중국 국방’이란 제목의 약 90쪽의 백서를 공개했다. 중국은 1988년 첫 백서를 발간한 후 통상 2년에 한번씩 발간해왔다. 이번 백서는 10번째로 2015년 발간 후 4년 만에 이뤄졌다. 특히 4년 전 백서 분량은 약 20쪽이었지만 이번 백서에는 사드, 남북문제, 미국, 일본, 호주, 대만 등 다양한 주제를 담으며 중국의 주장을 노골적으로 강조했다.

　백서는 “세계 경제와 전략 중심이 아시아태평양 지역으로 옮겨지고 있다. 이 지역에서 대국끼리 게임을 하면서 지역 안전에 불확실성을 가져왔다. 미국은 아태 군사 동맹을 강화하고 군사 배치 및 간섭을 확대하면서 이 지역에 복잡한 요소를 더했다”고 미국을 비난했다.

　남북문제에서도 중국이 주도적 역할을 하겠다는 뜻을 드러냈다. 특히 “한반도에서 긍정적인 진전이 있었지만 여전히 불확실성이 남았다. 중국은 한반도 같은 분쟁지역에서 정치적으로 건설적인 역할을 하고 있다”고 자평했다.

　중국은 일본과 호주에도 강한 경계를 드러냈다. 백서는 “일본이 전후 체제를 우회하기 위해 군사 안전 정책을 변경하면서 활발한 군사 움직임을 보이고 있다. 대외 지향적인 군사 움직임이 보인다”고 경계했다. 호주도 미국과 군사 동맹을 강화하면서 아태지역 안보 플레이어로 부상하고 있다고 진단했다. 특히 대만 문제에 대해서는 “평화통일 및 일국양제 방침을 견지한다. 중국을 분열하려는 시도와 외국의 내정간섭에 반대한다”고 또다시 미국을 겨냥했다.

　남아시아 지역에서 경쟁 중인 인도에도 날을 세웠다. 백서는 “남아시아에서 인도와 파키스탄의 충돌이 있다. 또 일부 국가의 영토 및 해양 분쟁, 민족과 종교 갈등이 끊임없이 발생하고 있다”고도 분석했다.

　백서는 “중국은 국방비 및 사용처를 투명하게 공개한다”며 현황을 표로 소개했다. 2012∼2017년 국내총생산(GDP) 대비 국방비 비중 평균은 1.3%로 러시아(4.4%) 미국 (3.5%) 인도(2.5%) 프랑스(2.3%)보다 낮다고 강조했다.

# 美-中, 화웨이 불씨 둔채 무역 2차휴전

도널드 트럼프 미국 대통령과 시진핑(習近平) 중국 국가주석이 지난달 29일 일본 오사카 주요 20개국(G20) 정상회의에서 80분간의 무역 담판을 갖고 신규 관세 부과 보류와 무역협상 재개를 약속한 ‘무역전쟁 2차 휴전’에 합의했다.

　트럼프 대통령은 이날 시 주석과 양자 정상회담을 마친 뒤 기자회견에서 “우리는 협상을 계속하기로 합의했다”며 “3250억 달러(약 375조5375억 원)어치(중국산 수입품)에 대한 관세를 부과하지 않기로 합의했다”고 밝혔다. 이어 “우리는 관세 부과를 보류하고 그들(중국)은 농산품을 구매할 것”이라고 설명했다. 두 정상은 지난해 12월 1일 아르헨티나 부에노스아이레스 G20 회의에서 추가 관세 인상을 보류하고 90일간 무역협상을 진행하는 ‘1차 휴전’에 합의한 바 있다.

　트럼프 대통령은 지난달 29일 “미국의 훌륭한 기업들이 화웨이에 제품을 계속 판매할 것”이라며 중국 최대 통신기업 화웨이에 대한 제재 완화를 시사했다. 다만 “화웨이를 마지막까지 남겨둘 것”이라며 “(그 거래가) 국가안보 우려를 일으키지 않는 한”이라고 단서를 달아 무역협상의 최후 카드로 남겨뒀다. 이와 함께 “중국인 학생들이 와서 우리의 훌륭한 학교와 대학을 이용하게 하길 원한다”고 덧붙였다.

　두 정상은 전날에도 비공식 만남을 가졌으나 이날 정상회담은 이례적으로 80분간 이어졌다. 트럼프 대통령은 “만약 우리가 합의하지 못하면 (관세 부과로) 돌아갈 것”이라며 “나는 서두르지 않는다. 올바른 합의를 하길 원한다”고 말했다. 중국 외교부에 따르면 시 주석은 회담에서 “중국의 주권과 존엄 관련 문제에서 중국은 반드시 핵심 이익을 수호할 것”이라고 강조했다.

　오사카 G20 정상회의는 이날 ‘공정한 무역’의 중요성을 강조하는 ‘오사카 선언’을 끝으로 폐막했다. G20 정상들은 세계 경제의 ‘하방 리스크가 크다’며 ‘리스크에 대처하기 위해 다양한 행동을 할 것’이라고 선언에서 밝혔다. ‘보호무역주의를 반대한다’는 취지의 표현은 미국의 반대로 지난해 아르헨티나 회의에 이어 다시 제외됐다.

# 방북 임박說시진핑, 비핵화 훼방꾼은 되지 말아야

시진핑 중국 국가주석이 이달 말 주요 20개국(G20) 정상회의 전에 북한을 방문해 김정은 국무위원장과 정상회담을 할 것이라는 관측이 나오고 있다. 2·28 하노이 북-미 정상회담 결렬 이후 북한이 일체의 대화를 거부하는 상황에서 이달 말 주요 20개국(G20) 정상회의를 전후해 시 주석의 방북이 이뤄진다면 동북아 정세에 미묘한 변화를 낳는 이벤트가 될 수 있다.

　비밀을 상대국에 대한 예우로 여기는 북-중 관계의 특성 탓에 시 주석 방북은 공식 발표가 있기 전까지 확인하기 어려운 게 사실이다. 김정은 북한 국무위원장이 이미 네 차례나 중국을 방문한 만큼 올해 상반기 안에 시 주석 답방이 이뤄질 것으로 예상됐지만 하노이 회담이 결렬되면서 시 주석 방북은 미뤄졌다.

이번 시 주석 방북 추진설은 미국과의 무역·기술전쟁에다 대만·홍콩을 둘러싼 내정간섭 논란까지 날카롭게 대립하는 상황에서 나왔다. 중국은 재작년 미중 무역갈등 초기에 그랬듯이 이번에도 미중 대결의 지렛대이자 반전 카드로 북핵 문제를 꺼내들 것이라는 관측이 나온다. 시 주석으로선 김정은의 후견인으로서 영향력을 확인하는 한편 G20에서 만날 도널드 트럼프 미국 대통령에게 북한 해법을 내밀며 미중 갈등의 휴전을 노릴 수도 있다.

　요즘 북한은 한미의 거듭된 대화 재개 촉구에도 묵묵부답으로 일관하고 있다. 그간 북한의 입장을 배려하던 문재인 대통령마저 북유럽 순방에서 거듭 북한이 먼저 핵 폐기 의지를 보이라고 촉구한 것은 그만큼 북한에 대한 답답함과 실망감을 반영하는 것이다.

　이런 시점에 시 주석의 방북이 이뤄진다면 북한이 대화 재개에 나서도록 만드는 계기가 될 수도 있다. 하지만 중국의 대북 외교는 김정은의 비핵화 결단을 압박하고 지원하는 것이어야지, 북-중 밀착을 과시하며 김정은이 딴 마음을 품게 하는 것이어선 안 된다. 특히 중국이 나서 국제사회의 대북제재 전선을 무너뜨린다면 돌이키기 어려운 결과를 낳을 뿐이다. 북한이 지난해 초 협상에 나올 수밖에 없었던 것도 중국까지 제재에 동참했기 때문이었다.

# 중국이 세계를 지배하면

캐나다는 미국의 요청을 받고 중국 최대 통신기업 화웨이의 최고재무책임자(CFO) 멍완저우(孟晩舟·46) 부회장을 밴쿠버 공항에서 체포했다가 미중 무역전쟁의 급류에 빨려 들어갔다. 중국은 캐나다인 2명을 억류하며 압박했고, 멍 부회장은 보석으로 풀려났다. 중국의 뒤끝이 얼마나 심했는지 캐나다 고가 패딩 브랜드인 ‘캐나다구스’ 주가마저 급락했다.　

　절대 강자인 미국엔 침묵하면서 미국을 돕는 동맹국에는 가차 없이 보복하는 대국의 민낯이 드러난 게 처음은 아니다. 북한 미사일을 막기 위해 미국이 한국에 사드(THAAD·고고도미사일방어체계)를 배치했을 때 중국은 한국을 때렸다. 중국이 단체관광객의 한국 방문을 막으며 한국 경제를 압박했을 때 그들을 평생 친구로 생각했던 한국인들은 당황할 수밖에 없었다.

　중국은 유엔과 같은 국제무대에선 서구의 패권에 대항하는 수평적 다자주의를 강조하지만 실제 행보는 혼란스럽다. 봉건시대 제후국을 대하듯이 중국 중심의 수직적 위계질서를 강조하고 역린(逆鱗)을 건드리면 크건 작건 본때를 보이는 패권국가의 전근대성도 보인다.

　인구 2만여 명의 남태평양 작은 섬나라 팔라우도 중국에 그렇게 당했다. 하루에 태어나는 중국 신생아 수밖에 안 되는 인구를 가진 이 작은 섬나라가 대만과 국교를 맺고 있으니 ‘하나의 중국’ 원칙을 일관되게 주장하고 있는 중국에는 눈에 든 가시나 다름없었을 것이다.

　중국 정부는 지난해 외교관계가 없다는 이유로 팔라우 단체여행을 막았다. 대만 항공사가 운영하는 팔라우 퍼시픽에어웨이스는 중국인 여행객이 절반으로 줄어들자 올해 7월 중국 노선을 폐지했다. 섬에 호텔을 짓고 건물을 사들이던 중국인 큰손 투자자도 손을 놓았다. 관광 등 서비스업 비중이 경제의 80% 이상을 차지하는 팔라우가 받은 타격은 엄청났다.

　중국은 세계 인구의 약 20%를 차지하는 인구대국이라는 점에서 소수의 인구로 세계 패권을 쥐었던 영국이나 미국과는 질적으로 다르다. 관광산업마저 무기화할 수 있는 규모의 경제와 힘을 갖고 있다. 여기에다 위안화가 달러와 같은 기축통화 반열에 오른다면 미국의 독자제재처럼 중국 금융시스템에서 퇴출시키는 것만으로 한 나라 경제를 위협할 수 있게 될 것이다. 한국, 캐나다, 팔라우에서 일어난 일들은 어쩌면 중국이 세계를 지배하면 벌어질 일의 예고편에 불과할지 모른다.

　이런 중국과 싫든 좋든 등을 맞대고 함께 살아가야 하는 게 한반도의 운명이다. 대중 외교를 강화하려고 한다면 한쪽으로 기울어진 ‘친중파’보다 중국과의 관계를 긍정적으로 보면서 잠재적 위험 요인까지 따지는 ‘낙관적 현실주의자’가 훨씬 더 많아야 한다. 정치 경제 사회 문화 등에서 특정 국가에 대한 쏠림은 없는지 점검하고 대비하는 중장기 국가 전략시스템도 가동해야 한다.

　팔라우는 중국인 관광객이 전체의 절반에 육박할 정도로 커진 뒤에야 중국에 의존하는 자국 관광산업의 취약점을 깨달았다. 뒤늦게 중국의 위협에 맞서 중국인 단체관광객 대신 1인당 매출액이 훨씬 큰 유럽이나 일본인 관광객 유치를 위해 친환경 고부가가치 관광산업을 집중 육성하기 시작했다. 산호초 보호를 위해 미국 하와이에 이어 선크림 사용을 선제적으로 금지한 이유다. 최근 팔라우 당국은 중국인 관광객은 줄었지만 1인당 매출액이 늘면서 전체 관광 매출은 증가했다고 발표했다. 미중 무역전쟁과 섬나라 팔라우에서 일어난 일들은 ‘전쟁의 승패는 싸우기 전에 판가름 난다’고 했던 손자의 말을 곱씹어 보게 한다.

# 화웨이 진영 싸움 확전 조짐

중국 통신장비업체 화웨이 창업자의 딸이자 최고재무책임자(CFO)인 멍완저우(孟晩舟) 부회장이 대이란 제재를 위반한 혐의로 캐나다에서 체포된 뒤 그 후폭풍이 미중 간 무역협상으로 번지고 있다. 미중은 1일 아르헨티나에서 가진 정상회담을 통해 무역전쟁 휴전에 합의하고 90일 협상에 돌입했지만 이 문제가 협상을 망가뜨릴 수 있는 뇌관으로 부상한 것이다.

　미국 측 협상 책임자인 로버트 라이트하이저 미무역대표부(USTR) 대표는 9일(현지 시간) CBS방송 인터뷰에서 “내 생각에 (90일은) 단호한 최종시한”이라며 “내가 (도널드 트럼프) 대통령과 이야기할 때 그는 3월 1일을 넘기는 것을 말하지 않는다”고 말했다. 그러면서 “90일이 지나면 관세가 인상될 것”이라고 강조했다.

　　‘미중 정상회담 만찬 당시 트럼프 대통령이 멍 부회장 체포 사실을 알고 있었느냐’는 질문엔 “알지 못했다. 분명히 말할 수 있다”며 “무역협상은 멍 부회장의 체포에 영향을 받지 않아야 한다”고 강조했다.

　하지만 멍 부회장 체포가 미중 무역협상에 악영향을 줄 것이라는 분석이 잇따르고 있다. 뉴욕타임스(NYT)는 “멍 부회장의 구금은 미중 경제 관계를 상당히 복잡하게 만들었다”며 “중국인들의 자존심인 화웨이 문제가 분노와 놀람을 일으켰다”고 전했다. 월스트리트저널(WSJ)은 “멍 부회장 체포가 중국의 민족주의를 자극하면서 시 주석의 양보를 어렵게 만들고 있다”고 평가했다.

　전날 베이징 주재 캐나다대사를 불러 항의했던 중국 외교부는 9일 테리 브랜스태드 주중 미국대사를 초치해 체포영장 철회를 요구했다. 러위청(樂玉成) 중국 외교부 부부장은 브랜스태드 대사에게 “미국은 중국 시민의 합법적인 권리와 이해를 침해했다”며 “중국은 미국의 행동에 따라 추가적인 조치를 취할 것”이라고 말했다고 중국 외교부는 밝혔다.

　캐나다도 무역사절단의 중국 방문 계획을 취소하며 맞대응에 나섰다. 캐나다 서부 브리티시컬럼비아주는 성명에서 “무역사절단의 방중 계획 취소는 멍 부회장과 관련한 사법적 절차 때문”이라고 밝혔다.

　결국 경제동맹체인 미국과 캐나다가 한 몸처럼 움직이고 있고, 자존심이 강한 중국도 물러서지 않으면서 강대강 대치 국면이 장기화될 가능성이 거론되고 있다. AP통신은 중국이 이번 사건에 대한 보복으로 캐나다인을 억류할 가능성이 있다고 전했다.

　한편 캐나다에서 체포된 멍 부회장은 자신의 결백을 주장하며 건강 문제를 들어 심리 기간 중 보석을 요청했다. 로이터통신 등에 따르면 멍 부회장은 9일 공개된 법정 진술서에서 “미국 신병 인도에 맞서기 위해 밴쿠버에 체류하고자 하며 인도된다면 미국에서 혐의에 맞서 싸우겠다”고 밝혔다. 이어 “심각한 고혈압과 건강에 대한 우려로 미국 인도 절차가 진행되는 동안 보석금을 내고 석방되기를 바란다”고 말했다. 7일 열린 첫 보석 심리에서 캐나다 검찰은 멍 부회장이 대이란 제재 위반 혐의를 받고 있다며 보석 불허를 요청했다. 다음 심리는 10일 재개된다.

# “한국인들, 중국이 기만할 것 우려해”

　“한국인들은 중국이 한국을 기만할 것이라고 우려한다.”

　천샹양(陳向陽) 중국 현대국제관계연구원 한반도연구실 부연구원은 5일 중국 장쑤(江蘇)성 옌청(鹽城)시에서 열린 제6차 한중 공공외교포럼에서 “중국에 대한 한국의 자신감이 좀 떨어져 보인다”며 이같이 주장했다. 한중 외교부가 공동 주최하고 한국국제교류재단과 중국공공외교협회가 주관한 이번 포럼은 ‘한중관계 향후 10년의 버팀목이 될 전면적 신뢰 구축’이라는 주제로 4, 5일 개최됐다. 한국의 사드(THAAD·고고도미사일방어체계) 배치에 대한 중국의 보복 이후 중국에 대한 부정적 여론이 부쩍 높아진 한국을 바라보는 중국 측의 속내를 엿볼 수 있는 자리였다.

　천 부연구원은 5일 “사드가 한중 상호 신뢰에 큰 (부정적) 영향을 줬다”며 “서로 피해를 주는 마지노선을 넘지 말아야 한다”고 주장했다. 이어 “중국은 최선을 다해 한중 협력의 중요성을 강조할 필요가 있고 한국은 한중 협력의 가치를 인식해야 한다”고 덧붙였다. 자오치정(趙啓正) 전 중국 국무원 신문판공실 주임은 “민족주의 정서가 (한중) 양자관계와 외교에 영향을 준다”며 “중국의 학자들이 (중국) 청년들에게 영향을 줘 극단적 민족주의를 극복해야 한다. 한국도 마찬가지”라고 지적했다.

　중국 측 인사들은 한미동맹 강화가 중국의 국익에 해를 끼친다는 인식도 드러냈다. 천 부연구원은 “한미동맹을 해체하라는 게 아니라 한미동맹이 강화돼 중국에 피해를 줄 것을 우려하는 것”이라고 주장했다.

　이호철 인천대 중국연구소장은 “한국은 한미동맹을 완화, 축소하거나 철폐하고 한중관계로만 외교정책을 돌릴(집중할) 수 없다”며 “한중 양국이 처한 이런 구조적 조건을 상호 이해하는 바탕에서 한중관계를 이끌어가야 한다”고 지적했다.

# 靑는 “中이 양보”-中은 “핵심이익 수호” 다른말

도널드 트럼프 미국 대통령이 2일(현지 시간) 트위터에 “중국이 현재 40%인 미국산 자동차에 대한 관세를 삭감·철폐하는 데 동의했다”고 밝혔다. 1일 미중 정상회담에서 미중 관세전쟁 ‘90일 휴전’에 합의한 뒤 중국이 즉각 미국에 양보하고 나섰음을 강조한 것이다.

　3일 영국 파이낸셜타임스(FT)에 따르면 이달 12일경 류허(劉鶴) 중국 부총리가 협상팀을 이끌고 워싱턴을 방문해 미국과 후속 무역 협상을 벌일 것으로 알려졌다. 하지만 중국 정부와 관영매체들은 미국 백악관과 달리 ‘협상 시한이 90일이고 그 안에 협상이 타결되지 못하면 미국이 다시 추가 관세를 부과할 것’이라는 사실을 전혀 밝히지 않고 있다. 오히려 중국 관영매체들은 “중국이 무역 협상과정에서 핵심 이익을 결연히 수호했다”는 점을 강조하고 있다.

　중국의 이런 태도는 트럼프 대통령이 요구하는 중국 경제의 구조개혁 과정에서 일방적인 양보는 없다고 천명한 것이어서 향후 무역 협상이 가시밭길을 걸을 것이라는 전망이 나온다. FT도 미중 정상의 합의를 “깨지기 쉬운 관세 휴전”이라고 표현하면서 “후속 협상은 어려움투성이”라고 지적했다.

　미중 관계 전문가인 왕융(王勇) 베이징(北京)대 교수는 홍콩 사우스차이나모닝포스트(SCMP)에 “중국은 여전히 경제, 특히 첨단기술 분야에서 미국에 의존하지 못하게 되는 최악의 시나리오에 대비해야 한다”고 지적했다. 그는 “중국이 (미국 기업의) 시장 접근성과 지식재산권 보호를 확대하는 것을 포함해 산업 정책을 조정하는 것은 가능하지만 경제 구조의 근본적인 변화는 불가능하다”고 말했다.

　이는 미중이 후속 협상의 영역으로 남겨둔 미국의 중국 경제 구조조정 요구가 시진핑 정권의 권력 유지 및 중국의 국가 주도 사회주의 시장경제 발전과 직결되기 때문이다. 미국은 중국의 기술 이전 강요, 지식재산권 침해, 비관세장벽 등 문제 해결 등을 요구하고 있다. 이는 중국이 2025년까지 최첨단 기술 제조업 분야에서 미국을 넘어서겠다는 ‘중국 제조 2025’를 무력화시키겠다는 것이다. 하지만 중국은 미국의 이런 요구를 ‘중국 특색 사회주의 제도’를 인정하지 않고 중국의 주권을 침해하는 불평등 협상이라며 반발하고 있다. 이 때문에 중국에서도 향후 무역 협상을 낙관할 수 없다는 우려가 나오는 것이다.

　미중 정상회담에서의 휴전을 놓고 “백악관 내의 온건파가 득점했다”는 평가도 나온다. 트럼프 대통령의 곁에서 강경 일변도의 무역정책을 주장하며 대중국 압박을 주도해 온 피터 나바로 백악관 무역·제조업 정책국장의 영향력이 줄어든 반면 타협을 주장해 온 래리 커들로 백악관 국가경제위원회(NEC) 위원장과 스티븐 므누신 재무장관의 입지가 커졌다는 것이다.

　인터넷 매체 악시오스는 ‘G20에서 이뤄진 외교의 승리’라는 제목의 기사에서 “이번 담판 결과는 백악관 내 초강경 보호무역주의자들에 대한 글로벌리스트(세계적 관여주의자)들의 승리”라며 “누구도 트럼프 대통령이 시 주석과의 만찬을 준비해올 것으로 생각하지 않았지만, 예상을 뛰어넘어 두 나라의 무역전쟁에 대한 휴전 합의가 이뤄졌다”고 보도했다.

# 대만 민진당 선거 참패 뒤엔... 中의 경제압박 있었다

“돈 벌어서 대만의 민진당 후원하는 것 아니냐.”

　중국 대륙에서 활동하는 대만 사업가 중에는 이런 얘기를 공개적으로 듣는 경우가 있다고 중국의 한 전문가는 전했다. 2016년 1월 독립 성향 민진당의 차이잉원(蔡英文) 총통이 당선된 이후 중국의 민진당 압박 분위기를 보여준다.

　지난달 24일 대만 지방선거에서 민진당이 국민당에 참패한 원인을 두고 중국의 경제적 압박도 주요한 요인 중 하나라는 분석이 나온다.

　이번 선거에서 광역 지방자치단체장 중 민진당 소속은 13명에서 6명으로 줄어든 반면 야당인 국민당 소속 당선자는 6명에서 15명으로 늘었다. 민진당은 20년간 지켜 온 텃밭인 제2 도시 가오슝(高雄) 시장직도 국민당 한궈위(韓國瑜) 후보에게 뺏겼다.

　중국은 차이 총통 집권 이후 대만행 관광객 쿼터를 축소하는가 하면 일부 농산물과 수산물 수출 제한 조치를 내렸다. 대만에서 공급받던 부품을 대륙 내에서 자체 조달하는 이른바 ‘홍색 공급망’의 가동 강화도 중소기업 부품 산업이 주력인 대만 경제에 큰 타격을 줬다.

　박한진 KOTRA 중국지역본부장은 30일 동아일보와의 통화에서 “차이 총통 집권 이후 중국은 대만으로 가는 단체관광객 규모를 40% 가까이 줄이는 등 제재를 가했다. 관광 분야에서 특히 어려움이 컸다”고 말했다.

　차이 총통 취임 당시 22개국이던 대만의 수교국이 올해 17개국으로 줄어들 만큼 외교적 고립 작전도 강화됐다. 이번 선거에서 민심이 민진당에 등을 돌린 주요인은 민생경제 악화다. 지난해 총 수출액 중 41.1%(홍콩 포함)를 차지할 정도로 대만의 중국 대륙에 대한 경제 의존도가 압도적인 상황에서 중국의 민진당 압박이 계속되자 대만 유권자들이 ‘명분보다는 실리’ ‘양안 갈등보다는 안정’을 선택한 것이라는 분석이 나온다.

　친중 성향 국민당 마잉주(馬英九) 전 총통의 10년 집권 후 대만 유권자들이 민진당의 차이 총통을 선택할 때는 얼마 정도의 양안 갈등도 예상했다. 차이 정부는 동남아와의 교류 확대 등 ‘신남방 정책’으로 돌파하려고 했다. 하지만 이번 선거는 “중국과 등을 돌리면 경제가 너무 힘들다”는 것을 보여주는 것이라는 관측이 많다.

　지방선거에서 차이 전 총통의 기반 지지층인 청년층 이탈이 심한 것이 이를 잘 보여준다. 강준영 한국외국어대 중국정치경제학과 교수는 “중국에서 취업을 희망하는 청년이 69%에 달한다”며 “대만 내 청년실업 문제가 심각한 상황에서 민진당이 중국과 계속 엇박자를 내자 청년 지지층이 등을 돌린 것”이라고 설명했다. 올해 3분기(7∼9월) 대만의 청년세대 실업률은 12.29%로 전체 실업률(3.76%)을 크게 웃도는 것으로 알려졌다.

　중국 대륙에서 경제활동을 하는 대만인은 150만여 명으로 추산된다. 이 중에는 전기·전자 부품 공장 임직원도 많다. 페이퍼컴퍼니를 세우거나 통계에 잡히지 않는 방식으로 경제활동을 하는 사람도 적지 않다. 중국에 진출한 사업가들은 국민당 지지와 집권을 위해 선거 때면 비행기를 타고 대만으로 가 투표를 하고 돌아올 정도로 적극적이다.

　중국 국무원 대만사무판공실 마샤오광(馬曉光) 대변인은 지난달 28일 기자회견을 열고 “중국과 대만의 도시 차원의 교류가 확대될 것”이라며 “중국 단체관광객이 가오슝으로 향하고 있다”고 말했다. 야당이 승리한 지역을 중심으로 교류를 확대하는 등 집권 민진당을 압박하면서도 친중국 지자체에 유화적인 조치가 병행될 수 있음을 시사했다.

# 中 과학자들 “유전자 편집 아기 강력 규탄” 성명

세계 최초로 유전자를 편집한 아기의 출생에 성공했다는 한 과학자의 주장으로 중국이 논란에 휩싸였다. 중국 당국은 사실관계 조사를 지시했다. 이 과학자와 관련된 대학 등은 모두 실험과의 연관성을 부인하고 나섰다.　

　중국인 과학자 허젠쿠이(賀建奎)는 26일 “유전자 편집 기술을 통해 에이즈(AIDS)에 면역력을 갖도록 유전자를 편집했다”며 “불임 치료를 받은 부부 7쌍의 배아에 대해 유전자 편집을 진행했고 이 중 한 쌍이 여자 쌍둥이를 출산했다”고 주장했다. 유전자 편집은 비정상 유전자를 잘라 내거나 정상 유전자를 삽입하는 것이지만 배아 유전자를 편집할 경우 다음 세대에 변형된 유전자가 유전될 수 있어 세계적으로 금지돼 왔다.

　중국인 과학자 122명은 이날 웨이보(중국의 트위터 격)에 “강력히 규탄한다”는 성명을 발표했다. 이들은 “유전자 편집은 (기술적으로는) 전혀 새로운 게 아니다. (하지만) 불확실성이 존재하고 윤리 문제에서 큰 위험이 존재한다”며 “엄격한 윤리와 안전성 검사를 거치지 않은 채 (다음 세대로) 유전될 수 있는 태아의 유전자를 편집한 시도에 결연히 반대한다”고 밝혔다. 이들은 “판도라의 상자가 열렸다”며 “만회하지 못하는 단계에 이르기 전에 상자를 닫을 기회가 아직 있다. 국가는 엄격하게 감독 관리를 시행해야 한다”고 지적했다. 중국 국가위생건강위원회는 광둥(廣東)성 위생건강위원회에 실태 조사를 지시했다.

　허젠쿠이가 재직 중인 난팡(南方)과학기술대 학술위원회는 “허젠쿠이는 올해 2월부터 무급 휴직 중이며 연구는 대학 바깥에서 진행된 것으로 실험 내용에 대해 알지 못한다”고 말했다.

# 中 모래폭풍 덮쳐...미세먼지에 황사까지 ‘컥컥’

27일은 대기 정체로 쌓인 미세먼지에 중국발 스모그와 황사가 겹치면서 숨이 턱턱 막히는 하루였다. 황사가 한반도에 영향을 준 것은 올가을 들어 처음이다.

　기상청과 국립환경과학원에 따르면 서울의 초미세먼지 농도는 이날 오후 3시 현재 m³당 48μg(마이크로그램·1μg은 100만분의 1g)으로 ‘나쁨’ 수준이었다. 경기와 인천은 각각 이날 오전 10시와 낮 12시에 초미세먼지 주의보가 발령됐다. 주의보는 m³당 75μg 이상인 초미세먼지가 2시간 이상 지속될 때 발령된다. 23일부터 한반도 상공 대기가 정체되면서 며칠째 국내에서 발생한 미세먼지가 그대로 쌓인 데다 이날 중국으로부터 밀려온 스모그가 더해져 농도가 더 짙어졌다.

　오후에는 황사까지 겹쳤다. 황사가 우리나라에 유입된 것은 5월 25일 이후 6개월 만이다. 26일 중국 내몽골 부근에서 발생한 황사 가운데 일부가 북서풍을 타고 서해상으로 밀려 내려와 27일 오후부터 한반도에 영향을 줬다. 중국으로부터 우리나라까지 날아온 황사는 대부분 초미세먼지(PM2.5·지름이 2.5μm 이하)보다 입자가 큰 미세먼지(PM10·지름 2.5μm 초과∼10μm 이하)였다.

　황사의 영향으로 이날 오후 1시 백령도의 미세먼지(PM10) 농도가 m³당 471μg까지 치솟았다. ‘매우 나쁨’(m³당 151μg 이상) 기준을 3배 이상으로 넘어선 것이다. 인천과 서울은 이날 오후 내내 가시거리가 2∼3km로 맑은 날의 5분의 1 수준에 그쳤다. 황사는 28일 아침까지 서울을 비롯한 수도권에 영향을 줄 것으로 보인다.

　다만 28일에는 전국의 미세먼지가 ‘보통’ 수준을 회복할 것으로 전망된다. 국립환경과학원은 “황사를 몰고 온 북서풍이 한반도 상공의 미세먼지를 외부로 밀어내 일부 남쪽 지역을 제외하면 미세먼지 농도가 크게 높지 않을 것으로 예측된다”고 말했다.

# AI 기술 미국 따라잡는 중국…‘삼성 반도체’도 잡는 날 오면

“그간 (미중 무역전쟁은) 고위급에서 싸우면 그만이라고 생각했어요. 하지만 최근 우리에게 영향을 준다고 확실히 느껴집니다. 우리 기업들에 압박이 정말 큽니다. 무역전쟁이 빨리 끝났으면 좋겠어요.”

　삼성전자가 15일 중국 베이징(北京)에서 연 ‘삼성 미래기술 포럼’ 패널 토론 현장. 상하이쑤이위안(上海燧原)과학기술유한공사 자오리둥(趙立東) 최고경영자(CEO)가 미중 무역전쟁으로 중국의 인공지능(AI), 반도체 산업이 처한 어려움을 비교적 솔직하게 털어놓았다.　

　자오리둥의 걱정에도 아랑곳없이 미국 상무부 산업안보국은 19일 AI, 로봇공학 등 첨단 기술 14개 항목에 대한 수출 제한 조치를 예고했다. 중국을 거론하지 않았지만 중국의 AI 반도체 굴기(굴起)를 막기 위해 휘두른 칼임이 분명했다. 중국도 가만있지 않는다. 시진핑(習近平) 중국 국가주석은 이달 초 “AI 핵심 기술을 선점, 장악하라”는 주문을 쏟아냈다.

　15일 포럼 패널로 나선 중국 바이두(百度)클라우드 셰광쥔(謝廣軍) 부사장은 “현재 미중 간 인공지능은 중국과 미국 모두 시작 단계다. 중국에 매우 좋은 앞날을 기대한다”며 중국 정부의 지원 등을 중국 AI 산업의 비교우위로 내세웠다. 미중 격차가 크지 않기 때문에 미국의 압박에도 문제없다는 자신감을 내비친 것이다.

　자오리둥도 “컴퓨터 첨단 반도체는 하루아침에 따라잡지 못하지만 AI는 (미국과) 출발선 사이의 거리가 그리 멀지 않다”고 말했다. 그는 “5년 전 중국에서 AI 반도체를 얘기하는 건 완전히 허황된 얘기로 들렸지만 지금은 정부의 지지, 자본 시장의 열광적인 성원, 국제적 기술과의 연결 등으로 발전이 가능해졌다”고 강조했다.

　삼성전자 DS(디바이스솔루션)부문 중국총괄 최철 부사장은 이날 포럼 시작 전 중국의 반도체 굴기에 대해 “우리가 경쟁력이 월등하다고 생각한다”고 말했다. 중국의 메모리반도체 생산 계획에 대해서도 “1등 하는 기업(삼성전자)은 시장을 따라가는 게 아니라 스스로 시장을 만들어낸다”며 자신감을 보였다.

　하지만 다음 날인 16일 중국 국가시장감독관리총국은 삼성전자, SK하이닉스, 마이크론의 메모리반도체 시장 독점 행위에 대한 “대량의 증거 자료를 확보했다”며 제재 가능성을 시사했다.

　세계 최대 반도체 시장인 중국이 한국 반도체 산업을 견제하겠다는 발톱을 드러낸 것이다. 미국의 첨단산업 기술 억제에 사력을 다해 맞서려는 중국은 반도체 산업에서도 한국을 뛰어넘기 위해 필사적으로 달려들 것이다.

　15일 포럼 토론 막판 샤오미(小米) AI제품부 지쉬(季旭) 사장 등 중국 기업 패널들은 “삼성은 중요한 협력 파트너” “메모리반도체 등에서 삼성의 더 많은 지원과 협력을 희망한다” “AI 산업은 반도체 의존도가 매우 높기 때문에 삼성에 크게 의지한다” 등의 덕담을 건넸다.

　지쉬는 “현재 미중 무역마찰은 삼성에 더욱 큰 기회”라고 말하기도 했다. 토론 사회를 맡은 삼성반도체 중국 화베이(華北)지역 어우양지(歐陽基) 부대표는 자오리둥의 무역전쟁 걱정에 “걱정 말라. 삼성이 있다”며 농담을 건넸다.

　하지만 포럼에 참석한 첨단기술 중국 기업들과 중국 정부는 “반도체에서 삼성을 제쳤다. 이제 삼성은 더 이상 필요 없다”고 말할 날도 하루빨리 오기를 고대하고 있는 듯했다.

# 中 3분기 성장률 6.5%...금융위기 이후 최저

중국의 3분기(7∼9월) 국내총생산(GDP) 성장률이 글로벌 금융위기 이후 최저치를 기록했다.

　중국 국가통계국은 3분기 GDP가 지난해 같은 기간 대비 6.5% 증가했다고 19일 밝혔다. 이는 글로벌 금융위기를 겪은 2009년 1분기 성장률(6.4%) 이후 최저치다. 이로써 중국의 분기별 GDP 성장률은 지난해 1분기 6.9%를 기록한 이래 하락세를 이어가고 있다. 올해 1분기와 2분기에는 각각 6.8%와 6.7%를 기록했다.

　중국 당국은 1∼3분기 평균 GDP 성장률이 6.7%이고 올해 목표치가 6.5%인만큼 종합적으로 볼 때 안정세를 유지하고 있다고 평가했다. 국가통계국은 이날 성명에서 “복합하고 엄중한 국제 정세 속에서도 시진핑 동지를 핵심으로 하는 당 중앙의 영도 하에서 국민경제는 전체적으로 안정적으로 운영되는 가운데 경제 구조 또한 부단히 선진화하고 있다”고 밝혔다. 이날 기자간담회에서도 마오셩용 국가통계국 대변인(毛盛勇)은 내년 성장 전망에 대해서도 “외부 불확실성이 커졌지만 우리는 외부 압력에 충분히 대응할 수 있을 것으로 보고 있다”고 밝혔다.

　다만 중국 GDP가 하락세를 이어가는 만큼 미중 무역전쟁의 여파가 본격화되는 4분기(10∼12월)부터는 중국 GDP 성장률이 더 큰 타격을 입을 수 있다는 전망도 나온다.

　한편 류허 경제담당 부총리는 이날 인민일보 등 관영 매체와의 인터뷰에서 “현재 미국과 접촉하고 있다”면서 “중미 무역마찰이 시장에 영향을 미쳤으나 솔직히 말해 실제 영향을 미치는 것보다는 심리적인 영향이 크다”고 밝혔다.

# 존엄한 국가 흥얼거린 죄...중 인터넷 스타 철창행

중국의 유명 인터넷 스타가 인터넷 개인 방송에서 중국 국가(國歌)를 장난스럽게 흥얼거렸다는 이유로 구류 처분을 받았다. 시진핑(習近平) 국가주석 시대 중국의 언론 인터넷 등 사회 통제가 전방위로 확대되는 단면을 보여준다는 지적이 나온다.

　상하이(上海)시 공안국(경찰청)은 14일 인터넷 생방송 진행자인 양카이리(楊凱莉·20)가 중국 국가(國歌)법을 위반해 행정구류 5일 처분을 내렸다고 밝혔다. 상하이 공안은 양카이리가 ‘엄숙히 서서 국가를 불러야 하고 불경한 행위를 하면 안 된다’는 법을 위반했다며 인터넷 방송도 법 적용의 예외 지역이 아니라고 강조했다.

　인터넷 생방송 진행자인 양카이리는 리거(莉哥)라는 예명으로 알려져 있다. 중국의 인기 동영상 앱 더우인('音)에서 팔로어가 4400만 명에 달하는 ‘왕훙(網紅)’이다. 왕훙은 중국에서 인터넷 스타를 가리킨다.　

　양카이리는 7일 중국판 유튜브인 후야(虎牙)에서 온라인 음악 축제를 소개하는 인터넷 생방송을 시작하면서 앉은 채로 “일어나라, 노예가 되기를 거부하는 인민이여”로 시작하는 중국 국가를 흥얼거리듯 불렀다. 불과 3초 정도밖에 안 되는 시간이었지만 “국가의 존엄을 모욕했다”는 비난이 쏟아졌다.

　이후 양카이리가 “국가를 진지하게 부르지 못해 진심으로 사과한다. 조국에도 사과한다”는 사과문을 두 차례 소셜미디어에 올렸지만 논란은 확산됐다. 후야 측은 양카이리의 방송 계정을 정지시켰다. 결국 양카이리는 사건 발생 일주일 만에 경찰의 구류 처분을 받는 신세가 됐다. 상하이 공안은 “법률의 마지노선에 도전하고 공공질서와 미풍양속을 위반하는 행위를 법에 따라 결연히 처벌하겠다”고 밝혔다.

# 中 “단둥-평양-서울-부산 연결”…일대일로 한반도 확장 첫 명시

중국이 북-중 접경지역인 랴오닝(遼寧)성 단둥(丹東)을 관문 삼아 일대일로(一帶一路·인프라 투자 등을 통한 중국의 해외 경제영토 확장)를 한반도로 확대하겠다는 계획을 처음 공개했다.

　랴오닝성 정부는 최근 랴오닝일보가 보도한 ‘랴오닝 일대일로 종합실험구 건설 총체 방안’ 전문에서 “단둥을 관문으로 한반도 내륙으로 연결한다”고 명시해 일대일로가 한반도로 확장된다는 점을 분명히 했다. 랴오닝성 정부는 이 문건에서 단둥∼평양∼서울∼부산을 철도와 도로, 통신망으로 상호 연결하겠다고 밝혔다. 그러면서 이 연결의 성격을 “남부 항구로 직통하는 것”이라고 설명했다. 일대일로를 태평양으로 확장하기 위해 부산까지 뻗어나가겠다는 의도를 드러낸 것으로 풀이된다. 신의주가 포함된 단둥∼평양 도로 건설 계획도 밝혔다.

　문건은 또 신의주와 단둥 사이 압록강의 섬 황금평에 있는 북-중 경제구, 단둥의 북-중 호시(互市)무역구를 단둥 내 중점 개발 개방 실험구와 함께 대북 경제협력의 중요한 지지대로 만들겠다고 명시했다. 중앙정부가 적절한 시기에 단둥특구를 건설하도록 노력하고 랴오닝성 선양(瀋陽) 다롄(大連) 단둥 공항과 북한 및 러시아 극동 도시 간 항공편 운항도 강화하기로 했다. 단둥 호시무역구를 국가 간 온라인 전자상거래 플랫폼으로 지원한다는 계획도 공개해 북-중 간 전자상거래가 현실화될 가능성도 커지고 있다. 랴오닝성 정부는 “북-중 양국 지도자의 중요한 합의를 지도로 삼아 대북 협력을 견고하게 계획한다”고 밝혀 일대일로를 한반도로 확장하려는 계획이 시진핑(習近平) 중국 국가주석과 김정은 북한 국무위원장의 3차례 정상회담에서 합의된 내용임을 시사했다.

　일대일로를 한반도로 확장하겠다는 계획은 랴오닝성을 허브로 하고 중국 한국 북한 일본 러시아 몽골이 협력하는 동북아경제회랑 건설과 함께 추진된다. 랴오닝성 정부는 “중국-러시아-몽골 경제회랑과 한중일+X 모델을 융합해 6개국 협력을 전면 심화하는 동북아 운명공동체를 건설하겠다”고 밝혔다. 한반도를 포함한 동북아 지역 개발을 랴오닝성을 중심으로 중국이 주도하겠다는 의도를 드러낸 것이다. 한중일+X 모델은 올해 5월 리커창(李克强) 중국 총리가 한중일 정상회의에서 제안한 것으로 한중일 자유무역협정(FTA)과 함께 다른 국가와의 자유무역 협력도 추진하자는 내용이다.

　랴오닝성 정부는 이를 위해 단둥∼훈춘∼러시아 블라디보스토크로 연결되는 철도 건설과 단둥항에서 블라디보스토크항으로 연결되는 해상 통로를 동시에 추진하겠다고 밝혔다. 횡으로는 북-중 접경지역을 따라 중국과 러시아를 연결하고, 종으로는 중국과 한반도를 연결하겠다는 것이다. 문건은 일대일로 동북아 관문의 지위가 두드러지는 시기를 2030년으로 명시해 이번 계획을 2030년까지 완성하는 것이 목표임을 시사했다. 시 주석은 12일 블라디보스토크에서 열린 동방경제포럼 연설에서 미국의 일방주의와 대비시킨 ‘동북아경제권’을 주창했다. 일대일로의 한반도 확장이 미국과의 패권 경쟁을 위한 것임도 배제할 수 없는 것이다.

　중국의 일대일로 한반도 확장 계획은 문재인 정부의 남북 경제협력 계획인 한반도신경제지도의 서울∼평양∼신의주∼단둥 고속철도 연결 계획과 겹친다. 외교 소식통은 “한국과 중국이 북한 인프라 투자 개발에 대해 협력하는 계기가 될 수도 있지만 중국이 신경제지도를 일대일로에 흡수하려 할 수도 있다”고 지적했다. 실제 랴오닝성의 철도 연결 계획 등 한반도 확장 계획은 서울과 부산이 포함돼 있음에도 북한과의 협력에서만 강조되고 한국과의 부분에서는 전혀 언급되지 않았다.

　유엔 안전보장이사회의 대북 제재가 유지되는 상황에서 대북 제재 위반 가능성이 있다는 지적도 나온다. 홍콩 사우스차이나모닝포스트(SCMP)는 “제재가 있는 한 중국이 지금 당장 계획을 추진하지는 않을 것”이라고 지적했다.

# 시진핑 9•9절 방북 사실상 무산... 대타 왕후닝 유력

북한의 정권수립(9·9절) 70주년을 기념해 시진핑(習近平) 중국 국가주석 대신 왕후닝(王호寧) 당 중앙서기처 서기 겸 정치국 상무위원이 방북할 것으로 3일 알려졌다.

　복수의 정부 당국자와 중국 소식통들은 “3, 4일 베이징에서 열리는 ‘중국-아프리카 협력포럼 정상회의에 외교력을 집중하고 있는 시 주석이 9·9절에 맞춰 방북하는 것은 물리적으로 어렵다”며 “북-중 관계를 고려해 영향력 있는 왕 서기를 보내 격을 갖추려 할 것”이라고 말했다. 이미 지난달쯤 시 주석의 방북이 불투명해진 뒤 왕 서기가 가는 것으로 정리된 분위기였다는 전언도 나온다.

　왕 서기는 북한 문제와 사상·선전을 담당하고 있어 ‘대리 방북’의 적임자라는 평이다. 올해 세 차례 김정은 북한 국무위원장이 중국을 방문했을 때 모두 영접을 나갔으며 북-중 정상회담에 배석했다. 박병광 국가안보전략연구원 책임연구위원은 “왕 서기는 미국 방문학자 경험이 있는 유일한 상무위원으로 향후 대미관계 조율이나 북한의 개혁개방과 관련해 조언할 수 있는 적절한 인물”이라고 말했다.

　외교가는 시 주석의 방북이 사실상 무산된 데는 도널드 트럼프 미국 대통령의 중국 비난과 경고가 작용했을 것으로 보고 있다. 김한권 국립외교원 교수는 “미중 무역분쟁이나 대만 문제보다 한반도 문제가 정책 우선순위에서 밀리는 상황인데 시 주석이 굳이 정치적 부담을 지고 긁어 부스럼을 만들 이유가 없다”고 분석했다.

　한편 북한이 2월 건군절과 유사한 수준으로 9·9절 열병식을 준비하고 있으며 대륙간탄도미사일(ICBM) 공개 징후도 포착되지 않은 것으로 알려졌다. 통일부 당국자는 “북한이 열병식에 물자를 쏟아부을 여력이 별로 없는 것으로 보고 있다”고 말했다.

# 中군용기, 한달만에 또 KADIZ 침범

중국 군용기 1대가 29일 동해와 남해에서 한국방공식별구역(KADIZ)에 무단으로 진입해 우리 공군 전투기가 대응 출격했다. 중국 군용기의 KADIZ 진입은 지난달 27일 이후 한 달여 만이다. 올 들어선 5번째다.

　군 당국에 따르면 이날 오전 7시 37분경 Y-9 정찰기로 추정되는 중국 군용기 1대가 이어도 서남쪽 KADIZ로 처음 진입한 후 대한해협을 따라 동해상으로 이동하면서 KADIZ 진입과 이탈을 반복했다. 이어 경북 포항 동북방 약 74km 지점을 거쳐 강원 강릉 동쪽 약 96km 상공까지 북상한 후 기수를 남쪽으로 돌려 진입한 경로를 따라 비행하면서 오전 11시 50분경 KADIZ를 완전히 빠져나갔다.

　군은 F-15K 등 전투기 10여 대를 긴급 출격시켜 중국 군용기에 대해 추적 감시 비행과 경고방송 등 전술 조치를 취했다고 밝혔다. 또 한중 직통망으로 우발적 충돌을 일으킬 수 있는 긴장 고조 행위 중단을 경고했다. 하지만 중국 군용기는 이를 무시한 채 4시간여 동안 KADIZ에서 비행을 계속했다. 중국 측은 “국제 공역에서 국제법을 위반하지 않고 정상적인 훈련 비행을 한 것”이라고 응답한 것으로 알려졌다. 이어도 주변 공역은 KADIZ를 비롯해 일본과 중국 방공식별구역이 중첩되는 곳이다.

　앞서 국방부는 지난달 중국 군용기의 KADIZ 무단 진입 직후 주한 중국국방무관을 초치해 엄중 항의하고, 재발 방지를 촉구한 바 있다. 군 당국자는 “중국군이 한반도 주변을 정찰하고 정찰기의 장거리 비행 능력을 과시하고자 KADIZ 진입 비행을 반복하는 것으로 보인다”고 말했다.

# 이란 원유 최대수입국 中, 美제재에 전전긍긍

이란의 최대 무역 파트너이자 이란 원유 최대 수입국인 중국이 7일부터 재개된 미국의 대(對)이란 경제·금융 제재로 고민에 빠졌다. 중국은 미국의 이란 제재에도 이란과 경제무역 관계를 유지하겠다고 밝혔지만 미국과의 무역전쟁으로 적지 않은 타격을 받고 있는 상황에서 또 다른 전선을 형성하는 데 대한 부담으로 전전긍긍하는 모습이다.

　중국 외교부는 8일 “이란과의 상업 협력은 개방적이고 투명하며 공정합법하다. 어떤 유엔 안보리 결의도 위반하지 않았다”며 “중국은 일방적 제재와 미국 국내법을 국외에 적용하는 방식을 반대해 왔다”고 강조했다. 지난해 중국과 이란의 무역 총액은 370억 달러(약 41조3179억 원)다. 전년 대비 20% 증가한 액수다. 중국은 이란에 대형 댐과 발전소, 교통시설 등 기초 인프라 건설을 위한 대규모 차관도 제공하고 있다.

　11월 발효될 2단계 제재 조치인 이란 원유 거래 차단은 중국에 더 부담을 안길 것으로 보인다. 중국이 이란으로부터 수입하는 원유는 하루 평균 65만 배럴. 현재 시장가치로 150억 달러(약16조7745억 원)어치다. 중국의 전체 원유 수입량 중 7%가 이란산이다. 이란으로선 수출량의 4분의 1이 넘는다. 중국 국유 석유기업들은 이란 주요 유전에 수십억 달러를 투자한 것으로 알려졌다.

　미국의 이란 제재가 중국 경제이익을 침해한다는 점에서 중국은 반발할 수밖에 없는 상황이다. 하지만 무역전쟁 과정에서 미국의 고율 관세 부과에 똑같은 규모의 보복 관세로 맞불을 놓는 것과 달리 이란 제재 문제와 관련해서는 중국의 속내가 복잡하다. 무역전쟁은 미국의 보호무역주의에 맞서 자유무역 이념을 수호한다는 명분으로 유럽과 다른 아시아 국가 등 국제사회에 공동 대응을 호소할 수 있었다.

# 中 vs 대만 올림픽 모델 충돌

“대만의 일부 정치세력이 2020년 도쿄 올림픽 때 타이완(Taiwan·대만)으로 참가 표기를 하자는 국민투표를 추진하면서 ‘올림픽 모델’에 도전하고 있다. 이 때문에 2019년 (대만) 타이중에서 열릴 예정인 제1회 동아시아청소년경기대회가 정치적 위협과 방해를 받고 있다.”

　중국이 주도하는 동아시아올림픽위원회(EAOC) 회의가 24일 베이징에서 열렸다. 위원장 류펑(劉鵬)은 타이중시의 대회 개최권 박탈을 주장하면서 위원들의 거수 표결로 개최권 박탈 여부를 결정할 것을 제안했다. 류펑은 중국 전국인민정치협상회의 외사위원회 부주임을 맡고 있는 중국 측 인사다.

　이날 회의에는 위원장 류펑과 중국 대만 한국 북한 일본 몽골 홍콩 마카오의 위원 등 모두 9명이 참석해 있었다. 류 위원장의 발언에 위원들은 술렁였다. 대만 측 위원은 “내년 대회는 대만이 처음으로 올림픽 룰을 적용해 개최하는 대회다. 동아시아 청소년들의 대회 참가 기회를 빼앗지 말라”고 항변했다. 일본 측 위원은 “이미 2014년에 타이중시 개최가 확정됐는데 지금 개최권 박탈을 결정하려는 것은 지나치다”는 의견을 냈다.

　하지만 류 위원장의 주장대로 표결은 이뤄졌고 개최권 박탈에 찬성 7표가 나왔다. 4년을 준비해온 국제대회가 한순간에 물거품이 되는 순간이었다. 이 대회는 내년 8월 24∼31일 열릴 예정이었다. 대만만 개최권 박탈에 반대표를 던졌고 일본은 기권했다. 차이잉원(蔡英文) 대만 총통은 이날 페이스북에 “중국이 정치적인 힘으로 대회 개최권을 난폭하게 박탈했다. 대만 국민은 이를 결코 받아들일 수 없다”며 반발했다. 라이칭더(賴淸德) 대만 행정원장(총리)은 “EAOC에 정식으로 항의하겠다”고 밝혔다.

　최근 대만에서는 도쿄 올림픽 때 대만이라는 표기로 참가해야 한다는 국민투표 청원운동이 벌어지고 있던 상황이었다. 현재 대만은 올림픽에 ‘차이니즈 타이베이(Chinese Taipei)’라는 이름으로만 참가가 가능하다. 1968년 멕시코 올림픽까지만 해도 대만으로 참가가 가능했고 대만 국기를 들고 대만 국가도 불렀다. 하지만 1970년대부터 중국의 국제사회 영향력이 커지면서 결국 국제올림픽위원회(IOC)는 1981년 대만이 중국의 일부라는 뜻의 ‘차이니즈 타이베이’라는 명칭으로만 올림픽에 참가할 수 있다고 결정했다. 류 위원장이 ‘올림픽 모델에 대한 도전’이라고 언급한 것도 이 때문이다. 2016년 차이 총통 집권 이후 대만 정부가 대만이 중국의 일부라는 ‘하나의 중국’ 원칙 수용을 거부해 중국과 대만의 관계가 최악으로 치닫던 상황에서 이번 일로 또 하나의 악재가 터진 것이다.

　“우리는 대만이라고 부르기 시작한다”는 표어를 내걸고 국민투표 청원운동을 주도하던 대만 육상 국가대표 출신 지정(紀政)은 “대만은 민주국가이고 (국민투표를 청원하는) 시민 권리 행사는 법률이 허락한 것”이라며 “개최권 박탈은 받아들일 수 없다”고 말했다. 지정은 대만이란 이름으로 참가한 1968년 멕시코 올림픽에서 동메달을 땄다.

　대만 문제를 이용해 중국을 압박하려는 미국과 중국 간의 갈등도 고조되고 있다. 중국 외교부는 24일 “대만을 중국과 별개 국가로 표기하고 있는 미국 항공사들에 홈페이지 등의 자료 수정을 촉구하라”며 “25일까지 수정되지 않으면 다음 단계 조치를 취하겠다”고 미국 정부에 최후통첩을 했다. 로이터통신은 25일 아메리칸항공, 델타항공, 하와이안항공이 중국의 요구를 수용해 표기를 수정했다고 보도했다.

# “관료부패가 가짜백신사태 불러” ...분노한 중민심 SNS 타고 확산

“(죄를 지은) 자기가 자기를 조사해?” “반응이 이렇게 굼떠서 어떻게 지도자를 하나.” “탐관오리들은 계속 직을 유지하고 장사꾼은 계속 돈을 번다. 인민은 계속 고통받는다.”

　가짜 영유아용 백신 사건이 중국을 강타한 24일 중국 공산당의 부패 조사기관인 중앙기율검사위원회는 가짜 백신을 제조한 창춘창성(長春長生)바이오테크놀로지 소재지인 지린(吉林)성 기율검사위원회가 이 사건 관련 책임자를 조사한다고 밝혔다. 하지만 이를 보도한 중국 신징(新京)보 웨이보(중국의 트위터 격) 계정에 달린 댓글에 나타난 중국 민심은 싸늘했다. 이 업체가 제조했으나 부적합 판정을 받은 DPT(디프테리아 백일해 파상풍) 백신 25개가 25만 명의 영유아에게 접종됐을 뿐 아니라 충칭(重慶)시에서도 한 업체의 부적합 백일해 백신이 14만여 영유아에게 접종된 사실이 추가로 밝혀졌다.

　중국 누리꾼들은 “관료들의 말을 듣고 싶지 않아. 행동을 보고 싶다” “(부패) 관련 관료가 (조사하는) 너희들보다 직이 높을 것이다” “중앙이 직접 사람을 보내 조사하라! 성 지도자는 부패했다” “사건 발생 후 여러 날이 지났다. 여론을 가리지 못한다. 책임을 물어야 한다” 등 노골적으로 중국 당국에 대한 불신과 적대감을 드러냈다.

　충칭시의 한 시민은 자녀가 문제의 백신을 접종받은 사실을 안 뒤 바로 인터넷에 “가짜 백신 피해자 권리 수호 조직을 만들자”고 올렸다. 얼마 뒤 공안(경찰)이 그의 집에 들이닥쳐 공안국으로 연행됐고 조사를 받은 뒤 풀려났다.

　리커창(李克强) 총리는 물론이고 아프리카 순방 중인 시진핑(習近平) 주석까지 공개적으로 진상 조사를 지시했지만 민심의 분노는 중국 공산당과 정부를 직접 향하고 있는 것이다. 중국 당국이 정부 비판 인터넷 댓글을 대대적으로 삭제하는 동안 소셜미디어가 정부에 대한 분노를 직접 표출하는 핵심 통로로 떠오른 점이 주목된다.

　이런 가운데 중국 국가식품의약품감독관리총국 쉬징허(徐景合) 부국장이 가짜 백신 사건과 관련한 관영 중국중앙(CC)TV의 저녁 메인뉴스 신원롄보(新聞聯報) 인터뷰 때 영국산 명품인 버버리 티셔츠를 입은 것이 부적절했다는 비판도 이어지고 있다. 22일 방영된 이 인터뷰에서 그는 3200위안(약 53만 원)짜리로 추정되는 파란색 티셔츠를 입었다. 중국 누리꾼들은 “위부터 아래까지 심하게 썩었다”는 비판부터 “집에서 가장 싼 옷일 것”이라는 비아냥까지 분노를 쏟아냈다. 한 베이징 시민은 본보에 “월급이 많지 않은 공무원이 버버리를 입고 버젓이 관영매체 인터뷰를 했다는 것 자체가 한심하다”고 지적했다.

# 16일 中-EU 정상, 경제동맹 재편 회담

16일 중국 베이징(北京)에서 열리는 20번째 유럽연합(EU)-중국 정상회담은 역대 어느 때보다 더 끈끈한 분위기를 연출할 것으로 보인다. 이번 정상회담의 핵심 의제는 경제다. 미국과 무역전쟁을 치르며 동병상련의 어려움을 겪고 있는 중국과 EU는 도널드 트럼프 미국 행정부의 ‘고립주의와 보호무역’에 맞서 ‘다자주의와 자유무역’의 공통분모를 강조할 예정이다.

　더 급한 쪽은 중국이다. 미국이 500억 달러 규모의 중국산 제품에 25%의 관세를 부과한 데 이어 2000억 달러 규모의 중국산 제품에 추가 관세 10%를 부여하겠다고 밝혔지만 중국은 이에 반격할 마땅한 카드가 없다. 작년 기준 미국의 대중 수출액은 약 1300억 달러에 불과하기 때문이다.

　중국은 올해가 중-EU 전략적 협력 동반자관계 15주년이 되는 해라는 점을 강조하면서 분위기를 띄우고 있다. 장클로드 융커 EU 집행위원장의 대화 상대인 리커창(李克强) 총리뿐 아니라 시진핑(習近平) 국가주석이 융커 위원장 및 도날트 투스크 EU의회 상임의장과 회견할 것임도 미리 공개했다.

　미중 무역전쟁을 미국 대 중국 구도가 아닌 미국 대 세계 구도로 만들려는 게 중국의 속셈이다. 중국은 이미 지난주 독일이 강력하게 요구해 온 중국 인권운동가 류샤오보(劉曉波)의 부인 류샤(劉霞)를 독일로 보내며 서방에 강력한 구애를 보냈다.

　중국의 이런 태도는 EU에 절호의 기회가 될 수 있다. EU도 미국과 무역전쟁을 하고 있지만 중국에 비하면 아직은 대응할 카드가 많다. 과거 중국의 시장진입 제한, 지식재산권 침해 등에 문제를 제기하며 불만을 나타냈던 EU는 이번 기회를 십분 활용해 중국의 양보를 얻어내려 하고 있다.

　이미 EU는 자동차와 화학 등에서 중국의 경제적 당근을 받았다. 지난주 리 총리는 베를린을 방문해 EU 최대국인 독일의 앙겔라 메르켈 총리와 회담을 갖고 200억 유로의 경제 관련 합의를 했다. 독일 자동차의 중국 생산 공장 허가뿐만 아니라 독일산 자율 주행차 개발에 도움을 주기로 했다. 중국에 수출하는 차도 미국에서 생산하고 있는 독일로서는 중국에 생산공장을 지으면 훨씬 낮은 비용으로 중국 시장에 차를 공급할 수 있게 된다. 또 독일 기업 바스프는 중국 기업과 합자를 하지 않고 지분 100%를 유지한 채 광둥(廣東)성에 100억 달러 규모의 화학 공장을 건설하는 내용의 협약을 체결했다. EU는 “정상회담에서 양측은 전략적 관계 확대를 할 예정이며 무역과 투자 분야가 가장 핵심 이슈가 될 것”이라고 밝혔다.

# 불붙은 무역전쟁... 中 ‘소방수’ 왕치산이 안보인다

미중 갈등을 해결할 ‘해결사’로 여겨졌던 왕치산(王岐山·사진) 중국 국가부주석이 미중 무역전쟁이 본격 발발했는데도 나서지 않아 배경이 주목된다.

　왕 부주석이 미국에 가 협상을 벌일 것이라는 관측도 나왔으나 중국의 여러 소식통은 10일 “현재로서는 왕 부주석이 미국에 갈 가능성은 낮다. 미중 무역문제 관련 중국 측 협상 대표를 류허(劉鶴) 국무원 부총리가 맡고 있는 상황에서 왕 부주석이 나설 이유가 적다”고 말하고 있다.

　올해 3월 전국인민대표대회(전국인대·한국의 국회 격)를 통해 화려하게 복귀한 왕 부주석이 외교·경제 분야 미중 갈등을 조정할 ‘소방대장’으로 기대를 받았다는 점에서 그가 무역전쟁 국면에서 나서지 않는 데는 말하지 못할 사정이 있는 것 아니냐는 관측이 나온다. 왕 부주석은 2009∼2012년 부총리 시절 미중 전략경제대화를 주도하는 등 미국과의 협상 경험이 풍부하고 미국에 상당한 인적 네트워크를 구축한 것으로 알려졌다. 그는 부주석 임명 전 테리 브랜스태드 주중 미국대사와 스티브 배넌 전 백악관 수석전략가 등 핵심 인사를 잇달아 비공개로 만났다. 부주석 임명 이후에도 미국 기업들의 주요 책임자들과 비공개 회담을 해온 것으로 전해졌다.

　하지만 미국의소리(VOA) 중문판 표현처럼 미중 무역전쟁의 화살이 활시위에 있는 일촉즉발 상황에서 왕 부주석은 보이지 않았다. 로이터통신은 8일 ‘소방대장 부주석이 미국의 무역전쟁 불길을 피하고 있다’는 제목의 기사에서 고위 서방 외교관을 인용해 “중국이 왕 부주석을 무역전쟁에 투입하기를 원하지 않는 것 같다”고 전했다.

　앞서 류 부총리가 참여한 5월 워싱턴 2차 미중 무역협상에서 양측은 서로 관세를 부과하지 않기로 합의했다. 당시 류 부총리는 인터뷰에서 “미중은 무역전쟁을 하지 않을 것”이라고 자신 있게 밝혔으나 불과 10여 일 뒤 백악관은 중국에 대한 관세 부과 계획을 발표했다.

　이 때문에 류 부총리는 매우 난처한 입장에 처했다. 류 부총리도 시진핑(習近平) 국가주석의 측근으로 통하지만 왕 부주석은 시 주석의 오른팔로 통하는 최측근 중의 최측근이다. 왕 부주석이 대미 협상에 투입돼 류 부총리처럼 실패하면 이에 대한 비판이 시 주석으로 직접 향할 것을 우려한 중국 당국이 왕 부주석 투입을 꺼리고 있다는 것이다. 한 외교 소식통은 “미중이 언젠가 협상 테이블에 앉겠지만 이번 무역전쟁은 장기전이 될 가능성이 높다”며 “무역전쟁 여파로 중국 경제성장이 둔화되고 경제가 침체돼 국민 생활에 영향을 미치면 중국 당국이 고민스러운 상황에 처할 것”이라고 말했다.

# 中의 반격…美반도체 마이크론 판매금지

(5판용/교열 안봄)　중국이 미국 반도체 기업인 마이크론 제품의 자국 내 판매 금지에 나섰다. 6일 미국과 관세 부과 발효를 앞두고 전격적으로 나온 이번 결정을 두고 무역전쟁의 신호탄이라는 분석이 나온다.

　현지시간 3일 블룸버그 등에 따르면 중국 푸저우(福州) 법원은 지난 2일 마이크론의 중국 내 판매 금지 예비 명령을 내렸다. 이 명령은 마이크론이 생산한 D램, 낸드플래시 관련 제품 등 26개 품목에 적용된다. 미국에 본사를 둔 마이크론은 지난해 매출의 절반 이상을 중국에서 올렸다.

　중국 법원의 이번 명령은 마이크론의 경쟁사인 대만 반도체 기업 UMC 발표를 통해 언론에 알려졌다. 중국 국유기업 푸젠진화와 손잡고 현지에 D램 생산공장을 짓고 있는 UMC는 지난해부터 마이크론과 특허 침해 및 영업 기밀 등을 놓고 법적 다툼을 벌여왔다. 마이크론은 지난해 12월 UMC 등이 D램 반도체 특허 및 영업비밀을 침해했다며 미국 캘리포니아 주 법원에 소송을 제기했고, UMC는 올 1월 마이크론을 상대로 중국 지방법원에 맞소송을 냈다.

　세계 최대 반도체 시장인 중국의 이번 결정으로 미국과 중국의 무역갈등이 IT분야로 본격화 된 것이라는 해석이 나온다. 앞서 미국은 중국 IT업체 화웨이, ZTE, 차이나모바일 등에 제재를 부과했고 미 반도체 업체 퀄컴도 중국의 견제로 네덜란드 반도체 회사 NXP의 인수에 어려움을 겪었다고 블룸버그는 전했다.

# 흔들리는 北제재… 美中 점검회의 중단

유엔 안전보장이사회 대북제재 결의를 중국 정부가 제대로 이행하고 있는지 점검하는 미국과 중국의 합동 실무회의가 올해부터 중단된 것으로 전해졌다.

　도널드 트럼프 미 행정부의 한 관계자는 22일(현지 시간) 본보와의 통화에서 “중국 측 사정으로 올해 들어 최근까지 미중 간 실무회의가 열리지 못하고 있다”고 전했다. 다른 소식통도 “김정은 국무위원장이 연초 신년사에서 평창 올림픽 참가를 발표한 이후 실무회의가 소집되지 않고 있다”고 말했다. 트럼프 대통령이 21일 트위터에 “최근 들어 국경에 구멍이 훨씬 더 많이 뚫리고 더 많은 것들이 흘러들어 가고 있다는 소문들이 돌고 있다”며 중국의 대북제재 이행을 공개적으로 촉구한 것도 미중 실무회의 중단과 관련이 있다는 관측이 나온다.

　대북제재 이행을 집중 점검하는 이 회의는 트럼프 행정부가 유엔 제재에도 중국 국경을 통해 은밀하게 북한과의 거래가 이뤄지면서 제재의 실효성이 떨어진다고 집요하게 문제 제기를 하자 중국 측의 제의로 지난해 초부터 시작됐다. 이후 워싱턴과 베이징을 오가며 2, 3개월에 한 번씩 비정기적으로 회의가 열렸다. 지난해 9월 27일 워싱턴 회의가 마지막이었다.

　유엔 안보리가 대북 수출입을 금지 또는 제한한 정제유와 석탄, 철, 수산물 등에 대해 중국 정부가 구체적인 통계를 제시하고 미국도 위성 관측 등을 통해 확보한 정보를 통해 확인하는 과정을 거치면서 충실한 제재 이행을 뒷받침하는 역할을 해왔다. 북한 대외무역의 95%를 차지하는 중국이 적극적으로 제재에 동참하면서 결국 북한이 대화 무대로 나오게 됐다는 게 미국의 평가였다. 트럼프 대통령도 기회가 될 때마다 시진핑(習近平) 중국 국가주석의 협조를 평가해 왔다. 하지만 한반도 해빙 무드의 영향에 더해 중국 정부가 실무회의에서 빠지면서 다시 대북제재에 구멍이 생기고 있는 게 아니냐는 관측이 나온다.

　문제는 트럼프 행정부가 중국을 압박해왔던 무역카드를 소진해 버린 시점이어서 대북제재를 강화할 카드가 마땅치 않다는 점이다. 미중은 두 차례의 협상 끝에 최근 관세 부과 유예 등에 합의해 논란을 일단락 지었다. 그동안 거의 매달 대북제재 대상을 발표해 왔던 미 재무부도 2월 이후 석 달째 제재 대상을 발표하지 않고 있다.

# 美-中, 관세전쟁 중단… 무역협상 타결

중국이 대중국 무역적자를 줄이기 위해 전방위로 압박해온 도널드 트럼프 미국 행정부에 결국 백기를 들었다. 중국은 미국산 농산물 수입을 늘리고 미국의 지식재산권을 보호하는 무역협상에 합의했다.

　스티븐 므누신 미국 재무장관과 류허(劉鶴) 중국 국무원 부총리가 각각 이끄는 미중 경제·무역대표단은 17, 18일(현지 시간) 워싱턴에서 2차 무역협상을 마무리하고 19일 합의 내용을 발표했다. 양국은 연간 3750억 달러(약 406조 원·지난해 미국 측 통계 기준)에 이르는 대중 무역적자를 절반 수준으로 줄이고, 중국의 지식재산권 침해를 막기 위한 제도적 정비를 하기로 합의했다.

　양국 대표단은 공동성명에서 “중국의 대미 상품수지 흑자를 상당 부분 줄이기 위한 조치를 하자는 데 공감대를 이뤘다”면서 “이를 위해 중국은 미국의 상품·서비스 구매를 늘릴 예정”이라고 밝혔다. 미국의 수출확대 품목으로는 트럼프 대통령의 정치적 기반 지역에서 주로 생산되는 농산물을 비롯해 자동차와 에너지 제품이 명시됐다. 앞서 논의된 품목 중 항공기 반도체 등은 목록에서 빠졌다. 구체적인 무역흑자 축소 목표액은 공동성명에 담기지 않았다. 미국은 연간 2000억 달러 감축을 명기하자고 했지만 중국이 거부한 것으로 알려졌다.

　이번 합의에도 불구하고 중국의 대미 무역 흑자가 큰 폭으로 줄어들기는 쉽지 않을 것이란 지적이 나온다. 소비대국 미국과 값싼 노동력을 기반으로 전 세계의 생산기지 역할을 하는 중국의 경제구조에 차이가 있어 양국의 무역 불균형은 근본적인 해법을 찾기 어렵다는 것이다. 특히 저가 농산물과 에너지 제품의 대중 수출을 늘리는 방식으로는 한계가 있다는 평가가 나온다. 뉴욕타임스는 “콩류인 대두에서 50억 달러, 천연가스 석탄 원유 등에서 90억 달러 정도의 수출 증가가 가능할 것”이라며 “고가의 최첨단 정보기술(IT) 제품과 항공기 및 방위산업 제품을 포함하더라도 트럼프 행정부의 목표치인 연간 2000억 달러의 적자 감축은 비현실적”이라고 분석했다.

　류 부총리는 “이번 무역협상의 최대 성과는 양측이 무역전쟁을 하지 않기로 했고 상호 관세 부과를 중지하기로 합의한 것”이라고 밝혔다. 공동성명에는 포함되지 않았지만 미중이 관세폭탄 전쟁을 끝내기로 했음을 밝힌 것이다. 중국 매체들은 이번 합의가 미국에 백기를 든 것으로 비치는 걸 막기 위해 “무역전쟁을 끝냈다!” “공동 승리”라는 표현 등을 강조했다.

# 中첫 국산 항모 시험항해... 연안 벗어나 원양으로 ‘군사 굴기’

중국의 첫 국산 항공모함이자 두 번째 항모인 산둥(山東)함이 8일 랴오닝(遼寧)성 다롄(大連)에서 시험 항해를 시작한 것으로 알려졌다. 시진핑(習近平) 중국 국가주석의 전용기와 김정은 북한 국무위원장의 전용기가 다롄 공항에서 목격돼 두 지도자가 역사적인 시험 항해를 지켜본 것으로 관측된다. 앞서 랴오닝성 해사국은 4∼11일 보하이(渤海) 해역과 서해 북부 해역에서 군사 임무가 펼쳐진다며 선박 진입을 금지한다고 공고했다

　중국 해군이 랴오닝함에 이어 산둥함까지 확보함에 따라 본토 해안선에서 수천 km 떨어진 곳에서도 군사 작전이 가능해졌고 해외에서 미국에 맞서 자국의 전략 이익을 도모할 능력을 갖게 됐다는 평가가 나온다. 중국의 2개 항모 보유는 근해 연안 방어에 치중했던 중국이 원양 해군으로 나가는 데 중요한 이정표가 될 것으로 보인다. 중국은 항모를 6척까지 늘릴 계획이다.

○　베일 벗은 산둥함의 전투능력

　중국은 우크라이나에서 들여온 구소련 항모를 개조해 2012년 5만5000t급 랴오닝함을 진수했다. 이를 통해 축적한 기술력을 바탕으로 만재배수량이 더 커진 산둥함(7만 t)을 만들어냈다. 모듈식 조립 방식으로 건조된 산둥함은 2013년 11월부터 건조에 착수해 지난해 4월 진수됐다. 시험 항해를 마친 뒤 내년에 실전 배치될 예정이다. 중국의 항모 이름은 바다에 접한 성의 이름을 순서대로 채용하며 산둥함 이후 진수되는 항모의 이름은 장쑤(江蘇)가 될 것으로 보인다.

　디젤 추진 항모인 산둥함은 길이 315m, 너비 75m에 최대 속도 31노트를 낼 수 있다. 스키점프 방식으로 이륙하는 젠(殲)-15 함재기 40대를 탑재 가능하다. 24대의 함재기를 탑재하는 랴오닝함에 비해 길이가 10m 정도 늘어났지만, 함재기는 16대나 더 실을 수 있어 중국이 그동안 상당한 능력의 최적화된 항모 설계 기술을 연마했음을 보여준다. 이륙 램프 경사도는 12도로 최고 14도인 랴오닝함보다 낮아졌다. 랴오닝함에 비해 함재기 이륙 거리 감축, 연료 절약, 무기 적재량 증가, 항모 구조 강화 등에서 많은 개선이 이뤄진 것으로 알려졌다.

　산둥함에는 대형 안테나 4개와 주변을 360도 감지해 해상 또는 공중 목표물 수십 개를 포착할 수 있는 S밴드 레이더가 탑재됐으며 수십 기의 중국산 단거리, 중거리 미사일이 실려 있다.

　다만 함재기를 발진시킬 때 미국 항모가 사용하는 전자식 사출 방식이 아닌 증기 사출 방식을 채택하고 있다. 증기 사출 방식이나 스키점프 이륙 방식은 전자식에 비해 함재기의 이륙 거리가 많이 필요하다. 또 비행기의 무게를 줄여야 하기 때문에 많은 무기를 탑재하기 어려우며, 결과적으로 작전 능력이 제약된다. 핵추진 항모가 아니기 때문에 며칠에 한 번씩 급유를 받아야 한다는 점도 약점으로 꼽힌다. 대양 작전을 벌이려면 방어능력이 취약한 여러 대의 대형 급유선을 함께 거느리고 움직여야 하기 때문이다.

○　원양엔 나갔어도, 미국 넘긴 역부족

　중국 해군은 그간 연해 방어 위주의 ‘황수(黃水·yellow water) 해군’에서 영해와 영토를 수호하는 ‘녹수(綠水·green water) 해군’에 이어 에너지 수송 노선을 수호하는 ‘남수(藍水·blue water) 해군’을 추구했다. 따라서 항모 전단들은 주로 인도양, 서태평양 중심으로 활동하면서 ‘일대일로(一帶一路·21세기 육상과 해상 실크로드 프로젝트)’ 가운데 ‘일로’ 구상을 지원하는 역할을 수행할 것으로 예상된다. 그러나 경우에 따라 한국과 일본의 전력을 견제하는 역할도 맡게 될 것으로 보여 동아시아 해양질서에 큰 변화가 생길 수밖에 없다.

　중국이 향후 3, 4척의 항모를 더 확보하고 핵 항모까지 손에 넣는다면 명실상부한 대양 해군 능력을 갖추게 된다. 그러면 각종 국제 현안에 대한 중국의 개입도 늘어나게 될 것으로 보인다.

　중국 해군이 비약적인 성장에도 불구하고 아시아·태평양 지역 내에만 4개의 항모전단을 운용하고 있으며, 전 세계적으로 10개의 항모전단을 보유한 미 해군력에 비해선 양적, 질적 수준 차이가 크다. 미국의 군함 총톤수는 950만 t에 이르지만 중국은 미국의 5%에도 못 미치는 군함 총톤수 50만 t 미만의 해군력을 보유하고 있다. 또 중국이 운용하는 젠-15 함재기의 전투 능력은 미 해군의 최신 함재기 F-35에 미치지 못한다고 군사전문가들은 평가한다. 여기에 미국이 20만 명 이상의 해병대 병력을 보유하고 있지만, 중국은 2만 명에서 10만 명으로 확대하기 위해 노력하는 중이다.

# 北달려간 왕이, 남북미중 회담 요구할듯

중국 왕이(王毅) 국무위원 겸 외교부장이 2일 오전 평양에 도착했다. 3일까지 평양에 머무는 왕 위원은 리용호 북한 외무상과 회담한 뒤 김정은 북한 국무위원장을 면담할 것으로 알려졌다.

　중국과 북한은 2일 오후까지 왕 위원의 평양 내 행보와 관련한 소식을 전하지 않았다. 왕 위원은 △북-미 정상회담 전후로 예상되는 시진핑(習近平) 중국 국가주석의 방북 일정을 확정하고 △남북 정상이 판문점선언에서 천명한 평화체제 협상은 중국이 참여한 남북미중 4자 회담이 돼야 한다는 점을 요구하는 등 북-미 정상회담을 앞두고 중국의 입장을 북한에 구체적으로 전할 것으로 관측된다. 남북 정상회담 결과에 대한 설명도 들을 것으로 알려졌다.

　남북 정상은 지난달 27일 판문점선언에서 평화체제 구축을 위해 “남북미 3자 또는 남북미중 4자 회담을 추진한다”고 밝혔다. 미국의소리(VOA) 중문판은 “왕 위원이 중국 ‘패싱’(배제)을 막기 위해 급하게 방북했다”는 분석을 내놓았다. 주한미군 철수 등 중국의 한반도 이해관계와 직결되는 평화협정 협상에서 중국이 배제돼서는 안 된다는 입장을 전하기 위한 적극적 개입 외교의 일환이라는 것이다.

　중국 외교부장의 방북은 2007년 7월 이후 11년 만이다. 왕 위원과 북한 사이에서 시 주석의 방북 시점이 어떻게 조율될지도 주목된다. 베이징 외교 당국자는 “(시 주석의 방북 시점이) 상식적으로 북-미 정상회담 전은 아닐 것”이라고 말했다. 시 주석이 방북에 이어 한국을 방문할 수 있다는 관측도 나온다.

# 中매체 “6·25 같은 의지로 美와 무역전쟁”

중국 공산당 기관지 런민(人民)일보의 자매지 환추(環球)시보는 8일 사설에서 “항미원조(抗美援朝·중국이 6·25전쟁 때 미국과 맞서 북한을 돕기 위해 참전한 것)의 의지로 결연히 도널드 트럼프 미 정부의 무역 공격을 쳐부술 것”이라고 주장했다. 이어 “항미원조는 미국 군대가 압록강변에 도달해 일어났다. 미국이 일으킨 무역전쟁은 중국의 핵심 이익에 충격을 줬다”며 “물러설 수 없는 위기감을 갖고 국가 근본 이익을 수호하기 위해 미국에 양보할 수 없다는 결연한 의지로 중국 사회가 응집하고 있다”고 강조했다. 환추시보는 또 “중국은 무역전쟁의 무기와 탄약이 매우 충분하다. 우리는 (전쟁에선) 희생을 치러야 함을 안다”고 주장했다.

　한편 미 해군 항공모함 루스벨트함(CVN-71)을 기함으로 하면서 이지스 순양함 벙커힐(CV-17), 미사일 구축함 샘프슨(DDG-102) 등으로 전단을 구성한 제9항모강습단이 6일부터 남중국해 남부 해역에서 싱가포르 해군과 연합 군사훈련을 벌이고 있다. 중국의 유일한 실전 배치 항공모함인 랴오닝(遼寧)함은 5일부터 남중국해 하이난(海南) 해역에서 40여 척의 군함과 잠수함, 훙(轟)-6K 전략 폭격기 12대 등과 함께 대규모 훈련을 진행하고 있다. 루스벨트함이 중국이 영유권을 주장하는 해역에 접근해 ‘항행의 자유’ 작전을 펼칠 경우 미중 항공모함 간 첫 대치가 발생할 수 있다는 우려가 나온다.

# 中, 작심 반격…미국산 콩에 보복관세

미국이 첨단 산업을 정조준해 1300개 중국산 수입품 품목에 25%의 ‘관세폭탄’을 떨어뜨렸다. 미국산 돼지고기와 농산물 등에 대한 중국의 보복관세에도 아랑곳하지 않고 예고한 대로 강펀치를 날렸다.

　미국무역대표부(USTR)는 3일(현지 시간) 25% 관세를 부과할 500억 달러(약 52조8600억 원)어치 중국산 수입품 1300개 품목을 발표했다. USTR가 이날 공개한 58페이지 분량의 관세 부과 목록에는 반도체, 통신장비, 리튬배터리 등 첨단 기술 제품부터 중장비, 철강, 금속 및 알루미늄 제품, 발전기, 오토바이, 항공기 부품, 무기, 의료기기 등이 골고루 포함됐다. 특히 고율 관세 부과 제품에는 중국이 내놓은 첨단산업 육성 전략인 ‘중국 제조(메이드 인 차이나) 2025’ 품목이 상당수 포함됐다.

　USTR는 이날 “중국은 미국 회사의 민감한 상업 정보와 무역 기밀에 접근하기 위한 컴퓨터 네트워크 무단 침입 및 기술 절취를 지원하고 실행했다”며 “(관세 부과 조치는) 미국 경제에 미친 피해와 중국의 해로운 정책, 절차, 행위를 없애기 위한 두 가지 측면에서 적절한 수준”이라고 밝혔다. 도널드 트럼프 미국 대통령은 중국에 대해 “무역적자의 리더”라고 비판했다.

　중국은 USTR의 관세 부과 품목 발표에 즉각 반발했다. 중국 상무부는 USTR의 발표 후 1시간여 만에 대변인 명의의 담화를 내고 “중국은 결연히 반대하고 조만간 법에 따라 미국산 상품에 대해 동등한 강도와 규모로 대등한 조치를 하겠다”고 경고했다. 중국은 미국의 철강과 알루미늄 보복 관세에 맞서 2일부터 돼지고기 등 30억 달러(약 3조1700억 원)어치 미국산 수입품에 고율 관세를 부과했다.

# 北 최고위급, ‘中의 실리콘밸리’ 중관춘 방문뒤 귀국

김정은 북한 노동당 위원장이거나 김 위원장의 여동생 김여정임이 확실해 보이는 북한 최고위 인사는 27일 중국 당국의 정상급 경호 의전을 받으며 베이징 서남부 곳곳을 누볐다. 특히 아버지 김정일이 방중 때마다 거의 빠짐없이 방문했던 ‘베이징의 실리콘밸리’ 중관춘(中關村)을 방문해 주목된다.　

　북한 최고위 인사가 머문 국빈 숙소 댜오위타이(釣魚臺) 정문(동문)은 이날도 무장경찰이 대거 배치돼 기자들의 취재를 막으며 삼엄한 분위기를 연출했다. 베이징의 한 소식통은 “북한 최고위 인사가 (할아버지) 김일성이 중국을 방문할 때마다 머물렀던 댜오위타이 18호각에서 하룻밤을 묵은 것으로 안다”고 전했다.

　이날 오전 9시 반경 최고위 인사가 탄 차량 등 2대가 중국 측 공안 사이드카 10여 대의 호위를 받으며 댜오위타이 정문을 빠져나왔다. 이 차량은 베이징의 실리콘밸리라 불리는 서부의 중관춘으로 향했다. 특히 이 인사는 중관춘 최대의 컴퓨터 전자기기 상가인 하이룽(海龍)빌딩을 방문한 것으로 알려졌다. 하이룽빌딩 내의 컴퓨터, 전자기기 상점들이 한국 서울 용산전자상가의 몇 배에 달하는 것으로 알려져 있다.

　김정일은 첫 중국 방문인 2000년 5월, 2010년 5월, 마지막 방문인 2011년 5월에 중관춘을 찾았다. 방중한 북한 최고위 인사가 김정일 방중 전통을 따라 중관춘을 방문해 컴퓨터 전자기기 상가를 둘러본 것은 이 인사가 과학기술에 관심이 많고 이 분야의 국산화 발전에 주력하고 있는 것과 관련이 있다는 관측이 나왔다.

　이날 최고위 인사의 방문지마다 삼엄한 통제로 통행이 금지되거나 심지어 도로를 쳐다보는 행위까지 금지해 중국 국민과 해외 여행객들이 불편을 호소하거나 불만을 표출했다.

　댜오위타이의 모든 출입구에는 공안이 배치됐으며 인근 200m 구간이 통제됐다. 취재진의 접근도 막았다. 이날 오전 중관춘 일대에도 교통 통제가 되면서 웨이보(중국의 트위터 격)에는 불편을 호소하는 시민들의 불만과 교통 통제 사진, 최고위 인사의 차량 행렬 사진 동영상 등이 잇따라 올라왔다. 한 시민은 “진싼팡(金三반·김씨네 3대 뚱보)이 왔는가 보다”라고 비꼬기도 했다. 진싼팡은 중국 누리꾼들이 김정은을 얕잡아 부르는 대표적 표현이다.

　이날 오전 11시경부터 베이징 남부에 있는 베이징기차역의 VIP(귀빈) 출입구가 통제되기 시작했고 11시 20분경부터는 베이징기차역 인근 도로 일부도 통제됐다. 낮 12시 반경부터 북한에서 온 ‘1호열차’가 대기했다. 오전 11시부터 자금성과 톈안먼(天安門)광장을 가르는 베이징 중심가 창안제(長安街) 일부와 톈안먼광장 서쪽의 인민대회당 서쪽 도로가 통제와 해제를 반복했다. 이 과정에서 공안들은 지나는 시민과 관광객들에게 인도에 서 있지 못하게 하고 통제된 도로를 쳐다보는 행위에 대해서조차 제지를 했다.

　이날 낮 12시 반경부터 베이징 남부 톈탄(天壇)공원 주변 도로가 통제됐다는 얘기도 들렸다. 이 최고위 인사는 중관춘을 출발해 인민대회당 북쪽 창안제를 거쳐 베이징기차역에 이르는 동선을 선택한 것으로 관측된다. 도중에 모처에서 점심식사를 한 것으로 알려졌다.

　한편 북-중 접경 지역인 랴오닝(遼寧)성 단둥(丹東)에서 압록강철교를 내려다볼 수 있는 중롄(中聯)호텔은 27일까지 압록강변을 바라보는 강변쪽 객실 예약을 중단했다.

# 중국, 北-美 대화 지원하고 ‘한반도 평화’ 후원자 돼야

중국 시진핑 국가주석은 어제 정의용 청와대 국가안보실장을 만나 남북, 북-미 정상회담 합의에 환영과 지지의 뜻을 나타내며 “성과를 내기 바란다”고 말했다. 정 실장은 양제츠 외교담당 국무위원, 왕이 외교부장과도 각각 면담과 함께 오찬과 만찬을 함께했다. 중국의 큰 연례 정치행사인 양회(兩會·전국인민대표대회와 전국인민정치협상회의) 기간에 시 주석을 비롯한 최고위 외교라인이 일제히 시간을 낸 것은 이례적이다. 한반도 정세의 급변에 대한 비상한 관심의 반영이자 ‘차이나 패싱(중국 배제)’ 우려가 작용한 것으로 볼 수 있다.

　중국은 남북, 북-미 정상회담에 환영과 지지를 표명하면서도 속내는 복잡하고 불편할 것이다. 그동안 북-미 간 대화를 주선하고 북핵 6자회담 의장국을 맡는 등 적극적 중재 역할을 맡았던 중국이다. 하지만 이번엔 북-미가 중국을 건너뛰고 한국의 중재로 역사적인 첫 정상회담을 열기로 했다. 더욱이 김정은 집권 이후 북-중 간에는 정상회담도 없었다. 이른바 ‘조중(朝中)혈맹’은 과거지사가 됐다지만 중국이 북-미 직거래를 한가롭게 팔짱 끼고 지켜볼 수만은 없는 처지다.

　특히 중국이 줄곧 북핵 해법으로 주장해온 북한의 핵·미사일 도발과 한미 연합군사훈련 동시 중단이라는 쌍중단(雙中斷)도 무색해졌다. 중국은 올림픽 기간의 연합훈련 연기가 사실상 중단 효과를 낸 것이라고 하지만, 북한은 한미 연합훈련도 양해한다며 중단 요구 없이 핵·미사일 실험 중단을 약속했다. 오히려 북한이 미국과의 직접 대화에 나선 것은 중국의 대북제재 협조였다. 중국이 대북제재를 보다 엄격하게 시행하자 북한은 중국 대신 미국을 향해 손짓하는 상황으로 전개됐다. 앞으로 북-미 관계가 급진전되면 미국과의 경쟁에서 북한을 우호적 완충지대로 두겠다는 중국의 대북 전략은 전면 수정이 불가피할 수도 있다.

　문재인 대통령도 어제 “우리가 이루려는 것은 지금까지 세계가 성공하지 못한 대전환의 길”이라며 “우리가 성공해낸다면 세계사적으로 극적인 변화가 만들어질 것이며 대한민국이 주역 될 것”이라고 했다. 북-미 관계의 변화는 분명 한반도는 물론 동북아시아 질서의 근본적 개편을 불러올 수 있다. 급속한 정세 변화에 중국도 경계심을 가질 수 있다. 하지만 중국의 역할을 갑자기 미국이 대신할 수는 없다. 북한이 거리를 두면서도 결국 생존을 의지해온 나라가 중국이다.

　지금까지 과정은 한반도 비핵화와 평화·안정의 수호, 대화·협상을 통한 해결이라는, 중국이 줄곧 내세운 북핵 3원칙의 실현 과정이다. 마침 중국은 국가주석의 3연임 금지 조항을 삭제하는 개헌안을 통과시켜 시 주석에게 막강한 지위와 권한을 부여했다. 앞으로 중국이 본격화할 ‘대국외교’의 첫 시험대는 북한 비핵화를 통한 새로운 동북아 평화질서 구축이 될 것이다. 중국이 북한의 후견국가를 자처하던 시절은 지났다. 동북아 지도국가로서 새로운 질서를 짜는 데 적극적인 역할을 해야 한다.

# 트럼프-김정은 5월 정상회담에..주변국은

미국과 북한이 최대한 빨리 대화해야 한다고 강조해온 중국 외교부는 9일 정례 브리핑에서 도널드 트럼프 미국 대통령이 5월 전 김정은 북한 노동당위원장과의 회담 의사를 밝힌 것에 대해 환영 의사를 나타냈다. 중국 관영 매체들은 이날 북미 정상회담 성사를 속보로 전하면서 놀라움을 감추지 못했다.

　평소 자극적인 표현이 많지 않은 중국 공산당 기관지 런민(人民)일보의 웹사이트 런민왕(網)도 ‘대사건! 트럼프가 5월 전 김정은과 회담에 동의’라는 제목으로 속보를 내보냈다. 런민왕은 다른 기사에서 “북핵 문제의 외교적 해결이 멀고 힘들지만 협상이 전쟁 발생의 우려를 없앨 수 있다”고 지적했다. 중국 정부 입장을 공식 대변하는 관영 신화(新華)통신도 ‘중대변화! 트럼프가 김정은과 5월 전 만난다’는 제목의 속보를 전했다. 신화통신은 다른 기사에서 “트럼프 대통령이 ‘한반도 문제에서 중대한 진전을 이루고 있다고 말했다’”며 “이와 동시에 미국은 합의에 이를 때까지 대북 제재를 계속할 것이라고 밝혔다”고 전했다.

　런민일보 자매지 환추시보(環球) 웹사이트 환추왕도 김정은과 회담 의사를 표명한 트럼프 대통령의 발언을 전한 정의용 대통령국가안보실장의 기자회견을 ‘중대성명’ ‘깜짝뉴스’라고 표현했다. 환추왕은 “너무 갑작스러운 일이다. 북한과 미국이 손을 잡고 기습했다”고 보도했다.

　외교 소식통들에 따르면 중국은 북미대화를 환영하면서도 향후 비핵화 협상에서 중국이 소외되는 일명 ‘차이나 패싱’이 불거질 것을 우려하는 분위기다. 중국이 대북 제재로 북중 관계가 악화되는 등의 대가를 치렀음에도 정작 대화 과정에서는 중국이 배제되는 것 아니냐는 것이다. 이 때문에 중국이 남북, 북미 정상회담 상황을 지켜보면서 북중 관계 개선에 나설 것이라는 전망이 나온다.

　소외론을 의식한 듯 중국 외교부는 “중국이 한반도 문제에서 적극적이고 건설적인 역할을 했고 국제사회의 인정을 받았다”는 점을 재차 강조하고 있다. 왕이(王毅) 중국 외교부장은 8일 기자회견에서 “중국이 주장한 쌍중단(북한 핵·미사일 실험과 한미 연합훈련 중단)이 정확한 처방이었음을 보여준다”며 “(역시 중국이 주장한) 쌍궤병행(비핵화와 평화협정 동시 추진) 방향에 따라 비핵화 과정에서 북한의 합리적인 안보 우려를 해결해야 한다”고 강조했다. 지금의 대화 국면이 중국이 주장해온 해결 방향에 따른 것이라는 주장으로 중국 소외론을 불식시키려는 의도로 해서된다.

# 中 국방비 8% 늘려 사상 최대… 美와 패권경쟁 가속

지난해 10월 19차 중국 공산당 전국대표대회(당 대회)에서 은퇴한 뒤 대외에 모습을 드러내지 않았던 왕치산(王岐山) 전 중앙기율검사위원회 서기가 5일 처음 공식석상을 통해 언론에 모습을 드러냈다. 시진핑(習近平) 국가주석의 오른팔로 국가부주석이 유력한 그는 이날 베이징(北京) 인민대회당에서 열린 전국인민대표대회(전국인대·한국의 국회 격) 개막식에서 시 주석과 리커창(李克强) 총리 등 7명의 상무위원(최고지도부)에 이어 모습을 드러냈다.

　리 총리가 지난 5년 평가와 2018년 정부 계획을 발표하는 업무보고를 읽어 내려가는 과정에서 실질적인 2인자가 리 총리가 아니라 왕 전 서기임을 보여주는 상징적인 장면이 포착됐다. 리 총리의 업무보고가 시작되자 시 주석을 제외하고 상무위원은 물론 정치국 위원, 중앙위원, 2970명의 전국인대 대표 모두 업무보고 책자를 펴들고 보고를 따라 읽어 내려갔다. 오로지 시 주석과 왕 전 서기만이 업무보고 책자에 손도 대지 않았고, 시선을 두지도 않았다. 시 주석은 리 총리의 업무보고가 시작된 지 40여 분 뒤에 책자를 펴들었으나 자신이 보고 싶은 곳만 넘겨봤다. ‘시왕’(習王·시진핑과 왕치산) 체제의 출범을 예고한 장면이라는 평가가 나온다.

　리 총리는 이날 1시간 50분의 업무보고에서 “시진핑 총서기(시 주석의 당 직책) 핵심적 지위를 결연히 수호해야 한다”고 말하는 등 13번이나 시 주석의 이름을 거론하며 시 주석에 대한 찬양을 이어갔다. 업무보고에 이어 시 주석의 임기 제한 폐지를 담은 헌법 수정안 초안이 상정됐다. 심의를 요청하며 개헌 이유를 설명하는 절차가 50여 분 진행됐으나 관영 중국중앙(CC)TV는 리 총리 보고만 생중계하고 개헌안 상정 과정은 중계하지 않았다. 여론의 반발 등 민감성을 고려한 것으로 보인다.

　하지만 인민대회당 현장은 임기 제한 철폐 찬양으로 가득 찼다. 개헌안 상정 때 나온 유일한 박수는 ‘2번을 초과해 연임하지 못하다’는 헌법 규정을 삭제한다고 소개할 때 나왔다.

　이날 전국인대에서는 올해 경제성장률 목표치로 지난해와 같은 6.5%가 제시됐다. 또한 지난해에 비해 국방예산이 8.1% 증가한 국가예산안이 보고됐다. 이로써 올해 중국의 국방예산은 역대 최대인 1조1069억 위안(약 189조1249억 원)에 달한다. 2016년 7.6%, 지난해 7%로 줄어들던 증가율이 다시 크게 늘어난 것이다.

　2016년 이전의 두 자릿수 증가율 회복은 아니지만 지난해 19차 당 대회에서 시 주석이 2050년까지 세계 1위 군사 강국이 되겠다는 강군몽(强軍夢)을 천명한 데 따라 중국이 미국과 군사패권 경쟁을 본격화하려는 것으로 풀이된다.

　중국은 최근 국가 안보가 위협에 처했다는 이유를 내세우면서 핵 추진 항공모함 건조 등 각종 군비 확장을 시도하고 있다.

　한편 리 총리는 미중 무역 전쟁을 염두에 둔 듯 미국을 겨냥해 “중국은 평등한 협상을 통해 무역 분쟁을 해결할 것을 주장한다. 보호무역주의를 반대하고 합법적인 권익을 결연히 수호해야 한다”고 강조했다.

# 中 지식인들 “마오시대로 돌아가나”...시진핑 장기집권 반발

중국 공산당이 25일 국가주석직의 2연임 임기(최장 10년) 제한을 없애는 헌법 개정안을 제안한 데 대한 중국 내 여론의 역풍이 확산되고 있다. ‘시진핑(習近平) 국가주석이 종신독재 집권의 마오쩌둥(毛澤東) 시대로 돌아가려는 것이냐’는 중국인들의 우려가 증폭되고 있기 때문이다.

　중국의 저명한 여성 사회학자인 리인허(李銀河) 중국 사회과학원 교수는 블로그에 “종신제 회복은 역사의 후퇴다. 중국을 마오(쩌둥) 시대로 돌아가게 만들 것”이라고 지적했다. 공산주의청년단(공청단) 기관지 중국청년보 산하 잡지 빙뎬(氷点)의 편집자였던 리다퉁(李大同)도 다음 달 개막하는 전국인민대표대회(한국의 국회 격)에 참석하는 천지닝(陳吉寧) 베이징(北京) 시장 등 베이징 인민대표 55명에게 공개서한을 보내 “개헌안에 반대표를 던질 것”을 호소했다. 중국 여성 기업가 왕잉(王瑛)은 “임기제 삭제는 배반이자 시대 흐름에 역행하는 것”이라며 “내게 침묵을 요구해도 결코 침묵하지 않을 것”이라는 성명을 발표했다. 미국에 망명한 톈안먼(天安門) 민주화운동 당시 학생지도자였던 왕단(王丹)은 100여 명의 중국 학자들과 함께 발표한 성명에서 “시진핑이 황제의 야심을 지니고 있음이 드러났다”고 말했다.

　일본 아사하신문은 27일 시 주석이 지난해 10월 19차 전국대표대회(당 대회) 직후 상하이(上海)에서 당 원로인 장쩌민(江澤民) 전 주석에게 임기 제한 철폐 의사를 밝혔는데 “절대 안 된다”는 장 전 주석의 강한 반발에 부딪혔다고 전했다.

　웨이보(중국판 트위터)에는 ‘나는 동의하지 않는다’ ‘이민’ ‘비행기에 탑승한다(登機)’ 등이 포함된 글까지 검열 대상이 됐다. 임기 제한 삭제안 발표 이후 이민 검색어 수치가 급등했고, ‘비행기에 탑승하다’의 중국어 발음이 ‘(황제) 등극’과 같기 때문이다. 종신제, 등극, 장기집권, 종신집권, 개헌반대 등의 단어는 검색조차 안 되고 있다. 영국 파이낸셜타임스(FT)는 중국 대표 포털 바이두(百度) 뉴스 직원을 익명으로 인용해 “최소 13개 인터넷 뉴스 기업이 당국으로부터 개헌 지지 기사를 상위에 올리라는 지시를 받았다”고 보도했다.

# “中, 남중국해 인공섬 ‘軍지휘센터’로 활용”

중국이 동남아시아 국가들과 영유권 분쟁을 겪고 있는 남중국해에 건설한 인공섬을 주변 군사시설을 지휘하는 센터로 이용하려 한다는 주장이 나왔다. 남중국해는 중국이 영유권을 주장하는 섬의 12해리 안에 미국이 군함을 진입시키는 ‘항행의 자유’ 작전을 지속하면서 군사적 긴장이 높아지고 있는 지역이다.

　18일 홍콩 사우스차이나모닝포스트(SCMP)에 따르면 미국 전략국제문제연구소(CSIS)는 항공사진을 통해 남중국해 스프래틀리 제도(중국명 난사·南沙군도)의 피어리크로스 암초(융수자오·永暑礁) 북서쪽에 중국이 최근 송신탑과 고주파 레이더 설비 등 통신장비를 집중 배치한 사실을 확인했다. 이 암초 10만 m² 부지에 길이 3000m 활주로뿐 아니라 폭격기와 공중급유기, 수송기를 위한 격납고도 설치됐다. 미국은 중국이 피어리크로스 암초뿐 아니라 스프래틀리 제도의 수비 암초(주비자오·渚碧礁), 미스치프 암초(메이지자오·美濟礁) 등 암초 7곳을 군사시설이 배치된 인공섬으로 조성하고 있다고 보고 있다. SCMP는 중화권 군사 전문가들을 인용해 “피어리크로스 시설이 중국이 조성한 군사시설 간 통신을 중개하는 기지로 사용될 수 있다”고 관측했다.

　이에 따라 미중 간 신경전이 거세지고 있다. 호주 주재 미국대사로 내정된 해리 해리스 미 태평양사령부 사령관은 14일 미 하원 군사위원회 청문회에서 스프래틀리 제도의 군사 기지화를 우려하면서 “중국이 인공섬에 고성능 군사 방어 장비를 갖춰 영유권을 주장할 것”이라고 지적했다.

# 中, 러시아판 사드배치… 美에 맞대응

중국이 최근 ‘러시아판 사드’로 불리는 지대공 미사일 S-400을 배치해 배경에 눈길이 쏠린다. 배치 장소는 알려지지 않았지만 산둥(山東)반도에 배치할 경우 한반도가 레이더 탐지 범위에 포함돼 한반도 유사시 한미 군의 움직임을 견제하기 위한 포석이라는 관측이 나왔다.

　19일 러시아 타스통신과 홍콩 밍(明)보에 따르면 러시아는 최근 중국에 미사일 통제소와 레이더 기지, 연료 공급 설비 등 S-400 지대공 미사일 시스템을 제공했다. 중국은 2014년 러시아로부터 S-400 3개 포대를 도입하기로 계약했고 이번에 첫 포대가 배치된 것이다. 중국은 2019년까지 도입을 마무리할 계획인 것으로 알려졌다.

　S-400은 사거리 400km, 최고 비행고도 185km에 이른다. 레이더는 700km 이내의 300개 표적을 추적할 수 있다. 여러 고도와 사거리의 각종 전투기 및 미사일을 동시에 격추할 수 있다. 전자기가 교란되는 환경에서도 운용이 가능하다. S-400은 F-35 스텔스기 등 미국의 첨단 전투기 격추 능력이 있다는 평가를 받아 왔다. 사거리 200km, 최대 고도 150km인 사드(THAAD·고고도미사일방어체계)보다 위협적이라는 분석도 있다.

　밍보는 S-400이 푸젠(福建)성과 같은 중국 남부 연안에 배치되면 대만 전역을 사거리에 둘 수 있어 대만 공군을 공격할 수 있다고 지적했다. 최근 중국에서 대만 무력통일론이 나오는 상황에 주목한 것이다.

　하지만 중국은 미국의 첨단 전투기 및 스텔스 순항미사일에 대응하기 위해 S-400 도입을 추진해 왔다는 관측도 나온다. 러시아도 블라디보스토크 등 북한 인접 극동지역에 S-400을 실전 배치했다.

# 中개혁개방 1번지 선전, 특구 장벽 허물었다

중국의 개혁개방을 상징하는 광둥(廣東)성 선전(深(수,천))시의 중국 제1호 경제특구와 나머지 본토 지역을 분할해 인적·물적 이동을 통제해온 장벽이 설치 36년 만에 완전히 역사 속으로 사라졌다. 더 이상 장벽이 필요 없을 정도로 중국 경제가 성장했음을 대내외에 알리는 상징적인 사건으로 평가된다.

　16일 중국 매체들에 따르면 국무원은 선전시 경제특구에 존재해 왔던 ‘선전경제특구관리선(제2관문선)’을 없애기로 공식 결정했다. “시내의 물리적 경계를 없애 해당 지역의 통합을 촉진할 수 있도록 해달라”는 광둥성 정부의 요청을 승인하는 형식이었다.

　선전시 남쪽의 홍콩과 경계선을 면한 일부 지역(327km²)은 덩샤오핑(鄧小平)이 1978년 개혁개방을 선언한 지 2년 뒤인 1980년 중국의 첫 경제특구로 지정됐다. 인구 3만 명의 작은 어촌 마을이었던 선전은 지금 세계적 정보기술(IT) 기업이 몰려 있는 중국의 실리콘밸리로 발돋움했다.

　중국은 선전에 경제특구를 지정하면서 특구 내 외국인의 자유로운 투자 등 경제 활동, 공장 설립, 면세 등을 보장했다. 하지만 특구 밖 중국인은 허가가 있어야 출입이 가능하도록 했다. 이를 위해 1982년 특구 주위에 136km에 달하는 철조망을 쳤다. 무장경찰이 지키는 초소 163곳과 검문소 10곳이 설치됐다. 경제특구 남쪽은 1997년에야 반환된 홍콩이었기 때문에 더더욱 통제가 심했다.

　사람들은 홍콩과 선전의 경계선을 ‘1선 관문’, 선전특구와 내륙의 경계선을 ‘2선 관문’이라 불렀다. 2선 관문의 존재는 선전의 중국 내 독특한 위상을 웅변하는 동시에 중국이 외부에 완전히 문을 연 것은 아니라는 점도 보여줬다. 2선 관문은 급격하게 서구 경제와 문화에 영향을 받아 중국 사회주의가 위기에 빠지는 일을 막는 역할을 했다.

　경제의 급속한 발전으로 2010년 선전경제특구는 선전시 전체로 확대됐다. 선전시 전역에 지하철이 다니면서 장벽이 유명무실해졌다. 오히려 장벽과 검문소 운영에 매년 수천만 위안이 들어갔고 교통 등 도시 발전의 장애물이 됐다. 시민들이 오갈 때 검문을 거쳐야 한다는 점도 불편을 초래했다. 2013년부터 장벽과 검문소 순찰도로 등에 대한 철거가 시작돼 현재도 진행되고 있지만 공식적으로 중국과 선전특구를 분할했던 이 경계선은 계속 존재해 왔다. 중국 매체들은 “이번 조치로 선전이 다시 한번 개혁개방의 최전선에 섰다”며 기대감을 나타냈다. 다만 국무원은 이번 조치 결정문에서 “선전과 홍콩 마카오 간 경계에 있는 1선 관문에 대한 관리 통제를 강화하라”고 지시했다.

# 美-中무역전쟁 일촉즉발

중국의 최대 전자상거래 업체 알리바바의 대표 쇼핑몰 타오바오(淘寶)가 또다시 미국 정부의 악덕시장 블랙리스트에 올랐다. 미국과 중국이 무역 금융 분야에서 티격태격하는 가운데 중국이 지난해 사상 최대 규모의 대미 무역흑자를 기록하면서 미중 무역전쟁이 폭풍전야라는 관측도 나왔다.

　14일 BBC 중문판에 따르면 미국 무역대표부(USTR)는 타오바오가 지식재산권 침해 상품으로 가득 차 있다며 2016년에 이어 지난해에도 악덕시장 명단에 포함시켰다. USTR는 미국의 지식재산권을 침해하고 미국 노동자에게 심각한 피해를 입히는 온라인 시장 25개와 오프라인 시장 18개를 지난해 악덕시장 리스트에 올렸다. 이 중 중국 온·오프라인 시장은 타오바오 등 9개로 20%를 차지했다. USTR는 타오바오의 짝퉁 상품 퇴치 노력은 인정했으나 “가짜 상품의 규모를 보여줄 객관적인 지표를 공개하지 않았고 짝퉁 판매량이 줄었다는 사실을 객관적으로 입증하지 못했다”고 재지정 이유를 밝혔다.

　알리바바는 즉각 반발하고 나섰다. 알리바바는 “무역 보호주의가 고개를 들어 고도로 정치화된 환경에서 도널드 트럼프 행정부로부터 점수를 따려는 USTR의 희생양이 됐다. USTR의 조치는 지식재산권을 보호하려는 것이 아니라 미국 정부의 지정학적 목표를 달성하기 위한 수단”이라고 지적했다. 　중국 해관총서(세관)가 12일 공개한 수치에 따르면 중국의 대미 무역흑자는 2758억 달러(약 293조 원)로 사상 최고치를 기록했다. 미국의소리(VOA) 중문판은 “이런 수치는 미중 무역관계의 긴장을 완화시키는 데 도움이 되지 않을 것”이라며 “미중 무역전쟁이 폭풍전야”라고 지적했다.

　이달 초 알리바바의 자회사로 모바일결제 업체인 앤트파이낸셜이 미국의 송금회사 머니그램을 인수합병하려던 계획이 미국 외국인투자심의위원회(CFIUS)에 의해 거부되기도 했다. 미국이 중국을 대표하는 알리바바를 제재하면서 무역전쟁에 시동을 걸고 있다는 관측이 나오는 이유다.

　미국 정부 당국은 중국산 철강 제품 등의 수입이 미국 국가 안보에 손해를 얼마나 끼치는지 조사를 끝낸 뒤 11일 트럼프 대통령에게 보고했다. 트럼프 대통령은 90일 안에 이에 대해 어떤 정책을 내놓을지 결정한다. 미국은 또 이달 중 중국 상품에 대한 각종 조사 결과를 발표할 예정이라고 VOA 중문판이 전했다.

　전문가들은 미국이 중국 상품에 대한 관세를 올리거나 수입 제한 조치를 실제로 본격화하면 중국 역시 같은 방식으로 보복할 가능성이 높다고 본다. 미국의 대중 주요 수출 상품인 보잉 항공기, 자동차, 집적회로, 대두뿐 아니라 할리우드 영화에도 규제를 가할 것이라는 우려가 미국 내에서 나왔다.

# 中측 “사드 해결 없인 한중관계 회복 없어”

“한반도 사드(THAAD·고고도미사일방어체계) 배치가 중한 관계의 걸림돌이다. 사드 문제에 대한 철저한 해결 없이는 한중 관계 회복은 없다.”

　웨이웨이(魏葦) 중국인민외교학회 부회장은 11일 베이징에서 열린 ‘한중 미래발전 싱크탱크 고위급 포럼’에서 이같이 말했다. 문재인 대통령의 방중을 이틀 앞둔 시점에 양국 간 상호 이해와 우호 분위기를 높이고 관계 개선 방향을 논의하기 위해 마련된 포럼이었지만 중국 측 참석자들은 사드 문제를 집중 거론했다.

　웨이 부회장은 관영 환추(環球)시보가 지난달 29일 제기한 ‘3불(不) 1한(限)’에 대해서도 언급했다. 3불 1한은 양국 합의 사항에 3불(사드 추가 배치하지 않고, 미국 미사일방어체계(MD)에 가입하지 않으며, 한미일 3국 동맹으로 발전하지 않는다)뿐 아니라 사드 사용 제한(1한)도 포함돼 있다는 주장이다. 웨이 부회장은 “중국 당국도 직접적으로 말하지는 않으나 3불 1한 입장은 같다”고 말했다. 한국의 한 외교 소식통은 “양국 합의에 결코 사드 사용 제한, 즉 1한이 없었는데 여론전을 펴는 것 같다”고 말했다.

　위훙쥔(于洪君) 전국정치협상회의 외사위원회 위원은 “복잡한 사정이 있지만 지난해 7월 한미의 사드 배치 결정은 적절하지 못했다”며 “이웃 국가의 이익 침해를 고려해야 한다”고 말했다. 위 위원은 “사드 문제로 한중 관계가 냉각된 것은 안타깝다”며 “책임은 한국에 있다”고 말했다. 우쓰커(吳思科) 차하얼학회 국제자문위원도 “사드 도입을 통해 한반도에서 평화를 추구하겠다는 결정은 도움이 되지 않는다”고 말했다.

　자오커진(趙可金) 칭화(淸華)대 사회과학학원 부원장은 “한국이 중국의 사드 반대를 과소평가하고 있다”며 “사드를 배치하는 이유를 알면서도 진실을 말하고 있지 않다”고 말했다. 미국의 MD 편입 등을 지적한 것으로 풀이된다. 이어 자오 부원장은 “한미 동맹이 계속 유지되면 한중 관계에도 걸림돌이 될 것”이라고 말했다. 한미일 3국 동맹이 아닌 ‘한미 동맹’ 자체를 한중 관계에 미치는 부정적인 요소로 지목한 것은 이례적이다.

　배기찬 안보전략연구원 고문은 “북한이 핵전력 완성을 공언한 상황에서도 중국은 한결같이 한국에 강경한 입장”이라며 “한국이 핵무장이라도 하기를 바라는 것이냐”고 반박했다.

　양측의 날 선 공방이 있었지만 문 대통령의 방중을 관계 개선의 기회로 삼아야 한다는 주문도 많았다. 한팡밍(韓方明) 중국 차하얼학회 회장(전국정협 외사위원회 부주임)은 “최근 (사드) 갈등은 양국의 이익에 부합하지 않는다”면서 “이번 양국 정상회담이 중요한 관계 개선의 계기가 되기를 기대한다”고 말했다.

　박은하 외교부 공공외교 대사는 “한 분야에서 발생한 갈등이 확산되지 않도록 관리할 필요가 있다는 것을 확인해 주었다”고 말했다.

# 北에 화난 中, 유엔결의 넘어 독자제재

중국 정부가 유엔 안전보장이사회 제재 이행을 넘어 여행 금지라는 대북 독자 제재를 취한 배경에는 시진핑(習近平) 중국 국가주석의 분노가 있다는 관측이다. 시 주석은 최근 쑹타오(宋濤) 공산당 대외연락부장을 북한에 특사로 보내 김정은 노동당 위원장에게 메시지를 보내려 했으나 거절당했다.

　미국 정부가 올해 8월 자국민의 북한 여행을 금지한 데 이어 중국 정부도 제한적으로 북한 여행 금지 조치를 내려 북한에 상당한 압박이 될 것으로 전망된다. 북한은 그동안 관광을 통해 매년 4400만 달러(약 478억 원)어치의 외화를 벌어들인 것으로 알려졌다. 이 중 중국이 80%를 차지한다. 중국 국가여유국이 2012년 마지막으로 공개한 통계에 따르면 23만7000명의 중국인이 북한을 여행했다.

　하지만 28일 북한 여행객이 집중된 단둥(丹東)과 선양(瀋陽)이 포함된 랴오닝(遼寧)성과 지린(吉林)성의 북한 여행은 허용한다는 얘기가 나온 만큼 실효성은 두고 봐야 한다는 지적도 있다. 이외 지역에서는 수요 감소로 이미 한동안 북한 관광객을 모집하지 않은 여행사도 적지 않다.

　이날 본보가 확인한 결과 베이징(北京)의 대형 A 여행사는 “북한 여행 상품은 계속 없었고 이번에 (금지) 통지를 받지 못했다”고 전했다. B 여행사는 “다음 달 7일 단둥을 출발하는 5일짜리 여행 상품이 있고, 다음 달 중순에는 왕복 비행기 여행 상품이 있다”면서도 “예약할 수 있을지는 불확실하다”고 답했다. 산둥(山東) 지역 여행사도 그동안 북한 여행 상품이 없었다는 반응이었다. 선양 지역 여행사는 “다음 달 16일에 4일짜리 여행상품이 있다”며 “하루 여행(신의주)은 언제든 출발 가능하다”고 답했다. 지린 지역 여행사는 “겨울에는 북한 여행 상품이 없다”고 밝혔다.

# 中학자 “중국, 주저말고 한미와 北 급변사태 논의해야”

쑹타오(宋濤) 공산당 대외연락부장이 시진핑(習近平) 중국 국가주석의 특사로 방북하기 전날 중국의 명문대 교수가 서울에서 열린 한국 정부 주최의 국제포럼에서 북한의 체제 붕괴 등 급변사태에 대비해 한미중 3국이 대화해야 한다고 주장했다. 북한의 핵·미사일 도발에 대한 중국 학계 내부의 반발이 확산되고 있다는 사례로 주목된다.

　상하이(上海) 소재 퉁지(同濟)대 정치및국제관계학원 샤리핑(夏立平) 원장(사진)은 16일 서울 그랜드힐튼호텔에서 열린 ‘2017 동북아 협력포럼’에서 “중국은 더 이상 주저하지 말고 미국 한국과 북한의 급변사태에 대한 논의에 나설 때”라고 밝혔다. 이어 3국의 ‘비상 계획 대화’를 제안하면서 △북한 체제가 붕괴될 때 누가 북한의 핵무기를 통제할 것인가 △북한의 난민 문제를 어떻게 다룰 것인가 △위기 시 북한의 국내 질서를 회복하는 책임을 누가 질 것인가 △위기 후 한반도의 정치적 정돈 등을 의제로 제시했다.

　그는 “중국도 보다 적극적으로 참여한 가운데 제재가 강화되고 있지만 북한이 핵을 포기할 것이라는 전망에는 비관론이 많다”면서 “북한이 중국의 외교적 노력을 무시하면서 핵과 미사일 도발을 계속하는 사태의 비중과 상황의 위험성 등을 고려할 때 이제 중국은 주저 없이 미국 한국과 진지한 대화에 나설 때”라며 이같이 주장했다.

　샤 원장은 이 같은 내용은 중국으로서는 매우 골치 아픈 주제들이지만 미중이 이와 관련해 대화와 협상을 벌이는 것은 양국 간 전략적 교착상태를 푸는 데도 도움이 될 것이라고 덧붙였다.

# 시진핑 특사 쑹타오 방북

“중국의 (대북) 특사가 목표를 진전시키길 바라고 있다.”

　17일 제주도에서 열린 한미 북핵 6자회담 수석대표 협의에 참석한 조셉 윤 미 국무부 대북정책특별대표는 회담 후 기자들과 만나 이렇게 말했다. 한국 측 이도훈 외교부 한반도평화교섭본부장 역시 “지금 시점에 상당히 의미를 부여할 수 있을 것 같다”며 이날 방북한 중국 쑹타오(宋濤) 대외연락부장이 얼어붙은 대북 관계를 녹일 메신저로 나서 주길 기대했다.

　실제 양측 대표는 두 달 넘게 ‘도발 휴지기’를 이어오고 있는 김정은의 의중을 분석했다. 특히 쑹 부장의 방북을 계기로 북한을 6자회담 등 다자외교 채널로 끌어들일 수 있을지 논의했다고 한다. 도널드 트럼프 미 대통령 역시 전날 트위터에 중국의 특사 파견과 관련해 “큰 움직임”이라고 평가했다. 외교가에선 트럼프 대통령이 당초 예상과는 달리 전날 북한을 테러지원국으로 재지정하지 않는 것도 중국의 향후 움직임을 지켜보겠다는 메시지로 해석하고 있다.

　다만 양측 대표는 제재·압박에 중점을 둔 대북 기조가 우선이라는 점은 분명히 했다. 윤 대표는 모두발언에서 “(북핵 문제에 대한) 진전을 만들 수 있을지 (여전히) 많은 숙제를 안고 있다”고 했다. 북한이 비핵화에 대한 구체적인 시그널을 주지 않는 이상 제재 완화 등 국면 전환은 쉽지 않을 거란 얘기다. 한 소식통에 따르면 양측 대표는 한미 정보 교류의 수준을 높여 제재 실효성을 확보하자고 협의한 것으로 알려졌다. 특히 윤 대표는 최근 중국에서 북한 노동자 철수 등 대북제재 효과가 있다고 말하면서 중국의 역할을 거듭 강조한 것으로 전해졌다.

# 아찔한 치파오

‘그 시절은 지나갔고 이제 거기 남은 건 아무 것도 없다‘. 왕가위 감독의 ‘화양연화‘의 한 대목이다. 이루지 못한 사랑을 아련하게 채색한 영화에서 배우 장만옥은 중국 전통의상 치파오를 수십 번 갈아입고 등장해 치명적 매력을 발산한다.

　▷치파오(旗袍)는 높은 옷깃, 치마 옆트임(slit)과 함께 몸에 착 달라붙는 것이 특징. 청나라를 지배한 만주족의 팔기군 복장에서 유래한 옷인데 남녀공용에서 여성의상으로 발전했다. 1920년대 상하이를 중심으로 고혹적 분위기를 강조한 개량형 치파오가 유행하기 시작했다. 1972년 미중 정상회담 때 닉슨 대통령의 부인 패트 여사가 “중국에 왜 인구가 많은지 알겠다”고 했다는 말이 있을 만큼 관능적이다. 2008년 베이징 올림픽에 이어 2010년 광저우 아시안게임에선 시상식 도우미 차림으로 속옷 라인까지 도드라지는 치파오를 선보여 논란을 빚었다.

　▷장만옥의 뒤를 이을 치파오의 새 강자가 나타났다. 9일 중국 국빈 만찬에 미중 퍼스트레이디가 나란히 치파오 블랙드레스 차림으로 등장한 것이다. 멜라니아는 소매에 모피가 달린 화려한 자수 드레스로, 펑리위안은 소매의 맨살이 비치는 시스루 드레스로 양보할 수 없는 패션 대결을 펼쳤다. 모델 출신의 멜라니아는 허벅지 중간까지 쭉 트인 현대적 치파오를 마놀로 블라닉의 킬힐과 매치해 한 걸음 옮길 때마다 돋보이는 각선미를 과시했다. 이 옷은 이탈리아 브랜드 구찌가 2016년 F/W시즌 치파오를 기반으로 내놓은 기성복이다. 펑리위안 은 무릎까지 트인, 상대적으로 정숙한 치파오를 택했다.

　▷이들의 패션 대결과 상관없이 이 날의 최종 승자는 단연 치파오. 중국은 치파오를 단정, 우아, 지성미를 갖춘 여성 외교의 복장이라고 선전한다. 미국 퍼스트레이디 덕분에 돈 한 푼 안 들이고 중국 전통의상의 맵시를 세계에 홍보한 셈이다. 패션은 문화를 넘어 산업적 측면에서도 중요하다. 세계적 디자이너들이 한복과 사랑에 빠져 우리 고유의 미학이 녹아든 옷을 내놓은 날은 언제일까. 치파오의 약진에 비하면 우리 옷의 세계화는 갈 길이 멀다.

# 시진핑 “어떤 국가도 외딴섬 되면 안돼” 북 압박

시진핑(習近平) 중국 국가주석이 18일 집권 2기의 대내외 정책을 천명하는 19차 전국대표대회 업무보고에서 “어떤 국가도 자신을 폐쇄시키는 외딴섬으로 돌아갈 수 없다”고 말했다. 시 주석은 한반도나 북한을 직접 거론하지 않았지만, 국제사회 고립을 자초하며 도발을 계속하는 북한을 겨냥했다는 분석이 나온다.

　시 주석은 이날 베이징(北京) 인민대회당에서 열린 당 대회 개막식 업무보고 가운데 대외정책을 공개하는 대목에서 “세계가 직면한 불안정성과 불확실성이 두드러지고 있다. 지역 분쟁 문제가 여기저기서 끊임없이 발생하고 있다”며 “어떤 국가도 인류가 직면한 각종 도전을 혼자 대응할 수 없다”며 이같이 말했다.

　그는 집권 2기 청사진으로 2단계 국가발전 전략을 제시했다. 1단계로 2020년까지 이룬 전면적 샤오캉(小康·모든 국민이 풍족하게 생활하는 것) 사회의 기초 위에서 2025년까지 사회주의 현대화를 기본적으로 실현하고, 2단계로 2035년부터 21세기 중엽까지 중국을 “부강한 민주 문명과 조화롭고 아름다운 사회주의 현대화 강국으로 만들겠다”고 했다. 그는 “2035년에 국방과 군대 현대화가 실현돼 21세기 중엽에 세계 일류 군대를 전면적으로 건설할 것”이라고도 했다. 자신의 임기(2022년) 이후까지 내다본 것은 장기 집권을 염두에 뒀다는 해석이 나온다.

　시 주석은 “당 전체가 중앙(시 주석)에 복종하고 당의 영도에 통일적으로 집중해야 한다”며 권력 집중을 예고했다. 자신의 사상을 “신시대의 중국 특색 사회주의”라고 밝혔다.

# 베이징대 부원장 “중, 북핵에 큰 책임 대북압박 높여야”

“중국이 (북핵 문제에서) 책임이 없다는 건 말이 안 된다.”

　왕이저우(王逸舟) 베이징(北京)대 국제관계학원 부원장(사진)은 최근 동아일보 인터뷰에서 “중국 정부는 우리에게 (북핵 문제에) 매우 큰 책임과 (한반도에) 매우 중요한 이익이 있음을 명확하게 인정할 필요가 있다. (따라서) 전쟁이 발생하지 않을 정도까지는 대북 압박의 강도를 높여야 한다”며 이같이 말했다.

　그는 이런 자신의 견해가 “북핵 문제에 대한 주요한 책임이 미국과 북한에 있고 우리(중국)의 책임은 부차적인 것이라고 생각하는 (중국) 정부 당국의 입장과 다르다”고 말했다. 중국이 북핵 문제의 직접적인 당사자가 아니기 때문에 중국의 역할에 한계가 있으며 미국이 직접 북한과 대화해 해결하라고 요구해온 중국 정부를 우회적으로 비판한 것으로 풀이된다.

　왕 부원장은 같은 대학의 자칭궈(賈慶國) 국제관계학원장과 함께 중국의 국제적 책임을 강조하는 자유주의학파의 대표적 인사다. 자 원장이 지난달 “중국이 한반도 전쟁 가능성을 인정하고 한미와의 소통 등으로 대비해야 한다”고 주장하자 주류 학파가 “중국 북핵 외교 핵심 원칙의 마지노선을 뒤집은 허튼소리”라며 공개적으로 정면충돌한 데 이어 중국 내에서 한반도 정책 노선 논쟁이 격화되고 있음을 보여준다.

　그는 자 원장의 견해에 대해 “대부분 지지한다”고 말했다. 이어 “중국이 개혁개방으로 갈수록 자 원장의 견해에 찬성하는 목소리가 많아질 것”이라고 전망했다. 특히 왕 부원장은 자 원장 논쟁에 대해 “(중국) 당국이 누가 맞고 누가 틀리다고 얘기하지 않았고 토론을 억누르지 않았다”며 “이는 좋은 일이며 과거에 비해 많은 변화가 생긴 것”이라고 밝혔다. 그는 “중국이 대북 정책을 조정하고 있다. 과거 약했던 제재 강도가 갈수록 커지고 제재의 방향도 엄중해지고 있다”고 말했다.

# ‘중국의 만델라’ 류사오보

간암 말기 판정을 받고 지난달 하순 가석방돼 입원 치료 중인 중국의 대표적인 반체제 인사 류샤오보(劉曉波·62)의 임종이 임박한 것으로 알려졌다. 중국 정부는 그동안 막아온 가족 면회를 허용하며 심각한 상황임을 알렸다고 한다. G20 정상회의 직전 해외의료진의 접견을 허용했던 중국 정부는 회담이 끝나자마자 류 씨의 해외 치료 요청을 거부했다. 이송의 안전을 우려했다지만 실제로는 해외의 반체제 활동에 불을 지를까 우려했을 것이다.

　▷1989년 천안문 사태 때 학생운동을 주도한 그는 반혁명선전선동죄로 징역 3년을 선고받았다. 1995년엔 천안문 사태 재평가를 요구했다가 9개월의 가택연금을, 이듬해엔 대만과의 평화통일을 주장했다가 노동교양 3년형을 선고받았다. 2008년엔 공산당 독재를 비판하는 ‘08헌장’ 서명을 주도했다가 징역 11년을 선고받는 등 총 4차례 연금 또는 수감됐다. 복역 중이던 2010년 중국에서 첫 노벨평화상을 받은 류 씨는 중국 밖에서는 ‘중국의 만델라’로 불린다. 하지만 중국 안에서는 그가 누군지도 모른다. 중국의 최대 포털 사이트 바이두(百度)는 그가 미국 단체의 부정한 돈을 지원받은 것처럼 묘사해 놓았다.

　▷대부분의 반체제 인사들이 당국의 탄압이 두려워 해외 망명을 택했지만 그는 끝내 중국에 남아 투쟁했다. 중국의 유명한 천체물리학자였던 팡리즈(方勵之)나 시각장애인 인권변호사 천광청(陳光誠)도 미국으로 망명하거나 탈출했다. 하지만 그도 부인은 자유로운 나라에서 살게 해주고 싶었던 것 같다. 그가 해외 치료를 원한 건 자신 때문이 아니라 10년째 가택연금 상태인 부인 류샤(劉霞·55)를 위해서였다고 외신은 전했다.

　▷중국에서 사회 불만으로 일어나는 집단시위는 연간 18만 건을 넘는다고 한다. 신장(新疆) 위구르 자치구에서는 매일같이 무장 독립투쟁이 일어난다. 대중이 모르는 사이에 투옥, 수감되는 인사가 부지기수라는 얘기다. 류 씨가 평생 외친 것은 자유 인권 평등 민주 법치였다. 이런 인류 보편적 가치조차 수용 못하는 중국이 세계 지도국가로 갈 길은 멀다.

# 트럼프 보호무역에 반기든 한중일, G20으로 확산돼야

한국과 중국, 일본 경제수장들이 어제 일본 요코하마에서 열린 한중일 재무장관·중앙은행총재회의에서 “모든 형태의 보호무역주의를 배격할 것”이라는 공동선언문을 발표했다. 한중일은 “우리는 무역이 생산성을 향상하고 일자리를 창출하는 등 경제성장의 가장 중요한 엔진이라는 점에 동의한다”며 높은 수준의 공조를 다짐했다. 도널드 트럼프 미국 대통령이 보호무역주의 기치 아래 자국 우선주의를 추구하는 상황에 세계 2위와 3위의 경제대국이 포함된 동북아 3개국 재무장관들이 한 목소리를 낸 것은 주목할 일이다.

　트럼프 대통령 취임 100일 동안 주요 국가들은 미국 눈치 보기에 급급하는 것이 사실이다. 3월 독일에서 열린 G20(주요 20개국) 재무장관·중앙은행총재 회의에서 미국 반대로 지난 3년 동안 공동선언문에 포함됐던 ‘보호주의를 배격한다’는 문구를 넣지 못했다. 한 달 전 크리스틴 라가르드 국제통화기금(IMF) 총재, 김용 세계은행 총재와 앙겔라 메르켈 독일 총리가 독일에서 보호무역주의 확산을 경계한다는 데 의견을 모으고도 지난달 워싱턴 G20 재무장관·중앙은행총재 회의에선 미국이 반발하는 바람에 갈등만 증폭됐다.

　라가르드 총재가 지난해 “1차 대전 이전 같은 보호무역주의는 재앙적인 결과를 초래할 것”이라며 글로벌 경제 성장을 해치고 통합과 사람들도 해친다고 지적했듯이 보호무역주의로 이익을 볼 수 있는 나라는 많지 않다. 특히 무역으로 먹고 사는 한국으로서는 보호무역주의의 암운이 사라지지 않으면 수출 회복세에 타격을 입을 공산이 크다. 시진핑 중국 국가 주석이 1월 스위스 다보스포럼에서 “중국은 자유무역의 수호자가 될 것”이라 해놓고 그 후의 표리부동(表裏不同)한 행동도 실망스럽다. 한국에 대해선 사드(THAAD·고고도미사일방어체계) 배치를 트집 잡아 한국제품 불매 운동을 조장하고 유커들의 한국 방문까지 막았다. 힘의 논리가 우선인 냉엄한 국제통상의 현실이다.

　그럼에도 한중일이 이번에 한 목소리로 자유무역을 수호하겠다고 천명한 것은 중대한 의미를 갖는다. 국경 없는 글로벌 경제에서 통상 문제는 국가안보와 직결되는 전략적 사안으로 격상됐다. 세계 경제와 교역의 20%를 차지하는 한중일 3국이 협력한다면 향후 G20 재무장관회의에서 트럼프의 보호무역주의에 대항하는 분위기를 조성할 수 있을 것이다. 한중일의 공동선언이 말로 그치지 말고 실질적인 정책공조로 이어지도록 해야 한다. 자유무역 정신은 되돌릴 수 없는 세계무역 흐름이라는 사실을 G20으로 확산되는 계기로 만들기 위해 노력할 필요가 있다.
