# Supplementary material for: Bilingual translations of intensifiers in Dong-A Ilbo’s news about China: A corpus-based discourse analysis approach
Source: PLoS One. 2024 Feb 6;19(2):e0292603. doi: 10.1371/journal.pone.0292603 (PMC10846699; doi:10.1371/journal.pone.0292603)
Supplement: S1 File — (ZIP) [file pone.0292603.s001.zip › corpus data/subcorpus of title.docx]

1、美의 中반도체 규제후 중국내 韓점유율 5.5%P↓

Korea’s share in Chinese chips imports drops by 5.5% points（RT）

美国对中国实施半导体限制后，中国进口韩国产半导体占有率减少5.5%（直译）

2、中, 우크라전쟁속 핵무장 박차…사막에 ICBM 격납고

China expedites nuclear armament amid tensions over Ukraine（RT）

中国在乌克兰战争中加快核武装步伐，在沙漠中建洲际导弹库（直译）

3、봉쇄 장기화에 흉흉한 상하이… 항구 폐쇄설도 번져

Extended COVID-19 lockdown in Shanghai sends panic （RT）

上海因封锁长期化而人心惶惶……甚至传出“关闭港口说” （直译）

4、北 도발에 대응할 열쇠 쥔 中, 두둔과 감싸기 더는 안 된다

China’s protection of North Korea should stop （RT）

掌握应对朝鲜挑衅钥匙的中国，不能再维护和包庇（直译）

5、조선 개항 이끈 중국 팽창주의 속내는

Hidden message in China’s expansionism that opens up Joseon’s ports（Direct Translation）

引导李氏朝鲜开放港口的中国，扩张主义的内心想法（增译）

6、1억명 하루 식수를 인공눈으로…베이징 ‘反환경 올림픽’ 논란

Controversy over Beijing Olympics’ artificial snow making （RT）

用1亿人一天的饮用水制造人工雪……北京“反环境奥运会”引发争议（直译）

7、안보리 무력화한 中, 지금 北 고삐 안 잡으면 되레 당할 것

China may face backstab N. Korea if it fails to take action（RT）

中国令安理会失去力气，如果现在不勒紧朝鲜，反而会遭受损失（直译）

8、베이징 올림픽 성화 14년만에 다시 타올라

Olympic torch lit up in Beijing in 14 years（DT）

北京奥运圣火时隔14年之后将再次被点燃（直译）

9、中대륙 못 도는 성화…베이징 인근 사흘만 달려

Beijing Olympic torch to be carried for only three days（RT）

2022年北京冬奥会圣火传递活动规模大幅缩减，只在北京附近传递三天（增译）

10、홍콩 反中매체 시티즌뉴스 폐간선언

Hong Kong’s Citizen News shuts down to protect its staff （AT）

香港反华网媒《众新闻》宣布停刊（直译）

11、G2 암울한 새해 전망…中, 인도에 밀리고 美, 코로나에 치이고

Dark clouds cast gloomy shadows on G2 economies（RT）

G2暗淡的新年展望：中国不及印度，美国被新冠疫情所困扰（直译）

12、中, 한미 가까워질수록 보복할 것…한국이 치러야할 불가피한 대가”

‘Beijing likely to retaliate for closer Seoul-Washington ties’（RT）

郑义溶：“南北关系难以以北京奥运会为契机取得改善”（转译）

13、정의용 “베이징올림픽 계기 남북관계 개선 어려워져”

Foreign minister: Improving inter-Korean ties at Beijing Olympics seems unlikely（DT）

郑义溶：“南北关系难以以北京奥运会为契机取得改善”（直译）

14、삼성전자 “中시안 공장, 코로나 확산에 탄력운영”

Samsung adjusts operations at its Xi’an chip plant amid（RT）

三星电子：“中国西安工厂因新冠疫情扩散实行弹性运营”（直译）

15、美 내년1분기 성장률 전망 5.2% → 2.2% U.S.

growth forecast in Q1 2022 lowered from 5.2% to 2.2%（DT）

美国明年第一季度经济增长率预测值从5.2%下调至2.2%（直译）

16、리그도 연기한 NHL, 베이징 겨울올림픽 안간다

NHL players to skip Beijing Olympics due to COVID-19（DT）

联赛延期的NHL，大概率不参加北京冬奥委会（直译）

17、“화이자-모더나 이외 백신, 오미크론 예방효과 거의 없어”

NYT: Vaccines except Pfizer and Moderna shots offer no protection against Omicron （AT）

美媒：“除辉瑞和莫德纳之外，其他疫苗几乎对奥密克戎毒株没有效果”（增译）

18、文“베이징올림픽 외교 보이콧 검토 안해”

Moon says he is not considering diplomatic boycott of Beijing Olympics（DT）

文在寅：“不考虑外交抵制北京奥运会”（直译）

19、정부, 세계 무역 15% 차지 CPTPP 가입 본격 추진

South Korea applies to join CPTPP after China’s bid（RT）

政府将正式推进占世界贸易15% 的CPTPP的加入工作（直译）

20、美 “베이징올림픽 외교적 보이콧”…中 “결연히 반격”

Moon’s premature announcement on diplomatic boycott of Beijing Olympics （转译）

美国宣布“外交抵制北京奥运会”，中国表示“坚决反击”（直译）

21、美 “베이징올림픽 외교적 보이콧”…中 “결연히 반격”

U.S. announces diplomatic boycott of Beijing Olympics （RT）

美国宣布“外交抵制北京奥运会”，中国表示“坚决反击”（直译）

22、홍콩, ‘보안법’ 1년만에 5500명 학교 떠나

Hong Kong security act causes 5,500 students and teachers to leave school（Additional translation)

香港《保安法》实行一年，5500名学生离校（直译）

中도 저출산-고령화… “4년뒤 ‘성인용 기저귀 〉 유아용’ 역전”

China faces demographic change（RT）

中国也迎来低生育-高龄化……英媒：“4年后大逆转，中国成人尿布需求将大于婴儿尿布”(直译）

서훈 내일 1박 2일 방중…양제츠와 종전선언 논의

NIS chief Suh Hoon to visit China on Thursday（RT）

徐薰明日访华，与杨洁篪讨论终战宣言（直译）

고향과 집

Homeland and home（DT）

故乡和家（直译）

美中 반도체 전쟁에 낀 하이닉스, 초격차 확대로 극복해야

Hynix stuck between U.S. and China for semiconductor war （RT）

陷入中美半导体战争的SK海力士，要以扩大绝对差距来摆脱困局（直译）

美 간 최종건 “현실적으로 中과 파트너십 필요”

Choi Jong-kun says partnership with China is needed realistically（DT）

在美国访问的崔钟建：“现实中需要与中国的伙伴关系”（直译）

中요소수 1만8700t 풀기로 일단 숨통

China decides to export 18,700 tons of urea solution to S. Korea（RT）

暂时松了口气，1.87万吨中国尿素将入境国内救急（直译）

“中헝다 사태 리스크 국제 경제 위협 우려”

Spillover effects of Evergrande liquidity crisis on the global economy（DT）

美联储：“担心中国恒大事态的风险威胁国际经济”（增译）

내년 1월 RCEP 발효… 日 “韓中과 첫 FTA 기대”

RCEP to take effect in next January（RT）

RCEP明年1月生效……日本：“期待与韩国、中国的首个自贸协定”（直译）

미군 2인자 “中군사력 증강 충격, 이대로면 美 추월”

Gen. Hyten: China’s military progress is ‘stunning’ （RT）

美军第二号人物：“中国增强军事力量令人震惊，再这样下去会赶超美国”(直译）

공급난 美中 가격 줄인상…한국도 수입물가 빨간불

Amid supply chain crisis, product price increases in U.S. and China

受全球供应难的影响中美物价持续上涨，韩国进口物价也亮起红灯（直译）

베이징올림픽, 방역 위해 중국 본토 관중만 입장

Spectators residing in China to be allowed entry at Beijing Olympics（RT）

北京冬奥会，处于防疫考虑将只允许中国本土观众入场（直译）

일본인 90% “中 싫어”…중국인 66% “日 싫어”

90% of Japanese ‘dislike China’ and 66% of Chinese ‘dislike Japan’（RT）

90%的日本人“讨厌中国”……66%的中国人“讨厌日本”（直译）

“中, 8월 극초음속 미사일 시험발사… 美 놀라게 했다”

Korea’s first space launch vehicle successfully lifts off China test-fired hypersonic missile, says Financial Times （RT）

《金融时报》：“中国8月份试射高超音速导弹……令美国大吃一惊”（直译）

시진핑, 떠나는 메르켈에 이례적 웃음… 한시 인용 덕담

Xi Jinping wishes farewell to German chancellor（RT）

习近平与即将卸任的默克尔视频会谈，破例展露笑颜……引用古语表示祝福（增译）

유가 80달러… 공급대란에 高물가 ‘쓰나미’까지 밀려오나

Soaring oil prices stir inflation fears（RT）

油价80美元……供应大乱中，高物价“海啸”也会袭来吗？（直译）

대규모 전력난에… 중, 호주산 석탄 다시 수입 ‘백기’

China imports coal from Australia again（RT）

面临大规模电力短缺，中国再次“举白旗”进口澳大利亚煤炭（直译）

‘파산 위기’ 中헝다 사태 여파 스웨덴까지 번져

Aftermath of China’s Evergrande woes hits Sweden（DT）

面临“破产危机”的中国恒大，瑞典也受到波及（直译）

정의용 “中 공세적 외교는 당연”…韓외교수장, 美서 ‘中두둔’ 논란

Foreign minister’s remark siding with China under fire（RT）

郑义溶：“中国采取攻势外交是理所当然的”……在美国发表维护中国的言论引发争议（直译）

9•11테러 20년, 美中‘힘의 오만’ 접고 경쟁•협력해야

U.S. and China cooperate while competing against each other（RT）

“9·11”恐怖袭击20周年，中美应该收起“力量的傲慢”，展开竞争和合作（直译）

中 베이징대 교수, 시진핑 ‘공동부유’ 정면비판

Beijing Univ. professor criticizes Xi's ‘common wealth’（DT）

北京大学教授张维迎正面批评习近平“共同富裕”（直译）

바이든, 아프간 철군 다음날 “이제 中-러 등 새 위협 대처해야”

Biden says U.S. should deal with new threats（RT）

拜登在从阿富汗撤军的第二天表示：“现在应该应对中俄等新威胁”（直译）

中 허난성 물폭탄에 최소 33명 사망…전세계 아이폰 절반 공급 공장도 타격

Heavy rain affects 3 million in China’s Henan province, 33 dead（RT）

中国河南省大暴雨至少造成33人死亡…全球一半苹果手机供应工厂也受到打击（直译）

文 만난 셔먼 “中과 대북정책 심도있게 논의할 것”

Sherman: U.S. will discuss with China over N. Korea policy（RT）

舍曼会见文在寅，“将与中国深入讨论对朝政策”（直译）

“中, 선양수용소 수감된 탈북자 50여명 北 보내”

Beijing sent over 50 defectors in detention back to N. Korea（RT）

自由亚洲电台：“中国将被关押在沈阳收容所的50多名‘脱北者’遣返朝鲜”（增译）

‘치사율 80%’ 원숭이B바이러스, 中서 첫 사망자

First human death due to monkey B virus reported in China（RT）

“致死率80%”，中国首例人类感染猴B病毒致死病例（直译）

北과 밀착하는 中 “美, 수십년간 北위협 반성해야”

China criticizes U.S. for several decades’ menace to N. Korea（RT）

中国与朝鲜靠近，“称美国应对数十年威胁朝鲜反省”（直译）

北 ‘코로나 국경봉쇄’ 1년만에 中과 교역 재개

N. Korea resumes trading with China in more than a year （RT）

北韩“新冠疫情封锁边境”时隔一年重启与中国的贸易（直译）

시진핑 “中 괴롭히면 머리 깨져 피 흘리게 될것”

Xi: Those bullying China will have their heads bashed and bloodied（DT）

习近平：“谁妄想欺负中国，必将头破血流”（直译）

공산당 창당 100주년 앞둔 베이징 ‘반계엄 상태’

Beijing is practically under martial law with 100th anniversary of Chinese Communist Party’s foundation（DT）

即将迎来建党100周年，北京处于“半戒严”状态（直译）

바이든, CIA 등에 “코로나 中실험실 기원 재조사하라”

Pres. Biden orders CIA to investigate origins of COVID-19 virus（RT）

拜登要求中情局“重新调查中国实验室新冠病毒起源” （直译）

中대사 “한국, 대만 언급 없었다면 좋았을것”

Chinese ambassador takes issue with S. Korea’s mentioning Taiwan（FT）

中国驻韩大使：“韩国如果没有提及台湾就好了”（直译）

두달만 뛰면 되는 中택한 김연경, 다음 목표는 美? 유럽?

Kim Yeon-koung chooses to play in China for her next season（RT）

选择只打两个月的中国联赛的金软景，下一个目标是美国还是欧洲？（直译）

홍콩, 대만공관 일방폐쇄… 교류 끊나

Hong Kong closes Taiwan trade office amid rise in diplomatic tensions（FT）

香港单方面关闭驻台湾办事处，中断交流（直译）

‘中, 달 이어 화성도 터치다운…‘우주굴기’ 전세계 과시

China lands on Mars following its touchdown on moon （RT）

中国探测器继月球后着陆火星……向全球展示“太空崛起”（直译）

中 22t 우주로켓 잔해 주말 지구 낙하 가능성

Debris of China’s 22-ton space rocket could fall onto Earth（RT）

中国重达22吨的运载火箭残骸有可能周末坠落地球（直译）

블링컨 “中 점점 더 공격적 행동…세계 지배국 되려 해”

Blinken remarks China’s “more aggressive” actions （FT）

布林肯：“中国采取越来越有攻击性的行动，想成为支配世界的国家”（直译）

“신의주역 가림막 철거…” 北中국경 봉쇄 해제 징후

Pyongyang- Beijing border looks set to reopen（RT）

“新义州火车站拆除遮布……”朝中边境有解除封锁迹象（直译）

美 ‘대북제재 완전한 이행’ 밝힌 날, 中은 한국에 ‘제재 완화 노력’ 촉구

China urges S. Korea to make efforts to alleviate N. Korea sanctions（RT）

美国表示“完全履行联合国对朝制裁”的当天，中国敦促“韩国努力放宽制裁”（直译）

현대건설기계, 中진출 최대 2500억원 수주

HCE wins orders worth of 250 billion won in China（RT）

2500亿韩元！现代建设机械承揽公司史上在华最大订单（直译）

중국, 자국 백신 접종 외국인 비자 발급 간소화 하기로

China simplifies visa requirements for foreigners inoculated with Chinese vaccine

中国决定对接种本国疫苗的外国人简化签证发放程序

바이든 정부 첫 안보지침 “동맹과 단합해 중에 대응” Biden administration plans to respond to China by working with allies

拜登政府推出第一个安保方针，“团结同盟应对中国”

시진핑 “회색코뿔소 - 블랙스완에 대비하라” 黨에 지시 Xi says China should prepare for crisis

习近平在党内指示：“做好应对灰犀牛与黑天鹅的预案”

시진핑 ‘파카’ 입었더니… 관련 의류회사 주가 10% 껑충 Anta Group’s shares get boost after Xi Jinping showcases parka

习近平穿上羽绒服亮相，相关服装公司股价猛涨10%

미국은 왜 중국의 일대일로 경계하나 Why does US counter China’s initiative?

美国为什么警惕中国的“一带一路”？

“달 표면 샘플 44년만에 가져왔다” 中들썩 China’s Chang’e-5 mission returns with Moon samples

“时隔44年带回月球表面样本”，中国一片欢腾

中관영언론 “김치-파오차이 완전히 다른 음식” Kimchi and paocai are two different foods,’ Chinese state media says

中国官方媒体：“韩式泡菜和中式泡菜是完全不同的食物”

“北, 中에 올해 4000억원대 석탄 밀수출" N. Korea smuggled over $400 million worth of coal to China

美国媒体：“朝鲜今年向中国走私价值4000亿韩元的煤炭”

美의회, 中견제 구상에 국방예산 2조원 책정 US Congress agrees on bill for counter-China initiative

美国议会就牵制中国构想制定2万亿韩元国防预算

中, 4년만에 한국게임 서비스 허가...한한령 풀리나 촉각 China gives approval to Korean gaming service in four years

中国4年以来首次批准韩国游戏服务，限韩令解除or只是个例？

BBC “중국 김치가 세계 표준?...중 언론 오보” BBC calls reports of kimchi by Chinese media false

英国广播公司（BBC）：“中国泡菜成为世界标准？……中国媒体纯属误报”

왕이가 띄운 ‘한중일FTA’, 아직은 외교•경제 리스크 크다 Risks over Korea-China-Japan FTA

王毅提出的“韩中日自贸协定”，目前外交和经济风险仍然很大

귀국 한국인 직원 감염… SK하이닉스 中공장 가동중단 SK Hynix Chinese plant suspends operation due to COVID-19 case

回国的韩国职员确诊感染新冠病毒... SK海力士中国工厂现已停产

“이소룡, 여전히 우리 가슴속에 있다” 탄생 80주년 행사 잇따라 Events commemorating Bruce Lee held in Greater China

“李小龙依然活在我们心中”，李小龙诞辰80周年纪念活动接连举行

미국 편에 서지 말라 압박하며 한한령은 풀지 않는 중오만 China stays arrogant to maintain restrictions on Korean wave content

傲慢的中国，施压韩国不要站在美国一边，却不解除“限韩令”

한국 온 中왕이 “세계에 미국만 있는 게 아니다” Chinese Foreign Minister visits South Korea

王毅访韩，“世界上不仅仅只有美国”

다시 찾아온 중국발 미세먼지 재앙... ‘저자세’ 외교로는 해결 요원하다 Ultra-fine dust from China blankets S. Korea again

来自中国的可吸入颗粒物之灾再次到来，依靠“低姿态”外交，解决将遥遥无期

文대통령, 中주도 RCEP 가입 서명 Pres. Moon signs China-led economic partnership

文在寅总统签署加入中国主导的RCEP协定

블랙핑크 판다 맨손 터치 논란 Chinese netizens, media attack Black Pink members for touching panda

BLACKPINK不戴手套触摸熊猫幼崽引发争议

현대차 “2025년까지 중에 수소트럭 3000대 보급” Hyundai to supply 3,000 hydrogen trucks to China

现代汽车：“到2025年为止，将在中国普及3000辆氢电卡车”

두산인프라, 中서 굴착기 생산 20만대 돌파 Doosan Infracore reaches 200,000 mark for excavator production in China

斗山工程机械在中国生产的挖掘机累计突破20万台

BTS “한국전쟁, 한미 고난의 역사”에 中 생트집 BTS faces backlash from Chinese fans over Van Fleet Award speech

BTS表示“韩国战争是韩美苦难的历史”，中方吹毛求疵

트럼프 “中 의존 영원히 끝낼것” 또 공격 Trump: U.S. will end its reliance on China once and for all

特朗普再次攻击中国，“将永远结束对中国的依赖”

中, 美영사관 보복 폐쇄 ‘전면전’China retaliates U.S. by closing its Consulate in Chengdu

中国报复，关闭美国领事馆，发动“全面战争”

“中어선, 동해 북한 수역서 오징어 5200억원어치 잡아들였다” China retaliates U.S. by closing its Consulate in Chengdu

研究结果：“中国渔船在朝鲜东部海域捞走价值5200亿韩元的鱿鱼”

‘휴스턴 中총영사관 폐쇄’ 갈등 고조 Chinese ships catch $440 million worth of squid in N. Korean waters

“关闭中国驻休斯敦总领事馆”矛盾愈演愈烈

美-中홍콩갈등 폭발 ‘헥시트’ 문이 열린다 U.S.-Sino tensions rise following closure of Chinese Consulate in Houston

美中香港矛盾爆发，“退出香港（HK-exit）”大门开启

코로나19가 왜 美-中대립의 속도를 높이나 Why COVID-19 further strains the U.S.-China relations?

新冠疫情为何加快美中对立速度？

中외교부 “한반도 안정 희망”... 北에 자제 촉구 China’s foreign ministry urges North Korea to remain calm

中国外交部：“希望韩半岛稳定”……敦促朝鲜克制

“베이징 집단감염 원인 유럽 수입연어 가능성” Imported salmon blamed for second coronavirus wave in Beijing

北京疾控中心官员：“北京集体感染原因可能是欧洲进口鲑鱼”

美 “中보복땐 한국 위해 뭐든 할 준비 돼” U.S. promises to protect S. Korea against any Chinese retaliatory action

美国：中国报复时，可以为韩国做任何事情

美 “中에 함께 맞서자“... EPN 참여 원칙과 실익 면밀 검토해야 U.S. wants S. Korea to stand by its side against China

美国：“一起对抗中国吧”……要仔细研究参与EPN的原则和实际利益

한국 조선 수주 점유율, 中과 격차 좁혀 S. Korea closes gap in shipbuilding order share with China

韩国造船订单占有率缩小与中国的差距

무역협회 “美中 홍콩갈등 격화땐 韓수출 타격” Worsened conflicts between U.S. and China over Hong Kong to undermine S. Korea’s export

韩贸易协会：中美香港矛盾激化将打击韩国出口

미중 갈등 속 中위안화 가치 급락... 유탄 피할 길 없는 한국경제 China lowers yuan amid conflicts with the U.S.

中美矛盾中人民币大幅贬值，无法避免流弹的韩国经济

‘기업인 신속통로’ 이용 삼성전자-SK이노 등 중에 인력 550여명 파견 S. Korean companies send around 550 employees to China

三星电子和SK Innovation等利用“企业人绿色通道”向中国派遣550多名人力

백악관 “中은 약탈경제” 新냉전 선포 White House attacks China’s ‘predatory economic practices’

白宫称“中国是掠夺性经济”，宣布新冷战

美中전방위 패권전쟁 개전...한생존전략 정립하라 S. Korea needs survival strategy amid U.S.-China dispute

中美全方位霸权战打响……韩国应确立生存战略

美, 양회 앞둔 中에 “또라이” “악랄한 독재정권” 원색비난 U.S. harshly criticizes China calling it authoritarian regime

美国在两会前夕不加掩饰地指责中国为“疯子”“恶劣的独裁政权”

트럼프 “WHO, 한달내 중서 독립 증명해야...아니면 탈퇴할수도” Pres. Trump asks WHO to prove independence from China

特朗普：“世卫组织要在一个月内证明独立性，否则美国可能退出”

美구축함, 상하이 인근 해상까지 접근 U.S. destroyer sails off coast of Shanghai

美驱逐舰接近上海附近海域

“미친 짓”까지 터져나온 美-中갈등 U.S.-China tensions are through the roof

美中矛盾加剧，甚至出现“疯狂之举”

美中갈등 속 시진핑 방한에 매달려선 우리 외교 길을 잃는다 The importance of not being swayed amid escalating U.S.-China tensions

在美中矛盾中执意寻求习近平访韩，将失去韩国外交道路

NYT “中, 해커 동원해 美코로나 백신자료 노려” China tries to hack COVID-19 research data, NYT reports

《纽约时报》：“中国利用黑客窥伺美国新冠疫苗资料”

美-中코로나 갈등으로 다시 무역전쟁 전운...수출처 다변화 등 전략짜야 U.S.-China conflicts on COVID-19 reignite tensions in global trade

美国与中国因新冠矛盾再起贸易战阴云……韩国应制定出口多元化等战略

‘코로나 우한硏발원설’ 목청 키우는 美...트럼프 “中끔찍한 실수” S. raises voice of criticizing Wuhan for causing COVID-19 outbreak

美国竭力宣称“新冠病毒武汉研究所起源说”，特朗普称“中国可怕的失误”

트럼프 “코로나 중서 발생 증거봤다” 보복관세 시사 Trump says he’s seen evidence coronavirus started in China lab

特朗普暗示要征收报复关税，称“已经看到新冠病毒在中国发生的证据”

트럼프 “中에 코로나 배상책임 물을것...진지하게 조사 진행중” Pres. Trump says to demand compensation for COVID-19 from China

特朗普：“将向中国追究新冠疫情赔偿责任……目前正在认真进行调查”

하얼빈 71명 집단감염에... 中, 다시 ‘準봉쇄조치’ With 71 cases in Harbin, China takes lockdown measures again

哈尔滨71人集体感染，中国再次采取“准封锁措施”

거세지는 ‘中코로나 책임론’... 메르켈 “中투명하게 공개해야” China under growing criticism for COVID-19 outbreak

愈演愈烈的“中国新冠疫情责任论”……默克尔：“中国应透明地予以公开”

中1분기―6.8% 사상첫 마이너스 성장 China’s economy records historic quarterly plunge due to coronavirus

中国第一季度经济增长率为-6.8%，历史上第一次负增长

中우한 코로나 사망자 축소 의혹 사실로 China’s cover-up of COVID-19 death toll in Wuhan is revealed

中国武汉市新冠肺炎死亡病例核增1290例

中기습 입국금지에 기업인 등 대혼란 China bars entry by almost all foreigners to contain virus pandemic

中国奇袭式禁止入境，导致企业人士等乱成一团

美확진자 8만5000명...中넘어 세계 최다 S. overtakes China for most confirmed COVID-19 cases

美国新冠肺炎确诊病例已超8.5万......超越中国成世界最大感染国

세계화 최대 수혜자 중국에 닥친 2차 위기 Another crisis awaits China, the biggest beneficiary of globalization

全球化最大受益者中国遭遇的第二次危机

싱하이밍 주한 中대사 “한국 기업인 격리 면제할수도” China could take exception to S. Koreans’ entry, says Ambassador Xing

中国驻韩国大使邢海明：“可以免除韩国企业人士的隔离”

시진핑 “韓中은 같은 배 탄 우호국...힘닿는한 돕겠다” ‘S. Korea and China are friendly nations on the same boat,’ says Xi

习近平致电文在寅：“中韩是同舟共济的友好邻邦……中国将提供力所能及的援助”

中외교부 “日의 입국제한 이해”...문제제기 안해 China understands Japan’s entry restrictions with little objection

中国外交部“对日本的入境限制表示理解”，不提出问题

“시진핑 방한 변함 없지만 코로나 극복 안되면 영향” The coronavirus outbreak could affect Xi’s S. Korea visit

韩国政府高官：“习近平访韩不会改变，但如果新冠疫情不能克服，将受到影响”

시진핑 “코로나19 발원지 찾아라” 지시 Xi Jinping instructs to find the origin of COVID-19

习近平指示“查明COVID-19的发源地”

中최상급 보건당국 “에어로졸 전파 가능성 존재” China’s health authorities acknowledges possibility of aerosol transmission

中国国家卫健委：“COVID-19存在气溶胶传播可能性”

환자 9배 폭증...중 신뢰잃은 ‘코로나 통계’ China’s coronavirus statistics loses credibility with 9-fold increase

患者暴增9倍，失去信任的中国“新冠肺炎”统计 /

中환자 증가폭 매일 신기록...홍콩서도 첫 사망자 The number of confirmed cases of coronavirus keeps increasing in China

中国新型冠状病毒感染病例增幅创造单日新纪录，香港出现首例死亡病例

中대사 “입국제한, WHO 근거 따라야” Chinese ambassador asks S. Korea to follow WHO stance

中国驻韩大使：“入境限制应根据世卫组织的规定”

WHO “전례없는 확산” 국제비상사태 선포 WHO declares Wuhan coronavirus outbreak a global health emergency

“史无前例的扩散”，WHO宣布将新型冠状病毒疫情列为“国际关注的突发公共卫生事件”

中서 무증상자發 집단전염...3차감염 사례도 Asymptomatic patient spreads the virus in China

中国出现无症状自发集体传染......第三次感染病例也出现

시진핑을 흔든 ‘우한의 기침’...성장률-리더십 위기 Wuhan’s cough shakes up Xi Jinping leadership

“武汉咳嗽”动摇习近平，增长率和领导能力面临危机

우한 폐렴 경보 Warnings of Wuhan pneumonia

武汉肺炎警报

中, 폐렴 발원지 우한시 뒤늦게 전면봉쇄 China shuts down Wuhan where pneumonia began

中国昨天才全面封锁肺炎发源地武汉市

美로 번진 우한폐렴, 대유행 조짐 ‘Wuhan pneumonia’ may become a global epidemic

“武汉肺炎”扩散到美国，出现大流行迹象

춘절 앞둔 中, ‘사스 공포’ 재현되나 ‘Fears of SARS’ rise in China ahead of Lunar New Year holiday

春节在即，中国会否再现“非典恐慌”？

중국몽 위협하는 새로운 세대의 등장 New generation poses threat to ‘Chinese Dream’

威胁中国梦的新一代登场

美-中1단계 무역 합의... 중국 수출 비상 걸린 한국 S.-China ‘Phase One’ deal

中美达成第一阶段贸易协议，韩国对华出口进入紧急状态

中, 美전역 사정권 SLBM 시험발사...러-이란과 합동훈련도 China test-launches SLBM that can reach U.S.

中国试射把美全境纳入射程的潜射导弹，还与俄、伊举行联合军演

美안팎 퍼지는 차이나 배싱…복잡한 글로벌 외교 방정식 China-bashing spreads in and outside U.S.

扩散至美国到处的“敲打中国”，复杂的全球外交方程式

中, 27년만에 최저성장 침체 그림자 짙어진다 China’s economy marks lowest quarterly growth in 27 years

中国出现27年来最低增长率，停滞阴影渐浓

홍콩시위대-중국군 첫 대치...부대 접근하자 “후폭풍 책임져야” China's military, Hong Kong protesters confront Sunday night

香港示威者首次与中国军队对峙，刚刚接近部队就被警告“要为后果负责任”

애국주의 들끓는 건국 70년… 中내부에서도 책임 목소리 Patriotism prevails in China on 70th anniversary of foundation

爱国主义沸腾的建国70年，中国内部也有责任的声音

시진핑 “어떤 힘도 중국 흔들지 못해” ‘No force can shake China,’ says Xi Jinping

习近平：“任何力量也无法撼动中国”

中, 위안화 환율 또 올려… 美는 中가구에 징벌관세 China sets its yuan midpoint weaker Friday

中国再次下调人民币汇率，美国对中国输美家具征收惩罚性关税

中, 홍콩시위 ‘색깔혁명‘ China views Hong Kong protests as 'color revolution'

中国认为香港示威是“颜色革命”

사드 처음 명시한 中국방백서… “긴장 고조” 美비난 China’s defense white paper makes mention of THAAD deployment in S. Korea

中国国防白皮书首次明确提及萨德系统，猛批萨德“加剧紧张”

美-中, 화웨이 불씨 둔채 무역 2차휴전 Trump, Xi agree to truce in U.S.-China trade war

美中贸易战二次停火，保留华为火种

방북 임박說시진핑, 비핵화 훼방꾼은 되지 말아야 Xi Jinping must not hinder denuclearization efforts

传习近平将访问朝鲜，不要成为无核化的干扰者

중국이 세계를 지배하면 If China rules the world

如果中国统治世界

화웨이 진영 싸움 확전 조짐 Huawei CFO’s arrest could impact U.S.-China trade talks

华为之争出现扩大征兆

“한국인들, 중국이 기만할 것 우려해” Chinese scholar argues S. Koreans concerned over China’s deception

“韩国人担心中国欺骗韩国”

靑는 “中이 양보”-中은 “핵심이익 수호” 다른말 U.S.-China tariffs war truce likely to prove fragile

美国“中国作出了让步”VS中国“捍卫了国家利益”，美中各说各话

대만 민진당 선거 참패 뒤엔... 中의 경제압박 있었다 Beijing’s economic pressure affected DPP’s election loss in Taiwan

台湾民进党选举惨败，背后是中国大陆的经济施压

中 과학자들 “유전자 편집 아기 강력 규탄” 성명 Chinese researcher claims world's first gene-edited babies

中国科学家们发表声明“强烈谴责基因编辑婴儿”

中 모래폭풍 덮쳐...미세먼지에 황사까지 ‘컥컥’ China's sandstorms hit Korea with fine and yellow dust

来自中国的沙尘暴从天而降，从微尘到黄沙“让人喘不过气来”

AI 기술 미국 따라잡는 중국…‘삼성 반도체’도 잡는 날 오면 China’s AI ambitions

人工智能技术紧追美国的中国……如果“三星半导体”也被追上的那天到来

中 3분기 성장률 6.5%...금융위기 이후 최저 China's third quarter GDP rises 6.5 percent year-on-year

中国第三季度增长率为6.5%，创金融危机后最低纪录

존엄한 국가 흥얼거린 죄...중 인터넷 스타 철창행 Chinese live-streamer detained for insulting national anthem

哼唱国歌获罪，中国“网红”铁窗行

中 “단둥-평양-서울-부산 연결”…일대일로 한반도 확장 첫 명시 China proposes to expand its Belt and Road Initiative

中国提出“连接丹东-平壤-首尔-釜山”，首次明确“一带一路”向韩半岛延伸

시진핑 9•9절 방북 사실상 무산... 대타 왕후닝 유력 Xi Jinping’s visit to North Korea seems unlikely

习近平事实上不可能“9·9节”访问北韩，王沪宁有可能代替前往

中군용기, 한달만에 또 KADIZ 침범 Chinese warplane enters KADIZ again

中国军机时隔一个月再次进入韩国东海防空识别区

이란 원유 최대수입국 中, 美제재에 전전긍긍 China anxious due to Trump's Iran sanctions

伊朗原油的最大进口国中国对美国的制裁战战兢兢

中 vs 대만 올림픽 모델 충돌 Taiwan slams China after sports event canceled

中国与台湾就奥运会模式产生冲突

“관료부패가 가짜백신사태 불러” ...분노한 중민심 SNS 타고 확산 Chinese people expose fury on social media over the vaccine scandal

“官僚腐败事件引发虚假疫苗事件”……愤怒的中国民心在社交媒体中扩散

16일 中-EU 정상, 경제동맹 재편 회담 E.U., China to discuss to reshape economic ties in Beijing

中国与欧盟16日举行领导人会晤，会谈重组经济同盟

불붙은 무역전쟁... 中 ‘소방수’ 왕치산이 안보인다 S.-China trade war in flames

火热的贸易战争……看不见中方“消防员”王岐山

中의 반격…美반도체 마이크론 판매금지 Beijing counterattack begins by banning Micron’s chip sales in China

中国进行回击……禁止美国半导体企业在华销售

흔들리는 北제재… 美中 점검회의 중단 China becoming a hole in North Korea sanctions

动摇的对北韩制裁……美中会议检查会议已告中断

美-中, 관세전쟁 중단… 무역협상 타결 U.S., China agree to abandon trade war

美与中国停止关税战争……贸易谈判达成一致

中첫 국산 항모 시험항해... 연안 벗어나 원양으로 ‘군사 굴기’ China puts its first natively-built air carrier to sea

中国第一艘国产航母试航……摆脱沿岸前往远洋的“军事崛起”

北달려간 왕이, 남북미중 회담 요구할듯 Chinese foreign minister likely to request a 4-nation summit

王毅访问北韩，似乎将要求举行南北美中四方会谈

中베이징대 학생들, 검열 뚫고 ‘미투’MeToo movement spreads in China

中国北京大学的学生们，突破审查喊出“我也是”

中매체 “6·25 같은 의지로 美와 무역전쟁” Chinese media: ‘Fight trade war with U.S. the same way as Korean War’

中国媒体：“用打抗美援朝的意志来打对美贸易战”

中, 작심 반격…미국산 콩에 보복관세 China retaliates with new levies on U.S. products

中国下决心反击……对美国产大豆实施报复性关税

北 최고위급, ‘中의 실리콘밸리’ 중관춘 방문뒤 귀국 China’s VIP security raises speculation of Kim Jong Un visit

北韩最高领导人参观“中国硅谷”中关村后回国

중국, 北-美 대화 지원하고 ‘한반도 평화’ 후원자 돼야 China should support a peaceful Korean Peninsula

中国应支持北韩与美国对话、成为“韩半岛和平”的襄助者

트럼프-김정은 5월 정상회담에..주변국은 China’s response to the announcement of Kim-Trump meeting

特朗普与金正恩5月举行元首会谈，周边各国怎么看？

中 국방비 8% 늘려 사상 최대… 美와 패권경쟁 가속 China expands defense budget by 8% for 2018

中国国防开支增加8%，创历史纪录，加快与美国霸权竞争

中 지식인들 “마오시대로 돌아가나”...시진핑 장기집권 반발 Xi Jinping abolishes the two-term limit on the presidency

中国知识分子反对习近平长期执政，“要回到毛泽东时代吗？”

“中, 남중국해 인공섬 ‘軍지휘센터’로 활용” China uses South China Sea islands as military control center

香港媒体：“中国把南中国海上的人工岛用作‘军事指挥中心’”

中, 러시아판 사드배치… 美에 맞대응 China deploys Russian surface-to-air missile system

为对抗美国，中国拟部署俄罗斯版“萨德”

中개혁개방 1번지 선전, 특구 장벽 허물었다 China removes historic border round Shenzhen economic zone

中国改革开放第一号深圳，撤销经济特区管理线

美-中무역전쟁 일촉즉발 U.S.-China trade war appears to be imminent

美中贸易战一触即发

中측 “사드 해결 없인 한중관계 회복 없어” China urges S. Korea to address THAAD issue

中方：“不解决萨德问题，中韩关系就不可能恢复”

北에 화난 中, 유엔결의 넘어 독자제재 China slaps sanctions on N. Korea on top of UNSC resolutions

中国生了气，超出联合国决议单方面制裁北韩

中학자 “중국, 주저말고 한미와 北 급변사태 논의해야” ‘China should talk with Korea and the U.S.,' says a Chinese scholar

中国学者：“中国不应犹豫，应与韩美磋商北韩剧变事态”

시진핑 특사 쑹타오 방북 Chinese envoy should warn against N. Korea's nuclear ambitions

习近平的特使宋涛访问北韩

아찔한 치파오 Chinese costume Qipao

令人晕眩的旗袍

시진핑 “어떤 국가도 외딴섬 되면 안돼” 북 압박 ‘No country can afford to retreat into self-isolation,’ says Xi Jinping

习近平称“任何国家都不能成为孤岛”，暗指北韩

베이징대 부원장 “중, 북핵에 큰 책임 대북압박 높여야” ‘China has a great responsibility in N.K. nuke,’ says Chinese scholar

北京大学副院长：“中国应对北韩加大压力”

‘중국의 만델라’ 류사오보 Liu Xiaobo, China’s Nelson Mandela

“中国的曼德拉”刘晓波

트럼프 보호무역에 반기든 한중일, G20으로 확산돼야 Korea, China, Japan say no to Trump's protectionism

扛起反对特朗普贸易保护主义旗帜的韩中日，要将之扩大到G20
