# Supplementary material for: Bilingual translations of intensifiers in Dong-A Ilbo’s news about China: A corpus-based discourse analysis approach
Source: PLoS One. 2024 Feb 6;19(2):e0292603. doi: 10.1371/journal.pone.0292603 (PMC10846699; doi:10.1371/journal.pone.0292603)
Supplement: S1 File — (ZIP) [file pone.0292603.s001.zip › corpus data/total corpus.docx]

美의 中반도체 규제후 중국내 韓점유율 5. 5%P↓

Korea’s share in Chinese chips imports drops by 5.5% points

美国对中国实施半导体限制后,中国进口韩国产半导体占有率减少5.5%

미국의 대중국 반도체 규제 이후 중국 수입 반도체 시장에서 한국산이 차지하는 비중이 줄어든 것으로 나타났다

The share of Korean exports in China’s semiconductor market has been reduced since Washington slapped sanctions against Beijing

据调查,美国对中国实施半导体限制后,韩国产半导体在中国进口半导体市场上所占的比重有所减少

전국경제인연합회는 2018년 대비 2021년 중국 수입 반도체 시장의 국가별 점유율 변화를 분석한 결과 한국은 5. 5%포인트가 낮아졌다고 25일 밝혔다

The Federation of Korean Industries announced Monday that Korea’s share in the Chinese semiconductor imports has fallen by 5 .5 percentage points from 2018 to 2021

全国经济家联合会25日表示,对与2018年相比,2021年中国进口半导体市场的各国占有率变化进行分析的结果显示,韩国下降了5.5个百分点

제재 당사자인 미국(0. 3%포인트 하락)보다 더 영향을 받은 것이다 대만(4. 4%포인트), 일본(1. 8%포인트), 아세안 6개국(0. 4%포인트)의 점유율은 미국의 규제 전보다 더 증가했다

The fall was even more drastic than that of America’s (fallen by 0. 3 percentage points), the architect of the anti-Chinese sanctions By contrast, the shares of other exporters in Asia rose compared to the pre-sanctions era as indicated by Taiwan (by 4. 4 percentage points), Japan (1. 8 percentage points), and six ASEAN countries (0. 4 percentage point)

这比制裁当事者美国(下降0.3个百分点)更受影响中国台湾(4.4个百分点) 日本(1.8个百分点) 东盟6国(0.4个百分点)的占有率比美国的限制规定前有所增加

미국 상무부는 2019년 4월∼2020년 9월 네 차례에 걸쳐 중국 화웨이, SMIC를 상대로 미국의 반도체 소프트웨어·장비를 활용해 생산한 반도체의 공급을 거래제한명단에 올리는 등의 방식으로 규제했다

From April 2019 to September 2020, the U S Department of Commerce put Huawei and SMIC on a trade blacklist over four times, blocking their supplies of semiconductors produced by using American software and equipment

美国商务部在2019年4月~2020年9月分四次以中国华为 SMIC为对象,以交易限制名单的方式限制了利用美国的半导体软件 设备生产的半导体供应

미국 제재 전인 2018년과 비교했을 때 지난해 중국의 전체 반도체 수입 규모는 37. 2%나 늘었다 한국산 수입도 6. 5% 늘긴 했으나 치열하게 경쟁 중인 대만산(57. 4%), 일본산(34. 8%) 등과 비교했을 땐 증가 폭이 작다

Compared to 2018, the size of China’s semiconductor imports swelled by 37. 2 percent last year Chinese imports increased by 6. 5 percent, but the rise was meager compared to the growth of the Taiwanese or Japanese imports (by 57. 4 and 34. 8 percent, respectively)

与美国制裁前的2018年相比,去年中国的半导体进口规模增加了37.2%韩国产进口虽然增加了6.5%,但与竞争激烈的台湾产(57.4%) 日本产(34.8%)等相比,增幅较小

중국의 한국 반도체 수입 비중이 줄어든 결정적 원인은 미국 규제의 영향으로 한국 기업들의 화웨이 공급이 중단됐기 때문이다

The biggest contributor to the fall in Korea’s share in the Chinese imports was American’s sanctions, which prevented Korean companies from supplying for Huawei

中国进口韩国半导体的比重减少的决定性原因是受美国限制的影响,韩国企业中断了华为的供应

제재가 시작된 2019년 기준 삼성전자와 SK하이닉스의 전체 매출에서 화웨이가 차지하는 비중은 각각 3. 2%(약 7조3700억 원)와 11. 4%(약 3조 원)였다

As of 2019 when the sanctions began to be imposed, the shares of Huawei in Samsung Electronics’ and SK Hynix’s revenues stood at 3. 2 percent (about 7. 37 trillion won) and 11. 4 percent (about 3 trillion won)

以制裁开始的2019年为基准,华为在三星电子和SK海力士的总销售额中所占的比重分别为3.2%(约7.37万亿韩元)和11.4%(约3万亿韩元)

中, 우크라전쟁속 핵무장 박차…사막에 ICBM 격납고

中国在乌克兰战争中加快核武装步伐,在沙漠中建洲际导弹库

중국 지도부가 미국에 맞선다는 명분으로 핵무장에 박차를 가하고 있다고 미국 월스트리트저널(WSJ)이 보도했다

China expedites nuclear armament amid tensions over Ukraine China has ramped up its efforts to develop nuclear weaponry under the pretext that it will get ready to face the United States, said The Wall Street Journal

据美国《华尔街日报》报道,中国领导层以对抗美国为由,正在加快核武装的步伐

우크라이나 전쟁에서 나온 블라디미르 푸틴 러시아 대통령의 핵 위협이 효과를 발휘했다는 판단을 한 것으로 풀이된다

This can be interpreted that nuclear threats from Russian President Vladimir Putin have worked out during the Russian invasion of Ukraine

分析认为,在乌克兰战争中出现的俄罗斯总统普京的核威胁发挥了效果

9일 WSJ는 중국 지도부 사정을 잘 아는 소식통을 인용해 “중국은 우크라이나 전쟁 전부터 핵전력 증강을 추진 중이었고 이번 전쟁을 통해 확신을 얻게 됐다”면서 “미국이 전쟁에 직접 개입을 자제하는 이유에 대해 러시아가 보유한 핵무기 때문이라는 결론을 내린 것으로 보인다”고 전했다

An anonymous source familiar with Chinese leadership was reported on Saturday by the WSJ that China pursued a nuclear buildup even before the Russia-Ukraine war, which convinced it of its nuclear pursuits, assuming that Beijing concluded that Washington’s hesitation to intervene is driven by Russia’s possession of nuclear weapons

《华尔街日报》9日援引熟悉中国领导层情况的消息人士的话报道说:“中国在乌克兰战争前就一直在推进核战斗力的增强,通过此次战争得到了确信,”“对于美国克制直接介入战争的原因,可能得出了是俄罗斯拥有的核武器的结论”

이어 “중국은 대만과 군사적 충돌이 발생했을 때 미국이 핵무기를 사용할 수 있다는 우려를 내세워 이에 맞대응하기 위한 핵무기를 늘리고 있다”고 덧붙였다

“Chinese leaders see a stronger nuclear arsenal as a way to deter the U S from getting directly involved in a potential conflict over Taiwan,” the source added

报道还说:“中国表示担心与台湾发生军事冲突时美国会使用核武器,因此正在增加核武器来应对”

중국과 대만 간 충돌이 발생할 경우 우크라이나 전쟁에서처럼 미국의 직접 개입을 막기 위해 핵무기를 증강하고 있다는 것이다

This means that China is enhancing its nuclear capabilities to keep the United States from directly intervening in any conflict with Taiwan just as in the ongoing war in Ukraine

也就是说,如果中国和台湾之间发生冲突,就像乌克兰战争一样,为了防止美国的直接介入,中国正在增强核武器

위성사진을 분석한 전문가들에 따르면 중국은 서부 사막 지역인 간쑤성 위먼(玉門) 인근에 있는 신형 대륙간탄도미사일(ICBM) 둥펑(DF)-41 격납고 의심 시설 100여 곳 건설의 마무리 작업을 서두르고 있다

Experts argue that satellite images show increased activity to get construction works on more than 100 sites believed to be silos for a new long-range IBCM called the DF-41 around Yumen, a desert region in western China

据分析卫星照片的专家们介绍,中国正在加紧建设位于西部沙漠地区甘肃省玉门附近的新型洲际弹道导弹“东风(DF-41)”机库可疑设施100多处

1월 촬영된 위성사진에서는 격납고를 가리고 있던 임시 장막이 모두 제거됐다 이는 정보 노출이 우려되는 민감한 작업이 끝났다는 것을 의미한다

Satellite images taken this January show temporary covers on silos having been removed, implying that confidential works involving sensitive information have already been finished

在1月份拍摄的卫星照片中,遮挡机库的临时帐篷全部被拆除这意味着担心信息泄露的敏感工作已经结束

핵탄두 탑재가 가능한 둥펑-41의 최대 사거리는 미국 본토 전역을 타깃으로 할 수 있는 1만5000km인 것으로 알려졌다

Carrying nuclear warheads, the DF-41 has an operational range of up to 15,000 kilometers, which can reach the U S

据悉,可搭载核弹头的“东风-41”的最大射程为1.5万公里,可以瞄准美国本土全境

중국 당국은 이 격납고에 대해 함구하고 있다

mainland China remains silent about these

中国当局对这个机库缄口不语

미국 전문가들은 현재 중국이 핵탄두 수백 개를 보유했을 것으로 추정하고 있다 2020년대 말 1000여 개까지 늘어날 것이라는 관측이 나온다

U S specialists speculate that China holds hundreds of nuclear warheads, expecting the number hereof to increase up to 1,000 by the late 2020s

美国专家推测,目前中国拥有数百枚核弹头有预测称,到2020年代末,这一数字将增至1000多枚

봉쇄 장기화에 흉흉한 상하이… 항구 폐쇄설도 번져

上海因封锁长期化而人心惶惶……甚至传出“关闭港口说”

중국 경제 수도 상하이의 신종 코로나바이러스 감염증(코로나19)으로 인한 봉쇄가 길어지면서 시민들의 공포가 커지고 있다

Extended COVID-19 lockdown in Shanghai sends panic Extended COVID-19 lockdown in Shanghai, the economic capital of China, is sending its citizens in panic

上海因封锁长期化而人心惶惶……甚至传出“关闭港口说”随着中国经济首都上海因新冠疫情的封锁时间延长,市民的恐惧正在加大

상하이 항구의 선적·하역 대기 선박도 봉쇄 이후 300척 이상 급증해 물류 장애가 더 심해질 것이라고 CNN이 보도했다 Further disruption in logistics is also expected as the number of ships waiting to load or discharge at Shanghai’s port has soared to more than 300 since the lockdown, according to CNN

据美国有线电视新闻网(CNN)报道,上海港等待装货卸货的船只在封锁后也激增至300艘以上,物流障碍将更加严重

상하이 봉쇄가 장기화하면서 지난달 28일 생산을 중단한 전기차 업체 테슬라의 공장 폐쇄도 계속될 것으로 보인다 As the lockdown continues, Tesla, which was forced to suspend production on last Monday, is likely to idle its production in Shanghai for the time being

随着上海封锁长期化,3月28日停产的电动汽车企业特斯拉也将持续关闭工厂

2019년 말 가동을 시작한 이래 최장기간 중단이다 It is the longest suspension of production since Tesla started its operation in the city in late 2019

这是自2019年末启动以来中断时间最长的一次

4일 중국 국가위생건강위원회에 따르면 전날 하루 중국 전역의 코로나19 확진자는 1만3137명으로 역대 최다인 2020년 2월 12일 1만5152명에 근접했다 According to National Health Commission of China on Monday, 13,137 new COVID-19 cases were reported across the country on the previous day, approaching its all-time high of 15,152 on Feb 12, 2020

据中国国家卫生健康委员会4日透露,前一天中国全国新冠确诊患者为1.3137万人,接近2020年2月12日1.5152万人的历史最高纪录

이날 상하이 신규 확진자는 9006명이었다 Shanghai reported 9,006 daily COVID-19 cases on the same day

当天上海新增确诊病例9006例

당초 시 당국이 밝힌 봉쇄 시한은 4일까지였지만 봉쇄는 계속되고 있다 Shanghai still remains in lockdown although city officials initially said the city will be placed under lockdown until Monday

当初上海市当局公布的封锁时限是4日,但封锁仍在继续

상하이 시민의 불안 심리가 커지면서 관련 소문도 확산되고 있다 Rumors are spreading as the citizens of Shanghai are increasingly experiencing anxiety

随着上海市民的不安心理增大,相关传闻也在扩散

3일 웨이보(중국판 트위터)에서는 상하이 한 병원에서 어린아이가 코로나19 치료를 못 받아 숨졌다는 소식과 동영상이 빠르게 퍼졌다 News and video spread fast on Weibo on Sunday that a child infected with COVID-19 died at a hospital in Shanghai after not being treated

3日微博上迅速传出了上海某医院一名儿童因新冠治疗无效死亡的消息和视频

이 동영상에서는 병상에 누운 아이에게 의료인이 다가가는 모습이 보이고 화면 밖에서 부모인 듯한 사람들의 고함이 들린다 In the video, medical staff is approaching a child lying on a bed and shouts of someone who appears to be the parents’ screams can be heard outside the camera

该视频中可以看到医务人员走近躺在病床上的孩子的样子,画面外还听到了像父母一样的人的喊叫声

상하이시 위생건강위원회는 이날 밤 이례적으로 성명을 발표하고 “해당 영상은 상하이 푸단대병원에서 고열로 의식을 잃은 아이를 응급처치 하려던 장면”이라고 공식 해명했다 그러면서 “아이는 의식을 회복했고 부모도 의료진에게 사과했다”고 전했다 The Shanghai Municipal Health Commission issued an unusual statement that night, officially clarifying that the child, who lost consciousness due to high fever, was offered emergency treatment at the Fudan University Hospital in Shanghai, adding that the child regained consciousness and the parents later apologized to the medical staff

上海市卫生健康委员会当晚破例发表声明,正式解释说:“相关视频是上海复旦大学医院对因高烧而昏迷的孩子进行急救的场面”声明说:“孩子恢复了意识,父母也向医疗人员道歉”

상하이에 채소 등을 공급하는 업체가 채소를 쌓아뒀다가 폐기했다거나, 세계 최대 물류항인 상하이항이 곧 폐쇄된다는 소문도 퍼지고 있다 Other rumors also spread that a company supplying vegetables had to discard piles of vegetables and the Port of Shanghai, the largest port in container throughput, will soon be closed

有传闻称,上海供应蔬菜的企业把蔬菜堆放不管后又废弃,世界最大的物流港上海港即将关闭等

이에 대해 상하이시 당국은 사실이 아니라고 해명하고 있지만 정부에 대한 불신이 누적된 시민들은 믿지 않는 분위기다 Despite the city authorities’ explanation that the rumors are not true, the citizens, who are having increasing levels of distrust of the government, do not seem to believe in them

对此,上海市当局虽然解释说不是事实,但对政府的不信任累积的市民却不相信

상하이시는 “봉쇄는 없다”고 공식 발표한 다음 날인 지난달 28일 전격적으로 봉쇄를 시작하는 등 이미 시민들의 신뢰를 잃은 상태다 The city of Shanghai has already lost the trust of its citizens as it began a lockdown on last Monday, the next day after it had announced that there would be no lockdown

上海市在正式宣布“不会封锁”的第二天即3月28日开始全面封锁,已经失去了市民的信任

北 도발에 대응할 열쇠 쥔 中, 두둔과 감싸기 더는 안 된다

掌握应对朝鲜挑衅钥匙的中国,不能再维护和包庇

윤석열 대통령 당선인이 어제 시진핑(習近平) 중국 국가주석과 첫 전화 통화를 했다 China’s protection of North Korea should stop South Korean President-elect Yoon Suk-yeol had the first conversation over the phone with Chinese President Xi Jinping

韩国当选总统尹锡悦昨天与中国国家主席习近平进行了首次通话

북한의 ‘괴물 ICBM’ 발사 다음날 이뤄진 통화다 It came the day after North Korea’s launch of the ‘monster ICBM'

这次通话是在朝鲜发射“怪物洲际导弹”的第二天进行

윤 당선인은 한반도 안보 상황과 북한의 도발에 대한 대응방안을 놓고 시 주석과 협의했다 Yoon discussed with Xi the security situation on the Korean Peninsula and responses to North Korea’s provocations

尹锡悦就韩半岛安保情况和应对朝鲜挑衅的方案与习主席进行了协商

북한은 이날 ICBM이 ‘화성-17’형임을 확인하며 ‘믿음직한 핵전쟁 억제수단’으로 완성됐다고 주장했다 North Korea confirmed on Friday that the ICBM was the Hwasong-17 and claimed that it had been completed as a reliable method to deter nuclear wars

朝鲜当天确认洲际导弹是“火星-17”型导弹,并声称是“可靠的核战争遏制手段”

‘괴물 ICBM’ 발사로 레드라인을 깬 북한은 핵 위협 수위를 노골적으로 높이고 있다 North Korea is baldly raising the level of nuclear provocations by breaking the red line with the launch of the ‘monster ICBM'

通过发射“怪物洲际导弹”打破红线的朝鲜正在露骨地提高核威胁水平

김정은 북한 국무위원장은 “용감히 쏘라”고 적은 친필 명령서를 하달했고, 평양 순안비행장을 찾아 발사 과정을 지켜봤다 North Korean leader Kim Jong Un delivered a handwritten order that read “Shoot with bravery” and visited Pyongyang Sunan Airport to watch the launch process

朝鲜国务委员长金正恩下达了亲笔命令书,要求“勇敢地发射”,并前往平壤顺安机场观看了发射过程

북한 매체는 이를 보도하면서 ‘핵’ 단어를 13번 사용했다 A North Korean news agency covered the story and used the word ‘nuclear’ 13 times

朝鲜媒体对此进行了报道,使用了13次“核”一词

핵실험을 비롯한 추가 도발을 언제라도 이어나갈 태세다 It seems like the country will continue further provocations, including nuclear tests

朝鲜摆出了随时准备继续进行核试验等追加挑衅的样子

북한의 망동을 막아야 할 중국의 대응은 실망스러웠던 게 사실이다 It is true that the response of China, a country supposed to prevent the North’s impulsive behavior, was disappointing

应该阻止朝鲜妄动的中国应对确实令人失望

중국은 그제 북한의 ICBM 발사에 “유관 각국이 대화, 협상의 올바른 방향을 견지하기 바란다”고 했다 “We hope each relevant country will maintain an appropriate direction for dialogues and negotiations,” said China on Thursday regarding the North’s ICBM launch

中国前天对朝鲜发射洲际导弹表示:“希望有关各国坚持对话 协商的正确方向”

규탄은커녕 주변국에 책임을 떠넘기는 듯한 뉘앙스다 Rather than condemning the North, China seems to be shifting the responsibility to other countries

不仅没有谴责,反而把责任推给周边国家

중국은 유엔 안보리에서 마땅히 내야 할 목소리도 내지 않고 있다 중국이 신냉전 기류에 편승해 북한의 도발 여지를 열어줬다는 비판을 피하기 어렵다 China is also not raising its voice in the U N Security Council, as it cannot avoid criticism that it took advantage of the New Cold War atmosphere and opened a room for North Korea’s provocations

中国也没有在联合国安理会发出应有的声音中国难免会受到“借助新冷战的潮流,为朝鲜打开了挑衅余地”的批评

중국이 대북 문제를 놓고 당장 윤 당선인과 협력할 가능성은 크지 않다 It is unlikely that China will work with Yoon on the North Korean issues

中国就对朝问题立即与尹锡悦合作的可能性不大

중국은 ‘당당한 외교’를 천명한 윤 당선인의 대중 정책에 불편한 기색을 내비쳐 왔다 The country has been expressing its discomfort with Yoon’s policy of ‘confident diplomacy’ toward China

中国一直对阐明“堂堂正正外交”的尹锡悦的对华政策表现出不满

그렇다고 중국이 북한의 핵 도발까지 마냥 방조하고 있을 여유는 없다 However, there is no reason for China to aid and abet North Korea’s nuclear provocations

即便如此,中国也没有余力帮助朝鲜进行核挑衅

북한의 핵기술 고도화는 3기 집권을 앞둔 시 주석에게 정치적 부담이자 안보 위협이다 North Korea’s sophistication of nuclear technology is a political burden and security threat to President Xi before his third term

朝鲜核技术的提升对于即将开始第三个任期的习主席来说既是政治负担,也是安保威胁

주변국의 핵 확산을 부추겨 지역 불안정을 가속화하는 요인이다 It is a factor accelerating regional destabilization by encouraging neighboring countries’ nuclear proliferation

这是助长周边国家核扩散 加速地区不稳定因素

중국은 지금이라도 북한이 도발을 중단하고 대화에 나서도록 압박에 나서야 한다 China should stop North Korea’s provocation and put pressure on the country to come to dialogues

哪怕从现起,中国也应该向朝鲜施压,要求朝鲜停止挑衅,进行对话

우선 국제사회의 대북 제재에 어깃장만 놓는 태도부터 바꿔야 한다 China should first change its attitude of obstructing the international community’s sanctions against North Korea

首先应该改变与国际社会对朝制裁对着干的态度

유엔 안보리 결의 2397호는 북한의 ICBM 발사시 자동으로 추가 제재를 부과하는 ‘트리거 조항’을 담고 있지만, 이 또한 결의안이 통과돼야 가능하다 The U N Security Resolution 2397 has a trigger clause that automatically applies additional sanctions against North Korea in case of its ICBM launch but it is only effective when the resolution is passed

联合国安理会第2397号决议虽然包含了朝鲜发射洲际导弹时自动追加制裁的“触发条款”,但这也要通过决议才能实现

중국의 동참은 국제사회의 일원으로서 마땅히 져야 할 의무다 China’s participation is a responsibility as a member of the international community

中国的参与是作为国际社会一员应该承担的义务

새 정부 또한 출범 직후 중국과 조속한 실무 협의를 할 수 있도록 준비해야 한다 The new South Korean administration should also prepare to begin close cooperation with China right after it takes office

新政府也应该做好准备,以便在上台后尽快与中国进行实务协商

북 핵 저지는 한중 양국이 국익이 걸린 공통분모이자 향후 관계를 좌우할 핵심 현안이다 Deterring North Korea’s nuclear weapons is a common ground on which the national interests of South Korea and China depend and a key issue that will determine the future relationship of the two

阻止朝鲜拥核是关系到韩中两国国家利益的共同分母,也是左右今后关系的核心问题

조선 개항 이끈 중국 팽창주의 속내는1882년 6월 조선 군사들이 궁궐에 난입한 직후 일본과 청은 즉각 출병에 나선다 Hidden message in China’s expansionism that opens up Joseon’s ports Right after Joseon soldiers invaded into the palace in June 1882, Japan and the Qing dynasty sent their troops to the Korean Peninsula

引导李氏朝鲜开放港口的中国,扩张主义的内心想法1882年6月朝鲜军人闯入宫中后,日本和清朝立即出兵

일본이 군함과 300명의 병력을 제물포로 보낸 데 이어 청이 광둥(廣東) 주둔군을 중심으로 3000명의 병력을 남양만에 상륙시킨다 While Japan dispatched warships and 300 soldiers to Jemulpo, 3,000 Qing troops landed on Namyang Bay

继日本派遣军舰和300人到济物浦之后,清朝也派遣以广东驻军为主的3000名兵力登陆南洋湾

일촉즉발의 위기에 양국 간 교섭이 시작되고, 청군은 군란의 배후에 있던 대원군을 톈진(天津)으로 납치하기에 이른다 After the two external forces started negotiations with the peninsula being a tinderbox on the verge of war, Qing troops hijacked and took Heungseon Daewongun, who was behind the coup, to Tianjin

在一触即发的危机中,两国开始交涉,清军甚至将兵乱背后的大院君绑至天津

미국 캘리포니아 데이비스대(UC데이비스) 교수로 동아시아 근대사를 연구한 저자는 이 책에서 19세기 후반 한중일 3국 간 국제관계가 근대 세계질서로 편입되는 과정을 추적했다 The author, a professor of East Asian modern history at University of California, Davis, traces how the relations of Korea, China and Japan were incorporated into the modern international order around the late 19th century

作者作为美国加利福尼亚大学戴维斯分校教授,一直研究东亚近代史,他在该书中追踪了19世纪后半期韩中日三国间的国际关系被并入近代世界秩序的过程

저자는 특히 중국 중심의 조공 체제가 와해된 역사적 사건으로 임오군란을 바라보고 있다 He describes the Imo Mutiny as a historical incident that dismantled the geopolitical system to pay tribute to the Qing dynasty

作者特别将寅午兵乱视为以中国为中心的朝贡体制瓦解的历史事件

당시 청은 군란을 진압한 후에도 한반도에 군대를 계속 주둔시키며 조선 내정에 깊이 간여했다 Even after suppressing the riots, it kept the troops stationed on the peninsula while meddling deeply in internal affairs of the Joseon dynasty

当时,清朝在镇压兵乱后继续在韩半岛驻扎军队,深度干预朝鲜内政

이는 유교 질서에 따라 종주국으로서 의례적 권한만 행사할 뿐, 조공국 내정에 간섭하지 않는 조공 체제 전통과 어긋나는 행태였다 The way it behaved ran against the conventional tribute system where a colonial master is entitled to rights only formally without interfering with a tributary state’s domestic politics

这是违背了根据儒教秩序,作为宗主国只行使礼仪性权限,不干涉朝贡国内政的朝贡体制传统的行为

중국이 이처럼 팽창주의로 기운 건 당시 일본, 러시아의 동아시아 침투가 직접적인 원인이었다 China’s leaning toward expansionism was directly driven by the invasion of Japan and Russia into East Asia

中国如此倾向于扩张主义,直接原因是当时日本 俄罗斯渗透到东亚地区

북중국은 물론 수도 베이징과도 멀지 않은 한반도는 자국 안보에 있어 핵심 완충국이었다는 것이다 The Korean Peninsula was considered by the Qing dynasty a core buffer state, not far from North China and the capital city of Beijing, from a perspective of national security

不仅是中国北部,离首都北京不远的韩半岛也是本国安全的核心缓冲国

흥미로운 건 아편전쟁 이후 서구 열강과 굴욕적 외교 조약을 맺은 청이 조선에 열강과의 조약을 통한 개항을 요구한 점이다 What’s interesting is that the Qing dynasty demanded a treaty to open Joseon‘s ports overseas after it was virtually forced to sign a humiliating diplomatic treaty with Western powers after the Opium Wars

有趣的是,鸦片战争以后,与西方列强签订屈辱性外交条约的清朝要求朝鲜通过与列强的条约开放港口

이는 서구 열강들을 끌어들여 일본, 러시아를 견제하려는 이른바 변형된 이이제이(以夷制夷) 전략이었다 Involving Western powers in the landscape, it intended to keep Japan and Russia in check In other words, the purpose of its diplomatic tactic was to take advantage of opponents to defeat another group of enemies

这是旨在拉拢西方列强牵制日本和俄罗斯的所谓“以夷制夷”战略

그런데 이것은 조선이 대등한 주권국 간의 외교 행위를 근간으로 하는 ‘근대 세계질서’에 편입되는 걸 의미했다 This turned out to be Joseon’s first step taken to be part of an international order of modern times under which sovereign states forge a diplomatic relationship in an equal position

但这意味着朝鲜被编入以对等主权国家之间的外交行为为基础的“近代世界秩序”

다시 말해 이 책 제목이 암시하듯 종주국을 정점으로 한 동아시아 세계질서가 사라지는 종막(終幕)이었던 셈이다 As the title of this book implies, it was the last phase of the East Asian order that had centered around a colonial master

换句话说,正如这本书的题目所暗示的那样,这是以宗主国为顶点的东亚世界秩序消失的终结

최근 베이징 겨울올림픽 판정 논란으로 반중 정서가 팽배한 가운데 중국을 어떻게 볼 것이냐가 화두가 되고 있다 With anti-Chinese sentiment prevailing following the judging controversy in the Beijing Winter Olympics, how we view China is becoming a hot potato

最近,由于北京冬奥会的裁判争议,反华情绪高涨,如何看待中国成为人们热议的话题

6·25전쟁의 분수령이 된 마오쩌둥의 참전 결정 이전에 19세기 청의 팽창주의가 한반도를 둘러싼 동아시아 세계질서를 바꾼 한 축이었음은 의미하는 바가 적지 않다 The expansionism-driven Qing dynasty in the 19th century reshaped the East Asian regional order surrounding the Korean Peninsula before Mao Zedong’s decision to join war turned out to be a watershed of the Korean War

在成为6·25战争分水岭的毛泽东决定中国参战之前,19世纪清朝的膨胀主义是改变围绕韩半岛的东亚世界秩序的一轴

1억명 하루 식수를 인공눈으로…베이징 ‘反환경 올림픽’ 논란중국이 2022년 베이징 겨울올림픽의 모든 설상 경기를 인공 눈으로 치르기로 하면서 환경에 악영향을 미칠 수 있다는 우려가 제기되고 있다 This has implications for us today Controversy over Beijing Olympics’ artificial snow making Concerns are growing that the 2022 Beijing Winter Olympics may have a negative impact on the environment as many competitions will be held on man-made snow

用1亿人一天的饮用水制造人工雪……北京“反环境奥运会”引发争议随着中国决定用人工雪进行2022年北京冬奥会的所有雪上比赛,有人担心会对环境产生负面影响

이번 대회 인공 눈의 양은 1억 명이 하루에 마시는 물의 양과 맞먹어 경기장 인근 주민들의 물 부족 사태가 벌어질 것이란 전망도 나온다 Almost a daily amount of drinking water for 100 million people will be turned artificially into snow over the Olympic period, increasing concerns that residents around event venues will face a severe water scarcity

有人预测说,此次大赛的人工雪量相当于1亿人每天喝的水量,因此,赛场附近居民将出现缺水现象

중국은 베이징 올림픽 개회식에서 작은 성화를 선보이며 ‘친환경 올림픽’이라고 강조했지만 이와 대조되는 현상이 벌어지고 있는 것이다 Although China promoted its pursuit of eco-friendliness during the opening ceremony with a smaller-sized flame on display, the very opposite is happening on the site

中国在北京奥运会开幕式上展示小圣火,强调“环保奥运会”,但与此形成鲜明对比的现象正在发生

미국 CNN은 이번 대회가 겨울올림픽을 열기에 적합하지 않은 기후에서 진행되고 있고, 지구온난화로 인한 기온 상승까지 겹쳐 인공 눈 제조에 더 많은 전력과 물이 소모될 것이라고 5일 보도했다 Beijing does not only have a climate not conducive to winter events but also has to spend more electricity and water producing snow amid growing global temperatures due to a warming planet, according to CNN on Saturday

美国有线电视新闻网(CNN)5日报道说,此次大会在不适合举办冬奥会的气候下进行,再加上全球变暖导致的气温上升,人工雪的制造将消耗更多的电力和水

국제올림픽위원회(IOC)에 따르면 이번 올림픽에서 인공눈을 만들기 위해 약 4900만 갤런(약 1억8548억 L)이 소모될 것으로 예측된다 이는 약 1억 명이 하루에 마시는 물의 양과 비슷하다 The International Olympic Committee estimated that around 49 million gallons of liquid water or 185 48 million litters will be used to provide artificial water to competition venues, which is equivalent to the volume of water that 100 million people drink a day

据国际奥委会透露,本届奥运会上,为了制造人工雪,预计将消耗约4900万加仑(约1.8548亿升)这与约1亿人每天喝的水量相似

야외 종목이 열리는 지역 중 상당수가 올겨울 극심한 가뭄 탓에 강설량이 부족해 인공눈에 대한 의존도가 커졌다는 분석도 나온다 As severe droughts have hit most of regions where outdoor competitions during the Olympics open over this winter, China has become highly dependent on artificial snow, say experts

有分析称,在举行户外项目的地区中,大部分地区由于今年冬天严重的干旱,降雪量不足,因此对人工雪的依赖度增大

야외 종목이 진행되는 장자커우(張家口) 지역은 평소에도 연평균 강설량이 200mm에 불과하다 With only an average snowfall of 200 millimeters a year, Zhangjiakou, a city which hosts outdoor competitions, is one of the driest regions in China

进行野外项目的张家口地区平时的年均降雪量也只有200毫米

1인당 사용 가능한 물의 양이 중국 전체 평균의 5분의 1도 안 되는 건조한 지역이다 An available amount of water per person in the city is less than a fifth of the national average

人均可用水量不到中国平均水平的五分之一,属于干燥地区

앞서 블룸버그는 이 지역의 스키장을 채우기 위해 200m³의 물이 필요하지만 53m³밖에 확보되지 못했다고 보도하기도 했다 Although it takes 200m³ of water to fill in ski resorts in Zhangjiakou, only 53m³ is reported to be secured as of now, said the Bloomberg

此前彭博社曾报道说,为了填满该地区的滑雪场,需要200立方米的水,但只确保了53立方米

중국은 ‘친환경 올림픽’을 실현했다며 자화자찬하는 분위기다 China seems to be proud of realizing a “eco-friendly” Olympic event

中国自称实现了“环保奥运会”

4일 개회식에서 올림픽 사상 가장 작은 성화를 선보인 장이머우 총감독은 “연료가 대량으로 쓰이는 대형 성화 대신 중국 정부의 환경친화적 아이디어를 전달한 것”이라고 했다 Director Zhang Yimou said that the smallest torch ever in history at the opening ceremony on Friday is a representation of the Chinese government’s green efforts to say no to a larger-sized flame that wastes a bulky fuel

在4日的开幕式上展示奥运会历史上最小的圣火的张艺谋总导演表示:“这是中国政府代替大量使用燃料的大型圣火传递的环保创意”

안보리 무력화한 中, 지금 北 고삐 안 잡으면 되레 당할 것북한의 중거리탄도미사일(IRBM) 도발에 대응하기 위한 유엔 안정보장이사회 회의가 4일 열렸지만 어떤 결과물로 내놓지 못한 채 종료됐다 China may face backstab N Korea if it fails to take action The UN Security Council meeting was held on Friday to come up with measures to respond to North Korea’s intermediate range ballistic missile issue but adjourned after concluding no outcomes

中国令安理会失去力气,如果现在不勒紧朝鲜,反而会遭受损失为应对朝鲜发射中程弹道导弹的挑衅,联合国安理会4日召开会议,但未能拿出任何结果就宣告结束

북한의 잇단 미사일 도발에 올해 들어 세 번째 열린 안보리 회의였지만 이번에도 안보리 이사국 과반의 공동대응 요구에 거부권을 쥔 중국과 러시아의 반대에 부딪쳤기 때문이다 It was the third Security Council meeting to be held this year on account of North Korea’s missile firing, but no agreement was reached as China and Russia vetoed against the joint action request made by more than half of the Security Council members

这是因为,虽然这是安理会今年以来第三次针对朝鲜接连发射导弹挑衅举行会议,但这次需要安理会过半数理事国共同应对的要求,也遭到了掌握否决权的中国和俄罗斯的反对

중국 측은 북한의 도발을 규탄하기는커녕 오히려 미국을 향해 “북한의 우려사항을 수용하는 정책과 행동을 보이라”고 주장했다 China did not even denounce North Korea’s provocative actions and urged the U S to exhibit policies and actions that accommodate North Korea’s concerns

中国方面不仅没有谴责朝鲜的挑衅,反而向美国主张“要表现出接受朝鲜担忧事项的政策和行动”

이번 안보리 회의에선 사거리 5000km의 IRBM 도발을 논의한 만큼 이전 두 차례 단거리미사일 때와는 다를 것이라는 기대도 없지 않았다 There were some expectations that the recent meeting, in which IRBM range of 5,000 kilometers was discussed, would be different from the outcomes of the previous two meetings

在此次安理会会议上,讨论了射程达5000公里的洲际弹道导弹挑衅问题,因此也不无期待认为此次与之前两次短程导弹不同

2017년 북한의 무더기 핵·미사일 도발 때 안보리는 중거리급 도발에도 북한 기관과 단체를 대북제재 명단에 추가하는 등 적극 대응했다 Back in 2018, the UN Security Council had come up with proactive measures to North Korea’s nuclear/missile threats, adding North Korean institutions and groups to its sanction list

2017年朝鲜进行大量核 导弹挑衅时,安理会曾将朝鲜机构和团体列入对朝制裁名单,积极应对

하지만 북한이 다시 4년여 만에 가장 높은 수위의 도발을 벌였는데도 안보리 차원의 성명 한 장도 나오지 않았다 Despite North Korea’s threats that have reached the highest ever in the last four years, the Security Council has not even issued a single statement

但是,朝鲜再次进行了4年多来最高级别的挑衅,但安理会却连一张声明都没有发表

중국 측이 이번엔 언론성명 초안을 본국에 보내 검토한다지만, 이 역시 흐지부지될 가능성이 적지 않다 China said that it would send a copy of a press statement draft back to its country for review, which may not happen as well

中方此次将媒体声明草案送交本国讨论,但是也很有可能不了了之

안보리 대응이 무산된 뒤 미국을 포함한 9개국은 공동성명을 내고 “안보리의 침묵은 북한을 더욱 대담하게 만들어 결의 위반을 당연시하고 국제평화를 계속 위협할 것”이라고 했다 Nine countries including the U S issued a joint statement saying that “the silence of the UN Security Council would only embolden North Korea, violating the resolutions and continually threatening international peace"

安理会应对告吹后,包括美国在内的9个国家发表联合声明说:“安理会的沉默会使朝鲜更加大胆,认为违反决议是理所当然的,将继续威胁国际和平”

안보리 이사국이 아닌 일본도 참여했지만 한국은 빠진 이 성명은 한낱 우려에 그치지 않는다 Japan, which is not a member of the Security Council, joined the statement, but South Korea was not involved

非安理会理事国的日本也参与 但韩国没有参与的这一声明,并不止于担忧

북한은 벌써 핵실험과 대륙간탄도미사일(ICBM) 도발까지 협박하고 있다 The statement, however, is simply an expression‎ of concern as North Korea is already making threats with nuclear tests and ICBM

朝鲜已经开始威胁进行核试验和洲际导弹挑衅

이 모든 게 중국이 감싸주고, 러시아가 거들고, 한국이 뒷짐 지고 있기에 벌어지는 일이다 This behavior is happening as it is encouraged by China, tolerated by Russia and Korea turning a blind eye

这一切都是因为中国包庇 俄罗斯帮助 韩国袖手旁观而发生的

세계 평화와 안전의 보루라는 유엔 안보리가 무력화된 데는 미·중 간 패권경쟁과 미·러 간 군사대치 같은 국제적 대결 정세와도 무관치 않다 The loss of power by the UN Security Council, which has been known as the keeper of international peace and safety, is partially due to the power struggle between the U S and China as well as armed conflict between the U S and Russia

被称为世界和平与安全堡垒的联合国安理会之所以失效,与美中之间的霸权竞争和美俄之间的军事对峙等国际对决局势不无关系

북한도 이런 신(新)냉전 기류에 편승해 한껏 도발을 벌이고 있다 North Korea is certainly taking advantage of this new Cold-War like situation

朝鲜也趁着这种新冷战氛围尽情地展开挑衅

하지만 지금 북한의 핵 질주에 제동을 걸지 못하면 결국 중국에 큰 골칫거리가 될 것이다 However, if we fail to put a stop to North Korea’s continuing nuclear threats, it will eventually develop into a massive problem for China as well

但是如果现在不能阻止朝鲜的核“疾驰”,最终会成为中国的一大难题

시진핑 주석이 굵직한 국제행사를 열 때마다 북한이 핵·미사일 도발로 잔칫상에 재를 뿌렸던 때가 불과 몇 년 전이다 China should not forget North Korea’s past launching of nuclear/missile threats whenever Xi Jinping tried to host major international events just a few years ago

几年前,习近平主席每次举行重大国际活动时,朝鲜都会用核 导弹挑衅在宴桌上撒灰

베이징 올림픽 성화 14년만에 다시 타올라14년 만에 중국 베이징에서 다시 올림픽의 불꽃이 타오른다 Olympic torch lit up in Beijing in 14 years The Olympic torch lights up Beijing in 14 years

北京奥运圣火时隔14年之后将再次被点燃时隔14年,中国北京将再次燃起奥运的火花

4일 오후 9시 열리는 개회식을 시작으로 2022 베이징 겨울올림픽이 17일간의 열전에 돌입한다 Starting with the opening ceremony that will take place at 9 p m on Friday, the 2022 Beijing Winter Games will begin its 17-day celebration of winter sports

以4日晚9点举行的开幕式为开端,2022年北京冬奥会将进入为期17天的激烈角逐

베이징은 올림픽 역사상 최초로 여름, 겨울 대회를 모두 개최하는 도시다 Beijing is the first city in Olympic history to hold both Summer and Winter Games

北京是奥运会历史上第一个同时举办夏季 冬季大会的城市

개회식이 열리는 중국 베이징 국가체육장은 2008년 베이징 여름올림픽 당시 개·폐회식, 육상, 남자축구 결승전 등을 치른 곳이다 Beijing National Stadium, where the opening ceremony is scheduled to take place, is the same stadium that hosted the opening and closing ceremonies of the 2008 Beijing Summer Games and was used as the main track and field stadium and the venue for the men’s 2008 gold medal soccer game

举行开幕式的中国北京国家体育场是2008年北京夏季奥运会当时举行开闭幕式 田径 男子足球决赛的地方

독특한 디자인으로 ‘냐오차오(鳥巢·새 둥지)’로도 불린다 It is known as the “Bird’s Nest” for its distinctive design

因其独特的设计被称为“鸟巢”

이번에는 개·폐회식 외에 따로 경기가 열리지는 않는다 The stadium, however, will not hold other sport events besides the opening and closing ceremonies

但这次除了开闭幕式以外,不会另外举行比赛

총감독 역시 2008년에도 개·폐회식 총감독을 했던 장이머우(張藝謀) 감독이 맡는다 Zhang Yimou, a Chinese film director, will be directing the opening and closing ceremonies as he did in the 2008 Beijing Summer Games

总导演也是2008年开闭幕式总导演张艺谋导演

이번 개회식에는 약 100분간 3000여 명의 공연자가 출연한다 이 중 95%가량은 10대다 Some 3,000 will perform in the opening ceremony that will last about 100 minutes, 95 percent of which will be teenagers

此次开幕式约100分钟,将有3000多名演出者参加其中95%左右是10多岁的年轻人

4시간 동안 열린 2008년 개회식 때는 1만5000여 명이 출연했다 신종 코로나바이러스 감염증(코로나19) 확산과 추운 날씨 등을 감안해 규모를 줄인 것 The opening ceremony for the Winter Games is simpler than that of the 2008 Beijing Olympics, which lasted about four hours with 15,000 performers, because of the COVID-19 pandemic and cold weather

在长达4个小时的2008年开幕式上,当时有1.5万多人参加了演出考虑到新型冠状病毒肺炎(COVID-19)疫情的扩散和寒冷的天气等,缩减了规模

게다가 미국을 비롯한 일부 서방 국가가 외교적 보이콧 의사를 드러내면서 외빈도 줄었다 Moreover, some Western countries including the U S claimed diplomatic boycott of the Beijing Winter Olympics, contributing to a lesser number of foreign missions

再加上美国等部分西方国家表现出外交抵制的意向,外宾也减少了

개최국인 중국의 시진핑 국가주석, 러시아의 블라디미르 푸틴 대통령 등의 참석이 예정돼 있다 Chinese President Xi Jinping of China and Russian President Vladimir Putin are expected to attend the opening ceremony

东道主中国的国家主席习近平 俄罗斯总统普京等将出席开幕式

개회식의 꽃인 최종 점화자, 점화 방식 등은 비밀에 부쳐져 있다 The identity of the final torchbearer and how the torch would be lit up remain confidential

开幕式之火的最终点火者 点火方式等都保密

2008년에는 중국 체조 영웅 리닝(59)이 와이어를 달고 경기장 지붕 안쪽 벽을 타고 달려가 성화대에 불을 붙였다 Back in 2008, Li Ning, a legendary Chinese gymnast, lit cauldron by gliding through the air, suspended on a high-wire

2008年,中国体操英雄李宁(59岁)身上挂着钢丝,沿着赛场屋顶内侧墙壁奔跑,点燃了圣火台

장 감독은 “창의적인 성화 점화 방법으로 사람들을 놀라게 하겠다”고 공언한 상태다 Director Zhang has already claimed that he would employ “a bold idea” to surprise spectators

张导演已经公开表示:“将以创意性的点燃圣火的方法让人们大吃一惊”

성화 연료로 수소를 써온 만큼 친환경 점화 방식에 대한 기대의 목소리도 나온다 Many expect to see eco-friendly way of lighting the cauldron, given that hydrogen has been used as energy source

由于一直使用氢气作为圣火燃料,因此也有人对环保点火方式表示期待

한편 최종 점화자 후보로는 역대 겨울올림픽에서 6개의 메달을 따낸 쇼트트랙 대표 왕멍(37), 2008년 대회 3관왕을 차지했던 체조 대표 저우카이(34) 등이 거론된다 Among candidates for the last torchbearer are Wang Meng, a decorated Chinese short-track speed skater who won six medals at the Olympics and Zou Kai, who won three gold medals at the 2008 Olympic Games transported by freight trains

另外,最终点火者候选人有在历届冬奥会上获得6枚奖牌的短道速滑代表王濛(37岁) 在2008年夏季奥运会上获得3冠王的体操代表邹凯(34岁)等

中대륙 못 도는 성화… Beijing Olympic torch to be carried for only three days

PS:此次北京冬奥会主题口号为“一起向未来”

베이징 인근 사흘만 달려2022 베이징 겨울올림픽 개회에 앞서 중국 전역을 돌아다녀야 할 성화(사진)가 신종 코로나바이러스 감염증(코로나19) 확산 우려로 한껏 움츠러들었다 The Olympic torch for the 2022 Beijing Winter Olympics, which would normally travel across China, will be carried only limitedly due to COVID-19

2022年北京冬奥会圣火传递活动规模大幅缩减,只在北京附近传递三天在2022年北京冬奥会开幕之前,本应辗转于中国全境的圣火(照片)因担心新型冠状病毒肺炎(新型冠状病毒)疫情的扩散而最大限度地“蜷缩”了

지난해 10월 20일 중국 베이징에 도착한 올림픽 성화는 4일 현재 베이징 올림픽타워에 보관돼 타고 있다 The Olympic torch that arrived in Beijing on Oct 20 last year is burning in the Beijing Olympic Tower as of Tuesday

截至4日,去年10月20日抵达中国北京的奥运圣火被保管在北京奥林匹克塔楼内

다음 달 2일부터 3일 동안 주자 1200명이 경기가 열리는 장소인 베이징 중심부부터 베이징 교외인 옌칭구와 허베이성 장자커우만을 달린다 Twelve hundred carriers will run from the center of Beijing to Yanqing District and Zhangjiakou in Hebei province, which are located in the outskirts of the capital city, for three days from February 2

从下月2日开始的3天时间里,将有1200名运动员从比赛举办场地北京中心地带开始,只在北京郊外的延庆区和河北省张家口区域进行圣火传递活动

왕복 거리로 따져도 300km 안팎이다 The round trip distance is only around 300 kilometers

如果算上往返距离,也只有300公里左右

2008년 베이징 여름올림픽 당시에는 주자 2만1880명이 5개 대륙 세계 19개 도시를 포함해 13만7000km를 누볐다 For the 2008 Beijing Summer Olympics, 21,880 runners carried the torch for 137,000 kilometers across 19 cities in five continents around the world

2008年北京夏季奥运会当时,2.188万名运动员跑遍了包括5个大陆世界19个城市在内的13.7万公里

최근 올림픽과 비교해도 봉송 규모가 크게 줄었다 It is also a lot less than the recent Olympics

与最近的奥运会相比,此次传递规模也大幅减少

도쿄 여름올림픽에서 성화는 지난해 3월 25일 후쿠시마를 시작으로 121일간 1만 명의 주자와 함께 일본 열도 2000km를 돌아 도쿄로 돌아왔다 For the Tokyo summer Olympics, the torch had been carried for 2,000 kilometers across Japan by 10,000 runners for 121 days starting from March 25, 2021 in Fukushima

在东京夏季奥运会上,圣火从去年3月25日福岛开始,在121天内与1万名火炬手一起绕行日本列岛2000公里返回东京

2018 평창 겨울올림픽 당시에는 2017년 11월 1일부터 101일 동안 7500명의 주자가 17개 시도와 강원도 시군 전체를 돌아 2018km를 달렸다 For the 2018 Pyeongchang Winter Olympics, 7,500 carriers ran 2,018 kilometers in 17 cities and provinces, as well as all around Gangwon Province, for 101 days from Nov 1, 2017

2018年平昌冬奥会当时,从2017年11月1日开始的101天里,7500名火炬手绕行了17个市道和江原道全市郡,跑了2018公里

홍콩 反中매체 시티즌뉴스 폐간선언Hong Kong’s Citizen News shuts down to protect its staff

香港反华网媒《众新闻》宣布停刊

홍콩의 반중(反中) 온라인 매체 시티즌뉴스가 2일 폐간했다 Hong Kong’s anti-China online news site Citizen News closed on Sunday

香港反华网络媒体《众新闻》(CitizenNews)2日停刊

날로 거세지는 당국의 언론 탄압으로 규모가 큰 언론사조차 최근 속속 폐간을 택하자 더 버티는 것이 불가능하다는 판단을 한 것으로 풀이된다 It appears the new site decided to cease operations after even large media outlets shut down one after another due to the growing suppression of the press from the authorities

分析认为,由于当局日益严厉地镇压媒体,最近连规模较大的媒体也陆续选择停刊,因此报纸认为不可能再坚持下去

지난해 6월 당시 홍콩 최대 일간지인 핑궈일보, 지난해 12월 29일 유명 온라인 매체 리창신문에 이어 약 반년 사이에 세 곳의 반중 언론이 문을 닫으면서 홍콩의 언론 자유가 사실상 사라졌다는 우려가 나온다 There are growing concerns that the freedom of press has virtually disappeared in Hong Kong as three anti-China media outlets shut down in the past half a year following the closures of Apple Daily, the largest daily newspaper in Hong Kong, in June, and Stand News on Dec 29

继去年6月香港最大的日报《苹果日报》和去年12月29日著名网媒《立场新闻》之后,大约半年内有3家反华媒体关门,有人担心香港的言论自由实际上已经消失

시티즌뉴스는 2일 페이스북에서 “위기의 시기에 배에 탄 모든 이의 안전을 우선 보장해야 한다 무거운 마음으로 폐간을 발표한다”고 이유를 밝혔다 On its official Facebook account, Citizen News made the announcement “with a heavy heart,” saying they must ensure the safety of everyone who are on board in a time of crisis

《众新闻》2日在脸书上写道:“在危机时期,首先要保障船上所有人的安全

저널리즘의 정신을 계승해 대중에 봉사하고 싶었지만 최근 2년간 홍콩 사회의 변화와 언론 환경의 악화로 시티즌뉴스라는 작은 배가 강한 바람과 파도에 부딪혔다고도 설명했다 It went on to say that they wanted to serve the public by inheriting the spirit of journalism, but due to changes in Hong Kong and worsening environment for media in the past two years, a small boat called Citizen News was hit by strong winds and waves

以沉重的心情宣布停刊”报方解释称,虽然想继承新闻业的精神,为大众服务,但近两年来香港社会的变化和舆论环境的恶化,使《众新闻》这条小船遭遇强风和波涛汹涌

설립자 겸 편집국장인 크리스 융은 3일 기자회견을 열고 “폐간 결정은 짧은 기간 안에 이뤄졌다 우리가 위험에 노출될 수도 있다는 점을 배제할 수 없었다”고 밝혔다 In a press conference on Monday, Citizen News’ founder and chief writer Chris Yeung said the decision to close Citizen News was made in a short time, adding they could not rule out that they might be exposed to some risks

创办人兼总编克里斯·杨(杨健兴)3日召开记者会表示:“停刊决定是在短时间内完成的不能排除我们可能会面临危险”

특히 결정적인 폐간 계기는 불과 4일 전 폐간한 리창신문의 선택이었다고도 했다 In particular, he said that the announcement made four days ago by Stand News to cease operations was the decisive reason to decide the closure

尤其是,其决定停刊的原因,是《立场新闻》4天前选择停刊

핑궈일보와 리창신문은 모두 폐간 직전 전현직 간부가 줄줄이 체포되고 회사 자산까지 동결되자 폐간을 택했다 Both Apple Daily and Stand News decided to shut down after former and incumbent executives were arrested one after another and company assets were frozen

在《苹果日报》和《立场新闻》停刊前,其前任和现任干部接连被捕,公司资产也被冻结,因而都选择了停刊

구성원에게 이런 일을 겪게 할 수 없었다는 뜻으로 해석된다 It appears Citizen News made the decision in order to protect its staff

分析认为,其意思是不能让成员经历这样的事情

시티즌뉴스는 2017년 1월 1일 창간한 직원 40명 규모의 소규모 온라인 매체다 Founded on Jan 1, 2017, Citizen News is a small online media outlet with 40 staff

《众新闻》是2017年1月1日创刊的40名职员规模的小型在线媒体

자유, 개방성, 다양성, 포용성 등을 추구하며 2019년 범죄인 인도법(송환법) 반대 및 2020년 홍콩 국가보안법 반대 시위에서 민주 진영의 목소리를 충실히 전했다 In pursuit of freedom, openness, diversity and inclusiveness, Citizen News faithfully delivered the voice of democracy during protests against the Hong Kong extradition bill in 2019 and Hong Kong security law in 2020

它追求自由 开放性 多样性 包容性,在2019年反对罪犯引渡法(遣返法)及2020年反对香港《国家安全法》的示威中,忠实地传达了民主阵营的声音

G2 암울한 새해 전망…中, 인도에 밀리고 美, 코로나에 치이고Dark clouds cast gloomy shadows on G2 economies

G2暗淡的新年展望:中国不及印度,美国被新冠疫情所困扰

세계 패권국 지위를 놓고 대립하고 있는 미국과 중국이 올해 나란히 힘든 한 해를 겪을 것이라고 주요 외신이 일제히 보도했다 The year of 2022 will place a burden on the shoulders of the top two global powerhouses in the never-ending fierce competition for leadership – the United States and China, according to major overseas news reports

主要外媒一致报道,就世界霸权国家地位对立的美国和中国今年将双双经历艰难的一年

중국은 신종 코로나바이러스 감염증(코로나19)에 따른 강경 봉쇄 정책, 부동산 부실, 전력난 등의 여파로 경제 성장이 대폭 둔화될 위험이 있다고 영국 텔레그래프가 지난해 12월 31일 보도했다 The Telegraph, a British newspaper, analyzed on Friday that China is likely to suffer a severe economic slowdown due to the consequences of tight border control following the COVID-19 pandemic, poor housing market conditions and power shortages

据英国《每日电讯报》去年12月31日报道,中国受新冠疫情带来的强硬封锁政策 房地产亏损 电力短缺等影响,经济增长存在大幅放缓的危险

미국 역시 코로나19 확산세, 인플레이션, 공급망 위기와 물류대란, 국제사회에서의 지도력 저하 등의 문제에 직면했다 Likewise, the United States has a hard time handling the spread of the virus, inflation, a supply chain crisis, a deadlock situation in logistics and decrease of global leadership

美国也面临着新冠疫情扩散趋势 通货膨胀 供应链危机和物流大乱 国际社会领导力低下等问题

집권 2년 차를 맞은 조 바이든 미 행정부 또한 ‘지뢰밭’에 직면했다고 미 정치매체 더힐이 1일 평가했다 This year is expected to be a “minefield” for U S President Joe Biden who has just started his second year at the White House, said the Hill, a U S politics magazine, on Saturday

美国政治媒体《国会山》1日评价说,迎来执政第二年的拜登政府也面临“地雷阵”

○ 中 성장률, 46년 만에 美에 뒤질 듯（（no translation））

○中国经济增长率将46年来首次落后于美国

일본 투자은행 노무라증권은 올해 중국 경제가 4. 3% 성장할 것으로 전망했다 미국 성장률 전망치는 이보다 0. 3%포인트 높은 4. 6%로 제시했다 Nomura Holdings Inc , a Japanese investment bank, expected the U S economy to grow 4 6 percent this year, 0. 3 percentage points up from the Chinese economic growth of 4. 3 percent as projected

日本投资银行野村证券预测,今年中国经济将增长4.3%美国经济增长率预测值为4.6%,比预测值高出0.3个百分点

세계은행 통계에 따르면 미국이 중국보다 높은 성장률을 기록하는 것은 1976년 이후 46년 만에 처음이다 The United States is expected to grow faster than China in 46 years, which is the first time since 1976

据世界银行统计,美国创下高于中国的增长率是自1976年以后46年来的第一次

중국의 경제 성장률은 1991년부터 2018년까지 약 30년간 연 6% 이하로 내려간 적이 없다 노무라증권은 확진자가 단 1명만 나와도 도시 전체를 봉쇄하는 중국 특유의 ‘제로(0) 코로나’ 정책이 장기화할수록 이에 따른 경제적 악영향이 클 것으로 내다봤다 （no translation）

中国的经济增长率从1991年到2018年的约30年间从未降至6%以下野村证券预测,只要出现一名确诊患者就要封锁整个城市的中国特有的“零新冠”政策越是长期化,带来的经济负面影响就越大

텔레그래프는 ‘떠오르는 거인’ 인도는 중국보다 약 2배 높은 8. 5% 성장이 예상된다며 인도 경제가 중국을 제치고 오랫동안 고성장을 구가할 것으로 내다봤다 The Telegraph projected that India, a “rising giant,” will grow by 8. 5 percent double China’s rate, predicting that the Indian economy will enjoy a long-lasting economic bonanza surpassing the Chinese economy

《每日电讯报》预测,“崛起的巨人”印度的经济增长率将达到8.5%,约为中国的2倍,印度经济将超越中国,长期保持高增长

프랑스 악사자산운용 역시 ‘세계의 공장’으로 불리는 중국의 생산 능력이 심각한 타격을 받을 것으로 내다봤다 AXA Investment Managers, a French investment management firm, also shared a pessimistic view of the production capabilities of China, the “world’s factory ”

法国乐师资产管理公司也预测,被称为“世界工厂”的中国生产能力将受到严重打击

중국은 한국의 최대 수출국일뿐 아니라 중국 현지에 생산 공장을 둔 한국 기업도 많기에 한국 경제 또한 악영향이 불가피할 것으로 보인다 The South Korean economy will be inevitably affected by a slowing Chinese economy not only because it is South Korea’s top export destination but also because it is home to many South Korean production facilities

中国不仅是韩国的最大出口国,在中国当地设立生产工厂的韩国企业也很多,预计韩国经济也将不可避免地受到负面影响

대만을 둘러싼 미국과의 갈등 격화 또한 우려를 낳고 있다 （no translation）

中国与美国围绕台湾的矛盾激化也令人担忧

시진핑(習近平) 중국 국가주석은 12월 31일 관영 중국중앙(CC)TV 생중계로 발표한 신년사에서 “조국의 완전한 통일은 양안(중국과 대만) 동포의 공통된 염원”이라며 대만을 압박했다 （no translation）

中国国家主席习近平在12月31日通过中国中央电视台现场直播发表的新年贺词中表示,“实现祖国的完全统一是两岸同胞的共同愿望,”对台湾施压

이날 그는 지난해 7월 열린 중국 공산당 100주년 기념식 사진들을 배경으로 놓고 신년사를 발표했다 （no translation）

当天,他发表了以去年7月召开的中国共产党100周年纪念仪式照片为背景的新年贺词

중국 군용기 또한 새해 첫날인 1일 오전 8시경 대만 방공식별구역(ADIZ)에 진입했다 （no translation）

中国军用飞机也在新年第一天1日上午8点左右进入了台湾防空识别区

이에 차이잉원(蔡英文) 대만 총통 또한 같은 날 페이스북 생중계 연설에서 “중국이 상황을 오판하지 말고 군사적 모험주의가 내부에서 확장되는 걸 막도록 일깨워줘야 한다”고 맞섰다 （no translation）

对此,台湾领导人蔡英文也在同一天脸书直播演讲中针锋相对地表示:“必须提醒中国,不要误判形势,防止军事冒险主义在内部扩张”

○ 집권 2년 차 바이든 ‘지뢰밭길’ 암울 최근 코로나19 일일 신규 확진자가 연일 사상 최고치를 경신하고 있는 미국에서는 새해 첫날에도 항공 대란이 이어지는 등 교통, 행정 기능이 타격을 받고 있다 （no translation）

○执政第二年的拜登面临暗淡的“地雷阵”美口最近日增新冠确诊患者连日刷新历史最高纪录,新年第一天还接连发生航空大乱等,交通 行政功能受到打击

항공편 추적 사이트 플라이트어웨어에 따르면 코로나19 감염으로 인한 조종사 부족, 폭설 등으로 1일 총 2655편의 운항이 취소됐다 （no translation）

据航班追踪网站“航班跟踪”透露,受新冠疫情导致飞行员不足 暴雪等影响,1日共取消了2655个航班

더힐은 이날 바이든 대통령이 코로나19와 오미크론 변이 확산, 전염병 대유행이 의료 및 금융체계에 미치는 여파, 인플레이션 등을 해결해야 한다고 지적했다 The Hill also pointed out that U S President Joe Biden should resolve the ramifications of pandemics including the ongoing situations with COVID-19 and the Omicron variant on medical and financial systems as well as inflation rates

《国会山》当天指出,拜登应该解决新冠疫情和奥密克戎变异毒株的扩散 传染病大流行对医疗及金融体系的影响 通货膨胀等问题

최근 그의 국정수행 지지율은 30∼40%대로 취임 후 최저 수준을 기록하고 있다 He has recently recorded the lowest approval rates in the 30-40 percent ranges since his inauguration

最近,拜登的国政支持率为30~40%,创下了就任以来的最低水平

집권 민주당의 선거전략가 조엘 페인은 “바이든은 코로나19 덕분에 대통령이 됐지만 이제는 코로나19 때문에 힘든 처지에 놓였다 그의 운은 코로나19와 함께 간다”고 했다 국내외 위험 요인도 많다 The ruling Democratic Party’s Democratic election strategist Joel Payne was quoted as saying, “Joe Biden is president because of COVID, but Dems are struggling right now because of COVID And until they can find someone to figure this out, people are going to be mad about COVID

执政的民主党的选举战略家乔尔·佩恩表示:“拜登得益于新冠疫情成为总统,但现在又因为新冠疫情处境艰难他的运气是伴随新冠疫情的”国内外危险因素也很多

바이든 대통령은 지난해 1월 도널드 트럼프 당시 대통령의 지지자가 트럼프의 대선 패배에 불복하며 의회에 난입한 사건이 발생한 지 1년이 되는 6일 대국민 연설을 하겠다고 예고했다 （no translation）

拜登预告,将在去年1月时任总统特朗普的支持者不服特朗普大选失利 闯入国会一年后的6日发表对国民演讲

그러자 트럼프 전 대통령 또한 같은 날 ‘맞불 회견’을 열겠다고 밝혔다 （no translation）

随后,特朗普也表示,将在同一天举行“针锋相对的记者会”

전현직 대통령의 충돌이 미국의 분열과 양극화를 심화시키고 있다는 우려가 높다 （no translation）

很多人担心前任 现任总统的冲突正在加剧美国的分裂和两极化

러시아의 우크라이나 침공 위협이 높아지고 있다는 지적이 나오는 가운데 바이든 대통령은 미 동부시간 2일 볼로디미르 젤렌스키 우크라이나 대통령과 통화해 우크라이나 영토 보전에 대한 의지를 재확인할 예정이라고 백악관이 밝혔다 （no translation）

有人指出,俄罗斯入侵乌克兰的威胁正在加大这种情况下,拜登将于美国东部时间2日与乌克兰总统泽连斯基通电话,再次确认保卫乌克兰领土的决心

바이든 대통령은 지난해 12월 30일 블라디미르 푸틴 러시아 대통령에게 “러시아가 우크라이나를 침공하면 가혹하게 제재할 것”이라고 경고했다 （no translation）

拜登去年12月30日向俄罗斯总统普京警告:“如果俄罗斯入侵乌克兰,将严厉制裁”

中, 한미 가까워질수록 보복할 것…한국이 치러야할 불가피한 대가”

郑义溶:“南北关系难以以北京奥运会为契机取得改善”

“한국이 미국에 가까워질수록 중국은 한국에 보복할 것이다 （no translation） 안타깝지만 이건 한국이 치러야 하는 피할 수 없는 대가다 （no translation）

韩国外交部长郑义溶表示:“原希望北京奥运会成为改善南北关系的契机,但从目前来看,这种期待实际上已变得越来越困难”

” 국제정치학계 분야의 대표적 석학인 존 미어샤이머 미국 시카고대 석좌교수(75·사진)는 최근 동아일보와의 신년 인터뷰에서 미중 갈등에 대해 “중국이 더 강력해질수록 한국의 안보 위협은 커질 것”이라며 “누가 (한국의) 대통령이 되든 한국과 미국이 어떤 관계를 맺을지가 가장 중요한 문제”라고 강조했다 ” ‘Beijing likely to retaliate for closer Seoul-Washington ties’ ohn J Mearsheimer (75), an emeritus professor of the University of Chicago and authority in international politics, had New Year’s interview with The Dong-A Ilbo “The stronger China becomes, the bigger security threat to Korea will be,” he said, commenting on conflict between Washington and Beijing “Whoever becomes the next South Korean president, what kind of ties South Korea and the U S will forge is the most important matter

（漏译）

시진핑(習近平) 중국 국가주석의 3연임으로 장기집권 체제를 다진 중국이 미국을 넘어서는 패권국이 되려고 시도하는 과정에서 한국을 향한 압박이 더욱 거세질 것이라는 얘기다 ” As Mearsheimer said as Chinese President Xi Jinping consolidated his long-term control of government by securing his three-consecutive terms, and thus Beijing seeks to become a hegemony exceeding Washington’s power and influence, it will increase pressure on South Korea

（漏译）

가장 영향력 있는 현실주의 이론가인 미어샤이머 교수는 미중 패권 경쟁을 가장 정확하게 예측한 인물로 꼽힌다 Mearsheimer, who is the most influential realistic theorist, is considered the expert who has most accurately predicted hegemonic competition between Washington and Beijing “

（漏译）

미어샤이머 교수는 “세계는 ‘2차 냉전’에 돌입하고 있다”며 “중국은 곧 미국과 동등한 힘을 갖게 되고, 앞으로 30년간 경제 성장을 이어간다면 미국을 제치고 가장 강력한 국가가 될 것”이라고 내다봤다 그는 또 “미중이 15년 이내에 대만을 두고 전쟁을 벌일 가능성이 높다고 본다”고 전망했다 The world is entering a second Cold War,” Mearsheimer said “China will have power on par with the U S soon, and if it continues economic growth over the next 30 years, it will become the most powerful country in the world, surpassing the U S There is a strong chance that the U S and China will stage a war over Taiwan within the next 15 years

（漏译）

특히 미어샤이머 교수는 한국의 이른바 ‘안미경중(안보는 미국, 경제는 중국)’ 외교에 대해 “한국이 한미 동맹에 전념하지 않는 것은 ‘어리석음의 극치(height of foolish)’가 될 것”이라고 강조했다 ” “Committing on South Korea’s so called ‘diplomacy of security with the U S and diplomacy of economy with China,” Mearsheimer said it would be ‘height of foolish’ for “South Korea not to concentrate on the Seoul-Washington alliance

（漏译）

사드(THAAD·고고도미사일방어체계) 사태에서 볼 수 있듯이 중국의 위협이 커질수록 한국은 미국과 안보 협력을 통한 생존을 선택할 수밖에 없다는 것 ” As evidenced by the Terminal High-Altitude Missile Defense system (THAAD), the stronger China’s threat becomes, the more South Korea will have to seek its survival through security cooperation with Washington

（漏译）

그는 “한국과 일본이 긴밀히 협력하면 중국의 위협에 더 잘 대응할 수 있을 것”이라고 조언했다 “If South Korea and Japan closely cooperate with each other, they will be able to better cope with China’s threat,” he advised

（漏译）

정의용 “베이징올림픽 계기 남북관계 개선 어려워져”정의용 외교부 장관이 “베이징 올림픽을 남북관계 개선의 계기로 삼길 희망했지만 현재로선 그런 기대가 사실상 어려워지고 있다“고 밝혔다 정 장관은 29일 서울 외교부 청사에서 열린 기자간담회에서 내년 2월 베이징 겨울올림픽에서 남북 정상회담 가능성 등을 묻는 질문에 이같이 말했다Foreign minister: Improving inter-Korean ties at Beijing Olympics seems unlikely South Korea’s Foreign Minister Chung Eui-yong said the country hoped that the Beijing Winter Olympics would serve as an opportunity to improve the inter-Korean ties, but now such expectation is unlikely to be met. Foreign Minister Chung told reporters at a press briefing held at the Foreign Ministry building in Seoul on Wednesday when asked about the possibility of an inter-Korean summit at the upcoming Beijing Winter Olympics in February

郑义溶29日在首尔外交部大楼举行的记者招待会上,就明年2月在北京冬奥会上举行南北首脑会晤的可能性等问题做出了上述回答受新冠疫情影响,朝鲜能否参加奥运会尚不明确在这种情况下,再加上美国还宣布进行外交抵制,因此他承认难以把冬奥会作为终战宣言的跳板

신종 코로나바이러스 감염증(코로나19) 여파로 북한의 올림픽 참석이 불투명한 가운데 미국도 외교적 보이콧을 선언한 상황에서 올림픽을 종전선언 등을 위한 발판으로 삼기 힘들다고 인정한 것 Under the circumstances where North Korea’s participation in the Olympics is unclear due to the spread of COVID-19 and the U S has announced a diplomatic boycott of the Beijing Olympics, Chung acknowledged that the Olympics will not likely to be a foothold for an end-of-war declaration

（漏译）

다만 정부 고위관계자는 “정 장관의 발언은 ‘올림픽’을 평화 프로세스에 활용하기 어려워졌다는 취지”라며 “오히려 올림픽 전이라도 남북관계에서 큰 진전이 있을 수 있는 것”이라고 전했다 However, a high-ranking government official said Foreign Minister Chung’s remarks mean that it has become difficult to leverage the Olympics to advance the peace process, adding there could be improvement of inter-Korean relations even before the Olympics

不过,政府一名高层官员表示:“郑义溶此话的意思,是难以把‘冬奥会’用于和平进程但是,反而是在冬奥会之前,南北关系也有可能取得重大进展”

정 장관은 베이징 올림픽 외교적 보이콧 관련해선 “검토하지 않고 있다”며 기존 입장을 확인했다 As for a possible boycott of the Beijing Olympics, Chung reiterated his position that Seoul is “not reviewing” a diplomatic boycott of the Olympics

对于外交抵制北京奥运会,郑义溶表示“没有考虑”,确认了原有的立场

문재인 대통령의 참석 가능성이 열려 있느냐는 질문에는 “현 단계에서 공유할 내용이 없다”고 말을 아꼈다 When asked if President Moon is open to attending the Olympics, Chung said he has nothing to share at the moment

对于“文在寅总统是否有可能出席冬奥会”的提问,他只是简单地回答说:“现阶段没有可以分享的内容”

청와대 내부에선 현재로선 문 대통령의 올림픽 참석이 어렵지 않겠느냐는 기류가 강한 것으로 알려졌다 It is known that the majority of opinion within Cheong Wa Dae is President Moon’s participation in the Olympics will be difficult for now

据悉,青瓦台内部强烈认为,从目前来看,文在寅难以参加冬奥会

정 장관은 한미가 조율 중인 종전선언 문안에 대해선 “사실상 합의가 돼 있는 상태”라고 밝혔다 “조율이 거의 끝난 상태”라던 기존 입장보다 한발 나아간 것 As for an end-of-war declaration, Chung said South Korea and the U S have effectively agreed on the draft, a step up from the previous announcement that talks with the U S are in final stages

郑义溶就韩美目前正在协调的《终战宣言》草案表示:“实际上已经达成协议”

또 “북한과 (종전선언 관련) 협의를 어떻게 진전시켜야 할지 검토하고 있다”며 “북한의 구체적인 반응이 있기를 기대하고 있다”고도 했다 He went on to say that Seoul is reviewing how to advance talks with Pyongyang over end-of-war declaration, adding he expects a concrete response from North Korea

这比“协调工作几乎已经结束”的原先立场更进了一步他还表示:“正在讨论如何与朝鲜进行(有关终战宣言的)谈判希望朝鲜做出具体回应”

정 장관은 일본군 위안부 문제 관련해선 “원죄가 어디 있는지 여러분이 잘 아시지 않느냐”며 과거사 문제로 인한 한일 관계 경색에 대한 책임을 일본 정부에 돌렸다 （no translation）

郑义溶就日军“慰安妇”问题表示,“大家不是很清楚原罪在哪里吗”将因历史问题导致韩日关系恶化的责任推给了日本政府

그러면서 “일본이 끝까지 우리가 2015년 (위안부) 합의를 그대로 지켜야 한다는 입장을 아주 완강하게 고수하고 있어 (한일 관계가) 전혀 진전을 이루지 못하고 있다”고도 했다 （no translation）

他还表示:“日本顽强地坚持我们要遵守2015年(慰安妇)协议的立场,(韩日关系)完全没有进展”

최근 일본이 조선인 강제노역 현장인 사도(佐渡) 광산을 세계문화유산에 등재하려는 움직임과 관련해선 “깊이 우려하고 깊은 유감을 표한다”고 비판했다 （no translation）

对于日本最近打算将朝鲜人强制劳役场——佐渡矿山载入世界文化遗产一事,郑义溶抨击说:“深表忧虑,深表遗憾”

정 장관은 우리 정부가 북한, 중국의 인권 문제에만 눈 감고 있다는 지적에 대해서는 “북한, 중국과는 특수한 관계에 있고 우리 안보와 직결돼 협력할 부분이 많기 때문”이라고 설명했다 When it comes to the criticism that Seoul is turning a blind eye to humanitarian issues in North Korea and China, Chung said South Korea is in special relations with North Korea and China, and there are many areas to cooperate with them as the two are directly related to the country’s national security

有人指出,韩国政府只对朝鲜和中国的人权问题视若不见对此,郑义溶解释说:“这是因为韩国与朝鲜和中国有着特殊的关系,而且与韩国安保直接相关,需要合作的部分很多”

삼성전자 “中시안 공장, 코로나 확산에 탄력운영”（（no translation））

三星电子:“中国西安工厂因新冠疫情扩散实行弹性运营”

신종 코로나바이러스 감염증(코로나19) 확산으로 중국 산시성 시안(西安)이 사실상 봉쇄된 가운데 삼성전자의 현지 반도체 생산이 일부 차질을 겪고 있다 Samsung adjusts operations at its Xi’an chip plant amid Samsung’s chip factory in Xi’an, China saw disruptions in production as the city is under a strict lockdown due to the spread of COVID-19

由于新型冠状病毒肺炎(COVID-19)疫情的扩散,中国陕西省西安市实际上已经被封锁,在这种情况下,三星电子在当地的半导体生产出现了一些问题

삼성전자는 29일 자사 뉴스룸을 통해 “중국 시안 반도체 사업장은 코로나19 확산세가 지속됨에 따라 생산라인의 탄력적 조정을 진행 중”이라고 밝혔다 Samsung Newsroom said on Wednesday that the company has decided to temporarily adjust operations at its manufacturing facilities in Xi’an, China due to the ongoing COVID-19 situation

三星电子29日通过本公司新闻室表示:“中国西安半导体事业场随着新冠疫情的持续扩散,正在进行生产线的灵活调整”

탄력적 조정은 반도체 생산이 평시보다 적은 수준으로 낮춰진다는 걸 뜻한다 The adjustment of operations means reducing chip production than usual

灵活调整意味着半导体生产将降到比平时低的水平

삼성전자는 “임직원의 안전과 건강을 최우선으로 고려해야 한다는 회사의 경영 방침에 따른 것”이라고 덧붙였다 “The decision was made in accordance with our commitment to protecting the health and safety of our employees and partners, which remains our top priority,” added Samsung Electronics

三星电子补充说:“这是根据公司的经营方针做出的决定,即应优先考虑员工的安全和健康”

산시성 성도이자 인구 1300만 명의 대도시인 시안은 이달 9∼22일 코로나19 신규 확진자가 206명 발생하면서 주민 외출이 전면 금지됐다 China locked down 13 million people in Xi’an, Shaanxi province after the city recorded 206 new COVID-19 cases between Dec 9 and Dec 22

作为陕西省省会 人口达1300万的大城市,西安市在本月9日至22日新增206例新冠病毒感染病例,政府随即实行全面禁止居民外出的防疫措施

중국 당국이 확진자가 1명이라도 생길 경우 해당 지역 전체를 봉쇄하고 주민 모두를 격리하는 정책을 시행하고 있기 때문이다 This is because Chinese authorities are placing a lockdown and isolating all residents if there is one confirmed COVID-19 case in the region

因为,中国当局正在实施只要出现1名感染病例,就封锁整个地区并隔离所有居民的政策

삼성전자는 그동안 중국 정부의 특별조치를 받아 공장을 정상 가동해 왔다 Samsung Electronics has been operating its production facilities normally under special measures from the Chinese government

三星电子此前适用中国政府的特别措施,工厂一直正常运转

하지만 지역 봉쇄가 길어지면서 임직원 출퇴근은 물론 물류까지 차질이 빚어지자 생산량 조절에 나섰다 However, the South Korean IT giant had to adjust its operations there as employees’ commuting efforts and logistics faced disruptions due to a prolonged lockdown

但是,随着地区封锁时间的延长,不仅是员工上下班,连物流也受到了影响,因此开始调节生产量

시안 반도체 공장은 삼성전자의 유일한 해외 메모리반도체 공장으로 2014년부터 낸드플래시를 생산하고 있다 The chip plant in Xi’an is Samsun Electronics’ only overseas memory chip plant and has been producing NAND flash memory chips since 2014

西安半导体工厂是三星电子唯一的海外存储半导体工厂,从2014年开始生产闪存芯片(NAND)

낸드플래시는 모바일 기기의 데이터 저장소뿐 아니라 서버, PC 등에서 데이터 저장에 필요한 솔리드스테이트드라이브(SSD) 제조에 쓰인다 NAND flash is used not only for data storage but also for manufacturing solid state drives (SSD) required for data storage in servers and PCs

闪存芯片不仅是移动设备的数据存储场所,还用于在服务器 PC等储存数据所需的固态硬盘(SSD)的制造

삼성전자의 낸드플래시 세계 시장 점유율은 40%가량인데, 삼성전자 전체 생산량 중 약 40%가 시안 공장에서 만들어지고 있다 Samsung Electronics has almost 40% share of the global NAND flash market and the Xi’an plant accounts for over 40% of the company’s total NAND flash production

三星电子在闪存芯片世界市场的占有率为40%左右,但在三星电子总产量中,约40%是在西安工厂生产

한편 시안에 있는 삼성SDI의 전기차용 배터리 공장은 현재 정상 가동 중이다 （no translation）

另外,位于西安的三星SDI的电动汽车电池工厂目前正常运转

삼성SDI는 코로나19 확산 정도와 중국 정부 조치에 따라 향후 공장 운영 방향을 결정할 계획이다 （no translation）

三星SDI将根据新冠疫情的扩散程度和中国政府的措施,决定今后的工厂运营方向

美 내년1분기 성장률 전망 5.2% → 2.2% U S growth forecast in Q1 2022 lowered from 5 2% to 2 2%

美国明年第一季度经济增长率预测值从5.2%下调至2.2%

신종 코로나바이러스 감염증(코로나19) 새 변이인 오미크론이 급속도로 퍼지면서 미국과 중국 등 세계 경제에 충격이 현실화될 조짐이다A fast spread of the Omicron variant is expected to hit the global economy, including the U S and China

随着新型冠状病毒肺炎(COVID-19)新变异毒株“奥密克戎(Omicron)”的迅速扩散,美国和中国等世界经济将势必受到不小冲击

27일 월스트리트저널(WSJ)에 따르면 각국의 경제 전문가들은 내년도 미국과 전 세계의 경제 전망을 하향 조정하고 있다 According to the Wall Street Journal on Tuesday, economic experts from around the world are lowering their growth forecast for the global economy, including the U S next year

据《华尔街日报》27日报道,各国经济专家正在下调明年美国和全世界的经济预测

무디스애널리틱스의 마크 잰디 수석이코노미스트는 내년 1분기(1∼3월) 미국의 국내총생산(GDP) 증가율을 기존의 5.2%(연율)에서 2.2%로 낮췄다 Mark Zandi, chief economist at Moody’s Analytics, downgraded his Q1 U S gross domestic product (GDP) forecast from 5.2% to 2.2%

穆迪分析公司首席经济学家马克?詹迪将明年第一季度(1~3月)美国国内生产总值(GDP)增长率从原来的5.2%(年率)下调至2.2%

그는 최근 항공대란에 따른 여행 감소와 스포츠 경기, 브로드웨이 공연 중단 등을 거론하며 “올여름 델타 변이 확산 때와 매우 비슷한 현상이 벌어지고 있다”고 진단했다 Citing reasons, such as less travel and cancellations of sporting events and Broadway shows, Zandi said the situation is very similar to the past summer, when Delta hit

他提到了最近发生的航空大乱导致的旅游减少 体育比赛和百老汇公演中断等,并诊断说:“正在出现与今年夏天德尔塔变异毒株扩散时非常相似的现象”

경제 연구기관인 판테온 매크로이코노믹스도 내년 1분기 미국의 성장률 전망치를 종전의 5%에서 3%로 낮췄다 Pantheon Macroeconomics also lowered its growth forecast for the U S in Q1 next year from 5% to 3%

经济研究机构万神殿宏观经济研究所也将明年第一季度美国经济增长率预测值从之前的5%下调到了3%

최근 세계은행(WB) 역시 중국의 내년 성장률 전망치를 5.4%에서 5.1%로 낮췄다 The World Bank recently cut its forecast for China’s economic growth next year from 5.4% to 5.1%

最近,世界银行(WB)也将中国明年的经济增长率预测值从5.4%下调到了5.1%

오미크론 변이가 봉쇄 조치의 장기화로 이어지면서 경제 활동이 내년에도 계속 위축될 것으로 예상했다 It predicted that the economy will continue to shrink next year as the spread of Omicron variant could lead to longer-lasting restrictions

据预测,随着奥密克戎变异毒株导致封锁措施的长期化,经济活动明年也将继续萎缩

독일의 중앙은행인 분데스방크도 독일의 내년 성장률을 6월에 전망한 5.2%에서 4.2%로 하향 조정했다 The Bundesbank, Germany’s central bank, also downgraded its economic growth forecast for Germany next year from 5.2% to 4.2%

德国中央银行德意志银行也将德国明年的增长率从6月预测的5.2%下调至4.2%

리그도 연기한 NHL, 베이징 겨울올림픽 안간다2022 베이징 겨울올림픽 흥행에 ‘빨간불’이 켜졌다 겨울올림픽의 꽃이자 최고 흥행 종목으로 꼽히는 아이스하키에 북미아이스하키리그(NHL) 선수들이 불참하기 때문이다 NHL players to skip Beijing Olympics due to COVID-19 The U S National Hockey League’s announcement that its players will not participate in the 2022 Beijing Olympics has poured cold water on the excitement for the winter games slated to be held next year Ice hockey is one of the most popular winter team sports, dubbed as the “flower of winter sports

联赛延期的NHL,大概率不参加北京冬奥委会2022年北京冬奥会票房亮起了“红灯”因为既是冬季奥运会之花,又是最受欢迎项目的冰球项目,北美职业冰球联盟(NHL)的选手们将不参加

미국 스포츠전문매체 ESPN은 22일 NHL 노사가 베이징 올림픽에 참가하지 않기로 합의했다고 보도했다 ” American sports channel ESPN reported Monday that the NHL and the NHL Players’ Association agreed not to participate the Beijing Olympic Games, amid rising concerns of the worsening coronavirus pandemic

美国体育专门媒体《ESPN》22日报道说,NHL劳资双方就不参加北京冬奥会成了协议

NHL의 올림픽 참가의 발목을 잡은 것은 신종 코로나바이러스 감염증(코로나19)의 확산 때문이다 NHL은 코로나19 오미크론 변이 확산으로 2021∼2022시즌 정규시즌 50경기를 연기했다 크리스마스 연휴에만 사흘간 쉬려고 하다가 이틀을 더해 22일부터 26일까지 리그를 중단하기로 했다 The NHL has already postponed 50 games in 2021-2022 regular season due to the spread of the Omicron variant of COVID-19, which is a change of the initial plan of taking a three-day Christmas holiday break to a pause of the regular season from Wednesday to Sunday

阻碍NHL参加奥运会的原因是新型冠状病毒肺炎(COVID-19)疫情的扩散NHL因新冠病毒奥密克戎(Omicron)变异毒株的扩散,推迟了2021～2022赛季常规赛50场比赛本想在圣诞节休假三天,但又决定从22日到26日中断联赛

ESPN은 “NHL은 내년 1월 10일까지 올림픽 불참을 결정하면 벌금 등의 페널티를 피할 수 있다”며 “조만간 불참과 관련한 공식 발표를 할 예정”이라고 전했다 ESPN reported that the NHL will soon publicly announce its decision on Olympic participation in 2022 as it can opt out of Olympic participation without financial penalty if it makes a decision on participation until Jan 10 next year

ESPN称:“如果NHL在明年1月10日之前决定不参加冬奥会,就可以避免罚款等处罚”

NHL은 1998년 나가노부터 2014년 소치까지 모든 올림픽에 나섰다 The NHL has participated in every Games from the 1998 Olympics in Nagano, Japan to the 2014 Olympics in Sochi, Russia,

"NHL计划不久后正式公布不参加奥运会的相关消息”NHL从1998年的长野到2014年的索契,参加了历届所有的冬季奥运会

하지만 2018년 평창 올림픽은 경제적으로 이득이 없고, 빠듯한 리그 일정 등을 이유로 불참했다 but it didn’t participate in the 2018 Pyeongchang Olympics, citing little economic benefit and a tight schedule

但是,2018年平昌冬奥会因经济上无法获益 联赛日程紧张等原因没有参加

NHL은 13억 인구를 지닌 거대 중국 시장을 고려해 베이징 올림픽은 참가하겠다고 했지만 결국 두 올림픽을 건너뛰고 2026년 밀라노-코르티나담페초 올림픽에 참가하게 됐다 The League initially announced that it would participate in the 2022 Beijing Olympics, a decision which appeared to be conscious of the 1 3 billion Chinese market, but it has reworked the plan and decided to skip the Beijing Olympics and go to the 2026 Milano Cortina Olympics

NHL考虑到拥有13亿人口的巨大的中国市场,表示要参加北京冬奥会,但最终决定连续两届缺席冬奥会,只参加2026年的米兰-科尔蒂纳丹佩佐冬奥会

베이징 올림픽은 해외 관중을 받지 않고 자국민에게만 입장권을 판매하기로 했다 As the Beijing Olympics tickets will be exclusively sold to spectators residing in mainland China,

北京冬奥会不接受海外观众,只向本国国民出售入场券

NHL 불참에 따른 티켓 판매 격감 등의 문제는 없겠지만 대회 권위 추락과 시청률 저하 등은 불가피할 것으로 전망된다 there will be no significant economic impact, such as a reduction in ticket sales, following the NHL’s announcement not to participate in the 2022 Beijing Olympics, but the Games’ reputation and viewership would seem to decline inevitably

虽然NHL不参赛将不会出现门票销售剧减等问题,但大赛权威下降和收视率下降等问题将不可避免

“화이자-모더나 이외 백신, 오미크론 예방효과 거의 없어”NYT: Vaccines except Pfizer and Moderna shots offer no protection against Omicron

美媒:“除辉瑞和莫德纳之外,其他疫苗几乎对奥密克戎毒株没有效果”

신종 코로나바이러스 감염증(코로나19) 백신 중 메신저리보핵산(mRNA) 방식인 화이자와 모더나를 제외한 나머지 백신들은 오미크론 변이 감염 예방 효과가 거의 없다고 미국 뉴욕타임스(NYT)가 19일(현지 시간) 보도했다 Most COVID-19 vaccines other than messenger RNA (mRNA) vaccines, such as Pfizer and Moderna vaccines, will not likely to offer protection against the Omicron variant, The New York Times (NYT) reported on Sunday (local time)

美国《纽约时报》当地时间19日报道说,新冠疫苗中,除了“信使核糖核酸(mRNA)”方式的辉瑞和莫德纳以外,其余的疫苗几乎没有预防感染奥密克戎变异毒株的效果

mRNA 방식이 아닌 다른 백신에 의존해 온 저소득 국가들을 중심으로 계속 감염이 늘고 변이가 출현할 것이라는 우려가 나온다 There are rising concerns that infections would increase and new variants would emerge in low-income countries, which have relied on vaccines other than mRNA vaccines

有人担心,以依靠mRNA方式以外的其他疫苗的低收入国家为中心,感染将继续增加,并出现变异

오미크론 변이가 코로나19 항체 치료제도 무력화한다는 연구 결과도 나왔다 A research found that the Omicron variant neutralizes antibody treatment for COVID-19

研究结果显示,奥密克戎毒株能使新冠抗体治疗药物失效

NYT는 “화이자, 모더나 백신만이 오미크론 변이로부터의 감염을 막을 수 있다는 연구 결과가 속속 발표되고 있다”고 보도했다 The NTY reported that only the Pfizer and Moderna shots appear to have provided protection against serious illness from Omicron

《纽约时报》说:“陆续发表的研究结果显示,只有辉瑞 莫德纳的疫苗才能防止奥密克戎毒株引起的感染"

영국 보건안전청(HSA)은 백신 접종자들을 분석한 결과 “아스트라제네카 백신을 맞은 지 6개월이 지나면 오미크론 감염 예방 효과가 0%대로 떨어진다”고 12일 밝혔다 The UK Health Security Agency (HAS) said on last Sunday that the effectiveness of the AstraZeneca vaccine against Omicron infection fell to 0 percent six months after vaccination

英国卫生安全局12日表示,对疫苗接种者进行分析的结果显示:“接种阿斯利康疫苗6个月后,预防感染奥密克戎毒株的效果将降至0%左右”

1회 접종 방식인 얀센 백신은 아프리카에서 수요가 급증하고 있지만 “오미크론 감염 예방 효과는 거의 무시해도 될 정도”라고 NYT는 지적했다 The Johnson & Johnson single-shot vaccine, whose demand is surging in Africa, “does little to nothing to stop the spread of Omicron,” NYT pointed out

《纽约时报》指出,虽然作为一针接种方式的强生疫苗在非洲的需求激增,但“预防奥密克戎感染的效果几乎可以忽略”

전 세계 백신 중 절반을 차지하는 중국 백신(시노팜, 시노백)에 대해선 “오미크론 감염을 거의 막지 못한다 중국, 멕시코, 브라질 등이 주로 이 백신을 접종했다”고 전했다 China’s Sinopharm and Sinovac vaccines, which account for almost half of COVID-19 vaccine doses delivered globally, “offer almost zero protection from Omicron infection,” NYT wrote, adding those vaccines are widely used in countries such as Mexico and Brazil

对于占全世界疫苗一半的中国疫苗(科兴 国药),报道称:“几乎无法防止感染奥密克戎主要是中国 墨西哥 巴西等国接种了该疫苗”

아프리카와 중남미 국가에서 주로 맞은 러시아의 스푸트니크 백신도 오미크론 예방 효과가 매우 낮다고 보도했다 The Russian Sputnik vaccine, which is being used in Africa and Latin America, shows “dismal rates of protection” against Omicron

主要在非洲和中南美国家注射的俄罗斯卫星疫苗的预防效果也很低

미국 존스홉킨스대 블룸버그공중보건대학원의 톨버트 니엔스와 연구원은 “부유한 선진국들은 첨단기술이 사용된 mRNA 백신 기술을 공유하지 않았고, 저소득 국가들은 다른 백신에 의존해야 했다 Wealthy countries did not share mRNA vaccine technology and as a result, low-income countries had to rely on non-mRNA vaccines, said Tolbert Nyenswah, a senior researcher with the Johns Hopkins Bloomberg School of Public Health

美国约翰斯霍普金斯大学布隆伯格公共保健研究生院研究员托尔伯特·尼恩斯表示:“富裕的发达国家没有共享使用尖端技术的mRNA疫苗技术,低收入国家只能依赖其他疫苗

그 결과 이들 국가에서 변이가 계속 나타나 코로나19 대유행을 연장시킬 것”이라고 지적했다 He pointed out that new variants will continue to emerge from those countries as a consequence, prolonging the pandemic

结果,这些国家不断发生变异,延长了新冠大流行”

20일 AP통신에 따르면 미국 제약사 리제네론과 일라이릴리는 미국 식품의약국(FDA)이 긴급사용을 승인해 미국 병원에서 가장 많이 사용 중인 자사의 항체 치료제가 오미크론에 듣지 않는 것으로 나타났다고 밝혔다 According to The Associated Press on Monday, U S Pharmaceutical companies Regeneron Pharmaceuticals and Eli Lilly and Company said their antibody treatments, which are being widely used in American hospitals after being authorized for emergency use by the Food and Drug Administration (FDA) have “diminished potency” verses Omicron Their antibody treatments are being used to prevent against severe disease

据美联社20日报道,美国制药公司“再生元”和“礼来”表示:“经美国食品医药局批准紧急使用,美国医院使用最多的本公司抗体药物对奥密克戎不起作用”

두 회사의 항체 치료제는 코로나19 환자가 중증으로 치닫는 것을 막는 데 쓰이고 있다 두 제약사는 새로운 항체 치료제를 신속하게 개발하겠다고 밝혔지만 AP통신은 “적어도 몇 달은 걸릴 것이다 의료진은 새 위기에 봉착하게 될 것”이라고 했다 The two companies said they can quickly develop new antibodies but it would take at least several months before the launch of those antibodies, The Associated Press reported, adding doctors will face yet another challenge

两家公司的抗体治疗药物正被用于防止新冠患者趋于重症两家制药公司表示将迅速开发新的抗体药物,但美联社表示:“至少需要几个月时间医疗人员将面临新的危机”

文“베이징올림픽 외교 보이콧 검토 안해”Moon says he is not considering diplomatic boycott of Beijing Olympics

文在寅:“不考虑外交抵制北京奥运会”

호주를 국빈 방문 중인 문재인 대통령이 13일 내년 2월 베이징 겨울올림픽 외교적 보이콧과 관련해 “한국 정부는 보이콧을 검토하고 있지 않다”고 밝혔다 South Korean President Moon Jae-in said on Monday that the South Korean government is not considering a diplomatic boycott of the Beijing Winter Olympics in February

正在对澳大利亚进行国事访问的韩国总统文在寅13日就一些国家外交抵制明年2月的北京冬奥会一事表示:“韩国政府不考虑抵制”

중국과 첨예하게 맞서고 있는 미국을 시작으로 호주 영국 등이 보이콧 행렬에 동참하고 있지만 문 대통령은 일단 선을 긋고 나선 것 During his state visit to Australia Moon drew a line while Australia and the U K have joined the boycott by the U S , which is in sharp conflict with China

从与中国针锋相对的美国开始,澳大利亚 英国等国家也加入了抵制行列,但文在寅与此划清了界限

문 대통령은 이날 호주 캔버라에서 스콧 모리슨 호주 총리와의 정상회담 뒤 가진 공동기자회견에서 “미국을 비롯한 어느 나라로부터도 (보이콧) 참가 권유를 받은 적이 없다”며 이같이 말했다 “We haven’t received any requests from any countries, including the U S , to participate,” said President Moon at a joint press conference following a summit meeting with Prime Minister of Australia Scott Morrison in Canberra on Monday

文在寅当天在澳大利亚堪培拉同澳大利亚总理斯科特·莫里森举行首脑会谈后举行的联合记者会上表示:“美国等任何国家从未劝说参与抵制北京冬奥会”

이어 “경제적인 측면, 한반도의 평화와 안정, 북한의 비핵화를 위해 중국의 건설적인 노력이 요구된다”며 “한국은 미국과의 굳건한 동맹을 기반으로 삼으면서 중국과도 조화로운 관계를 유지해 나갈 수 있도록 노력해 나가고 있다”고 했다 “Constructive efforts from China are required for the economy, peace, and stability on the Korean Peninsula, and the denuclearization of North Korea,” he added

他还表示:“无论从经济方面 韩半岛的和平与稳定还是朝鲜的无核化,我们都需要中国作出建设性的努力韩国正在努力以同美国建立稳固的同盟关系为基础,与中国保持和谐的关系”

미중 모두 종전선언의 중요한 관련국인 만큼 전략적 모호성 기조를 이어가며 균형외교를 펼치겠다는 의미로 풀이된다 “South Korea is trying to maintain a harmonious relationship with China while building on a solid alliance with the U S

分析认为,此话表明,美中都是发表《终战宣言》的重要相关国家,因此韩国将延续战略模糊性基调,展开均衡外交

이를 두고 미국 블룸버그통신은 ‘한국이 베이징 올림픽 보이콧을 놓고 미국과 결별했다(Breaks With US)’고 보도하며 “대북 화해를 핵심 목표로 삼은 문 대통령이 남은 임기 내 진전을 이루려면 중국의 도움이 필요할 것”이라고 해석했다 ” It seems that Moon is striving to achieve balanced diplomacy by continuing strategic ambiguity as both the U S and China are important to the future announcement of the end of the Korean War “South Korea breaks with the U S on a boycott of Beijing Olympics,” Bloomberg reported on the president’s statement “Moon has made reconciliation with North Korea one of his key policy objectives and, if he wants to make progress before his term in office ends next year, he will likely need Beijing’s help

美国彭博社就此报道说:“韩国在抵制北京奥运会问题上与美国闹掰了(BreaksWithUS)”报道分析称:“以对朝和解为核心目标的文在寅总统要想在剩下的任期内取得进展,需要中国的帮助”

정부, 세계 무역 15% 차지 CPTPP 가입 본격 추진정부가 세계 무역의 15%를 차지하는 ‘포괄적·점진적 환태평양경제동반자협정(CPTPP)’ 가입 추진을 공식화했다 ” South Korea applies to join CPTPP after China’s bid The South Korean government has officially announced to begin the application process to join the Comprehensive and Progressive Agreement for Trans-Pacific Partnership (CPTPP), a massive free trade deal taking up 15% of the world economy

政府将正式推进占世界贸易15%的CPTPP的加入工作韩国政府将正式推进占世界贸易15%的《全面 渐进的环太平洋经济伙伴协定(CPTPP)》的加入工作

중국에 대한 무역 의존도를 낮추고 교역을 다변화할 수 있는 기대가 나오지만 농산물 수입이 늘어날 것을 우려하는 농수산업계의 반발도 예상된다 While the deal is expected to serve as an opportunity to lower trade dependency on China and diversify Korea’s trade partnership, it is also feared to fuel steep opposition from local farmers and fisheries industry who are concerned about a fiercer competition against expanded imports

虽然出现了可以降低对中国的贸易依存度,实现交易多边化的期待,但是预计也会出现担心农产品进口增加的农水产业界的反对

13일 홍남기 경제부총리 겸 기획재정부 장관은 대외경제관계장관회의에서 “교역과 투자 확대를 통한 경제적, 전략적 가치 등을 고려해 CPTPP 가입을 본격 추진한다”며 “다양한 이해 관계자 등과 사회적 논의를 바탕으로 절차를 개시한다”고 밝혔다 “Considering the economic and strategic values derived from the expansion of trade and investments, we’ve decided to make an official bid to join the CPTPP,” said South Korean Finance Minister Hong Nam-ki at a meeting of Cabinet ministers on external economic affair on Monday “The process will begin based on our discussions with various groups of stakeholders,” Hong added

13日,经济副总理兼企划财政部长官洪楠基在对外经济长官会议中表示:“通过扩大贸易和投资的经济考虑 战略价值等,将正式推进加入CPTPP”,“将以与相关利害关系者等进行社会讨论为基础,启动程序”

정부가 2013년 CPTPP의 전신인 환태평양경제동반자협정(TPP)을 검토한 지 약 8년 만이다 The decision comes eight years after Seoul last considered making a bid for the membership of the TPP, the precursor to the CPTPP in 2013

这距离2013年政府讨论CPTPP的前身环太平洋经济伙伴协定(TPP)已有8年

CPTPP는 미국이 TPP를 탈퇴한 후 일본, 호주, 멕시코 등 11개국이 2018년 출범시킨 다자간 자유무역협정(FTA)이다 The CPTPP is a multilateral free trade deal launched in 2018 by 11 member states including Japan, Australia, and Mexico, after the U S exited the TPP trade deal

CPTPP是美国退出TPP后,日本 澳大利亚 墨西哥等11个国家于2018年签署的多边自由贸易协定(FTA)

일본이 의장국을 맡고 있고 올해 9월 중국과 대만이 가입을 신청했다 Currently, Japan is chairing the pact, with China and Taiwan having applied for membership in September this year

主席国由日本担任,今年9月中国也申请加入

CPTPP의 관세 철폐율은 최대 96% 수준으로 시장 개방도가 높다 The CPTPP boasts a high level of market openness, with a maximum 96% of tariff abolition rate

CPTPP的关税撤销率最高为96%,市场开放度很高

세계 무역의 15%를 차지하는 CPTPP에 가입하면 중국에 대한 무역 의존도가 낮아지고 수출시장이 다변화될 것으로 전망된다 Seoul is expecting to lower its trade dependency on China and diversify export markets by joining the CPTPP which accounts for 15% of global trade

CPTPP占世界贸易的15%,如果加入CPTPP,对中国的贸易依赖度会降低,出口市场也会变得多样化

올해 1월 한국개발연구원(KDI)은 “CPTPP 가입은 중국 의존도를 낮추고 통상 지형을 확대하는 데 매우 효과적”이라고 밝혔다 “Joining the CPTPP will prove highly effective in helping us lower our dependency on China and expand our trade landscape,” said an official from the Korea Development Institute (KDI) in January this year

今年1月,韩国开发研究院(KDI)曾表示,“加入CPTPP有助于降低对中国的依赖度,扩大通商领域”

미중 무역갈등이 지속되는 상황에서 안정적인 글로벌 공급망에 편입될 수 있다는 점도 장점이다 Another upside is to benefit from the stability of global supply chains amid the ongoing trade frictions between Washington and Beijing

在中美贸易矛盾持续的情况下,可以纳入稳定的全球供应链,这也是一大优点

특히 한국과 FTA를 체결하지 않은 멕시코와 처음으로 FTA를 체결하는 효과도 기대할 수 있다 The accession into the CPTPP can also be the chance to effectively sign a free trade deal with Mexica, a country that has yet to ink an FTA partnership with South Korea

特别是,还可以期待与没有和韩国签订FTA的墨西哥首次签订FTA的效果

반면 농수산업계는 농산물 수입이 늘어날 것을 우려하고 있다 By contrast, local farmers and fishermen are concerned about expanded imports of agricultural produces

相反,农水产业界则担心农产品进口增加

CPTPP 참여국 중 호주, 칠레, 캐나다 등 농업 강국이 많기 때문이다 In fact, the CPTPP boasts plenty of agricultural powerhouses such as Australia, Chile, and Canada

因为在CPTPP参与国中,澳大利亚 智利 加拿大等农业强国较多

이날 한국종합농업단체협의회는 성명을 내고 “상대적으로 가격 경쟁력이 높은 수입 농산물의 증가는 장기적으로 농업 생산기반 붕괴로 이어질 수 있다”고 반발했다 The Korea Agriculture Association Consultative Body issued a statement on Monday, saying that an increase in the price-competitive imported agricultural products can lead to a collapse of the country’s foundation of agricultural production in the longer term

当天,韩国综合农业团体协议会发表声明反驳说:“相对来说价格竞争力较高的进口农产品的增加会导致农业生产基础的长期崩溃”

실제 CPTPP에 가입하려면 공청회와 국회 보고 절차, 회원국과의 세부 협상이 필요해 2∼3년이 걸릴 것으로 보인다 Given additional procedures of public hearings, parliamentary reports, and discussions with member states, it will take another three to four years (转译）to complete the membership process

实际上,要想加入CPTPP,需要听证会 国会报告程序和与会员国的详细协商,预计需要2~3年的时间

정부가 가입 검토를 시작한 지 8년 만에 공식 가입 절차에 들어가 ‘뒷북 가입’으로 협상력만 떨어졌다는 지적도 나온다 Some point out Seoul’s belated bid to enter the process after eight years has only sapped its negotiating power

有人指出,政府开始讨论加入问题8年后才进入正式加入程序,因此“马后炮加入”导致谈判力下降

최종 가입을 위해 일본 등 참여국의 만장일치 동의가 필요하기 때문이다 A final accession requires a unanimous vote from all member states including Japan

因为为了最终加入,需要日本等参与国的一致同意

최원목 이화여대 법학전문대학원 교수는 “중국보다 가입이 한발 늦어져 한국의 협상력이 떨어질 수밖에 없다”며 “주도국인 일본은 강제징용 피해자에 대한 일본 기업의 배상 판결 등을 협상에 활용할 수 있다”고 분석했다 “Seoul’s negotiating power is relatively weaker because China has already begun the application process,” said Prof Choi Won-mok of Ewha Womans University “And Japan can capitalize on its chairmanship by playing the card of the court rulings on Korean victims of forced labor in rendering the final decision on Seoul’s membership

梨花女子大学法学专门研究生院教授崔元睦(音)分析说:“加入时间比中国晚了一步,因此韩国的谈判力必然会下降而作为主导国的日本也可以将日本企业对强制征用受害者的赔偿判决等活用在谈判中因此还有许多问题需要解决”

美 “베이징올림픽 외교적 보이콧”…中 “결연히 반격” U.S. announces diplomatic boycott of Beijing Olympics

美国宣布“外交抵制北京奥运会”,中国表示“坚决反击”

미국이 내년 2월 열리는 베이징 겨울올림픽에 대한 외교적 보이콧(diplomatic boycott)을 6일(현지 시간) 선언했다 The U.S. announced on Monday (local time) a diplomatic boycott of the Beijing Winter Olympics to be held in February.

美国在当地时间6日宣布,对明年2月举行的北京冬奥会进行“外交抵制”。这是把中国政府在新疆维吾尔自治区的人权压迫规定为“种族屠杀”,开始进行外交制裁

중국 당국의 신장(新疆)위구르 지역에 대한 인권탄압을 ‘제노사이드’(집단 학살)로 규정하면서 외교제재에 나선 것이다 중국의 공개적인 보복 경고에도 뉴질랜드 등이 보이콧 동참을 선언한 가운데 종전선언을 추진하고 있는 문재인 정부는 일단 올림픽 외교사절단 파견에 무게를 싣고 있다 It was a diplomatic measure based on the judgment that defined the Chinese authorities’ crimes against humanity in Xinjiang as genocide. While New Zealand and others announced to join the boycott despite China’s open warning for countermeasures, the South Korean government is leaning to sending an Olympics diplomatic mission.

尽管中国公开警告要进行报复,新西兰等仍宣布参与抵制,这种情况下,正在推进《终战宣言》的文在寅政府暂时倾向于向奥运会派遣外交使团

젠 사키 미국 백악관 대변인은 이날 “조 바이든 행정부는 베이징 겨울올림픽과 패럴림픽에 외교 및 공식 대표단을 파견하지 않기로 했다”고 밝혔다 “The Biden administration will not send any diplomatic or official representation to the Beijing 2022 Winter Olympics and Paralympic Games,” said White House press secretary Jen Psaki on Monday.

美国白宫发言人简·普萨基当天表示:“拜登政府决定不向北京冬奥会和残奥会派遣外交及官方代表团”

선수단은 파견하되 개·폐회식에 정부 고위급이나 정치권 인사들로 구성된 공식 사절단은 보내지 않는다는 의미다 While athletes will travel to China, an official mission consisting of high-ranking government and political figures will not be sent to the opening and closing ceremonies.

即,美国将派遣体育代表团,但不会派遣由政府高层人士或政界人士组成的正式使团参加开 闭幕式

사키 대변인은 “중국 신장에서 제노사이드와 인권 유린이 계속되고 있는 상황”이라며 “우리는 올림픽 경기의 팡파르에 동참할 수 없다”고 했다 The press secretary said that genocide and crimes against humanity and other human rights abuses are ongoing in Xinjiang. “We will not be contributing to the fanfare of the Games,” she said.

普萨基表示:“在中国新疆,‘种族歧视’和‘践踏人权’正在持续我们不能参加奥运会比赛的进行曲"

바이든 행정부는 다른 동맹국의 보이콧 참여도 사실상 공개 요청했다 The Biden administration practically asked its allies to join the boycott.

拜登政府实际上也公开要求其他盟国参与抵制

네드 프라이스 국무부 대변인은 “더 많은 국가로부터 (보이콧) 소식을 듣게 될 것”이라고 했다 State Department spokesman Ned Price said they will hear from more countries to join the boycott.

美国国务院发言人内德·普莱斯表示:“将从更多国家听到(抵制)消息”

이런 가운데 뉴질랜드가 7일 “베이징 올림픽에 정부 고위 대표단을 보내지 않기로 했다”고 밝혔다 New Zealand announced on Tuesday that its senior government representation will not be sent to the Beijing Olympics.

在这种情况下,新西兰7日表示:“决定不派遣政府高层代表团参加北京奥运会”

영국, 호주, 캐나다 등을 중심으로 보이콧 선언이 이어질 것으로 보인다 The U.K., Australia, and Canada are expected to follow suit.

预计,以英国 澳大利亚 加拿大等为中心,抵制宣言将会接连不断

중국은 “단호한 반격 조치를 취하겠다”며 강하게 반발했다 China strongly opposed and warned firm countermeasures.

中国强烈反对说:“将采取坚决的反击措施”

류펑위(劉鵬宇) 주미 중국대사관 대변인은 7일 “성공적인 올림픽 개최에 아무런 영향을 미치지 못할 정치적 조작”이라고 비판했다 “Politicians calling for boycott the 2022 Beijing Olympics are doing so for their own political interests and posturing. It has no impact whatsoever on the Beijing 2022 to be successfully held,” Liu Pengyu, the spokesperson of the Chinese embassy in the U.S., said on Tuesday.

中国驻美大使馆发言人刘鹏宇7日抨击说:“这是不会对奥运会成功举办产生任何影响的政治操作”

이에 앞서 자오리젠(趙立堅) 중국 외교부 대변인은 6일 정례 브리핑에서 “만약 미국이 독단적으로 행동한다면 중국은 반드시 반격하는 조치를 결연하게 취할 것”이라고 했다 “China will take resolute countermeasures against the U.S.’s dogmatic actions,” Zhao Lijian, the spokesperson for the Chinese Ministry of Foreign Affairs, said during a regular briefing on Monday.

中国外交部发言人赵立坚6日也在例行记者会上表示:“如果美国擅自行动,中国一定会坚决采取反击措施”

미국은 9, 10일 한국 등 110여 개국을 초청해 화상으로 여는 민주주의 정상회의에서도 보이콧 동참을 요청할 것으로 보이는 가운데 청와대는 일단 올림픽 불참 가능성에 거리를 두고 있다 （no translation）

预计美国将在9日 10日邀请韩国等110多个国家举行的民主峰会上也要求共同参与抵制,但青瓦台暂时对不参加奥运会的可能性保持距离

최영삼 외교부 대변인은 7일 정례 브리핑에서 “미국 측은 외교 경로를 통해 이번 결정을 우리 측에 미리 알렸다 다만 보이콧 동참을 요구해온 바는 없다”고 밝혔다 （no translation）

韩国外交部发言人崔永杉7日在例行记者会上表示:“美方通过外交渠道提前向我方通报了这一决定不过没有要求参与抵制”

그러면서 “우리 정부는 베이징 올림픽의 성공적 개최를 지지해 왔다 （no translation）

他还表示:“韩国政府一直支持北京奥运会的成功举办

올림픽이 세계 평화와 번영 및 남북관계에 기여하기를 희망한다”고 했다 （no translation）

希望奥运会能为世界和平 繁荣和南北关系做出贡献”

홍콩, ‘보안법’ 1년만에 5500명 학교 떠나지난 Hong Kong security act causes 5,500 students and teachers to leave school

香港《保安法》实行一年,5500名学生离校

1년 동안 홍콩에서 중고등학교를 그만둔 학생과 교사가 5000명을 넘어선 것으로 드러났다 More than 5,000 students and teachers have reportedly left secondary school in Hong Kong in the past year

一项调查结果显示,在过去一年里,香港初中和高中辍学和离职的学生和教师超过5000人退学的10名学生中有6人干脆表示将离开香港

학교를 그만둔 학생 10명 가운데 6명은 아예 홍콩을 떠나겠다고 밝혔다 Six out of 10 students who left school responded that they would leave Hong Kong for good

有分析认为,这是因为香港实施对进行反中活动的香港市民最高可判处无期徒刑的《国家保安法》等,香港的社会环境与《国家保安法》实施前发生了很大的变化

반중 활동을 한 홍콩 시민을 최대 무기징역에 처할 수 있도록 한 홍콩 국가보안법 시행 등으로 홍콩의 사회 환경이 보안법 시행 전과 크게 달라진 여파 때문이라는 분석이 나오고 있다 Since the Hong Kong Security Act, which allowed the state to sentence protestors in opposition to mainland China to life imprisonment at maximum, entered into force, Hong Kong’s social environment has radically changed, and this may have contributed to the large exodus of students and teachers

据香港媒体“HK01”2日报道,前一天香港初高中校长协会——香港中学校长会以140所初高中为对象

2일 홍콩 매체 HK01 등에 따르면 전날 홍콩 중고교 교장들의 모임인 홍콩중학교장회는 140개 중고교를 대상으로 조사한 결과 2020∼2021학년도 1년 동안 학생 4460명과 교사 987명이 학교를 그만둔 것으로 나타났다고 밝혔다 HK01, a Hong Kong-based online news portal, cited on Thursday the data gathered by the group of Hong Kong middle and high school principals, which surveyed of 140 middle and high schools in Hong Kong and revealed that a total of 4,460 students and 987 teachers have left school during the school year 2020-2021

进行调查的结果显示,2020年至2021学年度一年间有4460名学生和987名教师离开

학교당 평균 32명의 학생과 7명의 교사가 그만둔 셈이다 This is translated into an average of 32 students and seven teachers per school

这相当于平均每个学校有32名学生和7名教师离开

한 해 전 조사에서 각각 학생 2700명과 교사 498명이 그만둔 것보다 대폭 늘었다 The number of students and teachers who left school has increased considerably from the previous year, when 2,700 students and 498 teachers left school

在一年前的调查中,分别有2700名学生和498名教师辞职,相比之下大幅增加

특히 학교를 그만둔 학생 가운데 2643명(약 59.2%)은 홍콩을 떠나 다른 나라로 가겠다고 밝혔다 Of those students who left school, 2,643 students (59.2% of the respondents) said that they would leave Hong Kong and go to other countries

特别是,退学学生中有2643人(约59.2%)表示,将离开香港前往其他国家

중학교장회는 “학교를 그만두고 다른 나라로 이민을 선택하는 교사도 7배 이상 증가했다”며 “지난 1년 동안 학생과 교사의 이탈이 상당히 심각한 상황이라는 것은 명백한 사실”이라고 우려했다 The Association of Principals expressed concern that the number of teachers who left school and chose to emigrate to other countries has increased by seven-fold, clearly suggesting the gravity of the exodus of both students and teachers for the past year

初中校长会表示担忧说:“选择退学移民到其他国家的教师也增加了7倍以上

이런 사태의 이유로 보안법 시행으로 홍콩의 전반적인 사회 환경이 억압적으로 변하고 교육 정책과 커리큘럼 또한 친중 일색으로 바뀌면서 학생과 교사 모두 실망했기 때문이라는 분석이 나온다 Critics point out the oppressive social environment and pro-China education policies and curriculum upon the implementation of the Security Act, which gave rise to the disappointment of students and teachers, and cite it as the cause of the mass departure

在过去的一年里,学生和教师的离校现象非常严重,这是不争的事实”

신종 코로나바이러스 감염증(코로나19) 확산을 막기 위해 중국과 홍콩 간 왕래가 제한되면서 중국 본토 학생들이 홍콩 학교로 등하교할 수 없는 것도 영향을 미친 것으로 풀이된다 As the traffic between China and Hong Kong is restricted to stop the spread of COVID-19, students from mainland China are prohibited from commuting to Hong Kong schools, and this is presumed to have partly contributed to the departure

有分析认为,发生这种事态的原因是,随着《国家安全法》的实施,香港整体的社会环境变得压抑,教育政策和课程也变成了一边倒的“亲中”,学生和教师都感到失望据分析,为防止新冠疫情扩散,中国大陆和香港之间的往来受到限制,因此中国大陆学生无法到香港学校上下学,这也产生了一定的影响

중학교장회는 앞서 7월에도 당국에 “많은 학생과 교사들이 해외로 떠나고 있다”며 대책 마련을 요청했다 Back in July, the Association of Principals first called for the education department to take measures by stating that many students and teachers are exiting from Hong Kong to go toward other countries

此前,中学校长会曾于7月份向当局表示,“很多学生和教师正在前往海外”,要求制定对策

中도 저출산-고령화… “4년뒤 ‘성인용 기저귀 〉 유아용’ 역전”China faces demographic change

中国也迎来低生育-高龄化……英媒:“4年后大逆转,中国成人尿布需求将大于婴儿尿布”

중국에서 2025년부터 성인용 기저귀 판매량이 유아용 판매량을 추월할 것이라고 영국 파이낸셜타임스(FT)가 보도했다 중국이 겪고 있는 심각한 저출산 문제와 고령화 현상이 반영된 결과로 보인다 The Financial Times reported that the Chinese market for adult diapers could exceed infant products by 2025, reflecting China’s low birth rates and aging population trends

英国《金融时报》报道说,从2025年开始,中国成人尿布销量将超过婴儿尿布销量这可能是中国正在经历的严重的低生育问题和老龄化现象的反映

FT는 최근 중국의 유아용 기저귀 판매 수요가 점차 둔화되고 있는 반면 요실금을 앓는 고령층, 노인 요양시설 등을 위한 성인용 수요는 늘고 있다고 지난달 29일 보도했다 The article said on Monday that while the demand for children’s diapers was dwindling, there is surging demand for adult nappies at care homes

《金融时报》11月29日报道说,最近中国婴幼儿尿布销量逐渐减少,而为患尿失禁的高龄层和老人疗养设施等而使用的成人用尿布销量正在增加

중국 기업 관계자들은 2025년이면 연간 판매량에서 성인용 기저귀가 유아용을 앞설 것이라고 FT에 말했다 Chinese manufacturers told The Financial Times that the sales of adult diapers would exceed children’s diapers in terms of annual sales by 2025

中国企业有关人士向《金融时报》表示,到2025年,成人纸尿裤销量将超过婴儿纸尿裤

FT에 따르면 중국 1위 기저귀 판매사 유니참은 유아용보다 성인용에 마케팅 비용을 더 쓰고 있다 Unicharm, the best-selling diaper brand in China, is spending more of its marketing budget to adult diapers than baby diapers

据《金融时报》报道,中国第一大尿布销售商尤妮佳在成人用品上花费的市场营销费用比婴儿用品多

중국 후베이성에 있는 기저귀 생산 공장 관계자는 “유아용 생산 라인을 성인용으로 바꿨다”고 했다 A factory owner from a nappy factory in Hubei said that the factory’s manufacturing lines had shifted from baby diapers to adult diapers

中国湖北省尿布生产厂的有关人士表示:“已经将婴儿生产线改为成人生产线”

FT는 중국 기저귀 시장이 변곡점을 향해 가고 있다고 분석했다 The Financial Times said that China’s diaper market is gearing for a fundamental shift

《金融时报》分析说,中国尿布市场正走向拐点

홍콩계 증권사 CLSA의 분석에 따르면 지난해 중국의 기저귀 시장은 890억 달러(약 105조7943억 원)였는데 그중 성인용 시장은 10억 달러에 못 미쳤다 The Hong Kong-based investment and brokerage group CLSA predicts that within just over eight years the adult diaper market in China could be worth 16 billion dollars, from less than 1 billion dollars last year

据香港证券公司里昂证券(CLSA)的分析,去年中国的尿布市场为890亿美元(约105.7943万亿韩元),其中成人用尿布市场不到10亿美元

하지만 성인용 기저귀 시장은 2040년이면 300억 달러(약 35조7300억 원) 규모로 성장할 것으로 전망됐다 By 2040, the market size could rise to 30 billion dollars

但成人用尿布市场预计到2040年将增长到300亿美元(约35.73万亿韩元)

이는 중국 인구 구조의 변화 때문이다 This change is due to China’s changing demographics

这是因为中国人口结构的变化

세계은행 통계와 중국 인구조사에 따르면 중국 여성 1인당 평균 출산율은 1961년 5.9명에서 지난해 1.3명으로 줄었다 1952년 조사 이래 최저치다 According to World Bank and China census, the average birth rate of women in China fell from 5.9 in 1961 to 1.3 last year, the lowest since research started in 1952

据世界银行统计和中国人口调查,中国女性人均生育率从1961年的5.9人降至去年的1.3人这是1952年开始调查以来的最低值

프랑스 투자은행 나티시스는 지난해 중국의 고령층은 인구의 10%였지만 2050년이 되기 전에 25%로 늘어날 것이라고 전망했다 French investment bank Natixis predicts that China’s senior population, which accounted for 10% of the total population last year, could rise to 25% before 2050

法国投资银行——法国外贸银行预测,去年中国的老龄层是人口的10%,但到2050年将增长到25%

FT는 “기저귀 판매량의 변화는 인구통계학적 변화와 사회구조의 전환을 의미한다”고 전했다 （no translation）

《金融时报》说:“尿布销量的变化意味着人口统计学的变化和社会结构的转换”

앞서 고령화를 겪은 일본도 10년 전부터 성인용 기저귀 판매량이 유아용을 추월하는 현상이 벌어졌다 （no translation）

此前经历老龄化的日本也从10年前开始出现成人用尿布销量超过婴幼儿用尿布的现象

서훈 내일 1박 2일 방중…양제츠와 종전선언 논의서훈 청와대 국가안보실장이 2일부터 1박 2일 일정으로 중국을 방문한다 NIS chief Suh Hoon to visit China on Thursday Director of the National Intelligence Service Suh Hoon will visit China on Thursday for two days

徐薰明日访华,与杨洁篪讨论终战宣言青瓦台国家安保室室长徐薰将从2日起对中国进行为期两天的访问

미국과의 종전선언 논의가 마무리 단계에 접어든 가운데 서 실장은 이번 방중에서 종전선언과 관련해 중국 측에 협조를 구하고 북한을 대화 테이블로 이끌기 위한 협력 방안을 모색할 것으로 보인다 With the discussion with the U S regarding the announcement of the end of a war between the two Koreas in the finalization stage, Suh will ask for China’s cooperation for the batter and look for ways to bring North Korea to a discussion table during the visit

在韩美有关终战宣言的讨论进入收尾阶段的情况下,徐薰可能会在此次访华中就终战宣言寻求中国方面的协助,并为将朝鲜拉到对话桌前寻求合作方案

30일 외교 소식통에 따르면 서 실장은 2, 3일 중국을 방문해 양제츠(楊潔지) 중국 공산당 외교담당 정치국원과 회동한다 According to a diplomatic source on Tuesday, Suh will meet with Yang Jiechi, a member of the Politburo of the Chinese Communist Party in charge of foreign affairs, in China on Thursday and Friday

据外交消息人士30日透露,徐薰将于2日至3日访问中国,与中国共产党负责外交事务的政治局委员杨洁篪举行会晤

두 사람이 만나는 것은 지난해 8월 이후 1년 3개월여 만이다 The two are meeting each other for the first time since August last year

两人自去年8月以后时隔1年零3个月再次见面

청와대는 “한중 양국은 고위급이 다양한 계기를 통해 전략적 소통을 유지하고 있고, 그런 맥락 속에서 서 실장 방중을 중국 측과 협의 중에 있다”고 밝혔다 “South Korea and China have maintained strategic communication on various occasions between their senior leaders

青瓦台表示:“韩中两国高层通过各种契机保持战略沟通,在这种情况下,正在与中方协商徐薰室长访华事宜”

서 실장은 이번 방중에서 종전선언에 대한 중국의 협조를 당부하고 내년 2월 베이징 겨울올림픽에 대한 의견을 교환할 것으로 보인다 Against the backdrop, we are discussing the director’s visit to China,” said a member of Cheong Wa Dae Suh will ask for Beijing’s cooperation on the announcement of the end of the Korean War and share opinions about the Beijing Winter Olympics to be held in February during his visit

预计,徐薰此次访华期间将希望中国在终战宣言上给予协助,并就明年2月的北京冬奥会交换意见

미국이 베이징 올림픽에서 외교적 보이콧을 시사했고, 신종 코로나바이러스 감염증(코로나19) 상황이 지속되는 등 올림픽 흥행 실패를 우려하는 중국은 우리 정부의 협조를 구하는 것으로 알려졌다 China is asking for South Korea’s cooperation for the Olympics as the U S implied a diplomatic boycott of the Olympics and COVID-19 outbreaks continue

据悉,美国暗示将对北京奥运会进行外交抵制,中国担心新冠疫情持续等导致冬奥会票房失败,正在寻求韩国政府的协助

청와대 관계자는 “베이징 올림픽이 남북관계 개선을 위한 전기가 되고 동북아 세계평화에 기여하는 계기가 되길 바란다는 입장에는 변함이 없다”면서도 “(외교적 보이콧 등에 대해) 아직 정부가 특별한 입장을 가질 상황은 아니다”라고 했다 “We haven’t changed our stance that we hope the Beijing Olympics will be an opportunity for the improvement of inter-Korean relationship and contribute to peace in Northeast Asia,” said a member of Cheong Wa Dae “It is too early for the South Korean government to take a stance regarding the diplomatic boycott

青瓦台一名有关人士表示:“希望北京奥运会成为改善韩朝关系的转机,成为为东北亚世界和平做出贡献的契机,这一立场没有改变(就外交抵制等问题,)政府目前并没有特别的立场”

이번 방중에서 서 실장은 시진핑(習近平) 중국 국가주석의 방한 문제도 논의할 것으로 보인다 ” Suh will also discuss Chinese President Xi Jinping’s visit to South Korea

此次访华期间,徐薰还将讨论中国国家主席习近平访韩的问题

다만 코로나19 상황이 이어지고 있는 만큼 청와대는 화상 정상회담 방식도 고려하고 있는 것으로 알려졌다 As COVID-19 continues, Cheong Wa Dae is also considering a video conference summit meeting

据悉,由于新冠疫情仍在持续,青瓦台正在考虑举行视频首脑会谈的方式

고향과 집고향은 우리의 그리움이 향하는 곳이다 Homeland and home Homeland is where we long to be

故乡和家故乡是我们的思念所向的地方

그것은 자크 데리다에 따르면 “선영들이 묻혀 있는 땅” 혹은 “모든 여행과 모든 거리를 거기에서부터 가늠하는 부동의 장소” As French philosopher Jacques Derrida put it, it is “where our ancestors lie or the immovable place where all travel and distance begin

根据雅克?德里达的说法,这是一块“埋葬祖坟的土地”,或者是一个“从那里衡量一切旅行和距离的静止地点”

다 낯선 땅에 살던 사람들이 죽을 때 고향에 묻히기를 바라는 것은 그래서다 ” Perhaps this is the reason why some people who lived in foreign lands wish to be buried where they were born

因此,生活在陌生土地上的人们希望在死后安葬在故乡

그런데 지난주에 제1회 부천디아스포라문학상 수상을 위해 한국을 찾은 미국 작가 하 진은 고향을 그렇게 인식하지 않는다 But not for American writer Ha Jin, who visited Korea last week to receive the first Bucheon Diaspora Literary Award

但是,上周为领取第一届富川流散文学奖而来到韩国的美国作家哈金却不这样看待故乡

그에 따르면 고향은 인간이 뿌리를 내리고 살아가는 곳이다 He says that homeland is where one puts his/her roots down

根据他的说法,故乡是人类扎根生活的地方

두고 떠나온 곳이 아니라 어딘가에서 다시 만드는 유동적인 것 It is not some place that you left behind, but where you build

不是离开遗弃的地方,而是在某处重新创造的流动的东西

그의 삶을 생각하면 맞는 말이다 Ha’s words resonate his life

如果考虑到他的人生,这句话是对的

미국에서 유학 중이던 그는 1989년 톈안먼 대학살의 실상을 텔레비전으로 지켜보다가 망명을 택했다 He decided to seek asylum while watching televised scenes from the Tiananmen Square Massacre in 1989

在美国留学的他在1989年通过电视看到天安门事件的真实情况后,选择了流亡

그리고 중국을 배경으로 하는 작품들을 영어로 써서 전미도서상을 수상할 정도로 유명 작가가 되었다 He became famous for his literary work with Chinese background written in English, winning National Book Awards

他还用英语写出了以中国为背景的作品,获得了全美图书奖

중국 정부는 그를 배반자로 낙인찍고 몇십 년 동안 입국을 허용하지 않았다 The Chinese government regarded him as a traitor and forbid him to enter China for several years

中国政府把他打上叛徒的烙印,几十年来一直不允许他入境

그러나 그는 배반의 주체가 자신이 아니라 무고한 젊은이들을 학살한 국가라고 생각했다 However, he believes that the betrayal is done by the nation that killed innocent young people, not himself

但他认为背叛的主体不是自己,而是屠杀无辜年轻人的国家

중국은 보호해줘야 하는 “자식들을 잡아먹은 어미”였다 그에게 중국이 고향이 아니게 된 이유다 China was a “mother that had eaten her own children,” which is why he does not regard China as his homeland

对他来说那个国家是需要保护的“吃掉子女的母亲”这就是对他来说中国不是故乡的原因

그러나 그의 심리적 현실은 다르다 However, in psychological reality, things are different

但他的心理现实却不同

그는 미국이 집이라고 말하지만 중국으로 거듭 돌아간다 중국인이나 중국인 이민자를 소설에 매번 등장시키는 것도 심리적, 은유적인 의미에서 보면 귀향이나 다를 바 없기 때문이다 He refers to America as his home but constantly reverts to China, as seen from Chinese immigrant characters in his work

虽然他说美国是他的家,但他却一再回到中国因为每次小说中都出现中国人或中国移民,从心理和隐喻的意义上看,与“回乡”没有什么区别

그가 정체성이 이미 확립된 서른 살 무렵에 고향을 떠났기에 더욱 그러한지 모른다 Perhaps this is because he had left his homeland in his 30s when his ethical identity was already established

也许因为他在三十岁的时候就离开了故乡,认同感已经确立

그의 말과 다르게 미국이 진짜 고향, 진짜 집이 되는 것은 거의 불가능해 보인다 Unlike his words, the U S might not be his real homeland or home

与他的话不同,美国几乎不可能成为真正的故乡和家

그는 한국에 왔음에도 몇 시간이면 갈 수 있는 고향에 가지 못하고 그의 집이 있는 미국으로 쓸쓸히 돌아갔다 He returned to the U S after his visit to Korea without visiting his homeland, though it was just a few hours away

他虽然来到了韩国,但却无法回几个小时就能抵达的故乡,而是冷冷地回到了他的家所在的美国

하기야 지난 36년을 그렇게 살았다 Then again, he had lived that way for 36 years

过去的36年他就是这样生活的

부모가 세상을 떠났을 때도 고향에 가서 애도조차 할 수 없었다 He had not even been able to visit to mourn for his parents when they passed away

父母去世时,他连回老家哀悼都没有

가혹한 형벌이었다 It was a cruel punishment

这是残酷的刑罚

그러한 실존이 그를 디아스포라 작가로 만들었다 His being made him a diaspora writer whose works reveal wounds of the past

正是这种现实使他成为了一位流散作家

그가 쓴 시와 소설 주변에 상처가 아른거리는 이유다（（no translation））

这就是他写的诗和小说周围闪现伤痕的原因

美中 반도체 전쟁에 낀 하이닉스, 초격차 확대로 극복해야Hynix stuck between U S and China for semiconductor war

陷入中美半导体战争的SK海力士,要以扩大绝对差距来摆脱困局

네덜란드산 첨단 장비를 도입해 중국 장쑤성 우시 공장을 개선하려는 SK하이닉스의 계획이 미국 정부의 반대로 무산될 위기에 처했다고 로이터통신이 어제 보도했다 Reuters reported Wednesday that SK Hynix’s plan to overhaul its plant in Wuxi, Jiangsu Province by acquiring advanced equipment from the Netherlands is in jeopardy

据路透社昨天报道,SK海力士想要引进荷兰产尖端设备改善中国江苏省无锡工厂的计划,因美国政府的反对而面临流产的危机

“SK하이닉스가 미국과 중국의 지정학적 분쟁에서 다음 차례 희생양이 될 수 있다”고도 했다 “The potential setback could make SK Hynix the next victim of the geopolitical struggle between the U S and China,” it said

报道还说:“在美国和中国的地缘政治纷争中,SK海力士有可能成为下一个牺牲品”

미중 경제패권 전쟁의 한복판에 놓인 우리 반도체 기업들이 가장 우려하던 사태가 현실로 나타날 가능성이 커진 것이다 The biggest concerns of South Korean semiconductor players in the middle of the battle between the U S and China to seize economic hegemony might become a reality

处在美中经济霸权战争中心的韩国半导体企业最担心的事件很有可能成为现实

SK하이닉스의 3분기 세계 D램 반도체 시장 점유율은 27%로 삼성전자(44%)에 이은 2위다 SK Hynix had the second largest market share in the global D-RAM semiconductor sector in the third quarter with 27 percent, following Samsung Electronics’ 44 percent

SK海力士第三季度在世界DRAM(动态随机存取存储器)半导体市场的占有率为27%,仅次于三星电子(44%),位居第二

정부로부터 각종 보조금과 세제지원을 받으며 추격하는 미국 유럽연합 중국 경쟁업체들을 따돌리기 위해서는 첨단 기술, 장비에 대한 공격적 투자가 필수적이다 In order to hold the lead against competitors in the U S , the E U , and China backed by government subsidies and tax benefits, aggressive investment in cutting-edge technologies and equipment is a must

美国 欧盟 中国等国家的竞争企业得到政府的各种补贴和税制支援,SK海力士要想甩掉它们,必须对尖端技术和装备进行进攻性投资

특히 우시 공장은 이 회사 D램 제품 절반이 생산되는 중요 시설이어서 기업 경쟁력에 악영향이 미칠 수 있다 In particular, the plant in Wuxi is a key facility manufacturing half of the company’s D-RAM products

特别是无锡工厂是生产该公司一半DRAM产品的重要设施,有可能对企业竞争力产生负面影响

조 바이든 정부는 미국 및 동맹국 기술이 사용된 첨단 반도체 장비가 중국으로 들어가는 걸 절대 용납하지 않을 태세다 It can have a negative impact on the company’s competitiveness if the plan falls through The Biden administration seems to be strongly opposing the export of semiconductor equipment utilizing the technologies of the U S and its allies to China

拜登政府摆出了一副绝不允许使用美国及同盟国技术的尖端半导体设备进入中国的架势

중국의 군사력 강화에 악용될 수 있다는 게 표면적 이유지만 양과 질 모든 면에서 제조업 최강국에 올라서겠다는 ‘중국제조 2025’ 계획 등을 견제하는 게 주목적이다 The superficial reason is that it can be misused to strengthen China’s military power but the main purpose is to keep in check the ‘Made in China 2025’ plan, which aims to put the country as a leader of manufacturing both in terms of quantity and quality

表面上的理由是有可能被恶用于加强中国的军事力量,但其主要目的,是想牵制要在数量和质量所有方面成为制造业最强国的“中国制造2025”计划

미국 정부는 지난주 중국 현지 실리콘웨이퍼 생산량을 늘리려던 자국 반도체업체 인텔의 계획까지 포기시켰을 정도로 일체의 예외를 인정하지 않는 분위기다 The U S government seems to be allowing no exception as it dismissed last week its own semiconductor company Intel’s plan to increase silicon wafer production in China

美国政府上周甚至放弃了想要增加中国当地硅晶片产量的本国半导体企业英特尔的计划,似乎不允许一切例外

문제는 중국이 한국산 반도체 40%를 사가는 최대 고객이란 점이다 （no translation）

问题是中国是购买韩国产半导体40%的最大客户

홍콩을 통한 우회수출을 합하면 비중이 60%가 넘는다는 분석도 있다 （no translation）

有分析认为,如果加上通过香港的迂回出口,所占比重将超过60%

미국의 제동 때문에 중국 공장에 대한 시설투자를 제대로 못하면 중국은 미국 대신 애먼 한국 기업을 표적삼아 보복에 나설 가능성이 있다 （no translation）

如果因美国的掣肘而不能正常对中国工厂进行设备投资,中国可能会无辜的韩国企业而不是美国为目标进行报复

‘싸드 사태’ 때 그랬듯 전혀 다른 분야의 한국기업에까지 불똥이 튈 수도 있다 （no translation）

就像发生“萨德事件”时一样,完全不同领域的韩国企业也会受到牵连

이런 불편한 상황을 주도적으로 해결하는 방법은 어떤 갈등이 불거져도 중국 기업들이 한국산 반도체를 사지 않을 수 없도록 기술, 품질 초격차를 더 벌리는 길뿐이다 A solution to this uncomfortable situation is putting a wider gap against competitors in terms of technology and quality so that Chinese companies have no option but to buy semiconductors made by South Korean companies

解决这种尴尬状况的方法只有一个,那就是进一步拉大技术与质量上的绝对差距,无论出现什么矛盾,中国企业都不得不购买韩国产半导体

그런데도 SK하이닉스의 투자가 예정된 용인 반도체 클러스터는 주민 설득, 인허가가 지연돼 당초 계획보다 착공이 1년 이상 늦어지고 있다 Despite the circumstances, the construction of Yongin semiconductor cluster, in which SK Hynix plans to invest, is being postponed for over a year due to a delay in getting residents’ approval and licensing

但是,SK海力士计划投资的龙仁半导体集群工业园区,却因说服居民和批准推迟,开工时间比原计划推迟了一年以上

정부와 정치권은 반도체 산업 육성을 위한 특별법에 더 과감한 지원책과 규제완화 방안을 담아 서둘러 통과시켜야 한다 The government and the political circles should pass a special law to promote the growth of the semiconductor industry along with bolder measures to provide support and ease regulations

政府和政界必须为《培养半导体产业特别法》提供更果断的支援政策和放宽限制的方案,尽快予以通过

(（additional translation））
Hynix stuck between U S and China for semiconductor war Reuters reported Wednesday that SK Hynix’s plan to overhaul its plant in Wuxi, Jiangsu Province by acquiring advanced equipment from the Netherlands is in jeopardy “The potential setback could make SK Hynix the next victim of the geopolitical struggle between the U S and China,” it said The biggest concerns of South Korean semiconductor players in the middle of the battle between the U S and China to seize economic hegemony might become a reality SK Hynix had the second largest market share in the global D-RAM semiconductor sector in the third quarter with 27 percent, following Samsung Electronics’ 44 percent In order to hold the lead against competitors in the U S , the E U , and China backed by government subsidies and tax benefits, aggressive investment in cutting-edge technologies and equipment is a must In particular, the plant in Wuxi is a key facility manufacturing half of the company’s D-RAM products It can have a negative impact on the company’s competitiveness if the plan falls through The Biden administration seems to be strongly opposing the export of semiconductor equipment utilizing the technologies of the U S and its allies to China The superficial reason is that it can be misused to strengthen China’s military power but the main purpose is to keep in check the ‘Made in China 2025’ plan, which aims to put the country as a leader of manufacturing both in terms of quantity and quality The U S government seems to be allowing no exception as it dismissed last week its own semiconductor company Intel’s plan to increase silicon wafer production in China A solution to this uncomfortable situation is putting a wider gap against competitors in terms of technology and quality so that Chinese companies have no option but to buy semiconductors made by South Korean companies Despite the circumstances, the construction of Yongin semiconductor cluster, in which SK Hynix plans to invest, is being postponed for over a year due to a delay in getting residents’ approval and licensing The government and the political circles should pass a special law to promote the growth of the semiconductor industry along with bolder measures to provide support and ease regulations

美 간 최종건 “현실적으로 中과 파트너십 필요” Choi Jong-kun says partnership with China is needed realistically

在美国访问的崔钟建:“现实中需要与中国的伙伴关系”

최종건 외교부 1차관이 15일(현지 시간) 미국 워싱턴에서 한미 관계를 주제로 열린 전략포럼에서 “중국은 전략적 파트너이며 현실적으로 베이징과의 파트너십이 필요하다”며 한중 관계의 중요성을 강조했다 “China is a strategic partner and we realistically need a partnership with Beijing,“ South Korean Vice Minister of Foreign Affairs Choi Jong-kun said at a strategy forum with a topic of the relationship between South Korea and the U S in Washington, D C on Monday (local time),

韩国外交部第一次官崔钟建当地时间15日在华盛顿举行的以韩美关系为主题的战略论坛上表示,“中国是战略伙伴,现实中需要与北京的伙伴关系”,强调了韩中关系的重要性

이에 미국 행정부 전직 고위당국자들은 “한미 동맹이 장기적으로 약화하고 미국의 정책결정 과정에서 한국이 간과될 위험성이 있다”는 우려를 제기했다 emphasizing the importance of the relationship between South Korea and China Former high-ranking officials of the U S administration raised concerns that the ROK-U S alliance may weaken over time and South Korea may be overlooked in the process of U S ’s policy-making process In his keynote speech at the ROK-U S

对此,美国政府前高级官员表示担忧说:“担心韩美同盟长期削弱,美国在决策过程中可能会忽视韩国”

최 차관은 이날 워싱턴의 싱크탱크 전략국제문제연구소(CSIS)와 한국국제교류재단(KF)이 공동 주최한 한미전략포럼 기조연설에서 우선 한미동맹의 중요성을 역설했다 Strategic Forum co-hosted by the Center for Strategic and International Studies (CSIS), a think tank based in Washington, D C , and the Korea Foundation (KF), Choi first highlighted the importance of the ROK-U S alliance

崔钟建当天在由美国智库“战略与国际问题研究中心”和韩国国际交流财团共同主办的韩美战略论坛上发表主旨演讲,首先强调了韩美同盟的重要性

“한미 두 나라는 21세기의 동맹이 어떤 것인지를 전 세계에 보여주고 있다”며 한미 동맹이 전통적인 안보뿐 아니라 경제, 문화 분야에서도 파트너십을 진전시키고 있다고 평가했다 “South Korea and the U S are showing what a 21st-century alliance is about to the world,” he said, adding that the alliance of the two countries is leading partnership in not only security but also economy and culture

他指出,“韩美两国向全世界展示了21世纪的同盟是什么”,并评价称,韩美同盟不仅在传统安全领域,在经济 文化领域也在发展伙伴关系

그러나 그는 이어진 질의응답에서 중국에 대한 한국의 입장을 묻는 질문에 “그들은 전략적 파트너”라며 “다른 국내정책과 마찬가지로 외교정책 또한 한국인, 한국 중산층의 필요와 이해관계에 맞는 것이어야 한다”고 말했다 To a question asking where South Korea stands regarding China following the speech, he said China is a strategic partner “As with other domestic policies, foreign policies should be aligned with the needs and interests of South Koreans, especially the middle class in the country,” he said

但在接下来的问答环节,当被问及韩国的对华立场时,他答道:“他们是战略伙伴”

이어 “중국과의 교역 규모는 미국 및 일본을 합친 것보다 크고 그 시장에서 오는 큰 수익의 혜택을 즐기는 것은 우리 국민들”이라고 설명했다 “Trade volume with China is bigger than the U S and Japan combined and the beneficiaries of such a market are South Korean people,” he explained

"和其他国内政策一样,外交政策也应该符合韩国人 韩国中产阶层的需要和利害关系”

또 공급망 문제를 거론하며 “중국에서 오는 여러 품목에 대한 의존도는 우리 문제만이 아니라 모두의 문제”라고 지적했다 “Dependency on various products imported from China is not only South Korean but everybody’s issue,” he said on the subject of supply chain issues

他解释说:“与中国的贸易规模比美国和日本的总和还要大,享受从这一市场获得的巨大收益的是我们的国民”

북한 문제에 있어서도 “현실적으로 베이징과 파트너십이 필요하다”며 “우리가 좋든 싫든 간에 그것이 우리 정책의 현실”이라고 했다 （no translation）

他还提到了供应链问题,并指出:“对来自中国的各种产品的依赖程度不只是韩国的问题,而是所有人的问题”

한국이 지리적으로 중국에 가장 가까운 국가임을 상기시키며 “우리는 중국과 좋은 관계를 형성하려고 노력하고 있다”고 했다 （no translation）

他还就朝鲜问题表示:“现实中,我们有必要同北京建立伙伴关系无论我们愿不愿意,这就是我们政策的现实”

최 차관의 질의응답이 끝난 뒤 같은 자리에 패널로 참석한 랜들 슈라이버 전 미국 국방부 아시아태평양 담당 차관보는 “어느 한쪽은 뭔가를 중요하고 핵심적인 도전으로 보는데 다른 한쪽은 이를 받아들이지 않는 동맹관계를 가질 수는 없다”며 “(한국이) 그런 식으로 표류한다면 (한미)동맹이 점차 약화할 수 있다는 점에서 위험할 수 있다”고 했다 （no translation）

他提醒道,韩国是在地理位置上最接近中国的国家,并表示:“我们正在努力与中国建立良好的关系”崔钟建的问答结束后,作为专家出席当天活动的前美国国防部负责亚太事务的部长助理兰德尔·施莱佛说:“如果在一方看来是重要的 核心的挑战,而另一方不接受这种看法,同盟关系是不会拥有未来的”,“如果(韩国)以这种方式漂流,(韩美)同盟就可能逐步弱化,从这一点来看很危险”

中요소수 1만8700t 풀기로 일단 숨통China decides to export 18,700 tons of urea solution to S Korea

暂时松了口气,1.87万吨中国尿素将入境国内救急

중국 정부가 한국 기업들과 계약한 요소수 물량 1만8700t에 대한 수출 절차를 정상적으로 진행하겠다고 우리 정부에 통보했다 The Chinese government notified the South Korean government that it will export 18,700 tons of urea solution contracted with South Korean companies

中国政府向韩国政府通报说,将正常执行与韩国企业签订合同的1.87万吨尿素的出口程序

국내 소요량의 두세 달 치에 해당하는 물량이다 It is enough to cover two to three months of domestic demand

若这批尿素正常到货,可以满足韩国国内2至3个月的需求

이 중 차량용 요소 1만여 t은 이르면 이달 말에서 내달 초 사이 국내에 들어올 것으로 예상된다 일단 요소수 부족 사태에 대한 급한 불은 껐지만 중국이 요소수에 대한 수출 전 검사 제도를 중단하지는 않은 만큼 중국산 요소수 수입이 지속적으로 원활하게 이뤄질지는 미지수다 About 10,000 tons of urea for cars among the amount to be exported by China will arrive in South Korea as early as the end of this month or the beginning of the next month
It will resolve the shortage of urea solution in the short term, however, it still remains uncertain whether urea solution will be imported smoothly from China in the future as the country has not suspended its inspection system of urea solution before export
（（additional translation）） “After communicating with the Chinese government through various channels to
accelerate the import of Chinese urea solution, it has been confirmed that the amount previously contracted with South Korean companies will be imported normally,” the Ministry of Foreign Affairs said on Wednesday As it takes about two weeks from an application for pre-export inspection to its completion, the contracted amount will arrive in South Korea at the end of this month or the beginning of the next month

其中1万多吨车用尿素最早将于本月末至下月初进入国内

외교부는 10일 “중국산 요소 수입 절차의 조속한 진행을 위해 다양한 채널로 중국 측과 소통한 결과 우리 기업들이 이미 계약한 물량(1만8700t)에 대한 수출 절차가 진행될 것임을 확인했다”고 밝혔다 수출 전 검사 신청부터 검사 완료까지 약 2주가 걸리는 것을 감안하면 이달 말에서 내달 초까지 계약 물량이 국내에 들어올 수 있다는 것 또 1만8700t 중 7100t은 수출 전 검사를 신청한 상황이고, 이 중 A사가 수입하는 차량용 요소수 300t은 검사가 완료돼 다음 주 한국으로 들어온다고 외교부가 밝혔다 The ministry also added that applications had been submitted already for 7,100 tons out of 18,700 tons, and 300 tons imported by a company for automobiles have completed an inspection and will arrive in South Korea next week

虽然暂时解决了尿素溶液紧缺事态的燃眉之急,但是中国并没有中断尿素溶液的出口前检验制度,因此中国产尿素溶液的进口能否持续顺利进行还是未知数韩国外交部10日表示:“为了尽快启动中国产尿素的进口程序,通过多种渠道同中方进行了沟通,结果确认将执行韩国企业已签订合同物量(1.87万吨)的出口程序”从出口前的检查申请到检查结束大约需要2周的时间,考虑到这一点,从本月末到下月初,合同量可以进入国内外交部表示,在1.87万吨尿素中,有700吨已申请出口前检查,其中A公司进口的300吨车用尿素已完成检查,将于下周返抵达韩国

다만 중국이 사실상 요소수 수출에 제동을 건 만큼 앞으로 수급이 이전 수준으로 완전히 회복되기는 어려울 것으로 관측된다 However, as China has practically put a brake on its urea solution export, securing supply won’t go back to normal completely

但有观测认为,中国实际上已经停止了尿素出口,因此今后供求将很难完全恢复到以前的水平

외교부 당국자는 “단기적으로 (수출 절차가) 정상화된 것이라고 이해하면 된다”고 말했다 “It is a short-term normalization of the export process,” said a member of the ministry

外交部的一位官员表示:“可以理解为(出口程序)在短期内已经恢复正常”

“中헝다 사태 리스크 국제 경제 위협 우려”Spillover effects of Evergrande liquidity crisis on the global economy

美联储:“担心中国恒大事态的风险威胁国际经济”

미국 중앙은행인 연방준비제도(Fed·연준)가 파산 위기에 몰린 중국의 대형 부동산 회사 헝다(恒大) 사태의 파장이 미국 등 글로벌 경제로 확산될 수 있다고 8일(현지 시간) 경고했다 The U S Federal Reserve warned Monday that the liquidity crisis of Evergrande, China’s real estate developer company, may have spillover effects on the U S and global economy

美国中央银行——联邦储备委员会当地时间8日警告,面临破产危机的中国大型房地产公司恒大的事态可能波及美国等全球经济

올 9월만 해도 제롬 파월 연준 의장이 “헝다 문제는 중국에 국한된 것”이라며 글로벌 위기로의 확산 가능성을 일축한 것에 비하면 미국이 이 사태를 바라보는 시각이 달라진 것으로 풀이된다 The Fed’s change in tone provides a contrast to Federal Reserve Chair Jerome Powell’s statement back in September, which brushed off the concern that the Evergrande situation does not pose a threat beyond China, dismissing a possibility of spillovers to global economy

就在今年9月,美联储主席杰罗姆·鲍威尔还表示,“恒大问题只局限在中国”,否认了扩散为全球金融危机的可能性

연준은 이날 발간한 반기 금융안정보고서에서 “중국의 부동산 문제가 미국 금융 시스템에 리스크를 주고 있다”면서 이같이 짚었다 In its biannual report on financial stability, the Fed warned of the risk posed by China’s property developer Evergrande to the U S financial system

美联储在当天公布的《上半年金融稳定报告》中指出:“中国房地产问题给美国金融系统带来了风险”

연준은 보고서에서 “중국 경제와 금융 시스템의 규모, 전 세계와 무역 연계도 등을 감안했을 때, 중국 금융의 불안은 위기 심리의 악화를 통해 글로벌 금융시장에 압박을 주고 글로벌 경제 성장을 위협하며 미국에도 영향을 줄 수 있다”고 분석했다 “Given the size of China’s economy and financial system as well as its extensive trade linkages with the rest of the world, financial stresses in China could strain global financial markets through a deterioration of risk sentiment, pose risks to global economic growth, and affect the United States,” analyzed the Fed

美联储在报告中表示:“考虑到中国经济和金融系统的规模 其与全世界的贸易联系等方面,中国金融的不稳定可能通过心理上的恶化,给全球金融市场带来压力,威胁全球经济增长,对美国也会有影响”

연준은 또 “중국은 기업과 지방정부의 부채가 여전히 크고 금융 부문의 레버리지(차입금을 통한 투자)가 높으며 부동산 가치도 지나치게 오른 상태”라며 “이런 환경에서 중국이 레버리지가 높은 기관에 대한 규제에 집중할 경우, 헝다 사태에서 보듯이 부동산 섹터 등 부채가 높은 기업들에 위기를 초래할 가능성이 있다”고 했다 “In China, business and local government debt remain large the financial sector’s leverage is high, especially at small and medium-sized banks and real estate valuations are stretched,” the report further pointed out “In this environment, the ongoing regulatory focus on leveraged institutions has the potential to stress some highly indebted corporations, especially in the real estate sector, as exemplified by the recent concerns around China Evergrande Group,” the Fed stated in the report

美联储还表示:“中国企业和地方政府的负债仍大,通过金融部门的杠杆(借款投资)高,房地产投资价值也过分上涨”,“在这样的环境下,如果中国集中对高杠杆机关进行限制,就像恒大事件所示,可能给房地产领域等高负债企业造成危机”

헝다그룹은 그간 차입금을 바탕으로 공격적으로 사업을 확장해 왔지만 최근 당국이 부동산 규제에 나서면서 유동성 위기에 처한 상태다 Evergrande Group borrowed heavily to support its aggressive business expansion until the Chinese government began a crackdown on real estate debt, prompting a liquidity crisis

恒大集团此前一直以贷款为基础积极扩张事业,但最近随着政府出面限制房地产,陷入了流动性危机

연준은 이어 “위기가 금융회사에 전이되고 부동산 가격이 갑작스러운 조정을 겪거나 투자자들의 위험 감수 성향이 줄어들 경우 중국의 금융 시스템이 큰 압력을 받을 수 있다”고 덧붙였다 “Stresses could, in turn, propagate to the Chinese financial system through spillovers to financial firms, a sudden correction of real estate prices, or a reduction in investor risk appetite,” the Fed said

美联储还表示:“如果危机转移到金融公司,房地产价格突然发生调整,投资者的风险承受倾向减少,中国的金融体系可能会受到很大压力”

내년 1월 RCEP 발효… 日 “韓中과 첫 FTA 기대” RCEP to take effect in next January

RCEP明年1月生效……日本:“期待与韩国 中国的首个自贸协定”

내년 1월부터 중국, 일본 등 10개국 간에 역내포괄적경제동반자협정(RCEP)이 발효되면서 일본에서 경제 활성화에 대한 기대감이 나오고 있다 The Regional Comprehensive Economic Partnership, or RCEP, will come into force in January 2022, among 10 countries, including China and Japan

随着中国 日本等10个国家之间的区域全面经济伙伴关系协定(RCEP)将从明年1月起生效,日本国内出现了对搞活经济的期待

RCEP 발효로 일본 국내총생산(GDP)이 2.7% 상승할 것이라는 전망도 나왔다 Japan is particularly excited about economic invigoration, with its GDP projected to increase by 2.7%

有预测认为,随着RCEP的生效,日本国内生产总值将增长2.7%

한국도 RCEP에 서명했지만 아직 국회 비준이 끝나지 않아 내년 1월 발효국에는 포함되지 않았다 South Korea has signed the RCEP, but because it is yet to be ratified by the National Assembly, South Korea is not included in the list of countries where the RCEP will come into force

韩国虽然也在RCEP上签了字,但由于国会还没有批准,没有被包括在明年1月生效的国家之中

일본 외무성은 “호주와 뉴질랜드가 2일 RCEP 협정을 비준하면서 발효 조건이 충족됐다”며 “내년 1월 1일부터 일본, 중국, 호주, 뉴질랜드, 싱가포르, 베트남, 태국, 브루나이, 캄보디아, 라오스 등 10개국에서 RCEP가 발효된다”고 3일 발표했다 “With the ratification of the RCEP by Australia and New Zealand on Tuesday, the requirements for the regional trade pact to take effect have been satisfied,” stated the Japanese foreign ministry on Wednesday “From January 1, 2022, the RCEP will come into force in 10 countries, including Japan, China, Australia, New Zealand, Singapore, Vietnam, Thailand, Brunei, Cambodia, and Laos,”

日本外务省3日宣布:“随着澳大利亚和新西兰2日批准RCEP,RCEP生效的条件已经充分”,“从明年1月1日起,RCEP将在日本 中国 澳大利亚 新西兰 新加坡 越南 泰国 文莱 柬埔寨 老挝等10个国家生效”

우리나라를 비롯해 인도네시아, 말레이시아, 미얀마, 필리핀 등 5개국은 아직 비준서를 기탁하지 않았다 said the ministry Five countries including South Korea, Indonesia, Malaysia, Myanmar, and the Philippines have not deposited the ratification instrument

包括韩国在内,印度尼西亚 马来西亚 缅甸 菲律宾等5个国家尚未提交批准文件

RCEP는 가맹국 간에 상호 관세를 내려 역내 통상을 활성화하는 다국 간 자유무역협정(FTA)이다 The regional free trade agreement includes tariff reductions to promote regional trade

RCEP是成员国之间相互降低关税以活跃区域内贸易的多国间自由贸易协定

15개 서명국이 모두 비준을 마치게 되면 RCEP는 미국이 빠진 채 11개국이 참여 중인 포괄적·점진적 환태평양경제동반자협정(CPTPP)보다 규모가 큰 세계 최대 FTA가 된다 Once all 15 signatories ratify the instrument, the RCEP will become the world’s largest FTA, bigger in size than the Comprehensive and Progressive Agreement for Trans-Pacific Partnership (CPTPP), composed of 11 countries except the U S

如果15个签署国全部完成批准程序,RCEP将成为比没有美国参与 有11个国家签署的《全面与进步跨太平洋伙伴关系协定》(CPTPP)规模更大的世界第一大自贸协定

전체 무역 규모는 2019년 기준 5조6000억 달러(약 6600조 원·전 세계의 31.9%), GDP는 26조 달러(30.8%), 인구는 22억7000만 명(29.7%)이다 As of 2019, the RCEP covers a total trade volume of 5 6 trillion dollars (approximately 6,600 trillion won, taking up 31 9% of the world’s trade volume), a market of 2.27 billion people, roughly 29.7% of the world’s population, and 26 trillion dollars of GDP (30.8%)

以2019年为准,成员国整体贸易规模达5.6万亿美元(约6600万亿韩元,占全球31.9%),国内生产总值达26万亿美元(30.8%),人口22.7亿人(29.7%)

RCEP는 한중일 세 나라가 참여하고 향후 발전 가능성이 큰 동남아국가연합(ASEAN)이 포함된 FTA라는 점에서 주목을 받고 있다 The RCEP is expected to generate significant economic benefits, as it is an FTA that is joined by South Korea, China, and Japan, as well as ASEAN, which has a huge growth potential

RCEP是包括韩中日三国 今后发展潜能巨大的东盟在内的自贸区,因此备受关注

일본 정부 대변인인 마쓰노 히로카즈(松野博一) 관방장관은 4일 기자회견에서 “세계의 성장 센터 지역들과 우리나라의 연결로 지금보다 더 경제성장에 기여할 것”이라며 “(일본이) 주도적인 역할을 해 나가겠다”고 했다 “The RCEP will contribute more than ever to its economic development by connecting the world’s growth hub and Japan,” Chief Cabinet Secretary Hirokazu Matsuno, the Japanese government's top spokesperson, said in a press conference held on Thursday

日本政府发言人 官房长官松野博一4日在记者会上表示:“随着世界经济增长中心地区与我国连接,将为经济增长作出比现在更大的贡献”

니혼게이자이신문은 “일본이 한국, 중국과 맺은 첫 FTA가 움직이기 시작한다”며 “일본 GDP를 약 2.7% 끌어올리는 효과가 전망된다”고 분석했다 “Japan will take the lead,” said Mr Hirokazu The Nikkei reported that the first FTA signed by Japan with its counterparts South Korea and China will begin to take effect, stating, “[RCEP] is expected to increase Japan’s GDP by 2.7%

(日本)将发挥主导作用”《日本经济新闻》分析说:“日本与韩国和中国签订的第一个自贸协定将开始启动预计将带来将日本国内生产总值提高2.7%左右的效果”

요미우리신문은 “참가국은 경제 회복에 대한 기대감이 커지는 한편으로 아시아태평양 지역의 통상 분야에서 주도권을 잡으려는 중국에 대한 경계감도 높아지고 있다”고 전했다 ” The Yomiuri Shimbun stated that Nations joining the RCEP expect to see economic recovery at the same time, China is being checked by other countries, as it tries to take the hegemonic position in trade in the Asia-Pacific region

《读卖新闻》报道说:“参加国对经济复苏的期待越来越大,同时对想要在亚太地区贸易领域掌握主导权的中国的警戒心也越来越高”

인도도 RCEP 참여를 타진했으나 최종 서명에서 빠졌다 중국과의 무역에서 적자에 시달려 온 인도는 값싼 중국 제품의 공세가 거세질 것을 우려해 참여하지 않기로 했다 India deliberated on joining the RCEP, but it opted out of joining it out of concern that the RCEP would accelerate a flood of cheap Chinese imports, thereby exacerbating India’s trade deficit against China

印度也曾试图参与RCEP,但在最终签名时置身在外印度在与中国的贸易中饱受逆差困扰,担心廉价的中国产品的攻势会更加猛烈,所以决定不参与

미군 2인자 “中군사력 증강 충격, 이대로면 美 추월” Gen Hyten: China’s military progress is ‘stunning’

美军第二号人物:“中国增强军事力量令人震惊,再这样下去会赶超美国”

미군 서열 1위인 마크 밀리 합참의장이 중국의 극초음속 미사일 발사를 확인하며 이에 대한 우려를 나타낸 데 이어 존 하이튼 합참차장도 중국의 군사력 증강 속도를 두고 “충격적(stunning)”이라고 했다 Following Gen Mark Milley, chairman of the Joint Chiefs of Staff, who expressed concern over China’s test of a hypersonic missile, Gen John Hyten, vice chairman of the Joint Chiefs of Staff, said China’s growing military prowess is “stunning

继美军第一号人物 参谋长联席会议主席马克·米利确认中国发射高超音速导弹并表示担忧后,参谋长联席会议副主席约翰·海滕也就中国增强军事力量的速度表示“令人震惊(stunning)”

CNN 등에 따르면 하이튼 합참차장은 21일(현지 시간) “중국이 움직이는 속도는 충격적이다 이 속도와 궤적대로라면 중국은 러시아와 미국을 따라잡게 될 것”이라고 말했다 ” According to CNN, Gen Hyten said that the pace at which China is moving is stunning, adding that China will surpass Russia and the U S considering the pace China is moving and the trajectory it is on

美国有线电视新闻网(CNN)等媒体报道说,海滕当地时间21日表示:“中国的行动速度令人震惊按照这一速度和轨迹,中国将赶上俄罗斯和美国”

또 “이는 게임(의 판도)을 바꾸게 된다는 점에서 미국뿐 아니라 우리 동맹국의 문제이기도 하다”고 강조했다 “It’s not just the United States but the United States and our allies because that’s the thing that really changes the game,” Gen Hyten said

他强调:“这将改变游戏格局,这不仅是美国的问题,也是我们同盟国的问题”

그의 설명에 따르면 최근 5년간 중국이 수백 회의 극초음속 미사일 시험발사를 한 데 비해 미국은 9회밖에 하지 않았다 According to Gen Hyten, the U S has carried only nine hypersonic tests in the last five years whereas China has done hundreds of them

据海滕介绍,中国在最近5年里试射了数百枚高超音速导弹,而美国只发射了9枚

이런 큰 차이를 두고 그는 “좋지 않다”고 지적했다 “Single digits versus hundreds is not a good place,” Hyten pointed out

对于这一巨大差异,他指出,“这不好”

미국의 발사 실패에 대해선 “학습 과정으로 봐야 한다”며 “위험을 감수하고 실패에서 배워야 더 빨리 전진하는 것”이라고 했다 In regard of missile test failures of the U S , Hyten said, “Failure is part of the learning process,” adding the U S will move fast by taking risks and learning from failures

他就美国发射失败表示:“应该将其视为学习过程只有敢于冒险并从失败中学习,才能更快地前进”

하이튼 합참차장은 그러면서 김정은 북한 국무위원장이 과거의 실패한 시험발사를 통해 개발에 속도를 내는 법을 배웠다며 북한을 사례로 들었다 The vice chairman of the Joint Chiefs of Staff cited North Korean leader Kim Jong Un as an example, arguing Kim has learned the lesson of failed tests to speed up development

他还以朝鲜为例表示:“朝鲜国务委员长金正恩通过过去失败的试验发射,学会了加快开发速度”

김정은은 과학자와 엔지니어들이 실패했을 때도 아버지(김정일)와 달리 이들을 처형하지 않기로 결정했고 오히려 격려하며 실패에서 배우기도 했다는 것이다 Unlike his father (Kim Jong Il), Kim Jong Un has decided not to kill scientists and engineers when they failed and instead encourage them to learn by failing

据说,在科学家和工程师试验失败时,金正恩也和其父亲(金正日)不同,决定不处死他们,反而鼓励他们,从失败中学习

그는 “결국 그렇게 해서 세계 경제 순위 118위 국가인 북한이 대륙간탄도미사일(ICBM) 역량을 확보했다”고 덧붙였다 As a result, the 118th biggest economy in the world was able to build an ICBM nuclear capability

海滕说:“最终,世界经济排名第118名的朝鲜拥有了洲际弹道导弹力量”

공급난 美中 가격 줄인상…Amid supply chain crisis, product price increases in U S and China

受全球供应难的影响中美物价持续上涨,韩国进口物价也亮起红灯

한국도 수입물가 빨간불세계적으로 확산하고 있는 공급 대란의 여파로 미국과 중국을 비롯한 글로벌 경제의 인플레이션 압력이 커지고 있다 극심한 구인난과 공급망 위기에 처한 미국 기업들은 비용 증가를 견디다 못해 제품 가격을 줄줄이 인상하고 있다 The supply-chain disaster is putting inflationary pressure on global economy, including the U S and China U S businesses hit by labor shortage and supply chain crisis are raising product prices, after having endured ever-rising costs

受全球范围内的供应大乱的影响,美国和中国等全球经济的通货膨胀压力正在增大面临严重的招聘难和供应链危机的美国企业因无法承受费用的增加,正在接连上调产品价格

최근 전력난과 원자재 가격 급등의 직격탄을 맞은 ‘세계의 공장’ 중국도 물가가 크게 뛰었다 China, which is dubbed as the world’s factory, has been hit by electricity outage and rising costs of raw materials, has seen a huge increase in inflation

最近遭受电力难和原材料价格暴涨直接打击的“世界工厂”中国的物价也大幅上涨

중국의 인플레이션이 전 세계로 전이될 위험이 커진 가운데 한국 역시 수입물가에 비상이 걸렸다 As China’s inflation is likely to spread to the entire world, South Korea has been alarmed by import prices

在中国的通货膨胀扩散到全世界的危险增大的情况下,韩国的进口物价也进入了紧急状态

공급망 위기가 경기 회복을 저해함에 따라 미국의 3분기(7∼9월) 경제성장률은 앞선 분기의 반 토막 수준에 불과할 것이란 전망도 나오고 있다 Critics forecast that economic recovery interfered by supply chain crisis, U S ’s economy growth in the third quarter might be expected to be half of the previous quarter

有人预测说,随着供应链危机阻碍经济复苏,美国第三季度(7至9月)的经济增长率仅为前一季度的一半水准

27일(현지 시간) 뉴욕타임스(NYT)와 로이터통신 등에 따르면 이날 3분기 실적을 발표한 미국의 주요 기업들이 일제히 제품 가격 인상을 예고하고 나섰다 According to The New York Times and Reuters on Wednesday, major U S companies, which disclosed their third quarter earnings on the day, has hinted price rise

27日(当地时间),据《纽约时报》(NYT)和《路透社》等媒体报道,当天公布第三季度业绩的美国主要企业一致预告将上调产品价格

원자재와 인건비 상승에 따른 비용 증가분을 소비자에게 결국 전가하고 있는 것이다 They shift an increase in costs due to rise in commodity prices and labor costs to consumers

也就是说,将原材料和人工费上涨带来的费用增加部分最终转嫁给消费者

패스트푸드 체인 맥도날드는 인건비와 원자재 가격 상승분을 반영하기 위해 올해 제품 가격을 6%가량 인상한다고 밝혔다 McDonald’s announced that it would raise menu prices by about 6% due to increased ingredient and labor costs

快餐连锁店麦当劳表示,为了反映人工费和原材料价格的上涨部分,今年将把产品价格上调6%左右

맥도날드는 구인난으로 인해 올해 인건비가 벌써 10% 이상 올랐고 음식 재료와 기타 자재 값도 최대 4% 증가하는 등 비용 압박을 받고 있는 것으로 알려졌다 The fast-food giant is reportedly under heavy cost pressure from labor shortages that drove the company’s labor costs up by more than 10%, along with a maximum of 4% increase in the prices of ingredients and other materials

据悉,麦当劳因招聘难,今年的人工费已经上涨了10%以上,食品材料和其他材料的价格也最多增加了4%,正在承受费用压力

물가 상승에 대응해 제품 가격을 이미 1.5% 올린 식품기업 크래프트하인즈는 내년에도 이런 가격 정책 기조를 이어가겠다고 밝혀 추가적인 가격 인상 가능성을 내비쳤다 Kraft Heinz that already increased its product price by 1.5% in response to inflation stated that it would maintain its pricing strategy well into next year, implying a possibility of further price raise

为应对物价上涨,已经将产品价格上调1.5%的食品企业KraftHeinz表示,明年也将继续保持这种价格政策基调,暗示了进一步上调价格的可能性

코카콜라 역시 이날 실적을 발표하면서 “인건비와 물류비용이 높게 유지된다면 필요에 따라 가격을 올릴 수 있다”고 발표했다 Coca Cola announced in its earning report that it may increase the price of its beverages in the face of high labor and logistics costs

可口可乐也在当天公布业绩时表示:“如果人工费和物流费用保持较高水平,可以根据需要上调价格”

글로벌 제조기업 3M도 “폴리프로필렌 등 원자재 비용과 인건비 상승에 직면하고 있다”면서 “인플레이션과 공급망 압력에 대응하기 위해 제품 가격을 인상할 것”이라고 했다 3M, a global consumer goods company, also stated that it is facing higher costs related to polypropylene and high labor costs and that it would increase the price of its products to respond to inflation and supply chain pressure

全球制造企业3M也表示:“聚丙烯等原材料费用和人工费面临上涨为应对通货膨胀和供应链压力,将上调产品价格”

기업들의 이 같은 가격 인상 행렬은 최근 이례적인 인력난과 공급망 위기가 주요 원인이다 The price rally of many global companies is attributable to the unprecedented labor shortages and supply chain crisis

企业之所以出现这种上调价格的现象,主要原因是最近出现了罕见的人力难和供应链危机

미국에서는 월별 구인 건수가 계속 1000만 명을 넘을 정도로 일손이 많이 필요한 상황이지만 정작 일할 사람이 부족해 기업들이 앞다퉈 임금을 올리고 있는 실정이다 The number of monthly job openings in the U S has soared above 10 million, yet labor shortages are driving companies to raise wages

在美国,每月招聘人数持续超过1000万人,需要很多人手,但由于真正做事的人不足,各企业争先恐后地提高工资

또 항만과 육상 물류에 병목 현상이 생기면서 기업들은 단가가 비싼 항공 화물에 대한 의존도가 높아졌고 아예 자체 화물선을 띄우는 곳도 등장하고 있다베이징올림픽, 방역 위해 중국 본토 관중만 입장중국이 내년 2월 베이징 겨울올림픽을 최소 규모로 치르겠다고 밝혔다 A bottleneck at ports and land logistics have caused businesses to heavily rely on a high-rate air freight, and some businesses are even chartering their own container ships Spectators residing in China to be allowed entry at Beijing Olympics China has announced that the Beijing Winter Olympics set to start in February next year will be held on a minimum scale

另外,随着港口和陆地物流出现瓶颈现象,企业对单价较高的航空货物的依赖度提高,甚至出现了干脆启用自己的货船的企业北京冬奥会,处于防疫考虑将只允许中国本土观众入场中国方面表示,明年2月将以最小规模举办北京冬奥会

불필요한 활동과 절차를 줄이고 올림픽에 투입되는 인력도 대폭 감축할 방침이다 신종 코로나바이러스 감염증(코로나19) 확산을 막기 위한 조치다 It plans to reduce unnecessary activities and procedures and greatly cut down on the number of staff for the Olympics as a way to prevent the spread of COVID-19

政府计划减少不必要的活动和程序,大幅减少投入到奥运会的人力这是为了防止新型冠状病毒肺炎(COVID-19)疫情的扩散而采取的措施

26일 관영 신화통신 등 중국 매체들에 따르면 베이징 겨울올림픽 조직위원회는 전날 올림픽 방역 수칙이 담긴 방역수첩(매뉴얼)을 공개했다 1차로 공개된 이번 방역 매뉴얼은 선수와 대회 관계자들에게 적용되며 앞으로 한두 차례 더 보완될 수 있다 The Beijing Organizing Committee for the 2022 Olympic and Paralympic Games released Monday the first edition of the official manual containing COVID-19 infection prevention and control rules for the Olympics, according to the Chinese media The manual applies to athletes and officials and there could be further editions

26日,据《新华社》等中国媒体报道,北京冬奥会组委会在前一天公开了包含奥运会防疫守则的防疫手册(manual)第一次公开的此次防疫手册适用于运动员和大赛相关人士,以后还可以补充一两次

매뉴얼에 따르면 이번 올림픽을 위해 마련한 전용 교통편과 숙소, 부대시설, 경기장과 훈련장 등 모든 공간은 하나로 연결돼 운영된다 According to the manual, Olympics will be held in a “closed-loop” management system, where transportation, accommodation, facilities, stadiums and training centers are connected and operated as one

根据手册,为此次奥运会准备的专用交通工具和宿舍 附属设施 赛场和训练场等所有空间将连接在一起运营

이 공간들은 마치 거품(버블)을 덮어씌운 것처럼 다른 외부 공간과는 완전히 격리된 폐쇄 구역이 된다 As if covered with a bubble, participants will stay in closed areas that are completely isolated from outside

这些空间就像覆盖了泡沫一样,成为与其他外部空间完全隔离的封闭区域

대회 참가자들은 제한된 공간만 오갈 수 있으며 매일 코로나19 검사를 받아야 한다 Participants are allowed to move to and from limited spaces and must be tested for COVID-19 on a daily basis

大会参赛选手只能往返有限的空间,每天都要接受新冠病毒检查

코로나19 백신 접종을 완료하지 못한 대회 참가자는 중국 도착 후 21일간 베이징에서 격리해야 한다 Unvaccinated participants will have to serve a 21-day quarantine upon arrival in Beijing

未能接种新冠肺炎疫苗的参赛选手抵达中国后,将在北京隔离21天

올림픽 투입 인력을 최소화할 방침이어서 선수단 편의를 위해 활동했던 자원봉사자들도 사라질 것으로 보인다 With the Beijing Olympics set to be held with limited number of personnel, volunteers, who used to work for the convenience of athletes, are likely to disappear from Olympic venues as well

由于计划将投入奥运会的人力最小化,因此,为方便选手团而活动的志愿者也将消失

무관중으로 치러진 2020 도쿄 올림픽과 달리 관중을 입장시킬 계획이지만 규모를 최소화할 방침이며 중국 본토 밖에서 온 관중은 수용하지 않는다 Unlike the 2020 Tokyo Olympics, which were held without spectators, limited number of spectators will be allowed entry at the Beijing Olympics But those from outside mainland China will not be allowed to attend the Games

与以无观众的形式举行的2020年东京夏季奥运会不同,虽然计划让观众入场,但计划将规模最小化,不接纳来自中国本土以外的观众

베이징 올림픽을 앞두고 미국과 유럽연합(EU)의 집단 보이콧 움직임도 지속되고 있다 Meanwhile, actions to boycott the Beijing Olympics are continuing in the U S and the European Union (EU) ahead of the upcoming Olympics

面对北京冬奥会,美国和欧盟(EU)的集体抵制动向也在持续

하지만 전문가들은 보이콧이 실제로 이뤄질 가능성이 높지 않다고 보고 있다 Experts, however, say boycott is unlikely to actually happen

但专家认为,实际上抵制的可能性不大

김흥규 아주대 국제학부 정치외교학과 교수는 “미국이 최근 중국에 유화적인 모습을 보이고 있다”며 “중국과의 불화는 미국 여론에 악영향을 미칠 수 있다 “The U S is recently giving conciliatory signals to China,” said Kim Heung-gyu, a political science professor and Diplomacy at Ajou University

亚洲大学国际学部政治外交系教授金兴圭表示:“美国最近对中国表现出柔和的态度与中国的不和可能会对美国舆论产生负面影响”

내년 중간선거를 앞둔 조 바이든 정부가 그런 위험을 감당하려 하진 않을 것”이라고 분석했다 “Discord with China could have a negative impact on the Biden administration within the U S Washington will not be willing to take that risk ahead of next year’s midterm elections

面临明年中期选举的乔?拜登政府不会愿意承担这样的风险

일본인 90% “中 싫어”…중국인 66% “日 싫어”” 90% of Japanese ‘dislike China’ and 66% of Chinese ‘dislike Japan

”90%的日本人“讨厌中国”……66%的中国人“讨厌日本”调查显示,10名日本人中有9人对中国的印象是“不好”

일본인 10명 중 9명은 중국에 대한 인상을 “좋지 않다”고 답한 것으로 나타났다 일본에 대한 인상을 “좋지 않다”고 답한 중국인도 66. 1%다 In a recent survey, nine out of 10 Japanese answered they don’t have a positive impression of China while 66. 1 percent of Chinese expressed the same feeling toward Japan

中国人中对日本的印象回答“不好”的也占66.1%

중일 양국은 내년 9월 국교정상화 50주년을 앞두고 있지만 올해 양국 모두 상대에 대한 감정이 전년보다 악화됐다 It will be the 50th anniversary of the normalization of diplomatic relations between the two countries next September but their sentiment toward each other has worsened since last year

中日两国明年9月将迎来邦交正常化50周年,但今年两国都对对方的感情比前一年恶化

21일 마이니치신문에 따르면 일본의 비영리단체 겐론NPO와 중국 국제출판집단은 8월 21일∼9월 25일 18세 이상 남녀 1000명(일본), 1547명(중국)을 대상으로 상대국에 대한 인식을 조사했다 According to the Mainichi Shimbun on Thursday, Japanese non-profit organization Genron NPO and a Chinese group of international publishers conducted a survey of 1,000 Japanese and 1,547 Chinese aged 18 or over on the perception of each other from August 21 to September 25

据《每日新闻》21日报道,日本的非营利团体“言论NPO”和中国国际出版集团8月21日至9月25日以1000名18岁以上(日本)男女和1547名(中国)男女为对象,就对对方国家的认识进行了调查

일본인 가운데 중국에 부정적인 인상을 가진 사람은 지난해보다 1. 2%포인트 늘어난 90. 9%였다 2016년 조사에서 91. 6%로 조사를 시작한 이후 가장 높았는데 5년 만에 다시 90%를 넘었다 중국인 가운데 일본에 부정적인 인상을 가진 사람은 지난해보다 13. 2%포인트 급증한 66. 1%였다. The result was that 90. 9 percent of Japanese respondents had a negative perception of China, which is 1. 2 percentage points higher than that of last year The figure was the highest in 2016 at 91. 6 percent since the beginning of the survey and this was the first time it went over 90 percent in five years Among Chinese respondents, 66. 1 percent had a negative perception of Japan, up 13.2

在日本人中,对中国持否定态度的人比去年增加1.2个百分点,达到90.9%这是自2016年开始调查(91.6%)以来的最高值,也是5年后再次超过90%在中国人中,对日本持否定态度的人比去年增加13.2个百分点,达到66.1%

대일(對日) 부정적 인상은 센카쿠열도(중국명 댜오위다오) 갈등으로 양국이 충돌했던 2013년 90. 1%로 최고를 찍은 뒤 꾸준히 줄었지만 1년 만에 크게 늘어났다 2 percentage points from the previous year Anti-Japan sentiment was at its peak in 2013 at 90. 1 percent when the two countries collided regarding the Senkaku Islands in Japanese or the Diaoyudao Islands in Chinese and had been on a consistent decline since then before surging this year

对日负面印象在因尖阁列岛(中国称钓鱼岛)矛盾两国发生冲突的2013年达到90.1%后持续减少,但一年之后就大幅增加

구도 야스시(工藤泰志) 겐론NPO 대표는 20일 기자회견에서 “상호 군사적인 위협만 논의되고 양국 국민의 불안이 방치되고 있다”고 분석했다 “The two countries have only made military threats while neglecting their people’s anxiety,” Genron NPO’s CEO Yasushi Kudo said at a press conference on Wednesday

言论NPO负责人工藤泰志20日在记者会上分析说:“只讨论相互军事威胁,两国国民的不安被放置一边”

중일 국민이 갖고 있는 상대국에 대한 부정적 인식은 한일 국민보다 높았다 The negative perception of each other between Chinese and Japanese was more severe than the one between Korean and Japanese

中日国民对对方国家的否定认识高于韩日国民

겐론NPO가 한국 동아시아연구원(EAI)과 실시한 한일 국민 상호인식 조사 결과에 따르면 한국인이 갖고 있는 일본에 대한 부정적 인상은 지난해 71. 6%에서 올해 63. 2%로 줄었다 In a similar survey conducted by Genron NPO and East Asia Institute in South Korea, the share of South Koreans who had a negative impression of Japan decreased from 71. 6 percent last year to 63. 2 percent this year

言论NPO与韩国东亚研究院实施的韩日国民相互认识调查结果显示,韩国人对日本的否定印象从去年的71.6%减少到今年的63.2%

일본인이 가진 한국에 대한 부정적 인상은 같은 기간 46. 3%에서 48. 8%로 소폭 증가하는 데 그쳤다. Meanwhile, Japanese anti-Korean sentiment only increased a bit from 46. 3 percent to 48. 8 percent during the same period

同期,日本人对韩国的负面印象从46.3%小幅增加到48.8%

2015년 이후 일본인의 반중 감정은 반한 감정보다 2배 가까이 높은 상태가 지속되고 있다 Japanese anti-China sentiment has been almost twice higher than their anti-Korean sentiment since 2015

2015年以后,日本人的反华情绪处于持续比反韩情绪高出近2倍的状态

(（additional translation））Korea’s first space launch vehicle successfully lifts off Korea’s first space launch vehicle (KSLV-2), also known as Nuri, lifted off on Thursday from the Naro Space Center in Goheung County, South Jeolla Province The rocket soared to space, following the separation of the first-stage rocket, paring, and the second-stage rocket From design to development, production, and takeoff, the Nuri has been made with Korea’s homegrown rocket technology In the history of space rocket development, only 28 percent of the vehicles succeeded in the first launch The Naro has its first stage rocket that was built in Russia As the space rocket succeeded in the third attempt, it was loaded with a dummy satellite in contingency for failure South Korea is a late mover in the global space race due to many constraints arising from complicated security environment surrounding the Korean Peninsula In particular, the South Korea Ballistic Missile Range Guidelines shackled its development of not only military missiles but also the development of private spaceflights Thankfully, the Missile Guidelines were amended one by one, paving the way for the nation to realize its space ambitions, were completely abolished in the ROK-US summit held in May South Korea can now participate in the Artemis Accords, an American-led initiative for the Moon exploration The success of the Nuri will mark the first step for South Korea to become a leader in space exploration The nation will begin developing various private and military satellites, a Korea-specific global positioning system (GPS), and space materials, components, and gear development There is still a long way to go, as it is an industry requiring expensive costs and highly sophisticated technology However, space is infinitely huge, offering infinite opportunities for growth as such, we cannot afford to be reluctant The space industry has been expanding infinitely, from satellites and space launch vehicles to space tourism, air mobility, space Internet, and space debris treatment Countries that are leading in the sector are fiercely competing to get ahead of others The U S , moving beyond national security and technological competition, has kicked off private space tourism, and China has achieved remarkable growth in space development by building its own space station, sending astronauts into space and its aircraft on Mars Based on the achievements of the Nuri, South Korea should also begin in earnest its journey into space

“中, 8월 극초음속 미사일 시험발사… 美 놀라게 했다”China test-fired hypersonic missile, says Financial Times

《金融时报》:“中国8月份试射高超音速导弹……令美国大吃一惊”

중국이 8월 핵무기를 탑재할 수 있는 극초음속 미사일을 비밀리에 시험발사한 것으로 드러났다고 영국 파이낸셜타임스(FT)가 소식통을 인용해 16일 보도했다 China secretively test-fired in August a hypersonic missile that can have a nuclear warhead mounted, the Financial Times reported Saturday by quoting an informed source

英国《金融时报》16日援引消息人士的话报道说,中国于8月秘密试射了可搭载核武器的问超音速导弹

FT에 따르면 중국의 극초음속활공체(HGV)는 목표물에서 약 32km 거리에 떨어졌다 According to the U K daily, China’s hypersonic glider vehicle (HGV) flew before falling about 32 kilometers off the target

据《金融时报》报道,中国的高超音速滑翔飞行器(HGV)落在距离目标约32公里处

FT는 “중국 극초음속 무기의 진전을 보여준 이 테스트는 미국 정보기관을 놀라게 했다”고 전했다 “The test demonstrated the advancement of China’s hypersonic weapons, which has alarmed U S intelligence authorities,” the Financial Times said

《金融时报》说:“该测试显示了中国高超音速武器的进展,令美国情报机构大吃一惊”

중국이 극초음속 미사일을 보유한 것은 이미 알려졌지만 이번 시험발사로 미국이 알고 있는 것보다 중국의 극초음속 무기 관련 기술이 발전했다는 사실이 입증되었기 때문이다 중국 핵무기 정책 전문가인 테일러 프레이블 매사추세츠공대(MIT) 교수는 FT 인터뷰에서 “핵탄두를 탑재한 극초음속 미사일이 완성되면 미국의 미사일방어(MD) 체계를 무용지물로 만들 것”이라고 말했다 The fact China possesses a hypersonic missile was known already, but the test firing has demonstrated that China’s hypersonic weapons technology is more advanced than what Washington previously estimated

中国拥有高超音速导弹的事实早已为人所知,但通过此次试射,证明了中国的高超音速武器相关技术比美国所知道的要先进中国核武器政策专家 麻省理工学院教授泰勒·弗雷布尔在接受《金融时报》采访时表示:“搭载核弹头的高超音速导弹一旦完成,美国的导弹防御体系将成为无用之物”

극초음속 미사일은 음속의 5배에서 최대 20배 이상까지 속도를 내는 미사일로 전쟁의 판도를 바꿀 수 있는 ‘게임 체인저’로 불리는 첨단 무기다 The state-of-the-art weapons system, which is capable of flying at five to 20 times the speed of sound, is known as a game-changer in warfare

高超音速导弹的速度是音速的5倍到最多20倍以上,是可以改变战争格局的被称为“改变游戏规则者”的尖端武器

탄도미사일은 우주로 높이 올라 포물선을 그리며 날아가지만 극초음속 미사일은 대기 중에서 낮은 궤도로 날아가다가 목표물을 빠르게 타격한다 A ballistic missile soars into the space to fly in an arc, but a hypersonic missile flies at a low trajectory in the atmosphere before hitting the target instantly

弹道导弹上升至太空,画着抛物线飞行,但高超音速导弹在大气中以低轨道飞行,快速打击目标

순항미사일과 탄도미사일을 겨냥한 각국의 MD 체계를 무력화시킬 것으로 평가되고 있다 Experts say that the missile could incapacitate missile defense systems of various countries that are designed to target cruise missiles and ballistic missiles

有评价认为,这将使针对巡航导弹和弹道导弹的各国导弹防御系统失效

전 세계 어느 곳이든 1∼2시간 안에 타격할 수 있고 미사일방어망을 뚫을 수 있어 미국, 중국, 러시아 등 군사 강대국들은 극초음속 미사일 개발에 앞장서고 있다 Hypersonic missiles are capable of hitting a target anywhere in the world within one to two hours, and penetrating missile defense systems

高超音速导弹可以在1～2个小时里对全世界任何地方进行打击,能够突破导弹防御网,因此美国 中国 俄罗斯等军事强国正在带头开发高超音速导弹

북한도 지난달 28일 극초음속 미사일 ‘화성-8형’ 시험발사를 진행하며 경쟁에 가세했다 As such, global military powerhouses including the U S , China and Russia are racing to develop hypersonic missiles North Korea also joined the foray to develop such a missile by test-firing the Hwasong-8 hypersonic missile on Sept 28

朝鲜也于上个月28日试射了高超音速导弹“火星-8型”,加入了竞争行列

중국은 2019년 10월 건국 70주년 열병식에서 극초음속 미사일 ‘둥펑-17’을 처음 공개한 바 있다（no translation）

中国曾在2019年10月建国70周年阅兵式上首次公开高超音速导弹“东风-17”

같은 해 말 러시아는 ‘아방가르드’를 실전 배치했으며 지난해에는 신형 극초음속 순항미사일인 ‘지르콘’ 시험발사에 성공했다（no translation）

同年年底,俄罗斯实战部署了“前卫”导弹,去年成功试射了新型高超音速巡航导弹“锆石”

미국은 최근 극초음속 미사일 개발을 강도 높게 추진해 왔으나 현재까지는 중국, 러시아와의 경쟁에서 밀리고 있다는 평가를 받는다（no translation）

有评价认为,美国最近一直在大力推进高超音速导弹的开发,但到目前为止在与中国 俄罗斯的竞争中处于劣势

미국은 4월 B-52H 전략폭격기가 극초음속 미사일인 ‘AGM-183A ARRW’를 발사하는 시험을 했지만 성공하지 못했다（no translation）

美国4月进行了B-52H战略轰炸机发射高超音速导弹“AGM-183AARRW”的试验,但未能成功

시진핑, 떠나는 메르켈에 이례적 웃음… Xi Jinping wishes farewell to German chancellor Chinese

习近平与即将卸任的默克尔视频会谈,破例展露笑颜……

한시 인용 덕담시진핑(習近平) 중국 국가주석이 2005년부터 16년간 집권했으며 곧 퇴임을 앞둔 앙겔라 메르켈 독일 총리와 13일 화상회담을 가진 후 메르켈을 ‘라오펑유(老朋友·오랜 친구)’라고 부르며 극진히 예우했다 President Xi Jinping held farewell to German Chancellor Angela Merkel, who leaves office after 16 years in the chancellery since 2005, in a video meeting on Wednesday and called her an “old friend (lăo péngyŏu),” speaking very highly of the outgoing leader of Germany

引用古语表示祝福中国国家主席习近平13日与从2005年起执政16年并即将卸任的德国总理安格拉·默克尔举行视频会谈,并称默克尔为“老朋友”,给予了极高的礼遇

메르켈 총리가 재임 중 중국을 12차례나 방문했고 미중 갈등에서도 일방적으로 미국 편만 들지 않았다는 점을 높이 평가한 것으로 풀이된다 President Xi’s cordial greetings to Chancellor Merkel is likely to have been stemmed from Chancellor Merkel’s 12 visits to China during her tenure and her impartiality amid the U S -China tensions

默克尔在任期间曾12次访问中国,在中美矛盾中也没有单方面站在美国一边

메르켈 총리는 주독 미군 방위비 분담금 증액 압박 등을 가하는 도널드 트럼프 전 미 행정부와 상당한 마찰을 빚었다 The German chancellor frequently clashed with the Donald Trump administration over Trump’s intense pressure to spend more on defense and U S troops in Germany

默克尔与施加压力增加驻德美军防卫费分摊额等压力的前美国特朗普政府发生了相当大的摩擦

14일 공산당 기관지 런민일보는 전일 시 주석과 메르켈 총리가 화상회담을 가지면서 활짝 웃고 있는 사진을 1면에 게재했다 On Thursday, the People’s Daily, China’s largest newspaper, ran on the front page the photo of President Xi and Chancellor Merkel, both smiling, in a video meeting

中国共产党机关报《人民日报》14日在头版刊登了前一天习主席和默克尔总理进行视频会谈时露出灿烂笑容的照片

시 주석은 공개석상에서 거의 웃지 않는 것으로 유명하지만 이날 이례적으로 시종일관 미소를 지었다 President Xi rarely smiles in public, but he wore a pleasant smile throughout the meeting with Chancellor Merkel

习近平以在公开场合几乎不笑而闻名,但当天却一反常态地始终面带微笑

런민일보는 “메르켈이 재임하는 동안 중국과 독일 관계는 물론이고 중국과 유럽연합(EU)의 관계도 매우 돈독해졌다”고 평했다 The People’s Daily wrote that the China-Germany relations and the China-EU relations have been strengthened during Merkel’s time in office

《人民日报》评价说:“默克尔在任期间,不仅是中国和德国的关系,中国和欧盟的关系也变得非常深厚”

특히 시 주석이 “중국인은 정(情)과 의(義)를 중시하고 라오펑유를 잊지 않는다 “Chinese people put great emphasis on ties of friendship,” Xi was quoted as saying, adding, “We will never forget old friends,

习主席还特别强调:“中国人重情重义,不忘老朋友

중국의 대문은 언제라도 당신을 향해 활짝 열려 있다”고 강조했다고 덧붙였다 and China’s door will always be open to you

中国的大门随时都向你敞开”

중국 외교부 또한 홈페이지를 통해 시 주석이 이번 회담에서 ‘사람과 사람이 서로 아는 것이 제일 중요하고, 서로 알려면 상대의 마음을 알아야 한다(人之相識 貴在相知, 人之相知 貴在知心)’는 맹자의 구절을 인용했다고 밝혔다 ” The Chinese Foreign Ministry issued a press release stating that President Xi cited Mencius’ saying that goes, “The acquaintance of people lies in knowing each other In knowing each other, it is important to know the heart ”

中国外交部也在网站上表示,习近平在此次会谈中引用了孟子的一句话:“人之相识,贵在相知,人之相知,贵在知心”

또 메르켈의 재임 중 중국과 독일은 ‘제로섬’ 게임을 피하고 상호 이익을 얻을 수 있음을 증명했다고 치하했다 Xi applauded that Merkel’s chancellery proved that China and Germany could avoid playing a zero-sum game and were still be able to enjoy mutual benefits

他还称赞,默克尔在任期间,中国和德国证明了可以避免“零和”游戏,获得相互利益

관영 영자지 글로벌타임스도 메르켈이 주요국 지도자 중 중국을 가장 많이 방문했으며 실용적인 대중국 정책을 펼쳤다고 호평했다 The Global Times, China’s state-run English newspaper, also carried a favorable editorial, stating that Chancellor Merkel visited China the most among global leaders of major countries, and Germany adopted pragmatic China policies under her administration

中国官方英文报纸《环球时报》也评价说,默克尔在主要国家领导人中访问中国的次数最多,并推行了务实的对华政策

2005년 11월 취임한 메르켈은 신종 코로나바이러스 감염증(코로나19) 발생 전인 2019년 9월까지 총 12차례 중국을 방문했다 Since her inauguration in November 2005, Angela Merkel visited China 12 times through September 2019, before the breakout of the COVID-19 pandemic

2005年11月就任的默克尔到新冠疫情暴发之前的2019年9月为止,共访问了中国12次

같은 기간 시 주석은 세 차례 독일을 찾았다 Xi Jinping visited Germany three times during the same period

其间,习近平三次访问德国

두 사람은 코로나19 사태 후 전화와 화상회담으로 접촉을 이어갔다 The two leaders have communicated via phone and video meetings since the COVID-19 pandemic broke out

两人在新冠疫情暴发后通过电话和视频会谈进行了接触,

특히 올해에만 다섯 번 교류했다 In 2021 alone, Xi and Merkel contacted on five occasions

特别是仅今年就进行了5次交流

유가 80달러… 공급대란에 高물가 ‘쓰나미’까지 밀려오나Soaring oil prices stir inflation fears

油价80美元……供应大乱中,高物价“海啸”也会袭来吗

공급망 위기와 원자재 값 폭등이 겹치면서 세계 경제가 혼돈 속으로 빠져들고 있다 Supply chain crisis and a spike in raw material cost are driving the global economy into a chaos

随着供应链危机和原材料价格暴涨,世界经济陷入混乱

부품이 없어 공장이 멈추고, 배럴당 80달러까지 오른 유가는 글로벌 경기 회복의 발목을 잡고 있다 Plants are shut down due to lack of parts and oil prices, which rose to $80 per barrel, are holding back the recovery of global economy

由于没有零部件,工厂停止生产,油价每桶上涨到80美元,阻碍了全球经济的复苏

당장 미국 중국 등 거대 경제권의 경기가 둔화될 조짐을 보이고 있다 Large economies, including the U S and China are showing signs of slowdown

美国 中国等巨大经济圈的景气出现了放慢的征兆

이는 수출로 지탱하던 한국 경제에 직격탄이 될 수 있다 This could deal a blow to the Korean economy, which depends heavily on export

这有可能直接打击以出口支撑的韩国经济

유가로 인한 물가 상승까지 겹치면 내수 위축도 불가피한 상황이다 If soaring oil prices bring about inflation, domestic demand will inevitably shrink

如果再加上油价引起的物价上涨,内需萎缩也不可避免

11일(현지 시간) 미국 서부텍사스산원유(WTI) 가격은 7년 만에 배럴 당 80달러를 돌파했다 코로나 회복세와 난방용 수요가 겹친 탓이다 West Texas Intermediate (WTI) crude futures hit $80 a barrel for the first time in seven years on Monday (local time), caused by economic recovery from the COVID-19 pandemic and rising demand for heating

当地时间11日,美国西德克萨斯产原油价格时隔7年突破每桶80美元这是因为新冠恢复势头和取暖需求重迭

이 때문에 핀란드 노르디아뱅크는 내년 미국 성장률 예상치를 3.5%에서 1.5%로 낮췄다 As a result, Nordea Bank Finland lowered its forecast for U S economic growth next year to 1.5% from 3.5%

因此,芬兰北欧银行将明年美国经济增长率预测值从3.5%下调至1.5%

유가 폭등 탓에 물가가 오르고 소비가 둔화될 수밖에 없다는 뜻이다 This means soaring oil prices will press inflation upwards and reduce consumption

意思是,由于油价暴涨,物价必然会上涨,消费必然会趋缓

이런 흐름은 글로벌 경제와 한국 수출에 악영향을 줄 수밖에 없다 This trend will negatively affect the global economy and Korea’s export

这种趋势必然会对全球经济和韩国出口产生负面影响

공급망 위기도 악화되고 있다 The supply chain crisis is also worsening

供应链危机也在恶化

원자재와 중간재를 가리지 않고 병목 현상을 빚는데다 물류난까지 벌어지고 있다 There are difficulties in logistics not to mention bottlenecks in the production of raw materials and intermediate goods

不分原材料和中间材料,不仅出现瓶颈现象,还出现了物流困难

소비자가 제품을 제 때 공급받지 못하면서 물가가 가파르게 오르고 있다 Prices are rising sharply as consumers are not getting products on time

由于消费者不能及时得到产品供应,物价正在急剧上涨

물가가 오르면 기업들이 비싼 값에 팔려고 생산을 늘리는 게 상식이다 When prices rise, companies normally increase production to sell their products at higher prices

如果物价上涨,企业就会为了高价出售而增加生产,这是常识

하지만 생산 자체가 어려운 상황이어서 자칫 경기침체 속에 물가만 오르는 스태그플레이션 우려도 나오고 있다 However, under the circumstances, where production itself is difficult, there are concerns of stagflation, which refers to an economy that is experiencing no growth in production and an increase in inflation

但是,由于生产本身就很困难,因此有人担心经济停滞导致物价上涨的滞胀

한국 소비자물가는 9월까지 6개월 연속 2%대 상승률을 나타내며 당초 관리 목표인 1.8%를 넘어섰다 Korea’s consumer price index (CPI) showed an increase of 2% for six consecutive months through September, exceeding the target of 1. 8%

截止到9月,韩国消费者物价连续6个月上涨2%,超过了当初的管理目标1.8%

물가를 잡으려면 금리 인상 등 긴축에 나서야하지만 경기 회복에 찬물을 끼얹을 수 있다 Austerity measures, such as rise in interest rates, are necessary to control inflation but this could put a damper on economic recovery

要想控制物价,必须采取上调利率等紧缩措施,但这可能会给经济复苏泼冷水

한국은행이 어제 기준금리를 동결한 것도 이런 고민의 결과다 This is why the Bank of Korea (BOK) decided to freeze its key interest rate on Tuesday

韩国银行昨天冻结基准利率也是这种苦恼的结果

기업들은 원자재와 부품 공급을 위해 비상 체제를 가동하고 있다 Businesses are operating an emergency system to supply raw materials and parts

企业为了供应原材料和零部件,正在启动紧急体制

정부도 수출 호조에 취해 안일하게 대응할 때가 아니다 The government cannot afford to be complacent on brisk exports

政府也不能陶醉于出口好转,安逸地应对

공급망에 차질이 없도록 기업과 공동 대응에 나서고, 유가 상승에 따른 전기요금 영향도 면밀히 따져봐야 한다 It should take a joint response with businesses to ensure that there are no disruptions in the supply chain, and closely examine soaring oil prices’ impact on electricity cost

为了不影响供应链,应该与企业共同应对,并仔细分析油价上涨带来的电费影响

물가와 경기를 감안한 세심한 금리 정책도 쉽지 않은 과제이다 Another difficult challenge is establishing a meticulous interest rate policy, considering both prices and the economy

考虑到物价和景气的细致的利率政策,也是不容易的课题

상황을 지켜만 보기에는 글로벌 환경이 너무 긴박하다 The current situation worldwide is too urgent for the government to just sit back and observe it

仅凭观察情况,全球环境过于紧迫

대규모 전력난에… 중, 호주산 석탄 다시 수입 ‘백기’China imports coal from Australia again

面临大规模电力短缺,中国再次“举白旗”

미중 갈등 속에서 미국 편에 선 호주에 보복하기 위해 중국 당국이 호주산 석탄의 수입을 막았지만 석탄 부족 사태가 심각해지자 중국 수입업자들이 호주산 석탄을 하역하기 시작했다고 영국 파이낸셜타임스(FT)가 4일 보도했다 The Financial Times reported on Monday that Chinese importers started to unload Australian coal as demand for coal surges despite the import ban imposed by the Chinese authority on Australian coal in retaliation against Australia for taking sides with the U S amid the U S -China rivalry

进口澳大利亚煤炭英国《金融时报》4日报道,为了报复在中美矛盾中站在美国一边的澳大利亚,中国当局阻止了澳大利亚产煤炭的进口,但由于煤炭不足事态严重,中国进口企业开始装卸澳大利亚产煤炭

석탄 부족으로 발전소 운영이 중단되고 대규모 전력난으로 이어지자 사면초가 상황에 몰린 중국이 호주에 굴복했다는 분석이 나온다 Critics argue that China gave in to Australia in the face of the suspension of coal-fired power plants and resultant power outages

有分析认为,由于煤炭不足导致发电站运营中断,引发大规模电力难,面临四面楚歌状况的中国屈服于澳大利亚

FT에 따르면 지난달 말부터 중국 주요 항구에서는 바다에 대기 중이었던 호주 화물선에서 석탄을 하역하는 작업이 이뤄지고 있다 According to the newspaper, at major Chinese ports, coals are unloaded from Australian vessels that were stranded in the sea

据《金融时报》报道,从上月底开始,中国主要港口正在从在海上待命的澳大利亚货船上卸载煤炭

국제 선박중개회사 브래마 ACM의 닉 리스틱 화물책임자는 석탄 45만 t이 하역됐다고 전했다 Nick Ristic, lead dry cargo analyst at Braemar ACM Shipbroking, reported that approximately 45 tons of coal were unloaded so far

国际船舶经纪公司百力马-艾斯盟的货物负责人尼克·里斯提克表示:“45万吨煤炭已经卸货”

에너지컨설팅업체 케이플러도 지난달 선박 5척에서 호주산 석탄 38만3000t이 하역됐다고 FT에 밝혔다 Global energy consulting firm Kepler also admitted to The Financial Times that 383,000 tons of Australian coal were unloaded from five carriers last month

能源咨询企业Kpler也向《金融时报》表示,上个月从5艘船上卸载了38.3万吨澳大利亚产煤炭

현지 무역업자들은 중국 당국이 “통관을 허락한다”는 신호를 보낸 것으로 받아들이고 있다 The local traders consider the move as the Chinese authority’s signal that allows customs clearance

当地贸易商认为,中国当局发出了“允许通关”的信号

지난해 중국은 국영 에너지 기업과 제철소에 “호주산 석탄 수입을 중단하라”고 명령했다 세계 최대 석탄 수출국인 호주에 대한 무역 보복이었다 In 2020, the Chinese government ordered the state-owned energy corporations and steel mills to “stop importing Australian coal” in retaliation against Australia, the world’s biggest coal exporter

去年,中国命令国营能源企业和钢厂“停止进口澳大利亚产煤炭”这是对世界最大煤炭出口国澳大利亚的贸易报复

이 조치로 호주는 약 39억 달러(약 4조6352억 원)의 손실을 입었다 As a result, Australia incurred a loss of approximately 3. 9 billion dollars (approximately 4 6342 trillion won)

由于这一措施,澳大利亚蒙受了约39亿美元(约4.6352万亿韩元)的损失

호주산 석탄 수입이 금지되고 시진핑 중국 국가주석의 ‘친환경 저탄소’ 정책이 겹치면서 중국에서는 석탄 부족 사태가 벌어졌다 The import ban on Australian coal and Chinese President Xi Jinping’s “green energy initiative and carbon neutrality goal” initiated coal shortages in China

随着澳大利亚产煤炭被禁止进口,加上中国国家主席习近平推行“环保低碳”政策,中国出现了煤炭短缺现象

이는 중국 동북부의 전력난으로 이어져 일부 지역에서는 공장 가동이 중단되고 가정용 전기 공급도 제한되고 있다 This led to power outages in north-east China, leading to factory shutdown and leaving millions of homes in darkness and cold

这导致中国东北部的电力短缺,部分地区工厂停产,家庭用电也受到限制

지린성 등 중국 각 지방정부는 인도네시아, 러시아, 몽골, 카자흐스탄 등에서 석탄을 확보하기 위해 안간힘을 쓰고 있지만 세계적으로 석탄 수요가 늘면서 가격도 폭등해 수입하기 어려운 상황이다 The local governments, including Jilin province, are struggling to import coal from Indonesia, Russia, Mongolia, and Kazakhstan, but the surging global demand for coal raised the price, making it difficult for China to have access to coal imports

吉林省等中国各地方政府为在印度尼西亚 俄罗斯 蒙古国 哈萨克斯坦等地确保煤炭而竭尽全力,但随着世界煤炭需求的增加,价格也暴涨,因此很难进口

‘파산 위기’ 中헝다 사태 여파 스웨덴까지 번져약 355조 원에 달하는 천문학적 부채로 파산 위기에 직면한 중국 부동산회사 헝다그룹 사태의 후폭풍이 북유럽 스웨덴으로도 번졌다 Aftermath of China’s Evergrande woes hits Sweden The financial woes of China’s Evergrande Group at the risk of bankruptcy with about 355 trillion won in liabilities have spread to Sweden in Northern Europe

面临“破产危机”的中国恒大,瑞典也受到波及因约355万亿韩元的天文数字负债而面临破产危机的中国房地产公司恒达集团事件的后续风暴甚至蔓延到了北欧的瑞典

2일 블룸버그 등에 따르면 헝다 계열사 헝다뉴에너지자동차(헝다자동차)와 합작해 전기차를 개발하고 있는 스웨덴 자동차회사 ‘내셔널일렉트릭비이클스웨덴(NEVS)’은 최근 공장 직원 670명 중 절반에 가까운 300명을 해고했다 According to Bloomberg on Saturday, National Electric Vehicle Sweden AB (NEVS), a Swedish company jointly developing electric vehicles with Evergrande Group’s subsidiary, Evergrande New Energy Vehicle Group, recently dismissed 300 employees, which are almost half of its entire 670 workers at a factory

据彭博社2日报道,与恒达子公司恒达新能源汽车(恒达汽车)合作开发电动汽车的瑞典汽车公司“瑞典国家电动汽车(NEVS)”最近解雇了工厂670名职员中的近一半(300人)

내셔널일렉트릭 관계자는 “헝다자동차로부터 자금 조달이 이뤄지지 않아 감원은 물론 전기차 개발도 중단됐다”며 “공장을 사실상 폐쇄했다”고 밝혔다 “Due to the lack of funding from Evergrande New Energy Vehicle Group, we had to cut jobs and the development of electric vehicles has been suspended,” said a member of NEVS

瑞典国家电动汽车公司有关人士表示:“由于没有从恒达汽车公司调剂资金,因此不仅裁员,还中断了电动汽车的开发实际上已经关闭了工厂”

스테판 틸크 최고경영자(CEO) 역시 “헝다가 아닌 새로운 합작사와 투자자를 찾고 있다”고 밝혔다 “We are looking for a new partner and investors,” said Stefan Tilk, the CEO of NEVS

首席执行官斯蒂凡·蒂尔克也表示:“正在寻找新的合作公司和投资者,而不是恒大”

내년 전기차 양산을 준비했던 헝다자동차는 최근 중국 내 공장 설비업자에게도 대금을 지급하지 못했다 Evergrande New Energy Vehicle Group has been preparing for the mass production of electric vehicles next year but failed to pay a Chinese plant equipment company

准备明年批量生产电动汽车的恒大汽车最近也没能向中国国内的工厂设备业主支付货款

일부 직원의 월급도 밀렸고 연구소 연구원들을 위한 무료 식사 제공도 모두 중단했다 Some of its employees have not received salaries for some time and free meals for researchers at its R&D center have been suspended

部分职员的工资也被拖欠,研究所研究员的免费用餐也全部中断

헝다그룹이 지난달 말 자회사가 보유 중인 주식을 팔아 약 1조8300억 원의 자금을 확보했지만 올해 안에 갚아야 할 이자만 7500억 원에 달하는 등 위기가 계속되고 있다 While Evergrande Group sold shares owned by its subsidiary at the end of September to secure 1.83 trillion won but the group’s crisis will continue as it has 750 billion won of interests to pay until the end of this year

恒大集团于上月底出售子公司持有的股票,确保了约1.83万亿韩元的资金,但仅今年内要偿还的利息就达7500亿韩元,危机仍在持续

특히 주력 사업인 부동산은 중국 정부의 규제로 침체기에 접어들었고, 유망했던 전기차 사업마저 차질이 불가피해져 헝다그룹의 자력 회생은 사실상 불가능하다는 전망도 나온다 As its main business, which is real estate, has been slowing down due to the Chinese government’s regulations and its prominent electric vehicle business is struggling, some say that the group won’t be able to recover on its own

特别是,主力事业房地产因中国政府的限制性规定进入停滞期,曾有潜力的电动汽车事业也将不可避免地出现差池,因此有预测认为,恒大集团实际上不可能自力更生复苏

헝다그룹은 지난달 29일 달러화 채권 이자 약 559억 원을 채권자들에게 지급하지 못했다 Evergrande Group failed to pay 55.9 billion won of dollar bond interests to creditors on Wednesday

恒达集团上个月29日未能向债权人支付约559亿韩元的美元债券利息

앞서 같은 달 23일에도 달러화 채권 이자 약 993억 원을 지급하지 않고 30일 유예시켰다 It also missed the payment of 99.3 billion won of dollar bond interests on Sept 23 and delayed the payment on Thursday

在此前的同月23日也没有支付约993亿韩元的美元债券利息,而是推迟了30天

정의용 “中 공세적 외교는 당연”…韓외교수장, 美서 ‘中두둔’ 논란정의용Foreign minister’s remark siding with China under fire

郑义溶:“中国采取攻势外交是理所当然的”……

외교부 장관이 22일(현지 시간) 미국과 한국, 일본, 호주 등 동맹국들을 중국에 맞서는 하나의 연대로 묶는 것을 두고 “냉전시대 사고”라고 규정했다 South Korean Foreign Minister Chung Eui-yong described the U S -led initiative to create an alliance consisting of the country’s allies, including South Korea, Japan, and Australia, against China as an “old-style Cold War mentality

在美国发表维护中国的言论引发争议韩国外交部长郑义溶当地时间22日把美国 韩国 日本 澳大利亚等盟国为对抗中国而成立的联盟定义为“冷战思维”

미국에서 비판받는 중국의 ‘공세적(assertive) 외교’에 대해서는 “중국으로서는 당연한 일”이라고 했다 ” He also responded that it is “only natural” for China to employ “assertive diplomacy,” which is harshly criticized by the U S

他就在美国受到抨击的中国的“进攻性(assertive)外交”表示:“这对中国来说是理所当然的事情”

조 바이든 미국 행정부의 중국 견제 정책이 본격화하고 있는 시점에 한국의 외교수장이 미국에서 중국을 두둔하는 듯한 발언을 내놓은 것이어서 논란이 되고 있다 Coming as the Biden administration has begun in earnest to check China’s influence, the foreign minister’s remark quickly drew criticism for seemingly siding with China on the American soil

在美国拜登政府全面推行牵制中国政策的情况下,韩国的外交首长在美国做出了似乎维护中国的发言,引发了争议

유엔총회 참석차 미국을 방문 중인 정 장관은 이날 뉴욕의 싱크탱크 미국외교협회(CFR) 초청 대담에서 ‘최근 몇 년간 중국이 점점 더 공세적으로 돼 가고 있다고 보느냐’는 대담 진행자의 질문에 “그것은 당연한 일(only natural)” Chung, currently visiting the U S in attendance on President Moon Jae-in for the United Nations General Assembly, said that it was “only natural” for China to become more assertive for the past few years, in a conversation meeting hosted by New York-based think tank Council on Foreign Relations (CFR)

为出席联合国大会而正在美国访问的郑义溶当天出席纽约智库美国外交协会邀请的座谈中,就主持人“你是否认为近年来中国越来越变得具有进攻性”的提问表示:“那是理所当然的(onlynatural)”,

이라며 “중국은 경제적으로 더 강해지고 있고 지금은 20년 전의 중국이 아니다”라고 답변했다 “China has become an economic superpower, and China today is not the same as China 20 years ago

“中国在经济上变得更强,现在已经不是20年前的中国”

그는 “중국이 가진 것을 외교정책에 반영하기를 바라는 것은 자연스러운 일”이라며 “이것을 공세적이라고 부를 수 있는지 모르겠다”고 했다 It is natural for China to capitalize on its presence to gain a competitive edge in diplomacy, and I am unsure whether ‘assertiveness’ is the appropriate term to describe China’s diplomatic posture,” said Foreign Minister Chung

他表示:“中国希望在外交政策中反映自己拥有的东西是很自然的事情不知道能否将其称为进攻性”

“중국은 국제사회 일원으로 자신들의 목소리가 반영되기를 바라는 것”이라며 “우리는 그들이 우리에게 하려는 이야기에 귀 기울여야 한다”고도 했다 “China seeks to reflect its view as a member of global community, and we need to pay attention to what China is trying to say

他还表示:“中国希望作为国际社会的一员反映自己的声音我们应该倾听他们想对我们说的话”

진행자 파리드 자카리아 CNN 앵커가 인도태평양 지역의 외교 지형과 관련한 설명을 하면서 미국, 한국, 일본, 호주를 중국에 맞서는 하나의 블록으로 구분하려 하자 정 장관은 “그것은 중국 사람들이 말하듯이 냉전시대 사고(the mentality of Cold War)”라고 했다 ” When Fareed Zakaria, the host of CNN’s GPS and the moderator of the event, described the U S , South Korea, Japan, and Australia as one bloc in opposition to China in his illustration of the diplomatic landscape of the Indo-Pacific, Foreign Minister Chung said such distinction is “the outdated mentality of Cold War,” as declared by China

主持人 美国有线电视新闻网主播法里德·扎卡里亚似乎在说明印度太平洋地区的外交地形时要把美国 韩国 日本 澳大利亚区分为对抗中国的一个联盟,郑义溶对此表示:“那就是正如中国人们所说的冷战思维(thementalityofcoldwar)”

정 장관은 미국이 인도태평양 지역에서 중국 견제를 위한 주요 연합체로 삼고 있는 ‘쿼드(Quad)’ 가입에 대한 질문에는 “가입해야 할 긴급한 필요성을 느끼지 않는다”고 했고, 미중 두 나라를 두고서는 “어느 한쪽을 선택해야 한다고 생각하지 않는다”고 답변했다 Asked whether to join the Quad, the U S ’s strategic forum in the Indo-Pacific to contain China, Foreign Minister Chung said that South Korea does not feel the urgent need to join the forum and that it is not necessarily mandated to choose between U S and China

郑义溶就加入美国在印度太平洋地区作为牵制中国的主要联合体的“四方机制”(quad)的提问表示,“不觉得有必须加入的紧急必要性”,并认为“并不认为必须在美中两国应选一方”

9•11테러 20년, 美中‘힘의 오만’ 접고 경쟁•협력해야9·11테러 20주년을 맞은 11일, 미국 곳곳에선 희생자들을 기리는 추모식이 열렸다 U S and China cooperate while competing against each other Memorial ceremonies were held in various cities across the U S on Saturday that marked the 20th year of the Sept 11 attacks

“9·11”恐怖袭击20周年,中美应该收起“力量的傲慢”,展开竞争和合作迎来“9·11”恐怖事件20周年的11日,美国各地举行了悼念遇难者的仪式

2977명의 목숨을 앗아간 테러가 발생한 지 20년이라는 상징성에다 그 테러가 유발한 아프가니스탄 전쟁의 지난달 말 종료는 추모 분위기를 한층 엄숙하게 만들었다 The ceremonies were solemn on the 20th year of the the terrorist attacks that took 2,977 lives and the Afghanistan war that was ended at the end of August

夺走2977人生命的恐怖事件发生20年的象征性,再加上该恐怖事件引发的阿富汗战争上月底结束,使追悼氛围更加严肃

곳곳엔 ‘절대 잊지 않겠다(Never Forget)’는 문구가 내걸렸고, 전·현직 대통령 등 미국 지도자들은 한결같이 테러에 맞선 국민의 통합과 단결을 주문했다 Americans put up posts that go “Never Forget” and leaders including former and incumbent presidents asked for unity and consolidation against terrorism

到处都挂着“永不忘记(NeverForget)”的标语,前 现任总统等美国领导人一致要求国民团结应对恐怖袭击

세계 정상들도 국제적 연대를 강조했다 Global leaders also highlighted international coalition

世界首脑们也强调了国际联合

9·11테러는 21세기 벽두 자유주의 국제질서에 엄청난 공포와 분노를 불러오면서 유일 초강대국 미국의 파워를 시험대에 올린 사건이었다 The Sept 11 attacks were an incident that triggered tremendous fear and rage in the liberalist international order in early 21st century and put the power of the U S , the most powerful country on earth, on the testbed

“9·11”恐怖事件给21世纪初的自由主义国际秩序带来了巨大的恐惧和愤怒,将唯一超级大国美国的力量推上了试验台

10년 전 냉전 승리와 함께 일극(一極)의 질서를 이끌던 미국은 즉각 ‘테러와의 전쟁’을 선포하면서 아프간 전쟁, 그리고 이라크 침공까지 그 파워를 유감없이 발휘했다 하지만 아프간과 이라크는 미국의 ‘수렁’이었다 The U S , which won the cold war 10 years ago and led the international order, declared a war against terrorism that led to the Afghanistan war and the invasion of Iraq

10年前在冷战中取得胜利的同时引领一极秩序的美国立即宣布“与恐怖主义的战争”,在阿富汗战争和伊拉克战争中充分发挥了其力量但阿富汗和伊拉克是美国的“泥潭”

지난달 아프간에서 쫓겨나듯 철수하던 미군의 초라한 모습은 이를 상징적으로 보여줬다 But Afghanistan and Iraq were a pit for the U S The pitiable withdrawal of the U S troops from Afghanistan last month symbolized this

上个月像被赶出阿富汗一样撤退的美军寒酸的样子象征性地体现了这一点

미국은 이제 그 수렁에서 벗어나 중국과의 본격적인 패권경쟁에 온전히 집중하려 한다 The U S now plans to put itself out of the mire and focus wholly on the hegemony competition against China

美国现在想摆脱这个泥潭,完全集中精力与中国展开霸权竞争

조 바이든 행정부는 취임 직후부터 이전 행정부보다 훨씬 정교하고 치밀한 전략 아래 중국을 견제·포위하겠다는 뜻을 숨기지 않았다 The Biden administration did not hide its intention to hold China in check and envelop it with much more sophisticated and meticulous strategies than the previous administrations

拜登政府就任后就表示要在比前政府更加精巧 周密的战略下牵制和包围中国

물론 그것은 중국의 거센 도전을 막아내지 않고선 미국의 지위도 위태롭다는 위기의식의 산물이다 It comes from the sense of crisis that China’s fierce challenge could put the U S in a perilous position

当然,这是如果不阻止中国的强烈挑战,美国的地位也会受到威胁的危机意识的产物

이런 미국에 중국은 극도의 경계심을 나타내면서도 대결에는 대결로 맞서겠다는 결연한 태도를 보여왔다 China showed its firm resolution to confront the U S while remaining vigilant

对于这样的美国,中国表现出极度的警戒心,但对对抗却表现出坚决的态度

바이든 대통령이 10일 시진핑 중국 주석과 7개월 만에 가진 통화에서 상호 대화와 소통을 통해 국제적 책임을 다하자는 데 원론적으로 공감한 것은 그나마 다행이다 It is a good thing that U S President Joe Biden agreed to take international responsibilities through interaction and communication in a call with Chinese President Xi Jinping which he had for the first time in the past seven months

拜登10日与中国国家主席习近平时隔7个月再次通话,就通过相互对话和沟通尽到国际责任达成了尽管是原则性的共识,这是值得庆幸的事情

비록 입장차가 여전하지만 미중 정상의 9·11테러 20년 전날의 통화는 상징성이 크다 Even though they still had different standpoints, but the call, which happened a day before the 20th anniversary of the Sept 11 attacks, has a significant meaning

虽然立场差异依然存在,但美中首脑在“9·11”恐怖事件20周年前一天的通话具有很大的象征性

테러와의 전쟁 20년이 됐지만 그 전쟁은 한층 더 어려워졌다 It has been 20 years since the U S declared a war against terrorism, but the war became a lot more difficult to win

反恐战争已经过去20年,但这场战争变得更加艰难

미중이 협력해야 할 분야는 테러만이 아니다 Terrorism is not the only field that requires cooperation between the U S and China

美中需要合作的领域不仅仅是恐怖主义

기후변화는 물론 북핵 등 비확산 문제 등 산적해 있다 There are numerous fields that need cooperation such as climate change and non-proliferation of North Korea’s nuclear programs

不仅是气候变化,朝核等不扩散问题等堆积如山

국제정치에서 치열한 경쟁은 불가피하다 Fierce competition is inevitable in international politics

国际政治中激烈的竞争是不可避免的

하지만 강대국 간 대결, 힘의 정치가 지배하면 스스로는 물론 국제사회에 재앙을 가져올 뿐이다 But confrontation between superpowers and power politics can only bring a disaster to the global community

但如果强国之间的对抗 实力政治占据主导,不仅会给自己,还会给国际社会带来灾难

경쟁하면서 협력해야 한다 They should cooperate while competing against each other

要在竞争中合作

（（additional translation））U S and China cooperate while competing against each Memorial ceremonies were held in various cities across the U S on Saturday that marked the 20th year of the Sept 11 attacks The ceremonies were solemn on the 20th year of the the terrorist attacks that took 2,977 lives and the Afghanistan war that was ended at the end of August Americans put up posts that go “Never Forget” and leaders including former and incumbent presidents asked for unity and consolidation against terrorism Global leaders also highlighted international coalition The Sept 11 attacks were an incident that triggered tremendous fear and rage in the liberalist international order in early 21st century and put the power of the U S , the most powerful country on earth, on the testbed The U S , which won the cold war 10 years ago and led the international order, declared a war against terrorism that led to the Afghanistan war and the invasion of Iraq But Afghanistan and Iraq were a pit for the U S The pitiable withdrawal of the U S troops from Afghanistan last month symbolized this The U S now plans to put itself out of the mire and focus wholly on the hegemony competition against China The Biden administration did not hide its intention to hold China in check and envelop it with much more sophisticated and meticulous strategies than the previous administrations It comes from the sense of crisis that China’s fierce challenge could put the U S in a perilous position China showed its firm resolution to confront the U S while remaining vigilant It is a good thing that U S President Joe Biden agreed to take international responsibilities through interaction and communication in a call with Chinese President Xi Jinping which he had for the first time in the past seven months Even though they still had different standpoints, but the call, which happened a day before the 20th anniversary of the Sept 11 attacks, has a significant meaning It has been 20 years since the U S declared a war against terrorism, but the war became a lot more difficult to win Terrorism is not the only field that requires cooperation between the U S and China There are numerous fields that need cooperation such as climate change and non-proliferation of North Korea’s nuclear programs Fierce competition is inevitable in international politics But confrontation between superpowers and power politics can only bring a disaster to the global community They should cooperate while competing against each other

中 베이징대 교수, 시진핑 ‘공동부유’ 정면비판 Beijing Univ professor criticizes Xi's ‘common wealth’

北京大学教授张维迎正面批评习近平“共同富裕”

중국 베이징대의 한 경제학과 교수가 최근 시진핑(習近平) 중국 국가주석이 주창하고 있는 ‘공동부유(共同富裕)’에 정면으로 반기를 들었다 The initiative for "common wealth” proposed by Chinese President Xi Jinping has been met with direct criticism by an economics professor at Beijing University,

中国北京大学的一名经济学教授最近正面反对中国国家主席习近平提倡的“共同富裕”

정부의 과도한 개입으로 ‘공동부유’가 ‘공동빈곤’이 될 수 있다는 것이다 who argues that excessive government intervention can turn "common wealth” into “common poverty

他认为,如果政府过度介入,“共同富裕”可能会成为“共同贫困”

시 주석 집권 이후 반대 세력에 대한 대대적인 사정 작업과 감시·규제 확대로 정부 정책에 반대하는 목소리가 거의 사라진 가운데 나온 주장이어서 이목이 집중되고 있다 ” As opposing views of the government's policy directions have been subject to nationwide inspection, monitoring and regulation, the professor's argument is gathering public attention

习主席执政以来,由于对反对势力的大规模整顿工作和扩大监视和管制,反对政府政策的声音几乎消失,在这种情况下出现的主张备受关注

홍콩 사우스차이나모닝포스트(SCMP)는 4일 장웨이잉(張維迎·62·사진) 베이징대 경제학과 교수가 최근 공익성 민간학문기구인 ‘경제 50인 논단(CE50)’ 홈페이지에 올린 글을 통해 “시장의 힘에 대한 신뢰를 잃고 정부 개입에 자주 의존하면 공동빈곤으로 이어질 것”이라고 비판했다고 보도했다 Economics Professor Zhang Weiying at Beijing University was quoted as saying in an article posted on a website of CE50 – a private academic organization for the public good, “If we lose faith in market forces and rely on frequent government intervention, it will lead to common poverty,” according to the South China Morning Post's report on Saturday

香港《南华早报》4日报道,北京大学经济系教授张维迎(62岁,照片)最近在公益性民间学术机构“经济50人论坛(CE50)网站上发表文章说:“失去了对市场力量的信任,经常依赖政府介入,会共同导致贫困”

이어 그는 “기업가들이 부를 창출할 동기가 없다면 정부가 빈곤층에 줄 돈이 없어져 상류가 말라버린 강처럼 될 것”이라며 “계획경제는 빈곤층에 더 많은 복지를 제공하려 했지만 결과적으로 더 많은 빈곤층이 생겼다 시장 지향적 개혁을 앞당기는 것만이 보다 공정한 사회를 만들 수 있는 유일한 길”이라고 강조했다 “If entrepreneurs have no motivation to create wealth, the government will have no money to transfer – the charity will become a river without headwaters,” he said in his critical article Professor Zhang emphasized that a planned economic system tried to provide more welfare benefits to the poor but rather the result turned out to increase a level of poverty across society, adding that a market-oriented reform should happen as fast as possible so that a greater level of fairness can be shared further

他强调:“如果企业家没有创造财富的动机,政府就没有钱给贫困阶层,上游就会像干涸的河流一样计划经济试图为贫困阶层提供更多的福利,但结果出现了更多的贫困阶层只有提前市场指向性改革,才能创造更加公正的社会”

장 교수는 중국 시안에서 대학을 졸업하고 영국 옥스퍼드대에서 경제학 석·박사 학위를 받은 뒤 1994년부터 베이징대 교수로 재직 중이다 Mr Zhang has worked at Beijing University since 1994

张维迎毕业于中国西安大学,在英国牛津大学获得经济学硕士 博士学位,从1994年起担任北京大学教授,

2008년에는 베이징대 주요 싱크탱크인 국가발전연구원을 설립했다 College-educated in Xian, he obtained a master's and doctorate degree at Oxford University in Britain Back in 2008, he founded the National School of Development - a major think tank of Beijing University

2008年还成立了北京大学主要智库———国家发展研究院

장 교수는 2018년 10월에도 “중국의 지난 40년 고성장은 시장화, 기업가 정신, 서구 300년의 기술 축적으로 이룬 것이지 이른바 ‘중국모델’ 때문은 아니다”라고 주장해 파문을 일으키기도 했다 He stirred controversy by stating in October 2018 that the main contributor to China’s 40 years’ rapid growth is not the Chinese model of development but a combination of marketization, entrepreneurship and technological accumulation of 300 years of the West

张维迎2018年10月也曾主张,“中国过去40年的高增长是通过市场化 企业家精神 西欧300年的技术积累形成的,并不是因为所谓的‘中国模式’”,从而引发了风波

현재 CE50 홈페이지에 게재됐던 장 교수의 글은 내려진 상태며 장 교수의 개인 위챗(중국판 카카오톡) 계정에서도 삭제된 상태다 As of now, Zhang's critical article is deleted both on the website of CE50 and his personal WeChat account

目前张维迎登载在CE50网站上的文章已经被拿下,其个人微信(中国版kakaotalk)账号也被删除

위챗에서 해당 글을 전송하는 것도 안 되고 있다 The article is prohibited from being sent to other users on WeChat

在微信上发送相关文章也不行

바이든, 아프간 철군 다음날 “이제 中-러 등 새 위협 대처해야” Biden says U S should deal with new threats

拜登在从阿富汗撤军的第二天表示:“现在应该应对中俄等新威胁”

조 바이든 미국 대통령이 아프가니스탄 전쟁 종식을 선언하며 이제는 중국, 러시아 등 미국이 직면한 21세기의 위협에 대처할 때라고 강조했다U S President Joe Biden declared the end of a war in Afghanistan and emphasized that it is now time for the U S to deal with the 21st-century threats it is facing, such as China and Russia

美国总统拜登宣布阿富汗战争结束,并强调现在是应对中国 俄罗斯等美国面临的21世纪威胁的时候了

바이든 대통령은 미국이 아프간 철군을 완료한 지 하루 만인 지난달 31일(현지 시간) 백악관 대국민 연설에서 “세상이 바뀌고 있다”며 “우리는 중국과 심각한 경쟁 중이고, 러시아의 도전을 다루고 있으며, 사이버 공격과 핵 확산에 대응해야 한다”고 말했다 “The world is changing We’re engaged in a serious competition with China We’re dealing with the challenges on multiple fronts with Russia We’re confronted with cyberattacks and nuclear proliferation,” President Biden said on Tuesday (local time), just one day after the U S completed its withdrawal from Afghanistan, at the White House

拜登在美国完成从阿富汗撤军一天后的当地时间8月31日在白宫发表对国民演说他表示:“世界正在改变我们正在与中国进行严重的竞争,并在应对俄罗斯的挑战,还要应对网络攻击和核扩散”

“미국이 또 다른 10년을 아프간의 수렁에 빠지는 것을 중국과 러시아만큼 좋아할 나라는 없을 것”이라며 “우리는 2021년을 위한 새로운 도전에 맞설 역량을 보여줘야 한다”고도 했다 “And there’s nothing China or Russia would rather have, would want more in this competition than the United States to be bogged down another decade in Afghanistan,” he added “We have to shore up America’s competitiveness to meet these new challenges in the competition for the 21st century

他还表示:“没有比中国和俄罗斯更喜欢美国在阿富汗陷入另一个10年的泥潭的国家我们应该表现出应对2021年新挑战的力量”

바이든 대통령은 중동이 아닌 중국이라는 미국의 최대 위협에 집중할 필요성을 역설하며 “지난 20년간의 외교정책 페이지를 넘길 때”라고 강조했다 ” “As we turn the page on the foreign policy that has guided our nation the last two decades, we’ve got to learn from our mistakes,” the president said, emphasizing that the U S should focus on its biggest threat, which is China, not the Middle East

拜登强调有必要集中精力应队中国这一美国的最大威胁而不是中东他强调:“现在是翻过过去20年的外交政策一页的时候了”

연설에서 바이든 대통령은 미국이 ‘세계의 경찰’ 역할을 벗어던지고 국익을 바탕으로 현재와 미래의 외교안보 위협 대응에 집중하겠다는 외교정책 방향을 선명하게 드러냈다 His foreign policy direction was very clear in his statement that the U S will stop playing the role of the global policeman and focus on addressing the current and future threats in foreign affairs and security based on its own national interests

拜登在演讲中明确表示,其外交政策方向是,美国要摆脱“世界警察”的角色,以国家利益为基础,集中应对现在和未来的外交安保威胁

‘핵 확산’을 언급한 부분은 중국과 북한 등의 핵 위협이 커지는 상황을 감안한 것으로 보인다 He seems to have mentioned ‘nuclear proliferation’ in consideration of the growing nuclear threats from China and North Korea

分析认为,他提及“核扩散”的部分,可能是考虑到了中国和朝鲜等国的核威胁不断扩大的情况

이날 백악관은 북한의 영변 핵시설 재가동 움직임과 관련해 “(대북 접촉의) 문을 열어두고 있다”며 북한과의 대화 필요성을 재차 언급했다 The White House repeatedly mentioned on Tuesday the need for dialogues with North Korea regarding the North’s resumption of Yongbyon nuclear facilities by saying that they have left the door open

当天,白宫就朝鲜重启宁边核设施的动向表示,“(对朝鲜接触的)大门敞开着”,再次提及了同朝鲜对话的必要性

젠 사키 백악관 대변인은 브리핑에서 북한의 현재 핵 프로그램에 대한 평가 요청과 함께 ‘김정은과 접촉하려는 새로운 시도가 있었느냐’는 질문을 받고 “언제, 어디서나 전제조건 없이 만나겠다는 우리의 제안은 유지되고 있다”고 답변했다 “Our offer remains to meet anywhere, anytime without preconditions,” White House spokeswoman Jen Psaki said to a question asking about the current understanding of what North Korea is doing with their nuclear program and if there is any renewed outreach to Kim Jong Un and his regime

白宫发言人普萨奇在新闻发布会上就“是否有与金正恩接触的新尝试”的提问答称:“我们的提议是无论何时何地都要在没有前提条件的情况下会面”

방미 중인 노규덕 한반도평화교섭본부장은 이날 워싱턴 특파원들과의 간담회에서 “한미는 양국이 공동으로 추진할 수 있는 대북 인도적 분야 협의를 하는 등 북한에 관여할 다양한 방안을 협의 중”이라며 “북한이 호응한다면 언제든 추진하도록 만반의 준비를 한다는 게 양국의 공통된 입장”이라고 했다 “South Korea and the U S are discussing various measures to get involved in North Korean matters, including consultation on joint humanitarian assistance for the North,” Special Representative for Korean Peninsula Peace and Security Affairs Noh Kyu-duk said in a press conference during his visit to the U S. “South Korea and the U S share the common stance to be fully prepared to pursue things anytime as soon as North Korea reciprocates ”

正在访美的韩半岛和平交涉本部长鲁圭德当天在华盛顿特派记者会上表示:“韩美两国正在就可以共同推进的对朝人道领域等与朝鲜有关的多种方案进行磋商,两国的共同立场是,做好一切准备,一旦朝鲜响应就可以推进”

中 허난성 물폭탄에 최소 33명 사망…전세계 아이폰 절반 공급 공장도 타격Heavy rain affects 3 million in China’s Henan province, 33 dead

中国河南省大暴雨至少造成33人死亡…全球一半苹果手机供应工厂也受到打击

중국 중부 허난성 성도(省都) 정저우에 60년 만의 기록적인 폭우가 내린 가운데 22일 현재 최소 33명이 사망하고 25만6000명이 대피했다 Zhengzhou, the capital city of Henan Province, China, suffered the worst flood in 60 years, leaving at least 33 dead and around 256,000 evacuated as of Thursday

中国中部河南省省会郑州遭遇60年来罕见的特大暴雨,截至22日,至少造成33人死亡,25.6万人被迫撤离

300만 명이 넘는 이재민이 발생하는 등 최악의 홍수 사태에 대만의 차이잉원(蔡英文) 총통이 이례적으로 위로 메시지를 발표했다 With the number of the displaced hitting the mark of 3 million, Tsai Ing-wen, the president of Taiwan, sent her message of concern to Henan, setting a rare precedent

中国台湾省省长蔡英文罕见地就引发超过300万名灾民等最严重的洪灾表示慰问

22일 중국 허난성 당국에 따르면 이날 오전까지 확인된 사망자는 최소 33명으로 전날 25명보다 8명 증가했다 According to the provincial authorities of Henan, at least 33 were drowned by the downpour, killing 8 more from a day before

据中国河南省当局22日透露,截至当天上午,确认的死亡者至少有33人,比前一天的25人增加了8人

지하철 침수로 정저우에서만 12명이 사망한 것을 포함해 각 지역에서 산사태 등으로 매몰됐던 사람들이 확인되면서 사망자가 늘었다 Including the 12 passengers in Zhengzhou flood alone, the death toll soared with more victims found from the landslides in parts of the province

因地铁被淹,仅郑州就有12人死亡,随着各地区因泥石流等被埋的人得到确认,死亡人数有所增加

현재까지 발생한 이재민은 300만4000명이고, 25만6000명이 긴급 대피했다 The number of people affected by the flood stands at 3 million and 4,000, with 256,000 having been evacuated

截至目前,共有300.4万人受灾,25.6万人紧急避难

허난성은 “직접적 경제적 손실만 12억2000만 위안(약 2239억6500만 원)”이라고 밝혔다 “We’ve suffered direct economic losses worth 1.2 billion and 20 million yuan (around 223 9 billion and 65 million won),” a provincial official of Hanan said

河南省表示:“仅直接经济损失就达12.2亿元人民币(约2239.65亿韩元)”

허난성은 면적이 한국의 1,6배이며 인구는 1억 명 정도다 Hanan is 1.6 times larger than South Korea, with a population of 100 million

河南省的面积是韩国的1.6倍,人口数达1亿左右

중국의 비 피해가 심각해지자 그동안 중국과 첨예한 대립각을 세워온 대만의 차이 총통은 자신의 명의로 된 위로 메시지를 발표했다 이날 대만 중앙통신사에 따르면 차이 총통은 “불행히 숨진 사람과 그 가족들에게 애도를 표하고 재해 지역이 조기에 정상적인 생활로 돌아오기를 바란다”고 전했다 Tsai Ing-wen, the president of Taiwan, expressed her concern and sent her condolences to the victims, saying she hopes that “normal life could resume as soon as possible,” according to Taiwan’s Central News Agency

随着此次河南省雨灾越来越严重,此前一直与中国内地针锋相对的蔡英文也以自己的名义发表了慰问信息据台湾《中央通讯社》当天报道,蔡英文表示,“向不幸遇难的人及其家属表示哀悼,希望灾区早日恢复正常生活”

정저우에는 애플 아이폰을 위탁생산하는 대만 폭스콘 공장이 있어 아이폰 공급 차질도 예상된다 The supply of the iPhones is expected to be affected by the heavy rain as Taiwan’s Foxconn factory is located in Zhengzhou

郑州有委托生产苹果iPhone手机的台湾富士康工厂,预计iPhone的供应也会受到影响

폭스콘은 정저우에서 3개 공장을 운영하고 있으며 약 35만 명의 인력이 90개 생산 라인에 투입돼 있다 Foxconn operates three factories in Zhengzhou, with some 350,000 workers on 90 production lines

富士康在郑州运营3家工厂,约35万人力投入到90条生产线上

이곳에서 생산되는 아이폰은 전 세계 아이폰 물량의 절반 이상을 차지한다 Foxconn’s Zhengzhou factory is responsible for more than half of the global production of the iPhones

在这里生产的iPhone手机占全世界iPhone手机数量的一半以上

월스트리트저널(WSJ)은 폭스콘 직원들을 인용해 “20일 오후 폭스콘 공장 세 곳 모두 수 시간 동안 전기가 끊기고 일시 정전됐다”고 보도했다 “All three Foxconn factories in the city experienced hours of power outages Tuesday evening as the city went through a blackout,” the Wall Street Journal reported, quoting the employees of Foxconn

《华尔街日报》援引富士康职员的话报道说:“20日下午,富士康三家工厂全部停电数小时,并暂时断电”

한 직원은 공장 내 물이 허벅지까지 차오르면서 사람들이 생산 설비와 재고품들을 부랴부랴 옮겼다고 WSJ에 전했다 An assembly worker said he was submerged up to his thighs, with some workers moving production equipment and inventories to prevent them getting wet

一名职员向WSJ表示:“工厂内的水涨到大腿处,人们急忙搬运生产设备和库存品”

文 만난 셔먼 “中과 대북정책 심도있게 논의할 것” Sherman: U S will discuss with China over N Korea policy U S

舍曼会见文在寅,“将与中国深入讨论对朝政策”

한국을 방문 중인 미국 국무부 ‘넘버2’인 웬디 셔먼 부장관이 22일 문재인 대통령과 만나 “중국을 방문해 대북정책과 관련한 심도 있는 논의를 할 것”이라고 밝혔다 Deputy Secretary of State Wendy Sherman, the No 2 diplomat in the U S State Department, who is currently visiting South Korea, met President Moon Jae-in on Thursday and stated that she will have a thorough discussion on the U S policy towards North Korea in a forthcoming visit to China

正在韩国访问的美国副国务卿温迪·舍曼22日会见了韩国总统文在寅他表示:“将在访问中国时就对朝政策进行深入讨论”

미중은 최근 경제 이슈와 별개로 북한 문제 등 외교 현안 등에만 초점을 맞춘 정례 대화 채널을 다시 가동하기 시작한 것으로 알려져 북-미 대화 재개를 둘러싸고 미중이 어떤 협의를 할지 주목된다 Washington and Beijing are known to have recently resumed regular high-level talks strictly focused on diplomatic relations, including concerns over North Korea, while keeping a distance from the recent contention over economic matters

据悉,美中两国最近在经济问题之外,又重新启动了将焦点放在朝鲜问题等外交悬案等方面的定期对话渠道,因此,围绕朝美重启对话问题,美中将进行怎样的磋商备受关注

셔먼 부장관은 한국에 이어 25, 26일 중국을 방문해 왕이 중국 외교부장과 회담한다 U S Deputy Secretary of State Wendy Sherman and Chinese Foreign Minister Wang Yi are scheduled to meet on Sunday and Monday

舍曼将在访问韩国后,于25日至26日访问中国,并与中国外交部长王毅举行会谈

문 대통령은 이날 청와대에서 셔먼 부장관을 접견하고 “앞으로 북-미 대화 재개를 위해 적극 노력해 달라”고 당부했다 President Moon met Deputy Secretary Sherman at Cheong Wa Dae and requested that the U S play an active role to restart discussions between Washington and Pyongyang

文在寅当天在青瓦台接见舍曼,表示“希望今后为重启朝美对话积极努力”

이에 셔먼 부장관은 “북한이 미국의 대화 제의에 조기 호응해 오기를 기대한다”며 “대북정책과 관련해 긴밀히 조율된 노력을 함께해 나가길 바란다”고 답했다 “The U S looks forward to a prompt positive response on the resumption of dialogue from North Korea We hope to continue our closely coordinated efforts for dialogue with North Korea,” Ms Sherman said

对此,舍曼答道:“期待朝鲜对美国的对话提议早日作出响应”,“希望就对朝政策共同进行紧密协调的努力”

（增译）特别是,舍曼表示:“韩流明星‘防弹少年团’的《允许跳舞(PermissiontoDance)》在全世界很受欢迎,但因为韩美很合拍,所以不需要‘允许’”

외교 소식통에 따르면 미중은 도널드 트럼프 미 행정부 때 사실상 단절됐던 정례적 외교 채널을 최근 재가동했다 According to a person familiar with foreign affairs, the U S and China have recently reopened a diplomatic channel, which had been practically severed during the Trump administration

分析认为,这是在强调巩固的韩美同盟非常重要据外交消息人士称,中美最近重新启动了美国特朗普政府时期中断的例行外交渠道

소식통은 “이 채널에선 미중 양국이 협력 필요성을 제기한 북한 문제 등 외교 사안 중심의 협의가 이뤄질 것으로 보인다”며 “미중이 경제와 외교를 별개로 보는 투트랙 접근에 나선 것”이라고 전했다 An official declined to be named said that the resumed talks are expected to revolve around matters concerning diplomatic relations, including North Korea policy, which both Washington and Beijing agreed on the need to cooperate on The source further said that the two countries will adopt the two-track approach that separates economy from diplomacy

消息人士透露说:“在这个渠道中,中美两国可能会以提出合作必要性的朝鲜问题等外交悬案为中心进行磋商

“中, 선양수용소 수감된 탈북자 50여명 北 보내” Beijing sent over 50 defectors in detention back to NKorea

美中两国开始采取将经济和外交分开看待的‘双轨’处理方式

중국 정부가 랴오닝성 선양 수용소에 수감 중이던 탈북자 50여 명을 14일 북한으로 보냈다고 자유아시아방송(RFA)이 16일 보도했다 The Chinese government sent more than 50 North Korean defectors who had been in detention in Shenyang, Liaoning Province back to North Korea, Radio Free Asia reported on Friday

自由亚洲电台:“中国将被关押在沈阳收容所的50多名‘脱北者’遣返朝鲜”自由亚洲电台16日报道说,中国政府14日将关押在辽宁省沈阳收容所的50多名“脱北者”遣返朝鲜

중국은 이들을 4월부터 북송하려 했는데 신종 코로나바이러스 감염증(코로나19) 환자 유입을 우려한 북한이 몇 차례 거부해 늦어진 것으로 알려졌다 China had sought to repatriate them to the North since April, but Pyongyang reportedly declined to receive them several times due to concern over possible inflow of Covid-19 patients, which resulted in delays

据悉,中国原计划从4月份开始将这些人送回朝鲜,但由于朝鲜担心出现新冠患者,多次拒绝送回,所以被推迟

북송된 이들은 북한에서 극형을 면하기 어려울 것으로 보인다고 RFA는 전했다 RFA said the ill-fated defectors will most likely face execution in the North

据自由亚洲电台透露,被遣返朝鲜的这些人在朝鲜将很难避免被处以极刑

중국 당국은 선양 수용소에 1, 2년가량 수감돼 있던 탈북자들을 14일 단둥 국경 세관을 거쳐 북한으로 보냈다 The Chinese authority sent North Korean defectors, who had been detained at the Shenyang Detention Center for one or two years, via the customs office in Dandong, China on July 14

中国有关部门14日通过丹东边境海关,将被关押在沈阳收容所一两年左右的“脱北者”送往朝鲜

보도에 따르면 버스 2대가 탈북자들을 나눠 실었고 공안 수십 명이 오전 일찍부터 세관 주변에서 경계를 서며 사람들이 북송 장면을 촬영하지 못하게 한 것으로 전해졌다 According to the RFA report, the defectors were carried in two buses, and dozens of Chinese police officers were watching them around the customs office from early in the morning, while blocking people from taking photos or video

据报道,2辆巴士分运载“脱北者”,数十名公安从一早开始在海关周围站岗,不让人们拍摄遣返场面

북송된 50여 명 중에는 북한군 병사와 공군 파일럿 출신도 있었다 The 50-plus defectors, who were repatriated to the North, included a number of North Korean soldiers and Air Force pilots

被遣返朝鲜的50多人中,还有朝鲜士兵和空军飞行员出身

30대 탈북 여성은 중국인 남성과 결혼해 12세 아들을 뒀고, 중국에서 상당히 많은 돈을 벌었던 것으로 알려졌다 A 30-something female defector who married to a Chinese man has a 12-year-old son She reportedly earned a sizable amount of money in China

据悉,一名30多岁的女“脱北者”与一名中国男子结婚,生有一个12岁的儿子,还在中国赚了很多钱

한 소식통은 “이 여성은 두 번째 북송되는 것이어서 생사를 가늠할 길이 없다 “The woman was repatriated to the North for a second time, and there is no way of knowing her fate

一名消息人士表示:“这名女性是第二次被遣送回朝鲜,因此生死难以预料

남편이 아내를 구하기 위해 뇌물을 쓰려 했지만 통하지 않았다”고 전했다 Her husband tried to bribe officials to save her, to no avail,” an informed source said

丈夫为了救妻子曾想行贿,但行不通”

RFA에 따르면 선양 수용소에는 이날 북송된 50여 명 외에도 탈북자들이 더 남아 있다 According to RFA, the Shenyang Detention Center still has a number of North Korean defectors in detention, apart from the 50-plus repatriated defectors

据自由亚洲电台透露,沈阳收容所内除了当天遣返朝鲜的50多人以外,还有更多的“脱北者”

코로나19 방역 등을 위해 닫혀 있던 단둥 세관이 이날 하루 개통하면서 그동안 북한에 머물고 있던 화교와 북한 무역대표부 관계자 등 98명도 중국으로 이동했다 As the customs office in Dandong reopened on the day after shutdown for Covid-19 quarantine on the day, 98 people including Chinese nationals who were staying in the North and representatives of the North’s trade office moved to China

随着为防新冠疫情而关闭的丹东海关当天正式开通,此前滞留在朝鲜的华侨和朝鲜贸易代表部有关人员等98人也来到了中国

‘치사율 80%’ 원숭이B바이러스, 中서 첫 사망자원숭이로부터 매우 드물게 옮는 바이러스 감염증 환자가 중국과 미국에서 잇따라 발생했다 First human death due to monkey B virus reported in China Patients of rare viral infections transmitted from the monkey have been reported in China and the U S in succession

“致死率80%”,中国首例人类感染猴B病毒致死病例在中国和美国接连出现了来自于猴子身上非常罕见的病毒的感染患者

17일 중국 관영 매체 글로벌타임스에 따르면 베이징의 영장류 연구기관에서 일하던 53세 수의사가 ‘원숭이 B 바이러스’에 감염돼 치료받다가 5월 27일 사망했다 According to China’s state-run Global Times on Saturday, a 53-year-old veterinarian who was working at a primate laboratory in Beijing died on May 27 while taking treatment for “monkey B virus

据中国官方媒体《环球时报》17日报道,在北京一家专门从事非人灵长类研究机构工作的53岁兽医因感染“猴B病毒”而接受治疗,最终于5月27日死亡

이 수의사는 올해 3월 죽은 원숭이 2마리를 해부하면서 이 바이러스에 감염됐고, 그로부터 한 달 뒤 메스꺼움과 구토 증세를 보인 것으로 조사됐다 ” The veterinarian was infected with the virus while dissecting two dead monkeys in March this year He then came down with symptoms including nausea and vomiting beginning one month later

据调查,该兽医在今年3月解剖2只死猴子时感染了该病毒,并在一个月后出现了恶心和呕吐的症状

중국에서 사람이 이 바이러스에 감염된 것은 처음이라고 글로벌타임스는 전했다 The Global Times said it is the first case of human infection with the animal virus in China

《环球时报》报道称,这是中国首次有人感染这种病毒

이 수의사와 접촉한 사람들 중 추가 감염자는 없다고 한다 There are reportedly no additional infections among people who came into contact with the veterinarian

据说,接触过该兽医的人中没有新的感染者

원숭이 B 바이러스는 헤르페스 바이러스의 일종으로 감염된 원숭이에게 물리거나 긁힐 경우, 또는 원숭이의 분비물이 사람의 눈 점막 같은 곳에 튈 경우 전염될 수 있다 The monkey B virus is a type of the herpes virus A person can be infected with the virus if he or she is bitten or mauled by the infected monkey, or when secretion from the monkey is splattered onto human mucous membrane such as an eye

猴B病毒是疱疹病毒的一种,如果被感染的猴子咬伤或抓伤,或者猴子的分泌物溅到人的眼黏膜等部位,就可能会被传染

사람에게서 사람으로도 감염될 수 있는 바이러스로 치사율이 70∼80%에 이른다 The virus can transmit between humans, with a case fatality rate reaching as high as 70 to 80 percent

这是一种从人到人都能感染的病毒,致死率高达70%至80%

미국 텍사스주 북부 댈러스에서는 희소 감염병인 원숭이두창(Monkeypox) 바이러스 환자가 발생했다고 16일 미국 워싱턴포스트(WP)가 보도했다 Meanwhile The Washington Post reported Friday a person was infected with the monkeypox, a rare infectious disease in Dallas, Texas in the U S

据美国《华盛顿邮报》(WP)16日报道,美国得克萨斯州北部达拉斯发现了罕见传染病“猴头疮(Monkeypox)”病毒患者

댈러스카운티 보건당국은 최근 아프리카 나이지리아를 방문한 댈러스 주민이 이 바이러스에 감염된 사실을 확인했다고 이날 밝혔다 The health authority of the Dallas County said the Dallas resident who had visited Nigeria was confirmed as a patient infected with the virus

达拉斯县保健当局当天表示,已确认最近访问非洲尼日利亚的达拉斯居民感染了该病毒

이 환자는 이달 8일 나이지리아 라고스에서 미국 델타항공 비행기를 타고 애틀랜타를 경유해 9일 댈러스에 도착했다 The patient took a Delta Airline flight in Lagos, Nigeria on July 8 and arrived in Dallas on July 9 via Atlanta

该患者于本月8日从尼日利亚拉各斯乘坐美国达美航空公司的飞机,经亚特兰大于9日抵达达拉斯

미 질병통제예방센터(CDC)가 비행기에서 환자와 접촉한 사람들을 파악하고 연락을 취하고 있다 The U S Centers for Disease Control and Prevention is tracking and contacting people who came into contact with the patient inflight

美国疾病控制和预防中心(CDC)正在掌握在飞机上与患者接触的人,并与相关人员取得联系

댈러스카운티 당국은 “신종 코로나바이러스 감염증(코로나19) 예방을 위해 마스크 착용이 요구됐기에, 이 바이러스가 비행기나 공항에서 비말을 통해 다른 사람들에게 확산했을 위험은 낮다”고 밝혔다 “Due to mandatory mask requirement for the prevention of Covid-19, there is little chance that the virus has spread to other people through droplets,” the Dallas County health authority said

达拉斯县当局表示:“为了预防新型冠状病毒肺炎(COVID-19)疫情,要求佩戴口罩,因此该病毒在飞机或机场通过飞沫扩散到其他人的危险很低”

원숭이두창 바이러스는 우두 바이러스와 비슷한 종류로 1958년 처음 발견됐다 （no translation）

猴头疮病毒与牛痘病毒相似,于1958年首次被发现

사람이 감염되면 독감과 비슷한 증상을 보이면서 림프샘이 붓다가, 얼굴과 몸에 넓게 발진이 나타난다 （no translation）

如果人类感染,会出现与流感相似的症状,淋巴腺会肿胀,脸部和身体会出现大范围的疹子

감염자는 100명 중 1명꼴로 사망한다고 CDC는 밝혔다 （no translation）

美国疾病控制和预防中心表示,每100人中就有1人死亡

인간 감염 사례는 1970년 콩고민주공화국에서 최초로 확인됐다 （no translation）

人类感染事例在1970年刚果民主共和国首次得到确认

미국에서는 2003년 아프리카 가나에서 수입된 원숭이에게서 감염이 시작돼 47명의 감염자가 나온 바 있다（no translation）

在美国,2003年从非洲加纳进口的猴子身上开始感染,共出现了47名感染者

北과 밀착하는 中 “美, 수십년간 北위협 반성해야” China criticizes U S for several decades’ menace to NKorea

中国与朝鲜靠近,“称美国应对数十年威胁朝鲜反省”

중국이 미국을 향해 “수십 년 동안 북한에 가한 위협과 압박을 반성해야 한다”며 이례적으로 강도 높게 비판했다 China has raised an unprecedentedly critical voice against the United States that it is supposed to show some remorse for intimidating and pressing North Korea for several decades

中国罕见地强烈谴责美国称:“美国应该反省数十年来对朝鲜施加的威胁和压力”

4일 중국 외교부에 따르면 왕이(王毅) 중국 외교 담당 국무위원 겸 외교부장(사진)은 전날 칭화대에서 열린 제9차 세계평화포럼에 참석해 “한반도 핵 문제는 최근 30년 동안 질질 끌면서 우여곡절을 반복했다”며 이처럼 주장했다 Chinese State Councilor and Foreign Minister Wang Yi said in the 9th World Peace Forum in Tsinghua University on Saturday that nuclear issues on the Korean Peninsula have been dragged with ups and downs all the way for the past 30 years, according to the Chinese Foreign Ministry on Sunday

据中国外交部4日消息,中国负责外交事务的国务委员兼外交部长王毅前一天出席了在清华大学举行的第九届世界和平论坛,并在会上同时表示:“韩半岛核问题在最近30年里一直拖延,几经波折”

그는 “(북한과) 대화와 협상을 통한 평화적 해결이 기본 원칙이고, 한반도 비핵화와 평화체제 구축을 병행하는 게 올바른 길”이라고 강조했다 "The default option to make is to resolve issues peacefully based on dialogue and negotiations We should take the right path by working on denuclearization and peace-making efforts at the same time,” he said

他强调:“通过(与朝鲜)对话和谈判和平解决是基本原则,韩半岛无核化与和平体制的构建并行是正确的道路”

왕 부장은 “한반도의 일은 중국 문 앞의 일”이라며 “중국은 한반도의 안정을 위해 일관되게 건설적인 역할을 할 것”이라고 말했다 Minister Wang said that issues regarding the Korean Peninsula are unfolding right in front of China's doorstep, stating that the Chinese government will have a constructive role in ensuring stability on the Korean Peninsula with consistency

王毅表示:“韩半岛的事情是中国家门口的事情为了韩半岛的稳定,中国将一如既往地发挥建设性作用”

성 김 미국 국무부 특별대표가 북-미 대화 재개 가능성을 타진하기 위해 한국을 방문하는 등 최근 미국의 움직임에 대해선 “한반도 평화와 안정에 도움이 되는 모든 언행을 지지한다”고 밝혔다 Regarding Washington's recent moves including U S special representative for North Korea Sung Kim's visit to Seoul increase possibilities of the resumption of the U S -North Korea dialogue front, Mr Wang said that Beijing is supportive of all kinds of Washington's acts and statements that can bring peace and stability on the Korean Peninsula

对于美国国务院朝鲜事务特别代表金圣为试探朝美对话重启的可能性而访问韩国等最近美国的动向,他表示:“支持所有有助于韩半岛和平与稳定的言行”

그는 이날 연설에서 신장위구르와 홍콩의 인권 문제 등에 대한 서방의 비판에 대해 ‘중국 내정에 간섭하지 말라’는 입장을 다시 밝혔다 In the address on Saturday, Minister Wang clarified in response to criticism from the West for issues with the Xinjiang Uygur and human rights in Hong Kong that there should be no intervention in domestic affairs occurring in China

他在当天的演讲中就西方对新疆维吾尔自治区和香港人权问题等的谴责再次阐明了“不要干涉中国内政”的立场

대만에 대해서는 ‘분할할 수 없는 중국 영토의 일부’라고 전제한 뒤 “조국의 평화통일을 추진하는 것은 중국 정부가 견지해온 방침으로, 미국 일부 세력이 대만 독립 세력을 지원하는 것은 매우 잘못되고 위험한 것”이라고 경고했다 Defining Taiwan as an undividable part of Chinese territory, he argued that China's pursuit of a nationwide peaceful unification has been maintained for so long, warning that it is a wrongful and risky act for some forces in the United States to support the independence of Taiwan

他就台湾问题表示:“台湾是中国领土不可分割的一部分”他同时警告说:“推进祖国和平统一是中国政府一直坚持的方针,美国部分势力支援台独势力是非常错误和危险的”

왕 부장은 일본 정부의 후쿠시마 원전 오염수 해양 방류 결정에 대해 “일본 정부는 국제사회의 합리적인 관심을 충분히 경청하고 관련 국가와 국제기구의 협상 전에 함부로 태평양에 오염수를 방류해서는 안 된다”고 강조했다 As for Japan's decision to discharge contaminated nuclear water from nuclear reactors in Fukushima, the Chinese minister stressed that the Japanese government is not supposed to discharge contaminated water in the Pacific Ocean before it listens to the international community's reasonable concerns and worries and completes full negotiations with related neighboring nations and international organizations

对于日本政府决定向海洋排放核电站污染水,王毅强调:“日本政府应充分倾听国际社会的合理关注,在与有关国家和国际机构进行磋商之前,不能随意向太平洋排放污染水”

칭화대가 운영하는 ‘세계평화포럼 사무국’이 주최하는 세계평화포럼은 2012년에 발족됐다중국에서 비정부기구가 주최하는 유일한 국제안보 관련 글로벌 포럼이다 The World Peace Forum, which was founded by Tsinghua University in 2012, has become the only global forum to discuss international security issues by a non-governmental organization in China

清华大学运营的“世界和平论坛秘书处”主办的世界和平论坛成立于2012年这是中国唯一由非政府机构主办的与国际安保相关的全球论坛

北 ‘코로나 국경봉쇄’ 1년만에 中과 교역 재개 N Korea resumes trading with China in more than a year

北韩“新冠疫情封锁边境”时隔一年重启与中国的贸易

북한이 북-중 접경지역을 중심으로 최근 중국과 제한적으로 물자 교류를 재개했다 North Korea limitedly resumed resource trading with China recently, revolving around the border areas

朝鲜以中朝边境地区为中心,最近与中国进行了有限的物资交流

생필품 부족으로 불만이 쌓인 주민들을 달래기 위해 공식적으로는 국경 폐쇄 상태를 유지한 채 ‘비공식적인’ 교역에 나선 것으로 보인다 It seems that the North began “unofficial” trading while keeping the border officially closed to console residents who have complaints built up due to the lack of daily necessities

为了安抚因生活必需品不足而不满的居民,朝鲜官方在维持边境封闭状态的情况下开始了“非正式”交易

북한은 그동안 신종 코로나바이러스 감염증(코로나19) 확산에 대한 공포로 1년 넘게 국경을 봉쇄해 왔다 Pyongyang has closed off the border for longer than a year for fear that COVID-19 would spread

此前,北韩因对新型冠状病毒肺炎(COVID-19)扩散的恐惧,封锁边境超过一年

일각에선 이러한 교역 재개가 11일 북-중 우호협력조약 60주년을 앞두고 양국이 공조 체제를 조이는 신호라는 해석도 나온다 Some interpret this as a signal that the two countries started to tighten up coordination for the coming 60th year of signing an amicable treaty on July 11

也有人分析说,这种交易重启是在11日《中朝友好合作条约》签署60周年之际,两国收紧合作体制的信号

2일 정부 소식통에 따르면 북-중 무역 거점 도시인 중국 랴오닝(遼寧)성 단둥(丹東)시 등을 중심으로 지난달 말 일부 물자가 오간 것으로 전해졌다 According to a South Korean government source on Friday, some resources have been exchanged in late June in trading hubs including Dandong, Liaoning province in China

据韩国政府消息人士2日透露,上月底,以北韩-中国贸易据点城市中国辽宁省丹东市为中心,部分物资来往

소식통에 따르면 교류는 트럭 등을 이용해 육로로 매우 제한적인 수준에서 이뤄졌다고 한다 The source added that the exchange was done by land in a very limited level using trucks and other vehicles

据消息人士介绍,交流利用卡车等,通过陆路进行小范围的物资交流

시진핑 “中 괴롭히면 머리 깨져 피 흘리게 될것” Xi: Those bullying China will have their heads bashed and bloodied

习近平:“谁妄想欺负中国,必将头破血流”

시진핑(習近平·사진) 중국 국가주석이 1일 중국공산당 100주년을 맞아 “중화민족이 괴롭힘을 당하는 시대는 끝났다 Celebrating the 100th anniversary of the founding of the Communist Party of China on Thursday,

在7月1日中国共产党成立100周年之际,中国国家主席习近平(照片)表示:“中华民族受欺凌的时代已经结束了

외부 세력이 우리를 괴롭히면 14억 인민의 피와 살로 만든 강철 만리장성에 머리가 깨져 피가 흐를 것”이라고 밝혔다 Chinese President Xi Jinping said foreign forces that bully China will “find their heads bashed bloody against a great wall of steel forged by over 1· 4 billion Chinese people

任何外来势力妄想欺负我们,必将在14亿多中国人民用血肉筑成的钢铁长城面前碰得头破血流”

그는 “대만과의 완전한 통일을 이끌어내는 것이 새 의무”라며 미국 등 서방이 대만, 홍콩 문제 등에 개입하면 강경 대응할 뜻을 천명했다 ” President Xi declared his intention to take a resolute action if foreign countries, such as the U S intervene in its own issues with Taiwan and Hong Kong, saying it is China’s “historic task” to complete reunification with Taiwan

他表示,“同台湾实现祖国完全统一是新的义务”,如果美国等西方国家介入台湾 香港等问题,将采取强硬应对

1월 출범 후 내내 중국을 거세게 압박해온 조 바이든 미 행정부에 일종의 선전포고를 했다는 관측이 제기된다 His remarks are regarded as a declaration of war against the Joe Biden administration, which has continued pressure on China since it took office in January

有人分析认为,这是对1月份上台以后一直对中国施加强大压力的美国拜登政府的一种宣战

시 주석은 이날 베이징 톈안먼(天安門)광장의 창당 100주년 경축대회에서 “중화민족은 세계에서 가장 위대한 민족으로 5000년이란 유구한 문명과 역사를 가지고 인류문명 발전에 불멸의 공헌을 했다”며 “With a history of more than 5,000 years, China has made indelible contributions to the progress of human civilization,” President Xi said at the 100th anniversary celebrations held at Tiananmen Square on Thursday

习近平当天在北京天安门广场的建党100周年庆祝大会上表示,“中华民族是世界上伟大的民族,有着5000多年源远流长的文明历史,为人类文明进步作出了不可磨灭的贡献”

“누구도 주권과 영토를 보전하려는 중국의 굳은 결심과 확고한 의지, 강한 능력을 과소평가하면 안 된다”고 말했다 “No one should underestimate the resolve, the will, and the ability of the Chinese people to defend their national sovereignty and territorial integrity

“任何人都不要低估中国人民捍卫国家主权和领土完整的坚强决心 坚定意志 强大能力”

그는 마오쩌둥(毛澤東)이 1949년 중화인민공화국 건국을 선포한 톈안먼 망루 위 연단에 마오와 똑같은 회색 중산복을 입고 등장해 ‘사회주의 현대화 강국 전면 건설’이란 제2의 100년 목표를 제시했다 ” Speaking from the podium, where Mao Zedong proclaimed the People’s Republic of China in 1949, wearing the same gray buttoned suit like Mao’s, President Xi presented the country’s second centenary goal of fully building a modern socialist country and Chinese dream of national rejuvenation

习近平身穿毛泽东在1949年宣布中华人民共和国建国的天安门城楼上时一模一样的灰色中山服登场,他提出了“全面建设社会主义现代化强国”的第二个百年目标

첫 번째 100년 목표였던 ‘샤오캉(小康·모든 국민이 편안하고 풍족한 생활을 누림)’ 사회를 실현한 만큼 이제 국제사회에서 패권국 위치를 강화하는 데 힘쓰겠다는 뜻을 밝혔다 President Xi said that his country will strive to strengthen its hegemony in the international society now that the country has realized the first centenary goal of building a moderately prosperous society

他表示,既然实现了第一个百年目标——实现了“小康社会”,现在就要努力在国际社会上加强霸权国家的地位

공산당 창당 100주년 앞둔 베이징 ‘반계엄 상태’중국이 다음 달 1일 공산당 창당 100주년 기념일을 앞두고 사실상 반(半)계엄 상태에 돌입했다고 대만 쯔유(自由)시보가 23일 보도했다 Beijing is practically under martial law with 100th anniversary of Chinese Communist Party’s foundation The Taiwanese Liberty Times reported on Wednesday that China is practically under martial law with the 100th anniversary of the Chinese Communist Party’s foundation on next Thursday

即将迎来建党100周年,北京处于“半戒严”状态台湾《自由时报》23日报道,即将迎来7月1日中国共产党建党100周年纪念日之际,中国实际上已进入半戒严状态

100주년을 기념하는 불꽃 축제와 각종 문화 공연을 성대하게 준비하면서도 인력, 물자 이동 등을 철저히 차단해 공산당과 시진핑 국가주석의 장기 집권에 대한 불만을 막겠다는 의도로 풀이된다 While fireworks and other cultural performances are being prepared to celebrate the 100th anniversary, the movement of people and resources is strictly controlled to prevent any complaint against the long-term rule by the Communist Party and President Xi Jinping

分析认为,中国是在准备盛大的100周年烟花庆典和各种文化演出的同时,彻底切断人员和物资流动等,以防止对共产党和习近平国家主席长期执政的不满

중국 당국은 21일부터 수도 베이징으로 들어가는 모든 택배에 대해 2단계 전수조사를 실시하고 있다 The Chinese authorities have been conducting two-step examinations for all packages heading to Beijing since Monday

中国当局从21日开始对进入首都北京的所有快递进行了两阶段专门调查

우선 발신지 택배회사에서 베이징으로 보내는 모든 택배를 엑스레이 검사기로 검사한 후 보안검색 완료 표시를 붙인다 First, a shipping company of the dispatch location scans all packages with an X-ray machine and puts a sticker for a completed security check on them

首先用X光检查机检查发送地快递公司寄往北京的所有快递,然后贴上完成安全检查的标志

이후 택배가 도착한 베이징 현지에서 다시 검사를 진행한다 Then, another round of examinations is conducted in Beijing once packages arrive

之后在快递到达的北京当地再次进行检查

당국은 다음 달 1일까지 2단계 전수조사를 하겠다고 밝혔다 The authorities said such a two-step examination for all packages will be carried out until next Wednesday

当局表示,到7月1日为止,将展开第二阶段全面调查

이로 인한 배달 지체를 우려하는 목소리도 높다 Many people are worried about potential delays in shipping as a result

很多人担心会因此造成送货延误

온라인 쇼핑과 택배가 발달한 중국은 경제가 발달한 남부 광둥성, 상하이, 선전 등에서 생산된 물건이 택배를 통해 베이징으로 많이 들어온다 As a country with highly developed online shopping and shipping industries, many packages are delivered to Beijing from Guangdong Province, Shanghai, and Shenzhen in the southern part of the country

在网络购物和快递发达的中国,经济发达的南部广东省 上海 深圳等地生产的产品大部分都是通过快递进入北京

과거에는 2, 3일이면 충분했지만 2단계 전수조사가 실시된 후 1주일 이상 걸릴 것이란 지적이 나온다 What normally takes two to three days for shipping will take longer than a week due to the two-step examination

有人指出,过去2 3天就足够,但是在实施第二阶段专门调查之后,将需要一周以上的时间

일부 소비자들은 “온라인 주문을 통해 베이징 외곽에 있는 음식점에서 시내로 음식 배달을 시키는 것도 사실상 차단됐다”고 불만을 토로했다 “Ordering food online from a restaurant in the outskirts of Beijing has become practically impossible,” customers complained

部分消费者表示不满说:“通过网上预订让北京郊区的餐厅往市内送餐实际上也被切断了”

베이징 공안당국은 지난주부터 시내 임대주택 등을 대상으로 가택 방문조사를 하고 있다 （no translation）

北京公安部门从上周起以市内出租屋等为对象,展开了住宅访问调查

각 파출소에서 파견한 검사원들이 직접 집을 방문해 당초 신고된 거주자와 실제 거주자가 동일한지 확인하고 있다（no translation）

各派出所派遣的检查员亲自上门确认当初申报的居住者和实际居住者是否相同

특히 베이징에 거주하는 외국인에 대한 조사를 강화하는 것으로 알려졌다 （no translation）

据悉,特别加强了对居住在北京的外国人的调查

이 외에도 베이징 등 9개 주요 대도시에서는 드론을 포함한 모형 항공기, 연, 풍선 등 모든 비행물체를 띄우는 것 또한 금지됐다 （no translation）

除此之外,北京等9个主要大城市禁止放飞无人机 模型飞机 风筝 气球等所有飞行物体

‘중국의 암행어사’ 기관으로 불리는 공산당 중앙기율검사위원회는 최근 웹사이트를 통해 1930년대 공산당을 배반했던 사람들의 처참한 말로를 소개했다 （no translation）

被称为“中国暗行御史”机构的中共中央纪律检查委员会最近在网站上介绍了20世纪30年代背叛共产党的人的悲惨下场

기율검사위원회는 “당을 배반하지 않겠다는 것이 맹세로만 끝나서는 안 된다”며 공산당에 대한 절대 충성을 압박했다 （no translation）

纪律检查委员会表示,“不背叛党的承诺不能只停留在宣誓上”,向共产党员施加了绝对忠诚的压力

혹시라도 발생할 수 있는 반(反)공산당 행위를 아예 뿌리 뽑겠다는 의지로 해석된다 （no translation）

这表明了要根除反共产党行为的意志

바이든, CIA 등에 “코로나 中실험실 기원 재조사하라” Pres Biden orders CIA to investigate origins of COVID-19

拜登要求中情局“重新调查中国实验室新冠病毒起源”

신종 코로나바이러스 감염증(코로나19)의 기원에 대한 논란이 계속되는 가운데 조 바이든 미국 대통령이 미 정보당국의 판단이 엇갈린 상황이라며 추가 조사를 지시했다 virus As disputes about the origins of the COVID-19 virus continue, US President Joe Biden ordered further investigation into the matter based on the judgment that U S intelligence authorities have diverging opinions

新冠病毒的起源争论持续的情况下,美国总统拜登表示美国情报当局的判断出现分歧,指示进一步调查

바이든 대통령은 26일 성명에서 올 3월 코로나19가 동물과 인간의 접촉으로 시작됐는지, 실험실 사고로 발생했는지 등 기원을 분석하라고 중앙정보국(CIA) 등 정보기관에 지시했다고 밝혔다 President Biden announced in a statement on Wednesday that he ordered in March intelligence agencies, including the CIA, to investigate whether the COVID-19 virus started from contacts between humans and animals outside a lab or was accidentally leaked from a lab

拜登26日在声明中表示:“今年3月,我已向中央情报局等情报部门指示,分析新冠病毒的起源,是动物与人类的接触引起的,还是实验室事故引起的”

바이든 대통령은 최근 이와 관련한 보고를 받은 결과 정보당국이 분명한 결론에 이르지 못했다고 설명했다 The president explained that based on the recent briefings, he believed that the CIA and other intelligence agencies had not yet reached a consensus on the matter

拜登表示,最近听取与此相关的报告的结果,情报当局并没有得出明确的结论

정보기관 중 두 곳은 동물 기원설에, 한 곳은 실험실 유출설에 무게를 두고 있으나 모두 낮거나 중간 정도의 신뢰도만 있다고 밝혔다 He said two agencies lean toward the animal origination theory, while one agency puts more weight on the lab leakage theory – both with only a low or intermediate level of reliability

情报机构中,两处认为动物起源说,另一处认为实验室泄漏说,但均表示可信度低或中等

바이든 대통령은 분명한 결론에 가까워질 수 있도록 분석을 더 해 90일 이내에 다시 보고할 것을 정보당국에 지시했다 The president asked them to redouble their efforts to collect and analyze information that could bring us closer to a definitive conclusion and report back to him in 90 days

拜登指示情报当局,为了接近明确的结论,90天内要再次进行分析报告

미국이 결론이 나지 않은 정보당국의 활동을 공개한 것은 이례적이다 It is unusual for the U S to reveal the unconcluded activity of its intelligence agencies

美国公开没有得出结论的情报部门活动是非常罕见的事情

블룸버그는 미국이 중국 실험실 유출의 가능성을 배제하지 않고 있음을 보여주는 것이라고 분석했다 Bloomberg News reported that the U S intended to deliver a message that it is not excluding the possibility of the virus leaked from a lab in China

彭博社分析说:“这表明美国还没有排除中国实验室泄漏的可能性”

바이든 대통령은 성명에서 “중국이 완전하고 투명하며 증거에 기반한 국제적 조사에 협력할 수 있도록 미국은 같은 생각을 가진 전 세계 동맹들과 함께하겠다”며 중국을 압박했다 “The United States will also keep working with like-minded partners around the world to press China to participate in a full, transparent, evidence-based international investigation and to provide access to all relevant data and evidence,” Biden put pressure on China in the statement

拜登在声明中对中国施压称:“为了让中国配合完全 透明 以证据为基础的国际调查,美国将同全世界具有同样想法的同盟一起行动”

중국은 이에 대해 미국이 코로나19 기원을 정치화시킨다며 반발했다 China opposed the statement and criticized the U S for politicizing the origin of the COVID-19 virus

对此,中国反驳说:“美国企图把新冠病毒溯源政治化”

주미 중국대사관은 바이든 대통령의 성명은 언급하지 않은 채 “모종의 정치 세력이 코로나19 대유행에 대항해야 하는 긴급한 필요는 무시하고 비난 게임에 몰두하고 있다”고 27일 홈페이지를 통해 비난했다 “Since the outbreak of COVID-19 last year, some political forces have been fixated on political manipulation and blame game, while ignoring their people's urgent need to fight the pandemic,” the Chinese embassy in the U S made a statement on its website on Thursday without mentioning President Biden’s statement

中国驻美大使馆27日在网站上谴责说:“某种政治势力无视必须对抗新冠大流行的紧急需要,沉迷于谴责游戏”但内容中并未言及拜登的声明

세계보건기구(WHO)는 코로나19가 중국 우한바이러스연구소에서 유출됐을 가능성이 낮다는 보고서를 내놨지만 최근 월스트리트저널이 우한바이러스연구소가 유출지일 수 있다는 의혹을 보도하면서 발원지를 둘러싼 논란이 커지고 있다 Even though the World Health Organization published a report that the likelihood of the virus leaked from the Wuhan Institute of Virology in China is low, disputes about the origins of the virus are expanding as The Wall Street Journal reported that the lab could in fact be one

世界卫生组织曾发表报告说,新冠病毒来自中国武汉病毒研究所的可能性很低,但最近《华尔街日报》报道说,武汉病毒研究所可能是新冠病毒的发源地,从而使围绕起源的争议愈演愈烈

中대사 “한국, 대만 언급 없었다면 좋았을것”Chinese ambassador takes issue with S Korea’s mentioning T

中国驻韩大使:“韩国如果没有提及台湾就好了”

싱하이밍 주한 중국대사(사진)가 26일 한미 정상회담 공동성명에 대만해협과 남중국해 관련 내용이 “아예 없었다면 좋았을 것”이라고 말했다 aiwan Chinese Ambassador to South Korea Xing Haiming said on Wednesday that it would have been better if the issue of the Taiwan Strait and the South China Sea had not been mentioned in a joint press statement issued by the leaders of South Korea and the U S

中国驻韩大使邢海明(照片)26日表示,如果韩美首脑会谈联合声明中“根本没有台湾海峡和南海相关内容就好了”

우리 정부가 대만 관련 언급이 “매우 원론적인 내용”이라며 진화에 나선 상황에서 대만 문제 거론 자체를 문제 삼고 나선 것 following their summit The ambassador took issue with Taiwan being mentioned in the South Korea-U S joint statement while the South Korea government has attempted to ease China’s discomfort by saying a mention of Taiwan in the joint statement was theoretical and principled

在韩国政府就台湾问题阐明立场称是“非常原则性的内容” 出面灭火的情况下,中国却把韩国提及台湾问题本身视为问题

싱 대사는 “중국과 미국이 화해하기 위해 한국을 비롯한 중간 나라들이 좋은 역할을 하면 좋겠다”면서 한국에 미중 간 균형외교를 요구하고 나섰다 Ambassador Xing asked for a balanced diplomacy between the U S and China, saying he hopes that other nations including South Korea play a role in reconciling the U S and China

邢海明表示,“为了中美两国的和解,希望包括韩国在内的中间国家发挥良好作用”,要求韩国推行中美均衡外交

싱 대사는 이날 MBC 시사프로그램에 출연해 한미 성명에 “중국”이라는 표현이 빠진 데 대해 “(한국이) 많이 노력한다고 평가한다”면서도 “중국 단어가 나오지 않았지만 (중국을) 겨냥한 부분이 있는 것 같다”며 이같이 말했다 Talking to an MBC program on Wednesday, Ambassador Xing said while he appreciates Seoul’s efforts to not directly mention “China” in the joint statement, some part of it appears to be targeted at Beijing

邢海明当天出席MBC时事节目时,就韩美声明中没有使用“中国”一词一事表示:“我认为(韩国)付出了很大努力”

그는 “한중이 수교할 때 이미 대만이 중국의 일부분이라고 명확히 인정했다 He went on to say that Seoul recognized Taiwan as part of China when it established diplomatic relations with Beijing

但他同时表示,“虽然没有使用中国这一词,但似乎有针对(中国)的部分”

남중국해 문제도 우리는 주변국과 협력해서 해결하면 된다고 생각한다”고 했다 and he thinks the issue of the South China Sea can be addressed in cooperation with neighboring countries

他表示:“韩中建交时就已经明确承认台湾是中国的一部分

또 쿼드(미국 중국 일본 인도 4자 협의체) 언급을 겨냥해 “하나의 나라, 몇 개의 나라가 만드는 질서에 대해 우리는 좀 다르게 생각한다”면서 “미국은 자꾸 그룹을 만들어 중국을 포위하려는 경향이 있는데 (한국이) 우리 입장을 많이 고려해주면 대단히 고맙겠다”고 했다 As for the mention of the Quadrilateral Security Dialogue (QUAD) in the joint statement, Ambassador Xing said China has a different idea about the global order shaped by one country or a few countries, adding the US has a tendency to form groups to besiege China and it would be highly appreciated if South Korea could consider China’s position

我认为,关于南海问题,我们也可以同周边国家合作解决”另外,针对提及“四方安全对话(美国 澳大利亚 日本 印度四方协议体)”,邢海明表示:“对于一个国家或者几个国家建立的秩序,我们的想法有些不同,美国有着总是制造小集团包围中国的倾向,如果(韩国)多考虑我们(中国)的立场,将非常感谢”

한국의 쿼드 참여는 안 된다고 주장한 것 He made it clear that China is opposed to South Korea’s participation in QUAD

他是在主张,韩国不能参与“四轴安全对话”

한미가 정상회담을 통해 안보뿐 아니라 경제·첨단기술 분야로 동맹을 확대한 데 대해서도 싱 대사는 경계심을 드러냈다 （no translation）

韩美两国通过首脑会谈不仅在安全领域,还在经济 尖端技术领域扩大了同盟关系

그는 “중국은 미국처럼 (자국의) 기술을 다른 나라에 안 주는 식으로 통제한 적이 한 번도 없다”며 “(중국이) 10년 안에 22조 달러를 해외로부터 수입할 텐데 한국이 국익을 판단해 이런(중국) 시장을 활용해 경제 발전을 하는 게 맞다고 생각한다”고 했다 （no translation）

对此,邢海明表现出警戒心他说:“中国从来没有像美国一样,以不把(本国的)技术给别人的方式进行控制过,一次也没有,(中国)在10年内要从海外进口22万亿美元(商品),我认为,韩国判断国家利益,利用(中国的)这种市场发展经济,才是正确的”

다만 이날 싱 대사의 발언은 한미 정상회담 이후 나온 중국의 반발 수위에서 더 나아가지 않았다 However, Ambassador Xing’s reaction was not stronger than China’s protest made immediately after the South Korea-U S summit

但是,邢海明当天的发言并没有超出韩美首脑会谈以后中国的抗议程度

정부가 “불장난하지 말라”는 중국의 불만에 “한중은 특수관계”로 진화에 나서고 있는 점을 고려해 일단 우리 정부의 행보를 지켜보기로 한 것으로 보인다 Beijing appears to have decided to take a step back and watch Seoul’s actions down the road, considering that Seoul tried to appease Beijing by saying the two countries have “special ties” after Beijing warned not to “play with fire

对于中国称“不要玩火”表示不满,韩国政府以“韩中特殊关系”出面灭火,考虑到这一点,估计中国决定暂时观望韩国政府的动向

특히 한국마저 등을 돌릴 경우 동맹국을 규합한 미국의 중국 견제를 막아내기 힘들다는 판단이 작용한 것으로 알려졌다 ” China reportedly thought that it would be difficult to contain the efforts by the U S to unite its allies against China if South Korea were to turn its back on it

据悉,因为中国认为,如果连同韩国也转向美国,将很难阻止美国纠集盟国对中国进行牵制

싱 대사는 시진핑 주석 방한 계획에 대해서는 “현 상태에서는 확실하게 말할 게 없다”고 했다 As for plans for Chinese President Xi Jinping’s visit to South Korea, Ambassador Xing said there is nothing to say for certain at the moment

邢海明就习近平主席的访韩计划表示:“就目前状况而言,不能明确奉告”

두달만 뛰면 되는 中택한 김연경, 다음 목표는 美 유럽 Kim Yeon-koung chooses to play in China for her next season

选择只打两个月的中国联赛的金软景,下一个目标是美国还是欧洲

‘배구 여제’ 김연경(34·사진)의 중국행은 ‘예측 가능한 미래’였다 Kim Yeon-koung’s return to China was predictable

“排球女帝”金软景(34岁?照片)的中国之行是“可预测的未来”

그러나 그 다음 행선지가 어디가 될지는 여전히 예측 불가능한 상태다 However, it is still unknown where she will go afterward

但她的下一个目的地究竟会是哪里,仍然无法预测

20일 김연경의 에이전시 업무를 맡고 있는 라이언엣에 따르면 김연경은 상하이 연고 중국 리그 팀 광밍유베이와 계약 협상 마무리 단계를 밟고 있다 According to her agent Lianat on Thursday, Kim is in the final process of closing an agreement with the Shanghai Bright Ubest Women's Volleyball Club

20日,据负责金软景的代理业务的Lianat透露,金软景正在与中国联赛球队上海光明优倍队协商合同问题

예상 계약 기간은 한 시즌이다 The contract lasts one season

预计合同期为一个赛季

배구 이적 시장에 밝은 관계자는 “베이징자동차에서도 김연경에게 관심을 보였다 “Beijing BAIC Motor was also interested in Kim

熟悉排球转会市场的相关人士表示:“在北汽俱乐部方面,也对金软景表现出了兴趣

그러나 김연경이 2017∼2018 시즌 몸담았던 친정 팀을 더 선호한 것으로 알고 있다”며 However, it was said that Kim preferred the team that she was with during the 2017-2018 season,” said a source familiar with transfers in volleyball

但据悉,金软景更喜欢2017~2018赛季曾效力过的老东家球队”,

“김연경도 이제 30대 중반으로 중국 리그는 일정이 짧아 체력 부담이 작고 다음 무대를 준비하기가 쉽다”고 말했다 “The shorter Chinese league will be less physically straining and easier to prepare for the next season for Kim who is in her mid-30s

“金软景现在30多岁,中国联赛日程短,体力负担小,准备下一个舞台也很容易”

김연경은 4년 전 중국 리그로 진출할 때도 “경기 수가 많으면 체력적으로 부담스럽다”는 뜻을 밝혔다 ” When she first decided to play in the Chinese league four years ago, Kim mentioned that the higher number of games means more physically challenging

金软景在4年前进军中国联赛时也曾表示,“如果比赛场次多,体力上也会感到负担”

광밍유베이는 지난 시즌 12경기밖에 치르지 않았다 The Shanghai Bright Ubest Women's Volleyball Club only played 12 games last season,

在上个赛季,光明优倍队只进行了12场比赛

반면 김연경은 지난해 제천·MG새마을금고컵 대회부터 V리그 챔피언결정전까지 총 41경기를 뛰었다 while Kim played a total of 41 games last year from Jecheon-Korean Federation of Community Credit Cooperatives Volleyball Cup to V-league Championships

相反,金软景从去年堤川?MG新村金库杯大赛到V联赛冠军争夺战共参加了41场比赛

게다가 이재영-이다영 쌍둥이 자매가 학교 폭력 사태로 전력에서 이탈하면서 심리적 부담까지 안은 채 경기에 나서야 했다 As twin sisters Lee Jae-yeong and Lee Da-yeong dropped out of the team due to scandals about their history of bullying, Kim also had to play with psychological stress

再加上李在英和李多英双胞胎姐妹因学校暴力事件而失去战斗力,在心理负担下不得不参加比赛

아직 2021∼2022시즌 중국 리그 일정은 나오지 않은 상태지만 새 시즌 역시 길어도 두 달 안에 모든 일정이 마무리될 가능성이 높다 （no translation）

虽然2021~2022赛季中国联赛日程尚未出来,但新赛季最长两个月内所有日程都很有可能结束

김연경은 중국 리그 일정을 소화한 뒤 내년 1월 초에 다시 유럽 진출을 시도할 수 있다 （no translation）

金软景很有可能在完成中国联赛日程后,于明年1月初再次进军欧洲

올해 프로 리그가 막을 올린 미국 무대 진출도 가능하다 （no translation）

当然她也有可能进军今年职业联赛拉开帷幕的美国舞台

중국 리그가 끝난 뒤 흥국생명으로 돌아오는 것도 전혀 불가능한 일만은 아니다 （no translation）

中国女排联赛结束后,回到兴国生命也不是不可能的事情

김연경은 해외 리그 팀과 계약할 때는 자유계약선수(FA) 신분이지만 국내에서는 흥국생명에서 다음 시즌 일정의 40%(14경기) 이상을 소화해야 FA 자격을 얻는다 （no translation）

金软景在与海外联赛球队签约时虽然是自由签约选手(FA)身份,但在国内,只有在兴国生命队完成下赛季日程的40%(14场比赛)以上才能获得FA资格

홍콩, 대만공관 일방폐쇄… 교류 끊나 Hong Kong closes Taiwan trade office amid rise in diplomatic tensions

香港单方面关闭驻台湾办事处,中断交流

홍콩이 대만에서 외교 공관 역할을 해 왔던 경제무역문화판사처를 일방적으로 폐쇄했다Hong Kong closed the Hong Kong Economic, Trade and Cultural Office in Taiwan, which served as its representative office

香港单方面关闭了在台湾起到外交公馆作用的经贸文化办事处

이번 결정은 중국과 대만 관계가 악화하고 있는 가운데 나온 것이어서 중국 정부의 영향을 받은 홍콩이 사실상 대만과 교류 중단을 염두에 둔 것 아니냐는 분석도 나온다 The unilateral decision came amid strained ties between China and Taiwan, leading to speculations that Hong Kong is considering suspending ties with Taiwan affected by China’s efforts to diplomatically isolate Taiwan

还有分析说,此次决定是在中国大陆和台湾关系不断恶化的情况下做出的,受到中国政府影响的香港实际上可能考虑的是中断与台湾的交流

19일 홍콩 사우스차이나모닝포스트(SCMP) 등에 따르면 홍콩 정부는 전날 인터넷 홈페이지를 통해 주(駐)대만 경제무역문화판사처 운영을 잠정 중단한다고 밝혔다 According to the South China Morning Post (SCMP) on Wednesday, the government of Hong Kong announced on its website the previous day that it is temporarily suspending operations at its representative office in Taiwan

据香港《南华早报》等19日报道,香港政府前一天在其主页上表示,暂时停止运营驻台湾经济贸易文化办事处,

중단 사유와 운영 재개 시점 등에 대해서는 아무런 설명이 없었다 There was no explanation as to the reason for the suspension and when normal operations will be resumed

但没有对中断的原因和恢复运营的时间等没有作出说明

이에 대해 대만의 대중국 담당 부처인 대륙위원회는 “홍콩 정부가 일방적 결정을 내렸다”며 “깊은 유감을 표명한다”고 밝혔다 “We express deep regret at today’s unilateral decision by the Hong Kong government,” Taiwan’s Mainland Affairs Council said in a statement

对此,台湾负责中国大陆事务的大陆委员会表示:“香港政府单方面做出了决定对此深表遗憾“

주대만 경제무역문화판사처는 홍콩과 대만 사이의 경제·무역 교류를 촉진하기 위해 2011년 문을 열었다 The Hong Kong Economic, Trade and Cultural Office in Taiwan was opened in 2011 to facilitate economic and trade exchanges between Hong Kong and Taiwan

香港驻台湾经济贸易文化办事处旨在促进香港和台湾之间的经贸交流,成立于2011年

대만도 홍콩에 같은 역할을 하는 경제문화판사처를 두고 있다 Taiwan also has similar office in Hong Kong

台湾也在香港设立了起到同样作用的经济文化办事处

이들 기관은 공식적인 정부 기구는 아니지만 교민 보호 등 실질적인 영사관 역할을 수행해 왔다 Although these offices are not official government organizations, they have served as consulates, protecting their overseas citizens

这些机构虽然不是正式的政府机构,但起到了保护侨民等实质性的领事馆作用

홍콩과 대만이 이런 상주 기구를 운영하는 것과 달리 중국과 대만은 상대 지역에 상주 기구가 아예 없다 Unlike Hong Kong and Taiwan, which operate such representative offices, China and Taiwan do not run such offices in each other’s countries

与香港和台湾运营这种常驻机构不同,中国大陆和台湾在对方根本没有常驻机构

홍콩이 중국과 다른 정책을 펼 수 있었던 것은 시진핑(習近平) 중국 국가주석 집권 이전까지는 일국양제(一國兩制·한 국가 두 체제) 원칙이 비교적 잘 지켜졌기 때문이다 Hong Kong was able to establish its own policies because the principle of “one country, two systems” applied to Hong Kong before Xi Jinping came to power in 2013

香港之所以能够采取与中国不同的政策,是因为在中国国家主席习近平执政之前,香港比较遵守“一国两制”原则

하지만 2013년 시 주석 집권 이후 ‘양제’보다 ‘일국’ 원칙이 더 강조되면서 상황이 달라지기 시작했다 The situation began to change after 2013 as Xi Jinping underlined the adherence to the “one China” principle

但是,习近平2013年执政以后,相比“两制”更加强调“一国”原则,因此情况开始发生变化

특히 지난해에는 홍콩 국가보안법이 도입됐고 올해는 홍콩 선거제까지 개편되면서 홍콩의 중국화 현상이 뚜렷해지고 있다 Hong Kong is increasingly affected by China in recent years China passed a new national security law in Hong Kong last year and approved a plan to reform the electoral system of Hong Kong this year

特别是,去年实施了香港《国家安全法》,今年还改革了香港选举制,因此,香港的中国化现象日益明显

‘中, 달 이어 화성도 터치다운…‘우주굴기’ China lands on Mars following its touchdown on moon

中国探测器继月球后着陆火星……向全球展示“太空崛起”

전세계 과시중국이 화성 탐사용 무인이동 로봇(로버)을 화성 표면에 성공적으로 착륙시키면서 미국과 옛 소련에 이어 세 번째로 화성 표면에 탐사선을 착륙시킨 국가가 됐다 The Chinese unmanned rover Zhurong successfully landed on Mars, making China become the third country in the world to do so

中国成功将用于火星探测的无人移动机器人成功着陆到火星表面,成为继美国和前苏联后,第三个在火星表面成功着陆的国家

화성 표면 탐사를 위해 이동하는 로버를 보낸 것은 미국에 이어 두 번째다 Following the United States, China is the second nation across the globe to land a mobile rover on Mars for surface exploration

为探测火星表面而发射可移动的探测器,是继美国之后的第二个

중국 신화통신에 따르면 화성 주변 궤도를 돌던 중국 탐사선 톈원(天問) 1호에서 분리된 화성 탐사 로버 ‘주룽(祝融)’이 15일 오전 8시 18분(한국 시간) 화성 북반구 유토피아 평원 남쪽에 성공적으로 내려앉았다 Separating from the Tianwen-1 mission orbiting Mars, China’s red planet rover Zhurong sat down south of the Utopia Planitia region at 8:18 a m on Saturday (local time), reported Xinhua

据中国新华社报道,中国探测器“天问一号”此前一直在火星周边轨道上盘旋,携火星探测车“祝融”于韩国时间15日上午8时18分在火星北半球的乌托邦平原南侧成功着陆

시진핑 국가주석은 축전을 보내 “중국의 행성 탐사 장정에 중요한 한 걸음”이라며 “지구와 달 사이에서 이제는 행성 간 탐사로의 도약을 이뤄냈다”고 밝혔다 Chinese President Xi Jinping sent a congratulatory telegram, defining the landing as one of the greatest milestones in China’s interplanetary voyage He went on to say that China took a leap forward in interplanetary exploration along with its travel from Earth to the moon

中国国家主席习近平在贺电中表示:“迈出了中国星际探测征程的重要一步,实现了从地月糸到行星际的跨越”

지난해 7월 발사된 톈원 1호는 2월 10일 화성 궤도에 안착한 뒤 3개월 가까이 궤도를 돌며 착륙의 기회를 엿봤다 톈원은 ‘하늘에 묻는다’라는 뜻으로 중국 전국시대 초나라 시인 굴원의 시에서 따왔다 （no translation）

去年7月发射的“天问一号”于2月10日安全进入火星轨道后,在近三个月的时间里一直绕着轨道寻找着陆机会“天问”意为“问天”,取自中国战国时代楚国诗人屈原的诗

주룽은 중국 고대 신화에 나오는 불의 신 이름이다 （no translation）

“祝融”是中国古代神话中火神的名字

이날 착륙에 성공한 주룽은 크기가 가로 2. 6m, 세로 3m, 높이 1. 85m, 무게 240kg인 바퀴 6개짜리 이동형 로봇이다 The Zhurong is a six-wheeled mobile robot apparatus – 2.6 meters in width, 3 meters in length, 1. 85 meters in height and 240 kilograms in weight –

当天成功着陆火星的“祝融”火星车是长2.6米 宽3米 高1.85米 重240公斤 6个轮子的移动机器人

약 90 화성일(1 화성일은 지구로 따지면 24시간 37분) 동안 탐사를 하도록 설계됐다 designed to carry out an exploration mission for at least 90 Mars days or 24 hours and 37 minutes in earth days

其设计是利用约90个火星日(1个火星日按地球计算是24小时37分)时间进行探测

주룽에는 화성 탐사 로버 최초로 지하 100m까지 탐사할 수 있는 레이더 장비가 장착됐다 The rover robot is the first of its kind, which is equipped with a radar system that allows it to explore down to as deep as 100 meters

在“祝融”号探测器上首次安装了可以探测地下100米的雷达设备

중국은 이번 탐사에서 화성 표면에서 물과 얼음의 흔적을 찾고 토양과 암석 성분을 분석할 예정이다 China hopes to discover traces of water or ice and analyze soil and rock components

中国计划在此次探测中,在火星表面寻找水和冰的痕迹,并分析土壤和岩石成分

중국은 두 번의 도전 끝에 화성 탐사에 성공했다 China made it to Mars at the second try

经过两次挑战,中国终于成功实现火星探测

2011년 러시아와 함께 화성 탐사를 시도했지만 실패한 바 있다 Back in 2011, it attempted to explore the red planet in cooperation with Russia but to no avail

中国曾在2011年试图和俄罗斯一起探测火星,但以失败告终

시 주석은 “용감한 도전이 중국을 행성 탐사 분야에서 선진국 반열에 오르게 했다”고 치하했다 （no translation）

习近平称赞道:“你们勇于挑战追求卓越,使中国在行星探测领域进入世界先进国家行列”

중국은 최근 수년 새 화성 탐사와 달 탐사, 독자 우주정거장 건설 추진 등 ‘우주굴기’에 속도를 내며 미국을 위협하는 우주 강국으로 떠올랐다 China has recently sped up with its ambitious space programs on Mars and moon explorations, independent space stations, etc , rising as a rival in aerospace to the Unites States

中国在最近几年里不断加快探测火星和月球 建设独立空间站等“太空崛起”速度,跃升为威胁美国的太空强国

2019년 달 뒷면에 인류 최초로 탐사선 ‘창어 4호’를 착륙시킨 데 이어 지난해 12월 또 다른 탐사선 창어 5호를 달에 보내 월면토를 채취해 지구로 돌아왔다 In 2019, China sent the Chang’e-4 to the back side of the moon for the first time in human history Furthermore, last December, the Chang’e-5 traveled to the moon to collect and bring lunar soil to the Earth

继2019年人类首次在月球背面着陆探测飞船“嫦娥4号”后,去年12月又将另一艘探测飞船“嫦娥5号”发射到月球,采集月面土返回地球

지난달에는 중국 독자 우주정거장 ‘톈허(天和)’를 구성할 첫 구조물을 자국 발사체 ‘창정 5B호’에 실어 우주로 보냈다 （no translation）

上月,中国在本国发射体“长征5B”号上搭载中国独立太空站“天和”的首个构造物飞往太空

2024년 임무를 종료하는 국제우주정거장(ISS)이 폐쇄되면 톈허는 유일한 우주정거장이 된다 （no translation）

如果2024年完成任务的国际空间站(ISS)关闭,“天和”将成为唯一的空间站

2016년엔 미국에 앞서 세계 첫 양자통신위성 ‘모쯔(墨子)호’를 쏘아 올려 2400km 거리에서 무선 양자암호통신을 시현하는 데 성공했다 （no translation）

2016年,中国领先美国率先发射世界第一颗量子通信卫星“墨子”号,在2400公里远的距离上成功实现无线量子密码通信

미국은 중국의 우주 진출을 경계하고 있다 In the meantime, the United States is only throwing a vigilant glance at China’s growing presence in aerospace travel

美国正在警惕中国进入太空

이달 9일 창정 5B호가 통제력을 잃고 지구에 낙하하며 잔해 일부가 인도양에 떨어진 것과 관련해 미국은 “중국이 우주 파편에 관해 책임감 있는 국제기준을 충족하지 못하고 있다”며 빌 넬슨 미국 항공우주국(NASA) 국장 명의의 비판 성명을 내기도 했다 When China’s Changzheng-5 on May 9 lost control to fall back down to the Earth with debris scattered across the Indian Ocean, NASA Administrator Bill Nelson issued a critical statement, saying, “It is clear that China is failing to meet responsible standards regarding their space debris ” Behind tensions between the two nations lies a sense of rivalry in dominance in the field of space travel, analyze global media outlets

本月9日,美国宇航局就“长征5B”号失去控制并坠入地球,导致部分残骸落入印度洋一事表示:“中国没有忠实履行对太空碎片负责任的国际标准”美国宇航局还以局长比尔?尼尔森的名义发表声明对此批评

외신들은 이러한 갈등 이면에 우주 패권을 둔 경쟁의식이 있다고 해설했다中 22t 우주로켓 잔해 주말 지구 낙하 가능성중국의 로켓 ‘창정(長征)5B호’의 잔해가 주말경 지구 대기권에 진입할 것으로 보인다 Debris of China’s 22-ton space rocket could fall onto Earth Debris of China’s space rocket Changzheng (Long March) 5B will likely reenter the earth’s atmosphere around this weekend

外媒解释说,在这样的矛盾背后,存在着争夺太空霸权的竞争意识中国重达22吨的运载火箭残骸有可能周末坠落地球中国“长征5B”火箭的残骸将于周末左右进入地球大气层

한미 군 당국은 추락 예측지에 한반도가 포함되지는 않지만 유사시를 대비해 상호 공조 체계를 강화하기로 했다 The South Korean and U S military authorities said the Korean Peninsula is not included in candidate sites for the rocket’s crash, but they agreed to strengthen cooperation to prepare for an emergency situation

韩美军方决定,虽然预计坠落地点不包括韩半岛,但为应对突发情况,加强相互合作体系

공군 우주정보상황실은 7일 미 우주사령부 연합우주작전센터와 화상회의를 갖고 8, 9일경 지구 대기권에 진입할 것으로 예상되는 로켓 잔해 경로에 대한 감시정보를 지속 공유하기로 했다 The space information and situation room of the South Korean Air Force held a joint video meeting with the Joint Aerospace Operation Center of the U S Space Operations Command on Friday, and agreed to share surveillance information on the trajectory of debris from the rocket, which is expected to reenter the earth’s atmosphere on Saturday or Sunday

韩国空军太空情报状况室与美国太空司令部联合太空作战中心7日举行视频会议,决定将继续共享有关预计在8日或9日前后进入地球大气层的火箭残骸途径的监视信息

창정5B호 크기를 고려하면 대기권에서도 잔해가 소멸되지 않고 추락할 것으로 예측된다 Considering the size of the Changzheng 5B rocket, debris will likely fall onto the earth, rather than disappearing even after entering the atmosphere

韩美认为,考虑到“长征5B”的大小,它不会在大气层中焚烧干净,而是会坠落地球

일단 한미 군 당국은 잔해가 대서양에 추락할 것으로 보고 있으나, 대기권 진입 시 잔해가 본궤도를 이탈할 가능성도 배제하지 않는 것으로 알려졌다 The South Korean and U S military authorities predict the debris will fall into the Atlantic Ocean, but the possibility for the debris to deviate from its original trajectory when reentering the atmosphere cannot reportedly be ruled out

据悉,韩美军方认为残骸将坠落在大西洋,但不排除残骸进入大气层时脱离原轨道的可能性

중국은 지난달 29일 독자 우주정거장 구축을 위해 발사한 핵심 모듈인 ‘톈허(天和)’를 정상궤도에 안착시켰지만, 이를 실어 나른 창정5B호 로켓이 통제 불능 상태에 빠져 22. 5t에 달하는 잔해가 280km 상공에서 매일 1∼2km가량 지구로 낙하하는 중이다 China launched and put into orbit the Tianhe, a core module designed for constructing its own space station, on April 29, but the Changzheng 5B rocket, which transported the Tianhe, went out of control, and the debris weighing 22. 5 tons is falling onto the earth 1 to 2 kilometers daily from an altitude of 280 kilometers

中国为了建设独立的空间站,4月29日发射了核心模块“天和”,但运载火箭“长征5B”却陷入无法控制的状态,22

군 당국은 일단 미 우주사령부의 감시자산을 활용한 잔해 궤도 정보를 공유 받기로 했다 The South Korean military authority plans to share information on the projected trajectory of debris by using the U S

5吨的残骸每天从280公里上空坠落1至2公里左右

이와 함께 잔해가 한반도 상공으로 향하는 최악의 상황을 고려해 전력화를 진행 중인 ‘전자광학위성감시체계’나 ‘고출력레이저위성추적체계’ 등을 활용할 방침인 것으로 알려졌다 Space Operations Command’s surveillance assets In addition, to be prepared for the worst situation wherein debris falls into the skies over the Korean Peninsula, the military is reportedly considering using an electrooptical satellite surveillance system, or a high-performance radar satellite tracking system, which are under development for use in combat mission

韩国军方暂时决定利用美国太空司令部的监视资产共享残骸轨道情报据悉,考虑到残骸向韩半岛上空移动的最坏情况,也将充分利用正在进行战斗力化的“电子光学卫星监视体系”和“高输出激光卫星追踪体系”

블링컨 “中 점점 더 공격적 행동…세계 지배국 되려 해” Blinken remarks China’s “more aggressive” actions

布林肯:“中国采取越来越有攻击性的行动,想成为支配世界的国家”

토니 블링컨 미국 국무장관은 2일(현지 시간) “중국이 점점 더 억압적이고 공격적으로 행동하고 있다”고 비판했다“China is acting more repressively and aggressively,” U S Secretary of State Antony Blinken said on Sunday (local time)“What we've witnessed over the last several years is China acting more repressively at home and more aggressively abroad

美国国务卿安东尼·布林肯当地时间2日抨击中国称:“中国的行动越来越压制和具有攻击性”

미국의 대중국 정책 핵심은 이런 중국에 맞서 규칙에 기반한 국제사회의 질서를 지키는 것이라며 동맹들과의 협력을 재차 강조했다 He repeatedly emphasized cooperation with allies by saying that the key of the U S policy toward China is to uphold the international community’s rules-based order against China

他表示,美国对华政策的核心是对抗中国,维护以规则为基础的国际社会秩序,再次强调了与同盟之间的合作

블링컨 장관은 이날 CBS방송 시사 프로그램 ‘60분’과의 인터뷰에서 “우리는 중국이 국내에서 더 억압적으로, 해외에서도 더 공격적으로 행동하는 것을 봐왔다”고 했다 That is a fact,” said Blinken during an interview with CBS News’ “60 Minutes” on Sunday

布林肯当天在接受CBS电视台时事节目《60分钟》采访时表示:“我们看到中国在国内的行动更加压制,在海外的行动也更为攻击性”

중국은 규칙에 기초를 둔 국제질서에 도전하거나 이를 약화시킬 군사적, 경제적, 외교적 역량을 갖춘 나라라는 것이다 He argued that China is the one country in the world that has the military, economic, diplomatic capacity to undermine or challenge the rules-based order

也就是说,中国是具备挑战或削弱以规则为基础的国际秩序的军事 经济 外交力量的国家

‘중국의 목표가 무엇인 것 같으냐’는 질문에는 “중국은 자신들이 전 세계를 지배하는 국가가 될 수 있고, 돼야 한다고 믿는 것 같다”고 답변했다 “I think that over time, China believes that it can be and should be and will be the dominant country in the world,” he said when asked what the goal of China is

对于“中国的目标像是什么”的提问,他回答说:“中国似乎相信自己能够 必须成为统治全世界的国家”

다만 그는 진행자가 ‘미국이 중국과 군사적 대치 상황으로 나아가고 있느냐’고 묻자 “그런 상황에 도달하거나 그런 방향으로 가는 것조차 미국과 중국 모두의 이익에 심하게 반하는 일”이라고 선을 그었다 However, he drew a line by saying that it is profoundly against the interests of both China and the United States to get to that point or even to head in that direction when the host asked if he thinks the U S is heading towards some sort of military confrontation with China

但当主持人问道“美国是否正在走向同中国的军事对峙状况”时,他划清界限说:“达到这种状况或就算是走向这种局面,都严重违反美国和中国的利益”

블링컨 장관은 “조 바이든 대통령이 지식재산권 탈취를 포함해 중국이 취해 온 행동들에 대해 실제로 우려하고 있다”고도 전했다 “President Biden made clear that we have real concerns about the actions that China has taken, and that includes the theft of intellectual property,” said the secretary of state

布林肯还表示:“美国总统拜登实际上对中国采取的包括夺取知识产权在内的一系列行动感到担忧”

신장위구르족에 대한 중국 당국의 인권침해를 ‘집단학살(genocide)’이라고 부르며 중국의 인권 문제도 직접 겨냥했다 He even directly criticized China’s human rights issues by calling the Chinese authorities’ violation of human rights against the Uyghurs in Xinjiang a genocide

他将中国当局对新疆维吾尔族的人权侵害称为“种族灭绝”,直接将矛头指向中国的人权问题

그는 “우리는 중국을 다루지 않아도 되는 여유가 없다”며 “적대적인 부분이든, 경쟁적이거나 협력적인 부분이든 (중국과의) 관계는 정말로 복잡한 측면들이 있다”고 했다 “Look, we don't have the luxury of not dealing with China There are real complexities to the relationship, whether it's the adversarial piece, whether it's the competitive piece, whether it's the cooperative piece

他表示:“我们没有可以不处理中国问题的充裕。无论是敌对的部分,还是竞争的 合作的部分,(同中国的)关系确实存在复杂的方面”

그는 진행자가 중국의 국내총생산(GDP)이 2028년에 미국을 추월하게 될 것이라는 전망을 언급하자 “부국을 만드는 핵심은 인적 자원, 또 그 인적 자원의 잠재력을 극대화하는 국가의 능력에 달려 있다”며 “그런 점에서 우리는 (중국보다) 훨씬 더 좋은 위치에 있다”고 말했다 ” When the host mentioned that China's gross domestic product is expected to surpass the U S as early as 2028, Blinken said that what really makes the wealth of a nation is human resources and the ability of any one country to maximize its potential “I think we're in a much better place to maximize that (than China)

当主持人提及中国的国内生产总值将于2028年超过美国时,他表示:“打造富国的核心取决于将人力资源和将人力资源潜力极大化的国家能力。从这一点看,我们(比中国)的地位更高”

또 같은 가치관을 공유하는 동맹 및 파트너 국가들과 함께 협력할 것이라는 점도 재확인했다 ” He also reconfirmed that the U S will cooperate with its allies and partners that share the same values

此外,他还重申了将与拥有同样价值观的同盟及伙伴国家共同合作的立场

“신의주역 가림막 철거…” 北中국경 봉쇄 해제 Pyongyang- Beijing border looks set to reopen

“新义州火车站拆除遮布……”朝中边境有解除封锁迹象

징후북-중 교역이 이르면 이달 말 재개될 것으로 알려진 가운데 북한 신의주역에서 열차 가림막이 철거되는 등 북-중 간 국경 봉쇄 해제 징후가 속속 포착되고 있다 Amid reports that North Korea and China will resume trade at the end of April, evidence of opening the border between the two countries, including the removal of a train shield is emerging

据悉,朝鲜与中国的贸易最快将于本月末恢复。在这种情况下,在朝鲜新义州车站正在陆续发现拆除列车遮布等解除朝中边境封锁的迹象

24일 미국의 소리(VOA)에 따르면 위성사진 분석 결과 지난해 10월 북한 신의주역 일대에 설치됐던 열차 가림막이 지난달 31일 돌연 철거됐다 According to Voice of America on Saturday, satellite imagery showed that the tunnel-shaped shield installed in October 2020 near the Sinuiju station in North Korea was removed on March 31,

据“美国之音”广播电台24日透露,卫星照片分析结果显示,去年10月设置在朝鲜新义州车站一带的列车遮挡幕于3月31日突然被拆除

민간 위성사진업체 맥사테크놀로지의 지난달 16일자 위성사진에서는 신의주역에 설치된 약 400m 길이의 터널 형태 가림막이 확인됐다 15 days after the 400-meter cover was captured in satellite imagery released by Maxar Technologies

民间卫星照片企业Maxar科技在3月16日发行的卫星照片中,确认了设置在新义州车站的约400米长的隧道形状遮布

하지만 이달 사진에서는 이 가림막이 사라진 상태였다 가림막은 장기간 세워둔 열차를 눈이나 비 등으로부터 보호하는 목적으로 설치됐던 것으로 추정된다 It is presumed that the facility was built to protect trains from natural elements such as snow and rain while they were not in use

但在本月照片中,该遮布已经消失。据推测,遮布是为了保护长期放置的列车不受雪 雨等影响而设置的。

북한 신의주와 마주하고 있는 북-중 무역 거점 도시인 중국 랴오닝(遼寧)성 단둥(丹東)시와 인근 북-중 접경지 역에는 북한으로 보낼 대규모 물자가 대기 중인 것으로 알려졌다 It is also said that large volumes of goods are waiting in Liaoning and Dandong, which are Chinese trade hubs across Sinuiju, along with other border cities

据悉,与朝鲜新义州相对的朝中贸易据点城市——中国辽宁省丹东市和附近的朝中接壤地区即将发往朝鲜的大规模物资正在等待中

단둥시 기차역에선 평양 외곽 지역인 ‘서포’ 지명이 적힌 화물열차가 물자를 실은 채 세워져 있는 모습이 목격됐다 Freight trains marked with “Sopo,” a city on the outskirts of Pyongyang, were seen at a train station in Dandong

在丹东市火车站,有人看到停靠着一辆写有平壤郊区地名“西浦” 装有物资的货物列车

선박을 통한 북-중 간 교역은 지난달 재개됐다 Trade by ship between North Korea and China were resumed last month

通过船舶进行的朝中贸易已于3月重启

중국 해관총서에 따르면 지난달 북한은 중국으로부터 비료 등 1297만 달러 상당의 물자를 수입했다 Pyongyang imported 12. 97 million dollars’ worth goods including fertilizer in March, according to the Chinese General Administration of Customs

据中国海关总署统计,朝鲜3月从中国进口了化肥等价值1297万美元的物资

북-중 관계에 밝은 대북 소식통은 25일 “지난달 중국에서 북한으로 들어간 물자 대부분은 선박을 통해 들어갔다”고 말했다 “ Most imports from China were shipped to North Korea last month,” said a source familiar with the relations between the two countries

熟悉朝中关系的消息人士25日表示:“3月从中国进入朝鲜的大部分物资都是通过船只运进的”

이르면 27일부터 대북 지원용 비료를 실은 열차가 신의주로 들어갈 것으로 알려졌다 It is said that trains carrying fertilizer will head for the North starting Tuesday at the earliest

据悉,最快从27日开始,装载援朝化肥的列车将开往新义州

美 ‘대북제재 완전한 이행’ 밝힌 날, 中은 한국에 ‘제재 완화 노력’ China urges S Korea to make efforts to alleviate N Korea sanctions

美国表示“完全履行联合国对朝制裁”的当天,中国敦促“韩国努力放宽制裁”

촉구한미일 3국 안보실장들이 유엔 안전보장이사회 대북 제재 결의의 완전한 이행”을 강조했다고 미국이 밝힌 날 중국은 한국에 “북한의 합리적 안보 우려 해결을 위해 노력해야 한다”고 요구했다 Security advisors of South Korea, Japan and the U S highlighted the “imperative for full implementation” of relevant UN Security Council sanctions resolutions against Pyongyang

美国表示韩美日三国安保室室长强调“完全履行联合国安理会对朝鲜制裁决议”的当天,中国要求韩国“为解决朝鲜合理的安全关切而努力”

중국은 ‘북한의 합리적 안보 우려’를 북한 체제 보장과 대북 제재 완화 등을 가리키는 말로 써 왔다 On the same day, China demanded South Korea to make efforts to resolve reasonable security concerns of North Korea

中国一直将“朝鲜合理的安全忧虑”用在保障朝鲜体制和放宽对朝制裁等方面

미 백악관은 2일(현지 시간) 메릴랜드 아나폴리스 해군사관학교에서 열린 한미일 3국 안보실장 회의 뒤 낸 공동 언론성명에서 “3국 안보실장들은 북한을 비롯해 국제사회가 유엔 안보리 결의를 완전히 이행하는 것이 긴요하다는 데 동의했다”며 “핵 확산 방지와 한반도에서 (대북) 억지 강화 및 평화·안정 유지를 위해 협력해 나가기로 했다”고 강조했다 “북한의 핵 및 탄도미사일 프로그램에 대한 우려를 공유하고, 비핵화를 위한 한미일 3자 간 조율된 협력을 통해 이 문제들을 해결하려는 의지를 재확인했다”고도 밝혔다 China has been using the term “reasonable security concerns” to refer to guarantee the continuity of the North Korean regime and alleviate sanctions against the rogue state “They agreed on the imperative for full implementation of relevant UN Security Council resolutions by the international community, including North Korea, preventing proliferation, and cooperating to strengthen deterrence and maintain peace and stability on the Korean Peninsula,” said the White House in a joint statement after having trilateral talks at the U S Naval Academy in Maryland on Friday (local time) “National security advisors shared their concerns about North Korea’s nuclear and ballistic missile programs and reaffirmed their commitment to address and resolve these issues through concerted trilateral cooperation towards denuclearization ”

美国白宫当地时间2日在马里兰州安纳波利斯海军士官学校举行的韩美日三国安保室长会议结束后发表的联合媒体声明中强调:“三国安保室长一致认为,朝鲜等国际社会完全履行联合国安理会决议非常重要二国将为防止核扩散和在韩半岛加强(对朝)威慑并维护和平与稳定而继续合作”声明还表示:“三方就朝鲜核及弹道导弹计划的担忧达成了共识,同时重申了为实现无核化而通过韩美日三方协调合作解决这些问题的意志”

대북 억지를 강조하면서 북한과 중국에 대북 제재 준수를 압박한 것 The joint statement highlighted deterrence while pressing North Korea and China to observe the sanctions

即,在强调对朝遏制的同时,向朝鲜和中国施压,要求遵守对朝制裁

서훈 청와대 국가안보실장은 회의 뒤 기자들에게 “한미일은 북핵 문제의 시급성과 외교적 해결의 필요성에 대해 공감했고 북-미 협상의 조기 재개를 위한 노력이 계속돼야 한다는 데 뜻을 같이했다”고 했다 “South Korea, Japan and the U S agreed on the urgency and diplomatic necessity to resolve the North Korean nuclear issues and that our three countries should continue to make efforts for early resumption of the U S -North Korea talks,” said National Security Office Director Suh Hoon

青瓦台国家安全室室长徐薰在会议结束后接受记者采访时表示:“韩美日就朝核问题的紧迫性和外交解决的必要性达成了共识,一致认为应该继续努力使朝美谈判早日重启”

하지만 한미일 3국 조율을 통해 백악관이 밝힌 성명에 북-미 협상의 조속한 재개는 포함되지 않았다 But the resumption of talks was not included in the statement published by the White House

但通过韩美日三国协调,白宫发表的声明中并没有包括尽快重启朝美协商

반면 왕이(王毅) 중국 외교부장은 3일 중국 푸젠(福建)성 샤먼(廈門)에서 열린 한중 외교장관 회담에서 정의용 외교부 장관에게 “한반도 평화와 안정을 수호하고 북한의 합리적 안보 우려를 확실히 해결해야 한다”며 “각 측이 이를 위해 적극적으로 노력해야 한다”고 했다 “All related parties should actively try to maintain peace and stability on the Korean Peninsula and effectively resolve the concerns of North Korea,” said Chinese Foreign Minister Wang Yi in talks with his South Korean counterpart Chung Eui-yong on Saturday in Xiamen, China

另一方面,中国外长王毅3日在中国福建省厦门举行的韩中外长会谈上向韩国外长郑义溶表示:“一定要维护韩半岛和平与稳定,切实解决朝鲜合理安全关切，各方应为此作出积极努力”

왕 부장은 또 “중국은 한국과 5세대(5G) 이동통신, 반도체 집적회로 등 분야의 협력을 중점적으로 강화하고 질 높은 협력 파트너가 되기를 원한다”고도 했다 （no translation）

王毅还表示:“中国希望与韩国在5G移动通信 半导体集成电路等领域加强合作,成为高质量的合作伙伴”

반도체를 중국 견제를 위한 국가안보 이슈로 다루기 시작한 미국은 한미일 안보실장 회의에서 3국의 반도체 공급망 유지를 주요하게 논의한 것으로 알려졌다 （no translation）

据悉,把半导体作为牵制中国的国家安保焦点问题开始讨论半导体问题的美国,在韩美日安保室长会议上主要讨论了维持3国半导体供应链的问题

현대건설기계, 中진출 최대 2500억원 수주현대건설기계가 중국에서 역대 최대 규모의 수주를 했다 HCE wins orders worth of 250 billion won in China Hyundai Construction Equipment (HCE) has won its largest-ever orders in China

2500亿韩元现代建设机械承揽公司史上在华最大订单现代建设机械在中国承揽到了历届最大规模的订单

현대건설기계는 최근 중국 고객사들로부터 건설장비 2200여 대를 수주했다고 29일 밝혔다 현대건설기계가 중국에서 사업을 벌인 이후 가장 큰 규모의 수주다 HCE said on Monday that it has achieved orders of supplying 2,200 units of construction equipment to Chinese customers, the biggest ones since the company’s entry into the Chinese market

现代建设机械29日表示,最近从中国顾客公司接到了2200多台建设装备订单。这是现代建设机械在中国开展业务以来规模最大的订单

5.5∼85t급 굴착기와 휠로더 등으로 금액은 2500억 원에 달한다 지난해 현대건설기계의 중국 수주 규모가 7800여 대였던 걸 감안하면 지난해 연간 실적의 30%에 달한다 The orders, which are worth 250 billion won, include 5. 5- to 85-ton excavators and wheel loaders and account for approximately 30 percent of the company’s sales last year, considering that it received orders for 7,800 units of construction equipment in China

5.5~85吨级的挖掘机和轮式装载机等,合同金额达2500亿韩元，考虑到去年现代建设机械在中国的订单规模为7800多台,此次订单达到了去年整年业绩的30%

현대건설기계는 이번에 수주한 장비들을 5월까지 공급할 예정이다 The company plans to complete the delivery of the equipment by May

现代建设机械计划到5月为止供应此次承揽的装备

최근 중국 시장은 지난해 신종 코로나바이러스 감염증(코로나19)으로 인한 경기침체를 극복하기 위해 중국 정부 차원의 대대적인 사회간접자본(SOC) 투자가 이뤄지고 있다 The Chinese government is recently making great investments in social overhead capital (SOC) in order to cope with the economic downturn caused by the COVID-19 pandemic

最近中国市场为了克服去年因新型冠状病毒肺炎(COVID-19)疫情而导致的经济停滞,中国政府层面正在大规模进行社会间接资本(SOC)投资

현대건설기계는 중국 건설장비 시장이 호황기에 들어선 걸로 판단하고 이달 초 대형 굴착기, 수륙양용 굴착기, 파일해머, 니퍼 등 신제품 13종을 중국 시장에 선보였다 HCE introduced 13 new products, including large excavators, amphibious excavators, pile hammers, and nippers to the Chinese market early this month, thinking that the Chinese construction equipment market has entered a boom cycle

现代建设机械认为中国建设装备市场进入繁荣期,本月初在中国市场推出了大型挖掘机 水陆两用挖掘机 打桩机 钳子等13种新产品

철광석 가격이 1t당 170달러(약 20만 원)에 육박하는 등 원자재 가격이 강세를 띠면서 중국을 비롯한 신흥시장에서의 건설장비 수요 증가도 기대된다 An increase in demand for construction equipment is expected in emerging markets, including China, as the prices of raw materials are rapidly rising, with the price of iron ore approaching $170 per ton

随着铁矿石价格逼近每吨170美元(约20万韩元)等原材料价格走强,期待包括中国在内的新兴市场对建设装备的需求增加

현대건설기계는 올해 중국의 건설장비 수요가 지난해보다 8% 늘어난 31만5000여 대에 이를 것으로 보고 있다 HCE expects that this year’s demand for construction equipment in China will rise 8 percent year-on-year to 315,000 units

现代建设机械预测,今年中国的建设装备需求将比去年增加8%,达到31.5万多台

중국, 자국 백신 접종 외국인 비자 발급 간소화 하기로중국 정부가 중국산 코로나19 백신을 맞은 뒤 홍콩을 통해 입국하는 외국인에 대해 핵산검사 증명서 제출을 면제하는 등 비자 발급 절차를 간소화하기로 했다 China simplifies visa requirements for foreigners inoculated with Chinese vaccine The Chinese government has decided to simplify visa requirements for foreigners who enter the Chinese mainland via Hong Kong if they receive COVID-19 vaccines produced by China such as by exempting them from providing a negative PCR test result

中国决定对接种本国疫苗的外国人简化签证发放程序中国政府决定对接种中国产新冠疫苗后通过香港入境的外国人简化发放签证的程序,可免除提交核酸检查证明书等

14일 중국 관영 글로벌타임스에 따르면 중국 외교부 홍콩 주재 사무소는 12일 홈페이지를 통해 중국 백신을 2회 접종하거나 비자 신청 14일 전에 1회 맞은 사람은 중국 비자를 신청할 때 별도의 핵산검사 증명서와 건강 및 여행기록 증명서를 제출하지 않아도 된다고 밝혔다 According to the Chinese state-run media Global Times on Sunday, the Office of the Commissioner of the Chinese Ministry of Foreign Affairs in Hong Kong announced a new policy on its website on Friday where a negative PCR test result as well as health and travel certificates will not be required for any visa applicants who got two doses of the China-made vaccine or who got their first jab 14 days prior to the application

据中国官方媒体《环球时报》14日报道,中国外交部驻香港特派员公署12日在官方网站上表示,两次接种中国疫苗或申请签证14天前接种一次的人在申请中国签证时,可以不提交核酸检查证明书和健康及旅行记录证明书

중국은 현재 외국인이 중국에 입국할 때 72시간 이내 코로나19 핵산검사 음성 증명서 등을 제출하도록 하고 있다 Under the current policy, foreigners are required to provide a negative PCR test result that was issued within 72 hours before their arrival

目前,中国规定,外国人进入中国时,须提交72小时内新冠核酸检查阴性证明等

장례식 참석이나 친척 방문 등 인도적 목적으로 비자를 신청하는 경우도 중국산 백신을 맞았다면 간소화 대상으로 분류해 비자를 발급하기로 했다 The simplified application process will also be applied to those who apply for a visa for reasons such as visiting relatives or attending a funeral

对于以参加葬礼或探亲等人道主义目的申请签证的情况,如已接种中国产疫苗,就将其分类为简化手续对象并发放签证

이 조치는 15일부터 시행된다 The new process will be put in place on Monday

该措施将从15日开始执行

글로벌타임스는 “중국산 백신 접종자에 대해 핵산검사 음성 증명서 제출을 생략하도록 한 것은 중국산 백신에 대한 자신감을 보여주는 것”이라고 설명했지만 중국산 백신 접종자에 한해서만 비자 발급을 간소화하는 이번 조치는 중국산 백신의 보급을 확대하기 위한 것이란 분석이 많다 “China has simplified the visa policy for foreigners applying to enter the Chinese mainland via Hong Kong who have received China-produced COVID-19 vaccines, which experts said shows the authorities' confidence in the efficacy and safety of the vaccines,” said Global Times

《环球时报》解释说:“让接种中国疫苗的人省去提交核酸检查阴性证明的做法表现出对中国产疫苗的信心”但很多人分析说,这一措施仅针对中国疫苗接种者简化签证发放程序,目的是为了扩大中国产疫苗的普及

중국 입국을 위한 수요가 상당하다는 점에서 이들의 중국산 백신 접종을 늘리기 위한 조치라는 것이다 Many experts say, however, that streamlining visas is aimed at providing its vaccines more widely, considering that there are many people who wish to enter the Chinese mainland

也就是说,从为入境中国的相当数量的需求来看,这是为了增加这些人在中国的疫苗接种数量而采取的措施

다만 중국 내에서도 중국산 백신에 대한 불신으로 백신 접종 인구가 2월 말 현재 전체 인구의 3% 안팎에 그쳐 이번 조치의 실효성에 의문을 제기하는 시각도 있다 Meanwhile, only three percent of the Chinese population were inoculated as of late February due to a lack of public confidence in the vaccine, raising doubts about the impact of the new policy

但是,由于中国国内对中国产疫苗的不信任,疫苗接种人口在2月底仅占总人口的3%左右,因此有人对此举的实效性提出了疑问

바이든 정부 첫 안보지침 “동맹과 단합해 중에 대응”조 바이든 미국 행정부가 3일(현지 시간) 중국을 집중 겨냥하는 내용이 담긴 ‘잠정적 안보전략 지침’을 발표했다 Biden administration plans to respond to China by working with allies The Biden administration announced Wednesday (local time) a tentative security strategy that targets China

拜登政府推出第一个安保方针,“团结同盟应对中国”美国拜登政府当地时间3日公布了包含集中针对中国的“国家安保战略中间方针”

바이든 행정부 취임 이후 40여 일 만에 나온 것으로 향후 미국의 외교안보 정책의 방향과 청사진을 담고 있다 It came out 40 days after the new president took office and holds the direction and blueprints of diplomacy and security policies of the U S

拜登政府就任40多天后公布这一方针,包含着今后美国外交安保政策的方向和蓝图

앤서니 블링컨 국무장관은 이날 백악관의 지침 발표에 앞서 “중국은 21세기의 가장 큰 지정학적 시험”이라며 “중국은 안정적이고 개방된 국제시스템에 도전할 경제적, 외교적, 군사적, 기술적 힘을 가진 유일한 국가”라고 했다 U S Secretary of State Antony Blinken said China represented America’s “biggest geopolitical test of the 21st century

美国国务卿安东尼·布林肯当天在白宫发表指针之前表示:“中国是21世纪最大的地政学考验

또 “중국은 우리가 원하는 방식으로 전 세계가 작동하도록 하는 모든 규범과 가치에 도전이 되고 있다”고 밝혔다 ” He also said, “China is the only country with the economic, diplomatic, military and technological power to seriously challenge the stable and open international system— all the rules, values and relationships that make the world work the way we want it to

中国是唯一拥有挑战稳定 开放的国际体系的经济 外交 军事 技术力量的国家”他还表示:“中国正在向以我们希望的方式让全世界运转的所有规范和价值发起挑战”

블링컨 장관은 “우리가 빠져나간 자리를 중국이 채웠다”며 동맹 및 파트너 국가들과의 협력 강화로 이에 대응하겠다고 강조했다 ” 　 “Where we have pulled back, China has filled in,” he said and emphasized that he would respond to it by working with allies and partners

布林肯表示:“我们离开的地方,已被中国占据”

동맹들을 ‘힘을 배가시키는 미국의 독특한 자산’이라고 부른 뒤 “우리의 단합된 무게는 중국이 이를 무시하지 못하게 만든다”고 역설했다 “Our alliances are what the military calls force multipliers They’re our unique asset,” he said “Our combined weight is much harder for China to ignore

他强调,将通过加强同同盟及伙伴国家的合作予以应对他将同盟称为“使力量倍增的美国独特的资产”,并强调,“我们团结一心的力量让中国无法无视”

시진핑 “회색코뿔소 - 블랙스완에 대비하라” 黨에 지시시진핑(習近平) 중국 국가주석이 ‘회색코뿔소’와 ‘블랙스완’을 언급하며 중국이 직면한 위험을 강조했다 ” Xi says China should prepare for crisis Chinese President Xi Jinping has emphasized jeopardies faced by China by mentioning “white rhino” and “black swan

习近平在党内指示:“做好应对灰犀牛与黑天鹅的预案”中国国家主席习近平提及“灰犀牛”和“黑天鹅”,强调中国面临的风险

중국 관영 중국중앙(CC)TV에 따르면 시 주석은 지난달 28일 공산당 중앙정치국 집단학습에서 “각종 위험과 도전을 잘 예측해야 하며 각종 회색코뿔소와 블랙스완 사건에 잘 대비해야 한다”고 말했다 ” According to China's state-run channel CCTV, President Xi attended a group training for the Chinese Community Party’s central politburo on Thursday where he said, “China should properly forecast various jeopardies and challenges, and should be well prepared for white rhino and black swan incidents

据中国中央电视台报道,习近平1月28日在中共中央政治局集体学习时表示:“要善于预见和预判各种风险挑战,做好应对各种‘灰犀牛’和‘黑天鹅’事件的预案”

회색코뿔소는 예상할 수 있지만 간과하기 쉬운 위험을, 블랙스완은 발생할 확률은 낮지만 한 번 일어나면 큰 충격을 주는 위험을 의미한다 ” The white rhino refers to a jeopardy that can be predicted but can be easily overlooked, while the black swan is a risk that has a slim chance of occurring but causes massive damage once it occurs

“灰犀牛”是可以预料到 但容易忽视的危险,“黑天鹅”是指虽然发生概率较低 但一旦发生就会带来巨大冲击的危险

시 주석은 또 “세계적으로 100년간 전례 없는 대변화가 일어나는 가운데 복잡한 국제 정세의 영향을 깊이 인식하고 중국의 발전에 유리한 환경을 조성해야 한다”고 주문했다 As a massive transformation that is unprecedented over the past 100 years is now happening around the world, China should recognize and deeply understand complex international situations and create an environment favorable to China’s development, the Chinese president added

习近平还表示:“面对世界百年未有之大变局,要深刻认识错综复杂的国际局势的影响,为中国的发展创造良好环境”

이 같은 발언은 미국 조 바이든 행정부의 출범 이후에도 양국 대결 구도는 계속될 것이라는 전망이 제기되는 가운데 나왔다 Xi made the remarks as experts predicted that the U S will not change the stance of bilateral rivalry even after the inauguration of the Joe Biden administration

这一讲话的背景是,有预测认为,美国拜登政府上台以后,两国对峙格局还会持续下去

도널드 트럼프 행정부 때 악화된 미중 갈등은 현재 무역을 넘어 외교·안보 분야까지 확대된 상태다 U S -China conflicts that were aggravated during the Donald Trump administration has now expanded from trade to diplomacy and national security

在特朗普执政时期恶化的中美矛盾目前已经超越贸易领域,扩大到外交 安全领域

지난달 28일 미국의 유력 정치전문 매체 폴리티코에는 “중국 공산당 지도부의 균열을 통해 시 주석 교체를 도모해야 한다”는 내용의 전직 미국 고위당국자 익명 기고문까지 실렸다 （no translation）

1月28日,美国权威政治专门媒体《政客》甚至刊登了一篇匿名投稿,内容为“应该通过中国共产党领导层的分裂,来谋求更换习主席”

시 주석은 위기를 강조할 때마다 회색코뿔소와 블랙스완을 언급하고 있다 Xi has been mentioning white rhino and black swan whenever emphasizing a crisis

习近平每次强调危机时都会提及“灰犀牛”和“黑天鹅”

신종 코로나바이러스 감염증(코로나19)이 확산하던 지난해 2월엔 인도네시아 대통령, 말레이시아 총리 등과 전화통화를 하면서 “방역에 있어 회색코뿔소나 블랙스완을 어떻게 막을 수 있을지는 전 세계의 고민”이라고 했다 When the Covid-19 pandemic was spreading wildly in February last year, the Chinese president said that the entire world is agonizing how to prevent white rhino and black swan in the area of quarantine” in his calls with the Indonesian president and the Malaysian prime minister

新冠疫情扩散的去年2月,他与印度尼西亚总统 马来西亚总理等人通电话时表示:“在防疫方面,如何预防‘灰犀牛’和‘黑天鹅’,是全世界的难题”

또 중국이 28년 만에 가장 낮은 경제성장률을 발표하던 2019년 1월에도 “국제 정세가 예측하기 어렵고 주변 환경은 복잡하고 민감하다 When China was announcing the lowest economic growth rate in 28 years in January 2019, Xi said, “International situations are difficult to predict and surrounding environment is complex and delicate

此外,中国公布28年来最低经济增长率的2019年1月也曾表示:“国际形势难以预测,周边环境复杂敏感

블랙스완을 고도로 경계하고 회색코뿔소도 예방해야 한다”고 강조했다 We have to be highly vigilant against black swan and prevent white rhino

应该高度警惕‘黑天鹅’,预防‘灰犀牛’”

시 주석은 특히 회색코뿔소나 블랙스완 같은 위험을 막는 일을 전쟁에 비유하기도 했다 ” He even compared preventing jeopardies such as white rhino or black swan to a war

习近平还把防范“灰犀牛”和“黑天鹅”等危险比喻成战争

시 주석은 이번 정치국 학습에서 올해는 14차 5개년 계획의 첫해이자 공산당 창당 100년을 맞는 해로 경제사회 발전을 보장하는 것이 매우 중요하다고도 했다 （no translation）

习近平在这次政治局学习中还说:“今年是十四五规划开局之年,也是共产党建党100周年,保障经济社会发展非常重要”

시진핑 ‘파카’ 입었더니… Anta Group’s shares get boost after Xi Jinping showcases parka

习近平穿上羽绒服亮相,相关服装公司股价猛涨10%

관련 의류회사 주가 10% 껑충시진핑(習近平) 중국 국가주석이 18일 2022 베이징 겨울올림픽 공식 후원사인 안타(安踏)그룹 산하기업의 방수코트(파카)를 입고 등장하자 하루 뒤 홍콩 증시에 상장된 안타그룹 주가가 약 10% 상승했다 After Chinese President Xi Jinping made appearance clad in a parka from Anta Group on Monday, the company’s stock listed on the Hong Kong stock market jumped about 10 percent on Tuesday

中国国家主席习近平18日身穿2022北京冬奥会官方赞助商安踏集团下属企业的防水羽绒服登场,一天后,在香港股市上市的安踏集团股价上涨了约10%

홍콩 사우스차이나모닝포스트(SCMP)에 따르면 시 주석은 18일 올림픽 준비 상황을 점검하기 위해 베이징 외곽의 겨울올림픽 시설을 방문하면서 짙은 푸른색의 ‘아크테릭스’ 파카를 입었다 According to the South China Morning Post, President Xi was seen wearing a cobalt blue Arc’teryx parka when visiting facilities for the Winter Olympics out the outskirts of Beijing on the day to inspect preparation for the event

据香港《南华早报》报道,习近平18日访问北京郊区的冬奥会设施时,身穿深蓝色的“始祖鸟(Arcteryx)”大衣

원래 핀란드기업 아메르스포츠에 속한 브랜드였는데 2019년 ‘중국의 나이키’로 불리는 안타그룹에 인수됐다 The brand originally belonged to Amer Sports of Finland, but was taken over in 2019 by Anta Group, an official sponsor of the 2022 Winter Olympic Games in Beijing

始祖鸟原来是属于芬兰企业AmerSports的品牌,2019年被被称为“中国耐克”的安踏集团收购

아크테릭스 제품은 온라인 매장에서 보통 700∼2000달러(약 80만∼220만 원)에 판매된다 The Arc’teryx parka product generally costs 700 U S dollars to 2,000 dollars in online shops

始祖鸟产品在网络卖场中通常以700～2000美元(约80万～220万韩元)的价格销售

시 주석이 입은 제품의 정확한 가격은 알려지지 않았다 The exact price of Xi’s parka remained unknown

习主席所穿产品的具体价格尚不清楚

안타는 중국 스포츠용품 시장에서 미국 나이키(시장 점유율 23%), 독일 아디다스(20%)에 이어 점유율 15%로 3위를 차지하고 있다 Anta has 15 percent of the Chinese sports gear market share to rank third overall after Nike (23 percent share) and Adidas (20 percent)

安踏在中国体育用品市场上,继美国耐克(市场占有率23%) 德国阿迪达斯(20%)之后,以15%的占有率位居第三

시 주석은 2017년에도 안타 재킷을 입고 등장했다 Xi appeared wearing an Anta jacket in 2017 as well

习主席在2017年也穿着安踏夹克登场

2018년 평창 겨울올림픽에 등장한 중국 대표팀도 안타 제품을 유니폼으로 착용했다 The Chinese delegation that participated in the 2018 Winter Olympics in Pyeongchang, South Korea, also used Anta products as its uniforms

在2018年平昌冬奥会上登场的中国国家队也把安踏产品作为队服穿着

시 주석이 자국 의류회사를 지원하기 위해 일부러 이 모습을 공개했다는 분석이 제기된다 Analysts say Xi intentionally made such appearance to promote a Chinese apparel maker

有分析认为,习主席为了支援本国服装公司,故意公开了这一形象

실제 19일 안타그룹 주가는 10% 가까이 상승했다 In fact, Anta Group’s share jumped nearly 10 percent on Tuesday

事实上,19日安踏集团的股价上涨了近10%

미국은 왜 중국의 일대일로 경계하나 ‘아시아인들은 생각할 수 있는가(Can Asians Think) Why does US counter China’s initiative

美国为什么警惕中国的“一带一路”

’ 이 도발적인 문장은 1993년 키쇼어 마부바니가 쓴 책의 제목이다 “Can Asians Think ” is the provocative title of a book written by Kishore Mahbubani in 1993

“亚洲人会思考吗(CanAsiansThink)”这一挑衅性的句子是1993年基绍尔·马夫巴尼写的书名

싱가포르 리콴유공공정책대학원장인 마부바니는 당시 책을 통해 세계의 흐름이 변하고 있으며, 아시아가 서양에 가르칠 것이 더 많다고 경고했다 In the book, Mahbubani, dean of the Lee Kuan Yew School of Public Policy at National University of Singapore, warned that the tide was changing and Asia had more to teach the West

新加坡李光耀公共政策研究生院院长马夫巴尼当时通过此书警告说:“世界趋势正在发生变化,亚洲教给西方的东西更多”

아시아인이 생각할 수 있느냐는 식민주의적 사고가 아닌, 아시아인이 어떤 생각을 하는지를 연구할 시점이라면서 말이다 The author underlined that it was time to break away from the colonialist thinking, “Can Asians think ” but to study what Asians think

亚洲人会不会思考这并不是殖民主义思维,而是指现在应该研究亚洲人有什么想法

20여 년이 지나 달라진 아시아의 위상은 경제 수치로도 입증되고 있다 Twenty years later, Asia’s elevated status is demonstrated by economic figures

20多年间亚洲地位的变化,正在经济数据中得到证明

지난해 11월 한중일과 호주 등 15개국이 서명한 ‘역내포괄적경제동반자협정(RCEP)’은 인구 22억 명, 국내총생산(GDP) 규모 총 26조2000억 달러로 ‘세계 최대의 자유무역협정(FTA)’이라고 불렸다 The Regional Comprehensive Economic Partnership (RCEP) signed by 15 countries, including South Korea, China, Japan, and Australia in November last year was called the biggest free trade agreement (FTA) in the world since the combined population and gross domestic product (GDP) of the member countries totaled 2. 2 billion and 26. 2 trillion dollars, respectively

去年11月,韩 中 日 澳等15个国家签署的《区域全面经济伙伴协定(RCEP)》拥有22亿人口,国内生产总值(GDP)总规模达26.2万亿美元,被称为“世界上最大的自由贸易协定”

저자는 앞으로 세계 지형도는 ‘아메리카 퍼스트’가 아닌 ‘아시아 퍼스트’가 될 것이라 단언한다 In his new book, Mahbubani affirms that the global landscape will be rewritten from “America First” to “Asia First,

作者断言,今后世界地形图将不再是“美国第一”,而是“亚洲第一”

그러면서 잘 알려지지 않은 아시아의 여러 단면을 자세히 소개한다 ” introducing unknown sides of Asia

书中同时详细介绍了鲜为人知的亚洲各方面

첫 출발은 고대 아시아 문명이다 The author begins with the ancient Asian civilization

第一步是古代亚洲文明

그리스 문명 등 서구 중심으로 쓰인 세계사의 그늘에 가려진 인도와 서아시아, 동아시아 문명을 간략하게 정리한다 He gives a brief summary of the civilizations of India, West Asia, and East Asia, which were hidden in the shadow of the world history that focused on the West, such as Ancient Greek civilization

该书简略地整理了被以希腊文明等以西方为中心写就的世界史阴影笼罩的印度 西亚 东亚文明

그 뒤 아시아의 주요 정치, 경제, 사회, 문화에 관한 기본적인 지식과 사건을 제시한다 He then introduces the basic knowledge and events on major political, economic, social, and cultural aspects of Asia

随后,该书提示了亚洲主要政治 经济 社会 文化相关的基本知识和事件

호주와 러시아는 왜 일찍부터 ‘아시아화’에 뛰어들었는지, 미국은 왜 중국의 일대일로 프로젝트를 경계하는지 등 시의성 있는 주제도 다룬다 The author also deals with topics of the time, such as why Australia and Russia began Asianization early on and why the U S wants to counter China’s “One Belt One Road” initiative

该书还涉及了澳大利亚和俄罗斯为什么很早就开始“亚洲化” 美国为什么对中国的“一带一路”项目保持警惕等具有时宜性的主题

‘아시아의 관점에서 지난 20년은 조지 W 부시의 무능력, 버락 오바마의 무성의, 도널드 트럼프의 예측 불가능성의 시대’라거나, ‘서양의 오해와 달리 아시아는 중국 중심을 향해 움직이고 있지 않다’는 관점이 흥미롭다 It is interesting that the past 20 years, from the perspective of Asia, has been the era of incompetence by George W Bush, the era of insincerity by Barack Obama, and the era of uncertainty by Donald Trump, and that, contrary to popular belief in the West, Asia is not centered around China

“从亚洲的观点来看,过去20年是乔治·W·布什的无能力 贝拉克·奥巴马的无诚意 唐纳德·特朗普未能预测的时代”以及“与西方的误解不同,亚洲并不是向着以中国为中心移动”的观点非常有趣

동아시아를 넘어 인도와 동남아시아 등 광활한 아시아의 현주소를 훑어볼 수 있다는 것도 장점이다 While helping readers to look at the current status of entire Asia, including India and Southeast Asia,

超出东亚,还可以一览印度和东南亚等广阔的亚洲现状,是该书的又一优点

다만 아시아에 무지한 서구인을 독자로 설정하고 있다는 한계도 느껴진다 the book has its limits as it sets Westerners, who are ignorant of Asia, as its readers

但同时也让人感受到该书把对亚洲无知的西方人设定为读者的局限性

인도에서 태어난 저자는 미국과 유럽에서 국제 관계 전문가로 활동하고 현재는 싱가포르에 정착했다 Born in India, the author works as an international relations expert in the U S and Europe and is currently settled in Singapore

作者出生于印度,在美国和欧洲担任国际关系专家,目前定居在新加坡

원제는 ‘The Future is Asian’ The original title of the book is “The Future is Asian

英文书名为《TheFutureisAsian(未来是亚洲的)》

“달 표면 샘플 44년만에 가져왔다” 中들썩 “14억 중국 국민 전체가 들썩이고 있다 ” China’s Chang’e-5 mission returns with Moon samples

“时隔44年带回月球表面样本”,中国一片欢腾“全体14亿中国国民正在一片欢腾”

” 중국 무인 달 탐사선 ‘창어(嫦娥) 5호’가 달 표면 샘플을 채취해 17일 새벽 무사히 귀환했다 China’s “Chang’e-5” mission returned to Earth with Moon samples early on Thursday morning

中国无人月球探测器“嫦娥五号”采取月球表面样本后于17日凌晨安全返回

달 샘플 채취는 1976년 옛 소련의 ‘루나 24’ 로봇 탐사 이후 44년 만 This is the first lunar sample collection since the Soviet Luna mission brought its samples home 44 years ago

这是继1976年前苏联“月球24”机器人探测之后时隔44年再次采取月球样本

중국 주요 매체들은 일제히 “중국이 우주 강국의 대열에 들어섰다”며 자축하고 나섰다 Chinese media celebrated the achievement, saying that the nation has joined the ranks of space powers

中国主要媒体一致表示祝贺,称“中国进入了宇宙强国的行列”

중국 관영 신화통신은 이날 “중국이 달 샘플 채취에 성공하면서 미국과 옛 소련 이후 세 번째 달 탐사에 성공한 국가가 됐다”면서 “세 번째이긴 하지만 창어 5호는 기존 탐사와는 다른 점들이 많아 ‘세계 최초’라는 타이틀이 많다”고 강조했다 （no translation）

中国官方媒体新华社当天报道说,“中国在月球成功采取样本,成为继美国和前苏联之后第三个成功探测月球的国家”,并强调,“虽然是第三个,但是‘嫦娥五号’与以往探测有着很多不同的地方,拥有多个‘世界第一’的头衔”

신화통신에 따르면 창어 5호는 지금까지 인류가 가본 적 없는 용암 평원인 ‘폭풍우의 바다’에 착륙했다 이 지역 암석과 토양은 다른 지역에 비해 비교적 최근에 생성돼 달의 화산 활동 연구에 도움이 될 것이라는 기대가 있다 According to Xinhua News Agency, the Chang’e-5 spacecraft landed on a high volcanic region called “Mons Rümker ” Being relatively new, rock and soil from this terrain are expected to help understand the volcanic activity on the Moon

据新华社报道,“嫦娥五号”着陆在人类从未去过过的熔岩平原“暴风雨海洋”该地区的岩石和土壤与其他地区相比,是最近才形成的,因此有人期待对研究月球火山活动有所帮助

중국 관영 글로벌타임스는 “지난해 1월 창어 4호가 인류 최초로 달 뒷면에 착륙한 데 이어 창어 5호 역시 달의 새로운 지역을 처음으로 탐사하면서 ‘최초’ 타이틀을 이어가게 됐다”고 전했다 Chinese state media Global Times reported the Chang’e-5 mission has become the “first” venture that explored the terrain after the Chang’e-4 mission became the first probe to land on the far side of the Moon in January last year

中国的英文官方媒体《环球时报》报道说:“继去年1月‘嫦娥四号’首次登陆月球背面后,‘嫦娥五号’也首次探测月球新区域,延续了‘首次’的称号”

채취한 샘플 무게가 2kg에 이르는 것도 새로운 기록이다 It is also worth noting that two kilograms of samples have been retrieved,

采取的样本重量达到2公斤,这也是新记录

옛 소련의 루나 24가 채취한 330g의 약 6배에 달하는 양이다 six times the 330-gram sample collected by the Soviet Luna mission

这相当于前苏联“月球24”所提取的330克的6倍左右

창어 5호가 다량의 샘플을 채취할 수 있었던 것은 지구로 돌아올 때 이용할 연료를 크게 줄였기 때문이다 This was possible because Chang’e-5’s return journey to Earth required much less fuel

“嫦娥五号”之所以能够采取到大量样本,是因为它大大减少了返回地球时使用的燃料

과거 미국과 옛 소련의 경우 달착륙선이 자체 보유한 연료를 이용해 지구로 귀환했다 Unlike the Soviet Luna mission that used the fuel in the ascending vehicle to return to Earth,

过去美国和前苏联利用登月飞船自身拥有的燃料返回地球

하지만 창어 5호는 달 궤도에서 귀환선과 도킹하는 방식을 이용했기 때문에 상대적으로 적은 연료만으로도 귀환이 가능했다 a robotic vehicle docked with a module in the Chang’e-5 venture, which did not require as much fuel

但“嫦娥五号”由于利用了在月球轨道上与返回舱对接的方式,所以仅靠相对较少的燃料就可以返回

신화통신은 “달 궤도에서 창어 5호와 귀환선의 도킹 작업은 최고의 우주 기술”이라면서 “고속으로 이동하는 우주선 간 안전한 도킹을 위해 연구진이 661번 연습을 진행했다”고 전했다 （no translation）

新华社报道说:“‘嫦娥五号’在月球轨道上和返回舱的对接工作是最好的宇宙技术”,“为了高速移动的宇宙飞船之间的安全对接,研究人员进行了661次练习”

중국은 올해 7월 화성탐사선 ‘톈원(天問) 1호’를 쏘아 올렸고, 이번에 창어 5호가 임무를 마치고 귀환하는 등 ‘우주 굴기’ 계획을 착착 실행하고 있다 （no translation）

中国今年7月发射了火星探测器“天问一号”,此次又有“嫦娥五号”完成任务后返回,正在有条不紊地实施“太空崛起”计划

중국은 지난해 27차례 로켓 발사를 통해 66기의 비행체를 우주로 보냈고, 올해에도 40기 이상의 로켓을 우주로 쏘아 올렸다 （no translation）

中国去年通过27次火箭发射,将66个飞行体送入太空,今年也将40枚以上的火箭发射进入太空

내년에도 최소 80기 이상의 로켓을 우주로 발사할 계획이다 （no translation）

中国明年也计划最少向太空发射80枚以上的火箭

시진핑(習近平) 중국 국가주석은 이날 창어 5호의 무사 귀환을 자축하며 프로젝트에 참여한 연구진에게 당·정·군 수장의 명의로 축전을 보냈다 （no translation）

中国国家主席习近平当天祝贺“嫦娥五号”安全返回,并以党 政 军领导人的名义向参与该项目的研究人员发去了贺电

시 주석은 “이번 프로젝트를 통해 중국의 항공우주 기술에 큰 진전을 이뤘다”고 말했다 （no translation）

习近平表示:“通过此次项目,中国航空航天技术取得了重大进展”

中관영언론 “김치-파오차이 완전히 다른 음식” Kimchi and paocai are two different foods,’

中国官方媒体:“韩式泡菜和中式泡菜是完全不同的食物”

최근 중국 매체가 ‘김치 종주국’ 논란을 촉발시켜 비판을 받은 가운데 중국 관영 언론이 “김치와 파오차이(泡菜)는 완전히 다른 음식”이란 입장을 내놨다 Chinese state media says As the Chinese press is under criticism for causing controversy about where kimchi originated from, a Chinese state media company said kimchi and paocai are completely different

最近,中国媒体因引发“泡菜宗主国”争议而遭到批评,中国官方媒体也表明立场称:“韩式泡菜(Kimchi)和中式泡菜(Paocai)是完全不同的食物”

이번 논란을 ‘단순 번역 오류’라고 설명하면서 한발 물러서는 모양새다 It seems to take a step back by explaining the recent controversy as a misunderstanding in translation

此举似乎是后退了一步,将此次争议解释为“单纯的翻译错误”

중국 관영 영자지 글로벌타임스는 9일(현지 시간) “김치와 파오차이를 둘러싼 논란은 번역 오류로 인한 ‘시시한 소동’에 불과하다”며 두 음식의 차이점을 설명했다 “This confusion that stems from an innocent lost in translation ignited a feud,” China’s state-run English newspaper Global Times said, explaining differences between kimchi and paocai on Wednesday (local time)

中国官方英文报纸《环球时报》当地时间9日就两种食物的差异进行了说明:“围绕韩式泡菜和中式泡菜的争论不过是翻译错误引起的‘无聊之举’”

“두 음식 모두 중국어로 ‘파오차이’라 불리지만 만드는 방식과 재료는 전혀 다르다”라면서 “The two food genres, despite being both called paocai in Chinese term, vary a lot in making methods and selection of raw materials,” the newspaper said

“虽然两种食物用中文都叫‘泡菜’,但制作方法和材料完全不同”,

“발효 음식인 김치는 한국요리를 대표하는 반면 파오차이는 쓰촨성의 절임 채소에서 유래했다”고 전했다 “Kimchi refers to a kind of fermented cabbage dish that plays an integral role in Korean cuisine, while paocai, or Sichuan paocai, refers to pickled vegetables that are popular originally in Southwest China's Sichuan Province

“发酵食品泡菜(Kimchi)代表韩国料理,而泡菜(Paocai)则源于四川省腌制蔬菜”

이 신문은 기사에서 김치를 ‘Kimchi’라고 명확히 표기했는데, 기존의 중국 매체들은 김치라는 표현 자체를 쓰지 않고 파오차이로 통칭해왔다 ” While the English newspaper used the term ‘kimchi,’ other Chinese media companies use the term ‘paocai’ to refer to kimchi

该报在报道中将泡菜明确标注为“Kimchi”,而以往中国媒体则没有使用“Kimchi”这一表述,而是统称为“泡菜”

글로벌타임스는 또 서경덕 성신여대 교수의 요구로 중국 포털 바이두 백과사전이 8일 ‘한국 김치는 중국에서 유래했다’는 부분을 삭제했다며 “중국 전문가들은 이를(김치 기원 논란) ‘시시한 소동’이라고 보고 있다”고 설명하기도 했다 The Global Times also reported that Baidu Baike, China's Wikipedia-like platform, deleted the phrase "Korean kimchi originated from China” on Tuesday upon the request of Seo Kyoung-duk, a professor at South Korea's Sungshin Women's University

《环球时报》还报道称,应诚信女大教授徐敬德的要求,中国门户网站百度百科辞典8日删除了“韩国泡菜源于中国”的内容,并解释说“中国专家们认为这(泡菜起源争议)是‘无聊之举’”

이어 “바이두 이용자라면 누구나 등록·편집·수정할 수 있는 (백과사전) 시스템 때문에 벌어진 해프닝”이라고 주장했다 According to the newspaper, Chinese experts refuted it as "unnecessary fuss," adding that Baidu Baike is a website that can be edited or created by any registered user

报道还声称:“只要是百度用户,任何人都可以登记 编辑 修改(百科全书),这是这一系统问题引起的意外插曲”

그러면서도 “단순 번역 오류를 한국의 김치문화 옹호자들이 ‘(중국이) 우리 문화를 훔치려 한다’고 비판하면서 불화가 시작됐다”며 한국 측에 논란의 책임을 떠넘겼다 At the same time, the article pushed the responsibility for causing the controversy to South Korea, saying that this confusion that stems from an innocent lost in translation ignited a feud with defenders of kimchi culture on South Korean social media, who accused China of attempting to "steal our culture

报道还称,“韩国的泡菜文化拥护者拿着单纯的翻译错误,批判称‘(中国)想偷窃我们的文化’,由此引发了不和”,将争议的责任推给了韩国方面

이번 김치 논란을 촉발시켰던 중국 관영 환추시보도 이날 “김치(Kimchi)는 파오차이와는 다른 음식”이라고 전했다 " The state-run newspapers of China, which caused the kimchi controversy, also said on Wednesday that kimchi and paocai are different foods

引发此次泡菜争议的中国官方中文报纸《环球时报》当天也报道说:“Kimchi与泡菜不同”

김치 종주국을 둘러싼 논란은 지난달 29일 환추시보가 중국이 절임 채소인 파오차이 제조법을 국제표준화기구(ISO)에 등록한 것을 두고 “중국의 파오차이 산업이 국제 시장에서 기준이 됐다”며 “사실 한국이 파오차이 종주국이라는 주장은 이미 유명무실하다”고 주장하면서 시작됐다 한국의 김치와 중국의 파오차이는 엄연히 다른 음식임에도 양국이 김치의 표준규격을 놓고 신경전을 벌이는 듯한 오해를 부른 것 The recent controversy has been brought on due to a misunderstanding that South Korea and China were arguing about the standards of kimchi even though kimchi and paocai are two completely different foods

围绕泡菜宗主国的争论始于上个月29日《环球时报》就中国向国际标准化组织注册腌制蔬菜法一事的报道报道称:“中国的泡菜产业在国际市场上成了标准”,“事实上,韩国是泡菜宗主国的主张已经有名无实”尽管韩国的泡菜和中国的泡菜是完全不同的食物,却引发了两国就泡菜标准规格展开神经战的误会

이후 논란이 커졌지만 중국은 별다른 입장을 내놓지 않다가 9일 만에 ’번역 실수‘라고 한 것 While the controversy grew over the last nine days, China had not made any statement and later announced that is was a translation mistake

此后争议不断扩大,但中方没有做出特别的表态,仅过9天就说是“翻译失误”

조정은 세계김치연구소 전략기획본부장은 “이번 상황은 일단락됐지만 중국의 ‘김치공정’은 늘 대비해야 한다”며 “한국을 대표하는 음식 문화유산인 김치를 지켜내기 위해선 전략적 접근이 필요하다”고 강조했다 （no translation）

世界泡菜研究所战略企划本部长赵贞恩(音)强调:“这次情况暂告一段落,但应该长期做好应对中国‘泡菜工程’的准备”,“代表韩国的饮食文化遗产——泡菜,需要战略性的保护”

“北, 中에 올해 4000억원대 석탄 밀수출" N Korea smuggled over $400 million worth of coal to China

美国媒体:“朝鲜今年向中国走私价值4000亿韩元的煤炭”

유엔 제재로 석탄 수출이 금지된 북한이 올 1∼9월 중국에 최대 4억1000만 달러(약 4452억 원)어치의 석탄을 수출한 것으로 분석됐다North Korea is believed to have exported up to 401 million dollars worth of coal to China between January and September of this year despite UN sanctions banning North Korean coal exports

据分析,因联合国制裁而被禁止出口煤炭的朝鲜今年1月至9月向中国出口了价值最多可达4.1亿美元(约合4452亿韩元)的煤炭

북한 선박은 인공기를 버젓이 달고 운항하기도 해 대북 제재에 구멍이 크다는 지적이 나온다 Experts point out that there are holes in the sanctions as North Korean vessels openly operated flying the North Korean flag

有人指出,朝鲜船只还堂而皇之地悬挂国旗航行,显示对朝制裁存在很大的漏洞

월스트리트저널(WSJ)은 미 국무부 관료 인터뷰와 국무부가 제공한 위성사진을 토대로 “북한 선적 선박들이 지난 1년 동안 중국 닝보-저우산 지역으로 수백 차례 석탄을 직접 실어 날랐다”고 7일(현지 시간) 보도했다 The Wall Street Journal (WSJ) on Monday cited satellite images provided by the U S State Department showing that North Korean vessels have shipped hundreds of coal shipments to China’s Ningbo-Zhoushan area for the past year

《华尔街日报》以对美国国务院官员的采访和国务院提供的卫星照片为基础,当地时间7日报道说:“朝鲜船只在过去一年里向中国宁波-舟山地区直接运送了数百次煤炭”

구체적으로 8월 12일 촬영된 위성사진에는 인공기를 단 석탄 운반선 4척이 중국 닝보-저우산항 인근 해역에 중국 선박과 함께 정박한 것이 포착돼 석탄 불법 환적을 한 것으로 분석됐다 More specifically, a satellite image taken on Aug 12 showed four North Korea-flagged vessels anchoring off the Ningbo-Zhoushan area with Chinese ships, suggesting an illegal ship-to-ship transfer of coal

具体是在8月12日拍摄的卫星照片中,4艘悬挂朝鲜国旗的煤炭运输船在中国宁波-舟山港附近海域与中国船舶停泊在一起,分析认为是进行了非法转运

북한은 올해 들어 9월까지 410만 t의 석탄을 수출한 것으로 미 정부는 파악하고 있다 Washington believes Pyongyang has smuggled 4. 1 million tons of coals by Sep this year

据美国政府掌握的情况,朝鲜今年前9个月共出口了410万吨煤炭

1t당 80∼100달러에 팔렸다고 가정할 때 수출액은 3억3000만∼4억1000만 달러(약 3583억∼4452억 원)에 이른다 Assuming that the coals were sold for 80-100 dollars per ton, the total amount of export is between 330 million dollars and 410 million dollars

假设以每吨80美元至100美元的价格销售,出口额将达到33亿至4.1亿美元(约合3583亿至4452亿韩元)

미국을 비롯한 국제사회는 북한의 불법 환적에 대한 공동 감시 태세에 나선 상황이지만 북한 선박은 인공기까지 달고 운항했다고 WSJ는 전했다 그동안 외국 국적 선박을 동원하거나 선박의 명칭을 바꾸고, 선박위치식별장치(AIS)를 끄는 등 회피 수법을 써왔지만 이젠 대놓고 불법 환적에 나서고 있는 것 The international community including the U S is jointly monitoring North Korea’s illegal ship-to-ship transfer but the WSJ reported that North Korean vessels were sailing under the North Korean flag

《华尔街日报》报道称,以美国为首的国际社会虽然对朝鲜非法转运采取了共同监视态势,但朝鲜船舶还是挂着国旗航行。此前,朝鲜曾采用动用外国籍船舶 更换船舶名称 关闭船舶位置识别装置等回避手段,但现在却明目张胆地非法转运

미국 측은 중국이 유엔 제재를 무시하기 때문에 벌어지는 일이라고 비판한다 The U S accused China of ignoring the UN sanctions against North Korea

美国方面批评说,这是中国无视联合国制裁而发生的事情

미 국무부 고위 관리는 “북한에서 중국으로 (석탄을) 직접 운송하는 건 2017년 유엔 안보리의 제재 채택 이후 처음 목격하는 큰 변화”라며 “중국과 북한이 더 이상 제재 감시를 피하기 위해 위장하려 애쓰지 않고 있다”고 말했다 A senior U S State Department official said that a direct shipment of coal from North Korea to China is the first major change we have seen since the adoption of UN sanctions against North Korea in 2017, adding that China and North Korea are no longer trying to hide their smuggling activity

美国国务院高层官员说:“自2017年联合国安理会通过制裁决议以来,首次亲眼目睹了从朝鲜直接向中国运送煤炭的巨大变化”,“中国和朝鲜不再为逃避制裁监视而努力伪装”

美의회, 中견제 구상에 국방예산 2조원 책정US Congress agrees on bill for counter-China initiative

美国议会就牵制中国构想制定2万亿韩元

미국 의회가 2021회계연도 국방예산안을 담은 국방수권법(NDAA) 법안에 ‘태평양억지구상(Pacific Deterrence Initiative)’ 항목을 신설하고 22억 달러(약 2조3800억 원)를 배정했다 U S Congress has authorized the new National Defense Authorization Act (NDAA) for fiscal year 2021, agreeing to set aside 2. 2 billion dollars for the new Pacific Deterrence Initiative

国防预算美国议会在包含2021会计年度国防预算案的《国防授权法(NDAA)》法案中新设了“太平洋地区构想(PacificDeterrenceInitiative)”项目,并分配了22亿美元(约2.38万亿韩元)预算

사실상 중국을 겨냥한 국방예산을 신설하면서 내년 1월 조 바이든 행정부 출범 이후에도 미국의 대중 강경정책이 이어질 것임을 예고했다 By establishing a counter-China fund, the Congress signaled that the U S will maintain its tough stance on China even after Joe Biden takes office in January 2021

事实上,美国新设了针对中国的国防预算,这预示着明年1月拜登政府上台后,美国将继续采取强硬的对华政策

6일(현지 시간) 워싱턴포스트(WP)에 따르면 상하원이 내놓은 NDAA 법안 중 인도태평양 지역에 관한 부분에 태평양억지구상 항목이 추가됐다 According to The Washington Post on Dec 6 (local time), a new program called Pacific Deterrence Initiative has been added to the new NDAA released by Senate and House Armed Services Committees as part of the efforts to strengthen the U S military capability in the Indo-Pacific region

据《华盛顿邮报》(WP)6日(当地时间)透露,参众两院提出的NDAA法案中,关于印度太平洋地区的部分增加了“太平洋遏制构想”项目

법안에는 국방장관이 역내 미군 주둔 병력의 현대화 및 강화 방안이 담긴 계획을 수립하고, 인도태평양사령부와 협의를 거쳐 이 내용이 담긴 보고서를 2021년 2월 15일까지 의회에 제출하도록 돼 있다 Under the bill, the Secretary of Defense, in consultation with the Commander of United States Indo-Pacific Command, shall summit to the congressional defense committees a report that contains a detailed summary of the activities and resources of the Indo-Pacific Deterrence Initiative not later than Feb 15, 2021

法案规定,国防部长要制定包含地区内美军驻扎兵力现代化及强化方案的计划,并与印度太平洋司令部进行协商,在2021年2月15日之前向议会提交包含上述内容的报告书

법안은 “이 구상은 인도태평양 지역에서 미국의 억지력과 국방력, 준비태세를 강화하고 역내 동맹 및 파트너 국가들을 확신시키기 위해 우선시되는 활동들을 수행하기 위한 것”이라고 명시했다 The bill stipulates that the purpose of the initiative is to enhance the United States deterrence and defense posture in the Indo-Pacific region and to carry out activities necessary to assure allies and partners in the region

法案明确规定:“这一构想是为了强化美国在印度太平洋地区的威慑力 国防力和准备态势,并为了确保区域内同盟及伙伴国家,执行优先考虑的活动”

이 법안이 시행되면 미국이 인도태평양에서의 억지력을 높이기 위해 주한미군을 감축하지 않고 유지할 가능성이 높다 Once the bill goes into effect, it is highly likely that the U S will decide not to shrink its military presence in South Korea in order to enhance its military deterrence in the Indo-Pacific region

如果该法案得以实施,美国为了提高在印度太平洋的威慑力,很有可能不缩减驻韩美军,而是维持下去

한편으로는 미국이 한국을 향해 반중(反中) 연합전선에 참여하라는 압박이 심해질 수 있다는 분석도 나온다 But experts also say that the U S could increase its pressure on South Korea to join its anti-China front

另一方面,也有分析认为,美国向韩国施压,要求韩国加入反华联合阵线

中, 4년만에 한국게임 서비스 허가 한한령 풀리나 촉각China gives approval to Korean gaming service in four years

中国4年以来首次批准韩国游戏服务,限韩令解除or只是个例

중국 정부가 약 4년 만에 처음으로 한국 게임에 ‘판호(版號·중국 내 게임 서비스 허가)’를 내줬다 The Chinese government granted a business license to a South Korean gaming program for the first time in four years

中国政府时隔约4年首次向韩国游戏颁发了“版号(中国国内游戏服务许可)”

한국 게임에 대한 ‘한한령(限韓令·한류 제한령)’이 풀리는 신호탄이라는 기대와 일회성에 그칠 것이라는 부정적 전망이 동시에 나온다 Some expect that it signals the lessening of China’s regulations on South Korean gaming software while others dismiss it as a one-time exception

同时出现了解除对韩国游戏的“限韩令(韩流限制令)”信号弹的期待和仅限于一次性的负面预测

3일 게임사 컴투스에 따르면 2일 중국 국가신문출판서는 홈페이지에 컴투스의 모바일 역할수행게임(RPG)인 ‘서머너즈 워: 천공의 아레나’에 대해 판호를 발급했다고 공지했다 South Korean gaming developer Com2uS officially announced on Thursday that its mobile game Summoners War: Sky Arena received a license from China’s State Administration of Press and Publications the day before

3日,据韩国游戏开发商Com2uS透露,2日中国国家新闻出版署在网站上发布公告称,已向Com2uS的移动角色扮演游戏(RPG)《魔灵召唤:天空之役》下发了版号

2014년 6월 전 세계에 선보인 서머너즈 워는 컴투스의 해외 매출 중 약 80%를 차지하는 인기 게임이다 Since its global launch in June 2014, Summoners War: Sky Arena has become Com2uS’s flagship product, contributing to more than 80 percent of the developer’s overseas sales

2014年6月向全世界推出的《魔灵召唤(SummonersWar)》是Com2uS海外销售额中约占80%的人气游戏

컴투스는 2016년 말 중국 시장에 서머너즈 워를 선보이기 위해 판호 발급을 신청했다 Later 2016, Com2uS applied for a business license with the aim of helping Summoners War: Sky Arena penetrate the Chinese market

Com2uS为了2016年末在中国市场展示《魔灵召唤》,申请了版号发放

하지만 2017년 사드(THAAD·고고도미사일방어체계) 배치를 놓고 한중 간 갈등이 확대되면서 중국은 비공식적으로 한국산 게임 등 문화 콘텐츠에 대한 유통을 제한해왔다 However, the dispute between Seoul and Beijing over the deployment of Terminal High Altitude Area Defenseless (THAAD) only worsened their diplomatic relationship in 2017 while the Chinese government unofficially put restrictions on the distribution of South Korean pop cultural content including gaming services

但随着2017年围绕萨德(THAAD)部署问题韩中矛盾不断扩大,中国一直非正式地限制韩国游戏等文化产品的流通

특히 게임의 경우 2017년 초를 마지막으로 판호를 발급해주지 않았다 Early 2017 was the last time when it issues a business license to a South Korean game

特别是游戏,在2017年初最后一次发放之后就一直没有再发放版号

컴투스 관계자는 “오래전에 신청했던 건인 만큼 전혀 예상하지 못했고, 사전 연락도 없었다”고 말했다 “It was a long time ago that we applied for a license so we did not expect it to happen

Com2uS相关人士表示:“因为是很久以前申请的,所以完全没有想到,事前也没有联系”

중국 정부의 깜짝 판호 발급에 국내 게임업계에서는 세계 최대 게임 시장 중 하나인 중국 시장의 문이 다시 열리는 것 아니냐는 기대가 나오고 있다 We were not notified of the news before,” according to an executive at Com2uS

对于中国政府突然发放版号,韩国国内游戏业界期待称,作为世界最大的游戏市场之一的中国市场大门是否再次开启

중국 시장 진출을 앞두고 있는 넥슨의 모바일게임 던전앤파이터를 비롯해 과거 중국에서 인기를 얻었던 지식재산권(IP)을 활용한 게임들의 수출이 조만간 이루어질 수 있다는 것이다 China’s surprise issuance of the business license is increasing expectations across the South Korean gaming industry that one of the world’s largest gaming markets may reopen its door for South Korean gaming businesses

包括即将进军中国市场的Nexon手机游戏《地下城与勇士》在内,利用过去在中国人气颇高的知识产权(IP)的游戏的出口有望在不久的将来实现

이 같은 기대감에 3일 국내 주식시장에서 컴투스가 전날 대비 6. 19% 상승 마감한 것을 비롯해 위메이드(5. 75%), 펄어비스(14. 11%), 넷마블(3. 59%), 엔씨소프트(2. 21%) 등 게임사들의 주가가 강세를 보였다 Optimists anticipate that Beijing will soon allow Nexon’s flagship title Dungeon & Fighter, which is soon to attempt to make inroads into China, and other games based on intellectual property well-received by Chinese users to have access to its domestic market Such heightened market expectations translated into rising stock prices of South Korean gaming developers on Thursday

由于这种期待,3日国内股市上Com2uS比前一天上涨6.19%,Wemade(5.75%) Pearlabyss(14.11%) NetMarble(3.59%) NC软件(2.21%)等游戏公司的股价表现强势

다만 이번 조치로 중국 시장이 다시 열렸다고 판단하기는 이르다는 신중한 반응도 나오고 있다 중국이 한한령을 전면 철폐했다고 확신하기 위해서는 추가 판호 발급이 이어져야 한다는 것이다 Meanwhile, others concern that it is too early to conclude that the Chinese market is now accessible, saying that extra license issuances should follow in order for South Korean gaming developers to be assured of China completely removing restrictions on them

但也有人慎重地表示,认为此次措施使中国市场重新打开还为时尚早。为了确信中国已经全面撤销了限韩令,应该继续发放追加版号

위정현 한국게임학회장은 “중국이 판호를 줄줄이 내줄 것이라고 기대하기는 어렵다”며 “다만 이번 판호 발급을 지렛대로 삼아 정부와 업계 등이 치열한 외교적, 전략적 노력을 해야 한다”고 지적했다 （no translation）

韩国游戏学会会长俞正铉(音)指出:“很难期待中国会陆续发放版号”,“但是,韩国相关政府部门和游戏业界应把此次版号发放作为杠杆,今后更积极地致力于解决版号问题。”

BBC “중국 김치가 세계 표준 중 언론 오보” BBC calls reports of kimchi by Chinese media false

英国广播公司(BBC):“中国泡菜成为世界标准”

중국이 절임 채소인 파오차이 제조법을 국제표준화기구(ISO)에 등록한 것을 놓고 중국 관영매체가 김치 국제표준을 제정한 것처럼 보도한 것에 대해 영국 BBC가 ‘오보(false report)’라며 중국의 주장을 지적했다 Chinese media reported that China set an international standard for kimchi after the country received certification for pao cai, a type of Chinese fermented vegetables, from the International Organisation for Standardisation (ISO), and BBC described the report as “false

……中国媒体纯属误报”针对中国将腌制蔬菜“泡菜(Paocai)”的制作法登记到国际标准化组织一事,中国官方媒体报道似乎称中国制定了泡菜的国际标准,英国广播公司指出中国的主张,认为这是“误报”

BBC는 지난달 30일(현지 시간) ‘김치, 한중 문화 갈등을 발효하다’는 제목의 기사에서 “중국이 한국 전통 음식인 김치 제조법을 국제적으로 인정받았다는 오보에 한국이 반박하고 나섰다”며 “(김치 논란은) 한국과 중국 간 가장 최근에 벌어진 문화 분쟁”이라고 전했다 ” In an article entitled, “Kimchi ferments cultural feud between South Korea and China,” BBC said, “South Korea has rebuffed China after false reports that it had won global certification for its production of kimchi - a hallowed dish for Koreans

英国广播公司当地时间11月30日在题为《泡菜,令韩中文化矛盾发酵》的报道中称:“中国误报说(其)关于韩国传统饮食泡菜的制作方法得到了国际认可,韩国对此进行了反驳”,“(泡菜争议)是韩国和中国最近发生的文化纠纷”

앞서 지난달 29일 중국 관영매체 환추시보는 파오차이 국제표준을 제정했다고 보도하면서 ” It also said, “It's the latest cultural spat between the neighbours

此前,中国官方媒体《环球时报》10月月29日报道称,中国制定了“泡菜国际标准”,

“중국의 파오차이 산업이 국제 시장에서 기준이 됐다”면서 “사실 한국이 ‘파오차이 종주국’이라는 주장은 이미 유명무실하다”고 주장했다 ” Chinese state media Global Times reported on Sunday that the ISO status was "an international standard for the kimchi industry led by China,” adding that, contrary to popular belief, “pao cai” has not originated from South Korea

并声称“中国的泡菜产业已成为国际市场的标准,事实上,韩国是‘泡菜宗主国’的主张已经有名无实”

파오차이와 김치를 같은 음식인 것처럼 표현하고, 파오차이를 국제표준으로 등록한 것에 한국 김치까지 포함되는 것처럼 선전한 것이다 It described kimchi as the same dish as pao cai and claimed the award covered kimchi

相关报道将“中国泡菜(Paocai)”和“韩国泡菜(Kimchi)”形容为同样的食物,把登记为国际标准的“Paocai”包含韩国泡菜一样进行宣传

이에 한국 농림축산식품부는 즉각 2001년 유엔 국제식량농업기구 산하 국제식품규격위원회(CODEX)의 규격에 따라 김치는 국제규격으로 설정됐으며, 파오차이는 김치로 해석할 수 없다고 반박한 바 있다 The South Korean Ministry of Agriculture, Food and Rural Affairs refuted the claim, saying that international standards for kimchi were agreed by the United Nations in 2001 in line with CODEX Alimentarius It also said kimchi and pao cai are two different dishes

对此,韩国农林畜产食品部立即反驳道:根据2001年联合国国际粮农组织下属的国际食品法典委员会(CODEX)的规格,韩国泡菜被设定为国际标准,“Paocai”不能解释为韩国泡菜

BBC도 농식품부의 주장을 인용하면서 김치의 특성과 김장문화를 소개했다 BBC quoted the statement and introduced kimchi and kimjang, the communal act of making kimchi

”英国广播公司也援引农食部的主张,介绍了韩国泡菜的特性和腌泡文化”

“매운 염장 음식인 김치는 중국에서 파오차이라는 이름으로 공급되고 있기는 하지만 같은 이름의 중국 고유 음식이 있다”며 “ISO 문서에는 이번 식품 규격이 ‘김치에는 적용되지 않는다’고 적시돼 있는데도 일부 중국 언론은 이와 다르게 보도했다”고 지적했다 “Kimchi is often served in China under the name pao cai, but China has its own variant of the dish which it also calls pao cai,” it said “Although the ISO listing clearly says "this document does not apply to kimchi", some Chinese media suggested otherwise

属于辛辣腌制食品的泡菜虽然在中国以‘Paocai’的名称供应,但是中国也有相同名称的固有食品,国际标准化组织(ISO)文件中虽然指出这一食品规格‘不适用于韩国泡菜’,但部分中国媒体却进行了与此不同的报道”

이어 “김치는 채소를 소금에 절인 뒤 양념과 발효된 해산물을 넣고 항아리에 보관한다”며 “매년 김치를 만드는 김장은 유네스코 인류무형문화유산에 등재돼 있다”고 소개했다 （no translation）

报道还介绍说,“韩国泡菜是将蔬菜用盐腌制后放入调料和发酵的海产品并放入缸中保管,每年制作泡菜的腌制泡菜已被联合国教科文组织列入人类非物质文化遗产名录”

또 “한국은 김치 수요가 많아 중국에서 수입하지만 한국은 중국의 엄격한 규제로 수출길이 사실상 막혔다”고 덧붙였다 （no translation）

报道说:“韩国泡菜需求量大,需要从中国进口,但由于中国的严格限制,韩国的出口之路实际上已被堵住”

BBC는 최근 이어진 한국과 중국 간 문화 갈등 사례도 소개했다 （no translation）

英国广播公司还介绍了最近韩国和中国之间文化矛盾的事例

10월 방탄소년단(BTS)이 6·25전쟁 70주년을 맞아 한미 관계 발전에 기여한 공로로 밴플리트상을 받으며 “(한미) 양국이 함께 겪었던 고난의 역사”라는 표현을 쓰자 중국 누리꾼들이 “중국의 희생을 무시했다”며 집단으로 반발했다 （no translation）

10月防弹少年团(BTS)在6·25战争70周年之际,因对韩美关系发展做出的贡献而获得范弗里特奖,并使用了“(韩美)两国共同经历的苦难历史”的表述,引起中国网民集体抗议,称“无视中国的牺牲”

11월에는 중국 배우 쉬카이(許凱)가 중국 소셜미디어 웨이보에 ‘한복은 중국 의상’이라는 취지의 글을 올려 논란이 일었다 （no translation）

11月,中国演员许凯在中国社交媒体微博上发表题为《韩服是中国服装》的文章,引起了争议

왕이가 띄운 ‘한중일FTA’, 아직은 외교•경제 리스크 크다 ” Risks over Korea-China-Japan FTA

王毅提出的“韩中日自贸协定”,目前外交和经济风险仍然很大

최근 한국과 일본을 순방한 왕이 중국 외교부장이 ‘한중일 자유무역협정(FTA)’ 추진을 주장했다 Chinese Foreign Minister Wang Yi, who recently visited Seoul and Tokyo,

最近巡访韩国和日本的中国外交部长王毅主张推进“韩中日自由贸易协定”

왕 부장은 강경화 외교부 장관과의 회담에서 “한중일FTA를 적극 추진해보자”고 했고 일본 외무상과의 회담에서도 이를 강조했다 has proposed a push for a tripartite free trade agreement among the three neighboring countries of Asia

王毅部长在与外交部长康京和的会谈中表示,“要积极推进韩中日自贸协定”,在与日本外相的会谈中也强调了这一点

중국 환추시보는 “한중일 FTA는 동북아 지역협력 제도의 부족함을 보충하는데 효과적”이라며 분위기 띄우기에 나섰다 In a meeting with Korean Foreign Minister Kang Kyung-wha, Minister Wang Yi strongly proposed to establish a Korea-China-Japan FTA, and the same message was stressed during the meeting with his Japanese counterpart “The three-party FTA will prove to be effective in complementing the lack of cooperative institutions in the North East Asian region,” argued the Chinese daily Global Times

中国《环球时报》报道说:“韩中日自贸协定有效弥补了东北亚地区合作制度的不足”

한중일FTA는 기대 효과와 함께 경제적 외교적 리스크도 큰 만큼 신중하게 접근해야 한다 Given the economic and diplomatic risks it entails, however, a tripartite trade deal must be approached with caution

韩中日自贸协定在取得期待效果的同时,也存在很大的经济 外交风险,因此要慎重对待

한중일 FTA는 2013년부터 협상이 시작됐으나 핵심 분야에 대한 이견과 외교 갈등으로 협상이 부진했다 The first negotiation for a tri-party trade deal among Beijing, Seoul, and Tokyo, began in 2013, but there has been little progress owing to the differences of opinions in core agendas and their concomitant diplomatic disputes

韩中日自贸协定谈判始于2013年,但由于对核心领域的分歧和外交矛盾,谈判停滞不前

세계 경제의 24%를 차지하는 세 나라가 상품과 서비스 장벽을 없애면 경제성장을 촉진하는 효과가 생길 수 있으나 그만큼 위험도 크다 Once the three Asian economic powerhouses, which take up 24% of global GDP, remove the barriers for their products and services, it will certainly galvanize economic growth, but it will also invite as much risk

占世界经济24%的三个国家如果消除商品和服务壁垒,可能会产生促进经济增长的效果,但是其危险也很大

세계적인 제조업 강국인 3국은 반도체 자동차 철강 등 핵심 수출 산업에서 겹치는 부분이 많다 As big manufacturers in the world, the three Asian neighbors share many overlapping sectors of export such as semi-conductors, cars, and steel

三国作为世界性制造业强国,在半导体 汽车 钢铁等核心出口产业中有很多重叠的部分

한중 FTA가 2015년 발효됐는데 그 이상의 개방은 우리 산업에 타격을 줄 우려가 있다 The FTA with China took effect in 2015, and for Seoul,

韩中自贸协定于2015年生效,但进一步的开放令人担心有可能对韩国产业造成打击

한국과 일본도 자국 산업 보호를 위해 서로 개방을 꺼리는 분야가 많다 if the market is pried open further, it might deal a heavy blow to its vital industries

韩国和日本也为了保护本国产业,很多领域都不愿互相开放

한중일 FTA가 그동안 여러 차례 논의됐으나 더 이상 진전되지 못한 것도 이 때문이다 In fact, this is why the tri-party FTA has failed to make meaningful progress despite the series of talks and negotiations

韩中日自贸协定此前曾多次讨论过,但未能取得进一步进展,也是因为这个原因

정치 외교적인 측면에서의 함의도 빼놓을 수 없다 The political and diplomatic implications should be taken into account as well

政治外交层面的含义也必不可少

미국의 정권 교체를 앞두고 중국이 지역협력과 경제통상 문제를 꺼내드는 것은 동북아지역에서 미국의 영향력을 견제하려는 의도가 깔려 있다 Behind Beijing’s proposal for regional cooperation and trade deals amid Washington’s power transition is China’s intention to keep in check America’s influence in North East Asia

在美国政权交替之际,中国提出地区合作和经济贸易问题,其目的在于牵制美国在东北亚地区的影响力

그동안 한중일FTA 협상은 한일이 과거사 문제로 갈등하고 중국이 한국의 고고도미사일방어체계(THAAD)를 트집 잡아 중단되는 등 정치 외교적 영향을 크게 받았다 History attests to the fact that the three neighbors’ attempt to seal a free trade deal has been gravely swayed by political and diplomatic factors such as Beijing’s Seoul-bashing over the installation of the THAAD system and the historical disputes between Korea and Japan

此前,韩中日自贸协定谈判因韩日历史问题产生矛盾,中国也挑韩国“萨德(末段高空区域防御系统)”的毛病而中断,受到的政治外交影响很大

미국은 조 바이든 대통령 당선인이 ‘민주주의 정상회의’ 개최를 추진하면서 중국 견제에 나섰다 In response, U S President-elect Joe Biden is proposing to hold a “Summit for Democracy

美国当选总统拜登推进召开“民主首脑会议”,开始牵制中国

바이든 당선인은 대선 캠페인 과정에서 “세계 민주 국가들이 모여 민주주의 체제를 강화하고, (민주주의에) 역행하는 국가들에 맞서자”고 했는데 최근 본격화 움직임을 보이고 있다 ” During his campaign, Biden said the gathering will “bring together the world’s democracies to strengthen our democratic institutions, honestly confront nations that are backsliding,” and certainly there are signs that he is willing to put those words into action

拜登在竞选活动中曾表示,“世界民主国家聚集在一起,加强民主体制,对抗逆行(民主)的国家”,最近出现了正式动向

미중 양쪽에서 끌어당기는 힘이 커지면서 한국의 ‘전략적 모호성’은 또다시 시험대에 올라섰다 Korea’s “strategic ambiguity” is being tested yet again between the growing pressure from America and China

随着美中两国拉拉扯扯的力量增强,韩国的“战略模糊性”再次面临考验

미중의 패권 경쟁에서 등터지는 새우가 되지 않으려면 될지, 전략적 활용으로 국익을 높일 지는 정부 하기에 달렸다 Sandwiched between the two superpowers, Seoul must make a wise strategic choice for its own national interests

在美中霸权竞争中,韩国是不想成为“开背虾”就行,还是通过战略性利用提高国家利益,这取决于政府的行动

귀국 한국인 직원 감염… SK하이닉스 中공장 가동중단SK Hynix Chinese plant suspends operation due to COVID-19 case

回国的韩国职员确诊感染新冠病毒

SK하이닉스 중국 충칭(重慶) 공장에 파견을 나갔던 한국 직원이 신종 코로나바이러스 감염증(코로나19) 확진 판정을 받아 중국 현지 공장 가동이 중단됐다 A South Korean worker who was dispatched to SK Hynix’s plant in Chongqing, China tested positive for COVID-19, which resulted in the suspension of the Chinese plant’s operation

SK海力士中国工厂现已停产被派往SK海力士中国重庆工厂的韩国职员被确诊感染新型冠状病毒肺炎(COVID-19),中国当地工厂暂时中断生产

중국 충칭시 정부는 해당 공장에 근무하는 전 직원 2700여 명의 코로나19 전수 조사를 명령했다 The Chongqing city government ordered COVID-19 testing for all 2,700 employees working at the plant

中国重庆市政府下令对在相关工厂工作的2700多名全体员工进行新冠肺炎专项调查

29일 중국 관영 신화통신 및 재계에 따르면 SK하이닉스 직원 A 씨는 약 1년 6개월 동안의 중국 근무를 마치고 인천공항으로 돌아와 받은 코로나19 검사에서 28일 양성 판정을 받았다 According to China’s state-run Xinhua News Agency and sources in the South Korean business community on Sunday, an SK Hynix employee tested positive when he took COVID-19 test upon arrival on Sunday at Incheon International Airport after completing his 18-month secondment to China

29日,据中国官方新华通讯社及企业界透露,SK海力士职员A某结束在中国为期1年零6个月的工作后,回到仁川机场接受新冠病毒检查,并于28日被判定为阳性

A 씨가 확진 판정을 받은 직후 충칭시는 A 씨가 근무했던 SK하이닉스 공장에 대한 역학 조사를 진행하고, 현장 통제 및 소독 등 긴급 조치를 시행하는 것으로 알려졌다 Right after the man was confirmed as a COVID-19 patient, the city of Chongqing conducted epidemiological survey at, restricted access to, and decontaminated the SK Hynix plant where the Korean national worked

据悉,A某被确诊后,重庆市立即对A某曾工作的SK海力士工厂进行流行病学调查,并实行现场控制及消毒等紧急措施

재계 관계자는 “충칭시 및 SK하이닉스 등은 A 씨가 특별한 증상이 없었던 무증상 감염자로 추정하고 있다”라며 “The Chongqing city government and SK Hynix suspect that the employee is an asymptomatic case without displaying notable symptoms,” a South Korea business community source said

财界相关人士表示:“重庆市及SK海力士等机构推测A某是没有特别症状的无症状感染者”,

“충칭시는 A 씨가 묵었던 호텔 역시 운영을 중단하고 25일부터 이 호텔에 묵었던 투숙객들을 대상으로 핵산 검사를 진행하고 있다”고 말했다 “The City of Chongqing also suspended the operation of the hotel where the SK employee stayed, and started PCR testing in people who stayed at the hotel since November 25

“A某下榻的所住的重庆某假日酒店暂停营业,封闭管理,对酒店所有员工及11月25日以来的所有入住客人进行追踪和核酸检测”

가동이 중단된 SK하이닉스 충칭 공장은 반도체 패키징(포장) 등 후공정 작업을 하는 곳이다 SK Hynix’s Chongqing plant is a facility for late-state processing including semiconductor packaging

停止运转的SK海力士重庆工厂是进行半导体包装等后工序的地方

일반적으로 반도체 생산 공장은 잠시라도 전력 공급이 끊기면 생산 라인을 복구하기까지 수일이 걸리고, 피해액도 크다 Generally, if a semiconductor factory stops operation even momentarily due to power outage, it takes days before recovering production lines, and can cause massive damage

一般来说,半导体生产工厂一旦电力供应中断,恢复生产线需要数天时间,损失额也很大

반면 이번에 가동이 중단된 패키징 공장은 시 정부의 방역 조치가 완료되면 곧바로 정상 가동이 가능할 것으로 보인다 The chip packaging plant where operation has been suspended this time will likely resume operation as soon as the city authority completes quarantine measures

相反,此次停止运转的包装工厂在市政府防疫措施完成后,有望立即恢复正常运转

하지만 현재로서는 가동 재개 시점이 불투명해 공장 가동 중단 장기화 시 생산 차질을 피하긴 어려울 것으로 전망된다 However, it is unknown at this point in time when the plant will actually resume operation, and the company will inevitably face disruptions in production if the plant inevitably remains suspended for an extended period of time

但就目前而言,恢复生产的时间尚不明确,如果工厂长期停产,将很难避免生产受阻

SK하이닉스 측은 이날 “중국 정부에 적극 협조해 빠른 시일 안에 조업이 재개될 수 있도록 최선을 다하겠다”고 밝혔다 “We will fully cooperate with the Chinese government, and do our best to resume operation as soon as possible,” SK Hynix said Sunday

SK海力士方面当天表示:“将积极协助中国政府,尽最大努力尽快恢复生产”

“이소룡, 여전히 우리 가슴속에 있다” 탄생 80주년 행사 잇따라다 Events commemorating Bruce Lee held in Greater China

“李小龙依然活在我们心中”,李小龙诞辰80周年纪念活动接连举行

홍콩의 전설적 액션스타 이소룡(리샤오룽·李小龍·1940∼1973·사진)의 탄생 80주년을 맞아 중화권에서 그를 기리는 행사가 잇따르고 있Events remembering Bruce Lee (1940-1973), a legendary action movie star of Hong Kong, have been held in Greater China to mark the 80th year of his birth

在迎来香港传奇动作明星李小龙(1940~1973年?照片)诞辰80周年之际,中华圈接连举行各种活动纪念李小龙

28일 중국 관영 영자지 글로벌타임스에 따르면 이소룡 부친의 고향이자 이소룡이 유년 시절 잠시 거주한 광둥성 포산에서는 1940년 11월생인 이소룡을 기려 이달 초부터 ‘이소룡 탄생 80주년’ 행사가 열리고 있다 According to the report of Global Times, a state-owned English newspaper in China, published on Saturday, Foshan, Guangdong Province, has been holding an event marking the 80th year of his birth (November 1940) from early this month

28日,据中国官方英文报纸《环球时报》报道,在李小龙父亲的故乡 李小龙幼年时期暂时居住的广东省佛山,为了纪念1940年11月出生的李小龙,从本月初开始举行“李小龙诞辰80周年”活动

닮은 사람 찾기 콘테스트, 이소룡의 삶을 회고하는 온·오프라인 행사 등이 잇따른다 A look-alike contest, online/offline events to look back on his life and other events are held

寻找相似的人比赛 回顾李小龙人生的线上?线下活动等接连不断

포산 당국은 이런 행사를 통해 중국 무술이 아닌 전 세계의 무술문화 중심지로 발돋움하겠다는 속내를 드러내고 있다 The Foshan government intends to make the city the center of the world’s martial arts culture

佛山当局希望通过这样的活动,使佛山成为中国武术乃至全世界的武术文化中心

홍콩 우체국 또한 ‘세계 무술 속 이소룡의 유산’을 주제로 특별우표 발행에 나섰다 Hong Kong’s post office also issued special stamps under the theme of “Bruce Lee’s heritage in global martial arts

香港邮政局也以“世界武术中的李小龙的遗产”为主题,开始发行特别邮票

홍콩 센트럴 지역을 관통하는 일부 트램에도 이소룡의 탄생 80주년을 기념하는 광고가 전면에 실렸다 ” Trams running through Hong Kong’s central region also put up an advertisement commemorating the 80th year of his birth

贯通香港中环地区的部分有轨电车上也全面刊登了纪念李小龙诞辰80周年的广告

이 트램은 내년 1월까지 운영될 예정이다 They will operate until January

该有轨电车将运营到明年1月

웨이보 등 중국 소셜미디어에서도 여러 누리꾼이 이소룡 동영상과 사진을 공유하며 그의 탄신일을 기렸다 Internet users shared video clips and photos of Bruce Lee commemorating his birth on Chinese social networks including Weibo

在微博等中国社交网络媒体上,很多网民也分享了李小龙的视频和照片,缅怀他的诞辰日

글로벌타임스는 “이소룡은 많은 사람에게 쿵푸스타 이상이었다”면서 “Bruce Lee was more than just a kung fu star to many,” said Global Times

《环球时报》评价说:“对于很多人来说,李小龙已经超越了功夫明星本身”,

“중국인에 대한 서구의 고정 관념을 깼고 여전히 많은 사람의 가슴 속에 있다”고 평가했다 “He changed the stereotypes about Chinese people in western countries and is still remembered by a lot of fans

“他打破了西方对中国人的固有观念,依然活在很多人的心中”

딸 섀넌 리(李香凝·51)는 부친의 영어 이름 ‘브루스 리’를 딴 웹사이트 ‘브루스닷컴’에 글을 올려 “아버지는 세상을 떠났지만 그의 삶은 계속해서 우리에게 새로운 영감을 주고 있다”고 회고했다 （no translation）

女儿香农?李(李香凝?51岁)在以父亲英文名字“布鲁斯?李”命名的网站“布鲁斯com”上发表文章回忆说,“虽然父亲去世了,但他的生活继续给我们带来新的灵感”

이소룡은 미국 샌프란시스코에서 태어나 생후 3개월 때 홍콩으로 이주했다 ” 　 Bruce Lee was born in San Francisco, the U S , and moved to Hong Kong when he was three months old

李小龙出生于美国旧金山,出生3个月时移居中国香港

성년이 된 후 23편의 영화에 출연하며 1970, 80년대 전 세계 남성들에게 대중문화의 아이콘으로 떠올랐다 He appeared in 23 movies and became an icon of popular culture among men all over the world in the 1970s and 1980s

成年之后他出演了23部电影,成为了20世纪70 80年代全世界男性大众文化的代表人物

불과 33세에 요절한 점도 세계적 팬덤 현상을 부채질했다 The global fandom grew even bigger when he died at the tender age of 33

33岁时夭折这一点也助长了世界粉丝团现象

미국 편에 서지 말라 압박하며 한한령은 풀지 않는 중오만왕이 중국 외교부장이 어제 2박3일의 방한을 마치고 귀국했다 China stays arrogant to maintain restrictions on Korean wave content Chinese Foreign Minister Wang Yi returned to his home country on Friday after spending three days in South Korea

傲慢的中国,施压韩国不要站在美国一边,却不解除“限韩令”中国外交部长王毅昨天结束了为期3天的韩国访问回国

왕 부장은 짧은 일정에도 한국 대통령부터 국회의장, 여권 실세까지 두루 만났다 Despite a short stay, he met with many important figures in South Korea, including its president, the speaker of the National Assembly, and the leading members of the ruling party

王毅在短暂的日程中,与韩国总统 国会议长 执政党实权人物一一会面

한중 외교장관회담에 25분이나 지각하는 결례도 범했다 He was late for a meeting with the South Korean foreign minister by 25 minutes

在韩中外长会谈中,他还犯了迟到25分钟的失礼行为

그의 방한을 계기로 양국 간엔 폭넓은 협력 방안을 논의됐다지만 중국의 한한령(限韓令·한류 금지령) 해제 같은 핵심 현안에서는 아무런 진전이 없었다 While broad plans for bilateral cooperation were discussed between the two countries during his visit, there was no progress made in key issues, including China’s restrictions on the Korean wave content

虽然以他访韩为契机,两国间讨论了广泛的合作方案,但在解除中国“限韩令”等核心问题上没有任何进展

왕 부장 방한 목적은 미국의 정권 교체기를 맞아 주변국 분위기 탐색과 외교적 관리를 위한 것 그 이상도 이하도 아니었던 듯하다 The purpose of Wang’s visit to South Korea seems to have been to gauge a neighboring country’s current atmosphere and for diplomatic management in the face of the U S ’ change of administration – nothing more, nothing less

王毅访韩的目的似乎完完全全为了在美国政权交替之际探索周边国家的氛围和外交管理

중국 측은 양국 간 합의라며 10개 항을 열거했지만 거기에 핵심 의제는 없었다 While the Chinese side listed 10 items as agreements between the two countries, the list did not include any key agenda

中国方面列举了10项内容,称之为两国之间的协议,但其中并没核心议题

왕 부장은 시진핑 주석 방한에 코로나19 완전 통제라는 조건을 내세웠다 Wang also said Chinese President Xi Jinping’s possible visit to South Korea would require the complete containment of the COVID-19 virus

王毅在习近平主席访韩事宜上提出了“完全控制新冠疫情”的条件

사드(THAAD·고고도미사일방어체계) 문제는 “적절히 처리해야 한다”며 철수를 압박했고, 그 보복 조치인 한한령 해제 요청에는 “지속적 소통을 희망한다”고만 했다 He put pressure on the Terminal High Altitude Area Defense (THAAD) issue by demanding its withdrawal while only responded to South Korea’s request to lift the restrictions on the Korean wave content, which was a retaliatory measure against the deployment of THAAD, by saying that he hopes for continuous communication

对于萨德(THAAD,末段高空区域防御系统)问题,他称“应该妥善处理”,并施压要求拆除,而对于要求解除其报复措施“限韩令”,他只是表示“希望持续进行沟通”

중국은 그간 미국 트럼프 행정부의 전방위 공세에 시달려왔다 China has been suffering all-around attacks by the Trump administration

中国一直受到美国特朗普政府全方位攻势的困扰

조 바이든 시대에도 그런 미-중 긴장은 계속될 것이라는 관측이 지배적이다 Such tensions between the U S and China are likely to continue under the Biden administration, according to many experts

大部分观测认为,在拜登时代,美中之间的这种紧张局面还会持续下去

중국으로선 미국의 동맹인 한국·일본이 반중(反中)전선에 서지 않도록 주변국 외교에 부쩍 공을 들이고 있다 China is putting a lot of effort into diplomacy with South Korea and Japan, which are the allies of the U S , so that the neighboring countries won't side with the anti-China stance

作为中国,为了不让美国的盟友韩国和日本站在反华战线上,正在致力于周边国家的外交

이번에도 새삼 한중일 경제통합을 강조하며, 미국의 중국 정보기술·기업 퇴출에 맞선 ‘글로벌 데이터안보 이니셔티브’에 동참해줄 것을 요청했다 Even during this visit, Wang emphasized economic integration among South Korea, China, and Japan and requested South Korea to join the Global Initiative on Data Security, which was set up to fight the U S ' bans on Chinese information technologies and businesses

此次,中方也再次强调韩中日经济一体化,邀请韩国加入针对美国驱除中国信息技术 企业的“全球数据安全倡议”

바이든 시대의 미중 관계는 아직 예단하기 어렵다 The U S -China relations under Biden’s lead is hard to predict

拜登时代的中美关系现在还很难断言

트럼프 대통령은 예외지만 선거 때 중국을 맹비난했던 미국 대통령들은 당선 후엔 모두 중국과의 통상 확대에 치중했다 Except for President Trump, all U S presidents who harshly criticized China during the election period focused on trade expansion with China once they took the office

虽然特朗普总统是例外,但选举时猛烈抨击中国的美国总统们在当选后都侧重于扩大与中国的贸易

규범과 가치를 앞세운 바이든식 국제주의는 트럼프 시절의 무역전쟁 같은 거친 방식이 아닌, 치밀한 제도적 포위망으로 중국을 옥죌 가능성이 높다 The Biden-style internationalism based on rules and values will exercise its influence on China with meticulous systematic plans, rather than the Trump-style trade war

以规范和价值为导向的拜登式国际主义很有可能不会采取特朗普时期贸易战等粗暴方式,而是通过严密的制度包围网来勒紧中国

‘민주주의 정상회의’ 같은 가치연대 구상은 한국을 더는 피하기 어려운 선택의 기로에 서게 만들 수 있다 A formation of a value-based initiative, such as a ‘democracy summit,’ may put South Korea at the crossroads of unavoidable choices

像“民主首脑会议”这样的价值同盟构想,会使韩国站在难以避免的选择的十字路口

왕 부장은 “이 세계에 미국만 있는 게 아니다”고 했다 “America is not the only nation in the world,” Wang said,

王毅说:“这个世界上不仅仅只有美国”

한국을 향해 미국 편에 서지 말라는 압박일 것이다 which means pressure on South Korea not to side with the U S

这可能是在向韩国施压,不要站在美国一边

동아시아에도 중국만 있는 게 아니다 However, China is not the only country in East Asia, either

东亚地区也不是只有中国

자국의 거대시장 접근을 제멋대로 막고 풀며 치졸한 외교를 계속하는 한 중국은 어디에도 설 자리가 없을 것이다 The country will lose its ground if it continues its shameful diplomatic tactics by controlling other countries’ access to the large Chinese market

中国只要任性阻止进入本国的巨大市场,同时继续推行拙劣的外交,就不会有立足之地

중국에서 누구든 방한할 때마다 ‘황제 칙사처럼 구느냐’는 얘기가 왜 나오는지 중국은 알아야 한다 China should reflect on why some say that Chinese officials visiting South Korea act like an envoy of an emperor

中国应该知道,为什么每次中国来人访问时,都会出现“耍皇帝敕使派头”的说法

한국 온 中왕이 “세계에 미국만 있는 게 아니다”Chinese Foreign Minister visits South Korea “America is not the only nation in the world

王毅访韩,“世界上不仅仅只有美国”

방한 중인 왕이(王毅) 중국 국무위원 겸 외교부장이 26일 “세계에 미국만 있는 게 아니다 （no translation）

正在韩国访问的中国国务委员兼外交部长王毅26日表示:“世界上不仅仅只有美国

190여 국가가 있고 모두 독립 자주 국가다 There are 190 countries and each of them is a sovereign nation

有190多个国家,都是独立自主的国家

한국과 중국도 그렇다”고 밝혔다 They include China and South Korea,” Chinese State Councilor and Foreign Minister Wang Yi said on Thursday during his visit to South Korea

韩国和中国也是如此”

한중 협력이 미국의 영향을 받아서는 안 된다는 점을 강조한 것으로 풀이된다 His comment seems to stress that cooperation between South Korea and China should not be affected by the U S

这可以解释为是在强调韩中合作不应受到美国的影响

시진핑(習近平) 중국 국가주석의 방한에 대해서는 신종 코로나바이러스 감염증(코로나19)이 “완전히 통제돼야 한다”고 밝혀 사실상 한국 정부가 추진해 온 연내 방한이 무산됐음을 시사했다 （no translation）

对于中国国家主席习近平访韩,他表示“新冠疫情必须得到完全控制”,事实上暗示韩国政府一直推动的年内访韩计划已经无望实现

왕 부장은 이날 오전 외교부 청사에서 강경화 외교부 장관과 회담을 마친 뒤 기자들과 만나 ‘이번 방한이 한국 정부와 여권 인사들에게 미국 편에 서지 말라는 미중 경쟁 차원인가’라는 질문에 이같이 말하면서 “한중은 이웃 국가이고 친척처럼 자주 오가야 한다”고 말했다 It was his answer to a question during a press conference after a meeting with South Korean Foreign Minister Kang Kyung-wha at the office of the foreign minister on Thursday morning asking whether his visit is a way to press the South Korean government and the ruling party members not to side with the U S when it comes to the competition between the U S and China “China and South Korea are close neighbors and should visit each other more often, like relatives,” he added

王毅当天上午在外交部大楼与韩国外交部长康京和举行会谈后,对记者提出的“此次访韩是否为了中美竞争,要求韩国政府和执政党人士不要站在美国一边”的问题,回答说:“韩中是邻国,应该像亲戚一样经常来往”

왕 부장은 “우리(한중)는 전략적 협력 동반자 관계이니 전방위로 조율하고 협력해야 한다”고도 했다 He also said that the two countries are strategic cooperative partners and should engage in comprehensive coordination and cooperation

王毅表示:“我们(韩中)是战略合作伙伴关系,应该全方位协调和合作”

그는 강 장관과의 회담 모두 발언에서도 “국제, 지역 문제에 대해 전략적 소통을 할 생각이 있다”고 밝혔다 During his statement at a meeting with Kang, he said he is willing to have strategic discussions regarding the international and regional issues

他在和康京和会谈的开场发言中表示:“我们打算就国际和地区问题进行战略沟通”

강 장관에게 한국에 배치된 사드(THAAD·고고도미사일방어체계)에 대한 우려도 표시했다고 외교부 당국자가 전했다 A source from the foreign ministry said that Wang expresses his concerns about the Terminal High Altitude Area Defense (THAAD) system deployed to South Korea

韩国外交部负责官员称,他向康京和对在韩国部署的萨德(THAAD,末段高空区域防御系统)表示了担忧

왕 부장은 ‘시 주석의 연내 방한 가능성이 얼마나 되느냐’는 질문에는 취재진을 가리키며 “다들 마스크를 쓰고 있다”며 “중요한 것은 (코로나19를) 완전히 통제하는 것”이라고 말했다 When asked about a possible visit to South Korea by Chinese President Xi Jinping, Wang pointed to the reporter, commenting on the face masks worn by them

对于“习近平主席年内访韩的可能性有多大”的提问,王毅指着采访团说:“大家都戴着口罩,重要的是完全控制(新冠疫情)”

한국에서 코로나19가 재확산되고 있어 시 주석 방한이 어렵다고 밝힌 것이다 “What’s important is to fully contain the COVID-19 virus,” he said, implying that the president’s visit is unlikely due to the resurgence of COVID-19 cases in South Korea

意思是说,韩国的新冠疫情正在再度扩散,因此习近平主席难以访韩

다시 찾아온 중국발 미세먼지 재앙 Ultra-fine dust from China blankets S Korea again

来自中国的可吸入颗粒物之灾再次到来,依靠“低姿态”外交,解决将遥遥无期

‘저자세’ 외교로는 해결 요원하다코로나19 사태 와중에 일시 진정되는 듯 했던 미세먼지가 다시 기승을 부리고 있다 South Korea, which saw a temporary reduction in fine dust levels amid the COVID-19 pandemic, is suffering from high levels of fine dust again

新冠疫情期间似乎一度平静的可吸入颗粒物再次活动猖獗

그제 서울에 올 가을 들어 첫 초미세먼지주의보가 발령됐으며 수도권과 충청권 등 중서부 지역은 12일부터 나흘 연속으로 초미세먼지 농도가 ‘나쁨’ 수준을 이어갔다 Ultrafine dust warnings were issued for the first time this fall in Seoul on Sunday and the levels of ultrafine dust remained “bad” for four days in a row from Nov 12 in the Seoul metropolitan and Chungcheong areas

前天在首尔发布了入秋以来首次超细颗粒物警报,首都圈和忠清地区等中西部地区12日起连续四天,超细颗粒物浓度持续为“糟糕”水平

충남지역은 14, 16일 이틀간 비상저감조치가 발령됐으며, 어제도 광주전남과 부산경남을 제외한 전국 대부분이 ‘나쁨’ 수준으로 공기가 나빴다 Emergency fine dust reduction measures were issued in South Chungcheong Province on Nov 14 and 16 and almost all parts of the country except for Gwangju and Busan had “bad” fine dust levels on Monday

忠南地区14日和16日两天启动了紧急降尘措施,昨天除光州 全南 庆南和釜山之外的大部分都空气不佳,处于“糟糕”水平

우리나라 미세먼지는 고농도 시 최대 80%가 중국에서 유입될 정도로 중국의 영향이 압도적이다 Up to 80 percent of South Korea’s fine dust particles come from China

我国的可吸入颗粒物处于高浓度时,80%都来自中国,中国的影响是压倒性的

최근 특히 심해진 것도 편서풍을 타고 중국에서 날아온 대기오염물질이 국내에서 대기정체로 쌓였기 때문이라는 게 환경당국의 분석이다 Environment authorities say a surge in fine dust concentrations in recent years is attributable to the atmospheric stagnation, which causes the accumulation of airborne pollutants brought in from China on winds from the west

据韩国环境部门分析,最近特别趋于严重,也是由于乘着偏西风从中国飞来的空气污染物停留在韩国大气中

코로나19 사태로 떨어졌던 중국의 공장가동률이 100% 가까이 회복된 데다 겨울철 난방이 시작되면서 중국에서 유입되는 오염물질의 총량이 다시 늘어난 탓이다 China’s factory utilization rate, which dropped amid the COVID-19 crisis, has recovered almost to 100 percent and the total amount of pollutants coming from China has increased again as people have started to turn on their heating

因新冠疫情一度降低的中国工厂的开工率接近恢复至100%,再加上开始冬季供暖,从中国流入的污染物质的总量再次增加

올 들어 9월까지 중국 전역의 미세먼지 농도가 평균 11. 8% 감소하는 등 중국 대기질이 일부 개선됐다고는 하지만 코로나19 사태로 인한 착시현상이었을 뿐 중국발 미세먼지 재앙은 여전히 한국인의 건강과 일상을 망가뜨리고 있다 China’s air quality is said to have improved somewhat this year with the fine dust levels across the country reducing by an average of 11. 8 percent until September As it turns out, however, it was only a temporary illusion created by COVID-19 and the lives and health of Koreans are still damaged by the fine-dust disaster

截至今年9月,全中国的可吸入颗粒物浓度平均下降了11.8%,为中国空气质量部分改善,但这只是新冠疫情带来的错觉现象,来自中国的可吸入颗粒物灾难仍然在破坏韩国人的健康和生活

지난해 봄 한반도를 덮친 최악의 미세먼지 사태를 겪으며 중국발 미세먼지 해결이 국가적인 당면 과제로 떠오르자 문재인 정부도 뭔가 근본적인 대책을 강구하는 듯 싶었다 When the issue of fine dust coming from China became an urgent national task after the national suffered from the worst fine dust concentrations in spring of last year, the Moon Jae-in administration seemed to be coming up with some fundamental measures

去年春天,肆虐韩半岛的最严重的可吸入颗粒物事件,来自中国的雾霾解决成为国家面临的问题,文在寅政府似乎在寻求某种根本对策

하지만 그후 2년 가까이 지나도록 뭐하나 진전을 이룬게 없다 But little progress has been made since then

但之后近两年,并没有取得什么进展

양국 환경장관회의와 실무자 협의를 1년에 한두 번 열어 대응책을 논의하고 있다지만 여기서 나오는 대책이라는게 정보교류나 학술연구 수준을 넘지 못하고 있다 South Korea and China are having environment ministers’ meeting and working-level talks once or twice a year but the outcomes were no more than information exchange or academic research

虽说一年里举行了一两次两国环境部长会议和实务磋商讨论对策,但这里出来的对策并没有超出信息交流和学术研究的水平

문재인 대통령은 지난해 미세먼지 사태로 민심이 들끓자 반기문 전 UN사무총장을 위원장으로 하는 범국가적 대책기구인 국가기후환경회의를 출범시켰지만 그후엔 별다른 진전이 없다 To be sure, there will be no ways to fundamentally block fine dust from China unless China’s industrial and environmental policies change dramatically

文在寅总统在去年可吸入颗粒物事件引起民心沸腾后,成立了由前联合国秘书长潘基文为委员长的全国性对策机构——国家气候环境会议,但之后没有取得任何进展

중국은 여전히 “서울 미세먼지는 현지에서 배출된 것”이라며 자국의 책임을 인정하지 않는데도 우리 정부는 공식 항의조차 못한채 미온적으로 임하고 있다 However, if China significantly strengthens the pollutant emissions standards for the plants on China’s east coast and makes bold investments on reducing the total amount of emissions, the amount of fine dust currently hitting Korea will be reduced greatly

中国仍然声称“首尔的可吸入颗粒物是当地排放的”,不承认本国的责任,韩国政府甚至连正式抗议也没有提出,进行了不咸不淡的处理

물론 중국의 산업·환경정책이 획기적으로 바뀌지 않는한 중국발 대기오염물질의 내습을 근본적으로 차단할 방책은 없을 것이다 하지만 중국이 동부 연안 공장들의 오염물질 배출 기준을 대폭 강화하고 배출총량 저감을 위한 과감한 투자에 나선다면 현재 한국을 덮치고 있는 미세먼지의 상당량이 줄어들 것이다 미세먼지 사태는 국민들이 숨을 쉴 수 있냐 없냐는 생존의 문제인 만큼 저자세 대응을 버리고 중국에 근본적인 미세먼지 저감 대책 마련을 강력하게 촉구해야 한다 Since the fine dust crisis is a matter of survival for Koreans, the South Korean government should abandon its submissive attitude and strongly urge China to come up with fundamental solution to reduce fine dust pollution

当然,如果中国的产业和环境政策没有发生划时代的变化,就不会有从根本上阻止来自中国的大气污染物的来袭但是,如果中国大幅加强东部沿海工厂的污染物排放标准,并果断投资降低排放总量,那么目前席卷韩国的可吸入颗粒物将大幅减少可吸入颗粒物事态是关系到韩国国民能否呼吸的生存问题,应该放弃低姿态应对,强烈敦促中国制定根本性的减少可吸入颗粒物的对策

文대통령, 中주도 RCEP 가입 서명Chinese Foreign Minister visits South Korea “America is not the only nation in the world

文在寅总统签署加入中国主导的RCEP协定

문재인 대통령이 15일 세계 최대 규모의 다자 자유무역협정(FTA)인 역내포괄적경제동반자협정(RCEP)에 최종 서명했다 President Moon Jae-in officially signed the Regional Comprehensive Economic Partnership (RCEP), the world’s largest multilateral trade agreement on Sunday

文在寅总统15日最终签署了世界最大规模的多边自由贸易协定——《区域全面经济伙伴关系协定(RCEP)》

문 대통령은 이날 청와대에서 화상으로 열린 ‘제4차 RCEP 정상회의’에서 RCEP에 서명하며 “코로나(신종 코로나바이러스 감염증)의 도전과 보호무역 확산, 다자체제의 위기 앞에서 젊고 역동적인 아세안이 중심이 돼 세계 최대 규모의 자유무역협정을 체결하게 됐다 President Moon signed the partnership at the fourth RCEP summit, which took place virtually, at the presidential office on the day “The world’s largest free trade agreement has been signed with young dynamic ASEAN as its center in the face of the challenges of Covid-19, spread of protectionism, and a crisis in multilateralism,” Moon said when signing the agreement

文在寅当天在青瓦台通过视频举行的“第四届RCEP峰会”上签署《RCEP》时表示:“新冠疫情的挑战 贸易保护主义的扩散 多边体制危机面前,以年轻 充满活力的东盟为中心,签署了世界最大规模的自由贸易协定

우리는 자유무역 가치 수호를 행동으로 옮겼다”고 말했다 “We have translated into action the protection of the value of free trade

我们把守护自由贸易价值付诸了行动”

RCEP는 한중일과 아세안 10개국, 호주, 뉴질랜드 등 15개국이 참여하는 세계 최대 규모의 FTA이다 ” The world’s largest free trade agreement comprises 15 countries, including South Korea, China and Japan, plus 10 ASEAN countries, Australia and New Zealand

RCEP是由韩中日 东盟十国 澳大利亚 新西兰等15个国家参与的世界最大规模的自贸协定

무역 규모, 국내총생산(GDP), 인구 측면에서 전 세계 약 30%를 차지한다 Their combined trade volume, GCP, and population account for a third of the world's total

从贸易规模 国内生产总值 人口方面来看,约占全世界的30%

가맹국 사이에서 관세 문턱을 낮추고 체계적인 무역·투자 시스템을 확립해 교역을 활성화하자는 것이 기본 취지다 The purpose of the pact is to bolster trade by lowering tariffs among its members and establishing a systematic trade and investment system

其基本宗旨是在协定成员国之间降低关税门槛,确立系统的贸易和投资体系,促进贸易

일각에선 사실상 중국이 주도하는 RCEP 가입을 계기로 미중 갈등 속 한국 정부의 외교적 셈법이 더욱 복잡해질 것이라는 관측이 나온다 Some critics say that with South Korea’s joining the economic partnership that is effectively spearheaded by China, Seoul will face more complex diplomatic affairs amidst tension between Washington and Beijing

也有分析认为,事实上,以加入中国主导的RCEP为契机,在中美矛盾中韩国政府的外交算法将变得更加复杂

버락 오바마 전 미 대통령이 중국의 팽창을 막기 위해 2010년부터 환태평양경제동반자협정(TPP)을 추진하자 중국은 이 포위망을 뚫기 위해 2012년부터 RCEP 구축에 나서며 한국 참여를 종용해 왔다 When former U S President Barack Obama pushed to form the Trans-Pacific Pact from 2010 in a bid to block China’s expansion, Beijing started process to form the RCEP in 2012 to dodge obstacles to trade, and would urge Seoul to join TPP ended up collapsing as U S

美国前总统奥巴马为了阻止中国的膨胀,从2010年开始推进环太平洋经济伙伴关系协定(TPP),中国为了突破这个包围圈,从2012年开始构筑RCEP,鼓励韩国参与

TPP는 2017년 다자 체제를 거부한 트럼프 대통령이 탈퇴를 선언하며 좌초됐지만 2018년 일본 호주가 주축이 돼 CPTPP로 이름을 바꿔 발효됐다 President Donald Trump, who denied multilateralism, declared withdrawal from the trade agreement in 2017 However the pact was replaced with the name “Comprehensive and Progressive Agreement for Trans-Pacific Partnership (CPTPP)” spearheaded by Japan and Australia in 2018

TPP在2017年因拒绝多边体制的特朗普总统宣布退出而搁浅,但在2018年以日本 澳大利亚为主轴,改名为CPTPP后生效

조 바이든 미 대통령 당선인은 후보 시절부터 CPTPP 복귀를 시사해 왔다 U S President-elect Joe Biden has been indicating that the U S will return to the CPTPP ever since becoming Democratic presidential candidate

美国当选总统拜登从候选人时期开始就暗示要回归CPTPP

이에 청와대 관계자는 “중국은 다른 나라와 마찬가지로 RCEP에 참여한 15개국 중 하나”라면서도 “필요하다고 느끼면 우리도 CPTPP에 들어갈 수 있다”고 했다 “Like other countries China is one of the 15 countries that have joined the RCEP,” said an official at the presidential office “If deemed necessary, South Korea could also participate in the CPTPP

对此,青瓦台相关人士表示:“中国与其他国家一样,是参与RCEP的15个国家之一”,“如果觉得有必要,我们也可以进入CPTPP”

블랙핑크 판다 맨손 터치 논란맹목적 애국주의를 앞세워 K팝 그룹 방탄소년단(BTS)을 공격했던 중국 누리꾼과 관영매체가 이번에는 걸그룹 블랙핑크에 대한 공격에 나섰다 ” Chinese netizens, media attack Black Pink members for touching panda Chinese netizens and state-run media would attack South Korean boy band BTS by promoting blank nationalism

BLACKPINK不戴手套触摸熊猫幼崽引发争议前段时间以“盲目的”爱国主义攻击K-POP组合防弹少年团(BTS)的中国网民和官方媒体,这次又开始攻击女子组合BLACKPINK

이달 3일 중국 희귀동물 판다와 접촉한 동영상을 자체 유튜브 등에 공개한 블랙핑크가 자신들이 원하는 수준만큼 판다를 귀중하게 다루지 않았다고 주장했다 Now, they have launched attacks on South Korean girl group Black Pink en masse

BLACKPINK本月3日在YouTube等社交网路媒体(SNS)上公开了与中国珍稀动物大熊猫接触的视频,她们主张没有像自己所希望的那样重视大熊猫

관영 영자지 글로벌타임스는 6일 “블랙핑크 멤버들이 한국 에버랜드에 있는 생후 약 3개월의 새끼 판다 ‘푸바오’, 2016년 한국에 온 ‘화니’를 접촉할 때 짙은 화장을 했다 때때로 장갑과 마스크도 끼지 않았다”고 주장했다 They claimed that Blank Pink, which released on YouTube and other channels a video showing the music band’s members coming into contact with the rare animal giant panda on Tuesday, did not treat the animals cautiously and valuably enough to satisfy them China’s state-run English daily Global Times said Friday, “Blank Pink members wore deep makeup when they touched three-month-old baby panda ‘Fubao “at the Everland theme park in South Korea, and ‘Huani’ which came to Korea in 2016

官方英文杂志《环球时报》6日表示:“BLACKPINK成员在接触韩国爱宝乐园出生约3个月的熊猫幼崽‘福宝’,以及2016年来到韩国的‘华妮’(韩国呼名:爱宝)时,都化了浓妆

이어 “새끼 판다는 면역력이 약해 짙은 화장을 하거나 방역 장비를 착용하지 않으면 위험할 수 있다”며 “판다는 중국의 ‘국보’이고 해외에서 태어나더라도 일정한 시기가 되면 중국으로 돌아와야 하므로 중국의 소유”라고 덧붙였다 They failed to wear gloves and facemask as well ” The daily went on to say, “Baby pandas lack immunity and they could be in danger if people (coming into contact) wear strong makeup or fail to use protective equipment The panda is China’s ‘national treasure’ and even if one is born overseas, it should return to China after a certain period of time, and they belong to China

有时连手套和口罩都不戴”接着,该媒体补充表示,“熊猫幼崽的免疫力很弱,如果化浓妆或不佩戴防疫装备的话,可能会很危险大熊猫是中国的‘国宝’,即使在海外出生,到了一定时期也要回到中国,所以属于中国”

일부 누리꾼은 웨이보에 “한국에 있는 판다를 회수하자”는 글을 올렸다 ” Some Chinese netizens posted Weibo comments, reading “Let’s bring back the pandas from Korea

部分网民在微博上发表文章称“回收韩国的大熊猫吧”

전 세계 동물원의 판다는 모두 중국이 임대해 주는 형태다 ” All giant pandas in zoos around the world are rented, rather than sold or granted, by the Chinese government

全世界动物园的大熊猫都是由中国租借的

중국야생동물보호협회는 5일 성명에서 “한국 아이돌 멤버가 국보 판다를 장갑도 끼지 않은 채 만진 사태를 묵과할 수 없다”고 주장했다 （no translation）

中国野生动物保护协会在5日的声明中主张:“韩国偶像组合成员在没有戴手套的情况下触摸了国宝熊猫,对此不能置之不理”

에버랜드와 블랙핑크 측에 각각 판다에 대한 불법 접촉 행위를 즉시 멈추고, 해당 장면이 나온 동영상을 삭제하라고 공식 항의했다고도 덧붙였다 When uploading a teaser of Black Pink’s video on its Instagram page,

该协会还补充说,已正式向爱宝乐园和BLACKPINK方面提出抗议,要求他们立即停止非法接触大熊猫的行为,并删除出现相关场面的视频

같은 날 에버랜드는 블랙핑크의 영상 예고편을 인스타그램에 게재하며 “본 촬영은 담당 수의사와 사육사의 감독하에 철저한 소독과 방역 후 진행됐다”고 공지했다 Everland said, “Filming was done after thorough decontamination and quarantine under supervision by the responsible veterinarian and zookeepers

同一天,爱宝乐园在Instagram上登载了BLACKPINK的视频预告片,并公告称“正式拍摄是在负责兽医和饲养员的监督下,经过彻底的消毒和防疫后进行的”

중국이 반발하자 이 영상을 삭제했다 ” But the theme park removed the clip after Chinese netizens responded negatively

在中国方面提出抗议后,爱宝乐园删除了该视频

현대차 “2025년까지 중에 수소트럭 3000대 보급”현대자동차가 중국에서 수소전기트럭 보급에 나서며 중국 수소경제 사업 확대에 속도를 낸다 Hyundai to supply 3,000 hydrogen trucks to China Hyundai Motor Company will supply hydrogen-electric trucks to China, accelerating its hydrogen economy business expansion in the country

现代汽车:“到2025年为止,将在中国普及3000辆氢电卡车”现代汽车将在中国普及氢电卡车,加速扩大中国氢能经济事业

현대차는 중국 수도 베이징이 있는 ‘징진지(京津冀)’ 지역과 경제 도시 상하이가 있는 ‘창장강(長江·양쯔강) 삼각주’ 지역의 기업들과 수소경제 생태계 구축을 위한 업무협약(MOU)을 연이어 체결했다고 4일 밝혔다 The South Korean automaker announced on Wednesday that it signed memorandums of understanding (MoUs) to build a hydrogen economy ecosystem with businesses in the Jing-Jin-Ji metropolitan region where capital Beijing is located and the Yangtze Delta where Shanghai is located

现代汽车4日表示,与中国首都北京所在的“京津冀”地区 经济城市上海所在的“长江三角洲”地区的企业接连签订了构建氢能经济生态系统的业务协议(MOU)

우선 현대차는 지난달 27일 상하이전력고분유한공사, 상하이순화신에너지시스템유한공사, 상하이융화전과융자리스유한공사 등 창장강 삼각주 지역 내 주요 에너지 및 금융회사와 MOU를 맺었다 Hyundai Motor Company first signed MoUs with major energy and financial companies in the Yangtze Delta on October 27

首先,现代汽车于上月27日与上海电力股份有限公司 上海舜华新能源系统有限公司 上海荣华融资租赁有限公司等长江三角洲地区内的主要能源及金融公司签订了MOU

수소충전소 및 수소생산설비 구축, 수소전기차 운영을 위한 금융 서비스를 추진해 2025년까지 이 지역에 현대차의 수소전기트럭 3000대 이상을 보급하는 게 목표다 The goal is to supply over 3,000 hydrogen-electric trucks of Hyundai Motor Company to the region by building hydrogen charging stations and hydrogen production facilities and launching financial services to support hydrogen-electric cars

其目标是,建立氢气加氢站及氢气生产设备,推进氢燃料电动汽车运营金融服务,到2025年为止,在该地区普及现代汽车3000辆以上的氢电卡车

이어 4일에는 중국강연집단 안타이과기고분유한공사 및 허강집단 허베이철강공업기술복무유한공사와 MOU를 체결해 징진지 지역에서 수소충전소 구축과 수소전기트럭 시범운행, 2025년까지 수소전기트럭 1000여 대 보급 추진에 뜻을 모았다 Later on Wednesday, the automaker signed MoUs with Antai Science and Technology and Hebei Steel Industrial Technology Service to build hydrogen charging stations, pilot hydrogen-electric trucks, and supply over 1,000 hydrogen-electric trucks by 2025 in the Jing-Jin-Ji metropolitan region

本月4日,现代汽车又与中国钢铁集团安泰科技股份有限公司及河钢集团河北钢铁工业技术服务有限公司签订了MOU,在京津地区构建氢气加氢站和示范运行氢电卡车,并计划在2025年之前普及1000多辆氢电卡车

이인철 현대차 상용사업본부장(부사장)은 “중국 시장에 수소 차량 판매뿐만 아니라, 수소차 리스, 충전소 운영 등 수소 생태계 전반에 걸친 사업 생태계를 구축할 계획”이라고 말했다 （no translation）

现代汽车商用事业本部长(副社长)李仁哲表示:“我们计划在中国市场构建不仅销售氢燃汽车,还运营氢气汽车租赁 加氢站等整个氢生态系统的事业生态系”

현대차는 스위스에서도 초기 수소전기트럭 구매 비용에 부담을 느끼는 물류업체들의 부담을 덜고자 사용료를 받고 차를 대여하는 형태로 수소트럭 보급에 나선 바 있다 （no translation）

现代汽车在瑞士初期也为了减轻对购买氢电卡车费用感到负担的物流企业的负担,以收取使用费租借车辆的形式来普及氢电卡车

중국은 수소전기차에 쓰이는 수소의 원료인 ‘부생수소’(석유화학 공정의 부산물)가 풍부하고, 지속적인 산업 기반 확충으로 트럭 수요가 꾸준한 시장으로 꼽힌다, 수소전기트럭 시장 성장 잠재력이 충분한 것이다 （no translation）

中国氢电汽车所用的氢原料“附生氢”(石油化学工程的副产品)非常丰富,由于持续扩充产业基础,卡车需求一直占据市场,氢电卡车市场发展潜力非常充分

중앙정부는 물론 지방정부 차원에서도 수소전기차 보급 확대, 수소 생산과 충전에 필요한 기반 구축에 속도를 내고 있기도 하다 Hyundai Motor Company supplied hydrogen trucks by lending them for fees to reduce the cost burden of logistics companies for initial purchases

不仅是中央政府,地方政府也加快了氢燃料电动汽车的普及 构建氢气生产和加氢所需的基础

2030년까지 수소전기차 100만 대 보급을 추진 중인 정부 목표에 발맞춰 지난달 현대차도 2030년까지 중국에 수소전기트럭 2만7000대 이상을 수출하겠다는 목표를 제시했다 Doosan Infracore reaches 200,000 mark for excavator production in China Doosan Infracore announced on Tuesday that the company’s number of excavators produced in China surpassed 200,000 units

为了配合政府提出的到2030年为止普及100万辆氢电汽车的目标,现代汽车上月也提出了到2030年为止向中国出口2.7万辆氢电卡车以上的目标

같은 기간 북미와 유럽으로의 각 1만2000대, 2만5000대 수출 목표를 능가한다 （no translation）

这超过了同期向北美和欧洲分别出口1.2万辆和2.5万辆的目标

두산인프라, 中서 굴착기 생산 20만대 돌파두산인프라코어가 중국 내 굴착기 누적생산 20만 대를 돌파했다고 3일 밝혔다 （no translation）

斗山工程机械在中国生产的挖掘机累计突破20万台斗山工程机械有限公司3日表示,在中国国内生产的挖掘机累计突破了20万台

1994년 10월 중국 진출 이후 26년 만에 달성한 기록이다 It is a record reached in 26 years since the company first entered the Chinese market in October 1994

这是公司自1994年10月进军中国市场后时隔26年达成的纪录，

중국 옌타이에 공장을 가지고 있는 두산인프라코어는 2001년 누적생산 5000대를 넘어섰다 Doosan Infracore whose plants are located in Yantai in China reached the 5,000 mark for the accumulated production of excavators in 2001

在中国烟台拥有工厂的斗山工程机械在2001年累计生产超过了5000台

당시 중국 시장은 일본의 건설기계 업체들이 선점하고 있었지만, 공격적인 마케팅과 최신 제품 출시로 2000년대 이후 중국 내 해외 건설기계 업체 부문 점유율 1위 기업으로 부상했다 The Chinese market was dominated by Japanese construction machinery producers at the time, but the South Korean company grew to secure the largest market share as a foreign company in the country’s construction machinery sector since the 2000s by engaging in aggressive marketing tactics and launching new products

当时,日本建筑机械企业抢先占领中国市场,但随着攻击性的市场营销和最新产品的推出,自2000年代以后,在中国的海外建筑机械企业领域占有率跃居首位

2000년대 중반부터는 연간 생산 1만 대를 넘겼고, 2010년에는 연간 2만 대 생산을 초과하며 성장해갔다 The company’s annual production of excavators surpassed 10,000 units in the mid-2000s and 20,000 units in 2010

从2000年代中期开始年产量超过1万台,2010年年产量超过2万台,并逐渐增长

이번 20만 대 누적생산 기록은 중국에 진출한 해외 건설기계 회사 중 최초다 The accumulated production record of 200,000 units is the first among foreign construction machinery producers in China

此次20万台的累计生产记录在进军中国的海外建筑机械公司中尚属首次

두산인프라코어는 중국 현지 기업을 제외한 굴착기 시장에서 3분기(7∼9월) 점유율 22. 8%를 기록했다 Doosan Infracore has 22.8 percent of market share in the Chinese excavator market, excluding domestic companies, as of the third quarter this year

除了中国当地企业,斗山工程机械在挖掘机市场的第三季度(7~9月)占有率达到了22.8%

미국의 건설기계 업체 캐터필라와 함께 이 시장의 점유율 1, 2위를 다투고 있다 The South Korean company is competing against American company Caterpillar Inc for the No. 1 spot in the sector

斗山工程机械目前正与美国建筑机械企业卡特彼勒(Caterpillar)就市场占有率第一 二的位置展开激烈竞争

두산인프라코어는 지난해 중국에서 건설기계 1만5000여 대를 판매했다 Doosan Infracore sold over 15,000 units of construction machinery last year in China

斗山工程机械去年在中国销售了1.5万多台建筑机械

올해는 신종 코로나바이러스 감염증(코로나19)으로 인한 시장 침체에도 불구하고 3분기까지 1만4348대를 팔아 지난해 실적과 맞먹는 성과를 거두고 있다 Despite the market slowdown due to COVID-19, the company has sold 14,348 units for the first three quarters of this year, which is comparable to its sales last year

今年,虽然因新型冠状病毒肺炎(COVID-19)疫情导致市场停滞,但截止到第三季度共销售了1.4348万台,与去年业绩几乎持平

두산인프라코어는 중국 굴착기 시장의 성장세에 맞춰 신제품과 특수장비들을 계속 출시할 계획이다 Doosan Infracore will continue to launch new products and specialty equipment in line with the growth trend of the Chinese excavator market

斗山工程机械为了适应中国挖掘机市场的增长趋势,计划继续推出新产品和特殊装备

또한 중국 내 지역사회 발전을 위해 낙후지역 청소년 학업 지원과 교육환경 개선 사회공헌 활동도 추진하고 있다 （no translation）

另外,为了中国地区社会的发展,还推进支援落后地区青少年学业和改善教育环境的社会贡献活动

BTS “한국전쟁, 한미 고난의 역사”에 中 생트집 BTS faces backlash from Chinese fans over Van Fleet Award speech

BTS表示“韩国战争是韩美苦难的历史”,中方吹毛求疵

세계적 케이팝 그룹 방탄소년단(BTS)이 수상소감에서 6·25전쟁을 언급했다가 중국에서 거센 비판을 받고 있다 6·25전쟁과 관련해 ‘양국(한국 미국)’만 언급한 점을 두고 ‘중국을 무시했다’며 발끈한 것이다 World-famous K-pop band BTS has come under fire in China for not acknowledging China alongside South Korea and the United States in its acceptance speech

世界级K-POP组合防弹少年团(BTS)在获奖感言中提到“6.25战争”,在中国受到了强烈的批评对于625战争只提到“两国(韩国 美国)”,中国有关方面怒气冲冲地表示这是“无视中国”的行为

BTS는 7일(현지 시간) 미국 비영리단체 코리아소사이어티가 주는 ‘밴플리트상’을 받았다 BTS was given the “Van Fleet Award” by The Korea Society

防弹少年团于当地时间7日获得了美国非盈利团体韩国社交协会颁发的“范弗里特奖”

이 상은 6·25전쟁에 참전한 제임스 밴플리트 미 8군 사령관을 기리기 위한 것으로 1995년부터 매년 한미관계 증진에 기여한 개인이나 단체에 수여된다 The U S non-profit organization has given to individuals and groups that promoted the U S -South Korean relations since 1995 to honor James Van Fleet, Commander of the U S Eighth Army who fought in the Korean War

该奖项是为了纪念参加6?25战争的美军第8集团军司令官詹姆斯?范弗里特,从1995年开始每年授予为增进韩美关系做出贡献的个人或团体

김대중 전 대통령, 이건희 삼성그룹 회장, 대한상공회의소 등도 수상한 바 있다 It has been awarded to former South Korean President Kim Dae-jung, Samsung Group Chairman Lee Kun-hee and the Korea Chamber of Commerce and Industry

前总统金大中 三星集团总裁李健熙 大韩商工会议所等也曾获奖

이 자리에서 BTS의 리더 RM(본명 김남준)은 수상 소감으로 “올해는 한국전쟁 70주년으로 우리는 양국(한미)이 함께 겪었던 고난의 역사와 많은 남성과 여성의 희생을 영원히 기억해야 한다”라고 말했다 “This year marks the 70th anniversary of the Korean War,” said RM, the leader of the South Korean group, accepting the award “We will always remember the history of pain that our two nations shared together, and the sacrifices of countless men and women ”

防弹少年团的队长RM(本名金南俊)在发表获奖感言时说:“今年是韩国战争70周年,我们要永远铭记两国(韩美)共同经历的苦难历史和众多男性和女性的牺牲”

이 소감이 뒤늦게 중국에 알려지면서 중국 언론과 누리꾼들이 발끈한 것이다 His remarks have angered Chinese media and Internet users

该感想晚些时候在中国传开,引起了中国当地媒体和网民的热议

12일 중국 관영 환추시보는 “수상 소감 중 ‘양국이 겪었던 고난의 역사’라는 부분에 중국 누리꾼들이 분노하고 있다”고 보도했다 The Global Times reported on Monday that Chinese online users were enraged by the mention of “the history of pain that our two nations shared together

12日,中国官方媒体《环球时报》报道说:“在获奖感言中,中国网民对‘两国经历的苦难历史’这一表述感到愤怒”

신랑왕(新浪網) 텅쉰왕(騰訊網) 등 유명 뉴스 포털사이트에서는 관련 댓글에 “한국전쟁 당시 중국 군인들의 고귀한 희생을 무시한 것”이라면서 “BTS의 수상 소감은 미국의 침략과 아시아에 대한 간섭을 무시하는 발언”이라는 의견까지 등장했다 ” Some commented that the Korean boy band dismissed the noble sacrifices made by Chinese soldiers during the Korean War and that the acceptance speech ignored the American invasion and Asia’s interference on popular news portal websites

新浪网 腾讯网等知名新闻门户网站甚至在相关回帖中出现了“无视韩国战争当时中国军人的宝贵牺牲” “防弹少年团的获奖感想是无视美国侵略和对亚洲的干涉的发言”的意见

중국은 6·25전쟁을 ‘항미원조(抗美援朝·미국에 맞서 북한을 도움) 전쟁’이라고 부르고 있다 The Korean War is called the “war against the United States to assist North Korea” in China

中国将6?25战争称为“抗美援朝(对抗美国帮助北韩)战争”

특히 미중 갈등이 고조되는 와중에 올해 참전 70주년을 맞아 민족주의 애국주의 영웅주의 등의 의미를 담은 ‘항미원조 정신’을 강조하고 있다 Amid escalating tensions between the United States and China, Beijing is emphasizing the “sprit of helping North Korea against the United States,” which implies nationalism, patriotism and heroism to commemorate the 70th anniversary of the war

尤其是在美中矛盾达到高潮的情况下,今年迎来参战70周年,强调了包含民族主义 爱国主义 英雄主义等意义的“抗美援朝精神”

BTS에 대한 중국 누리꾼들의 과격한 반응도 이 연장선에서 나온 것으로 분석되고 있다 This possibly explains the angry reaction of Chinese fans to BTS’s speech

据分析,中国网民对防弹少年团的过激反应也是出于这一原因

트럼프 “中 의존 영원히 끝낼것” 또 공격 Trump: U S will end its reliance on China once and for all

特朗普再次攻击中国,“将永远结束对中国的依赖”

미국과 중국의 경제 갈등이 재점화하고 있다 Trade conflict between the U S and China has been reigniting in recent weeks

美国和中国的经济矛盾再次激化

도널드 트럼프 미국 대통령은 중국이 미국과의 무역에서 얻은 돈을 군비 강화에 쓰고 있다고 비난하면서 “중국에 대한 의존을 끝내겠다”고 강조했다 U S President Donald Trump lashed out at China for spending the money it earned from its trade with the U S on military expansion “We will end our dependence on China,” stressed Trump

美国总统特朗普指责中国用从与美国的贸易中获得的资金强化军备,并强调,“将结束对中国的依赖”

이에 중국 왕이(王毅) 외교담당 국무위원 겸 외교부장은 자국 정보기술(IT) 기업들을 겨냥한 미국의 압박을 즉각 비난하면서 맞섰고 중국 관영매체는 중국이 보유한 미국 국채 매각 가능성을 언급하며 보복을 경고했다 In response, Chinese State Councilor and Foreign Minister Wang Yi immediately criticized the U S for putting Chinese tech companies under pressure and the Chinese state media warned of retaliation, mentioning a possibility of selling U S government bonds

对此,中国负责外交事务的国务委员兼外交部长王毅立即针锋相对地抨击美国打压中国的信息技术企业,中国官方媒体提及中国出售持有的美国国债的可能性,警告对美国进行报复

트럼프 대통령은 7일(현지 시간) 노동절 휴일을 맞아 진행된 언론 브리핑에서 “지금까지 중국만큼 우리(미국)를 뜯어먹은 나라는 없었다”면서 “중국은 우리가 준 돈을 군사력 강화에 쓰고 있다”고 말했다 At a press conference held on Monday (local time) marking the Labor Day, President Trump said, “There’s been no country anywhere at any time that’s ripped us off like China has,” adding that China spends that money on building their military

特朗普当地时间7日在为迎接劳动节假日而举行的媒体吹风会上说:“迄今为止,没有一个国家像中国那样占我们(美国)的便宜”,“中国把我们给的钱用在加强军事力量上”

그는 이어 “내가 우리 군사력을 강화해서 망정이지, 안 그랬으면 중국에 추월당했을 것”이라며 “그것이 디커플링(탈동조화)이든, 우리가 계속 해온 막대한 관세든 간에 우리는 중국에 대한 의존을 영원히 끝낼 것”이라고 말했다 “It’s very lucky that I’ve been building ours up because otherwise we’d be dwarfed right now by China,” said Trump “We’ll end our reliance on China, once and for all, whether it’s decoupling or putting in massive tariffs like I’ve been doing already

他接着说:“幸亏我加强了我们的军事力量,否则就会被中国超越”,“不管是脱钩,还是我们一直在做的征收巨额关税,我们将永远结束对中国的依赖”

최근 트럼프 행정부는 화웨이와 틱톡, 위챗 등 중국의 IT 기업들을 국가안보 위협으로 규정하면서 강력한 규제에 나서고 있다 ” The Trump administration has recently cracked down on Chinese tech companies, such as Huawei, TikTok, and WeChat, officially designating them as national threats

最近,特朗普政府把华为 抖音 微信等中国的信息科技企业定性为威胁国家安全,采取了强有力的限制措施

특히 미국은 중국의 대표적인 반도체 기업 SMIC를 거래제한 기업 리스트에 올리는 방안까지 검토하고 나섰다 In particular, Washington is considering placing SMIC, China’s No. 1 foundry company, on its export restriction list

特别是,美国正在研究将中国的代表性半导体企业中芯国际(SMIC)列入交易限制企业名单的方案

금융 분야에서는 미국의 회계 기준을 지키지 않는 중국 기업들을 미 증시에서 퇴출시키겠다는 방안을 발표하는 등 파상 공세를 이어가고 있다 The U S is launching a series of attacks also in the finance sector, announcing plans to delist Chinese companies that fail to comply with U S accounting standards

在金融领域,美国政府宣布了把不遵守美国会计标准的中国企业赶出美国股市的方案等,持续着波浪式攻势

이에 중국은 8일 왕 부장 주도로 ‘글로벌 데이터 안보’에 관한 이니셔티브를 발표하며 미국에 대한 견제에 나섰다 （no translation）

对此,中国8日在王毅的主导下发表了“全球数据安全”倡议,开始牵制美国

막연히 ‘안보 위협’이라고 비난할 게 아니라 구체적인 규칙과 표준을 제정해 따져 보자는 것이다 （no translation）

不是盲目地指责其为“安全威胁”,而应该制定具体的规则和标准来衡量

왕 부장은 “중국 정부는 중국 기업에 대해 다른 나라 법을 위반하면서 국외 데이터를 제공하라고 하지 않을 것”이라며 “일부 국가가 안전을 핑계로 선두 기업을 공격하는 것은 노골적인 횡포”라며 미국 정부를 정조준했다 （no translation）

王毅表示,“中国政府不会要求中国企业违反其他国家法律,提供国外数据”,“部分国家以安全为借口攻击领头企业,这是赤裸裸的蛮横行为”,将矛头指向美国政府

또 중국 관영 글로벌타임스는 최근 “중국이 미국 국채를 상당 부분 매각할 수 있다”고 보도했다 （no translation）

另外,中国官方媒体《环球时报》英文版最近报道说:“中国可以出售相当一部分美国国债”

현재 중국 정부가 보유한 미 국채는 1조 달러(약 1200조 원) 이상인데, 이를 8000억 달러까지 점진적으로 낮출 수 있다는 것이다 （no translation）

报道称,目前中国政府持有的美国国债在1万亿美元(约1200万亿韩元)以上,可以逐渐将其减持至8000亿美元

그러면서 양국의 군사적 충돌 같은 극단적인 상황에서는 모든 보유 국채를 내다팔 수도 있다고도 덧붙였다 （no translation）

报道还称,在两国发生军事冲突等极端情况下,可以抛售所有持有的国债

미국 국채의 최대 보유국인 중국이 미 국채를 대량으로 팔면 달러화 가치가 폭락하고 금리가 급등하는 등 국제 금융시장이 마비되는 상황이 발생할 수 있다 （no translation）

中国是美国国债的最大持有国,如果中国大量抛售美国国债,可能会出现美元价值暴跌 利率暴涨等国际金融市场陷入瘫痪的状况

중국과의 경제 관계를 끊어버리겠다는 미국의 엄포에 대응해서도 자구책을 마련하고 있다 For its part, Beijing is bracing for Washington’s threat to cut off economic ties with China

针对美国要断绝与中国的经济关系的恫吓,中国也在准备自救对策

글로벌타임스는 6일 ‘중국은 미국의 디커플링 시도를 상쇄할 방안을 찾고 있다’는 제목의 칼럼에서 “중국은 자국에 적대적인 나라에는 등을 돌리고 현재 추진 중인 일대일로(一帶一路) 정책에 따라 유럽 및 아프리카 아시아 나라들과 긴밀한 경제적 파트너십을 형성할 것”이라며 In a column, “Beijing ponders measures to offset U S decoupling attempt” in The Global Times on Sunday, the writer said China will turn its back on all unfriendly economies and will seek to form closer economic partnerships along the Belt and Road Initiative, particularly with the economics of the European Union, Asia, and Africa

《环球时报》6日在题为《中国正在寻找可以抵消美国脱钩计划的方案》的专栏中报道说:“中国将背弃对本国敌对的国家,根据目前正在推进的一带一路政策,与欧洲及非洲 亚洲国家形成紧密的经济伙伴关系”,

“트럼프 행정부가 미중 간 디커플링에 나선 만큼 중국은 스스로 자신의 운명을 개척해야 한다”고 보도했다 The column added that Beijing should “take its own destiny in its own hands”

“特朗普政府已着手美中之间的脱钩,中国应该自己开拓自己的命运”

(（additional translation））
as the Trump administration has embarked on its bid to decouple itself from China China retaliates U S by closing its Consulate in Chengdu The U S has announced that it will completely revise its engagement policy toward China, harshly criticizing Chinese President Xi Jinping as a “true believer in bankrupt, totalitarian ideology ”

（增译）中国报复,关闭美国领事馆,发动“全面战争”美国强烈指责中国国家主席习近平是“破产的极权主义的信奉者”,并宣布将全面修改对华包容政策基调

(（additional translation））The Chinese government instructed the closure of the U S Consulate General in Chengdu, Sichuan in retaliation of the U S ’ closure of the Chinese Consulate General in Houston

（增译）为了报复美国关闭中国驻休斯敦总领事馆,中国政府下令关闭美国驻四川省成都总领事馆

(（additional translation））It is deemed that the two countries’ relations are on the brink of severing for the first time since the two established diplomatic ties 41 years ago as the bilateral tensions that have arisen over the U S -China trade disputes, blaming each other for the responsibility of COVID-19, and China’s enactment of Hong Kong national security law are now unfolding as a full-on diplomatic war

（增译）有分析认为,随着美中贸易纠纷 新冠疫情责任攻防战 香港维护国家安全法的制定等,水平不断提高的中美矛盾升级为全面外交战,两国建交41年来首次面临断交之前的状况

(（additional translation））U S Secretary of State Mike Pompeo delivered a speech titled “Communist China and the Free World’s Future” at the Richard Nixon Library in Yorba Linda, CA on Thursday (local time) “

（增译）美国国务卿迈克·蓬佩奥当地时间23日在加利福尼亚约巴林达的尼克松图书馆发表了题为《共产国家中国与自由世界的未来》的演讲

(（additional translation））President Nixon once said he feared he had created a ‘Frankenstein’ by opening the world to the CCP, and here we are,” Pompeo said This implies that the U S ’s engagement policy toward China, which has continued for about 50 years since President Nixon’s visit to China in 1972, has contributed to the growth of the country as the biggest adversary threatening the Western world in modern times

（增译）他在演说中表示:“前总统理查德·尼克松曾说,‘我们担心我们是不是让中国向世界开放,制造了怪物',现在我们正处于这种状况”他的意思是,自1972年时任美国总统尼克松访问中国后,美国持续了约50年的对华包容政策,成为中国成长为威胁西方世界的最大敌国的契机

(（additional translation））“We have to keep in mind that the CCP regime is a Marxist-Leninist regime,” Pompeo continued “We, the freedom-loving nations of the world, must induce China to change,” he added, encouraging the U S ’s allies to join in putting pressure on China

（增译）随后,蓬佩奥表示,“有必要牢记中国共产党的本质是马克思和列宁政权”,“全世界热爱自由的国家应该诱导中国的变化”,要求同盟国共同参与对华施压

(（additional translation））Regarding the decision to shutter the Chinese Consulate General in Houston, he claimed that the consulate was the hub of China’s spying and stealing of intellectual property rights

（增译）对于关闭中国驻休斯敦总领事馆的决定,他声称:“因为这是中国间谍活动和知识产权盗窃行为的中心”中国外交部24日向驻中国美国大使馆通报称:“撤销美国驻成都总领事馆的设立和运营许可”,“美方必须停止驻成都总领事馆的所有业务和活动”外交部表示:“此次措施是对美国不理智行为的正当而必要的应对”,“也符合国际法 国际关系基本准则和外交惯例”他接着补充说:“中国并不希望看到美国和中国现在所处的状况”,“希望美国立即撤回错误的措施,为两国关系正常化创造必要条件”

“中어선, 동해 북한 수역서 오징어 5200억원어치 잡아들였다” Chinese ships catch $440 million worth of squid in N Korean waters

研究结果:“中国渔船在朝鲜东部海域捞走价值5200亿韩元的鱿鱼”

중국의 암흑선단이 유엔 제재를 받고 있는 북한의 동해로 몰래 들어가 약 2년간 불법 조업으로 5200억 원어치가 넘는 오징어를 남획했다는 인공위성 분석 결과가 공개됐다 The analysis of satellite images has been revealed to show that Chinese “dark fleets” have illegally caught 440 million U S dollars worth of squid in North Korea’s East Sea, which is under the United Nations (U N ) sanctions, for about two years

最新披露的人造卫星照片分析结果显示,中国的黑暗船队偷偷进入遭受联合国制裁的朝鲜的东部海域,约2年间非法捕捞了价值5200亿韩元以上的鱿鱼

암흑선단은 선박의 위치를 송출하지 않거나 공개된 모니터링 시스템에 나타나지 않는 무허가 불법 선박이다 Dark fleets refer to illegal and unlicensed ships that do not send their locations nor appear on a public monitoring system

黑暗船队指的是不发送船舶位置或没有出现在公开的监控系统中的无许可非法船只

한국인 데이터 과학자와 국제 비정부기구가 주도한 국제 연구가 밝힌 결과다 The findings have been achieved from international research led by South Korean data scientists and international non-governmental organizations

这是韩国数据科学家和国际非政府组织主导的国际研究表明的结果

중국의 불법 조업 선단 때문에 영세한 북한 어민이 더 위험한 먼바다로 밀려났다는 사실도 확인됐다 The research has also confirmed that small-scale fishermen were pushed further out into the ocean due to the illegal fishing vessels

经确认,由于非法的中国捕捞船队,零散的朝鲜渔民被逼到了更危险的远海去作业

비영리 민간연구단체 ‘글로벌어업감시’와 한국해양수산개발원, 일본수산연구교육기구, 미국 캘리포니아대는 2017, 2018년 북한 동해에서 중국 어선들이 이 같은 세계 최대 규모의 불법 조업을 벌였다는 인공위성 정밀 분석 결과를 국제학술지 ‘사이언스 어드밴시스’에 22일 공개했다 ○중국 암흑선단 추적하는 국제 공조 중국 정부는 수년째 자국 어민의 남획으로 각국 정부와 환경단체의 비난을 듣고 있다 그럼에도 중국은 아무런 조치를 취하지 않고 있다 Non-profit private research organization Global Fishing Watch, Korea Maritime Institute, Japan Fisheries Research and Education Agency (FRA), and the University of California have published the precision analysis results of satellite images in the journal Science Advances on Wednesday to demonstrate that Chinese vessels have conducted the world’s largest illegal fishing from 2017 to 2018

非营利性民间研究团体“全球渔业监视”和韩国海洋水产开发院 日本水产研究教育机构 美国加利福尼亚大学22日在国际学术刊物《科学》上发表了人工卫星精密分析结果,认为2018年在2017中国渔船在朝鲜东部海域实施了这一世界最大规模的非法捕捞○国际合作追踪中国黑暗船队中国政府数年来因本国渔民的滥捕而受到各国政府和环境团体的指责尽管如此,中国并没有采取任何措施

해양 보호단체인 오세아나, 비영리 위성정보 분석단체인 스카이트루스, 구글은 급기야 2016년부터 인공위성을 동원해 세계 바다를 운항하는 어선 3만5000척을 추적하는 ‘글로벌어업감시’ 프로젝트를 벌이고 있다 인공위성과 선박 정보를 이용해 남획을 일삼는 대형 어선을 추적하겠다는 의도다 박재윤 글로벌어업감시 수석데이터과학자를 포함한 연구팀은 2017, 2018년 북한의 배타적 경제수역에 진입한 오징어잡이 선박을 집중 감시했다 이들 선박 가운데 상당수는 중국 앞바다에서 활동하던 암흑선단이 남해를 거쳐 동해로 진출한 것으로 추정된다 A research team, including senior data scientist Park Jae-yoon at Global Fishing Watch, has focused their monitoring efforts on squid fishing ships that entered the exclusive economic zone (EEZ) of North Korea in 2017 and 2018 Many of them are suspected to be dark fleets operating on the coast of China that have entered the East Sea through the South Sea

海洋保护团体“海洋环境保护组织” 非营利卫星信息分析团体Skytrus 谷歌为形势所迫从2016年开始展开了“全球渔业监视”项目,动用人造卫星追踪35000艘航行在世界海域的渔船其意图是追踪利用人造卫星和船舶信息追踪滥捕的大型渔船包括“全球渔业监视”首席数据科学家朴在润(音译)在内的研究团队集中监视了2017年和2018年进入朝鲜专属经济水域的鱿鱼捕捞船他们中大部分是中国近海活动的黑暗船队,据推测是经由南部海域出入东部海域

하지만 이들을 지속적으로 추적 감시할 방법이 마땅히 없는 실정이다 However, there is no measure to consistently track and monitor them for now

但实际情况是,一直没有持续追踪和监视他们的方法

박 수석과학자는 e메일 인터뷰에서 “한반도 동해 북측 수역은 암흑선단 활동이 심각하지만 인접국 간 협력이 이뤄지지 않아 불법 어로 활동이 제대로 감시되지 않고 있다”며 “ “Dark fleets are very active in the East Sea within North Korean waters, however, their illegal fishing is not being properly monitored due to the lack of cooperation among neighboring countries," said Park

朴在润在电子邮件采访中表示:“虽然黑暗船队在韩半岛东部海域朝鲜水域的活动十分严重,但邻近国家之间没有开展合作,非法捕捞活动真正没有得到监督”,

인공지능(AI)과 여러 위성 데이터를 바탕으로 암흑선단의 조업을 종합적으로 밝혀낼 곳으로 동해를 선택했다”고 말했다 "We chose the East Sea as a place to reveal the comprehensive picture of dark fleets' illegal fishing based on artificial intelligence (AI) and satellite data

“以人工智能和各种卫星数据为基础,选择了把东部海域作为综合性查明黑暗船队捕捞活动的地方”

연구팀은 네 가지 위성 관측 기술을 조합해 어떤 환경에서도 불법 어선을 추적 감시할 수 있는 기술을 개발했다 " The research team has developed technologies that enable the tracking of illegal fishing vessels under any conditions by combining four different satellite observation technologies

研究团队结合四种卫星观测技术,开发出了在任何环境下都能追踪 监视非法渔船的技术

먼저 미국의 위성영상 서비스 기업 플래닛랩스가 보유한 군집위성을 이용해 두 척의 배가 그물로 어류를 포획하는 쌍끌이 어선을 찾아 AI를 이용해 식별했다 They first utilized a satellite constellation owned by Planet Labs, an American satellite video service provider, to identify pair trawling boats

首先,利用美国卫星影像服务企业“星球实验室”拥有的集群卫星,找到了两艘船用网捕获鱼类的双拖渔船,利用人工智能进行了识别

여기에 구름이 낀 날에도 어선을 찾고 추적할 수 있는 위성 레이더(SAR) 3기를 동원해 선박 크기와 위치, 이동 경로를 추적했다 （no translation）

此外,在多云天气里,还动员了3个可以寻找和追踪渔船的卫星雷达来追踪船只的大小 位置和移动路线

마지막으로 선박 이름과 속력 등 정보를 자동으로 수집, 추적해 충돌을 감시하는 선박자동식별시스템(AIS)을 통해 선박의 공식적인 움직임을 추적했다 （no translation）

最后,通过自动收集并追踪船舶名称和速度等信息,通过监视碰撞的船舶自动识别系统(AIS),追踪了船舶的正式动向

추적 결과 연구팀은 2017년 796척, 2018년 588척의 쌍끌이 어선을 찾아냈다 （no translation）

根据追踪结果,研究团队在2017年和2018年分别找到了796艘和588艘双拖渔船

연구팀은 대부분의 오징어잡이 어선이 밤에는 불을 켜고 오징어를 유인해 잡는다는 점에 착안해 고감도 적외선감지기(VIIRS)를 장착한 위성을 동원해 이를 추적하는 데 성공했다 （no translation）

研究团队着眼于大部分捕鱿鱼渔船晚上开灯引诱捕鱿鱼这一点,动员了装有高灵敏度的光红外成像辐射仪(VIIRS)的卫星,成功追踪了捕鱿鱼船

이런 방식으로 2017년에는 108척, 2018년에는 130척의 오징어잡이 선박을 찾아냈다 （no translation）

通过这种方式,2017年和2018年分别找到了108艘和130艘捕捞鱿鱼的船只

공동연구팀이 2년간 수집한 위성 정보를 분석해 찾아낸 중국의 불법 선박은 1600척이 넘는다 The joint research team has identified over 1,600 Chinese illegal fishing vessels by analyzing satellite images collected for two years

共同研究团队通过分析两年间收集的卫星信息,找到了1600多艘中国非法船只

잡아들인 오징어는 16만4000t으로, 금액으로 환산하면 4억4000만 달러(약 5263억 원)어치에 해당하는 것으로 추정된다 The amount of squid caught by them is about 164,000 tons, which is estimated to be worth approximately 440 million dollars

捕获的鱿鱼为16.4万吨,折合成金额相当于4.4亿美元(约合5263亿韩元)

공식적으로 가장 많은 오징어 어획량을 올린 일본과 한국의 전체 어획량을 더한 것과 맞먹는 양이다 This is almost equivalent to the sum of both South Korea’s and Japan’s squid catch, which officially have the largest squid catch records

这相当于正式鱿鱼捕获量最高的日本和韩国的整体捕获量

박 수석과학자는 “이런 규모의 불법 선단은 중국 전체 원양어선의 3분의 1에 달하는 규모”라며 “한 국가의 상업 선단이 다른 나라 수역에서 저지른 불법 조업 사례 중 가장 큰 규모”라고 말했다 （no translation）

朴在润表示:“这种规模的非法船队占中国全部远洋渔船的三分之一”,“在一个国家的商业船队在其他国家水域进行的非法作业案例中,规模最大”

○영세한 북한 어민은 먼바다로 밀려나 연구팀은 선체 길이가 10∼20m에 불과하고 전구 몇 개만 달고 조업하는 작고 영세한 북한 어선들이 러시아 연안에서 오징어를 잡고 있는 상황을 포착했다 （no translation）

○零散的朝鲜渔民被赶到了远海研究团队发现,船体长度只有10～20米,只有几个灯泡进行作业的小而零星的朝鲜渔船在俄罗斯沿岸捕获鱿鱼

2018년에만 이런 활동은 3000회 이상 포착됐다 （no translation）

仅在2018年,这种活动就发现了3000多次

이정삼 한국해양수산개발원 연구위원은 “길이가 50m에 첨단 장비로 무장한 중국 쌍끌이 어선과의 경쟁에 밀려 북한 어민들이 인근 러시아 해안까지 가게 된 것으로 보인다”며 “이들이 타고 있는 소형 목선은 작고 열악해 이처럼 먼바다로 나가는 데 적합하지 않고 위험하다”고 말했다 （no translation）

韩国海洋水产开发院研究委员李正三(音译)表示:“在与长50米 用尖端装备武装起来的中国双拖渔船的竞争中败下阵来,朝鲜渔民们似乎因此前往附近的俄罗斯海岸”,“他们乘坐的小型木船又小又恶劣,不适合前往这么远的大海,也很危险”

실제로 최근 북한 어선 수백 척이 러시아나 일본 해안을 표류하고 일부 어민들이 숨진 채로 발견되고 있는 것도 중국 어선들의 북한 수역 진출과 무관하지 않다는 게 연구팀의 분석이다 （no translation）

事实上,最近有数百艘朝鲜渔船在俄罗斯或日本海岸附近漂流,部分渔民被发现死亡,研究团队分析认为,这也与中国渔船进入朝鲜水域不无关系

2018년 러시아 해역에서 북한 어선의 어로 활동이 2015년에 비해 약 6배 늘어났다는 사실도 이번에 드러나 해가 갈수록 중국 암흑선단의 횡포가 극심해지고 있는 것으로 나타났다 （no translation）

2018年,朝鲜渔船在俄罗斯海域的捕捞活动比2015年增加了约6倍的事实,这一次也被曝光,表明中国黑暗船队的横行霸道越来越严重

박 수석과학자는 “중국의 대규모 상업 어선단 때문에 영세 어민이 피해를 받는 사례는 라이베리아 등 서아프리카에서도 발생하고 있다”며 “위성 데이터와 AI를 이용해 국가 어업감시기구에 기술을 지원하면 지속 가능하고 공정한 어로 활동을 제공할 수 있다”고 말했다 （no translation）

朴在润表示:“因为中国大规模商业渔船团而遭受损失的零散渔民的事例,在利比里亚等西非国家也有发生”,“如果利用卫星数据和人工智能向国家渔业监督机构提供技术支援,可以提供持续的 公正的捕鱼活动”

중국 암흑선단의 불법 남획으로 동해의 어류 자원이 고갈되고 해양 생태계가 파괴되고 있는 것도 문제다 （no translation）

由于中国黑暗捕捞船队的非法滥捕,东部海域鱼类资源枯竭 海洋生态系统被破坏,也成为问题

2003년 이후 한국과 일본의 오징어 어획량은 각각 80%와 82% 줄어든 상태로, 배후에는 중국의 불법 조업이 있는 것으로 추정된다 （no translation）

2003年以后,韩国和日本的鱿鱼捕获量分别减少了80%和82%,据推测其背后原因就是中国的非法捕捞

박 수석과학자는 “오징어와 같이 국가 간 경계선을 넘나드는 어종을 관리하려면 정보 공유가 중요하다”며 “역내 국가들이 데이터와 과학적 접근을 바탕으로 지역 어업을 협력적으로 관리할 수 있는 메커니즘을 만들길 기대한다”고 말했다 （no translation）

朴在润表示:“如果想要管理像鱿鱼一样跨越国家间界线的鱼种,信息共享非常重要”,“期待地区内国家能以数据和科学对待为基础,建立合作管理地区渔业的机制”

‘휴스턴 中총영사관 폐쇄’ 갈등 고조 U S -Sino tensions rise

“关闭中国驻休斯敦总领事馆”矛盾愈演愈烈

미국이 텍사스주 휴스턴 주재 중국 총영사관에 폐쇄 조치를 내린 것과 관련해 도널드 트럼프 대통령이 “중국 공관을 추가로 닫는 것은 언제든지 가능하다”고 말했다 following closure of Chinese Consulate in Houston Over the latest closure of the Chinese Consulate in Houston, the U S , President Donald Trump said it is always possible to close more Chinese missions

就美国对中国驻得克萨斯州休斯敦总领事馆采取关闭措施一事,美国总统唐纳德?特朗普表示:“任何时候都有可能追加关闭中国领事馆”

중국의 강력한 반발과 전 세계의 우려에도 물러서지 않고 오히려 추가 조치 가능성을 언급하면서 대중 압박 수위를 최고조로 끌어올린 것이다 Despite the strong opposition from Beijing and worries from the international community, the U S president has fueled such concerns, hinting at the possibility of further cranking up the intensity of crackdowns against China

美方不顾中方的强烈反对和全世界的忧虑,反而提及追加措施的可能性,将对华施压水平提升到了最高潮

트럼프 대통령은 22일(현지 시간) 백악관 브리핑에서 이렇게 밝히며 “우리가 폐쇄한 곳(휴스턴 주재 중국 총영사관)에서 불이 났다고 생각했고 모두가 ‘불이야’라고 했지만 내 생각에 그들은 서류와 문서를 태운 것 같다”고 말했다 중국 총영사관이 미국 내 불법 활동과 관련된 기록을 없애려 했을 것이라는 취지의 발언이다 “We thought there was a fire in the one that we did close and everybody said ‘There’s a fire There’s a fire ’ But I guess they were burning documents or burning papers and I wonder what that’s all about,” President Trump said during a White House briefing, insinuating that there may have been attempts to destroy evidence of China’s illegal activities at the Consulate building

特朗普总统当地时间22日在白宫举行的新闻发布会上如此表示,“我认为我们关闭的地方(中国驻休斯敦总领事馆)着火了,所有人都说‘着火了’,但我认为他们好像烧毁了文件和档案”言下之意就是,中国总领事馆可能想删除有关中方在美国国内进行非法活动的记录

스티븐 비건 국무부 부장관은 이날 상원 외교위원회가 미국의 대중 정책을 주제로 개최한 청문회에서 이번 조치가 트럼프 대통령의 지시에 따른 것이었다고 확인했다 During a Senate Foreign Relations Committee on America’s China policy on Thursday, Deputy Secretary of State Stephen Biegun said the closure was in compliance with Trump’s instructions

当天,美国副国务卿史蒂芬?比根在参议院外交委员会以“美国的对华政策”为主题举行的听证会上确认,此次措施是根据特朗普总统的指示进行的

비건 부장관은 “중국의 미국 기술 탈취와 지식재산권 침해 등 현안마다 이어진 분쟁이 이런 조치를 내리게 된 배경”이라며 중국을 조목조목 비판했다 Biegun criticized Beijing, citing a series of disputes stemming from China’s theft of American technologies and infringing on its intellectual properties as background of the measure

比根副国务卿对中国进行了逐一批评,他说:“中国抢夺美国技术 侵犯知识产权等每个悬案都接连发生纠纷,所以才会采取这样的措施”

주미 중국대사관은 성명을 내고 “미국의 주장은 근거가 전혀 없는 견강부회”라고 비판했다 The Chinese embassy in America issued a statement, calling America’s allegation a groundless sophistry

中国驻美大使馆发表声明批判称,“美国的主张是毫无根据的牵强附会”

차이웨이(蔡偉) 휴스턴 주재 중국 총영사도 ABC방송과의 인터뷰에서 “미국의 결정에 큰 충격을 받았다”며 “미국 일부 정치인은 입만 열면 거짓말하는 수작을 집어치워라”라고 원색적으로 비난했다 During an interview on ABC, Cai Wei, Consul General of China in Houston, said he was greatly shocked by America’s decision, pouring a raw criticism against American politicians who he accused are “habitual liars

中国驻休斯敦总领事蔡伟在接受美国广播公司(ABC)的采访时也毫不客气地批评道:“因美方的决定,受到了很大的冲击。美国某些政客总是在撒谎,请收起那套忽悠的把戏吧”

美-中홍콩갈등 폭발 ‘헥시트’ 문이 열린다

美中香港矛盾爆发,“退出香港(HK-exit)”大门开启

미국이 지난달 29일(현지 시간) 홍콩에 대해 국방물자 수출 중단 및 첨단 기술의 수출 규제에 나섰다 중국의 홍콩 국가보안법 강행 처리에 대응하기 위해 홍콩의 특별지위를 박탈하는 작업에 본격 착수한 것이다 ” US-China tensions escalate China passed the Hong Kong security law, and, in response, the United States ended arms exports to Hong Kong and restricted the territory’s access to hi-tech products on Monday (local time) as it moves to strip away the special status of the territory

当地时间5月29日,美国对香港采取了中断国防物资出口及限制尖端技术出口的措施这是为了应对中国强行处理香港《国家安全法》,正式着手剥夺香港的特殊地位

한동안 물밑으로 가라앉았던 미중 간 갈등이 다시 격화되고, 홍콩의 앞날은 격랑에 빠지게 됐다 Amid rising tensions between Washington and Beijing, Hong Kong’s future is as uncertain as ever

一度沉于水底的中美矛盾再度激化,香港的前途陷入激流

로이터통신 등에 따르면 윌버 로스 미 상무장관은 이날 성명에서 “수출 허가 예외 등 홍콩에 특혜를 주는 미 상무부의 규정이 중단됐다”며 “다른 (특혜) 조치를 폐지할지는 검토 중”이라고 밝혔다 “Commerce Department regulations affording preferential treatment to Hong Kong over China, including the availability of export license exceptions, are suspended,” Reuters reported quoting U S Secretary of Commerce Wilbur Ross “Further actions to eliminate differential treatment are also being eval‎uated

据路透社等媒体报道,美国商务部长威尔伯·罗斯在当天的声明中表示,“美国商务部关于出口许可例外等给予香港特惠的规定已经停止”,“正在讨论是否废除其他(优惠)措施”

홍콩이 중국에 반환됐던 상징적인 날(1997년 7월 1일)을 코앞에 두고 내놓은 조치다 ” This came one day before the 23rd anniversary of Hong Kong’s handover by the United Kingdom in 1997

这是在香港回归中国的象征性日子(1997年7月1日)即将到来之际出台的措施

마이크 폼페이오 국무장관도 이날 성명에서 “국방물자 수출을 중단하고 (군과 민간의) 이중 용도 첨단기술 규제를 중국과 마찬가지로 홍콩에 적용하는 절차를 시작할 것”이라고 했다 “The United States will today end exports of U S -origin defense equipment and will take steps toward imposing the same restrictions on U S defense and dual-use technologies to Hong Kong as it does for China,” US Secretary of State Mike Pompeo said in his statement

美国国务卿迈克·蓬佩奥也在当天发表的声明中表示:“将停止出口国防物资,启动将(军民)双重用途尖端技术限制与中国一样适用于香港的程序”

그는 “이제는 더 이상 통제 품목의 수출에 대해 홍콩과 중국 본토를 분리할 수 없다”고 덧붙였다 “We can no longer distinguish between the export of controlled items to Hong Kong or to mainland China

他还说:“现在,对于管制产品的出口,香港和中国内地不会再分开”

미국이 이번 조치를 시작으로 홍콩에 적용되던 관세 특혜 철폐 등을 포함한 특별지위의 전면 박탈에 나설 경우 글로벌 금융자본과 인력이 대거 홍콩에서 빠져나가는 ‘헥시트’(홍콩+엑시트)가 현실화될 것이라는 우려가 나온다 " Concerns are rising that the U S removal of Hong Kong’s special status which includes lower trade tariffs would trigger “Hexit,” a compound of Hong Kong and exit that describes a situation where global capital and human resources scramble out of the city

有人担心,如果美国以此次措施为开端,全面剥夺包括取消对香港适用的关税特惠等在内的特别地位,全球金融资本和人力大举从香港撤出的“退出香港”将成为现实

캐리 람 홍콩 행정장관은 “미국의 어떠한 제재도 두렵지 않다”고 반발했다 Hong Kong’s Chief Executive Carrie Lam said she did not fear any restrictions from Washington

香港特区行政长官林郑月娥则反驳说:“我们不怕美国的任何制裁”

중국은 미국의 전방위 압박에도 불구하고 이날 홍콩보안법 제정을 완료했다 Despite the pressure from the United States, China formally adopted the security law on the same day

中国不顾美国的全方位压力,当天完成了香港国家安全法的制定

홍콩 사우스차이나모닝포스트(SCMP)에 따르면 중국 전국인민대표대회(전국인대) 상무위원회는 참석자 162명 전원의 찬성으로 홍콩보안법을 상정 15분 만에 전격 통과시켰다 According to South China Morning Post (SCMP), the legislation was passed unanimously at the National People’s Congress Standing Committee 15 minutes after it was put to vote

据香港《南华早报》报道,中国全国人大常务委员会在全体162名与会者的赞成下,在递交15分钟后,迅速通过了香港国家安全法

홍콩 정부는 홍콩의 실질적인 헌법인 기본법 부칙에 이 법을 즉시 삽입해 홍콩 주권 반환일인 7월 1일부터 시행할 것으로 보인다 The new law will be added as a supplementary provision to the Hong Kong Basic Law, which is the territory’s de facto constitution, and is expected to come into effect on July 1

香港特区政府将及时将该法纳入香港实质宪法《基本法》附则,并从香港回归日7月1日起正式实施

국가 전복, 테러, 외국 세력과 결탁 등의 행위를 금지하는 홍콩보안법을 어기면 최대 종신형에 처해진다 Under the new security law, anyone who commits acts of secession, subversion terrorism or collusion with foreigners can be sentenced to a life sentence

如果违反禁止颠覆国家 恐怖主义 勾结外国势力等行为的香港国家安全法,将被处以终身监禁

반중(反中) 인사 재판에는 홍콩 행정장관이 특정 판사를 지명할 수 있도록 했다 （no translation）

在反华人士审判中,允许香港特区行政长官提名特定法官

홍콩보안법이 통과되면서 홍콩의 대표적 민주화 인사인 조슈아 웡 홍콩 데모시스토당 비서장(24)과 반중 성향 일간지 핑궈(빈果)일보 사주 지미 라이 회장(72)이 곧 체포될 것이라는 관측이 나온다 （no translation）

观察人士认为,随着香港国家安全法的通过,香港代表性民主化人士——香港“众志”秘书长黄之锋(24岁)和具有反华倾向的《苹果日报》社长黎智英(72岁)将被逮捕

코로나19가 왜 美-中대립의 속도를 높이나 Why COVID-19 further strains the U S -China relations

新冠疫情为何加快美中对立速度

신종 코로나바이러스 감염증(코로나19)은 인류가 직면한 매우 보기 드문 생물 안보 재난이다 The COVID-19 pandemic is a very rare biological and security disaster facing mankind

新冠疫情是人类面临的一次非常罕见的生物安全灾难

중국과 미국 양국에 공통의 위협이며 미중이 협력해야만 전 세계인들과 함께 코로나19에 승리할 수 있다 It is a common threat both to the U S and China, and the world will be able to rise above the COVID-19 crisis when the two powers join hands

这是对中美两国的共同威胁,只有中美合作,才能与全世界人民一道,战胜新冠疫情

하지만 코로나19 이후 미중 관계가 계속 악화되고 양국 관계가 ‘신냉전’에서 불과 한 발짝밖에 떨어져 있지 않아 보인다 The COVID-19 pandemic, however, is further straining the U S -China relations and the two countries appear to be only one step away from entering a new Cold War

但自新冠疫情暴发以来,中美关系持续恶化,两国关系似乎距离“新冷战”仅一步之遥

코로나19는 미중 관계를 더욱 악화시키고 있다 The COVID-19 pandemic has aggravated the U S -China relations

新冠疫情正在使中美关系进一步恶化

도널드 트럼프 미국 대통령 집권 이후 미국의 ‘대중국 정책 발언 시스템’에 역사적인 후퇴가 나타났다 There has been a great setback in the history of the U S government policy statements on China since President Donald Trump took office

美国总统特朗普执政后,美国的“对华政策发言系统”出现了历史性的倒退

트럼프 정부는 강하게 중국을 ‘악마화’해 왔다 The Trump administration has kept “demonizing China

特朗普政府一直将中国“妖魔化”

이런 ‘중국 악마화’는 트럼프 정부와 극우 공화당 세력이 보여준, 중국인들로서는 이해하기 힘든 이른바 ‘(중국에 당한) 피해자 콤플렉스’를 대표한다 ” This demonization represents a “victim complex” of the Trump administration and far-right Republicans that Chinese find hard to understand

这种“中国妖魔化”代表了特朗普政府和极右共和党势力所表现出来的 中国人难以理解的所谓“(因中国而)受害者情结”

이 콤플렉스는 3년여 동안 트럼프 정부 대중국 정책의 기본 기조가 됐다 This complex has been the cornerstone of the Trump administration’s policy toward China for the past three years

这一情结在3年多的时间里成为特朗普政府对华政策的基本基调

코로나19가 원래 미중 협력의 기회였음에도 유감스럽게도 트럼프 정부는 이른바 미국 이익 우선 정책을 더욱 강력하게 진행하면서 중국에 대해 높은 대립 정서를 표출했다 Rather than using the COVID-19 pandemic as a chance to collaborate with China, the Trump administration strongly pursued its “America First” policy, expressing resentment against China

新冠疫情本来是美中合作的机会,但遗憾的是,特朗普政府更加强硬地推行所谓的美国利益优先政策,对中国表现出高度对立的情绪

이는 중국을 압박하는 주요한 수단이 됐다 It has become a major means to put China under pressure

这已经成为向中国施压的主要手段

우선 미국 내 코로나19의 심각한 상황은 트럼프 정부와 미국 공화당 우익 정치세력들이 보여온 반중(反中) 피해자 콤플렉스를 더욱 히스테릭하게 변화시켰다 As many as 70 percent of Americans today think China should be held responsible for the spread of COVID-19 The pandemic has fueled the anti-Asian American racism and discrimination

首先,美国国内新冠疫情的严重状况使特朗普政府和美国共和党右翼政治势力所表现出的反华受害者情结变得更加歇斯底里

트럼프 정부는 코로나19를, 중국을 더욱 압박하고 정치 경제적으로 중국과의 관계를 청산하는 기회로 본다 （no translation）

特朗普政府把新冠疫情看作是进一步向中国施压 在政治和经济上同中国清算关系的机会

두 번째로 코로나19는 미국의 중국에 대한 우려를 격화했다 （no translation）

第二,新冠疫情加剧了美国对中国的担忧

트럼프 정부가 추진하는 디커플링(관계 단절) 방향의 중국 정책을 강화시켰다 （no translation）

强化了特朗普政府推进的“脱钩”方向的中国政策

미국인은 코로나19가 중국에 큰 기회의 창을 열었다고 여긴다 （no translation）

美国人认为新冠疫情为中国打开了一个大机会之窗

중국이 전 세계에 대한 영향력을 더욱 강하게 추구하고 미국의 세계 리더 지위를 밀어내려 한다고 여긴다 （no translation）

他们认为,中国试图更加强烈地追求对全球的影响力,并挤掉美国在世界的领导地位

중국에 대한 과학기술 전쟁, 무역 전쟁, 언론 전쟁, 심지어 앞으로 금융 전쟁까지 강화해 산업망, 공급망, 가치망을 중국에서 빼내는 것은 중국의 굴기를 억제하는 것이고, 미국의 지속적인 패권 우위를 유지하는 것으로 생각한다 （no translation）

他们认为,加强对中国的科技战争 贸易战争 媒体战争,甚至今后的金融战争,把产业链 供应链 价值网从中国挖走,就是在遏制中国的崛起,也是在保持美国持续的霸权优势

세 번째 코로나19는 트럼프 정부가 자유자재로 사용하는 중국 압박의 정치적 도구가 됐다 （no translation）

第三,新冠疫情成为特朗普政府恣意用来向中国施压的政治工具

중국을 압박하면 미국이 코로나19 초기에 보여준 무능하고 효과가 낮은 대응에 대한 미국인들의 원망을 중국으로 돌릴 수 있다 （no translation）

如果向中国施压,美国就能把对美国初期应对新冠疫情时表现出来的无能 低效方式的怨恨转嫁给中国

이뿐 아니라 트럼프가 ‘당신들은 중국인을 증오해야 한다 （no translation）

不仅如此,特朗普还能声称,“你们应该憎恨中国人

중국인이 오늘날 이런 두려운 국면을 만들었다’고 말할 수 있게 됐다 （no translation）

中国人造就了今天这样可怕的局面”

이는 이미 트럼프 대통령의 대선 전략의 중요한 부분이 됐다 （no translation）

这已经成为特朗普大选战略的重要组成部分

미국에서 중국 문제는 이미 트럼프 대통령에 의해 완전히 정치화됐다 （no translation）

在美国,中国问题已经被特朗普总统完全政治化

마지막으로 코로나19는 미국 사회의 반중(反中), 혐중(嫌中), 중국에 대한 공포 정서를 높였고 트럼프의 중국 압박 정책은 미국 내에서 더 많은 지지를 얻게 됐다 （no translation）

最后,新冠疫情加剧了美国社会的反华 厌华 恐华情绪,特朗普向中国施压的政策在美国国内得到了更多的支持

오늘날 미국 국민의 70%가 ‘중국이 코로나19 확산에 대해 책임을 져야 한다’고 여긴다 （no translation）

今天,70%的美国民众认为,“中国应对新冠疫情的扩散负责”

코로나19는 미국 내 아시아계 주민에 대한 인종주의 차별과 배척을 격화시켰다 （no translation）

新冠疫情激化了美国国内对亚裔居民的种族主义歧视和排斥

다수의 미국 정치 엘리트와 국민들이 중국 정책을 보는 견해는 코로나19의 영향을 받아 1950년대와 비슷한 ‘신(新)매카시즘(정치적 반대자를 공산주의자로 매도하는 태도)’으로 돌아가기 시작했다 The views of many political elites and Americans on the U S policy towards China are being influenced by the COVID-19 pandemic and returning to the “New McCarthyism” in the 1950s

多数美国政治精英和国民对中国政策的看法受到新冠疫情的影响,开始回到与20世纪50年代相似的“新麦卡锡主义(将政治反对者攻击为共产主义者的态度)”

이성적이고 온화한 중국 정책의 목소리는 계속 밀려나고 있다 Rational and gentle opinions on Trump’s China policy are often being pushed aside

理性 温和的中国政策声音不断受到排挤

미중 관계는 양국 모두 진지하고 이성적인 정책과 책략, 반성이 필요한 시점에 도달했다 It is high time that both the U S and China reflect on themselves and come up with sincere and rational policies and strategies

中美关系已经到了两国都需要认真 理性的政策与策略 反思的时刻

국제 체계에서 강대국의 흥망성쇠는 종종 피할 수 없는 강대국 간 격렬한 권력 경쟁과 전략적 대립을 가져온다 The rise and fall of superpowers inevitably bring about fierce power competition and strategic confrontation

在国际体系中,大国兴衰往往带来不可避免的大国间激烈的权力竞争和战略对立

하지만 21세기의 오늘날 미중의 어떤 ‘신냉전’의 앞날도 세계 안정과 평화와 번영을 해칠 것이다 But the future of “New Cold War” between the U S and China in the 21st century will only undermine the stability, peace, and prosperity of the world

但是,在21世纪的今天,中美的任何“新冷战”的前途,都将损害世界的稳定 和平与繁荣

더욱이 동북아 지역 경제 발전에 재난과 같은 충격을 가져올 것이다 Moreover, it will bring about a disastrous shock to the economic development of Northeast Asia

尤其是,它将给东北亚地区经济发展带来灾难般的冲击

최근 미국의 지미 카터, 빌 클린턴, 조지 W 부시, 버락 오바마 전 대통령이 함께 목소리를 내 트럼프의 대내외 정책을 호되게 비판했다 Former U S presidents including Jimmy Carter, Bill Clinton, George W Bush, and Barack Obama recently lashed out on Trump’s policies at home and abroad,

最近,美国的吉米·卡特 比尔·克林顿 乔治·W·布什 贝拉克·奥巴马等前总统一起发出声音,严厉批评了特朗普的内外政策

그리고 미국 정책의 ‘재난적 실패’의 근원을 반성하라고 요구했다 calling on President Trump to look back on the cause of his “catastrophic failure” of policies

他们还要求反省美国政策“灾难性失败”的根源

이와 마찬가지로 중국 정부 역시 코로나19 사태 과정에서 중국이 보여준 수많은 문제 가운데 정치 경제 개혁을 촉진하고 이미지 개선을 가속화하는 미래의 길을 찾아야 할 필요가 있다 For its part, the Chinese government first needs to promote political reforms and improve national image among other problems it has presented in dealing with the COVID-19 pandemic

同样,中国政府也有必要在新冠疫情过程中表现出的诸多问题中找到一条促进政治经济改革 加快形象改善的未来道路

이렇게 할 때만 중국과 미국 양국이 계속해서 세계 다수 국가의 이해와 존중을 얻을 것이다 Only then will the U S and China continue to be understood and respected by many countries in the world

只有这样,才能使中美两国继续得到世界上多数国家的理解和尊重

中외교부 “한반도 안정 희망” China’s foreign ministry urges North Korea to remain calm

中国外交部:“希望韩半岛稳定”……敦促朝鲜克制

北에 자제 촉구북한이 16일 오후 개성 남북공동연락사무소를 폭파한 것에 대해 주요 외신들은 일제히 속보로 보도하며 “한반도 내 긴장이 높아지고 있다”고 전했다 Foreign news outlets reported that North Korea destroyed an inter-Korean liaison office in Kaesong on Tuesday, saying that the tensions on the Korean Peninsula is elevating

对于朝鲜16日下午炸毁开城南北共同联络办事处一事,主要外媒一致通过快讯进行了报道,称“韩半岛内部的紧张气氛正在加剧”

중국 외교부는 “한반도의 평화와 안정을 바란다”며 북측에 자제를 촉구했다 China’s Foreign Ministry urged the North to control itself, saying, “We want peace and stability of the Korean Peninsula

中国外交部表示,“希望韩半岛的和平与稳定”,敦促朝鲜保持克制

AP통신과 CNN 방송, 뉴욕타임스(NYT), 아사히신문 등은 이날 오후 통일부의 발표를 인용해 북한이 연락사무소 청사를 폭파했다는 사실을 전하면서 이번 폭파는 ” The Associated Press, CNN, The New York Times and Asahi Shimbun quoted the announcement of the South Korean Ministry of Unification to deliver that North Korea blew up a liaison office

美联社 美国有线电视新闻网(CNN) 《纽约时报》《朝日新闻》等媒体当天下午引用韩国统一部的发言报导了朝鲜炸毁联络办事处的事实,

13일 김여정 북한 노동당 제1부부장이 한국 정부가 탈북단체의 대북 전단 살포를 막지 못한 것을 비난하며 예고했던 것이라고 덧붙였다 Kim Yo Jong, first vice department director of the Central Committee of the Workers' Party of Korea, said in a statement, criticizing the South Korean government was not able to stop leaflet drops of North Korean defector groups, the media outlets added

并指出,朝鲜劳动党第一副部长金与正13日在抨击韩国政府没能阻止“脱北者”团体撒放反朝传单时已经预告了这次爆破

AP통신은 일부 전문가를 인용해 한국이 미국 주도의 대북 제재로 경협을 재개할 수 없는 것에 대해 북한이 답답함을 토로하고 있다고 분석했다 The Associated Press analyzed that North Korea was expressing its frustration on being unable to resume economic cooperation with the South due to U S -led sanctions against North Korea

美联社援引部分专家的话分析说,对于韩国因美国主导的对朝鲜制裁而无法重启经济合作,朝鲜吐露了不快

NYT는 “최근 남북 간의 화해 무드를 끝내겠다고 위협해온 북한이 남한에 대한 불만을 극적인 방법으로 표시했다”고 전했다 The New York Times reported that North Korea displayed its anger against the South in a dramatic way after threatening to end the reconciliatory mood between the two Koreas

《纽约时报》报道说:“最近威胁要结束南北和解氛围的朝鲜用极端的方式表达了对韩国的不满”

워싱턴포스트(WP)는 북한이 최근 몇 주간 한국에 점점 더 날카로운 어조로 비판해왔다면서 연락사무소 파괴가 갈등을 급격히 증폭시킬 것이라고 전망했다 The Washington Post said Pyongyang’s rhetoric against Seoul has become harsher for the past few weeks and projected that the demolition would drastically increase conflicts

《华盛顿邮报》报道说,朝鲜最近几周对韩国提出了越来越尖锐的指责,并预测朝鲜破坏联络办事处会迅速激化矛盾

아사히신문은 연락사무소가 문재인 정부에는 대북 정책의 성과를 상징하는 것이었다며 큰 타격이 될 수밖에 없을 것이라고 내다봤다 Asahi Shimbun reported that the liaison office symbolized achievements of South Korean President Moon Jae-in’s North Korea policies and thus the destruction was bound to be a heavy blow

《朝日新闻》预测说,联络事务所对文在寅政府来说象征着对朝政策的成果,这必然会成为巨大的打击

자오리젠(趙立堅) 중국 외교부 대변인은 이날 정례 브리핑에서 북한의 연락사무소 폭파에 관련한 질문을 받고 “북한과 한국은 같은 민족”이라며 “중국은 이웃 국가로서 한반도의 평화와 안정 유지를 일관되게 희망한다”고 밝혔다 South and North Korea are part of the same nation, and that as a close neighbor, China has always wished for peace and stability on the peninsula, Chinese Foreign Ministry spokesperson Zhao Lijian said in a press briefing

中国外交部发言人赵立坚在当天的例行记者会上就朝鲜炸毁联络办事处一事表示:“朝鲜和韩国是同一个民族”,“中国作为邻国,一贯希望维护韩半岛的和平与稳定”

스가 요시히데(菅義偉) 일본 관방장관은 “계속해서 미국, 한국 등과 함께 긴밀히 협력하면서 필요한 정보의 수집, 분석을 실시하고 정세를 주시하는 한편으로 경계, 감시에 전력을 기울이고 있는 중”이라고 밝혔다 Japanese Chief Cabinet Secretary Yoshihide Suga said in a press conference on Tuesday that his country was continuously cooperating with countries such as the U S and South Korea over matters regarding the North, collecting and analyzing necessary information and closely monitoring the situation

日本官房长官菅义伟表示:“将继续与美国 韩国等紧密合作,收集和分析必要的情报,关注局势,同时全力进行警戒和监视”

“베이징 집단감염 원인 유럽 수입연어 가능성”중국 베이징(北京) 남부 신파디(新發地) 농수산물 도매시장에서 시작된 신종 코로나바이러스 감염증(코로나19) 재확산이 베이징 이외 지역으로 확산되고 있다 Imported salmon blamed for second coronavirus wave in Beijing The resurgence of COVID-19 from the Xinfadi wholesale market of Beijing is spreading across the city and beyond

北京疾控中心官员:“北京集体感染原因可能是欧洲进口鲑鱼”源自中国北京南部新发地农水产品批发市场的新冠疫情再次蔓延至北京以外地区

중국 정부는 유럽에서 수입한 연어 등에 코로나19가 묻어서 유입됐을 가능성을 제기하고 나섰다 The Chinese government is proposing the possibility that the salmon imported from Europe may have been responsible for the reentry of the virus

中国政府提出,从欧洲进口的鲑鱼等很有可能是沾有新冠病毒后流入中国

15일 중국 국가위생건강위원회에 따르면 14일 하루 동안 베이징에서 신규 확진자가 36명 발생했다 According to the National Health Commission, a total of 36 cases were confirmed in Beijing alone on Sunday

据中国国家卫生健康委员会15日透露,14日一天,北京新增确诊病例36例

11일 신파디 시장발 첫 환자가 발생한 이후 나흘 동안 베이징에서 총 79명의 확진자가 나왔고, 베이징 16개 구 가운데 8개에서 환자가 확인됐다 During the four days since the outbreak of the first case from Xinfadi on Thursday, a total of 79 patients have been diagnosed with COVID-19 in Beijing, and the eight out of the 16 districts within the city were found to have produced patients

新发地市场11日出现首例患者后,4天内北京共确诊79例患者,北京16个区中有8个区出现确诊患者

베이징시는 14일 시민 7만6499명을 대상으로 코로나19 검사를 진행하는 등 대대적으로 검사를 하고 있어 신규 확진자 수가 빠르게 늘어날 것으로 예상된다 With a massive testing scheduled on 76,499 citizens of Beijing, it is expected that the number of newly-affected patients will surge apace

北京市14日对76499名市民进行新冠病毒检测,进行大检查,预计新增确诊病例将快速增长

쑨춘란(孫春蘭) 국무원 부총리는 14일 “베이징 코로나19 확산 위험이 매우 크다”고 우려했다 “Beijing is exposed to a high risk of COVID-19 contagion,” said Sun Chunlan, the vice premier of the People's Republic of China, on Sunday

国务院副总理孙春兰14日担心,“北京新冠疫情扩散的风险非常大”

베이징 외에 허베이(河北)성의 바오딩(保定)시에서 신파디 시장 상인의 일가족 3명이 확진 판정을 받았고, 쓰촨(四川)성에서도 신파디 시장과 관련된 의심 환자 1명이 확인됐다 （no translation）

除北京外,新发地市场的河北省保定市商户一家三口确诊病例,四川也确诊了一名与新发地市场有关的疑似患者

중국은 유럽에서 수입된 수산물이나 육류에 바이러스가 묻어 중국으로 들어왔을 가능성을 제기했다 （no translation）

中国认为,从欧洲进口的水产品和肉类有可能沾上了病毒进入中国

베이징 질병예방통제센터 양펑(楊鵬) 주임은 이날 관영 중국중앙(CC)TV에서 “바이러스가 어떻게 왔는지 불확실하다”면서도 “유전자 서열 분석을 통해 (신파디 시장에서 발견된 코로나19) 바이러스가 유럽 쪽에서 온 것임을 확인했다”고 밝혔다 （no translation）

北京疾病预防控制中心主任杨鹏当天在中国官方媒体中央电视台表示:“病毒怎么来的还不清楚”,“通过基因序列分析,确认了(新发地市场上发现的新冠病毒)来自欧洲方面”

이어 “해외 코로나19 상황이 아직 심각해 (해외에서) 육류와 수산물을 처리하는 과정에서 (감염자의) 바이러스에 오염돼 수입됐을 가능성이 있다”고 말했다 （no translation）

他还说:“国外的新冠疫情还比较严重,(国外)在处理肉类和水产品的过程中,可能受到(感染者的)病毒污染而进口”

중국 전문가들은 코로나19 바이러스의 생존 능력이 저온에서 극대화되기 때문에 냉동 냉장 유통 과정에서 바이러스가 유입됐을 가능성이 충분하다고 주장한다 （no translation）

中国专家主张,由于新冠病毒的生存能力在低温条件下得到极大提高,因此病毒完全有可能在冷链流通过程中流入中国

중국 소셜네트워크서비스(SNS)에는 “또 해외 유입(에 책임을 돌리나)”이라는 비판과 “해외 (수산물, 육류) 가공 직원들이 마스크를 쓰지 않는 걸 봤다”는 주장이 동시에 나왔다 The public sentiment on social media cut both ways, with some criticizing the government for blaming imported salmon and others arguing to have witnessed unmasked employees handling fish and meat at the market

中国社交网站上出现了“又把责任推给海外流入吗”的批评之声,同时也出现了“看到海外(水产品 肉类)加工职员不戴口罩”的主张

“후베이(湖北)성 우한(武漢)에서 퍼졌던 바이러스 역시 유입된 것”이라는 음모론까지 다시 고개를 들었다 Conspiracies have been voiced again, claiming that the virus spreading across Wuhan had also come from outside

“在湖北武汉传播的病毒也是从境外流入”的阴谋论也再次抬头

美 “中보복땐 한국 위해 뭐든 할 준비 돼” U S promises to protect S Korea against any Chinese retaliatory action U S

美国:中国报复时,可以为韩国做任何事情

키스 크라크 미국 국무부 경제담당 차관은 11일(현지 시간) 한국이 반중(反中) 경제블록구상인 ‘경제번영네트워크(EPN)’나 화웨이 제재 등에 동참해 중국의 보복 조치에 직면할 경우 “미국은 한국을 돕기 위해 무엇이든 할 준비가 돼 있다”고 밝혔다 Under Secretary of State for Economic Growth, Energy, and the Environment Keith Krach said on Thursday (local time) that Washington is ready to do its utmost to keep Seoul intact in the face of any retaliatory action that Beijing may take if Seoul joins the U S -led Economic Prosperity Network (EPN) or sanctions against Huawei

美国国务院主管经济的副国务卿奇斯?克拉克11日(当地时间)表示,如果韩国加入参与和中国对抗的经济构想“经济繁荣网络(EPN)” “制裁华为制裁”等,届时如果中国采取报复措施,美国将尽一切努力帮助韩国

또 “전 세계가 중국의 위협과 보복에 맞서기 위해 일어서야 한다”며 동맹 및 파트너 국가들에 미국의 강경한 대중정책 동참과 연대를 요구했다 He called upon U S allies and partners to agree to anti-China policy and build unity, emphasizing the urgency for the international community to come forward to fight against the threats and retaliations by the Chinese government

此外,他还呼吁盟国及伙伴国家加入美国强硬的对华政策并联合起来,“全世界都应该站出来,以对抗中国的威胁和报复”

크라크 차관은 이날 인도, 브라질 등 5개 국가 주요 언론사들과 진행한 전화 간담회에서 미국의 대중 경제제재 및 정책 구상에 대해 설명하며 이렇게 밝혔다 With major media networks from five countries including India and Brazil in presence, a telephone press conference was held on Thursday where Under Secretary Krach elaborated Washington’s economic sanctions and policy framework regarding issues with China

克拉克副国务卿当天在与印度 巴西等5个国家主要媒体举行的电话座谈会上,就美国对中国的经济制裁及政策构想进行了说明,并做出了上述表示

한국 언론사 중에서는 동아일보가 유일하게 간담회에 참여했다 The Dong-A Ilbo was the only South Korean media outlet that was invited to the telephone press conference

本报是韩国媒体中唯一一家参加座谈会的媒体

그는 미국이 우방들에 ‘미국의 대중정책에 동참해 달라’고 요구한 것과 관련해 “중국이나 미국 중 한쪽을 선택하라는 게 아니다”며 “선택은 누구에게나 열려 있지만 결국 어느 쪽을 신뢰할 것이냐의 문제”라고 설명했다 As for the request by Washington for its allies to join U S policy toward China, the under secretary said that it is not a matter of choosing either Washington or Beijing, adding that everyone is entitled to a choice and at the heart of the issue is trust

他就美国要求友邦“共同参与美国对华政策”一事解释说:“不是在中国和美国中间作出选择虽然选择对谁都开放,但归根结底还是信任哪一方的问题”

크라크 차관은 “한국은 전 세계의 경제적, 기술적 파워하우스이자 미국뿐 아니라 전 세계적으로 큰 무역 파트너”라며 한국과의 경제협력을 강조했다 Under Secretary Krach stressed the importance of economic cooperation between the United States and South Korea, saying that South Korea is one of the world’s biggest economic and technological powerhouses as well as one of the major trade partners not only to the United States but also to the rest of the world

克拉克副国务卿表示“韩国是全世界的经济和技术力量源泉(PowerHouse),不仅是美国,也是全世界的大贸易伙伴”,强调了与韩国的经济合作

특히 삼성전자에 대해선 “세계 3대 5세대(5G) 관련 기업 중 하나이며 가장 발달한 반도체 생산업체”라고 높이 평가했다 Meanwhile, he highly commended Samsung Electronics for being one of the world’s top three 5G players and boasting off a high level of semiconductor production technology

特别他对三星电子给予了高度评价,称其为“世界三大5G相关企业之一,也是最发达的半导体生产企业”

美 “中에 함께 맞서자“ U S wants S Korea to stand by its side against China U S

美国:“一起对抗中国吧”……

EPN 참여 원칙과 실익 면밀 검토해야키스 크라크 미국 국무부 경제차관은 11일 중국에 맞서는 새로운 경제블록 구상인 경제번영네트워크(EPN)에 한국이 참여해줄 것을 요청하며 중국의 보복 조치에 직면할 경우 “미국은 한국을 돕기 위해 무엇이든 할 것”이라고 말했다 Under Secretary of State for Economic Growth, Energy, and the Environment Keith Krach on Thursday asked South Korea to join the Economic Prosperity Network (EPN), a U S -led economic bloc initiative against China

要仔细研究参与EPN的原则和实际利益美国国务院主管经济的副国务卿奇斯·克拉克11日要求韩国参与与中国对抗的新经济版图构想——经济繁荣网络(EPN),并表示,如果中国采取报复措施,“美国将尽一切努力帮助韩国”

그러면서 “중국·미국 중 선택하라는 게 아니다 In his words, the United States would do whatever it takes to help South Korea handle any retaliatory action that China may take

他还说:“不是在中国和美国中间作出选择

선택은 누구에게나 열려 있지만 결국 어느 쪽을 신뢰하느냐의 문제”라고 했다 Krach made it clear that it is not a matter of choice between the United States and China, emphasizing that everyone has their own choice but it all comes down to trust

虽然选择对谁都开放,但归根结底还是信任哪一方的问题”

크라크 차관의 발언은 그간 미국이 추진해온 EPN 구상과 화웨이 제재 등 중국 견제 정책에 한국이 적극 참여해야 한다는 요청이다 The gist of Under Secretary Krach’s request is that Seoul should be supportive of the EPN initiative and a series of anti-Huawei campaigns that Washington has pushed for

克拉克的发言是要求韩国积极参与其间美国推进的EPN构想和制裁华为等牵制中国的政策

EPN은 중국을 배제하고 미국 주도의 새로운 글로벌 공급사슬(GSC)을 구축하겠다는 구상이다 The EPN initiative aims to build a new U S -centric global supply chain with China out of the picture

EPN的构想是排除中国,构建由美国主导的新的全球供应链(GSC)

중국의 일대일로(一帶一路) 세력권 확장에 맞선 미국의 안보전략이 인도태평양전략이라면, EPN은 경제 차원의 중국 견제전략이다 The U S government has promoted an economic rival of China's One Belt One Road (OBOR), the pivot of growing Chinese influence across the world, whereas the Indo-Pacific Strategy has served as Washington's anti-China national security scheme

如果说美国针对中国“一带一路”势力范围扩张的安保战略是印度太平洋战略,那么EPN则是经济层面上的牵制中国战略

여기에 미국의 동맹으로서 한국이 참여해야 한다는 본격적인 압박인 것이다 Against the backdrop, South Korea is, in effect, being pressed into taking the U S side as one of its allies

在这里,还有韩国作为美国的同盟必须参与的大举施压

미국은 중국식 국가주의적 자본주의에 맞서 민주주의와 인권, 투명성, 지적재산권 보호 같은 가치를 공유하는 자유주의적 자본주의 국가 간 연대의 필요성을 강조한다 The United States reiterates how significant it is for liberal capitalist countries to unite to uphold democracy, human rights, transparency and intellectual property rights against China's nationalist capitalism

美国针对中国式国家资本主义,强调共享民主主义 人权和透明度 保护知识产权等价值的自由主义资本主义国家间联合的必要性

하지만 문제는 EPN 구상은 아직 전혀 익지 않은 과일로 보인다는 점이다 However, the point is that the EPN initiative still seems to be nothing more than unripe fruit

但问题是,EPN的构想看起来还不是完全成熟的水果

중국 배제 이외에는 어떤 구속력을 가진 형태가 될지, 그 협력 내용은 무엇인지 제대로 구체화된 게 없다 Not much of details has been specified as to in what way its binding force takes effect or in what field cooperation works, except the overarching principle of keeping China out

除了中国之外,没有具体说明会形成具有何种约束力的形态,合作内容又是什么

오직 ‘세계의 공장’이 된 중국을 배제하겠다는 미국의 전략적 판단만 두드러질 뿐이다 This only makes it all the clearer that the U S strategically schemes to ostracize China, a k a the world's factory, from the global supply chain

只是突显了美国要将已经成为“世界工厂”的中国排除在外的战略判断

참가국들에 대한 중국의 보복 우려에 대해 미국은 “뭐든 돕겠다”고 한다 Washington promises to participating countries, which are concerned about any retaliatory action that Beijing may take, that it will do whatever it takes to help them

对于中国对参加国进行报复的担忧,美国表示“将尽力提供帮助”

미국이 나선다면 중국 보복에 맞선 충분한 대항력과 보상도 가능할 것이다 Theoretically, they may be able to wield strong power against China's backlash and ensure sufficient compensation if the U S has their back

如果美国出面的话,韩国也可以充分对抗中国报复,并获得相应的补偿

하지만 EPN 참여는 중국의 보복에 따른 직접적인 피해는 물론 산업구조 전반의 변화를 요구하는 사안이다 However, involvement in the EPN initiative is likely to come with the direct damage caused by China's retaliatory measures and demand industry-wise structural change

但是参与EPN不仅是中国报复造成的直接损失,也是要求整个产业结构变化的事项

특히 우리에겐 중국의 사드(THAAD·고고도미사일방어체계) 보복 당시 제3자연하던 미국의 태도가 여전히 아픈 기억으로 남아 있다 What is hurtful to South Korea, in retrospect, is the attitude that the United States showed toward China’s retaliation against its ally regarding the Terminal High Altitude Area Defense (THAAD) issue

特别是对于韩国来说,中国报复“萨德”系统当时美国扮演第三者的态度,仍然是一个痛苦的记忆

미국이 내건 대로 자유와 민주의 가치를 공유하는 국가연대에 동맹국인 한국이 빠질 이유는 없다 Obviously, South Korea as an U S ally has every reason to take part in an international coalition to safeguard freedom and democracy as Washington puts it

正如美国所提出的那样,作为同盟国的韩国没有理由缺席共享自由和民主价值的国家连带

EPN을 현재 중국 의존도가 높은 수출과 생산을 다변화할 수 있는 기회로 만들 수도 있다 With the EPN in place, it may increase chances of diversifying export and production routes that are heavily dependent on China

也可以让EPN成为目前对中国依存度较高的出口和生产多元化的机会

그러나 우리의 최대 교역국이자 세계 최대 시장에 완전히 등을 돌리는 것은 가능하지 않고 However, it is a far-fetched plot for South Korea to turn its back on its No. 1 trade partner and the world’s largest consumer market

但是,作为韩国的最大贸易国和世界最大市场,完全背离是不可能的,

미국도 그렇게까지 요구하진 못할 것이다 Also, Washington will less likely push it to that level

美国也不会要求那样做

범정부, 나아가 산업계 의견을 모으면서 참여의 원칙과 수준, 실익, 국제적 동향을 면밀히 따져가며 단단히 준비해야 한다 It is time to collect opinions across the South Korean government and industries and make thorough preparations while pondering upon the rationality of joining the EPN initiative, depth of participation and global trends

要在广泛征求泛政府乃至产业界意见的同时,认真考虑参与的原则 水平 实际利益和国际动向,做好充分准备

한국 조선 수주 점유율, 中과 격차 좁혀 S Korea closes gap in shipbuilding order share with China

韩国造船订单占有率缩小与中国的差距

앞서 가던 중국의 조선 수주 점유율이 주춤하며 추격하던 한국과의 격차가 대폭 좁혀졌다 The gap between South Korea and China in terms of their shares of shipbuilding orders closed significantly as current leader China’s share slowed down

一度领先的中国造船订单占有率停滞不前,与追赶的韩国的差距大幅缩小

중국이 자국 물량을 쏟아 부으면서 인위적으로 수주량을 끌어올리던 시도가 한계에 다다랐기 때문이다 China’s manipulative efforts to increase order volume by pouring in its own domestic volume has finally reached its limit

因为中国试图通过倾注大量本国货单来人为拉高订单量的做法已经达到了极限

9일 영국 조선 해운 시황 분석기관인 클라크슨리서치에 따르면 지난달 전 세계 선박 발주량은 총 57만 CGT(표준화물선 환산 톤수)로 141만 CGT를 기록한 전월보다 40% 정도 감소했다 According to Clarkson Research, a shipbuilding and shipping market conditions analytic institution in the U K , on Tuesday, the total amount of global shipbuilding orders placed last month was 570,000 CGT, which is 40 percent less than last month’s 1,410,000 CGT

9日,据英国造船海运市场分析机构“克拉克森研究”公司透露,上个月全世界船舶订货量共计57万CGT(修正总吨),比创下141万CGT的上个月减少了40%左右

국가별로는 지난달 중국이 27만 CGT(13척, 47%)를 수주했고, 한국은 23만 CGT(8척, 40%), 일본은 5만 CGT(2척, 9%)를 수주했다 By country, China, South Korea, and Japan won 270,000 CGT (13 ships 47 percent), 230,000 CGT (eight ships 40 percent), and 50,000 CGT (two ships nine percent) last month, respectively

按国家来看,上个月中国承揽了27万CGT(13艘,47%),韩国承揽了23万CGT(8艘,40%),日本承揽了5万CGT(2艘,9%)

올해 수주 실적은 중국이 세계 1위를 달리고 있다 This year’s overall performance is headed by China

在今年的订单业绩中,中国位居世界第一

그러나 한국과 중국의 점유율 격차는 점차 줄어들고 있다 However, the gap between South Korea and China is closing in terms of order shares

但韩国和中国的占有率差距正在逐渐缩小

1∼5월 국가별 누적 수주 실적은 중국 288만 GCT, 한국 90만 CGT, 일본 49만 CGT다 The accumulated orders received from January to May are 2.88 million CGT, 0 9 million CGT, and 0.49 million CGT each for China, South Korea, and Japan

1至5月,国别累计订单业绩分别为:中国288万GCT,韩国90万CGT,日本49万CGT

4월 한국과 중국의 월별 수주 점유율은 55%포인트까지 차이가 났지만 지난달 월별 수주 점유율 차이는 7%포인트까지 줄었다 The monthly shares of orders in April showed a 55 percentage point difference between South Korea and China but last month’s gap dropped to seven percentage points

4月份韩国和中国的单月订单占有率差距达到55个百分点,但上个月单月订单占有率差距缩小到7个百分点

이는 중국의 자국 발주 물량이 대폭 감소했기 때문이다 as China’s domestic order volume decreased significantly

这是由于中国本国订货大幅减少

지난달 수주량의 경우 한국은 4월과 비슷한 수준을 유지했지만 중국은 전월 대비 73% 급감했다 For last month’s orders, South Korea has maintained a similar level as April but China’s declined 73 percent compared to the previous month

从上个月订单量来看,韩国与4月份基本持平,但中国比前一个月剧减73%

특히 지난달 중국의 수주량 중 85%는 자국 발주 물량이었지만 한국은 전부 유럽과 아시아 국가 선주들로부터 수주한 물량이다 In particular, 85 percent of orders received by China last month were domestic while South Korea’s orders were all from businesses in Europe and Asia

特别是,上个月中国的订单中有85%是本国订单,而韩国全部是来自欧洲和亚洲国家的船主订单

이에 업계에서는 꾸준한 수주를 하고 있는 한국이 하반기(7∼12월)에 중국의 수주량을 제칠 것으로 보고 있다 Industry experts believe that South Korea’s order volume will surpass China’s in the second half of this year

对此,造船业界认为,一直不断接到订单的韩国将在下半年(7～12月)超过中国的订单量

한국은 최근 카타르와 대규모 액화천연가스(LNG) 운반선 건조 슬롯 계약을 체결했고, 러시아와 모잠비크에서도 한국이 강점을 가진 대형 LNG 발주 프로젝트가 예정돼 있기 때문이다 South Korea recently signed a contract with Qatar to reserve slots to build large LNG carriers and Russia and Mozambique are scheduled to launch large LNG shipbuilding projects, in which South Korea has strengths

因为韩国最近与卡塔尔签订了大规模液化天然气运输船建造合同,俄罗斯和莫桑比克也预定了韩国具有优势的大型LNG订货项目

무역협회 “美中 홍콩갈등 격화땐 韓수출 타격” Worsened conflicts between U S and China over Hong Kong to undermine S Korea’s export

韩贸易协会:中美香港矛盾激化将打击

홍콩보안법 제정을 둘러싼 미국과 중국의 갈등이 심각해지면 홍콩을 중계무역 기지로 활용하던 우리 수출에도 타격이 불가피하다는 전망이 나왔다 As the tensions between the U S and China regarding the enactment of the Hong Kong National Security law grow, their impact on the export of South Korea, which uses Hong Kong as the hub of intermediate trade, seems unavoidable

韩国出口有预测称,如果美国和中国围绕制定香港保安法的矛盾变得严重,那么将香港作为中转贸易基地的韩国出口也将不可避免地受到打击

29일 무역협회 국제무역통상연구원에 따르면 홍콩은 우리 기업들이 중국으로 재수출을 하기 위해 이용하는 중계무역의 요충지다 Hong Kong is an important hub of South Korean businesses’ intermediate trade to reexport to China, the international trade and commerce research center of the Korea International Trade Association (KITA) reported on Friday

据韩国贸易协会国际贸易通商研究院29日透露,香港是韩国企业为再出口中国而利用的转口贸易要塞

홍콩은 중국 본토로의 접근성이 좋고 부가가치세 환급, 낮은 법인세, 각종 비과세 등의 세제 혜택, 뛰어난 무역 인프라를 갖추고 있기 때문이다 Hong Kong has easy access to mainland China and features various tax benefits, such as value-added tax refund, low corporate tax, and tax exemptions, as well as outstanding trade infrastructure,

因为香港对中国内地的接近性很好,并拥有增值税退税 低法人税 各种免税等税收优惠,以及出色的贸易基础设施

이에 한국→홍콩→중국으로의 물류 이동이 활발한 상태다 which is why logistics movement from South Korea to Hong Kong to China is very active at the moment

因此,从韩国到香港再到中国大陆的物流移动非常活跃

지난해에만 한국에서 홍콩으로 간 수출의 90% 이상이 다시 중국으로 들어갔을 정도다 Last year alone, over 90 percent of exports from South Korea to Hong Kong eventually went to their destinations in mainland China

仅在去年,韩国出口到香港的90%以上都再次流入中国

특히 미국은 1992년부터 홍콩에 대해 비자 발급과 투자 유치, 법 집행 등에서 특별무역지위를 부여해 대우를 해왔다 In addition, the U S has granted a special trade status to Hong Kong in terms of issuing visas, attracting investment, and enforcing laws since 1992,

值得一提的是,自1992年起,美国在签证 吸引投资和执法等方面赋予香港特别贸易地位,给予香港待遇

이는 홍콩이 아시아의 대표 금융 물류 요충지로 성장하는 데 중요한 역할을 했다 which has played an important role in the growth of Hong Kong as one of the major finance and logistics hubs in Asia

这为香港发展成为亚洲代表性金融物流要冲之地发挥了重要作用

그러나 미국의 대(對)홍콩 제재가 강화되면 각종 혜택이 사라지는 것은 물론이고 외국계 자본의 대거 이탈도 예상된다 However, once the U S strengthens sanctions against Hong Kong, the benefits mentioned above will disappear and a massive amount of foreign capital will leave the region as its advantages

但如果美国加强对香港的制裁,不仅各种优惠将消失,而且外资也将大举撤离

금융과 물류 허브로서의 각종 이점이 사라지기 때문이다 as a finance and logistics hub will be lost

因为其作为金融和物流枢纽的各种优势将消失

무역협회는 “이런 사태가 발생하면 중국으로 직접 수출을 할 수밖에 없어 각종 물류비가 증가하고 중국 직수출을 위한 항공편 확보 등에 차질이 생길 수 있다”고 전망했다 “Under such a circumstance, direct export to China is the only available option, which will drive logistics costs and issues in securing direct flights to China for export,” the KITA predicted

贸易协会预测称:“如果发生这种事态,只能直接向中国出口,因此各种物流费用增加,为直接出口中国而确保航班等也可能会出现问题”

무역협회 관계자는 “홍콩은 우리나라의 네 번째 수요 수출 국가로 중계무역 기지로서 가치가 높았는데 홍콩의 금융, 서비스, 물류 기능이 약화되면 수출에 타격을 받을 수밖에 없다”고 말했다 “Hong Kong is the fourth largest export destination of South Korea and offers values as an intermediate trade hub If Hong Kong’s roles in finance, service, and logistics weaken, impact on South Korea’s export will be inevitable,” said a member of the KITA

贸易协会某官员说:“香港是我国第四大需求出口地,作为转口贸易基地价值很高,如果香港的金融 服务 物流功能减弱,韩国的出口势必将受到冲击”

미중 갈등 속 中위안화 가치 급락 China lowers yuan amid conflicts with the U S

中美矛盾中人民币大幅贬值,无法避免流弹的韩国经济

유탄 피할 길 없는 한국경제중국 위안화 환율이 달러당 7위안을 넘는 이른바 ‘포치(破七)’가 일어났다 China’s currency yuan broke through the psychologically important level of seven yuan against the dollar

中国人民币汇率出现了1美元兑换超过7元人民币的所谓“破七”

중국의 중앙은행인 런민(人民)은행이 25일 달러대비 위안화 기준환율을 전날에 비해 0. 38%오른 7. 1209위안으로 고시한 것이다 The People’s Bank of China put the yuan fixing at 7. 1209 per dollar on Monday, an increase by 0. 38 percent from the previous day

中国央行——中国人民银行25日公布了人民币对美元汇率中间价报7.1209元,较前一交易日上涨0.38%

달러당 7위안은 심리적 저항선으로 여겨져 지난해 8월 미중 무역전쟁이 격화될 당시 ‘포치’가 발생하자 미국은 즉각 중국을 환율조작국으로 지정한 바 있다 The U S officially named China a currency manipulator in August when the yuan became cheaper than the seven-yuan-to-dollar threshold in the midst of the trade war between the two most powerful countries in the world

1美元兑人民币7元被认为是心理防线,去年8月中美贸易战激化时发生“破七”后,美国立即将中国列为汇率操纵国

중국이 위안화 환율을 올린 것은 무역전쟁에 이어 최근 홍콩보안법, 코로나19 발원지 갈등을 둘러싼 미국의 파상 공격에 대한 반격조치로 해석될 여지가 있다 China lowering its currency value could be interpreted as a measure against repeated attacks of the U S regarding the Hong Kong security law and the origin of COVID-19 as well as the trade war

中国提高人民币汇率可以解释为,继贸易战争之后,又针对最近美国在香港国安法 新冠病毒发源地矛盾上的波浪式进攻采取了反击措施

미국이 압박을 가해오면 중국은 위안화 환율을 높여, 즉 자국의 화폐가치를 낮춰 수출경쟁력을 높이는 방식으로 대응할 것이라는 관측은 늘 제기돼 오던 것이다 Many have projected that China would lower the yuan to increase its competitiveness in exports if the U S puts pressure on the country

一直有观测认为,如果美国施加压力,中国将提高人民币汇率,即通过降低本国货币价值来提高出口竞争力

물론 중국이 1000조 원에 이르는 대규모 경기부양을 위해 통화량을 늘리고 재정적자를 확대한다는 방침을 밝힘에 따라 위안화 가치가 자연스럽게 떨어진 측면도 있다 The deval‎uation partly comes from China’s announcement to increase the money supply and expand the fiscal deficit for large scale pump-priming worth 1,000 trillion won

当然,随着中国宣布将实施1000万亿韩元的大规模经济扶持,将增加货币量 扩大财政赤字的方针,人民币价值自然下跌,这一方面是存在的

양국 관계가 우호적일 때 서로 양해될 수도 있겠지만 거의 극단으로 치닫는 미중 갈등의 양상을 보건대 미국은 수출 확대가 절실한 중국이 적극적으로 평가절하를 유도했다고 볼 가능성이 없지 않다 It would be excusable if the relations between the two countries are amicable, but the U S is likely to see this as China’s deliberate effort to expand exports as the conflicts between the two countries are elevated to extremes

在两国关系友好时,双方可能会互谅互让,但从中美矛盾几乎走向极端来看,美国很有可能认为迫切需要扩大出口的中国积极引导了货币贬值

작년 8월 환율조작국 지정은 올해 1월 15일 1차 미중 무역합의가 이뤄지면서 해제됐다 The U S reversed its decision to brand China as a currency manipulator as the two countries made the first trade agreement on January 15

随着今年1月15日首轮中美贸易协议的达成,去年8月的指称汇率操纵国的问题被解除

이번 ‘포치’를 계기로 미국이 재차 중국에 대해 환율조작국 지정 카드를 꺼내들 경우 어렵게 체결한 무역합의는 휴지조각이 되고 미중 갈등이 환율이슈라는 민감한 국면으로 접어들 우려가 있다 If the U S re-designates China as a currency manipulator for this, the trade agreement, which was signed with difficulty, would not be worth the paper and the conflicts would elevate to a more sensitive issue of currency

人们担心,如果美国以此次“破七”为契机再次对中国亮出指定汇率操纵国的牌,好不容易签署的贸易协议将成为一纸空文,中美矛盾有可能进入被称为汇率问题的敏感局面

미중 환율전쟁의 유탄을 맞을 수 있는 대표적인 국가가 한국이다 South Korea is one of the countries that could bear the brunt of the currency war between the U S and China

韩国是可能受到美中汇率战争流弹的代表性国家

대외의존도가 높으며 특히 미중에 치우친 무역구조 때문에 불안한 환율은 우리 기업과 정부 모두에 상당한 애로요인이다 Unstable currency could spell troubles both to the government and businesses as it has high dependency on exports, especially to the U S and China

由于对外依存度高,尤其是偏向美中的贸易结构,不稳定的汇率对于韩国企业和政府来说都是相当困难的因素

당장은 위안화와의 동조현상에 따른 원화 약세로 수출경쟁력에 도움이 되겠지만 국내 자본시장에서 달러가 빠져 나가 금융시장이 흔들릴 수도 있다 For the moment, it could boost exports as Korean won would be depreciated along with the yuan But the financial market may falter if dollars evade the South Korean market

眼下,由于与人民币的同步现象,韩元的弱势会对出口竞争力有所帮助,但也有可能导致美元从韩国国内资本市场流出,金融市场发生动摇

그렇다고 해도 당장 뾰족한 대처방안도 있을 수 없다 But there is no other way to go around this

但即便如此,也并不存在马上有用的应对方案

정부는 외환시장을 면밀히 모니터링하면서 국제적으로 용인되는 범위 내에서는 적극적으로 개입하는 노력은 게을리 하지 않아야한다 The South Korean government should monitor the foreign-exchange market meticulously and make every effort to intervene as long as it is globally acceptable

政府应该密切监控外汇市场,毫不懈怠地在国际上允许的范围内积极介入外汇市场

이런 어려움이 닥칠 때를 대비해서라도 평소에 기업의 대외 경쟁력과 재정건전성 확보를 통해 외부로부터의 파고(波高)를 막아낼 방파제를 튼튼하게 구축해야 한다 It should build robust seawalls to ward off external waves by securing global competitiveness and financial health to prepare for the difficult times

哪怕是防范这种困难的来临,也必须通过在平时确保企业的对外竞争力和财政健全性,构筑起阻断外部风浪的防波堤

‘기업인 신속통로’ 이용 삼성전자-SK이노 등 중에 인력 550여명 파견삼성전자 등 국내 주요 기업들이 21, 22일 이틀 동안 ‘한중 기업인 신속통로(입국 절차 간소화)’ 제도를 통해 550여명의 인력을 중국에 파견했다 S Korean companies send around 550 employees to China Major South Korean companies, including Samsung Electronics, have sent over 550 employees to China on Thursday and Friday through the “South Korea-China quick path for businesspeople” system

三星电子和SKInnovation等利用“企业人绿色通道”向中国派遣550多名人力三星电子等韩国国内主要企业21 22日两天内通过“韩中企业人绿色通道(入境程序简化)”制度向中国派遣了550多名人力

신종 코로나바이러스 감염증(코로나19) 확산으로 지연됐던 중국 내 공장 증설 등의 프로젝트가 빠르게 재개될 것으로 보인다 Projects in China that have been suspended due to the COVID-19 outbreak, such as plant capacity expansion, are expected to be resumed quickly

因新型冠状病毒感染症(COVID-19病毒)疫情扩散而被推迟的中国国内工厂增设等项目有望迅速重启

삼성전자는 22일 중국 시안(西安) 반도체 제2공장 증설을 위해 필요한 본사·협력업체 기술진 300여 명을 전세기 편으로 파견했다 Samsung Electronics has dispatched over 300 technical employees of its headquarters and partners on a chartered plane for the capacity expansion of its semiconductor plant 2 in Xi'an, China

三星电子22日向中国西安派遣了300多名总公司?合作企业技术人员,在当地增设半导体第二工厂

이 비행기에는 시안에서 배터리 공장을 운영하고 있는 삼성SDI의 인력 30여 명도 함께 탑승했다 Over 30 employees from Samsung SDI, which s operating a battery plant in Xi'an, were also on board

在西安经营电池工厂的三星SDI的30多名人力也一同乘坐了该飞机

이재용 삼성전자 부회장이 코로나19 사태 이후 첫 해외 출장지로 시안을 다녀온 뒤 3일 만에 이뤄진 조치다 Such a measure was implemented three days after Samsung Electronics Vice Chairman Lee Jae-yong visited the city on his first overseas business trip since the COVID-19 outbreak

这是三星电子副会长李在镕自新冠肺炎疫情后首次海外出差造访西安后,时隔3天采取的措施

기업인 신속통로는 기업인 등에 한해 출국 전후 각각 코로나19 검사를 받고 음성 판정이 나오면 현지에서 14일 의무격리를 면제해주는 조치다 The quick path system for businesspeople grants a 14-day quarantine wavier for businesspeople who test negative for COVID-19 both before departure and after arrival

企业人绿色通道是指,仅限于企业人,在出国前后分别接受新冠肺炎检查,一旦出现阴性判定,在当地免除14日义务隔离的措施

삼성전자가 이 제도로 대규모 인력을 해외 현장에 파견한 것은 이번이 처음이다 This is the first time the South Korean electronic giant used the system to send a large number of employees overseas

这是三星电子首次通过该制度向海外大规模派遣人力

4월 시안에 200여 명의 인력을 파견할 때는 이 제도가 생기기 전이라 ‘특별 입국’을 요청해 격리 조치 없이 인력을 파견했다 When the company dispatched around 200 employees to Xi'an in April before the introduction of the system, it requested “special entry” to bypass quarantine

4月份向西安派遣200多名人力时,由于该制度尚未出台,所以要求“特别入境”,在没有采取隔离措施的情况下派遣人力

시안 공장은 삼성전자의 유일한 해외 메모리 반도체 생산 기지로 총 150억 달러(약 18조4500억 원) 규모의 투자가 진행되고 있다 The Xi'an plant is the only overseas memory semiconductor manufacturing hub of Samsung Electronics with a total of 15 billion dollars of investment underway

西安工厂是三星电子唯一的海外存储半导体生产基地,总投资规模达150亿美元(约18.45万亿韩元)

기아자동차도 이날 전세기 편으로 중국 옌청(鹽城) 공장에 인력 100여 명을 파견했다 Kia Motors has also dispatched around 100 employees to its plant in Yancheng,

起亚汽车当天也乘坐包机向中国江苏盐城工厂派遣了100多名人力

SK이노베이션은 앞서 21일 옌청 배터리 공장 건설 현장에서 근무할 인력 120여 명을 전세기로 보냈다 China on a chartered plane while SK Innovation has sent about 120 employees on Thursday who will work in the construction site of the company’s Yancheng battery plant

此前,SKInnovation于21日用包机派遣了120多名今后会在盐城电池工厂建设现场工作的人员

백악관 “中은 약탈경제” 新냉전 선포도널드 트럼프 미국 행정부가 중국을 상대로 한 미국의 향후 전략 및 정책 방향을 담은 보고서를 공개했다 White House attacks China’s ‘predatory economic practices’ The U S government led by President Donald Trump has released a report on the country’s future strategy and policy directions regarding China

白宫称“中国是掠夺性经济”,宣布新冷战美国特朗普政府公开了一份报告,内容包括美国今后对中国的战略及政策方向

중국과의 협력이 아닌 공개 압박, 사실상의 중국 봉쇄 등 ‘경쟁적 접근(competitive approach)’을 하겠다는 점을 분명히 해 사실상 ‘신(新)냉전’을 선언했다는 평가가 나온다 The report is deemed as a practical announcement of a “new cold war” as it makes it clear that a “competitive approach” will be adopted for China, such as exercising public pressure and practically containing the country, rather than cooperation

有评论认为,这一文件事实上宣布了“新冷战”,明确表示将采取公开施压而不是与中国合作,事实上对中国进行封锁等“竞争性对待(competitiveapproach)”

워싱턴포스트(WP)는 백악관이 20일(현지 시간) 국방부 초안을 바탕으로 작성한 ‘미국의 대중국 전략 보고서’를 의회에 제출했다고 보도했다 The White House submitted a report titled “United States Strategic Approach to The People’s Republic of China” to Congress on Wednesday, The Washington Post said

《华盛顿邮报》报道说,白宫当地时间20日向国会提交了以国防部草案为基础编写的《美国对华战略报告》

백악관이 홈페이지에 공개한 16장 분량의 보고서는 “중국의 근본적인 경제 개혁 및 정치적 개방에 대한 기대는 실패로 끝났다 The 16-page report diagnosed that “a hope that deepening engagement would spur fundamental economic and political opening in the People’s Republic of China (PRC)” has failed and

白宫在其网站上公布的长达16页的报告说:“对中国根本性经济改革及政治开放的期待以失败告终

중국은 생명과 자유, 행복추구권에 대한 미국의 기본적인 신념을 흔드는 정책을 추진하고 있다”고 진단했다 that the Chinese Communist Party “promotes globally a value proposition that challenges the bedrock American belief in the unalienable right of every person to life, liberty, and the pursuit of happiness

中国正在推行动摇美国对生命 自由和追求幸福权的基本信念的政策”

이어 “이제 중국에 대해 경쟁적 접근을 할 것”이라고 선언했다 ” The report announced that a competitive approach will be adopted toward China

报告还宣布:“现在将对中国展开竞争”

보고서는 “대중 외교가 헛된 시도임이 확인되면 미국은 중국의 행동에 상응하는 비용을 지렛대로 사용해 미국의 이익을 보호하는 데 나서고 중국 정부에 대한 공개적인 압박을 확대할 것”이라고 밝혔다 “When quiet diplomacy proves futile, the United States will increase public pressure on the PRC government and take action to protect United States interests by leveraging proportional costs when necessary,” said the report

报告说,“如果证实对华外交是徒劳的尝试,美国将对中国的行动以相应的费用作为杠杆,以保护美国的利益,并加大对中国政府的公开施压”

중국의 악의적 행동, 투자, 의도 등을 언급하며 ‘악의적(malign)’이란 형용사를 8차례나 사용했다 The word “Malign” was used eight times throughout the report to describe China’s malicious bestment, and intention,ehavior, inv

报告提及中国的恶意行为 投资 意图等,使用了“恶意(malign)”的形容词

‘약탈적(predatory) 경제’란 표현도 등장했다 along with the phrase “predatory economic practices

报告中甚至出现了“掠夺性(predatory)经济”的说法

보고서는 또 ‘전략핵무기 3축체계(Nuclear Triad)’의 현대화로 힘을 통한 평화를 유지할 뜻을 강조했다 （no translation）

报告还强调,通过“战略核武器三轴体系(NuclearTriad)”的现代化,将维持通过力量实现的和平

극초음속 미사일체계, 사이버·우주 기반 무기의 실전 배치 등을 앞당기겠다는 계획도 담았다 （no translation）

报告中还包括提前进行极超音速导弹体系 基于网络和宇宙的武器的实战部署等计划

중국의 위협에 맞서기 위한 방안으로는 역내 동맹 및 파트너들과의 관계 강화를 언급했다 ” Strengthening relationships with regional alliances and partners was mentioned as one of the measures to address Chinese threats

作为应对中国威胁的方案,报告提到了加强与区域盟友及伙伴的关系

문재인 정부의 ‘신남방 정책’을 미국이 협력해야 할 역내 동맹국 정책으로도 꼽았다 The “New Southern” policy of the current South Korean administration was pointed as one of the policies of regional alliances with which the U S should cooperate

报告把文在寅政府的“新南方政策”认为是美国应该合作的区域内盟国政策

브루스 베넷 랜드연구소 선임연구원은 미국의소리(VOA) 방송에 “이번 보고서는 사실상 미국 정부가 중국에 대해 신냉전을 선포한 성격이 짙다”고 평가했다 Bruce Bennett, a principal researcher at RAND Corporation, said during an interview with Voice of America that the new report is practically Washington’s announcement of a new cold war on China

兰德研究所首席研究员布鲁斯·贝内特对“美国之音”广播电台评价称:“这一报告实际上具有十分浓厚的美国政府宣布对中国实行新冷战的性质”

또 중국 정부가 홍콩 국가보안법 제정에 나선 것에 대해 미국이 강력하게 반발해 미중 갈등의 새로운 뇌관으로 떠올랐다 （no translation）

此外,美国强烈反对中国政府制定香港“国家安全法”,这将成为中美矛盾的新导火索

트럼프 대통령은 “만약 그것(홍콩 국가보안법 제정)이 일어난다면 우리는 그 문제를 매우 강하게 다룰 것”이라고 경고했다 （no translation）

特朗普总统警告说:“如果发生(制定香港《国家保安法》),我们将非常严厉地处理这个问题”

美中전방위 패권전쟁 개전 S Korea needs survival strategy amid U S -China dispute

中美全方位霸权战打响……韩国应确立生存战略

한생존전략 정립하라미국과 중국이 코로나19 사태를 계기로 정치 외교안보 경제 전반에 이르는 대결을 본격화하고 있다 Signs of a heated power struggle between Washington and Beijing have been becoming clearer across the political, diplomatic and economic fields since the COVID-19 pandemic sparked tensions between them

美国和中国以新冠疫情为契机,正式展开了包罗政治 外交 安保 经济等的全面对决

미 백악관은 21일(현지시각) 의회에 제출한 보고서에서 ‘중국의 경제 정치 군사적 힘 확대가 미국의 이익과 전 세계 국가의 주권을 훼손했다’면서 “The CCP (the Chinese Communist Party)’s expanding use of economic, political, and military power to compel acquiescence from nation states harms vital American interests and undermines the sovereignty and dignity of countries and individuals around the world,” the White House said in a report submitted to the U S Congress

美国白宫当地时间21日在向国会提交的报告中称,“中国经济 政治 军事力量的扩大损害了美国的利益和世界各国的主权”,

‘지난 20년간 미국의 대중(對中) 정책에 대한 근본적 재검토가 필요하다’고 했다 It also pointed out the need to rethink the policies of the past two decades toward China

“有必要从根本上重新考虑过去20年间美国的对华政策”

미중 갈등이 코로나19에 대한 책임론과 경제전쟁을 넘어 장기적인 패권전쟁으로 나아가고 있다 Triggered by economic tensions and COVID-19 related accountability issues, the power struggle between Washington and Beijing has turned into a long-run warfare

中美矛盾正在超越对新冠疫情的责任论和经济战,走向长期霸权战

세계 1, 2위 경제대국인 미국과 중국은 트럼프 정부 출범 이후 노골적인 무역분쟁을 일으켜왔는데 The world’s two biggest economic powerhouses have constantly engaged in obvious trade war since the Trump administration was inaugurated

世界第一和第二经济大国美国和中国,自特朗普政府上台以来一直露骨地挑起贸易纠纷,

최근엔 코로나19 사태의 책임을 놓고 “악랄한 독재정권” “완전히 미쳤다”면서 정면충돌했다 These days, however, they have pointed an accusatory finger at each other since the COVID-19 pandemic took place, even using bluntly explicit phrases such as “a vicious authoritarian regime” and “totally insane

但最近围绕新冠疫情的责任,双方发生正面冲突,互相指称“恶劣的独裁政权”“彻底疯了”

중국이 전국인민대표대회에서 ‘홍콩 국가보안법’을 제정하겠다고 하자 미국이 강경 대응 방침을 밝히는 등 전선(戰線)은 점점 늘고 있다 ” As Beijing announced to enact national security laws regarding Hong Kong in its annual plenary session of the National People's Congress, Washington, in turn, reacted strongly by releasing a stringent action plan, which only shows the deepening of their conflict

在中国全国人民代表大会提出制定“香港国家安全法”后,美国表明了强硬应对方针,战线逐渐扩大

미국은 한국의 외교안보 동맹이지만 경제 분야에서는 대중 수출이 1위, 대미 수출이 2위다 China is South Korea’s largest export destination followed by the United States while Seoul and Washington are close diplomatic and security allies

美国是韩国的外交安保同盟,但在经济领域,韩国对华出口居第一位,对美出口居第二位

한국은 그동안 미중 사이에서 전략적 모호성을 유지해왔으나 점점 양자택일의 압력이 강해지고 있다 Being sandwiched between the two, the South Korean government has so far tried to convey strategic vagueness

此前,韩国在美中之间一直维持着战略模糊性,但二选一的压力越来越大

미국은 중국을 빼고 안보상 믿을 수 있는 나라들끼리 글로벌 공급망을 새로 짜자는 ’경제번영네트워크(EPN)＇을 들고 나와 한국의 참여를 압박하고 있다 However, it is currently under greater pressure to choose to side with either of them For example, Washington has recently pushed Seoul to join “Economic Prosperity Network,” proposing to build a new global supply network among trusted security partners except China

美国提出“经济繁荣网络(EPN)”,要求建立把中国排除在外 安保上可以信任的国家共同重新建立全球供应网,向韩国施压要求参与

중국 화웨이에 연간 10조 원 이상의 반도체를 수출하는 한국 기업들에게도 수출 중단 압력을 넣고 있다 South Korean semiconductor exporters are pressed into severing their more-than-10-trillion-won annual trade ties with China’s Huawei

对于每年向中国华为公司出口10万亿韩元以上半导体的韩国企业也施加了中断出口的压力

안보는 미국, 경제는 중국에 기대는 안미경중(安美經中) 구조를 가진 한국에는 크나큰 시련이다 Indeed, South Korea finds itself in a painful dilemma as it has high security dependence on the United States whereas its economy greatly relies on China

对于安保依赖美国 经济依赖中国,维持“安美经中”结构的韩国来说,这是巨大的考验

사드(THAADㆍ고고도미사일방어체계) 사태로 경제에 큰 타격을 입었던 악몽을 떠올릴 수밖에 없다 All of this reminds Seoul of the economic blow dealt by the THAAD issue

这不得不让人想起因萨德(THAAD,末段高空导弹防御系统)事件而使经济遭受重创的噩梦

그러나 한국 정부가 미중 신(新)냉전 시대를 맞아 제대로 대비하고 있는지 의문이다 Nevertheless, it is questionable whether the South Korean government gets fully ready for a new cold war era led by the United States and China

但是韩国政府在美中新冷战时代是否做好了应对准备还是个疑问

포스트 코로나 시대 글로벌 외교 안보 경제에 대한 총론 없이 남북관계와 시진핑 중국 국가 주석의 방한 등에만 관심을 쏟고 있는 것이 아닌지 걱정스럽다 What’s worrying is that it pays attention merely to inter-Korean relations and Chinese President Xi Jinping’s visit to South Korea without any holistic action plan in place regarding global economic, diplomatic and security issues emerging in the post-COVID-19 era

令人担心的是,韩国政府是否在缺乏后新冠时代全球外交 安保 经济的概论的情况下,只关心南北关系和中国国家主席习近平访韩等

북한의 천안함 폭침에 대응한 5·24조치에 대해 한국 정부가 사실상 폐기를 선언하자 당장 미국이 “비핵화 진전과 보조를 맞춰야 한다＂고 제동을 거는 등 한미간에 미묘한 냉기류마저 감돈다 As for Seoul’s de facto withdrawal from the May 24 measures taken following the North's torpedoing of the South's naval ship Cheonan, Washington expressed a feeling of unease by saying that the issue should be considered coupled with progress in denuclearization talks, leaving their relationship uncomfortable

韩国政府事实上宣布废除应对朝鲜炸沉天安舰的“5·24”措施后,美国立即表示“应该与无核化的进展步调一致”予以叫停等,韩美间甚至出现了微妙的冷气流

외교부는 지난해 미중 갈등 대응을 위해 외교전략조정회의를 출범시켰지만 올해 들어 단 한차례도 열지 않았다 To better respond to the U S -China tensions, the South Korean Ministry of Foreign Affairs embarked on a diplomatic strategy meeting last year, which has not happened at all since the turn of the year

韩国外交部去年成立了应对美中矛盾的外交战略协调会议,但今年以来一次也没有举行

오히려 문재인 대통령은 취임 3주년 기자회견에서 북-미대화만 바라보지 말고 남북간에 할 수 있는 일을 해야 한다고 강조했다 In contrast to that, South Korean President Moon Jae-in put emphasis on finding what the two Koreas can do, rather than expecting a great deal from the U S -North talks

反而,文在寅总统在就任3周年在记者会上强调,不能只期盼朝美对话,应该做南北之间可以做的事情

중국에 대한 경제의존도가 높은 상황에서 미국의 EPN 압박을 어떻게 풀어나갈지, 시 주석의 연내 방한이 추가적인 긴장요인으로 작용하는 것은 아닌지 정부의 외교 경제 전략을 총체적으로 재점검해야 한다 A thorough review should be conducted of the South Korean government’s diplomatic and economic policy frame to see how it can handle Washington’s push for the E P N initiative while considering its high economic reliance on China or to assume whether additional tension arises when President Xi visits Seoul this year

在经济对中国高度依赖的情况下,如何化解美国的EPN压力,习主席年内的访韩是否会成为进一步的紧张因素,需要政府重新审视外交经济战略

美, 양회 앞둔 中에 “또라이” “악랄한 독재정권” 원색비난 U S harshly criticizes China calling it authoritarian regime

美国在两会前夕不加掩饰地指责中国为“疯子”“恶劣的独裁政权”

중국의 최대 정치행사인 양회(兩會)를 앞두고 도널드 트럼프 미국 행정부가 중국에 대한 비난 수위를 최고조로 끌어올렸다 The United States has ratcheted up criticism of China in the run up to the annual plenary session of the National People's Congress, the biggest political event in Beijing

在中国最大的政治活动“两会”即将召开之际,美国特朗普政府把对中国的指责调到了最高潮

트럼프 대통령은 중국 정부의 입장 발표에 ‘또라이’ ‘얼간이’라며 막말을 퍼부었고 마이크 폼페이오 국무장관은 “악랄한 독재정권”이라고 중국 정부를 정조준했다 U S President Donald Trump used words such as “wacko” and “dope” describing the Chinese government’s recent statement, while Secretary of State Mike Pompeo called China “a brutal authoritarian regime

特朗普总统对中国政府发表的立场大放厥词,称其为“疯子”“傻瓜”,而国务卿蓬佩奥则将矛头指向中国政府,称其为“恶劣的独裁政权”

트럼프 대통령은 20일 트위터에 “중국의 어떤 또라이(wacko)가 방금 수십만 명을 죽인 바이러스에 대해 중국을 제외한 모든 이들을 비난하는 성명을 발표했다”며 ” “Some wacko in China just released a statement blaming everybody other than China for the Virus which has now killed hundreds of thousands of people,” said President Trump on his Twitter account on Wednesday

特朗普20日在推特上写道,“中国的某个疯子(wacko)就刚刚杀死数十万人的病毒,指责了除中国以外的所有人”,

“이 얼간이(dope)에게 이러한 전 세계적 대규모 살상을 저지른 것이 다름 아닌 중국의 무능이라는 것을 설명 좀 하라”고 적었다 “Please explain to this dope that it was the incompetence of China and nothing else that did this mass Worldwide killing

“对这个蠢人(dope)说明一下吧,导致如此大规模杀伤的,不是别人而是中国的无能”

누구를 겨냥한 것인지는 구체적으로 언급하지 않았지만 신종 코로나바이러스 감염증(코로나19)과 관련한 중국 외교부 등 주요 기관의 대변인일 가능성이 높다 （no translation）

虽然特朗普没有具体说明是针对谁的,但是很有可能是与新冠病毒有关的中国外交部等主要机关的发言人

폼페이오 장관은 이날 오전 기자회견에서 “중국은 1949년부터 악랄하고 권위주의적인 공산 정권에 의해 지배돼 왔다”며 “중국은 이데올로기적으로도, 정치적으로도 자유국가에 적대적”이라고 주장했다 ” “China has been ruled by a brutal, authoritarian regime, a communist regime, since 1949,” Secretary Pompeo said during a press conference on Wednesday morning, claiming, “Beijing is ideologically and politically hostile to free nations

蓬佩奥在当天上午举行的记者会上说,“自1949年起,中国一直被恶劣的 权威主义的共产政权统治”,“中国无论在意识形态上还是政治上都对自由国家怀有敌意”

이어 “이 전염병으로 9만 명에 이르는 미국인이 숨졌고, 3월 이후 3600만 명이 일자리를 잃었다 ” “This plague has cost roughly 90,000 American lives, more than 36 million Americans have lost their jobs since March,” Secretary Pompeo said

他还抨击称,“这一传染病造成9万美国人死亡,3月以来有3600万人失业

중국 공산당의 (대응) 실패로 전 세계적으로 최대 9조 달러의 피해를 입었다”고 비판했다 “Could be as much as nine trillion dollars, according to our estimates, cost imposition on the world of the Chinese Communist Party's failures”

因为中国共产党的(应对)失败,全世界遭受了最多达9万亿美元的损失”

폼페이오 장관은 이날 1억6200만 달러 규모의 해외 코로나19 피해 지원책을 발표했다 （no translation）

蓬佩奥当天宣布一项总额为1.62亿美元的新冠病毒受损海外援助计划

그는 미 국제개발처(USAID)와 함께 100억 달러의 지원을 약속한 것 외에 추가로 이를 지원한다는 점을 강조하면서 “중국이 내놓은 20억 달러는 세계에 끼친 (피해)비용에 비하면 쥐꼬리(paltry) 수준이다 （no translation）

他强调,除了与美国国际开发署(USAID)共同承诺提供100亿美元的援助外,还将进一步提供援助他同时揶揄称,“中国拿出的20亿美元与给世界造成的(损失)费用相比,只是九牛一毛(palary)

그거라도 약속한 대로 이행하기를 기대한다”고 꼬집었다 （no translation）

我们期待,即使那么一点,也能如实履行”

그는 이 자리에서 차이잉원(蔡英文) 대만 총통의 재취임을 거듭 축하했다 （no translation）

他在记者会上再次祝贺台湾领导人蔡英文再次当选

홍콩과 관련해서는 “중국으로부터 높은 수준의 자율성을 갖고 있는지에 대한 판단을 아직 내리지 않았고 현재 일어나는 일을 예의 주시하고 있다”고 밝혔다 （no translation）

关于香港,他表示:“对于香港是否(从中国)具有高度的自治,还没有做出判断,正在密切关注现在发生的事情”

반도체 위탁생산(파운드리) 업체인 대만 TSMC의 미국 투자와 상무부의 화웨이 수출 제재안 발표 등도 언급했다 （no translation）

他还提到了代工生产企业台积电(TSMC)的对美国投资和商务部发表针对华为的出口制裁案等

이런 트럼프 행정부의 거친 ‘중국 때리기’는 국내 정치용이라는 분석이 나온다 （no translation）

有分析认为,特朗普政府的这种粗暴的“打击中国”行为是出于美国国内政治考虑

공화당은 이미 중국 공격을 연말 대선은 물론이고 전국 주요 주지사 및 의원 선거의 핵심 전략으로 삼는 지침을 정했다 （no translation）

共和党已经制定了把攻击中国作为年底总统选举乃至全国主要州长及议员选举的核心战略的方针

공화당 캠페인 전략팀이 지난달 당에 배포한 메모에는 이와 함께 선거 경쟁자들을 친(親)중국파 혹은 중국에 대해 유약한 이미지로 공격하라는 등의 내용이 담겼다 （no translation）

共和党竞选战略小组上个月向党内散发的备忘录中,还包含要求把选举竞争对手作为亲华派或对华软弱的形象进行攻击等内容

거듭된 공격에 중국도 발끈했다 （no translation）

美国的一再攻击让中国也勃然大怒

중국 관영 영어방송 CGTN은 트위터에 ‘폼페이오 신뢰도 테스트’라는 영상(사진)을 올려 중국을 공격하는 폼페이오 장관을 우스꽝스럽게 묘사했다 （no translation）

中国官方英语电视台CGTN在推特上上传了名为“蓬佩奥可信度测试”的视频(照片),把攻击中国的蓬佩奥描述成小丑

21일 런민일보(人民日報)에 따르면 시진핑 주석은 셰이크 하시나 방글라데시 총리와 통화에서 “중국은 전염병 퇴치를 위한 국제 협력에 방해하는 행위에 반대한다”며 에둘러 미국의 공세를 비판했다 （no translation）

据《人民日报》21日报道,中国国家主席习近平在与孟加拉国总理谢赫·哈西娜通话中表示,“中国反对在妨碍防控传染病国际合作的行为”,委婉地批评了美国的攻势

트럼프 “WHO, 한달내 중서 독립 증명해야 Pres Trump asks WHO to prove independence from China U S

特朗普:“世卫组织要在一个月内证明独立性,否则美国可能退出”

아니면 탈퇴할수도”신종 코로나바이러스 감염증(코로나19) 발원지를 놓고 중국과 치열하게 대립하고 있는 도널드 트럼프 미국 대통령이 세계보건기구(WHO)의 친중 성향을 지적하며 ‘30일 안에 개선이 없으면 자금 지원을 영구 중단하거나 탈퇴할 수 있다’는 최후통첩을 날렸다 President Donald Trump who is in an intense confrontation with China regarding the source of COVID-19 has criticized the pro-China tendency of the World Health Organization (WHO) and given an ultimatum to permanently pull funding to the organization if it fails to “commit to major substantive improvements in the next 30 days

美国总统特朗普就新冠病毒发源地问题与中国发生激烈对立,他指出世界卫生组织的亲华倾向,并下最后通牒称,“如果30天内没有改善,将永久中断资金援助或者退出”

트럼프 대통령은 18일 테워드로스 아드하놈 거브러여수스 WHO 사무총장에게 보낸 4쪽짜리 서한에서 “30일 안에 중국으로부터의 독립성을 증명하는 개선안을 내놓지 않으면 자금 지원을 영원히 중단할 수 있음을 알린다 ” “If the World Health Organization does not commit to major substantive improvements within the next 30 days, I will make my temporary freeze of United States funding to the World Health Organization permanent and reconsider our membership in the organization,” read the four-page letter sent by President Trump to WHO Director-General Tedros Adhanom Ghebreyesus on Monday

特朗普18日在写给世界卫生组织总干事谭德塞的四页信函中声称:“如果30天内不拿出证明世卫组织在中国面前保持独立性的改善方案,美国将永久中断资金援助

우리의 회원 자격도 재고하겠다”고 주장했다 “The only way forward for the World Health Organization is if it can actually demonstrate independence from China,” the letter also said

我们也会重新考虑我们的会员资格”

WHO가 앞으로 나아갈 유일한 길은 중국으로부터의 독립을 보여주느냐에 달렸다며 “미국 이익에 부합하지 않는 조직에 납세자의 돈을 지원할 수 없다”고도 했다 “I cannot allow American taxpayer dollars to continue to finance an organization that, in its present state, is so clearly not serving America’s interests

他还表示,世卫组织今后的唯一出路在于能否展现在中国面前的独立性,“不能拿纳税人的钱向不符合美国利益的组织提供援助”

그는 취재진 앞에서도 WHO를 중국의 꼭두각시(puppet)라고 강력히 비판했다 ” President Trump also harshly criticized the WHO as a “puppet of China” in front of correspondents

他在记者面前,猛烈抨击世卫组织是中国的傀儡(puppet)

미국이 WHO에 연 4억5000만 달러의 분담금을 내는 반면 중국은 3800만 달러만 낸다는 점도 문제 삼았다 He also took an issue with the U S contributing 450 million dollars to the WHO while China pays 38 million dollars

他同时还提出异议称,美国每年向世卫组织支付4.5亿美元的分摊额,而中国只支付3800万美元

앨릭스 에이자 미 보건장관은 이날 사상 최초로 화상으로 열린 세계보건총회(WHA)에서“한 회원국이 투명성 의무를 조롱해 전 세계에 엄청난 희생을 초래했다 “At least one member country made a mockery of their transparency obligations, with tremendous costs for the entire world

美国卫生部长亚历克斯·阿扎当天在历史上首次通过视频召开的世界卫生大会上表示,“一个会员国嘲弄透明性义务,给全世界带来了巨大的牺牲

다시는 이런 일이 일어나면 안 된다”며 중국을 정면 비판했다 This cannot ever happen again,”

不能再发生这样的事情”,对中国提出了正面批评

마이크 폼페이오 국무장관은 중국의 반대로 대만의 WHA 참가 시도가 무산된 것을 비판하는 성명을 발표했다 said U S Secretary of Health and Human Services Alex Azar in an apparent reference to China during the World Health Assembly (WHA) meeting held via videoconference for the first time on Monday

美国国务卿蓬佩奥发表声明,批评台湾参加世界卫生大会的计划因中国的反对而流产

폼페이오 장관은 “WHO 사무총장은 대만을 WHA에 참가시킬 모든 법적 권한이 있음에도 중국의 압력 때문에 하지 않았다 Meanwhile, Secretary of State Mike Pompeo announced a statement criticizing the WHO for failing to allow Taiwan to participate in the WHA due to opposition from China

蓬佩奥说:“尽管世卫组织总干事有让台湾参加世界卫生大会的法律权限,但因为中国的压力,他没有这样做

사무총장의 독립성 결여가 WHO의 신뢰와 효율성을 훼손시켰다”고 지적했다 Pompeo wrote that the WHO director-general did not allow Taiwan in the WHA due to pressure from China despite all the legal authorities he has to do so and that the director-general’s lack of independence has damaged the WHO’s reliability and efficiency

总干事缺乏独立性破坏了世界卫生组织的信任和效率”

존 울리엇 미 백악관 국가안보회의(NSC) 대변인은 시진핑(習近平) 중국 국가주석이 WHA 화상 회의에서 “코로나19 피해국에 20억 달러를 지원하겠다”고 밝힌 것을 비난했다 National Security Council spokesman John Ullyot criticized Chinese President Xi Jinping’s announcement during the WHA meeting to provide two billion dollars to countries heavily affected by COVID-19,

美国白宫国家安全委员会发言人约翰·乌利奥特表示,对中国国家主席习近平视频会议上表示“将向新冠疫情受害国提供20亿美元援助”进行批评

그는 “점점 더 많은 나라가 중국의 책임을 묻는 것에서 주의를 분산하려는 시도”라고 지적했다 saying that it is an attempt to distract a growing number of countries asking for China’s responsibility

他指出:“这是为了从越来越多国家试图追究中国责任中分散注意力”

WHA는 19일 회의에서 코로나19 독립 조사 진행에 대한 결의안을 표결한다 （no translation）

世界卫生大会将在19日的会议上表决有关新冠病毒展开立调查的决议

194개 WHO 회원국 중 3분의 2인 129개국의 지지를 얻으면 통과된다 （no translation）

在194个世卫组织成员国中,如果得到三分之二的129个国家的支持,就可以通过该决议

이미 호주 영국 프랑스 러시아 등 122개국이 지지를 표명해 통과 가능성이 높다는 관측이 제기된다 （no translation）

澳大利亚 英国 法国 俄罗斯等122个国家已经表示支持,因此有观察人士认为通过的可能性很高

중국도 반격에 나섰다 （no translation）

中国队也展开了反击

관영 환추(環球)시보는 19일 “이 조사에 미국도 포함해야 한다 중국은 조사를 두려워하지 않는다”고 맞섰다 （no translation）

官方的《环球时报》19日称:“这项调查应该包括美国中国不怕调查”

상무부 역시 “향후 5년간 호주산 보리에 반덤핑 관세를 부과하겠다”고 밝혔다 （no translation）

商务部也表示,“今后5年内将对澳大利亚产大麦征收反倾销税”

호주산 보리와 쇠고기의 최대 수입국인 중국은 12일에도 일부 호주산 쇠고기의 수입을 금지했다 （no translation）

澳大利亚产大麦和牛肉的最大进口国中国12日还禁止了部分澳大利亚产牛肉的进口

호주가 미국 편에 서서 코로나19 조사를 촉구해 왔다는 이유로 경제 보복에 나섰다는 분석이 나온다 （no translation）

有分析认为,澳大利亚一直站在美国一边敦促调查新冠病毒,中国因此进行了经济报复

발끈한 호주 역시 세계무역기구(WTO)에 제소할 의사를 밝혔다 （no translation）

愤怒的澳大利亚也表示将向世界贸易组织提起诉讼

미중 갈등의 한복판에 있는 중국 최대 통신장비업체 화웨이는 18일 성명에서 “미국의 화웨이 제재는 자의적이고 치명적이며 결국 미국의 이익도 해칠 것”이라고 주장했다 （no translation）

处于中美矛盾中心的中国最大的通信设备制造商华为18日在声明中表示:“美国对华为的制裁是恣意的 致命的,最终也将损害美国的利益”

양국 갈등이 미 주식시장으로도 옮겨붙는 모습이다 （no translation）

两国的矛盾也转移到了美国股市上

로이터통신은 미 2위 증권거래소 나스닥이 중국 기업의 기업공개(IPO) 자격 및 회계감사 등을 강화하는 규제를 조만간 공개할 것이라고 전했다 （no translation）

据路透社报道,美国第二大证券交易所纳斯达克近日将公布加强中国企业首次公开募股(IPO)资格及会计审计等方面的限制

중국 금융당국 역시 중국 기업에 영국 런던 증시 상장을 독려하고 있다고 덧붙였다 （no translation）

报道称,中国金融当局也在鼓励中国企业在英国伦敦上市

美구축함, 상하이 인근 해상까지 접근U S destroyer sails off coast of Shanghai

美驱逐舰接近上海附近海域

미국과 중국 간에 경제뿐 아니라 군사 측면에서도 긴장감이 높아지고 있다 Tensions between the United States and China are intensifying not only in trade but also in the military

不仅是经济方面,美国和中国之间在军事方面的紧张感也在加剧

미 군함들이 이례적으로 중국 근해까지 진출했고, 양국이 최신 무기 개발 경쟁에도 나서고 있다 U S warships have unusually sailed through waters near China while both countries are engaged in a race to develop new weapons

美国军舰罕见地出现在中国近海,两国还展开了研发最新武器的竞争

홍콩 사우스차이나모닝포스트(SCMP)는 16일 베이징대 소속 연구기관인 남중국해전략태세감지계획의 발표를 인용해 미 해군 알레이버크급 구축함인 라파엘 페랄타함(DDG-115)이 최근 상하이(上海)에서 115해리(약 213km) 떨어진 해상까지 접근했다고 보도했다 The South China Morning Post reported on Saturday that USS Rafael Peralta (DDG-115), an Arleigh Burke-class destroyer, was seen about 213 kilometers off the coast of Shanghai with a picture released by the South China Sea Strategic Situation Probing Initiative, a think tank at Peking University

香港《南华早报》16日援引北京大学下属研究机构“南海战略态势感知计划”发表的消息称,美国海军“阿利·伯克”级驱逐舰“拉斐尔·佩拉尔塔”号(DDG-115)最近出现在距离上海115海里(约213公里)的海上

미 해군 태평양함대도 전날 트위터에 날짜를 특정하지 않은 ‘라파엘 페랄타함이 이번 주에 동중국해를 항해했다’고 공개했다 The U S Pacific Fleet also said in a Twitter post, “USS Rafael Peralta sails in the East China Sea this week,” without specifying the date

美国海军太平洋舰队前一天在推特上宣布,“‘拉斐尔·佩拉尔塔’号本周航行在东中国海”,但没有公布具体的日期

라파엘 페랄타함의 이번 항해는 중국 인민해방군이 14일부터 황해 보하이만에서 실사격 훈련을 하는 가운데 이뤄졌다 The sailing of the ship came as the People’s Liberation Army of China began live-fire drills in Bohai Bay of the Yellow Sea on Thursday,

“拉斐尔·佩拉尔塔”舰此次航行是在中国人民解放军14日开始在黄海的渤海湾进行实弹演习时进行的

항공모함이 참여할 것으로 예상되는 중국군의 훈련은 두 달 반 동안 계속된다 which is expected to involve aircraft carriers and will continue for two months and a half

预计将有航母参与的中国军队演习将持续两个半月

앞서 지난달 17일 다른 미 해군 구축함인 매케임벨함(DDG-85)도 산둥(山東)성 웨이하이(威海)에서 불과 42해리 떨어진 해상까지 접근한 바 있다 This is the second time a U S warship was seen off the coast of China in a month, following USS McCampbell (DDG-85), another U S

此前的上个月17日,另一艘美国海军驱逐舰“麦克坎贝尔”号(DDG-85)也曾接近距山东威海仅42海里的海域

한 달 새 두 번이나 미 군함이 중국 연안에 바짝 다가선 것이다 Navy destroyer, spotted 42 nautical miles off the coast of Weihai in Shandong on April 17

一个月内,美国军舰两次逼近中国沿岸

미국은 지속적으로 군사력 강화를 진행하고 있다 The United States are strengthening its military power

美国一直在加强军事力量

도널드 트럼프 미국 대통령은 15일(현지 시간) 우주군기(旗) 공개 행사에 참석해 “우리는 지금까지 본 적이 없는 놀라운 군사 장비를 개발 중”이며 Earlier on Friday, U S President Donald Trump said the United States was developing a “super duper missile,” unveiling the flag for his Space Force

美国总统特朗普当地时间15日出席太空军旗亮相活动时表示,“我们正在研制迄今为止从未有过的惊人军事装备”,

“이 기막힌 미사일은 지금 우리가 보유한 것보다 17배 빠르다고 들었다”고 과시했다 이어 “우리의 적국들이 있기 때문에 해야만 하는 일”이라고 덧붙였다 He went onto say that, “We have to do it with the adversaries we have out there, and I heard the other night it is 17 times faster than what they have right now

“我听说,这种令人不可思议的导弹比我们现在拥有的快17倍”他还表示,“因为有我们的敌国,所以这是必须要做的”

중국과 러시아가 극초음속 무기 개발을 추진하는 시점에서 트럼프 대통령이 이런 언급을 한 것은 중국과 러시아를 겨냥한 것으로 보인다고 CNN은 전했다 ” His remarks appear to target China and Russia that are developing hypersonic weapons, said CNN U S -China tensions are through the roof Chinese media outlets have blamed U S

”美国有线电视新闻网(CNN)报道说,在中国和俄罗斯推进开发高超音速武器的时刻,特朗普总统作出上述发言,可能是针对中国和俄罗斯

미 국방부는 이에 대한 언론의 질의에 구체적인 답변을 내놓지 않았다 （no translation）

美国国防部没有就媒体提出的问题给出具体答复

국방부 대변인인 로버트 카버 공군 중령은 CNN에 “극초음속 무기의 실전 배치는 기술 연구 및 공학에 있어 최우선 사항”이라며 “미국은 극초음속 무기 시스템의 개발을 위한 탄탄한 프로그램을 확보하고 있다”고 강조했다 （no translation）

美国防部发言人 空军中校罗伯特·卡佛对CNN表示,“高超音速武器的实战部睹,在技术研究及工程中是最优先事项”,并强调,“在高超音速武器系统开发方面,美国拥有稳定的项目”

“미친 짓”까지 터져나온 美-中갈등 미중 갈등이 선을 넘고 있다 （no translation）

美中矛盾加剧,甚至出现“疯狂之举”美中矛盾正在越过界限

“중국과 모든 관계를 끊을 수도 있다”는 도널드 트럼프 미국 대통령의 발언에 중국 언론은 “정신이상자”라고 비난했다 President Donald Trump for making a “lunatic” statement saying that he "could cut off the whole relationship" with China

美国总统特朗普声称,“可能与中国断绝一切关系”,中国媒体则抨击特朗普“精神失常”

미국이 관계를 끊으면 대만을 공격하자는 주장까지 나왔다 Some of them have made an aggressive remark to attack Taiwan if Washington severs the U S -China relations

甚至有人声称,如果美国断绝关系,就会攻击台湾

미국은 미국 증시에 상장된 중국 기업을 정조준했다 Meanwhile, the prime target of Washington is reportedly Chinese-based companies listed in the U S stock market

美国则瞄准了在美国股市上市的中国企业

중국 런민(人民)일보 자매지 환추(環球)시보는 15일 사설에서 전날 트럼프 대통령의 발언을 “미친 짓”이라고 비판하면서 The Global Times, a sister newspaper under the People's Daily, criticized President Trump’s remark as “lunacy” in its editorial on Friday

中国《人民日报》姐妹纸《环球时报》在15日的社论中批评特朗普前一天的发言是“疯狂之举”,

“중국은 과학기술 및 이와 관련 있는 경제, 인문·사회과학 등 미중 관계의 핵심 분야에서 실질적인 ‘관계 단절’에 대한 준비를 해야 한다”고 주장했다 “China should be prepared for a partial or complete decoupling in vital sectors, including technology, the economy, humanities, and social sciences,” it argued

并主张,“中国在科学技术以及与此相关的经济 人文 社会科学等美中关系的核心领域,应该做好‘实质性脱钩’的准备”

중국 글로벌타임스는 트럼프 대통령을 향해 “궁지에 몰린 짐승 같다” “미친 것처럼 보인다”고 원색적으로 비판했다 The newspaper gave President Trump raw criticism saying, “Trump is like a cornered beast doing something desperate,” and “Is Trump totally insane

《环球时报》英文版则毫不掩饰地抨击特朗普是“就像被逼入绝境的野兽”“看起来像是疯了”

진찬룽(金燦榮) 런민(人民)대 국제관계학원 부원장은 “미국이 일방적으로 관계를 끊으면 중국은 즉각 대만을 (무력)통일할 수 있다 중국은 미중 관계를 유지하기 위해 대만 문제를 해결하지 않았다”고 말했다 ” “In the past, we didn't solve the Taiwan question because we wanted to maintain the China-US relationship, and if the U S unilaterally cuts it off, we can just reunify Taiwan immediately since the Chinese mainland has an overwhelming advantage to solve this long-standing problem," said Jin Canrong, the associate dean of Renmin University of China's School of International Studies in Beijing

中国人民大学国际关系学院副院长金灿荣表示:“如果美国单方面断绝关系,中国可以立即(武力)统一台湾为了维护中美关系,中国没有解决台湾问题”

트럼프 대통령은 전날 폭스비즈니스 인터뷰에서 미국 증시에 상장하려는 중국 기업들은 미국 회계기준을 준수하도록 의무화하는 방안을 “매우 강하게” 살펴보고 있다고 밝혔다 President Trump told Fox Business on Thursday that his administration is looking "very strongly” at mandating Chinese companies to follow U S accounting standards when they pursue an IPO in the U S stock market

特朗普在前一天接受福克斯商业频道采访时称,他正在“非常强烈”地审视一项方案,准备要求在美国股市上市的中国企业必须遵守美国的会计准则

뉴욕증권거래소(NYSE)와 나스닥에 상장됐지만 미국 기업에 적용되는 일반회계 기준(GAAP)을 따르지 않는 중국 기업에 대한 제재를 검토할 수 있다는 뜻으로 풀이된다 He may intend to consider imposing sanctions against Chinese companies on NYSE or NASDAQ that do not stick to Generally Accepted Accounting Principles (GAAP), which U S firms have followed

分析认为,特朗普的意思是,可以考虑对在纽约证券交易所和纳斯达克上市但不遵守美国企业适用的一般会计标准(GAAP)的中国企业进行制裁

반도체, 의약품 등의 생산·공급시설을 미국에 유치해 공급망에서 중국을 제외하려는 움직임도 진행되고 있다 Washington has reportedly made some preparations to attract semiconductor and pharmaceutical fabricators and suppliers to the United States as a way to ostracize China from the global supply chain

同时,美国还在积极诱导半导体 医药品等生产 供应设施前往美国,试图将中国排除在供应链之外

대만과 남중국해에서도 미중 간 군사 긴장이 높아져 우발적 충돌 위험이 커지고 있다 Meanwhile, risks of accidental conflict between Washington and Beijing are mounting due to the ever-escalating military tensions over South China Sea and Taiwan

在台湾和中国南海,中美军事紧张加剧,偶发性冲突风险增加

미국은 최근 남중국해에 잇달아 군함과 전략폭격기를 보내는 등 중국에 대한 군사적 압박을 높이고 있다고 CNN이 보도했다 According to CNN, the United States is increasing military pressure on China by dispatching battleships and strategic bombers to South China Sea

据美国有线电视新闻网(CNN)报道,美国最近接连向中国南海派遣军舰和战略轰炸机,加大了对中国的军事压力

（（additional translation））U S -China tensions are through the roof Chinese media outlets have blamed U S President Donald Trump for making a “lunatic” statement saying that he "could cut off the whole relationship" with China Some of them have made an aggressive remark to attack Taiwan if Washington severs the U S -China relations Meanwhile, the prime target of Washington is reportedly Chinese-based companies listed in the U S stock market The Global Times, a sister newspaper under the People's Daily, criticized President Trump’s remark as “lunacy” in its editorial on Friday “China should be prepared for a partial or complete decoupling in vital sectors, including technology, the economy, humanities, and social sciences,” it argued The newspaper gave President Trump raw criticism saying, “Trump is like a cornered beast doing something desperate,” and “Is Trump totally insane ” “In the past, we didn't solve the Taiwan question because we wanted to maintain the China-US relationship, and if the U S unilaterally cuts it off, we can just reunify Taiwan immediately since the Chinese mainland has an overwhelming advantage to solve this long-standing problem," said Jin Canrong, the associate dean of Renmin University of China's School of International Studies in Beijing President Trump told Fox Business on Thursday that his administration is looking "very strongly” at mandating Chinese companies to follow U S accounting standards when they pursue an IPO in the U S stock market He may intend to consider imposing sanctions against Chinese companies on NYSE or NASDAQ that do not stick to Generally Accepted Accounting Principles (GAAP), which U S firms have followed Washington has reportedly made some preparations to attract semiconductor and pharmaceutical fabricators and suppliers to the United States as a way to ostracize China from the global supply chain Meanwhile, risks of accidental conflict between Washington and Beijing are mounting due to the ever-escalating military tensions over South China Sea and Taiwan According to CNN, the United States is increasing military pressure on China by dispatching battleships and strategic bombers to South China Sea

美中갈등 속 시진핑 방한에 매달려선 우리 외교 길을 잃는다 The importance of not being swayed amid escalating U S -China tensions

在美中矛盾中执意寻求习近平访韩,将失去韩国外交道路

시진핑 중국 국가주석이 그제 문재인 대통령과의 통화에서 “금년 중 방한하는 데 대한 굳은 의지는 변하지 않았다”고 말했다고 청와대가 전했다 Chinese President Xi Jinping expressed his continued commitment to visiting South Korea this year on a phone call to President Moon Jae-in on Wednesday, said the South Korean presidential office Cheong Wa Dae

青瓦台称,中国国家主席习近平前天在与文在寅总统通电话时表示:“今年访韩的坚定意志没有改变”

이에 문 대통령은 한중관계에서 시 주석 방한이 무엇보다 중요하다고 했다 In response, President Moon emphasized the importance of President Xi’s visit in the bilateral relations

对此,文在寅表示,在韩中关系上,习主席的访韩比什么都重要

이번 통화는 시 주석의 요청으로 이뤄졌다고 한다 The phone call was arranged at the request of President Xi

据悉,此次通话是应习近平主席的邀请进行的

양국 정상의 통화는 미중 갈등이 다시 격화되는 미묘한 시기에 이뤄졌다 The call took place as the U S -China tensions are escalating

两国首脑的通话是在美中矛盾再次激化的微妙时期进行的

무엇보다 중국은 한국을 자기 편으로 끌어들이려는 의도가 역력하다 It is clear that Beijing wants Seoul on its side

最重要的是,中国想将韩国拉到自己一边的意图非常明显

한국도 사드(THAAD·고고도미사일방어체계) 갈등을 마무리하기 위해 시 주석의 조속한 방한을 원하고 있다 while South Korea wants President Xi’s visit to the nation as soon as possible to resolve the conflicts caused by the deployment of THAAD

韩国也希望习主席尽快访问韩国,以结束萨德(THAAD,末段高空区域防御系统)矛盾

그런데 정작 중국 관영매체은 시 주석 방한과 관련한 내용은 전하지 않은 채 양국이 코로나19 공동방역을 두고 ‘비바람 속에서도 한 배를 타고 있다(風雨同舟)’고만 했다 However, the Chinese state media has made no mention of President Xi’s visit and simply stated the two nations are in the same boat against the COVID-19 crisis

但中国官方媒体却没有报道习主席访韩的相关内容,只是说两国在共同防控新冠疫情时“风雨同舟”

코로나19 발원지를 둘러싼 미중 대립은 모든 분야에서 확전을 예고하고 있다 It appears the tensions between the United States and China over the origins of the pandemic will spread to all the other areas

围绕新冠病毒起源的美中对立预示着在所有领域都将扩大战事

도널드 트럼프 미국 대통령은 코로나19 중국 책임론을 거론하며 관세 부과는 물론이고 중국에 편중된 글로벌 공급망을 바꾸겠다며 동맹국들의 협조를 요청했다 U S President Donald Trump argues that China is responsible for the coronavirus outbreak while threatening to impose higher tariffs on Chinese goods and requesting cooperation from allies to shift the global supply chain that is heavily dependent on China

美国总统特朗普提出“新冠病毒中国责任论”,表示“不仅要加征关税,还要改变偏重中国的全球供应网”,并要求盟国予以协助

중국과의 무역전쟁을 넘어 국제적 무역질서의 재편까지 예고한 셈이다 Trump’s intention seems to go beyond waging a trade war to reestablish the global trade order

这等于是预告了不仅与中国打贸易战,还要重组国际贸易秩序

이런 갈등 국면에서 한국이 어느 한쪽 편을 들다간 무역 보복을 당하거나 외교안보 공조의 틀이 흔들리는 위기에 빠질 수 있다 Against this backdrop, if Seoul sides with one of the two countries, it will either have to face economic retaliation or undermine the foundation of foreign security cooperation

在这种矛盾局面下,韩国如果站在某一方一边,可能会遭到贸易报复,或陷入外交安保合作框架动摇的危机

미중은 벌써부터 글로벌 편 가르기에도 나섰다 （no translation）

美中早已开始在全球划拨分边

미국은 한국을 비롯한 주요 동맹국 외교장관들을 모아 코로나19 대응을 위한 국제협력에서 중국을 견제하고 미국의 리더십을 따르라는 메시지를 보냈다 （no translation）

美国召集包括韩国在内的主要盟国的外交部长,在应对新冠疫情的国际合作中发出牵制中国 跟随美国领导的信息

중국도 전면 대응을 시작했다 （no translation）

中国也开始全面应对

중국은 미국 편을 들어 국제조사를 요구한 호주에 대해 쇠고기 수입금지로 보복에 나섰다 （no translation）

中国通过禁止进口牛肉,对站在美国一边要求进行国际调查的澳大利亚进行了报复

지난해 미중이 화웨이 제품 사용을 두고 국제사회에 선택을 강요하던 일이 보다 큰 폭으로 되풀이되고 있는 셈이다 （no translation）

可以说,去年美中在使用华为产品问题上强迫国际社会做出选择的事情正在以更大的幅度再次上演

연말 미국 대선 때까지 계속될 이번 미중 갈등은 과거와는 다른 차원의 파장을 낳을 수 있다 The U S -China conflicts, which are expected to continue until the U S presidential election scheduled in November, might have unprecedented consequences

此次美中矛盾将持续到年底美国总统选举为止,可能会在不同以往的层面上产生影响

미중 갈등이 무역전쟁에만 집중된다면 우리 경제의 체질 강화로 버틸 수 있겠지만, If the tensions remain only in trade, South Korea’s economy might be able to ride them out with its renewed strength

如果美中矛盾只集中于贸易战,韩国可以靠经济体质的加强撑过去,

이를 뛰어넘는 국제질서 전반의 변동을 낳는다면 선택지가 많지 않다 However, if they trigger a change in international order, South Korea will be left with only few options

但若出现超越这一层度导致整个国际秩序的变动,则选择余地不大

양쪽의 러브콜은 우리의 헤엄칠 공간을 넓히는 대신 잘못 운신했다간 설자리를 잃게 되는 양날의 칼과도 같다 Being wooed by both Washington and Beijing can be a double-edged sword for Seoul: The atmosphere might provide Seoul with more room for maneuver, but it can also leave it with no room at all if anything goes wrong

双方的橄榄枝将像是一把双刃剑,既能扩大韩国游泳的空间,但如果行动出错,韩国将失去立足之地

동맹을 돈으로만 판단하는 트럼프 대통령의 무리한 방위비분담금 요구로 동맹 피로감이 생긴 것도 현실이다 It is true that there is alliance fatigue because of President Trump’s tendency to determine the value of an ally purely based on money

特朗普只用金钱判断同盟,其提出的防卫费分摊额无理要求,令同盟感到疲劳,这也是现实

우리의 선택은 더욱 어려워졌다 South Korea is faced with a difficult choice,

我们的选择更加困难

이런 때일수록 지혜로운 외교가 필요하다 and we need wise foreign policies that can guide us through these trying times

越是这样的时候,越需要有智慧的外交

우선 한미동맹을 근간으로 한 우리 외교정책의 좌표를 분명히 하고 그 토대 위에서 한중협력의 구체적 수준을 정해야 한다 The South Korean government should set a clear direction for foreign policies with the South Korea-U S alliance at heart, based on which, it can decide the extent of cooperation with China

首先,要明确韩国以韩美同盟为根本的外交政策坐标,在此基础上确定韩中合作的具体水平

그래야 미중 양국이 막연한 기대감으로 우리를 끌어들이기 위해 압박하는 대신 실제로 협력이 가능한 분야를 두고 협의에 나설 것이다 This would encourage Washington and Beijing to come to the negotiating table with more realistic options rather than trying to draw us in with unrealistic expectations

只有这样,中美两国才不会以茫然的期待感来向韩国施压,并就真正可以合作的领域进行磋商

NYT “中, 해커 동원해 美코로나 백신자료 노려” China tries to hack COVID-19 research data, NYT reports

《纽约时报》:“中国利用黑客窥伺美国新冠疫苗资料”

전 세계가 신종 코로나바이러스 감염증(코로나19) 치료제 개발에 몰두하고 있는 가운데 중국 등 세계 10여 개국이 정부 차원에서 해커를 동원해 백신 관련 정보 쟁탈전을 벌이고 있다고 10일 뉴욕타임스(NYT)가 전했다 With the entire international community scrambling to find a cure to the novel type of coronavirus called COVID-19, some 10 countries including China are reportedly mobilizing hackers to get their hands on the information on COVID-19 vaccines, The New York Times reported on Sunday

《纽约时报》10日报道称,全世界都在埋头开发新型冠状病毒的治疗药物之际,中国等世界10多个国家正在政府层面动用黑客,展开有关疫苗的信息争夺战

미국 연방수사국(FBI)과 국토안보부는 조만간 “중국이 미국의 코로나19 치료제 및 백신 개발에 관한 연구 자료를 훔치려 하고 있다 미국의 관련 지식재산권과 공중보건 자료를 불법적 수단을 통해 획득하려 한다”는 경고문을 발표하기로 했다 “China is trying to steal the research data on the development of treatment and vaccines for COVID-19 from the United States It is aiming to get access to America’s intellectual property and public health data through illegal channels,” warned the FBI and the Department of Homeland Security

美国联邦调查局和国土安全部决定近期发布警告,警告“中国试图窃取美国新冠病毒治疗药物及疫苗开发相关研究资料企图通过非法手段获取美国的相关知识产权和公共卫生资料”

특히 중국이 정보 요원이 아닌 유학생, 교수, 연구원 등을 동원해 미 주요 대학과 민간 연구소의 코로나19 정보를 빼내려 하고 있다고 지적했다 They pointed out that Beijing is mobilizing students, professors, and researchers instead of agents to steal the information on COVID-19 from America’s universities and private research centers

警告特别指出,中国不是利用情报人员,而是动员留学生 教授 研究员等,试图窃取美国主要大学和民间研究所的新冠病毒情报

미국은 중국의 해외 인재 유치 프로젝트 ‘천인계획(千人計劃)’이 미국의 첨단 기술을 훔치려는 계획의 일환이라고 의심해 왔다 Washington has long held the suspicion that China’s “Thousand Talents Program” is part of the country’s broader scheme to poach America’s cutting-edge technologies

美国一直怀疑,中国吸引海外人才计划“千人计划”是窃取美国尖端科技计划的一部分

FBI는 조만간 주요 대학을 방문해 보안 강화를 촉구할 것으로 알려졌다 The FBI is planning to visit major universities in the U S to urge them to beef up security

据悉,联邦调查局将在近期访问主要大学,敦促加强保安

나노기술의 세계적 석학인 찰스 리버 미 하버드대 교수(61) 역시 1월 ‘천인계획’에 참여한 사실을 고의로 숨긴 혐의로 체포됐다 In January, Charles Lieber, a professor at Harvard University, was arrested for lying about his ties to the Chinese state-run recruitment program

纳米技术的世界级学者——美国哈佛大学教授查尔斯·利伯(61岁)1月份也因涉嫌故意隐瞒参与"千人计划"的事实而被捕

그는 2012∼2017년 코로나19 진원지로 꼽히는 후베이(湖北)성 우한이공대에서 수십억 원을 지원받았음에도 “참여 요청을 받은 적이 없다”고 거짓 진술을 했다 From 2012 to 2017, he was awarded millions of dollars from the Wuhan University of Technology but made a false statement about the payment he received

2012年至2017年,他在新冠病毒爆发地湖北省武汉理工大学获得数十亿韩元资助,但他谎称“从未收到过参与邀请”

도널드 트럼프 행정부는 줄곧 코로나19의 중국 유래설을 주장하며 중국과 맞서고 있다 U S President Donald Trump has been accusing China of being the epicenter of the coronavirus

特朗普政府一直主张新冠病毒“源自中国”,与中国针锋相对

중국 역시 거세게 반발하고 있어 해킹 논란이 가뜩이나 나쁜 미중 갈등을 더 악화시킬 것이라는 우려도 제기된다 Against this backdrop, China is retorting hard and experts voice the concern that the latest controversy might further fuel the bad blood between Washington and Beijing

中国也对此表示强烈反对。因此有人担心,黑客风波会进一步恶化本来就不好的美中矛盾

NYT는 한국, 이란, 베트남 등도 해커를 동원해 타국의 코로나19 정보 수집에 몰두하고 있다고 전했다 （no translation）

《纽约时报》报道说,韩国 伊朗 越南等国也动员黑客,正在收集其他国家的新冠病毒情报

특히 “한국 해커들이 세계보건기구(WHO)는 물론이고 북한, 일본, 미국 정부 관계자들의 이메일을 해킹해 정보를 수집하려 했다 （no translation）

报道称,特别是“韩国黑客不仅攻击世界卫生组织,也攻击朝鲜 日本 美国政府相关人士的电子邮件,试图收集情报

미국의 동맹국조차 미국의 통계를 의심하고 있음을 보여준다”고 보도했다 （no translation）

这表明连美国的盟国也在怀疑美国的统计数据”

美-中코로나 갈등으로 다시 무역전쟁 전운 U S -China conflicts on COVID-19 reignite tensions in global trade

美国与中国因新冠矛盾再起贸易战阴云……韩国应制定出口多元化等战略

수출처 다변화 등 전략짜야코스피 주가 지수가 4일 다시 1900선이 무너졌다 South Korea’s benchmark KOSPI has fallen below 1,900 points again on Monday

韩国综合股价指数(KOSPI)4日再次跌破1900点大关

그동안 소강상태를 보이던 미·중 무역갈등이 재개될지 모른다는 불안감으로 외국인 투자자들이 국내 주식시장에서 1조 원어치나 팔고 나갔기 때문이다 It is because foreign investors sold Korean stocks worth one trillion won due to the anxiety that the trade war between the U S and China in a state of lull could be resumed

因为此前一直呈现平稳状态的美中贸易矛盾有可能重新爆发的不安感,外国投资者在韩国股市抛售了1万亿韩元的股票

코로나19가 다른 나라에 비해 조기에 진정되고 있고, 경제활동도 조만간 본격적으로 재개될 것이라는 기대감을 가져온 한국경제에 찬물을 끼얹는 것이다 It threw a wet blanket to the South Korean economy, which started to have hopes that economic activities, would become more active soon as the country successfully flattened the COVID-19 curve compared to other countries

这给比其他国家更早平息了新冠疫情 带来经济活动也即将正式恢复的期待感的韩国经济泼了冷水

미중 무역전쟁 재발 가능성에 불을 붙인 것은 코로나19 확산에 대한 중국 책임론을 거론하며 1조 달러(약 1200조원)의 관세를 추가로 부과할 수 있다고 시사한 도널드 트럼프 미국 대통령의 발언이다 What triggered the possibility of a trade war between the world's two largest economies was U S President Donald Trump’s remarks that Washington may impose additional tariffs on one trillion dollars worth of Chinese goods, saying that China should be held responsible for the spread of the COVID-19 outbreak

美中出现再次爆发贸易战的可能性,是因为美国总统特朗普提及中国对新冠疫情扩散的责任论,暗示可以追加征收1万亿美元(约1200万亿韩元)的关税

한국의 수출은 이미 올 4월 실적이 작년 4월에 비해 23%나 급감했고 무역수지는 2012년 1월 이후 99개월 만에 처음 적자를 나타냈다 South Korea’s exports plummeted by 23 percent year-on-year in April, and the trade balance is in deficit for the first time in 99 months since January 2012

韩国出口方面,今年4月的业绩比去年4月锐减23%,贸易收支自2012年1月以后 时隔99个月首次出现逆差

트럼프 대통령이 11월 대선을 앞두고 유리한 여론형성을 위해 중국 때리기를 본격화할 경우 코로나 사태로 이미 수출시장과 글로벌 공급망에서 큰 타격을 입은 우리로서는 또 하나의 초대형 악재가 아닐 수 없다 If Trump starts to batter China to create public opinion favorable to him before the 2020 presidential election in November, it would be another mega-blow to South Korea, which was already severely affected in the exports market and global supply chains amid the prolonged pandemic

如果特朗普总统在11月大选前夕为了形成有利的舆论形势而大举敲打中国,对于因新冠疫情已在出口市场和全球供应链上遭受重创的韩国来说,无疑是又一个超大型不利因素

다소 희망적인 부분은 코로나 방역 과정에서 한국의 전반적인 국격이 높아졌고 경제적 저력 또한 세계시장에서 높이 평가받고 있다는 점이다 A flash of hope is that South Korea earned a good reputation in the curve-flattening process and its economic potential is highly assessed in the global market

多少有些希望的是,在防控新冠疫情的过程中,韩国的整体国格有所提高,经济潜力在世界市场上也得到高度评价

예컨대 앞으로 비대면 활동이 늘 것이라는 기대로 삼성전자와 SK하이닉스 2개 회사가 세계 시장에서 70% 이상을 차지하는 메모리반도체 D램의 4월 평균 가격이 39개월 만에 가장 큰 폭으로 올랐다 For instance, the price of DRAM memory chips recorded the highest increase in 39 months in April thanks to the projections that non face-to-face activities would increase in the future Samsung Electronics and SK Hynix accounted for more than 70 percent of the share in the global market last month

例如,由于期待今后非面对面活动增加,三星电子和SK海力士两家公司在世界市场上占据70%以上的存储器半导体DRAM,4月份平均价格创下了39个月以来的最大涨幅

반도체와 함께 또 다른 주력 수출상품 가운데 하나인 자동차분야에서도 현대차가 베트남 시장에서 일본 도요타를 제치고 판매 1위를 차지했다는 소식도 들린다 South Korea is also doing well in the automobile market as Hyundai Motor became the highest-selling brand in the Vietnamese market last month, exceeding Japan’s Toyota

与半导体一样,在另一个主要出口商品之一的汽车领域,也有消息称现代汽车在越南市场超过日本丰田,占据了销售第一位

그렇다고 해도 우리 앞에는 불안요소가 더 많다 The South Korean economy has still lots of stumbling blocks ahead

即使如此,我们面前的不安因素也更多

세계 경기 침체는 이제 시작일 뿐이고 미중 무역갈등은 적어도 미 대선이 끝나는 11월까지는 이어질 가능성이 높다 The global economy is at the beginning of a recession and the trade war is likely to be prolonged until the U S presidential election in November

世界经济衰退才刚刚开始,中美贸易矛盾至少将持续到美国大选结束的11月

그럴수록 우리가 자체적으로 할 수 있고 반드시 해야 할 일은 해야 한다 South Korea needs to do what we can and should do in this situation

越是这样,我们越要必须做可以自己做 必须做的事情

당장은 적극적 소비촉진을 통해 내수 경기를 살리고 각종 규제를 풀어 기업의 사기를 북돋우는 작업이다 The South Korean government should revitalize the domestic market by boosting consumption and raise morale of businesses by easing various regulations

眼下的工作是积极促进消费,搞活内需经济,解除各种限制,鼓舞企业的士气

길게는 보호무역 색채가 더욱 강해질 포스트 코로나시대의 수출환경에 맞춰 내수 비중을 높이고 수출 시장을 미국과 중국 중심에서 동남아 인도 유럽 등으로 다각화하는 노력에 박차를 가해야한다 In the long term, South Korea should increase the ratio of domestic consumption to cope with the post-COVID-19 era with stronger protectionism, while working on diversifying the exports market to the South East Asia, India, Europe and more

长期而言,为适应贸易保护色彩更加浓厚的“后新冠时代”的出口环境,韩国应努力提高内需比重,并加快加大努力,使出口市场从以美国和中国为中心向东南亚 印度 欧洲等多边化发展

‘코로나 우한硏발원설’ 목청 키우는 美 트럼프 “中끔찍한 실수”U S raises voice of criticizing Wuhan for causing COVID-19 outbreak

美国竭力宣称“新冠病毒武汉研究所起源说”,特朗普称“中国可怕的失误”

신종 코로나바이러스 감염증(코로나19)의 발원지를 둘러싼 미국과 중국 간의 갈등이 폭발 직전으로 치닫고 있다 Tensions between the U S and China are rising regarding the source of the COVID-19 outbreak

围绕新冠病毒的发源地,美国和中国之间的矛盾正在走向爆发之前

도널드 트럼프 미 대통령과 마이크 폼페이오 국무장관이 자극적 표현으로 중국 책임론을 제기하며 조사를 압박하자 중국은 ‘정치 쇼’라고 거칠게 반발했다 As U S President Donald Trump and Secretary of State Mike Pompeo have been blaming China for causing the outbreak and demanding an investigation with provocative remarks, China is harshly criticizing it as a “political show

美国总统特朗普和国务卿蓬佩奥以刺激性措辞提出“中国责任论”,并施压要求进行调查,中国对此强烈抗议,认为这是“政治秀”

CNN 등에 따르면 트럼프 대통령은 3일 ‘중국 연구소에서 바이러스가 나왔느냐’는 취재진의 질문을 받고 “중국이 끔찍한 실수를 저질렀다고 생각한다 ” “Personally, I think they made a horrible mistake, and they didn’t want to admit it,” Trump responded to a question on Sunday asking if he thinks the virus has originated from a Chinese research institute

据美国有线电视新闻网(CNN)等媒体报道,特朗普3日被记者问及“病毒是否出自中国研究所”时声称:“我认为中国犯下了可怕的错误

그들은 이를 덮으려 했지만 불을 끄지 못했고 실수를 인정하지 않는다”고 주장했다 “They tried to cover it, like a fire… They couldn’t put out the fire

他们试图掩盖此事,但未能灭火,不承认失误”

이어 “우한 연구소에서 무슨 일이 있었는지에 대한 보고를 받을 것이며 그것이 결정적일 것”이라고 강조했다 ” The president said he will get a report on the origins of the virus and how the Wuhan Institute of Virology might be involved, which would be “very conclusive

他还强调:“会收到武汉研究所发生了什么事情的报告,那是决定性的”

그는 지난달 30일에도 “바이러스가 중국 우한 연구소에서 유래했다는 증거를 봤다”며 관세 보복 등을 거론했다 ” He mentioned applying retaliatory tariffs on Thursday, saying that he has seen the evidence that the virus has originated from the Wuhan institute

他在4月30日也曾表示:“看到了病毒源自中国武汉研究所的证据”,提及了实施报复性关税等问题

같은 날 폼페이오 장관은 ABC방송 인터뷰에서 “코로나19가 우한의 연구소에서 나왔다는 것을 입증할 ‘엄청난 증거(enormous evidence)’가 있다”며 “중국 연구소의 실패로 세계가 바이러스에 노출된 것은 이번이 처음이 아니다”라고 지적했다 “There is enormous evidence that that’s where this began,” Pompeo said during an interview with ABC on Sunday, adding that this is not the first time that the world is exposed to viruses due to the failures of Chinese research institutes

同一天,蓬佩奥在接受美国广播公司(ABC)采访时指出,“有‘大量证据(enormousevidence)’可以证明,新冠病毒来自武汉研究所”,“中国研究所的失败导致全世界遭受新冠病毒,这不是第一次”

그는 중국이 의도적으로 바이러스를 퍼뜨렸는지, 우발적 사고였는지를 묻는 질문에 “알아야 할 것이 많다 To a question asking if the virus was spread intentionally by China or by mistake,

当记者问及中国是有意传播病毒还是偶发事故时,他说,“有很多事情需要了解

의문을 풀기 위해서라도 현지 조사가 필요하다 the secretary of state answered that there are many questions to be resolved, which require an on-site investigation

为了解开疑问,有必要进行现场调查

그곳에 가야 한다”며 중국 측을 압박했다 대통령의 최측근인 집권 공화당의 린지 그레이엄 상원의원 역시 “중국이 우한 연구소 조사에 협조할 때까지 제재를 가하는 법안을 마련했다”고 밝혔다 “We have come up with a bill to put sanctions on China until the country cooperates with an investigation into the Wuhan institute,” said Lindsey Graham, a Republican Senator and a close confidant of the president

应该去那里”,向中方施压特朗普总统最亲信的执政党共和党参议员林赛·格雷厄姆也表示:“制定了制裁中国的法案,直到中国协助武汉研究所调查为止”

미국 내에서는 코로나19 중국 발원설을 두고 우한 연구소가 생물학적 무기로 사용할 목적으로 바이러스를 만들었다는 설과 우한 연구소에서 사고로 우연히 유출됐다는 설이 나온다 In the U S , there are two theories regarding the origins of COVID-19 – the Wuhan institute produced the virus to use it as a biological weapon or the virus was leaked from the institute by accident

美国国内就新冠病毒“中国起源说”有两种说法,一是武汉研究所出于使用生物武器的目的制造了病毒,二是武汉研究所因事故偶然泄露

정치 매체 액시오스는 “생물학 무기설은 가능성이 낮고 사고설은 개연성이 있지만 직접 증거가 나오지 않았다”고 전했다 “The biological weapon hypothesis is unlikely while the accidental leakage scenario is more probable, but no direct evidence is not available yet,” said Axios, a website covering political news

政治媒体“Axios”报道说:“生物学武器说的可能性很低,事故说有一定可能性,但没有直接证据”

트럼프 대통령은 이날 중국의 ‘실수’라고 표현했고, 폼페이오 장관 역시 코로나19가 인공적으로 만들어진 것이 아니라는 것에 대해 “이를 의심할 이유가 없다”며 동의했다 Meanwhile, President Trump said it was Chinese “mistake” on Sunday and Secretary Pompeo also said there is no reason to doubt that COVID-19 has not been man-made

特朗普当天形容中国是“失误”,庞培也同意新冠病毒并非人工制造,“没有理由怀疑”

그럼에도 우발적 사고라면 어떤 식으로 유출됐는지를 알기 위해서라도 우한 연구소 조사가 불가피하다는 뜻을 강조한 것으로 해석된다 The two seem to have emphasized the necessity of an investigation into the Wuhan institute to find out how the virus has been leaked, even if it was an accident

分析认为,他们是在强调,即便如此,如果是偶发性事故,即使是为了了解是以何种方式泄露的,对武汉研究所的调查也不可避免

3일 AP통신에 따르면 미 국토안보부는 “중국 지도부가 의료물품 및 장비 비축을 위해 1월 초부터 의도적으로 코로나19의 심각성을 은폐했다”는 4장짜리 내부 보고서를 작성했다 The Associated Press reported on Sunday that Chinese leaders “intentionally concealed the severity” of the pandemic from the world in early January to stockpile medical supplies and equipment according to a four-page Department of Homeland Security intelligence report

据美联社3日报道,美国国土安全部3日制作了4页内部报告,内容为“中国领导层为储备医疗物品和设备,从1月初开始故意隐瞒了新冠病毒的严重性”

중국이 코로나19의 위험성을 세계보건기구(WHO)에 보고하는 것을 일부러 늦추면서 해외로부터 의료장비를 수입했고, 그 결과 올해 초 중국의 마스크 및 보호장갑 수입량이 급증했다는 것이다 The Chinese government deployed reporting on the dangers of COVID-19 to the World Health Organization (WHO) while importing medical supplies from foreign countries, leading to the country’s import surge of masks and protective gloves at the beginning of this year

也就是说,中国故意推迟向世界卫生组织报告新冠病毒的危险性,从而从国外进口医疗设备,结果今年初中国口罩及防护手套进口量猛增

양측은 대만의 WHO 회의 참석을 두고도 대립하고 있다 The U S and China are also confronting each other regarding Taiwan’s participation in a WHO meeting

双方就台湾参加世界卫星组织会议一事也针锋相对

WHO 최고 의결기관인 세계보건총회(WHA)는 18일 화상회의를 개최한다 The World Health Assembly (WHA), the highest policy-setting body of the WHO, will hold a video conference meeting on May 18

世卫组织最高决议机关世界卫生总会将于18日召开视频会议

미 국무부와 주유엔 미국대표부는 2일 트위터에 “대만의 WHO 가입을 지지한다”는 해시태그를 올렸다 The State Department and the U S representative at the United Nations posted a hashtag on Saturday, supporting Taiwan to become a member of the WHO

美国国务院和美国驻联合国代表部2日在推特上上传了“支持台湾加入世界卫生组织”的推特话题

미국의 주대만 대사관 역할을 하는 미국재대만협회(AIT) 역시 이날부터 매일 대만의 WHA 참여를 지지하는 글을 페이스북에 올릴 것이라고 밝혔다 The American Institute in Taiwan, which works as the Taiwanese embassy in the U S , announced on the same day that it will update daily posts on Facebook supporting Taiwan’s joining of the WHA

相当于美国驻台湾大使馆的美国在台协会也表示,“从当天开始,每天都会在脸书上传支持台湾参与世界卫生总会的文字”

중국은 “미국이 코로나19 이슈를 정치화하고 있다”고 강하게 반발했다 China is strongly opposing the U S ’s claim, saying that the U S is politicizing the COVID-19 issue

中国则认为,“美国正在把新冠问题政治化”,表示强烈反对

주제네바 중국대표부는 “이는 (대만이 중국의 일부라는) 하나의 중국 원칙을 위반한 것이고 대만 독립 세력에게 잘못된 신호를 줄 수 있다”고 비난했다 “It is in violation of the One-China policy that Taiwan is part of the country – it may send wrong signals to independent forces in Taiwan,” the Chinese representatives in Geneva said

中国驻日内瓦代表部批评称:“这违反了一个中国(台湾是中国的一部分))的原则,可能会给台独势力发出错误的信号”

트럼프 “코로나 중서 발생 증거봤다” 보복관세 시사 Trump says he’s seen evidence coronavirus started in China lab

特朗普暗示要征收报复关税,称“已经看到新冠病毒在中国发生的证据”

도널드 트럼프 미국 대통령이 “신종 코로나바이러스 감염증(코로나19)이 중국 우한(武漢) 바이러스연구소에서 발원했다는 증거를 봤다”고 주장하며 대중 관세를 검토하고 있다고 밝혔다 U S President Donald Trump indicated a possibility of imposing a tariff on China, maintaining that he had seen evidence that the Wuhan Institute of Virology is the origin of COVID-19

美国总统特朗普声称,“已经看到了新冠病毒源自中国武汉病毒研究所的证据”,表示正在研究对华关税

그가 재선을 위해 강력하고 구체적인 칼을 빼들었다는 분석이 제기된다 Critics see his message as a concrete and stringent attempt to win his re-election race

有分析认为,特朗普为了获得连任,亮出了强有力而具体的剑

올해 1월 1단계 무역합의를 통해 어렵사리 출구를 찾은 양국 무역전쟁이 다시 전면전으로 비화할 가능성도 배제할 수 없다 There is also a likelihood that the U S -China trade tensions can go up again despite their signing of a first-phase trade agreement in January

不能排除今年1月通过第一阶段贸易协议好不容易找到出口的两国贸易战争再次演变成全面战争的可能性

트럼프 대통령은 지난달 30일(현지 시간) 백악관 기자회견에서 ‘우한연구소 유래설에 대한 증거를 봤느냐’는 취재진 질문에 “그렇다 나는 봤다”고 두 차례 반복해서 답했다 When asked if President Trump has seen any concrete evidence that COVID-19 escaped from the virology institute in Wuhan in a press conference at the White House on Thursday (local time), he gave a clear yes twice

特朗普当地时间4月30日在白宫举行的记者招待会上,当记者问及“是否看到了武汉研究所源头说的证据”时,他两次重复回答:“是的看到了”

그는 “중국이 확산을 막지 못했거나 확산되도록 내버려뒀다”면서도 구체적인 증거는 밝히지 않았다 However, he did not disclose any detailed evidence although arguing that China failed to contain the spread of the virus or intentionally let it spread

他虽然表示,“中国没能阻止扩散或放任其扩散”,但并未透露具体证据

그는 관련 조사가 진행 중이라며 “머지않은 장래에 답을 얻을 것이다 그 결과가 중국에 대해 어떻게 느끼는지를 결정할 것”이라고 설명했다 “We should have the answer to that in the not-too-distant future and that will determine a lot how I feel about China,” President Trump said, adding that an investigation is underway

他解释称,相关调查正在进行中,“不久的将来就会得到答案，其结果将决定对中国有何感受”

그는 ‘중국을 응징하기 위해 채무 이행 중단을 검토하고 있느냐’는 질문에 “이 같은 일을 할 수 있지만 단지 관세를 부과함으로써 더 많은 돈을 얻을 것”이라고 말했다 President Trump also answered that merely introducing a tariff on China can bring more money to the United States, in response to a question if he would consider having the United States not fulfilling debt obligations to China as punishment for the virus

在回答“是否考虑停止履行债务以惩罚中国”的问题时,他说:“我可以做这样的事情,但只会通过征收关税获得更多的钱”

워싱턴포스트(WP)는 미국이 중국을 대상으로 ‘주권국은 타국 법정의 피고가 될 수 없다’는 국제법의 ‘주권 면제’ 조항을 박탈하는 방안을 논의하고 있다고 전했다 The U S government has reportedly been considering stripping China of sovereign immunity under the Foreign Sovereign Immunities Act (FISA), according to The Washington Post

《华盛顿邮报》报道称,美国正在针对中国讨论剥夺国际法中“主权国家不得成为他国法庭被告”的“主权豁免”条款

중국을 미 법정에 세워 손해배상을 받아내겠다는 의도다 It intends to sue China for coronavirus damages in a U S court

其意图是将中国告上美国法庭,以获得损害赔偿

CNN 역시 미국이 경제 제재, 채무상환 거부, 새 무역정책 등을 검토하고 있다고 보도했다 CNN also reported that Washington is thinking of economic sanctions, refusal of debt payment and new trade policy

美国有线电视新闻网(CNN)也报道说,美国正在研究经济制裁,拒绝偿还债务和新贸易政策等

미 17개 정보기관을 관장하는 국가정보국(DNI)은 이날 “정보기관들은 코로나바이러스가 사람이 만들거나 유전적으로 변형된 것이 아니라는 과학적 합의에 동의한다 발병이 우한연구소의 사고 결과인지, 감염된 동물과의 접촉으로 시작됐는지 판단하기 위해 조사하겠다”는 성명을 냈다 “The Intelligence Community also concurs with the wide scientific consensus that the COVID-19 virus was not manmade or genetically modified The IC will continue to rigorously examine emerging information and intelligence to determine whether the outbreak began through contact with infected animals or if it was the result of an accident at a laboratory in Wuhan,” the Office of Director of National Intelligence (DNI) said in a statement on Thursday Some experts see that the DNI, a U S governmental arm in charge of 17 national intelligence agencies, implicitly agreed that COVID-19 originated from Wuhan

掌管美国17个情报机构的国家情报局当天说:“情报机构同意科学上达成的协议,即新冠病毒并非由人制造或遗传性变形，为了判断发病是武汉研究所事故导致的结果,还是始于与受感染动物的接触,将进行调查”

사실상 ‘우한 발원설’을 지지했다는 관측이 나온다 （no translation）

事实上,有观察人士认为,这是在支持“武汉起源说”

수차례 ‘백신 개발에 최소 18개월이 걸린다’고 언급한 앤서니 파우치 알레르기전염병연구소(NIAID) 소장은 “내년 1월까지 수억 개의 백신 공급이 가능할 것”이라며 태도를 바꿨다 （no translation）

美国过敏症 传染病研究所所长安东尼·福奇曾多次表示,“疫苗的开发至少需要18个月”,如今改变态度说:“到明年1月为止,可以提供数亿支疫苗”

역시 대선과 무관하지 않다는 분석이 나온다 （no translation）

有分析认为,这也与大选不无关系

뉴욕타임스(NYT)는 “파우치 소장에 불만을 가진 대통령이 앨릭스 에이자 보건장관에게 직접 연내 개발을 지시했다 （no translation）

《纽约时报》报道说:“对福奇感到不满的特朗普总统亲自指示卫生部长亚历克斯·阿扎在年内开发

백신이 질병 및 사망을 야기해도 책임을 묻지 않는 식으로 개발을 앞당길 것”이라고 전했다 （no translation）

将加快疫苗的开发,即使疫苗引起疾病和死亡也不会追究其责任”

트럼프 행정부의 총공세는 코로나19에 따른 인명 피해 및 경제침체 장기화가 11월 대선에 악영향을 끼칠 것이란 우려 때문으로 풀이된다 （no translation）

据分析,特朗普政府的总攻势是因为担心新冠疫情造成的人员伤亡以及经济停滞的长期化会对11月的总统选举产生负面影响

반대파의 화살을 중국으로 돌리고 지지층을 결집시키기 위해 중국을 겨냥했다는 의미다 （no translation）

这意味着,他把反对派的矛头转向中国,为凝聚支持者而针对中国

트럼프 대통령은 지난달 29일 “중국이 나의 승리를 저지하기 위해 뭐든 할 것”이라고 주장했다 （no translation）

特朗普总统上个月29日主张:“中国为了阻止我的胜利,将用尽一切办法”

중국은 격렬히 반발했다 （no translation）

中国对此表示强烈反对

우첸(吳謙) 국방부 대변인은 지난달 30일 기자회견에서 “미 정치인이 책임을 회피하고 중국을 비난하는 것은 이기적이고 무책임하다”고 주장했다 （no translation）

中国国防部发言人吴谦4月30日在记者会上表示:“美国政客回避责任 指责中国,是自私和不负责任的行为”

전날 러위청(樂玉成) 외교부 부부장도 NBC 인터뷰에서 “중국에 근거 없는 혐의를 뒤집어씌우지 말라 （no translation）

前一天,中国外交部副部长乐玉成在接受美国全国广播公司(NBC)采访时表示:“不要把毫无根据的罪名转嫁给中国

중국에 배상금을 요구할 법적 근거가 없는데도 터무니없는 주장을 한다”며 “황당한 정치적 웃음거리”라고 일축했다 （no translation）

没有向中国要求赔偿的法律依据,却提出荒唐的主张”,“这是荒唐的政治笑话”

트럼프 “中에 코로나 배상책임 물을것 Pres Trump says to demand compensation for COVID-19 from China

特朗普:“将向中国追究新冠疫情赔偿责任……目前正在认真进行调查”

진지하게 조사 진행중”도널드 트럼프 미국 대통령이 “중국에 신종 코로나바이러스 감염증(코로나19) 확산에 대한 거액의 배상 책임을 묻겠다”고 밝혀 미중 공방이 가열되고 있다 As U S President Donald Trump said he plans to demand that China pay a "very substantial figure" as compensation for the coronavirus outbreak, tensions between the U S and China are rising

美国总统特朗普表示,“将向中国追究新冠病毒扩散的巨额赔偿责任”,美中攻防战正在升温

폴리티코 등에 따르면 트럼프 대통령은 27일 워싱턴 백악관 기자회견에서 미국이 독일처럼 중국에 배상을 청구할 수 있느냐는 질문에 “금액을 결정하지 못했지만 미국은 독일보다 더 많은 배상금을 요구할 수 있다”며 “코로나19로 인한 피해가 상당하다 이 피해는 미국에만 국한되는 게 아니라 전 세계적”이라고 답했다 최근 독일 최대 일간지 빌트는 중국에 코로나19로 인한 경제적 피해 배상금 1490억 유로(약 198조 원)를 청구해야 한다고 주장했다 “We have not determined the final amount It's very substantial We're talking about a lot more money than Germany's talking about,” President Trump answered to a question by a reporter about German newspaper Bild’s claim for alleged “invoice” of 149 billion euros to China for “coronavirus damage” during a White House press conference on Monday, according to Politico “If you look at the world, I mean, this is worldwide damage This is damage to the U S , but this is damage to the world

据《政治报》等媒体报道,特朗普27日在华盛顿白宫举行的记者招待会上,当被问及美国是否会像德国那样向中国提出赔偿时说:“虽然金额还没有决定,但美国可能会要求支付比德国更多的赔偿金”,“新冠病毒的损失相当大这一损失不仅仅局限于美国,而是全球性的”最近德国最大日报《图片报》主张,应该向中国要求1490亿欧元(约合198万亿韩元)的新冠病毒造成的经济损失赔偿

‘살균제 인체 주입’ 발언 논란으로 지난주 후반 코로나19 일일 정례 기자회견에 등장하지 않았던 트럼프 대통령은 이날 발언의 대부분을 중국 공격에 할애했다 그는 “중국이 코로나19 확산에 대한 책임을 져야 한다（no translation）

因“把杀菌剂注入人体”的发言引发争议,特朗普上周后半期没有出席新冠疫情例行记者会,他当天大部分发言都对中国进行了攻击他。再次强调:“中国应该对新冠病毒的扩散负责”

우리는 매우 진지하게 조사를 진행하고 있다”고 거듭 강조했다（no translation）

我们正在非常认真地进行调查

대중 강경파로 유명한 피터 나바로 백악관 무역제조업 정책국장도 폭스뉴스에 출연해 ‘중국이 미국에 저질 코로나19 검사장비를 수출했고 폭리를 취했다’고 비난했다 ” Peter Navarro, the White House’s Director of Trade and Manufacturing Policy and a well-known hardliner against China, appeared on Fox News and criticized China for exporting low-quality test kits for COVID-19 and making undue profits

以对华强硬派著称的白宫贸易制造业政策局局长彼得·纳瓦罗也参加福克斯新闻频道节目,指责“中国向美国出口劣质新冠病毒检察装备,并从中牟取暴利”

나바로 국장은 “이동 통제를 해제하고 미국인이 일터로 복귀하려면 코로나19 진단 검사를 늘려야 한다 He also said that more testing for the virus was vital to getting Americans currently in lockdown back to work but some Chinese test kits show incorrect results

纳瓦罗说:“要解除移动管制,让美国人重返工作岗位,必须增加新冠病毒诊断检查”

그런데 중국 장비로 검사하면 잘못된 결과가 나온다”며 “중국산 장비가 경제 정상화에 지장을 줄 것”이라고 지적했다 He argued that Chinese test kits will impact the normalization of the economy

但是,如果用中国设备进行检查,就会出现错误的结果”,“中国生产的设备会影响经济正常化”

국립보건원(NIH)은 24일 중국과 손잡고 코로나19 연구를 수행하던 비영리 연구소 ‘에코헬스얼라이언스’에 대한 자금 지원을 중단했다 （no translation）

美国国立保健院(NIH)24日中断了对与中国携手进行新冠病毒研究的非营利研究所“回声健康联盟”的资金援助

이 연구소는 박쥐가 사람에게 어떻게 코로나바이러스를 전파하는지를 연구해왔다 （no translation）

该研究所一直在研究蝙蝠如何向人类传播冠状病毒

미국 정부는 2015년부터 현재까지 370만 달러(약 46억 원)를 지원했지만 올해 보조금 중 아직 쓰이지 않은 약 37만 달러의 사용을 중지하라고 통보했다 （no translation）

美国政府通报说,从2015年至今,美国共提供了370万美元(约合46亿韩元)的援助,但今年补贴中尚未使用的约37万美元将被停止使用

폴리티코는 이 연구소가 중국 우한바이러스연구소와 손잡은 것이 트럼프 행정부의 심기를 거스른 것으로 보인다고 추측했다 （no translation）

《政治报》推测,该研究所与中国武汉病毒研究所联手,可能违背了特朗普政府的心意

미국에서는 우한바이러스연구소가 코로나바이러스를 고의로 만들어내거나 유출했다는 주장이 제기되고 있다 （no translation）

在美国,有人提出武汉病毒研究所故意制造或泄漏新冠病毒

피터 다스작 에코헬스얼라이언스 이사장은 “우한바이러스연구소와 협력했지만 돈을 주고받은 적은 없다”며 억울함을 호소했다 （no translation）

“回声健康联盟”理事长彼得·达斯萨克称,“虽然与武汉病毒研究所合作过,但从未有过金钱往来”,表示冤枉

트럼프 행정부는 최근 대만과의 협력도 부쩍 강화하고 있다 The Trump administration is also strengthening its cooperation with Taiwan

特朗普政府最近还加强了与台湾的合作

앨릭스 에이자 미 보건장관은 이날 천스중(陳時中) 대만 위생부장과 통화했다 Secretary of Health and Human Services Alex Azar talked to Taiwanese Minister of Health and Welfare Chen Shih-chung over the phone on Monday to reinforce cooperation between the two countries

美国卫生部长亚历克斯·阿扎当天与台湾“卫福部长”陈时中通了电话

코로나19 백신 및 치료제 개발 등에서 양국 협력을 강화하기 위한 목적이다 for the development of COVID-19 vaccines and treatments

其目的是加强双方在新冠病毒疫苗及治疗药物开发等方面的合作

블룸버그통신은 “두 장관이 30분간 대화를 나눴다 Bloomberg reported that the two leaders of health talked for 30 minutes,

彭博社评价说:“两人进行了30分钟的交谈

대만에 대한 미국의 강력한 지지를 보여준다”고 평가했다 which shows strong support of the U S for Taiwan

这显示了美国对台湾的有力支持”

대만 정부는 미국이 대만의 세계보건기구(WHO) 가입 및 국제보건 현안의 참여 기회를 늘려줄 것이라는 기대를 드러내고 있다 （no translation）

台湾当局期待美国增加台湾加入世界卫生组织及参与国际保健悬案的机会

중국 정부가 주장하는 ‘하나의 중국’ 원칙 때문에 대만은 WHO의 정식 회원국이 되지 못하고 ‘옵서버’ 지위만 갖고 있다 （no translation）

由于中国政府主张的“一个中国”原则,台湾不能成为世卫组织的正式成员国,只有“观察员”地位

대만은 친중 성향으로 유명한 테워드로스 거브러여수스 WHO 사무총장이 중국의 지지를 받아 2017년 7월 취임한 후 “WHO로부터 노골적인 홀대를 받고 있다 （no translation）

台湾声称,在以亲大陆倾向著称的世卫组织总干事谭德塞得到中国的支持在2017年7月就任后,“(台湾)受到世卫组织露骨的忽视

코로나19 발발 초기에도 제대로 된 정보를 공유받지 못했다”는 불만을 제기해왔다 （no translation）

新冠疫情爆发初期也没能得到正常的信息共享”,一直表示不满

하얼빈 71명 집단감염에 中, 다시 ‘準봉쇄조치’With 71 cases in Harbin, China takes lockdown measures again

哈尔滨71人集体感染,中国再次采取“准封锁措施”

중국이 신종 코로나바이러스 감염증(코로나19) 재확산을 막기 위해 인구 1085만 명인 헤이룽장(黑龍江)성 성도 하얼빈(哈爾濱)시에 대해 준(準)봉쇄 조치를 취했다 China has imposed lockdown on Harbin, the provincial capital of Heilongjiang province with 10. 85 million population to prevent the spread of COVID-19

为防止新冠病毒疫情再次扩散,中国对拥有1085万人口的黑龙江省省会哈尔滨市采取了准封锁措施

하얼빈시에서는 최근 해외에서 돌아온 감염자 1명이 71명을 연쇄·집단 감염시키는 사태가 발생했다 The city is suffering from an overseas infection that has spread to mass infection of 71 cases in the community

哈尔滨市最近发生了1名从海外回来的感染者导致71人连锁 集体感染的事件

하얼빈시 정부는 22일 “시내 모든 주거단지와 농촌마을의 출입구를 통제해 외부인과 외부인 차량의 진입을 금지한다”고 밝혔다 The municipal government of Harbin announced on Wednesday that it has banned entry to residential zones in the city and urban villages by people and vehicles registered elsewhere

哈尔滨市政府22日表示,“管制全市所有居民小区和农村村镇的出入口,禁止外来人员和外部车辆进入”

결혼식과 장례식도 금지했다 Weddings and funerals are also banned as well as large-scale events

婚礼和葬礼也被禁止

공연, 경기, 포럼, 전시 등 대형 행사도 중단시켰다 such as performances, sports events, forums, and exhibitions

演出 比赛 论坛 展览等大型活动也被叫停

중국은 그동안 “코로나19 통제에 성공했다”며 이동 통제 조치를 완화해 왔다 China had lifted isolation measures by assessing that it succeeded in controlling the virus pandemic

此前,中国一直表示“成功控制了新冠疫情”,放宽了移动管制措施

하지만 해외 유입에 이은 집단 감염이 다시 발생하면서 2차 유행 가능성이 우려되자 화들짝 놀란 중국 당국이 다시 강경 조치를 꺼내 든 것이다 However, as the mass infection caused by an imported case raised concerns on the possibility of a second outbreak, a wary Chinese government has imposed strict measures once again

但是,随着继海外流入病例之后再次发生集体感染,担心第二次流行的可能性,受惊吓的中国当局再次拿出了强硬措施

하얼빈시의 연쇄·집단 감염은 지난달 미국에서 홍콩과 베이징을 거쳐 돌아온 유학생 한(韓·여·22)모 씨로부터 시작됐다 The recent cluster case in Harbin started out with a Chinese student studying overseas named Han (female, 22), who returned home from the U S via Hong Kong and Beijing

哈尔滨市的连锁 集体感染始于上个月从美国经香港和北京回国的留学生韩某

가족과 이웃들이 감염됐고, 이들 중 일부가 방문한 하얼빈 시내 대형 병원 2곳에서 집단 감염이 발생했다 The virus spread to her family and neighbors, some of whom visited two major local hospitals, where the mass infection spread

其家人和邻居都受到了感染,其中一部分人访问的哈尔滨市内两家大型医院也发生了集体感染

이로 인해 하얼빈시뿐 아니라 랴오닝(遼寧)성 푸순(撫順)시와 네이멍구(內蒙古)자치구에서도 환자가 각각 1명씩 발생했다 This caused infections in Fushun of Liaoning and Inner Mongolia, both of which saw one confirmed case each

由此,不仅是哈尔滨市,辽宁省抚顺市和内蒙古自治区也分别出现了1名患者

하얼빈시 정부는 병원 내 집단 감염 당시 이곳에 있었던 4106명의 감염 여부를 조사하고 있다 The municipal government of Harbin is inspecting potential infections of 4,106 people who had been at the hospitals at that time

哈尔滨市政府正在对医院内集体感染当时在场的4106人进行是否感染的调查

특히 한 씨는 이달 초 상하이(上海)에도 다녀온 것으로 알려졌다 Han has reportedly travelled to Shanghai early this month as well

据悉,韩某本月初还去过上海

중국 내 코로나 재유행 범위가 확산될 수 있다는 우려가 나왔다 There are concerns that a massive outbreak could take place again in China

有人担心,新冠病毒的再流行范围可能会在中国内扩散

(（additional translation））The Chinese foreign ministry advised Wednesday people in China not to travel overseas and those who are outside of the nation not to travel to other countries

（增译)中国外交部21日下午表示:“在国内的人不要去海外旅行身在海外的人不要移动到其他国家"

(（additional translation））This is seen as a measure to prevent cross border movement of Chinese people ahead of the upcoming Labor Day holiday on May 1

（增译)这可以解释为,由于情况不妙,在5月1日劳动节长假之前,中国宣布了禁止中国人跨国移动的措施

거세지는 ‘中코로나 책임론’ 메르켈 “中투명하게 공개해야” China under growing criticism for COVID-19 outbreak

愈演愈烈的“中国新冠疫情责任论”……默克尔:“中国应透明地予以公开”

신종 코로나바이러스 감염증(코로나19) 확산에 대한 중국의 책임론이 갈수록 거세지고 있다 A sentiment that China is responsible for the outbreak of COVID-19 is growing

对于新冠疫情的扩散,“中国责任论”正在愈演愈烈

미국에 이어 유럽 각국 정상들도 코로나19 사태의 축소 의혹을 명확히 밝히라고 촉구했다 The leaders of the U S and some European countries are calling for a clear explanation about a suspicion that China had tried to reduce the extent of the outbreak

继美国之后,欧洲各国首脑也敦促中国明确查清瞒报新冠疫情的疑惑

로이터통신 등에 따르면 앙겔라 메르켈 독일 총리는 20일 “중국 정부는 코로나바이러스 기원과 초기 확산을 투명하게 공개해야 한다 According to Reuters, German Chancellor Angela Merkel said on Monday that the Chinese government should transparently reveal the origin and the initial spread of COVID-19

据路透社等媒体报道,德国总理默克尔20日表示:“中国政府应该透明地公开新冠病毒的起源和初期扩散

그래야 세계 모두가 교훈을 얻어 상황이 나아질 것”이라고 밝혔다 so that the entire world can learn lessons and make improvements

只有这样,全世界才能吸取教训,情况才会好转”

독일에서는 최근 대표 일간지인 빌트 등 언론을 통해 ‘코로나 중국 책임론’이 확산되기도 했다 The sentiment to put responsibility for the virus on China has been growing recently among German media companies, including tabloid newspaper Bild

最近在德国,“新冠疫情中国责任论”还通过主流媒体《图片报》等媒体扩散开来

에마뉘엘 마크롱 프랑스 대통령은 앞서 17일 파이낸셜타임스(FT)와의 인터뷰에서 “중국이 (코로나19 사태에) 더 잘 대응했다는 건 순진한 생각”이라며 “우리가 알 수 없는 일들이 벌어지고 있다”고 지적했다 “Let’s not be so naive as to say China has been much better at handling this There are clearly things that have happened that we don’t know about,” said French President Emmanuel Macron during an interview with The Financial Times on Friday

法国总统马克龙17日接受《金融时报》采访时指出:“认为中国妥善地应对了(新冠疫情),这是天真的想法”,“正在发生我们不知道的事情”

도미닉 라브 영국 외교장관도 16일 기자회견에서 “중국에서 어떻게 바이러스가 퍼졌는지 철저한 분석이 필요하다”고 밝혔다 U K Secretary of State for Foreign Affairs Dominic Raab also said during a press conference that a thorough investigation of how the virus has spread in China is needed

英国外交大臣拉布16日在记者会上也表示,“有必要对病毒在中国是如何传播的进行彻底的分析”

유럽은 미국과 달리 코로나19 사태와 관련해 중국 비판을 자제해 왔다 Unlike the U S , European countries had been refraining from criticizing China

欧洲与美国不同,在新冠疫情上一直克制对中国的批评

2017년 도널드 트럼프 대통령 취임 이후 미국과의 관계가 벌어지면서 중국에 대한 의존도가 커진 탓이다 until recently regarding the spread of the virus as their relations with the U S have grown apart while their dependence on China has increased since the election of U S President Donald Trump

这是因为2017年特朗普总统就任后,随着与美国的关系逐渐疏远,欧洲对中国的依赖度也随之增加

유럽의 기조가 바뀐 데 대해 미국 외교전문매체 포린폴리시는 “중국이 발표한 코로나 관련 데이터들은 신뢰를 잃었다 이를 토대로 세운 각국의 코로나 대책도 잘못 만들어졌다”고 설명했다 Regarding such a shift among European countries towards China, U S -based magazine Foreign Policy explained that COVID-19 data published by China has lost its reliability and the virus response measures of other countries based on such data are also misinformed

对于欧洲基调的改变,美国外交专业媒体《外交政策》表示:“中国公布的有关新冠疫情的数据失去了信任,以此为基础制定的各国新冠疫情对策也是错误的。“

중국은 코로나 발병 시기와 누적 사망자 숫자 등을 명확히 밝히지 않아 불신을 키운 바 있다 Distrust on China has grown as the country has failed to reveal the exact timing of the virus outbreak and the number of total deaths

中国没有明确地表明新冠疫情发病时间和累计死亡人数等,因此曾引起不信任

코로나가 자국에서 발병했음에도 자국 체제를 선전하고 다른 국가를 깎아내린 점도 반감을 샀다 （no translation）

新冠疫情在本国暴发后还宣传本国体制,贬低其他国家的做法也引起了反感

최근 주프랑스 중국 대사는 홈페이지에 “서구의 코로나 대응은 느림보”라는 글을 올려 프랑스 정부의 항의를 받았다 （no translation）

近日,中国驻法大使在其网站上发表文章《西欧应对新冠疫情行动迟缓》,遭到法国政府的抗议

인도적 명분을 내세워 의료물품을 지원해 의도적으로 유럽연합(EU)의 분열을 노린다는 지적도 있다 （no translation）

也有人指出,中国打着人道主义的旗号支援医疗物品,有意挑起欧盟的分裂

일각에서는 서구사회에 대한 가짜뉴스를 퍼뜨린다는 의혹도 나온다 （no translation）

部分人还怀疑中国在西方社会散布假新闻

자국 내 여론 환기를 위해 중국으로 화살을 돌리고 있다는 분석도 나온다 （no translation）

也有分析认为,为了唤起国内舆论,一些国家将矛头转向了中国

미국을 비롯해 프랑스 독일 영국 등의 코로나 사망자만 수만 명에 달할 정도로 피해가 크다 （no translation）

包括美国在内,法国 德国 英国等国的新冠疫情死亡人数达数万人,损失巨大

FT는 “서방 정치인들은 베이징을 공격해 자신들의 실패를 피하면서 비판 대상을 분산시키고 있다”고 전했다 （no translation）

《金融时报》报道说:“西方政客攻击北京,避免自己的失败,同时分散批评对象”

중국에 대한 비판이 전 세계로 확산되자 중국을 옹호해 온 세계보건기구(WHO)는 난처한 입장에 빠졌다 As criticism on China grows throughout the world, the World Health Organization, which has been defending China, has found itself in a tricky situation

随着对中国的批评扩散到全世界,一直拥护中国的世界卫生组织陷入了尴尬的立场

테워드로스 아드하놈 거브러여수스 WHO 사무총장은 20일 스위스 제네바 본부 언론 브리핑에서 “우리는 미국에 (정보를) 감춘 것이 아무것도 없다”고 강조했다 During a press conference held on Monday at the organization’s headquarters in Geneva, Switzerland, WHO Director-General Tedros Adhanom Ghebreyesus said they have not hidden anything to the U S

世卫组织总干事谭德塞20日在瑞士日内瓦总部的新闻发布会上强调:“我们从未向美国隐瞒(任何信息)”

中1분기―6. 8% 사상첫 마이너스 성장 China’s economy records historic quarterly plunge due to coronavirus

中国第一季度经济增长率为-6.8%,历史上第一次负增长

신종 코로나바이러스 감염증(코로나19) 사태 충격으로 중국의 1분기(1∼3월) 경제성장률이 통계 작성 이후 처음으로 마이너스 성장을 기록했다 Amid the spread of COVID-19, China has recorded a negative growth in the first quarter of the year for the first time since related statistics were first officially gathered

受新冠疫情冲击,中国第一季度(1～3月)的经济增长率自统计以来首次出现负增长

중국 정부는 고강도 경기 부양 조치를 예고했다 Accordingly, the Chinese government has signaled a tough economic stimulus program

中国政府预告将采取高强度经济刺激措施

중국 국가통계국 마오성융(毛盛勇) 대변인은 17일 기자회견에서 “1분기 국내총생산(GDP) 총액이 20조6504억 위안(약 3554조 원)으로 지난해 같은 기간에 비해 6. 8% 감소했다”고 밝혔다 Mao Shengyong, spokesperson of the National Bureau of Statistics of China, said in a press conference on Friday that China’s GDP decreased by 6. 8 percent to 20. 65 trillion yuan (approximately 3,554 trillion won) in the first quarter of the year on a year-on-year basis,

中国国家统计局发言人毛盛勇17日在记者会上表示:“第一季度国内生产总值(GDP)总额为20.6504万亿元,比去年同期减少了6.8%”

중국이 분기별 경제성장률을 발표하기 시작한 1992년 이후 28년 만의 첫 마이너스 성장이자 최저치다 marking both the lowest and the first negative figure since Beijing first announced quarterly growth rates in 1992

这是中国自1992年开始公布季度经济增长率以来,28年来的首次负增长和最低值

지난해 4분기 경제성장률인 6%에 비해서는 12. 8%포인트 급락한 것이기도 하다 The Chinese economy experienced a historic drop of 12. 8 percent points compared to the six percent increase during the fourth quarter of last year Previously,

这与去年第四季度6%的经济增长率相比,下降了12.8个百分点

중국의 연간 경제성장률이 마이너스를 기록한 것은 문화대혁명으로 중국 경제가 붕괴 직전까지 갔던 1976년(―1. 6%)이 마지막이었다 China recorded a negative yearly growth at -1. 6 percent in 1976 when the Cultural Revolution drove up risks of an economic meltdown

因文化大革命中国经济濒临崩溃的1976年(-1.6%)是最后一次出现负增长

올해 전체 경제성장률이 마이너스를 기록한다면 44년 만이다 It can repeat itself in 44 years if this year ends up with a negative growth

如果今年整体经济增长率出现负增长,这将是44年来首次出现负增长

마 대변인은 “1분기 2차산업(제조업) 생산이 9. 6%, 3차산업(서비스업) 생산이 5. 2% 감소했다”고 말했다 “The total production decreased by 9. 6 percent and 5. 2 percent in manufacturing and service industries, respectively, during the first quarter of the year,” said Mao

毛盛勇说,一季度第二产业(制造业)产值下降9.6%,第三产业(服务业)产值下降5.2%

1분기 산업생산은 지난해 같은 기간에 비해 8. 4% 하락했고, 소비 수요를 보여주는 소매 판매는 19%나 하락했다 Industrial production went down by 8. 4 percent while retail sales, a barometer of consumption demand, plummeted by a whopping 19 percent

一季度工业生产同比下降8.4%,反映消费需求的零售销售同比下降19%

중국 외교부는 21일 오후 “국내에 있는 사람들은 해외여행을 하지 말라 （no translation）

（漏译）

해외에 있는 사람은 다른 나라로 이동하지 말라”고 발표했다 （no translation）

（漏译）

상황이 심상치 않자 다음 달 1일 노동절 연휴를 앞두고 중국인들의 국경 간 이동 금지 조치를 발표한 것으로 해석된다（no translation）

（漏译）

中우한 코로나 사망자 축소 의혹 사실로 China’s cover-up of COVID-19 death toll in Wuhan is revealed

中国武汉市新冠肺炎死亡病例核增1290例

중국이 신종 코로나바이러스 감염증(코로나19) 사망자 수를 축소해 왔다는 의혹이 사실로 드러났다 The suspicion that the Chinese government has been underreporting the number of deaths from COVID-19 has been confirmed

中国缩小新型冠状病毒感染症(Covid19病毒)死亡人数的疑惑被证实

중국 신화(新華)통신에 따르면 17일 후베이(湖北)성 우한(武漢)시 정부는 16일까지 우한시의 누적 사망자 수가 기존 공식 발표보다 1290명이 많은 3869명이라고 밝혔다 According to Xinhua News Agency, the Wuhan municipal government in Hubei province reported on Friday that the total number of deaths in the city as of Thursday is 3,869, which is 1,290 higher than the previous official figure

据中国《新华通讯社》报道称,17日湖北省武汉市政府对外表示,截至16日,武汉市的新冠肺炎累计死亡人数为3869人,比此前官方公布的死亡人数多出1290人

우한시의 누적 확진자 수는 기존보다 325명 많은 5만333명이라고 공개했다 The city’s number of confirmed cases is also 325 higher than the previous figure at 50,333

公告称,武汉市累计确诊病例50333例,比原来增加325例

중국 보건 당국인 국가위생건강위원회(위건위)가 같은 날 2시간 전 발표한 16일까지의 우한시 누적 사망자 수는 2579명이었다 The death toll in Wuhan as of Thursday announced by the National Health Commission of China two hours earlier on Friday was 2,579

中国保健当局国家卫生健康委员会(简称“卫建委”)在同一天公布,截至16日,武汉市的新冠肺炎累计死亡人数为2579人

기존 공식 누적 사망자의 절반에 해당하는 숫자가 지금까지 집계에 포함되지 않은 셈이 된다 This means deaths equivalent to about 50 percent of the previously reported figure had not been included until now

这相当于现有官方累计死亡人数的一半,也就是说,到目前为止,这一数字还没有被列入统计数据

우한시 당국은 시 위건위와 질병예방통제센터, 공안(경찰) 등 여러 부서가 병원, 양로원, 장례 정보 시스템 등을 조사했다고 밝혔다 Control and Prevention, and the Public Security Bureau have worked together to come up with the new figure based on data from hospitals, nursing homes, funeral data systems, etc

武汉市政府表示,市建委 疾病预防控制中心和公安等多部门对医院 养老院 殡葬信息系统等进行了调查

우한시 당국은 △코로나19 사태 초기 환자 급증으로 환자들이 입원하지 못한 채 집에서 사망했고 △병원 과부하로 의료진의 지연 보고, 보고 누락, 오보(誤報) 현상이 나타났으며 △사망자 정보가 부정확해 중복되거나 잘못 보고 됐기 때문이라고 해명했다 The city authorities explained that such a gap in death toll has been caused by some patients died at home without having been treated in hospitals due to the surge of new cases in the early stage of the COVID-19 outbreak during the height of their treating efforts, hospitals were operating beyond their capacities and medical staff was preoccupied with saving and treating patients, resulting in belated, missed and mistaken reporting and the registered information of some of the deceased patients was incomplete, and there were repetitions and mistakes in the reporting

武汉市政府解释说,在新冠肺炎疫情初期,由于患者剧增,导致患者无法住院在家中死亡,而且由于医院超负荷运转,医疗人员忙于救治,客观上存在迟报 漏报 误报现象,导致死亡信息不准确

하지만 당국이 책임을 회피한 채 사망자 수 누락의 책임을 일선 의료진에게 떠넘긴 것 아니냐는 비판이 나왔다 This, however, led to criticism that the authorities are shifting the responsibility for unreported deaths to health care providers in the front line

但是,有人批评当局回避责任,把漏报死亡人数的责任推给一线医护人员

중국의 코로나19 통계는 그동안 오락가락 집계와 은폐 의혹으로 불신이 컸다 The distrust of the Chinese government’s COVID-19 statistics has been growing as figures were revised at times and suspicion for the government’s cover-up has been raised

此前,中国的新冠肺炎统计数据变化无常,被怀疑隐瞒事实,引起公众的不信任

중국 매체 차이신(財新)은 최근 공식 사망자 수보다 최소 2배 많은 유골함이 운반됐다고 의혹을 제기했다 Chinese media Caixin recently reported that the number of urns shipped for COVID-19 deaths in Wuhan looked to be more than double the city’s death toll

中国媒体《财新》最近质疑称,运走的骨灰盒至少是官方死亡人数的两倍

中기습 입국금지에 기업인 등 대혼란 China bars entry by almost all foreigners to contain virus pandemic

中国奇袭式禁止入境,导致企业人士等乱成一团

중국이 신종 코로나바이러스 감염증(코로나19) 방역을 이유로 사실상 국경을 봉쇄하는 초강경 조치를 발표했다 China has announced an extraordinarily strong measure of effectively blocking the border at the pretext of containing the coronavirus pandemic

中国以防控新冠肺炎疫情为由,事实上宣布了封锁国境的超强硬措施

중국 현지에서 생산 공장 및 판매법인을 운영하고 있는 국내 기업뿐 아니라 현재 중국 밖에서 머물고 있던 한국 교민 및 유학생들도 중국 입국이 사실상 불가능해지면서 큰 혼란을 피할 수 없게 됐다 Not only South Korean companies that operate production plants and sales units in mainland China but also South Korean nationals living there and students of Chinese universities who are currently staying outside China have been denied entry, which will cause inevitably significant chaos and confusion to them

不仅是在中国当地运营生产工厂及销售法人的国内企业,目前停留在中国以外的韩国侨民及留学生也因为无法进入中国而不可避免地出现混乱

이날 중국 외교부와 국가이민관리국은 28일 0시를 기준으로 중국 체류 비자와 거류허가증을 가진 외국인도 중국 입국을 금지하기로 했다 The Chinese ministry of foreign affairs and the national immigration bureau have decided to ban foreigners with visas for long-term stay and residential permits from entering China, effective midnight Saturday

当天,中国外交部和国家移民管理局决定,以28日零时为标准,禁止持中国居留签证和居留许可证的外国人进入中国

중국은 경제무역, 과학기술 활동 및 긴급한 인도주의 사유가 있으면 현지 중국 대사관, 영사관에 예외적으로 비자를 신청할 수 있다고 밝혔다 China said people could still apply for visas through local Chinese embassies and consulates if they have justification for a visit due to economy and trade, science and technology activities and urgent humanitarian reasons

中国表示,如有经贸 科技活动及紧急人道主义原因,可向当地驻华使馆 领事馆例外申请签证

하지만 주중한국대사관은 “중국 측에 어떤 경우에 예외 조치로 비자를 신청할 수 있는지 구체적인 설명을 요구했지만 중국 측이 분명한 답을 하지 않았다”고 전했다 However, the South Korean Embassy in China said, "We have asked Beijing for a detailed explanation about the circumstances in which foreigners can apply for a visa as an exception, but the Chinese government has not given any clear answer yet

但韩国驻华大使馆表示:“已经要求中方说明在什么情况下可以作为例外措施申请签证,但中方没有给出明确的答复”

중국 입국 금지 조치는 시진핑(習近平) 중국 국가주석을 포함한 주요 20개국(G20) 정상들이 화상 정상회의를 끝낸 직후 공동성명을 통해 " The entry ban into China was announced only one hour after the leaders of the G20 countries including Chinese President Xi Jinping issued a joint statement shortly after their video summit

包括中国国家主席习近平在内的二十国集团领导人特别视频峰会结束后不久发表联合声明称,“为了促进国际贸易,将以避免在国家间移动和贸易方面造成不必要障碍的方式进行合作”

“국제무역을 촉진하고 국가 간 이동과 무역에 불필요한 장애를 유발하지 않는 방식으로 대응하기 위해 협력할 것”이라고 밝힌 지 불과 약 1시간여 만에 발표됐다 The joint statement read, "We will cooperate to respond (to the pandemic) in a way that will promote international trade, and that will not cause unnecessary obstacles to travel and trade between countries

中国的措施是在上述声明发布一个多小时后发布的

중국의 기습적 입국금지 조치에 한국 정부도 적잖게 당황하는 분위기다 （no translation）

对于中国突然采取的禁止入境措施,韩国政府也感到非常惊慌

외교 고위당국자는 27일 기자들과 만나 “어제(26일) 갑작스럽게 입국금지를 통보 받았다 （no translation）

韩国外交高层官员27日对记者说:“昨天(26日)突然接到了禁止入境的通知

사전에 통보를 받지 못한 것에 대해 유감스럽게 생각한다”고 말했다 （no translation）

我对事先没有接到通知感到遗憾”

美확진자 8만5000명 中넘어 세계 최다 V S overtakes China for most confirmed COVID-19 cases

美国新冠肺炎确诊病例已超8.5万

26일(현지 시간) 미국의 신종 코로나바이러스 감염증(코로나19) 확진자 수가 8만5000명을 넘어서며 세계 최대 감염국이 됐다 The number of COVID-19 cases in the U S surpassed 85,000 on Thursday (local time), making the U S the country with the highest number of confirmed cases in the world

超越中国成世界最大感染国26日(当地时间),美国确诊的新型冠状病毒感染症(COVID19病毒)患者人数超过8.5万名,成为了世界最大的感染国

1월 21일 미국에서 첫 환자가 발생한 후 65일 만이다 It has been 65 days since the first COVID-19 case was reported in the country on Jan 21

自1月21日美国出现首例患者后,已经过了65天

글로벌 통계 사이트 월드오미터 등에 따르면 이날 미국 확진자 수는 전일 대비 1만6939명 증가한 8만5520명으로 중국(8만1340명)을 앞질렀다 According to global statistics site Worldometer, the number of new confirmed cases in the nation rose by 16,939 on Thursday, increasing the total number to 85,520, which is larger than China’s 81,340

据全球统计网站“Worldometer”实时统计数据显示,截至当天美国确诊患者为8.5520万人,比前一日增加了1.6939万人,超过了中国(8.1340万人)

사망자는 261명 증가한 1297명이다 The death toll climbed by 261 to reach 1,297 on the day

死亡人数增加了261人,达到1297人

이달 19일 1만 명을 돌파한 지 1주일 만에 8만 명을 넘어서는 등 증가 속도가 가파르다 The novel coronavirus is spreading at a rapid pace in the U S , with the number of confirmed cases in the country surpassing 80,000 a week after the total number reached 10,000 on last Thursday

自本月19日突破1万人大关后,短短一周时间,这一数字就超过了8万人,增速异常迅猛

미국 최대 도시 뉴욕이 속한 뉴욕주 환자가 3만7258명에 달한다 The New York state has 37,258 cases

美国最大城市纽约所属的纽约州患者达3.7258万人

데버라 벅스 백악관 코로나19 책임자는 “미 전체 감염자의 55%가 뉴욕과 인근 뉴저지주에서 발생했다 White House Coronavirus Task Force Coordinator, Deborah Birx said that 55% of those infected have come out of New York and New Jersey,

白宫新冠肺炎负责人戴伯拉?伯克斯说:“全美55%的感染者都在纽约和附近的新泽西州

시카고와 디트로이트의 환자 급증세도 예사롭지 않다”고 밝혔다 adding that Chicago and Detroit are also seeing a surge in new coronavirus cases

芝加哥和底特律的患者剧增趋势也非同寻常”

의료진 및 의료용품 부족도 심각해 이날 앤드루 쿠오모 뉴욕주지사는 산소호흡기 1개를 환자 2명이 함께 사용하는 방안을 승인했다 New York Gov Andrew Cuomo on Thursday approved hospitals to treat two patients with one ventilator, reflecting a lack of medical staff and equipment in the state

由于医护人员及医疗用品严重短缺,纽约州长安德鲁?库奥莫当天批准了两名患者共同使用一个氧气呼吸器的方案

블룸버그통신은 도널드 트럼프 행정부의 안이한 현실 인식, 허술한 초동대처가 현 상황을 야기했다며 “코로나19는 트럼프의 베트남전”이라고 질타했다 Bloomberg criticized the Trump administration for its poor judgement of the situation and lack of initial preparation, adding, “The coronavirus isn’t Trump’s Katrina It’s his Vietnam ”

彭博社指责说,唐纳德?特朗普政府安逸的现实认识 松懈的初期应对方式导致了目前的状况,并称“新冠肺炎疫情是特朗普的越战”

하지만 트럼프 대통령은 이날 기자회견에서도 “다음 달 12일 부활절 전 코로나19 확산 방지를 위한 사회적 거리 두기 조치를 풀겠다”는 기존 입장을 고수해 비판이 일고 있다 President Trump came under fire once again by saying he wants to reopen America before Easter, which falls on April 12, during a press conference

但是,特朗普总统在当天举行的记者招待会上依旧坚持“在下月12日复活节前解除为防止新冠肺炎疫情扩散的社会距离”的原有立场,受到了批评

이탈리아(8만589명)도 감염자 증가 속도가 빨라 조만간 중국을 앞지를 것으로 전망된다 （no translation）

意大利(8.0589万名)感染者的增加速度也很快,预计不久后会超过中国

일본 감염자는 하루 만에 역대 최고치인 96명 증가해 2099명(다이아몬드 프린세스호 포함)으로 늘었다 （no translation）

日本感染者在一天内增加了96名,新增确诊患者人数创历史新高,总患者人数达到了2099名(包括钻石公主号)

세계화 최대 수혜자 중국에 닥친 2차 위기최근 중국 소셜네트워크서비스(SNS) 웨이보에서는 이탈리아에서 유학 중인 중국인 야오야오(瑤瑤·가명) 씨의 귀국기가 화제다 Another crisis awaits China, the biggest beneficiary of globalization All eyes have recently been on a Chinese student named Yao Yao across Chinese social media platform Weibo, who successfully came back home from Italy where he studied

全球化最大受益者中国遭遇的第二次危机最近在中国社交媒体微博上,在意大利留学的中国人瑶瑶(化名)的回国记成为了热门话题

이탈리아의 신종 코로나바이러스 감염증(코로나19) 상황이 심각해지자 그는 고향인 광둥(廣東)성 선전으로 돌아가기로 결심했다 With the COVID-19 pandemic worsening across Italy, he decided to return to his hometown, Shenzhen in Guangdong Province, China

意大利新冠肺炎疫情趋于严重后,瑶瑶决定返回家乡广东深圳

아부다비, 베이징(北京)을 거쳐 선전에 도착할 때까지 28시간을 야오야오 씨는 아무것도 먹지도 마시지도 않고 마스크를 한 번도 벗지 않은 채 버텼다 Yao Yao did neither eat nor drink anything with his mask on for 28 hours all the way from Italy to his final destination via Abu Dhabi and Beijing

从阿布扎比 北京直到到达深圳,整整28个小时,瑶瑶不吃不喝,也没有摘过口罩,坚持了下来

야오야오 씨의 웃지 못할 사연에 중국 누리꾼들은 “교과서식 귀국”이라며 박수를 쳤다 His pitiful story has garnered widespread support online being described as a so-called “textbook way of return”

对于瑶瑶令人哭笑不得的遭遇,中国网友拍手叫好:“教科书式回国”

하지만 그처럼 “중국이 가장 안전하다”며 유럽과 미국을 ‘탈출’하는 중국인이 크게 늘자 이들로 인한 코로나19 역유입도 증가세다 However, the number of coronavirus infections from overseas are worryingly on the rise in China due to an increasing number of Chinese citizens evacuating European countries and the United States in the belief that China is a safer place to stay

但是,像他这样认为“中国最安全”并“逃离”欧美的中国人大幅增加后,由他们造成的新冠肺炎患者回流现象也呈上升趋势

중국 내 코로나19 신규 감염자 수가 ‘0’이라고 선전하던 중국 당국이 화들짝 놀랐다 The unexpected development has puzzled Chinese health authorities, which have confidently boasted about having none of new infections reported at home

这令宣传中国国内新冠肺炎新增病例为“零”的中国当局大吃一惊

이에 중국 당국은 입국 통제 조치를 전면화했다 In response, the Chinese government has put tight entry control in place

对此,中国当局全面采取了入境管制措施

외국발 베이징행 항공기들이 베이징에 바로 착륙하지 못하게 하는 제한 조치도 시작했다 while ordering international flights to Beijing to arrive at an airport near its capital city instead

中国还开始实施限制措施,禁止外国飞往北京的飞机直接降落在北京

중국 상황이 심각할 때 중국에 문을 걸어 잠근 세계 각국을 중국이 비판했던 것과는 상반된다 The recent prohibitive measures are not line with Beijing’s criticism over other countries closing the door to Chinese citizens at the peak of the pandemic across China

这与中国在形势严峻时对中国关上大门的世界各国进行批评的态度截然相反

하늘길이 통제되자 중국 항공업체들의 타격이 현실화됐다 A series of aviation limits have wreaked havoc on major Chinese airlines,

空中通道被控制后,中国航空企业受到的打击成为现实

지난달 에어차이나 등 중국 대표 항공사들의 승객 수가 지난해 같은 기간에 비해 80% 이상 급감했다 including Air China with the number of their passengers in February nosediving over a whopping 80 percent on a year-on-year basis

上月,中国国际航空公司等中国代表性航空公司的乘客数量比去年同期骤减八成以上

코로나19로 세계 각국이 국경을 닫거나 통제하자 글로벌 공급망이 타격을 입기 시작했다 Added to that, the global supply chain is suffering from the ever-tightening of border controls across the world

新冠肺炎疫情导致世界各国关闭或控制边境,全球供应链开始受到冲击

코로나19 확산세가 멈춘 중국은 공장을 다시 가동한다 It is a relief that China-based factories have resumed production since the slowdown of the virus at home

停止新冠肺炎疫情扩散趋势的中国重新启动工厂

하지만 세계 공급망 파괴로 원재료가 부족해 실제로는 상당수 공장이 제대로 운영되지 못한다 Nevertheless, their comeback does not translate into 100 percent operation as they fall short of raw material due to global supply disruptions

但是,由于世界供应链的破坏,原材料不足,实际上相当多工厂无法正常运营

중국 화난(華南) 지역 미국 상공회의소의 최근 조사에 응한 중국 남부지역 기업 237곳 가운데 32%가 원재료 공급 부족에 직면했다 According to the U S Chamber of Commerce’s survey of 237 businesses located in southern China, 32 percent face supply shortages of raw material

中国华南地区美国商会最近调查的237家中国南部地区企业中,32%面临原材料供应不足的问题

코로나19로 해외 각국에서 소비가 줄어든 것도 중국 경제에는 악영향을 주고 있다 Overall, the Chinese economy has been hit hard by the decreasing consumption demand across the globe

新冠肺炎疫情导致海外各国消费下降,也给中国经济带来不利影响

중국의 제조업 중심지 항저우(杭州)의 한 자동차 부품 관련 기업은 “지난달부터 공장을 가동했지만 중국 내 수요 감소뿐 아니라 한국 일본에 대한 수출도 지장을 받아 평상시 주문의 30%만 들어온다”고 미국 워싱턴포스트에 밝혔다 Despite the resumed operation last month, a Hangzhou-based auto part company sees its orders decline 30 percent in volume compared to normal levels due to decreases in demand not only in China but also in South Korea and Japan, according to The Washington Post

中国制造业中心杭州的一家汽车零部件相关企业对美国《华盛顿邮报》表示:“虽然从上个月开始启动工厂,但不仅中国国内需求减少,而且对韩国和日本的出口也受到影响,只有平时订单的三成”

미국 코어사이트리서치에 따르면 미국 신발 제조업체인 스티브매든은 제품 73%를, 미국 전자제품 기업 베스트바이는 60%를 중국에서 생산한다 U S shoemaker Steve Madden and electronics fabricator Best Buy produce 73 percent and 60 percent of their products within China, respectively, said U S analysis firm Coresight Research

据美国市场调查机构CoresightResearch统计,美国鞋类制造商史蒂夫·麦登公司生产的产品的73%和美国电子产品企业百思买公司生产产品的60%,是在中国生产的

코로나19로 미국 내 수요가 감소하면 중국 내 공장 생산도 줄어 중국 경제도 피해를 입는다는 것이다 This implies that decreasing demand in the U S market driven by the COVID-19 outbreak can shrink factory production in China, then affecting the Chinese economy as a whole

也就是说,如果新冠肺炎疫情在美国的需求减少,中国国内工厂生产也会减少,中国经济也会受到损失

중국 정부는 ‘코로나19 퇴치전에서의 승리’를 선언하려고 하지만 이제 글로벌 공급망 파괴와 소비 급감이 중국 경제에 미칠 타격을 걱정해야 하는 처지가 됐다 Beijing finds itself in a position to face the economic consequences of global supply disruptions and shrinking demand although it has been waiting for the perfect timing to declare its victory in the war against COVID-19

中国政府想宣传“在阻击新冠肺炎疫情的战争中取得胜利”,但现在不得不担心全球供应链的受损和消费锐减对中国经济带来的冲击

세계화의 가장 큰 수혜자인 중국은 세계화 단절의 가장 큰 피해자가 될 수 있기 때문이다 The world’s No. 1 beneficiary of globalization seems to be just a few steps away from becoming the biggest victim of a growing headwind in the globalized market

因为作为全球化的最大受益者,中国可能成为全球化断裂的最大受害者

코로나19의 대규모 확산이라는 1차 위기 이후 중국이 직면한 이 2차 위기는 1차 때와 달리 중국 정부 혼자 해결할 수 없다 The Chinese government alone is not able to fight against the second crisis coming on the scene unlike COVID-19 within the country

在新冠肺炎疫情大规模扩散的第一轮危机之后,中国面临的这场第二轮危机与第一次不同,中国政府无法独自解决

그런데 생산·소비·무역의 상호의존도가 가장 큰 세계 1, 2위 경제대국 미국과 중국이 코로나19 대처와 발원지 문제로 책임 회피와 소모적 갈등을 되풀이하고 있다 Regrettably, the United States and China, the two biggest economic powerhouses highly interdependent in terms of production, consumption and trade, are pointing an accusatory finger at each other while wasting time arguing over coronavirus responses and accountability as an epicenter

但是生产 消费 贸易相互依赖度最大的世界第一 第二经济大国美国和中国因为新冠肺炎疫情的对策和发源地问题,一再出现了回避责任和消耗性矛盾

미중이 이런 식으로 계속 싸우면 다른 나라들이 글로벌 경제 위기에 열심히 대처하려고 해도 효과가 떨어질 것이라는 우려가 크다 Worryingly, the ongoing U S -China tug-of-war will inevitably offset efforts by the rest of the world to beat the coronavirus-induced shock to the global economy

人们普遍担心,如果美中继续以这种方式进行斗争,即使其他国家努力应对全球经济危机,效果也会下降

싱하이밍 주한 中대사 “한국 기업인 격리 면제할수도” China could take exception to S Koreans’ entry, says Ambassador Xing

中国驻韩国大使邢海明:“可以免除韩国企业人士的隔离”

싱하이밍(邢海明) 주한 중국대사(사진)가 신종 코로나바이러스 감염증(코로나19)과 관련해 “중국은 상대국에서 근무하는 외국 국민들의 필수적인 왕래를 보장하며 중국을 방문하는 한국 국민들에게 맞춤형 편리를 제공할 것”이라고 밝혔다 Chinese ambassador mentions of exception on S Koreans with health certificate Chinese Ambassador to South Korea Xing Haiming said that essential visits by South Koreans to China are guaranteed and China will provide South Koreans visiting the country with the convenience they need

中国驻韩国大使邢海明(照片)就新型冠状病毒肺炎表示:“中国保障在对方国家工作的外国国民的必要往来,将为访问中国的韩国国民提供针对性的便利”

정부가 코로나19 음성 확인서나 건강증명서를 지참한 기업인에 대해 예외적으로 입국을 허용하도록 중국과 협의 중인 가운데 중국 고위 당국자가 이에 대해 긍정적인 입장을 시사한 것은 처음이다 It is the first time, since Seoul and Beijing started discussing applying exceptions to China’s entry ban, that a high-ranking Chinese official indicated a positive stance towards making an exception on South Korean businessmen carrying a health certificate or a certificate certifying that he/she has been tested negative for coronavirus

韩国政府正在与中国磋商,寻求允许持有新冠病毒检测阴性确认书或健康证明书的企业人士作为例外入境,中国高层官员对此表明肯定立场尚属首次

싱 대사는 17일 주한 중국대사관에서 진행된 동아일보와의 단독 인터뷰에서 한국 기업인에게 입국 시 격리 조치를 면제하는 방안에 대해 “한국에서 검사하고 (중국에) 내려서 한 번 더 검사해 발열 증상이 없으면 그렇게 되지 않을까 한다”며 이같이 말했다 In an exclusive interview with the Dong-A Ilbo held at the Chinese Embassy in Seoul on Tuesday, Ambassador Xing said it is likely that South Korean businessmen will be exempted from quarantine measures if they pass airport health checks in South Korea and China

邢海明17日在中国驻韩国大使馆接受《东亚日报》独家采访时,就韩国企业人士入境时免除隔离措施的方案表示:“在韩国接受过检查,(到中国后)再检查一次,如果没有发热症状,可能就可以”

중국은 17일 현재 24개 지방정부(성·시·자치구)에서 한국발 입국자들을 14일간 격리하고 있다 As of Tuesday, 24 Chinese local governments are putting visitors from South Korea in quarantine for two weeks

截至17日,中国的24个地方政府(省 市 自治区)对来自韩国的入境者实施14天隔离

싱 대사는 “한국이 한중 코로나19 대응 방역협력체제 1차 화상회의에서 건강확인증으로 문제가 없다는 것을 입증한다면 입국을 허용하는 원칙을 제언했고, 중국 정부는 이를 진지하게 검토하고 있다”고 전했다 시진핑(習近平) 중국 국가주석의 방한과 관련해 싱 대사는 “시 주석은 이미 문재인 대통령의 방한 요청을 수용했기 때문에 반드시 한국을 방문할 것”이라면서도 “시기는 결정된 상황이 아니다”라고 했다 또 방한 시기가 방일과 연계돼 있느냐고 묻자 “그렇지 않다”고 말했다 Ambassador Xing said China is seriously reviewing the proposal made by South Korea during their first video conference on response to COVID-19 to allow South Korean visitors entry into the country if they can prove their health status by submitting a health certificate

邢海明表示:“韩国在韩中应对新冠肺炎合作体制第一次视频会议上提出如果能证明健康确认证没有问题就允许入境的原则,中国政府正在对此进行认真研究”关于中国国家主席习近平的访韩,邢海明表示:“习近平主席已经接受了文在寅总统的访韩邀请,所以一定会访问韩国”但他同时表示:“时间并没有决定”当记者问及访韩时间是否与访日有关时,他表示:“不是”

시진핑 “韓中은 같은 배 탄 우호국 힘닿는한 돕겠다“ ‘S Korea and China are friendly nations on the same boat,’ says Xi

习近平致电文在寅:“中韩是同舟共济的友好邻邦……中国将提供力所能及的援助”

시진핑(習近平) 중국 국가주석이 신종 코로나바이러스 감염증(코로나19)과 관련해 문재인 대통령에게 감사와 위로를 담은 전문을 보냈다 Chinese President Xi Jinping sent a message of sympathy to his South Korean counterpart Moon Jae-in concerning the ongoing COVID-19 pandemic

中国国家主席习近平就新冠肺炎问题,致电文在寅总统表示感谢和慰问

14일 관영 중국중앙(CC)TV에 따르면 시 주석은 “중한은 같은 배를 탄 우호 국가”라며 “한국 정부와 사회 각계각층은 그동안 잇달아 중국의 방역 상황에 안부를 묻고 많은 도움을 줬다”고 했다 President Xi described that China and South Korea are friendly nations on the same boat, adding that the South Korean government and people from all walks of life have extended their sympathy to China and provided it with enormous support, according to Chinese state-run TV network China Central Television (CCTV) on Saturday

据中国官方媒体中央电视台14日报道,习近平主席在慰问电中表示,“中韩是守望相助 同舟共济的友好邻邦”,“韩国政府和社会各界此前纷纷向中方抗击疫情表达关心慰问并提供大量帮助”

지난달 20일 한중 정상 통화 당시 문 대통령이 “중국의 어려움이 우리의 어려움”이라며 코로나19 극복을 위한 양국 공조를 강조한 데 이어 시 주석이 전문을 통해 이를 재차 언급한 것이다 He repeated the importance of cooperation between the two nations in overcoming the novel coronavirus outbreak as stressed by President Moon over the phone on Feb 20 that China's difficulties are South Korea's difficulties

上个月20日,在韩中国家元首通话时,文在寅总统表示,“中国的困难是韩国的困难”,强调了两国为克服新冠肺炎疫情加强合作的重要性,习主席通过电文再次提及了这一点

시 주석은 “감염병에는 국경이 없고 세계 각국은 동고동락하는 운명 공동체”라며 “중국 정부와 인민은 한국이 현재 겪고 있는 어려움에 공감하며 힘닿는 데까지 (한국을) 돕겠다”고 강조했다 The Chinese leader emphasized that pandemics have no borders and all countries around the globe share a common future

习主席表示:“疫病没有国界,世界各国是休戚与共的命运共同体”,“中国政府和中国人民对韩方目前遭受的疫情和困难感同身受,将继续提供力所能及的援助”

이어 “나는 중한 관계 발전을 매우 중시하고, 문 대통령과 함께 노력해 양국의 전략적 협력 동반자 관계를 더 높은 수준으로 끌어올리길 바란다”고 덧붙였다 He went on to say that the Chinese government and people empathize with South Korea’s struggle, promising to do all the best to assist Seoul in weathering the outbreak

他还表示:“我高度重视中韩关系发展,愿同文在寅总统一道努力,推动中韩战略合作伙伴关系迈向更高水平”

앞서 양국은 13일 ‘한중 합동 코로나19 방역 체계’를 출범시키고 외교부와 방역당국 등 관계부처가 참석한 가운데 ‘한중 코로나19 대응 방역 협력 대화(국장급)’ 화상회의를 개최했다 In his message, Xi valued the relationship between Beijing and Seoul highly, hoping to work with President Moon to take their cooperative partnership to a higher level South Korea and China held a video conference on Friday among foreign affairs and public health officials to strengthen cooperation amid the COVID-19 pandemic while embarking on a joint quarantine system

此前,两国于13日成立了“韩中联合新冠肺炎防疫体系”,并举行了由外交部和防疫当局等相关部门参加的“韩中应对新冠肺炎疫情合作对话(局长级)”视频会议

中외교부 “日의 입국제한 이해” China understands Japan’s entry restrictions with little objection

中国外交部“对日本的入境限制表示理解”,不提出问题

문제제기 안해일본이 한국과 함께 중국에 대해서도 사실상 입국 금지 조치를 취했지만 중국의 반응은 한국 정부와 전혀 다르다 China has shown a somewhat different reactions from South Korea in the wake of Japan’s travel limits that in effect ban travel to and from its two neighboring nations

虽然日本对韩国和中国共同采取了事实上的禁止入境措施,但中国的反应与韩国政府截然不同

정부와 언론매체 모두 “이해할 수 있다”며 문제 제기를 하지 않았다 Both the Chinese government and media outlets have not raised any issue concerning the entry ban, describing it as “understandable

中国政府和媒体都表示“可以理解”,没有提出问题

최근 가까워지고 있는 중일 관계를 상징적으로 보여주는 장면이라는 분석이 나온다 ” It is analyzed that China’s reactions symbolically demonstrate the relationship between Beijing and Tokyo that has recently been improving

有分析认为,这一场面是最近日益亲近的中日关系的象征

중국 외교부 자오리젠(趙立堅) 대변인은 5일 일본의 입국 제한 조치에 대해 “중국이든 일본이든 자국과 외국 시민의 건강과 안전, 지역과 세계 공공 보건 안전을 위해 과학적이고 전문적인 조치를 취하는 데 대해 여러분 모두 이해할 수 있을 것”이라고 밝혔다 “Both China and Japan are taking science-based, professional and appropriate measures in order to protect the health and safety of their nationals and foreigners and uphold regional and global public health security, which I believe we all understand,” Chinese Foreign Ministry spokesperson Zhao Lijian talked about Japan’s entry limits on Thursday

中国外交部发言人赵立坚5日就日本限制入境措施表示:“无论是中国还是日本,为保障本国和外国市民的健康和安全,维护地区和世界公共卫生安全,采取科学 专业的措施,大家都能理解”

그는 “양국은 외교 경로로 긴밀히 소통을 유지하고 있다”고 밝혀 일본이 중국에 미리 조치를 알려왔음을 시사했다 “The two sides are in close communication through diplomatic channels ” His remarks imply that Tokyo gave advance notice to Beijing

他表示,“两国通过外交途径保持着密切的沟通”,暗示日本已经提前向中国通报了措施

배타적 성향의 환추(環球)시보도 아베 신조(安倍晋三) 일본 총리가 발표한 한국, 중국발 승객 14일 격리 소식을 전한 소셜네트워크서비스(SNS) 공식 계정 기사의 제목을 “이해할 수 있다”고 달았다 Even China’s state-run media Global Times used the word “understand” in the title of an article posted on its official social media account reporting a 14-day quarantine on travelers from China and South Korea that has recently been announced by Japanese Prime Minister Shinzo Abe

具有排外倾向的《环球时报》也刊登了日本首相安倍晋三宣布的对从韩国 中国出发的乘客隔离14天的消息,并在社交媒体的官方账号上报道时加上了“可以理解”的题目

중국 SNS 웨이보에는 비자 효력 취소에 따른 우려와 문의가 잇따랐지만 일본의 조치를 노골적으로 비난하는 글은 찾기 어려웠다 On Weibo, the Chinese social media network, few comments criticized Japan’s measures explicitly although many users poured out their concerns and inquiries about invalidation of tourist visas issued to Chinese citizens

中国社交媒体微博上虽然接连出现了关于取消签证效力的担忧和咨询,但是很难找到露骨地指责日本措施的文章

한 중국 누리꾼은 관련 기사에 “중국이 비상시기에 먼저 저렇게 했다 일본의 조치는 비난할 일이 아니다 한국도 저렇게 할 것을 제안한다”는 댓글을 달았다 In a comment on an article regarding Japan’s entry ban, a Weibo user argued that Japan is not to blame given that China took the same action in an emergency situation at an earlier time while proposing that Seoul should do the same

一位中国网民在相关报道下写道:“中国首先在非常时期这样做日本的措施不应该受到谴责, 我建议韩国也这样做。“

중국 정부와 매체의 반응은 신종 코로나바이러스 감염증(코로나19)의 역유입을 막는다는 명분으로 이미 중국 여러 지역에서 한국과 일본발 승객에 대해 14일 강제 격리 조치를 시행하는 것을 정당화하기 위한 측면이 있다 In some sense, such reaction from the Chinese government and media outlets is interpreted as a justification for the 14-day quarantine measure against travelers from Japan and South Korea taken earlier in many Chinese regions under the pretext to contain the outbreak of the novel coronavirus COVID-19 that can be caused by incoming visitors

中国政府和媒体的反应,某种程度上是为了正当化中国多个地区以防止新冠病毒肺炎回流为由,对来自韩国和日本的乘客实施14天强制隔离的措施

이와 함께 2018년 10월 아베 총리가 중국을 방문해 ‘새로운 중일관계’를 선언한 이후 양국 관계가 꾸준히 개선돼온 흐름과도 무관치 않다는 분석이 나온다 The coronavirus outbreak could affect Xi’s S Korea visit There is a growing consensus in the South Korean government that Chinese President Xi Jinping’s state visit to South Korea might have to be rescheduled with the coronavirus outbreak showing no sign of slowing down soon

还有分析认为,这与2018年10月安倍首相访华宣布“新中日关系”后两国关系不断改善的趋势不无关系

중국은 미중 갈등 속에서 일본을 끌어들이려 했고, 일본 역시 미국의 동맹 홀대 속에서 경제·안보를 위해 중국과의 관계 개선이 필요한 상황이다 On Tuesday, a senior official of the Ministry of Foreign Affairs said in a press conference, “A delay in President Xi’s visit to Japan, which was originally scheduled for April, has received much media coverage in Japan,” adding that his visit to South Korea might also be affected by the new coronavirus

中国试图在美中矛盾中拉拢日本,日本也在美国忽视同盟的情况下为了经济 安全需要改善与中国的关系

실제 코로나19가 중국에서 본격적으로 확산되기 시작한 1월 일본은 중국에 가장 먼저 마스크 등 방역 물품 지원 의사를 밝혔다 Although these remarks were about the agreement between Tokyo and Beijing to postpone the visit to Autumn, it is worth noting that it was the first time a high ranking South Korean official mentioned a possibility of delaying President Xi’s visit to Seoul

实际上,新冠病毒肺炎在中国正式开始扩散的1月份,日本最先向中国表明了支援口罩等防疫物品的意向

당시 중국 정부는 “어려울 때 친구가 진짜 친구”라며 일본에 대한 감사를 표했다 However, the official also said, “All preparations are proceeding according to the original plan

当时中国政府对日本表示感谢,称“困难时的朋友才是真正的朋友”

“시진핑 방한 변함 없지만 코로나 극복 안되면 영향”

韩国政府高官:“习近平访韩不会改变,但如果新冠疫情不能克服,将受到影响”

신종 코로나바이러스 감염증(코로나19) 사태 장기화로 시진핑(習近平·사진) 중국 국가주석의 상반기 방한 계획이 영향을 받을 수 있다는 기류가 외교 당국에서 확산되고 있다 ” Some experts say it would also be difficult to confirm‎ diplomatic schedules with Russia, which celebrates 30 years of diplomatic relations with South Korea this year

韩国外交当局普遍认为,由于新型冠状病毒感染症事态长期化,中国国家主席习近平上半年访韩的计划可能会受到影响

외교 고위당국자는 3일 기자들과 만나 “일본 언론에 4월 중으로 추진되던 시 주석의 방문 계획이 연기된다는 보도가 많이 나오고 있다”며 “(코로나19) 사태가 빨리 극복되지 않는다면 (시 주석 방한 일정에도) 영향이 있을 수도 있지 않을까 생각해 보게 된다”고 말했다 The latest coronavirus outbreak has disrupted the South Korean government’s plan to arrange Russian Foreign Minister Sergey Lavrov’s visit to Seoul for this month at the earliest

外交当局高层官员3日会见记者时表示:“日本媒体上有很多报道称习主席4月份的访问计划已被推迟”,“我认为,如果(新冠病毒)事态不能尽快克服,(习主席访韩日程)也会推迟”

코로나19 사태로 인해 4월로 추진하던 시 주석 방일 일정을 가을로 미루는 것을 중일이 조율하고 있다는 외신 기사를 거론한 것이지만, 정부 고위 당국자가 시 주석 방한도 코로나19의 영향을 받을 수 있다고 언급한 것은 처음이다 Reportedly, a number of working-level meetings have already been delayed or cancelled

这虽然提到的是外电关于中日正在协调因新冠疫情而推迟习主席4月份访日日程的报道,但这是韩国政府高层官员首次提到习主席的访韩也会受到新冠疫情的影响

다만 이 고위 당국자는 “기존의 협의 틀 속에서 변함없이 (상반기 방한) 일정을 추진하고 있다”고 덧붙였다 While the South Korean Ministry of Foreign Affairs is looking closely at the possibility of Washington imposing an entry ban on Seoul, the U S government announced that it would “tighten screening rules”

但是该官员补充说:“我们仍然在现有协议框架下推进(上半年访韩)日程”

올해로 수교 30주년을 맞은 러시아와의 외교 일정도 이른 시일 내 구체화하기는 어려워졌다는 평가가 나온다 （no translation）

有评价称,今年与俄罗斯迎来建交30周年,但对俄外交日程也很难在短时间内具体化

정부는 세르게이 라브로프 러시아 외교장관의 방한을 이르면 이달 중으로 추진하려 했으나 코로나19 여파로 어려워진 것으로 전해졌다 （no translation）

据悉,韩国政府原计划最早在本月内推进俄罗斯外长拉夫罗夫的访韩计划,但由于新冠疫情的影响而变得困难

이외에도 낮은 급에서의 외교 일정 다수는 이미 연기 및 취소되고 있는 것으로 알려졌다 （no translation）

据悉,除此之外,低级别的诸多外交日程已经延期或取消

외교 당국이 미국의 한국인 입국금지 등의 가능성에 촉각을 곤두세운 가운데 미국은 당분간 ‘검사 강화’ 수준에서 대처에 나선다는 입장을 밝혔다 （no translation）

在外交当局高度关注美国禁止韩国人入境等可能性的情况下,美国表明了暂时“加强检查”的立场

마이크 펜스 미 부통령은 2일(현지 시간) 기자회견에서 “이탈리아와 한국에서 오는 모든 직항편에 대해 공항에서 100% (발열) 검사가 이뤄질 것”이라고 말했다 “There will be a 100 percent screening on all direct flights at all airports across Italy and across South Korea,” said U S Vice President Mike Pence in a press conference on Monday

美国副总统迈克·彭斯当地时间2日在记者会上表示:“对意大利和韩国的所有直航航班,将在机场进行100%(发热)检查”

다만 도널드 트럼프 미 대통령은 같은 날 ‘여행 제한 강화를 검토하느냐’는 취재진의 질문에 “(코로나19가) 더 많이 발병하고 있는 특정 국가들에 대해 그렇게 할 것”이라고 답해 추가 대응 가능성을 시사했다 However, when asked about stricter travel restrictions, U S President Donald Trump said he would place further restrictions on countries hit particularly hard by the infection

但是,美国总统特朗普同一天在回答记者“是否考虑加强旅行限制”的提问时回答说:“对(新冠疫情)发病较多的特定国家会这样做”,暗示存在加强应对的可能性

일본 외무성은 2일 경북 경산시, 영천시, 칠곡군, 의성군, 성주군, 군위군 등 6개 지역의 감염증 위험정보를 기존 ‘레벨1’에서 ‘레벨3’으로 올렸다 Meanwhile, the Japanese Ministry of Foreign Affairs elevated Monday its warning against travel to six regions in South Korea including Gyeongsan, Yeongcheon, Chilgok, Uiseong, Seongju and Gunwi from “Level1” to “Level 3

日本外务省2日将庆尚北道庆山市 永川市 漆谷郡 义城郡 星州郡 军威郡等6个地区的感染症危险信息从原来的“等级1”提升至“等级3”

레벨3은 방문 중지를 권고하는 수준으로 4단계 중 두 번째로 심각한 단계다 ” Level 3, which advises the public not to travel, is the second highest level

等级3建议暂停访问,是四个级别中第二个严重级别

3일 오후 기준 한국인의 입국을 금지하거나 격리 등에 나선 국가는 총 89개국인 것으로 집계됐다 유엔 회원국(193개국) 절반에 달하는 46% 정도다 As of Tuesday afternoon, a total of 89 nations, which takes up 46 percent of the UN member countries, have banned the entry of or isolated South Korean nationals

据统计,截至3日下午,禁止韩国人入境或予以隔隔离的国家共有89个，占联合国会员国(193个国家)一半的46%。

해외에서 자가 및 지정시설에 격리 조치를 당하고 있는 한국인은 3일 오전 기준 1200명을 넘는다고 외교부는 밝혔다 According to the South Korean Ministry of Foreign Affairs, the number of Koreans who have self-isolated or have been quarantined exceeded 1,200 as of Tuesday morning

外交部表示,截至3日上午,在海外被隔离的韩国人超过1200人

중국에서 960명, 베트남에서 270명가량이 격리 중이며, 러시아 키르기스스탄 카타르 등지에 각각 10여 명이 격리 중인 것으로 알려졌다 It is reported that 960 and 270 of them are in China and Vietnam, respectively, while Russia, Kyrgyzstan and Qatar have more than 10 people each who are isolated

据悉,中国和越南分别有960人和270多人被隔离,俄罗斯 吉尔吉斯斯坦 卡塔尔等地各有10多人被隔离

시진핑 “코로나19 발원지 찾아라” 지시 Xi Jinping instructs to find the origin of COVID-19

习近平指示“查明COVID-19的发源地”

시진핑(習近平) 중국 국가주석이 신종 코로나바이러스 감염증(코로나19)의 발원지를 밝혀내라고 중국 과학자들에게 지시했다 Chinese President Xi Jinping has directed scientists to find the origin of COVID-19

中国国家主席习近平指示中国科学家尽快查明新型冠状病毒感染症(COVID-19)的发源地

최근 중국 관영매체와 당국 관계자들을 중심으로 중국이 발원지가 아닐 수 있다는 주장이 제기되는 가운데 나온 지시여서 배경이 주목된다 It is garnering attention as China’s official news agency and authorities recently argued that China may not be the origin of the virus

这是最近以中国官方媒体和当局有关人士为中心,提出中国可能不是发源地的情况下做出的指示,其背景备受关注

2일 관영 중국중앙(CC)TV에 따르면 시진핑은 이날 코로나19 관련 연구를 진행 중인 베이징(北京)의 군사의학연구원과 칭화(淸華)대 의학원을 시찰하면서 “인공지능(AI)과 빅데이터 등 새로운 기술을 활용해 (코로나19의) 근원을 추적하라 Xi ordered Beijing’s military medical research center and Tsinghua University’s medical institute to trace the origin [of the virus] and conduct research on the virus using new technologies such as AI and big data, according to the report of CCTV on Monday

2日,据中国中央电视台(CCTV)报道,习近平当天在视察正在进行新冠肺炎疫情相关研究的北京军事医学研究院和清华大学医学院时表示,“利用人工智能(AI)和大数据等新技术,追踪(新冠肺炎疫情的)根源

(코로나19) 병원(病源)이 어디에서 왔는지, 어디로 가는지 명확히 밝히고 검사의 정확도와 효율을 높이라”고 주문했다 He also asked them to clarify where the pathogen came from and where it is going, and increase the accuracy and efficiency of tests

明确说明(新冠肺炎疫情)病源究竟来自何处,去往何处,提高检查的准确度和效率”

일부 전문가들은 “코로나19의 발원지가 여러 곳일 수 있고, 발원 동물(숙주) 역시 여러 종일 수 있다”는 의견을 냈다 “The virus may have more than one origin and multiple hosts,” said some experts

一些专家提出,“新冠肺炎疫情的发源地可能有多处,发源动物(宿主)也可能有好几种”

중국 환추(環球)시보는 이달 초 “미국이 발원지일 수 있다”는 주장까지 펼쳐 코로나19가 세계로 확산되자 중국이 책임론에서 벗어나려는 의도라는 해석이 나온다 The Global Times even asserted that the U S may be the origin early this month, which is interpreted as an intention to avoid China’s responsibilities as the virus has spread across the world

中国《环球时报》本月初甚至主张“美国可能是发源地”,因此有人分析说,新冠肺炎疫情扩散到全世界后,中国试图摆脱责任论

中최상급 보건당국 “에어로졸 전파 가능성 존재”China’s health authorities acknowledges possibility of aerosol transmission

中国国家卫健委:“COVID-19存在气溶胶传播可能性”

중국의 보건당국 중 가장 상급기관이 에어로졸(대기 중에 떠도는 미세한 입자)에 의한 신종 코로나바이러스 감염증(코로나19) 전파 가능성을 처음으로 공식 인정했다 The highest health authorities in China have officially acknowledged the possibility of COVID-19 spreading through aerosol for the first time

在中国保健当局(卫生局)中的最上级机关首次正式承认了通过气溶胶(大气中漂浮的微小粒子)传播新型冠状病毒感染症(世界卫生组织将新冠病毒命名为COVID-19)的可能性

중국 국가위생건강위원회(위건위)는 19일 발표한 ‘코로나19 진료 방안(제6판)’에서 전파 경로에 관해 “상대적으로 밀폐된 환경에서 장시간 고농도의 에어로졸에 노출되면 에어로졸 전파 가능성이 존재한다”는 대목을 추가했다고 밝혔다 The National Health Commission of China newly added exposure to high concentrations of droplets in the air mixed with the virus in a closed environment for a long time as one of the possible routes of new coronavirus transmission in new guidelines for diagnosing and treating patients announced on Wednesday

中国国家卫生健康委员会(卫健委)在19日发布的《COVID-19诊疗方案(第六版)》中就传播途径表示,“在相对封闭的环境下,如果长时间暴露在高浓度的气溶胶中,病毒通过气溶胶传播的可能性较大”

위건위는 4일 발표한 진료 방안(제5판)에서는 “에어로졸 전파 경로는 아직 명확하지 않다”고 밝혔다 At the guidelines announced on February 4, the National Health Commission had said that the routes of aerosol transmission were not clear

卫健委在4日发布的诊疗方案(第五版)中表示,“气溶胶传播途径尚不明确”

하지만 이후 전문가와 일부 지방정부가 에어로졸 감염 가능성을 제기했다 Later, however, some experts and local governments suggested the possibility of aerosol transmission

但此后,专家和一些地方政府提出了通过气溶胶传播的可能性

8일에는 상하이(上海)시 정부가 “주요 전파 경로는 비말(침방울)을 통한 직접 전파, 에어로졸 전파, 접촉 전파로 확정할 수 있다”고 밝혔다 Also in Feb 8, the Shanghai government announced that transmission routes of the coronavirus included direct transmission, contact transmission and aerosol transmission

8日,上海市政府曾表示,“COVID-19主要传播途径确定为通过飞沫(唾液)的直接传播 气溶胶传播 接触传播”

하지만 다음 날인 9일 위건위가 “에어로졸 전파를 보여주는 증거가 없다”고 반박하면서 논란이 불거졌다 But controversies arose when the Chinese health agency refuted the theory saying that there is no evidence of aerosol transmission

但第二天的9日,卫健委反驳称“没有证据显示病毒可通过气溶胶进行传播”,引发了争议

비말은 상대적으로 무거워 통상 2m 범위를 벗어나지 못하는 반면 에어로졸은 가벼워 멀리 퍼진다 While droplets are comparatively heavy and cannot travel further than a radius of two meters most of the time, aerosol is lighter and spreads further

飞沫相对较重,通常无法超出2米范围,而气溶胶相对轻巧,传播距离较远

사무실 등 밀폐된 실내 공간에 남아 있는 에어로졸을 통한 집단 감염이 일어날 수도 있다 A cluster of infections may occur through aerosol left in closed spaces

通过留在办公室等密闭室内空间的气溶胶,有可能发生集体感染

환자 9배 폭증...중 신뢰잃은 ‘코로나 통계’ （no translation）

（漏译）

위건위는 또 후베이성에만 적용하던 ‘폐렴 증상이 있는 임상 진단 환자’ 분류 대목을 삭제한다고 밝혔다 （no translation）

卫健委还删除了只适用于湖北省的“有肺炎症状的临床诊断患者”分类条款

후베이성이 13일 이 항목으로 분류되는 환자를 확진 환자에 포함하면서 하루 사이에 후베이성의 환자가 1만4840명이나 늘어났다 （no translation）

13日,湖北省将列入这一项目的患者包括在确诊患者中,仅一天时间,湖北省的患者就增加了14840人

하지만 새로 나온 진료 방안을 적용하면 후베이성도 임상 진단 환자를 확진 환자에 포함시킬 필요가 없어져 통계상 후베이성의 환자 규모를 줄이려는 의도가 아니냐는 지적이 나온다 （no translation）

但有人指出,如果采用新诊疗方案,湖北省也没有必要将临床诊断患者包括在确诊患者中,因此,从统计上来看,此举是不是为了减少湖北省的患者规模

환자 9배 폭증 중 신뢰잃은 ‘코로나 통계’ China’s coronavirus statistics loses credibility with 9-fold increase

患者暴增9倍,失去信任的中国“新冠肺炎”统计

중국이 신종 코로나바이러스 감염증(코로나19) 확진 환자 판정에 대한 기준을 바꾸면서 후베이(湖北)성의 감염자와 사망자 수가 폭증했다 As China has changed the criteria to confirm‎ the Wuhan coronavirus patients, the numbers of infected cases and deaths from the virus in Hubei province soared

中国改变对新型冠状病毒感染症(COVID-19)确诊患者判定标准后,湖北省的感染者和死亡人数暴增

코로나19 발생지인 우한(武漢) 등 후베이성의 코로나19 확산세가 알려진 것보다 훨씬 심각하고 그동안 실상을 은폐·축소해온 것 아니냐는 의혹이 제기됐다 As a result, some are raising the suspicion that the new coronavirus has been spreading more rapidly than what has been reported and that the Chinese government has been hiding the true statistics

有人提出疑惑称,新冠病毒发生地武汉等湖北省的新冠病毒扩散趋势比人们所知的还要严重,一直以来是否隐瞒和缩小了事实真相

후베이성 위생건강위원회는 12일 하루 사이 확진 환자는 1만4840명, 사망자는 242명 늘었다고 13일 발표했다 The hygiene and health commission of Hubei province announced on Thursday that the number of confirmed patients rose by 14,840 with 242 more deaths in just one day on Wednesday,

湖北省卫生健康委员会13日公布,12日一天内新增确诊病例14840例,死亡242例

11일에 비해 확진 환자 수는 약 9배, 사망자 수는 약 2. 6배 증가한 것이다 which is nine times and 2.6 times higher than the Tuesday figures, respectively

与11日相比,确诊患者和死亡人数分别增加了约9倍和2.6倍

매일 오전 공식 집계를 발표해왔던 국가위생건강위원회는 이날 오후까지도 통계를 발표하지 않았다 The national hygiene and health commission, which had been releasing official figures every morning, had not announced statistics until Thursday afternoon

每天上午公布正式统计的国家卫生健康委员会直到当天下午,也没有公布统计结果

후베이성은 “환자들이 제때 확진 판정을 받아 치료 성공률을 높이기 위해 임상(치료) 진단을 확진 판정 기준으로 추가했다”고 해명했다 “Clinical diagnosis has been added as one of the criteria to confirm‎ infected cases in order to allow timely diagnosis of patients and increase treatment success rates,” explained Hubei province

湖北省解释说:“为使患者得到及时确诊 提高治疗成功率,把临床(治疗)诊断作为确诊的标准”

“그동안 핵산 검사를 통해 확진 판정을 내렸지만 이제 의료진의 판단 및 컴퓨터단층촬영(CT) 영상을 통해 확진 판정을 내릴 수 있도록 기준을 바꾸면서 확진자가 대폭 늘었다”는 설명이다 “So far, cases had been confirmed based on the RNA test, but now patients can be confirmed even with diagnosis by medical professionals and the CT scan, which is why the number of cases went up

“之前通过核酸检查确诊病例,现在改变标准,通过医疗团队的判断及计算机断层扫描(CT)影像,确诊病例大幅增加”

후베이성에 따르면 이런 ‘임상 진단’에 따른 확진 환자와 사망자는 각각 이날 증가 환자의 약 90%(1만332명)와 약 56%(135명)를 차지했다 ” According to Hubei province, the numbers of confirmed patients and deaths by clinical diagnosis account for 90 percent and 56 percent, respectively, of the increased figures on Thursday

据湖北省统计,这种“临床诊断”导致的确诊病例和死亡病例分别约占当天新增病例的90%(10332例)和约56%(135例)

후베이성은 “전국 다른 성(省)이 공표한 확진 판정 기준에 맞추기 위해 13일부터 ‘임상 진단’ 환자를 확진 환자에 포함시켜 발표하기로 했다”고 밝혔다 “We decided to include clinically-diagnosed patients as confirmed cases to match the confirmation criteria announced by other provinces,” said the Hubei provincial government,

湖北省说,“为符合全国其他省份公布的确诊标准,湖北省决定从13日起将‘临床诊断’患者纳入确诊病例范围并予以公布”

다른 성에서 이미 적용해 왔던 확진 판정 기준을 이제야 적용했다고 밝힌 것이다 which is an admission to the fact that the province just started to apply the confirmation criteria that have been applied by other provinces

即,此前已在其他省份适用的确诊标准现在才开始适用

특히 이 확진 분류의 근거인 중국 국가위생건강위원회 ‘코로나19 진단 방안’(제5판)은 일주일여 전인 4일에 이미 나온 것으로 확인됐다 （no translation）

值得一提的是,经确认,这一确诊分类的依据——中国国家卫生健康委员会《新冠病毒诊断方案》(第五版),早在一周多前的4日就已出台

홍콩 사우스차이나모닝포스트(SCMP)는 이날 “우한 등에서 코로나19로 의심되는 폐렴 환자와 사망자가 나와도 과부하에 걸린 의료진이 일반 폐렴으로 분류해 왔다”고 지적했다 （no translation）

香港《南华早报》当天指出,“在武汉等地发现疑似新冠病毒肺炎患者和死亡病例后,超负荷的医护人员一直将这些患者列为普通肺炎”

또 일본 후생노동성은 13일 크루즈선 ‘다이아몬드 프린세스’호에서 신규 감염자가 44명(승객 43명, 승무원 1명) 나왔다고 밝혔다 （no translation）

此外,日本厚生劳动省13日表示,“钻石公主”游轮号上新增感染者44人(乘客43人,乘务员1人)

이로써 크루즈선에서만 확진 환자가 218명 나왔고, 일본 내 감염자 수는 총 247명으로 늘었다 （no translation）

至此,仅游轮上就有218名确诊患者,日本国内感染人数增至247人

한국에서는 이날 추가 감염자가 확인되지 않았지만 보건당국과 의료계는 중국의 코로나19 확진 환자와 사망자 급증에 긴장하는 모습이다 （no translation）

韩国当天虽然没有确认的新增感染者,但是保健当局和医疗界对于中国的新冠病毒确诊患者和死亡者剧增非常紧张

질병관리본부는 중국 보건당국에 환자 수 급증에 대한 확인을 요청했다고 13일 밝혔다 （no translation）

疾病管理本部13日表示,已向中国保健当局要求确认患者人数剧增

中환자 증가폭 매일 신기록 홍콩서도 첫 사망자 The number of confirmed cases of coronavirus keeps increasing in China

中国新型冠状病毒感染病例增幅创造单日新纪录,香港出现首例死亡病例

중국에서 신종 코로나바이러스 감염증(신종 코로나)으로 인한 사망자가 400명을 넘어섰다 The coronavirus outbreak has killed 426 as of Tuesday, up 65 from the previous day

中国因感染新型冠状病毒而死亡的人数已超过400人

홍콩에서는 신종 코로나로 인한 첫 사망자가 나왔다 Hong Kong reported its first coronavirus death on Tuesday,

香港出现首例因感染新型冠状病毒死亡的病例

중국 본토 외에서 사망자가 발생한 것은 필리핀에 이어 두 번째다 confirm‎ing the second death outside mainland China following the Philippines

这是继菲律宾之后,中国本土以外发生的第二个死亡病例

4일 중국 내 확진 환자 수는 전날보다 3136명 증가한 총 2만438명에 달했다 The number of confirmed coronavirus cases in China has reached 20,438 on Tuesday, up 3,136 from the previous day

4日,中国国内确诊患者人数比前一天增加了3136人,共达20438人

하루 환자 증가 수가 처음으로 3000명을 넘어섰다 This is the first time that the number of new confirmed cases exceeded 3,000 a day

一天里患者增加数首次超过了3000名

사망자는 모두 426명으로 전날보다 65명이나 늘었다 The coronavirus outbreak has killed 426 as of Tuesday, up 65 from the previous day

死亡人数为426人,比前一天增加了65人

하루에 발생한 확진 환자와 사망자 수 모두 최고 증가치를 보여 갈수록 증가치가 높아지는 추세다 Amid the biggest daily increase of confirmed case and death toll on Tuesday since the virus was detected,

一天内出现的确诊患者和死亡者数都呈现出最高的增加值,呈现出日益增加的趋势

중국 정부가 공식적으로 밝힌 중증 환자가 2788명에 달하는 데다 신종 코로나 발생지인 후베이(湖北)성 우한(武漢)시에서는 5%가 넘는 사망률을 보이고 있어 앞으로도 사망자 증가세가 이어질 것이라는 우려가 나왔다 Chinese officials said 2,788 people are currently in critical condition and the mortality rate of coronavirus in Wuhan City, Hubei province, the epicenter of the decease, has exceeded 5%, sparking concerns that the new virus’ fatality rate will continue to grow

中国政府正式公布的重症病例达2788人,而且新型冠状病毒感染症发病地湖北省武汉市死亡率超过5%,因此有人担心今后死亡人数还会继续增加

또 홍콩 밍(明)보에 따르면 우한을 방문한 적이 있는 39세 남성이 지난달 31일 확진 판정 뒤 치료를 받다가 이날 사망했다 According to Ming Pao Daily News of Hong Kong, a 39-year-old man, who contracted the virus after travelling to Wuhan, died Tuesday while being treated since he was confirmed to have coronavirus on Friday

另据香港《明报》报道,一名曾到过武汉的39岁男子1月31日确诊后接受治疗,当天不治身亡

홍콩은 전날 선전(深(수,천))만 검문소 등 2곳을 제외하고 중국 본토와 연결되는 모든 검문소를 폐쇄하겠다고 발표했으나 홍콩 내에서 중국 본토와의 접경을 완전 봉쇄하라는 요구가 거세지고 있다 Hong Kong announced on Monday that it will close all border checkpoints with mainland China except for two, but Hong King citizens are calling for the full closure of border with mainland China

香港前一天宣布,除深圳湾检查站等两处外,所有与中国内地相连的检查站都将关闭,但香港境内要求完全封锁与中国内地接壤的边境的呼声日益高涨

中대사 “입국제한, WHO 근거 따라야” Chinese ambassador asks S Korea to follow WHO stance

中国驻韩大使:“入境限制应根据世卫组织的规定”

한국이 취한 (중국인 일부 입국 제한) 조치에 대해 제가 많이 평가하지 않겠다 We can base our decisions on WHO recommendations,’ says Chinese ambassador “I am not going to comment on Seoul's travel restrictions (entry restrictions on Chinese visitors),”

“对于韩国采取的(限制部分中国人入境)措施,我不会多加评价”

” 싱하이밍(邢海明) 주한 중국대사는 4일 주한 중국대사관에서 가진 기자회견에서 이날부터 시행된 정부의 후베이(湖北)성 방문 외국인 입국 제한 방침에 대한 중국의 입장을 묻는 질문에 이같이 답했다 said new Chinese Ambassador to South Korea Xing Haiming at a press conference held at the Chinese Embassy in Seoul on Tuesday

中国驻韩国大使邢海明4日在中国驻韩国大使馆举行的记者招待会上,就韩国政府从当天起实行限制访问过武汉的外国人入境的方针的提问做出了上述回答

신종 코로나바이러스 감염증(신종 코로나) 확산으로 한국 내 중국 비판 여론이 감지되는 상황에서 적극적인 반대 의사를 표하기보다 비교적 낮은 수위로 불쾌감을 표한 것이다 He showed his displeasure rather than actively voicing his opposition to the South Korean government’s decision in a situation where Korean citizens’ criticism towards China is elevating due to the spread of the new coronavirus from China

由于新型冠状病毒感染症的扩散,韩国国内出现了批评中国的舆论,在这样的情况下,与其说积极的反对,不如说是邢海明以比较低的程度表示了不快

싱 대사는 이날 세계보건기구(WHO) 입장을 인용하는 방식으로 한국이 강경한 입국제한 조치를 취하지 않길 바란다는 뜻을 밝혔다 The ambassador said that he does not want South Korea to take on strong entry restrictions while quoting the stance of the World Health Organization

邢海明当天援引世界卫生组织的立场表示,希望韩国不要采取强硬的入境限制措施

그는 “(WHO는) 이번 전염병이 국제여행 등을 불필요하게 방해할 이유는 없다고 했다”며 WHO Director-General Tedros Adhanom Ghebreyesus told the organization’s executive board that there was no need for measures that "unnecessarily interfere with international travel and trade" in containing the virus

他说:“(世界卫生组织)认为此次传染病没有理由不必要地妨碍国际旅行”

“세계적이고 과학적인 것은 WHO 근거인 만큼 (입국 제한 등은) WHO 근거에 따르면 되지 않을까 한다”고 말했다 “The WHO is the most scientific, authoritative organization (on health issues), and I believe that we can base our decisions on WHO recommendations,” the Chinese ambassador said

“世界性 科学性是世界卫生组织的根据,(限制入境等)根据世界卫生组织的规定就可以了吧”

그는 또한 “(한중이) 과학적인 태도로 역지사지하며 (전염병에) 대응해 나가자”고도 덧붙였다 "In the wake of such an issue, we can be seen as part of a community sharing the common destiny I hope we can respond to the disease outbreak while putting ourselves in each other's shoes "

他还表示:“(韩中)要以科学的态度换位思考,共同应对(传染病)”

한국이 대규모 인도적 지원을 결정한 것에 대해선 “전염병과의 투쟁에 큰 힘을 실어줘서 깊은 감사를 표하며, 이 따뜻한 선물을 영원히 잊지 않을 것”이라고 말했다 Xing also used the press briefing to express appreciation for Seoul's support for Beijing's fight against the coronavirus "South Korea, through various channels, has sent messages supporting China’s fight against the contagious disease, and provided us with precious other support," he said "We will not forget this warm support for good

”对于韩国决定提供大规模人道主义援助,他表示:“对与传染病的斗争给予了大力支持,对此深表感谢,我们永远不会忘记这份温暖的礼物”

중국에 500만 달러 지원을 약속한 외교부는 전날 화물기를 빌려 민간에서 제공한 마스크 300만 장 등 구호물품을 실어 중국 우한에 보냈다 " Seoul has decided to offer five million U S dollars in emergency assistance to China, along with three millions of protective masks and other relief items

”韩国外交部承诺向中国提供500万美元的援助,前一天租用货机将民间提供的300万个口罩等救援物资送往中国武汉

한국의 출입국 정책 강화를 두고 중국이 껄끄럽다는 속내를 내비치는 가운데, 정부는 여전히 추가 조치를 배제할 수 없다는 입장을 견지하고 있다 The South Korean government is keeping its stance on taking additional measures even though China spoke of its displeasure towards Seoul’s strengthened entry policy

针对中国对韩国强化出入境政策表现出不快的态度,韩国政府仍然坚持立场称,“不排除追加措施”

김인철 외교부 대변인은 4일 정례 브리핑에서 “여행경보 (상향 등) 조정 문제는 계속 검토해 나가고 있다”고 말했다 “The ministry is reviewing continuously the adjustment of travel warnings,” Kim In-cheol, spokesperson of the South Korean Foreign Ministry, told reporters on Tuesday

外交部发言人金仁哲4日在例行记者会上表示:“关于(向上)调整旅游警报等问题,正在继续进行讨论”

WHO “전례없는 확산” 국제비상사태 선포세계보건기구(WHO)가 신종 코로나바이러스 감염증(우한 폐렴)에 대해 ‘국제적 공중보건 비상사태(PHEIC)’를 선포했다 WHO declares Wuhan coronavirus outbreak a global health emergency The World Health Organization (WHO) has declared the coronavirus outbreak in China a public health emergency of international concern (PHEIC)

“史无前例的扩散”,WHO宣布将新型冠状病毒疫情列为“国际关注的突发公共卫生事件”世界卫生组织(WHO)宣布,将新型冠状病毒感染症(武汉肺炎)疫情列为“国际关注的突发公共卫生事件(英文简称PHEIC)”

테워드로스 아드하놈 거브러여수스 WHO 사무총장은 지난달 30일(현지 시간) 우한 폐렴에 대해 “지난 몇 주 동안 이전에 알지 못했던 병원체의 출현을 목격했고 전례가 없는 발병으로 확대했다”고 말했다 “Over the past few weeks we have witnessed the emergence of a previously unknown pathogen, which has escalated into an unprecedented outbreak,” said WHO Director General Tedros Adhanom Ghebreyesus on Thursday (local time)

世界卫生组织总干事谭德塞(TedrosAdhanomGhebreyesus)当地时间30日就武汉肺炎表示,“过去几周以来,人们目睹了之前所不知道的病原体的出现,并扩大为史无前例的发病”

다만 비상사태 선포는 “중국 내 발생 때문이 아니라 다른 나라에서 일어나는 일 때문”이라며 “(중국과) 교역과 여행을 방해하는 조치를 할 이유가 없다”고 주장했다 But he has made it clear that the declaration is not caused by the outbreak in China but is made due to what is happening outside the country He added that there is no reason to limit trade or travel to China

但他同时表示,宣布紧急状态“疫情不仅在中国国内大规模爆发,在其他国家也正发生疫情”

중국 내 우한 폐렴 확진 환자와 사망자 수 모두 1일 최대치로 증가하면서 확산 속도가 빨라지고 있다 The new coronavirus is spreading fast with the number of confirmed cases and deaths in China recording the biggest daily increase

并强调称,“没有理由采取妨碍(与中国)贸易和旅行的措施”

중국 내 확진 환자는 9692명으로 전날보다 1866명 늘었고, 사망자는 213명으로 43명 증가했다 The number of confirmed cases in China rose to 9,692, up by 1,866 from the previous day, and the death toll increased by 43 to 213

中国国内武汉肺炎确诊患者和死亡人数在1日均达到最高值,扩散速度正在加快

전 세계 확진 환자 수는 9831명으로 2003년 사스(SARS·중증급성호흡기증후군) 전 세계 환자 수 8098명을 넘어섰다 A total of 9,831 cases have been confirmed globally, exceeding 8,098 cases of severe acute respiratory syndrome (SARS) in 2003

中国内地确诊病例9692例,较前一日增加1866例，死亡213例,增加43例全球确诊病例9831例,超过2003年SARS全球病例数8098例

중국에서는 우한 폐렴의 ‘공기 감염’ 가능성까지 제기됐다 A possibility of an airborne infection has been raised in China

在中国甚至出现了武汉肺炎的“空气感染”可能性

중국질병예방통제센터(CDC)의 우쭌유(吳尊友) 수석전문가는 관영 중국중앙(CC)TV 인터뷰에서 “우한 폐렴은 사스와 많이 다르고 유행성감기(독감)의 전파 패턴과 훨씬 비슷하다”며 “Wuhan pneumonia is a lot different from SARS while much similar to the flu virus in terms of its transmission pattern,” a senior researcher at the Chinese Center for Disease Control and Prevention (CDC) said in an interview with CCTV

中国疾病预防控制中心(CDC)首席专家吴尊友在接受中国中央电视台(CCTV)采访时表示,“武汉肺炎与非典有很大不同,与流行性感冒(流感)的传播模式更相似

“우한 폐렴 감염자가 (특정한) 폐쇄공간에서 공기 중에 남긴 비말(飛沫·분비물)이나 에어로졸(미세한 고체 입자나 물방울)을 다른 사람이 흡입해 감염원 없는 감염을 일으킨다는 것을 조사 과정에서 발견했다”고 말했다 “A research study has found that the new coronavirus spreads through droplets or aerosol particles an infected person coughs out in a closed space

在调查过程发现,在(特定的)封闭空间内,其他人吸入武汉肺炎感染者留在空气中的飞沫(分泌物)或浮质(微固体粒子或水滴),引起无感染源的感染”

中서 무증상자發 집단전염 Asymptomatic patient spreads the virus in China

中国出现无症状自发集体传染

3차감염 사례도중국에서 ‘3차 감염’을 비롯해 신종 코로나바이러스 감염증(우한 폐렴) 집단 전염 사례가 잇따르면서 통제 가능한 범위를 넘어선 것 아니냐는 우려가 커지고 있다 There are growing concerns over China’s Wuhan pneumonia, which has shown signs of group transmission and third infection, with many becoming fearful that the epidemic is going out of control

第三次感染病例也出现随着中国连续出现包括“第3次感染”在内的新型冠状病毒感染症(以下武汉肺炎)集体传染事例,人们开始担心疫情是否超出了可控范围

29일 중국 허난(河南)성 안양(安陽)시 위생건강위원회에 따르면 후베이(湖北)성 우한(武漢)에 갔다가 안양 집에 돌아온 루(魯)모 씨에 의해 아버지(45)와 고모 2명이 전염됐다(2차 감염) According to the Sanitary Health Commission of Anyang City, Hunan province on Wednesday, the father (age 45) and two aunts of Mr Liu, who had visited Wuhan before returning to his home in Anyang, were infected with the virus

29日,据中国河南省安阳市卫生健康委员会透露,父亲(45岁)和2个姑姑被从湖北武汉回家的鲁某传染

이어 루 씨 아버지에게서 루 씨 어머니 저우(周·42)모 씨와 루 씨의 또 다른 고모가 다시 한번 감염(3차 감염)됐다 , which is secondary infection The virus also spread to Mr Liu's mother (aged 42) and another aunt, third infection

随后,鲁某父亲又传染给了鲁某的母亲周某和鲁某的另一个姑姑(第3次传染)

루 씨는 확진 환자임에도 10일 우한에서 돌아온 뒤 잠복기(최장 14일)를 지났는데도 증상이 나타나지 않아 무(無)증상 환자에 의한 집단 감염이 현실화됐다 Though Liu was confirmed of the virus, there had been no symptoms after the latent period (up to 14 days), which implies the virus' potential of group transmission via no symptom patients

鲁女士是确诊患者,但她10日从武汉回来后,已过了潜伏期(最长14天),症状仍未出现,无症状患者的集体感染已成现实

또 안후이(安徽)성 허페이(合肥)시에서는 동창 모임에 참석한 20대 6명이 집단으로 감염돼 28일 확진 판정을 받았다 In Hefei City of Anhui province, six people in their 20s were infected after attending a gathering, confirmed of the virus on Tuesday

另外,安徽省合肥市参加同学聚会的6名20多岁学生集体感染,28日确诊

안후이성 황산(黃山)시에서도 가족 6명이 잇따라 감염됐다 A family of six in Huangshan City, Anhui province were also found infected

安徽省黄山市也有6名家属相继被感染

28일까지 중국 본토의 우한 폐렴 확진 환자 누계는 6018명으로 2003년 사스(SARS·중증급성호흡기증후군) 유행 당시 중국 본토 최종 확진 환자 5327명을 넘어섰다 The number of accumulated confirmed cases in mainland China as of Tuesday reached 6018, outnumbering the cases in 2003 during the outbreak of the SAAS virus

截至28日,中国内地武汉肺炎确诊病例累计达到6018例,超过2003年非典流行时中国内地最终确诊病例5327例

우한 폐렴 사망자는 132명으로 전날보다 26명 늘었다 The death toll recorded 132, which is 26 higher from the previous day

武汉肺炎死亡人数为132人,比前一日增加26人

중동에서는 처음으로 아랍에미리트에서 우한 폐렴 확진 환자가 발생했다 A case was first confirmed in the Middle East for the first time in the Arab Emirates

另外,在中东 阿联酋出现首例武汉肺炎确诊病例

크리스티안 린드마이어 세계보건기구(WHO) 대변인은 28일(현지 시간) “감염자가 어느 정도 증상이 나타나야 우한 폐렴을 전파할 수 있는지는 단정할 수 없다”며 무증상 감염 가능성을 언급했다 Christian Lindmeier, spokesperson of the World Health Organization, said on Tuesday (local time) that the WHO cannot conclude the level of symptoms, which allows the infectee to spread the virus," hinting the chance of asymptomatic infections

世界卫生组织发言人克里斯蒂安?林德迈尔28日(当地时间)表示,“不能断定感染者在多大程度上出现症状才能传播武汉肺炎”,提到了无症状感染的可能性

하지만 박혜경 질병관리본부 중앙방역대책본부 총괄팀장은 “WHO 문건에는 그런 문구가 없고 무증상자로 인한 감염 근거는 없다고 돼 있다”고 말했다 However, Park Hye-Kyung, the head of the Central Headquarters for Emergency Operations at the Korean Center of Disease Control, objected to this statement, saying, "There is no such wording on the WHO document and asymptomatic infections are groundless"

但是疾病管理本部中央防疫对策本部总管组长朴惠京表示:“WHO文件中没有这样的文字,没有因无症状而感染的根据”

29일 국내에서 추가 확진 환자는 발생하지 않았다 No further confirmed case was reported in Korea on Wednesday

29日,韩国国内没有出现新增确诊患者

시진핑을 흔든 ‘우한의 기침’ Wuhan’s cough shakes up Xi Jinping leadership

“武汉咳嗽”动摇习近平,增长率和领导能力面临危机

성장률-리더십 위기 ‘신종 코로나바이러스 감염증(우한 폐렴)’ 사태로 시진핑(習近平) 중국 국가주석의 지도력이 적잖은 타격을 입고 있다 Chinese President Xi Jinping’s leadership is suffering a blow due to a pneumonia outbreak arose in central China

受“新型冠状病毒感染症(武汉肺炎)”事件的影响,中国国家主席习近平的领导力受到了不小的打击

중국 정부는 위기관리 능력에 문제점을 드러냈고, 경제에도 그림자가 드리울 것으로 보인다 The Chinese government has just revealed its inability to manage crisis, and the economy is likely to take a hit

中国政府在危机管理能力上暴露出了问题,经济也将蒙上阴影

미 뉴욕타임스(NYT)는 “시 주석이 수년 만에 가장 심각한 정치적 위기를 맞았다”고 지적했다 The New York Times reported that Xi is facing the most severe political crisis for the first time in several years

美国《纽约时报》指出:“习主席遭遇了数年来最严重的政治危机”

○ ‘시진핑 리더십’에 비판 고조 시 주석은 지난해 12월 30일 우한시 당국이 우한 폐렴 발병 사실을 밝힌 뒤 25일이 지난 이달 25일에야 처음으로 우한 폐렴 대응을 공식적으로 언급했다 President Xi officially announced the government’s response to the Wuhan coronavirus as late as Saturday, which is 25 days after the Wuhan authorities announced the breakout of Wuhan pneumonia on December 30 last year

○对“习近平领导力”的批评加剧自去年12月30日武汉市政府公布“武汉肺炎”发病情况后,25天之后的本月25日,习主席才首次正式提及应对“武汉肺炎”

정부의 미온적인 대응에 불만이 높아지자 27일 리커창(李克强) 총리는 시 주석의 지시로 폐렴 진원지인 후베이(湖北)성 우한(武漢)을 찾았다 As dissatisfaction towards the government’s nonchalant attitude grew, Chinese Premier Li Keqiang went to Wuhan City, Hebei province, which is the epicenter of the virus,

由于对政府的消极应对感到不满加剧,李克强总理27日在习近平主席的指示下访问了肺炎发源地湖北省武汉

그는 병원과 마트 등을 방문해 의료진과 주민들을 격려했다 and visited hospitals and marts to encourage medical staff and residents

他前往医院和超市等场所,鼓励医护人员和居民

임시 격리병동 건설 현장에서는 노동자들에게 “어려움이 있으면 말하라 해결해 주겠다”고 했다 “Tell me if you are having problems I will solve them for you,” he said to construction workers who were building a temporary isolation ward

在临时隔离病房建设现场,李克强向工人们说:“有困难就说吧我会帮你解决的”

현장에 모인 사람들은 “(어려움이) 없습니다 ”라고 외쳤다 “There is no problem ” the workers said

在场的人高喊:“没有(困难)

하지만 현지 매체에 따르면 하루 전까지 이들은 마스크와 의료용 장갑 등 물자 부족에 대한 어려움을 토로했다 But local news outlets reported that the workers talked about lack of resources such as masks and medical gloves a day before his visit

不过据当地媒体报道,直到前一天,他们还是透露了口罩和医用手套等物资短缺的困难

소셜미디어 웨이보에는 “각지에 도움을 구하더니 갑자기 말을 바꿨네 ” “참 중국적인 답”이라는 조소가 이어졌다 （no translation）

社交媒体微博中不断出现嘲笑声,“一直向各地寻求帮助,突然改口了”“真的是中国式回答”

정부의 보고 및 의사결정 체계에도 상당한 문제가 있음이 드러났다 There was a serious issue in the reporting and decision-making process as well

政府的报告及决策体系也暴露出相当多的问题

저우셴왕(周先旺) 우한시장은 관영 중국중앙(CC)TV에 “우리도 정보 공개에 대한 불만이 있다 “We are not happy with disclosure of information,” said Wuhan Mayor Zhou Xianwang

武汉市市长周先旺对官方的中国中央电视台说:“我们也对信息公开不满

지방정부인 우리는 관련 정보와 권한을 얻은 다음에야 정보를 공개할 수 있었다”고 불만을 표했다 “We, as a local government, were able to publicize information only after obtaining the authority and relevant information

作为地方政府,我们只有在获得相关信息和权限后才能公开信息”

중국 의료체계의 취약점도 드러났다 The Chinese medical system revealed vulnerabilities as well

中国医疗体系的薄弱环节也暴露出来

NYT에 따르면 우한 주민인 샤오시빙 씨(51)는 발열과 호흡 곤란을 겪었지만 우한시 병원 병상 부족으로 병원을 전전하다가 보름 이상 지난 26일에야 입원했다 According to The New York Times, Xiao Shibing (51), a resident in Wuhan, had a fever and difficulty in breathing about 15 days ago, but was hospitalized on Sunday because there were not enough beds in hospitals

据《纽约时报》报道,51岁的武汉居民肖西冰(音)虽然出现发烧和呼吸困难,但由于武汉市医院病床不足,一直辗转于医院,半个月后的26日才住进医院

아내 펑시우 씨는 “(환자를) 이리저리 공 차듯 한다”고 불만을 토로했다 “Hospitals kick around patients like they are a soccer ball,” said his wife Feng Xiu

妻子彭秀(音)不满地说:“(把病人)踢来踢去”

마궈창(馬國强) 후베이성 부서기 겸 우한시 서기는 26일 밤 기자회견에서 우한시 병동이 극도의 과부하 상태임을 인정했다 Wuhan Communist Party Secretary Ma Guoqiang acknowledged that hospitals in Wuhan were extremely overcrowded in a press conference on Sunday

湖北省委副书记 武汉市委书记马国强26日晚在记者招待会上承认,武汉市病区处于超负荷运转状态

그는 “최근 며칠간 매일 1만5000명의 발열 환자가 병원들에 몰려 길게 줄을 서는 문제가 발생했다”고 밝혔다 （no translation）

他说,“最近几天,每天都有15000名发烧患者涌进医院,出现排长队问题”

중국은 다음 달까지 우한시에 병상 1000개, 1300개 규모의 임시 격리 병동 2곳을 완공할 계획이다 （no translation）

中国计划到下个月为止在武汉市建设1000个病床 1300个规模的两个临时隔离病区

○ “경제성장률 4% 기록할 수도” 우한 폐렴 확산에 따른 노동 일수 감소, 관광 위축 등이 장기화하면 정부의 목표치인 성장률 6% 사수가 어려워질 가능성이 있다 If the virus reduces workdays and prolongs tourism, the Chinese government may not be able to meet the target growth rate of 6 percent

○“经济增长率可能降低到4%”如果“武汉肺炎”的扩散导致劳动天数减少 旅游萎缩等长期化,政府将很难达到6%的目标增长率

홍콩 사우스차이나모닝포스트(SCMP)는 미국의 중국 연구기관 플리넘을 인용해 “중국의 1분기 성장률이 4%대를 기록할 수 있다”고 전망했다 Hong Kong’s South China Morning Post projected that China’s growth for the first quarter could stand at the 4 percent range quoting a U S -based research center

香港《南华早报》援引美国中国研究机构“plenum”的话预测说:“中国第一季度的增长率可能会达到4%左右”

중국은 춘제 연휴를 다음 달 2일까지로 사흘 연장했으나 우한 폐렴이 폭증하면서 더 연장할 가능성이 크다 China has extended the spring festival holiday by three days until Sunday but is likely to extend it further due to the Wuhan pneumonia outbreak

中国把春节长假延长三天至下个月2日,但随着“武汉肺炎”的暴增,很有可能再次延长

금융 도시 상하이(上海)는 기업들에 다음 달 9일까지 업무를 재개하지 말라고 통보했다 The Shanghai government ordered businesses not to resume work until February 9

金融城市上海通知企业,在下月9日之前不要恢复业务

로이터통신은 상하이시의 이번 조치로 인해 해당 지역에 공장을 소유하고 있거나 현지 합작사를 운영하고 있는 테슬라, 제너럴모터스(GM), 폭스바겐이 피해를 입을 것이라고 전했다 Reuters reported that Tesla, General Motors and Volkswagen that are operating a joint venture or a factory in the region will suffer a loss

据路透社报道,上海市此举将使在相关地区拥有工厂或在当地经营合资公司的特斯拉 通用汽车和大众汽车蒙受损失

제조업 중심지인 쑤저우(蘇州)도 다음 달 8일 정오까지 업무를 중단하라고 기업들에 요구했다 （no translation）

制造业中心苏州也要求企业停止业务至下月8日中午

정보기술(IT) 기업 텐센트와 중국 최대 전자상거래 기업 알리바바 등은 직원들에게 14일간 재택근무를 하라고 통지한 것으로 알려졌다 （no translation）

据悉,信息技术企业腾讯和中国最大的电子商务企业阿里巴巴等已通知职员在家工作14天

자금성(紫禁城)과 만리장성, 상하이 디즈니랜드 등 주요 관광지도 잠정 폐쇄됐다 （no translation）

故宫 长城 上海迪斯尼乐园等主要旅游景点也暂时关闭

춘제 시즌이 피크인 영화 개봉도 잇따라 연기됐다 （no translation）

春节档期为高峰的电影上映也相继推迟

중국 농구리그(CBA)도 무기한 중단됐다 （no translation）

中国篮球联赛也无限期中断

베이징(北京)은 춘제 연휴가 끝나더라도 후베이성 등 지방에서 돌아온 시민들에게 14일간 집에서 자가 격리할 것을 권고했다 （no translation）

北京还建议,即使春节假期结束,从湖北等地方回来的市民在家自行隔离14天

일부 대기업은 의료진을 지원하기 위한 펀드 조성에 나섰다 （no translation）

一些大企业为了支援医疗团队,开始建立基金

바이두(百度)는 27일 우한 폐렴 치료를 위한 연구개발 등을 지원하기 위해 3억 위안(약 506억 원)에 달하는 기금을 조성하겠다고 밝혔다 （no translation）

百度27日表示,将筹集3亿元人民币(约合506亿韩元)的基金,用于支援治疗“武汉肺炎”的研发等

음식 배달 앱 메이탄도 의료진을 지원하기 위한 2억 위안(약 337억 원) 규모의 기금을 조성한다 （no translation）

送餐应用程序“美团”也将成立2亿元人民币(约合337亿韩元)的基金,支援医疗团队

이와 함께 우한 시내 의료진에 매일 1000회의 무료 테이크아웃 식사 제공권과 후베이성 내 의료진에 30만 대의 공용 자전거 무료 이용권을 약속했다 （no translation）

美团还承诺向武汉市内医务人员提供每天1000次的免费外卖券以及湖北省内医务人员免费使用30万辆公用自行车的优惠券

우한 폐렴 경보신종 코로나바이러스인 우한 폐렴이 확산 일로다 Warnings of Wuhan pneumonia Wuhan pneumonia caused by a novel coronavirus is spreading like wildfire

武汉肺炎警报新型冠状病毒引发的“武汉肺炎”呈扩散趋势

지난해 12월 31일 중국 후베이(湖北)성 성도(省都)인 우한(武漢)에서 발생한 지 한 달도 되지 않아 미국 1명을 포함해 8개국에서 확진 환자 581명이 발생했다 It has been less than a month since it first appeared in Wuhan, the capital city of Hubei province, on December 31, 2019, but the virus has already reached eight countries, including the United States, with 581 confirmed cases

去年12月31日,在中国湖北省省会武汉发生疫情后不到一个月,包括一名美国患者在内,全世界8个国家共确诊病例581例

이 중 사망자는 17명으로 모두 중국인이다 Seventeen people who have died are all Chinese

其中死亡17人,均为中国人

한국도 인천공항에서 감염자로 의심돼 격리된 중국인 1명이 확진 판정을 받았다 In the meanwhile, South Korea has also reported one confirmed case The Chinese female patient, who displayed symptoms at the Incheon International Airport, has been quarantined as of today

一名中国人在韩国仁川机场被疑感染,隔离检查后已被确诊

교통 요충지로 ‘중국의 배꼽’이라 불리는 인구 1100만 도시 우한은 23일 오전 10시부터 도시로 들어가고 나가는 모든 대중교통 운행이 중단됐다 Despite being China’s transport hub with a population of 11 million, Wuhan has suspended all public transport services in and out of the city as of 10 a m on Thursday,

作为交通要塞 有着“中国肚脐”之称的1100万人口的武汉,从23日上午10点开始,所有进城 出城的公共交通全部停运

중국 역사상 성도 봉쇄는 처음이다 becoming the first provincial capital city that has been shut down

这是中国历史上第一次封锁省会城市

▷우한 폐렴의 숙주는 박쥐나 뱀으로 알려져 있다 It is thought that the host of Wuhan pneumonia is bats or snakes

据悉,武汉肺炎的宿主是蝙蝠或蛇

사스(SARS·중증급성호흡기증후군)는 박쥐와 사향고양이, 메르스(MERS·중동호흡기증후군)는 낙타였다 Severe acute respiratory syndrome (SARS) started in bats or civet cats while Middle East respiratory syndrome (MERS) in camels

非典(SARS)的宿主是蝙蝠和麝香猫,中东呼吸综合征的宿主是骆驼

우한 폐렴 발원지인 수산물시장에서는 온갖 야생동물이 불법으로 거래돼 왔는데 두 번째 사망자도 수산물시장 가게 주인이었다 The outbreak began at a seafood market in Wuhan where a variety of wild animals are illegally traded, and the second person who died from the virus owned a store in the market

在武汉肺炎发源地水产市场,一直有各种野生动物的非法交易,第二名死亡者也是水产市场店主

잠복기는 짧게는 2, 3일, 길면 10∼12일이다 It can take up to 10 to 12 days to develop symptoms, which are similar to those of a common cold or a flu

潜伏期短则两三天,长则10-12天

증세는 감기나 독감과 비슷하고 치료제나 백신은 없다 There is no treatment or vaccine yet

症状与感冒和流感相似,没有治疗剂和疫苗

감염자의 침이나 콧물로 전파되므로 사람이 많은 곳에 갈 땐 마스크를 써야 한다 Given that Human-to-human transmission can occur when someone comes in contact with an infected person’s secretions such as saliva and mucus, it is advised to wear a mask when in public places

因为会以感染者的唾液或鼻涕传播,所以去人多的地方时要戴口罩

마스크에 바이러스가 묻을 수 있으므로 한 번 쓰고는 버리는 것이 좋다 A mask should not be reused as it might have viruses on it

口罩可能会沾上病毒,最好戴上一次就扔掉

▷홍콩 전문가들은 우한 폐렴이 2003년 사스 때처럼 대유행할 조짐이 있다고 경고한다 Experts in Hong Kong warn that the outbreak might evolve into a pandemic as SARS did in 2003

香港专家警告说,武汉肺炎有像2003年非典时期那样大流行的迹象

전염병 확산은 동물에서 인간→인간 간 전염→환자 가족과 의료진에 전염→대규모 발병 단계로 진행되는데 우한 폐렴은 마지막 단계 진입을 코앞에 두고 있다는 것이다 According to them, infectious diseases are first transmitted from animals to humans, from humans to humans and then from patients to their families and medical staff before they become a full-fledged pandemic, and Wuhan pneumonia is already in the third stage

传染病的扩散会经历从动物传染到人类 人际传染 传染给患者家属和医疗团队 进入大规模发病阶段的不同阶段,而武汉肺炎已是进入最后阶段在即

중국의 정보 통제와 뒷북 대응이 바이러스 확산을 가속화했다는 비판이 나온다 China has come under criticism for accelerating the spread of the virus with its cover-up and late response

有批评指出,中国的信息控制和马后炮应对加速了病毒的扩散

우한은 봉쇄됐지만 이미 수백만 우한 시민이 빠져나간 것으로 추정된다 Wuhan has been shut down, but it is estimated that millions of people have already left the city

武汉虽被封锁,但据估计已有数百万武汉市民走出城市

뉴욕타임스는 중국이 언론과 시민사회를 통제하고 정부가 정보를 독점하다 사태를 키웠다고 지적했다 The New York Times said the problem has exacerbated by the Chinese government’s tight control over information, the media and a civic society

《纽约时报》指出,中国控制媒体和市民社会,政府垄断信息,导致事态扩大

▷우한 봉쇄 소식에 중국 주가지수가 폭락했다 In the meantime, the lockdown of Wuhan has tumbled down China stocks

封锁武汉的消息导致中国股指暴跌

세계 경제가 입을 피해 규모도 2003년 사스 때를 능가할 것으로 보인다 It is expected that the economic damages caused by the novel coronavirus would be larger than those inflicted by SARS in 2003,

世界经济遭受的损失规模也将超过2003年非典时期

세계 경제에서 중국이 차지하는 비중이 2003년 8. 7%에서 올해는 20%로 커질 것으로 전망되기 때문이다 because China’s share of the global economy is estimated to increase to 20 percent from 8. 7 percent in 2003

因为在世界经济中,预计中国所占的比重将从2003年的8.7%增加到今年的20%

회복 기미를 보이던 한국 경제도 연초부터 악재를 만났다 This could also impact the South Korean economy, which was showing signs of recovery, from the beginning of the year

呈现恢复迹象的韩国经济也从年初开始就遇到了不利因素

5년 전 메르스 때처럼 소비가 얼어붙어 성장률 반등에 지장을 줄 우려가 있다 Consumption might freeze just like five years ago, delaying a rebound of the growth rate

人们担心,像5年前的“中东呼吸综合征”时一样,消费冻结会影响增长率的反弹

▷오늘 시작되는 설 연휴가 1차 고비다 The first crisis is the Luna New Year holiday, which starts today

今天开始的春节长假是第一个关口

이동이 많고 사람이 모여 감염 우려가 높지만 대다수 병원은 문을 닫는다 With many people traveling and gathering, the risk of transmission goes up while most hospitals close

虽然移动较多,人员聚集,感染的可能性较大,但大多数医院都关门

중국인 관광객 14만 명도 몰려들 것으로 예상된다 During the Lunar New Year holiday, more than 140,000 Chinese tourists are expected to flock to South Korea

预计将吸引14万名中国游客

정부 당국은 24시간 비상방역체계를 가동하고, 개인은 관련 정보에 귀를 열어둔 채 손 소독과 마스크 쓰기를 비롯한 전염병 예방행동수칙을 따라야 한다 The Korean government should operate a 24-hour emergency disease control system while citizens should closely follow the relevant news as well as the guidelines to prevent the spread of the virus such as washing hands and wearing masks

韩国政府相关部门应启动24小时应急防疫体系,个人应倾听相关信息,并遵循包括手消毒和戴口罩在内的传染病预防行为守则

中, 폐렴 발원지 우한시 뒤늦게 전면봉쇄China shuts down Wuhan where pneumonia began

中国昨天才全面封锁肺炎发源地

신종 코로나바이러스로 인해 발생한 ‘우한 폐렴’ 확진자와 사망자가 크게 늘자 중국 당국이 뒤늦게 바이러스 발원지인 후베이성(湖北)성 우한(武漢)시를 긴급 봉쇄했다 Amid increasing confirmed cases of “Wuhan pneumonia” and fatalities, the Chinese authorities has shut down the city where the virus began

武汉市因新型冠状病毒导致的“武汉肺炎”确诊病例和死亡病例大幅增加,中国当局事后紧急封锁了病毒发源地湖北省武汉市

인구 1100만 명의 우한시는 후베이성의 성도(省都)다 Wuhan is the capital city of Hubei province with a population of about 11 million

拥有1100万人口的武汉市是湖北省省会城市

성도급 도시를 봉쇄한 것은 사상 처음이다 It is the first time for the nation to shut down the capital city of a province

这是历史上第一次封锁省级城市

우한시 당국은 23일 오전 2시(현지 시간) 긴급 성명에서 “이날 오전 10시부터 버스와 지하철, 페리, 시외 장거리 버스 운행을 중단한다”며 In an emergency statement released at 2 a m on Thursday (local time), the city of Wuhan announced a suspension of bus, subway, ferry and intercity bus services as of 10 a m

当地时间23日凌晨2时,武汉市有关部门在一份紧急声明中说:“从当天上午10时起,将停止运营公交车 地铁 渡轮和长途汽车”,

“시민들은 특수한 상황이 아니면 우한을 떠나지 말라 항공편과 열차편도 임시 중단하고 재개 시점은 추후 통지하겠다”고 밝혔다 Those living in the city are advised not to leave the city, and airports and train stations have also been closed with no date or time set for the services to resume

“市民如不是特殊情况,不要离开武汉航班和列车也将暂时中断,届时将通知恢复时间”

중국 중부에 위치한 우한은 주변 9개 성(省)과 연결된 교통의 요충지이기도 하다 Located in the middle of China, Wuhan is a transport hub that connects nine neighboring provinces

位于中国中部的武汉也是连接周边9个省的交通要塞

전염병 대유행(팬데믹) 조짐이 보이자 다급해진 중국 당국이 초유의 조치를 내린 것으로 풀이된다 The unusual lockdown comes as the risk of the virus turning into a global pandemic grows

分析认为,在看到传染病大流行的征兆后,着急的中国当局采取了前所未有的措施

하지만 중국 내 우한 폐렴 확진 환자는 이날 600명으로 집계돼 전날보다 56명 늘었다 However, the confirmed cases have increased by 56 to 600 as of Thursday

但是据统计,当天中国国内武汉肺炎确诊患者为600人,比前一天增加了56人

중국 31개 성, 시(市) 가운데 27개에서 확진 또는 의심 환자가 발생했다 우한 폐렴 영향권에 들지 않은 지역은 서부의 간쑤(甘肅)성, 칭하이(靑海)성, 신장(新疆)위구르자치구, 티베트자치구 등 4곳만 남았다 Suspected and confirmed cases have been identified in 27 Chinese provinces out of 31, leaving only four provinces, Gansu, Qinghai, Xinjiang and Tibet, unaffected

中国31个省 区 市中有27个出现确诊或疑似患者没有进入武汉肺炎影响圈的地区只剩下西部的甘肃省 青海省 新疆维吾尔自治区和西藏自治区等4个

중남미의 멕시코 브라질 콜롬비아, 북미의 캐나다에서도 의심 환자가 발생하는 등 전 세계적으로 우한 폐렴 확산 우려가 커지고 있다 Fears of a global pandemic are growing as suspected cases have also been detected in Mexico, Brazil, Columbia and Canada

拉美的墨西哥 巴西和哥伦比亚,北美的加拿大也出现了疑似患者等,全世界对武汉肺炎扩散的担忧正在加大

이에 따라 외교부는 이날 중국 우한에 여행경보 2단계(여행 자제)를 발령했다 （no translation）

因此,韩国外交部当天向中国武汉发布了二级旅游警报(克制旅行)

우한 폐렴과 관련해 정부가 여행경보를 내린 것은 처음이다 （no translation）

这是政府首次就武汉肺炎问题发布旅游警报

현재 2단계 여행경보 지역은 미국과 무력 갈등을 빚고 있는 이란, 대규모 시위가 이어졌던 홍콩 등이다 （no translation）

目前,二级旅游警报地区目前包括与美国发生武力纠纷的伊朗 持续举行大规模示威的香港等地

외교부는 후베이성 전역엔 여행경보 1단계(여행 유의)를 발령했다 （no translation）

外交部向湖北省全境发布了一级旅游警报(注意旅行)

외교부는 “이 지역(우한 등)을 여행할 예정인 국민들은 여행 필요성을 신중히 검토해 주시고, 체류 중인 국민은 신변 안전에 특별히 유의해 달라”고 당부했다 （no translation）

外交部叮嘱道:“计划去该地区(武汉等)旅游的国民要慎重考虑旅行的必要性,滞留当地的国民要特别注意人身安全

질병관리본부는 신속한 현지 상황 파악 등을 위해 이날 중국 베이징(北京) 한국대사관에 역학조사관을 파견했다 （no translation）

”疾病管理本部为了迅速掌握当地情况,当天向中国北京韩国大使馆派遣了流行病学调查官

美로 번진 우한폐렴, 대유행 조짐 ‘Wuhan pneumonia’ may become a global epidemic

“武汉肺炎”扩散到美国,出现大流行迹象

미국에서 신종 코로나바이러스로 인한 ‘우한 폐렴’ 첫 확진 환자가 발생하고, 중국에서 환자 폭증세가 이어지면서 전 세계적인 대유행 조짐을 보이고 있다 “Wuhan pneumonia,” caused by a novel coronavirus, is showing signs of wide outbreak across the world, with the first U S case confirmed and infections spreading across China

美国出现首例因新型冠状病毒引起的“武汉肺炎”确诊病例,而在中国,患者人数持续暴增,全球出现大流行迹象

21일(현지 시간) 로이터통신에 따르면 미국 질병통제예방센터(CDC)는 최근 바이러스 발생지인 중국 후베이(湖北)성 우한(武漢)으로 여행을 다녀온 30대 남성이 우한 폐렴 확진 판정을 받았다고 밝혔다 According to Reuters News on Tuesday, the United States Center for Disease Control confirmed that a resident near Seattle, Washington was diagnosed with the Wuhan pneumonia after visiting Wuhan, China

据路透社当地时间21日报道,美国疾病控制和预防中心表示,最近一名在中国湖北省武汉旅游过的30多岁男性被确诊为武汉肺炎

미국 워싱턴주 시애틀 인근에 거주하는 이 남성은 15일 귀국한 뒤 치료를 받고 있다 He is now under treatment since returning to the U S last Wednesday

这名男子居住在美国华盛顿州西雅图附近,15日回国后正在接受治疗

아시아 외 대륙에서 확진 환자가 나온 것은 처음이다 It is the first confirmed case outside Asia

这是亚洲以外大陆首次发现确诊病例

중국 질병예방통제센터는 22일 우한 폐렴 확진 환자가 440명(대만 포함)으로 늘었다고 밝혔다 The Chinese Center for Disease Control announced Wednesday that the number of confirmed cases grew to 440 (including Taiwan),

中国疾病预防控制中心22日宣布,武汉肺炎确诊病例增至440例(含台湾)

하루 만에 130여 명이 증가한 것이다 meaning that around 130 cases increased in just one day

仅一天就增加了130多人

13개 중국 성(省), 시(市)에서 확진 환자가 발생했다 Confirmed patients were found across 13 cities and provinces across the nation

13个省 市出现了确诊病例

의심 환자까지 합치면 21개 성, 시가 영향권에 들어 중국 31개 성, 시의 68%에 달했다 When including suspected patients, the number of potentially infected areas would include 21 cities and provinces, accounting for 68% of China’s 31 cities and provinces

加上疑似患者,中国共有21个省 市受到影响,占中国31个省 市的68%

이날 마카오에서도 처음으로 확진 환자가 발생했다 Against this backdrop, Macao also confirmed its first confirmed case on the day

当天,澳门也出现首例确诊病例

중국 내 사망자도 6명에서 9명으로 늘어났다 Also the number of deaths increased from six to nine in China

中国国内死亡人数也从6人增至9人

당국이 관찰 중인 밀접 접촉자가 1394명에 달해 환자 급증세가 이어질 것으로 보인다 With 1,394 people who had close contract with the patients put under medical observation, the number of patients is expected to increase

医疗部门正在观察的密切接触者达1394人,预计患者人数将会持续激增

중국 관영 신화통신은 “바이러스의 변이 가능성이 있어 전염 상황이 더욱 확산될 위험이 있다”는 국가위생건강위원회 전문가팀의 지적을 전했다 （no translation）

中国官方媒体新华社引述国家卫生与健康委员会专家组的报告指出,“病毒可能发生变异,传染情况有进一步扩散的危险”

이들은 “지역사회 전파도 있다”고 밝혔다 （no translation）

专家们表示:“还有地区内传播”

일부 지역에서 집단 감염 사례가 있음을 인정한 것이다 （no translation）

等于是承认了一些地区存在集体感染病例

전문가팀의 중난산(鍾南山) 팀장은 “슈퍼 전파자 출현을 막아야 한다”고 경고했다 （no translation）

专家组组长钟南山警告说:“必须防止超级传播者出现”

홍콩 사우스차이나모닝포스트(SCMP)는 2003년 사스(SARS·중증급성호흡기증후군) 때와 같은 전면적 확산 단계에 진입할 것이라고 전망했다 （no translation）

香港《南华早报》预测说,将像2003年非典(SARS)时期一样进入全面扩散阶段

중국 14억 인구 중 4억5000만 명 이상이 이동하는 춘제(중국의 설·25일)가 다가와 대유행의 분수령이 될 것으로 보인다 The Chinese New Year Day (Jan 25), which more than 450 million out of 1. 4 billion Chinese travel, is expected to be a turning point of the epidemic

中国14亿人口中有超过4.5亿人以上移动的春节(25日)即将到来,预计将成为大流行的分水岭

사스 사태급 대응을 천명한 중국 당국은 우한으로 가거나 우한을 떠나지 말라는 우한 여행 자제 권고령을 내렸다 The Chinese authorities have urged people to stop travelling in and out of Wuhan

中国政府明确表示将采取非典级紧急应对,下达了“不要去武汉或离开武汉”的武汉旅游克制呼吁令

국내에서도 우한 폐렴과 유사한 증상을 보이는 ‘유증상자’ 4명이 추가로 발생했다 （no translation）

韩国国内也新增了4名类似武汉肺炎症状的“有症状者”

이로써 국내 우한 폐렴 유증상자는 19일 확진 판정을 받은 중국인 여성 A 씨(35)를 포함해 총 16명으로 늘었다 （no translation）

至此,包括19日被确诊的中国女患者A某(35岁)在内,国内武汉肺炎患者总数增至16人

새로 추가된 유증상자 중 3명에는 A 씨와 함께 우한에서 인천행 비행기를 탄 승객 및 공항 관계자가 포함된 것으로 알려졌다 （no translation）

据悉,新增加的“有症状者”中有3人是与A某一起从武汉飞往仁川的乘客及机场负责人

문재인 대통령은 22일 상황을 보고받은 뒤 “검역 및 예방 조치에 만전을 기함과 동시에 경제에 미치는 영향도 종합 점검하라”고 지시했다고 한정우 청와대 부대변인이 전했다 （no translation）

青瓦台副发言人韩正宇表示,文在寅总统在22日听取情况报告后指示,“在做好检疫和预防措施的同时,还要综合检查对经济的影响”

문 대통령은 전날 정부세종청사에서 열렸던 국무회의에서도 “방역에 각별히 힘써 달라”는 취지의 지시를 한 것으로 알려졌다 （no translation）

据悉,文在寅总统前一天在政府世宗办公大楼举行的国务会议上,也下达了“请格外注意防疫”的指示

춘절 앞둔 中, ‘사스 공포’ 재현되나‘Fears of SARS’ rise in China ahead of Lunar New Year holiday

春节在即,中国会否再现“非典恐慌”

중국 내에서 신종 코로나바이러스로 인한 ‘우한 폐렴’ 환자 수가 폭발적으로 늘어나면서 ‘중국의 방역체계가 뚫린 것 아니냐’는 우려가 커지고 있다 The sharp rise of “Wuhan pneumonia” due to a new coronavirus in China has prompted concerns over the Chinese government’s ability to contain infectious disease outbreak

随着中国国内新型冠状病毒引发的“武汉肺炎”患者数量急剧增加,人们越来越担心“中国的防疫体系是否已被突破”

중국 누리꾼들은 당국의 은폐, 늑장 대응 의혹까지 제기했다 Some Chinese Internet users are accusing the government of a cover-up and late response

中国网民甚至怀疑当局隐瞒真相,应对迟缓

2002∼2003년 사스(SARS·중증급성호흡기증후군) 발생 초기 중국 당국이 전염 사실을 은폐하는 바람에 초기 대응에 실패해 중국과 홍콩에서만 648명이 목숨을 잃은 전례가 되풀이되는 것 아니냐는 목소리도 나온다 while others worry if the virus could become as fatal as SARS, which killed 648 people across mainland China and Hong Kong in 2002 and 2003 after Beijing failed to contain the spread

有人质疑,2002年至2003年非典疫情爆发初期,中国有关部门隐瞒疫情,导致疫情初期应对不力,仅中国内地和香港就有648人死亡,这一先例是否会重演

우한 폐렴은 전염 규모와 범위 모두 걷잡을 수 없이 커지는 양상이다 “Wuhan pneumonia” are spreading rapidly and widely

武汉肺炎的传染规模和范围都呈不可控制的扩大态势

발원지인 후베이(湖北)성 우한(武漢)에서 18, 19일 이틀 동안 확진 환자가 136명 늘면서 기존 환자의 3배 이상으로 증가했다 In Wuhan, Hubei province, where the virus was first detected, confirmed cases tripled over the weekend by 136

发源地湖北武汉18日和19日两天新增确诊病例136例,是现有病例的3倍以上

또 베이징(北京) 남부 다싱(大興)구와 광둥(廣東)성 선전(深(수,천))시에서 각각 확진 환자 2명, 1명이 발생했고 선전시에서 8명, 저장(浙江)성에서 5명의 의심 환자가 나와 중국 전역으로 확산될 조짐을 보이고 있다 In addition, it appears that the virus is spreading across the country Two and one cases were confirmed in Daxing and Shenzhen, respectively, while eight suspected cases were found in Shenzhen and five in Zhejiang

另外,北京南部大兴区和广东省深圳市分别出现2名和1名确诊患者,深圳市出现8名疑似患者,浙江省出现5名疑似患者

‘전염력이 약하고 사람 간 전염 가능성이 낮다’고 강조해온 중국 당국의 발표와는 정면으로 배치되는 것이다 It contradicts the Chinese authorities’ claim that the virus is not highly contagious and not likely to be transmitted through human to human contact

这与中国有关部门强调“因为传染力很脆弱,人与人之间传染的可能性极低”的说法大相径庭

다싱구에는 지난해 운영을 시작한 베이징신공항이 있다 （no translation）

在大兴区,有一个去年投入运营的北京新机场

베이징시 당국은 확진 환자가 우한에 여행을 다녀왔다고 밝혔지만 언제 어떤 경로로 전염됐는지 공개하지 않았다 （no translation）

北京市有关部门表示,确诊患者曾到武汉旅游,但并未公开何时通过何种途径感染

상하이(上海)시 당국은 “일부 의심 환자에 대해 전염 방지 조치를 취했다”고 밝혔지만 의심 환자 수는 공개하지 않았다 （no translation）

上海市当局表示,“对部分疑似患者采取了防止传染措施”,但并未公开疑似患者人数

선전시 당국은 확진 환자 1명이 우한에 친척을 만나러 다녀온 66세 남성이라고 밝혔다 （no translation）

深圳市有关部门表示,一名确诊患者是曾去武汉探亲的66岁男子

하지만 인도 통신사인 PTI는 선전시 국제학교 교사인 인도인 프리티 마헤슈와리 씨(45·여)가 확진 판정을 받아 선전시 병원에서 격리 치료 중이라고 보도했다 （no translation）

但“印度报业托拉斯”通讯社报道说,深圳市国际学校教师 印度人普里蒂·马赫什瓦里(45岁,女)被确诊,目前正在深圳市医院隔离治疗

이 보도가 사실이라면 중국 당국이 인도인 환자 발생 사실을 숨긴 셈이 된다 （no translation）

如果该报道属实,那么相当于中国有关部门隐瞒了印度患者发病的事实

더욱이 이 여성은 우한에 간 적이 없다고 PTI는 전했다 （no translation）

更何况,印报托称,这名女子从未到过武汉

그동안 지방 당국 차원에서만 대응해오던 중국은 19일에야 국가위생건강위원회가 예방 대책을 발표하고 중국 전역에 실무대응팀을 파견했다 The Chinese government had dealt with the outbreak at the municipal level, but China’s National Health Commission announced prevention measures and sent working groups to provinces across the country on Sunday

此前,中国一直只从地方当局层面进行应对,直到19日国家卫生健康委员会才公布预防对策,并向全中国派遣了工作组

중국 당국은 “바이러스 전염의 원천을 찾지 못했고 전파 경로도 완전히 파악하지 못했다”고 밝혔다 The authorities said it had not identified the source of the outbreak as well as the exact transmission pathways

中国当局表示:“没有找到病毒传染的源头,也没有完全掌握传播途径”

한국 정부 당국자는 “중국 당국이 새로운 조사 방법을 적용했더니 우한에서 확진 환자 수가 크게 늘었다고 전해왔다”고 말했다 A source from the Korean government said, “The Chinese authorities applied a new method, which has driven up confirmed cases in Wuhan”

韩国政府高级官员表示:“据传,中国当局使用了新的检查方法,武汉的确诊患者人数大幅增加”

특히 중국은 춘제(春節·중국의 설)를 전후해 연인원 30억 명이 대이동할 것으로 예상돼 걷잡을 수 없는 대유행으로 악화할 가능성을 배제할 수 없다 The possibility of this turning into a major outbreak cannot be ruled out given that about 3 billion people are expected to travel over the Lunar New Year holiday

特别是中国春节前后将有30亿人大移动,不能排除因无法控制而发展成为大流行的可能性

중국 누리꾼들의 불신도 확산하고 있다 Concerns are rising among Chinese Internet users as well

中国网民的不信任也在扩散

한 누리꾼은 중국 소셜미디어 웨이보에 “전염력이 약하다더니 이렇게 급증했나 또 속이고 숨기는가”라고 썼다 “애국 바이러스라는 말은 더 이상 안 나오겠네”라고 비꼬는 글도 보였다 “The government said it is unlikely to be highly infectious Was it another cover-up ”, said a person Another person left a sarcastic comment saying, “The virus is no longer patriotic

某网民在中国社交媒体微博上留言说:“听说传染力很弱,患者如此激增又要骗人 隐瞒吗”还有人留言讽刺说:“不会再有人说爱国病毒了吧”

중국몽 위협하는 새로운 세대의 등장지난해 12월과 이달 대만 대선을 취재하기 위해 타이베이를 방문했다 ” New generation poses threat to ‘Chinese Dream’ This reporter visited Taipei last December to cover the presidential election in Taiwan

威胁中国梦的新一代登场去年12月和本月,为采访台湾大选,记者访问了台北

현장에서 만난 20대 대만 젊은이들은 자신을 “톈란두(天然獨)”라고 묘사했다 Young Taiwanese in their 20s described themselves “Born independent

记者在现场看到,一群20多岁的台湾年轻人形容自己是“天然独”

어릴 때부터 대만이 독립된 주권을 가진 국가라고 여기며 자랐다는 뜻이다 ” It means they were raised believing Taiwan is an independent country

意思是说,从小开始就认为台湾是拥有独立主权的国家

대학 4학년인 청(程·22)모 씨는 “그래서 우리는 중국이 제기한 일국양제(一國兩制·1국가 2체제) 통일 방안을 받아들일 수 없다”고 말했다 “This is why we cannot accept China’s one country, two systems,” said a Taiwanese university student Taiwanese

大四学生程某(22岁)说:“所以我们不能接受中国提出的‘一国两制’统一方案”

현지에서 만난 대만의 2030세대는 자신을 중국인이 아닌 대만인이라고 여겼다 who are in their 20s and 30s see themselves as Taiwanese, not Chinese

在当地见到的台湾20岁至39岁的人,认为自己不是中国人,而是台湾人

이들의 표심이 반중(反中) 성향 차이잉원(蔡英文) 총통 재선의 원동력이었다 Their votes were what secured the second term of Taiwanese President Tsai Ing-wen who opposes closer ties with China What has given them strong Taiwanese identity was the country’s democratization

他们的选票是反华倾向的蔡英文连任总统的原动力

1980년대에 태어난 젊은이들을 기성세대와 달라지게 한 건 대만의 민주화였다 Despite the democratic movement in the 1980s,

让20世纪80年代出生的年轻人与老一辈变得不同的,是台湾的民主化

1980년대 민주화운동을 거쳐 1996년 총통직선제가 도입되기 전 대만은 중국에서 건너온 외성인(外省人)이 주축이 된 국민당이 장기 집권한 권위주의 사회였다 Taiwan remained an authoritarian society with the Kuomintang party, which mostly consisted of Mainlanders, in power until 1996 when the first direct presidential election was held

经过上世纪80年代的民主化运动,直到1996年实行总统直选制之前,台湾是一个以来自中国的外省人为主的国民党长期执政的权威主义社会

1970년대 이전에 태어난 ‘구세대’는 중국과 통일해야 한다는 ‘톈란퉁(天然統)’ ‘당란퉁(當然統)’이 많다 반면 어릴 때부터 민주화를 경험한 젊은이들은 대만인으로서의 정체성이 강하다 Many from older generations, who were born before the 1970s, support unification with China whereas young Taiwanese who were born in a democratic society has strong Taiwanese identity

上世纪70年代以前出生的“老一代”有很多是“天然统”“当然统”,主张应当与中国统一相反,从小开始经历民主化的年轻人作为台湾人的认同感很强

자신이 중국인이냐 대만인이냐, 중국과 통일해야 하느냐 독립해야 하느냐 혼란을 겪는 40대 이상 대만인들과 다르다 The young Taiwanese are neither confused on their identity nor torn between unification and independence unlike older generations over 40

他们与搞不清自己到底是中国人还是台湾人 是应该与中国统一还是独立,头脑混乱的40岁以上台湾人不同

대만에서 만난 전문가들은 “젊은이들은 과거엔 취업 등 자신의 문제에만 관심을 가졌다 “Young people used to be only interested in personal issues such as finding a job,” said pundits in Taiwan

在台湾见到的专家说:“年轻人过去只关心就业等自身问题

하지만 자신들의 미래를 중국과 통일을 원하는 은퇴한 구세대에 맡겨서는 안 된다는 위기의식이 투표 열기로 이어졌다”고 주장했다 “The high turnout of young voters in the latest election, however, shows that they are determined not to leave their future in the hands of older generations

但是,不能将自己的未来交付给希望与中国统一的旧世代身上的危机意识,导致了投票热潮”

대만 대선이 차이 총통의 압승으로 끝나자 대만 언론들은 “이들이 이제 톈란두에서 톈란타이(天然臺)로 변하고 있다”고 분석했다 ” Following the landslide victory in the presidential election, Taiwanese newspapers reported that the younger generation is evolving from “born independent” into “born Taiwanese

在台湾大选以蔡英文的压倒性胜利结束后,台湾媒体分析说:“他们现在正从‘天然独’变成‘天然台’”

이들은 “이미 대만은 주권 국가이기 때문에 굳이 독립을 추구할 필요가 없고 현재의 대만 그 자체로 현상을 유지하기를 원한다”는 것이다 ” From their point of view, Taiwan is already an independent country, and there is no need to pursue independence

他们说,“台湾已经是一个主权国家,没有必要非要追求独立,而是希望维持目前台湾本身的现状”

대만 통일에 필요하다면 무력도 배제하지 않겠다는 시진핑(習近平) 중국 국가주석과는 대척점에 서 있는 셈이다 They want to maintain the status quo, which is the opposite of what Chinese President Xi Jinping wants

这与主张如果统一台湾需要不排除武力的中国国家主席习近平站在了对立的位置上

지난해 홍콩 시위 현장에서도 톈란두와 거의 똑같은 새로운 밀레니얼 세대의 출현을 목격했다 Last year, Hong Kong also witnessed the rise of the millennial generation in protests

去年在香港的示威现场,也目睹了与“天然独”几乎一模一样的新千年一代的出现

홍콩중문대 학생 찬모 씨(21·여)는 기자에게 “홍콩이 중국에 반환된 1990년대에 태어난 우리는 100% 홍콩에 속한 첫 세대”라고 말했다 A student at the Chinese University of Hong Kong said, “Born in the 1990s when Hong Kong was handed over to China, we are the first generation that belongs to Hong Kong 100 percent,”

香港中文大学学生陈某(21岁,女)对记者说:“我们出生于香港回归中国后的90年代,是百分之百属于香港的第一代人”

영국의 식민지가 아니면서 “중국과도 다른 체제를 보장받은 자유로운 사회 홍콩에서 교육 받으며 자란 우리는 중국인이 아닌 홍콩인”이라는 얘기였다 meaning they were Hong Kongers, not Chinese, who were educated in a free society with its own system independent of China

是在既不是英国殖民地同时也是“在受保障与中国不同体制的自由社会香港接受教育长大的,我们不是中国人,而是香港人”

‘중국 건국 100주년인 2049년까지 세계 강국으로 우뚝 서겠다’는 중국몽(夢)에는 일국양제의 성공도 포함된다 ” China aims to become a world power by 2049, which marks the 100th anniversary of its founding, and the success of “one country, two systems” is also part of the plan

“直到中国建国100周年的2049年成为世界强国”的中国梦中,也包括“一国两制”的成功

홍콩과 마카오의 일국양제를 발판으로 대만도 통일하겠다는 구상이다 Its aim is to unite with Taiwan based on the “one country and two systems” principal in Hong Kong and Macao

以香港和澳门的“一国两制”为基础,台湾也要实现统一

하지만 기존 세대와 완전히 다른 가치관을 가진 대만의 톈란두와 홍콩의 밀레니얼 세대가 새로운 변수로 떠올랐음을 세계가 목격했다 However, armed with new values, Taiwan’s “born independent” and Hong Kong’s Millennials are posing a threat to China’s plan

但是,全世界都看到,与过去一代人有着完全不同价值观的台湾的“天然独”和香港的千禧一代,已经成为新的变数

현장에서 이들이 중국인 정체성을 거부하는 것이 일시적 현상이 아니라는 점을 확인할 수 있었다 이들은 시간이 흐를수록 대만과 홍콩의 주축 세대가 될 것이다 It is clear that this anti-Chinese sentiment is not a passing trend, which will only grow over time

在现场,记者了解到,他们拒绝中国人认同感并非一时现象

대만과 홍콩에서 만난 전문가들은 “중국이 새로운 세대의 등장이라는 변화를 직시하지 못한 채 이를 중국에 대한 대항과 도전으로만 보고 강경 대응해 역효과가 났다는 것이 대만과 홍콩의 공통점”이라고 지적했다 Experts point out that, both in Taiwan and Hong Kong, China failed to recognize the rise of a new generation and took a hardline stance dismissing them as yet another group of protesters, which backfired

随着时间的推移,他们将成为台湾和香港的主轴一代。台湾和香港接受采访的专家表示:“中国未能直视新一代登场这一变化,仅认为这是对中国的对抗和挑战,予以强硬应对,这会带来相反的结果,这是台湾和香港的共同点”

어떤 정치세력이든 민심의 변화를 읽지 못하면 위기를 겪을 수밖에 없다는 점을 새삼 일깨워준다 This is a reminder that a crisis will ensue if politicians fail to read changing demands of the public

这再次提醒我们,不管是什么样的政治势力,如果不能了解民心的变化,就只能经历危机

美-中1단계 무역 합의 ‘Phase One’ deal

中美达成第一阶段贸易协议,韩国对华出口进入紧急状态

중국 수출 비상 걸린 한국미국과 중국이 2년 가까이 끌었던 무역전쟁에서 1단계 합의를 하며 휴전에 들어갔다 The almost-two-year trade war between the U S and China has entered a truce with the Phase One agreement

在中美持续近两年的贸易战争中,双方达成第一阶段协议,开始停火

세계 1, 2위 경제 대국들이 벌이는 싸움에 위축되고 불안했던 글로벌 경제는 일단 짙은 먹구름이 가셨다 Clouds over the global economy that had contracted and been unstable due to a battle between the world’s two largest economies are gone for now

受世界前两大经济大国的争斗影响,一度萎缩不安的全球经济暂时一扫乌云

그러나 중국 화웨이 제재를 비롯한 민감한 갈등은 2단계로 넘겨진 데다 1단계 합의 이행 과정이 순조롭지 못할 경우 언제든 관세 전쟁이 재개될 수 있어 불확실성은 여전하다 However, all sensitive issues, including sanctions against China’s Huawei, have been deferred to Phase Two In addition, a trade war can be resumed at any time in the event of a bumpy implementation of the Phase One agreement, which means uncertainties are still in place

但是,包括制裁中国华为在内的敏感矛盾移交到了第二阶段,如果再有第一阶段协议的履行过程不顺利,关税战争随时都有可能重启,不确定性依然存在

무엇보다 중국이 막대한 양의 미국 제품을 추가 수입하기로 함으로써 한국의 대중(對中) 수출에 큰 타격이 우려된다 More than anything, South Korea’s exports to China can be substantially affected as China has agreed to import a large volume of goods from the U S

最令人担忧的是,中国决定追加进口大量美国产品,这将对韩国的对华出口造成巨大打击

미중은 15일 중국이 미국산 제품을 2년 간 2000억 달러(약 232조 원) 어치 추가 구매하는데 합의했다 The U S and China came to an agreement on Wednesday that China will import 200 billion U S dollars worth of “Made in USA” products for two years

美中15日达成协议,中国将在两年内追加购买价值2000亿美元(约合232万亿韩元)的美国产品

대신 미국은 중국산 제품에 부과하던 15% 관세를 7. 5%로 줄이고, 작년 12월 부과 예정이었던 다른 제품들에 대한 추가 관세는 부과하지 않기로 했다 In return, the U S will lower the 15-percent tariffs currently imposed on Chinese products to 7. 5 percent and scrap its plan to impose additional tariffs on certain products from December 2019

相反,美国决定把对中国产品征收的15%关税下调至7.5%,不再对去年12月计划征收的其他产品征收追加关税

합의안에는 중국의 지식재산권 보호와 기술이전 강요 금지, 인위적인 위안화 절하 중단 등에 대한 약속도 담겼다 The agreement also contains China’s commitments to protecting intellectual property rights, prohibiting forceful requests for technology transfer, and no longer deval‎uing the yuan

协议中还包含了中国保护知识产权 禁止强迫技术转让 停止人为压低人民币等承诺

중국이 미국에서 추가 수입하기로 한 2000억 달러는 한국의 연간 총 수출액의 33%에 이르는 금액이다 An additional 200 billion dollars worth of goods and services to be imported by China from the U S are equivalent to 33 percent of South Korea’s total annual exports

中国决定从美国追加进口的2000亿美元商品,相当于韩国年总出口额的33%

농산물 뿐 아니라 공산품 에너지 서비스 등 다양한 제품들이 포함된다 China will import not only agricultural products but also industrial products, energy, and services

不仅包括农产品,还包括工业产品 能源 服务等多种产品

중국의 내수량이 크게 늘지 않는다면 그만큼 다른 나라에서 수입하던 양이 줄어들 것이다 Unless China’s domestic demand increases as much, its import from other countries is bound to shrink accordingly

如果中国的内需量没有大幅增加,那么从其他国家进口的量就会减少

국제통화기금(IMF)은 지난해 말 ‘미중 무역협정의 부작용’이란 보고서에서 미중 협상이 타결되면 한국 일본 유럽연합(EU) 등 다른 나라들이 타격을 입을 것이라는 분석을 내놨다 The International Monetary Fund (IMF) analyzed how the U S -China trade deal will negatively impact other countries, including South Korea, Japan, and the European Union, in its report titled, “Managed Trade: What Could be Possible Spillover Effects of a Potential Trade Agreement Between the U S and China

国际货币基金组织去年底在题为《美中贸易协定的副作用》的报告中分析说,如果美中谈判达成妥协,韩国 日本 欧盟等其他国家将遭受打击

중국의 수입액이 기존과 같을 때 한국은 최대 460억 달러(약 53조 원), 국내총생산(GDP)의 3% 가량 수출이 줄어들 것이라고 봤다 ” The report predicted that assuming China’s total imports to stay the same South Korea’s export can decrease up to 46 billion dollars or three percent of the country’s GDP

如果中国的进口额和以前一样,韩国最多将减少460亿美元(约合53万亿韩元)的出口,国内生产总值(GDP)将减少3%左右

한국은 미중 무역갈등의 중단에 안도할 틈도 없이 대중 수출에 직격탄을 맞을 가능성이 높아졌다 South Korea’s exports to China are likely to be dealt a direct blow without a brief moment of relaxation for the country from the ending of trade tensions between the two powerhouses

韩国没有时间因美中贸易矛盾的中断而暗松一口气,对华出口直接受到冲击的可能性加大

강대국들끼리의 ‘짬짜미 협정’으로 글로벌 무역체계가 인위적으로 재편됨에 따라 향후 경제에 미칠 영향의 불확실성도 커졌다 As the behind-the-door agreement is to artificially reshuffle the global trade system, its impact on the future economy remains very high

强国之间签订的“私下协定”人为地重组了全球贸易体系,对今后经济产生的影响不确定性也增大了

한국은 대중 수출이 전체의 27%나 차지한다 Given that China accounts for 27 percent of South Korea’s total exports,

韩国对华出口占总出口的27%

수출을 다변화해 대중 무역 의존도를 줄이고 미중 무역의 틈새시장을 찾는 일이 급해졌다 South Korea needs to diversify its export destinations to reduce dependency on trade with China and urgently find niche markets amid the large volume of U S -China trade

当务之急是实现出口多元化,减少对华贸易依存度,寻找中美贸易的缝隙市场

中, 美전역 사정권 SLBM 시험발사 China test-launches SLBM that can reach U S

中国试射把美全境纳入射程的潜射导弹,还与俄 伊举行联合军演

러-이란과 합동훈련도미국이 이란에 대해 전례 없이 강력한 제재를 지속하는 가운데 중국 러시아가 이란과 함께 인도양 북부와 오만해에서 사상 처음으로 해상 연합 군사훈련에 나선다 While the U S continues to apply unprecedentedly heavy sanctions against Iran, China, Russia, and Iran will launch maritime military drills for the first time in the northern Indian Ocean and the Sea of Oman

在美国继续对伊朗实施空前严厉的制裁的情况下,中国和俄罗斯将与伊朗一起在印度洋北部和阿曼海举行历史上首次海上联合军事演习

중국은 미국 전역을 타격할 수 있는 대륙간탄도미사일(ICBM)급 핵탄두 탑재 가능 잠수함발사탄도미사일(SLBM)도 시험 발사해 무력시위를 벌였다 In addition, China staged an armed protest by test-launching a submarine-launched ballistic missile (SLBM) that is capable of delivering an intercontinental ballistic missile-level nuclear warhead and reaching all over the U S

中国还试射了可以打击美国全境的洲际弹道导弹级核弹头的潜射弹道导弹,展示了肌肉

중국, 러시아가 손잡고 미국과 맞서는 모습이 지속되고 있어 주목된다 The continued cooperation of China and Russia against the U S is garnering attention

中国与俄罗斯携手与美国对抗的态势仍在持续,引起了人们关注

25일 AP통신과 이란 메르통신 등에 따르면 세 나라 해군이 참가하는 이번 훈련은 ‘해양 안보벨트’라는 이름으로 27∼30일 진행된다 According to The Associated Press and the Mehr News Agency of Iran on Wednesday, the joint military drills named the “Maritime Security Belt” wargame among the navy troops of the three countries will be carried out from Friday to next Monday

据美联社和伊朗梅尔通讯社25日报道,三国海军参加的这次演习名为“海洋安全带”,在27日至30日期间举行

이 지역은 걸프해의 입구이며 세계 최대 원유 수송 해역인 호르무즈 해협과도 가깝다 The location of the wargame is at the entrance of the Persian Gulf and close to the Strait of Hormuz, the world’s biggest crude oil transportation strait

该地区是海湾的入口,且靠近世界最大的原油运输海域霍尔木兹海峡

이란군은 “이번 훈련은 중동 지역의 국제 교역 안보 강화가 목적이다 “The objective of the wargame is to strengthen international trade security in the Middle East

伊朗军方说:“这次演习的目的是加强中东地区的国际贸易安全

이란, 러시아, 중국이 안보 경험을 교환하고, 테러와 해적 행위에 맞서는 게 가장 중요한 목표”라고 밝혔다 Iran, Russia, and China will share security experiences and train against terror attacks and pirates,” said an Iranian military source

伊朗 俄罗斯和中国交换安全经验 对抗恐怖主义和海盗行为,是最重要的目标”

하지만 이번 훈련은 미국의 중거리핵전력조약(INF) 탈퇴 및 경제 제재로 어려움을 겪는 이란을 지원하기 위한 것으로 중국과 러시아가 중동 지역에서 미국에 대항하는 움직임으로 보는 시각이 많다 However, many believe that the drills are for China and Russia to support Iran, which is struggling with the U S ’ withdrawal from the Intermediate-Range Nuclear Forces Treaty and economic sanctions, in opposition to the U S

但此次演习是为了支援因美国退出《中程核力量条约》及经济制裁而陷入困境的伊朗,很多人认为这是中国和俄罗斯在中东地区对抗美国的举动

미국은 이란에 대한 군사적 압박을 높이기 위해 우방국들과 걸프 해역에서 활동하는 해군 군사 연합체인 ‘호르무즈 호위연합’을 결성했고, 이에 대해 중국과 러시아는 ‘중동 지역의 안정을 위협한다’며 반발해왔다 （no translation）

为加大对伊朗的军事压力,美国与友好国家结成了在海湾海域活动的海军军事联盟“霍尔木兹护航联盟”,中国和俄罗斯对此表示,“威胁中东地区的稳定”

한국도 미국의 요청에 따라 호르무즈 해협에 파병할 가능성이 있다는 점에서 주목된다 （no translation）

值得关注的是,韩国也有可能根据美国的要求,向霍尔木兹海峡派兵

미국은 예멘 후티 반군이 자신들의 행위라고 밝힌 올해 9월 사우디아라비아 국영 석유기업 아람코의 석유 생산시설과 유전에 대한 무인기(드론) 및 미사일 공격에도 이란이 직접 개입했다며 이란에 대한 압박 강도를 높이고 있다 （no translation）

美国表示,伊朗直接介入了也门胡塞反政府武装宣称是自己所为的今年9月针对沙特阿拉伯国营石油企业阿美石油公司的石油生产设施和油田的无人机及导弹攻击,正在加大对伊朗的压力

미국은 아람코 피격 사태 후 사우디에 미군을 증파했고, 미사일방어(MD) 시스템도 추가 설치했다 （no translation）

美国在阿美石油公司遇袭事件发生后,向沙特增派了美军,并增设了导弹防御系统

이란은 이번 훈련을 중국, 러시아와의 군사 협력을 확대하는 계기로 활용할 의지를 내보였다 Iran expressed its intention to use the drills as an opportunity to expand military cooperation with China and Russia

伊朗表示,将利用此次演习扩大与中国 俄罗斯的军事合作

이란 파르스통신에 따르면 이란 해군의 호세인 한자디 소장은 “이번 훈련은 중국 러시아 해군과의 광범위한 협력의 한 부분이며, 여기에는 잠수함과 구축함 생산도 포함된다”고 말했다 “The wargame is part of extensive cooperation with the navy troops of China and Russia, which includes the production of submarines and destroyers,” said Hossein Khanzadi, the commander of the Iranian maritime branch, to the Fars News Agency

据伊朗法尔斯通讯社报道,伊朗海军少将何塞因·汉扎迪说,“这次演习是伊朗与中国 俄罗斯海军广泛合作的一部分,其中包括潜艇和驱逐舰的生产”

파키스탄처럼 반미 성향이 강한 나라가 향후 중국 러시아 이란의 합동 군사훈련에 참여할 수 있다는 전망도 나온다 Heavy anti-U S countries, such as Pakistan, are predicted to join the joint military wargame among China, Russia, and Iran in the future

也有预测称,像巴基斯坦这样反美倾向较强的国家今后有可能参加中国 俄罗斯 伊朗的联合军事演习

중국은 22일 보하이(渤海)에서 미국 전역을 사거리로 하는 ICBM급 SLBM인 ‘쥐랑(巨浪)-3’을 서쪽 방향으로 시험 발사했다 （no translation）

中国22日在渤海向西试射了射程为美国全境的洲际导弹级潜射导弹“巨浪-3”

중국 관영 관차저왕(觀察者網)은 쥐랑-3 발사 사실을 직접 언급하지 않으면서도 “베이징(北京)에서 (미사일 궤적인) 이상한 구름들이 목격됐다”며 “중국 정부가 20∼27일 보하이에서 군사 임무를 이유로 항행을 금지했다”고 보도했다 （no translation）

中国官方媒体“观察者网”没有直接提及发射“巨浪-3”的事实,但表示:“在北京看到了奇怪的云层(导弹轨迹)”,“中国政府以20日至27日在渤海实行军事任务为由发布了禁航令”

쥐랑-3은 중국의 094형 전략 핵추진 잠수함에서 발사된 것으로 알려졌다 （no translation）

据悉,“巨浪-3”是从中国的094型战略核动力潜艇上发射的

사거리가 1만 km에 달해 핵탄두를 탑재하고 미국 본토를 타격할 수 있는 것으로 평가된다 （no translation）

该导弹射程达1万公里,被认为可以搭载核弹头打击美国本土

美안팎 퍼지는 차이나 배싱… China-bashing spreads in and outside U S

扩散至美国到处的“敲打中国”,复杂的全球外交方程式

복잡한 글로벌 외교 방정식워싱턴의 한 중견 로펌에서 근무하는 P 변호사에겐 요즘 중국인 의뢰인이 부쩍 늘었다 A lawyer working at a mid-sized law firm in Washington is seeing a noticeable increase in Chinese clients recently

在华盛顿一家中坚律师事务所工作的P律师,最近中国委托人大幅增加

미국 내 중국 과학자들이 미국 정부로부터 감시를 당하고, 석연치 않은 이유로 활동을 제한당하고 있다는 것 Chinese scientists in the U S are under surveillance by the U S government and experiencing restrictions with unclear reasons

具体情况是,在美国的中国科学家受到美国政府的监视,并因不可捉摸的原因活动受到限制

법적 대응을 해주는 그는 “중국 과학자들한테 미국 정보요원들이 거의 일대일로 붙어서 밀착 감시하는 분위기”라며 “It seems like intelligence agents are assigned to Chinese scientists one-on-one for close surveillance,” said the lawyer said

他负责提供法律应对,就此表示:“美国情报人员几乎是一对一地对中国科学家进行密切监视”,“美中关系恶化,业务就越来越多,真好”

“미중 관계가 나빠지니 일은 많아져서 좋다”고 했다 “The bad relations between the U S and China are bringing more clients for me

“不会连民间领域的学者都如此严密地管理吧……”

‘설마 민간 분야 학자들까지 그렇게 치밀하게 관리할까…’ 궁금했지만 정색하는 P 변호사의 표정을 보니 틀린 말은 아닌 것 같았다 ” I was wondering if indeed researchers in the private sector are surveilled to such a degree but the look on the lawyer’s face seemed quite serious

记者虽然很好奇,但看到P律师表情严肃,看来这句话没有错

화웨이 사례에서 보듯 중국의 기술패권을 견제하려는 도널드 트럼프 행정부의 움직임은 이미 전방위로 확장하는 기류다 As seen in Huawei’s case, the Trump administration’s activities to check China’s technology hegemony are expanding in all directions

从华为的事例中可以看出,特朗普政府想要牵制中国的技术霸权,已经出现了全方位扩散的趋势

미국의 ‘중국 때리기(China bashing)’가 워싱턴 외교안보 분야의 분명한 흐름이라는 점도 부인할 수 없다 It is also undeniable that China-bashing is a clear direction in Washington’s foreign security

不可否认,美国的“敲打中国(Chinabashing)”是华盛顿外交安全领域的一个明显趋势

중국을 사실상 ‘적대국가’로 겨냥한 백악관과 국무부 고위 당국자들의 발언 강도는 점점 강해지고 있다 （no translation）

事实上,将中国视为“敌对国家”的白宫和国务院高层官员的发言强度正在逐渐加强

최근 홍콩 시위와 관련해 중국의 인권 상황을 문제 삼는 내용이 많아졌다 （no translation）

最近,关于香港示威,指责中国人权状况的内容越来越多

마이크 폼페이오 국무장관은 지난달 독일 베를린장벽 30주년을 기념해 진행한 연설에서조차 “중국 공산당이 국민을 억압하고 있다”며 중국을 비판했다 （no translation）

美国国务卿迈克·蓬佩奥上个月在德国柏林墙30周年纪念仪式上发表演讲时甚至批评中国说:“中国共产党在压迫国民”

옛 동독에 이어 중국까지 공산주의 문제점을 지적하는 방식이었지만 ‘1989년의 교훈: 자유와 우리의 미래’라는 제목의 연설 취지와는 연관성이 낮아 보였다 （no translation）

虽然他采取的是在继前东德之后连带指责中国存在的共产主义问题的方式,但看起来与题为《1989年的教训:自由和我们的未来》的演讲宗旨没有多大关联性

올해 70주년을 맞은 북대서양조약기구(NATO·나토)가 이달 초 정상회의 성명에서 중국을 겨냥한 것도 예사롭지 않다 It was also unusual for the North Atlantic Treaty Organization (NATO), which marked its 70th anniversary this year, to target China in its summit statement in early December

今年迎来70周年的北大西洋公约组织(北约)本月初在首脑会议声明中针对中国也不同寻常

중국의 아프리카 공략과 사이버 활동, 군사력 증강 등이 유럽에도 새로운 도전이 될 수 있다는 논리지만 지구 맞은편의 중국을 타깃으로 삼고 나서는 것은 다소 뜬금없다 It was based on the grounds that China’s attack on Africa, cyber activities, and growing military power pose new challenges to Europe, but it was rather random for the NATO to target the country on the opposite side of the Earth

中国攻略非洲 网络活动 增强军事能力等,虽然对于欧洲也可能成为新的挑战,但把地球对面的中国当作目标说事,多少有些莫名其妙

나토의 큰손 회원국인 미국의 그림자가 어른거린다 The U S , a NATO member with big influence, seems to be behind such targeting

浮现在人们眼前的是北约大腕成员国美国的身影

미국의 공격적인 대중 전략이 수출되는 느낌이랄까 as if the U S’ aggressive strategy against China is being exported

让人觉得美国出口了其攻击性的对华战略

중국 연구에 대한 강도를 높이는 싱크탱크도 많아졌다 In addition, more and more think tanks are reinforcing their research on China

加大对中国研究力度的智库也越来越多

전략국제문제연구소(CSIS)는 분야별로 중국을 연구하는 팀만 4개로 만들었다 The Center for Strategic and International Studies newly created four teams to study China by different fields

美国国际战略研究中心在不同领域专门成立了4个研究中国的小组

이달 들어서만 ‘중국의 부상과 글로벌 질서’ ‘중국의 인권 문제와 미국의 대응’ ‘중국의 정치적, 종교적 인권 문제’ 등 중국을 주제로 한 세미나가 잇따라 진행됐다 In December alone, there were a series of seminars on China, such as “China’s rise and global order,” “human rights issues in China and the U S ’ responses,” and “political, religious, and human rights issues in China

仅本月以来,就先后举行了“中国的崛起和全球秩序”“中国的人权问题和美国的应对”“中国的政治 宗教人权问题”等以中国为主题的研讨会

막상 현장에 가보면 중국인은 거의 없고 현지 학계 및 언론 인사와 홍콩, 대만, 한국 같은 외신기자들로 북적인다 ” Yet, it is hard to find Chinese participants at seminar venues Rather, they are filled with local researchers and media persons, as well as foreign reporters from Hong Kong, Taiwan, South Korea, etc

到现场一看,几乎没有中国人,倒是当地学界及媒体人士和香港 台湾 韩国等外国记者非常热闹

중국인은 견제 분위기가 워낙 강해서인지 얼굴을 내밀기도 어려운 눈치다 It seems like the Chinese find it uncomfortable to show up to such venues due to strong China-bashing sentiment

中国人似乎因为牵制氛围太强,很难露面

미국이 12일 중국과의 1단계 무역합의를 타결하면서 미중 사이 긴장감이 다소 누그러지는 듯하다 The tension between the U S and China seems to have been eased to a degree as the two reached a phase one trade agreement

随着美国12日与中国达成第一阶段贸易协议,美中之间的紧张气氛似乎有所缓和

그러나 지식재산권 등 훨씬 까다로운 내용을 다룰 2단계 협상이 남아 있다 However, phase two negotiations involving much trickier stuff, such as intellectual property rights, will be much tougher

但是,双方还有第二阶段谈判,涉及知识产权等更加棘手的内容

잠시 동결일 뿐 미중 관계가 좋아질 것이라고 기대하는 기류를 찾기 어렵다 The bilateral relation may be in truce now but there is no sign that it will improve going forward

这只是暂时的冻结,很难找到期待美中关系会好转的氛围

중국이 미국 외교안보 정책의 변수가 아닌 상수가 된 건 이미 오래전이다 It has been a long time since China has become a constant, rather than a variable, in the U S ’ foreign security policy

中国很久以前就不是美国外交安保政策的变数而是常数

이런 충돌 속에서 베이징의 반발은 더 거세지고 이로 인한 동북아 정세는 더 출렁이고 있다 Opposition from Beijing is growing amid such conflicts and the circumstances in Northeast Asia are becoming more unstable

在这种冲突中,北京的抗议更加强烈,由此引发的东北亚局势更加动荡

내년에는 ‘새로운 길’을 예고한 북한 문제까지 포함되면 미중 사이에서 풀어야 할 외교 함수도 더 복잡해진다 Considering the issue surrounding North Korea, which made threats about a “new path” from next year, diplomatic functions to be solved between the two powerhouses will become even more complicated

如果明年包括预告“新道路”的朝鲜问题在内,美中之间需要解决的外交函数也将变得更加复杂

이젠 중국 자체는 물론이고 미국의 대중 정책과 전략에 대한 더 많은 분석과 연구까지 필요한 시간이 다가오고 있다 More analysis and research, not only on China but also on the U S ’ policy and strategy towards China, will be needed in the near future

现在,不仅是中国本身,对美国对华政策和战略也要进行更多的分析和研究

中, 27년만에 최저성장 침체 그림자 짙어진다중국의 3분기(7∼9월) 경제성장률이 27년 만에 최저인 60%에 그쳤다 China’s economy marks lowest quarterly growth in 27 years China’s economy grew by 6 0 percent in the third quarter of this year, the lowest quarterly growth rate in 27 years

中国出现27年来最低增长率,停滞阴影渐浓中国第三季度(7月至9月)的经济增长率仅为6.0%,为27年来的最低值

한국 기업이 수출과 직접투자를 가장 많이 하는 중국 시장이 쪼그라들면서 한국 경제의 성장 발판이 흔들리고 있다는 분석이 나온다 Analysts say that the contracted Chinese market, the biggest destination of South Korea’s exports and direct investment, is likely to shake the foundation for South Korea’s economic growth

有分析认为,随着韩国企业出口和直接投资最多的中国市场萎缩,韩国经济的增长基础正在动摇

중국 국가통계국은 18일 “3분기 국내총생산(GDP)이 24조6865억 위안(약 4119조 원)으로 지난해 같은 기간보다 6.0% 증가했다”고 밝혔다 China’s National Bureau of Statistics said Friday that the country’s gross domestic product (GDP) for the July-September quarter recorded around 24. 687 trillion yuan (about 4,119 trillion won), a 6.0 increase from a year ago

中国国家统计局18日表示:“第三季度国内生产总值(GDP)为24.6865万亿元(约4119万亿韩元),比去年同期增长6.0%”

이 같은 성장률은 분기별 성장률 통계를 집계하기 시작한 1992년 이후 27년 만에 가장 낮은 것으로 당초 시장 전망치보다 0. 1%포인트 하락한 것이다 This marks the slowest quarterly growth since records began in 1992 and is a 0. 1 percentage point lower than analysts’ earlier expectations

这一增长率是自1992年开始统计季度增长率以来的最低值,比当初的市场预期下降了0.1个百分点

중국은 2015년 2분기 7. 0% 성장세를 나타낸 뒤 4년 동안 6%대 성장에 머물다가 6% 선이 무너질 상황에 몰렸다 Since logging 7. 0 percent in the second quarter of 2015, the growth of the world’s second largest economy has slowed down to around 6 percent for the following four years

中国自2015年第二季度出现7.0%的增长势头后,4年来一直停留在6%多,但现在接近了跌破6%

지난해 1분기부터는 분기 성장률이 매번 하락세를 보이고 있는 점을 감안하면 4분기 성장률은 5%대로 떨어질 가능성이 있다는 전망이 많다 Many speculate that the growth rate in the fourth quarter of 2019 will drop further to a range of 5 percent given this year’s growth trends

从去年第一季度开始,季度增长率每次都呈下降趋势,因此很多人预测第四季度增长率有可能下降到不到6%

3분기 중국 성장률이 시장 예상보다 낮아진 것은 미중 무역분쟁과 아프리카돼지열병(ASF)으로 수출과 내수 기반이 동시에 약해졌기 때문이다 The economic slowdown of the world’s most populous nation in the latest quarter is attributed to weakening demands for exports and domestic consumption due to its protracted trade war with the United States and the breakout of African swine fever (ASF)

第三季度中国经济增长率低于市场预期,原因是中美贸易纠纷和非洲猪瘟导致出口和内需基础同时减弱

미중 무역 갈등 여파로 중국의 9월 수출은 지난해 같은 달보다 3. 2% 줄었다 Affected by the trade spat with Washington, Beijing’s exports in September fell by 3. 2 percent from a year earlier

受中美贸易矛盾影响,中国9月份出口同比下降3.2%

제조업의 활력 정도를 보여주는 생산자물가지수(PPI)는 올 7∼9월 3개월 연속 감소해 디플레이션 우려까지 나오고 있다 In addition, the country’s producer price index (PPI) has declined for three consecutive months, sparking concern about a possible emergence of deflation

显示制造业活力的生产价格指数(PPI)在今年7月至9月连续3个月减少,甚至产生了通货紧缩的担心

여기에 ASF로 지난달 돼지고기 가격이 70% 올라 지난달 소비자물가가 3% 상승했다 The consumer price also rose by three percent last month, with the price of pork soaring by 70 percent as a result of ASF reports

再加上非洲猪瘟,上个月猪肉价格上涨了70%,上个月消费者物价上升了3%

그 여파로 소비가 감소하면서 성장률을 떨어뜨리는 효과가 나타났다 This further discouraged consumption, making a dent in the growth rate

受此影响,随着消费减少,产生了降低增长率的效果

영국 파이낸셜타임스(FT)는 “지방정부가 도로 다리 등 인프라 건설을 통해 성장률 목표를 달성해 왔지만 이런 사업도 말라가고 있다”고 했다 “The (Chinese) government has sought to use tax cuts and a flood of new infrastructure projects to power through the slowdown The data for the first nine months of the year show that some of those efforts are falling short,” the Financial Times said

英国《金融时报》报道说:“地方政府通过道路 桥梁等基础设施建设,实现了增长目标,但这样的事业也日渐枯竭”

중국 경제가 부진에 빠짐에 따라 제조업과 금융 부문에서 밀접하게 연관돼 있는 한국 경제가 타격을 받을 수 있다는 우려가 적지 않다 Analysts express concern that the South Korean economy would be dealt a blow from the slowing Chinese economy, as the two countries are closely linked to each other in terms of the manufacturing and finance industries

不少人担心,随着中国经济陷入低迷,在制造业和金融领域与中国密切相关的韩国经济将受到打击

한국경제연구원 등 민간 연구소들은 중국 성장률이 1%포인트 내려가면 한국 성장률은 0. 5%포인트 하락할 수 있다고 보고 있다 Private research institutes including the Korea Economic Research Institute analyzed that a 1 percent point decline in China’s growth would bring down the growth of South Korea by 0. 5 percentage point

韩国经济研究院等民间研究所认为,如果中国经济增长率下降1个百分点,韩国经济增长率将下降0.5个百分点

주원 현대경제연구원 경제연구실장은 “세계 경제 침체가 장기화되고 중국 정부의 각종 부양책도 먹히지 않으면서 성장 둔화가 내년까지 이어질 수 있고 한국 경제가 부정적 영향을 받을 수 있다”고 말했다 “If the slowdown of the global economy is protracted and the Chinese government’s stimulus policies fail to work, the Chinese economy may continue to shrink next year, negatively affecting the South Korean economy,” said Joo Won, deputy director of Economic Research Department at the Hyundai Research Institute

现代经济研究院经济研究室室长朱元(音译)表示:“随着世界经济长期低迷,中国政府的各种扶持政策也未能奏效,经济增长放缓现象可能会持续到明年,韩国经济可能会受到负面影响”

홍콩시위대-중국군 첫 대치 China's military, Hong Kong protesters confront Sunday night

香港示威者首次与中国军队对峙,刚刚接近部队就被警告“要为后果负责任”

부대 접근하자 “후폭풍 책임져야”홍콩 반중 시위대와 홍콩 주둔 중국 인민해방군이 6일 밤 잠시 대치했다 The anti-China protesters of Hong Kong had a brief confrontation with the People’s Liberation Army (PLA) troops in Hong Kong on Sunday night

香港反中示威者和中国人民解放军驻港部队6日晚间发生短暂对峙

6월부터 넉 달째 이어지고 있는 이번 시위에서 양측의 첫 직접 대치여서 긴장감이 고조됐다 Tensions were escalated as it was the first direct interaction between the PLA and protesters

在始于6月至今已延续四个多月的这次示威中,这是双方首次直接对峙,令紧张感达到了高潮

이날 시위대 수백 명은 카오룽 지역의 인민해방군 부대 근처에서 레이저 불빛을 부대 막사 건물에 비췄다 On Sunday, hundreds of protesters shone laser lights on the barracks of the PLA in Kowloon

当天,数百名示威者前往九龙地区的人民解放军部队营区附近,用激光灯光照射部队建筑物

중국군은 즉각 막사 옥상에서 노란 깃발을 들어 시위대에 경고 신호를 보냈다 The PLA instantly warned protesters by raising a yellow flag on the roof

中国军队立即在房屋顶上举起黄色旗帜,向示威者发出警告信号

깃발에는 중국 본토에서 사용하는 푸퉁화(普通話)와 영어로 “당신은 법을 어기고 있다 On the flag was written a phrase both in Putonghua and English, saying “You’re violating law

旗帜上用中国本土使用的普通話和英语写着:“你们正在违反法律

기소될 수 있다”는 문구가 적혔다 You may be prosecuted

可能会被起诉”

중국군은 홍콩에서 쓰는 광둥(廣東)어로 “이후 발생하는 후과는 모두 자신이 책임져야 한다”는 육성 경고도 했다 ” Later on, the PLA issued a voiced warning in Cantonese that the protesters “must take full responsibility for the consequences of their actions

中国军队还用在香港使用的广东话发出语音警告:“以后发生的后果,你们自己要负责任”

중국군은 이 과정에서 카메라로 시위대를 촬영하며 이들의 동태를 면밀히 감시했다 ” The troops monitored protesters with cameras and watched their movements closely

这一过程中,中国军队使用摄像机拍摄示威者,密切监视他们的动态

다만 시위대가 곧 부대 주변을 떠나면서 더 이상의 충돌은 일어나지 않았다 As protesters left the barracks soon, there was no direct conflict between the two sides

但是,随着示威者不久即离开部队周边,再没有发生冲突

사우스차이나모닝포스트(SCMP)는 “중국군이 유례없는 움직임으로 경고했다”고 전했다 The South China Morning Post reported that the Chinese military “issued unprecedented warnings” to Hong Kong protesters

《南华早报》报道称,“中国军队对前所未有的行动发出了警告”

중국군이 시위대에 발포하거나 유혈 진압에 나서면 시위 사태가 걷잡을 수 없는 방향으로 흘러갈 수도 있다는 우려가 나온다 Some express concern that a shooting or a violent suppression by the PLA could nudge the situation into an entirely unpredictable direction

有人担心,如果中国军队向示威者射击或流血镇压,示威事态有可能朝着无法控制的方向发展

친중파 세력들은 시위대의 이번 행동이 서방의 개입을 이끌어내려는 고의적 도발이 아니냐고 주장하고 있다 （no translation）

亲中派势力主张,示威者的此次行动可能是为了拉西方介入而实施的故意挑衅

5일부터 시작된 복면금지법 시행에 따른 대립도 점점 격화되고 있다 The conflict over the ban on face masks, which started on Saturday, has been intensifying as well

随着5日开始施行《禁止蒙面规例》,对立也越来越激化

홍콩 경찰은 7일 복면금지법 위반 혐의로 18세 대학생 및 38세 여성을 처음으로 기소했다 On Monday, the Hong Kong police brought the first charges under the anti-mask law to an 18-year-old college student and a 38-year-old woman

香港警方7日以涉嫌违反禁止蒙面法,首次起诉一名18岁的大学生和一名38岁的女子

이들은 5일 새벽 마스크를 쓰고 시위를 벌이다 체포됐다 The two were arrested on early Saturday morning while protesting with masks on

她们5日凌晨戴着口罩展开示威,随后被捕

교육 당국은 중·고등학교 교장들에게 “8일부터 마스크를 쓰고 등교하는 학생, 수업을 거부하는 학생, 인간 띠 시위를 벌이거나 구호를 외치는 학생들의 명단을 제출하라”고 지시했다 Starting from Tuesday, Hong Kong’s education authorities ordered the heads of middle and high schools across the city to submit the list of students who “wear a mask at school, refuse to attend classes, form a human chain or chant for protest

教育当局向初中和高中的校长们发出指示:“从8日起,要提交戴口罩上学的学生 拒绝上课的学生 展开手拉手示威或喊口号的学生的名单”

경찰은 6일 대학의 허락 없이 홍콩중문대와 침례대 안으로 진입해 시위대를 체포했고, 마스크를 착용한 시민들을 무차별적으로 체포하고 있는 것으로 알려졌다 ” On Sunday, the Hong Kong police broke into the premises of the Chinese University of Hong Kong and Hong Kong Baptist University to arrest protesters, and they are reportedly making indiscriminate arrests of random citizens wearing a mask

据称,警方6日在未经大学许可的情况下,进入香港中文大学和浸礼大学校内,将示威者和戴口罩市民全部逮捕

6일 삼서이보 지역에서는 60대 운전기사가 모는 택시 1대가 시위대를 향해 돌진해 2명이 차에 깔려 중상을 입었다 （no translation）

6日,在深水埗地区,一名60多岁的司机驾驶一辆出租车冲向示威人群,导致两人被车子轧倒,身受重伤

이후 시위대들이 기사를 끌어내 구타했고, 기사가 얼굴에 피를 흘리며 의식을 잃은 모습도 목격됐다 （no translation）

之后,人们看到,示威者们把司机拉出车外进行殴打,司机脸上流着血,失去意识

한 방송사 기자는 시위대가 던진 화염병을 맞아 얼굴에 화상을 입기도 했다 （no translation）

一名电视台记者被示威者投掷的火焰瓶砸中,脸部被烧伤

홍콩 지하철은 7일 오전에도 전체 지하철역(94곳) 중 39곳만 운행했다 The Hong Kong metro operated only 39 lines out of a total 94 on Monday morning

7日上午,香港地铁只运营了所有地铁站(94处)中的39处

이날 오후 6시부터는 내부 수리를 이유로 전체 노선을 폐쇄해 유령 도시를 방불케 했다 Starting from 6 p m , the entire lines were shut down for maintenance, turning the territory into a ghost city

当天下午6点起以内部修理为由关闭全部路线,令城市犹如一座幽灵城市

대형 쇼핑몰은 문을 닫았고 주요 마트들도 영업시간을 단축했다 Large shopping malls were closed, and major supermarkets reduced working hours

大型购物商场纷纷关门,主要超市也缩短了营业时间

마트에 생필품을 사려는 사람들이 장사진을 이뤄 물건이 동나는 광경도 목격됐다 Against this backdrop, many grocery stores were packed with people looking for food and daily necessities and saw their shelves emptying instantly

去超市购买生活必需品的人群排起长队,还出现了东西被抢购一空的情景

일부 시민들은 “전쟁 같은 분위기”라고 전했다 Some citizens described the scene as “wartime atmosphere”

部分市民认为“如同战争般的氛围”

한편 미국프로농구(NBA)의 유명 구단 휴스턴 로키츠의 대릴 모레이 단장은 시위대를 지지했다가 로키츠를 후원하던 중국 기업들이 스폰서 중단을 선언해 곤욕을 치렀다 （no translation）

此外,美国职业篮球(NBA)的著名球队休斯顿火箭队总经理达雷尔·莫雷因为支持示威者,导致赞助火箭队的中国企业纷纷中断赞助,遭受了屈辱

그는 6일 트위터에 “복잡한 사건에 대해 한쪽 편만 들었다”고 썼다 （no translation）

他在6日在推特上写道:“对于复杂的事件,仅支持了一方”

애국주의 들끓는 건국 70년… 中내부에서도 책임 목소리중국 건국 70주년 기념 열병식이 오전 10시(현지 시간)부터 베이징 중심 톈안먼(天安門)일대에서 시작된 1일 Patriotism prevails in China on 70th anniversary of foundation A military parade marking the 70th anniversary of China’s foundation started at 10 a m on last Tuesday at Tiananmen Square in central Beijing

爱国主义沸腾的建国70年,中国内部也有责任的声音10月1日,新中国成立70周年纪念阅兵式从上午10时(当地时间)开始在北京中心天安门一带举行

기자를 비롯한 외신 기자들은 열병식 시작 5시간 반 전인 오전 4시 반경 1차 보안검사를 거친 뒤 베이징 서부 미디어센터에 모였다 Foreign journalists including this reporter gathered at the Media Center in western Beijing after undergoing the first security inspection at around 4:30 a m , or about five and a half hours before the parade began

包括记者在内的外国记者在阅兵式开始前5个半小时,即上午4点半左右经过第一次安检后,聚集在北京西部的媒体中心

컴컴한 새벽, 교통통제로 텅 빈 도로를 달려 오전 6시경 도착한 톈안먼광장 인근 첸먼(前門) The reporters arrived at Chenmen near Tiananmen Square around 6 a m after traveling through a road that was completely empty due to traffic restrictions in the dark

漆黑的清晨,记者驱车行驶在交通管制下空荡荡的马路上,早上6时左右到达天安门广场附近的前门

열병식 참관 장소인 톈안먼광장 맨 앞으로 향하기 위해 2차 보안검사를 통과해야 했다 Passing a second security check was mandatory before moving to the forefront of the Square, the site of the military parade

为了前往观看阅兵式场所天安门广场的最前方,必须通过第二次安检

런민(人民)일보 신화통신 등 중국 관영매체 기자들은 전날인 지난달 30일 오후 11시 미디어센터에 집합했다 Reporters from China’s state-run media outlets including the People’s Daily and Xinhua News Agency gathered at the Media Center at 11 p m on the previous day

《人民日报》 新华社等中国官方媒体的记者于前一天的30日晚11时在媒体中心集合

이들이 톈안먼 일대에 도착한 시간은 1일 오전 1시 50분경이었다 Those reporters arrived at the Tiananmen Square area around 1:30 a m on Tuesday

他们到达天安门一带的时间是1日凌晨1点50分左右

열병식 참가 병사들은 이미 톈안먼을 지나는 창안(長安)대로에 집결했다 Soldiers who participated in the parade had already gathered at Changan Road, which is linked to Tiananmen Square

参加阅兵式的士兵们已经集结在长安大街上

하룻밤을 꼬박 거리에서 지냈지만 표정은 매우 밝았다 They spent awake all night on the street but looked excited

他们在街上呆了整整一夜,但表情很明朗

열병식 시작 전 현장에서 만난 중국인들도 벅찬 감정을 감추지 못했다 Chinese citizens this reporter encountered onsite before the parade’s opening also looked exuberant

阅兵式开始前在现场见到的中国人也掩饰不住激动的心情

3만여 관중은 각 지역에서 선발돼 열병식 참관 기회를 얻어 새벽부터 현장에 도착했다 More than 30,000 spectators, who had been selected from different regions across China and given a chance to witness the historic parade in person, already arrived at the site early in the morning

3万多名观众从各地选拔,获得了参观阅兵的机会,从凌晨就到达了现场

톈안먼 광장에서 “강한 조국의 군대”를 직접 보는 것은 중국인들의 꿈이었다 It was the Chinese people’s dream to watch in person a “strong military of their fatherland” at Tiananmen Square

在天安门广场亲眼看到“强大的祖国军队”是中国人的梦想

“어떤 세력도 우리 위대한 조국의 지위를 흔들 수 없다”고 선포한 시진핑(習近平) 중국 국가주석의 연설에 중국인들은 환호했다 Patriotism, in which “unity of the people” is considered to be of utmost importance, is thriving across China

“任何势力也无法动摇我们伟大祖国的地位”

시 주석이 “70년 전 중국의 건국으로부터 근대 이후 중국의 길고 긴 가난함과 약함, 괴롭힘을 당한 비참한 운명을 철저히 바꿨다”고 말한 것이 의미심장했다 （no translation）

中国国家主席习近平的宣布,引起了中国人在欢呼。习主席说:“70年前,新中国成立以后,彻底改变了近代以来中国漫长的积贫积弱 惨遭欺负的悲惨命运”此话意味深长

‘강해진 조국’에 대한 중국인들의 환호는 이제 미국은 물론이고 세계 어떤 나라도 중국을 건드릴 수 없다는 자부심으로 보였다 （no translation）

中国人民对“强大起来的祖国”的欢呼,展示了现在不仅是美国,世界上任何一个国家都无法撼动中国的自豪

그 자부심은 때로 외부 세계에 대한 호전적인 언어로 드러난다 （no translation）

这种自豪感有时通过对外部好战的语言表现出来

지금 중국 전역은 ‘인민의 단결’이 가장 중요하다는 애국주의로 들끓고 있다 （no translation）

现在,全中国上下都在为“人民的团结”最为重要的爱国主义而沸腾

국경절 연휴(1∼7일)에 개봉한 애국주의 영화 ‘나와 내 조국’ 등의 흥행 바람이 몰아치고 있다 “Me and My Fatherland,” a patriotic movie that was released at cinemas during the National Day holiday (October 1 – 7) across China, has become a box office hit

国庆节长假(1日至7日)上映的爱国主义电影《我和我的祖国》等票房火爆

‘중화민족의 위대한 부흥’이라는 민족주의가 애국주의를 떠받친다 Nationalism dubbed “great reconstruction of the Chinese people” is buttressing patriotism in the movie

“中华民族伟大复兴”的民族主义支撑着爱国主义

애국주의 자체는 탓할 일이 아니다 Patriotism per se should not be criticized

爱国主义本身不应该责怪

하지만 ‘강대국이 져야 할 무거운 책임’도 함께 깨달아 가고 있는지 중국 내부에서도 우려가 나온다 However, critics even within China have raised concerns that China is concurrently realizing the heavy responsibility weighing on the country as a superpower

但是,中国内部也有人担心,中国是否意识到了“强国应承担的沉重责任”

베이징대 국제관계학원 부원장을 지낸 왕이저우(王逸舟) 베이징대 교수는 인터뷰에서 “민족주의를 잘못 다루면 쇼비니즘(맹목적 애국주의)으로 변할 수 있다”고 경고했다 “If nationalism is mistreated, it can transform into chauvinism Notably,

曾任北京大学国际关系学院副院长的北京大学教授王逸舟在接受采访时警告说:“如果错误处理民族主义,可能会变成一种盲目的爱国主义

“특히 한 국가가 괴롭힘을 당한 역사가 있거나 주권 분쟁이 있으면, 민족주의는 상상하지 못한 (방식으로) 남을 해치는 결과를 낳을 수 있다”고 지적했다 if a country has history of being invaded and colonized or is in dispute over sovereignty, nationalism could end up doing harm on others (in an unimaginable way),” Professor Wang Yizhou at Beijing University said in an interview

他指出:“特别是一个国家,如果曾经遭受过欺负,或者存在主权争端,民族主义可能会产生意想不到地伤害他人的后果”

“좋은 외교는 반드시 좋은 내치, 좋은 사회의 기초가 있어야 한다”고 일갈한 “Good diplomacy should always be built on the basis of good public administration and good society”

他一语道破,“好外交必须有好内治 好的社会基础”

그가 주장하는 ‘인(仁)의 사회’로 가는 조건에는 “인민들이 더 개방적이고 호전적인 정서가 없는 사회” 등이 포함되어 있다 He promoted “society of virtue,” whose conditions he says include a society where people are more open and devoid of a sense of belligerence

他主张,走向“仁社会”的条件包括“人民更开放,没有好战情绪的社会”等

그는 다른 국가들이 중국의 굴기(굴起)에 왜 우려하고 위협을 느끼는지 중국이 제대로 인식하지 못하는 것을 걱정했다 （no translation）

他担心的是,其他国家为什么对中国崛起感到担忧和威胁,而中国却认识不到这一点

중국의 굴기가 갈림길에 놓였다고 한 그는 “높은 산 정상으로 갈수록 풍경이 아름답지만 고산 증세가 심해진다”고 했다 （no translation）

他说,中国的崛起已经走到了十字路口,“越是高山之巅,风景越美,但高山症状越严重”

지금은 과거 중국에는 없었던 문제, 즉 세계 속에서 더 큰 책임과 의무라는 문제를 마주하고 있다는 것인데, 애국주의만 지나치게 강조하다 눈을 가리는 게 아닌지 다시 돌아보게 하는 대목이다 （no translation）

现在中国遇到了过去没有的问题,即世界中更大的责任和义务问题,需要回头看看是不是因为过分强调爱国主义而遮住了眼睛

시진핑 “어떤 힘도 중국 흔들지 못해”‘No force can shake China,’ says Xi Jinping

习近平:“任何力量也无法撼动中国”

중국이 1일 건국 70주년을 맞아 베이징 톈안먼(天安門)광장에서 무기 전시장을 방불케 하는 대규모 열병식을 열어 국력을 과시했다 China showed off its national power on Tuesday by hosting a large-scale military parade that reminded of a weapon exhibition at Tiananmen Square in Beijing to celebrate the 70th anniversary of the People’s Republic of China

中国1日迎来建国70周年,在北京天安门广场举行了仿佛武器展示场的大规模阅兵式,展示了国力

시진핑(習近平) 국가주석은 미국을 겨냥해 “어떤 힘도 중국을 흔들어 놓을 수 없다”고 강조했다 “No force can shake China,” emphasized Chinese President Xi Jinping targeting the U S

国家主席习近平针对美国强调,“任何力量都撼动不了中国”

홍콩에는 일국양제를, 대만에는 평화통일을 언급하며 애국, 단결, 민족주의를 거듭 외쳤다 He enthusiastically spoke about patriotism, unity, and nationalism by mentioning “one country, two systems” for Hong Kong and peaceful unification for Taiwan

他一再呼吁香港要“一国两制” 台湾要“和平统一”以及“爱国 团结 民族主义”

이날 열병식은 건국 70주년을 자축하는 70번의 예포 발사와 오성홍기 게양식으로 시작했다 The military parade began with a salute of 70 guns and the Chinese red flag with five stars to celebrate the 70th anniversary

当天的阅兵式以庆祝建国70周年的70次礼炮发射和五星红旗升旗仪式开始

인민복 차림의 시 주석은 양 옆에 장쩌민(江澤民), 후진타오(胡錦濤) 전 주석을 대동한 채 “지난 70년 동안 중국은 눈부신 성장을 이뤄냈다 어떠한 힘도 우리의 지위를 흔들 수 없다 중국 인민과 중화민족의 전진을 막을 어떠한 세력도 있을 수 없다”고 말했다 “China has made remarkable growth for the past 70 years There is no force that can shake the status of this great nation No force can stop the Chinese people and the Chinese nation forging ahead,” said President Xi in a Maoist suit who stood between former Presidents Jiang Zemin and Hu Jintao

身着中山装的习主席率领在两旁的江泽民 胡锦涛两名前任国家主席出现,并表示:“过去的70年里,中国取得了辉煌的成果。任何力量都不能动摇我们的地位。任何势力都不能阻止中国人民和中华民族的前进”

미중 무역전쟁, 홍콩 반중 시위, 경제 둔화 등 내우외환에도 불구하고 미국의 압박에 굴하지 않겠다는 뜻으로 풀이된다 His speech is interpreted as China’s determination to stand tall against the U S ’ pressure despite challenges in and outside the country, including U S

这可以解释为,尽管存在美中贸易战 香港反华示威 经济增速放缓等内忧外患,中国不会屈服于美国的压力

그는 홍콩과 대만을 겨냥해 “평화통일, 일국양제의 원칙을 준수하며 홍콩과 마카오의 장기적 번영과 안정을 유지해야 한다”고 했다 -China trade war, the anti-China demonstration in Hong Kong, and economic slowdown “Forging ahead, we must remain committed to the strategy of peaceful reunification, and ’One Country, Two Systems’ to maintain long-term prosperity and stability of Hong Kong and Macao,” the Chinese president also said

他针对香港和台湾说,“要恪守和平统一 一国两制的原则,保持香港和澳门的长期繁荣稳定”

또 “중국의 내일은 훨씬 나아질 것”이라며 중화민족의 위대한 부흥을 뜻하는 ‘중국몽’ 실현을 위해 단합하자고 주문했다 He also called for unity to realize the Chinese Dream, which refers to the great prosperity of the Chinese nation, saying, “China’s tomorrow will be even more prosperous

他还表示,“中国的明天会更加美好”,希望大家团结起来,实现中华民族伟大复兴的“中国梦”

연설 말미에는 “중화인민공화국이여, 위대한 중국 공산당이여, 위대한 중국인들이여 영원하라”고 외쳤다 ” He ended his speech shouting, “Long live the great People’s Republic of China Long live the great Communist Party of China Long live the great Chinese people

在演讲的最后,他大声疾呼:“中华人民共和国万岁,伟大的中国共产党万岁,伟大的中国人民万岁”

시 주석은 톈안먼광장 앞 창안제(長安街)에서 미리 도열해 있던 59개 제대, 군사 1만5000명의 사열을 받았다 ” President Xi inspected 15,000 troops of 59 military units who lined up in Changan Avenue in front of Tiananmen Square

习主席检阅了事先在天安门广场前面长安街上列队的59个方队 15000名军人

최첨단 무기 전시장을 방불케 한 열병식은 그 자체로 미국을 향한 메시지였다 The military parade resembling an exhibition of cutting-edge weaponry was a message to the U S in itself

如同最尖端武器展示场的阅兵式本身就是向美国传达的信息

가장 이목을 끈 무기는 미국 수도 워싱턴을 타격할 수 있는 차세대 대륙간탄도미사일(ICBM) ‘둥펑-41’ The most eye-catching weapon was the Dongfeng-41, a next-generation intercontinental ballistic missile that can attack Washington D C

最引人注目的武器是能够打击美国首都华盛顿的新一代洲际导弹“东风-41”

이날 최초 공개된 이 미사일은 사거리가 1만4000km여서 전 세계가 사정권이다 The missile, which was revealed for the first time on Tuesday, has the maximum range of 14,000 kilometers, making it capable of reaching anywhere in the world

当天首次公开的该导弹射程为1.4万公里,全世界都在射程之内

최고 10개의 핵탄두를 장착할 수 있고 공격 목표의 오차 범위도 100m에 불과한 것으로 알려졌다 It can carry up to 10 nuclear warheads and the margin of error for a target is less than 100 meters

据悉,该导弹最高可携带10枚核弹头,攻击目标误差范围也只有100米

극초음속 활강 기술을 사용해 미국의 미사일방어체계(MD)를 뚫을 수 있는 것으로 알려진 ‘둥펑-17’, In addition, Beijing also revealed the Dongfeng-17, which is known to avoid the missile defense system of the U S

此外,中国还展示了使用极超音速滑降技术 可以突破美国导弹防御系统的“东风-17”,

항공모함 킬러로 불리는 극초음속 미사일 ‘둥펑-100’ 등도 선보였다 using the hypersonic descent technology, and the Dongfeng-100, a hypersonic missile known to be the killer of aircraft carriers

以及被称为“航母杀手”的极超音速导弹“东风-100”等

미국 F-35에 맞먹는 신형 스텔스 전투기 ‘J-20’, 미 군용헬기 블랙호크에 필적하는 ‘Z-20’도 가세했다 The J-20, a new stealth fighter comparable to the U S ’ F-35, and the Z-20, an equal for the U S ’ military helicopter Black Hawk, were also featured

与美国F-35相当的新型隐形战斗机“J-20”与美国军用直升机“黑鹰”相匹敌的“Z-20”也加入其中

중국 언론은 이날 열병식에 동원된 무기 중 40%가 최초로 공개됐다고 전했다 Chinese media reported that 40 percent of the weapon mobilized for the military parade was revealed for the first time

中国媒体报道说,当天参加阅兵式的武器中,40%是首次公开的

관영 글로벌타임스는 “어떤 위협에도 대응할 전략 핵무기를 갖고 있다는 뜻을 알렸다”고 했다 Chinese state-run media Global Times said it was “a message to the world that China has sufficient and reliable strategic nuclear power to respond to any ‘nuclear blackmail’ from any country

官方的《环球时报》报道说:“已告知中国拥有战略核武器,可以应对任何威胁”

미국뿐 아니라 한국, 일본 등 주변국에 대한 위협 효과도 있다는 분석도 나온다 ” Some say that China’s imposing demonstration targeted not only the U S but also its neighboring countries, including South Korea and Japan

有分析认为,这不仅对美国,对韩国 日本等周边国家也有威胁效果

이날 건국 70주년을 맞아 각국 지도자들도 축전을 보냈다 （no translation）

当天,为迎接建国70周年,各国领导人也纷纷发来贺电

중국은 블라디미르 푸틴 러시아 대통령, 도널드 트럼프 미국 대통령, 김정은 북한 국무위원장의 순서로 축전을 소개했다 （no translation）

中国按照俄罗斯总统普京 美国总统特朗普 朝鲜国务委员会委员长金正恩的顺序介绍了贺电

김 위원장은 축전에서 “(북한은) 나라의 안정과 핵심이익을 수호하고 지속적인 발전을 이룩하기 위한 중국의 투쟁을 전적으로 지지한다”며 “사회주의를 고수하고 빛내기 위한 한길에서 언제나 (중국과) 함께 있을 것”이라고 밝혔다고 조선중앙통신이 1일 보도했다 （no translation）

据朝鲜中央通讯社1日报道,金正恩在贺电中表示:“(朝鲜)完全支持中国为维护国家稳定和核心利益 实现持续发展而进行的斗争”,“在坚持社会主义 为国争光的道路上,朝鲜将永远与中国站在一起”

이어 ‘북-중 간 여러 차례의 상봉(정상회담)에서 이룩된 중요한 합의정신’을 언급하며 “새 시대의 요구와 두 나라 인민의 공동의 염원에 맞게 (양국 관계가) 날로 활력 있게 발전할 것이라고 굳게 믿는다”고 강조했다 （no translation）

金正恩还在贺电中谈到“中朝之间多次(首脑)会晤上取得的重要协议精神”,强调“深信(两国关系)将符合新时代的要求和两国人民共同的愿望,日益获得发展”

中, 위안화 환율 또 올려… 美는 中가구에 징벌관세 China sets its yuan midpoint weaker Friday

中国再次下调人民币汇率,美国对中国输美家具征收惩罚性关税

중국 중앙은행 런민(人民)은행이 9일 위안화 고시 환율을 또 올렸다 China’s central bank raised the official midpoint reference for the yuan yet again on Friday,

中国央行——中国人民银行9日再次下调人民币市场汇价

위안화 고시 환율은 전날 2008년 5월 이후 11년 만에 ‘포치(破七·달러당 7위안이 넘는 것)’를 돌파했고 이날 추가 상승했다 which broke a psychologically important level of 7 per dollar Thursday for the first time in 11 years since May 2008

人民币汇率自2008年5月后时隔11年首次突破了“1美元兑7元人民币”大关(破七),当天又进一步下跌

런민은행은 이날 오전 달러 대비 위안화 중간 환율을 7. 0136위안으로 고시했다 The People’s Bank of China set the yuan’s daily midpoint at 7. 0136 per dollar,

今天上午,中国人民银行公布人民币对美元汇率中间价为7.0136:1

전일 고시 환율은 7. 0039위안보다 0. 14% 상승(위안화 가치 하락)했다 a 0. 14 percent increase from Thursday’s rate of 7. 0039 yuan per dollar

前一交易日,人民币对美元汇率中间价为7.0039,上升了0.14%(人民币贬值)

위안화 고시 환율은 지난달 31일 이후 7거래일 연속 상승했다 The yuan’s rate against the greenback has risen for seven consecutive trading days since last Wednesday

自上个月31日以来,人民币对美元汇率已连续7个交易日走低

중국이 미국의 거센 반발에도 위안화 약세를 관세 및 환율전쟁 ‘무기’로 사용한다는 지적이 나오면서 미국도 중국에 대한 ‘맞불’ 제재에 나섰다 With China apparently weakening its local currency in response to trade pressure, the United States has also revealed another set of sanctions

有人指出,中国不顾美国的强烈反对,将人民币弱势作为关税及汇率战的“武器”,美国也对中国采取了“对攻”制裁

미 상무부는 8일(현지 시간) 중국산 목제 가구에 고율의 상계관세를 물리기로 결정했다고 AP통신 등이 전했다 The U S Department of Commerce announced Thursday (local time) the affirmative preliminary determination of the countervailing duty (CVD) investigation of imports of wooden cabinets and vanities from China, The Associated Press reported

据美联社报道,美国商务部8日(当地时间)决定对中国产木制家具征收高额补偿性关税

상계관세는 교역 상대국 정부가 수출 경쟁력을 높이기 위해 보조금을 지급하는 상품에 적용하는 징벌적 세금이다 Countervailing duty laws protect businesses and workers from the unfair pricing of subsidized imports

补偿性关税是贸易对象国政府为提高出口竞争力而对提供补助金的商品适用的惩罚性税金

중국이 지난해 미국에 수출한 나무 찬장과 화장대의 규모는 44억117만 달러(약 5조3000억 원)에 달한다 The United States imported wooden cabinets and vanities from China worth around 4. 41 billion U S dollars (about 5. 3 trillion won) last year

中国去年向美国出口的木橱和梳妆台规模达44.0117亿美元(约合53000亿韩元)

상무부는 이날 중국산 찬장과 화장대에 대한 상계관세 조사 결과, 중국 기업이 최저 10. 97%에서 최고 229. 24%의 국가 보조금을 받은 것으로 판정했다고 밝혔다 The Department of Commerce also said that according to its investigation, Chinese exporters received countervailable subsidies ranging from 10. 97 to 229. 24 percent

美国商务部当天宣布,对中国产橱柜和梳妆台的补偿性关税调查结果显示,中国企业获得的国家补贴最低为10.97%,最高为229.24%

상무부는 해당 기업으로부터 판정된 불공정 보조금 지급액만큼 현금을 징수하겠다고도 덧붙였다 and that it will collect cash deposits from importers of wooden cabinets and vanities from China

商务部还表示,将按相关企业判定的不公平补贴支付额征收相应的税金

이 조치는 내년 1월 30일 미 무역위원회(ITC)의 최종 판정을 거쳐 집행된다 The U S International Trade Commission (ITC) is scheduled to make its final CVD determination on Jan 30, 2020

该措施将在明年1月30日经过美国贸易委员会的最终裁决后执行

미국은 이날 중국 최대 통신장비업체 화웨이에 대한 수출규제 완화도 보류했다고 블룸버그 등이 전했다 Washington has also held off “on a decision about licenses for U S companies to restart business with Huawei Technologies Co ,” according to a report from Bloomberg

彭博社等报道称,美国当天还保留了放宽对中国最大通信设备企业华为的出口限制

윌버 로스 미국 상무장관은 지난달 30일 “화웨이와 거래하게 해 달라”는 미 기업의 요청에 “다음 주까지 응답할 수 있다”고 긍정적 반응을 보였다 U S Commerce Secretary Wilbur Ross had said last Tuesday that a decision on American businesses’ requests to resume sales could be made by next week

美国商务部长威尔伯·罗斯7月30日对美国企业提出的“请允许与华为进行交易”的要求称,“下周之内可以作出回应”,作出了正面的反应

하지만 양국의 대립이 격화되자 이를 무기한 연기한 것으로 풀이된다(（no translation））

但分析认为,随着两国对立的激化,这一措施已被无限期推迟.

상무부는 5월 이란과의 거래 정황을 이유로 화웨이와 계열사를 거래제한 블랙리스트에 올렸다 이후 화웨이와 거래를 원하는 미 기업은 반드시 정부의 사전 승인을 받아야 한다(（no translation））

5月,商务部以与伊朗的交易情况为由,将华为及其子公司列入限制交易的黑名单。此后,希望与华为进行交易的美国企业必须事先得到政府的批准

한편 금융시장에서는 ‘위안화 인상’의 다음 마지노선을 달러당 7. 2∼7. 3위안으로 전망한다고 9일 니혼게이자이신문이 보도했다(（no translation））

另据《日本经济新闻》9日报道,金融市场人士预计,“人民币下跌”的下一个底线为1美元兑7.2至7.3元人民币

다음 달 1일부터 3000억 달러 규모의 중국산 제품에 대해 10%의 추가 관세를 부과하겠다고 밝힌 도널드 트럼프 미 행정부의 발표를 추산한 결과다(（no translation））

这是根据特朗普政府宣布从下个月1日起对中国价值3000亿美元的产品征收10%追加关税进行推算的结果

3000억 달러는 중국의 전체 대미 수출의 약 60%이며, 여기에 10%의 관세를 부과하면 대미 수출 전체에 미치는 영향은 6%가 된다 7. 0136위안인 현 위안화 환율에 6%의 인상을 적용하면 달러당 7. 3위안이 된다고 니혼게이자이는 분석했다 일각에서는 위안화 환율이 7. 3위안까지 상승하면 중국 기업의 달러화 부채가 급증해 위안화 투매 및 가치 추가 하락이 일어날 수도 있다고 우려하고 있다 (（no translation））

3000亿美元是中国对美国出口总额的约60%,如果在此基础上加征10%的关税,对美国出口整体影响为6%据《日本经济新闻》分析,目前人民币汇率为7.0136元,若按6%的升值幅度计算,1美元将兑换7.3元人民币。也有人担心,如果人民币对美元汇率下跌到7.3:1,中国企业的美元负债将会剧增,从而引发人民币抛售及价值的进一步下跌

（（additional translation））China views Hong Kong protests as 'color revolution'

（增译）中国认为香港示威是“颜色革命”

（（additional translation））China has characterized the ongoing antigovernment protests in Hong Kong as a "color revolution" for the first time,

（增译）中国当局首次把香港示威定性为指称政权更迭运动的“颜色革命”

（（additional translation））a reference to uprisings aimed at replacing the government, sparking speculation

（增译）对此,有分析认为,以大规模示威持续的本周末为转折点,中国可能针对香港开始投入军队等武力介入

（（additional translation））that an armed intervention could be imminent Zhang Xiaoming, director of the Hong Kong and Macao Office of the State Council, made the remarks on Wednesday at a closed-door symposium in Shenzhen, Guangdong Province, which borders with Hong Kong "The movement about the extradition bill has gone bad, and it has the clear characteristics of a color revolution,"

（增译）中国国务院港澳办公室主任张晓明7日在与香港接壤的广东省深圳市举行的非公开座谈会上表示:“‘罪犯引渡法’事件已经变质，明确地出现了颜色革命的特点”

（（additional translation））Zhang noted More than 550 people attended the meeting, including Hong Kong deputies to the National People's Congress and members of the Chinese People's Political Consultative Conference "Hong Kong is facing the most serious situation since its return to China,” Zhang said "If the situation in Hong Kong continues to worsen into unrest that the Hong Kong Special Administrative Region government cannot control, the central government will not sit back and do nothing…

（增译）550名全国人大代表 全国政协(国家咨询机构)委员参加了当天的座谈会张晓明说:“目前形势是1997年香港回归以来最严重的一次”,“如果香港局势进一步恶化,香港特区政府不能控制动乱,中央(中国政府)绝不会坐视不管

（（additional translation））According to the Basic Law (of Hong Kong), the central authorities have ample methods as well as sufficient strength to promptly settle any possible turmoil," he said

（增译）”他提高声音表示:“根据香港基本法,中央有足够的办法和强大力量迅速镇压各种动乱

（（additional translation））Xiakedao, the official social media account of the Chinese Communist Party's newspaper Renminribao (People's Daily), which offers its interpretations of the party's intentions, said that this is the first time the Chinese central government used "color revolution" to describe the protests in Hong Kong, adding that it is a "clear signal" from Beijing to the protesters in Hong Kong Color revolution refers to various movements for a change of government in some countries in the former Soviet Union, Eastern Europe and the Middle East since the 1990s

（增译）”一直解释中国共产党意图的中国共产党机关报《人民日报》的社交媒体官方账号“侠客岛”表示,“中国当局首次判断香港示威是颜色革命,”认为这是中国当局向香港示威队伍发出了明确的信号。“颜色革命”是社会主义国家阵营开始崩溃的1990年代前苏联国家和东欧 中亚 中东等地区发生的政权更迭运动

（（additional translation））The Chinese leadership, led by President Xi Jinping, showed strong wariness about a color revolution early this year

（增译）中国国家主席习近平领导的领导层今年年初流露出对颜色革命强烈的警惕

（（additional translation））At a meeting with senior law-enforcement officials in January, China's State Councilor and Minister of Public Security Zhao Kezhi urged China's police to "stress the prevention and resistance of 'color revolutions' and firmly fight to protect China's political security" and to "strike back against all kinds of infiltration and subversive activities by hostile foreign forces "

（增译）中国国务委员兼公安部部长赵克志今年1月召集全国公安干警后表示:“要聚集所有公安的智慧和力量,阻止颜色革命要打击内外敌对势力渗透到各种破坏颠覆活动之中”

（（additional translation））According to Chinese media, Zhang quoted former Chinese leader Deng Xiaoping's remarks about sending Chinese troops to Hong Kong, saying, "Deng was far-sighted " Wang Zhimin, director of the Chinese Liaison Office in Hong Kong, said that the most urgent thing is to put the chaos under control to restore order, asserting that it is now a "battle of life and death" for Hong Kong’s future and "battle to defend Hong Kong"

（增译）中国当局将香港示威规定为“颜色革命”后,有人指出这是动用武力的全面介入的信号,也是基于此的判断碰巧在座谈会前一天的6日,深圳市举行了假定香港示威队伍的大规模镇压暴动演习中国媒体透露,张晓明就邓小平在过去所说的“如果有必要,可以向香港投入中国军队”的说法表示:“邓小平有先见之明”与张晓明一起主持座谈会的中国香港联络办公室主任王志民表示:“香港已经到了无路可退的地步”,“控制混乱局面 恢复秩序是最为紧迫的事情,这是关系到香港的未来和命运的战争和保卫战”

（增译）《环球时报》等中国媒体8日批评2014年香港民主化示威“雨伞革命”的领导人黄之锋 罗冠聪等人6日会见了美国驻香港总领事馆负责人,并抨击美国干涉香港问题如果中国真的动员军队,美国等外部势力就会介入其中,进而在香港引发政权颠覆运动此外,也有人谨慎地指出,在与美国展开贸易 关税 汇率等全面战争的情况下,如果再制造一个尖锐的矛盾战线,将会给中国领导层带来额外的负担

사드 처음 명시한 中국방백서… “긴장 고조” 美비난 China’s defense white paper makes mention of THAAD deployment in S Korea

中国国防白皮书首次明确提及萨德系统,猛批萨德“加剧紧张”

중국 국방부가 24일(현지 시간) 발표한 2019 국방백서에서 ‘사드(THAAD·고고도미사일방어체계)’를 거론하며 한미 양국에 날을 세웠다 China has raised issue with the United States’ deployment the THAAD antimissile defense system in South Korea in its defense white paper for the first time

中国国防部24日(当地时间)发表的《2019国防白皮书》中提到了“萨德”,并向韩美两国亮出了兵刃

백서는 “미국이 한국에 사드를 배치하면서 아시아태평양지역의 전략적 균형과 안보 이익을 엄중하게 훼손했다”고 주장했다 The white paper claimed that the THAAD deployment “gravely damaged” the strategic balance and security interest in the Asia-Pacific region

白皮书说,“美国在韩国部署萨德系统,严重损害了亚太地区的战略平衡和安全利益”

중국 국방부는 이날 ‘신시대 중국 국방’이란 제목의 약 90쪽의 백서를 공개했다 China’s defense ministry released the 90-odd-page white paper in question on Wednesday

中国国防部当天发表了约90页的白皮书,题目是《新时代的中国国防》

중국은 1988년 첫 백서를 발간한 후 통상 2년에 한번씩 발간해왔다 Since the first issuance in 1988, China has published its defense white paper usually every two years

中国自1988年发行第一本白皮书后,通常每两年发行一次

이번 백서는 10번째로 2015년 발간 후 4년 만에 이뤄졌다 This year’s white paper, the 10th edition, was released for the first time in four years

此次白皮书是2015年出版后时隔4年的第10次发表

특히 4년 전 백서 분량은 약 20쪽이었지만 이번 백서에는 사드, 남북문제, 미국, 일본, 호주, 대만 등 다양한 주제를 담으며 중국의 주장을 노골적으로 강조했다 The 2015 edition was about 20 pages long, while this year’s edition is much longer, explicitly highlighting Beijing’s claims on various security issues involving the THAAD, the inter-Korean issues, the United States, Japan, Australia and Taiwan

特别是,4年前的白皮书长约20页,但此次白皮书中包含了萨德 韩朝问题 美国 日本 澳大利亚 台湾等多方面主题,露骨地强调了中国的主张

백서는 “세계 경제와 전략 중심이 아시아태평양 지역으로 옮겨지고 있다 Nothing that the world’s economic and strategic center is moving toward the Asia-Pacific region,

白皮书说:“世界经济和战略重心正在向亚太地区转移

이 지역에서 대국끼리 게임을 하면서 지역 안전에 불확실성을 가져왔다 the white paper said that “games” played by great powers brought uncertainties to regional security,

在这个地区,大国之间博弈,给地区安全带来了不确定性

미국은 아태 군사 동맹을 강화하고 군사 배치 및 간섭을 확대하면서 이 지역에 복잡한 요소를 더했다”고 미국을 비난했다 accusing the United States of “adding complexity” to the region by strengthening military alliances and expanding military deployment and intervention

美国在加强亚太军事同盟 扩大军事部署及干涉的同时,给该地区增添了复杂因素”

남북문제에서도 중국이 주도적 역할을 하겠다는 뜻을 드러냈다 China also expressed its intention to play a leading role in the inter-Korean issues

这表明中国在韩朝问题上也将发挥主导作用

특히 “한반도에서 긍정적인 진전이 있었지만 여전히 불확실성이 남았다 “Despite positive progress made on the Korean Peninsula, there remain uncertainties,” the white paper said

白皮书特别强调自我评价称:“虽然半岛取得了积极进展,但仍然存在不确定性

중국은 한반도 같은 분쟁지역에서 정치적으로 건설적인 역할을 하고 있다”고 자평했다 “China is playing a politically constructive role in troubled regions such as the Korean Peninsula

中国在半岛等纷争地区发挥着政治上的建设性作用”

중국은 일본과 호주에도 강한 경계를 드러냈다 ” China was also wary about Japan and Australia

中国对日本和澳大利亚也表现出了强烈的警惕

백서는 “일본이 전후 체제를 우회하기 위해 군사 안전 정책을 변경하면서 활발한 군사 움직임을 보이고 있다 “Japan is making brisk military moves by changing its military security policy in order to circumvent the post-war regime

白皮书说:“日本为绕过战后体制而改变军事安全政策,军事活动十分活跃

대외 지향적인 군사 움직임이 보인다”고 경계했다 Externally oriented military moves are seen,” the white paper said

出现了对外指向性的军事动向”

호주도 미국과 군사 동맹을 강화하면서 아태지역 안보 플레이어로 부상하고 있다고 진단했다 It also viewed Australia as emerging security player in the region by strengthening its alliance with the U S

白皮书认为,澳大利亚也与美国加强军事同盟,正在跃升为亚太地区的安保玩家

특히 대만 문제에 대해서는 “평화통일 및 일국양제 방침을 견지한다 Regarding the Taiwan issue, the white paper again targeted Washington, noting that Beijing maintains its policy of seeking peaceful reunification and "one country, two systems

特别是在台湾问题上,白皮书表示“坚持和平统一和一国两制的方针

중국을 분열하려는 시도와 외국의 내정간섭에 반대한다”고 또다시 미국을 겨냥했다 （no translation）

反对分裂中国的企图和外国干涉中国内政”,把矛头指向美国

남아시아 지역에서 경쟁 중인 인도에도 날을 세웠다 （no translation）

中国还向在南亚地区展开竞争的印度也亮出了兵刃

백서는 “남아시아에서 인도와 파키스탄의 충돌이 있다 （no translation）

白皮书说:“在南亚,印度和巴基斯坦有冲突

또 일부 국가의 영토 및 해양 분쟁, 민족과 종교 갈등이 끊임없이 발생하고 있다”고도 분석했다 （no translation）

一些国家的领土和海洋争端 民族和宗教矛盾不断发生”

백서는 “중국은 국방비 및 사용처를 투명하게 공개한다”며 현황을 표로 소개했다 （no translation）

白皮书以表格形式介绍了当前的情况,称“中国公开透明地公开国防开支及用途”

2012∼2017년 국내총생산(GDP) 대비 국방비 비중 평균은 1. 3%로 러시아(4. 4%) 미국 (3. 5%) 인도(2. 5%) 프랑스(2. 3%)보다 낮다고 강조했다 （no translation）

白皮书强调,在2012年至2017年国内生产总值(GDP)相比,中国的国防开支占比平均为1.3%,低于俄罗斯(4.4%) 美国(3.5%) 印度(2.5%) 和法国(2.3%)

美-中, 화웨이 불씨 둔채 무역 2차휴전 Trump, Xi agree to truce in U S -China trade war

美中贸易战二次停火,保留华为火种

도널드 트럼프 미국 대통령과 시진핑(習近平) 중국 국가주석이 지난달 29일 일본 오사카 주요 20개국(G20) 정상회의에서 80분간의 무역 담판을 갖고 신규 관세 부과 보류와 무역협상 재개를 약속한 ‘무역전쟁 2차 휴전’에 합의했다 U S President Donald Trump and Chinese President Xi Jinping reached the second truce in which they agreed to resume trade negotiations and hold off on a new round of tariffs after sitting down with each other for 80 minutes at the G20 Summit held in Osaka, Japan on Saturday

美国总统唐纳德·特朗普和中国国家主席习近平6月29日在日本大阪的二十国集团(G20)首脑会议上举行80分钟的贸易谈判,就“第二次贸易战停火”达成协议,承诺暂停新增征收关税,并重启贸易谈判

트럼프 대통령은 이날 시 주석과 양자 정상회담을 마친 뒤 기자회견에서 “우리는 협상을 계속하기로 합의했다”며 “We agreed today that we would continue the negotiations,” said Trump after the bilateral meeting with Xi

特朗普总统当天在与习主席举行双边会谈后举行的记者招待会上表示:“我们同意继续进行协商,

“3250억 달러(약 375조5375억 원)어치(중국산 수입품)에 대한 관세를 부과하지 않기로 합의했다”고 밝혔다 “We agreed I would not be putting tariffs on 325 billion U S dollars that I would have the ability to put on if I wanted

决定不对价值3250亿美元(约合375.5375万亿韩元)的中国进口商品加征关税”

이어 “우리는 관세 부과를 보류하고 그들(중국)은 농산품을 구매할 것”이라고 설명했다 ” According to Trump, China has agreed to buy a "tremendous amount" of agricultural products immediately " (（additional translation））We are going to give them lists of things we want them to buy," Trump said

他接着解释说:“我们暂缓征收关税,他们(中国)将购买(美国)农产品”

두 정상은 지난해 12월 1일 아르헨티나 부에노스아이레스 G20 회의에서 추가 관세 인상을 보류하고 90일간 무역협상을 진행하는 ‘1차 휴전’에 합의한 바 있다 Back on Dec 1 last year, the two heads of state had agreed to delay an additional tariff increase and negotiate for 90 days at the G20 summit in Buenos Aires, Argentina

去年12月1日,两位首脑在阿根廷布宜诺斯艾利斯G20峰会上就保留追加关税 进行90天贸易谈判的“第一次停火”达成协议

트럼프 대통령은 지난달 29일 “미국의 훌륭한 기업들이 화웨이에 제품을 계속 판매할 것”이라며 중국 최대 통신기업 화웨이에 대한 제재 완화를 시사했다 Trump hinted on easing sanctions on Chinese communication company Huawei on Saturday “U S companies can sell their equipment to Huawei

特朗普总统6月29日表示,“美国的优秀企业将继续向华为销售产品”,暗示将放宽对中国最大的通信企业华为的制裁

다만 “화웨이를 마지막까지 남겨둘 것”이라며 “(그 거래가) 국가안보 우려를 일으키지 않는 한”이라고 단서를 달아 무역협상의 최후 카드로 남겨뒀다 We’re talking about equipment where there’s no great national security problem with it,” the U S president said

但他同时表示,“将把华为留到最后”,“前提是只要(该交易)不引起国家安保担忧”,留下这一线索作为贸易谈判的最后筹码

이와 함께 “중국인 학생들이 와서 우리의 훌륭한 학교와 대학을 이용하게 하길 원한다”고 덧붙였다 “We want to have Chinese students [go] to our great schools and great universities

同时他还表示:“希望中国学生能够前来美国,利用我们优秀的学校和大学”

두 정상은 전날에도 비공식 만남을 가졌으나 이날 정상회담은 이례적으로 80분간 이어졌다 ” Trump and Xi had also an unofficial meeting the previous day, but the bilateral summit exceptionally lasted for 80 minutes on Saturday

两位首脑在前一天也进行了非正式会谈,但是当天的首脑会谈破例地持续了80分钟

트럼프 대통령은 “만약 우리가 합의하지 못하면 (관세 부과로) 돌아갈 것”이라며 "My Plan B is that if we don't make a deal, I will tariff I am in no hurry, but things look very good,” Trump wrote in a post on Twitter

特朗普说:“如果我们无法达成协议,就会重新回到(征收关税)上来,”

“나는 서두르지 않는다 올바른 합의를 하길 원한다”고 말했다 “The quality of the transaction is far more important to me than speed”

“我不着急希望达成正确的协议”

중국 외교부에 따르면 시 주석은 회담에서 “중국의 주권과 존엄 관련 문제에서 중국은 반드시 핵심 이익을 수호할 것”이라고 강조했다 According to the Foreign Ministry of China, Xi emphasized that he would spare no effort to safeguard the benefits of China with regard to China’s sovereignty and dignity

据中国外交部透露,习主席在会谈中强调:“在事关中国主权和尊严的问题上,中国必须维护核心利益”

오사카 G20 정상회의는 이날 ‘공정한 무역’의 중요성을 강조하는 ‘오사카 선언’을 끝으로 폐막했다 The G20 Summit in Osaka closed with the “Osaka Declaration,” which emphasized the importance of fair trade

大阪G20峰会当天在强调“公平贸易”重要性的《大阪宣言》后闭幕

G20 정상들은 세계 경제의 ‘하방 리스크가 크다’며 ‘리스크에 대처하기 위해 다양한 행동을 할 것’이라고 선언에서 밝혔다 The heads of the G20 member states announced that the global economy was ridden with downside risks and they would take various actions to handle the risks

G20领导人在宣言中表示,世界经济“下行风险很大”,将“采取各种行动应对风险”

‘보호무역주의를 반대한다’는 취지의 표현은 미국의 반대로 지난해 아르헨티나 회의에 이어 다시 제외됐다 Opposition to protectionism was excluded from the announcement this time as well due to the opposition of the U S , following last year’s summit in Argentina

以“反对贸易保护主义”为主旨的措辞,因为美国的反对,继去年阿根廷会议之后再次被排除在宣言内容之外

방북 임박說시진핑, 비핵화 훼방꾼은 되지 말아야 Xi Jinping must not hinder denuclearization efforts

传习近平将访问朝鲜,不要成为无核化的干扰者

시진핑 중국 국가주석이 이달 말 주요 20개국(G20) 정상회의 전에 북한을 방문해 김정은 국무위원장과 정상회담을 할 것이라는 관측이 나오고 있다 Speculation is brewing that Chinese President Xi Jinping will meet Kim Jong Un, the young leader of North Korea, before visiting Osaka for the upcoming G20 summit late this month

有分析认为,中国国家主席习近平将在本月底的二十国集团(G20)首脑会议之前访问朝鲜,与朝鲜国务委员会委员长金正恩举行首脑会谈

2·28 하노이 북-미 정상회담 결렬 이후 북한이 일체의 대화를 거부하는 상황에서 이달 말 주요 20개국(G20) 정상회의를 전후해 시 주석의 방북이 이뤄진다면 동북아 정세에 미묘한 변화를 낳는 이벤트가 될 수 있다 With the communist regime refusing to have any dialogue since the fiasco in Hanoi, Mr Xi’s visit could serve as an event to bring about subtle change in the diplomatic landscape in North East Asia

自2月28日朝美首脑河内会谈破裂之后,朝鲜拒绝一切对话,在这种情况下,如果习近平主席在G20峰会前后访朝,这一活动将给东北亚局势带来微妙的变化

비밀을 상대국에 대한 예우로 여기는 북-중 관계의 특성 탓에 시 주석 방북은 공식 발표가 있기 전까지 확인하기 어려운 게 사실이다 It is hard to verify the visit before an official announcement as both Beijing and Pyongyang consider secrecy as a diplomatic courtesy

由于把秘密视为对对方国家礼遇的朝中关系的特性,习主席访朝在正式宣布之前,确实难以得到确认

김정은 북한 국무위원장이 이미 네 차례나 중국을 방문한 만큼 올해 상반기 안에 시 주석 답방이 이뤄질 것으로 예상됐지만 하노이 회담이 결렬되면서 시 주석 방북은 미뤄졌다 Pundits expected that President Xi would visit Pyongyang in return soon, given that Kim Jong Un has already visited China four times, but it has been put off after U S -North Korea summit in Hanoi fell apart

朝鲜国务委员会委员长金正恩已经四次访问中国,因此人们预计今年上半年内习主席会回访,但是随着河内会谈的破裂,习主席的访朝日程被推迟

이번 시 주석 방북 추진설은 미국과의 무역·기술전쟁에다 대만·홍콩을 둘러싼 내정간섭 논란까지 날카롭게 대립하는 상황에서 나왔다 The speculation over Xi’s upcoming visit came out amid the escalating disputes with Washington over trade and technology and the criticism over interfering in the internal affairs of Taiwan and Hong Kong

此次有关习主席访朝的说法,是在与美国的贸易 技术战以及围绕台湾 香港的内政干涉争议尖锐对立的情况下传出的

중국은 재작년 미중 무역갈등 초기에 그랬듯이 이번에도 미중 대결의 지렛대이자 반전 카드로 북핵 문제를 꺼내들 것이라는 관측이 나온다 Some experts say that Beijing will utilize Pyongyang as an ace up the sleeve and leverage against Washington as it did during the early phase of trade war in 2017

有分析认为,就像前年美中贸易矛盾初期一样,中国此次也将把朝核问题作为美中对抗的杠杆和反转筹码

시 주석으로선 김정은의 후견인으로서 영향력을 확인하는 한편 G20에서 만날 도널드 트럼프 미국 대통령에게 북한 해법을 내밀며 미중 갈등의 휴전을 노릴 수도 있다 The North Korean card will be both an opportunity for Xi to reaffirm his clout as backer of Kim Jong Un and a solution to present to President Trump during their meeting at G20 as a way to bring about a ceasefire for the on-going trade war

习主席一方面可以确认作为金正恩监护人的影响力,另一方面也可以向将在G20峰会中见面的美国总统特朗普提出解决朝鲜问题的方案,以期实现美中矛盾的停战

요즘 북한은 한미의 거듭된 대화 재개 촉구에도 묵묵부답으로 일관하고 있다 The North has remained silent despite the repeated requests for dialogue from Seoul and Washington

最近,朝鲜对韩美有关恢复对话的一再敦促始终保持沉默

그간 북한의 입장을 배려하던 문재인 대통령마저 북유럽 순방에서 거듭 북한이 먼저 핵 폐기 의지를 보이라고 촉구한 것은 그만큼 북한에 대한 답답함과 실망감을 반영하는 것이다 Even President Moon Jae-in, who has been much accommodating to Kim Jong Un, is urging the North Korean leader to show the will to disarm his nuclear weapons, which reflects Mr Moon’s frustration and disappointment towards Pyongyang

此前一直照顾朝鲜立场的文在寅总统,也在北欧巡访中多次敦促朝鲜首先表现出弃核的意志,这反映了对朝鲜的郁闷和失望

이런 시점에 시 주석의 방북이 이뤄진다면 북한이 대화 재개에 나서도록 만드는 계기가 될 수도 있다 President Xi’s visit to North Korea could serve as a turning point for the regime to engage in dialogues again

这种情况下,习主席访朝如果成行,将成为让朝鲜参加恢复对话的契机

하지만 중국의 대북 외교는 김정은의 비핵화 결단을 압박하고 지원하는 것이어야지, 북-중 밀착을 과시하며 김정은이 딴 마음을 품게 하는 것이어선 안 된다 However, China’s diplomatic tactics towards North Korean must be focused on pressuring the North to disarm the arsenal rather than boasting their ties, which will encourage Kim Jong Un to harbor an ulterior motive

但是,中国的对朝外交应该是对金正恩的无核化决断进行施压和支持,而不是展示朝中密切关系 让金正恩怀有异心

특히 중국이 나서 국제사회의 대북제재 전선을 무너뜨린다면 돌이키기 어려운 결과를 낳을 뿐이다 If China steps in to undermine the sanctions of international community, it will only beget more trouble

特别是中国如果出面摧毁国际社会的对朝制裁战线,只会造成难以挽回的后果

북한이 지난해 초 협상에 나올 수밖에 없었던 것도 중국까지 제재에 동참했기 때문이었다 It is noteworthy that North Korea had to come back to the negotiating table early last year because China participated in the sanctions

朝鲜在去年年初不得不参加协商,也是因为连中国也参与了对朝制裁

중국이 세계를 지배하면 If China rules the world

如果中国统治世界

캐나다는 미국의 요청을 받고 중국 최대 통신기업 화웨이의 최고재무책임자(CFO) 멍완저우(孟晩舟·46) 부회장을 밴쿠버 공항에서 체포했다가 미중 무역전쟁의 급류에 빨려 들어갔다 China has emphasized that it pursues egalitarian multilateralism to counter the West’s hegemony on the international stage, but its actual course of actions is confusing at best

应美国的要求,加拿大在温哥华机场逮捕了中国最大通信企业华为的副董事长 首席财务官孟晚舟(46岁),结果被中美贸易战争的激流所吞噬

중국은 캐나다인 2명을 억류하며 압박했고, 멍 부회장은 보석으로 풀려났다 （no translation）

中国扣留了两名加拿大人施压,孟晚舟则保释出狱

중국의 뒤끝이 얼마나 심했는지 캐나다 고가 패딩 브랜드인 ‘캐나다구스’ 주가마저 급락했다 （no translation）

中国的后劲之大,导致加拿大高价羽绒服品牌“加拿大鹅”的股价也暴跌

절대 강자인 미국엔 침묵하면서 미국을 돕는 동맹국에는 가차 없이 보복하는 대국의 민낯이 드러난 게 처음은 아니다 （no translation）

对绝对强者美国保持沉默,对帮助美国的盟国进行毫不留情的报复,这并不是中国第一次暴露出大国的真面目

북한 미사일을 막기 위해 미국이 한국에 사드(THAAD·고고도미사일방어체계)를 배치했을 때 중국은 한국을 때렸다 （no translation）

美国为阻止北韩导弹而在韩国部署萨德系统时,中国对韩国进行了打击

중국이 단체관광객의 한국 방문을 막으며 한국 경제를 압박했을 때 그들을 평생 친구로 생각했던 한국인들은 당황할 수밖에 없었다 （no translation）

当中国阻止团体游客访问韩国 压迫韩国经济时,认为他们是一生朋友的韩国人只能惊慌失措

중국은 유엔과 같은 국제무대에선 서구의 패권에 대항하는 수평적 다자주의를 강조하지만 실제 행보는 혼란스럽다 （no translation）

中国在联合国这样的国际舞台上强调对抗西方霸权的“水平多边主义”,但实际行动却非常混乱

봉건시대 제후국을 대하듯이 중국 중심의 수직적 위계질서를 강조하고 역린(逆鱗)을 건드리면 크건 작건 본때를 보이는 패권국가의 전근대성도 보인다 China stresses vertical hierarchical order centered on China as if it is treating neighboring countries as subordinate states during the feudal age If a neighboring country causes its wrath, it does not hesitate to retaliate against the country, be it big or small, just like a hegemonic country would do in the pre-modernistic era

就像封建时代对待诸侯国一样,中国强调以中国为中心的垂直等级秩序,一旦有人碰触其逆鳞,或多或少,总是显露出霸权国家的前近代性

인구 2만여 명의 남태평양 작은 섬나라 팔라우도 중국에 그렇게 당했다 Likewise Palau, a tiny Pacific island with a population of some 20,000, suffered damage from China’s retaliatory action

拥有2万多人口的南太平洋小岛国帕劳就曾受到中国的这种对待

하루에 태어나는 중국 신생아 수밖에 안 되는 인구를 가진 이 작은 섬나라가 대만과 국교를 맺고 있으니 The island country, whose population equals the number of daily newborns in China, formed diplomatic ties with Taiwan

只拥有中国一天新生儿人口的这个小岛国与台湾建交,

‘하나의 중국’ 원칙을 일관되게 주장하고 있는 중국에는 눈에 든 가시나 다름없었을 것이다 This angered China, which is consistently following "One China" policy

对于一贯主张“一个中国”原则的中国来说,这无疑是眼中刺

중국 정부는 지난해 외교관계가 없다는 이유로 팔라우 단체여행을 막았다 The Chinese government imposed a ban on group travels to Palau by Chinese tourists by citing lack of diplomatic ties

中国政府去年以没有外交关系为由,阻止了帕劳团体游

대만 항공사가 운영하는 팔라우 퍼시픽에어웨이스는 중국인 여행객이 절반으로 줄어들자 올해 7월 중국 노선을 폐지했다 As the number of Chinese tourists to the island state was halved, Palau Pacific Airways, run by a Taiwanese airliner, shut off its China routes in July

台湾航空公司运营的帕劳太平洋航空公司因中国游客减少一半,今年7月停飞了中国航线

섬에 호텔을 짓고 건물을 사들이던 중국인 큰손 투자자도 손을 놓았다 Large Chinse investors who had constructed hotels and bought large buildings on the island also dumped their properties there

在岛上建酒店 购买建筑的中国大腕投资者也放弃了投资

관광 등 서비스업 비중이 경제의 80% 이상을 차지하는 팔라우가 받은 타격은 엄청났다 Palau, whose service industry including tourism accounts for 80 percent of its economy, was dealt with a massive blow

旅游等服务业比重占经济80%以上的帕劳受到了巨大的打击

중국은 세계 인구의 약 20%를 차지하는 인구대국이라는 점에서 소수의 인구로 세계 패권을 쥐었던 영국이나 미국과는 질적으로 다르다 Since China's population accounts for 20 percent of the global population, it is completely different from the U K or the U S , which enjoyed supremacy with relatively small populations

中国是占世界人口约20%的人口大国,因此与以少数人口掌握世界霸权的英国和美国有着本质上的区别

관광산업마저 무기화할 수 있는 규모의 경제와 힘을 갖고 있다 China has so large economy of scale and power that it can even weaponize its own tourism industry

中国拥有着连旅游产业也能当作武器来使用的大规模经济和力量

여기에다 위안화가 달러와 같은 기축통화 반열에 오른다면 미국의 독자제재처럼 중국 금융시스템에서 퇴출시키는 것만으로 한 나라 경제를 위협할 수 있게 될 것이다 In addition, if the Chinese yuan emerges as a key currency like the U S dollar, it will be able to threaten the entire economy of a country simply by excluding economic players from China’s financial system, the way the U S does with its independent sanctions

而且,如果人民币能够进入美元等国际储备货币的行列,那么像美国的单方面制裁一样,只要驱离中国金融系统,就能威胁到一个国家的经济

한국, 캐나다, 팔라우에서 일어난 일들은 어쩌면 중국이 세계를 지배하면 벌어질 일의 예고편에 불과할지 모른다 What have happened in South Korea, Canada and Palau may herald what will happen when China comes to rule the world

在韩国 加拿大 帕劳发生的事情,也许只是中国支配世界后就会发生的预告而已

이런 중국과 싫든 좋든 등을 맞대고 함께 살아가야 하는 게 한반도의 운명이다 It is the fate of the Korean Peninsula that neighbors and should co-exist with China, whether we like it or not

无论喜欢还是讨厌,韩半岛的命运都是要与中国面对面生活下去

대중 외교를 강화하려고 한다면 한쪽으로 기울어진 ‘친중파’보다 중국과의 관계를 긍정적으로 보면서 잠재적 위험 요인까지 따지는 ‘낙관적 현실주의자’가 훨씬 더 많아야 한다 If South Korea is to strengthen its China diplomacy, Seoul needs more "optimistic realists" who carefully take into account potential risks while positively perceiving relations with China at the same time, rather than "pro-Chinese people" who are largely biased

要想加强对华外交,比起倾向于一方的“亲中派”,更应该是更多的“乐观的现实主义者”,即积极看待同中国的关系,同时盘算潜在的危险因素

정치 경제 사회 문화 등에서 특정 국가에 대한 쏠림은 없는지 점검하고 대비하는 중장기 국가 전략시스템도 가동해야 한다 Seoul should run a system of mid- to long-term national strategy that will examine whether the government is not excessively biased towards a certain country in the areas of politics, economy, society and culture, and prepare itself accordingly

同时也应该启动检查政治 经济 社会 文化等方面是否过于集中于特定国家并采取应对措施的国家中长期战略系统

팔라우는 중국인 관광객이 전체의 절반에 육박할 정도로 커진 뒤에야 중국에 의존하는 자국 관광산업의 취약점을 깨달았다 Palau learned the weaknesses of its tourism industry only after Chinese tourists accounted for nearly half its foreign tourists

帕劳在中国游客增加到接近总体的一半之后,才认识到了本国旅游产业过于依赖中国的弱点

뒤늦게 중국의 위협에 맞서 중국인 단체관광객 대신 1인당 매출액이 훨씬 큰 유럽이나 일본인 관광객 유치를 위해 친환경 고부가가치 관광산업을 집중 육성하기 시작했다 In a bid to counter China’s threat belatedly, Palau started to promote an eco-friendly, high value-added tourism industry in order to attract tourists from Europe and Japan, whose per-capita expenditure is larger than Chinese

为对抗中国的威胁,尽管为时略晚,帕劳开始集中培养环保高附加价值旅游产业,以吸引人均销售额远高于中国团体游客的欧洲或日本游客

산호초 보호를 위해 미국 하와이에 이어 선크림 사용을 선제적으로 금지한 이유다 For this reason, the island country started to preemptively ban the use of sun cream to protect its coral reefs, following Hawaii’s suit

这就是为了保护珊瑚礁,继美国夏威夷之后,帕劳率先禁止使用防晒霜的原因

최근 팔라우 당국은 중국인 관광객은 줄었지만 1인당 매출액이 늘면서 전체 관광 매출은 증가했다고 발표했다 The Palau government recently said while Chinese tourists have declined in number, it saw tourism revenue increase due to a hike in per-capita expenditure by foreign tourists

最近帕劳当局表示,虽然中国游客有所减少,但随着人均销售额增加,旅游总销售额也相应有所增加

미중 무역전쟁과 섬나라 팔라우에서 일어난 일들은 ‘전쟁의 승패는 싸우기 전에 판가름 난다’고 했던 손자의 말을 곱씹어 보게 한다 The U S -China trade war and what has happened in Palau remind us of the lesson in the "Act of War" that the winner and loser of a war are decided even before the war breaks out

美中贸易战和在岛国帕劳发生的事件,让我们回味一下孙子所说的“战争的胜败,在战争之前就已见分晓”

화웨이 진영 싸움 확전 조짐중국 통신장비업체 화웨이 창업자의 딸이자 최고재무책임자(CFO)인 멍완저우(孟晩舟) 부회장이 대이란 제재를 위반한 혐의로 캐나다에서 체포된 뒤 그 후폭풍이 미중 간 무역협상으로 번지고 있다 Huawei CFO’s arrest could impact U S -China trade talks Meng Wanzhou, Huawei Technologies' chief financial officer and the daughter of the company's founder, has been arrested in Canada on charges of violating U S sanctions against Iran, and her arrest is stirring a ripple effect on the on-going trade talks between Washington and Beijing

华为之争出现扩大征兆中国通信设备企业华为创始人的女儿 首席财务官(CFO) 副董事长孟晚舟因涉嫌违反(美国)对伊朗的制裁而在加拿大被捕,其后续影响正蔓延到美中两国之间的贸易谈判

미중은 1일 아르헨티나에서 가진 정상회담을 통해 무역전쟁 휴전에 합의하고 90일 협상에 돌입했지만 이 문제가 협상을 망가뜨릴 수 있는 뇌관으로 부상한 것이다 Meng’s arrest is fast emerging as a tinder box that could rupture the current talks, after the two countries agreed to a temporary 90-day truce at the summit meeting in Argentina on December 1

美中两国1日在阿根廷举行首脑会谈,就贸易战停战达成协议并进入为期90天的谈判,但这一问题却成为可能破坏谈判的导火索

미국 측 협상 책임자인 로버트 라이트하이저 미무역대표부(USTR) 대표는 9일(현지 시간) CBS방송 인터뷰에서 “내 생각에 (90일은) 단호한 최종시한”이라며 "As far as I'm concerned, it's a hard deadline," said U S Trade Representative Robert Lighthizer during his CBS interview on Sunday (local time)

美方谈判负责人 美国贸易代表办公室代表罗伯特·赖特希泽当地时间9日在接受CBS电视台采访时表示,“我认为(90天)是明确的最终时限,”

“내가 (도널드 트럼프) 대통령과 이야기할 때 그는 3월 1일을 넘기는 것을 말하지 않는다”고 말했다 "When I talked to the president of the United States, he's not talking about going beyond March

“我和(特朗普)总统说话时,他说不要超过3月1日”

그러면서 “90일이 지나면 관세가 인상될 것”이라고 강조했다 ” He stressed that at the end of 90 days, the tariffs will be raised

他同时强调:“90天之后,关税将提高”

‘미중 정상회담 만찬 당시 트럼프 대통령이 멍 부회장 체포 사실을 알고 있었느냐’는 질문엔 “알지 못했다 분명히 말할 수 있다”며 “무역협상은 멍 부회장의 체포에 영향을 받지 않아야 한다”고 강조했다 When asked if President Donald Trump had known about the arrest during his summit dinner with President Xi Jinping, Lighthizer denied such a possibility, emphasizing that trade talks must not be affected by Meng’s arrest

对于“美国总统特朗普在美中首脑会谈晚宴时是否知道逮捕孟晚舟一事”的提问,他回答说,“不知道这一点可以明确”他强调:“贸易谈判不应受到孟晚舟被捕一事影响”

하지만 멍 부회장 체포가 미중 무역협상에 악영향을 줄 것이라는 분석이 잇따르고 있다 However, many experts are of the view that the CFO’s arrest will have a negative impact on the talks between the U S and China

但有不少分析认为,孟晚舟被捕会对美中贸易谈判产生负面影响

뉴욕타임스(NYT)는 “멍 부회장의 구금은 미중 경제 관계를 상당히 복잡하게 만들었다”며 “Ms Meng’s detention has considerably complicated China’s economic relations with the United States,” said the New York Times,

《纽约时报》报道称,“孟晚舟被扣押使美中经济关系变得相当复杂”,

“중국인들의 자존심인 화웨이 문제가 분노와 놀람을 일으켰다”고 전했다 adding that it has ignited anger and astonishment in China, where Huawei is a source of national price

“中国人的自尊心——华为问题引发了愤怒和惊吓”

월스트리트저널(WSJ)은 “멍 부회장 체포가 중국의 민족주의를 자극하면서 시 주석의 양보를 어렵게 만들고 있다”고 평가했다 “The imbroglio has produced a nationalist backlash in China that could make it difficult for Mr Xi to make concessions to the U S ,” reported the Wall Street Journal

《华尔街日报》评论说:“孟晚舟被捕刺激了中国的民族主义,让习主席很难做出让步”

전날 베이징 주재 캐나다대사를 불러 항의했던 중국 외교부는 9일 테리 브랜스태드 주중 미국대사를 초치해 체포영장 철회를 요구했다 A day after calling in the Canadian envoy stationed in China for protest, the Chinese foreign ministry summoned U S Ambassador to China Terry Branstad to have the arrest warrant revoked

前一天召见加拿大驻北京大使表示抗议的中国外交部9日召见了美国驻华大使特里·布兰斯泰德,要求美方撤回拘捕令

러위청(樂玉成) 중국 외교부 부부장은 브랜스태드 대사에게 “미국은 중국 시민의 합법적인 권리와 이해를 침해했다”며 “중국은 미국의 행동에 따라 추가적인 조치를 취할 것”이라고 말했다고 중국 외교부는 밝혔다 “The actions of the U S seriously violated the lawful and legitimate rights of the Chinese citizen,” said Vice Foreign Minister Le Yucheng in a statement, adding that Beijing will take additional measures depending on the future actions of Washington

中国外交部表示,中国外交部副部长乐玉成对布兰斯泰德说:“美国侵害了中国市民的合法权益”,“中国将根据美国的行动采取追加措施”

캐나다도 무역사절단의 중국 방문 계획을 취소하며 맞대응에 나섰다 Canada has also made a response, canceling a trade mission’s trip to China

加拿大也取消了贸易使节团的访华计划,开始应对

캐나다 서부 브리티시컬럼비아주는 성명에서 “무역사절단의 방중 계획 취소는 멍 부회장과 관련한 사법적 절차 때문”이라고 밝혔다 The province of British Columbia issued a statement that it has suspended the China leg of its Asian forestry trade mission due to the international judicial process relating to Huawei’s chief financial officer

加拿大西部不列颠哥伦比亚州在声明中表示:“贸易使节团取消访华计划是因为与孟晚舟相关的司法程序”

결국 경제동맹체인 미국과 캐나다가 한 몸처럼 움직이고 있고, 자존심이 강한 중국도 물러서지 않으면서 강대강 대치 국면이 장기화될 가능성이 거론되고 있다 Pundits say that the intransigence between the world’s largest economies is likely to be prolonged as the economic alliance between the U S and Canada is fully aligned, and China is showing no signs of backtracking

结果,属于经济同盟体的美国和加拿大采取了一致行动,而自尊心强的中国也不甘示弱,因此有人提出,强强对峙局面有可能长期持续

AP통신은 중국이 이번 사건에 대한 보복으로 캐나다인을 억류할 가능성이 있다고 전했다 There is a possibility that China could detain Canadians as a retaliatory measure, the AP reported

美联社报道说,中国为了报复此次事件,有可能扣留加拿大人

한편 캐나다에서 체포된 멍 부회장은 자신의 결백을 주장하며 건강 문제를 들어 심리 기간 중 보석을 요청했다 （no translation）

另外,在加拿大被捕的孟晚舟主张自己的清白,以健康问题为由,在审理中申请保释

로이터통신 등에 따르면 멍 부회장은 9일 공개된 법정 진술서에서 “미국 신병 인도에 맞서기 위해 밴쿠버에 체류하고자 하며 인도된다면 미국에서 혐의에 맞서 싸우겠다”고 밝혔다 （no translation）

据路透社等媒体报道,孟晚舟在9日公开的法庭陈述书中表示,“为了对抗引渡美国,将滞留在温哥华,如果被引渡至美国,将在美国与嫌疑作斗争”

이어 “심각한 고혈압과 건강에 대한 우려로 미국 인도 절차가 진행되는 동안 보석금을 내고 석방되기를 바란다”고 말했다 （no translation）

她还表示:“考虑到严重的高血压和健康问题,希望在引渡至美国的过程中,交保释金后释放”

7일 열린 첫 보석 심리에서 캐나다 검찰은 멍 부회장이 대이란 제재 위반 혐의를 받고 있다며 보석 불허를 요청했다 （no translation）

在7日举行的首次保释审理中,加拿大检察机关表示,孟晚舟涉嫌违反对伊朗制裁,要求法院禁止其保释

다음 심리는 10일 재개된다 （no translation）

下一次审理将于10日举行

“한국인들, 중국이 기만할 것 우려해” Chinese scholar argues S Koreans concerned over China’s deception

“韩国人担心中国欺骗韩国”

“한국인들은 중국이 한국을 기만할 것이라고 우려한다 “Chen Xiangyang, an associate researcher of Korea Peninsula Program at the China Institutes of Contemporary International Relations, said South Koreans are concerned that China could deceive them

“韩国人担心中国会欺骗韩国”

” 천샹양(陳向陽) 중국 현대국제관계연구원 한반도연구실 부연구원은 5일 중국 장쑤(江蘇)성 옌청(鹽城)시에서 열린 제6차 한중 공공외교포럼에서 “중국에 대한 한국의 자신감이 좀 떨어져 보인다”며 이같이 주장했다 He made the remark at a South Korea-China public diplomacy forum held on Wednesday in Yancheng, Jiangsu Province in China, claiming that South Korea seems to have “low self-confidence” towards China

中国现代国际关系研究院韩半岛研究室副研究员陈向阳5日在中国江苏省盐城市举行的第6次韩中公共外交论坛上声称,“韩国对中国的自信似乎有所下降”

한중 외교부가 공동 주최하고 한국국제교류재단과 중국공공외교협회가 주관한 이번 포럼은 ‘한중관계 향후 10년의 버팀목이 될 전면적 신뢰 구축’이라는 주제로 4, 5일 개최됐다 Co-hosted by the foreign ministries of the two neighbor countries, the forum took place for two days from Tuesday on full-fledged trust building that would prop up the bilateral ties over the next 10 years, providing an opportunity to peek into inner thoughts of China toward South Korea

由韩中外交部共同主办 韩国国际交流财团和中国公共外交协会主管的此次论坛,以“构建韩中关系未来10年的支柱——全面信任”为主题,于4日和5日举行

한국의 사드(THAAD·고고도미사일방어체계) 배치에 대한 중국의 보복 이후 중국에 대한 부정적 여론이 부쩍 높아진 한국을 바라보는 중국 측의 속내를 엿볼 수 있는 자리였다 South Koreans' resentment against Beijing has significantly escalated since China’s retaliation against the South’s deployment of the U S missile defense system called THAAD

从这次论坛可以看出,在中国对韩国部署萨德系统进行报复后,韩国对中国的负面舆论骤然高涨以及中国方面对韩国的态度

천 부연구원은 5일 “사드가 한중 상호 신뢰에 큰 (부정적) 영향을 줬다”며 “The THAAD has had an enormous (negative) impact on mutual trust between South Korea and China,” Chen said

陈向阳5日主张:“萨德给韩中相互信任带来了巨大的(负面)影响,”

“서로 피해를 주는 마지노선을 넘지 말아야 한다”고 주장했다 “The two sides should not cross the ‘Maginot Line’ of doing damage to each other

“双方不应越过对双方都造成损害的马其诺防线”

이어 “중국은 최선을 다해 한중 협력의 중요성을 강조할 필요가 있고 한국은 한중 협력의 가치를 인식해야 한다”고 덧붙였다 ” He called on Beijing to “do its best” to emphasize the importance of bilateral cooperation, while urging Seoul to recognize the value of the ties

他补充说道:“中国需要尽全力强调韩中合作的重要性,韩国应该认识到韩中合作的价值

자오치정(趙啓正) 전 중국 국무원 신문판공실 주임은 “민족주의 정서가 (한중) 양자관계와 외교에 영향을 준다”며 （no translation）

”中国前国务院新闻办公室主任赵启正表示:“民族主义情绪给(韩中)双边关系和外交产生了影响

“중국의 학자들이 (중국) 청년들에게 영향을 줘 극단적 민족주의를 극복해야 한다 한국도 마찬가지”라고 지적했다 （no translation）

”“中国学者们应向(中国)青年施加影响,克服极端民族主义韩国也一样

중국 측 인사들은 한미동맹 강화가 중국의 국익에 해를 끼친다는 인식도 드러냈다 Chinese presenters also expressed their perception that a stronger South Korea-U S alliance would hurt China’s national interest

”中方人士还表现出了加强韩美同盟有损中国国家利益的认识

천 부연구원은 “한미동맹을 해체하라는 게 아니라 한미동맹이 강화돼 중국에 피해를 줄 것을 우려하는 것”이라고 주장했다 “It is not that China wants South Korea to dismantle the alliance but that it is concerned over a stronger South Korea-U S alliance doing damage to China,” Chen argued

陈向阳认为:“并不是要求解散韩美同盟,而是担心韩美同盟的加强会对中国造成损害

이호철 인천대 중국연구소장은 “한국은 한미동맹을 완화, 축소하거나 철폐하고 한중관계로만 외교정책을 돌릴(집중할) 수 없다”며 “South Korea cannot ease, reduce, or abolish its alliance with the U S to shift (the focus of) its diplomatic policy only on its relations with China,” said Lee Ho-chul, head of the Academy of Chinese Studies at South Korea’s Incheon National University

”仁川大学中国研究所所长李浩哲(音译)指出:“韩国不能集中于缓和 缩小或废除韩美同盟,只将外交政策转向韩中关系”,

“한중 양국이 처한 이런 구조적 조건을 상호 이해하는 바탕에서 한중관계를 이끌어가야 한다”고 지적했다 “South Korea and China should maintain their relations on the basis of mutual understanding of such a structural condition that the two countries are facing”

“应该在理解韩中两国所处的结构性条件的基础上,引领韩中关系的发展”

靑는 “中이 양보”-中은 “핵심이익 수호” U S -China tariffs war truce likely to prove fragile

美国“中国作出了让步”VS中国“捍卫了国家利益”,美中各说各话

다른말도널드 트럼프 미국 대통령이 2일(현지 시간) 트위터에 “중국이 현재 40%인 미국산 자동차에 대한 관세를 삭감·철폐하는 데 동의했다”고 밝혔다 “China has agreed to reduce and remove tariffs on cars coming into China from the US Currently, the tariff is 40 per cent,” U S President Donald Trump wrote on Twitter on Sunday

美国总统特朗普当地时间2日在推特上表示,“中国同意削减或撤销目前针对美国汽车的40%的关税”

1일 미중 정상회담에서 미중 관세전쟁 ‘90일 휴전’에 합의한 뒤 중국이 즉각 미국에 양보하고 나섰음을 강조한 것이다 It appears that his latest remarks are aimed at stressing China’s instant concession after it agreed to take a "90-day truce" from its tariff war with the United States during the summit meeting on Saturday

这是在强调,在12月1日美中首脑会晤上,中美就“90日停战”达成协议后,中国立刻向美国作出了让步

3일 영국 파이낸셜타임스(FT)에 따르면 이달 12일경 류허(劉鶴) 중국 부총리가 협상팀을 이끌고 워싱턴을 방문해 미국과 후속 무역 협상을 벌일 것으로 알려졌다 According to the Financial Times on Monday, China's Vice Premier Liu He will lead his team to visit Washington on December 12 for a follow-up discussion with Washington

据英国《金融时报》3日透露,中国副总理刘鹤率领协商小组将于本月12日访问华盛顿,与美国进行后续贸易谈判

하지만 중국 정부와 관영매체들은 미국 백악관과 달리 ‘협상 시한이 90일이고 그 안에 협상이 타결되지 못하면 미국이 다시 추가 관세를 부과할 것’이라는 사실을 전혀 밝히지 않고 있다 However, the Chinese government and state-run news media outlets are withholding the information from their public that "90 days are given as deadline, and if the two parties fail to strike a deal with the timeframe, the U S will impose tariffs again

但是,中国政府和官方媒体与美国白宫不同,完全没有表明以下事实:“谈判时限为90天,如果不能在谈判期限内达成协议,美国将重新征收追加关税”

오히려 중국 관영매체들은 “중국이 무역 협상과정에서 핵심 이익을 결연히 수호했다”는 점을 강조하고 있다 " If anything, the Chinese media are emphasizing that China has resolutely safeguarded its core interests in the negotiation with the U S

中国官方媒体反而强调,“中国在贸易谈判过程中坚决守护了核心利益”

중국의 이런 태도는 트럼프 대통령이 요구하는 중국 경제의 구조개혁 과정에서 일방적인 양보는 없다고 천명한 것이어서 향후 무역 협상이 가시밭길을 걸을 것이라는 전망이 나온다 Experts say that the following trade talks will face much trouble as Chinas has effectively declared that it will not make any unilateral concessions in meeting the demands from President Trump

中国的这一态度表明,在特朗普总统要求的中国经济结构改革过程中,中国不会单方面作出让步。因此,有预测认为,美中今后的贸易谈判将走过荆棘田

FT도 미중 정상의 합의를 “깨지기 쉬운 관세 휴전”이라고 표현하면서 “후속 협상은 어려움투성이”라고 지적했다 The Financial Times also described the agreement as "fragile tariffs truce," expecting the coming months between Washington and Beijing to be "fraught with pitfalls

《金融时报》也把美中首脑的协议称为“容易破裂的关税休战”,指出“后续谈判充满了困难”

미중 관계 전문가인 왕융(王勇) 베이징(北京)대 교수는 홍콩 사우스차이나모닝포스트(SCMP)에 “중국은 여전히 경제, 특히 첨단기술 분야에서 미국에 의존하지 못하게 되는 최악의 시나리오에 대비해야 한다”고 지적했다 （no translation）

美中关系专家 北京大学教授王勇在香港《南华早报》上指出:“中国仍然必须作好在经济,尤其是尖端技术领域无法依赖美国的最坏准备”

그는 “중국이 (미국 기업의) 시장 접근성과 지식재산권 보호를 확대하는 것을 포함해 산업 정책을 조정하는 것은 가능하지만 경제 구조의 근본적인 변화는 불가능하다”고 말했다 （no translation）

他说:“中国可以调整包括扩大(美国企业的)市场准入性和知识产权保护在内的产业政策,但经济结构不可能发生根本性的变化”

이는 미중이 후속 협상의 영역으로 남겨둔 미국의 중국 경제 구조조정 요구가 시진핑 정권의 권력 유지 및 중국의 국가 주도 사회주의 시장경제 발전과 직결되기 때문이다 （no translation）

这是因为,作为美中后续谈判的领域,美国提出的中国调整经济结构的要求,与习近平政权维持权力及中国由国家主导的社会主义市场经济发展息息相关

미국은 중국의 기술 이전 강요, 지식재산권 침해, 비관세장벽 등 문제 해결 등을 요구하고 있다 （no translation）

美国要求中国解决强迫转让技术 侵犯知识产权 非关税壁垒等问题

이는 중국이 2025년까지 최첨단 기술 제조업 분야에서 미국을 넘어서겠다는 ‘중국 제조 2025’를 무력화시키겠다는 것이다 （no translation）

这意味着,要让中国放弃在2025年之前在高科技制造业领域超越美国的“中国制造2025”

하지만 중국은 미국의 이런 요구를 ‘중국 특색 사회주의 제도’를 인정하지 않고 중국의 주권을 침해하는 불평등 협상이라며 반발하고 있다 （no translation）

但是,中国认为美国的这种要求是不承认“中国特色社会主义制度”,是侵害中国主权的不平等谈判

이 때문에 중국에서도 향후 무역 협상을 낙관할 수 없다는 우려가 나오는 것이다 （no translation）

因此,中国内部也担心今后的贸易谈判不能乐观

미중 정상회담에서의 휴전을 놓고 “백악관 내의 온건파가 득점했다”는 평가도 나온다 " Some pundits point out that the tariffs truce was arranged as the doves have gained ground in Washington

对于美中首脑会晤达成的休战,有人评价称,“白宫内的稳健派得了分”

트럼프 대통령의 곁에서 강경 일변도의 무역정책을 주장하며 대중국 압박을 주도해 온 피터 나바로 백악관 무역·제조업 정책국장의 영향력이 줄어든 반면 타협을 주장해 온 래리 커들로 백악관 국가경제위원회(NEC) 위원장과 스티븐 므누신 재무장관의 입지가 커졌다는 것이다 They say the conciliatory influence from Top White House economic advisor Larry Kudlow and Treasury Secretary Steven Mnuchin has grown against the waning clout of Peter Navarro, the director of Trade and Industrial Policy known for his hawkish economic view towards China

在特朗普总统身边主张强硬一边倒的贸易政策,主导对中国施压的白宫贸易 制造业政策局长皮特·纳瓦罗的影响力减少,而主张妥协的白宫国家经济委员会主席拉里?柯德和财政部长史蒂文·姆努钦占据了上风

인터넷 매체 악시오스는 ‘G20에서 이뤄진 외교의 승리’라는 제목의 기사에서 “이번 담판 결과는 백악관 내 초강경 보호무역주의자들에 대한 글로벌리스트(세계적 관여주의자)들의 승리”라며 “누구도 트럼프 대통령이 시 주석과의 만찬을 준비해올 것으로 생각하지 않았지만, 예상을 뛰어넘어 두 나라의 무역전쟁에 대한 휴전 합의가 이뤄졌다”고 보도했다 In an article titled "A win for diplomacy at the G20," U S -based Internet news site Axios said the result of the agreement was a "score one for the globalists" against hawkish protectionists, adding that "no one thought Donald Trump had prepared for his dinner with Chinese President Xi Jinping, but somehow an agreement was hashed out that calls a 90-day truce to the trade war between the two countries

网络媒体Axios在题为《G20峰会上的外交胜利》的报道中称,“虽然此次谈判结果不是全国主义者对白宫内超强硬的贸易保护主义者的胜利,”“但谁也没有预料特朗普总统准备了与习主席的晚宴,超出预想,两国达成了就贸易战停战的协议”

대만 민진당 선거 참패 뒤엔 中의 경제압박 있었다 " Beijing’s economic pressure affected DPP’s election loss in Taiwan

台湾民进党选举惨败,背后是中国大陆的经济施压“这不是赚了钱之后赞助台湾民进党吗”

“돈 벌어서 대만의 민진당 후원하는 것 아니냐 ” 중국 대륙에서 활동하는 대만 사업가 중에는 이런 얘기를 공개적으로 듣는 경우가 있다고 중국의 한 전문가는 전했다 （no translation）

中国的一位专家表示,在中国大陆活动的台湾企业家中,经常会公开听到这样的话

2016년 1월 독립 성향 민진당의 차이잉원(蔡英文) 총통이 당선된 이후 중국의 민진당 압박 분위기를 보여준다 （no translation）

2016年1月,具有独立倾向的民进党“总统”蔡英文当选后,出现了中国对民进党施压的氛围

지난달 24일 대만 지방선거에서 민진당이 국민당에 참패한 원인을 두고 중국의 경제적 압박도 주요한 요인 중 하나라는 분석이 나온다 Analysts say that Beijing’s economic pressure was one of the key factors in the landslide defeat of the ruling Democratic Progressive Party (DPP) to the rival Kuomintang (KMT) in the November 24 local elections in Taiwan

上个月24日台湾地方选举中民进党惨败于国民党,有分析认为,中国在经济施压也是主要原因之一

이번 선거에서 광역 지방자치단체장 중 민진당 소속은 13명에서 6명으로 줄어든 반면 야당인 국민당 소속 당선자는 6명에서 15명으로 늘었다 While the number of DPP candidates who were elected in the gubernatorial elections declined from 13 to six, that of KMT candidates who were elected increased from six to 15

在此次选举中,广域地方自治团体长中,民进党所属人员从13人减少到6人,相反,在野党国民党所属当选人从6人增至15人

민진당은 20년간 지켜 온 텃밭인 제2 도시 가오슝(高雄) 시장직도 국민당 한궈위(韓國瑜) 후보에게 뺏겼다 DPP also lost the mayoral seat of Kaohsiung, the second largest city and previous DPP stronghold where the party dominated for 20 years, to KMT’s Han Kuo-Yu

民进党还被国民党候选人韩国瑜夺走了守护了20年的第二大城市高雄的市长职位

중국은 차이 총통 집권 이후 대만행 관광객 쿼터를 축소하는가 하면 일부 농산물과 수산물 수출 제한 조치를 내렸다 Since Tsai Ing-wen assumed the presidency in Taiwan, China reduced the quota for Chinese tourists bound for Taiwan and imposed export restrictions on certain agricultural and fisheries products

中国在蔡英文执政后,不仅缩减了前往台湾的游客配额,还采取了限制出口农产品和水产品的措施

대만에서 공급받던 부품을 대륙 내에서 자체 조달하는 이른바 ‘홍색 공급망’의 가동 강화도 중소기업 부품 산업이 주력인 대만 경제에 큰 타격을 줬다 China also strengthened the operation of so-called "red supply chain" through which it self-supplies parts and components that were used to be supplied by Taiwan, dealing a significant blow to the Taiwanese economy that is focused on parts supply by small and medium size companies

大陆自行调配原本由台湾供应的零配件的所谓“红色供应网”的加强运转,也给以中小企业零配件产业为主力的台湾经济带来巨大打击

박한진 KOTRA 중국지역본부장은 30일 동아일보와의 통화에서 “차이 총통 집권 이후 중국은 대만으로 가는 단체관광객 규모를 40% 가까이 줄이는 등 제재를 가했다 관광 분야에서 특히 어려움이 컸다”고 말했다 “Since President Tsai’s inauguration, China has reduced the number of group tourists heading to Taiwan by nearly 40 percent as part of its sanctions Taiwan suffered especially more damage in the tourism industry,” Park Han-jin, chief of the Korea Trade Investment Promotion Agency (KOTRA) Chinese office, said in an interview with The Dong-A Ilbo on Friday

韩国贸易投资振兴公社中国地区本部长朴汉镇30日接受《东亚日报》电话采访时说:“蔡英文执政后,中国对前往台湾的旅游团规模减少了近40%，在旅游领域尤其困难。”

차이 총통 취임 당시 22개국이던 대만의 수교국이 올해 17개국으로 줄어들 만큼 외교적 고립 작전도 강화됐다 Beijing also intensified its strategy for Taipei’s diplomatic isolation, as evidenced by the number of countries that have diplomatic ties with Beijing, which has declined from 22 at the time of Tsai’s inauguration, to 17 this year

中国大陆还加强了外交孤立行动,蔡英文就任时台湾的“建交国”只有22个,到今年已减少至17个

이번 선거에서 민심이 민진당에 등을 돌린 주요인은 민생경제 악화다 The biggest reason voters snubbed DPP in the elections was deterioration of people’s livelihoods

在此次选举中,民心背离民进党的主要原因是民生经济恶化

지난해 총 수출액 중 41. 1%(홍콩 포함)를 차지할 정도로 대만의 중국 대륙에 대한 경제 의존도가 압도적인 상황에서 중국의 민진당 압박이 계속되자 대만 유권자들이 ‘명분보다는 실리’ ‘양안 갈등보다는 안정’을 선택한 것이라는 분석이 나온다 As Taiwan’s economic dependency on the Chinese Economy is so dominant, with China accounting for 41. 1 percent of Taiwan’s total export last year, Taiwanese voters chose "practical interests over justification" and "stability over cross-strait conflict" in the wake of Beijing’s constant pressure on DPP, analysts say

据分析,去年台湾对中国大陆的依赖度占总出口额的41.1%(含香港),台湾对中国大陆呈现绝对性依赖,在这种情况下,中国对民进党持续加压,台湾选民选择了“实利”“稳定”而不是“面子”,选择了“两岸稳定而不是矛盾”

친중 성향 국민당 마잉주(馬英九) 전 총통의 10년 집권 후 대만 유권자들이 민진당의 차이 총통을 선택할 때는 얼마 정도의 양안 갈등도 예상했다 When Taiwanese voters elected Tsai after a 10-year rule by former President pro-Beijing Ma Ying-jeou of KMT, watchers already expected a certain degree of cross-strait conflict

具有亲大陆倾向的国民党前领导人马英九执政10年后,台湾选民选择民进党“总统”蔡英文时,也预测到了两岸之间会出现一定程度的矛盾

차이 정부는 동남아와의 교류 확대 등 ‘신남방 정책’으로 돌파하려고 했다 The Tsai administration sought to overcome this situation through "New Southern Policy" by increasing exchange with Southeast Asia

蔡英文当局试图通过扩大与东南亚的交流等“新南方政策”来突破这一局面

하지만 이번 선거는 “중국과 등을 돌리면 경제가 너무 힘들다”는 것을 보여주는 것이라는 관측이 많다 However, analysts say that the latest elections have demonstrated that Taipei’s opposition to Beijing brings about severe consequences to the Taiwanese economy

但是,很多人分析认为,此次选举意味着“如果与中国背道而驰,经济将非常困难”

지방선거에서 차이 전 총통의 기반 지지층인 청년층 이탈이 심한 것이 이를 잘 보여준다 This has been clearly revealed by the withdrawal of support for DPP by young people, who are a significant part of former President Tsai’s supporters

在地方选举中,蔡英文的基础支持层——青年层的离脱现象非常严重,这充分说明了这一点

강준영 한국외국어대 중국정치경제학과 교수는 “중국에서 취업을 희망하는 청년이 69%에 달한다”며 “Young people who want to land jobs in China account for 69 percent of youth,” said Kang Joon-young, professor of Chinese politics and economy at Hankuk University of Foreign Studies

韩国外国语大学中国政治经济学教授姜俊英(音)解释说:“希望在中国就业的青年达69%”

“대만 내 청년실업 문제가 심각한 상황에서 민진당이 중국과 계속 엇박자를 내자 청년 지지층이 등을 돌린 것”이라고 설명했다 “As DPP continued to be unfriendly with Beijing amid a high youth unemployment rate in Taiwan, younger supporters chose to dump the party

“台湾内部青年失业问题非常严重,在这种情况下,民进党不断与中国步调不一致,导致青年支持层纷纷离开”

올해 3분기(7∼9월) 대만의 청년세대 실업률은 12. 29%로 전체 실업률(3. 76%)을 크게 웃도는 것으로 알려졌다 ” The youth unemployment in Taiwan came to 12. 29 percent in the third quarter of this year, which is far higher than the overall unemployment rate (3. 76 percent)

据悉,今年第3季度(7-9月)台湾的青年一代失业率为12.29%,大幅高于整体失业率(3.76%)

중국 대륙에서 경제활동을 하는 대만인은 150만여 명으로 추산된다 The number of Taiwanese who are engaged in economic activities in the Chinese continent is estimated at more than 1. 5 million

据推算,在中国大陆从事经济活动的台湾人达150多万人

이 중에는 전기·전자 부품 공장 임직원도 많다 Many of them are executives and employees of electronic and electrical parts and components suppliers

其中,电气 电子零部件工厂的员工也很多

페이퍼컴퍼니를 세우거나 통계에 잡히지 않는 방식으로 경제활동을 하는 사람도 적지 않다 A sizable number of people are also engaged in economic activities in China through ways that are not tallied in economic statistics, including operation of paper companies

有不少人建立皮包公司或以不包括在统计中的方式参与经济活动

중국에 진출한 사업가들은 국민당 지지와 집권을 위해 선거 때면 비행기를 타고 대만으로 가 투표를 하고 돌아올 정도로 적극적이다 Business people who are operating businesses in China are so proactive in politics that they even bother to fly to Taiwan to cast ballot during elections to help KMT win elections

进军中国的企业家们为了支持国民党和执政,在选举时会乘坐飞机去台湾投票,非常积极

중국 국무원 대만사무판공실 마샤오광(馬曉光) 대변인은 지난달 28일 기자회견을 열고 “중국과 대만의 도시 차원의 교류가 확대될 것”이라며 “중국 단체관광객이 가오슝으로 향하고 있다”고 말했다 “Exchange at the city level between China and Taiwan will expand,” said Ma Xiaoguang, the spokesman for Beijing's Taiwan Affairs Office, told a press conference on Wednesday

中国国务院台湾事务办公室发言人马晓光上个月28日在记者会上表示:“中国大陆和台湾城市之间的交流将进一步扩大”“大陆旅游团正在前往高雄”

야당이 승리한 지역을 중심으로 교류를 확대하는 등 집권 민진당을 압박하면서도 친중국 지자체에 유화적인 조치가 병행될 수 있음을 시사했다 “Chinese group tourists are heading to Kaohsiung ” He thus indicated that China will seek to pressure the ruling DPP by expanding exchange especially with Taiwanese regions where the opposition party won elections, while concurrently taking reconciliatory measures for pro-Beijing gubernatorial governments

中国大陆在以在野党获胜的地区为中心扩大交流等向执政党民进党施压的同时,也暗示了对亲大陆的地方自治团体采取柔和措施的可能性

中 과학자들 “유전자 편집 아기 강력 규탄”

中国科学家们发表声明“强烈谴责基因编辑婴儿”

성명세계 최초로 유전자를 편집한 아기의 출생에 성공했다는 한 과학자의 주장으로 중국이 논란에 휩싸였다 Chinese researcher claims world's first gene-edited babies

一名科学家声称成功完成世界上第一个使用基因编辑手段产生的婴儿,使中国卷入了争议

중국 당국은 사실관계 조사를 지시했다 Controversy has been stirred up across China by the news that a scientist, reportedly, succeeded in designing a baby, whose genes are biologically edited

中国政府有关部门下令调查事实真相

이 과학자와 관련된 대학 등은 모두 실험과의 연관성을 부인하고 나섰다 Chinese authorities ordered an investigation on the case A university, which the scientist in question belongs, has denied any correlation with the controversial testing

与该科学家有关的大学彻底否认与实验有关

중국인 과학자 허젠쿠이(賀建奎)는 26일 “유전자 편집 기술을 통해 에이즈(AIDS)에 면역력을 갖도록 유전자를 편집했다”며 Chinese scientist He Jiankui argued on Monday that he manipulated genes by using gene editing technology so that they can become immune to AIDS

中国科学家贺建奎26日表示:“通过基因编辑技术,产生了对艾滋病具有免疫力的基因,”

“불임 치료를 받은 부부 7쌍의 배아에 대해 유전자 편집을 진행했고 이 중 한 쌍이 여자 쌍둥이를 출산했다”고 주장했다 The measure was taken on embryos of seven married couples who received infertility treatment, one of which succeeded in giving birth to twin girls, according to the scientist

“对接受不孕治疗的7对夫妇的胚胎进行基因编辑后,从中诞生了一对双胞胎女婴”

유전자 편집은 비정상 유전자를 잘라 내거나 정상 유전자를 삽입하는 것이지만 Editing genes cut some abnormal parts of genes or insert normal ones

基因编辑是切除非正常基因或植入正常基因的手段,

배아 유전자를 편집할 경우 다음 세대에 변형된 유전자가 유전될 수 있어 세계적으로 금지돼 왔다 However, it has been globally prohibited that genes in embryos are edited because the edited genes can be passed down to the next generations

但编辑胚胎基因可能会导致变形的基因遗传到下一代,因此在世界上被禁止

중국인 과학자 122명은 이날 웨이보(중국의 트위터 격)에 “강력히 규탄한다”는 성명을 발표했다 On the same day, 122 Chinese scientists announced their condemnation against Prof

122名中国科学家当天通过微博发表了声明表示“强烈谴责”

이들은 “유전자 편집은 (기술적으로는) 전혀 새로운 게 아니다 He’s argument on Weibo, a Chinese version Twitter They explained that gene editing is not a novel technology from a technological view

他们表示:“基因编辑(在技术上)并不是新技术

(하지만) 불확실성이 존재하고 윤리 문제에서 큰 위험이 존재한다”며 However, uncertainties still exist and the technology can lead to ethical issues and risks, according to the scientists

(但是)它仍存在不确定性,

“엄격한 윤리와 안전성 검사를 거치지 않은 채 (다음 세대로) 유전될 수 있는 태아의 유전자를 편집한 시도에 결연히 반대한다”고 밝혔다 They expressed their strong opposition against such efforts to edit embryos’ genes without any strict ethical and safety considerations, citing that manipulated genes can affect the next generations genetically

在伦理问题上存在很大的危险,”“我们坚决反对在没有进行严格的伦理和安全性检查的情况下,编辑(会向下一代)遗传的胎儿基因的行为”

이들은 “판도라의 상자가 열렸다”며 “만회하지 못하는 단계에 이르기 전에 상자를 닫을 기회가 아직 있다 국가는 엄격하게 감독 관리를 시행해야 한다”고 지적했다 （no translation）

他们说,“潘多拉的盒子已被打开,”“在到达不能回头的阶段之前,还有机会盖上盒子国家应该严格进行监督管理”

중국 국가위생건강위원회는 광둥(廣東)성 위생건강위원회에 실태 조사를 지시했다 （no translation）

中国国家卫生健康委员会向广东省卫生健康委员会下达了调查实际情况的指示

허젠쿠이가 재직 중인 난팡(南方)과학기술대 학술위원회는 “허젠쿠이는 올해 2월부터 무급 휴직 중이며 연구는 대학 바깥에서 진행된 것으로 실험 내용에 대해 알지 못한다”고 말했다 The Academic Council at the Southern University of Science and Technology in Shenzhen, where Prof He belongs, said that he had been on unpaid leave since February The university said that it had been unaware of the test in question as it was conducted outside the campus

贺建奎供职的南方科学技术大学学术委员会表示:“从今年2月开始,贺建奎一直处于停薪留职状态,研究是在大学之外进行的,学校对实验内容一无所知”

中 모래폭풍 덮쳐 China's sandstorms hit Korea with fine and yellow dust

来自中国的沙尘暴从天而降,从微尘到黄沙“让人喘不过气来”

미세먼지에 황사까지 ‘컥컥’27일은 대기 정체로 쌓인 미세먼지에 중국발 스모그와 황사가 겹치면서 숨이 턱턱 막히는 하루였다 Tuesday was indeed a suffocating day in Korea as smog and yellow dust originated in China were added to the accumulated fine dust in the atmosphere

27日,因大气凝滞积累的微尘(可吸入颗粒物),加之源自中国的烟雾和沙尘暴赶在一块,整天都让人喘不过气来

황사가 한반도에 영향을 준 것은 올가을 들어 처음이다 It is the first time this fall yellow dust affected the Korean Peninsula

黄沙袭击韩半岛是今年入秋以来的第一次

기상청과 국립환경과학원에 따르면 서울의 초미세먼지 농도는 이날 오후 3시 현재 m³당 48μg(마이크로그램·1μg은 100만분의 1g)으로 ‘나쁨’ 수준이었다 According to the Korea Meteorological Administration and the National Institute of Environmental Research, the ultra-fine particle concentration level was "bad" with 48μg per m³ as of 3:00 p m Tuesday

据韩国气象厅和国立环境科学院透露,首尔的雾霾浓度截止当天下午3时为每立方米48μg(微克,100万分之1克),属于“恶劣”的水平

경기와 인천은 각각 이날 오전 10시와 낮 12시에 초미세먼지 주의보가 발령됐다 An ultra-fine dust watch was issued at 10:00 a m and 12:00 p m in Gyeonggi Province and Incheon City, respectively

京畿和仁川分别在当天上午10时和中午12时发布雾霾注意警报

주의보는 m³당 75μg 이상인 초미세먼지가 2시간 이상 지속될 때 발령된다 A watch is issued when ultra-fine dust that exceeds 75μg per m³ lasts for more than two hours

警报只有在每立方米75微克以上的雾霾持续2个小时以上时才会发布

23일부터 한반도 상공 대기가 정체되면서 며칠째 국내에서 발생한 미세먼지가 그대로 쌓인 데다 이날 중국으로부터 밀려온 스모그가 더해져 농도가 더 짙어졌다 As the atmosphere of the Korean Peninsula has stagnated since Friday, fine dust generated within Korea has been accumulated, added with smog from China, making find-dust even more concentrated

从23日开始韩半岛上空大气凝滞,连续几天后在国内发生的微尘直接堆积,再加上当天从中国袭来的烟雾,浓度进一步上升

오후에는 황사까지 겹쳤다 To make matters worse, yellow dust also overlapped with the current atmospheric situation

当天下午袭来的沙尘暴则更是雪上加霜

황사가 우리나라에 유입된 것은 5월 25일 이후 6개월 만이다 It has been six months since yellow dust inflowed into Korea after May 25

流入韩国的沙尘暴是自5月25日以后时隔6个月的首次

26일 중국 내몽골 부근에서 발생한 황사 가운데 일부가 북서풍을 타고 서해상으로 밀려 내려와 27일 오후부터 한반도에 영향을 줬다 Part of the yellow dust that was generated near Mongol in China on Monday blew into the Yellow Sea through northwest wind and affected the Korean Peninsula from Tuesday afternoon

26日在中国内蒙古附近发生的沙尘暴中的一部分人乘着西北风降临在西海上,从27日下午开始对韩半岛产生了影响

중국으로부터 우리나라까지 날아온 황사는 대부분 초미세먼지(PM2. 5·지름이 2. 5μm 이하)보다 입자가 큰 미세먼지(PM10·지름 2. 5μm 초과∼10μm 이하)였다 Yellow dust that flew into Korea from China were fine dust of which PM diameter is more than 2. 5μm but less than 10μm, meaning it has larger particles than ultra-fine dust of which the PM 2, 5 diameter is less than 2, 5μm

从中国飞到韩国的黄沙大部分是与细颗粒物(PM2.5,直径2.5微克以下)相比粒子更大的微尘(PM10,直径在2.5微克至10微克中间)

황사의 영향으로 이날 오후 1시 백령도의 미세먼지(PM10) 농도가 m³당 471μg까지 치솟았다 （no translation）

受沙尘暴的影响,当天下午1时白翎岛的微尘(PM10)浓度飙升至每立方米471微克

‘매우 나쁨’(m³당 151μg 이상) 기준을 3배 이상으로 넘어선 것이다 （no translation）

这一数据是“非常恶劣”(每立方米151微克以上)的3倍以上

인천과 서울은 이날 오후 내내 가시거리가 2∼3km로 맑은 날의 5분의 1 수준에 그쳤다 （no translation）

当天下午,首尔和仁川间的可视距离始终只有2~3公里,是晴天的五分之一水平

황사는 28일 아침까지 서울을 비롯한 수도권에 영향을 줄 것으로 보인다 （no translation）

预计,截止到28日早晨为止,沙尘暴将对包括首尔在内的首都地区产生影响

다만 28일에는 전국의 미세먼지가 ‘보통’ 수준을 회복할 것으로 전망된다 （no translation）

到了28日白天,全国的微尘有望恢复至“一般”水平

국립환경과학원은 “황사를 몰고 온 북서풍이 한반도 상공의 미세먼지를 외부로 밀어내 일부 남쪽 지역을 제외하면 미세먼지 농도가 크게 높지 않을 것으로 예측된다”고 말했다 （no translation）

国立环境科学院表示:“携带黄沙的西北风会将韩半岛上空的微尘推到外部,除了南方部分地区之外,微尘浓度将不会大幅上升”

AI 기술 미국 따라잡는 중국… China’s AI ambitions

人工智能技术紧追美国的中国……

‘삼성 반도체’도 잡는 날 오면“그간 (미중 무역전쟁은) 고위급에서 싸우면 그만이라고 생각했어요 하지만 최근 우리에게 영향을 준다고 확실히 느껴집니다 우리 기업들에 압박이 정말 큽니다 무역전쟁이 빨리 끝났으면 좋겠어요 （no translation）

如果“三星半导体”也被追上的那天到来“过去我一直认为(美中贸易战)只会在高层之间开战。但是最近确实感觉到对我们有影响，对我们企业来说压力很大。希望贸易战能尽快结束。”

” 삼성전자가 15일 중국 베이징(北京)에서 연 ‘삼성 미래기술 포럼’ 패널 토론 현장 （no translation）

三星电子15日在北京举行的“三星未来技术论坛”的小组讨论现场

상하이쑤이위안(上海燧原)과학기술유한공사 자오리둥(趙立東) 최고경영자(CEO)가 미중 무역전쟁으로 중국의 인공지능(AI), 반도체 산업이 처한 어려움을 비교적 솔직하게 털어놓았다 During a panel discussion session of the “Samsung Future Tech Forum” held in Beijing on Thursday, Chinese AI maker startup Suiyuan Technology CEO Zhao Lidong frankly talked about the difficulties Chinese AI and semiconductor industries are experiencing due to the trade war between the U S and China

上海燧原科技有限公司首席执行官赵立东比较坦率地吐露了受美中贸易战影响,中国的人工智能 半导体产业所处的困境

자오리둥의 걱정에도 아랑곳없이 미국 상무부 산업안보국은 19일 AI, 로봇공학 등 첨단 기술 14개 항목에 대한 수출 제한 조치를 예고했다 In spite of Zhao’s concerns, the U S Department of Commerce on Monday said it is considering curbs on exports of 14 advanced technologies, including artificial intelligence and robotics

19日,美国商务部产业安全局不顾赵立东的担忧,预告了将对人工智能 机器人工学等14个尖端技术项目限制出口的措施

중국을 거론하지 않았지만 중국의 AI 반도체 굴기(굴起)를 막기 위해 휘두른 칼임이 분명했다 It did not mention China but the move is intended to prevent China’s rise in the semiconductor industry

虽然没有提到中国,但很明显这是为了阻止中国的人工智能和半导体崛起而舞刀动枪

중국도 가만있지 않는다 China has taken an action, too

中国也不会坐视不理

시진핑(習近平) 중국 국가주석은 이달 초 “AI 핵심 기술을 선점, 장악하라”는 주문을 쏟아냈다 Earlier this month, Chinese President Xi Jinping urged innovation in the AI sector, asking the industry to focus on developing core technologies

中国国家主席习近平本月初曾提出“要掌握人工智能的核心技术”

15일 포럼 패널로 나선 중국 바이두(百度)클라우드 셰광쥔(謝廣軍) 부사장은 “현재 미중 간 인공지능은 중국과 미국 모두 시작 단계다 “The AI industry is still in the initial stage both in the U S and China

15日作为嘉宾出席论坛的中国百度云副总经理谢广军表示,“现在美中两国的人工智能都处于起步阶段

중국에 매우 좋은 앞날을 기대한다”며 중국 정부의 지원 등을 중국 AI 산업의 비교우위로 내세웠다 I wish the future will be bright for China,” said Xie Guangjun, vice general manager of Baidu Cloud, during the panel discussion He viewed that China would hold a dominant position in the field thanks to the Chinese government’s strong support

我们期待中国能有一个美好的未来,”指出中国人工智能有政府支援等比较优势

미중 격차가 크지 않기 때문에 미국의 압박에도 문제없다는 자신감을 내비친 것이다 He was confident that China would not be affected by U S pressure as the field is in its nascent stage of development in both countries

这也透露出自信,因为中美差距不大,所以对美国的压力没有感到问题

자오리둥도 “컴퓨터 첨단 반도체는 하루아침에 따라잡지 못하지만 AI는 (미국과) 출발선 사이의 거리가 그리 멀지 않다”고 말했다 “China will not be able to catch up with the leaders in the computer or semiconductor industry But when it comes to AI, both China and the U S are standing in the start line,” said Zhao Lidong

赵立东也表示:“电脑尖端半导体在一夜之间无法跟上,但人工智能距离(美国)起跑线并不遥远”

그는 “5년 전 중국에서 AI 반도체를 얘기하는 건 완전히 허황된 얘기로 들렸지만 지금은 정부의 지지, 자본 시장의 열광적인 성원, 국제적 기술과의 연결 등으로 발전이 가능해졌다”고 강조했다 “AI and semiconductor sounded like building castles in the air for China five years ago But it is not a dream anymore with the government’s support, investment from the capital market, and collaboration with international companies

他说:“5年前在中国人工智能半导体是完全不切实际的说法,但现在由于有政府的支持 资本市场的狂热支持 国际上的技术连接等,发展变为可能”

삼성전자 DS(디바이스솔루션)부문 중국총괄 최철 부사장은 이날 포럼 시작 전 중국의 반도체 굴기에 대해 “우리가 경쟁력이 월등하다고 생각한다”고 말했다 " "I think we are far more competitive than China," Choi Cheol, executive vice president and head of the Device Solutions China regional office at Samsung Electronics, said Thursday about China’s rise in the semiconductor industry

三星电子DS(设备解决方案)部门中国总管副总裁崔哲(音)当天在论坛开始前,就中国的半导体崛起表示:“我认为我们的竞争力非常强”

중국의 메모리반도체 생산 계획에 대해서도 “1등 하는 기업(삼성전자)은 시장을 따라가는 게 아니라 스스로 시장을 만들어낸다”며 자신감을 보였다 “The leader (Samsung Electronics) does not follow the market trend, but it creates a market by itself,” he expressed confidence when talking about China’s plans to mass produce its own memory chips

对于中国的存储器半导体生产计划,他充满自信地说:“第一的企业(三星电子)不是跟随市场,而是自己制造市场”

하지만 다음 날인 16일 중국 국가시장감독관리총국은 삼성전자, SK하이닉스, 마이크론의 메모리반도체 시장 독점 행위에 대한 “대량의 증거 자료를 확보했다”며 제재 가능성을 시사했다 On the following day, however, China’s State Administration for Market Regulation (SAMR) said they found “massive evidence” of anti-competitive behavior by the world’s top three chip makers, Samsung Electronics, SK Hynix, and Micron, suggesting sanctions against them

但是第二天16日,中国国家市场监督管理总局就三星电子 SK海力士 美光垄断存储半导体市场的行为表示,“掌握了大量的证据资料”,暗示了制裁的可能性

세계 최대 반도체 시장인 중국이 한국 반도체 산업을 견제하겠다는 발톱을 드러낸 것이다 China, which has the largest semiconductor market in the world, is making a move to keep the Korean semiconductor industry in check

世界最大半导体市场中国露出了要牵制韩国半导体产业的爪子

미국의 첨단산업 기술 억제에 사력을 다해 맞서려는 중국은 반도체 산업에서도 한국을 뛰어넘기 위해 필사적으로 달려들 것이다 While fighting against the U S ’ export ban on AI, China would make desperate efforts to catch up with Korea in the semiconductor market
(（additional translation））It seemed that Beijing and Chinese tech companies could not wait to see the day when they can say that China has overtaken Samsung in the semiconductor market and it is not a competitor anymore

中国试图用自己的力量对抗美国的尖端产业技术,在半导体产业上也会为了超越韩国而拼尽全力

15일 포럼 토론 막판 샤오미(小米) AI제품부 지쉬(季旭) 사장 등 중국 기업 패널들은 “삼성은 중요한 협력 파트너” “메모리반도체 등에서 삼성의 더 많은 지원과 협력을 희망한다” “AI 산업은 반도체 의존도가 매우 높기 때문에 삼성에 크게 의지한다” 등의 덕담을 건넸다 （no translation）

在15日论坛讨论最后阶段,小米的人工智能产品部总经理小米季旭等中国企业嘉宾们表示:“三星是重要的合作伙伴”,“希望在存储器半导体等领域得到三星更多的支持和合作”,“人工智能因为半导体依存度问,十分倚重三星”

지쉬는 “현재 미중 무역마찰은 삼성에 더욱 큰 기회”라고 말하기도 했다 （no translation）

季旭甚至表示:“现在美中贸易摩擦对三星来说是一个更大的机会”

토론 사회를 맡은 삼성반도체 중국 화베이(華北)지역 어우양지(歐陽基) 부대표는 자오리둥의 무역전쟁 걱정에 “걱정 말라 삼성이 있다”며 농담을 건넸다 （no translation）

担任讨论主持人的三星半导体中国华北地区副代表欧阳基就赵立东对贸易战的担心表示:“不要担心,有三星在”

하지만 포럼에 참석한 첨단기술 중국 기업들과 중국 정부는 “반도체에서 삼성을 제쳤다 이제 삼성은 더 이상 필요 없다”고 말할 날도 하루빨리 오기를 고대하고 있는 듯했다 （no translation）

但是,出席论坛的中国尖端技术企业和中国政府似乎都在期待中国早日赶超三星,“半导体领域超过了三星，现在不再需要三星了”

中 3분기 성장률 6. 5% China's third quarter GDP rises 6. 5 percent year-on-year

中国第三季度增长率为6.5%,创金融危机后最低纪录

금융위기 이후 최저중국의 3분기(7∼9월) 국내총생산(GDP) 성장률이 글로벌 금융위기 이후 최저치를 기록했다 China GDP growth rate hit the lowest point since the Great Recession in the third quarter of the year

中国第三季度(7~9月)国内生产总值(GDP)增长率创下全球金融危机后的最低纪录

중국 국가통계국은 3분기 GDP가 지난해 같은 기간 대비 6. 5% 증가했다고 19일 밝혔다 China’s National Bureau of Statistics said Friday that the country’s GDP expanded 6. 5 percent year-on-year in the third quarter of the year

中国国家统计局19日表示,第三季度GDP同比增长6.5%

이는 글로벌 금융위기를 겪은 2009년 1분기 성장률(6. 4%) 이후 최저치다 The figure is the lowest growth rate since 6. 4 percent that was posted in the first quarter of 2009, when the world was engulfed in the Great Recession

这是经历全球金融危机的2009年第一季度增长率(6.4%)以来的最低值

이로써 중국의 분기별 GDP 성장률은 지난해 1분기 6. 9%를 기록한 이래 하락세를 이어가고 있다 China’s quarterly GDP growth has continued to slide since recording 6. 9 percent in the first quarter of 2017

中国季度GDP增长率从去年第一季度的6.9%起持续下跌

올해 1분기와 2분기에는 각각 6. 8%와 6. 7%를 기록했다 The country’s GDP came to 6. 8 percent and 6. 7 percent, respectively, in the first and second quarters of this year

今年第一季度和第二季度分别为6.8%和6.7%

중국 당국은 1∼3분기 평균 GDP 성장률이 6. 7%이고 올해 목표치가 6. 5%인만큼 종합적으로 볼 때 안정세를 유지하고 있다고 평가했다 Given that the average GDP growth rate over the past three quarters was 6. 7 percent and that this year’s target is 6. 5 percent, its GDP growth continues to be stable in general, the Chinese government says

中国当局认为,1~3季度平均GDP增长率为6.7%,今年的目标值为6.5%,综合来看,维持了稳定趋势

국가통계국은 이날 성명에서 “복합하고 엄중한 국제 정세 속에서도 시진핑 동지를 핵심으로 하는 당 중앙의 영도 하에서 국민경제는 전체적으로 안정적으로 운영되는 가운데 경제 구조 또한 부단히 선진화하고 있다”고 밝혔다 “The national economy has operating on a stable basis overall under the leadership of the Communist Party’s Central Committee led by Comrade Xi Jinping despite complex and grave international politics, while the economic structure is constantly advancing,” the National Bureau of Statistics of China said in a statement on the day

国家统计局当天在声明中说,“在严重复杂的国际形势下,在以习近平同志为核心的党中央的领导下,国民经济整体运营稳定,经济结构也正在不断优化”

이날 기자간담회에서도 마오셩용 국가통계국 대변인(毛盛勇)은 내년 성장 전망에 대해서도 “외부 불확실성이 커졌지만 우리는 외부 압력에 충분히 대응할 수 있을 것으로 보고 있다”고 밝혔다 “External uncertainty has grown, but it is expected that we will sufficiently cope with external pressure,” National Bureau of Statistics spokesman Mao Shengyong also told a press meeting

在当天的记者会上,中国国家统计局发言人毛盛勇就明年经济增长的前景表示:“虽然外部不确定性增大,但我们认为完全能够应对外部压力”

다만 중국 GDP가 하락세를 이어가는 만큼 미중 무역전쟁의 여파가 본격화되는 4분기(10∼12월)부터는 중국 GDP 성장률이 더 큰 타격을 입을 수 있다는 전망도 나온다 Since China’s GDP growth has continued to slump, however, its GDP growth rate will take a bigger hit beginning in this year’s fourth quarter, when the ongoing trade war between the U S and China will start to take its toll in earnest

但是,随着中国GDP持续走下坡路,有分析认为,从美中贸易战争余波全面蔓延的第四季度(10~12月)开始,中国GDP增长率将遭受更大的打击

한편 류허 경제담당 부총리는 이날 인민일보 등 관영 매체와의 인터뷰에서 “현재 미국과 접촉하고 있다”면서 “중미 무역마찰이 시장에 영향을 미쳤으나 솔직히 말해 실제 영향을 미치는 것보다는 심리적인 영향이 크다”고 밝혔다 （no translation）

另一方面,中国主管经济的副总理刘鹤当天在接受《人民日报》等官方媒体的采访时表示:“现在与美国进行接触”,“虽然中美贸易摩擦对市场产生了影响,但说实话,比起对市场产生的影响,心理上的影响更大”

존엄한 국가 흥얼거린 죄 Chinese live-streamer detained for insulting national anthem

哼唱国歌获罪,中国“网红”铁窗行

중 인터넷 스타 철창행중국의 유명 인터넷 스타가 인터넷 개인 방송에서 중국 국가(國歌)를 장난스럽게 흥얼거렸다는 이유로 구류 처분을 받았다 A popular Chinese live-streamer has been detained for singing the national anthem not seriously enough

中国知名“网红”因在网络个人秀节目中开玩笑似地哼唱中国国歌,受到了拘留处分

시진핑(習近平) 국가주석 시대 중국의 언론 인터넷 등 사회 통제가 전방위로 확대되는 단면을 보여준다는 지적이 나온다 Pundits say that the latest episode reflects the expansion of censorship by Chinese President Xi Jinping into Chinese media outlets as well as the Internet

有人认为,这从侧面体现了习近平时代中国对媒体网络等的社会管理正在向全方位扩大

상하이(上海)시 공안국(경찰청)은 14일 인터넷 생방송 진행자인 양카이리(楊凱莉·20)가 중국 국가(國歌)법을 위반해 행정구류 5일 처분을 내렸다고 밝혔다 The Shanghai Police Department said on Sunday that Yang Kaili, a 20-year-old Chinese live-streamer, has been detained for five days for violating China’s national anthem law

上海市公安局14日表示,网络直播主持人杨凯莉因违反《中国国歌法》,5日对她下达了行政拘留处分

상하이 공안은 양카이리가 ‘엄숙히 서서 국가를 불러야 하고 불경한 행위를 하면 안 된다’는 법을 위반했다며 인터넷 방송도 법 적용의 예외 지역이 아니라고 강조했다 The police department said Yang Kaili broke the national anthem law stipulating that one must stand and refrain from disrespectful acts when the anthem is played, stressing that the arena of the Internet is not immune to such law

上海公安部门表示,杨凯莉违反了“应严肃地起立唱国歌,不能对国歌不敬”的国歌法,并强调,因特网直播也不能成为法外之地

인터넷 생방송 진행자인 양카이리는 리거(莉哥)라는 예명으로 알려져 있다 Yang Kaili, who is also known as Li Ge, is a popular live-streamer in China

网络直播主持人杨凯莉以“莉哥”的艺名广为人知

중국의 인기 동영상 앱 더우인('音)에서 팔로어가 4400만 명에 달하는 ‘왕훙(網紅)’이다 왕훙은 중국에서 인터넷 스타를 가리킨다 She is a wanghung, a Chinese expression‎ referring to a major Internet celebrity, with more than 44 million followers on TikTok, a popular streaming app

作为中国知名网红,她在人气视频应用软件“抖音”上有4400万名粉丝

양카이리는 7일 중국판 유튜브인 후야(虎牙)에서 온라인 음악 축제를 소개하는 인터넷 생방송을 시작하면서 앉은 채로 “일어나라, 노예가 되기를 거부하는 인민이여”로 시작하는 중국 국가를 흥얼거리듯 불렀다 The Internet celebrity hummed the national anthem while broadcasting on Huya, a Chinese version of YouTube, to introduce an online music festival on October 7

“网红”在中国是网络明星的代称杨凯莉7日在中国版“优兔”——虎牙上进行介绍在线音乐庆典的网络直播时,坐在椅子上像哼唱一样唱起了“起来不愿做奴隶的人们”开头的国歌

불과 3초 정도밖에 안 되는 시간이었지만 “국가의 존엄을 모욕했다”는 비난이 쏟아졌다 The singing lasted only for three seconds, but she was quickly under fire for insulting the “dignity of the national anthem

虽然只有3秒时间,但很多人指责她“侮辱了国歌的尊严”

이후 양카이리가 “국가를 진지하게 부르지 못해 진심으로 사과한다 조국에도 사과한다”는 사과문을 두 차례 소셜미디어에 올렸지만 논란은 확산됐다 ” Yang issued two apologies on social media, saying that she would like to apologize for singing the national anthem in an unserious manner, and that she was sorry to her motherland as well

之后,杨凯莉两次在社交媒体上发文道歉,表示“没能真诚地演唱国歌,真心道歉向祖国道歉”

후야 측은 양카이리의 방송 계정을 정지시켰다 But the controversy spread, and Huya decided to shut down Yang Kaili’s broadcasting account

虎牙方面停止了杨凯莉的直播账号

결국 양카이리는 사건 발생 일주일 만에 경찰의 구류 처분을 받는 신세가 됐다 Eventually, Yang faced detention a week after the incident

最终,杨凯莉在事件发生一周后,遭到了警方的拘留处分

상하이 공안은 “법률의 마지노선에 도전하고 공공질서와 미풍양속을 위반하는 행위를 법에 따라 결연히 처벌하겠다”고 밝혔다 The Shanghai Police Department said that it would strictly punish acts that challenge the parameters of law and violate the public order and fine custom of the country

上海市公安部门表示:“将依法坚决惩处挑战法律马其诺防线 违反公共秩序和良风美俗的行为”

中 “단둥-평양-서울-부산 연결”…일대일로 한반도 확장 첫 명시 China proposes to expand its Belt and Road Initiative

中国提出“连接丹东-平壤-首尔-釜山”,首次明确“一带一路”

중국이 북-중 접경지역인 랴오닝(遼寧)성 단둥(丹東)을 관문 삼아 일대일로(一帶一路·인프라 투자 등을 통한 중국의 해외 경제영토 확장)를 한반도로 확대하겠다는 계획을 처음 공개했다 China has revealed a plan to expand its “Belt and Road Initiative” (intended to interconnect infrastructure corridors and promote economic growth) with Dandong, a city in Liaoning province that borders North Korea, as a gateway

向韩半岛延伸中国首次公开了把中国-北韩交界地区辽宁省丹东市作为关门 把“一带一路”向韩半岛延伸的计划

랴오닝성 정부는 최근 랴오닝일보가 보도한 ‘랴오닝 일대일로 종합실험구 건설 총체 방안’ 전문에서 “단둥을 관문으로 한반도 내륙으로 연결한다”고 명시해 일대일로가 한반도로 확장된다는 점을 분명히 했다 The province of Liaoning has proposed a plan to the government to “link its city Dandong with the Korean Peninsula” via rail and road as part of its contribution to China’s “Belt and Road Initiative,” according to the official Liaoning Daily

辽宁省政府在最近《辽宁日报》报道的《辽宁“一带一路”综合实验区建设总体方案》全文中明确指出,“以丹东为关门,连接韩半岛内陆,”明确表示“一带一路”向韩半岛延伸

랴오닝성 정부는 이 문건에서 단둥∼평양∼서울∼부산을 철도와 도로, 통신망으로 상호 연결하겠다고 밝혔다 그러면서 이 연결의 성격을 “남부 항구로 직통하는 것”이라고 설명했다 일대일로를 태평양으로 확장하기 위해 부산까지 뻗어나가겠다는 의도를 드러낸 것으로 풀이된다 The province has suggested a rail, road, and communications link from the city of Dandong to the North Korean capital Pyongyang and then on to Seoul and Busan in the South, saying that the link would be a direct route to a port in the south, referring to the city of Busan through which it can expand its plan to the Pacific

辽宁省政府在该文件中表示,将用铁路 公路和通信网把丹东-平壤-首尔-釜山相互连接起来,并解释称,这一连接的性质是“直接通向南部港口”分析认为,这是为了把“一带一路”向太平洋扩张而要延伸到釜山的意图

신의주가 포함된 단둥∼평양 도로 건설 계획도 밝혔다 The province is also pushing for a new road between Dandong and Pyongyang through Sinuiju

方案中还公布了包括新义州在内的丹东-平壤的公路建设计划

문건은 또 신의주와 단둥 사이 압록강의 섬 황금평에 있는 북-중 경제구, 단둥의 북-중 호시(互市)무역구를 단둥 내 중점 개발 개방 실험구와 함께 대북 경제협력의 중요한 지지대로 만들겠다고 명시했다 The document also said that the province will further develop a North Korea-China economic zone in Hwanggumpyong Island and the two countries’ border trade zone in Dandong into the important pillars of economic cooperation with the North along with Dandong’s key development zone for experiments

文件还明确表示,将把位于新义州和丹东之间的鸭绿江岛屿黄金坪的北韩-中国经济区 丹东的北韩-中国互市贸易区,与丹东的重点开发开放实验区一起,打造对北韩经济合作的重要支持区

중앙정부가 적절한 시기에 단둥특구를 건설하도록 노력하고 랴오닝성 선양(瀋陽) 다롄(大連) 단둥 공항과 북한 및 러시아 극동 도시 간 항공편 운항도 강화하기로 했다 Liaoning is also seeking central government approval at due time to establish a special economic zone in Dandong while increasing flight routes between airports in Shenyang, Dalian, Dandong and North Korea and Russia’s Far Eastern cities

中央政府决定,努力在适当时期建设丹东特区,并加强辽宁省沈阳 大连 丹东机场和北韩 俄罗斯远东城市之间的航班

단둥 호시무역구를 국가 간 온라인 전자상거래 플랫폼으로 지원한다는 계획도 공개해 북-중 간 전자상거래가 현실화될 가능성도 커지고 있다 The document also included a plan to support the cross-border trade zone in Dandong as an online e-commerce platform for the two countries

文件还公布了支援丹东互市贸易区成为国家之间网上电子商务平台的计划,中国与北韩之间电子商务成为现实的可能性也越来越大

랴오닝성 정부는 “북-중 양국 지도자의 중요한 합의를 지도로 삼아 대북 협력을 견고하게 계획한다”고 밝혀 일대일로를 한반도로 확장하려는 계획이 시진핑(習近平) 중국 국가주석과 김정은 북한 국무위원장의 3차례 정상회담에서 합의된 내용임을 시사했다 “Guided by the important consensus by the leaders of China and North Korea, (we) steadily make plans of cooperation with North Korea,” said the provincial government, indicating that the initiative’s expansion into the Korean Peninsula had been agreed upon by President Xi Jinping and North Korean leader Kim Jong Un at previous three rounds of bilateral summits

辽宁省政府表示,“把中朝两国领导人的重要协议作为指导,巩固地规划了对北韩合作,”表明把“一带一路”向韩半岛延伸的计划是中国国家主席习近平和金正恩委员长的三次首脑会谈上达成协议的内容

일대일로를 한반도로 확장하겠다는 계획은 랴오닝성을 허브로 하고 중국 한국 북한 일본 러시아 몽골이 협력하는 동북아경제회랑 건설과 함께 추진된다 Beijing’s such plan is pushed alongside the establishment of “Northeast Asian Economic Corridors,” for which China, South and North Korea, Japan, Russia, and Mongolia work together

把“一带一路”延伸至韩半岛的计划,将以辽宁省为枢纽,与中国 韩国 北韩 日本 俄罗斯 蒙古国合作的东北亚经济走廊建设一起推进

랴오닝성 정부는 “중국-러시아-몽골 경제회랑과 한중일+X 모델을 융합해 6개국 협력을 전면 심화하는 동북아 운명공동체를 건설하겠다”고 밝혔다 The Liaoning province said that it will create a group that shares a common destiny in Northeast Asia by converging economic corridors that connect China, Russia, and Mongolia with a framework called the “China-Japan-South Korea plus X model” so that the six countries can enhance cooperation

辽宁省政府表示:“将结合中-俄-蒙经济走廊和韩中日+X模式,建立全面深化六国合作的东北亚命运共同体”

한반도를 포함한 동북아 지역 개발을 랴오닝성을 중심으로 중국이 주도하겠다는 의도를 드러낸 것이다 This embodies the country’s intention to take the lead in the development of the Northeast Asian region with Liaoning as a hub

这体现了中国以辽宁省为中心,主导包括韩半岛在内的东北亚地区开发的意图

한중일+X 모델은 올해 5월 리커창(李克强) 중국 총리가 한중일 정상회의에서 제안한 것으로 한중일 자유무역협정(FTA)과 함께 다른 국가와의 자유무역 협력도 추진하자는 내용이다 The “China-Japan-South Korea plus X model,” first suggested by Chinese Premier Li Keqiang at a trilateral summit in May, calls for free trades with other countries including a free trade agreement between the three countries

韩中日+X模式是今年5月中国总理李克强在韩中日首脑会议上提议的,内容包括推进韩中日自贸协定和与其他国家的自由贸易合作

랴오닝성 정부는 이를 위해 단둥∼훈춘∼러시아 블라디보스토크로 연결되는 철도 건설과 단둥항에서 블라디보스토크항으로 연결되는 해상 통로를 동시에 추진하겠다고 밝혔다 （no translation）

辽宁省政府表示,为此,将同时推进连接丹东-珲春-俄罗斯符拉迪沃斯托克的铁路建设和丹东港至符拉迪沃斯托克港的海上通道

횡으로는 북-중 접경지역을 따라 중국과 러시아를 연결하고, 종으로는 중국과 한반도를 연결하겠다는 것이다 （no translation）

横着要沿着北韩-中国边境地区,连接中国和俄罗斯,纵向要连接中国和韩半岛

문건은 일대일로 동북아 관문의 지위가 두드러지는 시기를 2030년으로 명시해 이번 계획을 2030년까지 완성하는 것이 목표임을 시사했다 （no translation）

文件明确指出,突显东北亚关口地位的时间点是2030年,表示目标是到2030年完成这一计划

시 주석은 12일 블라디보스토크에서 열린 동방경제포럼 연설에서 미국의 일방주의와 대비시킨 ‘동북아경제권’을 주창했다 （no translation）

习近平主席12日在符拉迪沃斯托克举行的东方经济论坛的演讲中倡导了与美国的单方主义形成对比的“东北亚经济圈”

일대일로의 한반도 확장이 미국과의 패권 경쟁을 위한 것임도 배제할 수 없는 것이다 （no translation）

也不能排除“一带一路”向韩半岛延伸是为了与美国争霸

중국의 일대일로 한반도 확장 계획은 문재인 정부의 남북 경제협력 계획인 한반도신경제지도의 서울∼평양∼신의주∼단둥 고속철도 연결 계획과 겹친다 An expanded Belt and Road Initiative of China overlaps with South Korean President Moon Jae-in’s inter-Korean economic policy called “New Economic Map” that plans to connect cities from Seoul to Pyongyang, Sinuiju, and Dandong

中国的“一带一路”向韩半岛延伸计划与文在寅政府的南北经济合作计划——韩半岛新经济地图的首尔-平壤-新义州-丹东高速铁路连接计划相重叠

외교 소식통은 “한국과 중국이 북한 인프라 투자 개발에 대해 협력하는 계기가 될 수도 있지만 중국이 신경제지도를 일대일로에 흡수하려 할 수도 있다”고 지적했다 “This may be an opportunity for South Korea and China to cooperate on the development of North Korea’s infrastructure, but it is also possible that China may try to absorb South Korea’s policy into its own initiative,” said a diplomatic source

外交消息灵通人士指出:“虽然这可能成为韩国和中国就北韩基础设施投资开发进行合作的契机,但中国也有可能试图将新经济地纳入‘一带一路’”

실제 랴오닝성의 철도 연결 계획 등 한반도 확장 계획은 서울과 부산이 포함돼 있음에도 북한과의 협력에서만 강조되고 한국과의 부분에서는 전혀 언급되지 않았다 In fact, while Seoul and Busan are included in Beijing’s plan for expansion, the document only emphasizes cooperation with the North while not mentioning any of it with the South

实际上,辽宁省铁路连接计划等向韩半岛延伸的计划中,就包括首尔和釜山在内,但只强调了与北韩的合作,丝毫没有提及与韩国之间的部分

유엔 안전보장이사회의 대북 제재가 유지되는 상황에서 대북 제재 위반 가능성이 있다는 지적도 나온다 Some say that U N Security Council resolutions on the North Korean regime are likely to be violated in the process of expanding China’s initiative into the Korean Peninsula

有人指出,在联合国安理会维持对北韩制裁的情况下,“一带一路”向韩半岛延伸有可能违反对北制裁

홍콩 사우스차이나모닝포스트(SCMP)는 “제재가 있는 한 중국이 지금 당장 계획을 추진하지는 않을 것”이라고 지적했다 “China was unlikely to push forward any infrastructure projects with the North while sanctions were still in place,” said Hong Kong’s South China Morning Post (SCMP)

香港《南华早报》指出:“只要有制裁,中国就不会马上推进计划”

시진핑 9•9절 방북 사실상 무산 Xi Jinping’s visit to North Korea seems unlikely

习近平事实上不可能“9·9节”访问北韩,王沪宁有可能代替前往

대타 왕후닝 유력북한의 정권수립(9·9절) 70주년을 기념해 시진핑(習近平) 중국 국가주석 대신 왕후닝(王호寧) 당 중앙서기처 서기 겸 정치국 상무위원이 방북할 것으로 3일 알려졌다 Wang Huning, a member of China’s Politburo Standing Committee and secretary of the party’s Secretariat, is set to travel to Pyongyang as President Xi Jinping’s envoy around Sept 9 to celebrate North Korea’s 70th founding anniversary, sources said Monday

记者3日获悉,为纪念北韩政权成立(9·9节)70周年,中共中央政治局常委 书记处书记王沪宁将代替中国国家主席习近平访问北韩

복수의 정부 당국자와 중국 소식통들은 “3, 4일 베이징에서 열리는 ‘중국-아프리카 협력포럼 정상회의에 외교력을 집중하고 있는 시 주석이 9·9절에 맞춰 방북하는 것은 물리적으로 어렵다”며 Multiple government officials and diplomatic sources said that it would be physically difficult for President Xi to make time for a visit to Pyongyang because “for now, the Chinese government appears focused on a China-Africa cooperation forum” being held in Beijing from Monday to Tuesday

多名政府官员和中国消息人士表示:“习主席专心致志于3日-4日在北京举行的中非合作论坛峰会,事实上难以在9·9节访问北韩”,

“북-중 관계를 고려해 영향력 있는 왕 서기를 보내 격을 갖추려 할 것”이라고 말했다 In view of the North Korea-China relationship, the party’s senior official Wang will be sent on behalf of Xi, according to the sources

“考虑到中朝关系,要派有影响力的王(沪宁)书记,才够级别”

이미 지난달쯤 시 주석의 방북이 불투명해진 뒤 왕 서기가 가는 것으로 정리된 분위기였다는 전언도 나온다 Some say that the Chinese government had already decided to dispatch Wang around last month considering President Xi’s busy schedule

也有传言说,习主席北韩之行在上个月就开始变得前景不明,后来就发展为由王(沪宁)书记前往

왕 서기는 북한 문제와 사상·선전을 담당하고 있어 ‘대리 방북’의 적임자라는 평이다 （no translation）

王沪宁负责北韩问题和思想 宣传,因此被认为是“代为访问北韩”的合适人选

올해 세 차례 김정은 북한 국무위원장이 중국을 방문했을 때 모두 영접을 나갔으며 북-중 정상회담에 배석했다 （no translation）

北韩国务委员会委员长金正恩今年三次访问中国时,都是王沪宁出面迎接,并陪同出席北韩-中国首脑会谈

박병광 국가안보전략연구원 책임연구위원은 “왕 서기는 미국 방문학자 경험이 있는 유일한 상무위원으로 향후 대미관계 조율이나 북한의 개혁개방과 관련해 조언할 수 있는 적절한 인물”이라고 말했다 （no translation）

韩国国家安全战略研究院责任研究员朴炳光(音译)表示:“王(沪宁)书记是有访美经验的唯一一名常委,是可以在调整对美关系和为北韩改革开放建言献策的合适人选”

외교가는 시 주석의 방북이 사실상 무산된 데는 도널드 트럼프 미국 대통령의 중국 비난과 경고가 작용했을 것으로 보고 있다 Diplomatic sources speculate that in fact, it was U S President Trump’s condemnation of and warning against China that stopped Xi from visiting the North

外交界认为,习主席平壤之行事实上已经无望,这是美国总统特朗普对中国的谴责和警告起到了作用

김한권 국립외교원 교수는 “미중 무역분쟁이나 대만 문제보다 한반도 문제가 정책 우선순위에서 밀리는 상황인데 “The issue of the Korean Peninsula is not a policy priority for China as it should deal with a trade spat with the United States as well as the issue of China-Taiwan relations

韩国国立外交院金汉权(音译)教授分析称:“韩半岛问题比中美贸易问题和台湾问题在政策优先度上靠后,

시 주석이 굳이 정치적 부담을 지고 긁어 부스럼을 만들 이유가 없다”고 분석했다 So, there is no need for President Xi to make a potentially uncomfortable decision in a political sense and risk borrowing trouble,” said Kim Han-kwon from the Korea National Diplomacy Academy

这种情况下习近平没有必要承担政治负担自找麻烦”

한편 북한이 2월 건군절과 유사한 수준으로 9·9절 열병식을 준비하고 있으며 대륙간탄도미사일(ICBM) 공개 징후도 포착되지 않은 것으로 알려졌다 （no translation）

据悉,北韩正在准备的9·9节阅兵规模大致与2月份建军节相仿,目前尚未捕捉到将公开洲际弹道导弹的征兆

통일부 당국자는 “북한이 열병식에 물자를 쏟아부을 여력이 별로 없는 것으로 보고 있다”고 말했다 （no translation）

韩国统一部高级官员表示:“似乎北韩也没有余力向阅兵式投入大量物资”

中군용기, 한달만에 또 KADIZ 침범 Chinese warplane enters KADIZ again

中国军机时隔一个月再次进入韩国东海防空识别区

중국 군용기 1대가 29일 동해와 남해에서 한국방공식별구역(KADIZ)에 무단으로 진입해 우리 공군 전투기가 대응 출격했다A Chinese military aircraft entered South Korea’s Air Defense Identification Zone (KADIZ) without notice on Wednesday, prompting a South Korean fighter jet to make a sortie in response

一架中国军用飞机29日擅自进入南海和东海上空的韩国防空识别区,韩国空军战机升空应对

중국 군용기의 KADIZ 진입은 지난달 27일 이후 한 달여 만이다 It has been a month since a Chinese warplane entered the KADIZ on July 27 and

这是自上个月27日以来,中国军机时隔一个多月再次进入韩国防空识别区,

올 들어선 5번째다 marks a fifth unauthorized entry this year

也是今年以来的第五次

군 당국에 따르면 이날 오전 7시 37분경 Y-9 정찰기로 추정되는 중국 군용기 1대가 이어도 서남쪽 KADIZ로 처음 진입한 후 대한해협을 따라 동해상으로 이동하면서 KADIZ 진입과 이탈을 반복했다 At around 7:37 a m , a Chinese military jet, presumably a Y-9 reconnaissance aircraft, first entered the KADIZ from southwest of Ieo Island and flied along the Straits of Korea toward the East Sea, entering and exiting it on and off, according to military authorities

据韩国军方透露,当天上午7时37分左右,一架据推测是Y-9侦察机的中国军用飞机进入离于岛西南方的韩国防空识别区,然后沿大韩海峡向东海方向移动,多次进出韩国防空识别区

이어 경북 포항 동북방 약 74km 지점을 거쳐 강원 강릉 동쪽 약 96km 상공까지 북상한 후 기수를 남쪽으로 돌려 진입한 경로를 따라 비행하면서 The Chinese military jet moved northwards to Pohang, flying at an altitude of 74 kilometers, and kept northwards to Gangneung, climbing up to an altitude of 96 kilometers

随后,该机经过庆北浦项东北方向约74公里处,北上至江原道江陵东侧约96公里上空,然后调转机头向南,沿来路飞行,

오전 11시 50분경 KADIZ를 완전히 빠져나갔다 The jet then turned southwards and completely exited the KADIZ at around 11:50 a m

于上午11时50分左右完全脱离韩国防空识别区

군은 F-15K 등 전투기 10여 대를 긴급 출격시켜 중국 군용기에 대해 추적 감시 비행과 경고방송 등 전술 조치를 취했다고 밝혔다 The military said it scrambled 10 aircrafts, including F-15K fighter jets, to track and monitor it and sent a warning message

韩国军方表示,紧急出动了F-15K等10多架战斗机,对中国军机采取跟踪监视飞行和警告广播等战术措施

또 한중 직통망으로 우발적 충돌을 일으킬 수 있는 긴장 고조 행위 중단을 경고했다 In addition, it warned of any actions that could raise tensions and cause accidental conflicts via a South Korea-China hot line

军方还通过韩中直通网,警告中方停止加剧局势紧张 引发偶然性冲突的行为

하지만 중국 군용기는 이를 무시한 채 4시간여 동안 KADIZ에서 비행을 계속했다 But the Chinese jet ignored the warning and stayed inside the KADIZ for four hours

但中国军机置之不理,在韩国防空识别区连续飞行4个多小时

중국 측은 “국제 공역에서 국제법을 위반하지 않고 정상적인 훈련 비행을 한 것”이라고 응답한 것으로 알려졌다 China reportedly said the sortie was an ordinary military operation in international airspace that does not violate international laws

据悉,中方回应称,“在国际空域进行了正常的训练飞行,没有违反国际法”

이어도 주변 공역은 KADIZ를 비롯해 일본과 중국 방공식별구역이 중첩되는 곳이다 The KADIZ around the Ieo Island overlaps with air defense identification zone (ADIZ) of Japan and China

离于岛周边区域,是韩国 日本和中国三国防空识别区重叠的地区

앞서 국방부는 지난달 중국 군용기의 KADIZ 무단 진입 직후 주한 중국국방무관을 초치해 엄중 항의하고, 재발 방지를 촉구한 바 있다 The South Korean Ministry of Defense called in the Chinese defense attaché at the Chinese embassy in Seoul earlier in last month right after a Chinese warplane made an unauthorized entry into the KADIZ to lodge a complaint and urged China to prevent similar incident from happening again

在此之前,韩国国防部曾在上个月中国军机擅自闯入韩国防空识别区后,召见中国国防武官提出严正抗议,并敦促防止再次发生类似事件

군 당국자는 “중국군이 한반도 주변을 정찰하고 정찰기의 장거리 비행 능력을 과시하고자 KADIZ 진입 비행을 반복하는 것으로 보인다”고 말했다 It appears Chinese military is constantly entering the KADIZ in order to reconnoiter the Korean Peninsula and show off the ability of its reconnaissance aircraft to fly long distance

军方高级官员表示:“中国军队为了侦察韩半岛周边地区,并展示侦察机的远程飞行能力,所以反复飞行进入韩国防空识别区”

이란 원유 최대수입국 中, 美제재에 전전긍긍 China anxious due to Trump's Iran sanctions

伊朗原油的最大进口国中国对美国的制裁战战兢兢

이란의 최대 무역 파트너이자 이란 원유 최대 수입국인 중국이 7일부터 재개된 미국의 대(對)이란 경제·금융 제재로 고민에 빠졌다 China, which is Iran’s largest trading partner and largest crude oil importer, is in trouble as the United States resumed economic and financial sanctions against Iran on Tuesday

中国是伊朗最大的贸易伙伴 伊朗原油的最大进口国,从7日起因美国恢复对伊朗实施经济和金融制裁而陷入了烦恼

중국은 미국의 이란 제재에도 이란과 경제무역 관계를 유지하겠다고 밝혔지만 China revealed that it would maintain its economic relationship with Iran despite U S sanctions against the partner country

中国虽然表示,尽管美国对伊朗进行经济制裁,仍将维持与伊朗的经贸关系,

미국과의 무역전쟁으로 적지 않은 타격을 받고 있는 상황에서 또 다른 전선을 형성하는 데 대한 부담으로 전전긍긍하는 모습이다 중국 외교부는 8일 “이란과의 상업 협력은 개방적이고 투명하며 공정합법하다 China, however, appears to be nervous as it is uncomfortable in forming another war while it is already suffering conspicuously from the trade war with the United States

但在因与美国的贸易战争而受到不少打击的情况下,对与美国形成新的战线感到负担 战战兢兢。中国外交部8日强调:“与伊朗的商业合作既开放又透明,是公正合法的

어떤 유엔 안보리 결의도 위반하지 않았다”며 “중국은 일방적 제재와 미국 국내법을 국외에 적용하는 방식을 반대해 왔다”고 강조했다 （no translation）

不违反任何联合国安理会决议,”“中国一向反对单方面制裁和美国在国际上适用国内法的方式”

지난해 중국과 이란의 무역 총액은 370억 달러(약 41조3179억 원)다 （no translation）

去年中国和伊朗的贸易额达到了370亿美元(约413179亿韩元),

전년 대비 20% 증가한 액수다 （no translation）

比前年增加20%

중국은 이란에 대형 댐과 발전소, 교통시설 등 기초 인프라 건설을 위한 대규모 차관도 제공하고 있다 （no translation）

中国还向伊朗提供大坝和发电厂 交通设施等基础设施建设的大规模贷款

11월 발효될 2단계 제재 조치인 이란 원유 거래 차단은 중국에 더 부담을 안길 것으로 보인다 The second sanctions, which block trading Iran’s crude oil from November, will pose additional burden on China

预计将于11月生效的第二阶段制裁措施——阻止伊朗原油交易,将会给中国带来更大负担

중국이 이란으로부터 수입하는 원유는 하루 평균 65만 배럴 The amount of crude oil China imports from Iran is an average of 650,000 barrels per day,

中国从伊朗进口的原油每天平均达65万桶,

현재 시장가치로 150억 달러(약16조7745억 원)어치다 which is worth 15 billion U S dollars in market value

按目前市场价格计算相当于150亿美元(约合167745亿韩元)

중국의 전체 원유 수입량 중 7%가 이란산이다 Some 7 percent of China's overall imported crude oil is from Iran

中国的原油进口量中,7%来自伊朗

이란으로선 수출량의 4분의 1이 넘는다 That is, one-fourth of Iran’s export

对于伊朗来说,对华出口超过了其总出口量的四分之一

중국 국유 석유기업들은 이란 주요 유전에 수십억 달러를 투자한 것으로 알려졌다 Chinese state run oil companies have reportedly invested billions of dollars to Iran’s major oilfields

据悉,中国国有石油企业向伊朗主要油田投资了数十亿美元

미국의 이란 제재가 중국 경제이익을 침해한다는 점에서 중국은 반발할 수밖에 없는 상황이다 It is not a surprise for China to resist U S sanctions against Iran as it violates China’s economic profit

美国对伊制裁侵害了中国的经济利益,中国不得不表示反对

하지만 무역전쟁 과정에서 미국의 고율 관세 부과에 똑같은 규모의 보복 관세로 맞불을 놓는 것과 달리 이란 제재 문제와 관련해서는 중국의 속내가 복잡하다 It is, however, complicating for China in terms of sanctions against Iran, unlike fighting back with retaliatory tariffs with the same scale during the course of a trade war

但与在贸易战争过程中针对美国高额关税征收同等规模的报复关税不同,涉及制裁伊朗问题,中国的想法非常复杂

무역전쟁은 미국의 보호무역주의에 맞서 자유무역 이념을 수호한다는 명분으로 유럽과 다른 아시아 국가 등 국제사회에 공동 대응을 호소할 수 있었다 China was able to appeal for joint response of the international society including Europe and other Asian countries under the name of protecting the ideology of free trade, which is against Trump's protectionism

贸易战争以对抗美国贸易保护主义 维护自由贸易理念的名义,可以向欧盟和其他亚洲国家等国际社会呼吁共同应对

（（additional translation））It is difficult for China to appeal to the international society for help

（增译）但是涉及制裁伊朗,中国很难要求国际社会发出一致声音

（（additional translation））Moreover, U S President Donald Trump wrote on Twitter on Tuesday that anyone who trades with Iran cannot trade with the United States Chinese corporations that engage in transactions with Iran may become the target of U S sanctions

（增译）况且,特朗普总统7日在推特上斩钉截铁地表示:“无论是谁,只要与伊朗进行交易,都无法与美国进行交易”与伊朗进行交易的中国企业可能会成为美国制裁的目标

（（additional translation））China has also been responding sensitively to such secondary boycott with sanctions imposed on North Korea as well European corporations begin halting investments in Iran as Trump reimposed economic sanctions on Iran, which shall also be a burden to China

（增译）中国在对北韩制裁问题上也对上述二级抵制(制裁第三方)方式表现出敏感的反应美国恢复对伊朗制裁之后,欧洲企业中断对伊朗的投资 从伊朗撤走,这种情况对中国而言也是负担

（增译）中国共产党机关报《人民日报》的姐妹纸《环球时报》7日表示,“美国制裁伊朗,在(与中国)贸易战争以外,在(其他领域)点燃了新的火球,”把对伊朗制裁定义为美中矛盾的新因素《环球时报》指出:“包括中国在内,向伊朗投资的国家面临了困境”,“中国很难抵制美国的方式”该报表示,“中国反对美国的单方面制裁(但)围绕伊朗问题,不能随意与美国对立”,“如何维持利益之间的平衡,是中国面临的外交挑战”

中 vs 대만 올림픽 모델 충돌 Taiwan slams China after sports event canceled

中国与台湾就奥运会模式产生冲突

“대만의 일부 정치세력이 2020년 도쿄 올림픽 때 타이완(Taiwan·대만)으로 참가 표기를 하자는 국민투표를 추진하면서 ‘올림픽 모델’에 도전하고 있다 （no translation）

“台湾的一些政治势力正在推动以台湾名义参加2020年东京奥运会的国民投票,对‘奥运会模式’发起挑战”

이 때문에 2019년 (대만) 타이중에서 열릴 예정인 제1회 동아시아청소년경기대회가 정치적 위협과 방해를 받고 있다 （no translation）

因此,将在2019年在(台湾)台中举行的第一届东亚青少年运动会受到了政治性威胁和妨碍

” 중국이 주도하는 동아시아올림픽위원회(EAOC) 회의가 24일 베이징에서 열렸다 （no translation）

由中国主导的东亚奥委会会议24日在北京召开

위원장 류펑(劉鵬)은 타이중시의 대회 개최권 박탈을 주장하면서 위원들의 거수 표결로 개최권 박탈 여부를 결정할 것을 제안했다 At the China-led East Asia Olympic Committee meeting held in Beijing on Tuesday, Committee Chairman Liu Peng insisted on the revocation of Taichung City to host the sports event and asked for a vote of hands to determine the issue

委员长刘鹏主张剥夺台中市的大会主办权,并提议通过委员们的举手表决决定是否剥夺举办权

류펑은 중국 전국인민정치협상회의 외사위원회 부주임을 맡고 있는 중국 측 인사다 Liu is also serving the deputy chief of the Foreign Commission of Chinese People's Political Consultative Conference

刘鹏是担任中国全国人民政治协商会议外事委员会副主任的中方人士

이날 회의에는 위원장 류펑과 중국 대만 한국 북한 일본 몽골 홍콩 마카오의 위원 등 모두 9명이 참석해 있었다 The meeting was attended by nine commission members: representatives from China, Taiwan, South Korea, North Korea, Japan, Mongolia, Hong Kong, Macao and Chairman Liu Peng

刘鹏和来自中国大陆 台湾 韩国 北韩 日本 蒙古 香港 澳门等地的9名委员出席了当天的会议

류 위원장의 발언에 위원들은 술렁였다 The audience began to stir at Liu’s remark

对于刘鹏的发言,委员们议论纷纷

대만 측 위원은 “내년 대회는 대만이 처음으로 올림픽 룰을 적용해 개최하는 대회다 （no translation）

台湾方面的委员抗辩称:“明年的大会是台湾首次适用奥运规则举办的大赛

동아시아 청소년들의 대회 참가 기회를 빼앗지 말라”고 항변했다 （no translation）

不要夺走东亚青少年参加比赛的机会”

일본 측 위원은 “이미 2014년에 타이중시 개최가 확정됐는데 지금 개최권 박탈을 결정하려는 것은 지나치다”는 의견을 냈다 （no translation）

日本方委员提出异议称,“2014年确定由台中市举办,现在决定剥夺其主办权有点过分”

하지만 류 위원장의 주장대로 표결은 이뤄졌고 개최권 박탈에 찬성 7표가 나왔다 However, the vote was carried out and seven voted in favor of cancelling hosting rights

但是,根据刘鹏的主张进行了投票表决,结果有7票赞成剥夺举办权

4년을 준비해온 국제대회가 한순간에 물거품이 되는 순간이었다 It meant that four years of preparation had gone to waste

准备了4年的国际大赛瞬间变成了泡影

이 대회는 내년 8월 24∼31일 열릴 예정이었다 The event was scheduled to take place for eight years from August 24 next year

该大会原计划于明年8月24日至31日举行

대만만 개최권 박탈에 반대표를 던졌고 일본은 기권했다 Only Taiwan voted against the proposal and Japan abstained

只有台湾投票反对剥夺举办权,日本弃权

차이잉원(蔡英文) 대만 총통은 이날 페이스북에 “중국이 정치적인 힘으로 대회 개최권을 난폭하게 박탈했다 Taiwanese President Tsai Ing-wen condemned the decision at a Facebook comment: “China is taking advantage of its political power to violently revoke our right host the event

台湾总统蔡英文当天在脸书上表示反对称:“中国以政治力量粗暴剥夺了大会主办权

대만 국민은 이를 결코 받아들일 수 없다”며 반발했다 The Taiwanese people will not accept the decision

台湾国民绝对不接受这一决定”

라이칭더(賴淸德) 대만 행정원장(총리)은 “EAOC에 정식으로 항의하겠다”고 밝혔다 ” Taiwanese Premier William Lai also said that Taiwan would make an official protest to the EAOC”

台湾行政院长(总理)赖清德表示:“将正式向东亚奥委会提出抗议”

최근 대만에서는 도쿄 올림픽 때 대만이라는 표기로 참가해야 한다는 국민투표 청원운동이 벌어지고 있던 상황이었다 （no translation）

最近在台湾出现了以台湾名义参加东京奥运会的国民投票请愿运动

현재 대만은 올림픽에 ‘차이니즈 타이베이(Chinese Taipei)’라는 이름으로만 참가가 가능하다 （no translation）

目前,台湾只能以“中国台北(ChineseTaipei)”的名义参加奥运会

1968년 멕시코 올림픽까지만 해도 대만으로 참가가 가능했고 대만 국기를 들고 대만 국가도 불렀다 （no translation）

直到1968年墨西哥奥运会,台湾还可以以台湾名义参加奥运会,还拿着台湾国旗,高喊台湾为国家

하지만 1970년대부터 중국의 국제사회 영향력이 커지면서 결국 국제올림픽위원회(IOC)는 1981년 대만이 중국의 일부라는 뜻의 ‘차이니즈 타이베이’라는 명칭으로만 올림픽에 참가할 수 있다고 결정했다 （no translation）

但是,随着从20世纪70年代起中国的国际社会影响力越来越大,国际奥委会在1981年决定,台湾只能以意为中国一部分的“中国台北”的名义参加奥运会

류 위원장이 ‘올림픽 모델에 대한 도전’이라고 언급한 것도 이 때문이다 （no translation）

刘鹏提及“挑战奥运会模式”也是出于这个原因

2016년 차이 총통 집권 이후 대만 정부가 대만이 중국의 일부라는 ‘하나의 중국’ 원칙 수용을 거부해 중국과 대만의 관계가 최악으로 치닫던 상황에서 이번 일로 또 하나의 악재가 터진 것이다 （no translation）

2016年蔡英文执政以后,台湾政府拒绝接受意为台湾是中国一部分的“一个中国”原则,使得中国大陆和台湾之间的关系陷入僵局,这次再次爆发出一个不利消息

“우리는 대만이라고 부르기 시작한다”는 표어를 내걸고 국민투표 청원운동을 주도하던 대만 육상 국가대표 출신 지정(紀政)은 “대만은 민주국가이고 (국민투표를 청원하는) 시민 권리 행사는 법률이 허락한 것”이라며 “개최권 박탈은 받아들일 수 없다”고 말했다 （no translation）

打出“我们开始叫台湾”的标语 主导国民投票请愿运动的台湾田径国家队出身的纪政表示:“台湾是民主国家,法律允许使用(请愿国民投票)公民权利”,“不能接受剥夺举办权”

지정은 대만이란 이름으로 참가한 1968년 멕시코 올림픽에서 동메달을 땄다 （no translation）

纪政曾在以台湾名义参加的1968年墨西哥奥运会上获得铜牌

대만 문제를 이용해 중국을 압박하려는 미국과 중국 간의 갈등도 고조되고 있다 （no translation）

利用台湾问题向中国施压的美国和中国之间的矛盾也愈演愈烈

중국 외교부는 24일 “대만을 중국과 별개 국가로 표기하고 있는 미국 항공사들에 홈페이지 등의 자료 수정을 촉구하라”며 “25일까지 수정되지 않으면 다음 단계 조치를 취하겠다”고 미국 정부에 최후통첩을 했다 （no translation）

中国外交部24日向美国政府发出了最后通牒,要求美方“敦促把台湾和中国标注为不同国家的美国航空公司修正网站资料,如果25日之前不修改,将采取下一阶段措施”

로이터통신은 25일 아메리칸항공, 델타항공, 하와이안항공이 중국의 요구를 수용해 표기를 수정했다고 보도했다 （no translation）

路透社25日报道说,美洲航空 达美航空 夏威夷航空公司接受中国的要求,已经修改了标注

“관료부패가 가짜백신사태 불러” 분노한 중민심 SNS 타고 확산 Chinese people expose fury on social media over the vaccine scandal

“官僚腐败事件引发虚假疫苗事件”……愤怒的中国民心在社交媒体中扩散

“(죄를 지은) 자기가 자기를 조사해 ” “반응이 이렇게 굼떠서 어떻게 지도자를 하나 ” “탐관오리들은 계속 직을 유지하고 장사꾼은 계속 돈을 번다 인민은 계속 고통받는다” 가짜 영유아용 백신 사건이 중국을 강타한 24일 중국 공산당의 부패 조사기관인 중앙기율검사위원회는 가짜 백신을 제조한 창춘창성(長春長生)바이오테크놀로지 소재지인 지린(吉林)성 기율검사위원회가 이 사건 관련 책임자를 조사한다고 밝혔다 China’s Central Commission for Discipline Inspection said Tuesday that the Jilin Provincial Discipline Inspection Commission would launch an investigation into vaccine manufacturer Changchun Changsheng Biotechnology, which caused the latest vaccine scandal in the country

“(犯罪的人)自己调查自己”“反应这样迟钝,领导人是怎么当的”“贪官污吏继续留在位置上,商人继续赚钱老百姓继续受苦”24日,在遭受造假婴幼儿疫苗事件打击的中国,中国共产党的腐败调查机构——中央纪律检查委员会表示,生产造假疫苗的长春长生生物技术公司的所在地吉林省的纪律检查委员会正在调查与该事件相关的责任人

하지만 이를 보도한 중국 신징(新京)보 웨이보(중국의 트위터 격) 계정에 달린 댓글에 나타난 중국 민심은 싸늘했다 However, an account of the Xinjiang News on Weibo, a Chinese platform likened to Twitter, was flooded with widespread anger and distrust since the news broke

但是报道这一消息的中国《新京报》微博账号上出现的回帖中,中国的民心却反应十分冷淡

이 업체가 제조했으나 부적합 판정을 받은 DPT(디프테리아 백일해 파상풍) 백신 25개가 25만 명의 영유아에게 접종됐을 뿐 아니라 충칭(重慶)시에서도 한 업체의 부적합 백일해 백신이 14만여 영유아에게 접종된 사실이 추가로 밝혀졌다 It has been additionally revealed that more than 250,000 doses of the mandatory diphtheria, pertussis and tetanus (DPT) vaccine manufactured by Changchun Changsheng Biotechnology found defective have been administered to children, and the faulty pertussis vaccine manufactured by another company has been also sold for 140,000 children in the city of Chongqing

该公司生产的被判定为不合格的DPT(百白破:百日咳 白喉 破伤风)疫苗接种了25万名婴儿,后来在重庆又发现了某公司的一种不合格疫苗在14万多名婴幼儿中接种

중국 누리꾼들은 “관료들의 말을 듣고 싶지 않아 행동을 보고 싶다” “(부패) 관련 관료가 (조사하는) 너희들보다 직이 높을 것이다” “중앙이 직접 사람을 보내 조사하라 성 지도자는 부패했다” Chinese people have reacted with fury and bluntly exposed their distrust and hostility towards the Chinese authorities, leaving online comments such as “I do not want to hear what officials have to say I want them to act on,” “Related officials (to corruption scandals) may have a higher position than you (investigators),” “The central government should directly send officials to investigate The provincial leaders are corrupt,” and

中国网民说:“不想听官僚们的话想看他们的行动”“贪腐官员的职别比你们(调查人员)还要高”“中央直接派人调查省里的领导是腐败分子”

“사건 발생 후 여러 날이 지났다 여론을 가리지 못한다 책임을 물어야 한다” 등 노골적으로 중국 당국에 대한 불신과 적대감을 드러냈다 “Several days have passed since the incident broke out Public opinions cannot be dismissed, and they should be held responsible”

“事件发生都过了好几天了舆论真假难辨必须追究责任”等,露骨地流露出对中国当局的不信任和敌对感

충칭시의 한 시민은 자녀가 문제의 백신을 접종받은 사실을 안 뒤 바로 인터넷에 “가짜 백신 피해자 권리 수호 조직을 만들자”고 올렸다 （no translation）

重庆市的一名市民在得知子女接种问题疫苗的事实后立即在网上留言说:“要建立‘假疫苗受害者权利保护组织’”

얼마 뒤 공안(경찰)이 그의 집에 들이닥쳐 공안국으로 연행됐고 조사를 받은 뒤 풀려났다 （no translation）

不久后公安(警察)来到了他家,他被带到公安局接受调查后才获释

리커창(李克强) 총리는 물론이고 아프리카 순방 중인 시진핑(習近平) 주석까지 공개적으로 진상 조사를 지시했지만 민심의 분노는 중국 공산당과 정부를 직접 향하고 있는 것이다 Chinese President Xi Jinping, who is currently on trips to Africa, as well as Premier Li Keqiang have ordered a thorough investigation into the scandal, but the public’s anger is also pointing to the country’s Communist Party and the government

不仅是总理李克强,连同正在非洲多国访问的国家主席习近平也公开指示调查真相,但是民心的愤怒直接针对中国共产党和政府

중국 당국이 정부 비판 인터넷 댓글을 대대적으로 삭제하는 동안 소셜미디어가 정부에 대한 분노를 직접 표출하는 핵심 통로로 떠오른 점이 주목된다 It is noteworthy that social media have ironically served as a key channel for people to express their frustration towards the government while the authorities have been working to delete anti-government online comments

中国当局大举删除批评政府的网络留言的过程中,社交媒体成为直接表露对政府愤怒情绪的核心通道,这一点倍受关注

이런 가운데 중국 국가식품의약품감독관리총국 쉬징허(徐景合) 부국장이 가짜 백신 사건과 관련한 관영 중국중앙(CC)TV의 저녁 메인뉴스 신원롄보(新聞聯報) 인터뷰 때 영국산 명품인 버버리 티셔츠를 입은 것이 부적절했다는 비판도 이어지고 있다 Meanwhile, Xu Jinghe, deputy director of China’s Food and Drug Administration, has also come under fire due to his choice of clothes for an interview with the main evening news program of China’s state-run CCTV

在这种情况下,中国国家食品医药品监督管理总局副局长徐景合在接受官方媒体中央电视台“新闻联播”采访时,身穿英国名牌巴宝莉体恤,也遭到批评,认为很不合适

22일 방영된 이 인터뷰에서 그는 3200위안(약 53만 원)짜리로 추정되는 파란색 티셔츠를 입었다 At the interview aired Sunday, he appeared in a blue T-shirt of Burberry, one of the UK’s luxury brands, which is estimated to be priced at 3,200 yuan (around 530,000 won)

在22日播出的这一采访中,他穿了一件被推测为3200元人民币(约合53万韩元)的蓝色体恤

중국 누리꾼들은 “위부터 아래까지 심하게 썩었다”는 비판부터 “집에서 가장 싼 옷일 것”이라는 비아냥까지 분노를 쏟아냈다 Online message boards have been again inundated with sarcastic comments such as “All are seriously rotten from top to bottom” and “That may be the cheapest shirt he owns”

中国网民们愤怒地批评道:“从上到下都严重腐烂”,“这可能是他家里最便宜的衣服”

한 베이징 시민은 본보에 “월급이 많지 않은 공무원이 버버리를 입고 버젓이 관영매체 인터뷰를 했다는 것 자체가 한심하다”고 지적했다 “The fact that a public official who does not have a high monthly salary takes part in an interview of a state-run media wearing a luxurious Burberry shirt itself is disgraceful,” a Beijing citizen pointed out

一名北京市民指出:“工资不高的公务员们居然穿着巴宝莉,堂而皇然地接受官方媒体的采访,这本身就令人寒心”

16일 中-EU 정상, 경제동맹 재편 회담 E U , China to discuss to reshape economic ties in Beijing

中国与欧盟16日举行领导人会晤,会谈重组经济同盟

16일 중국 베이징(北京)에서 열리는 20번째 유럽연합(EU)-중국 정상회담은 역대 어느 때보다 더 끈끈한 분위기를 연출할 것으로 보인다 The 20th E U -China summit will be held in Beijing on Monday and is expected to become the sweetest-ever honeymoon in their relationship

第20届欧盟-中国领导人会晤16日在中国北京举行,预计将会比以往历届峰会演绎出更加沉闷的氛围

이번 정상회담의 핵심 의제는 경제다 Economic issue will be one of key agendas given that the European Union and China alike are struggling in a trade war with the United States

此次会晤的核心议题是经济

미국과 무역전쟁을 치르며 동병상련의 어려움을 겪고 있는 중국과 EU는 도널드 트럼프 미국 행정부의 ‘고립주의와 보호무역’에 맞서 ‘다자주의와 자유무역’의 공통분모를 강조할 예정이다 It is expected that the two sides will put mutual emphasis on multilateralism and free trade as their common ground in their opposition to the Trump administration’s policy direction of isolationism and protectionism

同样与美国进行贸易战 遭受同病相怜之苦的中国和欧盟,将针对美国特朗普政府的“孤立主义和保护贸易”,强调“多边主义和自由贸易”的共同分母

더 급한 쪽은 중국이다 （no translation）

更为急迫的是中国

미국이 500억 달러 규모의 중국산 제품에 25%의 관세를 부과한 데 이어 2000억 달러 규모의 중국산 제품에 추가 관세 10%를 부여하겠다고 밝혔지만 중국은 이에 반격할 마땅한 카드가 없다 （no translation）

美国对500亿美元的中国输美产品征收了25%的关税,接着又表示将对2000亿美元中国输美产品加征10%的关税,但中国没有合适的回应之策

작년 기준 미국의 대중 수출액은 약 1300억 달러에 불과하기 때문이다 （no translation）

因为去年美国的对华出口额只有1300亿美元

중국은 올해가 중-EU 전략적 협력 동반자관계 15주년이 되는 해라는 점을 강조하면서 분위기를 띄우고 있다 （no translation）

中国强调今年是中欧战略合作伙伴关系建立15周年,努力营造气氛

장클로드 융커 EU 집행위원장의 대화 상대인 리커창(李克强) 총리뿐 아니라 시진핑(習近平) 국가주석이 융커 위원장 및 도날트 투스크 EU의회 상임의장과 회견할 것임도 미리 공개했다 （no translation）

不仅是欧盟委员会主席容克的对话伙伴李克强总理,中方还提前公布了中国国家主席习近平主席将会见容克和欧洲理事会主席唐纳德·图斯克的消息

미중 무역전쟁을 미국 대 중국 구도가 아닌 미국 대 세계 구도로 만들려는 게 중국의 속셈이다 During the scheduled summit with its European counterpart, the Chinese government seemingly hopes the current U S -China trade war will grow into a broader face-off between the United States and the rest of the world

中国的算盘是把美中贸易战转变成美国对世界的格局,而不是美国对中国的格局

중국은 이미 지난주 독일이 강력하게 요구해 온 중국 인권운동가 류샤오보(劉曉波)의 부인 류샤(劉霞)를 독일로 보내며 서방에 강력한 구애를 보냈다 Last week, it was mainly a symbolic gesture that the Chinese government sent Liu Xia, the widow of the Chinese human rights activist Liu Xiaobo, to Germany upon its strong requests, in an attempt to win over the hearts of the West

中国上周已将德国强烈要求的中国人权运动家刘晓波的夫人刘霞送往德国,向西方发出了强烈的求爱信

중국의 이런 태도는 EU에 절호의 기회가 될 수 있다 Such a move from the Chinese side could bring a golden opportunity for the European Union during this summit

中国的这种态度可能成为欧盟的绝佳机会

EU도 미국과 무역전쟁을 하고 있지만 중국에 비하면 아직은 대응할 카드가 많다 Even in the trade war with the United States, the European Union still has many more cards up their sleeves than China does

虽然欧盟也与美国在打贸易战,但与中国相比,现在还有很多应对措施

과거 중국의 시장진입 제한, 지식재산권 침해 등에 문제를 제기하며 불만을 나타냈던 EU는 이번 기회를 십분 활용해 중국의 양보를 얻어내려 하고 있다 One of E U strategies is to secure some major concessions from China regarding a series of long-standing issues, about which the European Union has complained,

欧盟过去对中国限制市场准入 侵犯知识产权等问题提出过异议,正充分利用此次机会,争取中国的让步

이미 EU는 자동차와 화학 등에서 중국의 경제적 당근을 받았다 such as China’s market entry restriction and infringement of intellectual property

欧盟已经在汽车和化学等领域从中国得到了甜头

지난주 리 총리는 베를린을 방문해 EU 최대국인 독일의 앙겔라 메르켈 총리와 회담을 갖고 200억 유로의 경제 관련 합의를 했다 （no translation）

李克强总理上周访问欧盟最大国家德国,在柏林与德国总理默克尔举行会谈,双方达成了200亿欧元的经济协议

독일 자동차의 중국 생산 공장 허가뿐만 아니라 독일산 자율 주행차 개발에 도움을 주기로 했다 （no translation）

中国不仅拿出了德国汽车在中国设立生产工厂的许可,还决定对德国开发自动驾驶汽车提供帮助

중국에 수출하는 차도 미국에서 생산하고 있는 독일로서는 중국에 생산공장을 지으면 훨씬 낮은 비용으로 중국 시장에 차를 공급할 수 있게 된다 （no translation）

在美国生产汽车出口中国的德国,如果在中国建厂,就可以以更低的费用向中国市场提供汽车

또 독일 기업 바스프는 중국 기업과 합자를 하지 않고 지분 100%를 유지한 채 광둥(廣東)성에 100억 달러 규모의 화학 공장을 건설하는 내용의 협약을 체결했다 （no translation）

另外,德国企业巴斯夫与中国签订了协议,不与中国企业合资,将以100%控股的方式在广东省建立100亿美元规模的化学工厂

EU는 “정상회담에서 양측은 전략적 관계 확대를 할 예정이며 무역과 투자 분야가 가장 핵심 이슈가 될 것”이라고 밝혔다 The European Union commented that the two sides would expand their strategic relationship and mainly discuss trade and investment

欧盟表示:“峰会上,双方将扩大战略关系,贸易和投资领域将成为最核心的议题”

불붙은 무역전쟁 中 ‘소방수’ 왕치산이 안보인다 U S -China trade war in flames

火热的贸易战争……看不见中方“消防员”王岐山

미중 갈등을 해결할 ‘해결사’로 여겨졌던 왕치산(王岐山·사진) 중국 국가부주석이 미중 무역전쟁이 본격 발발했는데도 나서지 않아 배경이 주목된다 Chinese Vice President Wang Qishan who is thought of as a person to solve conflicts between the United States and China has not responded to the U S -China trade war, garnering much attention of the public as a result

中国国家副主席王岐山(照片)被认为是中美矛盾的“解决者”,但此次中美正式爆发贸易战争时却不见露面,其背景备受关注

왕 부주석이 미국에 가 협상을 벌일 것이라는 관측도 나왔으나 중국의 여러 소식통은 10일 “현재로서는 왕 부주석이 미국에 갈 가능성은 낮다 Some projected that Wang would visit Washington to negotiate, but many Chinese experts say, “The possibility of him visiting the United States is low

虽然有人分析王岐山可能赴美进行谈判,但多名中方消息人士10日表示:“目前王岐山副主席去美国的可能性很低

미중 무역문제 관련 중국 측 협상 대표를 류허(劉鶴) 국무원 부총리가 맡고 있는 상황에서 왕 부주석이 나설 이유가 적다”고 말하고 있다 because he doesn’t have reasons to get involved in the negotiation currently headed by Chinese Vice Premier Liu He

目前由国务院副总理刘鹤担任中美贸易摩擦中方谈判代表,这种情况下,由王岐山出面的理由很少”

올해 3월 전국인민대표대회(전국인대·한국의 국회 격)를 통해 화려하게 복귀한 왕 부주석이 외교·경제 분야 미중 갈등을 조정할 ‘소방대장’으로 기대를 받았다는 점에서 그가 무역전쟁 국면에서 나서지 않는 데는 말하지 못할 사정이 있는 것 아니냐는 관측이 나온다 ” Wang who made a huge comeback at the National People’s Congress in March was expected to put off the fire between the two countries, which makes some to suspect that he has a reason not to get involved in the trade war

在今年3月的全国人民代表大会(相当于韩国的国会)上华丽回归的王岐山被寄予厚望,外界猜测他可能会担任调解外交 经济领域美中矛盾的“消防队长”

왕 부주석은 2009∼2012년 부총리 시절 미중 전략경제대화를 주도하는 등 미국과의 협상 경험이 풍부하고 미국에 상당한 인적 네트워크를 구축한 것으로 알려졌다 He has vast experiences in negotiating with the United States such as leading the U S -China Strategic Economic Dialogue while he served as a vice minister from 2009 to 2012, and also established a broad network in the United States

对于他在贸易战局面下不出面说话,有分析认为其中有难言之隐据悉,王岐山2009年至2012年担任副总理时主导中美战略经济对话,有着丰富的对美谈判经验,在美国拥有相当多的人脉

그는 부주석 임명 전 테리 브랜스태드 주중 미국대사와 스티브 배넌 전 백악관 수석전략가 등 핵심 인사를 잇달아 비공개로 만났다 He had a series of private meetings with U S ambassador to China Terry Branstad and former White House advisor Steve Bannon before he was appointed as vice president

他在被任命副主席之前,曾接连秘密会见美国驻华大使特里·布兰斯塔德和前白宫首席秘书官史蒂夫·班农等核心人士

부주석 임명 이후에도 미국 기업들의 주요 책임자들과 비공개 회담을 해온 것으로 전해졌다 Even after the appointment, he reportedly had many more private meetings with American businessmen

据悉,在出任副主席之后,王岐山还一直与美国企业主要负责人进行非公开会谈

하지만 미국의소리(VOA) 중문판 표현처럼 미중 무역전쟁의 화살이 활시위에 있는 일촉즉발 상황에서 왕 부주석은 보이지 않았다 But he is nowhere to be seen in the imminent trade war, as reported by Voice of America

但正如“美国之音”中文版的描述,在中美贸易战箭在弦上一触即发的情况下,也没有看到王岐山露面

로이터통신은 8일 ‘소방대장 부주석이 미국의 무역전쟁 불길을 피하고 있다’는 제목의 기사에서 고위 서방 외교관을 인용해 “중국이 왕 부주석을 무역전쟁에 투입하기를 원하지 않는 것 같다”고 전했다 Reuters reported that Beijing appeared to be reluctant for Wang to get involved in the article “China’s ‘firefighter’ vice president avoids flames of U S trade war ”

路透社8日在题为《消防队长副主席在避开美国的贸易战火》的报道中,援引一名西方高级外交官的话报道称:“中国似乎不愿意把王岐山投入贸易战”

앞서 류 부총리가 참여한 5월 워싱턴 2차 미중 무역협상에서 양측은 서로 관세를 부과하지 않기로 합의했다 （no translation）

此前,在今年5月刘鹤参与的华盛顿第二次中美贸易协商中,中美双方达成协议,决定互不加征关税

당시 류 부총리는 인터뷰에서 “미중은 무역전쟁을 하지 않을 것”이라고 자신 있게 밝혔으나 불과 10여 일 뒤 백악관은 중국에 대한 관세 부과 계획을 발표했다 （no translation）

当时,刘鹤在接受采访时曾自信地表示:“美中不会进行贸易战争,”但仅仅10多天后,白宫发表了对中国的加征关税计划

이 때문에 류 부총리는 매우 난처한 입장에 처했다 （no translation）

刘鹤因此陷入了尴尬的处境

류 부총리도 시진핑(習近平) 국가주석의 측근으로 통하지만 왕 부주석은 시 주석의 오른팔로 통하는 최측근 중의 최측근이다 （no translation）

刘鹤虽然也是国家主席习近平的亲信,但王岐山被称为习近平的“左膀右臂”,是亲信中的亲信

왕 부주석이 대미 협상에 투입돼 류 부총리처럼 실패하면 이에 대한 비판이 시 주석으로 직접 향할 것을 우려한 중국 당국이 왕 부주석 투입을 꺼리고 있다는 것이다 （no translation）

如果王岐山参与对美磋商并像刘鹤一样失败,由此而引起的批评之声就会直接指向习近平,因此中国政府不愿投入王岐山

한 외교 소식통은 “미중이 언젠가 협상 테이블에 앉겠지만 이번 무역전쟁은 장기전이 될 가능성이 높다”며 “The United States and China will come to the negotiation table one day, but the trade war is likely to be prolonged,” said a diplomacy expert

一名外交消息灵通人士表示:“虽然中美两国将会坐到谈判桌前,但此次贸易战争很有可能会长期化

“무역전쟁 여파로 중국 경제성장이 둔화되고 경제가 침체돼 국민 생활에 영향을 미치면 중국 당국이 고민스러운 상황에 처할 것”이라고 말했다 “Beijing would face a dilemma if the trade war slows down China’s economic growth and people’s lives are affected by it”

如果受贸易战的影响,中国经济增速放缓 经济停滞,国民生活受到影响,中国政府将陷入烦恼的状况”

中의 반격…美반도체 마이크론 판매금지(5판용/교열 안봄) Beijing counterattack begins by banning Micron’s chip sales in China

中国进行回击……禁止美国半导体企业在华销售

중국이 미국 반도체 기업인 마이크론 제품의 자국 내 판매 금지에 나섰다 China has banned Micron from selling its chips in China amid escalating trade tension between China and the United States

中国开始禁止美国芯片生产商美光科技公司在中国销售产品

6일 미국과 관세 부과 발효를 앞두고 전격적으로 나온 이번 결정을 두고 무역전쟁의 신호탄이라는 분석이 나온다 The ban came before the United States is set to impose tariffs on Chinese imported products Saturday

有分析认为,在6日美国加征关税决定生效前夕突然出现的这一决定,是贸易战争的信号弹

현지시간 3일 블룸버그 등에 따르면 중국 푸저우(福州) 법원은 지난 2일 마이크론의 중국 내 판매 금지 예비 명령을 내렸다 The Fuzhou Intermediate People’s Court on Monday ordered Micron to temporarily cease selling its chip products in China, according to Bloomburg

据彭博社当地时间3日报道,中国福州法院于本月2日发布了禁止美光在中国境内销售产品的预告

이 명령은 마이크론이 생산한 D램, 낸드플래시 관련 제품 등 26개 품목에 적용된다 The order covers Micron's 26 chip products, including DRAM and NAND flash memory chips

该决定适用于美光生产的固态盘 内存条 闪存等26种产品

미국에 본사를 둔 마이크론은 지난해 매출의 절반 이상을 중국에서 올렸다 The U S -based chip maker earned more than half of its revenue from China last year

总部设在美国的美光公司去年在中国的销售额占其总销售一半以上

중국 법원의 이번 명령은 마이크론의 경쟁사인 대만 반도체 기업 UMC 발표를 통해 언론에 알려졌다 The injuction order by a Chinese court was revealed by Taiwan-based chip maker and Micron's competition UMC

据悉,中国法院的这一命令是通过美光公司的竞争对手——台湾半导体企业UMC公布的

중국 국유기업 푸젠진화와 손잡고 현지에 D램 생산공장을 짓고 있는 UMC는 지난해부터 마이크론과 특허 침해 및 영업 기밀 등을 놓고 법적 다툼을 벌여왔다 UMC, which is constructing DRAM production facility in partnership with China's state enterprise Fujian Jinhua, has been in a legal battle with Micron since last year over patent and trade secret infringement

与中国国有企业福建晋华联手在当地建设DRAM生产工厂的UMC,从去年起与美光展开了专利侵权和商业机密的诉讼

마이크론은 지난해 12월 UMC 등이 D램 반도체 특허 및 영업비밀을 침해했다며 미국 캘리포니아 주 법원에 소송을 제기했고, In December last year, Micron filed a lawsuit against UMC in the District Court for the Northern District of California, alleging infringement on its DRAM patents and intellectual property rights In response,

去年12月,美光向美国加州法院提起诉讼,声称UMC侵犯DRAM半导体专利及商业机密,

UMC는 올 1월 마이크론을 상대로 중국 지방법원에 맞소송을 냈다 UMC filed patent infringement lawsuits against Micron with the mainland China courts in January this year

UMC则于今年1月向中国地方法院提起了针对美光的诉讼

세계 최대 반도체 시장인 중국의 이번 결정으로 미국과 중국의 무역갈등이 IT분야로 본격화 된 것이라는 해석이 나온다 Some say the sales ban decision made in the largest chip market in the world suggests that the trade war between the United States and China has intensified in the IT field

有分析认为,世界最大半导体市场的中国的这一决定,将使得美国和中国的贸易摩擦大举扩散到IT领域

앞서 미국은 중국 IT업체 화웨이, ZTE, 차이나모바일 등에 제재를 부과했고 미 반도체 업체 퀄컴도 중국의 견제로 네덜란드 반도체 회사 NXP의 인수에 어려움을 겪었다고 블룸버그는 전했다 Bloomberge reported that the United States earlier imposed sanctions against Chinese tech companies, including Huawei, ZTE and China Mobile, and U S mobile chipmaker Qualcomm had difficulty acquiring Dutch semiconductor company NXP as Chinese regulators delayed the approval of acquisition

彭博社报道说,美国此前对中国的IT企业华为 ZTE 中国移动通信等实施了制裁,美国芯片制造商高通也因中国的牵制而在收购荷兰芯片公司NXP遭遇困难

흔들리는 北제재… 美中 점검회의 중단 China becoming a hole in North Korea sanctions

动摇的对北韩制裁……美中会议检查会议已告中断

유엔 안전보장이사회 대북제재 결의를 중국 정부가 제대로 이행하고 있는지 점검하는 미국과 중국의 합동 실무회의가 올해부터 중단된 것으로 전해졌다 （no translation）

据悉,负责检查中国是否如实执行联合国安理会对北韩制裁决议的美中联合工作会议,从今年起就宣告停顿

도널드 트럼프 미 행정부의 한 관계자는 22일(현지 시간) 본보와의 통화에서 “중국 측 사정으로 올해 들어 최근까지 미중 간 실무회의가 열리지 못하고 있다”고 전했다 （no translation）

美国特朗普政府的一名相关人士当地时间22日在接受本报的电话采访时表示:“由于中方的缘故,今年以来中美两国间的工作会议尚未举行”

다른 소식통도 “김정은 국무위원장이 연초 신년사에서 평창 올림픽 참가를 발표한 이후 실무회의가 소집되지 않고 있다”고 말했다 （no translation）

另一名消息人士也表示:“自从金正恩在新年致辞中宣布参加平昌冬奥会之后,就没有召开过工作会议”

트럼프 대통령이 21일 트위터에 U S President Donald Trump disapproved China’s move to ease sanctions against North Korea Monday (local time) on Twitter

有消息称,特朗普总统21日在推特上表示:

“최근 들어 국경에 구멍이 훨씬 더 많이 뚫리고 더 많은 것들이 흘러들어 가고 있다는 소문들이 돌고 있다”며 중국의 대북제재 이행을 공개적으로 촉구한 것도 미중 실무회의 중단과 관련이 있다는 관측이 나온다 （no translation）（（additional translation））
“The word is that recently the Border [between North Korea and China] has become much more porous and more has been filtering in,” he said “I want this to happen, and North Korea to be VERY successful, but only after signing ”

“最近,有传闻说,中国与北韩边境上出现了越来越多的漏洞,越来越多的东西流入北韩,”公开敦促中国履行对朝北韩制裁有分析认为,此举也与中断美中工作会议有关。

대북제재 이행을 집중 점검하는 이 회의는 트럼프 행정부가 유엔 제재에도 중국 국경을 통해 은밀하게 북한과의 거래가 이뤄지면서 제재의 실효성이 떨어진다고 집요하게 문제 제기를 하자 중국 측의 제의로 지난해 초부터 시작됐다 （no translation）

这一会议负责集中检查对北韩制裁决议的执行情况,是因为特朗普政府坚持提出,尽管联合国通过了制裁决议,但通过中国国境仍有人暗中与北韩进行交易,制裁的实际效果低下,因此从去年年初起,根据中方的建议启动

이후 워싱턴과 베이징을 오가며 2, 3개월에 한 번씩 비정기적으로 회의가 열렸다 （no translation）

此后中美双方往返于华盛顿和北京之间,每隔二三个月不定期地举行一次会议

지난해 9월 27일 워싱턴 회의가 마지막이었다 （no translation）

去年9月27日的华盛顿会议是最后一次

유엔 안보리가 대북 수출입을 금지 또는 제한한 정제유와 석탄, 철, 수산물 등에 대해 중국 정부가 구체적인 통계를 제시하고 미국도 위성 관측 등을 통해 확보한 정보를 통해 확인하는 과정을 거치면서 충실한 제재 이행을 뒷받침하는 역할을 해왔다 （no translation）

关于联合国安理会禁止或者限制对北韩进出口的提炼油 煤炭 铁和水产品,中国政府提交了具体的统计报告,美国也通过卫星观测等途径得到的情报进行确认,该会议则发挥了支持忠实执行的作用

북한 대외무역의 95%를 차지하는 중국이 적극적으로 제재에 동참하면서 결국 북한이 대화 무대로 나오게 됐다는 게 미국의 평가였다 （no translation）

美国评价称,占北韩对外贸易95%的中国积极参与制裁,最终使北韩走向了对话的舞台

트럼프 대통령도 기회가 될 때마다 시진핑(習近平) 중국 국가주석의 협조를 평가해 왔다 하지만 한반도 해빙 무드의 영향에 더해 중국 정부가 실무회의에서 빠지면서 다시 대북제재에 구멍이 생기고 있는 게 아니냐는 관측이 나온다 문제는 트럼프 행정부가 중국을 압박해왔던 무역카드를 소진해 버린 시점이어서 대북제재를 강화할 카드가 마땅치 않다는 점이다 He warns that China’s border control should be tighter at least until North Korea agrees to denuclearize Trump mentioned Kim Jong Un’s second visit to China and suspected Chinese President Xi Jinping was behind North Korea’s threat to reconsider the summit with the United States China has been long misunderstood as a “hole” in North Korea sanctions and thus an obstacle of North Korea’s denuclearization

特朗普总统也是每有机会就赞赏中国国家主席习近平的协助。但是有观测认为,受韩半岛解冻气氛的影响以及中国政府缺席工作会议,对北韩制裁可能会出现漏洞问题是,特朗普政府已把对中国施压的贸易筹码用尽,因此没有适合加强对北制裁的筹码

미중은 두 차례의 협상 끝에 최근 관세 부과 유예 등에 합의해 논란을 일단락 지었다 （no translation）（（additional translation））As a result of China participating in the “maximum pressure and sanctions” against North Korea of the Trump administration, however, China should take the credit for bringing North Korea to the negotiation table China has recently been assuaging Pyongyang by increasing North Korea-China exchanges This would also be related to the suspension of the U S -China working level discussions this year, which have been reviewing progresses made in the United Nation’s sanctions against North Korea Pyongyang suddenly took a hardline stance as its relations with China thawed, and threatened to cancel the summit with the United States China would have cajoled the North to engage in brinkmanship or at least is doing nothing about Pyongyang’s brinkmanship tactic Another possibility is that China would have advised Kim to strain relations with the South by cancelling the South-North high-level summit, refusing to admit South Korean journalists at the closing of its nuclear test site, and raising an issue of the South Korea-U S joint military drills China is even more sensitive than North Korea about U S Forces in Korea or forward deployment of U S forces such as unfolding strategic military assets because it looks at issues on the Korean Peninsula from the framework of the hegemony race with the United States in Northeast Asia Thus, China could be trying to put pressure and conciliate North Korea to recover its influence during the process of complete denuclearization, a nuclear-free Korean Peninsula

美中两国在经过两次磋商后,最近就推迟征收关税等问题达成协议,暂时搁置了争议

그동안 거의 매달 대북제재 대상을 발표해 왔던 미 재무부도 2월 이후 석 달째 제재 대상을 발표하지 않고 있다 （no translation）

此前几乎每个月都要公布对北韩制裁对象的美国财政部,也从2月份起连续3个月没有公布制裁对象

美-中, 관세전쟁 중단… 무역협상 타결 South Korean President Moon Jae-in and U S

美与中国停止关税战争……贸易谈判达成一致

중국이 대중국 무역적자를 줄이기 위해 전방위로 압박해온 도널드 트럼프 미국 행정부에 결국 백기를 들었다 President Donald Trump met this morning in Washington D C to discuss measures to prevent North Korea from discontinuing denuclearization (（additional translation））North Korea’s sudden change in posture has raised not only U S -Sino distrust regarding North Korean sanctions but also doubt regarding South Korea-U S cooperative efforts North Korea could wrongly assume that alienating measures were fruitful But as U S Vice President Mike Spence warned, if Kim believes he can play Trump, it is a miscalculation Unless Kim delivers on his promise of “complete denuclearization,” China will not be able to compensate North Korea in any form or shape China must refrain from encouraging North Korea from taking an alternative path or taking any action that can raise suspicions U S ,

中国最终向为减少美国对华贸易赤字全面施压的美国特朗普政府举起了白旗

중국은 미국산 농산물 수입을 늘리고 미국의 지식재산권을 보호하는 무역협상에 합의했다 China agree to abandon trade war Beijing and Washington have agreed to substantially reduce America’s massive trade deficit with China and stop tit-for-tat trade war Beijing pledged to purchase more American goods including agricultural products and strengthen cooperation on protecting intellectual property The United States and China issued a joint statement after delegations led by U S

中美在谈判中达成协议,中国将增加对美国产农产品的进口,并保护美国知识产权

스티븐 므누신 미국 재무장관과 류허(劉鶴) 중국 국무원 부총리가 각각 이끄는 미중 경제·무역대표단은 17, 18일(현지 시간) 워싱턴에서 2차 무역협상을 마무리하고 19일 합의 내용을 발표했다 Treasury Secretary Steven Mnuchin and Chinese Vice-Premier Liu He had the second round of talks last week

由美国财政部长姆努钦 中国国务院副总理刘鹤分别率领的美中经济贸易代表团,当地时间17日~18日在华盛顿举行第二轮贸易磋商,并于19日公布了协议内容

양국은 연간 3750억 달러(약 406조 원·지난해 미국 측 통계 기준)에 이르는 대중 무역적자를 절반 수준으로 줄이고, 중국의 지식재산권 침해를 막기 위한 제도적 정비를 하기로 합의했다 The two sides agreed to reduce America’s trade deficit with china which hit 372. 5 billion dollars last year and examine institutional measures to prevent China’s infringement on U S intellectual property rights

两国一致同意强化制度,把每年达到3750亿美元(约合406万亿韩元,去年美方统计基准)的美国对华贸易赤字减少一半,并防止中国侵犯知识产权

양국 대표단은 공동성명에서 “중국의 대미 상품수지 흑자를 상당 부분 줄이기 위한 조치를 하자는 데 공감대를 이뤘다”면서 "There was a consensus on taking effective measures to substantially reduce the United States trade deficit in goods with China," the joint statement said

两国代表团在共同声明中表示:“双方就为减少中国对美商品收支顺差达成共识,”“为此,中国将增加对美国商品和服务的购买”

“이를 위해 중국은 미국의 상품·서비스 구매를 늘릴 예정”이라고 밝혔다 "To this end, China will significantly increase purchases of U S goods and services

协议表示,美国扩大出口的项目,

미국의 수출확대 품목으로는 트럼프 대통령의 정치적 기반 지역에서 주로 생산되는 농산물을 비롯해 자동차와 에너지 제품이 명시됐다 " Agricultural products, which are mainly produced in areas of Trump’s strong support base, automotive and energy products,

主要是特朗普总统政治基础地区所生产的农产品以及汽车和能源产品

앞서 논의된 품목 중 항공기 반도체 등은 목록에서 빠졌다 구체적인 무역흑자 축소 목표액은 공동성명에 담기지 않았다 are included in the list of items for increasing U S exports Semiconductors and aircrafts were not included, although they were discussed

减少具体贸易顺差的目标值,并没有出现在共同声明中

미국은 연간 2000억 달러 감축을 명기하자고 했지만 중국이 거부한 것으로 알려졌다 The statement provided no dollar amounts on how much China would increase its purchase of American products The United States reportedly offered to specify the amount of 200 billion dollars, but China is known to have refused to do so

据悉,美国提议写明每年减少2000亿美元,但遭到中国拒绝

이번 합의에도 불구하고 중국의 대미 무역 흑자가 큰 폭으로 줄어들기는 쉽지 않을 것이란 지적이 나온다 However, economists say the agreement would not practically help reduce America’s trade deficit,

也有人指出,即使达成这一协议,但中国大幅减少对美贸易顺差并不容易

소비대국 미국과 값싼 노동력을 기반으로 전 세계의 생산기지 역할을 하는 중국의 경제구조에 차이가 있어 양국의 무역 불균형은 근본적인 해법을 찾기 어렵다는 것이다 citing that the multi-billion-dollar trade imbalance stems from the different economic structure with the United States being the world’s largest consumer and China being the world’s factory on the strength of its cheap labor costs

据分析,以消费大国美国和廉价劳动力为基础,发挥全世界生产基地作用的中国经济结构有差异,很难找到从根本上解决两国贸易不均衡的办法

특히 저가 농산물과 에너지 제품의 대중 수출을 늘리는 방식으로는 한계가 있다는 평가가 나온다 There is skepticism about how much the imbalance will be reduced by merely increasing China’s purchase of American goods such as agricultural and energy products

特别是有评论认为,以增加低价农产品和能源产品对华出口的方式,作用有限

뉴욕타임스는 “콩류인 대두에서 50억 달러, 천연가스 석탄 원유 등에서 90억 달러 정도의 수출 증가가 가능할 것”이라며 According to The New York Times, there could be 5 billion-dollar increase in soybeans and 9 billion-dollar increase in liquefied natural gas, oil and coal

《纽约时报》分析说:“豆类大豆增加50亿美元,天然气 煤炭 原油等方面增加90亿美元的出口,是有可能的

“고가의 최첨단 정보기술(IT) 제품과 항공기 및 방위산업 제품을 포함하더라도 트럼프 행정부의 목표치인 연간 2000억 달러의 적자 감축은 비현실적”이라고 분석했다 However, even if the advanced technological and defense industrial products are included, it would add up to only a small fraction of the 200 billion-dollar decrease total, and these are unrealistic numbers

即使包括高价的最尖端信息技术产品和飞机 军火,减少特朗普政府的目标值2000亿美元的逆差,是不现实的”

류 부총리는 “이번 무역협상의 최대 성과는 양측이 무역전쟁을 하지 않기로 했고 상호 관세 부과를 중지하기로 합의한 것”이라고 밝혔다 （no translation）

刘鹤表示:“这次贸易磋商的最大成果,是双方达成协议,不打贸易战,并停止相互征收关税”

공동성명에는 포함되지 않았지만 미중이 관세폭탄 전쟁을 끝내기로 했음을 밝힌 것이다 （no translation）

其意思是,虽然没有包括在共同声明内,但美中决定结束关税炸弹之战

중국 매체들은 이번 합의가 미국에 백기를 든 것으로 비치는 걸 막기 위해 “무역전쟁을 끝냈다 （no translation）

中国媒体为了防止显得此次协议是中国向美国举白旗,强调了“贸易战结束了”

” “공동 승리”라는 표현 등을 강조했다 （no translation）

“共同胜利”等用词

中첫 국산 항모 시험항해 China puts its first natively-built air carrier to sea

中国第一艘国产航母试航……摆脱沿岸前往远洋的“军事崛起”

연안 벗어나 원양으로 ‘군사 굴기’중국의 첫 국산 항공모함이자 두 번째 항모인 산둥(山東)함이 8일 랴오닝(遼寧)성 다롄(大連)에서 시험 항해를 시작한 것으로 알려졌다 The Shandong, China’s first domestically built aircraft carrier and the country’s second aircraft carrier, reportedly sailed Tuesday on a sea trial in Dalian, Liaoning Province

据悉,中国首艘国产航母 也是第二艘航母——“山东”号8日在辽宁省大连开始试航

시진핑(習近平) 중국 국가주석의 전용기와 김정은 북한 국무위원장의 전용기가 다롄 공항에서 목격돼 두 지도자가 역사적인 시험 항해를 지켜본 것으로 관측된다 It appears the leaders of China and North Korea watched the historic sea trial together as their private jets were spotted at the Dalian Airport

人们在机场看到了中国国家主席习近平的专机和北韩国务委员会委员长金正恩的专机,有分析认为,两位领导人观看了中国国产航母的历史性试航

앞서 랴오닝성 해사국은 4∼11일 보하이(渤海) 해역과 서해 북부 해역에서 군사 임무가 펼쳐진다며 선박 진입을 금지한다고 공고했다 Earlier, the Liaoning Maritime Safety Administration announced a ban on all ship entry saying there will be a military mission taking place in the Bohai sea and northern part of the Yellow Sea

此前,辽宁省海事局发布公告说,从4日至11日将在渤海湾海域展开军事任务,禁止船舶进入

중국 해군이 랴오닝함에 이어 산둥함까지 확보함에 따라 본토 해안선에서 수천 km 떨어진 곳에서도 군사 작전이 가능해졌고 해외에서 미국에 맞서 자국의 전략 이익을 도모할 능력을 갖게 됐다는 평가가 나온다 Two aircraft carriersㅡLiaoning and Shandongㅡmade it possible for China to conduct military operations at places thousands of kilometers away from its mainland coastline Also, it acquired the capability to pursue strategic interest in the face of the United States

有评价称,中国海军随着继辽宁舰之后拥有山东舰,在离本土海岸线数千公里的地方也可以展开军事行动,拥有了在海外对抗美国 寻求本国战略利益的能力

중국의 2개 항모 보유는 근해 연안 방어에 치중했던 중국이 원양 해군으로 나가는 데 중요한 이정표가 될 것으로 보인다 The possession of two aircraft carriers will be an important milestone for the Chinese Navy, which focused more on offshore defense, in becoming a Navy capable of conducting operations out in the open ocean beyond offshore areas

中国拥有两艘航母,预计将使原本致力于近海沿岸防御的中国海军走向远洋海军的重要里程碑

중국은 항모를 6척까지 늘릴 계획이다 China plans to increase the number of aircraft carriers to six

中国计划把航母的数量增至6艘

○ 베일 벗은 산둥함의 전투능력 중국은 우크라이나에서 들여온 구소련 항모를 개조해 2012년 5만5000t급 랴오닝함을 진수했다 China put their first 55,000-ton aircraft carrier, the Liaoning, which was purchased as an unfinished hulk from Ukraine, into active service in 2012

○掀开面纱的山东舰的战斗能力中国对从乌克兰引进的旧苏联航母进行改造,55000吨级的辽宁舰于2012年下水

이를 통해 축적한 기술력을 바탕으로 만재배수량이 더 커진 산둥함(7만 t)을 만들어냈다 China could build the 70,000-ton Shandong, which has bigger full load displacement, based on the technologies accumulated from building Liaoning

通过以此积累的技术力量,中国生产了满载排水量更大的山东舰(7万吨级)

모듈식 조립 방식으로 건조된 산둥함은 2013년 11월부터 건조에 착수해 지난해 4월 진수됐다 The Shandong, which was built on modular assembly technology, started its construction in November 2013 and was first launched in April 2018

山东舰采用模块组建方式建造,从2013年11月着手建造,去年4月份下水

시험 항해를 마친 뒤 내년에 실전 배치될 예정이다 It will be put into active service in 2019

预计在完成试航后,将于明年投入实战部署

중국의 항모 이름은 바다에 접한 성의 이름을 순서대로 채용하며 산둥함 이후 진수되는 항모의 이름은 장쑤(江蘇)가 될 것으로 보인다 Chinese aircraft carriers are named after the name of the province adjacent to the sea The next aircraft carrier is likely to be named Jiangsu

中国航母的名称以临海的省份命名,预计“山东”号之后的航母将被命名为“江苏”号

디젤 추진 항모인 산둥함은 길이 315m, 너비 75m에 최대 속도 31노트를 낼 수 있다 （no translation）

“山东”号是柴油动力航母,长315m 宽75米,最大航速为31节

스키점프 방식으로 이륙하는 젠(殲)-15 함재기 40대를 탑재 가능하다 （no translation）

该舰可以搭载40架滑跃式起飞的歼-15舰载机

24대의 함재기를 탑재하는 랴오닝함에 비해 길이가 10m 정도 늘어났지만, 함재기는 16대나 더 실을 수 있어 중국이 그동안 상당한 능력의 최적화된 항모 설계 기술을 연마했음을 보여준다 （no translation）

与搭载24架舰载机的辽宁舰相比,山东舰长度增加了10米左右,但舰载机数量增加了16架,可见中国在这段时间里研发出了能力最适化的航母设计技术

이륙 램프 경사도는 12도로 최고 14도인 랴오닝함보다 낮아졌다 （no translation）

起飞甲板的倾斜度为12度,比最大14度的辽宁舰略有下降

랴오닝함에 비해 함재기 이륙 거리 감축, 연료 절약, 무기 적재량 증가, 항모 구조 강화 등에서 많은 개선이 이뤄진 것으로 알려졌다 （no translation）

据悉,与辽宁舰相比,山东舰舰载机在缩短起飞距离 节约燃料 增加武器装载量 强化航母结构等方面取得了诸多改善

산둥함에는 대형 안테나 4개와 주변을 360도 감지해 해상 또는 공중 목표물 수십 개를 포착할 수 있는 S밴드 레이더가 탑재됐으며 수십 기의 중국산 단거리, 중거리 미사일이 실려 있다 The Shandong is equipped with four large antennas and an S-band radar that can detect dozens of maritime or aerial targets and is carrying dozens of homegrown short and mid-range missiles

山东舰上安装了4个大型天线和可以探测周边360度的海上或空中目标的S波段雷达,装载了数十枚中国产短程导弹和中程导弹

다만 함재기를 발진시킬 때 미국 항모가 사용하는 전자식 사출 방식이 아닌 증기 사출 방식을 채택하고 있다 （no translation）

不过,在启动舰载机时,采用的并不是美国航母使用的电子式喷射方式,而是蒸汽喷射方式

증기 사출 방식이나 스키점프 이륙 방식은 전자식에 비해 함재기의 이륙 거리가 많이 필요하다 （no translation）

相比电子式喷射方式,蒸汽喷射和滑跃方式的起飞,需要更长的距离

또 비행기의 무게를 줄여야 하기 때문에 많은 무기를 탑재하기 어려우며, 결과적으로 작전 능력이 제약된다 （no translation）

另外,由于需要减轻飞机重量,所以很难装载很多武器,最终导致其作战能力受到制约

핵추진 항모가 아니기 때문에 며칠에 한 번씩 급유를 받아야 한다는 점도 약점으로 꼽힌다 （no translation）

因为不是核动力航母,所以每隔几天就要加一次油,这也是一大弱点

대양 작전을 벌이려면 방어능력이 취약한 여러 대의 대형 급유선을 함께 거느리고 움직여야 하기 때문이다 （no translation）

因为如果要开展远洋作战,就要率领多艘防御能力薄弱的大型供油船一同行动

○ 원양엔 나갔어도, 미국 넘긴 역부족 중국 해군은 그간 연해 방어 위주의 ‘황수(黃水·yellow water) 해군’에서 영해와 영토를 수호하는 ‘녹수(綠水·green water) 해군’에 이어 에너지 수송 노선을 수호하는 ‘남수(藍水·blue water) 해군’을 추구했다 （no translation）

○远涉重洋,但仍不敌美国中国海军此前从以沿海防御为主的“黄海海军”起步,后来发展成为守卫领海和领土的“绿海海军”,后来又寻求成为守护能源运输路线的“蓝海海军”

따라서 항모 전단들은 주로 인도양, 서태평양 중심으로 활동하면서 ‘일대일로(一帶一路·21세기 육상과 해상 실크로드 프로젝트)’ 가운데 ‘일로’ 구상을 지원하는 역할을 수행할 것으로 예상된다 （no translation）

预计中国航母编队主要是以印度洋 西太平洋为中心进行活动,并发挥支援“一带一路”(丝绸之路经济带和21世纪海上丝绸之路)中“一路”的作用

그러나 경우에 따라 한국과 일본의 전력을 견제하는 역할도 맡게 될 것으로 보여 동아시아 해양질서에 큰 변화가 생길 수밖에 없다 （no translation）

但是,根据具体情况,也将起到牵制韩国和日本的作用,因此必然将导致东亚海洋秩序发生巨大变化

중국이 향후 3, 4척의 항모를 더 확보하고 핵 항모까지 손에 넣는다면 명실상부한 대양 해군 능력을 갖추게 된다 China will have a true ocean navy once it owns nuclear aircraft carriers as well as three to four more aircraft carriers

如果中国今后再拥有三四艘航母,就将拥有名副其实的大洋海军的能力

그러면 각종 국제 현안에 대한 중국의 개입도 늘어나게 될 것으로 보인다 Then, there will likely be more intervention by China in various international issues

预计,中国对各种国际悬案的介入也将有所增加

중국 해군이 비약적인 성장에도 불구하고 아시아·태평양 지역 내에만 4개의 항모전단을 운용하고 있으며, 전 세계적으로 10개의 항모전단을 보유한 미 해군력에 비해선 양적, 질적 수준 차이가 크다 （no translation）

尽管中国海军飞速成长,但与仅在亚太地区就拥有4个航母编队 在全球范围内拥有10个航母编队的美国海军相比,其数量和质量仍有很大差距

미국의 군함 총톤수는 950만 t에 이르지만 중국은 미국의 5%에도 못 미치는 군함 총톤수 50만 t 미만의 해군력을 보유하고 있다 （no translation）

美国军舰总吨数达950万吨,但中国仅拥有不到50万吨的海军军力,不到美国的5%

또 중국이 운용하는 젠-15 함재기의 전투 능력은 미 해군의 최신 함재기 F-35에 미치지 못한다고 군사전문가들은 평가한다 （no translation）

军事专家们同时认为,中国运用的歼-15舰载机的战斗能力,也不如美国海军最新战机F-35

여기에 미국이 20만 명 이상의 해병대 병력을 보유하고 있지만, 중국은 2만 명에서 10만 명으로 확대하기 위해 노력하는 중이다 （no translation）

此外,美国拥有超过20万名的海军陆战队兵力,但中国仍在为从2万人增至10万人而努力

北달려간 왕이, 남북미중 회담 요구할듯 Chinese foreign minister likely to request a 4-nation summit

王毅访问北韩,似乎将要求举行南北美中四方会谈

중국 왕이(王毅) 국무위원 겸 외교부장이 2일 오전 평양에 도착했다 Chinese State Councilor and Foreign Minister Wang Yi arrived in Pyongyang Wednesday morning

中国国务委员兼外交部长王毅2日上午抵达平壤

3일까지 평양에 머무는 왕 위원은 리용호 북한 외무상과 회담한 뒤 김정은 북한 국무위원장을 면담할 것으로 알려졌다 Mr Wang, who will be staying in Pyongyang one more day, is expected to have a meeting with North Korean Foreign Minister Ri Young Ho and have a face-to-face talk with North Korean leader Kim Jong Un

据悉,王毅将在平壤逗留至3日,将与北韩外相李勇浩进行会谈,并会见北韩国务委员会委员长金正恩

중국과 북한은 2일 오후까지 왕 위원의 평양 내 행보와 관련한 소식을 전하지 않았다 The leaders of the two Koreas announced last Friday at Panmunjom Declaration to “push forward a 3-nation (South Korea, North Korea and the United States) or 4-nation (South Korea, North Korea, the United States and China) summit” in order to build a peace regime

截至2日下午,中国和北韩都没有报道有关王毅在平壤境内行踪的消息

왕 위원은 △북-미 정상회담 전후로 예상되는 시진핑(習近平) 중국 국가주석의 방북 일정을 확정하고 △남북 정상이 판문점선언에서 천명한 평화체제 협상은 중국이 참여한 남북미중 4자 회담이 돼야 한다는 점을 요구하는 등 북-미 정상회담을 앞두고 중국의 입장을 북한에 구체적으로 전할 것으로 관측된다 （no translation）

据观察,王毅此行是在北韩-美国首脑会谈之前,向北韩表明中方的具体立场,其中包括△中国国家主席习近平可能在北韩-美国首脑会谈前后访问北韩 △南北韩领导人在《板门店宣言》中阐明的和平机制磋商必须是有中国参与的南北美中四方会谈等

남북 정상회담 결과에 대한 설명도 들을 것으로 알려졌다 （no translation）

据悉,王毅还将听取有关南北韩首脑会谈结果的介绍

남북 정상은 지난달 27일 판문점선언에서 평화체제 구축을 위해 “남북미 3자 또는 남북미중 4자 회담을 추진한다”고 밝혔다 （no translation）

南北首脑4月27日在《板门店宣言》中表示,为了构建和平机制,“将推进南韩 北韩 美国三方或南北美中四方会谈”

미국의소리(VOA) 중문판은 “왕 위원이 중국 ‘패싱’(배제)을 막기 위해 급하게 방북했다”는 분석을 내놓았다 “Mr Wang urgently visited North Korea to avoid China ‘passing’ (excluding),” analyzed the Chinese version of Voice of America (VOA)

“美国之音”中文版分析称,“王毅为了阻止‘跳过’中国而急忙访问北韩”

주한미군 철수 등 중국의 한반도 이해관계와 직결되는 평화협정 협상에서 중국이 배제돼서는 안 된다는 입장을 전하기 위한 적극적 개입 외교의 일환이라는 것이다 In other words, this was part of China’s diplomacy of active participation to send the message that China should not be left out in peace treaty negotiations, which are directly related to China’s interest with the Korean Peninsula such as the withdrawal of USFK

这是中国积极介入韩半岛局势外交的一部分,其的的是要表明中方的立场,在驻韩美军撤离等直接关系到中国在韩半岛利害关系的和平协定谈判,中国不能被排在外

중국 외교부장의 방북은 2007년 7월 이후 11년 만이다 Chinese Foreign Minister’s visit to North Korea is the first time since July 2007

这是中国外交部长自2007年7月以来时隔11年再次访问北韩

왕 위원과 북한 사이에서 시 주석의 방북 시점이 어떻게 조율될지도 주목된다 Attention is being paid to how Chinese President Xi Jinping’s visit to North Korea will be adjusted by Wang Yi and the North Korean leadership

王毅和北韩方面如何协调习近平主席的访问时间备受瞩目

베이징 외교 당국자는 “(시 주석의 방북 시점이) 상식적으로 북-미 정상회담 전은 아닐 것”이라고 말했다 “President Xi’s visit to North Korea will commonsensically not take place before North Korea–U S summit,” said the Chinese diplomatic authorities

北京外交当局者表示:“从常识来看,(习主席访问北韩的时间)不会在北韩-美国首脑会谈之前”

시 주석이 방북에 이어 한국을 방문할 수 있다는 관측도 나온다 There are also views that President Xi may visit South Korea following his visit to North Korea

也有观测称,习主席在访问北韩后可能会访问韩国

（（additional translation））MeToo movement spreads in China The MeToo movement is spreading across prestigious universities in China in spite of government authorities’ censorship

（增译）中国北京大学的学生们,突破审查喊出“我也是”中国名牌大学的大学生突破政府部门的审查,正在扩散“我也是(#MeToo)”运动

（（additional translation））At Beijing University, students called for disclosure of data related to previous sexual harassment events, which was denied by the school But the issue has escalated,

（增译）在北京大学,有学生要求透明地公开过去性骚扰事件的相关资料,学校方面虽然堵住了他们的嘴,但是校园里贴出了支持学生的大字报,影响正在扩大

（（additional translation））with students putting up hand-written posters supporting the disclosure Students at Renmin University protested against a professor suspected of sexual harassment

（增译）在人民大学,学生们针对怀疑性骚扰的教授展开了抗议示威

（（additional translation））The students’ organized movement is seen as the first of its kind since the Tiananmen Square protest

（增译）有人评价说,自1989年天安门事件之后,首次出现了有组织的学生运动

（（additional translation））Weishin, a student at Beijing University, uploaded on Monday a posting criticizing the school’s action on his WeChat account

（增译）北京大学学生岳昕23日在微信(相当于韩国的可可聊天)正式账户上以公开信方式,上传了批评学校措施的文章

（（additional translation））On April 8, eight students including Weishin called for the disclosure of information regarding the suicide of Kaoyen, who took her own life after suffering from sexual assault by Professor Shunyang in 1998

（增译）据文章称,包括岳昕在内的8名北京大学学生,9日申请公开“1998年因遭受该校教授沈阳性暴力后不堪痛苦而自杀的高岩事件”的相关资料

（（additional translation））The school, however, threatened that such behavior would “affect graduation”

（增译）但是学校方威胁称,“还想顺利毕业吗

（（additional translation））and replied on last Friday that “no information was available ”

（增译）,20日回复学生称“没有可提供的资料”

（（additional translation））Early morning on Monday, a school faculty member and Weishin’s mother broke into his room

（增译）23日凌晨,校方相关人士和岳昕的母亲闯入岳昕正在睡觉的宿舍

（（additional translation））The faculty member demanded that Weishin should “discard all data stored on his mobile phone and computer demanding information disclosure of the event and write a pledge promising not to intervene in the future ”

（增译）有关人士要求岳昕“删除手机和电脑中有关要求公开信息的全部资料,并写下保证书不再介入此事

（（additional translation））According to Weishin, the school informed Weishin’s mother of distorted information and pressured her to take him home and lock him up

（增译）据岳昕称,校方制造假消息并向其母亲施压,要求把岳昕带回家管教

（（additional translation））The Financial Times reported that other students that joined Weishin were also suffering from threats and surveillance A poster titled “We support warrior Weishin” was posted Monday night at Beijing University

（增译）《金融时报》报道称,要求公开信息的其他学生也因校方的恐吓和监视而痛苦不堪23日夜间,北京大学贴出了“声援勇士岳昕”为题的大字报

（（additional translation））However, China forbids such postings publicly

（增译）中国在法律上禁止贴大字报

（（additional translation））The author of the poster said that BBC Chinese website reported that school security guards tore down the posters and Beijing University was barred from the search engine for some time Photos of Weishin’s letter and posters continue to upload on websites, despite government censoring

（增译）大字报的作者在BBC的中文版上表示:“几个小时后,学校警卫们撕掉了大字报,‘北京大学’一度成为网络不能搜索的敏感词”岳昕的公开信和大字报照片因中国当局的审查被删除后,仍在继续上传

（（additional translation））Last week, some 40 students at Renmin University held a protest in front of the classroom of an Economics professor suspected of harassing a female student, demanding that the professor clarify the case Security guards blocked students from entering classrooms and escorted the professor away The Financial Times reported that the professor was eventually fired “I’ve never seen a protest before on school grounds,” said a student who informed of the event There are rumors that a teaching assistant at Tsinghua University sexually assaulted a student, but the school remains silent

（增译）上周,在人民大学被疑对女学生进行性骚扰的经济学教授上课的教室前,有40多名学生展开示威,并要求“学校和教授就此事作出答复”保安人员们阻止示威学生进入教室,并带着教授离开据《金融时报》报道,据悉,问题教授最终已被解雇听到示威消息的一名中国大学生对本报表示,“校园内的示威,我一次也没有见过,”感到十分惊讶据悉,最近清华大学也有助教性侵学生的说法,但校方一直保持沉默

中매체 “6·25 같은 의지로 美와 무역전쟁” Chinese media: ‘Fight trade war with U S the same way as Korean War’

中国媒体:“用打抗美援朝的意志来打对美贸易战”

중국 공산당 기관지 런민(人民)일보의 자매지 환추(環球)시보는 8일 사설에서 “항미원조(抗美援朝·중국이 6·25전쟁 때 미국과 맞서 북한을 돕기 위해 참전한 것)의 의지로 결연히 도널드 트럼프 미 정부의 무역 공격을 쳐부술 것”이라고 주장했다 In an editorial on Sunday, the Global Times, the sister paper of the ruling Communist Party's official People's Daily, said, “A strategic resolution is being established in China, which is to fight the Trump administration's trade aggression in the same way the country fought U S troops during the Korean War (1950-53)

中国共产党机关报《人民日报》的姐妹刊《环球时报》在8日的社论中指出:“要以抗美援朝(中国在6·25战争时期为对抗美国帮助北韩而参加的战争)的意志,坚决粉碎美国特朗普政府的贸易攻击”

이어 “항미원조는 미국 군대가 압록강변에 도달해 일어났다 미국이 일으킨 무역전쟁은 중국의 핵심 이익에 충격을 줬다”며 We should fight today's trade war with the same strategic spirit that fears no sacrifice or loss, and force the United States to burn the very stick that it wields at China ” “China's participation in the Korea War was due to U S military's approach to the Yalu River, which is the Chinese border

社论说:“抗美援朝的发生是因为美国军队打到了鸭绿江边,今天美国发起的贸易战也在冲击中国的核心利益线”

“물러설 수 없는 위기감을 갖고 국가 근본 이익을 수호하기 위해 미국에 양보할 수 없다는 결연한 의지로 중국 사회가 응집하고 있다”고 강조했다 Today's trade war is also due to U S acts that are hurting China's core interests,” the newspaper added “A sense of crisis that we have no place to retreat is uniting the Chinese society

社论强调:“我们无处可退的危机感,和为维护国家根本利益决不可对美方让步的坚定意志正在中国全社会凝聚起来”

환추시보는 또 “중국은 무역전쟁의 무기와 탄약이 매우 충분하다 우리는 (전쟁에선) 희생을 치러야 함을 안다”고 주장했다 We know there will be sacrifices but we understand better that there is no limit to the greed of hegemony China has enough ammunition to fight this trade war

《环球时报》还主张,“中国打贸易战的武器和弹药十分充足，我们知道将付出一些牺牲”

한편 미 해군 항공모함 루스벨트함(CVN-71)을 기함으로 하면서 이지스 순양함 벙커힐(CV-17), 미사일 구축함 샘프슨(DDG-102) 등으로 전단을 구성한 제9항모강습단이 6일부터 남중국해 남부 해역에서 싱가포르 해군과 연합 군사훈련을 벌이고 있다 ” The U S ninth aircraft carrier battalion combat team, which is headed by the flagship USS Theodore Roosevelt (CVN 71) and comprised of the guide-missile destroyer USS Halsey (DDG 97), and the cruiser USS Bunker Hill (CG 52), has been conducting a joint military drill with the Singaporean navy in southern waters of the South China Sea since Friday

此外,随着美国海军“罗斯福”号航空母舰(CVN-71)起航,由“邦克山”号宙斯盾巡洋舰(CV-17) “桑普森”号导弹驱逐舰(DDG-102)等组成战团的第九航母打击团,6日起在南中国海南部海域与新加坡海军展开了联合军事演习

중국의 유일한 실전 배치 항공모함인 랴오닝(遼寧)함은 5일부터 남중국해 하이난(海南) 해역에서 40여 척의 군함과 잠수함, 훙(轟)-6K 전략 폭격기 12대 등과 함께 대규모 훈련을 진행하고 있다 The Liaoning, the only Chinese aircraft carrier that has been put to combat mission, is conducting a large-scale military exercise with more than 40 battleships and submarines, and 12 H-6K strategic bombers in Hainan waters in the South China Sea from Thursday

中国唯一的实战部署航空母舰“辽宁”号从5日起在南中国海海南海域与40多艘军舰 潜艇以及12架轰-6K战略轰炸机一道,进行大规模演习

루스벨트함이 중국이 영유권을 주장하는 해역에 접근해 ‘항행의 자유’ 작전을 펼칠 경우 미중 항공모함 간 첫 대치가 발생할 수 있다는 우려가 나온다 Watchers raise concern that if the USS Theodore conducts “freedom of navigation” operations by approaching waters that China claims sovereignty, U S and Chinese aircraft carriers could get into their first ever standoff

如果“罗斯福”号接近中国主张拥有主权的海域展开“航行自由”行动,人们担心会出现美中两国航空母舰之间的首次对峙

中, 작심 반격…미국산 콩에 보복관세 China retaliates with new levies on U S products

中国下决心反击……对美国产大豆实施报复性关税

미국이 첨단 산업을 정조준해 1300개 중국산 수입품 품목에 25%의 ‘관세폭탄’을 떨어뜨렸다 The United States slapped tariffs of 25 percent on 1,300 Chinese imported goods targeting the country’s high-tech industries

美国瞄准中国的尖端产业,对1300种中国产进口产品引爆了25%的“关税炸弹”

미국산 돼지고기와 농산물 등에 대한 중국의 보복관세에도 아랑곳하지 않고 예고한 대로 강펀치를 날렸다 It stood firm to its hawkish stance notwithstanding China’s retaliatory tariffs on U S pork and agricultural products

不顾中国将对美国产猪肉和农产品实施报复性关税,按预告打出了强烈的一拳

미국무역대표부(USTR)는 3일(현지 시간) 25% 관세를 부과할 500억 달러(약 52조8600억 원)어치 중국산 수입품 1300개 품목을 발표했다 The U S Trade Representative (USTR) announced on Tuesday (local time) 1,300 items of Chinese imports worth 50 billion U S dollars (some 52. 86 trillion won) that would be subject to tariffs of 25 percent

美国贸易代表部当地时间3日公布了征收25%关税 总额500亿美元(约合52.86万亿韩元)的1300项中国产进口产品

USTR가 이날 공개한 58페이지 분량의 관세 부과 목록에는 반도체, 통신장비, 리튬배터리 등 첨단 기술 제품부터 중장비, 철강, 금속 및 알루미늄 제품, 발전기, 오토바이, 항공기 부품, 무기, 의료기기 등이 골고루 포함됐다 The 58 page list of tariff items announced by the USTR included high tech products such as semiconductors, telecommunication equipment, lithium batteries and various other products such as heavy equipment, steel, metal, aluminum products, power generators, motorcycles, aircraft parts, weapons and medical instruments

美国贸易代表部当天公布的58页征税目录中,包括半导体 通信设备 锂电池等尖端技术产品,以及钢铁 金属 铝制品 发电机 摩托车 航空器配件 武器 医疗器械等各种产品

특히 고율 관세 부과 제품에는 중국이 내놓은 첨단산업 육성 전략인 ‘중국 제조(메이드 인 차이나) 2025’ 품목이 상당수 포함됐다 In particular, several items under the Made in China 2025 industrial development program are to be imposed higher rates

特别是高关税产品中,包括了中国推出的尖端产业培养战略“中国制造2025”中相当数量的品种

USTR는 이날 “중국은 미국 회사의 민감한 상업 정보와 무역 기밀에 접근하기 위한 컴퓨터 네트워크 무단 침입 및 기술 절취를 지원하고 실행했다”며 “China has supported and executed unlawful network infringement and technology theft that have allowed access of sensitive commercial information and confidential trade information of U S businesses,” the USTR said

美国贸易代表部当天声称,“中国为获得美国公司的敏感商业情报和贸易机密,支持并进行电脑网络的无端入侵和技术窃取,”

“(관세 부과 조치는) 미국 경제에 미친 피해와 중국의 해로운 정책, 절차, 행위를 없애기 위한 두 가지 측면에서 적절한 수준”이라고 밝혔다 “The tariff measure is appropriate considering China’s damage on the US economy and eliminating China’s harmful policy, procedures and acts

(征收关税的措施)从对美国经济遭受的损失和为消除中国的有害政策 程序和行为这两个层面上,水平都是合适的”

도널드 트럼프 미국 대통령은 중국에 대해 “무역적자의 리더”라고 비판했다 ” U S President Donald Trump criticized China as the “leader of trade deficit”

美国总统特朗普批评中国是“贸易赤字的领袖”

중국은 USTR의 관세 부과 품목 발표에 즉각 반발했다 China immediately resisted to the USTR’s announcement,

中国则立即反对美国贸易代表部公布征税品种

중국 상무부는 USTR의 발표 후 1시간여 만에 대변인 명의의 담화를 내고 “중국은 결연히 반대하고 조만간 법에 따라 미국산 상품에 대해 동등한 강도와 규모로 대등한 조치를 하겠다”고 경고했다 and the Chinese Ministry of Commerce issued a spokesperson statement an hour later, warning, “China stands resolutely opposed and shall impose measures equally strong and similar in scale on U S products in accordance with laws

中国商务部在美国贸易代表部宣布后1个多小时,就以发言人名义发表声明警告说:“中国坚决反对美方的做法,近期将依法对美国产商品采取同等强度和规模的对等措施”

중국은 미국의 철강과 알루미늄 보복 관세에 맞서 2일부터 돼지고기 등 30억 달러(약 3조1700억 원)어치 미국산 수입품에 고율 관세를 부과했다 ” Since Monday, China has imposed high tariffs on U S imports worth three billion dollars (about 3. 17 trillion won) including pork, in retaliation to U S measures to impose tariffs on Chinese steel and aluminum products

中国针对美国的钢铝报复关税,从2日开始对猪肉等30亿美元(约合31700亿韩元)美国产品征收高率进口关税

北 최고위급, ‘中의 실리콘밸리’ China’s VIP security raises speculation of Kim Jong Un visit

北韩最高领导人参观“中国硅谷”中关村后回国

중관춘 방문뒤 귀국김정은 북한 노동당 위원장이거나 김 위원장의 여동생 김여정임이 확실해 보이는 북한 최고위 인사는 27일 중국 당국의 정상급 경호 의전을 받으며 베이징 서남부 곳곳을 누볐다 Speculation about a visit to Beijing by North Korean leader Kim Jong Un or another high-level Pyongyang official was running high Tuesday amid talk of preparations for a North Korea-U S meeting North Korea’s senior leadership, who must be Kim Jong Un or his sister Kim Jong Un, has wrapped up an unprecedented whirlwind trip to the Chinese capital Monday while receiving Chinese authorities’ tight levels of security

疑似北韩劳动党委员长金正恩或者金正恩妹妹金与正的北韩最高层人士,27日接受中国当局最高级警卫的礼宾待遇,穿梭在北京西南部各处

특히 아버지 김정일이 방중 때마다 거의 빠짐없이 방문했던 ‘베이징의 실리콘밸리’ 중관춘(中關村)을 방문해 주목된다 The senior leadership reportedly visited Zhongguancun in Beijing, which is known as "China's Silicon Valley" and the favored venue of Kim’s father, Kim Jong Il, during his visits to Beijing

特别是,北韩领导层人士访问了父亲金正日访华时必去的“北京硅谷”中关村,备受关注

북한 최고위 인사가 머문 국빈 숙소 댜오위타이(釣魚臺) 정문(동문)은 이날도 무장경찰이 대거 배치돼 기자들의 취재를 막으며 삼엄한 분위기를 연출했다 The roads around the Diaoyutai State Guesthouse, where the North Korean leadership stayed, were closed amid a spate of road closures and tight security measures

北韩最高领导层人士下榻的国宾馆——钓鱼台国宾馆当天也部署了大量武装警察,阻止记者们的采访,营造了森严的气氛

베이징의 한 소식통은 “북한 최고위 인사가 (할아버지) 김일성이 중국을 방문할 때마다 머물렀던 댜오위타이 18호각에서 하룻밤을 묵은 것으로 안다”고 전했다 A large security presence remained until Tuesday morning when the distinctive green train carrying the North Korean leadership set off on its return journey to Pyongyang

北京的一位消息人士透露说:“据我所知,北韩最高领导层人士住在(爷爷)金日成每次访华时下榻的钓鱼台国宾馆第18号楼”

이날 오전 9시 반경 최고위 인사가 탄 차량 등 2대가 중국 측 공안 사이드카 10여 대의 호위를 받으며 댜오위타이 정문을 빠져나왔다 Along with a cordon a couple of hundred meters away, a convoy of vehicles accompanied by an escort of police on motorbikes left the compound at around 9 30 a m on Tuesday

当天上午9时30分左右,最高领导层人士乘坐的两辆汽车在10多辆中国公安车辆的护卫下,驶出了钓鱼台正门

이 차량은 베이징의 실리콘밸리라 불리는 서부의 중관춘으로 향했다 Residents living near one of the most heavily guarded buildings in Beijing reported unusually tight levels of security, and locals and foreign tourists complained about a spate of unexpected road closures

汽车驶向被称为北京硅谷的城西中关村

특히 이 인사는 중관춘 최대의 컴퓨터 전자기기 상가인 하이룽(海龍)빌딩을 방문한 것으로 알려졌다 （no translation）

据悉,这位人士访问了中关村最大的电脑电子器械商街——海龙大厦

하이룽빌딩 내의 컴퓨터, 전자기기 상점들이 한국 서울 용산전자상가의 몇 배에 달하는 것으로 알려져 있다 （no translation）

据悉,海龙大厦内的电脑电子器械商店已达到韩国首尔龙山电子商街的数倍

김정일은 첫 중국 방문인 2000년 5월, 2010년 5월, 마지막 방문인 2011년 5월에 중관춘을 찾았다 （no translation）

金正日首次访问中国的2000年5月以及2010年5月 2011年5月都访问了中关村

방중한 북한 최고위 인사가 김정일 방중 전통을 따라 중관춘을 방문해 컴퓨터 전자기기 상가를 둘러본 것은 이 인사가 과학기술에 관심이 많고 이 분야의 국산화 발전에 주력하고 있는 것과 관련이 있다는 관측이 나왔다 （no translation）

访问中国的北韩最高领导层人士根据金正日访问中国的传统造访中关村,参观了电脑电子设备商业街有分析认为,北韩领导层人士对科技非常感兴趣,与致力于发展该领域的国产化有关

이날 최고위 인사의 방문지마다 삼엄한 통제로 통행이 금지되거나 심지어 도로를 쳐다보는 행위까지 금지해 중국 국민과 해외 여행객들이 불편을 호소하거나 불만을 표출했다 （no translation）

在当天北韩领导人的访问中,中方进行交通管制甚至禁止人们围观道路,引起中国国民和海外游客的抱怨和不满

댜오위타이의 모든 출입구에는 공안이 배치됐으며 인근 200m 구간이 통제됐다 （no translation）

在钓鱼台的所有出入口都有公安,附近的200米区间被管制,记者被阻止接近

취재진의 접근도 막았다 （no translation）

当天上午,在中关村一带,随着进行交通管制,

이날 오전 중관춘 일대에도 교통 통제가 되면서 웨이보(중국의 트위터 격)에는 불편을 호소하는 시민들의 불만과 교통 통제 사진, 최고위 인사의 차량 행렬 사진 동영상 등이 잇따라 올라왔다 （no translation）

市民们表达不满和交通管制 北韩领导人车队的照片接连不断出现在微博(中国的推特)上

한 시민은 “진싼팡(金三반·김씨네 3대 뚱보)이 왔는가 보다”라고 비꼬기도 했다 （no translation）

一位市民甚至讽刺说:“应该是金三胖来了”

진싼팡은 중국 누리꾼들이 김정은을 얕잡아 부르는 대표적 표현이다 （no translation）

金三胖是中国网民贬低金正恩的典型称呼

이날 오전 11시경부터 베이징 남부에 있는 베이징기차역의 VIP(귀빈) 출입구가 통제되기 시작했고 11시 20분경부터는 베이징기차역 인근 도로 일부도 통제됐다 （no translation）

从当天上午11点开始,位于北京南部的北京火车站的贵宾出入口开始管制,从11点20分开始,北京火车站附近的道路也被部分管制

낮 12시 반경부터 북한에서 온 ‘1호열차’가 대기했다 오전 11시부터 자금성과 톈안먼(天安門)광장을 가르는 베이징 중심가 창안제(長安街) 일부와 톈안먼광장 서쪽의 인민대회당 서쪽 도로가 통제와 해제를 반복했다 There are rumors claiming that from 12:30 p m on Tuesday, the motorcade was once again spotted on the streets of Beijing passing by Tiananmen Square apparently en route to the railway station where the train that arrived on Monday night was waiting

从中午12点30分开始,来自北韩的“1号”专列等候在站内从上午11时开始,分割紫禁城和天安门广场的北京中心道路长安街和天安门广场西侧的道路不时被管制和解除

이 과정에서 공안들은 지나는 시민과 관광객들에게 인도에 서 있지 못하게 하고 통제된 도로를 쳐다보는 행위에 대해서조차 제지를 했다 （no translation）

在这个过程中,公安们要求路过的市民和游客们不要站在人行道上,并且制止人们眺望被管制的道路

이날 낮 12시 반경부터 베이징 남부 톈탄(天壇)공원 주변 도로가 통제됐다는 얘기도 들렸다 （no translation）

还有消息说,当天中午12点30分左右,北京南部天坛公园周边的道路被管制

이 최고위 인사는 중관춘을 출발해 인민대회당 북쪽 창안제를 거쳐 베이징기차역에 이르는 동선을 선택한 것으로 관측된다 （no translation）

据观测,北韩领导人从中关村出发后,选择了经过人民大会堂北侧长安街到达北京火车站的路线

도중에 모처에서 점심식사를 한 것으로 알려졌다 （no translation）

据悉,途中还在某处吃了午饭

한편 북-중 접경 지역인 랴오닝(遼寧)성 단둥(丹東)에서 압록강철교를 내려다볼 수 있는 중롄(中聯)호텔은 27일까지 압록강변을 바라보는 강변쪽 객실 예약을 중단했다 （no translation）

另外,北韩与中国接壤的辽宁省丹东,能看到鸭绿江铁桥的中联酒店,中断了截至27日能看到鸭绿江边的客房的预约

중국, 北-美 대화 지원하고 ‘한반도 평화’ 후원자 돼야 China should support a peaceful Korean Peninsula

中国应支持北韩与美国对话 成为“韩半岛和平”的襄助者

중국 시진핑 국가주석은 어제 정의용 청와대 국가안보실장을 만나 남북, 북-미 정상회담 합의에 환영과 지지의 뜻을 나타내며 “성과를 내기 바란다”고 말했다 Chinese President Xi Jinping welcomed and expressed support for the inter-Korean and the North Korea-U S summits on Monday in a meeting with South Korean President Moon Jae-in’s top security advisor Chung Eui-yong, who led the special envoys to North Korea, hoping that it could make a good result

中国国家主席习近平昨天会见了青瓦台国家安保室长郑义溶,对南北韩 北韩与美国的首脑会晤表示欢迎和支持,并表示“希望取得成果”

정 실장은 양제츠 외교담당 국무위원, 왕이 외교부장과도 각각 면담과 함께 오찬과 만찬을 함께했다 Chung also had luncheon and dinner meetings with China’s top foreign policy adviser Yang Jiechi and Foreign Minister Wang Yi, respectively

郑义溶与负责外交事务的国务委员杨洁篪和外交部长王毅分别会晤,并各自共进午餐和晚餐

중국의 큰 연례 정치행사인 양회(兩會·전국인민대표대회와 전국인민정치협상회의) 기간에 시 주석을 비롯한 최고위 외교라인이 일제히 시간을 낸 것은 이례적이다 It is unprecedented that Xi and China’s top diplomatic officers made time to attend the briefing by the special envoy given that the Chinese People's Political Consultative Conference and National People's Congress are currently taking place

在中国的重大年度政治活动“两会”(全国人民代表大会和全国人民政治协商会议)期间,包括习近平主席在内的中国外交最高层都抽出时间来,是相当罕见的

한반도 정세의 급변에 대한 비상한 관심의 반영이자 ‘차이나 패싱(중국 배제)’ 우려가 작용한 것으로 볼 수 있다 This shows the Chinese leadership is paying keen attention on the developments surrounding the Korean Peninsula while raising concerns over China being excluded from the historical diplomatic breakthrough

可以说,这反映出对韩半岛局势剧变的高度关注,同时也是因为担心出现“越过中国(排除中国)”

중국은 남북, 북-미 정상회담에 환영과 지지를 표명하면서도 속내는 복잡하고 불편할 것이다 Beijing should feel uncomfortable although they welcomed the decision made by the U S , South and North Korean leaders

中国对南北韩和北韩-美国首脑会晤表示欢迎和支持,但恐怕内心是复杂而不舒服的

그동안 북-미 간 대화를 주선하고 북핵 6자회담 의장국을 맡는 등 적극적 중재 역할을 맡았던 중국이다 Until now, it has arranged North Korea-U S dialogues and chaired the six-party talks, playing an active role of mediator

一段时间以来,一直是中国牵线北韩与美国之间的对话 担任北韩核问题六方会谈主席国,发挥了积极的斡旋作用

하지만 이번엔 북-미가 중국을 건너뛰고 한국의 중재로 역사적인 첫 정상회담을 열기로 했다 But this time around, U S President Donald Trump and North Korean leader Kim Jong Un have agreed to hold a meeting after South Korean President Moon’s delegation delivered the leaders’ direct message, for the first time in history

但此次北韩和美国跳过中国 通过韩国的斡旋决定举行历史性的首次首脑会谈

더욱이 김정은 집권 이후 북-중 간에는 정상회담도 없었다 Not a single summit between Pyongyang and Beijing had been taken place since Kim Jong Un took power

而且,在金正恩执政后,北韩与中国之间还没有举行过首脑会谈

이른바 ‘조중(朝中)혈맹’은 과거지사가 됐다지만 중국이 북-미 직거래를 한가롭게 팔짱 끼고 지켜볼 수만은 없는 처지다 Let alone restoring blood alliance, China has to do something to exert greater influence while seeking to shape the geopolitical order on the peninsula

所谓“北韩-中国血盟”已成为过去之事,但中国却不能袖手旁观北韩和美国直接交易

특히 중국이 줄곧 북핵 해법으로 주장해온 북한의 핵·미사일 도발과 한미 연합군사훈련 동시 중단이라는 쌍중단(雙中斷)도 무색해졌다 China has long argued the denuclearization would be achieved only by ceasing both South Korea-U S joint military drills and nuclear or missile provocations by the North

尤其是,中国一直提出的北韩核问题解决方案,即同时暂停北韩核导弹挑衅和韩美联合军事演习的“双暂停”主张也变得黯然失色

중국은 올림픽 기간의 연합훈련 연기가 사실상 중단 효과를 낸 것이라고 하지만, 북한은 한미 연합훈련도 양해한다며 중단 요구 없이 핵·미사일 실험 중단을 약속했다 오히려 북한이 미국과의 직접 대화에 나선 것은 중국의 대북제재 협조였다 But Kim Jong Un pledged that the regime would refrain from any further nuclear or missile tests, not demanding a cessation of joint military exercise between South Korea and the United States It was China’s faithful implementation of the sanctions that pushed North Korea to the negotiating table

虽然中国表示奥运会期间暂停联合军演就是实际上的暂停,但北韩后来又声称谅解韩美举行联合军演,没有要求暂停就承诺暂停核导试验。相反,北韩直接同美国进行对话,是因为中国对对北韩制裁的协助

중국이 대북제재를 보다 엄격하게 시행하자 북한은 중국 대신 미국을 향해 손짓하는 상황으로 전개됐다 China adding pressure on the North with the stricter sanctions and their effective implementation has led Kim Jong Un to invite Trump for talks

中国更为严格地实施对北韩的制裁后,北韩不是向中国 反而开始向美国“招手”

앞으로 북-미 관계가 급진전되면 미국과의 경쟁에서 북한을 우호적 완충지대로 두겠다는 중국의 대북 전략은 전면 수정이 불가피할 수도 있다 If the unprecedented meeting between Kim and Trump takes place and if diplomatic developments occur, the Chinese government may have no other option but to completely change its North Korea policy, which aims to have its neighboring ally as a strategic buffer zone in vying for geopolitical power with the United States

今后,如果北韩与美国关系迅速发展,在与美国的竞争中将北韩视为友好的缓冲地带的中国,可能将不可避免地全面修改其对北韩战略

문재인 대통령도 어제 “우리가 이루려는 것은 지금까지 세계가 성공하지 못한 대전환의 길”이라며 “We are trying to achieve what the world has failed to do so far,” South Korean President Moon Jae-in said Monday

韩国总统文在寅昨天也表示:“我们想要实现的,是目前世界没有成功过的大转变之路

“우리가 성공해낸다면 세계사적으로 극적인 변화가 만들어질 것이며 대한민국이 주역 될 것”이라고 했다 “If we succeed, there will be dramatic changes in world history and the Republic of Korea will have played the leading role

如果我们取得成功,将是世界史上戏剧性的变化,韩国将是其主角”

북-미 관계의 변화는 분명 한반도는 물론 동북아시아 질서의 근본적 개편을 불러올 수 있다 ” Any developments on the North Korea-U S relations are bound to transform the geopolitical order not only on the Korean Peninsula but also in Northeast Asia

北韩与美国关系的变化必然会引发韩半岛乃至东北亚秩序的根本性改变

급속한 정세 변화에 중국도 경계심을 가질 수 있다 Thus, it is natural Beijing keeps its eyes wide open to changes of the situation

对于迅速的变化,中国也会起戒心

하지만 중국의 역할을 갑자기 미국이 대신할 수는 없다 북한이 거리를 두면서도 결국 생존을 의지해온 나라가 중국이다 Nevertheless, China, the one and only ally that North Korea relies on economically and politically, cannot be replaced by the United States

但是,美国不能突然代替中国的作用，北韩虽然与中国保持距离,但最终依赖生存的国家还是中国

지금까지 과정은 한반도 비핵화와 평화·안정의 수호, 대화·협상을 통한 해결이라는, 중국이 줄곧 내세운 북핵 3원칙의 실현 과정이다 Three principles in China’s strategy for achieving North Korean denuclearization include promoting denuclearization of the Korean Peninsula, protecting peace and stability, seeking dialogues and negotiations

迄今为止的过程,是中国提出并坚持的韩半岛无核化 维护半岛和平与稳定 通过对话和协商解决等北韩核问题三原则的实现过程

마침 중국은 국가주석의 3연임 금지 조항을 삭제하는 개헌안을 통과시켜 시 주석에게 막강한 지위와 권한을 부여했다 China has amended its constitution to abolish the two-term limit for the country's president, allowing Xi Jinping to lead for life

中国刚刚通过了删除禁止国家主席三连任条款的修宪案,赋予了习近平主席强大的地位和权限

앞으로 중국이 본격화할 ‘대국외교’의 첫 시험대는 북한 비핵화를 통한 새로운 동북아 평화질서 구축이 될 것이다 China’s foreign policy – so-called “great power diplomacy” that is to be carried out in earnest, will be put under scrutiny The first testing stand is to see if Beijing will be able to establish peace and order in the region through denuclearization

中国今后全面展开“大国外交”的第一个试验台,将是通过实现北韩无核化构建东北亚新的和平秩序

중국이 북한의 후견국가를 자처하던 시절은 지났다 China does not view itself as a guardian of the reclusive regime any longer

中国以朝鲜的“保护国”自居的时代已经过去

동북아 지도국가로서 새로운 질서를 짜는 데 적극적인 역할을 해야 한다 As a world power and a responsible member of the international community, it needs to take the leading role in reshaping the order in Northeast Asia

作为东北亚的领导国家,在制定新秩序的过程中,应当发挥积极的作用

트럼프-김정은 5월 정상회담에 주변국은미국과 북한이 최대한 빨리 대화해야 한다고 강조해온 중국 외교부는 9일 정례 브리핑에서 도널드 트럼프 미국 대통령이 5월 전 김정은 북한 노동당위원장과의 회담 의사를 밝힌 것에 대해 환영 의사를 나타냈다 China’s response to the announcement of Kim-Trump meeting China officially welcomed the announcement that U S President Donald Trump and North Korean leader Kim Jong Un planned to hold a meeting by May on Friday at the Foreign Ministry’s regular press briefing

特朗普与金正恩5月举行元首会谈,周边各国怎么看一直强调美国和北韩应尽早进行对话的中国外交部,9日在例行记者会上对美国总统特朗普表示要在5月之前与北韩劳动党委员长举行会谈表示欢迎

중국 관영 매체들은 이날 북미 정상회담 성사를 속보로 전하면서 놀라움을 감추지 못했다 Chinese state-run media have also expressed surprise, reporting break news about the would-be first meeting between the two leaders

中国官方媒体当天也以快讯报道美国与北韩将举行首脑会谈,表示感到十分惊讶

평소 자극적인 표현이 많지 않은 중국 공산당 기관지 런민(人民)일보의 웹사이트 런민왕(網)도 ‘대사건 The People’s Daily website, which tends to refrain from using provocative wording in its reports, titled its breaking news “Big event

平时不太使用刺激性表述的中国共产党机关报《人民日报》的官网人民网,也使用“重大事件

트럼프가 5월 전 김정은과 회담에 동의’라는 제목으로 속보를 내보냈다 Trump agrees to hold a meeting with Kim Jong Un by May,” using a word it may not usually choose for a title

特朗普同意5月之前与金正恩会晤”的题目,播发了快讯

런민왕은 다른 기사에서 “북핵 문제의 외교적 해결이 멀고 힘들지만 협상이 전쟁 발생의 우려를 없앨 수 있다”고 지적했다 The website also pointed out in another article that although it would be hard and tough to diplomatically resolve North Korea’s nuclear issue, negotiation may allay concerns of a possible war breaking out

人民网还在另外一篇报道中指出,“虽然北韩核问题的外交解决前路漫漫而且艰难,但谈判可以消除爆发战争的忧虑”

중국 정부 입장을 공식 대변하는 관영 신화(新華)통신도 ‘중대변화 The Xinhua News Agency, China’s official state mouthpiece, also reported the announcement of the North Korea-U S meeting with a title “Significant change

正式代言中国政府立场的官方媒体新华通讯社也播发快讯“重大变化

트럼프가 김정은과 5월 전 만난다’는 제목의 속보를 전했다 Trump meets with Kim Jong Un by May

特朗普与金正恩将在5月之内会面”

신화통신은 다른 기사에서 “트럼프 대통령이 ‘한반도 문제에서 중대한 진전을 이루고 있다고 말했다’”며 “이와 동시에 미국은 합의에 이를 때까지 대북 제재를 계속할 것이라고 밝혔다”고 전했다 ” “President Trump said that great progress had been made but there would be no prospect of lifting sanctions until a deal was reached,” it also said in another report

新华社的另一篇报道称,“特朗普总统表示,‘韩半岛问题正在出现重大进展’他还表示在达成协议前美国将继续对北韩进行制裁”

런민일보 자매지 환추시보(環球) 웹사이트 환추왕도 김정은과 회담 의사를 표명한 트럼프 대통령의 발언을 전한 정의용 대통령국가안보실장의 기자회견을 ‘중대성명’ ‘깜짝뉴스’라고 표현했다（no translation）

《人民日报》的姊妹报纸《环球时报》的官网环球网也把韩国总统国家安保室长郑义溶在记者会上宣布美国总统特朗普愿意与金正恩会面的消息称为“重大声明”“突发新闻”

환추왕은 “너무 갑작스러운 일이다（no translation）

环球网报道称,“太过突然了

북한과 미국이 손을 잡고 기습했다”고 보도했다，（no translation）

北韩与美国联手发动了突然袭击”

외교 소식통들에 따르면 중국은 북미대화를 환영하면서도 향후 비핵화 협상에서 중국이 소외되는 일명 ‘차이나 패싱’이 불거질 것을 우려하는 분위기다Diplomatic sources say that Beijing, while welcoming the news, would also have concerns about it being possibly bypassed by Washington and Pyongyang in future negotiations on denuclearization, despite its support for the international community’s economic sanctions at the cost of the bilateral relations with North Korea

据外交消息人士透露,中国一方面欢迎北韩与美国对话,同时也颇为担心在今后的无核化谈判中会出现中国被排除在外的“越顶中国”局面

중국이 대북 제재로 북중 관계가 악화되는 등의 대가를 치렀음에도 정작 대화 과정에서는 중국이 배제되는 것 아니냐는 것이다（no translation）

尽管中国因对北韩制裁付出了与北韩关系恶化的代价,但在真正的对话进程中却被排斥在外

이 때문에 중국이 남북, 북미 정상회담 상황을 지켜보면서 북중 관계 개선에 나설 것이라는 전망이 나온다 This leads some experts to speculate that China will make efforts to improve its relationship with North Korea, keeping a keen eye on how the inter-Korean and North Korea-U S summit meetings play out

因此有预测称,中国将视南北韩 北韩与美国首脑会晤的情况,着手改善与北韩的关系

소외론을 의식한 듯 중국 외교부는 “중국이 한반도 문제에서 적극적이고 건설적인 역할을 했고 국제사회의 인정을 받았다”는 점을 재차 강조하고 있다 China’s Foreign Ministry, as if conscious of the speculation that it may be excluded in the process of future negotiations, has repeatedly stressed that it has played an active and constructive role in addressing issues on the Korean Peninsula and has been recognized by the international community

中国外交部似乎意识到了“排斥论”,再次强调称,“中国在韩半岛问题上发挥了积极和建设性的作用,得到了国际社会的认可”

왕이(王毅) 중국 외교부장은 8일 기자회견에서 “중국이 주장한 쌍중단(북한 핵·미사일 실험과 한미 연합훈련 중단)이 정확한 처방이었음을 보여준다”며 “(역시 중국이 주장한) 쌍궤병행(비핵화와 평화협정 동시 추진) 방향에 따라 비핵화 과정에서 북한의 합리적인 안보 우려를 해결해야 한다”고 강조했다 지금의 대화 국면이 중국이 주장해온 해결 방향에 따른 것이라는 주장으로 중국 소외론을 불식시키려는 의도로 해서된다 This can be seen as Beijing’s efforts to dispel such speculation by arguing that the current dialogue is based on the direction continuously pushed forwarded by the Chinese government

中国外交部长王毅在8日的记者会上也强调,“这也证明,中国提出的双暂停(北韩暂停核导试验和韩美暂停联合军演)是正确的处方。根据(同样由中方提出的)双轨并行(同时推进无核化与和平协定)方向,在无核化进程中应解决北韩的合理安全关切”分析认为,中国是想强调目前的对话局面正是朝着中国所提出的解决方向前进,以此来驳斥“排斥论”

中 국방비 8% 늘려 사상 최대… 美와 패권경쟁 가속 China expands defense budget by 8% for 2018

中国国防开支增加8%,创历史纪录,加快与美国霸权竞争

지난해 10월 19차 중국 공산당 전국대표대회(당 대회)에서 은퇴한 뒤 대외에 모습을 드러내지 않았던 왕치산(王岐山) 전 중앙기율검사위원회 서기가 5일 처음 공식석상을 통해 언론에 모습을 드러냈다 Wang Qishan, the former Chinese Secretary of the Central Commission for Discipline Inspection who remained reclusive since his retirement following the 19th National Congress of the Communist Party of China in October last year, made a public appearance on Monday, a first since the congress

去年10月份中国共产党第十九次全国代表大会(十九大)上退下来之后一直没有公开露面的前中央纪律检查委员会书记王岐山,5日首次在公开场合出现在媒体面前

시진핑(習近平) 국가주석의 오른팔로 국가부주석이 유력한 그는 이날 베이징(北京) 인민대회당에서 열린 전국인민대표대회(전국인대·한국의 국회 격) 개막식에서 시 주석과 리커창(李克强) 총리 등 7명의 상무위원(최고지도부)에 이어 모습을 드러냈다 A right-hand man of Chinese President Xi Jinping and highly probable vice president of China, Wang Qishan showed up at the opening ceremony of the National People’s Congress (NPC) in Beijing, along with the seven members of China’s politburo standing committee including President Xi and Premier Li Keqiang

作为中国国家主席习近平的左膀右臂,王岐山很有可能出任国家副主席,当天出现在北京人民大会堂举行的全国人民代表大会(全人大 相当于韩国的国会)开幕式上,排在习近平主席 李克强总理等7名中共政治局常委(最高领导层)后面

리 총리가 지난 5년 평가와 2018년 정부 계획을 발표하는 업무보고를 읽어 내려가는 과정에서 실질적인 2인자가 리 총리가 아니라 왕 전 서기임을 보여주는 상징적인 장면이 포착됐다 （no translation）

在李克强朗读评价过去5年和2018年政府计划的政府工作报告的过程中,记者捕捉到了象征实质性二号人物不是李克强总理而是王岐山前书记的场面

리 총리의 업무보고가 시작되자 시 주석을 제외하고 상무위원은 물론 정치국 위원, 중앙위원, 2970명의 전국인대 대표 모두 업무보고 책자를 펴들고 보고를 따라 읽어 내려갔다 （no translation）

李克强刚刚开始作政府工作报告,除习近平主席外,包括其他政治局常委 政治局委员 中央委员和2970名全国人大代表,都打开工作报告的小册子跟着阅读

오로지 시 주석과 왕 전 서기만이 업무보고 책자에 손도 대지 않았고, 시선을 두지도 않았다 （no translation）

只有习近平和王岐山没有打开工作报告小册子,连眼神也没有瞄一下

시 주석은 리 총리의 업무보고가 시작된 지 40여 분 뒤에 책자를 펴들었으나 자신이 보고 싶은 곳만 넘겨봤다 （no translation）

习近平在李克强开始作政府工作报告40多分钟后才打开了小册子,但也只扫了几眼自己想看的内容

‘시왕’(習王·시진핑과 왕치산) 체제의 출범을 예고한 장면이라는 평가가 나온다 （no translation）

有评论认为,这是预告“习王(习近平与王岐山)”体制上台的场面

리 총리는 이날 1시간 50분의 업무보고에서 “시진핑 총서기(시 주석의 당 직책) 핵심적 지위를 결연히 수호해야 한다”고 말하는 등 13번이나 시 주석의 이름을 거론하며 시 주석에 대한 찬양을 이어갔다 （no translation）

李克强在当天长达1个小时50分钟的政府工作报告中,包括“坚决拥护以习近平总书记(习近平的党内职务)的核心地位”等在内,先后13次提到习近平的名字,不断赞扬习近平

업무보고에 이어 시 주석의 임기 제한 폐지를 담은 헌법 수정안 초안이 상정됐다 （no translation）

李克强的政府工作报告结束后,关于取消习近平国家主席任期限制的宪法修正案草案被提交审议

심의를 요청하며 개헌 이유를 설명하는 절차가 50여 분 진행됐으나 관영 중국중앙(CC)TV는 리 총리 보고만 생중계하고 개헌안 상정 과정은 중계하지 않았다 （no translation）

提交审议并解释修宪理由的程序长达50多分钟,但中国官方的中央电视台只实况转播了李克强总理作政府工作报告,没有转播提交宪法修正案草案的过程

여론의 반발 등 민감성을 고려한 것으로 보인다 （no translation）

估计是考虑到舆论反对等敏感性

하지만 인민대회당 현장은 임기 제한 철폐 찬양으로 가득 찼다 （no translation）

但是,人民大会堂现场,却充满了赞成取消任期限制的声音

개헌안 상정 때 나온 유일한 박수는 ‘2번을 초과해 연임하지 못하다’는 헌법 규정을 삭제한다고 소개할 때 나왔다 （no translation）

提交宪法修正案草案时,出现的唯一一次掌声,就是在介绍“取消不得连任两届以上”的限制时

이날 전국인대에서는 올해 경제성장률 목표치로 지난해와 같은 6. 5%가 제시됐다 The NPC gathering on Monday presented a target for this year’s economic growth at 6. 5 percent, the same as the previous year

当天的全国人大会议上,中国提出了与去年持平的6.5%的经济增长率目标

또한 지난해에 비해 국방예산이 8. 1% 증가한 국가예산안이 보고됐다 And the national budget has expanded by 8. 1 percent compared to last year

政府工作报告中提出,国防预算将比去年增长8.1%

이로써 올해 중국의 국방예산은 역대 최대인 1조1069억 위안(약 189조1249억 원)에 달한다 This year, China’s defense budget for 2018 stands at 1. 1069 trillion yuan (174. 6 billion U S dollars), an all-time high

由此,中国今年的国防预算将达到创纪录的11069亿元人民币(约合1891249亿韩元)

2016년 7. 6%, 지난해 7%로 줄어들던 증가율이 다시 크게 늘어난 것이다 The country’s defense budget was on the decline, with the increase rates of the defense budget recorded at 7. 6 percent in 2016 and 7 percent in 2017, respectively

继2016年和2017年先后缩减7.6%和7%之后,中国国防预算再次出现大幅增加

2016년 이전의 두 자릿수 증가율 회복은 아니지만 지난해 19차 당 대회에서 시 주석이 2050년까지 세계 1위 군사 강국이 되겠다는 강군몽(强軍夢)을 천명한 데 따라 중국이 미국과 군사패권 경쟁을 본격화하려는 것으로 풀이된다 While it is far from the two-digit increase rates witnessed before 2016, the modest surge in China’s defense budget this year reflects the country’s plan to engage in a competition for military hegemony against the United States in line with President Xi’s announcement at the 19th national congress to make his country No. 1 military powerhouse by 2050

分析认为,虽然中国军费开支没有恢复到2016年之前的两位数增长率,但在去年的十九大上,习近平主席阐明了到2050年成为世界第一大军事强国的强军梦之后,中国启动了与美国进行军事霸权的竞争

중국은 최근 국가 안보가 위협에 처했다는 이유를 내세우면서 핵 추진 항공모함 건조 등 각종 군비 확장을 시도하고 있다 Citing threats to its national security, China is recently expanding military spending by building nuclear-powered aircraft carriers

中国以最近国家安全处于威胁之中为由,正在尝试建造核动力航母等各种军备扩张

한편 리 총리는 미중 무역 전쟁을 염두에 둔 듯 미국을 겨냥해 “중국은 평등한 협상을 통해 무역 분쟁을 해결할 것을 주장한다 “China claims to resolve trade conflicts through fair negotiations

此外,李克强总统似乎是着眼于美中贸易战争,针对美国强调称,“中国主张通过平等协商来解决贸易争端

보호무역주의를 반대하고 합법적인 권익을 결연히 수호해야 한다”고 강조했다 We must oppose protectionism while resolutely upholding our legitimate rights and interests,” said Premier Li, in an apparent reference to a trade war with the United States

必须反对贸易保护主义,坚决维护合法权益”

中 지식인들 “마오시대로 돌아가나” Xi Jinping abolishes the two-term limit on the presidency

中国知识分子反对习近平长期执政,“要回到毛泽东时代吗

시진핑 장기집권 반발중국 공산당이 25일 국가주석직의 2연임 임기(최장 10년) 제한을 없애는 헌법 개정안을 제안한 데 대한 중국 내 여론의 역풍이 확산되고 있다 The Communist Party of China on Sunday proposed to remove a constitution clause limiting presidential service to just two terms (10 years at maximum) in office, inflaming public opinion in the country

中国共产党25日建议修改宪法,删除“国家主席连续任职不得超过两届(最长10年)”的内容,中国国内舆论的反对之声正在扩散

‘시진핑(習近平) 국가주석이 종신독재 집권의 마오쩌둥(毛澤東) 시대로 돌아가려는 것이냐’는 중국인들의 우려가 증폭되고 있기 때문이다 Many Chinese are now concerned that the country could go back to the days when Mao Zedong had indefinite grip on power

这是因为,中国人的担心正在加剧,“国家主席习近平想回到终身掌权的毛泽东时代吗”

중국의 저명한 여성 사회학자인 리인허(李銀河) 중국 사회과학원 교수는 블로그에 “종신제 회복은 역사의 후퇴다 Chinese Academy of Social Sciences Professor and prominent sociologist Li Yinhe wrote in her blog that “Recovering the lifelong control is a regression of history

中国著名女性社会学家 中国社会科学院教授李银河在博客上指出,“恢复终身制是历史的倒退

중국을 마오(쩌둥) 시대로 돌아가게 만들 것”이라고 지적했다 It is returning China to the days of Ma Zedong

将会让中国回到毛(泽东)时代”

공산주의청년단(공청단) 기관지 중국청년보 산하 잡지 빙뎬(氷点)의 편집자였던 리다퉁(李大同)도 다음 달 개막하는 전국인민대표대회(한국의 국회 격)에 참석하는 천지닝(陳吉寧) 베이징(北京) 시장 등 베이징 인민대표 55명에게 공개서한을 보내 “개헌안에 반대표를 던질 것”을 호소했다 ” Li Datong, who is former editor-in-chief of a four-page weekly supplement of China Youth Daily, sent an official letter to 55 representatives of the people, including Beijing Mayor Chen Jining, appealing to vote against the Communist Party’s proposal when they attend the National People’s Congress scheduled to start next month

曾任中国共青团机关报下属《中国青年报》冰点栏目编辑的李大同也向出席将于下个月开幕的全国人民代表大会(相当于韩国的国会)的北京市市长陈吉宁等55名北京人大代表致公开信,呼吁“对修宪方案投反对票”

중국 여성 기업가 왕잉(王瑛)은 “임기제 삭제는 배반이자 시대 흐름에 역행하는 것”이라며 “내게 침묵을 요구해도 결코 침묵하지 않을 것”이라는 성명을 발표했다 Wang Ying, a female entrepreneur, issued a statement saying, “The removal of presidential term limits is a betrayal and going against the times

中国女性企业家王瑛发表声明说,“删除任期限制是背叛,是开时代倒车,”“即使要求我沉默,我也不会沉默”

미국에 망명한 톈안먼(天安門) 민주화운동 당시 학생지도자였던 왕단(王丹)은 100여 명의 중국 학자들과 함께 발표한 성명에서 “시진핑이 황제의 야심을 지니고 있음이 드러났다”고 말했다 I will not keep silent no matter how they require me to ” Tiananmen protest student leader Wang Dan, who exiled from China and now lives in the United States, said in his statement released with 100 Chinese scholars that “It has been revealed that Xi Jinping has had the ambition of becoming an emperor

天安门民主化运动时期的学生领袖 现流亡美国的王丹也和100余名中国学者一道发表声明称,“这暴露了习近平有皇帝野心”

일본 아사하신문은 27일 시 주석이 지난해 10월 19차 전국대표대회(당 대회) 직후 상하이(上海)에서 당 원로인 장쩌민(江澤民) 전 주석에게 임기 제한 철폐 의사를 밝혔는데 “절대 안 된다”는 장 전 주석의 강한 반발에 부딪혔다고 전했다 ” The Asahi Shimbun of Japan reported Tuesday that Xi Jinping met with a strong opposition from former Chinese President Jiang Zemin in October last year when he suggested scrapping the presidential term limits right after the 19th National People’s Congress

日本《朝日新闻》27日报道称,习近平曾在去年10月的第十九次全国代表大会(党代会)后不久,在上海向共产党元老 前国家主席江泽民表示想废除任期限制,但江泽民表示“绝对不行”,遇到了强烈反对

웨이보(중국판 트위터)에는 ‘나는 동의하지 않는다’ ‘이민’ ‘비행기에 탑승한다(登機)’ 등이 포함된 글까지 검열 대상이 됐다 Sensitive words, such as “disagree,” “emigrate” and “to board a plane,” have become the subject to censorship at China's Twitter-like social network Weibo

微博(中国版推特)上,包括“我不同意”“移民”“登机”等词汇的文章成为审查对象

임기 제한 삭제안 발표 이후 이민 검색어 수치가 급등했고, ‘비행기에 탑승하다’의 중국어 발음이 ‘(황제) 등극’과 같기 때문이다 This is because the number of the word “emigrate” being searched on the Internet has skyrocketed following the announcement of a list of proposed amendments to China’s constitution and the phrase “to board a plane” and “to ascend the throne” are homophonous

这是因为,在宣布建议删除任期限制后,有关移民的检索词使用迅速增加,而中文“登机”一词的发音与“(皇帝)登基”相同

종신제, 등극, 장기집권, 종신집권, 개헌반대 등의 단어는 검색조차 안 되고 있다 Words including lifelong control, ascension, long-term seizure of power and opposition to constitutional reform are blocked from Sina Weibo search results

终身制 登机 长期执政 反对修宪等词汇已经不能检索

영국 파이낸셜타임스(FT)는 중국 대표 포털 바이두(百度) 뉴스 직원을 익명으로 인용해 “최소 13개 인터넷 뉴스 기업이 당국으로부터 개헌 지지 기사를 상위에 올리라는 지시를 받았다”고 보도했다 “It, along with at least 13 other internet news companies, had received edicts from authorities to prioritize articles supportive of the proposed constitutional change,” The Financial Times quoted an unnamed source at Baidu

英国《金融时报》援引中国主要门户网站百度新闻匿名职工的话报道称,“至少有13家互联网新闻企业接到了当局要求在显著位置刊登支持修宪报道的指示”

“中, 남중국해 인공섬 ‘軍지휘센터’로 활용” China uses South China Sea islands as military control center

香港媒体:“中国把南中国海上的人工岛用作‘军事指挥中心’”

중국이 동남아시아 국가들과 영유권 분쟁을 겪고 있는 남중국해에 건설한 인공섬을 주변 군사시설을 지휘하는 센터로 이용하려 한다는 주장이 나왔다 （no translation）

有消息称,中国在与东南亚国家存在领土主权争议的南中国海上建设人工岛,并企图把这些岛当作指挥周边军事设施的中心

남중국해는 중국이 영유권을 주장하는 섬의 12해리 안에 미국이 군함을 진입시키는 ‘항행의 자유’ 작전을 지속하면서 군사적 긴장이 높아지고 있는 지역이다 （no translation）

随着美国持续实施“航行自由”行动 派遣军舰进入中国在南中国海上主张拥有主权的岛屿12海里内,南中国海上的军事紧张正在加剧

18일 홍콩 사우스차이나모닝포스트(SCMP)에 따르면 미국 전략국제문제연구소(CSIS)는 항공사진을 통해 남중국해 스프래틀리 제도(중국명 난사·南沙군도)의 피어리크로스 암초(융수자오·永暑礁) 북서쪽에 중국이 최근 송신탑과 고주파 레이더 설비 등 통신장비를 집중 배치한 사실을 확인했다 The U S Center for Strategic and International Studies confirmed that China recently deployed communications equipment such as transmitting tower, and high frequency radar equipment in the northwest part of Fiery Cross Reef in Spratly Island of the South China Sea, The Hong Kong South China Morning Post said on Sunday

据香港《南华早报》18日报道,美国战略国际问题研究所通过航空照片,确认了中国最近在南中国海斯普拉特利群岛(中国称南沙群岛)的FieryCrossReef(中国称永暑礁)的西北部,集中部署了电信塔和高频率雷达设备等通信装备

이 암초 10만 m² 부지에 길이 3000m 활주로뿐 아니라 폭격기와 공중급유기, 수송기를 위한 격납고도 설치됐다 Some 3,000-meter-long landing strip, not to mention a hangar for aerial tanker and air carriers, were deployed on the 100,000 square meter reef

在该暗礁上,中国在10万平方米的地面上不仅建造了长度达3000米的跑道,还为轰炸机 空中加油机 运输机等设立了机库

미국은 중국이 피어리크로스 암초뿐 아니라 스프래틀리 제도의 수비 암초(주비자오·渚碧礁), 미스치프 암초(메이지자오·美濟礁) 등 암초 7곳을 군사시설이 배치된 인공섬으로 조성하고 있다고 보고 있다 The United States believe that China is creating seven reefs including not only the Fiery Cross Reef but also the Subi Reef and Mischief Reef as an artificial island for military facility

美国同时认为,中国不仅在Fiery环暗礁上,而且也还把斯普拉特斯群岛的苏比暗礁(中国名渚碧礁) 美斯济夫暗礁(中国名美济礁)等7个暗礁建设成了部署军事设施的人工岛

SCMP는 중화권 군사 전문가들을 인용해 “피어리크로스 시설이 중국이 조성한 군사시설 간 통신을 중개하는 기지로 사용될 수 있다”고 관측했다 “The Fiery Cross facility may be used as a mediation base for communication among military facilities created by China,” the newspaper reported by citing military experts for the China region

《南华早报》引用中华圈军事专家的话分析称,“Fiery环暗礁上的设施,是中国建设的军事设施中被用作通信中转的基地”

이에 따라 미중 간 신경전이 거세지고 있다 As such, tensions are rising between the United States and China

这一形势正在加剧美中之间的神经战

호주 주재 미국대사로 내정된 해리 해리스 미 태평양사령부 사령관은 14일 미 하원 군사위원회 청문회에서 스프래틀리 제도의 군사 기지화를 우려하면서 “중국이 인공섬에 고성능 군사 방어 장비를 갖춰 영유권을 주장할 것”이라고 지적했다 Harry Harris, United States Navy Admiral, who has been designated as the U S ambassador to Australia, expressed concerns over militarization of the Spratly Islands during the House Armed Services Committee Hearing on Wednesday, saying, “China is attempting to assert de facto sovereignty over disputed maritime features by further militarizing its man-made bases”

被提名担任美国驻澳大利亚大使的美军太平洋司令部司令哈里·哈利斯14日在美国国会众议院军事委员会的听证会上,对斯普拉特利群岛的军事基地化表示忧虑,并指出,“中国将在人工岛上部署高性能军事防御装备后,声称拥有其主权”

中, 러시아판 사드배치… 美에 맞대응 China deploys Russian surface-to-air missile system

为对抗美国,中国拟部署俄罗斯版“萨德”

중국이 최근 ‘러시아판 사드’로 불리는 지대공 미사일 S-400을 배치해 배경에 눈길이 쏠린다 China’s recent deployment of Russian S-400 surface-to-air missile system is drawing attention about China’s motive

中国不久将部署被称为“俄罗斯版萨德”的S-400地对空导弹系统,其背景令人关注

배치 장소는 알려지지 않았지만 산둥(山東)반도에 배치할 경우 한반도가 레이더 탐지 범위에 포함돼 한반도 유사시 한미 군의 움직임을 견제하기 위한 포석이라는 관측이 나왔다 (（additional translation））（The S-400 is often compared to the U S 's Terminal High Altitude Air Defense system） China has not confirmed where the defense system is located However, if the S-400 is deployed anywhere in the Shandong Province, the Korean Peninsula will be within the radar surveillance range In this context, it has been speculated that China aims to check moving of South Korean and U S forces in time of emergency in the Korean Peninsula

分析认为,虽然部署地点尚不清楚,但如果选择在山东半岛部署,韩半岛将进入雷达的探测范围,一旦韩半岛发生战争,将成为牵制韩美军队动向的布子

19일 러시아 타스통신과 홍콩 밍(明)보에 따르면 러시아는 최근 중국에 미사일 통제소와 레이더 기지, 연료 공급 설비 등 S-400 지대공 미사일 시스템을 제공했다 According to Russia’s TASS and Hong Kong’s Ming Pao on Friday, Russia has recently delivered the S-400 surface-to-air missile system to China

据俄罗斯塔斯通讯社和香港《明报》19日报道,俄罗斯最近向中国提供了导弹控制中心 雷达基地 燃料供应设备等S-400地对空导弹系统

중국은 2014년 러시아로부터 S-400 3개 포대를 도입하기로 계약했고 이번에 첫 포대가 배치된 것이다 The S-400 system includes missile control station, radar station and fueling installations China and Russia signed a contract in 2014 to ship three sets of defense system, and the first system has been deployed this time

中国2014年签署了从俄罗斯引进3套S-400系统的协议,目前部署的是第一套

중국은 2019년까지 도입을 마무리할 계획인 것으로 알려졌다 China plans to complete deployment of all three systems by 2019

据悉,中国计划在2019年前结束S-400系统的引进

S-400은 사거리 400km, 최고 비행고도 185km에 이른다 The S-400 system has a maximum range of 400 kilometers and a maximum altitude of 185 kilometers

S-400的射程为400公里,最高飞行高速为185公里,

레이더는 700km 이내의 300개 표적을 추적할 수 있다 Its radar can track up to 300 targets within 700 kilometers

雷达可以追踪700公里以内的目标,

여러 고도와 사거리의 각종 전투기 및 미사일을 동시에 격추할 수 있다 It can shoot down multiple fighters and missiles flying at different ranges and altitudes simultaneously

可以同时击落不同高度和不同射程的战斗机和导弹

전자기가 교란되는 환경에서도 운용이 가능하다 The system is operable, while electric devices are disturbed

在电磁波受到干扰的情况下,它也能继续工作

S-400은 F-35 스텔스기 등 미국의 첨단 전투기 격추 능력이 있다는 평가를 받아 왔다 The S-400 has been eval‎uated that it can shoot down U S ’s latest fighters such as the F-35 stealth

S-400被认为具有击落F-35隐形战斗机等美国尖端战斗机的能力

사거리 200km, 최대 고도 150km인 사드(THAAD·고고도미사일방어체계)보다 위협적이라는 분석도 있다 and more threatening than the THAAD with a maximum range of 200 kilometers and a maximum altitude of 150 kilometers

还有分析认为,它比射程200公里 最大高度150公里的萨德更具威胁

밍보는 S-400이 푸젠(福建)성과 같은 중국 남부 연안에 배치되면 대만 전역을 사거리에 둘 수 있어 대만 공군을 공격할 수 있다고 지적했다 Ming Pao reported that, if the S-400 is located in the southern coast of mainland China, such as the Fujian Province, the entire Taiwan falls in the system’s range and China can attack Taiwanese air force

《明报》称,如果S-400部署在福建省等中国东南沿海,可以把台湾全境纳入射程,对台湾空军进行打击

최근 중국에서 대만 무력통일론이 나오는 상황에 주목한 것이다 This argument is based on a recent development in China where unification by force is taking a foothold

最近中国出现了武力统一台湾的说法,引起了关注

하지만 중국은 미국의 첨단 전투기 및 스텔스 순항미사일에 대응하기 위해 S-400 도입을 추진해 왔다는 관측도 나온다 In contrast, it has been suggested that China has imported the S-400 in order to destabilize U S ’s latest fighter and stealth cruise missile

但是,也有分析认为,中国为了应对美国的尖端战斗机和隐形巡航导弹,才推动S-400系统的引进

러시아도 블라디보스토크 등 북한 인접 극동지역에 S-400을 실전 배치했다 Russia also deployed the S-400 in far eastern areas near North Korean borders such as Vladivostok

俄罗斯也在符拉迪沃斯托克等靠近北韩的远东地区实战部署了S-400系统

中개혁개방 1번지 선전, 특구 장벽 허물었다 China removes historic border round Shenzhen economic zone

中国改革开放第一号深圳,撤销经济特区管理线

중국의 개혁개방을 상징하는 광둥(廣東)성 선전(深(수,천))시의 중국 제1호 경제특구와 나머지 본토 지역을 분할해 인적·물적 이동을 통제해온 장벽이 설치 36년 만에 완전히 역사 속으로 사라졌다 The wall that has been restricting the movement of people and materials by separating the 1st special economic zone and the rest of the mainland areas of Shenzhen, which represents the Chinese economic reform, has vanished into the history after 36 years since its designation

广东省深圳市是中国改革开放的象征。曾经将中国这第一个经济特区和大陆其他地区分隔 在人员物资移动方面进行限制的特区管理线,在设立36年后彻底成为历史

더 이상 장벽이 필요 없을 정도로 중국 경제가 성장했음을 대내외에 알리는 상징적인 사건으로 평가된다 It is eval‎uated as a symbolic incident, which proves China’s economy has grown so much that it no longer needs walls

分析认为,这一决定具有很大的象征意义,它向国内外宣告,中国经济已经发展到了不再需要隔离线的程度

16일 중국 매체들에 따르면 국무원은 선전시 경제특구에 존재해 왔던 ‘선전경제특구관리선(제2관문선)’을 없애기로 공식 결정했다 （no translation）

据中国媒体16日报道,中国国务院正式决定,撤销深圳经济特区管理线(第二道关门)

“시내의 물리적 경계를 없애 해당 지역의 통합을 촉진할 수 있도록 해달라”는 광둥성 정부의 요청을 승인하는 형식이었다 （no translation）

广东省政府此前请求“取消这一市内的实质性边境,以促进相关地区的协调发展”,并得到了中央政府的批准

선전시 남쪽의 홍콩과 경계선을 면한 일부 지역(327km²)은 덩샤오핑(鄧小平)이 1978년 개혁개방을 선언한 지 2년 뒤인 1980년 중국의 첫 경제특구로 지정됐다 Partial region in southern Shenzhen neighboring the border line with Hong Kong was designated as China’s first special economic zone in 1980, two years after late Chinese leader Deng Xiaoping announced the Chinese economic reform in 1978

邓小平1978年宣布改革开放,并在两年后的1980年,将深圳市南部与香港相邻的部分地区(面积327平方公里),宣布为中国头一个经济特区

인구 3만 명의 작은 어촌 마을이었던 선전은 지금 세계적 정보기술(IT) 기업이 몰려 있는 중국의 실리콘밸리로 발돋움했다 Shenzhen was once a small fishermen’s village with about 30,000 population, but it has now grown into the Silicon Valley of China, in which the world-class information technology (IT) companies have gathered up

当时仅是一个3万人口小渔村的深圳,如今已成为中国的硅谷,集中了众多世界级信息技术企业

중국은 선전에 경제특구를 지정하면서 특구 내 외국인의 자유로운 투자 등 경제 활동, 공장 설립, 면세 등을 보장했다 China guaranteed economic activities of foreigners, such as free investment, establishment of factories and tax exemption, in the Shenzhen economic zone

中国在宣布深圳成为特区后,为在特区内投资的外国人提供了自由投资等经济活动 设立工厂 免税等优惠条件

하지만 특구 밖 중국인은 허가가 있어야 출입이 가능하도록 했다 But Chinese people outside of the zone could only enter and leave it under permission

但是,特区外的中国人必须经过批准才能出入特区,

이를 위해 1982년 특구 주위에 136km에 달하는 철조망을 쳤다 Along with 136-kilometer wire fence around the zone since 1982, 163 guard posts

为此还在1982年在特区周围设立了长达136公里的铁丝网,

무장경찰이 지키는 초소 163곳과 검문소 10곳이 설치됐다 and 10 checkpoints protected by armed police were installed

并设立了由武装警察值守的163个哨所和10处检查站

경제특구 남쪽은 1997년에야 반환된 홍콩이었기 때문에 더더욱 통제가 심했다 As Hong Kong, the southern part of the zone, was returned to China in 1997, the Shenzhen economic zone has even more strict restrictions

由于经济特区以南就是1997年才归还中国的香港,因而管制更加严格

사람들은 홍콩과 선전의 경계선을 ‘1선 관문’, 선전특구와 내륙의 경계선을 ‘2선 관문’이라 불렀다 People called the border line between Hong Kong and Shenzhen “the first gateway,” and called the border line between the Shenzhen economic zone and the mainland China “the second gateway

人们把香港与深圳之间的边境线称为“一线关”,把深圳与内地之间的管理线称为“二线关”

2선 관문의 존재는 선전의 중국 내 독특한 위상을 웅변하는 동시에 중국이 외부에 완전히 문을 연 것은 아니라는 점도 보여줬다 ” The existence of the second gateway not only spoke for the unique status of Shenzhen in China, but also showed that China has not opened its doors completely to the outside world just yet

二线关的存在,说明了深圳在中国国内独特的地位,同时也说明,中国并没有完全对外打开大门

2선 관문은 급격하게 서구 경제와 문화에 영향을 받아 중국 사회주의가 위기에 빠지는 일을 막는 역할을 했다 The second gateway was quickly influenced by the Western economy and culture, and prevented Chinese socialism from being in danger

二线关还发挥了阻止中国社会主义因迅速受西方经济和文化影响而陷入危机的作用

경제의 급속한 발전으로 2010년 선전경제특구는 선전시 전체로 확대됐다 Thanks to the rapid economic growth, the Shenzhen special economic zone was expanded to the entire city of Shenzhen in 2010

随着经济的迅速发展,深圳经济特区在2010年扩大到深圳全市

선전시 전역에 지하철이 다니면서 장벽이 유명무실해졌다 As subway is operated all over the city of Shenzhen, the walls became useless

随着深圳市地铁贯通市内各地,原有的管理线变得有名无实

오히려 장벽과 검문소 운영에 매년 수천만 위안이 들어갔고 교통 등 도시 발전의 장애물이 됐다 Instead, the walls and checkpoints cost tens of millions of yuans every year, becoming obstacles for urban development

相反,管理线和检查站的运营每年还需要投入数千万元人民币,并成为交通等城市发展的障碍

시민들이 오갈 때 검문을 거쳐야 한다는 점도 불편을 초래했다 The fact that the citizens had to be inspected when going back and forth also caused inconvenience

市民们来来往往必须经过检查,也带来了不便

2013년부터 장벽과 검문소 순찰도로 등에 대한 철거가 시작돼 현재도 진행되고 있지만 공식적으로 중국과 선전특구를 분할했던 이 경계선은 계속 존재해 왔다 The walls, checkpoints and patrol roads have been being taken down since 2013 and they are still being taken down, but this border line, which officially divided China and Shenzhen, still exists

虽然取消管理线和检查站巡察路的工作早在2013年就已开始,目前也在进行之中,但在制度上,分割深圳特区与中国大陆其他地区的这一隔离线仍然存在

중국 매체들은 “이번 조치로 선전이 다시 한번 개혁개방의 최전선에 섰다”며 기대감을 나타냈다 The news media of Chia reported that this action made Shenzhen to take the lead in Chinese economic reform once again, showing their anticipation

中国媒体称,“由于这一措施,深圳再次成为改革开放的最前线”,表现出期待

다만 국무원은 이번 조치 결정문에서 “선전과 홍콩 마카오 간 경계에 있는 1선 관문에 대한 관리 통제를 강화하라”고 지시했다 But the Chinese State Council ordered in the decision letter of this action to reinforce management and restriction about the first gateway located in the border line between Shenzhen and Hong Kong/Macau

不过,国务院在相关决定中指示,“要加强深圳与香港 澳门之间边境上的一线关的管理管辖”

美-中무역전쟁 일촉즉발 U S -China trade war appears to be imminent

美中贸易战一触即发

중국의 최대 전자상거래 업체 알리바바의 대표 쇼핑몰 타오바오(淘寶)가 또다시 미국 정부의 악덕시장 블랙리스트에 올랐다 Taobao, the marketplace of China’s biggest e-commerce Alibaba, has yet again made the U S government’s annual list of the world’s most notorious markets

中国最大的电子商务企业阿里巴巴的代表性卖场淘宝再次被列入美国政府的不良市场黑名单

미국과 중국이 무역 금융 분야에서 티격태격하는 가운데 중국이 지난해 사상 최대 규모의 대미 무역흑자를 기록하면서 미중 무역전쟁이 폭풍전야라는 관측도 나왔다 While the United States and China continue to wrangle over trade, some watchers say that last year’s record-high trade surplus of China with the United States may soon trigger a trade war between the two superpowers

有分析认为,美国和中国在贸易金融领域磕磕碰碰之际,中国去年对美贸易创下有史以来的最大顺差,美中已处于贸易战争的前夜

14일 BBC 중문판에 따르면 미국 무역대표부(USTR)는 타오바오가 지식재산권 침해 상품으로 가득 차 있다며 2016년에 이어 지난해에도 악덕시장 명단에 포함시켰다 Last year, the U S Trade Representative (USTR) put Taobao on its blacklist for the second year in a row over suspected counterfeits on the shopping platform in violation of intellectual property (IP) rights, BBC China reported Sunday

据BBC中文网站14日报道,美国贸易代表部声称淘宝上充斥着侵犯知识产权的商品,继2016年之后,去年再次把淘宝列入不良市场名单

USTR는 미국의 지식재산권을 침해하고 미국 노동자에게 심각한 피해를 입히는 온라인 시장 25개와 오프라인 시장 18개를 지난해 악덕시장 리스트에 올렸다 The USTR placed 25 online markets and 18 physical markets on its list of notorious markets for sales of pirated and counterfeit goods that infringe on IP rights and cause severe damage to U S workers

美国贸易代表部把侵犯美国知识产权和给美国劳工带来严重损害的25家在线商家和18家线下市场列入黑名单

이 중 중국 온·오프라인 시장은 타오바오 등 9개로 20%를 차지했다 Taobao and another eight Chinese online and offline markets were included in the list, taking up 20 percent share

其中中国的在线和线下市场,包括淘宝在内共有9家,占总数的20%

USTR는 타오바오의 짝퉁 상품 퇴치 노력은 인정했으나 “가짜 상품의 규모를 보여줄 객관적인 지표를 공개하지 않았고 짝퉁 판매량이 줄었다는 사실을 객관적으로 입증하지 못했다”고 재지정 이유를 밝혔다 알리바바는 즉각 반발하고 나섰다 Alibaba did not hesitate to defend itself,

美国贸易代表部虽然承认淘宝为驱逐假货作出了努力,但仍然以“没有公开显示假冒商品规模的客观指标,无法客观证明假货销售量的减少”为由,再次将其列入黑名单，阿里巴巴立即表示反对

알리바바는 “무역 보호주의가 고개를 들어 고도로 정치화된 환경에서 도널드 트럼프 행정부로부터 점수를 따려는 USTR의 희생양이 됐다 saying that it has become “a scapegoat for the USTR to win points in a highly-politicized environment”

阿里巴巴指出,“在贸易保护主义抬头 高度政治化的环境中,阿里巴巴成了企图在特朗普政府拿到高分的美国贸易代表部的牺牲品

USTR의 조치는 지식재산권을 보호하려는 것이 아니라 미국 정부의 지정학적 목표를 달성하기 위한 수단”이라고 지적했다 and that the USTR’s action is “not about intellectual property protection, but just another instrument to achieve the U S government’s geopolitical objectives（no translation）

美国贸易代表部的措施不是为了保护知识产权,而是实现美国政府地缘目标的工具”

중국 해관총서(세관)가 12일 공개한 수치에 따르면 중국의 대미 무역흑자는 2758억 달러(약 293조 원)로 사상 최고치를 기록했다 （no translation）

根据中国海关总署12日公布的数字,中国对美国的贸易顺差为2758亿美元(约合293万亿韩元),是有史以来的最高纪录

미국의소리(VOA) 중문판은 “이런 수치는 미중 무역관계의 긴장을 완화시키는 데 도움이 되지 않을 것”이라며 “미중 무역전쟁이 폭풍전야”라고 지적했다 （no translation）

“美国之音”中文版指出,“这一数字无助于缓和美中贸易关系的紧张，美中已处于贸易战争的前夜”

이달 초 알리바바의 자회사로 모바일결제 업체인 앤트파이낸셜이 미국의 송금회사 머니그램을 인수합병하려던 계획이 미국 외국인투자심의위원회(CFIUS)에 의해 거부되기도 했다 Early this month, the Committee on Foreign Investment in the United States (CFIUS) rejected the plan of Ant Financial, online payment services provider and affiliate of Alibaba, to acquire the U S -based money transfer company MoneyGram, feeding speculation that Washington is about to start a trade war with Beijing by reining in its representative conglomerate Alibaba

本月初,阿里巴巴的子公司 移动结算企业蚂蚁金服收购美国汇款公司“速汇金”的计划,也遭到了美国外资投资委员会(CFIUS)的拒绝

미국이 중국을 대표하는 알리바바를 제재하면서 무역전쟁에 시동을 걸고 있다는 관측이 나오는 이유다 （no translation）

正因如此,有分析认为,美国制裁代表中国的阿里巴巴,标志着贸易战争已经打响

미국 정부 당국은 중국산 철강 제품 등의 수입이 미국 국가 안보에 손해를 얼마나 끼치는지 조사를 끝낸 뒤 11일 트럼프 대통령에게 보고했다 （no translation）

美国政府有关部门结束了就进口中国产钢铁产品等进对美国国家安全有多大损害进行的调查,11日向特朗普总统提交了报告

트럼프 대통령은 90일 안에 이에 대해 어떤 정책을 내놓을지 결정한다 （no translation）

特朗普将在90天内决定采取什么政策

미국은 또 이달 중 중국 상품에 대한 각종 조사 결과를 발표할 예정이라고 VOA 중문판이 전했다 （no translation）

“美国之音”报道称,美国还将在本月内公布对各种中国商品的调查结果

전문가들은 미국이 중국 상품에 대한 관세를 올리거나 수입 제한 조치를 실제로 본격화하면 중국 역시 같은 방식으로 보복할 가능성이 높다고 본다 Experts say that U S measures to raise tariffs on products made in China and import restrictions are likely to be met with China’s countermeasures in the same manner

专家们认为,如果美国针对中国商品提高关税或采取事实上的大规模限制措施,中国很可能会以同样方式进行报复

미국의 대중 주요 수출 상품인 보잉 항공기, 자동차, 집적회로, 대두뿐 아니라 할리우드 영화에도 규제를 가할 것이라는 우려가 미국 내에서 나왔다 Some U S pundits are voicing concern that in response, China may tighten regulations on key U S exported goods such as Boeing’s airplanes, automobiles, integrated circuits, soybeans as well as Hollywood films

美国国内担心,不仅波音飞机 汽车 集成电路 大豆等美国主要对华出口商品受到影响,中国还会对好莱坞电影进行限制

中측 “사드 해결 없인 한중관계 회복 없어” China urges S Korea to address THAAD issue

中方:“不解决萨德问题,中韩关系就不可能恢复”

“한반도 사드(THAAD·고고도미사일방어체계) 배치가 중한 관계의 걸림돌이다 “The deployment of the THAAD system in South Korea is an obstacle to China’s relations with the nation

“在韩半岛部署的萨德是中韩关系的绊脚石

사드 문제에 대한 철저한 해결 없이는 한중 관계 회복은 없다 Restoring the relationship would not be possible without first resolving THAAD issues thoroughly

萨德问题如果得不到彻底的解决,中韩关系就不可能恢复”

” 웨이웨이(魏葦) 중국인민외교학회 부회장은 11일 베이징에서 열린 ‘한중 미래발전 싱크탱크 고위급 포럼’에서 이같이 말했다 ” Wei Wei, the vice president of the Chinese People’s Institute of Foreign Affairs (CPIFA), was quoted as saying at the Korea-China Future Development Think Tank High-level Forum, which was held in Beijing on Monday

中国人民外交学会副会长魏苇11日在北京举行的“韩中未来发展智库高级别论坛”作出了上述表示

문재인 대통령의 방중을 이틀 앞둔 시점에 양국 간 상호 이해와 우호 분위기를 높이고 관계 개선 방향을 논의하기 위해 마련된 포럼이었지만 중국 측 참석자들은 사드 문제를 집중 거론했다 While the forum was designed to promote mutual understanding and amity between the two countries and discuss ways to improve their relations, the focal point of discussion was the Terminal High Altitude Area Defense missile system

在文在寅总统访华两天之前举行的这一论坛,目的是提高两国间的相互理解和友好氛围 讨论改善关系的方向,但中方与会者集中谈到了萨德问题

웨이 부회장은 관영 환추(環球)시보가 지난달 29일 제기한 ‘3불(不) 1한(限)’에 대해서도 언급했다 The vice president of CPIFA also mentioned the “Three Bans, One Restriction,” a phrase proposed in an article in the Chines state-run newspaper Global Times on October 29

魏苇谈到了中国官方媒体《环球时报》11月29日提出的“三不一限”问题

3불 1한은 양국 합의 사항에 3불(사드 추가 배치하지 않고, 미국 미사일방어체계(MD)에 가입하지 않으며, 한미일 3국 동맹으로 발전하지 않는다)뿐 아니라 사드 사용 제한(1한)도 포함돼 있다는 주장이다 The phase refers to China’s stance that South Korea and China have reached an agreement on a restriction of making use of the THAAD system as well as on three bans (deploying additional THAAD systems, joining the U S Missile Defense system and advancing a tripartite alliance between South Korea, the U S and Japan)

“三不一限”的主张,不仅包括两国协议中的三不(不追加部署萨德系统 不加入美国导弹防御系统 不发展韩美日三国同盟),还包括限制萨德的使用

웨이 부회장은 “중국 당국도 직접적으로 말하지는 않으나 3불 1한 입장은 같다”고 말했다 （no translation）

魏苇表示,“中国当局虽然没有直接说出口,但对于三不一限的立场是一致的”

한국의 한 외교 소식통은 “양국 합의에 결코 사드 사용 제한, 즉 1한이 없었는데 여론전을 펴는 것 같다”고 말했다 （no translation）

韩国某外交消息人士表示,“两国协议没有限制萨德的使用,即没有一限,这是(中方)在打舆论战”

위훙쥔(于洪君) 전국정치협상회의 외사위원회 위원은 “복잡한 사정이 있지만 지난해 7월 한미의 사드 배치 결정은 적절하지 못했다”며 “The situation is complicated, but their decision to deploy the THAAD system in South Korea in July was inappropriate,” said a member of the Committee of Foreign Affairs of the Chinese People’s Political Consultative Conference

中国全国政治协商会议外事委员会委员于洪君表示,“虽然有复杂的事由,但去年韩美关于部署萨德的决定是不合适的

“이웃 국가의 이익 침해를 고려해야 한다”고 말했다 “South Korea should have considered the possibility of infringing upon the interests of their neighbors I am afraid that the relations have cooled off owing to the THAAD issues The responsibility lies in South Korea”

应该想到,这是对邻国利益的侵害”

위 위원은 “사드 문제로 한중 관계가 냉각된 것은 안타깝다”며 “책임은 한국에 있다”고 말했다 （no translation）

他表示,“中韩关系因为萨德问题降温,让人感到难过，责任在于韩国“

우쓰커(吳思科) 차하얼학회 국제자문위원도 “사드 도입을 통해 한반도에서 평화를 추구하겠다는 결정은 도움이 되지 않는다”고 말했다 （no translation）

察哈尔学会国际顾问委员吴思科也表示,“引进萨德,对在半岛构建和平没有任何帮助”

자오커진(趙可金) 칭화(淸華)대 사회과학학원 부원장은 “한국이 중국의 사드 반대를 과소평가하고 있다”며 “사드를 배치하는 이유를 알면서도 진실을 말하고 있지 않다”고 말했다 （no translation）

清华大学社会科学院副院长赵可金表示,“韩国低估了中国对萨德的反对，韩国明知道部署萨德的原因,但没有讲真话”

미국의 MD 편입 등을 지적한 것으로 풀이된다 （no translation）

分析认为,他是指韩国加入了美国的防导体系

이어 자오 부원장은 “한미 동맹이 계속 유지되면 한중 관계에도 걸림돌이 될 것”이라고 말했다 （no translation）

赵可金还表示,“如果继续维持韩美同盟,它将成为韩中关系的绊脚石”

한미일 3국 동맹이 아닌 ‘한미 동맹’ 자체를 한중 관계에 미치는 부정적인 요소로 지목한 것은 이례적이다 （no translation）

指称“韩美同盟”本身而不是“韩美日三国同盟”,是对韩中关系产生负面影响的因素,这是十分罕见的

배기찬 안보전략연구원 고문은 “북한이 핵전력 완성을 공언한 상황에서도 중국은 한결같이 한국에 강경한 입장”이라며 “한국이 핵무장이라도 하기를 바라는 것이냐”고 반박했다 （no translation）

韩国安保战略研究院顾问裴基灿(音译)反驳道:“在北韩宣称完成核武装的情况下,中国仍对韩国采取强硬的立场,难道中国希望韩国也走向核武装吗”

양측의 날 선 공방이 있었지만 문 대통령의 방중을 관계 개선의 기회로 삼아야 한다는 주문도 많았다 （no translation）

虽然双方攻防十分尖锐,但也就应把文在寅总统访华当作改善两国关系的机会进行了很多探讨

한팡밍(韓方明) 중국 차하얼학회 회장(전국정협 외사위원회 부주임)은 “최근 (사드) 갈등은 양국의 이익에 부합하지 않는다”면서 “이번 양국 정상회담이 중요한 관계 개선의 계기가 되기를 기대한다”고 말했다 （no translation）

中国察哈尔学会会长(全国政协外事委员会副主任)韩方明表示,“最近的(萨德)矛盾不符合两国的利益,期待此次两国元首会谈成为改善这一重要关系的契机”

박은하 외교부 공공외교 대사는 “한 분야에서 발생한 갈등이 확산되지 않도록 관리할 필요가 있다는 것을 확인해 주었다”고 말했다 （no translation）

韩国外交部公共外交大使朴恩河(音译)表示,“论坛一致认为,某一领域发生的矛盾需要妥善管理,使它不扩散”

北에 화난 中, 유엔결의 넘어 독자제재 China slaps sanctions on N Korea on top of UNSC resolutions

中国生了气,超出联合国决议单方面制裁

중국 정부가 유엔 안전보장이사회 제재 이행을 넘어 여행 금지라는 대북 독자 제재를 취한 배경에는 시진핑(習近平) 중국 국가주석의 분노가 있다는 관측이다 （no translation）

北韩分析人士认为,中国政府超出执行联合国安理会决议的范围,下令实行禁止赴北韩旅游的单方面制裁,其背景是中国国家主席习近平对北韩感到愤怒

시 주석은 최근 쑹타오(宋濤) 공산당 대외연락부장을 북한에 특사로 보내 김정은 노동당 위원장에게 메시지를 보내려 했으나 거절당했다 （no translation）

习近平不久前派中国共产党对外联络部长宋涛访问北韩,想向北韩劳动党委员长金正恩转达口信,但遭到了拒绝

미국 정부가 올해 8월 자국민의 북한 여행을 금지한 데 이어 중국 정부도 제한적으로 북한 여행 금지 조치를 내려 북한에 상당한 압박이 될 것으로 전망된다 The Chinese government has limitedly banned its people from traveling to North Korea following a U S ban on its citizens travelling to the reclusive country, putting a strain on North Korea’s finances

在美国政府今年8月禁止本国公民前往北韩旅行后,中国政府也下令限制赴北韩旅行,将对北韩形成相当大的压力

북한은 그동안 관광을 통해 매년 4400만 달러(약 478억 원)어치의 외화를 벌어들인 것으로 알려졌다 이 중 중국이 80%를 차지한다 North Korea reportedly has been earning 44 million U S dollars annually from tourism, with China taking up 80 percent of the total

据悉,北韩每年通过旅游项目赚取相当于4400万美元(约合478亿韩元)的外汇其中,中国占80%

중국 국가여유국이 2012년 마지막으로 공개한 통계에 따르면 23만7000명의 중국인이 북한을 여행했다 According to the statistics released by the China National Tourism Administration in 2012, 237,000 Chinese travelled to North Korea

根据中国国家旅游局2012年最后一次公开的统计,有23.7万名中国人赴北韩旅游

하지만 28일 북한 여행객이 집중된 단둥(丹東)과 선양(瀋陽)이 포함된 랴오닝(遼寧)성과 지린(吉林)성의 북한 여행은 허용한다는 얘기가 나온 만큼 실효성은 두고 봐야 한다는 지적도 있다 Some point out that the effect of the travel ban remains to be seen as some regions, where most of the Chinese travelers to North Korea come from, including Dandong, Shenyang, Liaoning Province and Jilin Province are exempt from the ban

但是,随着28日有消息说包括北韩游客集中的丹东和沈阳在内,辽宁省和吉林省仍在允许赴北韩旅游,限令的实效性有待观望

이외 지역에서는 수요 감소로 이미 한동안 북한 관광객을 모집하지 않은 여행사도 적지 않다 Other regions are seeing a reduction in demands, resulting in travelling agencies not offering travelling packages to North Korea

此外,由于需求减少,上述地区有不少旅行社已经有一段时间不再募集游客

이날 본보가 확인한 결과 베이징(北京)의 대형 A 여행사는 “북한 여행 상품은 계속 없었고 이번에 (금지) 통지를 받지 못했다”고 전했다 “We do not have travelling packages to North Korea and have not received any notices banning travel to the North,” said a large travelling agency in Beijing

据本报今天确认的结果,北京的A大型旅行社表示,“北韩游旅行商品还是没有,这一次没有接到(禁止)通知”

B 여행사는 “다음 달 7일 단둥을 출발하는 5일짜리 여행 상품이 있고, 다음 달 중순에는 왕복 비행기 여행 상품이 있다”면서도 “We have a five-day package departing from Dandong to North Korea on December 7 and a shuttle flight from Dandong to North Korea in the middle of December,” said another agency

B旅行社回答,“下个月7日有从丹东出发的(北韩)5日游商品,下个月中旬有往返双飞的(北韩游)商品

“예약할 수 있을지는 불확실하다”고 답했다 “But we are not sure if making reservations would be possible”

但不清楚能不能预约”

산둥(山東) 지역 여행사도 그동안 북한 여행 상품이 없었다는 반응이었다 An agency in Shandong region said they have not offered travelling packages to North Korea for some time

山东地区的旅行社也都表示,没有赴北韩的旅行团

선양 지역 여행사는 “다음 달 16일에 4일짜리 여행상품이 있다”며 “하루 여행(신의주)은 언제든 출발 가능하다”고 답했다 An agency operating in Shenyang said that it has a four-day package to North Korea starting on December 16 but a day’s trip to Sinuiju can leave anytime,

沈阳地区的旅行社表示,“下个月16日有4日游的旅游团(新义州)一日游哪天都能出发”

지린 지역 여행사는 “겨울에는 북한 여행 상품이 없다”고 밝혔다 while the agency we spoke to in Jilin Province said that it does not have travelling packages to North Korea during winter times

吉林地区的旅行社表示,“冬天没有赴北韩的旅游团”

中학자 “중국, 주저말고 한미와 北 급변사태 논의해야” ‘China should talk with Korea and the U S ,' says a Chinese scholar

中国学者:“中国不应犹豫,应与韩美磋商北韩剧变事态”

쑹타오(宋濤) 공산당 대외연락부장이 시진핑(習近平) 중국 국가주석의 특사로 방북하기 전날 중국의 명문대 교수가 서울에서 열린 한국 정부 주최의 국제포럼에서 북한의 체제 붕괴 등 급변사태에 대비해 한미중 3국이 대화해야 한다고 주장했다 A day before Song Tao, the head of the International Liaison Department of the Communist Party of China, visited North Korea as Chinese President Xi Jinping’s special envoy, a professor at one of China’s most prestigious universities earned people’s attention in Seoul by speaking about the need for trilateral dialogue in preparation for sudden changes in circumstances such as the possible collapse of the North Korean regime

在中国共产党对外联络部长宋涛作为中国国家主席习近平特使访问北韩的前一天,中国名门大学的一名教授在由韩国政府在首尔主办举行的国际论坛上主张,为防范北韩体制崩溃等剧变事态,韩美中三国应开展对话

북한의 핵·미사일 도발에 대한 중국 학계 내부의 반발이 확산되고 있다는 사례로 주목된다 His comment suggests that Chinese scholars are increasingly voicing their opposition to the nuclear and missile threats from North Korea

这一事例说明,围绕北韩核武器和导弹挑衅,中国学界内部的反对之声正在扩散,引起了外界的关注

상하이(上海) 소재 퉁지(同濟)대 정치및국제관계학원 샤리핑(夏立平) 원장(사진)은 16일 서울 그랜드힐튼호텔에서 열린 ‘2017 동북아 협력포럼’에서 “중국은 더 이상 주저하지 말고 미국 한국과 북한의 급변사태에 대한 논의에 나설 때”라고 밝혔다 “China should no longer be hesitant in discussing abrupt changes that can possibly occur in North Korea with the United States and Korea,” said Xia Liping, dean and professor of School of Political Science & International Relations at Tongji University in Shanghai, at the “2017 Northeast Asia Peace and Cooperation Forum” held at Grand Hilton Seoul last Thursday

上海同济大学政治和国际关系学院院长夏立平(照片)16日在首尔大希尔顿饭店举行的“2017东北亚合作论坛”上表示,“中国不能再犹豫不决,现在是时候与美国和韩国就北韩的剧变事态进行磋商了”

이어 3국의 ‘비상 계획 대화’를 제안하면서 △북한 체제가 붕괴될 때 누가 북한의 핵무기를 통제할 것인가 △북한의 난민 문제를 어떻게 다룰 것인가 △위기 시 북한의 국내 질서를 회복하는 책임을 누가 질 것인가 △위기 후 한반도의 정치적 정돈 등을 의제로 제시했다 Proposing to hold a trilateral “emergency plan dialogue,” he suggested agendas include which party would control in the case where the North Korea regime collapses how we should handle the refugee issue of the North which party should take the responsibility of restoring national order within North Korea in times of urgency and how the Korean Peninsula can be politically stabilized after a crisis

他还提议三国举行“紧急计划对话”,提出了北韩体制崩溃时谁来掌控北韩的核武器 如何处理北韩难民问题 发生危机时谁来负责恢复北韩国内的秩序 危机后如何政治整顿韩半岛等议题

그는 “중국도 보다 적극적으로 참여한 가운데 제재가 강화되고 있지만 북한이 핵을 포기할 것이라는 전망에는 비관론이 많다”면서 “Though sanctions on the North are being strengthened with China’s more active participation, pessimistic views prevail on whether North Korea will drop nuclear development as a result,” said the scholar

他表示,“由于中国也积极参与,对北韩的制裁正在得到加强,但在北韩弃核这一前景上,很多人持悲观论

“북한이 중국의 외교적 노력을 무시하면서 핵과 미사일 도발을 계속하는 사태의 비중과 상황의 위험성 등을 고려할 때 이제 중국은 주저 없이 미국 한국과 진지한 대화에 나설 때”라며 이같이 주장했다 “Considering that North Korea continues to carry out nuclear and missile provocations despite China’s diplomatic efforts, now is the time for us to start a serious dialogue with the United States and Korea without hesitation

考虑到北韩无视中国的外交努力,并继续进行核武器和导弹的挑衅等事态的比重和状况的危险性,现在中国应毫不迟疑地与美国和韩国进行真诚的对话”

샤 원장은 이 같은 내용은 중국으로서는 매우 골치 아픈 주제들이지만 미중이 이와 관련해 대화와 협상을 벌이는 것은 양국 간 전략적 교착상태를 푸는 데도 도움이 될 것이라고 덧붙였다 ” Xia added that as much as such agendas are tricky in nature, dialogue and negotiation on them will help the United States and China to ease the strategic stalemate in their relations

夏立平表示,上述内容是中国感到感到非常头痛的议题,但美中就此展开对话和磋商,将有助于打开两国战略胶着状态

시진핑 특사 쑹타오 방북“중국의 (대북) 특사가 목표를 진전시키길 바라고 있다 Chinese envoy should warn against N Korea's nuclear ambitions Song Tao, head of the Chinese Communist Party’s International Liaison Department, arrived in North Korea Friday as a special envoy of President Xi Jinping in a 4-day trip

习近平的特使宋涛访问北韩“中国(赴北韩)特使希望目的取得进展”

한국 측 이도훈 외교부 한반도평화교섭본부장 역시 “지금 시점에 상당히 의미를 부여할 수 있을 것 같다”며 이날 방북한 중국 쑹타오(宋濤) 대외연락부장이 얼어붙은 대북 관계를 녹일 메신저로 나서 주길 기대했다 ” 17일 제주도에서 열린 한미 북핵 6자회담 수석대표 협의에 참석한 조셉 윤 미 국무부 대북정책특별대표는 회담 후 기자들과 만나 이렇게 말했다 （no translation）

北韩核问题六方会谈韩美两国的首席代表磋商17日在济州岛举行,美国国务院北韩政策特别代表尹汝尚在会谈结束后对记者作出了上述表示韩方首席代表 外交部韩半岛和平交涉本部长李道勋也表示,“在当前时间点,应该赋予它相当大的意义,”期待当天赴北韩访问的中国共产党对外联络部长宋涛能够融化冰冻的涉北韩关系

실제 양측 대표는 두 달 넘게 ‘도발 휴지기’를 이어오고 있는 김정은의 의중을 분석했다 （no translation）

事实上,韩美代表就北韩持续两个多月的“挑衅休眠期”中金正恩的意图进行了分析

（（additional translation））This is the first minister-level visit in two years since Politburo Standing Committee member Liu Yunshan paid a visit to North Korea in October 2015 The Chinese government said the main objective of Song’s visit was to report the North on the 19th National Congress of the Communist Party of China But the talks will likely be centered on resolving the North Korean nuclear issue as the visit comes after U S President Donald Trump’s travel to South Korea, China and Japan and his talks with South Korean President Moon Jae-in and Chinese President Xi Jinping on ways to resolve nuclear crisis with North Korea Song’s visit to North Korea is expected to be an important turning point in resuming talks with the North Song will reportedly deliver a letter or verbal message from President Xi to reiterate China’s firm commitment to “denuclearization on the Korean Peninsula” and show that Xi is willing to improve relations with North Korea in his second term as president Song could meet with North Korean leader Kim Jong Un and persuade him into suspending nuclear and missile tests and coming to the negotiating table If North Korea responds positively to China’s suggestions, a summit meeting between China and North Korea that has not been held in six years since Kim assumed power could take place

（漏译）

특히 쑹 부장의 방북을 계기로 북한을 6자회담 등 다자외교 채널로 끌어들일 수 있을지 논의했다고 한다 （no translation）

据悉,他们尤其对以宋涛访问北韩为契机,能否把北韩拉进六方会谈等多边外交渠道进行了磋商

도널드 트럼프 미 대통령 역시 전날 트위터에 중국의 특사 파견과 관련해 “큰 움직임”이라고 평가했다 In the meantime, Trump showed his expectation, calling the visit “a big move” on his twitter accounts Thursday The United States lowered the threshold for resuming talks with North Korea in time for the Chinese envoy’s visit to the North

美国总统特朗普前一天也在推特上就中国派特使前往北韩一事,认为这是一个“大动作”

（（additional translation））U S Defense Secretary James Mattis also stated the same day that North Korea would have a change for dialogue if it suspends nuclear tests and development and export of weapons The United States reportedly asked China to deliver its message to the North that the regime could be in big trouble if it ignores deterrence capability of the United States and the North should proclaim its will to give up nuclear program in exchange for dialogue But the situation is not optimistic as North Korea said Friday on Rodong Sinmun, the official newspaper of the Workers' Party of Korea, that, “We will never put issues related to our interest and the safety of our people on the negotiating table ” If Kim eventually refuses to accept China’s suggestions, Xi will likely put further pressure on the North by cutting off all shipments of oil to the country This could spoil the current mood for dialogue, making nuclear crisis with North Korea even worse China remains to be North Korea’s only ally as of now, but it cannot forever side with the North if Beijing decides that Pyongyang is not in its interest anymore Chinese special envoy’s visit to the North should not give the regime time to complete its nuclear development China should clarify to Kim Jong Un that this is his last chance to resolve the nuclear crisis Kim should also realize that the time is not on his side

외교가에선 트럼프 대통령이 당초 예상과는 달리 전날 북한을 테러지원국으로 재지정하지 않는 것도 중국의 향후 움직임을 지켜보겠다는 메시지로 해석하고 있다 （no translation）

外交界普遍认为,特朗普前一天推翻人们的猜测 没有把北韩再次列为支持恐怖主义国家,也可以解读为观察中国今后举动的信号

다만 양측 대표는 제재·압박에 중점을 둔 대북 기조가 우선이라는 점은 분명히 했다 （no translation）

但是,韩美代表明确表示,以制裁和施压为重点的对北韩政策基调仍然是优先课题

윤 대표는 모두발언에서 “(북핵 문제에 대한) 진전을 만들 수 있을지 (여전히) 많은 숙제를 안고 있다”고 했다 （no translation）

尹汝尚在磋商开场白中表示,“能否(在北韩核问题上)取得进展,(仍然)面临着诸多老难题”

북한이 비핵화에 대한 구체적인 시그널을 주지 않는 이상 제재 완화 등 국면 전환은 쉽지 않을 거란 얘기다 （no translation）

只要北韩没有就无核化发出具体的信号,就不会轻易转入放宽制裁的局面

한 소식통에 따르면 양측 대표는 한미 정보 교류의 수준을 높여 제재 실효성을 확보하자고 협의한 것으로 알려졌다 （no translation）

据某消息人士透露,韩美代表就提高韩美情报交流的水平 确保制裁的实效性等进行了磋商

특히 윤 대표는 최근 중국에서 북한 노동자 철수 등 대북제재 효과가 있다고 말하면서 중국의 역할을 거듭 강조한 것으로 전해졌다 （no translation）

尤其值得一提的是,尹汝尚表示,最近中国清退北韩劳工等,对北韩的制裁有效果,再次强调了中国的作用

아찔한 치파오‘그 시절은 지나갔고 이제 거기 남은 건 아무 것도 없다‘ Chinese costume Qipao “The old good time is already gone and nothing is left there now”

令人晕眩的旗袍“那个时代已经过去现在什么也没有留下”

왕가위 감독의 ‘화양연화‘의 한 대목이다 These lines are from a scene in the Chinese movie “The Most Beautiful Moment in Life” by director Wang Ga Yi

这是王家卫导演的电影《花样年华》中的一句台词

이루지 못한 사랑을 아련하게 채색한 영화에서 배우 장만옥은 중국 전통의상 치파오를 수십 번 갈아입고 등장해 치명적 매력을 발산한다 In the movie, which presents a sad story of lost love, actress Maggie Cheung appears in different scenes, changing traditional Chinese costume Qipao dozens of times to display fatal seduction

在这部含蓄描写无法实现的爱情的电影中,演员张曼玉换了几十身中国传统服装旗袍上场,散发出致命的魅力

▷치파오(旗袍)는 높은 옷깃, 치마 옆트임(slit)과 함께 몸에 착 달라붙는 것이 특징 Qipao is characterized by the rising collar, slit on the skirt and tight style

?旗袍的特征,是高领 高开衩和紧贴身材

청나라를 지배한 만주족의 팔기군 복장에서 유래한 옷인데 남녀공용에서 여성의상으로 발전했다 The costume originated from the Eight Banner Army of the Manchurian tribe, who ruled the Qing Dynasty, and developed into traditional costume for both males and females

旗袍来源于统治清朝的满族八旗军服装,后来由男女共用发展成为女性专用

1920년대 상하이를 중심으로 고혹적 분위기를 강조한 개량형 치파오가 유행하기 시작했다 A remodeled Qipao, which emphasized seductive looks, started to be in vogue primarily around Shanghai in the 1920s

1920年代以上海为中心,开始流行强调迷人氛围的改良型旗袍

1972년 미중 정상회담 때 닉슨 대통령의 부인 패트 여사가 “중국에 왜 인구가 많은지 알겠다”고 했다는 말이 있을 만큼 관능적이다 The attire is so sensual that former U S First Lady Pat Nixon even reportedly said, “I now know why China has so large a population

1972年美中元首会晤时,尼克松总统的夫人帕特女士对旗袍的性感度曾有过这样的表述:“我明白了为什么中国的人口会这么多”

2008년 베이징 올림픽에 이어 2010년 광저우 아시안게임에선 시상식 도우미 차림으로 속옷 라인까지 도드라지는 치파오를 선보여 논란을 빚었다 ” In the award ceremony at the 2010 Guangzhou Asian Games and the 2008 Beijing Summer Olympics, ceremonial helpers showcased Qipao that even revealed their underwear lines, sparking controversy

2008年北京奥运会以及其后的2010年广州亚运会上,颁装仪式上的服务员身穿的旗袍甚至暴露出内衣的线条,引起了一时的争议

▷장만옥의 뒤를 이을 치파오의 새 강자가 나타났다 New Qipao fashion icons to succeed Maggie Cheung have emerged

?继张曼玉之后,出现了旗袍的新强者

9일 중국 국빈 만찬에 미중 퍼스트레이디가 나란히 치파오 블랙드레스 차림으로 등장한 것이다 U S and Chinese first ladies made public appearance, wearing black Qipao dresses, side by side at the state visit dinner in China on Thursday

在9日中国国宾晚宴上,美中两国第一夫人都穿着旗袍登场

멜라니아는 소매에 모피가 달린 화려한 자수 드레스로, 펑리위안은 소매의 맨살이 비치는 시스루 드레스로 양보할 수 없는 패션 대결을 펼쳤다 U S First Lady Melania Trump was seen wearing highly colorful dress decorated with needlework, with fur attached on the sleeves, while Chinese First Lady Peng Liyuan was seen wearing see-through dress, which lightly revealed her skin under the sleeve

梅拉尼亚是袖子上带有毛皮的华丽刺绣旗袍,彭丽媛则是袖口看得出小臂的薄裙,展开了互不逊色的时装对抗

모델 출신의 멜라니아는 허벅지 중간까지 쭉 트인 현대적 치파오를 마놀로 블라닉의 킬힐과 매치해 한 걸음 옮길 때마다 돋보이는 각선미를 과시했다 The model-turned U S first lady was wearing modern a Qipao dress, which was boldly open up to half of her thigh, in combination with kill heels from Manolo Blahnik

模特出身的梅拉尼亚身穿露到大腿中段的现代式旗袍,配以莫罗伯拉尼克高跟鞋,每一步都展现出曲线美

이 옷은 이탈리아 브랜드 구찌가 2016년 F/W시즌 치파오를 기반으로 내놓은 기성복이다 Her clothing is ready-made attire that Italian luxury brand Gucci introduced in the Fall/Winter season in 2016

这身服装是意大利品牌古驰在2016年秋冬季的旗袍为基础推出的成衣

펑리위안 은 무릎까지 트인, 상대적으로 정숙한 치파오를 택했다 The Chinese first lady picked a relatively mature Qipao dress that is open up to the knees

彭丽媛选择的则是开至膝盖 相对端庄的旗袍

▷이들의 패션 대결과 상관없이 이 날의 최종 승자는 단연 치파오 Irrespective of their duel, the ultimate winner of the competition was Qipao

?两人的时装对抗中,当天最终的胜利者无疑是旗袍

중국은 치파오를 단정, 우아, 지성미를 갖춘 여성 외교의 복장이라고 선전한다 China widely promoted Qipao as attire for feminine diplomacy that exudes the beauty of tidiness, elegance and intellectuality

中国方面宣称称,旗袍是具有端庄 优雅 知性美的女性外交服装

미국 퍼스트레이디 덕분에 돈 한 푼 안 들이고 중국 전통의상의 맵시를 세계에 홍보한 셈이다 Thanks to the U S first lady, China has effectively promoted the nice appearance of its traditional Chinese costume

托美国第一夫人的福,中国没掏一分钱,却在全世界宣传了中国传统服装的风姿

패션은 문화를 넘어 산업적 측면에서도 중요하다 Fashion is important from the perspective of both culture and industry

时装不仅是一种文化,在产业领域也十分重要

세계적 디자이너들이 한복과 사랑에 빠져 우리 고유의 미학이 녹아든 옷을 내놓은 날은 언제일까 We wonder when Korea will come to see world-renowned fashion designers fall in love with traditional Korean costume “hanbok,” design and sell clothing that features the aesthetic of uniquely Korean tradition

世界级设计师们什么时候才会陷入韩服的美,设计出融韩国固有美学的服装呢

치파오의 약진에 비하면 우리 옷의 세계화는 갈 길이 멀다 Korean costume has a long way to go to be globalized when compared with Qipao, which has made great strides

与旗袍的跃进相比,韩国服装的世界化还有很远的路要走

시진핑 “어떤 국가도 외딴섬 되면 안돼” ‘No country can afford to retreat into self-isolation,’ says Xi Jinping

习近平称“任何国家都不能成为孤岛”,暗指北韩

북 압박시진핑(習近平) 중국 국가주석이 18일 집권 2기의 대내외 정책을 천명하는 19차 전국대표대회 업무보고에서 “어떤 국가도 자신을 폐쇄시키는 외딴섬으로 돌아갈 수 없다”고 말했다 During the 19th National Congress of the Communist Party of China on Wednesday where the Chinese leadership presents its national and foreign policy approaches for the second term of Chinese President Xi Jinping, Xi said, “No country can afford to retreat into self-isolation

中国国家主席习近平18日在中国共产党第19次全国代表大会上作报告,阐述执政第二个任期的对内外政策。他表示,“任何国家都不能回到自我封闭的孤岛状态”

시 주석은 한반도나 북한을 직접 거론하지 않았지만, 국제사회 고립을 자초하며 도발을 계속하는 북한을 겨냥했다는 분석이 나온다 ” Xi was not referring to the Korean Peninsula or North Korea directly, but he seemed to regard North Korea that is not stopping provocation while being isolated from the international community

有分析认为,习近平虽然没有直接提及韩半岛和北韩,但针对的是自我孤立于国际社会 继续挑衅的北韩

시 주석은 이날 베이징(北京) 인민대회당에서 열린 당 대회 개막식 업무보고 가운데 대외정책을 공개하는 대목에서 “세계가 직면한 불안정성과 불확실성이 두드러지고 있다 While China’s foreign policy for the next term was being presented during the opening of the Communist Party Congress in Beijing, Xi said, “The instability and uncertainty that the world faces become more visible

习近平当天在北京人民大会堂举行的党代会开幕会上作工作报告,在公开对外政策时表示,“世界面临的不稳定性正在突显

지역 분쟁 문제가 여기저기서 끊임없이 발생하고 있다”며 “어떤 국가도 인류가 직면한 각종 도전을 혼자 대응할 수 없다”며 이같이 말했다 Regional conflicts are occurring continuously in many places No country alone can address the many challenges facing mankind

地区争端问题不断发生，任何国家也无力独自应对人类所面临的各种挑战”

그는 집권 2기 청사진으로 2단계 국가발전 전략을 제시했다 ” Xi announced his two-phased national development strategy as a blue print for his second term

作为执政第二个任期的蓝图,他提出了两阶段的国家发展战略

1단계로 2020년까지 이룬 전면적 샤오캉(小康·모든 국민이 풍족하게 생활하는 것) 사회의 기초 위에서 2025년까지 사회주의 현대화를 기본적으로 실현하고, During the first phase of development, Xi aims at prosperity of every citizen until 2020 and modernization of socialism until 2025

第一个阶段是在到2020年全面建成小康社会的基础上,到2025年基本实现社会主义现代化

2단계로 2035년부터 21세기 중엽까지 중국을 “부강한 민주 문명과 조화롭고 아름다운 사회주의 현대화 강국으로 만들겠다”고 했다 As for the second phase of development, Xi said he would make China as a strong and modernized nation of prosperous democratic culture and harmonious and graceful socialism between 2035 and the middle of the 21th Century

第二个阶段是从2035年起到21世纪中叶,把中国建设成为“富强 民主 文明 和谐 美丽的社会主义现代化强国”

그는 “2035년에 국방과 군대 현대화가 실현돼 21세기 중엽에 세계 일류 군대를 전면적으로 건설할 것”이라고도 했다 “National defense and military forces will be modernized by 2035 and China will nurture one of the strongest and advanced military forces in the world by the middle of the 21th Century,” said Xi

他表示,“到2035年,将实现国防和军队的现代化,到21世纪中叶,将全面建设世界一流军队”

자신의 임기(2022년) 이후까지 내다본 것은 장기 집권을 염두에 뒀다는 해석이 나온다 Speaking about plans for 2022 and beyond after his second term ends, Xi seems to consider seizing power much longer

有分析认为,他对任期(2022年)以后的事情进行展望,是有长期执政的想法

시 주석은 “당 전체가 중앙(시 주석)에 복종하고 당의 영도에 통일적으로 집중해야 한다”며 권력 집중을 예고했다 Xi talked about concentration of power by saying, “The whole party should obey the leadership and follow guidance by the leadership harmoniously

习近平表示,“全党要服从党中央(习主席),集中统一到党的领导,”预告了权力集中

자신의 사상을 “신시대의 중국 특색 사회주의”라고 밝혔다 ” He branded his philosophy as “socialism with Chinese characteristics for a new era

他把自己的思想称为“新时代中国特色社会主义”

베이징대 부원장 “중, 북핵에 큰 책임 대북압박 높여야”“중국이 (북핵 문제에서) 책임이 없다는 건 말이 안 된다 ” ‘China has a great responsibility in N K nuke,’ says Chinese scholar “China cannot say it does not have a responsibility (in North Korea’s nuclear issues),”

北京大学副院长:“中国应对北韩加大压力”“中国(在北韩问题上)没有责任,这种说法是不对的”

” 왕이저우(王逸舟) 베이징(北京)대 국제관계학원 부원장(사진)은 최근 동아일보 인터뷰에서 “중국 정부는 우리에게 (북핵 문제에) 매우 큰 책임과 (한반도에) 매우 중요한 이익이 있음을 명확하게 인정할 필요가 있다 said Wang Yizhou, Deputy Dean of the School of International Studies at Peking University, in a recent interview with the Dong-A Ilbo “The Chinese government needs to clearly acknowledge that we have a great responsibility (in Pyongyang’s nuclear issues) as well as important interests (in the Korean Peninsula),” the international relations expert added

北京大学国际关系学院副院长王逸舟(照片)最近在接受《东亚日报》采访时表示:“中国政府有必要承认,我们(在北韩问题上)负有很大的责任,(在韩半岛)有着重要的利益

(따라서) 전쟁이 발생하지 않을 정도까지는 대북 압박의 강도를 높여야 한다”며 이같이 말했다 “(Therefore,) Pressure on the North should be raised to a level where a war would not break out

因此,只要不到引发战争的程度,中国应该加大对北韩的压力”

그는 이런 자신의 견해가 “북핵 문제에 대한 주요한 책임이 미국과 북한에 있고 우리(중국)의 책임은 부차적인 것이라고 생각하는 (중국) 정부 당국의 입장과 다르다”고 말했다 He clarified that his opinion was “apart from the (Chinese) government’s stance that views the United States and North Korea as countries with key responsibility for Pyongyang’s nuclear issues while China has only a secondary responsibility

他表示,自己的这一看法,“和中国政府的立场不同,中国政府认为北韩核问题的主要责任在于美国和北韩,我们(中国)负有次要责任”

중국이 북핵 문제의 직접적인 당사자가 아니기 때문에 중국의 역할에 한계가 있으며 미국이 직접 북한과 대화해 해결하라고 요구해온 중국 정부를 우회적으로 비판한 것으로 풀이된다 ” This is an indirect criticism of the Chinese government, which has insisted that Beijing’s role is limited as it is not a direct party involved and thus called upon Washington to have a direct dialogue with Pyongyang to resolve issues

分析认为,王逸舟是在委婉地批评中国政府,因为中国政府认为自己不是北韩核问题的直接当事人,中国的作用有限,要求美国直接与北韩进行对话来解决

왕 부원장은 같은 대학의 자칭궈(賈慶國) 국제관계학원장과 함께 중국의 국제적 책임을 강조하는 자유주의학파의 대표적 인사다 Professor Wang, together with Jia Qingguo, Dean of the School of International Studies at Peking University, is one of the well-known scholars of liberalism school of thought who emphasize China’s responsibility in the international community

王逸舟与北京大学国际关系学院院长的贾庆国一样,是中国国内强调中国国际责任的自由主义学派的代表性人物

자 원장이 지난달 “중국이 한반도 전쟁 가능성을 인정하고 한미와의 소통 등으로 대비해야 한다”고 주장하자 주류 학파가 “중국 북핵 외교 핵심 원칙의 마지노선을 뒤집은 허튼소리”라며 공개적으로 정면충돌한 데 이어 중국 내에서 한반도 정책 노선 논쟁이 격화되고 있음을 보여준다 Professor Jia said last month, “China should acknowledge the possibility of a war breaking out on the Korean Peninsula and should prepare against it through communication with the United States and South Korea,” publicly drawing criticism from scholars of mainstream school of thought who dismissed his remark as “nonsense that reversed the Maginot Line of China’s core principles in diplomacy towards North Korea’s nuclear issues ” Such a collision indicates that disputes over policy lines on the Korean Peninsula are being intensified in China

贾庆国9月份声称,“中国应承认韩半岛战争的可能性并与美国进行沟通,”与抨击这是“巅覆中国在北韩核问题上外交原则的胡说八道”的主流学派公开发生意见冲突王逸舟的表态再次显示,中国内部关于韩半岛政策路线的争议正在激化

그는 자 원장의 견해에 대해 “대부분 지지한다”고 말했다 Professor Wang said that he “mostly supports” Professor Jia’s opinions,

王逸舟表示,“大部分支持”贾庆国的观点

이어 “중국이 개혁개방으로 갈수록 자 원장의 견해에 찬성하는 목소리가 많아질 것”이라고 전망했다 adding, “As China continues to pursue reform and openness, more would agree on Professor Jia’s views

他预测,“中国越是改革开放,赞成贾院长的声音会越来越多”

특히 왕 부원장은 자 원장 논쟁에 대해 “(중국) 당국이 누가 맞고 누가 틀리다고 얘기하지 않았고 토론을 억누르지 않았다”며 “이는 좋은 일이며 과거에 비해 많은 변화가 생긴 것”이라고 밝혔다 ” He also said that “The (Chinese) authority did not say which side was right or wrong when the dispute arose, and they did not suppress discussion either,” viewing this as “a positive development and a great change from the past

对于贾庆国言论引起的争议,他表示,“(中国)政府没有说谁对谁错,没有压制讨论。这是好事,比过去有了很多变化”

그는 “중국이 대북 정책을 조정하고 있다 ” “China is currently adjusting its policy towards North Korea,” the scholar also commented

他还表示,“中国正在调整对北韩的政策

과거 약했던 제재 강도가 갈수록 커지고 제재의 방향도 엄중해지고 있다”고 말했다 “The intensity of sanctions, which was low in the past, is getting higher and the direction of sanctions is also becoming severe

过去微弱的制裁强度正在越来越大,制裁的方向也在变得更为严厉”

‘중국의 만델라’ 류사오보간암 말기 판정을 받고 지난달 하순 가석방돼 입원 치료 중인 중국의 대표적인 반체제 인사 류샤오보(劉曉波·62)의 임종이 임박한 것으로 알려졌다 ” Liu Xiaobo, China’s Nelson Mandela Liu Xiaobo, 61, a Chinese dissident who are on medical parole after being diagnosed with late-stage liver cancer, is said to be in a critical condition

“中国的曼德拉”刘晓波据悉,中国代表性反体制人士刘晓波(62岁)被确诊肝癌晚期,上个月下旬获得保外就医,临终就在眼前

중국 정부는 그동안 막아온 가족 면회를 허용하며 심각한 상황임을 알렸다고 한다 Beijing said he is in a critical condition and finally allowed his family to visit him

据说,中国政府允许一直以来阻止的家人探视,说明情况已十分严重

G20 정상회의 직전 해외의료진의 접견을 허용했던 중국 정부는 회담이 끝나자마자 류 씨의 해외 치료 요청을 거부했다 While it permitted Western doctors to see him right before the G20 summit, it rejected Liu’s request for medical treatment abroad immediately after the summit

中国政府在二十国集团峰会前夕允许海外医疗人员接触刘晓波,但等到会谈一结束,就拒绝了让刘晓波到海外接受治疗的请求

이송의 안전을 우려했다지만 실제로는 해외의 반체제 활동에 불을 지를까 우려했을 것이다 Though the Chinese government claimed that it was concerned about transportation, it would in fact have worried about his dissident activities outside China

虽然其理由是担心移送中出现安全问题,但实际上是担心此举给海外的反体制运动火上浇油

▷1989년 천안문 사태 때 학생운동을 주도한 그는 반혁명선전선동죄로 징역 3년을 선고받았다 Liu who led a student movement during the Tiananmen Square protest in 1989 was sentenced to a three-year prison term for inciting the subversion of state power

?刘晓波曾主导1989年天安门事件时的学生运动,因反革命煽动罪被判有期徒刑3年

1995년엔 천안문 사태 재평가를 요구했다가 9개월의 가택연금을, 이듬해엔 대만과의 평화통일을 주장했다가 노동교양 3년형을 선고받았다 After he requested reassessment of the protest in 1995, he was put under house arrest for nine months When he called for a peaceful unification with Taiwan in 1996, he was sentenced to three years in a labor camp

1995年因要求重新评价天安门事件,被判居家软禁9个月。第二年因主张与台湾和平统一,被判劳动教养3年

2008년엔 공산당 독재를 비판하는 ‘08헌장’ 서명을 주도했다가 징역 11년을 선고받는 등 총 4차례 연금 또는 수감됐다 He initiated a drive to sign the Chapter 08 criticizing the Communist Party of China, only to be sentenced to 11 years in prison

2008年因主导签名抨击共产党独裁的“08宪章”,被判有期徒刑11年,前后四次被软禁或判刑入狱

복역 중이던 2010년 중국에서 첫 노벨평화상을 받은 류 씨는 중국 밖에서는 ‘중국의 만델라’로 불린다 In total, he was put under arrest or imprisoned for four times When he was in prison, he became the first Chinese who won the Nobel Peace Prize in 2010

他在服刑中的2010年,作为首名中国人获得诺贝尔和平奖,在中国境外被称为“中国的曼德拉”

하지만 중국 안에서는 그가 누군지도 모른다 He is called China’s Nelson Mandela outside China, but no one knows who he is in China

但是,在中国,没有人知道他是谁

중국의 최대 포털 사이트 바이두(百度)는 그가 미국 단체의 부정한 돈을 지원받은 것처럼 묘사해 놓았다 Baidu, the largest portal website in China, described him as a person who took corrupt money from a U S organization

中国的最大门户网站百度上,他被描述为接受美国团体不法资金的人

▷대부분의 반체제 인사들이 당국의 탄압이 두려워 해외 망명을 택했지만 그는 끝내 중국에 남아 투쟁했다 While most dissidents chose to exile abroad due to the pressure from the authorities, he eventually did not leave and struggled in China

?大部分反体制人士因为担心政府的镇压,选择了流亡海外,但刘晓波最终留在中国进行斗争

중국의 유명한 천체물리학자였던 팡리즈(方勵之)나 시각장애인 인권변호사 천광청(陳光誠)도 미국으로 망명하거나 탈출했다 Fang Lizhi, a famous Chinese astrophysicist, and Chen Guangcheng, a blind human rights lawyer, exiled or escaped to the U S

中国著名的天体物理学家方励之和患有视觉障碍的人权律师陈光诚也流亡或逃往美国

하지만 그도 부인은 자유로운 나라에서 살게 해주고 싶었던 것 같다 Liu apparently wanted his wife to live in a free country

但是,刘晓波希望让他的夫人生活在自由的国度

그가 해외 치료를 원한 건 자신 때문이 아니라 10년째 가택연금 상태인 부인 류샤(劉霞·55)를 위해서였다고 외신은 전했다 Foreign media outlet reported that he wanted to get medical treatment abroad not for himself but for his wife Liu Xia, 54, who has also been under house arrest for 10 years

据外媒报道,刘晓波希望到海外接受治疗,并不是为了自己,而是为了10年来处于居家软禁状态的夫人刘霞(55岁)

▷중국에서 사회 불만으로 일어나는 집단시위는 연간 18만 건을 넘는다고 한다 It is said that more than 180,000 protests per year are held out of discontent about society

?在中国,每年因社会不满而发生的集体示威事件超过18万起

신장(新疆) 위구르 자치구에서는 매일같이 무장 독립투쟁이 일어난다 The Xinjiang Uyghur Autonomous Region has an armed protest for independence every day

在新疆维吾尔自治区,几乎每天都发生武装独立斗争

대중이 모르는 사이에 투옥, 수감되는 인사가 부지기수라는 얘기다 It means numerous people are imprisoned without the knowledge of the Chinese general public

在人们不知情的情况下入狱关押的人不知其数

류 씨가 평생 외친 것은 자유 인권 평등 민주 법치였다 Liu has called for freedom, human rights, equality, democracy and rule of law for his life

刘晓波平生呼吁的,是自由 人权 平等 民主和法制

이런 인류 보편적 가치조차 수용 못하는 중국이 세계 지도국가로 갈 길은 멀다 There is a long way to go for a country that cannot accept such universal values to become a world leader

中国连这样的人类普遍价值都不允许,离世界领导国家还有很远的路要走

트럼프 보호무역에 반기든 한중일, G20으로 확산돼야 Korea, China, Japan say no to Trump's protectionism

扛起反对特朗普贸易保护主义旗帜的韩中日,要将之扩大到G20

한국과 중국, 일본 경제수장들이 어제 일본 요코하마에서 열린 한중일 재무장관·중앙은행총재회의에서 “모든 형태의 보호무역주의를 배격할 것”이라는 공동선언문을 발표했다 Korean, Chinese, and Japanese financial policy makers met at the Trilateral Finance Ministers and Central Bank Governors' Meeting held on Thursday in Yokohama, Japan and announced a communique saying that they will "reject any form of protectionism

韩国 中国和日本的经济首脑们昨天齐聚日本的横滨,举行了韩中日财长和央行行长会议,发表了名为《抵制所有形式的贸易保护主义》的共同宣言

한중일은 “우리는 무역이 생산성을 향상하고 일자리를 창출하는 등 경제성장의 가장 중요한 엔진이라는 점에 동의한다”며 높은 수준의 공조를 다짐했다 " The three financial ministers reaffirmed a strong coordination by saying, "We agree that trade enhances productivity as the most vital engine for economic growth, including job creation

韩中日3国表示:“我们一致认为,贸易是提高生产率 创造就业岗位等推动经济增长的最为重要的引擎”,决定进行更高水平的协作

도널드 트럼프 미국 대통령이 보호무역주의 기치 아래 자국 우선주의를 추구하는 상황에 세계 2위와 3위의 경제대국이 포함된 동북아 3개국 재무장관들이 한 목소리를 낸 것은 주목할 일이다 " It is noticeable that the financial leaders of the three Northeast Asian nations raised one voice against U S

在美国总统特朗普祭出贸易保护主义的大旗,追求本国利益优先的情况下,包括世界第二大和第三大经济体的东北亚三国的财长们发出一致的声音,令人瞩目

트럼프 대통령 취임 100일 동안 주요 국가들은 미국 눈치 보기에 급급하는 것이 사실이다 President Donald Trump who is currently hoisting the banner of America-first banner under protectionism Indeed, major advanced leaders have been busy reading President Trump's face for 100 days since his inauguration

在特朗普总统就任100天的时间里,各主要国家忙于看美国的脸色是事实

3월 독일에서 열린 G20(주요 20개국) 재무장관·중앙은행총재 회의에서 미국 반대로 지난 3년 동안 공동선언문에 포함됐던 ‘보호주의를 배격한다’는 문구를 넣지 못했다 Under the rejection from the U S , the phrase "reject protectionism," which had appeared for three consecutive years, was excluded from the communique made in the G20 Meeting of Finance Ministers and Central Bank Governors held in Berlin in March this year

3月在德国举行的G20财长和央行行长会议上,由于美国的反对,共同宣言中没有写入过去3年都一直写入的“抵制贸易保护主义”的文句

한 달 전 크리스틴 라가르드 국제통화기금(IMF) 총재, 김용 세계은행 총재와 앙겔라 메르켈 독일 총리가 독일에서 보호무역주의 확산을 경계한다는 데 의견을 모으고도 지난달 워싱턴 G20 재무장관·중앙은행총재 회의에선 미국이 반발하는 바람에 갈등만 증폭됐다 Furthermore, though IMF Managing Director Christine Lagarde, World Bank President Jim Yong Kim, and German Chancellor Angela Merkel all agreed to raise vigilance upon rising protectionism, only conflicts heightened as the U S opposed their idea at the G20 Meeting of Finance Ministers and Central Bank Governors held in Washington last month

一个月之前,国际货币基金组织总裁克里斯蒂·拉加德,世界银行总裁金庸,德国总理默克尔在德国就警惕贸易保护主义的扩散达成一致,但即便如此,在上个月举行的华盛顿G20财长和央行行长会议上,由于美国的反对,更加扩大了分歧

라가르드 총재가 지난해 “1차 대전 이전 같은 보호무역주의는 재앙적인 결과를 초래할 것”이라며 글로벌 경제 성장을 해치고 통합과 사람들도 해친다고 지적했듯이 보호무역주의로 이익을 볼 수 있는 나라는 많지 않다 Just as Lagarde warned last year that the protectionism seen before World War I would lead to disastrous aftermath, not many countries can benefit from protectionism, which hurts global economic growth, inclusivism, and people

拉加德总裁去年曾指出,“像第一次世界之前的贸易保护主义,将会带来灾难性的后果”,将会危及全球经济增长,对全球整合甚至对人也会带来危害。如她所指出的那样,由于贸易保护主义而获利的国家并不多

특히 무역으로 먹고 사는 한국으로서는 보호무역주의의 암운이 사라지지 않으면 수출 회복세에 타격을 입을 공산이 크다 Unless the haunting shadows of protectionism stops lingering, the export-driven Korea will be the hardest hit

尤其像韩国这样靠贸易吃饭的国家,如果贸易保护主义的乌云不散,出口恢复的势头很可能遭到打击

시진핑 중국 국가 주석이 1월 스위스 다보스포럼에서 “중국은 자유무역의 수호자가 될 것”이라 해놓고 그 후의 표리부동(表裏不同)한 행동도 실망스럽다 It is also disappointing to see two-faced Chinese President Xi Jinping who once claimed "China will become the guardian of free trade" at the Davos Forum held in Switzerland in January, and then turned towards higher trade barriers

中国国家主席习近平1月在瑞士达沃斯世界经济论坛上曾表示,“中国将成为自由贸易的守护者”,但之后表里行动不一,令人失望

한국에 대해선 사드(THAAD·고고도미사일방어체계) 배치를 트집 잡아 한국제품 불매 운동을 조장하고 유커들의 한국 방문까지 막았다 Beijing encouraged Chinese consumers to boycott Korean products as a protest against the deployment of THAAD, and even blocked their people from visiting Korea

抓住韩国部署萨德(THAAD,末端高空区域导弹防御系统)的小辫子,助长抵制韩货的运动,阻止游客访韩

힘의 논리가 우선인 냉엄한 국제통상의 현실이다 These recent movements portray the stark reality of international trade where the way of the closed fist prevails

力量逻辑第一,这是当今国际通商的现实

그럼에도 한중일이 이번에 한 목소리로 자유무역을 수호하겠다고 천명한 것은 중대한 의미를 갖는다 Therefore, it is very meaningful that Korea, China and Japan have raised one voice to protect free trade against all odds

尽管如此,韩中日此次共同发声,表示捍卫自由贸易,具有重大的意义

국경 없는 글로벌 경제에서 통상 문제는 국가안보와 직결되는 전략적 사안으로 격상됐다 In this borderless global economy, trade has become a strategic matter directly linked to national security

在没有国境的全球经济中,通商问题上升为和国家安全直接相关的问题

세계 경제와 교역의 20%를 차지하는 한중일 3국이 협력한다면 향후 G20 재무장관회의에서 트럼프의 보호무역주의에 대항하는 분위기를 조성할 수 있을 것이다 Under close coordination among the three nations, which account for 20 percent of global economy and trade, they may win more supporters to confront Trump's protectionism

占世界经济和贸易量20%的韩中日3国如果能够合作,就可以在今后召开的G20财长会议上营造对抗特朗普贸易保护主义的氛围

한중일의 공동선언이 말로 그치지 말고 실질적인 정책공조로 이어지도록 해야 한다 In addition, their communique should go beyond words and actually translate into visible policy coordination

韩中日共同宣言不应该停留在口头上,应该落实到实质性的政策协作中

자유무역 정신은 되돌릴 수 없는 세계무역 흐름이라는 사실을 G20으로 확산되는 계기로 만들기 위해 노력할 필요가 있다 More efforts should be made to hoisting the flag of free trade, which is a resistless global trade trend at G20 meetings

自由贸易的精神是不可逆的世界贸易的潮流,应该努力创造契机,把这一事实扩散到G20中
