# Supplementary material for: Bilingual translations of intensifiers in Dong-A Ilbo’s news about China: A corpus-based discourse analysis approach
Source: PLoS One. 2024 Feb 6;19(2):e0292603. doi: 10.1371/journal.pone.0292603 (PMC10846699; doi:10.1371/journal.pone.0292603)
Supplement: S1 File — (ZIP) [file pone.0292603.s001.zip › corpus data/subcorpus of English translation.docx]

# **Korea’s share in Chinese chips imports drops by 5.5% points**

The share of Korean exports in China’s semiconductor market has been reduced since Washington slapped sanctions against Beijing.

The Federation of Korean Industries announced Monday that Korea’s share in the Chinese semiconductor imports has fallen by 5.5 percentage points from 2018 to 2021. The fall was even more drastic than that of America’s (fallen by 0.3 percentage points), the architect of the anti-Chinese sanctions. By contrast, the shares of other exporters in Asia rose compared to the pre-sanctions era as indicated by Taiwan (by 4.4 percentage points), Japan (1.8 percentage points), and six ASEAN countries (0.4 percentage point).

From April 2019 to September 2020, the U.S. Department of Commerce put Huawei and SMIC on a trade blacklist over four times, blocking their supplies of semiconductors produced by using American software and equipment.

Compared to 2018, the size of China’s semiconductor imports swelled by 37.2 percent last year. Chinese imports increased by 6.5 percent, but the rise was meager compared to the growth of the Taiwanese or Japanese imports (by 57.4 and 34.8 percent, respectively).

The biggest contributor to the fall in Korea’s share in the Chinese imports was American’s sanctions, which prevented Korean companies from supplying for Huawei. As of 2019 when the sanctions began to be imposed, the shares of Huawei in Samsung Electronics’ and SK Hynix’s revenues stood at 3.2 percent (about 7.37 trillion won) and 11.4 percent (about 3 trillion won).

# **China expedites nuclear armament amid tensions over Ukraine**

China has ramped up its efforts to develop nuclear weaponry under the pretext that it will get ready to face the United States, said The Wall Street Journal. This can be interpreted that nuclear threats from Russian President Vladimir Putin have worked out during the Russian invasion of Ukraine.

An anonymous source familiar with Chinese leadership was reported on Saturday by the WSJ that China pursued a nuclear buildup even before the Russia-Ukraine war, which convinced it of its nuclear pursuits, assuming that Beijing concluded that Washington’s hesitation to intervene is driven by Russia’s possession of nuclear weapons. “Chinese leaders see a stronger nuclear arsenal as a way to deter the U.S. from getting directly involved in a potential conflict over Taiwan,” the source added. This means that China is enhancing its nuclear capabilities to keep the United States from directly intervening in any conflict with Taiwan just as in the ongoing war in Ukraine.

Experts argue that satellite images show increased activity to get construction works on more than 100 sites believed to be silos for a new long-range IBCM called the DF-41 around Yumen, a desert region in western China. Satellite images taken this January show temporary covers on silos having been removed, implying that confidential works involving sensitive information have already been finished. Carrying nuclear warheads, the DF-41 has an operational range of up to 15,000 kilometers, which can reach the U.S. mainland. China remains silent about these silos.

U.S. specialists speculate that China holds hundreds of nuclear warheads, expecting the number hereof to increase up to 1,000 by the late 2020s.

### **Extended COVID-19 lockdown in Shanghai sends panic**

Extended COVID-19 lockdown in Shanghai, the economic capital of China, is sending its citizens in panic. Further disruption in logistics is also expected as the number of ships waiting to load or discharge at Shanghai’s port has soared to more than 300 since the lockdown, according to CNN. As the lockdown continues, Tesla, which was forced to suspend production on last Monday, is likely to idle its production in Shanghai for the time being. It is the longest suspension of production since Tesla started its operation in the city in late 2019.

According to National Health Commission of China on Monday, 13,137 new COVID-19 cases were reported across the country on the previous day, approaching its all-time high of 15,152 on Feb. 12, 2020. Shanghai reported 9,006 daily COVID-19 cases on the same day. Shanghai still remains in lockdown although city officials initially said the city will be placed under lockdown until Monday.

Rumors are spreading as the citizens of Shanghai are increasingly experiencing anxiety. News and video spread fast on Weibo on Sunday that a child infected with COVID-19 died at a hospital in Shanghai after not being treated. In the video, medical staff is approaching a child lying on a bed and shouts of someone who appears to be the parents’ screams can be heard outside the camera.

The Shanghai Municipal Health Commission issued an unusual statement that night, officially clarifying that the child, who lost consciousness due to high fever, was offered emergency treatment at the Fudan University Hospital in Shanghai, adding that the child regained consciousness and the parents later apologized to the medical staff. Other rumors also spread that a company supplying vegetables had to discard piles of vegetables and the Port of Shanghai, the largest port in container throughput, will soon be closed. Despite the city authorities’ explanation that the rumors are not true, the citizens, who are having increasing levels of distrust of the government, do not seem to believe in them. The city of Shanghai has already lost the trust of its citizens as it began a lockdown on last Monday, the next day after it had announced that there would be no lockdown.

# **China’s protection of North Korea should stop**

South Korean President-elect Yoon Suk-yeol had the first conversation over the phone with Chinese President Xi Jinping. It came the day after North Korea’s launch of the ‘monster ICBM.’ Yoon discussed with Xi the security situation on the Korean Peninsula and responses to North Korea’s provocations. North Korea confirmed on Friday that the ICBM was the Hwasong-17 and claimed that it had been completed as a reliable method to deter nuclear wars.

North Korea is baldly raising the level of nuclear provocations by breaking the red line with the launch of the ‘monster ICBM.’ North Korean leader Kim Jong Un delivered a handwritten order that read “Shoot with bravery” and visited Pyongyang Sunan Airport to watch the launch process. A North Korean news agency covered the story and used the word ‘nuclear’ 13 times. It seems like the country will continue further provocations, including nuclear tests.

It is true that the response of China, a country supposed to prevent the North’s impulsive behavior, was disappointing. “We hope each relevant country will maintain an appropriate direction for dialogues and negotiations,” said China on Thursday regarding the North’s ICBM launch. Rather than condemning the North, China seems to be shifting the responsibility to other countries. China is also not raising its voice in the U.N. Security Council, as it cannot avoid criticism that it took advantage of the New Cold War atmosphere and opened a room for North Korea’s provocations.

It is unlikely that China will work with Yoon on the North Korean issues. The country has been expressing its discomfort with Yoon’s policy of ‘confident diplomacy’ toward China. However, there is no reason for China to aid and abet North Korea’s nuclear provocations. North Korea’s sophistication of nuclear technology is a political burden and security threat to President Xi before his third term. It is a factor accelerating regional destabilization by encouraging neighboring countries’ nuclear proliferation.

China should stop North Korea’s provocation and put pressure on the country to come to dialogues. China should first change its attitude of obstructing the international community’s sanctions against North Korea. The U.N. Security Resolution 2397 has a trigger clause that automatically applies additional sanctions against North Korea in case of its ICBM launch but it is only effective when the resolution is passed. China’s participation is a responsibility as a member of the international community. The new South Korean administration should also prepare to begin close cooperation with China right after it takes office. Deterring North Korea’s nuclear weapons is a common ground on which the national interests of South Korea and China depend and a key issue that will determine the future relationship of the two.

# **Hidden message in China’s expansionism that opens up Joseon’s ports**

Right after Joseon soldiers invaded into the palace in June 1882, Japan and the Qing dynasty sent their troops to the Korean Peninsula. While Japan dispatched warships and 300 soldiers to Jemulpo, 3,000 Qing troops landed on Namyang Bay. After the two external forces started negotiations with the peninsula being a tinderbox on the verge of war, Qing troops hijacked and took Heungseon Daewongun, who was behind the coup, to Tianjin. 　

The author, a professor of East Asian modern history at University of California, Davis, traces how the relations of Korea, China and Japan were incorporated into the modern international order around the late 19th century. He describes the Imo Mutiny as a historical incident that dismantled the geopolitical system to pay tribute to the Qing dynasty. Even after suppressing the riots, it kept the troops stationed on the peninsula while meddling deeply in internal affairs of the Joseon dynasty. The way it behaved ran against the conventional tribute system where a colonial master is entitled to rights only formally without interfering with a tributary state’s domestic politics.

China’s leaning toward expansionism was directly driven by the invasion of Japan and Russia into East Asia. The Korean Peninsula was considered by the Qing dynasty a core buffer state, not far from North China and the capital city of Beijing, from a perspective of national security.

What’s interesting is that the Qing dynasty demanded a treaty to open Joseon‘s ports overseas after it was virtually forced to sign a humiliating diplomatic treaty with Western powers after the Opium Wars. Involving Western powers in the landscape, it intended to keep Japan and Russia in check. In other words, the purpose of its diplomatic tactic was to take advantage of opponents to defeat another group of enemies. This turned out to be Joseon’s first step taken to be part of an international order of modern times under which sovereign states forge a diplomatic relationship in an equal position. As the title of this book implies, it was the last phase of the East Asian order that had centered around a colonial master. 　

With anti-Chinese sentiment prevailing following the judging controversy in the Beijing Winter Olympics, how we view China is becoming a hot potato. The expansionism-driven Qing dynasty in the 19th century reshaped the East Asian regional order surrounding the Korean Peninsula before Mao Zedong’s decision to join war turned out to be a watershed of the Korean War. This has implications for us today.

### Controversy over Beijing Olympics’ artificial snow making

Concerns are growing that the 2022 Beijing Winter Olympics may have a negative impact on the environment as many competitions will be held on man-made snow. Almost a daily amount of drinking water for 100 million people will be turned artificially into snow over the Olympic period, increasing concerns that residents around event venues will face a severe water scarcity. Although China promoted its pursuit of eco-friendliness during the opening ceremony with a smaller-sized flame on display, the very opposite is happening on the site.

Beijing does not only have a climate not conducive to winter events but also has to spend more electricity and water producing snow amid growing global temperatures due to a warming planet, according to CNN on Saturday. The International Olympic Committee estimated that around 49 million gallons of liquid water or 185.48 million litters will be used to provide artificial water to competition venues, which is equivalent to the volume of water that 100 million people drink a day.

As severe droughts have hit most of regions where outdoor competitions during the Olympics open over this winter, China has become highly dependent on artificial snow, say experts. With only an average snowfall of 200 millimeters a year, Zhangjiakou, a city which hosts outdoor competitions, is one of the driest regions in China. An available amount of water per person in the city is less than a fifth of the national average. Although it takes 200m³ of water to fill in ski resorts in Zhangjiakou, only 53m³ is reported to be secured as of now, said the Bloomberg.

China seems to be proud of realizing a “eco-friendly” Olympic event. Director Zhang Yimou said that the smallest torch ever in history at the opening ceremony on Friday is a representation of the Chinese government’s green efforts to say no to a larger-sized flame that wastes a bulky fuel.

# **China may face backstab N. Korea if it fails to take action**

The UN Security Council meeting was held on Friday to come up with measures to respond to North Korea’s intermediate range ballistic missile issue but adjourned after concluding no outcomes. It was the third Security Council meeting to be held this year on account of North Korea’s missile firing, but no agreement was reached as China and Russia vetoed against the joint action request made by more than half of the Security Council members. China did not even denounce North Korea’s provocative actions and urged the U.S. to exhibit policies and actions that accommodate North Korea’s concerns.

There were some expectations that the recent meeting, in which IRBM range of 5,000 kilometers was discussed, would be different from the outcomes of the previous two meetings. Back in 2018, the UN Security Council had come up with proactive measures to North Korea’s nuclear/missile threats, adding North Korean institutions and groups to its sanction list. Despite North Korea’s threats that have reached the highest ever in the last four years, the Security Council has not even issued a single statement. China said that it would send a copy of a press statement draft back to its country for review, which may not happen as well.

Nine countries including the U.S. issued a joint statement saying that “the silence of the UN Security Council would only embolden North Korea, violating the resolutions and continually threatening international peace.” Japan, which is not a member of the Security Council, joined the statement, but South Korea was not involved. The statement, however, is simply an expression‎ of concern as North Korea is already making threats with nuclear tests and ICBM. This behavior is happening as it is encouraged by China, tolerated by Russia and Korea turning a blind eye.

The loss of power by the UN Security Council, which has been known as the keeper of international peace and safety, is partially due to the power struggle between the U.S. and China as well as armed conflict between the U.S. and Russia. North Korea is certainly taking advantage of this new Cold-War like situation. However, if we fail to put a stop to North Korea’s continuing nuclear threats, it will eventually develop into a massive problem for China as well. China should not forget North Korea’s past launching of nuclear/missile threats whenever Xi Jinping tried to host major international events just a few years ago.

# **Olympic torch lit up in Beijing in 14 years**

The Olympic torch lights up Beijing in 14 years. Starting with the opening ceremony that will take place at 9 p.m. on Friday, the 2022 Beijing Winter Games will begin its 17-day celebration of winter sports. Beijing is the first city in Olympic history to hold both Summer and Winter Games.

Beijing National Stadium, where the opening ceremony is scheduled to take place, is the same stadium that hosted the opening and closing ceremonies of the 2008 Beijing Summer Games and was used as the main track and field stadium and the venue for the men’s 2008 gold medal soccer game. It is known as the “Bird’s Nest” for its distinctive design. The stadium, however, will not hold other sport events besides the opening and closing ceremonies. Zhang Yimou, a Chinese film director, will be directing the opening and closing ceremonies as he did in the 2008 Beijing Summer Games. Some 3,000 will perform in the opening ceremony that will last about 100 minutes, 95 percent of which will be teenagers.

The opening ceremony for the Winter Games is simpler than that of the 2008 Beijing Olympics, which lasted about four hours with 15,000 performers, because of the COVID-19 pandemic and cold weather. Moreover, some Western countries including the U.S. claimed diplomatic boycott of the Beijing Winter Olympics, contributing to a lesser number of foreign missions. Chinese President Xi Jinping of China and Russian President Vladimir Putin are expected to attend the opening ceremony.

The identity of the final torchbearer and how the torch would be lit up remain confidential. Back in 2008, Li Ning, a legendary Chinese gymnast, lit cauldron by gliding through the air, suspended on a high-wire. Director Zhang has already claimed that he would employ “a bold idea” to surprise spectators. Many expect to see eco-friendly way of lighting the cauldron, given that hydrogen has been used as energy source. Among candidates for the last torchbearer are Wang Meng, a decorated Chinese short-track speed skater who won six medals at the Olympics and Zou Kai, who won three gold medals at the 2008 Olympic Games.

transported by freight trains, as the two countries are resuming bilateral trade.

Back in January 2020, the North shut down its borders with China and halted operation of passenger trains to help prevent the spread of COVID-19. And seven months later, it completely halted freight trains operated at long intervals.

The South Korean government is paying close attention to the resumption of trade between North Korea and China, while trying to analyze its meaning. “It seems that preparation for resumption of trade in border regions between the North and China was completed early this month,” a government source said. “The two countries may have engaged in a war of nerve over the timing of resumption, and they apparently have taken action this time.”

# **Beijing Olympic torch to be carried for only three days**

The Olympic torch for the 2022 Beijing Winter Olympics, which would normally travel across China, will be carried only limitedly due to COVID-19.

The Olympic torch that arrived in Beijing on Oct. 20 last year is burning in the Beijing Olympic Tower as of Tuesday. Twelve hundred carriers will run from the center of Beijing to Yanqing District and Zhangjiakou in Hebei province, which are located in the outskirts of the capital city, for three days from February 2. The round trip distance is only around 300 kilometers. For the 2008 Beijing Summer Olympics, 21,880 runners carried the torch for 137,000 kilometers across 19 cities in five continents around the world.

It is also a lot less than the recent Olympics. For the Tokyo summer Olympics, the torch had been carried for 2,000 kilometers across Japan by 10,000 runners for 121 days starting from March 25, 2021 in Fukushima. For the 2018 Pyeongchang Winter Olympics, 7,500 carriers ran 2,018 kilometers in 17 cities and provinces, as well as all around Gangwon Province, for 101 days from Nov. 1, 2017.

# **Hong Kong’s Citizen News shuts down to protect its staff**

Hong Kong’s anti-China online news site Citizen News closed on Sunday. It appears the new site decided to cease operations after even large media outlets shut down one after another due to the growing suppression of the press from the authorities. There are growing concerns that the freedom of press has virtually disappeared in Hong Kong as three anti-China media outlets shut down in the past half a year following the closures of Apple Daily, the largest daily newspaper in Hong Kong, in June, and Stand News on Dec. 29.

On its official Facebook account, Citizen News made the announcement “with a heavy heart,” saying they must ensure the safety of everyone who are on board in a time of crisis. It went on to say that they wanted to serve the public by inheriting the spirit of journalism, but due to changes in Hong Kong and worsening environment for media in the past two years, a small boat called Citizen News was hit by strong winds and waves.

In a press conference on Monday, Citizen News’ founder and chief writer Chris Yeung said the decision to close Citizen News was made in a short time, adding they could not rule out that they might be exposed to some risks. In particular, he said that the announcement made four days ago by Stand News to cease operations was the decisive reason to decide the closure. Both Apple Daily and Stand News decided to shut down after former and incumbent executives were arrested one after another and company assets were frozen. It appears Citizen News made the decision in order to protect its staff.

Founded on Jan. 1, 2017, Citizen News is a small online media outlet with 40 staff. In pursuit of freedom, openness, diversity and inclusiveness, Citizen News faithfully delivered the voice of democracy during protests against the Hong Kong extradition bill in 2019 and Hong Kong security law in 2020.

# **Dark clouds cast gloomy shadows on G2 economies**

The year of 2022 will place a burden on the shoulders of the top two global powerhouses in the never-ending fierce competition for leadership – the United States and China, according to major overseas news reports. The Telegraph, a British newspaper, analyzed on Friday that China is likely to suffer a severe economic slowdown due to the consequences of tight border control following the COVID-19 pandemic, poor housing market conditions and power shortages. Likewise, the United States has a hard time handling the spread of the virus, inflation, a supply chain crisis, a deadlock situation in logistics and decrease of global leadership. This year is expected to be a “minefield” for U.S. President Joe Biden who has just started his second year at the White House, said the Hill, a U.S. politics magazine, on Saturday.

Nomura Holdings Inc., a Japanese investment bank, expected the U.S. economy to grow 4.6 percent this year, 0.3 percentage points up from the Chinese economic growth of 4.3 percent as projected. The United States is expected to grow faster than China in 46 years, which is the first time since 1976.

The Telegraph projected that India, a “rising giant,” will grow by 8.5 percent double China’s rate, predicting that the Indian economy will enjoy a long-lasting economic bonanza surpassing the Chinese economy. AXA Investment Managers, a French investment management firm, also shared a pessimistic view of the production capabilities of China, the “world’s factory.” The South Korean economy will be inevitably affected by a slowing Chinese economy not only because it is South Korea’s top export destination but also because it is home to many South Korean production facilities.

The Hill also pointed out that U.S. President Joe Biden should resolve the ramifications of pandemics including the ongoing situations with COVID-19 and the Omicron variant on medical and financial systems as well as inflation rates. He has recently recorded the lowest approval rates in the 30-40 percent ranges since his inauguration. The ruling Democratic Party’s Democratic election strategist Joel Payne was quoted as saying, “Joe Biden is president because of COVID, but Dems are struggling right now because of COVID. And until they can find someone to figure this out, people are going to be mad about COVID.”

# **‘Beijing likely to retaliate for closer Seoul-Washington ties’**

ohn J. Mearsheimer (75), an emeritus professor of the University of Chicago and authority in international politics, had New Year’s interview with The Dong-A Ilbo. “The stronger China becomes, the bigger security threat to Korea will be,” he said, commenting on conflict between Washington and Beijing. “Whoever becomes the next South Korean president, what kind of ties South Korea and the U.S. will forge is the most important matter.”

As Mearsheimer said as Chinese President Xi Jinping consolidated his long-term control of government by securing his three-consecutive terms, and thus Beijing seeks to become a hegemony exceeding Washington’s power and influence, it will increase pressure on South Korea. Mearsheimer, who is the most influential realistic theorist, is considered the expert who has most accurately predicted hegemonic competition between Washington and Beijing.

“The world is entering a second Cold War,” Mearsheimer said. “China will have power on par with the U.S. soon, and if it continues economic growth over the next 30 years, it will become the most powerful country in the world, surpassing the U.S. There is a strong chance that the U.S. and China will stage a war over Taiwan within the next 15 years.”

“Committing on South Korea’s so called ‘diplomacy of security with the U.S. and diplomacy of economy with China,” Mearsheimer said it would be ‘height of foolish’ for “South Korea not to concentrate on the Seoul-Washington alliance.”

As evidenced by the Terminal High-Altitude Missile Defense system (THAAD), the stronger China’s threat becomes, the more South Korea will have to seek its survival through security cooperation with Washington. “If South Korea and Japan closely cooperate with each other, they will be able to better cope with China’s threat,” he advised.

# **Foreign minister: Improving inter-Korean ties at Beijing Olympics seems unlikely**

South Korea’s Foreign Minister Chung Eui-yong said the country hoped that the Beijing Winter Olympics would serve as an opportunity to improve the inter-Korean ties, but now such expectation is unlikely to be met.

Foreign Minister Chung told reporters at a press briefing held at the Foreign Ministry building in Seoul on Wednesday when asked about the possibility of an inter-Korean summit at the upcoming Beijing Winter Olympics in February. Under the circumstances where North Korea’s participation in the Olympics is unclear due to the spread of COVID-19 and the U.S. has announced a diplomatic boycott of the Beijing Olympics, Chung acknowledged that the Olympics will not likely to be a foothold for an end-of-war declaration. However, a high-ranking government official said Foreign Minister Chung’s remarks mean that it has become difficult to leverage the Olympics to advance the peace process, adding there could be improvement of inter-Korean relations even before the Olympics.

As for a possible boycott of the Beijing Olympics, Chung reiterated his position that Seoul is “not reviewing” a diplomatic boycott of the Olympics. When asked if President Moon is open to attending the Olympics, Chung said he has nothing to share at the moment. It is known that the majority of opinion within Cheong Wa Dae is President Moon’s participation in the Olympics will be difficult for now.

As for an end-of-war declaration, Chung said South Korea and the U.S. have effectively agreed on the draft, a step up from the previous announcement that talks with the U.S. are in final stages. He went on to say that Seoul is reviewing how to advance talks with Pyongyang over end-of-war declaration, adding he expects a concrete response from North Korea.

When it comes to the criticism that Seoul is turning a blind eye to humanitarian issues in North Korea and China, Chung said South Korea is in special relations with North Korea and China, and there are many areas to cooperate with them as the two are directly related to the country’s national security.

### Samsung adjusts operations at its Xi’an chip plant amid

Samsung’s chip factory in Xi’an, China saw disruptions in production as the city is under a strict lockdown due to the spread of COVID-19.

Samsung Newsroom said on Wednesday that the company has decided to temporarily adjust operations at its manufacturing facilities in Xi’an, China due to the ongoing COVID-19 situation. The adjustment of operations means reducing chip production than usual. “The decision was made in accordance with our commitment to protecting the health and safety of our employees and partners, which remains our top priority,” added Samsung Electronics.

China locked down 13 million people in Xi’an, Shaanxi province after the city recorded 206 new COVID-19 cases between Dec. 9 and Dec. 22. This is because Chinese authorities are placing a lockdown and isolating all residents if there is one confirmed COVID-19 case in the region. Samsung Electronics has been operating its production facilities normally under special measures from the Chinese government. However, the South Korean IT giant had to adjust its operations there as employees’ commuting efforts and logistics faced disruptions due to a prolonged lockdown.

The chip plant in Xi’an is Samsun Electronics’ only overseas memory chip plant and has been producing NAND flash memory chips since 2014. NAND flash is used not only for data storage but also for manufacturing solid state drives (SSD) required for data storage in servers and PCs. Samsung Electronics has almost 40% share of the global NAND flash market and the Xi’an plant accounts for over 40% of the company’s total NAND flash production.

# **U.S. growth forecast in Q1 2022 lowered from 5.2% to 2.2%**

A fast spread of the Omicron variant is expected to hit the global economy, including the U.S. and China.

According to the Wall Street Journal on Tuesday, economic experts from around the world are lowering their growth forecast for the global economy, including the U.S. next year. Mark Zandi, chief economist at Moody’s Analytics, downgraded his Q1 U.S. gross domestic product (GDP) forecast from 5.2% to 2.2%. Citing reasons, such as less travel and cancellations of sporting events and Broadway shows, Zandi said the situation is very similar to the past summer, when Delta hit. Pantheon Macroeconomics also lowered its growth forecast for the U.S. in Q1 next year from 5% to 3%.

The World Bank recently cut its forecast for China’s economic growth next year from 5.4% to 5.1%. It predicted that the economy will continue to shrink next year as the spread of Omicron variant could lead to longer-lasting restrictions. The Bundesbank, Germany’s central bank, also downgraded its economic growth forecast for Germany next year from 5.2% to 4.2%.

### NHL players to skip Beijing Olympics due to COVID-19

The U.S. National Hockey League’s announcement that its players will not participate in the 2022 Beijing Olympics has poured cold water on the excitement for the winter games slated to be held next year. Ice hockey is one of the most popular winter team sports, dubbed as the “flower of winter sports.”

American sports channel ESPN reported Monday that the NHL and the NHL Players’ Association agreed not to participate the Beijing Olympic Games, amid rising concerns of the worsening coronavirus pandemic.

The NHL has already postponed 50 games in 2021-2022 regular season due to the spread of the Omicron variant of COVID-19, which is a change of the initial plan of taking a three-day Christmas holiday break to a pause of the regular season from Wednesday to Sunday. ESPN reported that the NHL will soon publicly announce its decision on Olympic participation in 2022 as it can opt out of Olympic participation without financial penalty if it makes a decision on participation until Jan. 10 next year.

The NHL has participated in every Games from the 1998 Olympics in Nagano, Japan to the 2014 Olympics in Sochi, Russia, but it didn’t participate in the 2018 Pyeongchang Olympics, citing little economic benefit and a tight schedule. The League initially announced that it would participate in the 2022 Beijing Olympics, a decision which appeared to be conscious of the 1.3 billion Chinese market, but it has reworked the plan and decided to skip the Beijing Olympics and go to the 2026 Milano Cortina Olympics.

As the Beijing Olympics tickets will be exclusively sold to spectators residing in mainland China, there will be no significant economic impact, such as a reduction in ticket sales, following the NHL’s announcement not to participate in the 2022 Beijing Olympics, but the Games’ reputation and viewership would seem to decline inevitably.

# **NYT: Vaccines except Pfizer and Moderna shots offer no protection against Omicron**

Most COVID-19 vaccines other than messenger RNA (mRNA) vaccines, such as Pfizer and Moderna vaccines, will not likely to offer protection against the Omicron variant, The New York Times (NYT) reported on Sunday (local time). There are rising concerns that infections would increase and new variants would emerge in low-income countries, which have relied on vaccines other than mRNA vaccines. A research found that the Omicron variant neutralizes antibody treatment for COVID-19.

The NTY reported that only the Pfizer and Moderna shots appear to have provided protection against serious illness from Omicron. The UK Health Security Agency (HAS) said on last Sunday that the effectiveness of the AstraZeneca vaccine against Omicron infection fell to 0 percent six months after vaccination. The Johnson & Johnson single-shot vaccine, whose demand is surging in Africa, “does little to nothing to stop the spread of Omicron,” NYT pointed out. China’s Sinopharm and Sinovac vaccines, which account for almost half of COVID-19 vaccine doses delivered globally, “offer almost zero protection from Omicron infection,” NYT wrote, adding those vaccines are widely used in countries such as Mexico and Brazil. The Russian Sputnik vaccine, which is being used in Africa and Latin America, shows “dismal rates of protection” against Omicron.

Wealthy countries did not share mRNA vaccine technology and as a result, low-income countries had to rely on non-mRNA vaccines, said Tolbert Nyenswah, a senior researcher with the Johns Hopkins Bloomberg School of Public Health. He pointed out that new variants will continue to emerge from those countries as a consequence, prolonging the pandemic.

According to The Associated Press on Monday, U.S. Pharmaceutical companies Regeneron Pharmaceuticals and Eli Lilly and Company said their antibody treatments, which are being widely used in American hospitals after being authorized for emergency use by the Food and Drug Administration (FDA) have “diminished potency” verses Omicron. Their antibody treatments are being used to prevent against severe disease. The two companies said they can quickly develop new antibodies but it would take at least several months before the launch of those antibodies, The Associated Press reported, adding doctors will face yet another challenge.

# **Moon says he is not considering diplomatic boycott of Beijing Olympics**

South Korean President Moon Jae-in said on Monday that the South Korean government is not considering a diplomatic boycott of the Beijing Winter Olympics in February. During his state visit to Australia. Moon drew a line while Australia and the U.K. have joined the boycott by the U.S., which is in sharp conflict with China.

“We haven’t received any requests from any countries, including the U.S., to participate,” said President Moon at a joint press conference following a summit meeting with Prime Minister of Australia Scott Morrison in Canberra on Monday. “Constructive efforts from China are required for the economy, peace, and stability on the Korean Peninsula, and the denuclearization of North Korea,” he added. “South Korea is trying to maintain a harmonious relationship with China while building on a solid alliance with the U.S.”

It seems that Moon is striving to achieve balanced diplomacy by continuing strategic ambiguity as both the U.S. and China are important to the future announcement of the end of the Korean War. “South Korea breaks with the U.S. on a boycott of Beijing Olympics,” Bloomberg reported on the president’s statement. “Moon has made reconciliation with North Korea one of his key policy objectives and, if he wants to make progress before his term in office ends next year, he will likely need Beijing’s help.”

# **South Korea applies to join CPTPP after China’s bid**

The South Korean government has officially announced to begin the application process to join the Comprehensive and Progressive Agreement for Trans-Pacific Partnership (CPTPP), a massive free trade deal taking up 15% of the world economy. While the deal is expected to serve as an opportunity to lower trade dependency on China and diversify Korea’s trade partnership, it is also feared to fuel steep opposition from local farmers and fisheries industry who are concerned about a fiercer competition against expanded imports.

“Considering the economic and strategic values derived from the expansion of trade and investments, we’ve decided to make an official bid to join the CPTPP,” said South Korean Finance Minister Hong Nam-ki at a meeting of Cabinet ministers on external economic affair on Monday. “The process will begin based on our discussions with various groups of stakeholders,” Hong added. The decision comes eight years after Seoul last considered making a bid for the membership of the TPP, the precursor to the CPTPP in 2013.

The CPTPP is a multilateral free trade deal launched in 2018 by 11 member states including Japan, Australia, and Mexico, after the U.S. exited the TPP trade deal. Currently, Japan is chairing the pact, with China and Taiwan having applied for membership in September this year.

The CPTPP boasts a high level of market openness, with a maximum 96% of tariff abolition rate. Seoul is expecting to lower its trade dependency on China and diversify export markets by joining the CPTPP which accounts for 15% of global trade.

“Joining the CPTPP will prove highly effective in helping us lower our dependency on China and expand our trade landscape,” said an official from the Korea Development Institute (KDI) in January this year. Another upside is to benefit from the stability of global supply chains amid the ongoing trade frictions between Washington and Beijing. The accession into the CPTPP can also be the chance to effectively sign a free trade deal with Mexica, a country that has yet to ink an FTA partnership with South Korea.

By contrast, local farmers and fishermen are concerned about expanded imports of agricultural produces. In fact, the CPTPP boasts plenty of agricultural powerhouses such as Australia, Chile, and Canada. The Korea Agriculture Association Consultative Body issued a statement on Monday, saying that an increase in the price-competitive imported agricultural products can lead to a collapse of the country’s foundation of agricultural production in the longer term.

Given additional procedures of public hearings, parliamentary reports, and discussions with member states, it will take another three to four years to complete the membership process. Some point out Seoul’s belated bid to enter the process after eight years has only sapped its negotiating power. A final accession requires a unanimous vote from all member states including Japan.

“Seoul’s negotiating power is relatively weaker because China has already begun the application process,” said Prof. Choi Won-mok of Ewha Womans University. “And Japan can capitalize on its chairmanship by playing the card of the court rulings on Korean victims of forced labor in rendering the final decision on Seoul’s membership.

# **Moon’s premature announcement on diplomatic boycott of Beijing Olympics**

South Korean President Moon Jae-in said that the South Korean government is not considering a diplomatic boycott of the Beijing Winter Olympics on Monday at a joint press conference during his state visit to Australia. “We haven’t received any requests from any countries, including the U.S., to participate,” Moon said. “South Korea is trying to maintain a harmonious relationship with China while building on a solid alliance with the U.S.”

Even though President Moon’s statement is one reconfirm‎s the government’s stance, which has been announced by the presidential office Cheong Wa Dae and the Ministry of Foreign Affairs since last week, it carries a different weight. Since the U.S. announced a diplomatic boycott of the Olympics, citing China’s violation of human rights, Cheong Wa Dae said it’s not considering it and the Vice Minister of Foreign Affairs said the country will play the role as the host country of the previous Olympics. As the president repeated the same stance, the fact that South Korea is not (currently) considering it started to sound like the country will not participate in a boycott.

Of course, South Korea is not in the same situation as other countries that quickly decided to join the boycott, including Australia, which responded to China’s wolf warrior diplomacy with a nuclear submarine alliance. However, South Korea’s stance looks more like that of France to refuse the boycott, which has historically showcased resistance to following the U.S., than that of Japan, which seems to be positively responding to the boycott but pondering a way to avoid it. In fact, China complimented South Korea for being an “Olympics family.”

South Korea is trying to balance between the U.S. and China under the name of strategic ambiguity in the midst of the two countries’ tensions. It may be an unavoidable choice to protect national interests between the U.S., an ally on which South Korea’s security depends, and China, the closest neighbor and the biggest trading partner. However, such balancing is a highly advanced act. A small misstep can cause critical damage. As the U.S. and China collide with each other for different ideologies, South Korea will find itself in a precarious situation.

For smart diplomacy between the U.S. and China, South Korea should strike a balance for each matter between universal values and national interests under clear principles and rules. Sending delicate messages is more important than anything else. South Korea has not expressed any concerns about China’s violation of human rights, which is the root cause of the boycott. This resulted in South Korea looking like siding with China. It is surely a failure of message management, which is likely to cause misunderstanding by the U.S. South Korea should not make the mistake of thinking that the U.S. will understand anything as an ally. Balancing between the two sides requires skills.

# **U.S. announces diplomatic boycott of Beijing Olympics**

The U.S. announced on Monday (local time) a diplomatic boycott of the Beijing Winter Olympics to be held in February. It was a diplomatic measure based on the judgment that defined the Chinese authorities’ crimes against humanity in Xinjiang as genocide. While New Zealand and others announced to join the boycott despite China’s open warning for countermeasures, the South Korean government is leaning to sending an Olympics diplomatic mission.

“The Biden administration will not send any diplomatic or official representation to the Beijing 2022 Winter Olympics and Paralympic Games,” said White House press secretary Jen Psaki on Monday. While athletes will travel to China, an official mission consisting of high-ranking government and political figures will not be sent to the opening and closing ceremonies. The press secretary said that genocide and crimes against humanity and other human rights abuses are ongoing in Xinjiang. “We will not be contributing to the fanfare of the Games,” she said.

The Biden administration practically asked its allies to join the boycott. State Department spokesman Ned Price said they will hear from more countries to join the boycott. New Zealand announced on Tuesday that its senior government representation will not be sent to the Beijing Olympics. The U.K., Australia, and Canada are expected to follow suit.

China strongly opposed and warned firm countermeasures. “Politicians calling for boycott the 2022 Beijing Olympics are doing so for their own political interests and posturing. It has no impact whatsoever on the Beijing 2022 to be successfully held,” Liu Pengyu, the spokesperson of the Chinese embassy in the U.S., said on Tuesday. “China will take resolute countermeasures against the U.S.’s dogmatic actions,” Zhao Lijian, the spokesperson for the Chinese Ministry of Foreign Affairs, said during a regular briefing on Monday.

# **Hong Kong security act causes 5,500 students and teachers to leave school**

More than 5,000 students and teachers have reportedly left secondary school in Hong Kong in the past year. Six out of 10 students who left school responded that they would leave Hong Kong for good. Since the Hong Kong Security Act, which allowed the state to sentence protestors in opposition to mainland China to life imprisonment at maximum, entered into force, Hong Kong’s social environment has radically changed, and this may have contributed to the large exodus of students and teachers.

HK01, a Hong Kong-based online news portal, cited on Thursday the data gathered by the group of Hong Kong middle and high school principals, which surveyed of 140 middle and high schools in Hong Kong and revealed that a total of 4,460 students and 987 teachers have left school during the school year 2020-2021. This is translated into an average of 32 students and seven teachers per school. The number of students and teachers who left school has increased considerably from the previous year, when 2,700 students and 498 teachers left school.

Of those students who left school, 2,643 students (59.2% of the respondents) said that they would leave Hong Kong and go to other countries. The Association of Principals expressed concern that the number of teachers who left school and chose to emigrate to other countries has increased by seven-fold, clearly suggesting the gravity of the exodus of both students and teachers for the past year.

Critics point out the oppressive social environment and pro-China education policies and curriculum upon the implementation of the Security Act, which gave rise to the disappointment of students and teachers, and cite it as the cause of the mass departure. As the traffic between China and Hong Kong is restricted to stop the spread of COVID-19, students from mainland China are prohibited from commuting to Hong Kong schools, and this is presumed to have partly contributed to the departure. Back in July, the Association of Principals first called for the education department to take measures by stating that many students and teachers are exiting from Hong Kong to go toward other countries.

# **China faces demographic change**

The Financial Times reported that the Chinese market for adult diapers could exceed infant products by 2025, reflecting China’s low birth rates and aging population trends. 　

The article said on Monday that while the demand for children’s diapers was dwindling, there is surging demand for adult nappies at care homes. Chinese manufacturers told The Financial Times that the sales of adult diapers would exceed children’s diapers in terms of annual sales by 2025.

Unicharm, the best-selling diaper brand in China, is spending more of its marketing budget to adult diapers than baby diapers. A factory owner from a nappy factory in Hubei said that the factory’s manufacturing lines had shifted from baby diapers to adult diapers. The Financial Times said that China’s diaper market is gearing for a fundamental shift.

The Hong Kong-based investment and brokerage group CLSA predicts that within just over eight years the adult diaper market in China could be worth 16 billion dollars, from less than 1 billion dollars last year. By 2040, the market size could rise to 30 billion dollars.

This change is due to China’s changing demographics. According to World Bank and China census, the average birth rate of women in China fell from 5.9 in 1961 to 1.3 last year, the lowest since research started in 1952. French investment bank Natixis predicts that China’s senior population, which accounted for 10% of the total population last year, could rise to 25% before 2050.

# **NIS chief Suh Hoon to visit China on Thursday**

Director of the National Intelligence Service Suh Hoon will visit China on Thursday for two days. With the discussion with the U.S. regarding the announcement of the end of a war between the two Koreas in the finalization stage, Suh will ask for China’s cooperation for the batter and look for ways to bring North Korea to a discussion table during the visit.

According to a diplomatic source on Tuesday, Suh will meet with Yang Jiechi, a member of the Politburo of the Chinese Communist Party in charge of foreign affairs, in China on Thursday and Friday. The two are meeting each other for the first time since August last year. “South Korea and China have maintained strategic communication on various occasions between their senior leaders. Against the backdrop, we are discussing the director’s visit to China,” said a member of Cheong Wa Dae.

Suh will ask for Beijing’s cooperation on the announcement of the end of the Korean War and share opinions about the Beijing Winter Olympics to be held in February during his visit. China is asking for South Korea’s cooperation for the Olympics as the U.S. implied a diplomatic boycott of the Olympics and COVID-19 outbreaks continue. “We haven’t changed our stance that we hope the Beijing Olympics will be an opportunity for the improvement of inter-Korean relationship and contribute to peace in Northeast Asia,” said a member of Cheong Wa Dae. “It is too early for the South Korean government to take a stance regarding the diplomatic boycott.”

Suh will also discuss Chinese President Xi Jinping’s visit to South Korea. As COVID-19 continues, Cheong Wa Dae is also considering a video conference summit meeting.

# **Homeland and home**

Homeland is where we long to be. As French philosopher Jacques Derrida put it, it is “where our ancestors lie or the immovable place where all travel and distance begin.” Perhaps this is the reason why some people who lived in foreign lands wish to be buried where they were born. But not for American writer Ha Jin, who visited Korea last week to receive the first Bucheon Diaspora Literary Award. He says that homeland is where one puts his/her roots down. It is not some place that you left behind, but where you build.

Ha’s words resonate his life. He decided to seek asylum while watching televised scenes from the Tiananmen Square Massacre in 1989. He became famous for his literary work with Chinese background written in English, winning National Book Awards. The Chinese government regarded him as a traitor and forbid him to enter China for several years. However, he believes that the betrayal is done by the nation that killed innocent young people, not himself. China was a “mother that had eaten her own children,” which is why he does not regard China as his homeland. 　

However, in psychological reality, things are different. Ha refers to America as his home but constantly reverts to China, as seen from Chinese immigrant characters in his work. Perhaps this is because he had left his homeland in his 30s when his ethical identity was already established. Unlike his words, the U.S. might not be his real homeland or home. 　

He returned to the U.S. after his visit to Korea without visiting his homeland, though it was just a few hours away. Then again, he had lived that way for 36 years. He had not even been able to visit to mourn for his parents when they passed away. It was a cruel punishment. His being made him a diaspora writer whose works reveal wounds of the past.

# **Hynix stuck between U.S. and China for semiconductor war**

Reuters reported Wednesday that SK Hynix’s plan to overhaul its plant in Wuxi, Jiangsu Province by acquiring advanced equipment from the Netherlands is in jeopardy. “The potential setback could make SK Hynix the next victim of the geopolitical struggle between the U.S. and China,” it said. The biggest concerns of South Korean semiconductor players in the middle of the battle between the U.S. and China to seize economic hegemony might become a reality.

SK Hynix had the second largest market share in the global D-RAM semiconductor sector in the third quarter with 27 percent, following Samsung Electronics’ 44 percent. In order to hold the lead against competitors in the U.S., the E.U., and China backed by government subsidies and tax benefits, aggressive investment in cutting-edge technologies and equipment is a must. In particular, the plant in Wuxi is a key facility manufacturing half of the company’s D-RAM products. It can have a negative impact on the company’s competitiveness if the plan falls through.

The Biden administration seems to be strongly opposing the export of semiconductor equipment utilizing the technologies of the U.S. and its allies to China. The superficial reason is that it can be misused to strengthen China’s military power but the main purpose is to keep in check the ‘Made in China 2025’ plan, which aims to put the country as a leader of manufacturing both in terms of quantity and quality. The U.S. government seems to be allowing no exception as it dismissed last week its own semiconductor company Intel’s plan to increase silicon wafer production in China.

A solution to this uncomfortable situation is putting a wider gap against competitors in terms of technology and quality so that Chinese companies have no option but to buy semiconductors made by South Korean companies. Despite the circumstances, the construction of Yongin semiconductor cluster, in which SK Hynix plans to invest, is being postponed for over a year due to a delay in getting residents’ approval and licensing. The government and the political circles should pass a special law to promote the growth of the semiconductor industry along with bolder measures to provide support and ease regulations.

# **Hynix stuck between U.S. and China for semiconductor war**

Reuters reported Wednesday that SK Hynix’s plan to overhaul its plant in Wuxi, Jiangsu Province by acquiring advanced equipment from the Netherlands is in jeopardy. “The potential setback could make SK Hynix the next victim of the geopolitical struggle between the U.S. and China,” it said. The biggest concerns of South Korean semiconductor players in the middle of the battle between the U.S. and China to seize economic hegemony might become a reality.

SK Hynix had the second largest market share in the global D-RAM semiconductor sector in the third quarter with 27 percent, following Samsung Electronics’ 44 percent. In order to hold the lead against competitors in the U.S., the E.U., and China backed by government subsidies and tax benefits, aggressive investment in cutting-edge technologies and equipment is a must. In particular, the plant in Wuxi is a key facility manufacturing half of the company’s D-RAM products. It can have a negative impact on the company’s competitiveness if the plan falls through.

The Biden administration seems to be strongly opposing the export of semiconductor equipment utilizing the technologies of the U.S. and its allies to China. The superficial reason is that it can be misused to strengthen China’s military power but the main purpose is to keep in check the ‘Made in China 2025’ plan, which aims to put the country as a leader of manufacturing both in terms of quantity and quality. The U.S. government seems to be allowing no exception as it dismissed last week its own semiconductor company Intel’s plan to increase silicon wafer production in China.

A solution to this uncomfortable situation is putting a wider gap against competitors in terms of technology and quality so that Chinese companies have no option but to buy semiconductors made by South Korean companies. Despite the circumstances, the construction of Yongin semiconductor cluster, in which SK Hynix plans to invest, is being postponed for over a year due to a delay in getting residents’ approval and licensing. The government and the political circles should pass a special law to promote the growth of the semiconductor industry along with bolder measures to provide support and ease regulations.

# **Choi Jong-kun says partnership with China is needed realistically**

“China is a strategic partner and we realistically need a partnership with Beijing,“ South Korean Vice Minister of Foreign Affairs Choi Jong-kun said at a strategy forum with a topic of the relationship between South Korea and the U.S. in Washington, D.C. on Monday (local time), emphasizing the importance of the relationship between South Korea and China. Former high-ranking officials of the U.S. administration raised concerns that the ROK-U.S. alliance may weaken over time and South Korea may be overlooked in the process of U.S.’s policy-making process.

In his keynote speech at the ROK-U.S. Strategic Forum co-hosted by the Center for Strategic and International Studies (CSIS), a think tank based in Washington, D.C., and the Korea Foundation (KF), Choi first highlighted the importance of the ROK-U.S. alliance. “South Korea and the U.S. are showing what a 21st-century alliance is about to the world,” he said, adding that the alliance of the two countries is leading partnership in not only security but also economy and culture.

To a question asking where South Korea stands regarding China following the speech, he said China is a strategic partner. “As with other domestic policies, foreign policies should be aligned with the needs and interests of South Koreans, especially the middle class in the country,” he said. “Trade volume with China is bigger than the U.S. and Japan combined and the beneficiaries of such a market are South Korean people,” he explained. “Dependency on various products imported from China is not only South Korean but everybody’s issue,” he said on the subject of supply chain issues.

# **China decides to export 18,700 tons of urea solution to S. Korea**

The Chinese government notified the South Korean government that it will export 18,700 tons of urea solution contracted with South Korean companies. It is enough to cover two to three months of domestic demand. About 10,000 tons of urea for cars among the amount to be exported by China will arrive in South Korea as early as the end of this month or the beginning of the next month. It will resolve the shortage of urea solution in the short term, however, it still remains uncertain whether urea solution will be imported smoothly from China in the future as the country has not suspended its inspection system of urea solution before export.

“After communicating with the Chinese government through various channels to accelerate the import of Chinese urea solution, it has been confirmed that the amount previously contracted with South Korean companies will be imported normally,” the Ministry of Foreign Affairs said on Wednesday. As it takes about two weeks from an application for pre-export inspection to its completion, the contracted amount will arrive in South Korea at the end of this month or the beginning of the next month. The ministry also added that applications had been submitted already for 7,100 tons out of 18,700 tons, and 300 tons imported by a company for automobiles have completed an inspection and will arrive in South Korea next week.

However, as China has practically put a brake on its urea solution export, securing supply won’t go back to normal completely. “It is a short-term normalization of the export process,” said a member of the ministry.

# **Spillover effects of Evergrande liquidity crisis on the global economy**

The U.S. Federal Reserve warned Monday that the liquidity crisis of Evergrande, China’s real estate developer company, may have spillover effects on the U.S. and global economy. The Fed’s change in tone provides a contrast to Federal Reserve Chair Jerome Powell’s statement back in September, which brushed off the concern that the Evergrande situation does not pose a threat beyond China, dismissing a possibility of spillovers to global economy.

In its biannual report on financial stability, the Fed warned of the risk posed by China’s property developer Evergrande to the U.S. financial system. “Given the size of China’s economy and financial system as well as its extensive trade linkages with the rest of the world, financial stresses in China could strain global financial markets through a deterioration of risk sentiment, pose risks to global economic growth, and affect the United States,” analyzed the Fed.

“In China, business and local government debt remain large; the financial sector’s leverage is high, especially at small and medium-sized banks; and real estate valuations are stretched,” the report further pointed out. “In this environment, the ongoing regulatory focus on leveraged institutions has the potential to stress some highly indebted corporations, especially in the real estate sector, as exemplified by the recent concerns around China Evergrande Group,” the Fed stated in the report.

Evergrande Group borrowed heavily to support its aggressive business expansion until the Chinese government began a crackdown on real estate debt, prompting a liquidity crisis. “Stresses could, in turn, propagate to the Chinese financial system through spillovers to financial firms, a sudden correction of real estate prices, or a reduction in investor risk appetite,” the Fed said.

# **RCEP to take effect in next January**

The Regional Comprehensive Economic Partnership, or RCEP, will come into force in January 2022, among 10 countries, including China and Japan. Japan is particularly excited about economic invigoration, with its GDP projected to increase by 2.7%. South Korea has signed the RCEP, but because it is yet to be ratified by the National Assembly, South Korea is not included in the list of countries where the RCEP will come into force.

“With the ratification of the RCEP by Australia and New Zealand on Tuesday, the requirements for the regional trade pact to take effect have been satisfied,” stated the Japanese foreign ministry on Wednesday. “From January 1, 2022, the RCEP will come into force in 10 countries, including Japan, China, Australia, New Zealand, Singapore, Vietnam, Thailand, Brunei, Cambodia, and Laos,” said the ministry. Five countries including South Korea, Indonesia, Malaysia, Myanmar, and the Philippines have not deposited the ratification instrument.

The regional free trade agreement includes tariff reductions to promote regional trade. Once all 15 signatories ratify the instrument, the RCEP will become the world’s largest FTA, bigger in size than the Comprehensive and Progressive Agreement for Trans-Pacific Partnership

(CPTPP), composed of 11 countries except the U.S. As of 2019, the RCEP covers a total trade volume of 5.6 trillion dollars (approximately 6,600 trillion won, taking up 31.9% of the world’s trade volume), a market of 2.27 billion people, roughly 29.7% of the world’s population, and 26 trillion dollars of GDP (30.8%). The RCEP is expected to generate significant economic benefits, as it is an FTA that is joined by South Korea, China, and Japan, as well as ASEAN, which has a huge growth potential.

“The RCEP will contribute more than ever to its economic development by connecting the world’s growth hub and Japan,” Chief Cabinet Secretary Hirokazu Matsuno, the Japanese government's top spokesperson, said in a press conference held on Thursday. “Japan will take the lead,” said Mr. Hirokazu.

The Nikkei reported that the first FTA signed by Japan with its counterparts South Korea and China will begin to take effect, stating, “[RCEP] is expected to increase Japan’s GDP by 2.7%.” The Yomiuri Shimbun stated that Nations joining the RCEP expect to see economic recovery; at the same time, China is being checked by other countries, as it tries to take the hegemonic position in trade in the Asia-Pacific region.

India deliberated on joining the RCEP, but it opted out of joining it out of concern that the RCEP would accelerate a flood of cheap Chinese imports, thereby exacerbating India’s trade deficit against China.

# **Gen. Hyten: China’s military progress is ‘stunning’**

Following Gen. Mark Milley, chairman of the Joint Chiefs of Staff, who expressed concern over China’s test of a hypersonic missile, Gen. John Hyten, vice chairman of the Joint Chiefs of Staff, said China’s growing military prowess is “stunning.” According to CNN, Gen. Hyten said that the pace at which China is moving is stunning, adding that China will surpass Russia and the U.S. considering the pace China is moving and the trajectory it is on. “It’s not just the United States but the United States and our allies because that’s the thing that really changes the game,” Gen. Hyten said.

According to Gen. Hyten, the U.S. has carried only nine hypersonic tests in the last five years whereas China has done hundreds of them. “Single digits versus hundreds is not a good place,” Hyten pointed out. In regard of missile test failures of the U.S., Hyten said, “Failure is part of the learning process,” adding the U.S. will move fast by taking risks and learning from failures.

The vice chairman of the Joint Chiefs of Staff cited North Korean leader Kim Jong Un as an example, arguing Kim has learned the lesson of failed tests to speed up development. Unlike his father (Kim Jong Il), Kim Jong Un has decided not to kill scientists and engineers when they failed and instead encourage them to learn by failing. As a result, the 118th biggest economy in the world was able to build an ICBM nuclear capability.

# **Amid supply chain crisis, product price increases in U.S. and China**

The supply-chain disaster is putting inflationary pressure on global economy, including the U.S. and China. U.S. businesses hit by labor shortage and supply chain crisis are raising product prices, after having endured ever-rising costs. China, which is dubbed as the world’s factory, has been hit by electricity outage and rising costs of raw materials, has seen a huge increase in inflation.

As China’s inflation is likely to spread to the entire world, South Korea has been alarmed by import prices. Critics forecast that economic recovery interfered by supply chain crisis, U.S.’s economy growth in the third quarter might be expected to be half of the previous quarter.

According to The New York Times and Reuters on Wednesday, major U.S. companies, which disclosed their third quarter earnings on the day, has hinted price rise. They shift an increase in costs due to rise in commodity prices and labor costs to consumers.

McDonald’s announced that it would raise menu prices by about 6% due to increased ingredient and labor costs. The fast-food giant is reportedly under heavy cost pressure from labor shortages that drove the company’s labor costs up by more than 10%, along with a maximum of 4% increase in the prices of ingredients and other materials. Kraft Heinz that already increased its product price by 1.5% in response to inflation stated that it would maintain its pricing strategy well into next year, implying a possibility of further price raise. Coca Cola announced in its earning report that it may increase the price of its beverages in the face of high labor and logistics costs. 3M, a global consumer goods company, also stated that it is facing higher costs related to polypropylene and high labor costs and that it would increase the price of its products to respond to inflation and supply chain pressure.

The price rally of many global companies is attributable to the unprecedented labor shortages and supply chain crisis. The number of monthly job openings in the U.S. has soared above 10 million, yet labor shortages are driving companies to raise wages. A bottleneck at ports and land logistics have caused businesses to heavily rely on a high-rate air freight, and some businesses are even chartering their own container ships.

# **Spectators residing in China to be allowed entry at Beijing Olympics**

China has announced that the Beijing Winter Olympics set to start in February next year will be held on a minimum scale. It plans to reduce unnecessary activities and procedures and greatly cut down on the number of staff for the Olympics as a way to prevent the spread of COVID-19.

The Beijing Organizing Committee for the 2022 Olympic and Paralympic Games released Monday the first edition of the official manual containing COVID-19 infection prevention and control rules for the Olympics, according to the Chinese media. The manual applies to athletes and officials and there could be further editions.

According to the manual, Olympics will be held in a “closed-loop” management system, where transportation, accommodation, facilities, stadiums and training centers are connected and operated as one. As if covered with a bubble, participants will stay in closed areas that are completely isolated from outside. Participants are allowed to move to and from limited spaces and must be tested for COVID-19 on a daily basis. Unvaccinated participants will have to serve a 21-day quarantine upon arrival in Beijing.

With the Beijing Olympics set to be held with limited number of personnel, volunteers, who used to work for the convenience of athletes, are likely to disappear from Olympic venues as well. Unlike the 2020 Tokyo Olympics, which were held without spectators, limited number of spectators will be allowed entry at the Beijing Olympics. But those from outside mainland China will not be allowed to attend the Games.

Meanwhile, actions to boycott the Beijing Olympics are continuing in the U.S. and the European Union (EU) ahead of the upcoming Olympics. Experts, however, say boycott is unlikely to actually happen. “The U.S. is recently giving conciliatory signals to China,” said Kim Heung-gyu, a political science professor and Diplomacy at Ajou University. “Discord with China could have a negative impact on the Biden administration within the U.S. Washington will not be willing to take that risk ahead of next year’s midterm elections.”

# **90% of Japanese ‘dislike China’ and 66% of Chinese ‘dislike Japan’**

In a recent survey, nine out of 10 Japanese answered they don’t have a positive impression of China while 66.1 percent of Chinese expressed the same feeling toward Japan. It will be the 50th anniversary of the normalization of diplomatic relations between the two countries next September but their sentiment toward each other has worsened since last year.

According to the Mainichi Shimbun on Thursday, Japanese non-profit organization Genron NPO and a Chinese group of international publishers conducted a survey of 1,000 Japanese and 1,547 Chinese aged 18 or over on the perception of each other from August 21 to September 25. The result was that 90.9 percent of Japanese respondents had a negative perception of China, which is 1.2 percentage points higher than that of last year. The figure was the highest in 2016 at 91.6 percent since the beginning of the survey and this was the first time it went over 90 percent in five years.

Among Chinese respondents, 66.1 percent had a negative perception of Japan, up 13.2 percentage points from the previous year. Anti-Japan sentiment was at its peak in 2013 at 90.1 percent when the two countries collided regarding the Senkaku Islands in Japanese or the Diaoyudao Islands in Chinese and had been on a consistent decline since then before surging this year. “The two countries have only made military threats while neglecting their people’s anxiety,” Genron NPO’s CEO Yasushi Kudo said at a press conference on Wednesday.

The negative perception of each other between Chinese and Japanese was more severe than the one between Korean and Japanese. In a similar survey conducted by Genron NPO and East Asia Institute in South Korea, the share of South Koreans who had a negative impression of Japan decreased from 71.6 percent last year to 63.2 percent this year. Meanwhile, Japanese anti-Korean sentiment only increased a bit from 46.3 percent to 48.8 percent during the same period. Japanese anti-China sentiment has been almost twice higher than their anti-Korean sentiment since 2015.

# **Korea’s first space launch vehicle successfully lifts off**

Korea’s first space launch vehicle (KSLV-2), also known as Nuri, lifted off on Thursday from the Naro Space Center in Goheung County, South Jeolla Province. The rocket soared to space, following the separation of the first-stage rocket, paring, and the second-stage rocket.

From design to development, production, and takeoff, the Nuri has been made with Korea’s homegrown rocket technology. In the history of space rocket development, only 28 percent of the vehicles succeeded in the first launch. The Naro has its first stage rocket that was built in Russia. As the space rocket succeeded in the third attempt, it was loaded with a dummy satellite in contingency for failure.

South Korea is a late mover in the global space race due to many constraints arising from complicated security environment surrounding the Korean Peninsula. In particular, the South Korea Ballistic Missile Range Guidelines shackled its development of not only military missiles but also the development of private spaceflights. Thankfully, the Missile Guidelines were amended one by one, paving the way for the nation to realize its space ambitions, were completely abolished in the ROK-US summit held in May. South Korea can now participate in the Artemis Accords, an American-led initiative for the Moon exploration.

The success of the Nuri will mark the first step for South Korea to become a leader in space exploration. The nation will begin developing various private and military satellites, a Korea-specific global positioning system (GPS), and space materials, components, and gear development. There is still a long way to go, as it is an industry requiring expensive costs and highly sophisticated technology. However, space is infinitely huge, offering infinite opportunities for growth; as such, we cannot afford to be reluctant.

The space industry has been expanding infinitely, from satellites and space launch vehicles to space tourism, air mobility, space Internet, and space debris treatment. Countries that are leading in the sector are fiercely competing to get ahead of others. The U.S., moving beyond national security and technological competition, has kicked off private space tourism, and China has achieved remarkable growth in space development by building its own space station, sending astronauts into space and its aircraft on Mars. Based on the achievements of the Nuri, South Korea should also begin in earnest its journey into space.

# **China test-fired hypersonic missile, says Financial Times**

China secretively test-fired in August a hypersonic missile that can have a nuclear warhead mounted, the Financial Times reported Saturday by quoting an informed source.

According to the U.K. daily, China’s hypersonic glider vehicle (HGV) flew before falling about 32 kilometers off the target. “The test demonstrated the advancement of China’s hypersonic weapons, which has alarmed U.S. intelligence authorities,” the Financial Times said. The fact China possesses a hypersonic missile was known already, but the test firing has demonstrated that China’s hypersonic weapons technology is more advanced than what Washington previously estimated.

The state-of-the-art weapons system, which is capable of flying at five to 20 times the speed of sound, is known as a game-changer in warfare. A ballistic missile soars into the space to fly in an arc, but a hypersonic missile flies at a low trajectory in the atmosphere before hitting the target instantly. Experts say that the missile could incapacitate missile defense systems of various countries that are designed to target cruise missiles and ballistic missiles.

Hypersonic missiles are capable of hitting a target anywhere in the world within one to two hours, and penetrating missile defense systems. As such, global military powerhouses including the U.S., China and Russia are racing to develop hypersonic missiles. North Korea also joined the foray to develop such a missile by test-firing the Hwasong-8 hypersonic missile on Sept. 28.

# **Xi Jinping wishes farewell to German chancellor**

Chinese President Xi Jinping held farewell to German Chancellor Angela Merkel, who leaves office after 16 years in the chancellery since 2005, in a video meeting on Wednesday and called her an “old friend (lăo péngyŏu),” speaking very highly of the outgoing leader of Germany. President Xi’s cordial greetings to Chancellor Merkel is likely to have been stemmed from Chancellor Merkel’s 12 visits to China during her tenure and her impartiality amid the U.S.-China tensions. The German chancellor frequently clashed with the Donald Trump administration over Trump’s intense pressure to spend more on defense and U.S. troops in Germany.

On Thursday, the People’s Daily, China’s largest newspaper, ran on the front page the photo of President Xi and Chancellor Merkel, both smiling, in a video meeting. President Xi rarely smiles in public, but he wore a pleasant smile throughout the meeting with Chancellor Merkel. The People’s Daily wrote that the China-Germany relations and the China-EU relations have been strengthened during Merkel’s time in office. “Chinese people put great emphasis on ties of friendship,” Xi was quoted as saying, adding, “We will never forget old friends, and China’s door will always be open to you.”

The Chinese Foreign Ministry issued a press release stating that President Xi cited Mencius’ saying that goes, “The acquaintance of people lies in knowing each other. In knowing each other, it is important to know the heart.” Xi applauded that Merkel’s chancellery proved that China and Germany could avoid playing a zero-sum game and were still be able to enjoy mutual benefits.

The Global Times, China’s state-run English newspaper, also carried a favorable editorial, stating that Chancellor Merkel visited China the most among global leaders of major countries, and Germany adopted pragmatic China policies under her administration. Since her inauguration in November 2005, Angela Merkel visited China 12 times through September 2019, before the breakout of the COVID-19 pandemic. Xi Jinping visited Germany three times during the same period. The two leaders have communicated via phone and video meetings since the COVID-19 pandemic broke out. In 2021 alone, Xi and Merkel contacted on five occasions.

# **Soaring oil prices stir inflation fears**

Supply chain crisis and a spike in raw material cost are driving the global economy into a chaos. Plants are shut down due to lack of parts and oil prices, which rose to $80 per barrel, are holding back the recovery of global economy. Large economies, including the U.S. and China are showing signs of slowdown. This could deal a blow to the Korean economy, which depends heavily on export. If soaring oil prices bring about inflation, domestic demand will inevitably shrink.

West Texas Intermediate (WTI) crude futures hit $80 a barrel for the first time in seven years on Monday (local time), caused by economic recovery from the COVID-19 pandemic and rising demand for heating. As a result, Nordea Bank Finland lowered its forecast for U.S. economic growth next year to 1.5% from 3.5%. This means soaring oil prices will press inflation upwards and reduce consumption. This trend will negatively affect the global economy and Korea’s export.

The supply chain crisis is also worsening. There are difficulties in logistics not to mention bottlenecks in the production of raw materials and intermediate goods. Prices are rising sharply as consumers are not getting products on time. When prices rise, companies normally increase production to sell their products at higher prices. However, under the circumstances, where production itself is difficult, there are concerns of stagflation, which refers to an economy that is experiencing no growth in production and an increase in inflation.

Korea’s consumer price index (CPI) showed an increase of 2% for six consecutive months through September, exceeding the target of 1.8%. Austerity measures, such as rise in interest rates, are necessary to control inflation but this could put a damper on economic recovery. This is why the Bank of Korea (BOK) decided to freeze its key interest rate on Tuesday.

Businesses are operating an emergency system to supply raw materials and parts. The government cannot afford to be complacent on brisk exports. It should take a joint response with businesses to ensure that there are no disruptions in the supply chain, and closely examine soaring oil prices’ impact on electricity cost. Another difficult challenge is establishing a meticulous interest rate policy, considering both prices and the economy. The current situation worldwide is too urgent for the government to just sit back and observe it.

# **China imports coal from Australia again**

The Financial Times reported on Monday that Chinese importers started to unload Australian coal as demand for coal surges despite the import ban imposed by the Chinese authority on Australian coal in retaliation against Australia for taking sides with the U.S. amid the U.S.-China rivalry. Critics argue that China gave in to Australia in the face of the suspension of coal-fired power plants and resultant power outages.

According to the newspaper, at major Chinese ports, coals are unloaded from Australian vessels that were stranded in the sea. Nick Ristic, lead dry cargo analyst at Braemar ACM Shipbroking, reported that approximately 45 tons of coal were unloaded so far. Global energy consulting firm Kepler also admitted to The Financial Times that 383,000 tons of Australian coal were unloaded from five carriers last month. The local traders consider the move as the Chinese authority’s signal that allows customs clearance.

In 2020, the Chinese government ordered the state-owned energy corporations and steel mills to “stop importing Australian coal” in retaliation against Australia, the world’s biggest coal exporter. As a result, Australia incurred a loss of approximately 3.9 billion dollars (approximately 4.6342 trillion won).

The import ban on Australian coal and Chinese President Xi Jinping’s “green energy initiative and carbon neutrality goal” initiated coal shortages in China. This led to power outages in north-east China, leading to factory shutdown and leaving millions of homes in darkness and cold. The local governments, including Jilin province, are struggling to import coal from Indonesia, Russia, Mongolia, and Kazakhstan, but the surging global demand for coal raised the price, making it difficult for China to have access to coal imports.

# **Aftermath of China’s Evergrande woes hits Sweden**

The financial woes of China’s Evergrande Group at the risk of bankruptcy with about 355 trillion won in liabilities have spread to Sweden in Northern Europe.

According to Bloomberg on Saturday, National Electric Vehicle Sweden AB (NEVS), a Swedish company jointly developing electric vehicles with Evergrande Group’s subsidiary, Evergrande New Energy Vehicle Group, recently dismissed 300 employees, which are almost half of its entire 670 workers at a factory.

“Due to the lack of funding from Evergrande New Energy Vehicle Group, we had to cut jobs and the development of electric vehicles has been suspended,” said a member of NEVS. “We are looking for a new partner and investors,” said Stefan Tilk, the CEO of NEVS.

Evergrande New Energy Vehicle Group has been preparing for the mass production of electric vehicles next year but failed to pay a Chinese plant equipment company. Some of its employees have not received salaries for some time and free meals for researchers at its R&D center have been suspended.

　

While Evergrande Group sold shares owned by its subsidiary at the end of September to secure 1.83 trillion won but the group’s crisis will continue as it has 750 billion won of interests to pay until the end of this year. As its main business, which is real estate, has been slowing down due to the Chinese government’s regulations and its prominent electric vehicle business is struggling, some say that the group won’t be able to recover on its own.

Evergrande Group failed to pay 55.9 billion won of dollar bond interests to creditors on Wednesday. It also missed the payment of 99.3 billion won of dollar bond interests on Sept. 23 and delayed the payment on Thursday.

# **Foreign minister’s remark siding with China under fire**

South Korean Foreign Minister Chung Eui-yong described the U.S.-led initiative to create an alliance consisting of the country’s allies, including South Korea, Japan, and Australia, against China as an “old-style Cold War mentality.” He also responded that it is “only natural” for China to employ “assertive diplomacy,” which is harshly criticized by the U.S. Coming as the Biden administration has begun in earnest to check China’s influence, the foreign minister’s remark quickly drew criticism for seemingly siding with China on the American soil.

Chung, currently visiting the U.S. in attendance on President Moon Jae-in for the United Nations General Assembly, said that it was “only natural” for China to become more assertive for the past few years, in a conversation meeting hosted by New York-based think tank Council on Foreign Relations (CFR). “China has become an economic superpower, and China today is not the same as China 20 years ago. It is natural for China to capitalize on its presence to gain a competitive edge in diplomacy, and I am unsure whether ‘assertiveness’ is the appropriate term to describe China’s diplomatic posture,” said Foreign Minister Chung. “China seeks to reflect its view as a member of global community, and we need to pay attention to what China is trying to say.”

When Fareed Zakaria, the host of CNN’s GPS and the moderator of the event, described the U.S., South Korea, Japan, and Australia as one bloc in opposition to China in his illustration of the diplomatic landscape of the Indo-Pacific, Foreign Minister Chung said such distinction is “the outdated mentality of Cold War,” as declared by China. Asked whether to join the Quad, the U.S.’s strategic forum in the Indo-Pacific to contain China, Foreign Minister Chung said that South Korea does not feel the urgent need to join the forum and that it is not necessarily mandated to choose between U.S. and China.

# **U.S. and China cooperate while competing against each other**

Memorial ceremonies were held in various cities across the U.S. on Saturday that marked the 20th year of the Sept. 11 attacks. The ceremonies were solemn on the 20th year of the the terrorist attacks that took 2,977 lives and the Afghanistan war that was ended at the end of August. Americans put up posts that go “Never Forget” and leaders including former and incumbent presidents asked for unity and consolidation against terrorism. Global leaders also highlighted international coalition. 　

The Sept. 11 attacks were an incident that triggered tremendous fear and rage in the liberalist international order in early 21st century and put the power of the U.S., the most powerful country on earth, on the testbed. The U.S., which won the cold war 10 years ago and led the international order, declared a war against terrorism that led to the Afghanistan war and the invasion of Iraq. But Afghanistan and Iraq were a pit for the U.S. The pitiable withdrawal of the U.S. troops from Afghanistan last month symbolized this. 　

The U.S. now plans to put itself out of the mire and focus wholly on the hegemony competition against China. The Biden administration did not hide its intention to hold China in check and envelop it with much more sophisticated and meticulous strategies than the previous administrations. It comes from the sense of crisis that China’s fierce challenge could put the U.S. in a perilous position. China showed its firm resolution to confront the U.S. while remaining vigilant. 　

It is a good thing that U.S. President Joe Biden agreed to take international responsibilities through interaction and communication in a call with Chinese President Xi Jinping which he had for the first time in the past seven months. Even though they still had different standpoints, but the call, which happened a day before the 20th anniversary of the Sept. 11 attacks, has a significant meaning. It has been 20 years since the U.S. declared a war against terrorism, but the war became a lot more difficult to win. Terrorism is not the only field that requires cooperation between the U.S. and China. There are numerous fields that need cooperation such as climate change and non-proliferation of North Korea’s nuclear programs. Fierce competition is inevitable in international politics. But confrontation between superpowers and power politics can only bring a disaster to the global community. They should cooperate while competing against each other.

### U.S. and China cooperate while competing against each

Memorial ceremonies were held in various cities across the U.S. on Saturday that marked the 20th year of the Sept. 11 attacks. The ceremonies were solemn on the 20th year of the the terrorist attacks that took 2,977 lives and the Afghanistan war that was ended at the end of August. Americans put up posts that go “Never Forget” and leaders including former and incumbent presidents asked for unity and consolidation against terrorism. Global leaders also highlighted international coalition. 　

The Sept. 11 attacks were an incident that triggered tremendous fear and rage in the liberalist international order in early 21st century and put the power of the U.S., the most powerful country on earth, on the testbed. The U.S., which won the cold war 10 years ago and led the international order, declared a war against terrorism that led to the Afghanistan war and the invasion of Iraq. But Afghanistan and Iraq were a pit for the U.S. The pitiable withdrawal of the U.S. troops from Afghanistan last month symbolized this. 　

The U.S. now plans to put itself out of the mire and focus wholly on the hegemony competition against China. The Biden administration did not hide its intention to hold China in check and envelop it with much more sophisticated and meticulous strategies than the previous administrations. It comes from the sense of crisis that China’s fierce challenge could put the U.S. in a perilous position. China showed its firm resolution to confront the U.S. while remaining vigilant. 　

It is a good thing that U.S. President Joe Biden agreed to take international responsibilities through interaction and communication in a call with Chinese President Xi Jinping which he had for the first time in the past seven months. Even though they still had different standpoints, but the call, which happened a day before the 20th anniversary of the Sept. 11 attacks, has a significant meaning. It has been 20 years since the U.S. declared a war against terrorism, but the war became a lot more difficult to win. Terrorism is not the only field that requires cooperation between the U.S. and China. There are numerous fields that need cooperation such as climate change and non-proliferation of North Korea’s nuclear programs. Fierce competition is inevitable in international politics. But confrontation between superpowers and power politics can only bring a disaster to the global community. They should cooperate while competing against each other.

# **Beijing Univ. professor criticizes Xi's ‘common wealth’**

The initiative for "common wealth” proposed by Chinese President Xi Jinping has been met with direct criticism by an economics professor at Beijing University, who argues that excessive government intervention can turn "common wealth” into “common poverty.” As opposing views of the government's policy directions have been subject to nationwide inspection, monitoring and regulation, the professor's argument is gathering public attention. 　

Economics Professor Zhang Weiying at Beijing University was quoted as saying in an article posted on a website of CE50 – a private academic organization for the public good, “If we lose faith in market forces and rely on frequent government intervention, it will lead to common poverty,” according to the South China Morning Post's report on Saturday. “If entrepreneurs have no motivation to create wealth, the government will have no money to transfer – the charity will become a river without headwaters,” he said in his critical article. Professor Zhang emphasized that a planned economic system tried to provide more welfare benefits to the poor but rather the result turned out to increase a level of poverty across society, adding that a market-oriented reform should happen as fast as possible so that a greater level of fairness can be shared further.

Mr. Zhang has worked at Beijing University since 1994. College-educated in Xian, he obtained a master's and doctorate degree at Oxford University in Britain. Back in 2008, he founded the National School of Development - a major think tank of Beijing University. He stirred controversy by stating in October 2018 that the main contributor to China’s 40 years’ rapid growth is not the Chinese model of development but a combination of marketization, entrepreneurship and technological accumulation of 300 years of the West.

As of now, Zhang's critical article is deleted both on the website of CE50 and his personal WeChat account. The article is prohibited from being sent to other users on WeChat.

### Biden says U.S. should deal with new threats

U.S. President Joe Biden declared the end of a war in Afghanistan and emphasized that it is now time for the U.S. to deal with the 21st-century threats it is facing, such as China and Russia.

“The world is changing. We’re engaged in a serious competition with China. We’re dealing with the challenges on multiple fronts with Russia. We’re confronted with cyberattacks and nuclear proliferation,” President Biden said on Tuesday (local time), just one day after the U.S. completed its withdrawal from Afghanistan, at the White House. “And there’s nothing China or Russia would rather have, would want more in this competition than the United States to be bogged down another decade in Afghanistan,” he added. “We have to shore up America’s competitiveness to meet these new challenges in the competition for the 21st century.”

“As we turn the page on the foreign policy that has guided our nation the last two decades, we’ve got to learn from our mistakes,” the president said, emphasizing that the U.S. should focus on its biggest threat, which is China, not the Middle East. His foreign policy direction was very clear in his statement that the U.S. will stop playing the role of the global policeman and focus on addressing the current and future threats in foreign affairs and security based on its own national interests. He seems to have mentioned ‘nuclear proliferation’ in consideration of the growing nuclear threats from China and North Korea.

The White House repeatedly mentioned on Tuesday the need for dialogues with North Korea regarding the North’s resumption of Yongbyon nuclear facilities by saying that they have left the door open. “Our offer remains to meet anywhere, anytime without preconditions,” White House spokeswoman Jen Psaki said to a question asking about the current understanding of what North Korea is doing with their nuclear program and if there is any renewed outreach to Kim Jong Un and his regime. “South Korea and the U.S. are discussing various measures to get involved in North Korean matters, including consultation on joint humanitarian assistance for the North,” Special Representative for Korean Peninsula Peace and Security Affairs Noh Kyu-duk said in a press conference during his visit to the U.S. “South Korea and the U.S. share the common stance to be fully prepared to pursue things anytime as soon as North Korea reciprocates.”

# **Heavy rain affects 3 million in China’s Henan province, 33 dead**

Zhengzhou, the capital city of Henan Province, China, suffered the worst flood in 60 years, leaving at least 33 dead and around 256,000 evacuated as of Thursday. With the number of the displaced hitting the mark of 3 million, Tsai Ing-wen, the president of Taiwan, sent her message of concern to Henan, setting a rare precedent.

According to the provincial authorities of Henan, at least 33 were drowned by the downpour, killing 8 more from a day before. Including the 12 passengers in Zhengzhou flood alone, the death toll soared with more victims found from the landslides in parts of the province. The number of people affected by the flood stands at 3 million and 4,000, with 256,000 having been evacuated. “We’ve suffered direct economic losses worth 1.2 billion and 20 million yuan (around 223.9 billion and 65 million won),” a provincial official of Hanan said. Hanan is 1.6 times larger than South Korea, with a population of 100 million.

Tsai Ing-wen, the president of Taiwan, expressed her concern and sent her condolences to the victims, saying she hopes that “normal life could resume as soon as possible,” according to Taiwan’s Central News Agency.

The supply of the iPhones is expected to be affected by the heavy rain as Taiwan’s Foxconn factory is located in Zhengzhou. Foxconn operates three factories in Zhengzhou, with some 350,000 workers on 90 production lines. Foxconn’s Zhengzhou factory is responsible for more than half of the global production of the iPhones. “All three Foxconn factories in the city experienced hours of power outages Tuesday evening as the city went through a blackout,” the Wall Street Journal reported, quoting the employees of Foxconn. An assembly worker said he was submerged up to his thighs, with some workers moving production equipment and inventories to prevent them getting wet.

# **Sherman: U.S. will discuss with China over N. Korea policy**

U.S. Deputy Secretary of State Wendy Sherman, the No. 2 diplomat in the U.S. State Department, who is currently visiting South Korea, met President Moon Jae-in on Thursday and stated that she will have a thorough discussion on the U.S. policy towards North Korea in a forthcoming visit to China. Washington and Beijing are known to have recently resumed regular high-level talks strictly focused on diplomatic relations, including concerns over North Korea, while keeping a distance from the recent contention over economic matters. U.S. Deputy Secretary of State Wendy Sherman and Chinese Foreign Minister Wang Yi are scheduled to meet on Sunday and Monday.

President Moon met Deputy Secretary Sherman at Cheong Wa Dae and requested that the U.S. play an active role to restart discussions between Washington and Pyongyang. “The U.S. looks forward to a prompt positive response on the resumption of dialogue from North Korea. We hope to continue our closely coordinated efforts for dialogue with North Korea,” Ms. Sherman said.

According to a person familiar with foreign affairs, the U.S. and China have recently reopened a diplomatic channel, which had been practically severed during the Trump administration. An official declined to be named said that the resumed talks are expected to revolve around matters concerning diplomatic relations, including North Korea policy, which both Washington and Beijing agreed on the need to cooperate on. The source further said that the two countries will adopt the two-track approach that separates economy from diplomacy.

# **Beijing sent over 50 defectors in detention back to N. Korea**

The Chinese government sent more than 50 North Korean defectors who had been in detention in Shenyang, Liaoning Province back to North Korea, Radio Free Asia reported on Friday. China had sought to repatriate them to the North since April, but Pyongyang reportedly declined to receive them several times due to concern over possible inflow of Covid-19 patients, which resulted in delays. RFA said the ill-fated defectors will most likely face execution in the North.

The Chinese authority sent North Korean defectors, who had been detained at the Shenyang Detention Center for one or two years, via the customs office in Dandong, China on July 14. According to the RFA report, the defectors were carried in two buses, and dozens of Chinese police officers were watching them around the customs office from early in the morning, while blocking people from taking photos or video.

The 50-plus defectors, who were repatriated to the North, included a number of North Korean soldiers and Air Force pilots. A 30-something female defector who married to a Chinese man has a 12-year-old son. She reportedly earned a sizable amount of money in China. “The woman was repatriated to the North for a second time, and there is no way of knowing her fate. Her husband tried to bribe officials to save her, to no avail,” an informed source said. According to RFA, the Shenyang Detention Center still has a number of North Korean defectors in detention, apart from the 50-plus repatriated defectors.

As the customs office in Dandong reopened on the day after shutdown for Covid-19 quarantine on the day, 98 people including Chinese nationals who were staying in the North and representatives of the North’s trade office moved to China.

# **First human death due to monkey B virus reported in China**

Patients of rare viral infections transmitted from the monkey have been reported in China and the U.S. in succession.

According to China’s state-run Global Times on Saturday, a 53-year-old veterinarian who was working at a primate laboratory in Beijing died on May 27 while taking treatment for “monkey B virus.” The veterinarian was infected with the virus while dissecting two dead monkeys in March this year. He then came down with symptoms including nausea and vomiting beginning one month later. The Global Times said it is the first case of human infection with the animal virus in China. There are reportedly no additional infections among people who came into contact with the veterinarian.

The monkey B virus is a type of the herpes virus. A person can be infected with the virus if he or she is bitten or mauled by the infected monkey, or when secretion from the monkey is splattered onto human mucous membrane such as an eye. The virus can transmit between humans, with a case fatality rate reaching as high as 70 to 80 percent.

Meanwhile The Washington Post reported Friday a person was infected with the monkeypox, a rare infectious disease in Dallas, Texas in the U.S. The health authority of the Dallas County said the Dallas resident who had visited Nigeria was confirmed as a patient infected with the virus.

The patient took a Delta Airline flight in Lagos, Nigeria on July 8 and arrived in Dallas on July 9 via Atlanta. The U.S. Centers for Disease Control and Prevention is tracking and contacting people who came into contact with the patient inflight. “Due to mandatory mask requirement for the prevention of Covid-19, there is little chance that the virus has spread to other people through droplets,” the Dallas County health authority said.

# **China criticizes U.S. for several decades’ menace to N. Korea**

China has raised an unprecedentedly critical voice against the United States that it is supposed to show some remorse for intimidating and pressing North Korea for several decades.

Chinese State Councilor and Foreign Minister Wang Yi said in the 9th World Peace Forum in Tsinghua University on Saturday that nuclear issues on the Korean Peninsula have been dragged with ups and downs all the way for the past 30 years, according to the Chinese Foreign Ministry on Sunday. "The default option to make is to resolve issues peacefully based on dialogue and negotiations. We should take the right path by working on denuclearization and peace-making efforts at the same time,” he said.

Minister Wang said that issues regarding the Korean Peninsula are unfolding right in front of China's doorstep, stating that the Chinese government will have a constructive role in ensuring stability on the Korean Peninsula with consistency. Regarding Washington's recent moves including U.S. special representative for North Korea Sung Kim's visit to Seoul increase possibilities of the resumption of the U.S.-North Korea dialogue front, Mr. Wang said that Beijing is supportive of all kinds of Washington's acts and statements that can bring peace and stability on the Korean Peninsula.

In the address on Saturday, Minister Wang clarified in response to criticism from the West for issues with the Xinjiang Uygur and human rights in Hong Kong that there should be no intervention in domestic affairs occurring in China. Defining Taiwan as an undividable part of Chinese territory, he argued that China's pursuit of a nationwide peaceful unification has been maintained for so long, warning that it is a wrongful and risky act for some forces in the United States to support the independence of Taiwan. As for Japan's decision to discharge contaminated nuclear water from nuclear reactors in Fukushima, the Chinese minister stressed that the Japanese government is not supposed to discharge contaminated water in the Pacific Ocean before it listens to the international community's reasonable concerns and worries and completes full negotiations with related neighboring nations and international organizations.

The World Peace Forum, which was founded by Tsinghua University in 2012, has become the only global forum to discuss international security issues by a non-governmental organization in China.

# **N. Korea resumes trading with China in more than a year**

North Korea limitedly resumed resource trading with China recently, revolving around the border areas. It seems that the North began “unofficial” trading while keeping the border officially closed to console residents who have complaints built up due to the lack of daily necessities. Pyongyang has closed off the border for longer than a year for fear that COVID-19 would spread. Some interpret this as a signal that the two countries started to tighten up coordination for the coming 60th year of signing an amicable treaty on July 11.

According to a South Korean government source on Friday, some resources have been exchanged in late June in trading hubs including Dandong, Liaoning province in China. The source added that the exchange was done by land in a very limited level using trucks and other vehicles.

# **Xi: Those bullying China will have their heads bashed and bloodied**

Celebrating the 100th anniversary of the founding of the Communist Party of China on Thursday, Chinese President Xi Jinping said foreign forces that bully China will “find their heads bashed bloody against a great wall of steel forged by over 1.4 billion Chinese people.” President Xi declared his intention to take a resolute action if foreign countries, such as the U.S. intervene in its own issues with Taiwan and Hong Kong, saying it is China’s “historic task” to complete reunification with Taiwan. His remarks are regarded as a declaration of war against the Joe Biden administration, which has continued pressure on China since it took office in January.

“With a history of more than 5,000 years, China has made indelible contributions to the progress of human civilization,” President Xi said at the 100th anniversary celebrations held at Tiananmen Square on Thursday. “No one should underestimate the resolve, the will, and the ability of the Chinese people to defend their national sovereignty and territorial integrity.”

Speaking from the podium, where Mao Zedong proclaimed the People’s Republic of China in 1949, wearing the same gray buttoned suit like Mao’s, President Xi presented the country’s second centenary goal of fully building a modern socialist country and Chinese dream of national rejuvenation. President Xi **said** that his country will strive to strengthen its hegemony in the international society now that the country has realized the first centenary goal of building a moderately prosperous society.

# **Beijing is practically under martial law with 100th anniversary of Chinese Communist Party’s foundation**

The Taiwanese Liberty Times reported on Wednesday that China is practically under martial law with the 100th anniversary of the Chinese Communist Party’s foundation on next Thursday. While fireworks and other cultural performances are being prepared to celebrate the 100th anniversary, the movement of people and resources is strictly controlled to prevent any complaint against the long-term rule by the Communist Party and President Xi Jinping.

The Chinese authorities have been conducting two-step examinations for all packages heading to Beijing since Monday. First, a shipping company of the dispatch location scans all packages with an X-ray machine and puts a sticker for a completed security check on them. Then, another round of examinations is conducted in Beijing once packages arrive. The authorities said such a two-step examination for all packages will be carried out until next Wednesday.

Many people are worried about potential delays in shipping as a result. As a country with highly developed online shopping and shipping industries, many packages are delivered to Beijing from Guangdong Province, Shanghai, and Shenzhen in the southern part of the country. What normally takes two to three days for shipping will take longer than a week due to the two-step examination. “Ordering food online from a restaurant in the outskirts of Beijing has become practically impossible,” customers complained.

# **Pres. Biden orders CIA to investigate origins of COVID-19 virus**

As disputes about the origins of the COVID-19 virus continue, U.S. President Joe Biden ordered further investigation into the matter based on the judgment that U.S. intelligence authorities have diverging opinions.

President Biden announced in a statement on Wednesday that he ordered in March intelligence agencies, including the CIA, to investigate whether the COVID-19 virus started from contacts between humans and animals outside a lab or was accidentally leaked from a lab.

The president explained that based on the recent briefings, he believed that the CIA and other intelligence agencies had not yet reached a consensus on the matter. He said two agencies lean toward the animal origination theory, while one agency puts more weight on the lab leakage theory – both with only a low or intermediate level of reliability. The president asked them to redouble their efforts to collect and analyze information that could bring us closer to a definitive conclusion and report back to him in 90 days.

It is unusual for the U.S. to reveal the unconcluded activity of its intelligence agencies. Bloomberg News reported that the U.S. intended to deliver a message that it is not excluding the possibility of the virus leaked from a lab in China. “The United States will also keep working with like-minded partners around the world to press China to participate in a full, transparent, evidence-based international investigation and to provide access to all relevant data and evidence,” Biden put pressure on China in the statement.

China opposed the statement and criticized the U.S. for politicizing the origin of the COVID-19 virus. “Since the outbreak of COVID-19 last year, some political forces have been fixated on political manipulation and blame game, while ignoring their people's urgent need to fight the pandemic,” the Chinese embassy in the U.S. made a statement on its website on Thursday without mentioning President Biden’s statement.

Even though the World Health Organization published a report that the likelihood of the virus leaked from the Wuhan Institute of Virology in China is low, disputes about the origins of the virus are expanding as The Wall Street Journal reported that the lab could in fact be one.

# **Chinese ambassador takes issue with S. Korea’s mentioning Taiwan**

Chinese Ambassador to South Korea Xing Haiming said on Wednesday that it would have been better if the issue of the Taiwan Strait and the South China Sea had not been mentioned in a joint press statement issued by the leaders of South Korea and the U.S. following their summit. The ambassador took issue with Taiwan being mentioned in the South Korea-U.S. joint statement while the South Korea government has attempted to ease China’s discomfort by saying a mention of Taiwan in the joint statement was theoretical and principled. Ambassador Xing asked for a balanced diplomacy between the U.S. and China, saying he hopes that other nations including South Korea play a role in reconciling the U.S. and China.

Talking to an MBC program on Wednesday, Ambassador Xing said while he appreciates Seoul’s efforts to not directly mention “China” in the joint statement, some part of it appears to be targeted at Beijing. He went on to say that Seoul recognized Taiwan as part of China when it established diplomatic relations with Beijing and he thinks the issue of the South China Sea can be addressed in cooperation with neighboring countries.

As for the mention of the Quadrilateral Security Dialogue (QUAD) in the joint statement, Ambassador Xing said China has a different idea about the global order shaped by one country or a few countries, adding the U.S. has a tendency to form groups to besiege China and it would be highly appreciated if South Korea could consider China’s position. He made it clear that China is opposed to South Korea’s participation in QUAD.

However, Ambassador Xing’s reaction was not stronger than China’s protest made immediately after the South Korea-U.S. summit. Beijing appears to have decided to take a step back and watch Seoul’s actions down the road, considering that Seoul tried to appease Beijing by saying the two countries have “special ties” after Beijing warned not to “play with fire.” China reportedly thought that it would be difficult to contain the efforts by the U.S. to unite its allies against China if South Korea were to turn its back on it. As for plans for Chinese President Xi Jinping’s visit to South Korea, Ambassador Xing said there is nothing to say for certain at the moment.

# **Kim Yeon-koung chooses to play in China for her next season**

Kim Yeon-koung’s return to China was predictable. However, it is still unknown where she will go afterward.

According to her agent Lianat on Thursday, Kim is in the final process of closing an agreement with the Shanghai Bright Ubest Women's Volleyball Club. The contract lasts one season.

“Beijing BAIC Motor was also interested in Kim. However, it was said that Kim preferred the team that she was with during the 2017-2018 season,” said a source familiar with transfers in volleyball. “The shorter Chinese league will be less physically straining and easier to prepare for the next season for Kim who is in her mid-30s.”

When she first decided to play in the Chinese league four years ago, Kim mentioned that the higher number of games means more physically challenging. The Shanghai Bright Ubest Women's Volleyball Club only played 12 games last season, while Kim played a total of 41 games last year from Jecheon-Korean Federation of Community Credit Cooperatives Volleyball Cup to V-league Championships. As twin sisters Lee Jae-yeong and Lee Da-yeong dropped out of the team due to scandals about their history of bullying, Kim also had to play with psychological stress.

# **Hong Kong closes Taiwan trade office amid rise in diplomatic tensions**

Hong Kong closed the Hong Kong Economic, Trade and Cultural Office in Taiwan, which served as its representative office. The unilateral decision came amid strained ties between China and Taiwan, leading to speculations that Hong Kong is considering suspending ties with Taiwan affected by China’s efforts to diplomatically isolate Taiwan.

According to the South China Morning Post (SCMP) on Wednesday, the government of Hong Kong announced on its website the previous day that it is temporarily suspending operations at its representative office in Taiwan. There was no explanation as to the reason for the suspension and when normal operations will be resumed. “We express deep regret at today’s unilateral decision by the Hong Kong government,” Taiwan’s Mainland Affairs Council said in a statement.

The Hong Kong Economic, Trade and Cultural Office in Taiwan was opened in 2011 to facilitate economic and trade exchanges between Hong Kong and Taiwan. Taiwan also has similar office in Hong Kong. Although these offices are not official government organizations, they have served as consulates, protecting their overseas citizens.

Unlike Hong Kong and Taiwan, which operate such representative offices, China and Taiwan do not run such offices in each other’s countries. Hong Kong was able to establish its own policies because the principle of “one country, two systems” applied to Hong Kong before Xi Jinping came to power in 2013. The situation began to change after 2013 as Xi Jinping underlined the adherence to the “one China” principle. Hong Kong is increasingly affected by China in recent years. China passed a new national security law in Hong Kong last year and approved a plan to reform the electoral system of Hong Kong this year.

# **China lands on Mars following its touchdown on moon**

The Chinese unmanned rover Zhurong successfully landed on Mars, making China become the third country in the world to do so. Following the United States, China is the second nation across the globe to land a mobile rover on Mars for surface exploration.

Separating from the Tianwen-1 mission orbiting Mars, China’s red planet rover Zhurong sat down south of the Utopia Planitia region at 8:18 a.m. on Saturday (local time), reported Xinhua. Chinese President Xi Jinping sent a congratulatory telegram, defining the landing as one of the greatest milestones in China’s interplanetary voyage. He went on to say that China took a leap forward in interplanetary exploration along with its travel from Earth to the moon. 　

The Zhurong is a six-wheeled mobile robot apparatus – 2.6 meters in width, 3 meters in length, 1.85 meters in height and 240 kilograms in weight – designed to carry out an exploration mission for at least 90 Mars days or 24 hours and 37 minutes in earth days. The rover robot is the first of its kind, which is equipped with a radar system that allows it to explore down to as deep as 100 meters. China hopes to discover traces of water or ice and analyze soil and rock components. China made it to Mars at the second try. Back in 2011, it attempted to explore the red planet in cooperation with Russia but to no avail.

China has recently sped up with its ambitious space programs on Mars and moon explorations, independent space stations, etc., rising as a rival in aerospace to the Unites States. In 2019, China sent the Chang’e-4 to the back side of the moon for the first time in human history. Furthermore, last December, the Chang’e-5 traveled to the moon to collect and bring lunar soil to the Earth.

In the meantime, the United States is only throwing a vigilant glance at China’s growing presence in aerospace travel. When China’s Changzheng-5 on May 9 lost control to fall back down to the Earth with debris scattered across the Indian Ocean, NASA Administrator Bill Nelson issued a critical statement, saying, “It is clear that China is failing to meet responsible standards regarding their space debris.” Behind tensions between the two nations lies a sense of rivalry in dominance in the field of space travel, analyze global media outlets.

# **Debris of China’s 22-ton space rocket could fall onto Earth**

Debris of China’s space rocket Changzheng (Long March) 5B will likely reenter the earth’s atmosphere around this weekend. The South Korean and U.S. military authorities said the Korean Peninsula is not included in candidate sites for the rocket’s crash, but they agreed to strengthen cooperation to prepare for an emergency situation.

The space information and situation room of the South Korean Air Force held a joint video meeting with the Joint Aerospace Operation Center of the U.S. Space Operations Command on Friday, and agreed to share surveillance information on the trajectory of debris from the rocket, which is expected to reenter the earth’s atmosphere on Saturday or Sunday. Considering the size of the Changzheng 5B rocket, debris will likely fall onto the earth, rather than disappearing even after entering the atmosphere. The South Korean and U.S. military authorities predict the debris will fall into the Atlantic Ocean, but the possibility for the debris to deviate from its original trajectory when reentering the atmosphere cannot reportedly be ruled out.

China launched and put into orbit the Tianhe, a core module designed for constructing its own space station, on April 29, but the Changzheng 5B rocket, which transported the Tianhe, went out of control, and the debris weighing 22.5 tons is falling onto the earth 1 to 2 kilometers daily from an altitude of 280 kilometers.

The South Korean military authority plans to share information on the projected trajectory of debris by using the U.S. Space Operations Command’s surveillance assets. In addition, to be prepared for the worst situation wherein debris falls into the skies over the Korean Peninsula, the military is reportedly considering using an electrooptical satellite surveillance system, or a high-performance radar satellite tracking system, which are under development for use in combat mission.

# **Blinken remarks China’s “more aggressive” actions**

“China is acting more repressively and aggressively,” U.S. Secretary of State Antony Blinken said on Sunday (local time). He repeatedly emphasized cooperation with allies by saying that the key of the U.S. policy toward China is to uphold the international community’s rules-based order against China.

“What we've witnessed over the last several years is China acting more repressively at home and more aggressively abroad. That is a fact,” said Blinken during an interview with CBS News’ “60 Minutes” on Sunday. He argued that China is the one country in the world that has the military, economic, diplomatic capacity to undermine or challenge the rules-based order. “I think that over time, China believes that it can be and should be and will be the dominant country in the world,” he said when asked what the goal of China is. However, he drew a line by saying that it is profoundly against the interests of both China and the United States to get to that point or even to head in that direction when the host asked if he thinks the U.S. is heading towards some sort of military confrontation with China.

“President Biden made clear that we have real concerns about the actions that China has taken, and that includes the theft of intellectual property,” said the secretary of state. He even directly criticized China’s human rights issues by calling the Chinese authorities’ violation of human rights against the Uyghurs in Xinjiang a genocide. “Look, we don't have the luxury of not dealing with China. There are real complexities to the relationship, whether it's the adversarial piece, whether it's the competitive piece, whether it's the cooperative piece.”

When the host mentioned that China's gross domestic product is expected to surpass the U.S. as early as 2028, Blinken said that what really makes the wealth of a nation is human resources and the ability of any one country to maximize its potential. “I think we're in a much better place to maximize that (than China).” He also reconfirmed that the U.S. will cooperate with its allies and partners that share the same values.

# **Pyongyang- Beijing border looks set to reopen**

Amid reports that North Korea and China will resume trade at the end of April, evidence of opening the border between the two countries, including the removal of a train shield is emerging.

According to Voice of America on Saturday, satellite imagery showed that the tunnel-shaped shield installed in October 2020 near the Sinuiju station in North Korea was removed on March 31, 15 days after the 400-meter cover was captured in satellite imagery released by Maxar Technologies. It is presumed that the facility was built to protect trains from natural elements such as snow and rain while they were not in use.

It is also said that large volumes of goods are waiting in Liaoning and Dandong, which are Chinese trade hubs across Sinuiju, along with other border cities. Freight trains marked with “Sopo,” a city on the outskirts of Pyongyang, were seen at a train station in Dandong.

Trade by ship between North Korea and China were resumed last month. Pyongyang imported 12.97 million dollars’ worth goods including fertilizer in March, according to the Chinese General Administration of Customs.“ Most imports from China were shipped to North Korea last month,” said a source familiar with the relations between the two countries. It is said that trains carrying fertilizer will head for the North starting Tuesday at the earliest.

# **hina urges S. Korea to make efforts to alleviate N. Korea sanctions**

Security advisors of South Korea, Japan and the U.S. highlighted the “imperative for full implementation” of relevant UN Security Council sanctions resolutions against Pyongyang. On the same day, China demanded South Korea to make efforts to resolve reasonable security concerns of North Korea. China has been using the term “reasonable security concerns” to refer to guarantee the continuity of the North Korean regime and alleviate sanctions against the rogue state. 　

“They agreed on the imperative for full implementation of relevant UN Security Council resolutions by the international community, including North Korea, preventing proliferation, and cooperating to strengthen deterrence and maintain peace and stability on the Korean Peninsula,” said the White House in a joint statement after having trilateral talks at the U.S. Naval Academy in Maryland on Friday (local time). “National security advisors shared their concerns about North Korea’s nuclear and ballistic missile programs and reaffirmed their commitment to address and resolve these issues through concerted trilateral cooperation towards denuclearization.” The joint statement highlighted deterrence while pressing North Korea and China to observe the sanctions.

“South Korea, Japan and the U.S. agreed on the urgency and diplomatic necessity to resolve the North Korean nuclear issues and that our three countries should continue to make efforts for early resumption of the U.S.-North Korea talks,” said National Security Office Director Suh Hoon. But the resumption of talks was not included in the statement published by the White House. 　

“All related parties should actively try to maintain peace and stability on the Korean Peninsula and effectively resolve the concerns of North Korea,” said Chinese Foreign Minister Wang Yi in talks with his South Korean counterpart Chung Eui-yong on Saturday in Xiamen, China.

# **HCE wins orders worth of 250 billion won in China**

Hyundai Construction Equipment (HCE) has won its largest-ever orders in China. HCE said on Monday that it has achieved orders of supplying 2,200 units of construction equipment to Chinese customers, the biggest ones since the company’s entry into the Chinese market. The orders, which are worth 250 billion won, include 5.5- to 85-ton excavators and wheel loaders and account for approximately 30 percent of the company’s sales last year, considering that it received orders for 7,800 units of construction equipment in China. The company plans to complete the delivery of the equipment by May.

The Chinese government is recently making great investments in social overhead capital (SOC) in order to cope with the economic downturn caused by the COVID-19 pandemic. HCE introduced 13 new products, including large excavators, amphibious excavators, pile hammers, and nippers to the Chinese market early this month, thinking that the Chinese construction equipment market has entered a boom cycle.

An increase in demand for construction equipment is expected in emerging markets, including China, as the prices of raw materials are rapidly rising, with the price of iron ore approaching $170 per ton. HCE expects that this year’s demand for construction equipment in China will rise 8 percent year-on-year to 315,000 units.

# **China simplifies visa requirements for foreigners inoculated with Chinese vaccine**

The Chinese government has decided to simplify visa requirements for foreigners who enter the Chinese mainland via Hong Kong if they receive COVID-19 vaccines produced by China such as by exempting them from providing a negative PCR test result.

According to the Chinese state-run media Global Times on Sunday, the Office of the Commissioner of the Chinese Ministry of Foreign Affairs in Hong Kong announced a new policy on its website on Friday where a negative PCR test result as well as health and travel certificates will not be required for any visa applicants who got two doses of the China-made vaccine or who got their first jab 14 days prior to the application.

Under the current policy, foreigners are required to provide a negative PCR test result that was issued within 72 hours before their arrival. The simplified application process will also be applied to those who apply for a visa for reasons such as visiting relatives or attending a funeral. The new process will be put in place on Monday.

“China has simplified the visa policy for foreigners applying to enter the Chinese mainland via Hong Kong who have received China-produced COVID-19 vaccines, which experts said shows the authorities' confidence in the efficacy and safety of the vaccines,” said Global Times. Many experts say, however, that streamlining visas is aimed at providing its vaccines more widely, considering that there are many people who wish to enter the Chinese mainland.

Meanwhile, only three percent of the Chinese population were inoculated as of late February due to a lack of public confidence in the vaccine, raising doubts about the impact of the new policy.

# **Biden administration plans to respond to China by working with allies**

The Biden administration announced Wednesday (local time) a tentative security strategy that targets China. It came out 40 days after the new president took office and holds the direction and blueprints of diplomacy and security policies of the U.S. 　

U.S. Secretary of State Antony Blinken said China represented America’s “biggest geopolitical test of the 21st century.” He also said, “China is the only country with the economic, diplomatic, military and technological power to seriously challenge the stable and open international system— all the rules, values and relationships that make the world work the way we want it to.” 　

“Where we have pulled back, China has filled in,” he said and emphasized that he would respond to it by working with allies and partners. “Our alliances are what the military calls force multipliers. They’re our unique asset,” he said. “Our combined weight is much harder for China to ignore.”

# **Xi says China should prepare for crisis**

Chinese President Xi Jinping has emphasized jeopardies faced by China by mentioning “white rhino” and “black swan.”

According to China's state-run channel CCTV, President Xi attended a group training for the Chinese Community Party’s central politburo on Thursday where he said, “China should properly forecast various jeopardies and challenges, and should be well prepared for white rhino and black swan incidents.” The white rhino refers to a jeopardy that can be predicted but can be easily overlooked, while the black swan is a risk that has a slim chance of occurring but causes massive damage once it occurs.

As a massive transformation that is unprecedented over the past 100 years is now happening around the world, China should recognize and deeply understand complex international situations and create an environment favorable to China’s development, the Chinese president added. Xi made the remarks as experts predicted that the U.S. will not change the stance of bilateral rivalry even after the inauguration of the Joe Biden administration. U.S.-China conflicts that were aggravated during the Donald Trump administration has now expanded from trade to diplomacy and national security.

Xi has been mentioning white rhino and black swan whenever emphasizing a crisis. When the Covid-19 pandemic was spreading wildly in February last year, the Chinese president said that the entire world is agonizing how to prevent white rhino and black swan in the area of quarantine” in his calls with the Indonesian president and the Malaysian prime minister. When China was announcing the lowest economic growth rate in 28 years in January 2019, Xi said, “International situations are difficult to predict and surrounding environment is complex and delicate. We have to be highly vigilant against black swan and prevent white rhino.” He even compared preventing jeopardies such as white rhino or black swan to a war.

# **Anta Group’s shares get boost after Xi Jinping showcases parka**

After Chinese President Xi Jinping made appearance clad in a parka from Anta Group on Monday, the company’s stock listed on the Hong Kong stock market jumped about 10 percent on Tuesday.

According to the South China Morning Post, President Xi was seen wearing a cobalt blue Arc’teryx parka when visiting facilities for the Winter Olympics out the outskirts of Beijing on the day to inspect preparation for the event. The brand originally belonged to Amer Sports of Finland, but was taken over in 2019 by Anta Group, an official sponsor of the 2022 Winter Olympic Games in Beijing. The Arc’teryx parka product generally costs 700 U.S. dollars to 2,000 dollars in online shops. The exact price of Xi’s parka remained unknown.

Anta has 15 percent of the Chinese sports gear market share to rank third overall after Nike (23 percent share) and Adidas (20 percent). Xi appeared wearing an Anta jacket in 2017 as well. The Chinese delegation that participated in the 2018 Winter Olympics in Pyeongchang, South Korea, also used Anta products as its uniforms. Analysts say Xi intentionally made such appearance to promote a Chinese apparel maker. In fact, Anta Group’s share jumped nearly 10 percent on Tuesday.

# **Why does US counter China’s initiative?**

“Can Asians Think?” is the provocative title of a book written by Kishore Mahbubani in 1993. In the book, Mahbubani, dean of the Lee Kuan Yew School of Public Policy at National University of Singapore, warned that the tide was changing and Asia had more to teach the West. The author underlined that it was time to break away from the colonialist thinking, “Can Asians think?” but to study what Asians think.

Twenty years later, Asia’s elevated status is demonstrated by economic figures. The Regional Comprehensive Economic Partnership (RCEP) signed by 15 countries, including South Korea, China, Japan, and Australia in November last year was called the biggest free trade agreement (FTA) in the world since the combined population and gross domestic product (GDP) of the member countries totaled 2.2 billion and 26.2 trillion dollars, respectively. In his new book, Mahbubani affirms that the global landscape will be rewritten from “America First” to “Asia First,” introducing unknown sides of Asia.

The author begins with the ancient Asian civilization. He gives a brief summary of the civilizations of India, West Asia, and East Asia, which were hidden in the shadow of the world history that focused on the West, such as Ancient Greek civilization. He then introduces the basic knowledge and events on major political, economic, social, and cultural aspects of Asia. The author also deals with topics of the time, such as why Australia and Russia began Asianization early on and why the U.S. wants to counter China’s “One Belt One Road” initiative.

It is interesting that the past 20 years, from the perspective of Asia, has been the era of incompetence by George W. Bush, the era of insincerity by Barack Obama, and the era of uncertainty by Donald Trump, and that, contrary to popular belief in the West, Asia is not centered around China. While helping readers to look at the current status of entire Asia, including India and Southeast Asia, the book has its limits as it sets Westerners, who are ignorant of Asia, as its readers.

Born in India, the author works as an international relations expert in the U.S. and Europe and is currently settled in Singapore. The original title of the book is “The Future is Asian.”

# **China’s Chang’e-5 mission returns with Moon samples**

China’s “Chang’e-5” mission returned to Earth with Moon samples early on Thursday morning. This is the first lunar sample collection since the Soviet Luna mission brought its samples home 44 years ago. Chinese media celebrated the achievement, saying that the nation has joined the ranks of space powers.

According to Xinhua News Agency, the Chang’e-5 spacecraft landed on a high volcanic region called “Mons Rümker.” Being relatively new, rock and soil from this terrain are expected to help understand the volcanic activity on the Moon. Chinese state media Global Times reported the Chang’e-5 mission has become the “first” venture that explored the terrain after the Chang’e-4 mission became the first probe to land on the far side of the Moon in January last year.

It is also worth noting that two kilograms of samples have been retrieved, six times the 330-gram sample collected by the Soviet Luna mission. This was possible because Chang’e-5’s return journey to Earth required much less fuel. Unlike the Soviet Luna mission that used the fuel in the ascending vehicle to return to Earth, a robotic vehicle docked with a module in the Chang’e-5 venture, which did not require as much fuel.

# **Kimchi and paocai are two different foods,’ Chinese state media says**

As the Chinese press is under criticism for causing controversy about where kimchi originated from, a Chinese state media company said kimchi and paocai are completely different. It seems to take a step back by explaining the recent controversy as a misunderstanding in translation.

“This confusion that stems from an innocent lost in translation ignited a feud,” China’s state-run English newspaper Global Times said, explaining differences between kimchi and paocai on Wednesday (local time). “The two food genres, despite being both called paocai in Chinese term, vary a lot in making methods and selection of raw materials,” the newspaper said. “Kimchi refers to a kind of fermented cabbage dish that plays an integral role in Korean cuisine, while paocai, or Sichuan paocai, refers to pickled vegetables that are popular originally in Southwest China's Sichuan Province.” While the English newspaper used the term ‘kimchi,’ other Chinese media companies use the term ‘paocai’ to refer to kimchi.

The Global Times also reported that Baidu Baike, China's Wikipedia-like platform, deleted the phrase "Korean kimchi originated from China” on Tuesday upon the request of Seo Kyoung-duk, a professor at South Korea's Sungshin Women's University. According to the newspaper, Chinese experts refuted it as "unnecessary fuss," adding that Baidu Baike is a website that can be edited or created by any registered user. At the same time, the article pushed the responsibility for causing the controversy to South Korea, saying that this confusion that stems from an innocent lost in translation ignited a feud with defenders of kimchi culture on South Korean social media, who accused China of attempting to "steal our culture."

The state-run newspapers of China, which caused the kimchi controversy, also said on Wednesday that kimchi and paocai are different foods. The recent controversy has been brought on due to a misunderstanding that South Korea and China were arguing about the standards of kimchi even though kimchi and paocai are two completely different foods. While the controversy grew over the last nine days, China had not made any statement and later announced that is was a translation mistake.

# **N. Korea smuggled over $400 million worth of coal to China**

North Korea is believed to have exported up to 401 million dollars worth of coal to China between January and September of this year despite UN sanctions banning North Korean coal exports. Experts point out that there are holes in the sanctions as North Korean vessels openly operated flying the North Korean flag.

The Wall Street Journal (WSJ) on Monday cited satellite images provided by the U.S. State Department showing that North Korean vessels have shipped hundreds of coal shipments to China’s Ningbo-Zhoushan area for the past year. More specifically, a satellite image taken on Aug. 12 showed four North Korea-flagged vessels anchoring off the Ningbo-Zhoushan area with Chinese ships, suggesting an illegal ship-to-ship transfer of coal.

Washington believes Pyongyang has smuggled 4.1 million tons of coals by Sep. this year. Assuming that the coals were sold for 80-100 dollars per ton, the total amount of export is between 330 million dollars and 410 million dollars. The international community including the U.S. is jointly monitoring North Korea’s illegal ship-to-ship transfer but the WSJ reported that North Korean vessels were sailing under the North Korean flag.

The U.S. accused China of ignoring the UN sanctions against North Korea. A senior U.S. State Department official said that a direct shipment of coal from North Korea to China is the first major change we have seen since the adoption of UN sanctions against North Korea in 2017, adding that China and North Korea are no longer trying to hide their smuggling activity.

# **US Congress agrees on bill for counter-China initiative**

U.S. Congress has authorized the new National Defense Authorization Act (NDAA) for fiscal year 2021, agreeing to set aside 2.2 billion dollars for the new Pacific Deterrence Initiative. By establishing a counter-China fund, the Congress signaled that the U.S. will maintain its tough stance on China even after Joe Biden takes office in January 2021.

According to The Washington Post on Dec. 6 (local time), a new program called Pacific Deterrence Initiative has been added to the new NDAA released by Senate and House Armed Services Committees as part of the efforts to strengthen the U.S. military capability in the Indo-Pacific region. Under the bill, the Secretary of Defense, in consultation with the Commander of United States Indo-Pacific Command, shall summit to the congressional defense committees a report that contains a detailed summary of the activities and resources of the Indo-Pacific Deterrence Initiative not later than Feb. 15, 2021. The bill stipulates that the purpose of the initiative is to enhance the United States deterrence and defense posture in the Indo-Pacific region and to carry out activities necessary to assure allies and partners in the region.

Once the bill goes into effect, it is highly likely that the U.S. will decide not to shrink its military presence in South Korea in order to enhance its military deterrence in the Indo-Pacific region. But experts also say that the U.S. could increase its pressure on South Korea to join its anti-China front.

# **China gives approval to Korean gaming service in four years**

The Chinese government granted a business license to a South Korean gaming program for the first time in four years. Some expect that it signals the lessening of China’s regulations on South Korean gaming software while others dismiss it as a one-time exception.

South Korean gaming developer Com2uS officially announced on Thursday that its mobile game Summoners War: Sky Arena received a license from China’s State Administration of Press and Publications the day before. Since its global launch in June 2014, Summoners War: Sky Arena has become Com2uS’s flagship product, contributing to more than 80 percent of the developer’s overseas sales.

Later 2016, Com2uS applied for a business license with the aim of helping Summoners War: Sky Arena penetrate the Chinese market. However, the dispute between Seoul and Beijing over the deployment of Terminal High Altitude Area Defenseless (THAAD) only worsened their diplomatic relationship in 2017 while the Chinese government unofficially put restrictions on the distribution of South Korean pop cultural content including gaming services. Early 2017 was the last time when it issues a business license to a South Korean game. “It was a long time ago that we applied for a license so we did not expect it to happen. We were not notified of the news before,” according to an executive at Com2uS.

China’s surprise issuance of the business license is increasing expectations across the South Korean gaming industry that one of the world’s largest gaming markets may reopen its door for South Korean gaming businesses. Optimists anticipate that Beijing will soon allow Nexon’s flagship title Dungeon & Fighter, which is soon to attempt to make inroads into China, and other games based on intellectual property well-received by Chinese users to have access to its domestic market. Such heightened market expectations translated into rising stock prices of South Korean gaming developers on Thursday.

Meanwhile, others concern that it is too early to conclude that the Chinese market is now accessible, saying that extra license issuances should follow in order for South Korean gaming developers to be assured of China completely removing restrictions on them.

# **BBC calls reports of kimchi by Chinese media false**

Chinese media reported that China set an international standard for kimchi after the country received certification for pao cai, a type of Chinese fermented vegetables, from the International Organisation for Standardisation (ISO), and BBC described the report as “false.”

In an article entitled, “Kimchi ferments cultural feud between South Korea and China,” BBC said, “South Korea has rebuffed China after false reports that it had won global certification for its production of kimchi - a hallowed dish for Koreans.” It also said, “It's the latest cultural spat between the neighbours.”

Chinese state media Global Times reported on Sunday that the ISO status was "an international standard for the kimchi industry led by China,” adding that, contrary to popular belief, “pao cai” has not originated from South Korea. It described kimchi as the same dish as pao cai and claimed the award covered kimchi.

The South Korean Ministry of Agriculture, Food and Rural Affairs refuted the claim, saying that international standards for kimchi were agreed by the United Nations in 2001 in line with CODEX Alimentarius. It also said kimchi and pao cai are two different dishes.

BBC quoted the statement and introduced kimchi and kimjang, the communal act of making kimchi. “Kimchi is often served in China under the name pao cai, but China has its own variant of the dish which it also calls pao cai,” it said. “Although the ISO listing clearly says "this document does not apply to kimchi", some Chinese media suggested otherwise.”

# **Risks over Korea-China-Japan FTA**

Chinese Foreign Minister Wang Yi, who recently visited Seoul and Tokyo, has proposed a push for a tripartite free trade agreement among the three neighboring countries of Asia. In a meeting with Korean Foreign Minister Kang Kyung-wha, Minister Wang Yi strongly proposed to establish a Korea-China-Japan FTA, and the same message was stressed during the meeting with his Japanese counterpart. “The three-party FTA will prove to be effective in complementing the lack of cooperative institutions in the North East Asian region,” argued the Chinese daily Global Times. Given the economic and diplomatic risks it entails, however, a tripartite trade deal must be approached with caution.

The first negotiation for a tri-party trade deal among Beijing, Seoul, and Tokyo, began in 2013, but there has been little progress owing to the differences of opinions in core agendas and their concomitant diplomatic disputes. Once the three Asian economic powerhouses, which take up 24% of global GDP, remove the barriers for their products and services, it will certainly galvanize economic growth, but it will also invite as much risk. As big manufacturers in the world, the three Asian neighbors share many overlapping sectors of export such as semi-conductors, cars, and steel. The FTA with China took effect in 2015, and for Seoul, if the market is pried open further, it might deal a heavy blow to its vital industries. In fact, this is why the tri-party FTA has failed to make meaningful progress despite the series of talks and negotiations.

The political and diplomatic implications should be taken into account as well. Behind Beijing’s proposal for regional cooperation and trade deals amid Washington’s power transition is China’s intention to keep in check America’s influence in North East Asia. History attests to the fact that the three neighbors’ attempt to seal a free trade deal has been gravely swayed by political and diplomatic factors such as Beijing’s Seoul-bashing over the installation of the THAAD system and the historical disputes between Korea and Japan.

In response, U.S. President-elect Joe Biden is proposing to hold a “Summit for Democracy.” During his campaign, Biden said the gathering will “bring together the world’s democracies to strengthen our democratic institutions, honestly confront nations that are backsliding,” and certainly there are signs that he is willing to put those words into action. Korea’s “strategic ambiguity” is being tested yet again between the growing pressure from America and China. Sandwiched between the two superpowers, Seoul must make a wise strategic choice for its own national interests.

# **SK Hynix Chinese plant suspends operation due to COVID-19 case**

A South Korean worker who was dispatched to SK Hynix’s plant in Chongqing, China tested positive for COVID-19, which resulted in the suspension of the Chinese plant’s operation. The Chongqing city government ordered COVID-19 testing for all 2,700 employees working at the plant.

According to China’s state-run Xinhua News Agency and sources in the South Korean business community on Sunday, an SK Hynix employee tested positive when he took COVID-19 test upon arrival on Sunday at Incheon International Airport after completing his 18-month secondment to China. Right after the man was confirmed as a COVID-19 patient, the city of Chongqing conducted epidemiological survey at, restricted access to, and decontaminated the SK Hynix plant where the Korean national worked.

“The Chongqing city government and SK Hynix suspect that the employee is an asymptomatic case without displaying notable symptoms,” a South Korea business community source said. “The City of Chongqing also suspended the operation of the hotel where the SK employee stayed, and started PCR testing in people who stayed at the hotel since November 25.

SK Hynix’s Chongqing plant is a facility for late-state processing including semiconductor packaging. Generally, if a semiconductor factory stops operation even momentarily due to power outage, it takes days before recovering production lines, and can cause massive damage. The chip packaging plant where operation has been suspended this time will likely resume operation as soon as the city authority completes quarantine measures. However, it is unknown at this point in time when the plant will actually resume operation, and the company will inevitably face disruptions in production if the plant inevitably remains suspended for an extended period of time.

“We will fully cooperate with the Chinese government, and do our best to resume operation as soon as possible,” SK Hynix said Sunday.

# **Events commemorating Bruce Lee held in Greater China**

Events remembering Bruce Lee (1940-1973), a legendary action movie star of Hong Kong, have been held in Greater China to mark the 80th year of his birth.

According to the report of Global Times, a state-owned English newspaper in China, published on Saturday, Foshan, Guangdong Province, has been holding an event marking the 80th year of his birth (November 1940) from early this month. A look-alike contest, online/offline events to look back on his life and other events are held. The Foshan government intends to make the city the center of the world’s martial arts culture. 　

Hong Kong’s post office also issued special stamps under the theme of “Bruce Lee’s heritage in global martial arts.” Trams running through Hong Kong’s central region also put up an advertisement commemorating the 80th year of his birth. They will operate until January. 　

Internet users shared video clips and photos of Bruce Lee commemorating his birth on Chinese social networks including Weibo. “Bruce Lee was more than just a kung fu star to many,” said Global Times. “He changed the stereotypes about Chinese people in western countries and is still remembered by a lot of fans.” 　

Bruce Lee was born in San Francisco, the U.S., and moved to Hong Kong when he was three months old. He appeared in 23 movies and became an icon of popular culture among men all over the world in the 1970s and 1980s. The global fandom grew even bigger when he died at the tender age of 33.

# **China stays arrogant to maintain restrictions on Korean wave content**

Chinese Foreign Minister Wang Yi returned to his home country on Friday after spending three days in South Korea. Despite a short stay, he met with many important figures in South Korea, including its president, the speaker of the National Assembly, and the leading members of the ruling party. He was late for a meeting with the South Korean foreign minister by 25 minutes. While broad plans for bilateral cooperation were discussed between the two countries during his visit, there was no progress made in key issues, including China’s restrictions on the Korean wave content.

The purpose of Wang’s visit to South Korea seems to have been to gauge a neighboring country’s current atmosphere and for diplomatic management in the face of the U.S.’ change of administration – nothing more, nothing less. While the Chinese side listed 10 items as agreements between the two countries, the list did not include any key agenda. Wang also said Chinese President Xi Jinping’s possible visit to South Korea would require the complete containment of the COVID-19 virus. He put pressure on the Terminal High Altitude Area Defense (THAAD) issue by demanding its withdrawal while only responded to South Korea’s request to lift the restrictions on the Korean wave content, which was a retaliatory measure against the deployment of THAAD, by saying that he hopes for continuous communication.

China has been suffering all-around attacks by the Trump administration. Such tensions between the U.S. and China are likely to continue under the Biden administration, according to many experts. China is putting a lot of effort into diplomacy with South Korea and Japan, which are the allies of the U.S., so that the neighboring countries won't side with the anti-China stance. Even during this visit, Wang emphasized economic integration among South Korea, China, and Japan and requested South Korea to join the Global Initiative on Data Security, which was set up to fight the U.S.' bans on Chinese information technologies and businesses.

The U.S.-China relations under Biden’s lead is hard to predict. Except for President Trump, all U.S. presidents who harshly criticized China during the election period focused on trade expansion with China once they took the office. The Biden-style internationalism based on rules and values will exercise its influence on China with meticulous systematic plans, rather than the Trump-style trade war. A formation of a value-based initiative, such as a ‘democracy summit,’ may put South Korea at the crossroads of unavoidable choices.

“America is not the only nation in the world,” Wang said, which means pressure on South Korea not to side with the U.S. However, China is not the only country in East Asia, either. The country will lose its ground if it continues its shameful diplomatic tactics by controlling other countries’ access to the large Chinese market. China should reflect on why some say that Chinese officials visiting South Korea act like an envoy of an emperor.

### Chinese Foreign Minister visits South Korea

“America is not the only nation in the world. There are 190 countries and each of them is a sovereign nation. They include China and South Korea,” Chinese State Councilor and Foreign Minister Wang Yi said on Thursday during his visit to South Korea. His comment seems to stress that cooperation between South Korea and China should not be affected by the U.S.

It was his answer to a question during a press conference after a meeting with South Korean Foreign Minister Kang Kyung-wha at the office of the foreign minister on Thursday morning asking whether his visit is a way to press the South Korean government and the ruling party members not to side with the U.S. when it comes to the competition between the U.S. and China. “China and South Korea are close neighbors and should visit each other more often, like relatives,” he added. He also said that the two countries are strategic cooperative partners and should engage in comprehensive coordination and cooperation. During his statement at a meeting with Kang, he said he is willing to have strategic discussions regarding the international and regional issues. A source from the foreign ministry said that Wang expresses his concerns about the Terminal High Altitude Area Defense (THAAD) system deployed to South Korea.

When asked about a possible visit to South Korea by Chinese President Xi Jinping, Wang pointed to the reporter, commenting on the face masks worn by them. “What’s important is to fully contain the COVID-19 virus,” he said, implying that the president’s visit is unlikely due to the resurgence of COVID-19 cases in South Korea.

# **Ultra-fine dust from China blankets S. Korea again**

South Korea, which saw a temporary reduction in fine dust levels amid the COVID-19 pandemic, is suffering from high levels of fine dust again. Ultrafine dust warnings were issued for the first time this fall in Seoul on Sunday and the levels of ultrafine dust remained “bad” for four days in a row from Nov. 12 in the Seoul metropolitan and Chungcheong areas. Emergency fine dust reduction measures were issued in South Chungcheong Province on Nov. 14 and 16 and almost all parts of the country except for Gwangju and Busan had “bad” fine dust levels on Monday.

Up to 80 percent of South Korea’s fine dust particles come from China. Environment authorities say a surge in fine dust concentrations in recent years is attributable to the atmospheric stagnation, which causes the accumulation of airborne pollutants brought in from China on winds from the west. China’s factory utilization rate, which dropped amid the COVID-19 crisis, has recovered almost to 100 percent and the total amount of pollutants coming from China has increased again as people have started to turn on their heating. China’s air quality is said to have improved somewhat this year with the fine dust levels across the country reducing by an average of 11.8 percent until September. As it turns out, however, it was only a temporary illusion created by COVID-19 and the lives and health of Koreans are still damaged by the fine-dust disaster.

When the issue of fine dust coming from China became an urgent national task after the national suffered from the worst fine dust concentrations in spring of last year, the Moon Jae-in administration seemed to be coming up with some fundamental measures. But little progress has been made since then. South Korea and China are having environment ministers’ meeting and working-level talks once or twice a year but the outcomes were no more than information exchange or academic research.

To be sure, there will be no ways to fundamentally block fine dust from China unless China’s industrial and environmental policies change dramatically. However, if China significantly strengthens the pollutant emissions standards for the plants on China’s east coast and makes bold investments on reducing the total amount of emissions, the amount of fine dust currently hitting Korea will be reduced greatly. Since the fine dust crisis is a matter of survival for Koreans, the South Korean government should abandon its submissive attitude and strongly urge China to come up with fundamental solution to reduce fine dust pollution.

# **Pres. Moon signs China-led economic partnership**

President Moon Jae-in officially signed the Regional Comprehensive Economic Partnership (RCEP), the world’s largest multilateral trade agreement on Sunday.

President Moon signed the partnership at the fourth RCEP summit, which took place virtually, at the presidential office on the day. “The world’s largest free trade agreement has been signed with young dynamic ASEAN as its center in the face of the challenges of Covid-19, spread of protectionism, and a crisis in multilateralism,” Moon said when signing the agreement. “We have translated into action the protection of the value of free trade.”

The world’s largest free trade agreement comprises 15 countries, including South Korea, China and Japan, plus 10 ASEAN countries, Australia and New Zealand. Their combined trade volume, GCP, and population account for a third of the world's total. The purpose of the pact is to bolster trade by lowering tariffs among its members and establishing a systematic trade and investment system.

Some critics say that with South Korea’s joining the economic partnership that is effectively spearheaded by China, Seoul will face more complex diplomatic affairs amidst tension between Washington and Beijing. When former U.S. President Barack Obama pushed to form the Trans-Pacific Pact from 2010 in a bid to block China’s expansion, Beijing started process to form the RCEP in 2012 to dodge obstacles to trade, and would urge Seoul to join.

TPP ended up collapsing as U.S. President Donald Trump, who denied multilateralism, declared withdrawal from the trade agreement in 2017. However the pact was replaced with the name “Comprehensive and Progressive Agreement for Trans-Pacific Partnership (CPTPP)” spearheaded by Japan and Australia in 2018. U.S. President-elect Joe Biden has been indicating that the U.S. will return to the CPTPP ever since becoming Democratic presidential candidate.

“Like other countries China is one of the 15 countries that have joined the RCEP,” said an official at the presidential office. “If deemed necessary, South Korea could also participate in the CPTPP.”

# **Chinese netizens, media attack Black Pink members for touching panda**

Chinese netizens and state-run media would attack South Korean boy band BTS by promoting blank nationalism. Now, they have launched attacks on South Korean girl group Black Pink en masse. They claimed that Blank Pink, which released on YouTube and other channels a video showing the music band’s members coming into contact with the rare animal giant panda on Tuesday, did not treat the animals cautiously and valuably enough to satisfy them.

China’s state-run English daily Global Times said Friday, “Blank Pink members wore deep makeup when they touched three-month-old baby panda ‘Fubao “at the Everland theme park in South Korea, and ‘Huani’ which came to Korea in 2016. They failed to wear gloves and facemask as well.” The daily went on to say, “Baby pandas lack immunity and they could be in danger if people (coming into contact) wear strong makeup or fail to use protective equipment. The panda is China’s ‘national treasure’ and even if one is born overseas, it should return to China after a certain period of time, and they belong to China.”

Some Chinese netizens posted Weibo comments, reading “Let’s bring back the pandas from Korea.” All giant pandas in zoos around the world are rented, rather than sold or granted, by the Chinese government.

When uploading a teaser of Black Pink’s video on its Instagram page, Everland said, “Filming was done after thorough decontamination and quarantine under supervision by the responsible veterinarian and zookeepers.” But the theme park removed the clip after Chinese netizens responded negatively.

# **Hyundai to supply 3,000 hydrogen trucks to China**

Hyundai Motor Company will supply hydrogen-electric trucks to China, accelerating its hydrogen economy business expansion in the country.

The South Korean automaker announced on Wednesday that it signed memorandums of understanding (MoUs) to build a hydrogen economy ecosystem with businesses in the Jing-Jin-Ji metropolitan region where capital Beijing is located and the Yangtze Delta where Shanghai is located.

Hyundai Motor Company first signed MoUs with major energy and financial companies in the Yangtze Delta on October 27. The goal is to supply over 3,000 hydrogen-electric trucks of Hyundai Motor Company to the region by building hydrogen charging stations and hydrogen production facilities and launching financial services to support hydrogen-electric cars.

Later on Wednesday, the automaker signed MoUs with Antai Science and Technology and Hebei Steel Industrial Technology Service to build hydrogen charging stations, pilot hydrogen-electric trucks, and supply over 1,000 hydrogen-electric trucks by 2025 in the Jing-Jin-Ji metropolitan region.

Hyundai Motor Company supplied hydrogen trucks by lending them for fees to reduce the cost burden of logistics companies for initial purchases.

# **Doosan Infracore reaches 200,000 mark for excavator production in China**

Doosan Infracore announced on Tuesday that the company’s number of excavators produced in China surpassed 200,000 units. It is a record reached in 26 years since the company first entered the Chinese market in October 1994.

Doosan Infracore whose plants are located in Yantai in China reached the 5,000 mark for the accumulated production of excavators in 2001. The Chinese market was dominated by Japanese construction machinery producers at the time, but the South Korean company grew to secure the largest market share as a foreign company in the country’s construction machinery sector since the 2000s by engaging in aggressive marketing tactics and launching new products. The company’s annual production of excavators surpassed 10,000 units in the mid-2000s and 20,000 units in 2010.

The accumulated production record of 200,000 units is the first among foreign construction machinery producers in China. Doosan Infracore has 22.8 percent of market share in the Chinese excavator market, excluding domestic companies, as of the third quarter this year. The South Korean company is competing against American company Caterpillar Inc. for the No. 1 spot in the sector.

Doosan Infracore sold over 15,000 units of construction machinery last year in China. Despite the market slowdown due to COVID-19, the company has sold 14,348 units for the first three quarters of this year, which is comparable to its sales last year. Doosan Infracore will continue to launch new products and specialty equipment in line with the growth trend of the Chinese excavator market.

# **BTS faces backlash from Chinese fans over Van Fleet Award speech**

World-famous K-pop band BTS has come under fire in China for not acknowledging China alongside South Korea and the United States in its acceptance speech.

BTS was given the “Van Fleet Award” by The Korea Society. The U.S. non-profit organization has given to individuals and groups that promoted the U.S.-South Korean relations since 1995 to honor James Van Fleet, Commander of the U.S. Eighth Army who fought in the Korean War. It has been awarded to former South Korean President Kim Dae-jung, Samsung Group Chairman Lee Kun-hee and the Korea Chamber of Commerce and Industry.

“This year marks the 70th anniversary of the Korean War,” said RM, the leader of the South Korean group, accepting the award. “We will always remember the history of pain that our two nations shared together, and the sacrifices of countless men and women.” His remarks have angered Chinese media and Internet users.

The Global Times reported on Monday that Chinese online users were enraged by the mention of “the history of pain that our two nations shared together.” Some commented that the Korean boy band dismissed the noble sacrifices made by Chinese soldiers during the Korean War and that the acceptance speech ignored the American invasion and Asia’s interference on popular news portal websites.

The Korean War is called the “war against the United States to assist North Korea” in China. Amid escalating tensions between the United States and China, Beijing is emphasizing the “sprit of helping North Korea against the United States,” which implies nationalism, patriotism and heroism to commemorate the 70th anniversary of the war. This possibly explains the angry reaction of Chinese fans to BTS’s speech.

# **Trump: U.S. will end its reliance on China once and for all**

Trade conflict between the U.S. and China has been reigniting in recent weeks. U.S. President Donald Trump lashed out at China for spending the money it earned from its trade with the U.S. on military expansion. “We will end our dependence on China,” stressed Trump. In response, Chinese State Councilor and Foreign Minister Wang Yi immediately criticized the U.S. for putting Chinese tech companies under pressure and the Chinese state media warned of retaliation, mentioning a possibility of selling U.S. government bonds.

At a press conference held on Monday (local time) marking the Labor Day, President Trump said, “There’s been no country anywhere at any time that’s ripped us off like China has,” adding that China spends that money on building their military. “It’s very lucky that I’ve been building ours up because otherwise we’d be dwarfed right now by China,” said Trump. “We’ll end our reliance on China, once and for all, whether it’s decoupling or putting in massive tariffs like I’ve been doing already.”

The Trump administration has recently cracked down on Chinese tech companies, such as Huawei, TikTok, and WeChat, officially designating them as national threats. In particular, Washington is considering placing SMIC, China’s No. 1 foundry company, on its export restriction list. The U.S. is launching a series of attacks also in the finance sector, announcing plans to delist Chinese companies that fail to comply with U.S. accounting standards.

For its part, Beijing is bracing for Washington’s threat to cut off economic ties with China. In a column, “Beijing ponders measures to offset U.S. decoupling attempt” in The Global Times on Sunday, the writer said China will turn its back on all unfriendly economies and will seek to form closer economic partnerships along the Belt and Road Initiative, particularly with the economics of the European Union, Asia, and Africa. The column added that Beijing should “take its own destiny in its own hands” as the Trump administration has embarked on its bid to decouple itself from China.

# **China retaliates U.S. by closing its Consulate in Chengdu**

The U.S. has announced that it will completely revise its engagement policy toward China, harshly criticizing Chinese President Xi Jinping as a “true believer in bankrupt, totalitarian ideology.” The Chinese government instructed the closure of the U.S. Consulate General in Chengdu, Sichuan in retaliation of the U.S.’ closure of the Chinese Consulate General in Houston.

It is deemed that the two countries’ relations are on the brink of severing for the first time since the two established diplomatic ties 41 years ago as the bilateral tensions that have arisen over the U.S.-China trade disputes, blaming each other for the responsibility of COVID-19, and China’s enactment of Hong Kong national security law are now unfolding as a full-on diplomatic war.

U.S. Secretary of State Mike Pompeo delivered a speech titled “Communist China and the Free World’s Future” at the Richard Nixon Library in Yorba Linda, CA on Thursday (local time) “President Nixon once said he feared he had created a ‘Frankenstein’ by opening the world to the CCP, and here we are,” Pompeo said. This implies that the U.S.’s engagement policy toward China, which has continued for about 50 years since President Nixon’s visit to China in 1972, has contributed to the growth of the country as the biggest adversary threatening the Western world in modern times.

“We have to keep in mind that the CCP regime is a Marxist-Leninist regime,” Pompeo continued. “We, the freedom-loving nations of the world, must induce China to change,” he added, encouraging the U.S.’s allies to join in putting pressure on China. Regarding the decision to shutter the Chinese Consulate General in Houston, he claimed that the consulate was the hub of China’s spying and stealing of intellectual property rights.

# **Chinese ships catch $440 million worth of squid in N. Korean waters**

The analysis of satellite images has been revealed to show that Chinese “dark fleets” have illegally caught 440 million U.S. dollars worth of squid in North Korea’s East Sea, which is under the United Nations (U.N.) sanctions, for about two years. Dark fleets refer to illegal and unlicensed ships that do not send their locations nor appear on a public monitoring system. The findings have been achieved from international research led by South Korean data scientists and international non-governmental organizations. The research has also confirmed that small-scale fishermen were pushed further out into the ocean due to the illegal fishing vessels.

Non-profit private research organization Global Fishing Watch, Korea Maritime Institute, Japan Fisheries Research and Education Agency (FRA), and the University of California have published the precision analysis results of satellite images in the journal Science Advances on Wednesday to demonstrate that Chinese vessels have conducted the world’s largest illegal fishing from 2017 to 2018.

A research team, including senior data scientist Park Jae-yoon at Global Fishing Watch, has focused their monitoring efforts on squid fishing ships that entered the exclusive economic zone (EEZ) of North Korea in 2017 and 2018. Many of them are suspected to be dark fleets operating on the coast of China that have entered the East Sea through the South Sea. However, there is no measure to consistently track and monitor them for now. “Dark fleets are very active in the East Sea within North Korean waters, however, their illegal fishing is not being properly monitored due to the lack of cooperation among neighboring countries," said Park. "We chose the East Sea as a place to reveal the comprehensive picture of dark fleets' illegal fishing based on artificial intelligence (AI) and satellite data."

The research team has developed technologies that enable the tracking of illegal fishing vessels under any conditions by combining four different satellite observation technologies. They first utilized a satellite constellation owned by Planet Labs, an American satellite video service provider, to identify pair trawling boats.

The joint research team has identified over 1,600 Chinese illegal fishing vessels by analyzing satellite images collected for two years. The amount of squid caught by them is about 164,000 tons, which is estimated to be worth approximately 440 million dollars. This is almost equivalent to the sum of both South Korea’s and Japan’s squid catch, which officially have the largest squid catch records.

# **U.S.-Sino tensions rise following closure of Chinese Consulate in Houston**

Over the latest closure of the Chinese Consulate in Houston, the U.S., President Donald Trump said it is always possible to close more Chinese missions. Despite the strong opposition from Beijing and worries from the international community, the U.S. president has fueled such concerns, hinting at the possibility of further cranking up the intensity of crackdowns against China.

“We thought there was a fire in the one that we did close and everybody said ‘There’s a fire! There’s a fire!’ But I guess they were burning documents or burning papers and I wonder what that’s all about,” President Trump said during a White House briefing, insinuating that there may have been attempts to destroy evidence of China’s illegal activities at the Consulate building.

During a Senate Foreign Relations Committee on America’s China policy on Thursday, Deputy Secretary of State Stephen Biegun said the closure was in compliance with Trump’s instructions. Biegun criticized Beijing, citing a series of disputes stemming from China’s theft of American technologies and infringing on its intellectual properties as background of the measure.

The Chinese embassy in America issued a statement, calling America’s allegation a groundless sophistry. During an interview on ABC, Cai Wei, Consul General of China in Houston, said he was greatly shocked by America’s decision, pouring a raw criticism against American politicians who he accused are “habitual liars.”

# **US-China tensions escalate**

China passed the Hong Kong security law, and, in response, the United States ended arms exports to Hong Kong and restricted the territory’s access to hi-tech products on Monday (local time) as it moves to strip away the special status of the territory. Amid rising tensions between Washington and Beijing, Hong Kong’s future is as uncertain as ever.

“Commerce Department regulations affording preferential treatment to Hong Kong over China, including the availability of export license exceptions, are suspended,” Reuters reported quoting U.S. Secretary of Commerce Wilbur Ross. “Further actions to eliminate differential treatment are also being eval‎uated.” This came one day before the 23rd anniversary of Hong Kong’s handover by the United Kingdom in 1997. “The United States will today end exports of U.S.-origin defense equipment and will take steps toward imposing the same restrictions on U.S. defense and dual-use technologies to Hong Kong as it does for China,” US Secretary of State Mike Pompeo said in his statement. “We can no longer distinguish between the export of controlled items to Hong Kong or to mainland China."

Concerns are rising that the U.S. removal of Hong Kong’s special status which includes lower trade tariffs would trigger “Hexit,” a compound of Hong Kong and exit that describes a situation where global capital and human resources scramble out of the city. Hong Kong’s Chief Executive Carrie Lam said she did not fear any restrictions from Washington.

Despite the pressure from the United States, China formally adopted the security law on the same day. According to South China Morning Post (SCMP), the legislation was passed unanimously at the National People’s Congress Standing Committee 15 minutes after it was put to vote. The new law will be added as a supplementary provision to the Hong Kong Basic Law, which is the territory’s de facto constitution, and is expected to come into effect on July 1.

Under the new security law, anyone who commits acts of secession, subversion terrorism or collusion with foreigners can be sentenced to a life sentence.

# **Why COVID-19 further strains the U.S.-China relations?**

The COVID-19 pandemic is a very rare biological and security disaster facing mankind. It is a common threat both to the U.S. and China, and the world will be able to rise above the COVID-19 crisis when the two powers join hands. The COVID-19 pandemic, however, is further straining the U.S.-China relations and the two countries appear to be only one step away from entering a new Cold War.

The COVID-19 pandemic has aggravated the U.S.-China relations. There has been a great setback in the history of the U.S. government policy statements on China since President Donald Trump took office. The Trump administration has kept “demonizing China.” This demonization represents a “victim complex” of the Trump administration and far-right Republicans that Chinese find hard to understand. This complex has been the cornerstone of the Trump administration’s policy toward China for the past three years. Rather than using the COVID-19 pandemic as a chance to collaborate with China, the Trump administration strongly pursued its “America First” policy, expressing resentment against China. It has become a major means to put China under pressure.

As many as 70 percent of Americans today think China should be held responsible for the spread of COVID-19. The pandemic has fueled the anti-Asian American racism and discrimination. The views of many political elites and Americans on the U.S. policy towards China are being influenced by the COVID-19 pandemic and returning to the “New McCarthyism” in the 1950s. Rational and gentle opinions on Trump’s China policy are often being pushed aside.

It is high time that both the U.S. and China reflect on themselves and come up with sincere and rational policies and strategies. The rise and fall of superpowers inevitably bring about fierce power competition and strategic confrontation. But the future of “New Cold War” between the U.S. and China in the 21st century will only undermine the stability, peace, and prosperity of the world. Moreover, it will bring about a disastrous shock to the economic development of Northeast Asia. Former U.S. presidents including Jimmy Carter, Bill Clinton, George W. Bush, and Barack Obama recently lashed out on Trump’s policies at home and abroad, calling on President Trump to look back on the cause of his “catastrophic failure” of policies. For its part, the Chinese government first needs to promote political reforms and improve national image among other problems it has presented in dealing with the COVID-19 pandemic. Only then will the U.S. and China continue to be understood and respected by many countries in the world.

# **China’s foreign ministry urges North Korea to remain calm**

Foreign news outlets reported that North Korea destroyed an inter-Korean liaison office in Kaesong on Tuesday, saying that the tensions on the Korean Peninsula is elevating. China’s Foreign Ministry urged the North to control itself, saying, “We want peace and stability of the Korean Peninsula.”

The Associated Press, CNN, The New York Times and Asahi Shimbun quoted the announcement of the South Korean Ministry of Unification to deliver that North Korea blew up a liaison office. Kim Yo Jong, first vice department director of the Central Committee of the Workers' Party of Korea, said in a statement, criticizing the South Korean government was not able to stop leaflet drops of North Korean defector groups, the media outlets added.

The Associated Press analyzed that North Korea was expressing its frustration on being unable to resume economic cooperation with the South due to U.S.-led sanctions against North Korea. The New York Times reported that North Korea displayed its anger against the South in a dramatic way after threatening to end the reconciliatory mood between the two Koreas. The Washington Post said Pyongyang’s rhetoric against Seoul has become harsher for the past few weeks and projected that the demolition would drastically increase conflicts. Asahi Shimbun reported that the liaison office symbolized achievements of South Korean President Moon Jae-in’s North Korea policies and thus the destruction was bound to be a heavy blow.

South and North Korea are part of the same nation, and that as a close neighbor, China has always wished for peace and stability on the peninsula, Chinese Foreign Ministry spokesperson Zhao Lijian said in a press briefing. Japanese Chief Cabinet Secretary Yoshihide Suga said in a press conference on Tuesday that his country was continuously cooperating with countries such as the U.S. and South Korea over matters regarding the North, collecting and analyzing necessary information and closely monitoring the situation.

# **Imported salmon blamed for second coronavirus wave in Beijing**

The resurgence of COVID-19 from the Xinfadi wholesale market of Beijing is spreading across the city and beyond. The Chinese government is proposing the possibility that the salmon imported from Europe may have been responsible for the reentry of the virus.

According to the National Health Commission, a total of 36 cases were confirmed in Beijing alone on Sunday. During the four days since the outbreak of the first case from Xinfadi on Thursday, a total of 79 patients have been diagnosed with COVID-19 in Beijing, and the eight out of the 16 districts within the city were found to have produced patients.

With a massive testing scheduled on 76,499 citizens of Beijing, it is expected that the number of newly-affected patients will surge apace. “Beijing is exposed to a high risk of COVID-19 contagion,” said Sun Chunlan, the vice premier of the People's Republic of China, on Sunday.

The public sentiment on social media cut both ways, with some criticizing the government for blaming imported salmon and others arguing to have witnessed unmasked employees handling fish and meat at the market. Conspiracies have been voiced again, claiming that the virus spreading across Wuhan had also come from outside.

# **U.S. promises to protect S. Korea against any Chinese retaliatory action**

U.S. Under Secretary of State for Economic Growth, Energy, and the Environment Keith Krach said on Thursday (local time) that Washington is ready to do its utmost to keep Seoul intact in the face of any retaliatory action that Beijing may take if Seoul joins the U.S.-led Economic Prosperity Network (EPN) or sanctions against Huawei. He called upon U.S. allies and partners to agree to anti-China policy and build unity, emphasizing the urgency for the international community to come forward to fight against the threats and retaliations by the Chinese government.

With major media networks from five countries including India and Brazil in presence, a telephone press conference was held on Thursday where Under Secretary Krach elaborated Washington’s economic sanctions and policy framework regarding issues with China. The Dong-A Ilbo was the only South Korean media outlet that was invited to the telephone press conference.
As for the request by Washington for its allies to join U.S. policy toward China, the under secretary said that it is not a matter of choosing either Washington or Beijing, adding that everyone is entitled to a choice and at the heart of the issue is trust.

Under Secretary Krach stressed the importance of economic cooperation between the United States and South Korea, saying that South Korea is one of the world’s biggest economic and technological powerhouses as well as one of the major trade partners not only to the United States but also to the rest of the world. Meanwhile, he highly commended Samsung Electronics for being one of the world’s top three 5G players and boasting off a high level of semiconductor production technology.

# **U.S. wants S. Korea to stand by its side against China**

U.S. Under Secretary of State for Economic Growth, Energy, and the Environment Keith Krach on Thursday asked South Korea to join the Economic Prosperity Network (EPN), a U.S.-led economic bloc initiative against China. In his words, the United States would do whatever it takes to help South Korea handle any retaliatory action that China may take. Krach made it clear that it is not a matter of choice between the United States and China, emphasizing that everyone has their own choice but it all comes down to trust.

The gist of Under Secretary Krach’s request is that Seoul should be supportive of the EPN initiative and a series of anti-Huawei campaigns that Washington has pushed for. The EPN initiative aims to build a new U.S.-centric global supply chain with China out of the picture. The U.S. government has promoted an economic rival of China's One Belt One Road (OBOR), the pivot of growing Chinese influence across the world, whereas the Indo-Pacific Strategy has served as Washington's anti-China national security scheme. Against the backdrop, South Korea is, in effect, being pressed into taking the U.S. side as one of its allies.

The United States reiterates how significant it is for liberal capitalist countries to unite to uphold democracy, human rights, transparency and intellectual property rights against China's nationalist capitalism. However, the point is that the EPN initiative still seems to be nothing more than unripe fruit. Not much of details has been specified as to in what way its binding force takes effect or in what field cooperation works, except the overarching principle of keeping China out. This only makes it all the clearer that the U.S. strategically schemes to ostracize China, a.k.a. the world's factory, from the global supply chain.

Washington promises to participating countries, which are concerned about any retaliatory action that Beijing may take, that it will do whatever it takes to help them. Theoretically, they may be able to wield strong power against China's backlash and ensure sufficient compensation if the U.S. has their back. However, involvement in the EPN initiative is likely to come with the direct damage caused by China's retaliatory measures and demand industry-wise structural change. What is hurtful to South Korea, in retrospect, is the attitude that the United States showed toward China’s retaliation against its ally regarding the Terminal High Altitude Area Defense (THAAD) issue.

Obviously, South Korea as an U.S. ally has every reason to take part in an international coalition to safeguard freedom and democracy as Washington puts it. With the EPN in place, it may increase chances of diversifying export and production routes that are heavily dependent on China. However, it is a far-fetched plot for South Korea to turn its back on its No.1 trade partner and the world’s largest consumer market. Also, Washington will less likely push it to that level. It is time to collect opinions across the South Korean government and industries and make thorough preparations while pondering upon the rationality of joining the EPN initiative, depth of participation and global trends.

# **S. Korea closes gap in shipbuilding order share with China**

The gap between South Korea and China in terms of their shares of shipbuilding orders closed significantly as current leader China’s share slowed down. China’s manipulative efforts to increase order volume by pouring in its own domestic volume has finally reached its limit.

According to Clarkson Research, a shipbuilding and shipping market conditions analytic institution in the U.K., on Tuesday, the total amount of global shipbuilding orders placed last month was 570,000 CGT, which is 40 percent less than last month’s 1,410,000 CGT. By country, China, South Korea, and Japan won 270,000 CGT (13 ships; 47 percent), 230,000 CGT (eight ships; 40 percent), and 50,000 CGT (two ships; nine percent) last month, respectively.

This year’s overall performance is headed by China. However, the gap between South Korea and China is closing in terms of order shares. The accumulated orders received from January to May are 2.88 million CGT, 0.9 million CGT, and 0.49 million CGT each for China, South Korea, and Japan. The monthly shares of orders in April showed a 55 percentage point difference between South Korea and China but last month’s gap dropped to seven percentage points as China’s domestic order volume decreased significantly. For last month’s orders, South Korea has maintained a similar level as April but China’s declined 73 percent compared to the previous month. In particular, 85 percent of orders received by China last month were domestic while South Korea’s orders were all from businesses in Europe and Asia.

Industry experts believe that South Korea’s order volume will surpass China’s in the second half of this year. South Korea recently signed a contract with Qatar to reserve slots to build large LNG carriers and Russia and Mozambique are scheduled to launch large LNG shipbuilding projects, in which South Korea has strengths.

# **Worsened conflicts between U.S. and China over Hong Kong to undermine S. Korea’s export**

As the tensions between the U.S. and China regarding the enactment of the Hong Kong National Security law grow, their impact on the export of South Korea, which uses Hong Kong as the hub of intermediate trade, seems unavoidable.

Hong Kong is an important hub of South Korean businesses’ intermediate trade to reexport to China, the international trade and commerce research center of the Korea International Trade Association (KITA) reported on Friday. Hong Kong has easy access to mainland China and features various tax benefits, such as value-added tax refund, low corporate tax, and tax exemptions, as well as outstanding trade infrastructure, which is why logistics movement from South Korea to Hong Kong to China is very active at the moment. Last year alone, over 90 percent of exports from South Korea to Hong Kong eventually went to their destinations in mainland China. In addition, the U.S. has granted a special trade status to Hong Kong in terms of issuing visas, attracting investment, and enforcing laws since 1992, which has played an important role in the growth of Hong Kong as one of the major finance and logistics hubs in Asia.

However, once the U.S. strengthens sanctions against Hong Kong, the benefits mentioned above will disappear and a massive amount of foreign capital will leave the region as its advantages as a finance and logistics hub will be lost. “Under such a circumstance, direct export to China is the only available option, which will drive logistics costs and issues in securing direct flights to China for export,” the KITA predicted.

“Hong Kong is the fourth largest export destination of South Korea and offers values as an intermediate trade hub. If Hong Kong’s roles in finance, service, and logistics weaken, impact on South Korea’s export will be inevitable,” said a member of the KITA.

# **China lowers yuan amid conflicts with the U.S.**

China’s currency yuan broke through the psychologically important level of seven yuan against the dollar. The People’s Bank of China put the yuan fixing at 7.1209 per dollar on Monday, an increase by 0.38 percent from the previous day. The U.S. officially named China a currency manipulator in August when the yuan became cheaper than the seven-yuan-to-dollar threshold in the midst of the trade war between the two most powerful countries in the world.

China lowering its currency value could be interpreted as a measure against repeated attacks of the U.S. regarding the Hong Kong security law and the origin of COVID-19 as well as the trade war. Many have projected that China would lower the yuan to increase its competitiveness in exports if the U.S. puts pressure on the country.

The deval‎uation partly comes from China’s announcement to increase the money supply and expand the fiscal deficit for large scale pump-priming worth 1,000 trillion won. It would be excusable if the relations between the two countries are amicable, but the U.S. is likely to see this as China’s deliberate effort to expand exports as the conflicts between the two countries are elevated to extremes.

The U.S. reversed its decision to brand China as a currency manipulator as the two countries made the first trade agreement on January 15. If the U.S. re-designates China as a currency manipulator for this, the trade agreement, which was signed with difficulty, would not be worth the paper and the conflicts would elevate to a more sensitive issue of currency.

South Korea is one of the countries that could bear the brunt of the currency war between the U.S. and China. Unstable currency could spell troubles both to the government and businesses as it has high dependency on exports, especially to the U.S. and China. For the moment, it could boost exports as Korean won would be depreciated along with the yuan. But the financial market may falter if dollars evade the South Korean market.

But there is no other way to go around this. The South Korean government should monitor the foreign-exchange market meticulously and make every effort to intervene as long as it is globally acceptable. It should build robust seawalls to ward off external waves by securing global competitiveness and financial health to prepare for the difficult times.

# **S. Korean companies send around 550 employees to China**

Major South Korean companies, including Samsung Electronics, have sent over 550 employees to China on Thursday and Friday through the “South Korea-China quick path for businesspeople” system. Projects in China that have been suspended due to the COVID-19 outbreak, such as plant capacity expansion, are expected to be resumed quickly.

Samsung Electronics has dispatched over 300 technical employees of its headquarters and partners on a chartered plane for the capacity expansion of its semiconductor plant 2 in Xi'an, China. Over 30 employees from Samsung SDI, which s operating a battery plant in Xi'an, were also on board. Such a measure was implemented three days after Samsung Electronics Vice Chairman Lee Jae-yong visited the city on his first overseas business trip since the COVID-19 outbreak.

The quick path system for businesspeople grants a 14-day quarantine wavier for businesspeople who test negative for COVID-19 both before departure and after arrival. This is the first time the South Korean electronic giant used the system to send a large number of employees overseas. When the company dispatched around 200 employees to Xi'an in April before the introduction of the system, it requested “special entry” to bypass quarantine.

The Xi'an plant is the only overseas memory semiconductor manufacturing hub of Samsung Electronics with a total of 15 billion dollars of investment underway.

Kia Motors has also dispatched around 100 employees to its plant in Yancheng, China on a chartered plane while SK Innovation has sent about 120 employees on Thursday who will work in the construction site of the company’s Yancheng battery plant.

# **White House attacks China’s ‘predatory economic practices’**

The U.S. government led by President Donald Trump has released a report on the country’s future strategy and policy directions regarding China. The report is deemed as a practical announcement of a “new cold war” as it makes it clear that a “competitive approach” will be adopted for China, such as exercising public pressure and practically containing the country, rather than cooperation.

The White House submitted a report titled “United States Strategic Approach to The People’s Republic of China” to Congress on Wednesday, The Washington Post said. The 16-page report diagnosed that “a hope that deepening engagement would spur fundamental economic and political opening in the People’s Republic of China (PRC)” has failed and that the Chinese Communist Party “promotes globally a value proposition that challenges the bedrock American belief in the unalienable right of every person to life, liberty, and the pursuit of happiness.” The report announced that a competitive approach will be adopted toward China.

“When quiet diplomacy proves futile, the United States will increase public pressure on the PRC government and take action to protect United States interests by leveraging proportional costs when necessary,” said the report. The word “Malign” was used eight times throughout the report to describe China’s malicious behavior, investment, and intention, along with the phrase “predatory economic practices.”

Strengthening relationships with regional alliances and partners was mentioned as one of the measures to address Chinese threats. The “New Southern” policy of the current South Korean administration was pointed as one of the policies of regional alliances with which the U.S. should cooperate. Bruce Bennett, a principal researcher at RAND Corporation, said during an interview with Voice of America that the new report is practically Washington’s announcement of a new cold war on China.

# **S. Korea needs survival strategy amid U.S.-China dispute**

Signs of a heated power struggle between Washington and Beijing have been becoming clearer across the political, diplomatic and economic fields since the COVID-19 pandemic sparked tensions between them. “The CCP (the Chinese Communist Party)’s expanding use of economic, political, and military power to compel acquiescence from nation states harms vital American interests and undermines the sovereignty and dignity of countries and individuals around the world,” the White House said in a report submitted to the U.S. Congress. It also pointed out the need to rethink the policies of the past two decades toward China. Triggered by economic tensions and COVID-19 related accountability issues, the power struggle between Washington and Beijing has turned into a long-run warfare.

The world’s two biggest economic powerhouses have constantly engaged in obvious trade war since the Trump administration was inaugurated. These days, however, they have pointed an accusatory finger at each other since the COVID-19 pandemic took place, even using bluntly explicit phrases such as “a vicious authoritarian regime” and “totally insane.” As Beijing announced to enact national security laws regarding Hong Kong in its annual plenary session of the National People's Congress, Washington, in turn, reacted strongly by releasing a stringent action plan, which only shows the deepening of their conflict.

China is South Korea’s largest export destination followed by the United States while Seoul and Washington are close diplomatic and security allies. Being sandwiched between the two, the South Korean government has so far tried to convey strategic vagueness. However, it is currently under greater pressure to choose to side with either of them. For example, Washington has recently pushed Seoul to join “Economic Prosperity Network,” proposing to build a new global supply network among trusted security partners except China. South Korean semiconductor exporters are pressed into severing their more-than-10-trillion-won annual trade ties with China’s Huawei. Indeed, South Korea finds itself in a painful dilemma as it has high security dependence on the United States whereas its economy greatly relies on China. All of this reminds Seoul of the economic blow dealt by the THAAD issue.

Nevertheless, it is questionable whether the South Korean government gets fully ready for a new cold war era led by the United States and China. What’s worrying is that it pays attention merely to inter-Korean relations and Chinese President Xi Jinping’s visit to South Korea without any holistic action plan in place regarding global economic, diplomatic and security issues emerging in the post-COVID-19 era. As for Seoul’s de facto withdrawal from the May 24 measures taken following the North's torpedoing of the South's naval ship Cheonan, Washington expressed a feeling of unease by saying that the issue should be considered coupled with progress in denuclearization talks, leaving their relationship uncomfortable.

To better respond to the U.S.-China tensions, the South Korean Ministry of Foreign Affairs embarked on a diplomatic strategy meeting last year, which has not happened at all since the turn of the year. In contrast to that, South Korean President Moon Jae-in put emphasis on finding what the two Koreas can do, rather than expecting a great deal from the U.S.-North talks. A thorough review should be conducted of the South Korean government’s diplomatic and economic policy frame to see how it can handle Washington’s push for the E.P.N. initiative while considering its high economic reliance on China or to assume whether additional tension arises when President Xi visits Seoul this year.

# **U.S. harshly criticizes China calling it authoritarian regime**

The United States has ratcheted up criticism of China in the run up to the annual plenary session of the National People's Congress, the biggest political event in Beijing. U.S. President Donald Trump used words such as “wacko” and “dope” describing the Chinese government’s recent statement, while Secretary of State Mike Pompeo called China “a brutal authoritarian regime.”

“Some wacko in China just released a statement blaming everybody other than China for the Virus which has now killed hundreds of thousands of people,” said President Trump on his Twitter account on Wednesday. “Please explain to this dope that it was the incompetence of China and nothing else that did this mass Worldwide killing.”

“China has been ruled by a brutal, authoritarian regime, a communist regime, since 1949,” Secretary Pompeo said during a press conference on Wednesday morning, claiming, “Beijing is ideologically and politically hostile to free nations.”

“This plague has cost roughly 90,000 American lives, more than 36 million Americans have lost their jobs since March,” Secretary Pompeo said. “Could be as much as nine trillion dollars, according to our estimates, cost imposition on the world of the Chinese Communist Party's failures.”

# **Pres. Trump asks WHO to prove independence from China**

U.S. President Donald Trump who is in an intense confrontation with China regarding the source of COVID-19 has criticized the pro-China tendency of the World Health Organization (WHO) and given an ultimatum to permanently pull funding to the organization if it fails to “commit to major substantive improvements in the next 30 days.”

“If the World Health Organization does not commit to major substantive improvements within the next 30 days, I will make my temporary freeze of United States funding to the World Health Organization permanent and reconsider our membership in the organization,” read the four-page letter sent by President Trump to WHO Director-General Tedros Adhanom Ghebreyesus on Monday.

“The only way forward for the World Health Organization is if it can actually demonstrate independence from China,” the letter also said. “I cannot allow American taxpayer dollars to continue to finance an organization that, in its present state, is so clearly not serving America’s interests.”

President Trump also harshly criticized the WHO as a “puppet of China” in front of correspondents. He also took an issue with the U.S. contributing 450 million dollars to the WHO while China pays 38 million dollars.

“At least one member country made a mockery of their transparency obligations, with tremendous costs for the entire world. This cannot ever happen again,” said U.S. Secretary of Health and Human Services Alex Azar in an apparent reference to China during the World Health Assembly (WHA) meeting held via videoconference for the first time on Monday.

Meanwhile, Secretary of State Mike Pompeo announced a statement criticizing the WHO for failing to allow Taiwan to participate in the WHA due to opposition from China. Pompeo wrote that the WHO director-general did not allow Taiwan in the WHA due to pressure from China despite all the legal authorities he has to do so and that the director-general’s lack of independence has damaged the WHO’s reliability and efficiency.

National Security Council spokesman John Ullyot criticized Chinese President Xi Jinping’s announcement during the WHA meeting to provide two billion dollars to countries heavily affected by COVID-19, saying that it is an attempt to distract a growing number of countries asking for China’s responsibility.

# **U.S. destroyer sails off coast of Shanghai**

Tensions between the United States and China are intensifying not only in trade but also in the military. U.S. warships have unusually sailed through waters near China while both countries are engaged in a race to develop new weapons.

The South China Morning Post reported on Saturday that USS Rafael Peralta (DDG-115), an Arleigh Burke-class destroyer, was seen about 213 kilometers off the coast of Shanghai with a picture released by the South China Sea Strategic Situation Probing Initiative, a think tank at Peking University. The U.S. Pacific Fleet also said in a Twitter post, “USS Rafael Peralta sails in the East China Sea this week,” without specifying the date.

The sailing of the ship came as the People’s Liberation Army of China began live-fire drills in Bohai Bay of the Yellow Sea on Thursday, which is expected to involve aircraft carriers and will continue for two months and a half.

This is the second time a U.S. warship was seen off the coast of China in a month, following USS McCampbell (DDG-85), another U.S. Navy destroyer, spotted 42 nautical miles off the coast of Weihai in Shandong on April 17.

The United States are strengthening its military power. Earlier on Friday, U.S. President Donald Trump said the United States was developing a “super duper missile,” unveiling the flag for his Space Force. He went onto say that, “We have to do it with the adversaries we have out there, and I heard the other night it is 17 times faster than what they have right now.” His remarks appear to target China and Russia that are developing hypersonic weapons, said CNN.

# **U.S.-China tensions are through the roof**

Chinese media outlets have blamed U.S. President Donald Trump for making a “lunatic” statement saying that he "could cut off the whole relationship" with China. Some of them have made an aggressive remark to attack Taiwan if Washington severs the U.S.-China relations. Meanwhile, the prime target of Washington is reportedly Chinese-based companies listed in the U.S. stock market.

The Global Times, a sister newspaper under the People's Daily, criticized President Trump’s remark as “lunacy” in its editorial on Friday. “China should be prepared for a partial or complete decoupling in vital sectors, including technology, the economy, humanities, and social sciences,” it argued.

The newspaper gave President Trump raw criticism saying, “Trump is like a cornered beast doing something desperate,” and “Is Trump totally insane?”

“In the past, we didn't solve the Taiwan question because we wanted to maintain the China-US relationship, and if the U.S. unilaterally cuts it off, we can just reunify Taiwan immediately since the Chinese mainland has an overwhelming advantage to solve this long-standing problem," said Jin Canrong, the associate dean of Renmin University of China's School of International Studies in Beijing.

President Trump told Fox Business on Thursday that his administration is looking "very strongly” at mandating Chinese companies to follow U.S. accounting standards when they pursue an IPO in the U.S. stock market. He may intend to consider imposing sanctions against Chinese companies on NYSE or NASDAQ that do not stick to Generally Accepted Accounting Principles (GAAP), which U.S. firms have followed. Washington has reportedly made some preparations to attract semiconductor and pharmaceutical fabricators and suppliers to the United States as a way to ostracize China from the global supply chain.

Meanwhile, risks of accidental conflict between Washington and Beijing are mounting due to the ever-escalating military tensions over South China Sea and Taiwan. According to CNN, the United States is increasing military pressure on China by dispatching battleships and strategic bombers to South China Sea.

# **U.S.-China tensions are through the roof**

Chinese media outlets have blamed U.S. President Donald Trump for making a “lunatic” statement saying that he "could cut off the whole relationship" with China. Some of them have made an aggressive remark to attack Taiwan if Washington severs the U.S.-China relations. Meanwhile, the prime target of Washington is reportedly Chinese-based companies listed in the U.S. stock market.

The Global Times, a sister newspaper under the People's Daily, criticized President Trump’s remark as “lunacy” in its editorial on Friday. “China should be prepared for a partial or complete decoupling in vital sectors, including technology, the economy, humanities, and social sciences,” it argued.

The newspaper gave President Trump raw criticism saying, “Trump is like a cornered beast doing something desperate,” and “Is Trump totally insane?”

“In the past, we didn't solve the Taiwan question because we wanted to maintain the China-US relationship, and if the U.S. unilaterally cuts it off, we can just reunify Taiwan immediately since the Chinese mainland has an overwhelming advantage to solve this long-standing problem," said Jin Canrong, the associate dean of Renmin University of China's School of International Studies in Beijing.

President Trump told Fox Business on Thursday that his administration is looking "very strongly” at mandating Chinese companies to follow U.S. accounting standards when they pursue an IPO in the U.S. stock market. He may intend to consider imposing sanctions against Chinese companies on NYSE or NASDAQ that do not stick to Generally Accepted Accounting Principles (GAAP), which U.S. firms have followed. Washington has reportedly made some preparations to attract semiconductor and pharmaceutical fabricators and suppliers to the United States as a way to ostracize China from the global supply chain.

Meanwhile, risks of accidental conflict between Washington and Beijing are mounting due to the ever-escalating military tensions over South China Sea and Taiwan. According to CNN, the United States is increasing military pressure on China by dispatching battleships and strategic bombers to South China Sea.

# **The importance of not being swayed amid escalating U.S.-China tensions**

Chinese President Xi Jinping expressed his continued commitment to visiting South Korea this year on a phone call to President Moon Jae-in on Wednesday, said the South Korean presidential office Cheong Wa Dae. In response, President Moon emphasized the importance of President Xi’s visit in the bilateral relations. The phone call was arranged at the request of President Xi.

The call took place as the U.S.-China tensions are escalating. It is clear that Beijing wants Seoul on its side while South Korea wants President Xi’s visit to the nation as soon as possible to resolve the conflicts caused by the deployment of THAAD. However, the Chinese state media has made no mention of President Xi’s visit and simply stated the two nations are in the same boat against the COVID-19 crisis.

It appears the tensions between the United States and China over the origins of the pandemic will spread to all the other areas. U.S. President Donald Trump argues that China is responsible for the coronavirus outbreak while threatening to impose higher tariffs on Chinese goods and requesting cooperation from allies to shift the global supply chain that is heavily dependent on China. Trump’s intention seems to go beyond waging a trade war to reestablish the global trade order. Against this backdrop, if Seoul sides with one of the two countries, it will either have to face economic retaliation or undermine the foundation of foreign security cooperation.

The U.S.-China conflicts, which are expected to continue until the U.S. presidential election scheduled in November, might have unprecedented consequences. If the tensions remain only in trade, South Korea’s economy might be able to ride them out with its renewed strength. However, if they trigger a change in international order, South Korea will be left with only few options. Being wooed by both Washington and Beijing can be a double-edged sword for Seoul: The atmosphere might provide Seoul with more room for maneuver, but it can also leave it with no room at all if anything goes wrong.

It is true that there is alliance fatigue because of President Trump’s tendency to determine the value of an ally purely based on money. South Korea is faced with a difficult choice, and we need wise foreign policies that can guide us through these trying times. The South Korean government should set a clear direction for foreign policies with the South Korea-U.S. alliance at heart, based on which, it can decide the extent of cooperation with China. This would encourage Washington and Beijing to come to the negotiating table with more realistic options rather than trying to draw us in with unrealistic expectations.

# **China tries to hack COVID-19 research data, NYT reports**

With the entire international community scrambling to find a cure to the novel type of coronavirus called COVID-19, some 10 countries including China are reportedly mobilizing hackers to get their hands on the information on COVID-19 vaccines, The New York Times reported on Sunday.

“China is trying to steal the research data on the development of treatment and vaccines for COVID-19 from the United States. It is aiming to get access to America’s intellectual property and public health data through illegal channels,” warned the FBI and the Department of Homeland Security. They pointed out that Beijing is mobilizing students, professors, and researchers instead of agents to steal the information on COVID-19 from America’s universities and private research centers.

Washington has long held the suspicion that China’s “Thousand Talents Program” is part of the country’s broader scheme to poach America’s cutting-edge technologies. The FBI is planning to visit major universities in the U.S. to urge them to beef up security. In January, Charles Lieber, a professor at Harvard University, was arrested for lying about his ties to the Chinese state-run recruitment program. From 2012 to 2017, he was awarded millions of dollars from the Wuhan University of Technology but made a false statement about the payment he received.

U.S. President Donald Trump has been accusing China of being the epicenter of the coronavirus. Against this backdrop, China is retorting hard and experts voice the concern that the latest controversy might further fuel the bad blood between Washington and Beijing.

# **U.S.-China conflicts on COVID-19 reignite tensions in global trade**

South Korea’s benchmark KOSPI has fallen below 1,900 points again on Monday. It is because foreign investors sold Korean stocks worth one trillion won due to the anxiety that the trade war between the U.S. and China in a state of lull could be resumed. It threw a wet blanket to the South Korean economy, which started to have hopes that economic activities, would become more active soon as the country successfully flattened the COVID-19 curve compared to other countries.

What triggered the possibility of a trade war between the world's two largest economies was U.S. President Donald Trump’s remarks that Washington may impose additional tariffs on one trillion dollars worth of Chinese goods, saying that China should be held responsible for the spread of the COVID-19 outbreak.

South Korea’s exports plummeted by 23 percent year-on-year in April, and the trade balance is in deficit for the first time in 99 months since January 2012. If Trump starts to batter China to create public opinion favorable to him before the 2020 presidential election in November, it would be another mega-blow to South Korea, which was already severely affected in the exports market and global supply chains amid the prolonged pandemic.

A flash of hope is that South Korea earned a good reputation in the curve-flattening process and its economic potential is highly assessed in the global market. For instance, the price of DRAM memory chips recorded the highest increase in 39 months in April thanks to the projections that non face-to-face activities would increase in the future. Samsung Electronics and SK Hynix accounted for more than 70 percent of the share in the global market last month. South Korea is also doing well in the automobile market as Hyundai Motor became the highest-selling brand in the Vietnamese market last month, exceeding Japan’s Toyota.

The South Korean economy has still lots of stumbling blocks ahead. The global economy is at the beginning of a recession and the trade war is likely to be prolonged until the U.S. presidential election in November. South Korea needs to do what we can and should do in this situation. The South Korean government should revitalize the domestic market by boosting consumption and raise morale of businesses by easing various regulations. In the long term, South Korea should increase the ratio of domestic consumption to cope with the post-COVID-19 era with stronger protectionism, while working on diversifying the exports market to the South East Asia, India, Europe and more.

# **S. raises voice of criticizing Wuhan for causing COVID-19 outbreak**

Tensions between the U.S. and China are rising regarding the source of the COVID-19 outbreak. As U.S. President Donald Trump and Secretary of State Mike Pompeo have been blaming China for causing the outbreak and demanding an investigation with provocative remarks, China is harshly criticizing it as a “political show.”

“Personally, I think they made a horrible mistake, and they didn’t want to admit it,” Trump responded to a question on Sunday asking if he thinks the virus has originated from a Chinese research institute. “They tried to cover it, like a fire… They couldn’t put out the fire.” The president said he will get a report on the origins of the virus and how the Wuhan Institute of Virology might be involved, which would be “very conclusive.” He mentioned applying retaliatory tariffs on Thursday, saying that he has seen the evidence that the virus has originated from the Wuhan institute.

“There is enormous evidence that that’s where this began,” Pompeo said during an interview with ABC on Sunday, adding that this is not the first time that the world is exposed to viruses due to the failures of Chinese research institutes.

To a question asking if the virus was spread intentionally by China or by mistake, the secretary of state answered that there are many questions to be resolved, which require an on-site investigation. “We have come up with a bill to put sanctions on China until the country cooperates with an investigation into the Wuhan institute,” said Lindsey Graham, a Republican Senator and a close confidant of the president.

In the U.S., there are two theories regarding the origins of COVID-19 – the Wuhan institute produced the virus to use it as a biological weapon; or the virus was leaked from the institute by accident. “The biological weapon hypothesis is unlikely while the accidental leakage scenario is more probable, but no direct evidence is not available yet,” said Axios, a website covering political news. Meanwhile, President Trump said it was Chinese “mistake” on Sunday and Secretary Pompeo also said there is no reason to doubt that COVID-19 has not been man-made. The two seem to have emphasized the necessity of an investigation into the Wuhan institute to find out how the virus has been leaked, even if it was an accident.

The Associated Press reported on Sunday that Chinese leaders “intentionally concealed the severity” of the pandemic from the world in early January to stockpile medical supplies and equipment according to a four-page Department of Homeland Security intelligence report. The Chinese government deployed reporting on the dangers of COVID-19 to the World Health Organization (WHO) while importing medical supplies from foreign countries, leading to the country’s import surge of masks and protective gloves at the beginning of this year.

The U.S. and China are also confronting each other regarding Taiwan’s participation in a WHO meeting. The World Health Assembly (WHA), the highest policy-setting body of the WHO, will hold a video conference meeting on May 18. The State Department and the U.S. representative at the United Nations posted a hashtag on Saturday, supporting Taiwan to become a member of the WHO. The American Institute in Taiwan, which works as the Taiwanese embassy in the U.S., announced on the same day that it will update daily posts on Facebook supporting Taiwan’s joining of the WHA.

China is strongly opposing the U.S.’s claim, saying that the U.S. is politicizing the COVID-19 issue. “It is in violation of the One-China policy that Taiwan is part of the country – it may send wrong signals to independent forces in Taiwan,” the Chinese representatives in Geneva said.

# **Trump says he’s seen evidence coronavirus started in China lab**

U.S. President Donald Trump indicated a possibility of imposing a tariff on China, maintaining that he had seen evidence that the Wuhan Institute of Virology is the origin of COVID-19. Critics see his message as a concrete and stringent attempt to win his re-election race. There is also a likelihood that the U.S.-China trade tensions can go up again despite their signing of a first-phase trade agreement in January.

When asked if President Trump has seen any concrete evidence that COVID-19 escaped from the virology institute in Wuhan in a press conference at the White House on Thursday (local time), he gave a clear yes twice. However, he did not disclose any detailed evidence although arguing that China failed to contain the spread of the virus or intentionally let it spread. “We should have the answer to that in the not-too-distant future and that will determine a lot how I feel about China,” President Trump said, adding that an investigation is underway.

President Trump also answered that merely introducing a tariff on China can bring more money to the United States, in response to a question if he would consider having the United States not fulfilling debt obligations to China as punishment for the virus.

The U.S. government has reportedly been considering stripping China of sovereign immunity under the Foreign Sovereign Immunities Act (FISA), according to The Washington Post. It intends to sue China for coronavirus damages in a U.S. court. CNN also reported that Washington is thinking of economic sanctions, refusal of debt payment and new trade policy.

“The Intelligence Community also concurs with the wide scientific consensus that the COVID-19 virus was not manmade or genetically modified. The IC will continue to rigorously examine emerging information and intelligence to determine whether the outbreak began through contact with infected animals or if it was the result of an accident at a laboratory in Wuhan,” the Office of Director of National Intelligence (DNI) said in a statement on Thursday. Some experts see that the DNI, a U.S. governmental arm in charge of 17 national intelligence agencies, implicitly agreed that COVID-19 originated from Wuhan.

# **Pres. Trump says to demand compensation for COVID-19 from China**

As U.S. President Donald Trump said he plans to demand that China pay a "very substantial figure" as compensation for the coronavirus outbreak, tensions between the U.S. and China are rising.

“We have not determined the final amount. It's very substantial. We're talking about a lot more money than Germany's talking about,” President Trump answered to a question by a reporter about German newspaper Bild’s claim for alleged “invoice” of 149 billion euros to China for “coronavirus damage” during a White House press conference on Monday, according to Politico. “If you look at the world, I mean, this is worldwide damage. This is damage to the U.S., but this is damage to the world.”

Peter Navarro, the White House’s Director of Trade and Manufacturing Policy and a well-known hardliner against China, appeared on Fox News and criticized China for exporting low-quality test kits for COVID-19 and making undue profits. He also said that more testing for the virus was vital to getting Americans currently in lockdown back to work but some Chinese test kits show incorrect results. He argued that Chinese test kits will impact the normalization of the economy.

The Trump administration is also strengthening its cooperation with Taiwan. Secretary of Health and Human Services Alex Azar talked to Taiwanese Minister of Health and Welfare Chen Shih-chung over the phone on Monday to reinforce cooperation between the two countries for the development of COVID-19 vaccines and treatments. Bloomberg reported that the two leaders of health talked for 30 minutes, which shows strong support of the U.S. for Taiwan.

# **With 71 cases in Harbin, China takes lockdown measures again**

China has imposed lockdown on Harbin, the provincial capital of Heilongjiang province with 10.85 million population to prevent the spread of COVID-19. The city is suffering from an overseas infection that has spread to mass infection of 71 cases in the community.

The municipal government of Harbin announced on Wednesday that it has banned entry to residential zones in the city and urban villages by people and vehicles registered elsewhere. Weddings and funerals are also banned as well as large-scale events such as performances, sports events, forums, and exhibitions.

China had lifted isolation measures by assessing that it succeeded in controlling the virus pandemic. However, as the mass infection caused by an imported case raised concerns on the possibility of a second outbreak, a wary Chinese government has imposed strict measures once again.

The recent cluster case in Harbin started out with a Chinese student studying overseas named Han (female, 22), who returned home from the U.S. via Hong Kong and Beijing. The virus spread to her family and neighbors, some of whom visited two major local hospitals, where the mass infection spread. This caused infections in Fushun of Liaoning and Inner Mongolia, both of which saw one confirmed case each. The municipal government of Harbin is inspecting potential infections of 4,106 people who had been at the hospitals at that time.

Han has reportedly travelled to Shanghai early this month as well. There are concerns that a massive outbreak could take place again in China.

The Chinese foreign ministry advised Wednesday people in China not to travel overseas and those who are outside of the nation not to travel to other countries. This is seen as a measure to prevent cross border movement of Chinese people ahead of the upcoming Labor Day holiday on May 1.

# **China under growing criticism for COVID-19 outbreak**

A sentiment that China is responsible for the outbreak of COVID-19 is growing. The leaders of the U.S. and some European countries are calling for a clear explanation about a suspicion that China had tried to reduce the extent of the outbreak.

According to Reuters, German Chancellor Angela Merkel said on Monday that the Chinese government should transparently reveal the origin and the initial spread of COVID-19 so that the entire world can learn lessons and make improvements. The sentiment to put responsibility for the virus on China has been growing recently among German media companies, including tabloid newspaper Bild. “Let’s not be so naive as to say China has been much better at handling this. There are clearly things that have happened that we don’t know about,” said French President Emmanuel Macron during an interview with The Financial Times on Friday. U.K. Secretary of State for Foreign Affairs Dominic Raab also said during a press conference that a thorough investigation of how the virus has spread in China is needed.

Unlike the U.S., European countries had been refraining from criticizing China until recently regarding the spread of the virus as their relations with the U.S. have grown apart while their dependence on China has increased since the election of U.S. President Donald Trump. Regarding such a shift among European countries towards China, U.S.-based magazine Foreign Policy explained that COVID-19 data published by China has lost its reliability and the virus response measures of other countries based on such data are also misinformed. Distrust on China has grown as the country has failed to reveal the exact timing of the virus outbreak and the number of total deaths.

As criticism on China grows throughout the world, the World Health Organization, which has been defending China, has found itself in a tricky situation. During a press conference held on Monday at the organization’s headquarters in Geneva, Switzerland, WHO Director-General Tedros Adhanom Ghebreyesus said they have not hidden anything to the U.S.

# **China’s economy records historic quarterly plunge due to coronavirus**

Amid the spread of COVID-19, China has recorded a negative growth in the first quarter of the year for the first time since related statistics were first officially gathered. Accordingly, the Chinese government has signaled a tough economic stimulus program.

Mao Shengyong, spokesperson of the National Bureau of Statistics of China, said in a press conference on Friday that China’s GDP decreased by 6.8 percent to 20.65 trillion yuan (approximately 3,554 trillion won) in the first quarter of the year on a year-on-year basis, marking both the lowest and the first negative figure since Beijing first announced quarterly growth rates in 1992. The Chinese economy experienced a historic drop of 12.8 percent points compared to the six percent increase during the fourth quarter of last year.

Previously, China recorded a negative yearly growth at -1.6 percent in 1976 when the Cultural Revolution drove up risks of an economic meltdown. It can repeat itself in 44 years if this year ends up with a negative growth.

“The total production decreased by 9.6 percent and 5.2 percent in manufacturing and service industries, respectively, during the first quarter of the year,” said Mao. Industrial production went down by 8.4 percent while retail sales, a barometer of consumption demand, plummeted by a whopping 19 percent.

# **China’s cover-up of COVID-19 death toll in Wuhan is revealed**

The suspicion that the Chinese government has been underreporting the number of deaths from COVID-19 has been confirmed. According to Xinhua News Agency, the Wuhan municipal government in Hubei province reported on Friday that the total number of deaths in the city as of Thursday is 3,869, which is 1,290 higher than the previous official figure. The city’s number of confirmed cases is also 325 higher than the previous figure at 50,333.

The death toll in Wuhan as of Thursday announced by the National Health Commission of China two hours earlier on Friday was 2,579. This means deaths equivalent to about 50 percent of the previously reported figure had not been included until now.

Control and Prevention, and the Public Security Bureau have worked together to come up with the new figure based on data from hospitals, nursing homes, funeral data systems, etc. The city authorities explained that such a gap in death toll has been caused by some patients died at home without having been treated in hospitals due to the surge of new cases in the early stage of the COVID-19 outbreak; during the height of their treating efforts, hospitals were operating beyond their capacities and medical staff was preoccupied with saving and treating patients, resulting in belated, missed and mistaken reporting; and the registered information of some of the deceased patients was incomplete, and there were repetitions and mistakes in the reporting. This, however, led to criticism that the authorities are shifting the responsibility for unreported deaths to health care providers in the front line.

The distrust of the Chinese government’s COVID-19 statistics has been growing as figures were revised at times and suspicion for the government’s cover-up has been raised. Chinese media Caixin recently reported that the number of urns shipped for COVID-19 deaths in Wuhan looked to be more than double the city’s death toll.

# **China bars entry by almost all foreigners to contain virus pandemic**

China has announced an extraordinarily strong measure of effectively blocking the border at the pretext of containing the coronavirus pandemic. Not only South Korean companies that operate production plants and sales units in mainland China but also South Korean nationals living there and students of Chinese universities who are currently staying outside China have been denied entry, which will cause inevitably significant chaos and confusion to them.

The Chinese ministry of foreign affairs and the national immigration bureau have decided to ban foreigners with visas for long-term stay and residential permits from entering China, effective midnight Saturday. China said people could still apply for visas through local Chinese embassies and consulates if they have justification for a visit due to economy and trade, science and technology activities and urgent humanitarian reasons.

However, the South Korean Embassy in China said, "We have asked Beijing for a detailed explanation about the circumstances in which foreigners can apply for a visa as an exception, but the Chinese government has not given any clear answer yet."

The entry ban into China was announced only one hour after the leaders of the G20 countries including Chinese President Xi Jinping issued a joint statement shortly after their video summit. The joint statement read, "We will cooperate to respond (to the pandemic) in a way that will promote international trade, and that will not cause unnecessary obstacles to travel and trade between countries.”

# **S. overtakes China for most confirmed COVID-19 cases**

The number of COVID-19 cases in the U.S. surpassed 85,000 on Thursday (local time), making the U.S. the country with the highest number of confirmed cases in the world. It has been 65 days since the first COVID-19 case was reported in the country on Jan. 21.

According to global statistics site Worldometer, the number of new confirmed cases in the nation rose by 16,939 on Thursday, increasing the total number to 85,520, which is larger than China’s 81,340. The death toll climbed by 261 to reach 1,297 on the day. The novel coronavirus is spreading at a rapid pace in the U.S., with the number of confirmed cases in the country surpassing 80,000 a week after the total number reached 10,000 on last Thursday.

The New York state has 37,258 cases. White House Coronavirus Task Force Coordinator, Deborah Birx said that 55% of those infected have come out of New York and New Jersey, adding that Chicago and Detroit are also seeing a surge in new coronavirus cases. New York Gov. Andrew Cuomo on Thursday approved hospitals to treat two patients with one ventilator, reflecting a lack of medical staff and equipment in the state.

Bloomberg criticized the Trump administration for its poor judgement of the situation and lack of initial preparation, adding, “The coronavirus isn’t Trump’s Katrina. It’s his Vietnam.” President Trump came under fire once again by saying he wants to reopen America before Easter, which falls on April 12, during a press conference.

# **Another crisis awaits China, the biggest beneficiary of globalization**

All eyes have recently been on a Chinese student named Yao Yao across Chinese social media platform Weibo, who successfully came back home from Italy where he studied. With the COVID-19 pandemic worsening across Italy, he decided to return to his hometown, Shenzhen in Guangdong Province, China.

Yao Yao did neither eat nor drink anything with his mask on for 28 hours all the way from Italy to his final destination via Abu Dhabi and Beijing. His pitiful story has garnered widespread support online being described as a so-called “textbook way of return.” However, the number of coronavirus infections from overseas are worryingly on the rise in China due to an increasing number of Chinese citizens evacuating European countries and the United States in the belief that China is a safer place to stay. The unexpected development has puzzled Chinese health authorities, which have confidently boasted about having none of new infections reported at home.

In response, the Chinese government has put tight entry control in place while ordering international flights to Beijing to arrive at an airport near its capital city instead. The recent prohibitive measures are not line with Beijing’s criticism over other countries closing the door to Chinese citizens at the peak of the pandemic across China. A series of aviation limits have wreaked havoc on major Chinese airlines, including Air China with the number of their passengers in February nosediving over a whopping 80 percent on a year-on-year basis.

Added to that, the global supply chain is suffering from the ever-tightening of border controls across the world. It is a relief that China-based factories have resumed production since the slowdown of the virus at home. Nevertheless, their comeback does not translate into 100 percent operation as they fall short of raw material due to global supply disruptions. According to the U.S. Chamber of Commerce’s survey of 237 businesses located in southern China, 32 percent face supply shortages of raw material.

Overall, the Chinese economy has been hit hard by the decreasing consumption demand across the globe. Despite the resumed operation last month, a Hangzhou-based auto part company sees its orders decline 30 percent in volume compared to normal levels due to decreases in demand not only in China but also in South Korea and Japan, according to The Washington Post. U.S. shoemaker Steve Madden and electronics fabricator Best Buy produce 73 percent and 60 percent of their products within China, respectively, said U.S. analysis firm Coresight Research. This implies that decreasing demand in the U.S. market driven by the COVID-19 outbreak can shrink factory production in China, then affecting the Chinese economy as a whole.

Beijing finds itself in a position to face the economic consequences of global supply disruptions and shrinking demand although it has been waiting for the perfect timing to declare its victory in the war against COVID-19. The world’s No.1 beneficiary of globalization seems to be just a few steps away from becoming the biggest victim of a growing headwind in the globalized market. The Chinese government alone is not able to fight against the second crisis coming on the scene unlike COVID-19 within the country.

Regrettably, the United States and China, the two biggest economic powerhouses highly interdependent in terms of production, consumption and trade, are pointing an accusatory finger at each other while wasting time arguing over coronavirus responses and accountability as an epicenter. Worryingly, the ongoing U.S.-China tug-of-war will inevitably offset efforts by the rest of the world to beat the coronavirus-induced shock to the global economy.

# **China could take exception to S. Koreans’ entry, says Ambassador Xing**

Chinese ambassador mentions of exception on S. Koreans with health certificate

Chinese Ambassador to South Korea Xing Haiming said that essential visits by South Koreans to China are guaranteed and China will provide South Koreans visiting the country with the convenience they need. It is the first time, since Seoul and Beijing started discussing applying exceptions to China’s entry ban, that a high-ranking Chinese official indicated a positive stance towards making an exception on South Korean businessmen carrying a health certificate or a certificate certifying that he/she has been tested negative for coronavirus.

In an exclusive interview with the Dong-A Ilbo held at the Chinese Embassy in Seoul on Tuesday, Ambassador Xing said it is likely that South Korean businessmen will be exempted from quarantine measures if they pass airport health checks in South Korea and China. As of Tuesday, 24 Chinese local governments are putting visitors from South Korea in quarantine for two weeks.

Ambassador Xing said China is seriously reviewing the proposal made by South Korea during their first video conference on response to COVID-19 to allow South Korean visitors entry into the country if they can prove their health status by submitting a health certificate.

# **‘S. Korea and China are friendly nations on the same boat,’ says Xi**

Chinese President Xi Jinping sent a message of sympathy to his South Korean counterpart Moon Jae-in concerning the ongoing COVID-19 pandemic.

President Xi described that China and South Korea are friendly nations on the same boat, adding that the South Korean government and people from all walks of life have extended their sympathy to China and provided it with enormous support, according to Chinese state-run TV network China Central Television (CCTV) on Saturday. He repeated the importance of cooperation between the two nations in overcoming the novel coronavirus outbreak as stressed by President Moon over the phone on Feb. 20 that China's difficulties are South Korea's difficulties.

The Chinese leader emphasized that pandemics have no borders and all countries around the globe share a common future. He went on to say that the Chinese government and people empathize with South Korea’s struggle, promising to do all the best to assist Seoul in weathering the outbreak. In his message, Xi valued the relationship between Beijing and Seoul highly, hoping to work with President Moon to take their cooperative partnership to a higher level.

South Korea and China held a video conference on Friday among foreign affairs and public health officials to strengthen cooperation amid the COVID-19 pandemic while embarking on a joint quarantine system.

# **China understands Japan’s entry restrictions with little objection**

China has shown a somewhat different reactions from South Korea in the wake of Japan’s travel limits that in effect ban travel to and from its two neighboring nations. Both the Chinese government and media outlets have not raised any issue concerning the entry ban, describing it as “understandable.” It is analyzed that China’s reactions symbolically demonstrate the relationship between Beijing and Tokyo that has recently been improving.

“Both China and Japan are taking science-based, professional and appropriate measures in order to protect the health and safety of their nationals and foreigners and uphold regional and global public health security, which I believe we all understand,” Chinese Foreign Ministry spokesperson Zhao Lijian talked about Japan’s entry limits on Thursday. “The two sides are in close communication through diplomatic channels.” His remarks imply that Tokyo gave advance notice to Beijing.

Even China’s state-run media Global Times used the word “understand” in the title of an article posted on its official social media account reporting a 14-day quarantine on travelers from China and South Korea that has recently been announced by Japanese Prime Minister Shinzo Abe.

On Weibo, the Chinese social media network, few comments criticized Japan’s measures explicitly although many users poured out their concerns and inquiries about invalidation of tourist visas issued to Chinese citizens. In a comment on an article regarding Japan’s entry ban, a Weibo user argued that Japan is not to blame given that China took the same action in an emergency situation at an earlier time while proposing that Seoul should do the same.

In some sense, such reaction from the Chinese government and media outlets is interpreted as a justification for the 14-day quarantine measure against travelers from Japan and South Korea taken earlier in many Chinese regions under the pretext to contain the outbreak of the novel coronavirus COVID-19 that can be caused by incoming visitors.

# **The coronavirus outbreak could affect Xi’s S. Korea visit**

There is a growing consensus in the South Korean government that Chinese President Xi Jinping’s state visit to South Korea might have to be rescheduled with the coronavirus outbreak showing no sign of slowing down soon.

On Tuesday, a senior official of the Ministry of Foreign Affairs said in a press conference, “A delay in President Xi’s visit to Japan, which was originally scheduled for April, has received much media coverage in Japan,” adding that his visit to South Korea might also be affected by the new coronavirus. Although these remarks were about the agreement between Tokyo and Beijing to postpone the visit to Autumn, it is worth noting that it was the first time a high ranking South Korean official mentioned a possibility of delaying President Xi’s visit to Seoul. However, the official also said, “All preparations are proceeding according to the original plan.”

Some experts say it would also be difficult to confirm‎ diplomatic schedules with Russia, which celebrates 30 years of diplomatic relations with South Korea this year. The latest coronavirus outbreak has disrupted the South Korean government’s plan to arrange Russian Foreign Minister Sergey Lavrov’s visit to Seoul for this month at the earliest. Reportedly, a number of working-level meetings have already been delayed or cancelled.

While the South Korean Ministry of Foreign Affairs is looking closely at the possibility of Washington imposing an entry ban on Seoul, the U.S. government announced that it would “tighten screening rules.” “There will be a 100 percent screening on all direct flights at all airports across Italy and across South Korea,” said U.S. Vice President Mike Pence in a press conference on Monday. However, when asked about stricter travel restrictions, U.S. President Donald Trump said he would place further restrictions on countries hit particularly hard by the infection.

Meanwhile, the Japanese Ministry of Foreign Affairs elevated Monday its warning against travel to six regions in South Korea including Gyeongsan, Yeongcheon, Chilgok, Uiseong, Seongju and Gunwi from “Level1” to “Level 3.” Level 3, which advises the public not to travel, is the second highest level.

As of Tuesday afternoon, a total of 89 nations, which takes up 46 percent of the UN member countries, have banned the entry of or isolated South Korean nationals. According to the South Korean Ministry of Foreign Affairs, the number of Koreans who have self-isolated or have been quarantined exceeded 1,200 as of Tuesday morning. It is reported that 960 and 270 of them are in China and Vietnam, respectively, while Russia, Kyrgyzstan and Qatar have more than 10 people each who are isolated.

# **Xi Jinping instructs to find the origin of COVID-19**

Chinese President Xi Jinping has directed scientists to find the origin of COVID-19. It is garnering attention as China’s official news agency and authorities recently argued that China may not be the origin of the virus.

Xi ordered Beijing’s military medical research center and Tsinghua University’s medical institute to trace the origin [of the virus] and conduct research on the virus using new technologies such as AI and big data, according to the report of CCTV on Monday. He also asked them to clarify where the pathogen came from and where it is going, and increase the accuracy and efficiency of tests.

“The virus may have more than one origin and multiple hosts,” said some experts.

The Global Times even asserted that the U.S. may be the origin early this month, which is interpreted as an intention to avoid China’s responsibilities as the virus has spread across the world.

# **China’s health authorities acknowledges possibility of aerosol transmission**

The highest health authorities in China have officially acknowledged the possibility of COVID-19 spreading through aerosol for the first time.

The National Health Commission of China newly added exposure to high concentrations of droplets in the air mixed with the virus in a closed environment for a long time as one of the possible routes of new coronavirus transmission in new guidelines for diagnosing and treating patients announced on Wednesday.

At the guidelines announced on February 4, the National Health Commission had said that the routes of aerosol transmission were not clear. Later, however, some experts and local governments suggested the possibility of aerosol transmission. Also in Feb. 8, the Shanghai government announced that transmission routes of the coronavirus included direct transmission, contact transmission and aerosol transmission. But controversies arose when the Chinese health agency refuted the theory saying that there is no evidence of aerosol transmission.

While droplets are comparatively heavy and cannot travel further than a radius of two meters most of the time, aerosol is lighter and spreads further. A cluster of infections may occur through aerosol left in closed spaces.

# **China’s coronavirus statistics loses credibility with 9-fold increase**

As China has changed the criteria to confirm‎ the Wuhan coronavirus patients, the numbers of infected cases and deaths from the virus in Hubei province soared. As a result, some are raising the suspicion that the new coronavirus has been spreading more rapidly than what has been reported and that the Chinese government has been hiding the true statistics.

The hygiene and health commission of Hubei province announced on Thursday that the number of confirmed patients rose by 14,840 with 242 more deaths in just one day on Wednesday, which is nine times and 2.6 times higher than the Tuesday figures, respectively. The national hygiene and health commission, which had been releasing official figures every morning, had not announced statistics until Thursday afternoon.

“Clinical diagnosis has been added as one of the criteria to confirm‎ infected cases in order to allow timely diagnosis of patients and increase treatment success rates,” explained Hubei province. “So far, cases had been confirmed based on the RNA test, but now patients can be confirmed even with diagnosis by medical professionals and the CT scan, which is why the number of cases went up.” According to Hubei province, the numbers of confirmed patients and deaths by clinical diagnosis account for 90 percent and 56 percent, respectively, of the increased figures on Thursday.

“We decided to include clinically-diagnosed patients as confirmed cases to match the confirmation criteria announced by other provinces,” said the Hubei provincial government, which is an admission to the fact that the province just started to apply the confirmation criteria that have been applied by other provinces.

# **The number of confirmed cases of coronavirus keeps increasing in China**

The coronavirus outbreak has killed 426 as of Tuesday, up 65 from the previous day. Hong Kong reported its first coronavirus death on Tuesday, confirm‎ing the second death outside mainland China following the Philippines.

The number of confirmed coronavirus cases in China has reached 20,438 on Tuesday, up 3,136 from the previous day. This is the first time that the number of new confirmed cases exceeded 3,000 a day. Amid the biggest daily increase of confirmed case and death toll on Tuesday since the virus was detected, Chinese officials said 2,788 people are currently in critical condition and the mortality rate of coronavirus in Wuhan City, Hubei province, the epicenter of the decease, has exceeded 5%, sparking concerns that the new virus’ fatality rate will continue to grow.

According to Ming Pao Daily News of Hong Kong, a 39-year-old man, who contracted the virus after travelling to Wuhan, died Tuesday while being treated since he was confirmed to have coronavirus on Friday. Hong Kong announced on Monday that it will close all border checkpoints with mainland China except for two, but Hong King citizens are calling for the full closure of border with mainland China.

# **Chinese ambassador asks S. Korea to follow WHO stance**

We can base our decisions on WHO recommendations,’ says Chinese ambassador

“I am not going to comment on Seoul's travel restrictions (entry restrictions on Chinese visitors),” said new Chinese Ambassador to South Korea Xing Haiming at a press conference held at the Chinese Embassy in Seoul on Tuesday.

He showed his displeasure rather than actively voicing his opposition to the South Korean government’s decision in a situation where Korean citizens’ criticism towards China is elevating due to the spread of the new coronavirus from China.

The ambassador said that he does not want South Korea to take on strong entry restrictions while quoting the stance of the World Health Organization. WHO Director-General Tedros Adhanom Ghebreyesus told the organization’s executive board that there was no need for measures that "unnecessarily interfere with international travel and trade" in containing the virus.

“The WHO is the most scientific, authoritative organization (on health issues), and I believe that we can base our decisions on WHO recommendations,” the Chinese ambassador said. "In the wake of such an issue, we can be seen as part of a community sharing the common destiny. I hope we can respond to the disease outbreak while putting ourselves in each other's shoes."

Xing also used the press briefing to express appreciation for Seoul's support for Beijing's fight against the coronavirus. "South Korea, through various channels, has sent messages supporting China’s fight against the contagious disease, and provided us with precious other support," he said. "We will not forget this warm support for good." Seoul has decided to offer five million U.S. dollars in emergency assistance to China, along with three millions of protective masks and other relief items.

The South Korean government is keeping its stance on taking additional measures even though China spoke of its displeasure towards Seoul’s strengthened entry policy. “The ministry is reviewing continuously the adjustment of travel warnings,” Kim In-cheol, spokesperson of the South Korean Foreign Ministry, told reporters on Tuesday

# **WHO declares Wuhan coronavirus outbreak a global health emergency**

The World Health Organization (WHO) has declared the coronavirus outbreak in China a public health emergency of international concern (PHEIC). “Over the past few weeks we have witnessed the emergence of a previously unknown pathogen, which has escalated into an unprecedented outbreak,” said WHO Director General Tedros Adhanom Ghebreyesus on Thursday (local time). But he has made it clear that the declaration is not caused by the outbreak in China but is made due to what is happening outside the country. He added that there is no reason to limit trade or travel to China.

The new coronavirus is spreading fast with the number of confirmed cases and deaths in China recording the biggest daily increase. The number of confirmed cases in China rose to 9,692, up by 1,866 from the previous day, and the death toll increased by 43 to 213. A total of 9,831 cases have been confirmed globally, exceeding 8,098 cases of severe acute respiratory syndrome (SARS) in 2003.

A possibility of an airborne infection has been raised in China. “Wuhan pneumonia is a lot different from SARS while much similar to the flu virus in terms of its transmission pattern,” a senior researcher at the Chinese Center for Disease Control and Prevention (CDC) said in an interview with CCTV. “A research study has found that the new coronavirus spreads through droplets or aerosol particles an infected person coughs out in a closed space.

# **Asymptomatic patient spreads the virus in China**

There are growing concerns over China’s Wuhan pneumonia, which has shown signs of group transmission and third infection, with many becoming fearful that the epidemic is going out of control.

According to the Sanitary Health Commission of Anyang City, Hunan province on Wednesday, the father (age 45) and two aunts of Mr. Liu, who had visited Wuhan before returning to his home in Anyang, were infected with the virus, which is secondary infection. The virus also spread to Mr. Liu's mother (aged 42) and another aunt, third infection. Though Liu was confirmed of the virus, there had been no symptoms after the latent period (up to 14 days), which implies the virus' potential of group transmission via no symptom patients. In Hefei City of Anhui province, six people in their 20s were infected after attending a gathering, confirmed of the virus on Tuesday. A family of six in Huangshan City, Anhui province were also found infected.

The number of accumulated confirmed cases in mainland China as of Tuesday reached 6018, outnumbering the cases in 2003 during the outbreak of the SAAS virus. The death toll recorded 132, which is 26 higher from the previous day. A case was first confirmed in the Middle East for the first time in the Arab Emirates.

Christian Lindmeier, spokesperson of the World Health Organization, said on Tuesday (local time) that the WHO cannot conclude the level of symptoms, which allows the infectee to spread the virus," hinting the chance of asymptomatic infections. However, Park Hye-Kyung, the head of the Central Headquarters for Emergency Operations at the Korean Center of Disease Control, objected to this statement, saying, "There is no such wording on the WHO document and asymptomatic infections are groundless." No further confirmed case was reported in Korea on Wednesday.

# **Wuhan’s cough shakes up Xi Jinping leadership**

Chinese President Xi Jinping’s leadership is suffering a blow due to a pneumonia outbreak arose in central China. The Chinese government has just revealed its inability to manage crisis, and the economy is likely to take a hit. The New York Times reported that Xi is facing the most severe political crisis for the first time in several years.

President Xi officially announced the government’s response to the Wuhan coronavirus as late as Saturday, which is 25 days after the Wuhan authorities announced the breakout of Wuhan pneumonia on December 30 last year.

As dissatisfaction towards the government’s nonchalant attitude grew, Chinese Premier Li Keqiang went to Wuhan City, Hebei province, which is the epicenter of the virus, and visited hospitals and marts to encourage medical staff and residents. “Tell me if you are having problems. I will solve them for you,” he said to construction workers who were building a temporary isolation ward. “There is no problem!” the workers said. But local news outlets reported that the workers talked about lack of resources such as masks and medical gloves a day before his visit.

There was a serious issue in the reporting and decision-making process as well. “We are not happy with disclosure of information,” said Wuhan Mayor Zhou Xianwang. “We, as a local government, were able to publicize information only after obtaining the authority and relevant information.

The Chinese medical system revealed vulnerabilities as well. According to The New York Times, Xiao Shibing (51), a resident in Wuhan, had a fever and difficulty in breathing about 15 days ago, but was hospitalized on Sunday because there were not enough beds in hospitals. “Hospitals kick around patients like they are a soccer ball,” said his wife Feng Xiu. Wuhan Communist Party Secretary Ma Guoqiang acknowledged that hospitals in Wuhan were extremely overcrowded in a press conference on Sunday.

If the virus reduces workdays and prolongs tourism, the Chinese government may not be able to meet the target growth rate of 6 percent. Hong Kong’s South China Morning Post projected that China’s growth for the first quarter could stand at the 4 percent range quoting a U.S.-based research center.

China has extended the spring festival holiday by three days until Sunday but is likely to extend it further due to the Wuhan pneumonia outbreak. The Shanghai government ordered businesses not to resume work until February 9. Reuters reported that Tesla, General Motors and Volkswagen that are operating a joint venture or a factory in the region will suffer a loss.

# **Warnings of Wuhan pneumonia**

Wuhan pneumonia caused by a novel coronavirus is spreading like wildfire. It has been less than a month since it first appeared in Wuhan, the capital city of Hubei province, on December 31, 2019, but the virus has already reached eight countries, including the United States, with 581 confirmed cases. Seventeen people who have died are all Chinese. In the meanwhile, South Korea has also reported one confirmed case. The Chinese female patient, who displayed symptoms at the Incheon International Airport, has been quarantined as of today. Despite being China’s transport hub with a population of 11 million, Wuhan has suspended all public transport services in and out of the city as of 10 a.m. on Thursday, becoming the first provincial capital city that has been shut down.

It is thought that the host of Wuhan pneumonia is bats or snakes. Severe acute respiratory syndrome (SARS) started in bats or civet cats while Middle East respiratory syndrome (MERS) in camels. The outbreak began at a seafood market in Wuhan where a variety of wild animals are illegally traded, and the second person who died from the virus owned a store in the market. It can take up to 10 to 12 days to develop symptoms, which are similar to those of a common cold or a flu. There is no treatment or vaccine yet. Given that Human-to-human transmission can occur when someone comes in contact with an infected person’s secretions such as saliva and mucus, it is advised to wear a mask when in public places. A mask should not be reused as it might have viruses on it.

Experts in Hong Kong warn that the outbreak might evolve into a pandemic as SARS did in 2003. According to them, infectious diseases are first transmitted from animals to humans, from humans to humans and then from patients to their families and medical staff before they become a full-fledged pandemic, and Wuhan pneumonia is already in the third stage. China has come under criticism for accelerating the spread of the virus with its cover-up and late response. Wuhan has been shut down, but it is estimated that millions of people have already left the city. The New York Times said the problem has exacerbated by the Chinese government’s tight control over information, the media and a civic society.

In the meantime, the lockdown of Wuhan has tumbled down China stocks. It is expected that the economic damages caused by the novel coronavirus would be larger than those inflicted by SARS in 2003, because China’s share of the global economy is estimated to increase to 20 percent from 8.7 percent in 2003. This could also impact the South Korean economy, which was showing signs of recovery, from the beginning of the year. Consumption might freeze just like five years ago, delaying a rebound of the growth rate.

The first crisis is the Luna New Year holiday, which starts today. With many people traveling and gathering, the risk of transmission goes up while most hospitals close. During the Lunar New Year holiday, more than 140,000 Chinese tourists are expected to flock to South Korea. The Korean government should operate a 24-hour emergency disease control system while citizens should closely follow the relevant news as well as the guidelines to prevent the spread of the virus such as washing hands and wearing masks.

# **China shuts down Wuhan where pneumonia began**

Amid increasing confirmed cases of “Wuhan pneumonia” and fatalities, the Chinese authorities has shut down the city where the virus began. Wuhan is the capital city of Hubei province with a population of about 11 million. It is the first time for the nation to shut down the capital city of a province.

In an emergency statement released at 2 a.m. on Thursday (local time), the city of Wuhan announced a suspension of bus, subway, ferry and intercity bus services as of 10 a.m. Those living in the city are advised not to leave the city, and airports and train stations have also been closed with no date or time set for the services to resume.

Located in the middle of China, Wuhan is a transport hub that connects nine neighboring provinces. The unusual lockdown comes as the risk of the virus turning into a global pandemic grows.

However, the confirmed cases have increased by 56 to 600 as of Thursday. Suspected and confirmed cases have been identified in 27 Chinese provinces out of 31, leaving only four provinces, Gansu, Qinghai, Xinjiang and Tibet, unaffected. Fears of a global pandemic are growing as suspected cases have also been detected in Mexico, Brazil, Columbia and Canada.

# **‘Wuhan pneumonia’ may become a global epidemic**

“Wuhan pneumonia,” caused by a novel coronavirus, is showing signs of wide outbreak across the world, with the first U.S. case confirmed and infections spreading across China.

According to Reuters News on Tuesday, the United States Center for Disease Control confirmed that a resident near Seattle, Washington was diagnosed with the Wuhan pneumonia after visiting Wuhan, China He is now under treatment since returning to the U.S. last Wednesday. It is the first confirmed case outside Asia.

The Chinese Center for Disease Control announced Wednesday that the number of confirmed cases grew to 440 (including Taiwan), meaning that around 130 cases increased in just one day. Confirmed patients were found across 13 cities and provinces across the nation. When including suspected patients, the number of potentially infected areas would include 21 cities and provinces, accounting for 68% of China’s 31 cities and provinces. Against this backdrop, Macao also confirmed its first confirmed case on the day. Also the number of deaths increased from six to nine in China. With 1,394 people who had close contract with the patients put under medical observation, the number of patients is expected to increase.

The Chinese New Year Day (Jan. 25), which more than 450 million out of 1.4 billion Chinese travel, is expected to be a turning point of the epidemic. The Chinese authorities have urged people to stop travelling in and out of Wuhan.

# **‘Fears of SARS’ rise in China ahead of Lunar New Year holiday**

The sharp rise of “Wuhan pneumonia” due to a new coronavirus in China has prompted concerns over the Chinese government’s ability to contain infectious disease outbreak. Some Chinese Internet users are accusing the government of a cover-up and late response while others worry if the virus could become as fatal as SARS, which killed 648 people across mainland China and Hong Kong in 2002 and 2003 after Beijing failed to contain the spread.

“Wuhan pneumonia” are spreading rapidly and widely. In Wuhan, Hubei province, where the virus was first detected, confirmed cases tripled over the weekend by 136. In addition, it appears that the virus is spreading across the country. Two and one cases were confirmed in Daxing and Shenzhen, respectively, while eight suspected cases were found in Shenzhen and five in Zhejiang. It contradicts the Chinese authorities’ claim that the virus is not highly contagious and not likely to be transmitted through human to human contact.

The Chinese government had dealt with the outbreak at the municipal level, but China’s National Health Commission announced prevention measures and sent working groups to provinces across the country on Sunday. The authorities said it had not identified the source of the outbreak as well as the exact transmission pathways. A source from the Korean government said, “The Chinese authorities applied a new method, which has driven up confirmed cases in Wuhan.” The possibility of this turning into a major outbreak cannot be ruled out given that about 3 billion people are expected to travel over the Lunar New Year holiday.

Concerns are rising among Chinese Internet users as well. “The government said it is unlikely to be highly infectious. Was it another cover-up?”, said a person. Another person left a sarcastic comment saying, “The virus is no longer patriotic.”

# **New generation poses threat to ‘Chinese Dream’**

This reporter visited Taipei last December to cover the presidential election in Taiwan. Young Taiwanese in their 20s described themselves “Born independent.” It means they were raised believing Taiwan is an independent country. “This is why we cannot accept China’s one country, two systems,” said a Taiwanese university student.

Taiwanese who are in their 20s and 30s see themselves as Taiwanese, not Chinese. Their votes were what secured the second term of Taiwanese President Tsai Ing-wen who opposes closer ties with China. What has given them strong Taiwanese identity was the country’s democratization.

Despite the democratic movement in the 1980s, Taiwan remained an authoritarian society with the Kuomintang party, which mostly consisted of Mainlanders, in power until 1996 when the first direct presidential election was held. Many from older generations, who were born before the 1970s, support unification with China whereas young Taiwanese who were born in a democratic society has strong Taiwanese identity. The young Taiwanese are neither confused on their identity nor torn between unification and independence unlike older generations over 40. “Young people used to be only interested in personal issues such as finding a job,” said pundits in Taiwan. “The high turnout of young voters in the latest election, however, shows that they are determined not to leave their future in the hands of older generations.”

Following the landslide victory in the presidential election, Taiwanese newspapers reported that the younger generation is evolving from “born independent” into “born Taiwanese.” From their point of view, Taiwan is already an independent country, and there is no need to pursue independence. They want to maintain the status quo, which is the opposite of what Chinese President Xi Jinping wants.

Last year, Hong Kong also witnessed the rise of the millennial generation in protests. A student at the Chinese University of Hong Kong said, “Born in the 1990s when Hong Kong was handed over to China, we are the first generation that belongs to Hong Kong 100 percent,” meaning they were Hong Kongers, not Chinese, who were educated in a free society with its own system independent of China.”

China aims to become a world power by 2049, which marks the 100th anniversary of its founding, and the success of “one country, two systems” is also part of the plan. Its aim is to unite with Taiwan based on the “one country and two systems” principal in Hong Kong and Macao. However, armed with new values, Taiwan’s “born independent” and Hong Kong’s Millennials are posing a threat to China’s plan. It is clear that this anti-Chinese sentiment is not a passing trend, which will only grow over time.

Experts point out that, both in Taiwan and Hong Kong, China failed to recognize the rise of a new generation and took a hardline stance dismissing them as yet another group of protesters, which backfired. This is a reminder that a crisis will ensue if politicians fail to read changing demands of the public.

# **S.-China ‘Phase One’ deal**

The almost-two-year trade war between the U.S. and China has entered a truce with the Phase One agreement. Clouds over the global economy that had contracted and been unstable due to a battle between the world’s two largest economies are gone for now. However, all sensitive issues, including sanctions against China’s Huawei, have been deferred to Phase Two. In addition, a trade war can be resumed at any time in the event of a bumpy implementation of the Phase One agreement, which means uncertainties are still in place. More than anything, South Korea’s exports to China can be substantially affected as China has agreed to import a large volume of goods from the U.S.

The U.S. and China came to an agreement on Wednesday that China will import 200 billion U.S. dollars worth of “Made in USA” products for two years. In return, the U.S. will lower the 15-percent tariffs currently imposed on Chinese products to 7.5 percent and scrap its plan to impose additional tariffs on certain products from December 2019. The agreement also contains China’s commitments to protecting intellectual property rights, prohibiting forceful requests for technology transfer, and no longer deval‎uing the yuan.

An additional 200 billion dollars worth of goods and services to be imported by China from the U.S. are equivalent to 33 percent of South Korea’s total annual exports. China will import not only agricultural products but also industrial products, energy, and services. Unless China’s domestic demand increases as much, its import from other countries is bound to shrink accordingly.

The International Monetary Fund (IMF) analyzed how the U.S.-China trade deal will negatively impact other countries, including South Korea, Japan, and the European Union, in its report titled, “Managed Trade: What Could be Possible Spillover Effects of a Potential Trade Agreement Between the U.S. and China?” The report predicted that assuming China’s total imports to stay the same South Korea’s export can decrease up to 46 billion dollars or three percent of the country’s GDP.

South Korea’s exports to China are likely to be dealt a direct blow without a brief moment of relaxation for the country from the ending of trade tensions between the two powerhouses. As the behind-the-door agreement is to artificially reshuffle the global trade system, its impact on the future economy remains very high. Given that China accounts for 27 percent of South Korea’s total exports, South Korea needs to diversify its export destinations to reduce dependency on trade with China and urgently find niche markets amid the large volume of U.S.-China trade.

# **China test-launches SLBM that can reach U.S.**

While the U.S. continues to apply unprecedentedly heavy sanctions against Iran, China, Russia, and Iran will launch maritime military drills for the first time in the northern Indian Ocean and the Sea of Oman. In addition, China staged an armed protest by test-launching a submarine-launched ballistic missile (SLBM) that is capable of delivering an intercontinental ballistic missile-level nuclear warhead and reaching all over the U.S. The continued cooperation of China and Russia against the U.S. is garnering attention.

According to The Associated Press and the Mehr News Agency of Iran on Wednesday, the joint military drills named the “Maritime Security Belt” wargame among the navy troops of the three countries will be carried out from Friday to next Monday. The location of the wargame is at the entrance of the Persian Gulf and close to the Strait of Hormuz, the world’s biggest crude oil transportation strait. “The objective of the wargame is to strengthen international trade security in the Middle East. Iran, Russia, and China will share security experiences and train against terror attacks and pirates,” said an Iranian military source.

However, many believe that the drills are for China and Russia to support Iran, which is struggling with the U.S.’ withdrawal from the Intermediate-Range Nuclear Forces Treaty and economic sanctions, in opposition to the U.S.

Iran expressed its intention to use the drills as an opportunity to expand military cooperation with China and Russia. “The wargame is part of extensive cooperation with the navy troops of China and Russia, which includes the production of submarines and destroyers,” said Hossein Khanzadi, the commander of the Iranian maritime branch, to the Fars News Agency. Heavy anti-U.S. countries, such as Pakistan, are predicted to join the joint military wargame among China, Russia, and Iran in the future.

# **China-bashing spreads in and outside U.S.**

A lawyer working at a mid-sized law firm in Washington is seeing a noticeable increase in Chinese clients recently. Chinese scientists in the U.S. are under surveillance by the U.S. government and experiencing restrictions with unclear reasons. “It seems like intelligence agents are assigned to Chinese scientists one-on-one for close surveillance,” said the lawyer said. “The bad relations between the U.S. and China are bringing more clients for me.”

I was wondering if indeed researchers in the private sector are surveilled to such a degree but the look on the lawyer’s face seemed quite serious. As seen in Huawei’s case, the Trump administration’s activities to check China’s technology hegemony are expanding in all directions. It is also undeniable that China-bashing is a clear direction in Washington’s foreign security.

It was also unusual for the North Atlantic Treaty Organization (NATO), which marked its 70th anniversary this year, to target China in its summit statement in early December. It was based on the grounds that China’s attack on Africa, cyber activities, and growing military power pose new challenges to Europe, but it was rather random for the NATO to target the country on the opposite side of the Earth. The U.S., a NATO member with big influence, seems to be behind such targeting as if the U.S.’ aggressive strategy against China is being exported.

In addition, more and more think tanks are reinforcing their research on China. The Center for Strategic and International Studies newly created four teams to study China by different fields. In December alone, there were a series of seminars on China, such as “China’s rise and global order,” “human rights issues in China and the U.S.’ responses,” and “political, religious, and human rights issues in China.” Yet, it is hard to find Chinese participants at seminar venues. Rather, they are filled with local researchers and media persons, as well as foreign reporters from Hong Kong, Taiwan, South Korea, etc. It seems like the Chinese find it uncomfortable to show up to such venues due to strong China-bashing sentiment.

The tension between the U.S. and China seems to have been eased to a degree as the two reached a phase one trade agreement. However, phase two negotiations involving much trickier stuff, such as intellectual property rights, will be much tougher. The bilateral relation may be in truce now but there is no sign that it will improve going forward.

It has been a long time since China has become a constant, rather than a variable, in the U.S.’ foreign security policy. Opposition from Beijing is growing amid such conflicts and the circumstances in Northeast Asia are becoming more unstable. Considering the issue surrounding North Korea, which made threats about a “new path” from next year, diplomatic functions to be solved between the two powerhouses will become even more complicated. More analysis and research, not only on China but also on the U.S.’ policy and strategy towards China, will be needed in the near future.

# **China’s economy marks lowest quarterly growth in 27 years**

China’s economy grew by 6.0 percent in the third quarter of this year, the lowest quarterly growth rate in 27 years. Analysts say that the contracted Chinese market, the biggest destination of South Korea’s exports and direct investment, is likely to shake the foundation for South Korea’s economic growth.

China’s National Bureau of Statistics said Friday that the country’s gross domestic product (GDP) for the July-September quarter recorded around 24. 687 trillion yuan (about 4,119 trillion won), a 6.0 increase from a year ago. This marks the slowest quarterly growth since records began in 1992 and is a 0.1 percentage point lower than analysts’ earlier expectations. Since logging 7.0 percent in the second quarter of 2015, the growth of the world’s second largest economy has slowed down to around 6 percent for the following four years. Many speculate that the growth rate in the fourth quarter of 2019 will drop further to a range of 5 percent given this year’s growth trends.

The economic slowdown of the world’s most populous nation in the latest quarter is attributed to weakening demands for exports and domestic consumption due to its protracted trade war with the United States and the breakout of African swine fever (ASF). Affected by the trade spat with Washington, Beijing’s exports in September fell by 3.2 percent from a year earlier. In addition, the country’s producer price index (PPI) has declined for three consecutive months, sparking concern about a possible emergence of deflation. The consumer price also rose by three percent last month, with the price of pork soaring by 70 percent as a result of ASF reports. This further discouraged consumption, making a dent in the growth rate. “The (Chinese) government has sought to use tax cuts and a flood of new infrastructure projects to power through the slowdown. The data for the first nine months of the year show that some of those efforts are falling short,” the Financial Times said.

Analysts express concern that the South Korean economy would be dealt a blow from the slowing Chinese economy, as the two countries are closely linked to each other in terms of the manufacturing and finance industries. Private research institutes including the Korea Economic Research Institute analyzed that a 1 percent point decline in China’s growth would bring down the growth of South Korea by 0.5 percentage point. “If the slowdown of the global economy is protracted and the Chinese government’s stimulus policies fail to work, the Chinese economy may continue to shrink next year, negatively affecting the South Korean economy,” said Joo Won, deputy director of Economic Research Department at the Hyundai Research Institute.

# **China's military, Hong Kong protesters confront Sunday night**

The anti-China protesters of Hong Kong had a brief confrontation with the People’s Liberation Army (PLA) troops in Hong Kong on Sunday night. Tensions were escalated as it was the first direct interaction between the PLA and protesters.

On Sunday, hundreds of protesters shone laser lights on the barracks of the PLA in Kowloon. The PLA instantly warned protesters by raising a yellow flag on the roof. On the flag was written a phrase both in Putonghua and English, saying “You’re violating law. You may be prosecuted.” Later on, the PLA issued a voiced warning in Cantonese that the protesters “must take full responsibility for the consequences of their actions.”

The troops monitored protesters with cameras and watched their movements closely. As protesters left the barracks soon, there was no direct conflict between the two sides. The South China Morning Post reported that the Chinese military “issued unprecedented warnings” to Hong Kong protesters. Some express concern that a shooting or a violent suppression by the PLA could nudge the situation into an entirely unpredictable direction.

The conflict over the ban on face masks, which started on Saturday, has been intensifying as well. On Monday, the Hong Kong police brought the first charges under the anti-mask law to an 18-year-old college student and a 38-year-old woman. The two were arrested on early Saturday morning while protesting with masks on. Starting from Tuesday, Hong Kong’s education authorities ordered the heads of middle and high schools across the city to submit the list of students who “wear a mask at school, refuse to attend classes, form a human chain or chant for protest.” On Sunday, the Hong Kong police broke into the premises of the Chinese University of Hong Kong and Hong Kong Baptist University to arrest protesters, and they are reportedly making indiscriminate arrests of random citizens wearing a mask.

The Hong Kong metro operated only 39 lines out of a total 94 on Monday morning. Starting from 6 p.m., the entire lines were shut down for maintenance, turning the territory into a ghost city. Large shopping malls were closed, and major supermarkets reduced working hours. Against this backdrop, many grocery stores were packed with people looking for food and daily necessities and saw their shelves emptying instantly. Some citizens described the scene as “wartime atmosphere.”

# **Patriotism prevails in China on 70th anniversary of foundation**

A military parade marking the 70th anniversary of China’s foundation started at 10 a.m. on last Tuesday at Tiananmen Square in central Beijing. Foreign journalists including this reporter gathered at the Media Center in western Beijing after undergoing the first security inspection at around 4:30 a.m., or about five and a half hours before the parade began. The reporters arrived at Chenmen near Tiananmen Square around 6 a.m. after traveling through a road that was completely empty due to traffic restrictions in the dark. Passing a second security check was mandatory before moving to the forefront of the Square, the site of the military parade.

Reporters from China’s state-run media outlets including the People’s Daily and Xinhua News Agency gathered at the Media Center at 11 p.m. on the previous day. Those reporters arrived at the Tiananmen Square area around 1:30 a.m. on Tuesday. Soldiers who participated in the parade had already gathered at Changan Road, which is linked to Tiananmen Square. They spent awake all night on the street but looked excited.

Chinese citizens this reporter encountered onsite before the parade’s opening also looked exuberant. More than 30,000 spectators, who had been selected from different regions across China and given a chance to witness the historic parade in person, already arrived at the site early in the morning. It was the Chinese people’s dream to watch in person a “strong military of their fatherland” at Tiananmen Square.

Patriotism, in which “unity of the people” is considered to be of utmost importance, is thriving across China. “Me and My Fatherland,” a patriotic movie that was released at cinemas during the National Day holiday (October 1 – 7) across China, has become a box office hit. Nationalism dubbed “great reconstruction of the Chinese people” is buttressing patriotism in the movie. Patriotism per se should not be criticized. However, critics even within China have raised concerns that China is concurrently realizing the heavy responsibility weighing on the country as a superpower.

“If nationalism is mistreated, it can transform into chauvinism. Notably, if a country has history of being invaded and colonized or is in dispute over sovereignty, nationalism could end up doing harm on others (in an unimaginable way),” Professor Wang Yizhou at Beijing University said in an interview. “Good diplomacy should always be built on the basis of good public administration and good society.” He promoted “society of virtue,” whose conditions he says include a society where people are more open and devoid of a sense of belligerence.

# **‘No force can shake China,’ says Xi Jinping**

China showed off its national power on Tuesday by hosting a large-scale military parade that reminded of a weapon exhibition at Tiananmen Square in Beijing to celebrate the 70th anniversary of the People’s Republic of China. “No force can shake China,” emphasized Chinese President Xi Jinping targeting the U.S. He enthusiastically spoke about patriotism, unity, and nationalism by mentioning “one country, two systems” for Hong Kong and peaceful unification for Taiwan.

The military parade began with a salute of 70 guns and the Chinese red flag with five stars to celebrate the 70th anniversary. “China has made remarkable growth for the past 70 years. There is no force that can shake the status of this great nation. No force can stop the Chinese people and the Chinese nation forging ahead,” said President Xi in a Maoist suit who stood between former Presidents Jiang Zemin and Hu Jintao. His speech is interpreted as China’s determination to stand tall against the U.S.’ pressure despite challenges in and outside the country, including U.S.-China trade war, the anti-China demonstration in Hong Kong, and economic slowdown.

“Forging ahead, we must remain committed to the strategy of peaceful reunification, and ’One Country, Two Systems’ to maintain long-term prosperity and stability of Hong Kong and Macao,” the Chinese president also said. He also called for unity to realize the Chinese Dream, which refers to the great prosperity of the Chinese nation, saying, “China’s tomorrow will be even more prosperous.” He ended his speech shouting, “Long live the great People’s Republic of China! Long live the great Communist Party of China! Long live the great Chinese people!”

President Xi inspected 15,000 troops of 59 military units who lined up in Changan Avenue in front of Tiananmen Square. The military parade resembling an exhibition of cutting-edge weaponry was a message to the U.S. in itself. The most eye-catching weapon was the Dongfeng-41, a next-generation intercontinental ballistic missile that can attack Washington D.C. The missile, which was revealed for the first time on Tuesday, has the maximum range of 14,000 kilometers, making it capable of reaching anywhere in the world. It can carry up to 10 nuclear warheads and the margin of error for a target is less than 100 meters.

In addition, Beijing also revealed the Dongfeng-17, which is known to avoid the missile defense system of the U.S. using the hypersonic descent technology, and the Dongfeng-100, a hypersonic missile known to be the killer of aircraft carriers. The J-20, a new stealth fighter comparable to the U.S.’ F-35, and the Z-20, an equal for the U.S.’ military helicopter Black Hawk, were also featured. Chinese media reported that 40 percent of the weapon mobilized for the military parade was revealed for the first time. Chinese state-run media Global Times said it was “a message to the world that China has sufficient and reliable strategic nuclear power to respond to any ‘nuclear blackmail’ from any country.” Some say that China’s imposing demonstration targeted not only the U.S. but also its neighboring countries, including South Korea and Japan.

# **China sets its yuan midpoint weaker Friday**

China’s central bank raised the official midpoint reference for the yuan yet again on Friday, which broke a psychologically important level of 7 per dollar Thursday for the first time in 11 years since May 2008.

The People’s Bank of China set the yuan’s daily midpoint at 7.0136 per dollar, a 0.14 percent increase from Thursday’s rate of 7.0039 yuan per dollar. The yuan’s rate against the greenback has risen for seven consecutive trading days since last Wednesday.

With China apparently weakening its local currency in response to trade pressure, the United States has also revealed another set of sanctions. The U.S. Department of Commerce announced Thursday (local time) the affirmative preliminary determination of the countervailing duty (CVD) investigation of imports of wooden cabinets and vanities from China, The Associated Press reported. Countervailing duty laws protect businesses and workers from the unfair pricing of subsidized imports. The United States imported wooden cabinets and vanities from China worth around 4.41 billion U.S. dollars (about 5.3 trillion won) last year.

The Department of Commerce also said that according to its investigation, Chinese exporters received countervailable subsidies ranging from 10.97 to 229.24 percent and that it will collect cash deposits from importers of wooden cabinets and vanities from China. The U.S. International Trade Commission (ITC) is scheduled to make its final CVD determination on Jan. 30, 2020.

Washington has also held off “on a decision about licenses for U.S. companies to restart business with Huawei Technologies Co.,” according to a report from Bloomberg. U.S. Commerce Secretary Wilbur Ross had said last Tuesday that a decision on American businesses’ requests to resume sales could be made by next week.

# **China views Hong Kong protests as 'color revolution'**

China has characterized the ongoing antigovernment protests in Hong Kong as a "color revolution" for the first time, a reference to uprisings aimed at replacing the government, sparking speculation that an armed intervention could be imminent.

Zhang Xiaoming, director of the Hong Kong and Macao Office of the State Council, made the remarks on Wednesday at a closed-door symposium in Shenzhen, Guangdong Province, which borders with Hong Kong. "The movement about the extradition bill has gone bad, and it has the clear characteristics of a color revolution," Zhang noted. More than 550 people attended the meeting, including Hong Kong deputies to the National People's Congress and members of the Chinese People's Political Consultative Conference.

"Hong Kong is facing the most serious situation since its return to China,” Zhang said. "If the situation in Hong Kong continues to worsen into unrest that the Hong Kong Special Administrative Region government cannot control, the central government will not sit back and do nothing… According to the Basic Law (of Hong Kong), the central authorities have ample methods as well as sufficient strength to promptly settle any possible turmoil," he said.

Xiakedao, the official social media account of the Chinese Communist Party's newspaper Renminribao (People's Daily), which offers its interpretations of the party's intentions, said that this is the first time the Chinese central government used "color revolution" to describe the protests in Hong Kong, adding that it is a "clear signal" from Beijing to the protesters in Hong Kong. Color revolution refers to various movements for a change of government in some countries in the former Soviet Union, Eastern Europe and the Middle East since the 1990s.

The Chinese leadership, led by President Xi Jinping, showed strong wariness about a color revolution early this year. At a meeting with senior law-enforcement officials in January, China's State Councilor and Minister of Public Security Zhao Kezhi urged China's police to "stress the prevention and resistance of 'color revolutions' and firmly fight to protect China's political security" and to "strike back against all kinds of infiltration and subversive activities by hostile foreign forces."

According to Chinese media, Zhang quoted former Chinese leader Deng Xiaoping's remarks about sending Chinese troops to Hong Kong, saying, "Deng was far-sighted." Wang Zhimin, director of the Chinese Liaison Office in Hong Kong, said that the most urgent thing is to put the chaos under control to restore order, asserting that it is now a "battle of life and death" for Hong Kong’s future and "battle to defend Hong Kong."

# **China’s defense white paper makes mention of THAAD deployment in S. Korea**

China has raised issue with the United States’ deployment the THAAD antimissile defense system in South Korea in its defense white paper for the first time. The white paper claimed that the THAAD deployment “gravely damaged” the strategic balance and security interest in the Asia-Pacific region.

China’s defense ministry released the 90-odd-page white paper in question on Wednesday. Since the first issuance in 1988, China has published its defense white paper usually every two years. This year’s white paper, the 10th edition, was released for the first time in four years. The 2015 edition was about 20 pages long, while this year’s edition is much longer, explicitly highlighting Beijing’s claims on various security issues involving the THAAD, the inter-Korean issues, the United States, Japan, Australia and Taiwan.

Nothing that the world’s economic and strategic center is moving toward the Asia-Pacific region, the white paper said that “games” played by great powers brought uncertainties to regional security, accusing the United States of “adding complexity” to the region by strengthening military alliances and expanding military deployment and intervention.

China also expressed its intention to play a leading role in the inter-Korean issues. “Despite positive progress made on the Korean Peninsula, there remain uncertainties,” the white paper said. “China is playing a politically constructive role in troubled regions such as the Korean Peninsula.”

China was also wary about Japan and Australia. “Japan is making brisk military moves by changing its military security policy in order to circumvent the post-war regime. Externally oriented military moves are seen,” the white paper said. It also viewed Australia as emerging security player in the region by strengthening its alliance with the U.S. Regarding the Taiwan issue, the white paper again targeted Washington, noting that Beijing maintains its policy of seeking peaceful reunification and "one country, two systems."

# **Trump, Xi agree to truce in U.S.-China trade war**

U.S. President Donald Trump and Chinese President Xi Jinping reached the second truce in which they agreed to resume trade negotiations and hold off on a new round of tariffs after sitting down with each other for 80 minutes at the G20 Summit held in Osaka, Japan on Saturday.

“We agreed today that we would continue the negotiations,” said Trump after the bilateral meeting with Xi. “We agreed I would not be putting tariffs on 325 billion U.S. dollars that I would have the ability to put on if I wanted.” According to Trump, China has agreed to buy a "tremendous amount" of agricultural products immediately. "We are going to give them lists of things we want them to buy," Trump said. Back on Dec. 1 last year, the two heads of state had agreed to delay an additional tariff increase and negotiate for 90 days at the G20 summit in Buenos Aires, Argentina.

Trump hinted on easing sanctions on Chinese communication company Huawei on Saturday. “U.S. companies can sell their equipment to Huawei. We’re talking about equipment where there’s no great national security problem with it,” the U.S. president said. “We want to have Chinese students [go] to our great schools and great universities.”

Trump and Xi had also an unofficial meeting the previous day, but the bilateral summit exceptionally lasted for 80 minutes on Saturday. "My Plan B is that if we don't make a deal, I will tariff. I am in no hurry, but things look very good,” Trump wrote in a post on Twitter. “The quality of the transaction is far more important to me than speed.” According to the Foreign Ministry of China, Xi emphasized that he would spare no effort to safeguard the benefits of China with regard to China’s sovereignty and dignity.

The G20 Summit in Osaka closed with the “Osaka Declaration,” which emphasized the importance of fair trade. The heads of the G20 member states announced that the global economy was ridden with downside risks and they would take various actions to handle the risks. Opposition to protectionism was excluded from the announcement this time as well due to the opposition of the U.S., following last year’s summit in Argentina.

# **Xi Jinping must not hinder denuclearization efforts**

Speculation is brewing that Chinese President Xi Jinping will meet Kim Jong Un, the young leader of North Korea, before visiting Osaka for the upcoming G20 summit late this month. With the communist regime refusing to have any dialogue since the fiasco in Hanoi, Mr. Xi’s visit could serve as an event to bring about subtle change in the diplomatic landscape in North East Asia.

It is hard to verify the visit before an official announcement as both Beijing and Pyongyang consider secrecy as a diplomatic courtesy. Pundits expected that President Xi would visit Pyongyang in return soon, given that Kim Jong Un has already visited China four times, but it has been put off after U.S.-North Korea summit in Hanoi fell apart.

The speculation over Xi’s upcoming visit came out amid the escalating disputes with Washington over trade and technology and the criticism over interfering in the internal affairs of Taiwan and Hong Kong. Some experts say that Beijing will utilize Pyongyang as an ace up the sleeve and leverage against Washington as it did during the early phase of trade war in 2017. The North Korean card will be both an opportunity for Xi to reaffirm his clout as backer of Kim Jong Un and a solution to present to President Trump during their meeting at G20 as a way to bring about a ceasefire for the on-going trade war.

The North has remained silent despite the repeated requests for dialogue from Seoul and Washington. Even President Moon Jae-in, who has been much accommodating to Kim Jong Un, is urging the North Korean leader to show the will to disarm his nuclear weapons, which reflects Mr. Moon’s frustration and disappointment towards Pyongyang.

President Xi’s visit to North Korea could serve as a turning point for the regime to engage in dialogues again. However, China’s diplomatic tactics towards North Korean must be focused on pressuring the North to disarm the arsenal rather than boasting their ties, which will encourage Kim Jong Un to harbor an ulterior motive. If China steps in to undermine the sanctions of international community, it will only beget more trouble. It is noteworthy that North Korea had to come back to the negotiating table early last year because China participated in the sanctions.

# **If China rules the world**

China has emphasized that it pursues egalitarian multilateralism to counter the West’s hegemony on the international stage, but its actual course of actions is confusing at best. China stresses vertical hierarchical order centered on China as if it is treating neighboring countries as subordinate states during the feudal age. If a neighboring country causes its wrath, it does not hesitate to retaliate against the country, be it big or small, just like a hegemonic country would do in the pre-modernistic era.

Likewise Palau, a tiny Pacific island with a population of some 20,000, suffered damage from China’s retaliatory action. The island country, whose population equals the number of daily newborns in China, formed diplomatic ties with Taiwan. This angered China, which is consistently following "One China" policy.

The Chinese government imposed a ban on group travels to Palau by Chinese tourists by citing lack of diplomatic ties. As the number of Chinese tourists to the island state was halved, Palau Pacific Airways, run by a Taiwanese airliner, shut off its China routes in July. Large Chinse investors who had constructed hotels and bought large buildings on the island also dumped their properties there. Palau, whose service industry including tourism accounts for 80 percent of its economy, was dealt with a massive blow.

Since China's population accounts for 20 percent of the global population, it is completely different from the U.K. or the U.S., which enjoyed supremacy with relatively small populations. China has so large economy of scale and power that it can even weaponize its own tourism industry. In addition, if the Chinese yuan emerges as a key currency like the U.S. dollar, it will be able to threaten the entire economy of a country simply by excluding economic players from China’s financial system, the way the U.S. does with its independent sanctions. What have happened in South Korea, Canada and Palau may herald what will happen when China comes to rule the world.

It is the fate of the Korean Peninsula that neighbors and should co-exist with China, whether we like it or not. If South Korea is to strengthen its China diplomacy, Seoul needs more "optimistic realists" who carefully take into account potential risks while positively perceiving relations with China at the same time, rather than "pro-Chinese people" who are largely biased. Seoul should run a system of mid- to long-term national strategy that will examine whether the government is not excessively biased towards a certain country in the areas of politics, economy, society and culture, and prepare itself accordingly.

Palau learned the weaknesses of its tourism industry only after Chinese tourists accounted for nearly half its foreign tourists. In a bid to counter China’s threat belatedly, Palau started to promote an eco-friendly, high value-added tourism industry in order to attract tourists from Europe and Japan, whose per-capita expenditure is larger than Chinese. For this reason, the island country started to preemptively ban the use of sun cream to protect its coral reefs, following Hawaii’s suit. The Palau government recently said while Chinese tourists have declined in number, it saw tourism revenue increase due to a hike in per-capita expenditure by foreign tourists. The U.S.-China trade war and what has happened in Palau remind us of the lesson in the "Act of War" that the winner and loser of a war are decided even before the war breaks out.

# **Huawei CFO’s arrest could impact U.S.-China trade talks**

Meng Wanzhou, Huawei Technologies' chief financial officer and the daughter of the company's founder, has been arrested in Canada on charges of violating U.S. sanctions against Iran, and her arrest is stirring a ripple effect on the on-going trade talks between Washington and Beijing. Meng’s arrest is fast emerging as a tinder box that could rupture the current talks, after the two countries agreed to a temporary 90-day truce at the summit meeting in Argentina on December 1.

"As far as I'm concerned, it's a hard deadline," said U.S. Trade Representative Robert Lighthizer during his CBS interview on Sunday (local time). "When I talked to the president of the United States, he's not talking about going beyond March.” He stressed that at the end of 90 days, the tariffs will be raised.

When asked if President Donald Trump had known about the arrest during his summit dinner with President Xi Jinping, Lighthizer denied such a possibility, emphasizing that trade talks must not be affected by Meng’s arrest.

However, many experts are of the view that the CFO’s arrest will have a negative impact on the talks between the U.S. and China. “Ms. Meng’s detention has considerably complicated China’s economic relations with the United States,” said the New York Times, adding that it has ignited anger and astonishment in China, where Huawei is a source of national price. “The imbroglio has produced a nationalist backlash in China that could make it difficult for Mr. Xi to make concessions to the U.S.,” reported the Wall Street Journal.

A day after calling in the Canadian envoy stationed in China for protest, the Chinese foreign ministry summoned U.S. Ambassador to China Terry Branstad to have the arrest warrant revoked. “The actions of the U.S. seriously violated the lawful and legitimate rights of the Chinese citizen,” said Vice Foreign Minister Le Yucheng in a statement, adding that Beijing will take additional measures depending on the future actions of Washington.

Canada has also made a response, canceling a trade mission’s trip to China. The province of British Columbia issued a statement that it has suspended the China leg of its Asian forestry trade mission due to the international judicial process relating to Huawei’s chief financial officer.

Pundits say that the intransigence between the world’s largest economies is likely to be prolonged as the economic alliance between the U.S. and Canada is fully aligned, and China is showing no signs of backtracking. There is a possibility that China could detain Canadians as a retaliatory measure, the AP reported.

# **Chinese scholar argues S. Koreans concerned over China’s deception**

“Chen Xiangyang, an associate researcher of Korea Peninsula Program at the China Institutes of Contemporary International Relations, said South Koreans are concerned that China could deceive them. He made the remark at a South Korea-China public diplomacy forum held on Wednesday in Yancheng, Jiangsu Province in China, claiming that South Korea seems to have “low self-confidence” towards China.

Co-hosted by the foreign ministries of the two neighbor countries, the forum took place for two days from Tuesday on full-fledged trust building that would prop up the bilateral ties over the next 10 years, providing an opportunity to peek into inner thoughts of China toward South Korea. South Koreans' resentment against Beijing has significantly escalated since China’s retaliation against the South’s deployment of the U.S. missile defense system called THAAD.

“The THAAD has had an enormous (negative) impact on mutual trust between South Korea and China,” Chen said. “The two sides should not cross the ‘Maginot Line’ of doing damage to each other.” He called on Beijing to “do its best” to emphasize the importance of bilateral cooperation, while urging Seoul to recognize the value of the ties.

Chinese presenters also expressed their perception that a stronger South Korea-U.S. alliance would hurt China’s national interest. “It is not that China wants South Korea to dismantle the alliance but that it is concerned over a stronger South Korea-U.S. alliance doing damage to China,” Chen argued.

“South Korea cannot ease, reduce, or abolish its alliance with the U.S. to shift (the focus of) its diplomatic policy only on its relations with China,” said Lee Ho-chul, head of the Academy of Chinese Studies at South Korea’s Incheon National University. “South Korea and China should maintain their relations on the basis of mutual understanding of such a structural condition that the two countries are facing.”

# **U.S.-China tariffs war truce likely to prove fragile**

“China has agreed to reduce and remove tariffs on cars coming into China from the US. Currently, the tariff is 40 per cent,” U.S. President Donald Trump wrote on Twitter on Sunday. It appears that his latest remarks are aimed at stressing China’s instant concession after it agreed to take a "90-day truce" from its tariff war with the United States during the summit meeting on Saturday.

According to the Financial Times on Monday, China's Vice Premier Liu He will lead his team to visit Washington on December 12 for a follow-up discussion with Washington. However, the Chinese government and state-run news media outlets are withholding the information from their public that "90 days are given as deadline, and if the two parties fail to strike a deal with the timeframe, the U.S. will impose tariffs again." If anything, the Chinese media are emphasizing that China has resolutely safeguarded its core interests in the negotiation with the U.S.

Experts say that the following trade talks will face much trouble as Chinas has effectively declared that it will not make any unilateral concessions in meeting the demands from President Trump. The Financial Times also described the agreement as "fragile tariffs truce," expecting the coming months between Washington and Beijing to be "fraught with pitfalls."

Some pundits point out that the tariffs truce was arranged as the doves have gained ground in Washington. They say the conciliatory influence from Top White House economic advisor Larry Kudlow and Treasury Secretary Steven Mnuchin has grown against the waning clout of Peter Navarro, the director of Trade and Industrial Policy known for his hawkish economic view towards China.

In an article titled "A win for diplomacy at the G20," U.S.-based Internet news site Axios said the result of the agreement was a "score one for the globalists" against hawkish protectionists, adding that "no one thought Donald Trump had prepared for his dinner with Chinese President Xi Jinping, but somehow an agreement was hashed out that calls a 90-day truce to the trade war between the two countries."

# **Beijing’s economic pressure affected DPP’s election loss in Taiwan**

Analysts say that Beijing’s economic pressure was one of the key factors in the landslide defeat of the ruling Democratic Progressive Party (DPP) to the rival Kuomintang (KMT) in the November 24 local elections in Taiwan.

While the number of DPP candidates who were elected in the gubernatorial elections declined from 13 to six, that of KMT candidates who were elected increased from six to 15. DPP also lost the mayoral seat of Kaohsiung, the second largest city and previous DPP stronghold where the party dominated for 20 years, to KMT’s Han Kuo-Yu.

Since Tsai Ing-wen assumed the presidency in Taiwan, China reduced the quota for Chinese tourists bound for Taiwan and imposed export restrictions on certain agricultural and fisheries products. China also strengthened the operation of so-called "red supply chain" through which it self-supplies parts and components that were used to be supplied by Taiwan, dealing a significant blow to the Taiwanese economy that is focused on parts supply by small and medium size companies.

“Since President Tsai’s inauguration, China has reduced the number of group tourists heading to Taiwan by nearly 40 percent as part of its sanctions. Taiwan suffered especially more damage in the tourism industry,” Park Han-jin, chief of the Korea Trade Investment Promotion Agency (KOTRA) Chinese office, said in an interview with The Dong-A Ilbo on Friday.

Beijing also intensified its strategy for Taipei’s diplomatic isolation, as evidenced by the number of countries that have diplomatic ties with Beijing, which has declined from 22 at the time of Tsai’s inauguration, to 17 this year. The biggest reason voters snubbed DPP in the elections was deterioration of people’s livelihoods. As Taiwan’s economic dependency on the Chinese Economy is so dominant, with China accounting for 41.1 percent of Taiwan’s total export last year, Taiwanese voters chose "practical interests over justification" and "stability over cross-strait conflict" in the wake of Beijing’s constant pressure on DPP, analysts say.

When Taiwanese voters elected Tsai after a 10-year rule by former President pro-Beijing Ma Ying-jeou of KMT, watchers already expected a certain degree of cross-strait conflict. The Tsai administration sought to overcome this situation through "New Southern Policy" by increasing exchange with Southeast Asia. However, analysts say that the latest elections have demonstrated that Taipei’s opposition to Beijing brings about severe consequences to the Taiwanese economy.

This has been clearly revealed by the withdrawal of support for DPP by young people, who are a significant part of former President Tsai’s supporters. “Young people who want to land jobs in China account for 69 percent of youth,” said Kang Joon-young, professor of Chinese politics and economy at Hankuk University of Foreign Studies. “As DPP continued to be unfriendly with Beijing amid a high youth unemployment rate in Taiwan, younger supporters chose to dump the party.” The youth unemployment in Taiwan came to 12.29 percent in the third quarter of this year, which is far higher than the overall unemployment rate (3.76 percent).

The number of Taiwanese who are engaged in economic activities in the Chinese continent is estimated at more than 1.5 million. Many of them are executives and employees of electronic and electrical parts and components suppliers. A sizable number of people are also engaged in economic activities in China through ways that are not tallied in economic statistics, including operation of paper companies. Business people who are operating businesses in China are so proactive in politics that they even bother to fly to Taiwan to cast ballot during elections to help KMT win elections.

“Exchange at the city level between China and Taiwan will expand,” said Ma Xiaoguang, the spokesman for Beijing's Taiwan Affairs Office, told a press conference on Wednesday. “Chinese group tourists are heading to Kaohsiung.” He thus indicated that China will seek to pressure the ruling DPP by expanding exchange especially with Taiwanese regions where the opposition party won elections, while concurrently taking reconciliatory measures for pro-Beijing gubernatorial governments.

# **Chinese researcher claims world's first gene-edited babies**

Controversy has been stirred up across China by the news that a scientist, reportedly, succeeded in designing a baby, whose genes are biologically edited. Chinese authorities ordered an investigation on the case. A university, which the scientist in question belongs, has denied any correlation with the controversial testing.

Chinese scientist He Jiankui argued on Monday that he manipulated genes by using gene editing technology so that they can become immune to AIDS. The measure was taken on embryos of seven married couples who received infertility treatment, one of which succeeded in giving birth to twin girls, according to the scientist. Editing genes cut some abnormal parts of genes or insert normal ones. However, it has been globally prohibited that genes in embryos are edited because the edited genes can be passed down to the next generations.

On the same day, 122 Chinese scientists announced their condemnation against Prof. He’s argument on Weibo, a Chinese version Twitter. They explained that gene editing is not a novel technology from a technological view. However, uncertainties still exist and the technology can lead to ethical issues and risks, according to the scientists. They expressed their strong opposition against such efforts to edit embryos’ genes without any strict ethical and safety considerations, citing that manipulated genes can affect the next generations genetically.

The Academic Council at the Southern University of Science and Technology in Shenzhen, where Prof. He belongs, said that he had been on unpaid leave since February. The university said that it had been unaware of the test in question as it was conducted outside the campus.

# **China's sandstorms hit Korea with fine and yellow dust**

Tuesday was indeed a suffocating day in Korea as smog and yellow dust originated in China were added to the accumulated fine dust in the atmosphere. It is the first time this fall yellow dust affected the Korean Peninsula.

According to the Korea Meteorological Administration and the National Institute of Environmental Research, the ultra-fine particle concentration level was "bad" with 48μg per m³ as of 3:00 p.m. Tuesday. An ultra-fine dust watch was issued at 10:00 a.m. and 12:00 p.m. in Gyeonggi Province and Incheon City, respectively. A watch is issued when ultra-fine dust that exceeds 75μg per m³ lasts for more than two hours. As the atmosphere of the Korean Peninsula has stagnated since Friday, fine dust generated within Korea has been accumulated, added with smog from China, making find-dust even more concentrated.

To make matters worse, yellow dust also overlapped with the current atmospheric situation. It has been six months since yellow dust inflowed into Korea after May 25. Part of the yellow dust that was generated near Mongol in China on Monday blew into the Yellow Sea through northwest wind and affected the Korean Peninsula from Tuesday afternoon. Yellow dust that flew into Korea from China were fine dust of which PM diameter is more than 2.5μm but less than 10μm, meaning it has larger particles than ultra-fine dust of which the PM 2.5 diameter is less than 2.5μm.

# **China’s AI ambitions**

During a panel discussion session of the “Samsung Future Tech Forum” held in Beijing on Thursday, Chinese AI maker startup Suiyuan Technology CEO Zhao Lidong frankly talked about the difficulties Chinese AI and semiconductor industries are experiencing due to the trade war between the U.S. and China.

In spite of Zhao’s concerns, the U.S. Department of Commerce on Monday said it is considering curbs on exports of 14 advanced technologies, including artificial intelligence and robotics. It did not mention China but the move is intended to prevent China’s rise in the semiconductor industry. China has taken an action, too. Earlier this month, Chinese President Xi Jinping urged innovation in the AI sector, asking the industry to focus on developing core technologies.

“The AI industry is still in the initial stage both in the U.S. and China. I wish the future will be bright for China,” said Xie Guangjun, vice general manager of Baidu Cloud, during the panel discussion. He viewed that China would hold a dominant position in the field thanks to the Chinese government’s strong support. He was confident that China would not be affected by U.S. pressure as the field is in its nascent stage of development in both countries.

“China will not be able to catch up with the leaders in the computer or semiconductor industry. But when it comes to AI, both China and the U.S. are standing in the start line,” said Zhao Lidong. “AI and semiconductor sounded like building castles in the air for China five years ago. But it is not a dream anymore with the government’s support, investment from the capital market, and collaboration with international companies."

"I think we are far more competitive than China," Choi Cheol, executive vice president and head of the Device Solutions China regional office at Samsung Electronics, said Thursday about China’s rise in the semiconductor industry. “The leader (Samsung Electronics) does not follow the market trend, but it creates a market by itself,” he expressed confidence when talking about China’s plans to mass produce its own memory chips.

On the following day, however, China’s State Administration for Market Regulation (SAMR) said they found “massive evidence” of anti-competitive behavior by the world’s top three chip makers, Samsung Electronics, SK Hynix, and Micron, suggesting sanctions against them.

China, which has the largest semiconductor market in the world, is making a move to keep the Korean semiconductor industry in check. While fighting against the U.S.’ export ban on AI, China would make desperate efforts to catch up with Korea in the semiconductor market.

It seemed that Beijing and Chinese tech companies could not wait to see the day when they can say that China has overtaken Samsung in the semiconductor market and it is not a competitor anymore.

# **China's third quarter GDP rises 6.5 percent year-on-year**

China GDP growth rate hit the lowest point since the Great Recession in the third quarter of the year. China’s National Bureau of Statistics said Friday that the country’s GDP expanded 6.5 percent year-on-year in the third quarter of the year. The figure is the lowest growth rate since 6.4 percent that was posted in the first quarter of 2009, when the world was engulfed in the Great Recession. China’s quarterly GDP growth has continued to slide since recording 6.9 percent in the first quarter of 2017. The country’s GDP came to 6.8 percent and 6.7 percent, respectively, in the first and second quarters of this year.

Given that the average GDP growth rate over the past three quarters was 6.7 percent and that this year’s target is 6.5 percent, its GDP growth continues to be stable in general, the Chinese government says. “The national economy has operating on a stable basis overall under the leadership of the Communist Party’s Central Committee led by Comrade Xi Jinping despite complex and grave international politics, while the economic structure is constantly advancing,” the National Bureau of Statistics of China said in a statement on the day. “External uncertainty has grown, but it is expected that we will sufficiently cope with external pressure,” National Bureau of Statistics spokesman Mao Shengyong also told a press meeting.

Since China’s GDP growth has continued to slump, however, its GDP growth rate will take a bigger hit beginning in this year’s fourth quarter, when the ongoing trade war between the U.S. and China will start to take its toll in earnest.

# **Chinese live-streamer detained for insulting national anthem**

A popular Chinese live-streamer has been detained for singing the national anthem not seriously enough. Pundits say that the latest episode reflects the expansion of censorship by Chinese President Xi Jinping into Chinese media outlets as well as the Internet.

The Shanghai Police Department said on Sunday that Yang Kaili, a 20-year-old Chinese live-streamer, has been detained for five days for violating China’s national anthem law. The police department said Yang Kaili broke the national anthem law stipulating that one must stand and refrain from disrespectful acts when the anthem is played, stressing that the arena of the Internet is not immune to such law.

Yang Kaili, who is also known as Li Ge, is a popular live-streamer in China. She is a wanghung, a Chinese expression‎ referring to a major Internet celebrity, with more than 44 million followers on TikTok, a popular streaming app.

The Internet celebrity hummed the national anthem while broadcasting on Huya, a Chinese version of YouTube, to introduce an online music festival on October 7. The singing lasted only for three seconds, but she was quickly under fire for insulting the “dignity of the national anthem.”

Yang issued two apologies on social media, saying that she would like to apologize for singing the national anthem in an unserious manner, and that she was sorry to her motherland as well. But the controversy spread, and Huya decided to shut down Yang Kaili’s broadcasting account. Eventually, Yang faced detention a week after the incident. The Shanghai Police Department said that it would strictly punish acts that challenge the parameters of law and violate the public order and fine custom of the country.

# **China proposes to expand its Belt and Road Initiative**

China has revealed a plan to expand its “Belt and Road Initiative” (intended to interconnect infrastructure corridors and promote economic growth) with Dandong, a city in Liaoning province that borders North Korea, as a gateway.

The province of Liaoning has proposed a plan to the government to “link its city Dandong with the Korean Peninsula” via rail and road as part of its contribution to China’s “Belt and Road Initiative,” according to the official Liaoning Daily. The province has suggested a rail, road, and communications link from the city of Dandong to the North Korean capital Pyongyang and then on to Seoul and Busan in the South, saying that the link would be a direct route to a port in the south, referring to the city of Busan through which it can expand its plan to the Pacific. The province is also pushing for a new road between Dandong and Pyongyang through Sinuiju.

The document also said that the province will further develop a North Korea-China economic zone in Hwanggumpyong Island and the two countries’ border trade zone in Dandong into the important pillars of economic cooperation with the North along with Dandong’s key development zone for experiments. Liaoning is also seeking central government approval at due time to establish a special economic zone in Dandong while increasing flight routes between airports in Shenyang, Dalian, Dandong and North Korea and Russia’s Far Eastern cities.

The document also included a plan to support the cross-border trade zone in Dandong as an online e-commerce platform for the two countries. “Guided by the important consensus by the leaders of China and North Korea, (we) steadily make plans of cooperation with North Korea,” said the provincial government, indicating that the initiative’s expansion into the Korean Peninsula had been agreed upon by President Xi Jinping and North Korean leader Kim Jong Un at previous three rounds of bilateral summits.

Beijing’s such plan is pushed alongside the establishment of “Northeast Asian Economic Corridors,” for which China, South and North Korea, Japan, Russia, and Mongolia work together. The Liaoning province said that it will create a group that shares a common destiny in Northeast Asia by converging economic corridors that connect China, Russia, and Mongolia with a framework called the “China-Japan-South Korea plus X model” so that the six countries can enhance cooperation. This embodies the country’s intention to take the lead in the development of the Northeast Asian region with Liaoning as a hub. The “China-Japan-South Korea plus X model,” first suggested by Chinese Premier Li Keqiang at a trilateral summit in May, calls for free trades with other countries including a free trade agreement between the three countries.

An expanded Belt and Road Initiative of China overlaps with South Korean President Moon Jae-in’s inter-Korean economic policy called “New Economic Map” that plans to connect cities from Seoul to Pyongyang, Sinuiju, and Dandong. “This may be an opportunity for South Korea and China to cooperate on the development of North Korea’s infrastructure, but it is also possible that China may try to absorb South Korea’s policy into its own initiative,” said a diplomatic source. In fact, while Seoul and Busan are included in Beijing’s plan for expansion, the document only emphasizes cooperation with the North while not mentioning any of it with the South.

Some say that U.N. Security Council resolutions on the North Korean regime are likely to be violated in the process of expanding China’s initiative into the Korean Peninsula. “China was unlikely to push forward any infrastructure projects with the North while sanctions were still in place,” said Hong Kong’s South China Morning Post (SCMP).

# **Xi Jinping’s visit to North Korea seems unlikely**

Wang Huning, a member of China’s Politburo Standing Committee and secretary of the party’s Secretariat, is set to travel to Pyongyang as President Xi Jinping’s envoy around Sept. 9 to celebrate North Korea’s 70th founding anniversary, sources said Monday.

Multiple government officials and diplomatic sources said that it would be physically difficult for President Xi to make time for a visit to Pyongyang because “for now, the Chinese government appears focused on a China-Africa cooperation forum” being held in Beijing from Monday to Tuesday. In view of the North Korea-China relationship, the party’s senior official Wang will be sent on behalf of Xi, according to the sources. Some say that the Chinese government had already decided to dispatch Wang around last month considering President Xi’s busy schedule.

Diplomatic sources speculate that in fact, it was U.S. President Trump’s condemnation of and warning against China that stopped Xi from visiting the North. “The issue of the Korean Peninsula is not a policy priority for China as it should deal with a trade spat with the United States as well as the issue of China-Taiwan relations. So, there is no need for President Xi to make a potentially uncomfortable decision in a political sense and risk borrowing trouble,” said Kim Han-kwon from the Korea National Diplomacy Academy.

# **Chinese warplane enters KADIZ again**

A Chinese military aircraft entered South Korea’s Air Defense Identification Zone (KADIZ) without notice on Wednesday, prompting a South Korean fighter jet to make a sortie in response. It has been a month since a Chinese warplane entered the KADIZ on July 27 and marks a fifth unauthorized entry this year.

At around 7:37 a.m., a Chinese military jet, presumably a Y-9 reconnaissance aircraft, first entered the KADIZ from southwest of Ieo Island and flied along the Straits of Korea toward the East Sea, entering and exiting it on and off, according to military authorities. The Chinese military jet moved northwards to Pohang, flying at an altitude of 74 kilometers, and kept northwards to Gangneung, climbing up to an altitude of 96 kilometers. The jet then turned southwards and completely exited the KADIZ at around 11:50 a.m.

The military said it scrambled 10 aircrafts, including F-15K fighter jets, to track and monitor it and sent a warning message. In addition, it warned of any actions that could raise tensions and cause accidental conflicts via a South Korea-China hot line. But the Chinese jet ignored the warning and stayed inside the KADIZ for four hours.

China reportedly said the sortie was an ordinary military operation in international airspace that does not violate international laws. The KADIZ around the Ieo Island overlaps with air defense identification zone (ADIZ) of Japan and China.

The South Korean Ministry of Defense called in the Chinese defense attaché at the Chinese embassy in Seoul earlier in last month right after a Chinese warplane made an unauthorized entry into the KADIZ to lodge a complaint and urged China to prevent similar incident from happening again. It appears Chinese military is constantly entering the KADIZ in order to reconnoiter the Korean Peninsula and show off the ability of its reconnaissance aircraft to fly long distance.

# **China anxious due to Trump's Iran sanctions**

China, which is Iran’s largest trading partner and largest crude oil importer, is in trouble as the United States resumed economic and financial sanctions against Iran on Tuesday. China revealed that it would maintain its economic relationship with Iran despite U.S. sanctions against the partner country. China, however, appears to be nervous as it is uncomfortable in forming another war while it is already suffering conspicuously from the trade war with the United States.

The second sanctions, which block trading Iran’s crude oil from November, will pose additional burden on China. The amount of crude oil China imports from Iran is an average of 650,000 barrels per day, which is worth 15 billion U.S. dollars in market value. Some 7 percent of China's overall imported crude oil is from Iran. That is, one-fourth of Iran’s export. Chinese state run oil companies have reportedly invested billions of dollars to Iran’s major oilfields.

It is not a surprise for China to resist U.S. sanctions against Iran as it violates China’s economic profit. It is, however, complicating for China in terms of sanctions against Iran, unlike fighting back with retaliatory tariffs with the same scale during the course of a trade war. China was able to appeal for joint response of the international society including Europe and other Asian countries under the name of protecting the ideology of free trade, which is against Trump's protectionism.

It is difficult for China to appeal to the international society for help. Moreover, U.S. President Donald Trump wrote on Twitter on Tuesday that anyone who trades with Iran cannot trade with the United States. Chinese corporations that engage in transactions with Iran may become the target of U.S. sanctions. China has also been responding sensitively to such secondary boycott with sanctions imposed on North Korea as well. European corporations begin halting investments in Iran as Trump reimposed economic sanctions on Iran, which shall also be a burden to China.

# **Taiwan slams China after sports event canceled**

At the China-led East Asia Olympic Committee meeting held in Beijing on Tuesday, Committee Chairman Liu Peng insisted on the revocation of Taichung City to host the sports event and asked for a vote of hands to determine the issue. Liu is also serving the deputy chief of the Foreign Commission of Chinese People's Political Consultative Conference.

The meeting was attended by nine commission members: representatives from China, Taiwan, South Korea, North Korea, Japan, Mongolia, Hong Kong, Macao and Chairman Liu Peng. The audience began to stir at Liu’s remark.

However, the vote was carried out and seven voted in favor of cancelling hosting rights. It meant that four years of preparation had gone to waste. The event was scheduled to take place for eight years from August 24 next year. Only Taiwan voted against the proposal and Japan abstained. Taiwanese President Tsai Ing-wen condemned the decision at a Facebook comment: “China is taking advantage of its political power to violently revoke our right host the event. The Taiwanese people will not accept the decision.” Taiwanese Premier William Lai also said that Taiwan would make an official protest to the EAOC.”

# **Chinese people expose fury on social media over the vaccine scandal**

China’s Central Commission for Discipline Inspection said Tuesday that the Jilin Provincial Discipline Inspection Commission would launch an investigation into vaccine manufacturer Changchun Changsheng Biotechnology, which caused the latest vaccine scandal in the country. However, an account of the Xinjiang News on Weibo, a Chinese platform likened to Twitter, was flooded with widespread anger and distrust since the news broke. It has been additionally revealed that more than 250,000 doses of the mandatory diphtheria, pertussis and tetanus (DPT) vaccine manufactured by Changchun Changsheng Biotechnology found defective have been administered to children, and the faulty pertussis vaccine manufactured by another company has been also sold for 140,000 children in the city of Chongqing.

Chinese people have reacted with fury and bluntly exposed their distrust and hostility towards the Chinese authorities, leaving online comments such as “I do not want to hear what officials have to say. I want them to act on,” “Related officials (to corruption scandals) may have a higher position than you (investigators),” “The central government should directly send officials to investigate. The provincial leaders are corrupt,” and “Several days have passed since the incident broke out. Public opinions cannot be dismissed, and they should be held responsible.”

Chinese President Xi Jinping, who is currently on trips to Africa, as well as Premier Li Keqiang have ordered a thorough investigation into the scandal, but the public’s anger is also pointing to the country’s Communist Party and the government. It is noteworthy that social media have ironically served as a key channel for people to express their frustration towards the government while the authorities have been working to delete anti-government online comments.

Meanwhile, Xu Jinghe, deputy director of China’s Food and Drug Administration, has also come under fire due to his choice of clothes for an interview with the main evening news program of China’s state-run CCTV. At the interview aired Sunday, he appeared in a blue T-shirt of Burberry, one of the UK’s luxury brands, which is estimated to be priced at 3,200 yuan (around 530,000 won). Online message boards have been again inundated with sarcastic comments such as “All are seriously rotten from top to bottom” and “That may be the cheapest shirt he owns.” “The fact that a public official who does not have a high monthly salary takes part in an interview of a state-run media wearing a luxurious Burberry shirt itself is disgraceful,” a Beijing citizen pointed out.

# **E.U., China to discuss to reshape economic ties in Beijing**

The 20th E.U.-China summit will be held in Beijing on Monday and is expected to become the sweetest-ever honeymoon in their relationship. Economic issue will be one of key agendas given that the European Union and China alike are struggling in a trade war with the United States. It is expected that the two sides will put mutual emphasis on multilateralism and free trade as their common ground in their opposition to the Trump administration’s policy direction of isolationism and protectionism.

During the scheduled summit with its European counterpart, the Chinese government seemingly hopes the current U.S.-China trade war will grow into a broader face-off between the United States and the rest of the world. Last week, it was mainly a symbolic gesture that the Chinese government sent Liu Xia, the widow of the Chinese human rights activist Liu Xiaobo, to Germany upon its strong requests, in an attempt to win over the hearts of the West.

Such a move from the Chinese side could bring a golden opportunity for the European Union during this summit. Even in the trade war with the United States, the European Union still has many more cards up their sleeves than China does. One of E.U. strategies is to secure some major concessions from China regarding a series of long-standing issues, about which the European Union has complained, such as China’s market entry restriction and infringement of intellectual property.

The European Union commented that the two sides would expand their strategic relationship and mainly discuss trade and investment.

# **S.-China trade war in flames**

Chinese Vice President Wang Qishan who is thought of as a person to solve conflicts between the United States and China has not responded to the U.S.-China trade war, garnering much attention of the public as a result.

Some projected that Wang would visit Washington to negotiate, but many Chinese experts say, “The possibility of him visiting the United States is low because he doesn’t have reasons to get involved in the negotiation currently headed by Chinese Vice Premier Liu He.”

Wang who made a huge comeback at the National People’s Congress in March was expected to put off the fire between the two countries, which makes some to suspect that he has a reason not to get involved in the trade war. He has vast experiences in negotiating with the United States such as leading the U.S.-China Strategic Economic Dialogue while he served as a vice minister from 2009 to 2012, and also established a broad network in the United States. He had a series of private meetings with U.S. ambassador to China Terry Branstad and former White House advisor Steve Bannon before he was appointed as vice president. Even after the appointment, he reportedly had many more private meetings with American businessmen.

But he is nowhere to be seen in the imminent trade war, as reported by Voice of America. Reuters reported that Beijing appeared to be reluctant for Wang to get involved in the article “China’s ‘firefighter’ vice president avoids flames of U.S. trade war.”

“The United States and China will come to the negotiation table one day, but the trade war is likely to be prolonged,” said a diplomacy expert. “Beijing would face a dilemma if the trade war slows down China’s economic growth and people’s lives are affected by it.”

# **Beijing counterattack begins by banning Micron’s chip sales in China**

China has banned Micron from selling its chips in China amid escalating trade tension between China and the United States. The ban came before the United States is set to impose tariffs on Chinese imported products Saturday.

The Fuzhou Intermediate People’s Court on Monday ordered Micron to temporarily cease selling its chip products in China, according to Bloomburg. The order covers Micron's 26 chip products, including DRAM and NAND flash

memory chips. The U.S.-based chip maker earned more than half of its revenue from China last year.

The injuction order by a Chinese court was revealed by Taiwan-based chip maker and Micron's competition UMC. UMC, which is constructing DRAM production facility in partnership with China's state enterprise Fujian Jinhua, has been in a legal battle with Micron since last year over patent and trade secret infringement. In December last year, Micron filed a lawsuit against UMC in the District Court for the Northern District of California, alleging infringement on its DRAM patents and intellectual property rights. In response, UMC filed patent infringement lawsuits against Micron with the mainland China courts in January this year.

Some say the sales ban decision made in the largest chip market in the world suggests that the trade war between the United States and China has intensified in the IT field. Bloomberge reported that the United States earlier imposed sanctions against Chinese tech companies, including Huawei, ZTE and China Mobile, and U.S. mobile chipmaker Qualcomm had difficulty acquiring Dutch semiconductor company NXP as Chinese regulators delayed the approval of acquisition.

# **China becoming a hole in North Korea sanctions**

1. S. President Donald Trump disapproved China’s move to ease sanctions against North Korea Monday (local time) on Twitter. “The word is that recently the Border [between North Korea and China] has become much more porous and more has been filtering in,” he said. “I want this to happen, and North Korea to be VERY successful, but only after signing!” He warns that China’s border control should be tighter at least until North Korea agrees to denuclearize. Trump mentioned Kim Jong Un’s second visit to China and suspected Chinese President Xi Jinping was behind North Korea’s threat to reconsider the summit with the United States.

   China has been long misunderstood as a “hole” in North Korea sanctions and thus an obstacle of North Korea’s denuclearization. As a result of China participating in the “maximum pressure and sanctions” against North Korea of the Trump administration, however, China should take the credit for bringing North Korea to the negotiation table. China has recently been assuaging Pyongyang by increasing North Korea-China exchanges. This would also be related to the suspension of the U.S.-China working level discussions this year, which have been reviewing progresses made in the United Nation’s sanctions against North Korea. Pyongyang suddenly took a hardline stance as its relations with China thawed, and threatened to cancel the summit with the United States. China would have cajoled the North to engage in brinkmanship or at least is doing nothing about Pyongyang’s brinkmanship tactic.

   Another possibility is that China would have advised Kim to strain relations with the South by cancelling the South-North high-level summit, refusing to admit South Korean journalists at the closing of its nuclear test site, and raising an issue of the South Korea-U.S. joint military drills. China is even more sensitive than North Korea about U.S. Forces in Korea or forward deployment of U.S. forces such as unfolding strategic military assets because it looks at issues on the Korean Peninsula from the framework of the hegemony race with the United States in Northeast Asia. Thus, China could be trying to put pressure and conciliate North Korea to recover its influence during the process of complete denuclearization, a nuclear-free Korean Peninsula.

   South Korean President Moon Jae-in and U.S. President Donald Trump met this morning in Washington D.C. to discuss measures to prevent North Korea from discontinuing denuclearization. North Korea’s sudden change in posture has raised not only U.S.-Sino distrust regarding North Korean sanctions but also doubt regarding South Korea-U.S. cooperative efforts. North Korea could wrongly assume that alienating measures were fruitful. But as U.S. Vice President Mike Spence warned, if Kim believes he can play Trump, it is a miscalculation. Unless Kim delivers on his promise of “complete denuclearization,” China will not be able to compensate North Korea in any form or shape. China must refrain from encouraging North Korea from taking an alternative path or taking any action that can raise suspicions.

# **U.S., China agree to abandon trade war**

Beijing and Washington have agreed to substantially reduce America’s massive trade deficit with China and stop tit-for-tat trade war. Beijing pledged to purchase more American goods including agricultural products and strengthen cooperation on protecting intellectual property.

The United States and China issued a joint statement after delegations led by U.S. Treasury Secretary Steven Mnuchin and Chinese Vice-Premier Liu He had the second round of talks last week. The two sides agreed to reduce America’s trade deficit with china which hit 372.5 billion dollars last year and examine institutional measures to prevent China’s infringement on U.S. intellectual property rights.

"There was a consensus on taking effective measures to substantially reduce the United States trade deficit in goods with China," the joint statement said. "To this end, China will significantly increase purchases of U.S. goods and services." Agricultural products, which are mainly produced in areas of Trump’s strong support base, automotive and energy products, are included in the list of items for increasing U.S. exports. Semiconductors and aircrafts were not included, although they were discussed. The statement provided no dollar amounts on how much China would increase its purchase of American products. The United States reportedly offered to specify the amount of 200 billion dollars, but China is known to have refused to do so.

However, economists say the agreement would not practically help reduce America’s trade deficit, citing that the multi-billion-dollar trade imbalance stems from the different economic structure with the United States being the world’s largest consumer and China being the world’s factory on the strength of its cheap labor costs. There is skepticism about how much the imbalance will be reduced by merely increasing China’s purchase of American goods such as agricultural and energy products. According to The New York Times, there could be 5 billion-dollar increase in soybeans and 9 billion-dollar increase in liquefied natural gas, oil and coal. However, even if the advanced technological and defense industrial products are included, it would add up to only a small fraction of the 200 billion-dollar decrease total, and these are unrealistic numbers.

# **China puts its first natively-built air carrier to sea**

The Shandong, China’s first domestically built aircraft carrier and the country’s second aircraft carrier, reportedly sailed Tuesday on a sea trial in Dalian, Liaoning Province. It appears the leaders of China and North Korea watched the historic sea trial together as their private jets were spotted at the Dalian Airport. Earlier, the Liaoning Maritime Safety Administration announced a ban on all ship entry saying there will be a military mission taking place in the Bohai sea and northern part of the Yellow Sea.

Two aircraft carriersㅡLiaoning and Shandongㅡmade it possible for China to conduct military operations at places thousands of kilometers away from its mainland coastline. Also, it acquired the capability to pursue strategic interest in the face of the United States. The possession of two aircraft carriers will be an important milestone for the Chinese Navy, which focused more on offshore defense, in becoming a Navy capable of conducting operations out in the open ocean beyond offshore areas. China plans to increase the number of aircraft carriers to six.

China put their first 55,000-ton aircraft carrier, the Liaoning, which was purchased as an unfinished hulk from Ukraine, into active service in 2012. China could build the 70,000-ton Shandong, which has bigger full load displacement, based on the technologies accumulated from building Liaoning. The Shandong, which was built on modular assembly technology, started its construction in November 2013 and was first launched in April 2018. It will be put into active service in 2019. Chinese aircraft carriers are named after the name of the province adjacent to the sea. The next aircraft carrier is likely to be named Jiangsu.

The Shandong is equipped with four large antennas and an S-band radar that can detect dozens of maritime or aerial targets and is carrying dozens of homegrown short and mid-range missiles.

China will have a true ocean navy once it owns nuclear aircraft carriers as well as three to four more aircraft carriers. Then, there will likely be more intervention by China in various international issues.

# **Chinese foreign minister likely to request a 4-nation summit**

Chinese State Councilor and Foreign Minister Wang Yi arrived in Pyongyang Wednesday morning. Mr. Wang, who will be staying in Pyongyang one more day, is expected to have a meeting with North Korean Foreign Minister Ri Young Ho and have a face-to-face talk with North Korean leader Kim Jong Un.

The leaders of the two Koreas announced last Friday at Panmunjom Declaration to “push forward a 3-nation (South Korea, North Korea and the United States) or 4-nation (South Korea, North Korea, the United States and China) summit” in order to build a peace regime.

“Mr. Wang urgently visited North Korea to avoid China ‘passing’ (excluding),” analyzed the Chinese version of Voice of America (VOA). In other words, this was part of China’s diplomacy of active participation to send the message that China should not be left out in peace treaty negotiations, which are directly related to China’s interest with the Korean Peninsula such as the withdrawal of USFK.

Chinese Foreign Minister’s visit to North Korea is the first time since July 2007. Attention is being paid to how Chinese President Xi Jinping’s visit to North Korea will be adjusted by Wang Yi and the North Korean leadership. “President Xi’s visit to North Korea will commonsensically not take place before North Korea–U.S. summit,” said the Chinese diplomatic authorities. There are also views that President Xi may visit South Korea following his visit to North Korea.

# **MeToo movement spreads in China**

The MeToo movement is spreading across prestigious universities in China in spite of government authorities’ censorship. At Beijing University, students called for disclosure of data related to previous sexual harassment events, which was denied by the school. But the issue has escalated, with students putting up hand-written posters supporting the disclosure. Students at Renmin University protested against a professor suspected of sexual harassment. The students’ organized movement is seen as the first of its kind since the Tiananmen Square protest.

Weishin, a student at Beijing University, uploaded on Monday a posting criticizing the school’s action on his WeChat account. On April 8, eight students including Weishin called for the disclosure of information regarding the suicide of Kaoyen, who took her own life after suffering from sexual assault by Professor Shunyang in 1998. The school, however, threatened that such behavior would “affect graduation” and replied on last Friday that “no information was available.”

Early morning on Monday, a school faculty member and Weishin’s mother broke into his room. The faculty member demanded that Weishin should “discard all data stored on his mobile phone and computer demanding information disclosure of the event and write a pledge promising not to intervene in the future.” According to Weishin, the school informed Weishin’s mother of distorted information and pressured her to take him home and lock him up. The Financial Times reported that other students that joined Weishin were also suffering from threats and surveillance.

A poster titled “We support warrior Weishin” was posted Monday night at Beijing University. However, China forbids such postings publicly. The author of the poster said that BBC Chinese website reported that school security guards tore down the posters and Beijing University was barred from the search engine for some time. Photos of Weishin’s letter and posters continue to upload on websites, despite government censoring.

Last week, some 40 students at Renmin University held a protest in front of the classroom of an Economics professor suspected of harassing a female student, demanding that the professor clarify the case. Security guards blocked students from entering classrooms and escorted the professor away. The Financial Times reported that the professor was eventually fired. “I’ve never seen a protest before on school grounds,” said a student who informed of the event. There are rumors that a teaching assistant at Tsinghua University sexually assaulted a student, but the school remains silent.

# **Chinese media: ‘Fight trade war with U.S. the same way as Korean War’**

In an editorial on Sunday, the Global Times, the sister paper of the ruling Communist Party's official People's Daily, said, “A strategic resolution is being established in China, which is to fight the Trump administration's trade aggression in the same way the country fought U.S. troops during the Korean War (1950-53). We should fight today's trade war with the same strategic spirit that fears no sacrifice or loss, and force the United States to burn the very stick that it wields at China.”

“China's participation in the Korea War was due to U.S. military's approach to the Yalu River, which is the Chinese border. Today's trade war is also due to U..S acts that are hurting China's core interests,” the newspaper added. “A sense of crisis that we have no place to retreat is uniting the Chinese society. We know there will be sacrifices but we understand better that there is no limit to the greed of hegemony. China has enough ammunition to fight this trade war.”

The U.S. ninth aircraft carrier battalion combat team, which is headed by the flagship USS Theodore Roosevelt (CVN 71) and comprised of the guide-missile destroyer USS Halsey (DDG 97), and the cruiser USS Bunker Hill (CG 52), has been conducting a joint military drill with the Singaporean navy in southern waters of the South China Sea since Friday.

The Liaoning, the only Chinese aircraft carrier that has been put to combat mission, is conducting a large-scale military exercise with more than 40 battleships and submarines, and 12 H-6K strategic bombers in Hainan waters in the South China Sea from Thursday. Watchers raise concern that if the USS Theodore conducts “freedom of navigation” operations by approaching waters that China claims sovereignty, U.S. and Chinese aircraft carriers could get into their first ever standoff.

# **China retaliates with new levies on U.S. products**

The United States slapped tariffs of 25 percent on 1,300 Chinese imported goods targeting the country’s high-tech industries. It stood firm to its hawkish stance notwithstanding China’s retaliatory tariffs on U.S. pork and agricultural products.

The U.S. Trade Representative (USTR) announced on Tuesday (local time) 1,300 items of Chinese imports worth 50 billion U.S. dollars (some 52.86 trillion won) that would be subject to tariffs of 25 percent. The 58 page list of tariff items announced by the USTR included high tech products such as semiconductors, telecommunication equipment, lithium batteries and various other products such as heavy equipment, steel, metal, aluminum products, power generators, motorcycles, aircraft parts, weapons and medical instruments. In particular, several items under the Made in China 2025 industrial development program are to be imposed higher rates.

“China has supported and executed unlawful network infringement and technology theft that have allowed access of sensitive commercial information and confidential trade information of U.S. businesses,” the USTR said. “The tariff measure is appropriate considering China’s damage on the US economy and eliminating China’s harmful policy, procedures and acts.” U.S. President Donald Trump criticized China as the “leader of trade deficit.”

China immediately resisted to the USTR’s announcement, and the Chinese Ministry of Commerce issued a spokesperson statement an hour later, warning, “China stands resolutely opposed and shall impose measures equally strong and similar in scale on U.S. products in accordance with laws.”

Since Monday, China has imposed high tariffs on U.S. imports worth three billion dollars (about 3.17 trillion won) including pork, in retaliation to U.S. measures to impose tariffs on Chinese steel and aluminum products.

# **China’s VIP security raises speculation of Kim Jong Un visit**

Speculation about a visit to Beijing by North Korean leader Kim Jong Un or another high-level Pyongyang official was running high Tuesday amid talk of preparations for a North Korea-U.S. meeting.

North Korea’s senior leadership, who must be Kim Jong Un or his sister Kim Jong Un, has wrapped up an unprecedented whirlwind trip to the Chinese capital Monday while receiving Chinese authorities’ tight levels of security. The senior leadership reportedly visited Zhongguancun in Beijing, which is known as "China's Silicon Valley" and the favored venue of Kim’s father, Kim Jong Il, during his visits to Beijing.

The roads around the Diaoyutai State Guesthouse, where the North Korean leadership stayed, were closed amid a spate of road closures and tight security measures. A large security presence remained until Tuesday morning when the distinctive green train carrying the North Korean leadership set off on its return journey to Pyongyang.

Along with a cordon a couple of hundred meters away, a convoy of vehicles accompanied by an escort of police on motorbikes left the compound at around 9.30 a.m. on Tuesday. Residents living near one of the most heavily guarded buildings in Beijing reported unusually tight levels of security, and locals and foreign tourists complained about a spate of unexpected road closures.

There are rumors claiming that from 12:30 p.m. on Tuesday, the motorcade was once again spotted on the streets of Beijing passing by Tiananmen Square apparently en route to the railway station where the train that arrived on Monday night was waiting.

# **China should support a peaceful Korean Peninsula**

Chinese President Xi Jinping welcomed and expressed support for the inter-Korean and the North Korea-U.S. summits on Monday in a meeting with South Korean President Moon Jae-in’s top security advisor Chung Eui-yong, who led the special envoys to North Korea, hoping that it could make a good result. Chung also had luncheon and dinner meetings with China’s top foreign policy adviser Yang Jiechi and Foreign Minister Wang Yi, respectively. It is unprecedented that Xi and China’s top diplomatic officers made time to attend the briefing by the special envoy given that the Chinese People's Political Consultative Conference and National People's Congress are currently taking place. This shows the Chinese leadership is paying keen attention on the developments surrounding the Korean Peninsula while raising concerns over China being excluded from the historical diplomatic breakthrough.

Beijing should feel uncomfortable although they welcomed the decision made by the U.S., South and North Korean leaders. Until now, it has arranged North Korea-U.S. dialogues and chaired the six-party talks, playing an active role of mediator. But this time around, U.S. President Donald Trump and North Korean leader Kim Jong Un have agreed to hold a meeting after South Korean President Moon’s delegation delivered the leaders’ direct message, for the first time in history. Not a single summit between Pyongyang and Beijing had been taken place since Kim Jong Un took power. Let alone restoring blood alliance, China has to do something to exert greater influence while seeking to shape the geopolitical order on the peninsula.

China has long argued the denuclearization would be achieved only by ceasing both South Korea-U.S. joint military drills and nuclear or missile provocations by the North. But Kim Jong Un pledged that the regime would refrain from any further nuclear or missile tests, not demanding a cessation of joint military exercise between South Korea and the United States. It was China’s faithful implementation of the sanctions that pushed North Korea to the negotiating table. China adding pressure on the North with the stricter sanctions and their effective implementation has led Kim Jong Un to invite Trump for talks. If the unprecedented meeting between Kim and Trump takes place and if diplomatic developments occur, the Chinese government may have no other option but to completely change its North Korea policy, which aims to have its neighboring ally as a strategic buffer zone in vying for geopolitical power with the United States.

“We are trying to achieve what the world has failed to do so far,” South Korean President Moon Jae-in said Monday. “If we succeed, there will be dramatic changes in world history and the Republic of Korea will have played the leading role.” Any developments on the North Korea-U.S. relations are bound to transform the geopolitical order not only on the Korean Peninsula but also in Northeast Asia. Thus, it is natural Beijing keeps its eyes wide open to changes of the situation. Nevertheless, China, the one and only ally that North Korea relies on economically and politically, cannot be replaced by the United States.

Three principles in China’s strategy for achieving North Korean denuclearization include promoting denuclearization of the Korean Peninsula, protecting peace and stability, seeking dialogues and negotiations. China has amended its constitution to abolish the two-term limit for the country's president, allowing Xi Jinping to lead for life. China’s foreign policy – so-called “great power diplomacy” that is to be carried out in earnest, will be put under scrutiny. The first testing stand is to see if Beijing will be able to establish peace and order in the region through denuclearization. China does not view itself as a guardian of the reclusive regime any longer. As a world power and a responsible member of the international community, it needs to take the leading role in reshaping the order in Northeast Asia.

# **China’s response to the announcement of Kim-Trump meeting**

China officially welcomed the announcement that U.S. President Donald Trump and North Korean leader Kim Jong Un planned to hold a meeting by May on Friday at the Foreign Ministry’s regular press briefing. Chinese state-run media have also expressed surprise, reporting break news about the would-be first meeting between the two leaders.

The People’s Daily website, which tends to refrain from using provocative wording in its reports, titled its breaking news “Big event! Trump agrees to hold a meeting with Kim Jong Un by May,” using a word it may not usually choose for a title. The website also pointed out in another article that although it would be hard and tough to diplomatically resolve North Korea’s nuclear issue, negotiation may allay concerns of a possible war breaking out. The Xinhua News Agency, China’s official state mouthpiece, also reported the announcement of the North Korea-U.S. meeting with a title “Significant change! Trump meets with Kim Jong Un by May.” “President Trump said that great progress had been made but there would be no prospect of lifting sanctions until a deal was reached,” it also said in another report.

Diplomatic sources say that Beijing, while welcoming the news, would also have concerns about it being possibly bypassed by Washington and Pyongyang in future negotiations on denuclearization, despite its support for the international community’s economic sanctions at the cost of the bilateral relations with North Korea. This leads some experts to speculate that China will make efforts to improve its relationship with North Korea, keeping a keen eye on how the inter-Korean and North Korea-U.S. summit meetings play out.

China’s Foreign Ministry, as if conscious of the speculation that it may be excluded in the process of future negotiations, has repeatedly stressed that it has played an active and constructive role in addressing issues on the Korean Peninsula and has been recognized by the international community. This can be seen as Beijing’s efforts to dispel such speculation by arguing that the current dialogue is based on the direction continuously pushed forwarded by the Chinese government.

# **China expands defense budget by 8% for 2018**

Wang Qishan, the former Chinese Secretary of the Central Commission for Discipline Inspection who remained reclusive since his retirement following the 19th National Congress of the Communist Party of China in October last year, made a public appearance on Monday, a first since the congress. A right-hand man of Chinese President Xi Jinping and highly probable vice president of China, Wang Qishan showed up at the opening ceremony of the National People’s Congress (NPC) in Beijing, along with the seven members of China’s politburo standing committee including President Xi and Premier Li Keqiang.

The NPC gathering on Monday presented a target for this year’s economic growth at 6.5 percent, the same as the previous year. And the national budget has expanded by 8.1 percent compared to last year. This year, China’s defense budget for 2018 stands at 1.1069 trillion yuan (174.6 billion U.S. dollars), an all-time high. The country’s defense budget was on the decline, with the increase rates of the defense budget recorded at 7.6 percent in 2016 and 7 percent in 2017, respectively.

While it is far from the two-digit increase rates witnessed before 2016, the modest surge in China’s defense budget this year reflects the country’s plan to engage in a competition for military hegemony against the United States in line with President Xi’s announcement at the 19th national congress to make his country No. 1 military powerhouse by 2050.

Citing threats to its national security, China is recently expanding military spending by building nuclear-powered aircraft carriers.

“China claims to resolve trade conflicts through fair negotiations. We must oppose protectionism while resolutely upholding our legitimate rights and interests,” said Premier Li, in an apparent reference to a trade war with the United States.

# **Xi Jinping abolishes the two-term limit on the presidency**

The Communist Party of China on Sunday proposed to remove a constitution clause limiting presidential service to just two terms (10 years at maximum) in office, inflaming public opinion in the country. Many Chinese are now concerned that the country could go back to the days when Mao Zedong had indefinite grip on power.

Chinese Academy of Social Sciences Professor and prominent sociologist Li Yinhe wrote in her blog that “Recovering the lifelong control is a regression of history. It is returning China to the days of Ma Zedong.” Li Datong, who is former editor-in-chief of a four-page weekly supplement of China Youth Daily, sent an official letter to 55 representatives of the people, including Beijing Mayor Chen Jining, appealing to vote against the Communist Party’s proposal when they attend the National People’s Congress scheduled to start next month. Wang Ying, a female entrepreneur, issued a statement saying, “The removal of presidential term limits is a betrayal and going against the times. I will not keep silent no matter how they require me to.” Tiananmen protest student leader Wang Dan, who exiled from China and now lives in the United States, said in his statement released with 100 Chinese scholars that “It has been revealed that Xi Jinping has had the ambition of becoming an emperor.”

The Asahi Shimbun of Japan reported Tuesday that Xi Jinping met with a strong opposition from former Chinese President Jiang Zemin in October last year when he suggested scrapping the presidential term limits right after the 19th National People’s Congress.

Sensitive words, such as “disagree,” “emigrate” and “to board a plane,” have become the subject to censorship at China's Twitter-like social network Weibo. This is because the number of the word “emigrate” being searched on the Internet has skyrocketed following the announcement of a list of proposed amendments to China’s constitution and the phrase “to board a plane” and “to ascend the throne” are homophonous. Words including lifelong control, ascension, long-term seizure of power and opposition to constitutional reform are blocked from Sina Weibo search results.

“It, along with at least 13 other internet news companies, had received edicts from authorities to prioritize articles supportive of the proposed constitutional change,” The Financial Times quoted an unnamed source at Baidu.

# **China uses South China Sea islands as military control center**

The U.S. Center for Strategic and International Studies confirmed that China recently deployed communications equipment such as transmitting tower, and high frequency radar equipment in the northwest part of Fiery Cross Reef in Spratly Island of the South China Sea, The Hong Kong South China Morning Post said on Sunday. Some 3,000-meter-long landing strip, not to mention a hangar for aerial tanker and air carriers, were deployed on the 100,000 square meter reef. The United States believe that China is creating seven reefs including not only the Fiery Cross Reef but also the Subi Reef and Mischief Reef as an artificial island for military facility. “The Fiery Cross facility may be used as a mediation base for communication among military facilities created by China,” the newspaper reported by citing military experts for the China region.

As such, tensions are rising between the United States and China. Harry Harris, United States Navy Admiral, who has been designated as the U.S. ambassador to Australia, expressed concerns over militarization of the Spratly Islands during the House Armed Services Committee Hearing on Wednesday, saying, “China is attempting to assert de facto sovereignty over disputed maritime features by further militarizing its man-made bases.”

# **China deploys Russian surface-to-air missile system**

China’s recent deployment of Russian S-400 surface-to-air missile system is drawing attention about China’s motive. The S-400 is often compared to the U.S.'s Terminal High Altitude Air Defense system. China has not confirmed where the defense system is located. However, if the S-400 is deployed anywhere in the Shandong Province, the Korean Peninsula will be within the radar surveillance range. In this context, it has been speculated that China aims to check moving of South Korean and U.S. forces in time of emergency in the Korean Peninsula.

According to Russia’s TASS and Hong Kong’s Ming Pao on Friday, Russia has recently delivered the S-400 surface-to-air missile system to China. The S-400 system includes missile control station, radar station and fueling installations. China and Russia signed a contract in 2014 to ship three sets of defense system, and the first system has been deployed this time. China plans to complete deployment of all three systems by 2019.

The S-400 system has a maximum range of 400 kilometers and a maximum altitude of 185 kilometers. Its radar can track up to 300 targets within 700 kilometers. It can shoot down multiple fighters and missiles flying at different ranges and altitudes simultaneously. The system is operable, while electric devices are disturbed. The S-400 has been eval‎uated that it can shoot down U.S.’s latest fighters such as the F-35 stealth and more threatening than the THAAD with a maximum range of 200 kilometers and a maximum altitude of 150 kilometers.

Ming Pao reported that, if the S-400 is located in the southern coast of mainland China, such as the Fujian Province, the entire Taiwan falls in the system’s range and China can attack Taiwanese air force. This argument is based on a recent development in China where unification by force is taking a foothold.

In contrast, it has been suggested that China has imported the S-400 in order to destabilize U.S.’s latest fighter and stealth cruise missile. Russia also deployed the S-400 in far eastern areas near North Korean borders such as Vladivostok.

# **China removes historic border round Shenzhen economic zone**

The wall that has been restricting the movement of people and materials by separating the 1st special economic zone and the rest of the mainland areas of Shenzhen, which represents the Chinese economic reform, has vanished into the history after 36 years since its designation. It is eval‎uated as a symbolic incident, which proves China’s economy has grown so much that it no longer needs walls.

Partial region in southern Shenzhen neighboring the border line with Hong Kong was designated as China’s first special economic zone in 1980, two years after late Chinese leader Deng Xiaoping announced the Chinese economic reform in 1978. Shenzhen was once a small fishermen’s village with about 30,000 population, but it has now grown into the Silicon Valley of China, in which the world-class information technology (IT) companies have gathered up.

China guaranteed economic activities of foreigners, such as free investment, establishment of factories and tax exemption, in the Shenzhen economic zone. But Chinese people outside of the zone could only enter and leave it under permission. Along with 136-kilometer wire fence around the zone since 1982, 163 guard posts and 10 checkpoints protected by armed police were installed. As Hong Kong, the southern part of the zone, was returned to China in 1997, the Shenzhen economic zone has even more strict restrictions.

People called the border line between Hong Kong and Shenzhen “the first gateway,” and called the border line between the Shenzhen economic zone and the mainland China “the second gateway.” The existence of the second gateway not only spoke for the unique status of Shenzhen in China, but also showed that China has not opened its doors completely to the outside world just yet. The second gateway was quickly influenced by the Western economy and culture, and prevented Chinese socialism from being in danger.

Thanks to the rapid economic growth, the Shenzhen special economic zone was expanded to the entire city of Shenzhen in 2010. As subway is operated all over the city of Shenzhen, the walls became useless. Instead, the walls and checkpoints cost tens of millions of yuans every year, becoming obstacles for urban development. The fact that the citizens had to be inspected when going back and forth also caused inconvenience. The walls, checkpoints and patrol roads have been being taken down since 2013 and they are still being taken down, but this border line, which officially divided China and Shenzhen, still exists.

The news media of Chia reported that this action made Shenzhen to take the lead in Chinese economic reform once again, showing their anticipation. But the Chinese State Council ordered in the decision letter of this action to reinforce management and restriction about the first gateway located in the border line between Shenzhen and Hong Kong/Macau.

# **U.S.-China trade war appears to be imminent**

Taobao, the marketplace of China’s biggest e-commerce Alibaba, has yet again made the U.S. government’s annual list of the world’s most notorious markets. While the United States and China continue to wrangle over trade, some watchers say that last year’s record-high trade surplus of China with the United States may soon trigger a trade war between the two superpowers.

Last year, the U.S. Trade Representative (USTR) put Taobao on its blacklist for the second year in a row over suspected counterfeits on the shopping platform in violation of intellectual property (IP) rights, BBC China reported Sunday. The USTR placed 25 online markets and 18 physical markets on its list of notorious markets for sales of pirated and counterfeit goods that infringe on IP rights and cause severe damage to U.S. workers. Taobao and another eight Chinese online and offline markets were included in the list, taking up 20 percent share.

Alibaba did not hesitate to defend itself, saying that it has become “a scapegoat for the USTR to win points in a highly-politicized environment” and that the USTR’s action is “not about intellectual property protection, but just another instrument to achieve the U.S. government’s geopolitical objectives.”

Early this month, the Committee on Foreign Investment in the United States (CFIUS) rejected the plan of Ant Financial, online payment services provider and affiliate of Alibaba, to acquire the U.S.-based money transfer company MoneyGram, feeding speculation that Washington is about to start a trade war with Beijing by reining in its representative conglomerate Alibaba.

Experts say that U.S. measures to raise tariffs on products made in China and import restrictions are likely to be met with China’s countermeasures in the same manner. Some U.S. pundits are voicing concern that in response, China may tighten regulations on key U.S. exported goods such as Boeing’s airplanes, automobiles, integrated circuits, soybeans as well as Hollywood films.

# **China urges S. Korea to address THAAD issue**

“The deployment of the THAAD system in South Korea is an obstacle to China’s relations with the nation. Restoring the relationship would not be possible without first resolving THAAD issues thoroughly.”

Wei Wei, the vice president of the Chinese People’s Institute of Foreign Affairs (CPIFA), was quoted as saying at the Korea-China Future Development Think Tank High-level Forum, which was held in Beijing on Monday. While the forum was designed to promote mutual understanding and amity between the two countries and discuss ways to improve their relations, the focal point of discussion was the Terminal High Altitude Area Defense missile system.

The vice president of CPIFA also mentioned the “Three Bans, One Restriction,” a phrase proposed in an article in the Chines state-run newspaper Global Times on October 29. The phase refers to China’s stance that South Korea and China have reached an agreement on a restriction of making use of the THAAD system as well as on three bans (deploying additional THAAD systems, joining the U.S. Missile Defense system and advancing a tripartite alliance between South Korea, the U.S. and Japan).

“The situation is complicated, but their decision to deploy the THAAD system in South Korea in July was inappropriate,” said a member of the Committee of Foreign Affairs of the Chinese People’s Political Consultative Conference. “South Korea should have considered the possibility of infringing upon the interests of their neighbors. I am afraid that the relations have cooled off owing to the THAAD issues. The responsibility lies in South Korea.”

# **China slaps sanctions on N. Korea on top of UNSC resolutions**

The Chinese government has limitedly banned its people from traveling to North Korea following a U.S. ban on its citizens travelling to the reclusive country, putting a strain on North Korea’s finances. North Korea reportedly has been earning 44 million U.S. dollars annually from tourism, with China taking up 80 percent of the total. According to the statistics released by the China National Tourism Administration in 2012, 237,000 Chinese travelled to North Korea.

Some point out that the effect of the travel ban remains to be seen as some regions, where most of the Chinese travelers to North Korea come from, including Dandong, Shenyang, Liaoning Province and Jilin Province are exempt from the ban. Other regions are seeing a reduction in demands, resulting in travelling agencies not offering travelling packages to North Korea.

“We do not have travelling packages to North Korea and have not received any notices banning travel to the North,” said a large travelling agency in Beijing.

“We have a five-day package departing from Dandong to North Korea on December 7 and a shuttle flight from Dandong to North Korea in the middle of December,” said another agency. “But we are not sure if making reservations would be possible.”

An agency in Shandong region said they have not offered travelling packages to North Korea for some time. An agency operating in Shenyang said that it has a four-day package to North Korea starting on December 16 but a day’s trip to Sinuiju can leave anytime, while the agency we spoke to in Jilin Province said that it does not have travelling packages to North Korea during winter times.

# **‘China should talk with Korea and the U.S.,' says a Chinese scholar**

A day before Song Tao, the head of the International Liaison Department of the Communist Party of China, visited North Korea as Chinese President Xi Jinping’s special envoy, a professor at one of China’s most prestigious universities earned people’s attention in Seoul by speaking about the need for trilateral dialogue in preparation for sudden changes in circumstances such as the possible collapse of the North Korean regime. His comment suggests that Chinese scholars are increasingly voicing their opposition to the nuclear and missile threats from North Korea.

“China should no longer be hesitant in discussing abrupt changes that can possibly occur in North Korea with the United States and Korea,” said Xia Liping, dean and professor of School of Political Science & International Relations at Tongji University in Shanghai, at the “2017 Northeast Asia Peace and Cooperation Forum” held at Grand Hilton Seoul last Thursday. Proposing to hold a trilateral “emergency plan dialogue,” he suggested agendas include which party would control in the case where the North Korea regime collapses; how we should handle the refugee issue of the North; which party should take the responsibility of restoring national order within North Korea in times of urgency; and how the Korean Peninsula can be politically stabilized after a crisis.

“Though sanctions on the North are being strengthened with China’s more active participation, pessimistic views prevail on whether North Korea will drop nuclear development as a result,” said the scholar. “Considering that North Korea continues to carry out nuclear and missile provocations despite China’s diplomatic efforts, now is the time for us to start a serious dialogue with the United States and Korea without hesitation.”

Xia added that as much as such agendas are tricky in nature, dialogue and negotiation on them will help the United States and China to ease the strategic stalemate in their relations.

# **Chinese envoy should warn against N. Korea's nuclear ambitions**

Song Tao, head of the Chinese Communist Party’s International Liaison Department, arrived in North Korea Friday as a special envoy of President Xi Jinping in a 4-day trip. This is the first minister-level visit in two years since Politburo Standing Committee member Liu Yunshan paid a visit to North Korea in October 2015. The Chinese government said the main objective of Song’s visit was to report the North on the 19th National Congress of the Communist Party of China. But the talks will likely be centered on resolving the North Korean nuclear issue as the visit comes after U.S. President Donald Trump’s travel to South Korea, China and Japan and his talks with South Korean President Moon Jae-in and Chinese President Xi Jinping on ways to resolve nuclear crisis with North Korea. Song’s visit to North Korea is expected to be an important turning point in resuming talks with the North.

Song will reportedly deliver a letter or verbal message from President Xi to reiterate China’s firm commitment to “denuclearization on the Korean Peninsula” and show that Xi is willing to improve relations with North Korea in his second term as president. Song could meet with North Korean leader Kim Jong Un and persuade him into suspending nuclear and missile tests and coming to the negotiating table. If North Korea responds positively to China’s suggestions, a summit meeting between China and North Korea that has not been held in six years since Kim assumed power could take place.

In the meantime, Trump showed his expectation, calling the visit “a big move” on his twitter accounts Thursday. The United States lowered the threshold for resuming talks with North Korea in time for the Chinese envoy’s visit to the North. U.S. Defense Secretary James Mattis also stated the same day that North Korea would have a change for dialogue if it suspends nuclear tests and development and export of weapons. The United States reportedly asked China to deliver its message to the North that the regime could be in big trouble if it ignores deterrence capability of the United States and the North should proclaim its will to give up nuclear program in exchange for dialogue.

But the situation is not optimistic as North Korea said Friday on Rodong Sinmun, the official newspaper of the Workers' Party of Korea, that, “We will never put issues related to our interest and the safety of our people on the negotiating table.” If Kim eventually refuses to accept China’s suggestions, Xi will likely put further pressure on the North by cutting off all shipments of oil to the country. This could spoil the current mood for dialogue, making nuclear crisis with North Korea even worse.

China remains to be North Korea’s only ally as of now, but it cannot forever side with the North if Beijing decides that Pyongyang is not in its interest anymore. Chinese special envoy’s visit to the North should not give the regime time to complete its nuclear development. China should clarify to Kim Jong Un that this is his last chance to resolve the nuclear crisis. Kim should also realize that the time is not on his side.

# **Chinese costume Qipao**

“The old good time is already gone and nothing is left there now.” These lines are from a scene in the Chinese movie “The Most Beautiful Moment in Life” by director Wang Ga Yi. In the movie, which presents a sad story of lost love, actress Maggie Cheung appears in different scenes, changing traditional Chinese costume Qipao dozens of times to display fatal seduction.

Qipao is characterized by the rising collar, slit on the skirt and tight style. The costume originated from the Eight Banner Army of the Manchurian tribe, who ruled the Qing Dynasty, and developed into traditional costume for both males and females. A remodeled Qipao, which emphasized seductive looks, started to be in vogue primarily around Shanghai in the 1920s. The attire is so sensual that former U.S. First Lady Pat Nixon even reportedly said, “I now know why China has so large a population.” In the award ceremony at the 2010 Guangzhou Asian Games and the 2008 Beijing Summer Olympics, ceremonial helpers showcased Qipao that even revealed their underwear lines, sparking controversy.

New Qipao fashion icons to succeed Maggie Cheung have emerged. U.S. and Chinese first ladies made public appearance, wearing black Qipao dresses, side by side at the state visit dinner in China on Thursday. U.S. First Lady Melania Trump was seen wearing highly colorful dress decorated with needlework, with fur attached on the sleeves, while Chinese First Lady Peng Liyuan was seen wearing see-through dress, which lightly revealed her skin under the sleeve. The model-turned U.S. first lady was wearing modern a Qipao dress, which was boldly open up to half of her thigh, in combination with kill heels from Manolo Blahnik. Her clothing is ready-made attire that Italian luxury brand Gucci introduced in the Fall/Winter season in 2016. The Chinese first lady picked a relatively mature Qipao dress that is open up to the knees.

Irrespective of their duel, the ultimate winner of the competition was Qipao. China widely promoted Qipao as attire for feminine diplomacy that exudes the beauty of tidiness, elegance and intellectuality. Thanks to the U.S. first lady, China has effectively promoted the nice appearance of its traditional Chinese costume. Fashion is important from the perspective of both culture and industry. We wonder when Korea will come to see world-renowned fashion designers fall in love with traditional Korean costume “hanbok,” design and sell clothing that features the aesthetic of uniquely Korean tradition. Korean costume has a long way to go to be globalized when compared with Qipao, which has made great strides.

# **‘No country can afford to retreat into self-isolation,’ says Xi Jinping**

During the 19th National Congress of the Communist Party of China on Wednesday where the Chinese leadership presents its national and foreign policy approaches for the second term of Chinese President Xi Jinping, Xi said, “No country can afford to retreat into self-isolation.” Xi was not referring to the Korean Peninsula or North Korea directly, but he seemed to regard North Korea that is not stopping provocation while being isolated from the international community.

While China’s foreign policy for the next term was being presented during the opening of the Communist Party Congress in Beijing, Xi said, “The instability and uncertainty that the world faces become more visible. Regional conflicts are occurring continuously in many places. No country alone can address the many challenges facing mankind.”

Xi announced his two-phased national development strategy as a blue print for his second term. During the first phase of development, Xi aims at prosperity of every citizen until 2020 and modernization of socialism until 2025. As for the second phase of development, Xi said he would make China as a strong and modernized nation of prosperous democratic culture and harmonious and graceful socialism between 2035 and the middle of the 21th Century. “National defense and military forces will be modernized by 2035 and China will nurture one of the strongest and advanced military forces in the world by the middle of the 21th Century,” said Xi. Speaking about plans for 2022 and beyond after his second term ends, Xi seems to consider seizing power much longer.

Xi talked about concentration of power by saying, “The whole party should obey the leadership and follow guidance by the leadership harmoniously.” He branded his philosophy as “socialism with Chinese characteristics for a new era.”

# **‘China has a great responsibility in N.K. nuke,’ says Chinese scholar**

“China cannot say it does not have a responsibility (in North Korea’s nuclear issues),” said Wang Yizhou, Deputy Dean of the School of International Studies at Peking University, in a recent interview with the Dong-A Ilbo. “The Chinese government needs to clearly acknowledge that we have a great responsibility (in Pyongyang’s nuclear issues) as well as important interests (in the Korean Peninsula),” the international relations expert added. “(Therefore,) Pressure on the North should be raised to a level where a war would not break out.

He clarified that his opinion was “apart from the (Chinese) government’s stance that views the United States and North Korea as countries with key responsibility for Pyongyang’s nuclear issues while China has only a secondary responsibility.” This is an indirect criticism of the Chinese government, which has insisted that Beijing’s role is limited as it is not a direct party involved and thus called upon Washington to have a direct dialogue with Pyongyang to resolve issues.

Professor Wang, together with Jia Qingguo, Dean of the School of International Studies at Peking University, is one of the well-known scholars of liberalism school of thought who emphasize China’s responsibility in the international community. Professor Jia said last month, “China should acknowledge the possibility of a war breaking out on the Korean Peninsula and should prepare against it through communication with the United States and South Korea,” publicly drawing criticism from scholars of mainstream school of thought who dismissed his remark as “nonsense that reversed the Maginot Line of China’s core principles in diplomacy towards North Korea’s nuclear issues.” Such a collision indicates that disputes over policy lines on the Korean Peninsula are being intensified in China.

Professor Wang said that he “mostly supports” Professor Jia’s opinions, adding, “As China continues to pursue reform and openness, more would agree on Professor Jia’s views.” He also said that “The (Chinese) authority did not say which side was right or wrong when the dispute arose, and they did not suppress discussion either,” viewing this as “a positive development and a great change from the past.”

“China is currently adjusting its policy towards North Korea,” the scholar also commented. “The intensity of sanctions, which was low in the past, is getting higher and the direction of sanctions is also becoming severe.”

# **Liu Xiaobo, China’s Nelson Mandela**

Liu Xiaobo, 61, a Chinese dissident who are on medical parole after being diagnosed with late-stage liver cancer, is said to be in a critical condition. Beijing said he is in a critical condition and finally allowed his family to visit him. While it permitted Western doctors to see him right before the G20 summit, it rejected Liu’s request for medical treatment abroad immediately after the summit. Though the Chinese government claimed that it was concerned about transportation, it would in fact have worried about his dissident activities outside China.

Liu who led a student movement during the Tiananmen Square protest in 1989 was sentenced to a three-year prison term for inciting the subversion of state power. After he requested reassessment of the protest in 1995, he was put under house arrest for nine months. When he called for a peaceful unification with Taiwan in 1996, he was sentenced to three years in a labor camp. He initiated a drive to sign the Chapter 08 criticizing the Communist Party of China, only to be sentenced to 11 years in prison. In total, he was put under arrest or imprisoned for four times. When he was in prison, he became the first Chinese who won the Nobel Peace Prize in 2010. He is called China’s Nelson Mandela outside China, but no one knows who he is in China. Baidu, the largest portal website in China, described him as a person who took corrupt money from a U?S. organization.

While most dissidents chose to exile abroad due to the pressure from the authorities, he eventually did not leave and struggled in China. Fang Lizhi, a famous Chinese astrophysicist, and Chen Guangcheng, a blind human rights lawyer, exiled or escaped to the U.S. Liu apparently wanted his wife to live in a free country. Foreign media outlet reported that he wanted to get medical treatment abroad not for himself but for his wife Liu Xia, 54, who has also been under house arrest for 10 years.

It is said that more than 180,000 protests per year are held out of discontent about society. The Xinjiang Uyghur Autonomous Region has an armed protest for independence every day. It means numerous people are imprisoned without the knowledge of the Chinese general public. Liu has called for freedom, human rights, equality, democracy and rule of law for his life. There is a long way to go for a country that cannot accept such universal values to become a world leader.

# **Korea, China, Japan say no to Trump's protectionism**

Korean, Chinese, and Japanese financial policy makers met at the Trilateral Finance Ministers and Central Bank Governors' Meeting held on Thursday in Yokohama, Japan and announced a communique saying that they will "reject any form of protectionism." The three financial ministers reaffirmed a strong coordination by saying, "We agree that trade enhances productivity as the most vital engine for economic growth, including job creation." It is noticeable that the financial leaders of the three Northeast Asian nations raised one voice against U.S. President Donald Trump who is currently hoisting the banner of America-first banner under protectionism.

Indeed, major advanced leaders have been busy reading President Trump's face for 100 days since his inauguration. Under the rejection from the U.S., the phrase "reject protectionism," which had appeared for three consecutive years, was excluded from the communique made in the G20 Meeting of Finance Ministers and Central Bank Governors held in Berlin in March this year. Furthermore, though IMF Managing Director Christine Lagarde, World Bank President Jim Yong Kim, and German Chancellor Angela Merkel all agreed to raise vigilance upon rising protectionism, only conflicts heightened as the U.S. opposed their idea at the G20 Meeting of Finance Ministers and Central Bank Governors held in Washington last month.

Just as Lagarde warned last year that the protectionism seen before World War I would lead to disastrous aftermath, not many countries can benefit from protectionism, which hurts global economic growth, inclusivism, and people. Unless the haunting shadows of protectionism stops lingering, the export-driven Korea will be the hardest hit. It is also disappointing to see two-faced Chinese President Xi Jinping who once claimed "China will become the guardian of free trade" at the Davos Forum held in Switzerland in January, and then turned towards higher trade barriers. Beijing encouraged Chinese consumers to boycott Korean products as a protest against the deployment of THAAD, and even blocked their people from visiting Korea. These recent movements portray the stark reality of international trade where the way of the closed fist prevails.

Therefore, it is very meaningful that Korea, China and Japan have raised one voice to protect free trade against all odds. In this borderless global economy, trade has become a strategic matter directly linked to national security. Under close coordination among the three nations, which account for 20 percent of global economy and trade, they may win more supporters to confront Trump's protectionism. In addition, their communique should go beyond words and actually translate into visible policy coordination. More efforts should be made to hoisting the flag of free trade, which is a resistless global trade trend at G20 meetings.
