# Supplementary material for: Bilingual translations of intensifiers in Dong-A Ilbo’s news about China: A corpus-based discourse analysis approach
Source: PLoS One. 2024 Feb 6;19(2):e0292603. doi: 10.1371/journal.pone.0292603 (PMC10846699; doi:10.1371/journal.pone.0292603)
Supplement: S1 File — (ZIP) [file pone.0292603.s001.zip › corpus data/subcorpus of Chinese translation.docx]

# **美国对中国实施半导体限制后，中国进口韩国产半导体占有率减少5.5%**

据调查，美国对中国实施半导体限制后，韩国产半导体在中国进口半导体市场上所占的比重有所减少。

　全国经济家联合会25日表示，对与2018年相比，2021年中国进口半导体市场的各国占有率变化进行分析的结果显示，韩国下降了5.5个百分点。这比制裁当事者美国（下降0.3个百分点）更受影响。中国台湾（4.4个百分点）、日本（1.8个百分点）、东盟6国（0.4个百分点）的占有率比美国的限制规定前有所增加。

　美国商务部在2019年4月~2020年9月分四次以中国华为、SMIC为对象，以交易限制名单的方式限制了利用美国的半导体软件、设备生产的半导体供应。

　与美国制裁前的2018年相比，去年中国的半导体进口规模增加了37.2%。韩国产进口虽然增加了6.5%，但与竞争激烈的台湾产（57.4%）、日本产（34.8%）等相比，增幅较小。

　中国进口韩国半导体的比重减少的决定性原因是受美国限制的影响，韩国企业中断了华为的供应。以制裁开始的2019年为基准，华为在三星电子和SK海力士的总销售额中所占的比重分别为3.2%（约7.37万亿韩元）和11.4%（约3万亿韩元）。

# **中国在乌克兰战争中加快核武装步伐，在沙漠中建洲际导弹库**

据美国《华尔街日报》报道，中国领导层以对抗美国为由，正在加快核武装的步伐。分析认为，在乌克兰战争中出现的俄罗斯总统普京的核威胁发挥了效果。

　《华尔街日报》9日援引熟悉中国领导层情况的消息人士的话报道说：“中国在乌克兰战争前就一直在推进核战斗力的增强，通过此次战争得到了确信，”“对于美国克制直接介入战争的原因，可能得出了是俄罗斯拥有的核武器的结论。”报道还说：“中国表示担心与台湾发生军事冲突时美国会使用核武器，因此正在增加核武器来应对。”也就是说，如果中国和台湾之间发生冲突，就像乌克兰战争一样，为了防止美国的直接介入，中国正在增强核武器。

　据分析卫星照片的专家们介绍，中国正在加紧建设位于西部沙漠地区甘肃省玉门附近的新型洲际弹道导弹“东风（DF-41）”机库可疑设施100多处。在1月份拍摄的卫星照片中，遮挡机库的临时帐篷全部被拆除。这意味着担心信息泄露的敏感工作已经结束。据悉，可搭载核弹头的“东风-41”的最大射程为1.5万公里，可以瞄准美国本土全境。中国当局对这个机库缄口不语。

　美国专家推测，目前中国拥有数百枚核弹头。有预测称，到2020年代末，这一数字将增至1000多枚。

### 上海因封锁长期化而人心惶惶……甚至传出“关闭港口说”

- [Font size down](https://www.donga.com/cn/List/article/all/20220405/3294646/1)
- [Font size up](https://www.donga.com/cn/List/article/all/20220405/3294646/1)

[한국어](https://www.donga.com/cn/List/article/all/20220405/3294646/1?m=kor)

- [Share this article on Facebook](https://www.donga.com/cn/List/article/all/20220405/3294646/1)
- [Share this article on Twitter](https://www.donga.com/cn/List/article/all/20220405/3294646/1)

# **上海因封锁长期化而人心惶惶……甚至传出“关闭港口说”**

随着中国经济首都上海因新冠疫情的封锁时间延长，市民的恐惧正在加大。据美国有线电视新闻网（CNN）报道，上海港等待装货卸货的船只在封锁后也激增至300艘以上，物流障碍将更加严重。随着上海封锁长期化，3月28日停产的电动汽车企业特斯拉也将持续关闭工厂。这是自2019年末启动以来中断时间最长的一次。

　据中国国家卫生健康委员会4日透露，前一天中国全国新冠确诊患者为1.3137万人，接近2020年2月12日1.5152万人的历史最高纪录。当天上海新增确诊病例9006例。当初上海市当局公布的封锁时限是4日，但封锁仍在继续。 　随着上海市民的不安心理增大，相关传闻也在扩散。3日微博上迅速传出了上海某医院一名儿童因新冠治疗无效死亡的消息和视频。该视频中可以看到医务人员走近躺在病床上的孩子的样子，画面外还听到了像父母一样的人的喊叫声。

　上海市卫生健康委员会当晚破例发表声明，正式解释说：“相关视频是上海复旦大学医院对因高烧而昏迷的孩子进行急救的场面。”声明说：“孩子恢复了意识，父母也向医疗人员道歉。”有传闻称，上海供应蔬菜的企业把蔬菜堆放不管后又废弃，世界最大的物流港上海港即将关闭等。对此，上海市当局虽然解释说不是事实，但对政府的不信任累积的市民却不相信。上海市在正式宣布“不会封锁”的第二天即3月28日开始全面封锁，已经失去了市民的信任。

# **掌握应对朝鲜挑衅钥匙的中国，不能再维护和包庇**

韩国当选总统尹锡悦昨天与中国国家主席习近平进行了首次通话。这次通话是在朝鲜发射“怪物洲际导弹”的第二天进行。尹锡悦就韩半岛安保情况和应对朝鲜挑衅的方案与习主席进行了协商。朝鲜当天确认洲际导弹是“火星-17”型导弹，并声称是“可靠的核战争遏制手段”。

　　通过发射“怪物洲际导弹”打破红线的朝鲜正在露骨地提高核威胁水平。朝鲜国务委员长金正恩下达了亲笔命令书，要求“勇敢地发射”，并前往平壤顺安机场观看了发射过程。朝鲜媒体对此进行了报道，使用了13次“核”一词。朝鲜摆出了随时准备继续进行核试验等追加挑衅的样子。

　应该阻止朝鲜妄动的中国应对确实令人失望。中国前天对朝鲜发射洲际导弹表示：“希望有关各国坚持对话、协商的正确方向。”不仅没有谴责，反而把责任推给周边国家。中国也没有在联合国安理会发出应有的声音。中国难免会受到“借助新冷战的潮流，为朝鲜打开了挑衅余地”的批评。

　中国就对朝问题立即与尹锡悦合作的可能性不大。中国一直对阐明“堂堂正正外交”的尹锡悦的对华政策表现出不满。即便如此，中国也没有余力帮助朝鲜进行核挑衅。朝鲜核技术的提升对于即将开始第三个任期的习主席来说既是政治负担，也是安保威胁。这是助长周边国家核扩散、加速地区不稳定因素。

哪怕从现起， 中国也应该向朝鲜施压，要求朝鲜停止挑衅，进行对话。首先应该改变与国际社会对朝制裁对着干的态度。联合国安理会第2397号决议虽然包含了朝鲜发射洲际导弹时自动追加制裁的“触发条款”，但这也要通过决议才能实现。中国的参与是作为国际社会一员应该承担的义务。新政府也应该做好准备，以便在上台后尽快与中国进行实务协商。阻止朝鲜拥核是关系到韩中两国国家利益的共同分母，也是左右今后关系的核心问题。

# **引导李氏朝鲜开放港口的中国，扩张主义的内心想法**

 1882年6月朝鲜军人闯入宫中后，日本和清朝立即出兵。继日本派遣军舰和300人到济物浦之后，清朝也派遣以广东驻军为主的3000名兵力登陆南洋湾。在一触即发的危机中，两国开始交涉，清军甚至将兵乱背后的大院君绑至天津。

　作者作为美国加利福尼亚大学戴维斯分校教授，一直研究东亚近代史，他在该书中追踪了19世纪后半期韩中日三国间的国际关系被并入近代世界秩序的过程。作者特别将寅午兵乱视为以中国为中心的朝贡体制瓦解的历史事件。当时，清朝在镇压兵乱后继续在韩半岛驻扎军队，深度干预朝鲜内政。这是违背了根据儒教秩序，作为宗主国只行使礼仪性权限，不干涉朝贡国内政的朝贡体制传统的行为。

　中国如此倾向于扩张主义，直接原因是当时日本、俄罗斯渗透到东亚地区。不仅是中国北部，离首都北京不远的韩半岛也是本国安全的核心缓冲国。

　有趣的是，鸦片战争以后，与西方列强签订屈辱性外交条约的清朝要求朝鲜通过与列强的条约开放港口。这是旨在拉拢西方列强牵制日本和俄罗斯的所谓“以夷制夷”战略。但这意味着朝鲜被编入以对等主权国家之间的外交行为为基础的“近代世界秩序”。换句话说，正如这本书的题目所暗示的那样，这是以宗主国为顶点的东亚世界秩序消失的终结。

　最近，由于北京冬奥会的裁判争议，反华情绪高涨，如何看待中国成为人们热议的话题。在成为6·25战争分水岭的毛泽东决定中国参战之前，19世纪清朝的膨胀主义是改变围绕韩半岛的东亚世界秩序的一轴。

# **用1亿人一天的饮用水制造人工雪……北京“反环境奥运会”引发争议**

随着中国决定用人工雪进行2022年北京冬奥会的所有雪上比赛，有人担心会对环境产生负面影响。有人预测说，此次大赛的人工雪量相当于1亿人每天喝的水量，因此，赛场附近居民将出现缺水现象。中国在北京奥运会开幕式上展示小圣火，强调“环保奥运会”，但与此形成鲜明对比的现象正在发生。

　美国有线电视新闻网（CNN）5日报道说，此次大会在不适合举办冬奥会的气候下进行，再加上全球变暖导致的气温上升，人工雪的制造将消耗更多的电力和水。据国际奥委会透露，本届奥运会上，为了制造人工雪，预计将消耗约4900万加仑（约1.8548亿升）。这与约1亿人每天喝的水量相似。

　有分析称，在举行户外项目的地区中，大部分地区由于今年冬天严重的干旱，降雪量不足，因此对人工雪的依赖度增大。进行野外项目的张家口地区平时的年均降雪量也只有200毫米。人均可用水量不到中国平均水平的五分之一，属于干燥地区。此前彭博社曾报道说，为了填满该地区的滑雪场，需要200立方米的水，但只确保了53立方米。

　中国自称实现了“环保奥运会”。在4日的开幕式上展示奥运会历史上最小的圣火的张艺谋总导演表示：“这是中国政府代替大量使用燃料的大型圣火传递的环保创意。”

# **中国令安理会失去力气，如果现在不勒紧朝鲜，反而会遭受损失**

 为应对朝鲜发射中程弹道导弹的挑衅，联合国安理会4日召开会议，但未能拿出任何结果就宣告结束。这是因为，虽然这是安理会今年以来第三次针对朝鲜接连发射导弹挑衅举行会议，但这次需要安理会过半数理事国共同应对的要求，也遭到了掌握否决权的中国和俄罗斯的反对。中国方面不仅没有谴责朝鲜的挑衅，反而向美国主张“要表现出接受朝鲜担忧事项的政策和行动”。

　在此次安理会会议上，讨论了射程达5000公里的洲际弹道导弹挑衅问题，因此也不无期待认为此次与之前两次短程导弹不同。2017年朝鲜进行大量核、导弹挑衅时，安理会曾将朝鲜机构和团体列入对朝制裁名单，积极应对。但是，朝鲜再次进行了4年多来最高级别的挑衅，但安理会却连一张声明都没有发表。中方此次将媒体声明草案送交本国讨论，但是也很有可能不了了之。

　安理会应对告吹后，包括美国在内的9个国家发表联合声明说：“安理会的沉默会使朝鲜更加大胆，认为违反决议是理所当然的，将继续威胁国际和平。”非安理会理事国的日本也参与、但韩国没有参与的这一声明，并不止于担忧。朝鲜已经开始威胁进行核试验和洲际导弹挑衅。这一切都是因为中国包庇、俄罗斯帮助、韩国袖手旁观而发生的。

　被称为世界和平与安全堡垒的联合国安理会之所以失效，与美中之间的霸权竞争和美俄之间的军事对峙等国际对决局势不无关系。朝鲜也趁着这种新冷战氛围尽情地展开挑衅。但是如果现在不能阻止朝鲜的核“疾驰”，最终会成为中国的一大难题。几年前，习近平主席每次举行重大国际活动时，朝鲜都会用核、导弹挑衅在宴桌上撒灰。

# **北京奥运圣火时隔14年之后将再次被点燃**

时隔14年，中国北京将再次燃起奥运的火花。以4日晚9点举行的开幕式为开端，2022年北京冬奥会将进入为期17天的激烈角逐。北京是奥运会历史上第一个同时举办夏季、冬季大会的城市。

　举行开幕式的中国北京国家体育场是2008年北京夏季奥运会当时举行开闭幕式、田径、男子足球决赛的地方。因其独特的设计被称为“鸟巢”。但这次除了开闭幕式以外，不会另外举行比赛。总导演也是2008年开闭幕式总导演张艺谋导演。此次开幕式约100分钟，将有3000多名演出者参加。其中95%左右是10多岁的年轻人。在长达4个小时的2008年开幕式上，当时有1.5万多人参加了演出。考虑到新型冠状病毒肺炎（COVID-19）疫情的扩散和寒冷的天气等，缩减了规模。再加上美国等部分西方国家表现出外交抵制的意向，外宾也减少了。东道主中国的国家主席习近平、俄罗斯总统普京等将出席开幕式。

　开幕式之火的最终点火者、点火方式等都保密。2008年，中国体操英雄李宁（59岁）身上挂着钢丝，沿着赛场屋顶内侧墙壁奔跑，点燃了圣火台。张导演已经公开表示：“将以创意性的点燃圣火的方法让人们大吃一惊。”由于一直使用氢气作为圣火燃料，因此也有人对环保点火方式表示期待。另外，最终点火者候选人有在历届冬奥会上获得6枚奖牌的短道速滑代表王濛（37岁）、在2008年夏季奥运会上获得3冠王的体操代表邹凯（34岁）等。PS：此次北京冬奥会主题口号为“一起向未来”。

# **2022年北京冬奥会圣火传递活动规模大幅缩减，只在北京附近传递三天**

在2022年北京冬奥会开幕之前，本应辗转于中国全境的圣火（照片）因担心新型冠状病毒肺炎(新型冠状病毒)疫情的扩散而最大限度地“蜷缩”了。

　截至4日，去年10月20日抵达中国北京的奥运圣火被保管在北京奥林匹克塔楼内。从下月2日开始的3天时间里，将有1200名运动员从比赛举办场地北京中心地带开始，只在北京郊外的延庆区和河北省张家口区域进行圣火传递活动。如果算上往返距离，也只有300公里左右。2008年北京夏季奥运会当时，2.188万名运动员跑遍了包括5个大陆世界19个城市在内的13.7万公里。

　与最近的奥运会相比，此次传递规模也大幅减少。在东京夏季奥运会上，圣火从去年3月25日福岛开始，在121天内与1万名火炬手一起绕行日本列岛2000公里返回东京。2018年平昌冬奥会当时，从2017年11月1日开始的101天里，7500名火炬手绕行了17个市道和江原道全市郡，跑了2018公里。

# **香港反华网媒《众新闻》宣布停刊**

香港反华网络媒体《众新闻》（Citizen News）2日停刊。分析认为，由于当局日益严厉地镇压媒体，最近连规模较大的媒体也陆续选择停刊，因此报纸认为不可能再坚持下去。继去年6月香港最大的日报《苹果日报》和去年12月29日著名网媒《立场新闻》之后，大约半年内有3家反华媒体关门，有人担心香港的言论自由实际上已经消失。

　《众新闻》2日在脸书上写道：“在危机时期，首先要保障船上所有人的安全。以沉重的心情宣布停刊。”报方解释称，虽然想继承新闻业的精神，为大众服务，但近两年来香港社会的变化和舆论环境的恶化，使《众新闻》这条小船遭遇强风和波涛汹涌。

　创办人兼总编克里斯·杨（杨健兴）3日召开记者会表示：“停刊决定是在短时间内完成的。不能排除我们可能会面临危险。”尤其是，其决定停刊的原因，是《立场新闻》4天前选择停刊。在《苹果日报》和《立场新闻》停刊前，其前任和现任干部接连被捕，公司资产也被冻结，因而都选择了停刊。分析认为，其意思是不能让成员经历这样的事情。

　《众新闻》是2017年1月1日创刊的40名职员规模的小型在线媒体。它追求自由、开放性、多样性、包容性，在2019年反对罪犯引渡法（遣返法）及2020年反对香港《国家安全法》的示威中，忠实地传达了民主阵营的声音。

# **G2暗淡的新年展望：中国不及印度，美国被新冠疫情所困扰**

主要外媒一致报道，就世界霸权国家地位对立的美国和中国今年将双双经历艰难的一年。据英国《每日电讯报》去年12月31日报道，中国受新冠疫情带来的强硬封锁政策、房地产亏损、电力短缺等影响，经济增长存在大幅放缓的危险。美国也面临着新冠疫情扩散趋势、通货膨胀、供应链危机和物流大乱、国际社会领导力低下等问题。美国政治媒体《国会山》1日评价说，迎来执政第二年的拜登政府也面临“地雷阵”。

○ 中国经济增长率将46年来首次落后于美国

日本投资银行野村证券预测，今年中国经济将增长4.3%。美国经济增长率预测值为4.6%，比预测值高出0.3个百分点。据世界银行统计，美国创下高于中国的增长率是自1976年以后46年来的第一次。

中国的经济增长率从1991年到2018年的约30年间从未降至6%以下。野村证券预测，只要出现一名确诊患者就要封锁整个城市的中国特有的“零新冠”政策越是长期化，带来的经济负面影响就越大。

《每日电讯报》预测，“崛起的巨人”印度的经济增长率将达到8.5%，约为中国的2倍，印度经济将超越中国，长期保持高增长。法国乐师资产管理公司也预测，被称为“世界工厂”的中国生产能力将受到严重打击。中国不仅是韩国的最大出口国，在中国当地设立生产工厂的韩国企业也很多，预计韩国经济也将不可避免地受到负面影响。

中国与美国围绕台湾的矛盾激化也令人担忧。中国国家主席习近平在12月31日通过中国中央电视台现场直播发表的新年贺词中表示，“实现祖国的完全统一是两岸同胞的共同愿望，”对台湾施压。当天，他发表了以去年7月召开的中国共产党100周年纪念仪式照片为背景的新年贺词。

中国军用飞机也在新年第一天1日上午8点左右进入了台湾防空识别区。对此，台湾领导人蔡英文也在同一天脸书直播演讲中针锋相对地表示：“必须提醒中国，不要误判形势，防止军事冒险主义在内部扩张。”

○ 执政第二年的拜登面临暗淡的“地雷阵”

美口最近日增新冠确诊患者连日刷新历史最高纪录，新年第一天还接连发生航空大乱等，交通、行政功能受到打击。据航班追踪网站“航班跟踪”透露，受新冠疫情导致飞行员不足、暴雪等影响，1日共取消了2655个航班。

《国会山》当天指出，拜登应该解决新冠疫情和奥密克戎变异毒株的扩散、传染病大流行对医疗及金融体系的影响、通货膨胀等问题。最近，拜登的国政支持率为30~40%，创下了就任以来的最低水平。执政的民主党的选举战略家乔尔·佩恩表示：“拜登得益于新冠疫情成为总统，但现在又因为新冠疫情处境艰难。他的运气是伴随新冠疫情的。”

国内外危险因素也很多。拜登预告，将在去年1月时任总统特朗普的支持者不服特朗普大选失利、闯入国会一年后的6日发表对国民演讲。随后，特朗普也表示，将在同一天举行“针锋相对的记者会”。很多人担心前任、现任总统的冲突正在加剧美国的分裂和两极化。

有人指出，俄罗斯入侵乌克兰的威胁正在加大。这种情况下，拜登将于美国东部时间2日与乌克兰总统泽连斯基通电话，再次确认保卫乌克兰领土的决心。拜登去年12月30日向俄罗斯总统普京警告：“如果俄罗斯入侵乌克兰，将严厉制裁。”

# **郑义溶：“南北关系难以以北京奥运会为契机取得改善”**

韩国外交部长郑义溶表示：“原希望北京奥运会成为改善南北关系的契机，但从目前来看，这种期待实际上已变得越来越困难。”

郑义溶29日在首尔外交部大楼举行的记者招待会上，就明年2月在北京冬奥会上举行南北首脑会晤的可能性等问题做出了上述回答。受新冠疫情影响，朝鲜能否参加奥运会尚不明确。在这种情况下，再加上美国还宣布进行外交抵制，因此他承认难以把冬奥会作为终战宣言的跳板。不过，政府一名高层官员表示：“郑义溶此话的意思，是难以把‘冬奥会’用于和平进程。但是，反而是在冬奥会之前，南北关系也有可能取得重大进展。”

对于外交抵制北京奥运会，郑义溶表示“没有考虑”，确认了原有的立场。对于“文在寅总统是否有可能出席冬奥会”的提问，他只是简单地回答说：“现阶段没有可以分享的内容。”据悉，青瓦台内部强烈认为，从目前来看，文在寅难以参加冬奥会。

郑义溶就韩美目前正在协调的《终战宣言》草案表示：“实际上已经达成协议。”这比“协调工作几乎已经结束”的原先立场更进了一步。他还表示：“正在讨论如何与朝鲜进行（有关终战宣言的）谈判。希望朝鲜做出具体回应。”

郑义溶就日军“慰安妇”问题表示，“大家不是很清楚原罪在哪里吗?”将因历史问题导致韩日关系恶化的责任推给了日本政府。他还表示：“日本顽强地坚持我们要遵守2015年（慰安妇）协议的立场，（韩日关系）完全没有进展。”对于日本最近打算将朝鲜人强制劳役场——佐渡矿山载入世界文化遗产一事，郑义溶抨击说：“深表忧虑，深表遗憾。”

有人指出，韩国政府只对朝鲜和中国的人权问题视若不见。对此，郑义溶解释说：“这是因为韩国与朝鲜和中国有着特殊的关系，而且与韩国安保直接相关，需要合作的部分很多。”

# **三星电子：“中国西安工厂因新冠疫情扩散实行弹性运营”**

由于新型冠状病毒肺炎（COVID-19）疫情的扩散，中国陕西省西安市实际上已经被封锁，在这种情况下，三星电子在当地的半导体生产出现了一些问题。

　三星电子29日通过本公司新闻室表示：“中国西安半导体事业场随着新冠疫情的持续扩散，正在进行生产线的灵活调整。”灵活调整意味着半导体生产将降到比平时低的水平。三星电子补充说：“这是根据公司的经营方针做出的决定，即应优先考虑员工的安全和健康。”

　作为陕西省省会、人口达1300万的大城市，西安市在本月9日至22日新增206例新冠病毒感染病例，政府随即实行全面禁止居民外出的防疫措施。因为，中国当局正在实施只要出现1名感染病例，就封锁整个地区并隔离所有居民的政策。三星电子此前适用中国政府的特别措施，工厂一直正常运转。但是，随着地区封锁时间的延长，不仅是员工上下班，连物流也受到了影响，因此开始调节生产量。

　西安半导体工厂是三星电子唯一的海外存储半导体工厂，从2014年开始生产闪存芯片（NAND）。闪存芯片不仅是移动设备的数据存储场所，还用于在服务器、PC等储存数据所需的固态硬盘（SSD）的制造。三星电子在闪存芯片世界市场的占有率为40%左右，但在三星电子总产量中，约40%是在西安工厂生产。

　另外，位于西安的三星SDI的电动汽车电池工厂目前正常运转。三星SDI将根据新冠疫情的扩散程度和中国政府的措施，决定今后的工厂运营方向。

# **美国明年第一季度经济增长率预测值从5.2%下调至2.2%**

随着新型冠状病毒肺炎（COVID-19）新变异毒株“奥密克戎（Omicron）”的迅速扩散，美国和中国等世界经济将势必受到不小冲击。

　据《华尔街日报》27日报道，各国经济专家正在下调明年美国和全世界的经济预测。穆迪分析公司首席经济学家马克•詹迪将明年第一季度（1~3月）美国国内生产总值(GDP)增长率从原来的5.2%（年率）下调至2.2%。他提到了最近发生的航空大乱导致的旅游减少、体育比赛和百老汇公演中断等，并诊断说：“正在出现与今年夏天德尔塔变异毒株扩散时非常相似的现象。”经济研究机构万神殿宏观经济研究所也将明年第一季度美国经济增长率预测值从之前的5%下调到了3%。

　最近，世界银行（WB）也将中国明年的经济增长率预测值从5.4%下调到了5.1%。据预测，随着奥密克戎变异毒株导致封锁措施的长期化，经济活动明年也将继续萎缩。德国中央银行德意志银行也将德国明年的增长率从6月预测的5.2%下调至4.2%。

# **联赛延期的NHL，大概率不参加北京冬奥委会**

2022年北京冬奥会票房亮起了“红灯”。因为既是冬季奥运会之花，又是最受欢迎项目的冰球项目，北美职业冰球联盟（NHL）的选手们将不参加。

　美国体育专门媒体《ESPN》 22日报道说，NHL劳资双方就不参加北京冬奥会成了协议。阻碍NHL参加奥运会的原因是新型冠状病毒肺炎（COVID-19）疫情的扩散。

　NHL因新冠病毒奥密克戎（Omicron）变异毒株的扩散，推迟了2021∼2022赛季常规赛50场比赛。本想在圣诞节休假三天，但又决定从22日到26日中断联赛。ESPN称：“如果NHL在明年1月10日之前决定不参加冬奥会，就可以避免罚款等处罚。NHL计划不久后正式公布不参加奥运会的相关消息。”

NHL从1998年的长野到2014年的索契，参加了历届所有的冬季奥运会。但是，2018年平昌冬奥会因经济上无法获益、联赛日程紧张等原因没有参加。NHL考虑到拥有13亿人口的巨大的中国市场，表示要参加北京冬奥会，但最终决定连续两届缺席冬奥会，只参加2026年的米兰-科尔蒂纳丹佩佐冬奥会。

　北京冬奥会不接受海外观众，只向本国国民出售入场券。虽然NHL不参赛将不会出现门票销售剧减等问题，但大赛权威下降和收视率下降等问题将不可避免。

# **美媒：“除辉瑞和莫德纳之外，其他疫苗几乎对奥密克戎毒株没有效果”**

美国《纽约时报》当地时间19日报道说，新冠疫苗中，除了“信使核糖核酸（mRNA）”方式的辉瑞和莫德纳以外，其余的疫苗几乎没有预防感染奥密克戎变异毒株的效果。有人担心，以依靠mRNA方式以外的其他疫苗的低收入国家为中心，感染将继续增加，并出现变异。研究结果显示，奥密克戎毒株能使新冠抗体治疗药物失效。

《纽约时报》说：“陆续发表的研究结果显示，只有辉瑞、莫德纳的疫苗才能防止奥密克戎毒株引起的感染。”英国卫生安全局12日表示，对疫苗接种者进行分析的结果显示：“接种阿斯利康疫苗6个月后，预防感染奥密克戎毒株的效果将降至0%左右。”《纽约时报》指出，虽然作为一针接种方式的强生疫苗在非洲的需求激增，但“预防奥密克戎感染的效果几乎可以忽略”。对于占全世界疫苗一半的中国疫苗（科兴、国药），报道称：“几乎无法防止感染奥密克戎。主要是中国、墨西哥、巴西等国接种了该疫苗。”主要在非洲和中南美国家注射的俄罗斯卫星疫苗的预防效果也很低。

美国约翰斯霍普金斯大学布隆伯格公共保健研究生院研究员托尔伯特·尼恩斯表示：“富裕的发达国家没有共享使用尖端技术的mRNA疫苗技术，低收入国家只能依赖其他疫苗。结果，这些国家不断发生变异，延长了新冠大流行。”

据美联社20日报道，美国制药公司“再生元”和“礼来”表示：“经美国食品医药局批准紧急使用，美国医院使用最多的本公司抗体药物对奥密克戎不起作用。”两家公司的抗体治疗药物正被用于防止新冠患者趋于重症。两家制药公司表示将迅速开发新的抗体药物，但美联社表示：“至少需要几个月时间。医疗人员将面临新的危机。”

# **文在寅：“不考虑外交抵制北京奥运会”**

正在对澳大利亚进行国事访问的韩国总统文在寅13日就一些国家外交抵制明年2月的北京冬奥会一事表示：“韩国政府不考虑抵制。”从与中国针锋相对的美国开始，澳大利亚、英国等国家也加入了抵制行列，但文在寅与此划清了界限。

文在寅当天在澳大利亚堪培拉同澳大利亚总理斯科特·莫里森举行首脑会谈后举行的联合记者会上表示：“美国等任何国家从未劝说参与抵制北京冬奥会。”他还表示：“无论从经济方面、韩半岛的和平与稳定还是朝鲜的无核化，我们都需要中国作出建设性的努力。韩国正在努力以同美国建立稳固的同盟关系为基础，与中国保持和谐的关系。”分析认为，此话表明，美中都是发表《终战宣言》的重要相关国家，因此韩国将延续战略模糊性基调，展开均衡外交。

美国彭博社就此报道说：“韩国在抵制北京奥运会问题上与美国闹掰了（Breaks With US）。”报道分析称：“以对朝和解为核心目标的文在寅总统要想在剩下的任期内取得进展，需要中国的帮助。”

# **政府将正式推进占世界贸易15% 的CPTPP的加入工作**

韩国政府将正式推进占世界贸易15%的《全面、渐进的环太平洋经济伙伴协定（CPTPP）》的加入工作。虽然出现了可以降低对中国的贸易依存度，实现交易多边化的期待，但是预计也会出现担心农产品进口增加的农水产业界的反对。

　 13日，经济副总理兼企划财政部长官洪楠基在对外经济长官会议中表示：“通过扩大贸易和投资的经济考虑、战略价值等，将正式推进加入CPTPP”，“将以与相关利害关系者等进行社会讨论为基础，启动程序。”这距离2013年政府讨论CPTPP的前身环太平洋经济伙伴协定（TPP）已有8年。

　 CPTPP是美国退出TPP后，日本、澳大利亚、墨西哥等11个国家于2018年签署的多边自由贸易协定（FTA）。主席国由日本担任，今年9月中国也申请加入。

　 CPTPP的关税撤销率最高为96%，市场开放度很高。CPTPP占世界贸易的15%，如果加入CPTPP，对中国的贸易依赖度会降低，出口市场也会变得多样化。

　 今年1月，韩国开发研究院（KDI）曾表示，“加入CPTPP有助于降低对中国的依赖度，扩大通商领域。”在中美贸易矛盾持续的情况下，可以纳入稳定的全球供应链，这也是一大优点。特别是，还可以期待与没有和韩国签订FTA的墨西哥首次签订FTA的效果

　 相反，农水产业界则担心农产品进口增加。因为在CPTPP参与国中，澳大利亚、智利、加拿大等农业强国较多。当天，韩国综合农业团体协议会发表声明反驳说：“相对来说价格竞争力较高的进口农产品的增加会导致农业生产基础的长期崩溃。”

　 实际上，要想加入CPTPP，需要听证会、国会报告程序和与会员国的详细协商，预计需要2~3年的时间。有人指出，政府开始讨论加入问题8年后才进入正式加入程序，因此“马后炮加入”导致谈判力下降。因为为了最终加入，需要日本等参与国的一致同意。梨花女子大学法学专门研究生院教授崔元睦（音）分析说：“加入时间比中国晚了一步，因此韩国的谈判力必然会下降。而作为主导国的日本也可以将日本企业对强制征用受害者的赔偿判决等活用在谈判中。因此还有许多问题需要解决。”

# **美国宣布“外交抵制北京奥运会”，中国表示“坚决反击”**

美国在当地时间6日宣布，对明年2月举行的北京冬奥会进行“外交抵制”。这是把中国政府在新疆维吾尔自治区的人权压迫规定为“种族屠杀”，开始进行外交制裁。尽管中国公开警告要进行报复，新西兰等仍宣布参与抵制，这种情况下，正在推进《终战宣言》的文在寅政府暂时倾向于向奥运会派遣外交使团。

美国白宫发言人简·普萨基当天表示：“拜登政府决定不向北京冬奥会和残奥会派遣外交及官方代表团。”即，美国将派遣体育代表团，但不会派遣由政府高层人士或政界人士组成的正式使团参加开、闭幕式。普萨基表示：“在中国新疆，‘种族歧视’和‘践踏人权’正在持续。我们不能参加奥运会比赛的进行曲。”

拜登政府实际上也公开要求其他盟国参与抵制。美国国务院发言人内德·普莱斯表示：“将从更多国家听到（抵制）消息。”在这种情况下，新西兰7日表示：“决定不派遣政府高层代表团参加北京奥运会。”预计，以英国、澳大利亚、加拿大等为中心，抵制宣言将会接连不断。

中国强烈反对说：“将采取坚决的反击措施。”中国驻美大使馆发言人刘鹏宇7日抨击说：“这是不会对奥运会成功举办产生任何影响的政治操作。”中国外交部发言人赵立坚6日也在例行记者会上表示：“如果美国擅自行动，中国一定会坚决采取反击措施。”

预计美国将在9日、10日邀请韩国等110多个国家举行的民主峰会上也要求共同参与抵制，但青瓦台暂时对不参加奥运会的可能性保持距离。韩国外交部发言人崔永杉7日在例行记者会上表示：“美方通过外交渠道提前向我方通报了这一决定。不过没有要求参与抵制。”他还表示：“韩国政府一直支持北京奥运会的成功举办。希望奥运会能为世界和平、繁荣和南北关系做出贡献。”

# **香港《保安法》实行一年，5500名学生离校**

一项调查结果显示，在过去一年里，香港初中和高中辍学和离职的学生和教师超过5000人。退学的10名学生中有6人干脆表示将离开香港。有分析认为，这是因为香港实施对进行反中活动的香港市民最高可判处无期徒刑的《国家保安法》等，香港的社会环境与《国家保安法》实施前发生了很大的变化。

据香港媒体“HK01”2日报道，前一天香港初高中校长协会——香港中学校长会以140所初高中为对象进行调查的结果显示，2020年至2021学年度一年间有4460名学生和987名教师离开。这相当于平均每个学校有32名学生和7名教师离开。在一年前的调查中，分别有2700名学生和498名教师辞职，相比之下大幅增加。

特别是，退学学生中有2643人（约59.2%）表示，将离开香港前往其他国家。初中校长会表示担忧说：“选择退学移民到其他国家的教师也增加了7倍以上。在过去的一年里，学生和教师的离校现象非常严重，这是不争的事实。”

有分析认为，发生这种事态的原因是，随着《国家安全法》的实施，香港整体的社会环境变得压抑，教育政策和课程也变成了一边倒的“亲中”，学生和教师都感到失望。据分析，为防止新冠疫情扩散，中国大陆和香港之间的往来受到限制，因此中国大陆学生无法到香港学校上下学，这也产生了一定的影响。此前，中学校长会曾于7月份向当局表示，“很多学生和教师正在前往海外”，要求制定对策。

# **中国也迎来低生育-高龄化……英媒：“4年后大逆转，中国成人尿布需求将大于婴儿尿布”**

英国《金融时报》报道说，从2025年开始，中国成人尿布销量将超过婴儿尿布销量。这可能是中国正在经历的严重的低生育问题和老龄化现象的反映。

《金融时报》11月29日报道说，最近中国婴幼儿尿布销量逐渐减少，而为患尿失禁的高龄层和老人疗养设施等而使用的成人用尿布销量正在增加。中国企业有关人士向《金融时报》表示，到2025年，成人纸尿裤销量将超过婴儿纸尿裤。

据《金融时报》报道，中国第一大尿布销售商尤妮佳在成人用品上花费的市场营销费用比婴儿用品多。中国湖北省尿布生产厂的有关人士表示：“已经将婴儿生产线改为成人生产线。”《金融时报》分析说，中国尿布市场正走向拐点。

据香港证券公司里昂证券（CLSA）的分析，去年中国的尿布市场为890亿美元（约105.7943万亿韩元），其中成人用尿布市场不到10亿美元。但成人用尿布市场预计到2040年将增长到300亿美元（约35.73万亿韩元）。

这是因为中国人口结构的变化。据世界银行统计和中国人口调查，中国女性人均生育率从1961年的5.9人降至去年的1.3人。这是1952年开始调查以来的最低值。法国投资银行——法国外贸银行预测，去年中国的老龄层是人口的10%，但到2050年将增长到25%。”

《金融时报》说：“尿布销量的变化意味着人口统计学的变化和社会结构的转换。”此前经历老龄化的日本也从10年前开始出现成人用尿布销量超过婴幼儿用尿布的现象。

# **徐薰明日访华，与杨洁篪讨论终战宣言**

青瓦台国家安保室室长徐薰将从2日起对中国进行为期两天的访问。在韩美有关终战宣言的讨论进入收尾阶段的情况下，徐薰可能会在此次访华中就终战宣言寻求中国方面的协助，并为将朝鲜拉到对话桌前寻求合作方案。

据外交消息人士30日透露，徐薰将于2日至3日访问中国，与中国共产党负责外交事务的政治局委员杨洁篪举行会晤。两人自去年8月以后时隔1年零3个月再次见面。青瓦台表示：“韩中两国高层通过各种契机保持战略沟通，在这种情况下，正在与中方协商徐薰室长访华事宜。”

预计，徐薰此次访华期间将希望中国在终战宣言上给予协助，并就明年2月的北京冬奥会交换意见。据悉，美国暗示将对北京奥运会进行外交抵制，中国担心新冠疫情持续等导致冬奥会票房失败，正在寻求韩国政府的协助。青瓦台一名有关人士表示：“希望北京奥运会成为改善韩朝关系的转机，成为为东北亚世界和平做出贡献的契机，这一立场没有改变。（就外交抵制等问题，）政府目前并没有特别的立场。”

此次访华期间，徐薰还将讨论中国国家主席习近平访韩的问题。据悉，由于新冠疫情仍在持续，青瓦台正在考虑举行视频首脑会谈的方式。

# **故乡和家**

故乡是我们的思念所向的地方。根据雅克•德里达的说法，这是一块“埋葬祖坟的土地”，或者是一个“从那里衡量一切旅行和距离的静止地点”。因此，生活在陌生土地上的人们希望在死后安葬在故乡。但是，上周为领取第一届富川流散文学奖而来到韩国的美国作家哈金却不这样看待故乡。根据他的说法，故乡是人类扎根生活的地方。不是离开遗弃的地方，而是在某处重新创造的流动的东西。

　如果考虑到他的人生，这句话是对的。在美国留学的他在1989年通过电视看到天安门事件的真实情况后，选择了流亡。他还用英语写出了以中国为背景的作品，获得了全美图书奖。中国政府把他打上叛徒的烙印，几十年来一直不允许他入境。但他认为背叛的主体不是自己，而是屠杀无辜年轻人的国家。对他来说那个国家是需要保护的“吃掉子女的母亲”。这就是对他来说中国不是故乡的原因。

　但他的心理现实却不同。虽然他说美国是他的家，但他却一再回到中国。因为每次小说中都出现中国人或中国移民，从心理和隐喻的意义上看，与“回乡”没有什么区别。也许因为他在三十岁的时候就离开了故乡，认同感已经确立。与他的话不同，美国几乎不可能成为真正的故乡和家。

　他虽然来到了韩国，但却无法回几个小时就能抵达的故乡，而是冷冷地回到了他的家所在的美国。过去的36年他就是这样生活的。父母去世时，他连回老家哀悼都没有。这是残酷的刑罚。正是这种现实使他成为了一位流散作家。这就是他写的诗和小说周围闪现伤痕的原因。

# **陷入中美半导体战争的SK海力士，要以扩大绝对差距来摆脱困局**

据路透社昨天报道，SK海力士想要引进荷兰产尖端设备改善中国江苏省无锡工厂的计划，因美国政府的反对而面临流产的危机。报道还说：“在美国和中国的地缘政治纷争中，SK海力士有可能成为下一个牺牲品。”处在美中经济霸权战争中心的韩国半导体企业最担心的事件很有可能成为现实。

SK海力士第三季度在世界DRAM（动态随机存取存储器）半导体市场的占有率为27%，仅次于三星电子（44%），位居第二。美国、欧盟、中国等国家的竞争企业得到政府的各种补贴和税制支援，SK海力士要想甩掉它们，必须对尖端技术和装备进行进攻性投资。特别是无锡工厂是生产该公司一半DRAM产品的重要设施，有可能对企业竞争力产生负面影响。

拜登政府摆出了一副绝不允许使用美国及同盟国技术的尖端半导体设备进入中国的架势。表面上的理由是有可能被恶用于加强中国的军事力量，但其主要目的，是想牵制要在数量和质量所有方面成为制造业最强国的“中国制造2025”计划。美国政府上周甚至放弃了想要增加中国当地硅晶片产量的本国半导体企业英特尔的计划，似乎不允许一切例外。

问题是中国是购买韩国产半导体40%的最大客户。有分析认为，如果加上通过香港的迂回出口，所占比重将超过60%。如果因美国的掣肘而不能正常对中国工厂进行设备投资，中国可能会无辜的韩国企业而不是美国为目标进行报复。就像发生“萨德事件”时一样，完全不同领域的韩国企业也会受到牵连。

解决这种尴尬状况的方法只有一个，那就是进一步拉大技术与质量上的绝对差距，无论出现什么矛盾，中国企业都不得不购买韩国产半导体。但是，SK海力士计划投资的龙仁半导体集群工业园区，却因说服居民和批准推迟，开工时间比原计划推迟了一年以上。政府和政界必须为《培养半导体产业特别法》提供更果断的支援政策和放宽限制的方案，尽快予以通过。

# **在美国访问的崔钟建：“现实中需要与中国的伙伴关系”**

韩国外交部第一次官崔钟建当地时间15日在华盛顿举行的以韩美关系为主题的战略论坛上表示，“中国是战略伙伴，现实中需要与北京的伙伴关系”，强调了韩中关系的重要性。对此，美国政府前高级官员表示担忧说：“担心韩美同盟长期削弱，美国在决策过程中可能会忽视韩国。”

崔钟建当天在由美国智库“战略与国际问题研究中心”和韩国国际交流财团共同主办的韩美战略论坛上发表主旨演讲，首先强调了韩美同盟的重要性。他指出，“韩美两国向全世界展示了21世纪的同盟是什么”，并评价称，韩美同盟不仅在传统安全领域，在经济、文化领域也在发展伙伴关系。

但在接下来的问答环节，当被问及韩国的对华立场时，他答道：“他们是战略伙伴。和其他国内政策一样，外交政策也应该符合韩国人、韩国中产阶层的需要和利害关系。”他解释说：“与中国的贸易规模比美国和日本的总和还要大，享受从这一市场获得的巨大收益的是我们的国民。”他还提到了供应链问题，并指出：“对来自中国的各种产品的依赖程度不只是韩国的问题，而是所有人的问题。”他还就朝鲜问题表示：“现实中，我们有必要同北京建立伙伴关系。无论我们愿不愿意，这就是我们政策的现实。”他提醒道，韩国是在地理位置上最接近中国的国家，并表示：“我们正在努力与中国建立良好的关系。”

崔钟建的问答结束后，作为专家出席当天活动的前美国国防部负责亚太事务的部长助理兰德尔·施莱佛说：“如果在一方看来是重要的、核心的挑战，而另一方不接受这种看法，同盟关系是不会拥有未来的”，“如果（韩国）以这种方式漂流，（韩美）同盟就可能逐步弱化，从这一点来看很危险。”

# **暂时松了口气，1.87万吨中国尿素将入境国内救急**

中国政府向韩国政府通报说，将正常执行与韩国企业签订合同的1.87万吨尿素的出口程序。若这批尿素正常到货，可以满足韩国国内2至3个月的需求。其中1万多吨车用尿素最早将于本月末至下月初进入国内。虽然暂时解决了尿素溶液紧缺事态的燃眉之急，但是中国并没有中断尿素溶液的出口前检验制度，因此中国产尿素溶液的进口能否持续顺利进行还是未知数。

韩国外交部10日表示：“为了尽快启动中国产尿素的进口程序，通过多种渠道同中方进行了沟通，结果确认将执行韩国企业已签订合同物量（1.87万吨）的出口程序。”从出口前的检查申请到检查结束大约需要2周的时间，考虑到这一点，从本月末到下月初，合同量可以进入国内。外交部表示，在1.87万吨尿素中，有700吨已申请出口前检查，其中A公司进口的300吨车用尿素已完成检查，将于下周返抵达韩国。

　但有观测认为，中国实际上已经停止了尿素出口，因此今后供求将很难完全恢复到以前的水平。外交部的一位官员表示：“可以理解为（出口程序）在短期内已经恢复正常。”

# **美联储：“担心中国恒大事态的风险威胁国际经济”**

美国中央银行——联邦储备委员会当地时间8日警告，面临破产危机的中国大型房地产公司恒大的事态可能波及美国等全球经济。就在今年9月，美联储主席杰罗姆·鲍威尔还表示，“恒大问题只局限在中国”，否认了扩散为全球金融危机的可能性。

美联储在当天公布的《上半年金融稳定报告》中指出：“中国房地产问题给美国金融系统带来了风险。”美联储在报告中表示：“考虑到中国经济和金融系统的规模、其与全世界的贸易联系等方面，中国金融的不稳定可能通过心理上的恶化，给全球金融市场带来压力，威胁全球经济增长，对美国也会有影响。”

美联储还表示：“中国企业和地方政府的负债仍大，通过金融部门的杠杆（借款投资）高，房地产投资价值也过分上涨”，“在这样的环境下，如果中国集中对高杠杆机关进行限制，就像恒大事件所示，可能给房地产领域等高负债企业造成危机。”恒大集团此前一直以贷款为基础积极扩张事业，但最近随着政府出面限制房地产，陷入了流动性危机。美联储还表示：“如果危机转移到金融公司，房地产价格突然发生调整，投资者的风险承受倾向减少，中国的金融体系可能会受到很大压力。”

# **RCEP明年1月生效……日本：“期待与韩国、中国的首个自贸协定”**

随着中国、日本等10个国家之间的区域全面经济伙伴关系协定（RCEP）将从明年1月起生效，日本国内出现了对搞活经济的期待。有预测认为，随着RCEP的生效，日本国内生产总值将增长2.7%。韩国虽然也在RCEP上签了字，但由于国会还没有批准，没有被包括在明年1月生效的国家之中。

日本外务省3日宣布：“随着澳大利亚和新西兰2日批准RCEP，RCEP生效的条件已经充分”，“从明年1月1日起，RCEP将在日本、中国、澳大利亚、新西兰、新加坡、越南、泰国、文莱、柬埔寨、老挝等10个国家生效。”包括韩国在内，印度尼西亚、马来西亚、缅甸、菲律宾等5个国家尚未提交批准文件。

RCEP是成员国之间相互降低关税以活跃区域内贸易的多国间自由贸易协定。如果15个签署国全部完成批准程序，RCEP将成为比没有美国参与、有11个国家签署的《全面与进步跨太平洋伙伴关系协定》（CPTPP）规模更大的世界第一大自贸协定。以2019年为准，成员国整体贸易规模达5.6万亿美元（约6600万亿韩元，占全球31.9%），国内生产总值达26万亿美元（30.8%），人口22.7亿人（29.7%）。RCEP是包括韩中日三国、今后发展潜能巨大的东盟在内的自贸区，因此备受关注。

日本政府发言人、官房长官松野博一4日在记者会上表示：“随着世界经济增长中心地区与我国连接，将为经济增长作出比现在更大的贡献。（日本）将发挥主导作用。”

《日本经济新闻》分析说：“日本与韩国和中国签订的第一个自贸协定将开始启动。预计将带来将日本国内生产总值提高2.7%左右的效果。”《读卖新闻》报道说：“参加国对经济复苏的期待越来越大，同时对想要在亚太地区贸易领域掌握主导权的中国的警戒心也越来越高。”

印度也曾试图参与RCEP，但在最终签名时置身在外。印度在与中国的贸易中饱受逆差困扰，担心廉价的中国产品的攻势会更加猛烈，所以决定不参与。

# **美军第二号人物：“中国增强军事力量令人震惊，再这样下去会赶超美国”**

继美军第一号人物、参谋长联席会议主席马克·米利确认中国发射高超音速导弹并表示担忧后，参谋长联席会议副主席约翰·海滕也就中国增强军事力量的速度表示“令人震惊（stunning）。”美国有线电视新闻网（CNN）等媒体报道说，海滕当地时间21日表示：“中国的行动速度令人震惊。按照这一速度和轨迹，中国将赶上俄罗斯和美国。”他强调：“这将改变游戏格局，这不仅是美国的问题，也是我们同盟国的问题。”

据海滕介绍，中国在最近5年里试射了数百枚高超音速导弹，而美国只发射了9枚。对于这一巨大差异，他指出，“这不好”。他就美国发射失败表示：“应该将其视为学习过程。只有敢于冒险并从失败中学习，才能更快地前进。”他还以朝鲜为例表示：“朝鲜国务委员长金正恩通过过去失败的试验发射，学会了加快开发速度。”据说，在科学家和工程师试验失败时，金正恩也和其父亲（金正日）不同，决定不处死他们，反而鼓励他们，从失败中学习。海滕说：“最终，世界经济排名第118名的朝鲜拥有了洲际弹道导弹力量。”

# **受全球供应难的影响中美物价持续上涨，韩国进口物价也亮起红灯**

受全球范围内的供应大乱的影响，美国和中国等全球经济的通货膨胀压力正在增大。面临严重的招聘难和供应链危机的美国企业因无法承受费用的增加，正在接连上调产品价格。最近遭受电力难和原材料价格暴涨直接打击的“世界工厂”中国的物价也大幅上涨。在中国的通货膨胀扩散到全世界的危险增大的情况下，韩国的进口物价也进入了紧急状态。有人预测说，随着供应链危机阻碍经济复苏，美国第三季度(7至9月)的经济增长率仅为前一季度的一半水准。

　27日(当地时间)，据《纽约时报》(NYT)和《路透社》等媒体报道，当天公布第三季度业绩的美国主要企业一致预告将上调产品价格。也就是说，将原材料和人工费上涨带来的费用增加部分最终转嫁给消费者。

　快餐连锁店麦当劳表示，为了反映人工费和原材料价格的上涨部分，今年将把产品价格上调6%左右。据悉，麦当劳因招聘难，今年的人工费已经上涨了10%以上，食品材料和其他材料的价格也最多增加了4%，正在承受费用压力。为应对物价上涨，已经将产品价格上调1.5%的食品企业Kraft Heinz表示，明年也将继续保持这种价格政策基调，暗示了进一步上调价格的可能性。可口可乐也在当天公布业绩时表示：“如果人工费和物流费用保持较高水平，可以根据需要上调价格。”全球制造企业3M也表示：“聚丙烯等原材料费用和人工费面临上涨。为应对通货膨胀和供应链压力，将上调产品价格。

　企业之所以出现这种上调价格的现象，主要原因是最近出现了罕见的人力难和供应链危机。在美国，每月招聘人数持续超过1000万人，需要很多人手，但由于真正做事的人不足，各企业争先恐后地提高工资。另外，随着港口和陆地物流出现瓶颈现象，企业对单价较高的航空货物的依赖度提高，甚至出现了干脆启用自己的货船的企业。

# **北京冬奥会，处于防疫考虑将只允许中国本土观众入场**

中国方面表示，明年2月将以最小规模举办北京冬奥会。政府计划减少不必要的活动和程序，大幅减少投入到奥运会的人力。这是为了防止新型冠状病毒肺炎(COVID-19)疫情的扩散而采取的措施。

　26日，据《新华社》等中国媒体报道，北京冬奥会组委会在前一天公开了包含奥运会防疫守则的防疫手册(manual)。第一次公开的此次防疫手册适用于运动员和大赛相关人士，以后还可以补充一两次。

　根据手册，为此次奥运会准备的专用交通工具和宿舍、附属设施、赛场和训练场等所有空间将连接在一起运营。这些空间就像覆盖了泡沫一样，成为与其他外部空间完全隔离的封闭区域。大会参赛选手只能往返有限的空间，每天都要接受新冠病毒检查。未能接种新冠肺炎疫苗的参赛选手抵达中国后，将在北京隔离21天。

由于计划将投入奥运会的人力最小化，因此，为方便选手团而活动的志愿者也将消失。与以无观众的形式举行的2020年东京夏季奥运会不同，虽然计划让观众入场，但计划将规模最小化，不接纳来自中国本土以外的观众。

　面对北京冬奥会，美国和欧盟(EU)的集体抵制动向也在持续。但专家认为，实际上抵制的可能性不大。亚洲大学国际学部政治外交系教授金兴圭表示：“美国最近对中国表现出柔和的态度。与中国的不和可能会对美国舆论产生负面影响。面临明年中期选举的乔•拜登政府不会愿意承担这样的风险。”

# **90%的日本人“讨厌中国”……66%的中国人“讨厌日本”**

调查显示，10名日本人中有9人对中国的印象是“不好”。中国人中对日本的印象回答“不好”的也占66.1%。中日两国明年9月将迎来邦交正常化50周年，但今年两国都对对方的感情比前一年恶化。

据《每日新闻》21日报道，日本的非营利团体“言论NPO”和中国国际出版集团8月21日至9月25日以1000名18岁以上（日本）男女和1547名（中国）男女为对象，就对对方国家的认识进行了调查。在日本人中，对中国持否定态度的人比去年增加1.2个百分点，达到90.9%。这是自2016年开始调查（91.6%）以来的最高值，也是5年后再次超过90%。

在中国人中，对日本持否定态度的人比去年增加13.2个百分点，达到66.1%。对日负面印象在因尖阁列岛（中国称钓鱼岛）矛盾两国发生冲突的2013年达到90.1%后持续减少，但一年之后就大幅增加。言论NPO负责人工藤泰志20日在记者会上分析说：“只讨论相互军事威胁，两国国民的不安被放置一边。”

中日国民对对方国家的否定认识高于韩日国民。言论NPO与韩国东亚研究院实施的韩日国民相互认识调查结果显示，韩国人对日本的否定印象从去年的71.6%减少到今年的63.2%。同期，日本人对韩国的负面印象从46.3%小幅增加到48.8%。2015年以后，日本人的反华情绪处于持续比反韩情绪高出近2倍的状态。

# **《金融时报》：“中国8月份试射高超音速导弹……令美国大吃一惊”**

英国《金融时报》16日援引消息人士的话报道说，中国于8月秘密试射了可搭载核武器的问超音速导弹。

据《金融时报》报道，中国的高超音速滑翔飞行器（HGV）落在距离目标约32公里处。《金融时报》说：“该测试显示了中国高超音速武器的进展，令美国情报机构大吃一惊。”中国拥有高超音速导弹的事实早已为人所知，但通过此次试射，证明了中国的高超音速武器相关技术比美国所知道的要先进。中国核武器政策专家、麻省理工学院教授泰勒·弗雷布尔在接受《金融时报》采访时表示：“搭载核弹头的高超音速导弹一旦完成，美国的导弹防御体系将成为无用之物。”

高超音速导弹的速度是音速的5倍到最多20倍以上，是可以改变战争格局的被称为“改变游戏规则者”的尖端武器。弹道导弹上升至太空，画着抛物线飞行，但高超音速导弹在大气中以低轨道飞行，快速打击目标。有评价认为，这将使针对巡航导弹和弹道导弹的各国导弹防御系统失效。

高超音速导弹可以在1∼2个小时里对全世界任何地方进行打击，能够突破导弹防御网，因此美国、中国、俄罗斯等军事强国正在带头开发高超音速导弹。朝鲜也于上个月28日试射了高超音速导弹“火星-8型”，加入了竞争行列。中国曾在2019年10月建国70周年阅兵式上首次公开高超音速导弹“东风-17”。同年年底，俄罗斯实战部署了“前卫”导弹，去年成功试射了新型高超音速巡航导弹“锆石”。

有评价认为，美国最近一直在大力推进高超音速导弹的开发，但到目前为止在与中国、俄罗斯的竞争中处于劣势。美国4月进行了B-52H战略轰炸机发射高超音速导弹“AGM-183A ARRW”的试验，但未能成功。

# **习近平与即将卸任的默克尔视频会谈，破例展露笑颜……引用古语表示祝福**

中国国家主席习近平13日与从2005年起执政16年并即将卸任的德国总理安格拉·默克尔举行视频会谈，并称默克尔为“老朋友”，给予了极高的礼遇。默克尔在任期间曾12次访问中国，在中美矛盾中也没有单方面站在美国一边。默克尔与施加压力增加驻德美军防卫费分摊额等压力的前美国特朗普政府发生了相当大的摩擦。

中国共产党机关报《人民日报》14日在头版刊登了前一天习主席和默克尔总理进行视频会谈时露出灿烂笑容的照片。习近平以在公开场合几乎不笑而闻名，但当天却一反常态地始终面带微笑。《人民日报》评价说：“默克尔在任期间，不仅是中国和德国的关系，中国和欧盟的关系也变得非常深厚。”习主席还特别强调：“中国人重情重义，不忘老朋友。中国的大门随时都向你敞开。”

中国外交部也在网站上表示，习近平在此次会谈中引用了孟子的一句话：“人之相识，贵在相知，人之相知，贵在知心。”他还称赞，默克尔在任期间，中国和德国证明了可以避免“零和”游戏，获得相互利益。

中国官方英文报纸《环球时报》也评价说，默克尔在主要国家领导人中访问中国的次数最多，并推行了务实的对华政策。2005年11月就任的默克尔到新冠疫情暴发之前的2019年9月为止，共访问了中国12次。其间，习近平三次访问德国。两人在新冠疫情暴发后通过电话和视频会谈进行了接触，特别是仅今年就进行了5次交流。

# **油价80美元……供应大乱中，高物价“海啸”也会袭来吗？**

随着供应链危机和原材料价格暴涨，世界经济陷入混乱。由于没有零部件，工厂停止生产，油价每桶上涨到80美元，阻碍了全球经济的复苏。美国、中国等巨大经济圈的景气出现了放慢的征兆。这有可能直接打击以出口支撑的韩国经济。如果再加上油价引起的物价上涨，内需萎缩也不可避免。

当地时间11日，美国西德克萨斯产原油价格时隔7年突破每桶80美元。这是因为新冠恢复势头和取暖需求重迭。因此，芬兰北欧银行将明年美国经济增长率预测值从3.5%下调至1.5%。意思是，由于油价暴涨，物价必然会上涨，消费必然会趋缓。这种趋势必然会对全球经济和韩国出口产生负面影响。

供应链危机也在恶化。不分原材料和中间材料，不仅出现瓶颈现象，还出现了物流困难。由于消费者不能及时得到产品供应，物价正在急剧上涨。如果物价上涨，企业就会为了高价出售而增加生产，这是常识。但是，由于生产本身就很困难，因此有人担心经济停滞导致物价上涨的滞胀。

截止到9月，韩国消费者物价连续6个月上涨2%，超过了当初的管理目标1.8%。要想控制物价，必须采取上调利率等紧缩措施，但这可能会给经济复苏泼冷水。韩国银行昨天冻结基准利率也是这种苦恼的结果。

企业为了供应原材料和零部件，正在启动紧急体制。政府也不能陶醉于出口好转，安逸地应对。为了不影响供应链，应该与企业共同应对，并仔细分析油价上涨带来的电费影响。考虑到物价和景气的细致的利率政策，也是不容易的课题。仅凭观察情况，全球环境过于紧迫。

# **面临大规模电力短缺，中国再次“举白旗”进口澳大利亚煤炭**

英国《金融时报》4日报道，为了报复在中美矛盾中站在美国一边的澳大利亚，中国当局阻止了澳大利亚产煤炭的进口，但由于煤炭不足事态严重，中国进口企业开始装卸澳大利亚产煤炭。有分析认为，由于煤炭不足导致发电站运营中断，引发大规模电力难，面临四面楚歌状况的中国屈服于澳大利亚。

据《金融时报》报道，从上月底开始，中国主要港口正在从在海上待命的澳大利亚货船上卸载煤炭。国际船舶经纪公司百力马-艾斯盟的货物负责人尼克·里斯提克表示：“45万吨煤炭已经卸货。”能源咨询企业Kpler也向《金融时报》表示，上个月从5艘船上卸载了38.3万吨澳大利亚产煤炭。当地贸易商认为，中国当局发出了“允许通关”的信号。

去年，中国命令国营能源企业和钢厂“停止进口澳大利亚产煤炭”。这是对世界最大煤炭出口国澳大利亚的贸易报复。由于这一措施，澳大利亚蒙受了约39亿美元（约4.6352万亿韩元）的损失。

随着澳大利亚产煤炭被禁止进口，加上中国国家主席习近平推行“环保低碳”政策，中国出现了煤炭短缺现象。这导致中国东北部的电力短缺，部分地区工厂停产，家庭用电也受到限制。吉林省等中国各地方政府为在印度尼西亚、俄罗斯、蒙古国、哈萨克斯坦等地确保煤炭而竭尽全力，但随着世界煤炭需求的增加，价格也暴涨，因此很难进口。

# **面临“破产危机”的中国恒大，瑞典也受到波及**

因约355万亿韩元的天文数字负债而面临破产危机的中国房地产公司恒达集团事件的后续风暴甚至蔓延到了北欧的瑞典。

据彭博社2日报道，与恒达子公司恒达新能源汽车（恒达汽车）合作开发电动汽车的瑞典汽车公司“瑞典国家电动汽车（NEVS）”最近解雇了工厂670名职员中的近一半（300人）。

瑞典国家电动汽车公司有关人士表示：“由于没有从恒达汽车公司调剂资金，因此不仅裁员，还中断了电动汽车的开发。实际上已经关闭了工厂。”首席执行官斯蒂凡·蒂尔克也表示：“正在寻找新的合作公司和投资者，而不是恒大。”

准备明年批量生产电动汽车的恒大汽车最近也没能向中国国内的工厂设备业主支付货款。部分职员的工资也被拖欠，研究所研究员的免费用餐也全部中断。

恒大集团于上月底出售子公司持有的股票，确保了约1.83万亿韩元的资金，但仅今年内要偿还的利息就达7500亿韩元，危机仍在持续。特别是，主力事业房地产因中国政府的限制性规定进入停滞期，曾有潜力的电动汽车事业也将不可避免地出现差池，因此有预测认为，恒大集团实际上不可能自力更生复苏。

恒达集团上个月29日未能向债权人支付约559亿韩元的美元债券利息；在此前的同月23日也没有支付约993亿韩元的美元债券利息，而是推迟了30天。

# **郑义溶：“中国采取攻势外交是理所当然的”……在美国发表维护中国的言论引发争议**

韩国外交部长郑义溶当地时间22日把美国、韩国、日本、澳大利亚等盟国为对抗中国而成立的联盟定义为“冷战思维”。他就在美国受到抨击的中国的“进攻性（assertive）外交”表示：“这对中国来说是理所当然的事情。”在美国拜登政府全面推行牵制中国政策的情况下，韩国的外交首长在美国做出了似乎维护中国的发言，引发了争议。

为出席联合国大会而正在美国访问的郑义溶当天出席纽约智库美国外交协会邀请的座谈中，就主持人“你是否认为近年来中国越来越变得具有进攻性”的提问表示：“那是理所当然的（only natural）”，“中国在经济上变得更强，现在已经不是20年前的中国。”他表示：“中国希望在外交政策中反映自己拥有的东西是很自然的事情。不知道能否将其称为进攻性。”他还表示：“中国希望作为国际社会的一员反映自己的声音。我们应该倾听他们想对我们说的话。”

主持人、美国有线电视新闻网主播法里德·扎卡里亚似乎在说明印度太平洋地区的外交地形时要把美国、韩国、日本、澳大利亚区分为对抗中国的一个联盟，郑义溶对此表示：“那就是正如中国人们所说的冷战思维（the mentality of cold war）。”郑义溶就加入美国在印度太平洋地区作为牵制中国的主要联合体的“四方机制”（quad）的提问表示，“不觉得有必须加入的紧急必要性”，并认为“并不认为必须在美中两国应选一方。”

# **“9·11”恐怖袭击20周年，中美应该收起“力量的傲慢”，展开竞争和合作**

迎来“9·11”恐怖事件20周年的11日，美国各地举行了悼念遇难者的仪式。夺走2977人生命的恐怖事件发生20年的象征性，再加上该恐怖事件引发的阿富汗战争上月底结束，使追悼氛围更加严肃。到处都挂着“永不忘记（Never Forget）”的标语，前、现任总统等美国领导人一致要求国民团结应对恐怖袭击。世界首脑们也强调了国际联合。

“9·11”恐怖事件给21世纪初的自由主义国际秩序带来了巨大的恐惧和愤怒，将唯一超级大国美国的力量推上了试验台。10年前在冷战中取得胜利的同时引领一极秩序的美国立即宣布“与恐怖主义的战争”，在阿富汗战争和伊拉克战争中充分发挥了其力量。但阿富汗和伊拉克是美国的“泥潭”。上个月像被赶出阿富汗一样撤退的美军寒酸的样子象征性地体现了这一点。

美国现在想摆脱这个泥潭，完全集中精力与中国展开霸权竞争。拜登政府就任后就表示要在比前政府更加精巧、周密的战略下牵制和包围中国。当然，这是如果不阻止中国的强烈挑战，美国的地位也会受到威胁的危机意识的产物。对于这样的美国，中国表现出极度的警戒心，但对对抗却表现出坚决的态度。

拜登10日与中国国家主席习近平时隔7个月再次通话，就通过相互对话和沟通尽到国际责任达成了尽管是原则性的共识，这是值得庆幸的事情。虽然立场差异依然存在，但美中首脑在“9·11”恐怖事件20周年前一天的通话具有很大的象征性。反恐战争已经过去20年，但这场战争变得更加艰难。美中需要合作的领域不仅仅是恐怖主义。不仅是气候变化，朝核等不扩散问题等堆积如山。国际政治中激烈的竞争是不可避免的。但如果强国之间的对抗、实力政治占据主导，不仅会给自己，还会给国际社会带来灾难。要在竞争中合作。

# **北京大学教授张维迎正面批评习近平“共同富裕”**

中国北京大学的一名经济学教授最近正面反对中国国家主席习近平提倡的“共同富裕”。他认为，如果政府过度介入，“共同富裕”可能会成为“共同贫困”。习主席执政以来，由于对反对势力的大规模整顿工作和扩大监视和管制，反对政府政策的声音几乎消失，在这种情况下出现的主张备受关注。

香港《南华早报》4日报道，北京大学经济系教授张维迎（62岁，照片）最近在公益性民间学术机构“经济50人论坛（CE50）网站上发表文章说：“失去了对市场力量的信任，经常依赖政府介入，会共同导致贫困。”他强调：“如果企业家没有创造财富的动机，政府就没有钱给贫困阶层，上游就会像干涸的河流一样。计划经济试图为贫困阶层提供更多的福利，但结果出现了更多的贫困阶层。只有提前市场指向性改革，才能创造更加公正的社会。”

张维迎毕业于中国西安大学，在英国牛津大学获得经济学硕士、博士学位，从1994年起担任北京大学教授，2008年还成立了北京大学主要智库———国家发展研究院。张维迎2018年10月也曾主张，“中国过去40年的高增长是通过市场化、企业家精神、西欧300年的技术积累形成的，并不是因为所谓的‘中国模式’”，从而引发了风波。

目前张维迎登载在CE50网站上的文章已经被拿下，其个人微信（中国版kakaotalk）账号也被删除。在微信上发送相关文章也不行。

# **拜登在从阿富汗撤军的第二天表示：“现在应该应对中俄等新威胁”**

美国总统拜登宣布阿富汗战争结束，并强调现在是应对中国、俄罗斯等美国面临的21世纪威胁的时候了。

拜登在美国完成从阿富汗撤军一天后的当地时间8月31日在白宫发表对国民演说。他表示：“世界正在改变。我们正在与中国进行严重的竞争，并在应对俄罗斯的挑战，还要应对网络攻击和核扩散。”他还表示：“没有比中国和俄罗斯更喜欢美国在阿富汗陷入另一个10年的泥潭的国家。我们应该表现出应对2021年新挑战的力量。”

拜登强调有必要集中精力应队中国这一美国的最大威胁而不是中东。他强调：“现在是翻过过去20年的外交政策一页的时候了。”拜登在演讲中明确表示，其外交政策方向是，美国要摆脱“世界警察”的角色，以国家利益为基础，集中应对现在和未来的外交安保威胁。分析认为，他提及“核扩散”的部分，可能是考虑到了中国和朝鲜等国的核威胁不断扩大的情况。

当天，白宫就朝鲜重启宁边核设施的动向表示，“（对朝鲜接触的）大门敞开着”，再次提及了同朝鲜对话的必要性。白宫发言人普萨奇在新闻发布会上就“是否有与金正恩接触的新尝试”的提问答称：“我们的提议是无论何时何地都要在没有前提条件的情况下会面。”正在访美的韩半岛和平交涉本部长鲁圭德当天在华盛顿特派记者会上表示：“韩美两国正在就可以共同推进的对朝人道领域等与朝鲜有关的多种方案进行磋商，两国的共同立场是，做好一切准备，一旦朝鲜响应就可以推进。”

# **中国河南省大暴雨至少造成33人死亡…全球一半苹果手机供应工厂也受到打击**

# 中国中部河南省省会郑州遭遇60年来罕见的特大暴雨，截至22日，至少造成33人死亡，25.6万人被迫撤离。中国台湾省省长蔡英文罕见地就引发超过300万名灾民等最严重的洪灾表示慰问。 　据中国河南省当局22日透露，截至当天上午，确认的死亡者至少有33人，比前一天的25人增加了8人。因地铁被淹，仅郑州就有12人死亡，随着各地区因泥石流等被埋的人得到确认，死亡人数有所增加。截至目前，共有300.4万人受灾，25.6万人紧急避难。河南省表示：“仅直接经济损失就达12.2亿元人民币(约2239.65亿韩元)。”河南省的面积是韩国的1.6倍，人口数达1亿左右。 　随着此次河南省雨灾越来越严重，此前一直与中国内地针锋相对的蔡英文也以自己的名义发表了慰问信息。据台湾《中央通讯社》当天报道，蔡英文表示，“向不幸遇难的人及其家属表示哀悼，希望灾区早日恢复正常生活。” 　郑州有委托生产苹果iPhone手机的台湾富士康工厂，预计iPhone的供应也会受到影响。富士康在郑州运营3家工厂，约35万人力投入到90条生产线上。在这里生产的iPhone手机占全世界iPhone手机数量的一半以上。《华尔街日报》援引富士康职员的话报道说：“20日下午，富士康三家工厂全部停电数小时，并暂时断电。”一名职员向WSJ表示：“工厂内的水涨到大腿处，人们急忙搬运生产设备和库存品。” **舍曼会见文在寅，“将与中国深入讨论对朝政策”**

正在韩国访问的美国副国务卿温迪·舍曼22日会见了韩国总统文在寅。他表示：“将在访问中国时就对朝政策进行深入讨论。”据悉，美中两国最近在经济问题之外，又重新启动了将焦点放在朝鲜问题等外交悬案等方面的定期对话渠道，因此，围绕朝美重启对话问题，美中将进行怎样的磋商备受关注。舍曼将在访问韩国后，于25日至26日访问中国，并与中国外交部长王毅举行会谈。

文在寅当天在青瓦台接见舍曼，表示“希望今后为重启朝美对话积极努力”。对此，舍曼答道：“期待朝鲜对美国的对话提议早日作出响应”，“希望就对朝政策共同进行紧密协调的努力。”

特别是，舍曼表示：“韩流明星‘防弹少年团’的《允许跳舞（Permission to Dance）》在全世界很受欢迎，但因为韩美很合拍，所以不需要‘允许’。”分析认为，这是在强调巩固的韩美同盟非常重要。

据外交消息人士称，中美最近重新启动了美国特朗普政府时期中断的例行外交渠道。消息人士透露说：“在这个渠道中，中美两国可能会以提出合作必要性的朝鲜问题等外交悬案为中心进行磋商。美中两国开始采取将经济和外交分开看待的‘双轨’处理方式。”

# **自由亚洲电台：“中国将被关押在沈阳收容所的50多名‘脱北者’遣返朝鲜”**

自由亚洲电台16日报道说，中国政府14日将关押在辽宁省沈阳收容所的50多名“脱北者”遣返朝鲜。据悉，中国原计划从4月份开始将这些人送回朝鲜，但由于朝鲜担心出现新冠患者，多次拒绝送回，所以被推迟。据自由亚洲电台透露，被遣返朝鲜的这些人在朝鲜将很难避免被处以极刑。

中国有关部门14日通过丹东边境海关，将被关押在沈阳收容所一两年左右的“脱北者”送往朝鲜。据报道，2辆巴士分运载“脱北者”，数十名公安从一早开始在海关周围站岗，不让人们拍摄遣返场面。

被遣返朝鲜的50多人中，还有朝鲜士兵和空军飞行员出身。据悉，一名30多岁的女“脱北者”与一名中国男子结婚，生有一个12岁的儿子，还在中国赚了很多钱。一名消息人士表示：“这名女性是第二次被遣送回朝鲜，因此生死难以预料。丈夫为了救妻子曾想行贿，但行不通。”据自由亚洲电台透露，沈阳收容所内除了当天遣返朝鲜的50多人以外，还有更多的“脱北者”。

随着为防新冠疫情而关闭的丹东海关当天正式开通，此前滞留在朝鲜的华侨和朝鲜贸易代表部有关人员等98人也来到了中国。

# **“致死率80%”，中国首例人类感染猴B病毒致死病例**

在中国和美国接连出现了来自于猴子身上非常罕见的病毒的感染患者。

　据中国官方媒体《环球时报》17日报道，在北京一家专门从事非人灵长类研究机构工作的53岁兽医因感染“猴B病毒”而接受治疗，最终于5月27日死亡。据调查，该兽医在今年3月解剖2只死猴子时感染了该病毒，并在一个月后出现了恶心和呕吐的症状。《环球时报》报道称，这是中国首次有人感染这种病毒。据说，接触过该兽医的人中没有新的感染者。

　猴B病毒是疱疹病毒的一种，如果被感染的猴子咬伤或抓伤，或者猴子的分泌物溅到人的眼黏膜等部位，就可能会被传染。这是一种从人到人都能感染的病毒，致死率高达70%至80%。

　据美国《华盛顿邮报》(WP)16日报道，美国得克萨斯州北部达拉斯发现了罕见传染病“猴头疮(Monkeypox)”病毒患者。达拉斯县保健当局当天表示，已确认最近访问非洲尼日利亚的达拉斯居民感染了该病毒。

　该患者于本月8日从尼日利亚拉各斯乘坐美国达美航空公司的飞机，经亚特兰大于9日抵达达拉斯。美国疾病控制和预防中心(CDC)正在掌握在飞机上与患者接触的人，并与相关人员取得联系。达拉斯县当局表示：“为了预防新型冠状病毒肺炎(COVID-19)疫情，要求佩戴口罩，因此该病毒在飞机或机场通过飞沫扩散到其他人的危险很低。”

　猴头疮病毒与牛痘病毒相似，于1958年首次被发现。如果人类感染，会出现与流感相似的症状，淋巴腺会肿胀，脸部和身体会出现大范围的疹子。美国疾病控制和预防中心表示，每100人中就有1人死亡。人类感染事例在1970年刚果民主共和国首次得到确认。在美国，2003年从非洲加纳进口的猴子身上开始感染，共出现了47名感染者。

# **中国与朝鲜靠近，“称美国应对数十年威胁朝鲜反省”**

中国罕见地强烈谴责美国称：“美国应该反省数十年来对朝鲜施加的威胁和压力。”

据中国外交部4日消息，中国负责外交事务的国务委员兼外交部长王毅前一天出席了在清华大学举行的第九届世界和平论坛，并在会上同时表示：“韩半岛核问题在最近30年里一直拖延，几经波折。”他强调：“通过（与朝鲜）对话和谈判和平解决是基本原则，韩半岛无核化与和平体制的构建并行是正确的道路。”

王毅表示：“韩半岛的事情是中国家门口的事情。为了韩半岛的稳定，中国将一如既往地发挥建设性作用。”对于美国国务院朝鲜事务特别代表金圣为试探朝美对话重启的可能性而访问韩国等最近美国的动向，他表示：“支持所有有助于韩半岛和平与稳定的言行。”

他在当天的演讲中就西方对新疆维吾尔自治区和香港人权问题等的谴责再次阐明了“不要干涉中国内政”的立场。他就台湾问题表示：“台湾是中国领土不可分割的一部分。”他同时警告说：“推进祖国和平统一是中国政府一直坚持的方针，美国部分势力支援台独势力是非常错误和危险的。”对于日本政府决定向海洋排放核电站污染水，王毅强调：“日本政府应充分倾听国际社会的合理关注，在与有关国家和国际机构进行磋商之前，不能随意向太平洋排放污染水。”

清华大学运营的“世界和平论坛秘书处”主办的世界和平论坛成立于2012年。这是中国唯一由非政府机构主办的与国际安保相关的全球论坛。

# **北韩“新冠疫情封锁边境”时隔一年重启与中国的贸易**

朝鲜以中朝边境地区为中心，最近与中国进行了有限的物资交流。为了安抚因生活必需品不足而不满的居民，朝鲜官方在维持边境封闭状态的情况下开始了“非正式”交易。此前，北韩因对新型冠状病毒肺炎(COVID-19)扩散的恐惧，封锁边境超过一年。也有人分析说，这种交易重启是在11日《中朝友好合作条约》签署60周年之际，两国收紧合作体制的信号。

　据韩国政府消息人士2日透露，上月底，以北韩-中国贸易据点城市中国辽宁省丹东市为中心，部分物资来往。据消息人士介绍，交流利用卡车等，通过陆路进行小范围的物资交流。

# **习近平：“谁妄想欺负中国，必将头破血流”**

在7月1日中国共产党成立100周年之际，中国国家主席习近平（照片）表示：“中华民族受欺凌的时代已经结束了。任何外来势力妄想欺负我们，必将在14亿多中国人民用血肉筑成的钢铁长城面前碰得头破血流！”他表示，“同台湾实现祖国完全统一是新的义务”，如果美国等西方国家介入台湾、香港等问题，将采取强硬应对。有人分析认为，这是对1月份上台以后一直对中国施加强大压力的美国拜登政府的一种宣战。

习近平当天在北京天安门广场的建党100周年庆祝大会上表示，“中华民族是世界上伟大的民族，有着5000多年源远流长的文明历史，为人类文明进步作出了不可磨灭的贡献。”“任何人都不要低估中国人民捍卫国家主权和领土完整的坚强决心、坚定意志、强大能力！”习近平身穿毛泽东在1949年宣布中华人民共和国建国的天安门城楼上时一模一样的灰色中山服登场，他**提出了**“全面建设社会主义现代化强国”的第二个百年目标。他表示，既然实现了第一个百年目标——实现了“小康社会”，现在就要努力在国际社会上加强霸权国家的地位。

# **即将迎来建党100周年，北京处于“半戒严”状态**

台湾《自由时报》23日报道，即将迎来7月1日中国共产党建党100周年纪念日之际，中国实际上已进入半戒严状态。分析认为，中国是在准备盛大的100周年烟花庆典和各种文化演出的同时，彻底切断人员和物资流动等，以防止对共产党和习近平国家主席长期执政的不满。

中国当局从21日开始对进入首都北京的所有快递进行了两阶段专门调查。首先用X光检查机检查发送地快递公司寄往北京的所有快递，然后贴上完成安全检查的标志。之后在快递到达的北京当地再次进行检查。当局表示，到7月1日为止，将展开第二阶段全面调查。

很多人担心会因此造成送货延误。在网络购物和快递发达的中国，经济发达的南部广东省、上海、深圳等地生产的产品大部分都是通过快递进入北京。有人指出，过去2、3天就足够，但是在实施第二阶段专门调查之后，将需要一周以上的时间。部分消费者表示不满说：“通过网上预订让北京郊区的餐厅往市内送餐实际上也被切断了。”

北京公安部门从上周起以市内出租屋等为对象，展开了住宅访问调查。各派出所派遣的检查员亲自上门确认当初申报的居住者和实际居住者是否相同。据悉，特别加强了对居住在北京的外国人的调查。

除此之外，北京等9个主要大城市禁止放飞无人机、模型飞机、风筝、气球等所有飞行物体。

被称为“中国暗行御史”机构的中共中央纪律检查委员会最近在网站上介绍了20世纪30年代背叛共产党的人的悲惨下场。纪律检查委员会表示，“不背叛党的承诺不能只停留在宣誓上”，向共产党员施加了绝对忠诚的压力。这表明了要根除反共产党行为的意志。

# **拜登要求中情局“重新调查中国实验室新冠病毒起源”**

新冠病毒的起源争论持续的情况下，美国总统拜登表示美国情报当局的判断出现分歧，指示进一步调查。

拜登26日在声明中表示：“今年3月，我已向中央情报局等情报部门指示，分析新冠病毒的起源，是动物与人类的接触引起的，还是实验室事故引起的。”

拜登表示，最近听取与此相关的报告的结果，情报当局并没有得出明确的结论。情报机构中，两处认为动物起源说，另一处认为实验室泄漏说，但均表示可信度低或中等。拜登指示情报当局，为了接近明确的结论，90天内要再次进行分析报告。

美国公开没有得出结论的情报部门活动是非常罕见的事情。彭博社分析说：“这表明美国还没有排除中国实验室泄漏的可能性。”拜登在声明中对中国施压称：“为了让中国配合完全、透明、以证据为基础的国际调查，美国将同全世界具有同样想法的同盟一起行动。”

对此，中国反驳说：“美国企图把新冠病毒溯源政治化。”中国驻美大使馆27日在网站上谴责说：“某种政治势力无视必须对抗新冠大流行的紧急需要，沉迷于谴责游戏。”但内容中并未言及拜登的声明。

世界卫生组织曾发表报告说，新冠病毒来自中国武汉病毒研究所的可能性很低，但最近《华尔街日报》报道说，武汉病毒研究所可能是新冠病毒的发源地，从而使围绕起源的争议愈演愈烈。

# **中国驻韩大使：“韩国如果没有提及台湾就好了”**

中国驻韩大使邢海明（照片）26日表示，如果韩美首脑会谈联合声明中“根本没有台湾海峡和南海相关内容就好了”。在韩国政府就台湾问题阐明立场称是“非常原则性的内容”、出面灭火的情况下，中国却把韩国提及台湾问题本身视为问题。邢海明表示，“为了中美两国的和解，希望包括韩国在内的中间国家发挥良好作用”，要求韩国推行中美均衡外交。

邢海明当天出席MBC时事节目时，就韩美声明中没有使用“中国”一词一事表示：“我认为（韩国）付出了很大努力”。但他同时表示，“虽然没有使用中国这一词，但似乎有针对（中国）的部分。”他表示：“韩中建交时就已经明确承认台湾是中国的一部分。我认为，关于南海问题，我们也可以同周边国家合作解决。”

另外，针对提及“四方安全对话（美国、澳大利亚、日本、印度四方协议体）”，邢海明表示：“对于一个国家或者几个国家建立的秩序，我们的想法有些不同，美国有着总是制造小集团包围中国的倾向，如果（韩国）多考虑我们（中国）的立场，将非常感谢。”他是在主张，韩国不能参与“四轴安全对话”。

韩美两国通过首脑会谈不仅在安全领域，还在经济、尖端技术领域扩大了同盟关系。对此，邢海明表现出警戒心。他说：“中国从来没有像美国一样，以不把（本国的）技术给别人的方式进行控制过，一次也没有，（中国）在10年内要从海外进口22万亿美元（商品），我认为，韩国判断国家利益，利用（中国的）这种市场发展经济，才是正确的。”

但是，邢海明当天的发言并没有超出韩美首脑会谈以后中国的抗议程度。对于中国称“不要玩火”表示不满，韩国政府以“韩中特殊关系”出面灭火，考虑到这一点，估计中国决定暂时观望韩国政府的动向。据悉，因为中国认为，如果连同韩国也转向美国，将很难阻止美国纠集盟国对中国进行牵制。邢海明就习近平主席的访韩计划表示：“就目前状况而言，不能明确奉告。”

# **选择只打两个月的中国联赛的金软景，下一个目标是美国还是欧洲？**

“排球女帝”金软景(34岁•照片)的中国之行是“可预测的未来”。但她的下一个目的地究竟会是哪里，仍然无法预测。

　20日，据负责金软景的代理业务的Lianat透露，金软景正在与中国联赛球队上海光明优倍队协商合同问题。预计合同期为一个赛季。

　熟悉排球转会市场的相关人士表示：“在北汽俱乐部方面，也对金软景表现出了兴趣。但据悉，金软景更喜欢2017~2018赛季曾效力过的老东家球队”，“金软景现在30多岁，中国联赛日程短，体力负担小，准备下一个舞台也很容易。”

　金软景在4年前进军中国联赛时也曾表示，“如果比赛场次多，体力上也会感到负担。”在上个赛季，光明优倍队只进行了12场比赛。相反，金软景从去年堤川•MG新村金库杯大赛到V联赛冠军争夺战共参加了41场比赛。再加上李在英和李多英双胞胎姐妹因学校暴力事件而失去战斗力，在心理负担下不得不参加比赛。

　虽然2021~2022赛季中国联赛日程尚未出来，但新赛季最长两个月内所有日程都很有可能结束。金软景很有可能在完成中国联赛日程后，于明年1月初再次进军欧洲。当然她也有可能进军今年职业联赛拉开帷幕的美国舞台。

　中国女排联赛结束后，回到兴国生命也不是不可能的事情。金软景在与海外联赛球队签约时虽然是自由签约选手(FA)身份，但在国内，只有在兴国生命队完成下赛季日程的40%(14场比赛)以上才能获得FA资格。

# **香港单方面关闭驻台湾办事处，中断交流**

# 香港单方面关闭了在台湾起到外交公馆作用的经贸文化办事处。还有分析说，此次决定是在中国大陆和台湾关系不断恶化的情况下做出的，受到中国政府影响的香港实际上可能考虑的是中断与台湾的交流。 据香港《南华早报》等19日报道，香港政府前一天在其主页上表示，暂时停止运营驻台湾经济贸易文化办事处，但没有对中断的原因和恢复运营的时间等没有作出说明。对此，台湾负责中国大陆事务的大陆委员会表示：“香港政府单方面做出了决定。对此深表遗憾。” 香港驻台湾经济贸易文化办事处旨在促进香港和台湾之间的经贸交流，成立于2011年。台湾也在香港设立了起到同样作用的经济文化办事处。这些机构虽然不是正式的政府机构，但起到了保护侨民等实质性的领事馆作用。 与香港和台湾运营这种常驻机构不同，中国大陆和台湾在对方根本没有常驻机构。香港之所以能够采取与中国不同的政策，是因为在中国国家主席习近平执政之前，香港比较遵守“一国两制”原则。但是，习近平2013年执政以后，相比“两制”更加强调“一国”原则，因此情况开始发生变化。特别是，去年实施了香港《国家安全法》，今年还改革了香港选举制，因此，香港的中国化现象日益明显。 **中国探测器继月球后着陆火星……向全球展示“太空崛起”**

中国成功将用于火星探测的无人移动机器人成功着陆到火星表面，成为继美国和前苏联后，第三个在火星表面成功着陆的国家。为探测火星表面而发射可移动的探测器，是继美国之后的第二个。

据中国新华社报道，中国探测器“天问一号”此前一直在火星周边轨道上盘旋，携火星探测车“祝融”于韩国时间15日上午8时18分在火星北半球的乌托邦平原南侧成功着陆。中国国家主席习近平在贺电中表示：“迈出了中国星际探测征程的重要一步，实现了从地月糸到行星际的跨越。”

去年7月发射的“天问一号”于2月10日安全进入火星轨道后，在近三个月的时间里一直绕着轨道寻找着陆机会。“天问”意为“问天”，取自中国战国时代楚国诗人屈原的诗。“祝融”是中国古代神话中火神的名字。

当天成功着陆火星的“祝融”火星车是长2.6米、宽3米、高1.85米、重240公斤、6个轮子的移动机器人。其设计是利用约90个火星日（1个火星日按地球计算是24小时37分）时间进行探测。在“祝融”号探测器上首次安装了可以探测地下100米的雷达设备。中国计划在此次探测中，在火星表面寻找水和冰的痕迹，并分析土壤和岩石成分。经过两次挑战，中国终于成功实现火星探测。中国曾在2011年试图和俄罗斯一起探测火星，但以失败告终。习近平称赞道：“你们勇于挑战追求卓越，使中国在行星探测领域进入世界先进国家行列。”

中国在最近几年里不断加快探测火星和月球、建设独立空间站等“太空崛起”速度，跃升为威胁美国的太空强国。继2019年人类首次在月球背面着陆探测飞船“嫦娥4号”后，去年12月又将另一艘探测飞船“嫦娥5号”发射到月球，采集月面土返回地球。

上月，中国在本国发射体“长征5B”号上搭载中国独立太空站“天和”的首个构造物飞往太空。如果2024年完成任务的国际空间站（ISS）关闭，“天和”将成为唯一的空间站。

2016年，中国领先美国率先发射世界第一颗量子通信卫星“墨子”号，在2400公里远的距离上成功实现无线量子密码通信。

美国正在警惕中国进入太空。本月9日，美国宇航局就“长征5B”号失去控制并坠入地球，导致部分残骸落入印度洋一事表示：“中国没有忠实履行对太空碎片负责任的国际标准。”美国宇航局还以局长比尔•尼尔森的名义发表声明对此批评。外媒解释说，在这样的矛盾背后，存在着争夺太空霸权的竞争意识。

# **中国重达22吨的运载火箭残骸有可能周末坠落地球**

中国“长征5B”火箭的残骸将于周末左右进入地球大气层。韩美军方决定，虽然预计坠落地点不包括韩半岛，但为应对突发情况，加强相互合作体系。

韩国空军太空情报状况室与美国太空司令部联合太空作战中心7日举行视频会议，决定将继续共享有关预计在8日或9日前后进入地球大气层的火箭残骸途径的监视信息。韩美认为，考虑到“长征5B”的大小，它不会在大气层中焚烧干净，而是会坠落地球。据悉，韩美军方认为残骸将坠落在大西洋，但不排除残骸进入大气层时脱离原轨道的可能性。

中国为了建设独立的空间站，4月29日发射了核心模块“天和”，但运载火箭“长征5B”却陷入无法控制的状态，22.5吨的残骸每天从280公里上空坠落1至2公里左右。

韩国军方暂时决定利用美国太空司令部的监视资产共享残骸轨道情报。据悉，考虑到残骸向韩半岛上空移动的最坏情况，也将充分利用正在进行战斗力化的“电子光学卫星监视体系”和“高输出激光卫星追踪体系”。

# **布林肯：“中国采取越来越有攻击性的行动，想成为支配世界的国家”**

美国国务卿安东尼·布林肯当地时间2日抨击中国称：“中国的行动越来越压制和具有攻击性。”他表示，美国对华政策的核心是对抗中国，维护以规则为基础的国际社会秩序，再次强调了与同盟之间的合作。

布林肯当天在接受CBS电视台时事节目《60分钟》采访时表示：“我们看到中国在国内的行动更加压制，在海外的行动也更为攻击性。”也就是说，中国是具备挑战或削弱以规则为基础的国际秩序的军事、经济、外交力量的国家。对于“中国的目标像是什么”的提问，他回答说：“中国似乎相信自己能够、必须成为统治全世界的国家。”但当主持人问道“美国是否正在走向同中国的军事对峙状况”时，他划清界限说：“达到这种状况或就算是走向这种局面，都严重违反美国和中国的利益。”

布林肯还表示：“美国总统拜登实际上对中国采取的包括夺取知识产权在内的一系列行动感到担忧。”他将中国当局对新疆维吾尔族的人权侵害称为“种族灭绝”，直接将矛头指向中国的人权问题。他表示：“我们没有可以不处理中国问题的充裕。无论是敌对的部分，还是竞争的、合作的部分，（同中国的）关系确实存在复杂的方面。”

当主持人提及中国的国内生产总值将于2028年超过美国时，他表示：“打造富国的核心取决于将人力资源和将人力资源潜力极大化的国家能力。从这一点看，我们（比中国）的地位更高。”此外，他还重申了将与拥有同样价值观的同盟及伙伴国家共同合作的立场。

# **“新义州火车站拆除遮布……”朝中边境有解除封锁迹象**

据悉，朝鲜与中国的贸易最快将于本月末恢复。在这种情况下，在朝鲜新义州车站正在陆续发现拆除列车遮布等解除朝中边境封锁的迹象。

据“美国之音”广播电台24日透露，卫星照片分析结果显示，去年10月设置在朝鲜新义州车站一带的列车遮挡幕于3月31日突然被拆除。民间卫星照片企业Maxar科技在3月16日发行的卫星照片中，确认了设置在新义州车站的约400米长的隧道形状遮布。但在本月照片中，该遮布已经消失。据推测，遮布是为了保护长期放置的列车不受雪、雨等影响而设置的。

据悉，与朝鲜新义州相对的朝中贸易据点城市——中国辽宁省丹东市和附近的朝中接壤地区即将发往朝鲜的大规模物资正在等待中。在丹东市火车站，有人看到停靠着一辆写有平壤郊区地名“西浦”、装有物资的货物列车。

通过船舶进行的朝中贸易已于3月重启。据中国海关总署统计，朝鲜3月从中国进口了化肥等价值1297万美元的物资。熟悉朝中关系的消息人士25日表示：“3月从中国进入朝鲜的大部分物资都是通过船只运进的。”据悉，最快从27日开始，装载援朝化肥的列车将开往新义州。

# **美国表示“完全履行联合国对朝制裁”的当天，中国敦促“韩国努力放宽制裁”**

美国表示韩美日三国安保室室长强调“完全履行联合国安理会对朝鲜制裁决议”的当天，中国要求韩国“为解决朝鲜合理的安全关切而努力”。中国一直将“朝鲜合理的安全忧虑”用在保障朝鲜体制和放宽对朝制裁等方面。

美国白宫当地时间2日在马里兰州安纳波利斯海军士官学校举行的韩美日三国安保室长会议结束后发表的联合媒体声明中强调：“三国安保室长一致认为，朝鲜等国际社会完全履行联合国安理会决议非常重要。二国将为防止核扩散和在韩半岛加强（对朝）威慑并维护和平与稳定而继续合作。”声明还表示：“三方就朝鲜核及弹道导弹计划的担忧达成了共识，同时重申了为实现无核化而通过韩美日三方协调合作解决这些问题的意志。”即，在强调对朝遏制的同时，向朝鲜和中国施压，要求遵守对朝制裁。

青瓦台国家安全室室长徐薰在会议结束后接受记者采访时表示：“韩美日就朝核问题的紧迫性和外交解决的必要性达成了共识，一致认为应该继续努力使朝美谈判早日重启。”但通过韩美日三国协调，白宫发表的声明中并没有包括尽快重启朝美协商。

另一方面，中国外长王毅3日在中国福建省厦门举行的韩中外长会谈上向韩国外长郑义溶表示：“一定要维护韩半岛和平与稳定，切实解决朝鲜合理安全关切。各方应为此作出积极努力。”王毅还表示：“中国希望与韩国在5G移动通信、半导体集成电路等领域加强合作，成为高质量的合作伙伴。”据悉，把半导体作为牵制中国的国家安保焦点问题开始讨论半导体问题的美国，在韩美日安保室长会议上主要讨论了维持3国半导体供应链的问题。

# **2500亿韩元！现代建设机械承揽公司史上在华最大订单**

现代建设机械在中国承揽到了历届最大规模的订单。

　现代建设机械29日表示，最近从中国顾客公司接到了2200多台建设装备订单。这是现代建设机械在中国开展业务以来规模最大的订单。5.5~85吨级的挖掘机和轮式装载机等，合同金额达2500亿韩元。考虑到去年现代建设机械在中国的订单规模为7800多台，此次订单达到了去年整年业绩的30%。现代建设机械计划到5月为止供应此次承揽的装备。

　最近中国市场为了克服去年因新型冠状病毒肺炎(COVID-19)疫情而导致的经济停滞，中国政府层面正在大规模进行社会间接资本(SOC)投资。现代建设机械认为中国建设装备市场进入繁荣期，本月初在中国市场推出了大型挖掘机、水陆两用挖掘机、打桩机、钳子等13种新产品。

　随着铁矿石价格逼近每吨170美元(约20万韩元)等原材料价格走强，期待包括中国在内的新兴市场对建设装备的需求增加。现代建设机械预测，今年中国的建设装备需求将比去年增加8%，达到31.5万多台。

# **中国决定对接种本国疫苗的外国人简化签证发放程序**

中国政府决定对接种中国产新冠疫苗后通过香港入境的外国人简化发放签证的程序，可免除提交核酸检查证明书等。

据中国官方媒体《环球时报》14日报道，中国外交部驻香港特派员公署12日在官方网站上表示，两次接种中国疫苗或申请签证14天前接种一次的人在申请中国签证时，可以不提交核酸检查证明书和健康及旅行记录证明书。

目前，中国规定，外国人进入中国时，须提交72小时内新冠核酸检查阴性证明等。对于以参加葬礼或探亲等人道主义目的申请签证的情况，如已接种中国产疫苗，就将其分类为简化手续对象并发放签证。该措施将从15日开始执行。

《环球时报》解释说：“让接种中国疫苗的人省去提交核酸检查阴性证明的做法表现出对中国产疫苗的信心。”但很多人分析说，这一措施仅针对中国疫苗接种者简化签证发放程序，目的是为了扩大中国产疫苗的普及。也就是说，从为入境中国的相当数量的需求来看，这是为了增加这些人在中国的疫苗接种数量而采取的措施。

但是，由于中国国内对中国产疫苗的不信任，疫苗接种人口在2月底仅占总人口的3%左右，因此有人对此举的实效性提出了疑问。

# **拜登政府推出第一个安保方针，“团结同盟应对中国”**

美国拜登政府当地时间3日公布了包含集中针对中国的“国家安保战略中间方针”。拜登政府就任40多天后公布这一方针，包含着今后美国外交安保政策的方向和蓝图。

美国国务卿安东尼·布林肯当天在白宫发表指针之前表示：“中国是21世纪最大的地政学考验。中国是唯一拥有挑战稳定、开放的国际体系的经济、外交、军事、技术力量的国家。”他还表示：“中国正在向以我们希望的方式让全世界运转的所有规范和价值发起挑战。”

布林肯表示：“我们离开的地方，已被中国占据。”他强调，将通过加强同同盟及伙伴国家的合作予以应对。他将同盟称为“使力量倍增的美国独特的资产”，并强调，“我们团结一心的力量让中国无法无视。”

# **习近平在党内指示：“做好应对灰犀牛与黑天鹅的预案”**

中国国家主席习近平提及“灰犀牛”和“黑天鹅”，强调中国面临的风险。

据中国中央电视台报道，习近平1月28日在中共中央政治局集体学习时表示：“要善于预见和预判各种风险挑战，做好应对各种‘灰犀牛’和‘黑天鹅’事件的预案。”“灰犀牛”是可以预料到、但容易忽视的危险，“黑天鹅”是指虽然发生概率较低、但一旦发生就会带来巨大冲击的危险。

习近平还表示：“面对世界百年未有之大变局，要深刻认识错综复杂的国际局势的影响，为中国的发展创造良好环境。”这一讲话的背景是，有预测认为，美国拜登政府上台以后，两国对峙格局还会持续下去。在特朗普执政时期恶化的中美矛盾目前已经超越贸易领域，扩大到外交、安全领域。1月28日，美国权威政治专门媒体《政客》甚至刊登了一篇匿名投稿，内容为“应该通过中国共产党领导层的分裂，来谋求更换习主席”。

习近平每次强调危机时都会提及“灰犀牛”和“黑天鹅”。新冠疫情扩散的去年2月，他与印度尼西亚总统、马来西亚总理等人通电话时表示：“在防疫方面，如何预防‘灰犀牛’和‘黑天鹅’，是全世界的难题。”此外，中国公布28年来最低经济增长率的2019年1月也曾表示：“国际形势难以预测，周边环境复杂敏感。应该高度警惕‘黑天鹅’，预防‘灰犀牛’。”习近平还把防范“灰犀牛”和“黑天鹅”等危险比喻成战争。

习近平在这次政治局学习中还说：“今年是十四五规划开局之年，也是共产党建党100周年，保障经济社会发展非常重要。”

# **习近平穿上羽绒服亮相，相关服装公司股价猛涨10%**

中国国家主席习近平18日身穿2022北京冬奥会官方赞助商安踏集团下属企业的防水羽绒服登场，一天后，在香港股市上市的安踏集团股价上涨了约10%。

　据香港《南华早报》报道，习近平18日访问北京郊区的冬奥会设施时，身穿深蓝色的“始祖鸟（Arcteryx）”大衣。始祖鸟原来是属于芬兰企业Amer Sports的品牌，2019年被被称为“中国耐克”的安踏集团收购。始祖鸟产品在网络卖场中通常以700∼2000美元（约80万∼220万韩元）的价格销售。习主席所穿产品的具体价格尚不清楚。

　安踏在中国体育用品市场上，继美国耐克（市场占有率23%）、德国阿迪达斯（20%）之后，以15%的占有率位居第三。习主席在2017年也穿着安踏夹克登场。在2018年平昌冬奥会上登场的中国国家队也把安踏产品作为队服穿着。有分析认为，习主席为了支援本国服装公司，故意公开了这一形象。事实上，19日安踏集团的股价上涨了近10%。

# **美国为什么警惕中国的“一带一路”？**

　　“亚洲人会思考吗（Can Asians Think）？”

　这一挑衅性的句子是1993年基绍尔·马夫巴尼写的书名。新加坡李光耀公共政策研究生院院长马夫巴尼当时通过此书警告说：“世界趋势正在发生变化，亚洲教给西方的东西更多。”亚洲人会不会思考？这并不是殖民主义思维，而是指现在应该研究亚洲人有什么想法。

　20多年间亚洲地位的变化，正在经济数据中得到证明。去年11月，韩、中、日、澳等15个国家签署的《区域全面经济伙伴协定（RCEP）》拥有22亿人口，国内生产总值（GDP）总规模达26.2万亿美元，被称为“世界上最大的自由贸易协定”。作者断言，今后世界地形图将不再是“美国第一”，而是“亚洲第一”。书中同时详细介绍了鲜为人知的亚洲各方面。

　第一步是古代亚洲文明。该书简略地整理了被以希腊文明等以西方为中心写就的世界史阴影笼罩的印度、西亚、东亚文明。随后，该书提示了亚洲主要政治、经济、社会、文化相关的基本知识和事件。该书还涉及了澳大利亚和俄罗斯为什么很早就开始“亚洲化”、美国为什么对中国的“一带一路”项目保持警惕等具有时宜性的主题。

　　“从亚洲的观点来看，过去20年是乔治·W·布什的无能力、贝拉克·奥巴马的无诚意、唐纳德·特朗普未能预测的时代”以及“与西方的误解不同，亚洲并不是向着以中国为中心移动”的观点非常有趣。超出东亚，还可以一览印度和东南亚等广阔的亚洲现状，是该书的又一优点。但同时也让人感受到该书把对亚洲无知的西方人设定为读者的局限性。

　作者出生于印度，在美国和欧洲担任国际关系专家，目前定居在新加坡。英文书名为《The Future is Asian（未来是亚洲的）》。

# **“时隔44年带回月球表面样本”，中国一片欢腾**

“全体14亿中国国民正在一片欢腾。”

　中国无人月球探测器“嫦娥五号”采取月球表面样本后于17日凌晨安全返回。这是继1976年前苏联“月球24”机器人探测之后时隔44年再次采取月球样本。中国主要媒体一致表示祝贺，称“中国进入了宇宙强国的行列”。

　中国官方媒体新华社当天报道说，“中国在月球成功采取样本，成为继美国和前苏联之后第三个成功探测月球的国家”，并强调，“虽然是第三个，但是‘嫦娥五号’与以往探测有着很多不同的地方，拥有多个‘世界第一’的头衔。”

　据新华社报道，“嫦娥五号”着陆在人类从未去过过的熔岩平原“暴风雨海洋”。该地区的岩石和土壤与其他地区相比，是最近才形成的，因此有人期待对研究月球火山活动有所帮助。中国的英文官方媒体《环球时报》报道说：“继去年1月‘嫦娥四号’首次登陆月球背面后，‘嫦娥五号’也首次探测月球新区域，延续了‘首次’的称号。”

　采取的样本重量达到2公斤，这也是新记录。这相当于前苏联“月球24”所提取的330克的6倍左右。“嫦娥五号”之所以能够采取到大量样本，是因为它大大减少了返回地球时使用的燃料。过去美国和前苏联利用登月飞船自身拥有的燃料返回地球。但“嫦娥五号”由于利用了在月球轨道上与返回舱对接的方式，所以仅靠相对较少的燃料就可以返回。

　新华社报道说：“‘嫦娥五号’在月球轨道上和返回舱的对接工作是最好的宇宙技术”，“为了高速移动的宇宙飞船之间的安全对接，研究人员进行了661次练习。”

　中国今年7月发射了火星探测器“天问一号”，此次又有“嫦娥五号”完成任务后返回，正在有条不紊地实施“太空崛起”计划。中国去年通过27次火箭发射，将66个飞行体送入太空，今年也将40枚以上的火箭发射进入太空。中国明年也计划最少向太空发射80枚以上的火箭。　

　中国国家主席习近平当天祝贺“嫦娥五号”安全返回，并以党、政、军领导人的名义向参与该项目的研究人员发去了贺电。习近平表示：“通过此次项目，中国航空航天技术取得了重大进展。”

# **中国官方媒体：“韩式泡菜和中式泡菜是完全不同的食物”**

最近，中国媒体因引发“泡菜宗主国”争议而遭到批评，中国官方媒体也表明立场称：“韩式泡菜（Kimchi）和中式泡菜（Paocai）是完全不同的食物。”此举似乎是后退了一步，将此次争议解释为“单纯的翻译错误”。

中国官方英文报纸《环球时报》当地时间9日就两种食物的差异进行了说明：“围绕韩式泡菜和中式泡菜的争论不过是翻译错误引起的‘无聊之举’。”“虽然两种食物用中文都叫‘泡菜’，但制作方法和材料完全不同”，“发酵食品泡菜（Kimchi）代表韩国料理，而泡菜（Paocai）则源于四川省腌制蔬菜”。该报在报道中将泡菜明确标注为“Kimchi”，而以往中国媒体则没有使用“Kimchi”这一表述，而是统称为“泡菜”。

《环球时报》还报道称，应诚信女大教授徐敬德的要求，中国门户网站百度百科辞典8日删除了“韩国泡菜源于中国”的内容，并解释说“中国专家们认为这（泡菜起源争议）是‘无聊之举’”。报道还声称：“只要是百度用户，任何人都可以登记、编辑、修改（百科全书），这是这一系统问题引起的意外插曲。”报道还称，“韩国的泡菜文化拥护者拿着单纯的翻译错误，批判称‘（中国）想偷窃我们的文化’，由此引发了不和”，将争议的责任推给了韩国方面。

引发此次泡菜争议的中国官方中文报纸《环球时报》当天也报道说：“Kimchi与泡菜不同。”

围绕泡菜宗主国的争论始于上个月29日《环球时报》就中国向国际标准化组织注册腌制蔬菜法一事的报道。报道称：“中国的泡菜产业在国际市场上成了标准”，“事实上，韩国是泡菜宗主国的主张已经有名无实。”尽管韩国的泡菜和中国的泡菜是完全不同的食物，却引发了两国就泡菜标准规格展开神经战的误会。此后争议不断扩大，但中方没有做出特别的表态，仅过9天就说是“翻译失误”。

世界泡菜研究所战略企划本部长赵贞恩（音）强调：“这次情况暂告一段落，但应该长期做好应对中国‘泡菜工程’的准备”，“代表韩国的饮食文化遗产——泡菜，需要战略性的保护。”

# **美国媒体：“朝鲜今年向中国走私价值4000亿韩元的煤炭”**

据分析，因联合国制裁而被禁止出口煤炭的朝鲜今年1月至9月向中国出口了价值最多可达4.1亿美元（约合4452亿韩元）的煤炭。有人指出，朝鲜船只还堂而皇之地悬挂国旗航行，显示对朝制裁存在很大的漏洞。

　《华尔街日报》以对美国国务院官员的采访和国务院提供的卫星照片为基础，当地时间7日报道说：“朝鲜船只在过去一年里向中国宁波-舟山地区直接运送了数百次煤炭。”具体是在8月12日拍摄的卫星照片中，4艘悬挂朝鲜国旗的煤炭运输船在中国宁波-舟山港附近海域与中国船舶停泊在一起，分析认为是进行了非法转运。

　据美国政府掌握的情况，朝鲜今年前9个月共出口了410万吨煤炭。假设以每吨80美元至100美元的价格销售，出口额将达到3.3亿至4.1亿美元（约合3583亿至4452亿韩元）。《华尔街日报》报道称，以美国为首的国际社会虽然对朝鲜非法转运采取了共同监视态势，但朝鲜船舶还是挂着国旗航行。此前，朝鲜曾采用动用外国籍船舶、更换船舶名称、关闭船舶位置识别装置等回避手段，但现在却明目张胆地非法转运。

　美国方面批评说，这是中国无视联合国制裁而发生的事情。美国国务院高层官员说：“自2017年联合国安理会通过制裁决议以来，首次亲眼目睹了从朝鲜直接向中国运送煤炭的巨大变化”，“中国和朝鲜不再为逃避制裁监视而努力伪装。”

# **美国议会就牵制中国构想制定2万亿韩元国防预算**

美国议会在包含2021会计年度国防预算案的《国防授权法(NDAA)》法案中新设了“太平洋地区构想(Pacific Deterrence Initiative)”项目，并分配了22亿美元(约2.38万亿韩元)预算。事实上，美国新设了针对中国的国防预算，这预示着明年1月拜登政府上台后，美国将继续采取强硬的对华政策。

　 据《华盛顿邮报》(WP)6日(当地时间)透露，参众两院提出的NDAA法案中，关于印度太平洋地区的部分增加了“太平洋遏制构想”项目。法案规定，国防部长要制定包含地区内美军驻扎兵力现代化及强化方案的计划，并与印度太平洋司令部进行协商，在2021年2月15日之前向议会提交包含上述内容的报告书。法案明确规定：“这一构想是为了强化美国在印度太平洋地区的威慑力、国防力和准备态势，并为了确保区域内同盟及伙伴国家，执行优先考虑的活动。”

　 如果该法案得以实施，美国为了提高在印度太平洋的威慑力，很有可能不缩减驻韩美军，而是维持下去。另一方面，也有分析认为，美国向韩国施压，要求韩国加入反华联合阵线。

# **中国4年以来首次批准韩国游戏服务，限韩令解除or只是个例？**

中国政府时隔约4年首次向韩国游戏颁发了“版号(中国国内游戏服务许可)”。同时出现了解除对韩国游戏的“限韩令(韩流限制令)”信号弹的期待和仅限于一次性的负面预测。

　3日，据韩国游戏开发商Com2uS透露，2日中国国家新闻出版署在网站上发布公告称，已向Com2uS的移动角色扮演游戏(RPG)《魔灵召唤：天空之役》下发了版号。2014年6月向全世界推出的《魔灵召唤(Summoners War)》是Com2uS海外销售额中约占80%的人气游戏。

　Com2uS为了2016年末在中国市场展示《魔灵召唤》，申请了版号发放。但随着2017年围绕萨德(THAAD)部署问题韩中矛盾不断扩大，中国一直非正式地限制韩国游戏等文化产品的流通。特别是游戏，在2017年初最后一次发放之后就一直没有再发放版号。Com2uS相关人士表示：“因为是很久以前申请的，所以完全没有想到，事前也没有联系。”

　对于中国政府突然发放版号，韩国国内游戏业界期待称，作为世界最大的游戏市场之一的中国市场大门是否再次开启。包括即将进军中国市场的Nexon手机游戏《地下城与勇士》在内，利用过去在中国人气颇高的知识产权(IP)的游戏的出口有望在不久的将来实现。由于这种期待，3日国内股市上Com2uS比前一天上涨6.19%，Wemade(5.75%)、Pearlabyss(14.11%)、NetMarble(3.59%)、NC软件(2.21%)等游戏公司的股价表现强势。

　但也有人慎重地表示，认为此次措施使中国市场重新打开还为时尚早。为了确信中国已经全面撤销了限韩令，应该继续发放追加版号。韩国游戏学会会长俞正铉(音)指出：“很难期待中国会陆续发放版号”，“但是，韩国相关政府部门和游戏业界应把此次版号发放作为杠杆，今后更积极地致力于解决版号问题。”

# **英国广播公司（BBC）：“中国泡菜成为世界标准？……中国媒体纯属误报”**

针对中国将腌制蔬菜“泡菜（Paocai）”的制作法登记到国际标准化组织一事，中国官方媒体报道似乎称中国制定了泡菜的国际标准，英国广播公司指出中国的主张，认为这是“误报”。

英国广播公司当地时间11月30日在题为《泡菜，令韩中文化矛盾发酵》的报道中称：“中国误报说（其）关于韩国传统饮食泡菜的制作方法得到了国际认可，韩国对此进行了反驳”，“（泡菜争议）是韩国和中国最近发生的文化纠纷。”

此前，中国官方媒体《环球时报》10月月29日报道称，中国制定了“泡菜国际标准”，并声称“中国的泡菜产业已成为国际市场的标准，事实上，韩国是‘泡菜宗主国’的主张已经有名无实”。相关报道将“中国泡菜（Paocai）”和“韩国泡菜（Kimchi）”形容为同样的食物，把登记为国际标准的“Paocai”包含韩国泡菜一样进行宣传。

对此，韩国农林畜产食品部立即反驳道：根据2001年联合国国际粮农组织下属的国际食品法典委员会（CODEX）的规格，韩国泡菜被设定为国际标准，“Paocai”不能解释为韩国泡菜。”

英国广播公司也援引农食部的主张，介绍了韩国泡菜的特性和腌泡文化。“属于辛辣腌制食品的泡菜虽然在中国以‘Paocai’的名称供应，但是中国也有相同名称的固有食品，国际标准化组织（ISO）文件中虽然指出这一食品规格‘不适用于韩国泡菜’，但部分中国媒体却进行了与此不同的报道。”

报道还介绍说，“韩国泡菜是将蔬菜用盐腌制后放入调料和发酵的海产品并放入缸中保管，每年制作泡菜的腌制泡菜已被联合国教科文组织列入人类非物质文化遗产名录。”报道说：“韩国泡菜需求量大，需要从中国进口，但由于中国的严格限制，韩国的出口之路实际上已被堵住。”

英国广播公司还介绍了最近韩国和中国之间文化矛盾的事例。10月防弹少年团（BTS）在6·25战争70周年之际，因对韩美关系发展做出的贡献而获得范弗里特奖，并使用了“（韩美）两国共同经历的苦难历史”的表述，引起中国网民集体抗议，称“无视中国的牺牲”。11月，中国演员许凯在中国社交媒体微博上发表题为《韩服是中国服装》的文章，引起了争议。

# **王毅提出的“韩中日自贸协定”，目前外交和经济风险仍然很大**

最近巡访韩国和日本的中国外交部长王毅主张推进“韩中日自由贸易协定”。王毅部长在与外交部长康京和的会谈中表示，“要积极推进韩中日自贸协定”，在与日本外相的会谈中也强调了这一点。中国《环球时报》报道说：“韩中日自贸协定有效弥补了东北亚地区合作制度的不足。”韩中日自贸协定在取得期待效果的同时，也存在很大的经济、外交风险，因此要慎重对待。

韩中日自贸协定谈判始于2013年，但由于对核心领域的分歧和外交矛盾，谈判停滞不前。占世界经济24%的三个国家如果消除商品和服务壁垒，可能会产生促进经济增长的效果，但是其危险也很大。三国作为世界性制造业强国，在半导体、汽车、钢铁等核心出口产业中有很多重叠的部分。韩中自贸协定于2015年生效，但进一步的开放令人担心有可能对韩国产业造成打击。韩国和日本也为了保护本国产业，很多领域都不愿互相开放。韩中日自贸协定此前曾多次讨论过，但未能取得进一步进展，也是因为这个原因。

政治外交层面的含义也必不可少。在美国政权交替之际，中国提出地区合作和经济贸易问题，其目的在于牵制美国在东北亚地区的影响力。此前，韩中日自贸协定谈判因韩日历史问题产生矛盾，中国也挑韩国“萨德（末段高空区域防御系统）”的毛病而中断，受到的政治外交影响很大。

美国当选总统拜登推进召开“民主首脑会议”，开始牵制中国。拜登在竞选活动中曾表示，“世界民主国家聚集在一起，加强民主体制，对抗逆行（民主）的国家”，最近出现了正式动向。随着美中两国拉拉扯扯的力量增强，韩国的“战略模糊性”再次面临考验。在美中霸权竞争中，韩国是不想成为“开背虾”就行，还是通过战略性利用提高国家利益，这取决于政府的行动。

# **回国的韩国职员确诊感染新冠病毒... SK海力士中国工厂现已停产**

被派往SK海力士中国重庆工厂的韩国职员被确诊感染新型冠状病毒肺炎(COVID-19)，中国当地工厂暂时中断生产。中国重庆市政府下令对在相关工厂工作的2700多名全体员工进行新冠肺炎专项调查。

　29日，据中国官方新华通讯社及企业界透露，SK海力士职员A某结束在中国为期1年零6个月的工作后，回到仁川机场接受新冠病毒检查，并于28日被判定为阳性。据悉，A某被确诊后，重庆市立即对A某曾工作的SK海力士工厂进行流行病学调查，并实行现场控制及消毒等紧急措施。

　财界相关人士表示：“重庆市及SK海力士等机构推测A某是没有特别症状的无症状感染者”，“A某下榻的所住的重庆某假日酒店暂停营业，封闭管理，对酒店所有员工及11月25日以来的所有入住客人进行追踪和核酸检测。”

　停止运转的SK海力士重庆工厂是进行半导体包装等后工序的地方。一般来说，半导体生产工厂一旦电力供应中断，恢复生产线需要数天时间，损失额也很大。相反，此次停止运转的包装工厂在市政府防疫措施完成后，有望立即恢复正常运转。但就目前而言，恢复生产的时间尚不明确，如果工厂长期停产，将很难避免生产受阻。　

SK海力士方面当天表示：“将积极协助中国政府，尽最大努力尽快恢复生产。”

# **“李小龙依然活在我们心中”，李小龙诞辰80周年纪念活动接连举行**

在迎来香港传奇动作明星李小龙(1940~1973年•照片)诞辰80周年之际，中华圈接连举行各种活动纪念李小龙。

　28日，据中国官方英文报纸《环球时报》报道，在李小龙父亲的故乡、李小龙幼年时期暂时居住的广东省佛山，为了纪念1940年11月出生的李小龙，从本月初开始举行“李小龙诞辰80周年”活动。寻找相似的人比赛、回顾李小龙人生的线上•线下活动等接连不断。佛山当局希望通过这样的活动，使佛山成为中国武术乃至全世界的武术文化中心。

　香港邮政局也以“世界武术中的李小龙的遗产”为主题，开始发行特别邮票。贯通香港中环地区的部分有轨电车上也全面刊登了纪念李小龙诞辰80周年的广告。该有轨电车将运营到明年1月。

　在微博等中国社交网络媒体上，很多网民也分享了李小龙的视频和照片，缅怀他的诞辰日。《环球时报》评价说：“对于很多人来说，李小龙已经超越了功夫明星本身”，“他打破了西方对中国人的固有观念，依然活在很多人的心中。”女儿香农•李(李香凝•51岁)在以父亲英文名字“布鲁斯•李”命名的网站“布鲁斯.com”上发表文章回忆说，“虽然父亲去世了，但他的生活继续给我们带来新的灵感。”

　李小龙出生于美国旧金山，出生3个月时移居中国香港。成年之后他出演了23部电影，成为了20世纪70、80年代全世界男性大众文化的代表人物。33岁时夭折这一点也助长了世界粉丝团现象。

# **傲慢的中国，施压韩国不要站在美国一边，却不解除“限韩令”**

中国外交部长王毅昨天结束了为期3天的韩国访问回国。王毅在短暂的日程中，与韩国总统、国会议长、执政党实权人物一一会面。在韩中外长会谈中，他还犯了迟到25分钟的失礼行为。虽然以他访韩为契机，两国间讨论了广泛的合作方案，但在解除中国“限韩令”等核心问题上没有任何进展。

王毅访韩的目的似乎完完全全为了在美国政权交替之际探索周边国家的氛围和外交管理。中国方面列举了10项内容，称之为两国之间的协议，但其中并没核心议题。王毅在习近平主席访韩事宜上提出了“完全控制新冠疫情”的条件。对于萨德（THAAD，末段高空区域防御系统）问题，他称“应该妥善处理”，并施压要求拆除，而对于要求解除其报复措施“限韩令”，他只是表示“希望持续进行沟通”。

中国一直受到美国特朗普政府全方位攻势的困扰。大部分观测认为，在拜登时代，美中之间的这种紧张局面还会持续下去。作为中国，为了不让美国的盟友韩国和日本站在反华战线上，正在致力于周边国家的外交。此次，中方也再次强调韩中日经济一体化，邀请韩国加入针对美国驱除中国信息技术、企业的“全球数据安全倡议”。

拜登时代的中美关系现在还很难断言。虽然特朗普总统是例外，但选举时猛烈抨击中国的美国总统们在当选后都侧重于扩大与中国的贸易。以规范和价值为导向的拜登式国际主义很有可能不会采取特朗普时期贸易战等粗暴方式，而是通过严密的制度包围网来勒紧中国。像“民主首脑会议”这样的价值同盟构想，会使韩国站在难以避免的选择的十字路口。

王毅说：“这个世界上不仅仅只有美国。”这可能是在向韩国施压，不要站在美国一边。东亚地区也不是只有中国。中国只要任性阻止进入本国的巨大市场，同时继续推行拙劣的外交，就不会有立足之地。中国应该知道，为什么每次中国来人访问时，都会出现“耍皇帝敕使派头”的说法。

# **王毅访韩，“世界上不仅仅只有美国”**

正在韩国访问的中国国务委员兼外交部长王毅26日表示：“世界上不仅仅只有美国。有190多个国家，都是独立自主的国家。韩国和中国也是如此。”这可以解释为是在强调韩中合作不应受到美国的影响。对于中国国家主席习近平访韩，他表示“新冠疫情必须得到完全控制”，事实上暗示韩国政府一直推动的年内访韩计划已经无望实现。

王毅当天上午在外交部大楼与韩国外交部长康京和举行会谈后，对记者提出的“此次访韩是否为了中美竞争，要求韩国政府和执政党人士不要站在美国一边”的问题，回答说：“韩中是邻国，应该像亲戚一样经常来往。”王毅表示：“我们（韩中）是战略合作伙伴关系，应该全方位协调和合作。”他在和康京和会谈的开场发言中表示：“我们打算就国际和地区问题进行战略沟通。”韩国外交部负责官员称，他向康京和对在韩国部署的萨德（THAAD，末段高空区域防御系统）表示了担忧。

对于“习近平主席年内访韩的可能性有多大”的提问，王毅指着采访团说：“大家都戴着口罩，重要的是完全控制（新冠疫情）。”意思是说，韩国的新冠疫情正在再度扩散，因此习近平主席难以访韩。

# **来自中国的可吸入颗粒物之灾再次到来，依靠“低姿态”外交，解决将遥遥无期**

 新冠疫情期间似乎一度平静的可吸入颗粒物再次活动猖獗。前天在首尔发布了入秋以来首次超细颗粒物警报，首都圈和忠清地区等中西部地区12日起连续四天，超细颗粒物浓度持续为“糟糕”水平。忠南地区14日和16日两天启动了紧急降尘措施，昨天除光州、全南、庆南和釜山之外的大部分都空气不佳，处于“糟糕”水平。

　 我国的可吸入颗粒物处于高浓度时，80%都来自中国，中国的影响是压倒性的。据韩国环境部门分析，最近特别趋于严重，也是由于乘着偏西风从中国飞来的空气污染物停留在韩国大气中。因新冠疫情一度降低的中国工厂的开工率接近恢复至100%，再加上开始冬季供暖，从中国流入的污染物质的总量再次增加。截至今年9月，全中国的可吸入颗粒物浓度平均下降了11.8%，为中国空气质量部分改善，但这只是新冠疫情带来的错觉现象，来自中国的可吸入颗粒物灾难仍然在破坏韩国人的健康和生活。

　 去年春天，肆虐韩半岛的最严重的可吸入颗粒物事件，来自中国的雾霾解决成为国家面临的问题，文在寅政府似乎在寻求某种根本对策。但之后近两年，并没有取得什么进展。虽说一年里举行了一两次两国环境部长会议和实务磋商讨论对策，但这里出来的对策并没有超出信息交流和学术研究的水平。

　 文在寅总统在去年可吸入颗粒物事件引起民心沸腾后，成立了由前联合国秘书长潘基文为委员长的全国性对策机构——国家气候环境会议，但之后没有取得任何进展。中国仍然声称“首尔的可吸入颗粒物是当地排放的”，不承认本国的责任，韩国政府甚至连正式抗议也没有提出，进行了不咸不淡的处理。

　 当然，如果中国的产业和环境政策没有发生划时代的变化，就不会有从根本上阻止来自中国的大气污染物的来袭。但是，如果中国大幅加强东部沿海工厂的污染物排放标准，并果断投资降低排放总量，那么目前席卷韩国的可吸入颗粒物将大幅减少。可吸入颗粒物事态是关系到韩国国民能否呼吸的生存问题，应该放弃低姿态应对，强烈敦促中国制定根本性的减少可吸入颗粒物的对策。

# **文在寅总统签署加入中国主导的RCEP协定**

文在寅总统15日最终签署了世界最大规模的多边自由贸易协定——《区域全面经济伙伴关系协定（RCEP）》。

文在寅当天在青瓦台通过视频举行的“第四届RCEP峰会”上签署《RCEP》时表示：“新冠疫情的挑战、贸易保护主义的扩散、多边体制危机面前，以年轻、充满活力的东盟为中心，签署了世界最大规模的自由贸易协定。我们把守护自由贸易价值付诸了行动。”RCEP是由韩中日、东盟十国、澳大利亚、新西兰等15个国家参与的世界最大规模的自贸协定。从贸易规模、国内生产总值、人口方面来看，约占全世界的30%。其基本宗旨是在协定成员国之间降低关税门槛，确立系统的贸易和投资体系，促进贸易。

也有分析认为，事实上，以加入中国主导的RCEP为契机，在中美矛盾中韩国政府的外交算法将变得更加复杂。美国前总统奥巴马为了阻止中国的膨胀，从2010年开始推进环太平洋经济伙伴关系协定（TPP），中国为了突破这个包围圈，从2012年开始构筑RCEP，鼓励韩国参与。TPP在2017年因拒绝多边体制的特朗普总统宣布退出而搁浅，但在2018年以日本、澳大利亚为主轴，改名为CPTPP后生效。美国当选总统拜登从候选人时期开始就暗示要回归CPTPP。对此，青瓦台相关人士表示：“中国与其他国家一样，是参与RCEP的15个国家之一”，“如果觉得有必要，我们也可以进入CPTPP。”

# **BLACKPINK不戴手套触摸熊猫幼崽引发争议**

前段时间以“盲目的”爱国主义攻击K-POP组合防弹少年团(BTS)的中国网民和官方媒体，这次又开始攻击女子组合BLACKPINK。BLACKPINK本月3日在YouTube等社交网路媒体(SNS)上公开了与中国珍稀动物大熊猫接触的视频，她们主张没有像自己所希望的那样重视大熊猫。

　官方英文杂志《环球时报》6日表示：“BLACKPINK成员在接触韩国爱宝乐园出生约3个月的熊猫幼崽‘福宝’，以及2016年来到韩国的‘华妮’(韩国呼名：爱宝)时，都化了浓妆。有时连手套和口罩都不戴。”接着，该媒体补充表示，“熊猫幼崽的免疫力很弱，如果化浓妆或不佩戴防疫装备的话，可能会很危险。大熊猫是中国的‘国宝’，即使在海外出生，到了一定时期也要回到中国，所以属于中国。”

　部分网民在微博上发表文章称“回收韩国的大熊猫吧”。全世界动物园的大熊猫都是由中国租借的。

中国野生动物保护协会在5日的声明中主张：“韩国偶像组合成员在没有戴手套的情况下触摸了国宝熊猫，对此不能置之不理。”该协会还补充说，已正式向爱宝乐园和BLACKPINK方面提出抗议，要求他们立即停止非法接触大熊猫的行为，并删除出现相关场面的视频。

　同一天，爱宝乐园在Instagram上登载了BLACKPINK的视频预告片，并公告称“正式拍摄是在负责兽医和饲养员的监督下，经过彻底的消毒和防疫后进行的”。在中国方面提出抗议后，爱宝乐园删除了该视频。

# **现代汽车：“到2025年为止，将在中国普及3000辆氢电卡车”**

现代汽车将在中国普及氢电卡车，加速扩大中国氢能经济事业。

　现代汽车4日表示，与中国首都北京所在的“京津冀”地区、经济城市上海所在的“长江三角洲”地区的企业接连签订了构建氢能经济生态系统的业务协议(MOU)。

　首先，现代汽车于上月27日与上海电力股份有限公司、上海舜华新能源系统有限公司、上海荣华融资租赁有限公司等长江三角洲地区内的主要能源及金融公司签订了MOU。其目标是，建立氢气加氢站及氢气生产设备，推进氢燃料电动汽车运营金融服务，到2025年为止，在该地区普及现代汽车3000辆以上的氢电卡车。

　本月4日，现代汽车又与中国钢铁集团安泰科技股份有限公司及河钢集团河北钢铁工业技术服务有限公司签订了MOU，在京津地区构建氢气加氢站和示范运行氢电卡车，并计划在2025年之前普及1000多辆氢电卡车。现代汽车商用事业本部长(副社长)李仁哲表示：“我们计划在中国市场构建不仅销售氢燃汽车，还运营氢气汽车租赁、加氢站等整个氢生态系统的事业生态系。”现代汽车在瑞士初期也为了减轻对购买氢电卡车费用感到负担的物流企业的负担，以收取使用费租借车辆的形式来普及氢电卡车。

　中国氢电汽车所用的氢原料“附生氢”(石油化学工程的副产品)非常丰富，由于持续扩充产业基础，卡车需求一直占据市场，氢电卡车市场发展潜力非常充分。不仅是中央政府，地方政府也加快了氢燃料电动汽车的普及、构建氢气生产和加氢所需的基础。

　为了配合政府提出的到2030年为止普及100万辆氢电汽车的目标，现代汽车上月也提出了到2030年为止向中国出口2.7万辆氢电卡车以上的目标。这超过了同期向北美和欧洲分别出口1.2万辆和2.5万辆的目标。

# **斗山工程机械在中国生产的挖掘机累计突破20万台**

斗山工程机械有限公司3日表示，在中国国内生产的挖掘机累计突破了20万台。这是公司自1994年10月进军中国市场后时隔26年达成的纪录。

　在中国烟台拥有工厂的斗山工程机械在2001年累计生产超过了5000台。当时，日本建筑机械企业抢先占领中国市场，但随着攻击性的市场营销和最新产品的推出，自2000年代以后，在中国的海外建筑机械企业领域占有率跃居首位。从2000年代中期开始年产量超过1万台，2010年年产量超过2万台，并逐渐增长。

此次20万台的累计生产记录在进军中国的海外建筑机械公司中尚属首次。除了中国当地企业，斗山工程机械在挖掘机市场的第三季度(7~9月)占有率达到了22.8%。斗山工程机械目前正与美国建筑机械企业卡特彼勒(Caterpillar)就市场占有率第一、二的位置展开激烈竞争。

　斗山工程机械去年在中国销售了1.5万多台建筑机械。今年，虽然因新型冠状病毒肺炎(COVID-19)疫情导致市场停滞，但截止到第三季度共销售了1.4348万台，与去年业绩几乎持平。斗山工程机械为了适应中国挖掘机市场的增长趋势，计划继续推出新产品和特殊装备。另外，为了中国地区社会的发展，还推进支援落后地区青少年学业和改善教育环境的社会贡献活动。

# **BTS表示“韩国战争是韩美苦难的历史”，中方吹毛求疵**

世界级K-POP组合防弹少年团(BTS)在获奖感言中提到“6.25战争”，在中国受到了强烈的批评。对于6•25战争只提到“两国(韩国、美国)”，中国有关方面怒气冲冲地表示这是“无视中国”的行为。

　防弹少年团于当地时间7日获得了美国非盈利团体韩国社交协会颁发的“范弗里特奖”。该奖项是为了纪念参加6•25战争的美军第8集团军司令官詹姆斯•范弗里特，从1995年开始每年授予为增进韩美关系做出贡献的个人或团体。前总统金大中、三星集团总裁李健熙、大韩商工会议所等也曾获奖。

　防弹少年团的队长RM(本名金南俊)在发表获奖感言时说：“今年是韩国战争70周年，我们要永远铭记两国(韩美)共同经历的苦难历史和众多男性和女性的牺牲。”该感想晚些时候在中国传开，引起了中国当地媒体和网民的热议。

　12日，中国官方媒体《环球时报》报道说：“在获奖感言中，中国网民对‘两国经历的苦难历史’这一表述感到愤怒。”新浪网、腾讯网等知名新闻门户网站甚至在相关回帖中出现了“无视韩国战争当时中国军人的宝贵牺牲”、“防弹少年团的获奖感想是无视美国侵略和对亚洲的干涉的发言”的意见。

　中国将6•25战争称为“抗美援朝(对抗美国帮助北韩)战争”。尤其是在美中矛盾达到高潮的情况下，今年迎来参战70周年，强调了包含民族主义、爱国主义、英雄主义等意义的“抗美援朝精神”。据分析，中国网民对防弹少年团的过激反应也是出于这一原因。

# **特朗普再次攻击中国，“将永远结束对中国的依赖”**

美国和中国的经济矛盾再次激化。美国总统特朗普指责中国用从与美国的贸易中获得的资金强化军备，并强调，“将结束对中国的依赖”。对此，中国负责外交事务的国务委员兼外交部长王毅立即针锋相对地抨击美国打压中国的信息技术企业，中国官方媒体提及中国出售持有的美国国债的可能性，警告对美国进行报复。

特朗普当地时间7日在为迎接劳动节假日而举行的媒体吹风会上说：“迄今为止，没有一个国家像中国那样占我们（美国）的便宜”，“中国把我们给的钱用在加强军事力量上。”他接着说：“幸亏我加强了我们的军事力量，否则就会被中国超越”，“不管是脱钩，还是我们一直在做的征收巨额关税，我们将永远结束对中国的依赖。”

最近，特朗普政府把华为、抖音、微信等中国的信息科技企业定性为威胁国家安全，采取了强有力的限制措施。特别是，美国正在研究将中国的代表性半导体企业中芯国际（SMIC）列入交易限制企业名单的方案。在金融领域，美国政府宣布了把不遵守美国会计标准的中国企业赶出美国股市的方案等，持续着波浪式攻势。

对此，中国8日在王毅的主导下发表了“全球数据安全”倡议，开始牵制美国。不是盲目地指责其为“安全威胁”，而应该制定具体的规则和标准来衡量。王毅表示，“中国政府不会要求中国企业违反其他国家法律，提供国外数据”，“部分国家以安全为借口攻击领头企业，这是赤裸裸的蛮横行为”，将矛头指向美国政府。

另外，中国官方媒体《环球时报》英文版最近报道说：“中国可以出售相当一部分美国国债。”报道称，目前中国政府持有的美国国债在1万亿美元（约1200万亿韩元）以上，可以逐渐将其减持至8000亿美元。报道还称，在两国发生军事冲突等极端情况下，可以抛售所有持有的国债。中国是美国国债的最大持有国，如果中国大量抛售美国国债，可能会出现美元价值暴跌、利率暴涨等国际金融市场陷入瘫痪的状况。

针对美国要断绝与中国的经济关系的恫吓，中国也在准备自救对策。《环球时报》6日在题为《中国正在寻找可以抵消美国脱钩计划的方案》的专栏中报道说：“中国将背弃对本国敌对的国家，根据目前正在推进的一带一路政策，与欧洲及非洲、亚洲国家形成紧密的经济伙伴关系”，“特朗普政府已着手美中之间的脱钩，中国应该自己开拓自己的命运。”

# **中国报复，关闭美国领事馆，发动“全面战争”**

美国强烈指责中国国家主席习近平是“破产的极权主义的信奉者”，并宣布将全面修改对华包容政策基调。为了报复美国关闭中国驻休斯敦总领事馆，中国政府下令关闭美国驻四川省成都总领事馆。

有分析认为，随着美中贸易纠纷、新冠疫情责任攻防战、香港维护国家安全法的制定等，水平不断提高的中美矛盾升级为全面外交战，两国建交41年来首次面临断交之前的状况。

美国国务卿迈克·蓬佩奥当地时间23日在加利福尼亚约巴林达的尼克松图书馆发表了题为《共产国家中国与自由世界的未来》的演讲。他在演说中表示：“前总统理查德·尼克松曾说，‘我们担心我们是不是让中国向世界开放，制造了怪物'，现在我们正处于这种状况。”他的意思是，自1972年时任美国总统尼克松访问中国后，美国持续了约50年的对华包容政策，成为中国成长为威胁西方世界的最大敌国的契机。　

随后，蓬佩奥表示，“有必要牢记中国共产党的本质是马克思和列宁政权”，“全世界热爱自由的国家应该诱导中国的变化”，要求同盟国共同参与对华施压。对于关闭中国驻休斯敦总领事馆的决定，他声称：“因为这是中国间谍活动和知识产权盗窃行为的中心。”　

中国外交部24日向驻中国美国大使馆通报称：“撤销美国驻成都总领事馆的设立和运营许可”，“美方必须停止驻成都总领事馆的所有业务和活动。”外交部表示：“此次措施是对美国不理智行为的正当而必要的应对”，“也符合国际法、国际关系基本准则和外交惯例。”　

他接着补充说：“中国并不希望看到美国和中国现在所处的状况”，“希望美国立即撤回错误的措施，为两国关系正常化创造必要条件。”

# **研究结果：“中国渔船在朝鲜东部海域捞走价值5200亿韩元的鱿鱼”**

最新披露的人造卫星照片分析结果显示，中国的黑暗船队偷偷进入遭受联合国制裁的朝鲜的东部海域，约2年间非法捕捞了价值5200亿韩元以上的鱿鱼。黑暗船队指的是不发送船舶位置或没有出现在公开的监控系统中的无许可非法船只。这是韩国数据科学家和国际非政府组织主导的国际研究表明的结果。经确认，由于非法的中国捕捞船队，零散的朝鲜渔民被逼到了更危险的远海去作业。

　非营利性民间研究团体“全球渔业监视”和韩国海洋水产开发院、日本水产研究教育机构、美国加利福尼亚大学22日在国际学术刊物《科学》上发表了人工卫星精密分析结果，认为2018年在2017中国渔船在朝鲜东部海域实施了这一世界最大规模的非法捕捞。

○ 国际合作追踪中国黑暗船队

　中国政府数年来因本国渔民的滥捕而受到各国政府和环境团体的指责。尽管如此，中国并没有采取任何措施。海洋保护团体“海洋环境保护组织”、非营利卫星信息分析团体Skytrus、谷歌为形势所迫从2016年开始展开了“全球渔业监视”项目，动用人造卫星追踪35000艘航行在世界海域的渔船。其意图是追踪利用人造卫星和船舶信息追踪滥捕的大型渔船。

　包括“全球渔业监视”首席数据科学家朴在润（音译）在内的研究团队集中监视了2017年和2018年进入朝鲜专属经济水域的鱿鱼捕捞船。他们中大部分是中国近海活动的黑暗船队，据推测是经由南部海域出入东部海域。但实际情况是，一直没有持续追踪和监视他们的方法。朴在润在电子邮件采访中表示：“虽然黑暗船队在韩半岛东部海域朝鲜水域的活动十分严重，但邻近国家之间没有开展合作，非法捕捞活动真正没有得到监督”，“以人工智能和各种卫星数据为基础，选择了把东部海域作为综合性查明黑暗船队捕捞活动的地方。”

　研究团队结合四种卫星观测技术，开发出了在任何环境下都能追踪、监视非法渔船的技术。首先，利用美国卫星影像服务企业“星球实验室”拥有的集群卫星，找到了两艘船用网捕获鱼类的双拖渔船，利用人工智能进行了识别。

　此外，在多云天气里，还动员了3个可以寻找和追踪渔船的卫星雷达来追踪船只的大小、位置和移动路线。最后，通过自动收集并追踪船舶名称和速度等信息，通过监视碰撞的船舶自动识别系统（AIS），追踪了船舶的正式动向。根据追踪结果，研究团队在2017年和2018年分别找到了796艘和588艘双拖渔船。

　研究团队着眼于大部分捕鱿鱼渔船晚上开灯引诱捕鱿鱼这一点，动员了装有高灵敏度的光红外成像辐射仪（VIIRS）的卫星，成功追踪了捕鱿鱼船。通过这种方式，2017年和2018年分别找到了108艘和130艘捕捞鱿鱼的船只。

　共同研究团队通过分析两年间收集的卫星信息，找到了1600多艘中国非法船只。捕获的鱿鱼为16.4万吨，折合成金额相当于4.4亿美元（约合5263亿韩元）。这相当于正式鱿鱼捕获量最高的日本和韩国的整体捕获量。朴在润表示：“这种规模的非法船队占中国全部远洋渔船的三分之一”，“在一个国家的商业船队在其他国家水域进行的非法作业案例中，规模最大。”

○ 零散的朝鲜渔民被赶到了远海

　研究团队发现，船体长度只有10∼20米，只有几个灯泡进行作业的小而零星的朝鲜渔船在俄罗斯沿岸捕获鱿鱼。仅在2018年，这种活动就发现了3000多次。韩国海洋水产开发院研究委员李正三（音译）表示：“在与长50米、用尖端装备武装起来的中国双拖渔船的竞争中败下阵来，朝鲜渔民们似乎因此前往附近的俄罗斯海岸”，“他们乘坐的小型木船又小又恶劣，不适合前往这么远的大海，也很危险。”

　事实上，最近有数百艘朝鲜渔船在俄罗斯或日本海岸附近漂流，部分渔民被发现死亡，研究团队分析认为，这也与中国渔船进入朝鲜水域不无关系。2018年，朝鲜渔船在俄罗斯海域的捕捞活动比2015年增加了约6倍的事实，这一次也被曝光，表明中国黑暗船队的横行霸道越来越严重。

　朴在润表示：“因为中国大规模商业渔船团而遭受损失的零散渔民的事例，在利比里亚等西非国家也有发生”，“如果利用卫星数据和人工智能向国家渔业监督机构提供技术支援，可以提供持续的、公正的捕鱼活动。”

　由于中国黑暗捕捞船队的非法滥捕，东部海域鱼类资源枯竭、海洋生态系统被破坏，也成为问题。2003年以后，韩国和日本的鱿鱼捕获量分别减少了80%和82%，据推测其背后原因就是中国的非法捕捞。朴在润表示：“如果想要管理像鱿鱼一样跨越国家间界线的鱼种，信息共享非常重要”，“期待地区内国家能以数据和科学对待为基础，建立合作管理地区渔业的机制。”

# **“关闭中国驻休斯敦总领事馆”矛盾愈演愈烈**

就美国对中国驻得克萨斯州休斯敦总领事馆采取关闭措施一事，美国总统唐纳德•特朗普表示：“任何时候都有可能追加关闭中国领事馆。” 美方不顾中方的强烈反对和全世界的忧虑，反而提及追加措施的可能性，将对华施压水平提升到了最高潮。

　特朗普总统当地时间22日在白宫举行的新闻发布会上如此表示，“我认为我们关闭的地方（中国驻休斯敦总领事馆）着火了，所有人都说‘着火了’，但我认为他们好像烧毁了文件和档案。”言下之意就是，中国总领事馆可能想删除有关中方在美国国内进行非法活动的记录。

　当天，美国副国务卿史蒂芬•比根在参议院外交委员会以“美国的对华政策”为主题举行的听证会上确认，此次措施是根据特朗普总统的指示进行的。比根副国务卿对中国进行了逐一批评，他说：“中国抢夺美国技术、侵犯知识产权等每个悬案都接连发生纠纷，所以才会采取这样的措施。”

　中国驻美大使馆发表声明批判称，“美国的主张是毫无根据的牵强附会。”中国驻休斯敦总领事蔡伟在接受美国广播公司（ABC）的采访时也毫不客气地批评道：“因美方的决定，受到了很大的冲击。美国某些政客总是在撒谎，请收起那套忽悠的把戏吧。”

# **美中香港矛盾爆发，“退出香港（HK-exit）”大门开启**

当地时间5月29日，美国对香港采取了中断国防物资出口及限制尖端技术出口的措施。这是为了应对中国强行处理香港《国家安全法》，正式着手剥夺香港的特殊地位。一度沉于水底的中美矛盾再度激化，香港的前途陷入激流。

据路透社等媒体报道，美国商务部长威尔伯·罗斯在当天的声明中表示，“美国商务部关于出口许可例外等给予香港特惠的规定已经停止”，“正在讨论是否废除其他（优惠）措施。”这是在香港回归中国的象征性日子（1997年7月1日）即将到来之际出台的措施。美国国务卿迈克·蓬佩奥也在当天发表的声明中表示：“将停止出口国防物资，启动将（军民）双重用途尖端技术限制与中国一样适用于香港的程序。”他还说：“现在，对于管制产品的出口，香港和中国内地不会再分开。”

有人担心，如果美国以此次措施为开端，全面剥夺包括取消对香港适用的关税特惠等在内的特别地位，全球金融资本和人力大举从香港撤出的“退出香港”将成为现实。香港特区行政长官林郑月娥则反驳说：“我们不怕美国的任何制裁。”

中国不顾美国的全方位压力，当天完成了香港国家安全法的制定。据香港《南华早报》报道，中国全国人大常务委员会在全体162名与会者的赞成下，在递交15分钟后，迅速通过了香港国家安全法。香港特区政府将及时将该法纳入香港实质宪法《基本法》附则，并从香港回归日7月1日起正式实施。

如果违反禁止颠覆国家、恐怖主义、勾结外国势力等行为的香港国家安全法，将被处以终身监禁。在反华人士审判中，允许香港特区行政长官提名特定法官。观察人士认为，随着香港国家安全法的通过，香港代表性民主化人士——香港“众志”秘书长黄之锋（24岁）和具有反华倾向的《苹果日报》社长黎智英（72岁）将被逮捕。

# **新冠疫情为何加快美中对立速度？**

 新冠疫情是人类面临的一次非常罕见的生物安全灾难。这是对中美两国的共同威胁，只有中美合作，才能与全世界人民一道，战胜新冠疫情。但自新冠疫情暴发以来，中美关系持续恶化，两国关系似乎距离“新冷战”仅一步之遥。

　新冠疫情正在使中美关系进一步恶化。美国总统特朗普执政后，美国的“对华政策发言系统”出现了历史性的倒退。特朗普政府一直将中国“妖魔化”。这种“中国妖魔化”代表了特朗普政府和极右共和党势力所表现出来的、中国人难以理解的所谓“（因中国而）受害者情结”。这一情结在3年多的时间里成为特朗普政府对华政策的基本基调。新冠疫情本来是美中合作的机会，但遗憾的是，特朗普政府更加强硬地推行所谓的美国利益优先政策，对中国表现出高度对立的情绪。这已经成为向中国施压的主要手段。

　首先，美国国内新冠疫情的严重状况使特朗普政府和美国共和党右翼政治势力所表现出的反华受害者情结变得更加歇斯底里。特朗普政府把新冠疫情看作是进一步向中国施压、在政治和经济上同中国清算关系的机会。

　第二，新冠疫情加剧了美国对中国的担忧。强化了特朗普政府推进的“脱钩”方向的中国政策。美国人认为新冠疫情为中国打开了一个大机会之窗。他们认为，中国试图更加强烈地追求对全球的影响力，并挤掉美国在世界的领导地位。他们认为，加强对中国的科技战争、贸易战争、媒体战争，甚至今后的金融战争，把产业链、供应链、价值网从中国挖走，就是在遏制中国的崛起，也是在保持美国持续的霸权优势。

　第三，新冠疫情成为特朗普政府恣意用来向中国施压的政治工具。如果向中国施压，美国就能把对美国初期应对新冠疫情时表现出来的无能、低效方式的怨恨转嫁给中国。不仅如此，特朗普还能声称，“你们应该憎恨中国人。中国人造就了今天这样可怕的局面。”这已经成为特朗普大选战略的重要组成部分。在美国，中国问题已经被特朗普总统完全政治化。最后，新冠疫情加剧了美国社会的反华、厌华、恐华情绪，特朗普向中国施压的政策在美国国内得到了更多的支持。

　今天，70%的美国民众认为，“中国应对新冠疫情的扩散负责”。新冠疫情激化了美国国内对亚裔居民的种族主义歧视和排斥。多数美国政治精英和国民对中国政策的看法受到新冠疫情的影响，开始回到与20世纪50年代相似的“新麦卡锡主义（将政治反对者攻击为共产主义者的态度）”。理性、温和的中国政策声音不断受到排挤。

　中美关系已经到了两国都需要认真、理性的政策与策略、反思的时刻。在国际体系中，大国兴衰往往带来不可避免的大国间激烈的权力竞争和战略对立。但是，在21世纪的今天，中美的任何“新冷战”的前途，都将损害世界的稳定、和平与繁荣。尤其是，它将给东北亚地区经济发展带来灾难般的冲击。最近，美国的吉米·卡特、比尔·克林顿、乔治·W·布什、贝拉克·奥巴马等前总统一起发出声音，严厉批评了特朗普的内外政策。他们还要求反省美国政策“灾难性失败”的根源。同样，中国政府也有必要在新冠疫情过程中表现出的诸多问题中找到一条促进政治经济改革、加快形象改善的未来道路。只有这样，才能使中美两国继续得到世界上多数国家的理解和尊重。

# **中国外交部：“希望韩半岛稳定”……敦促朝鲜克制**

对于朝鲜16日下午炸毁开城南北共同联络办事处一事，主要外媒一致通过快讯进行了报道，称“韩半岛内部的紧张气氛正在加剧”。中国外交部表示，“希望韩半岛的和平与稳定”，敦促朝鲜保持克制。

　美联社、美国有线电视新闻网（CNN）、《纽约时报》《朝日新闻》等媒体当天下午引用韩国统一部的发言报导了朝鲜炸毁联络办事处的事实，并指出，朝鲜劳动党第一副部长金与正13日在抨击韩国政府没能阻止“脱北者”团体撒放反朝传单时已经预告了这次爆破。

　美联社援引部分专家的话分析说，对于韩国因美国主导的对朝鲜制裁而无法重启经济合作，朝鲜吐露了不快。《纽约时报》报道说：“最近威胁要结束南北和解氛围的朝鲜用极端的方式表达了对韩国的不满。”《华盛顿邮报》报道说，朝鲜最近几周对韩国提出了越来越尖锐的指责，并预测朝鲜破坏联络办事处会迅速激化矛盾。《朝日新闻》预测说，联络事务所对文在寅政府来说象征着对朝政策的成果，这必然会成为巨大的打击。

　中国外交部发言人赵立坚在当天的例行记者会上就朝鲜炸毁联络办事处一事表示：“朝鲜和韩国是同一个民族”，“中国作为邻国，一贯希望维护韩半岛的和平与稳定。”日本官房长官菅义伟表示：“将继续与美国、韩国等紧密合作，收集和分析必要的情报，关注局势，同时全力进行警戒和监视。”

# **北京疾控中心官员：“北京集体感染原因可能是欧洲进口鲑鱼”**

源自中国北京南部新发地农水产品批发市场的新冠疫情再次蔓延至北京以外地区。中国政府提出，从欧洲进口的鲑鱼等很有可能是沾有新冠病毒后流入中国。

　据中国国家卫生健康委员会15日透露，14日一天，北京新增确诊病例36例。新发地市场11日出现首例患者后，4天内北京共确诊79例患者，北京16个区中有8个区出现确诊患者。

　北京市14日对76499名市民进行新冠病毒检测，进行大检查，预计新增确诊病例将快速增长。国务院副总理孙春兰14日担心，“北京新冠疫情扩散的风险非常大。”

　除北京外，新发地市场的河北省保定市商户一家三口确诊病例，四川也确诊了一名与新发地市场有关的疑似患者。

　中国认为，从欧洲进口的水产品和肉类有可能沾上了病毒进入中国。北京疾病预防控制中心主任杨鹏当天在中国官方媒体中央电视台表示：“病毒怎么来的还不清楚”，“通过基因序列分析，确认了（新发地市场上发现的新冠病毒）来自欧洲方面。”

　他还说：“国外的新冠疫情还比较严重，（国外）在处理肉类和水产品的过程中，可能受到（感染者的）病毒污染而进口。”中国专家主张，由于新冠病毒的生存能力在低温条件下得到极大提高，因此病毒完全有可能在冷链流通过程中流入中国。

　中国社交网站上出现了“又把责任推给海外流入吗？”的批评之声，同时也出现了“看到海外（水产品、肉类）加工职员不戴口罩”的主张。“在湖北武汉传播的病毒也是从境外流入”的阴谋论也再次抬头。

# **美国：中国报复时，可以为韩国做任何事情**

Posted June. 15, 2020 13:17

Updated June. 15, 2020 22:48

- [Font size down](https://www.donga.com/cn/List/article/all/20200615/2091976/1)
- [Font size up](https://www.donga.com/cn/List/article/all/20200615/2091976/1)

[한국어](https://www.donga.com/cn/List/article/all/20200615/2091976/1?m=kor)

- [Share this article on Facebook](https://www.donga.com/cn/List/article/all/20200615/2091976/1)
- [Share this article on Twitter](https://www.donga.com/cn/List/article/all/20200615/2091976/1)


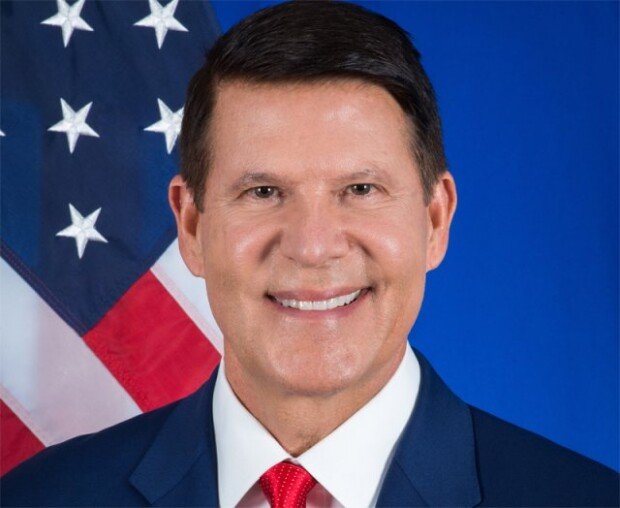


美国国务院主管经济的副国务卿奇斯•克拉克11日(当地时间)表示，如果韩国加入参与和中国对抗的经济构想“经济繁荣网络(EPN)”、“制裁华为制裁”等，届时如果中国采取报复措施，美国将尽一切努力帮助韩国。此外，他还呼吁盟国及伙伴国家加入美国强硬的对华政策并联合起来，“全世界都应该站出来，以对抗中国的威胁和报复”。
　克拉克副国务卿当天在与印度、巴西等5个国家主要媒体举行的电话座谈会上，就美国对中国的经济制裁及政策构想进行了说明，并做出了上述表示。本报是韩国媒体中唯一一家参加座谈会的媒体。
　他就美国要求友邦“共同参与美国对华政策”一事解释说：“不是在中国和美国中间作出选择。虽然选择对谁都开放，但归根结底还是信任哪一方的问题。”
　克拉克副国务卿表示“韩国是全世界的经济和技术力量源泉(Power House)，不仅是美国，也是全世界的大贸易伙伴”，强调了与韩国的经济合作。特别他对三星电子给予了高度评价，称其为“世界三大5G相关企业之一，也是最发达的半导体生产企业”。

# **美国：“一起对抗中国吧”……要仔细研究参与EPN的原则和实际利益**

美国国务院主管经济的副国务卿奇斯·克拉克11日要求韩国参与与中国对抗的新经济版图构想——经济繁荣网络（EPN），并表示，如果中国采取报复措施，“美国将尽一切努力帮助韩国”。他还说：“不是在中国和美国中间作出选择。虽然选择对谁都开放，但归根结底还是信任哪一方的问题。”

　克拉克的发言是要求韩国积极参与其间美国推进的EPN构想和制裁华为等牵制中国的政策。EPN的构想是排除中国，构建由美国主导的新的全球供应链（GSC）。如果说美国针对中国“一带一路”势力范围扩张的安保战略是印度太平洋战略，那么EPN则是经济层面上的牵制中国战略。在这里，还有韩国作为美国的同盟必须参与的大举施压。

　美国针对中国式国家资本主义，强调共享民主主义、人权和透明度、保护知识产权等价值的自由主义资本主义国家间联合的必要性。但问题是，EPN的构想看起来还不是完全成熟的水果。除了中国之外，没有具体说明会形成具有何种约束力的形态，合作内容又是什么。只是突显了美国要将已经成为“世界工厂”的中国排除在外的战略判断。

　对于中国对参加国进行报复的担忧，美国表示“将尽力提供帮助”。如果美国出面的话，韩国也可以充分对抗中国报复，并获得相应的补偿。但是参与EPN不仅是中国报复造成的直接损失，也是要求整个产业结构变化的事项。特别是对于韩国来说，中国报复“萨德”系统当时美国扮演第三者的态度，仍然是一个痛苦的记忆。

　正如美国所提出的那样，作为同盟国的韩国没有理由缺席共享自由和民主价值的国家连带。也可以让EPN成为目前对中国依存度较高的出口和生产多元化的机会。但是，作为韩国的最大贸易国和世界最大市场，完全背离是不可能的，美国也不会要求那样做。要在广泛征求泛政府乃至产业界意见的同时，认真考虑参与的原则、水平、实际利益和国际动向，做好充分准备。

# **韩国造船订单占有率缩小与中国的差距**

一度领先的中国造船订单占有率停滞不前，与追赶的韩国的差距大幅缩小。因为中国试图通过倾注大量本国货单来人为拉高订单量的做法已经达到了极限。

　9日，据英国造船海运市场分析机构“克拉克森研究”公司透露，上个月全世界船舶订货量共计57万CGT（修正总吨），比创下141万CGT的上个月减少了40%左右。按国家来看，上个月中国承揽了27万CGT（13艘，47%），韩国承揽了23万CGT（8艘，40%），日本承揽了5万CGT（2艘，9%）。

　在今年的订单业绩中，中国位居世界第一。但韩国和中国的占有率差距正在逐渐缩小。1至5月，国别累计订单业绩分别为：中国288万GCT，韩国90万CGT，日本49万CGT。4月份韩国和中国的单月订单占有率差距达到55个百分点，但上个月单月订单占有率差距缩小到7个百分点。这是由于中国本国订货大幅减少。从上个月订单量来看，韩国与4月份基本持平，但中国比前一个月剧减73%。特别是，上个月中国的订单中有85%是本国订单，而韩国全部是来自欧洲和亚洲国家的船主订单。

对此，造船业界认为，一直不断接到订单的韩国将在下半年（7∼12月）超过中国的订单量。因为韩国最近与卡塔尔签订了大规模液化天然气运输船建造合同，俄罗斯和莫桑比克也预定了韩国具有优势的大型LNG订货项目。

# **韩贸易协会：中美香港矛盾激化将打击韩国出口**

有预测称，如果美国和中国围绕制定香港保安法的矛盾变得严重，那么将香港作为中转贸易基地的韩国出口也将不可避免地受到打击。

　据韩国贸易协会国际贸易通商研究院29日透露，香港是韩国企业为再出口中国而利用的转口贸易要塞。因为香港对中国内地的接近性很好，并拥有增值税退税、低法人税、各种免税等税收优惠，以及出色的贸易基础设施。因此，从韩国到香港再到中国大陆的物流移动非常活跃。仅在去年，韩国出口到香港的90%以上都再次流入中国。值得一提的是，自1992年起，美国在签证、吸引投资和执法等方面赋予香港特别贸易地位，给予香港待遇。这为香港发展成为亚洲代表性金融物流要冲之地发挥了重要作用。

　但如果美国加强对香港的制裁，不仅各种优惠将消失，而且外资也将大举撤离。因为其作为金融和物流枢纽的各种优势将消失。贸易协会预测称：“如果发生这种事态，只能直接向中国出口，因此各种物流费用增加，为直接出口中国而确保航班等也可能会出现问题。”

　贸易协会某官员说：“香港是我国第四大需求出口地，作为转口贸易基地价值很高，如果香港的金融、服务、物流功能减弱，韩国的出口势必将受到冲击。”

# **中美矛盾中人民币大幅贬值，无法避免流弹的韩国经济**

 中国人民币汇率出现了1美元兑换超过7元人民币的所谓“破七”。中国央行——中国人民银行25日公布了人民币对美元汇率中间价报7.1209元，较前一交易日上涨0.38%。1美元兑人民币7元被认为是心理防线，去年8月中美贸易战激化时发生“破七”后，美国立即将中国列为汇率操纵国。

　中国提高人民币汇率可以解释为，继贸易战争之后，又针对最近美国在香港国安法、新冠病毒发源地矛盾上的波浪式进攻采取了反击措施。一直有观测认为，如果美国施加压力，中国将提高人民币汇率，即通过降低本国货币价值来提高出口竞争力。

　当然，随着中国宣布将实施1000万亿韩元的大规模经济扶持，将增加货币量、扩大财政赤字的方针，人民币价值自然下跌，这一方面是存在的。在两国关系友好时，双方可能会互谅互让，但从中美矛盾几乎走向极端来看，美国很有可能认为迫切需要扩大出口的中国积极引导了货币贬值。

　随着今年1月15日首轮中美贸易协议的达成，去年8月的指称汇率操纵国的问题被解除。人们担心，如果美国以此次“破七”为契机再次对中国亮出指定汇率操纵国的牌，好不容易签署的贸易协议将成为一纸空文，中美矛盾有可能进入被称为汇率问题的敏感局面。

韩国是可能受到美中汇率战争流弹的代表性国家。由于对外依存度高，尤其是偏向美中的贸易结构，不稳定的汇率对于韩国企业和政府来说都是相当困难的因素。眼下，由于与人民币的同步现象，韩元的弱势会对出口竞争力有所帮助，但也有可能导致美元从韩国国内资本市场流出，金融市场发生动摇。

　但即便如此，也并不存在马上有用的应对方案。政府应该密切监控外汇市场，毫不懈怠地在国际上允许的范围内积极介入外汇市场。哪怕是防范这种困难的来临，也必须通过在平时确保企业的对外竞争力和财政健全性，构筑起阻断外部风浪的防波堤。

# **三星电子和SK Innovation等利用“企业人绿色通道”向中国派遣550多名人力**

三星电子等韩国国内主要企业21、22日两天内通过“韩中企业人绿色通道(入境程序简化)”制度向中国派遣了550多名人力。因新型冠状病毒感染症(COVID-19病毒)疫情扩散而被推迟的中国国内工厂增设等项目有望迅速重启。

　三星电子22日向中国西安派遣了300多名总公司•合作企业技术人员，在当地增设半导体第二工厂。在西安经营电池工厂的三星SDI的30多名人力也一同乘坐了该飞机。这是三星电子副会长李在镕自新冠肺炎疫情后首次海外出差造访西安后，时隔3天采取的措施。

　企业人绿色通道是指，仅限于企业人，在出国前后分别接受新冠肺炎检查，一旦出现阴性判定，在当地免除14日义务隔离的措施。这是三星电子首次通过该制度向海外大规模派遣人力。4月份向西安派遣200多名人力时，由于该制度尚未出台，所以要求“特别入境”，在没有采取隔离措施的情况下派遣人力。

　西安工厂是三星电子唯一的海外存储半导体生产基地，总投资规模达150亿美元(约18.45万亿韩元)。

　起亚汽车当天也乘坐包机向中国江苏盐城工厂派遣了100多名人力。此前，SK Innovation于21日用包机派遣了120多名今后会在盐城电池工厂建设现场工作的人员。

# **白宫称“中国是掠夺性经济”，宣布新冷战**

 美国特朗普政府公开了一份报告，内容包括美国今后对中国的战略及政策方向。有评论认为，这一文件事实上宣布了“新冷战”，明确表示将采取公开施压而不是与中国合作，事实上对中国进行封锁等“竞争性对待（competitive approach）”。

　《华盛顿邮报》报道说，白宫当地时间20日向国会提交了以国防部草案为基础编写的《美国对华战略报告》。白宫在其网站上公布的长达16页的报告说：“对中国根本性经济改革及政治开放的期待以失败告终。中国正在推行动摇美国对生命、自由和追求幸福权的基本信念的政策。”报告还宣布：“现在将对中国展开竞争。”

　报告说，“如果证实对华外交是徒劳的尝试，美国将对中国的行动以相应的费用作为杠杆，以保护美国的利益，并加大对中国政府的公开施压”。报告提及中国的恶意行为、投资、意图等，使用了“恶意（malign）”的形容词。报告中甚至出现了“掠夺性（predatory）经济”的说法。

　报告还强调，通过“战略核武器三轴体系（Nuclear Triad）”的现代化，将维持通过力量实现的和平。报告中还包括提前进行极超音速导弹体系、基于网络和宇宙的武器的实战部署等计划。

　作为应对中国威胁的方案，报告提到了加强与区域盟友及伙伴的关系。报告把文在寅政府的“新南方政策”认为是美国应该合作的区域内盟国政策。兰德研究所首席研究员布鲁斯·贝内特对“美国之音”广播电台评价称：“这一报告实际上具有十分浓厚的美国政府宣布对中国实行新冷战的性质。”

　此外，美国强烈反对中国政府制定香港“国家安全法”，这将成为中美矛盾的新导火索。特朗普总统警告说：“如果发生（制定香港《国家保安法》），我们将非常严厉地处理这个问题。”

# **中美全方位霸权战打响……韩国应确立生存战略**

美国和中国以新冠疫情为契机，正式展开了包罗政治、外交、安保、经济等的全面对决。美国白宫当地时间21日在向国会提交的报告中称，“中国经济、政治、军事力量的扩大损害了美国的利益和世界各国的主权”，“有必要从根本上重新考虑过去20年间美国的对华政策”。中美矛盾正在超越对新冠疫情的责任论和经济战，走向长期霸权战。

　世界第一和第二经济大国美国和中国，自特朗普政府上台以来一直露骨地挑起贸易纠纷，但最近围绕新冠疫情的责任，双方发生正面冲突，互相指称“恶劣的独裁政权”“彻底疯了”。在中国全国人民代表大会提出制定“香港国家安全法”后，美国表明了强硬应对方针，战线逐渐扩大。

　美国是韩国的外交安保同盟，但在经济领域，韩国对华出口居第一位，对美出口居第二位。此前，韩国在美中之间一直维持着战略模糊性，但二选一的压力越来越大。美国提出“经济繁荣网络（EPN）”，要求建立把中国排除在外、安保上可以信任的国家共同重新建立全球供应网，向韩国施压要求参与。对于每年向中国华为公司出口10万亿韩元以上半导体的韩国企业也施加了中断出口的压力。对于安保依赖美国、经济依赖中国，维持“安美经中”结构的韩国来说，这是巨大的考验。这不得不让人想起因萨德（THAAD，末段高空导弹防御系统）事件而使经济遭受重创的噩梦。

　但是韩国政府在美中新冷战时代是否做好了应对准备还是个疑问。令人担心的是，韩国政府是否在缺乏后新冠时代全球外交、安保、经济的概论的情况下，只关心南北关系和中国国家主席习近平访韩等。韩国政府事实上宣布废除应对朝鲜炸沉天安舰的“5·24”措施后，美国立即表示“应该与无核化的进展步调一致”予以叫停等，韩美间甚至出现了微妙的冷气流。

　韩国外交部去年成立了应对美中矛盾的外交战略协调会议，但今年以来一次也没有举行。反而，文在寅总统在就任3周年在记者会上强调，不能只期盼朝美对话，应该做南北之间可以做的事情。在经济对中国高度依赖的情况下，如何化解美国的EPN压力，习主席年内的访韩是否会成为进一步的紧张因素，需要政府重新审视外交经济战略

# **美国在两会前夕不加掩饰地指责中国为“疯子”“恶劣的独裁政权”**

在中国最大的政治活动“两会”即将召开之际，美国特朗普政府把对中国的指责调到了最高潮。特朗普总统对中国政府发表的立场大放厥词，称其为“疯子”“傻瓜”，而国务卿蓬佩奥则将矛头指向中国政府，称其为“恶劣的独裁政权”。

　特朗普20日在推特上写道，“中国的某个疯子（wacko）就刚刚杀死数十万人的病毒，指责了除中国以外的所有人”，“对这个蠢人（dope）说明一下吧，导致如此大规模杀伤的，不是别人而是中国的无能”。虽然特朗普没有具体说明是针对谁的，但是很有可能是与新冠病毒有关的中国外交部等主要机关的发言人。

　蓬佩奥在当天上午举行的记者会上说，“自1949年起，中国一直被恶劣的、权威主义的共产政权统治”，“中国无论在意识形态上还是政治上都对自由国家怀有敌意。”他还抨击称，“这一传染病造成9万美国人死亡，3月以来有3600万人失业。因为中国共产党的（应对）失败，全世界遭受了最多达9万亿美元的损失。”

　蓬佩奥当天宣布一项总额为1.62亿美元的新冠病毒受损海外援助计划。他强调，除了与美国国际开发署（USAID）共同承诺提供100亿美元的援助外，还将进一步提供援助。他同时揶揄称，“中国拿出的20亿美元与给世界造成的（损失）费用相比，只是九牛一毛（palary）。我们期待，即使那么一点，也能如实履行。”

　他在记者会上再次祝贺台湾领导人蔡英文再次当选。关于香港，他表示：“对于香港是否（从中国）具有高度的自治，还没有做出判断，正在密切关注现在发生的事情。”他还提到了代工生产企业台积电（TSMC）的对美国投资和商务部发表针对华为的出口制裁案等。

　有分析认为，特朗普政府的这种粗暴的“打击中国”行为是出于美国国内政治考虑。共和党已经制定了把攻击中国作为年底总统选举乃至全国主要州长及议员选举的核心战略的方针。共和党竞选战略小组上个月向党内散发的备忘录中，还包含要求把选举竞争对手作为亲华派或对华软弱的形象进行攻击等内容。

　美国的一再攻击让中国也勃然大怒。中国官方英语电视台CGTN在推特上上传了名为“蓬佩奥可信度测试”的视频（照片），把攻击中国的蓬佩奥描述成小丑。据《人民日报》21日报道，中国国家主席习近平在与孟加拉国总理谢赫·哈西娜通话中表示，“中国反对在妨碍防控传染病国际合作的行为”，委婉地批评了美国的攻势。

# **特朗普：“世卫组织要在一个月内证明独立性，否则美国可能退出”**

美国总统特朗普就新冠病毒发源地问题与中国发生激烈对立，他指出世界卫生组织的亲华倾向，并下最后通牒称，“如果30天内没有改善，将永久中断资金援助或者退出”。

　特朗普18日在写给世界卫生组织总干事谭德塞的四页信函中声称：“如果30天内不拿出证明世卫组织在中国面前保持独立性的改善方案，美国将永久中断资金援助。我们也会重新考虑我们的会员资格。”他还表示，世卫组织今后的唯一出路在于能否展现在中国面前的独立性，“不能拿纳税人的钱向不符合美国利益的组织提供援助”。

　他在记者面前，猛烈抨击世卫组织是中国的傀儡（puppet）。他同时还提出异议称，美国每年向世卫组织支付4.5亿美元的分摊额，而中国只支付3800万美元。

　美国卫生部长亚历克斯·阿扎当天在历史上首次通过视频召开的世界卫生大会上表示，“一个会员国嘲弄透明性义务，给全世界带来了巨大的牺牲。不能再发生这样的事情”，对中国提出了正面批评。美国国务卿蓬佩奥发表声明，批评台湾参加世界卫生大会的计划因中国的反对而流产。蓬佩奥说：“尽管世卫组织总干事有让台湾参加世界卫生大会的法律权限，但因为中国的压力，他没有这样做。总干事缺乏独立性破坏了世界卫生组织的信任和效率。”

　美国白宫国家安全委员会发言人约翰·乌利奥特表示，对中国国家主席习近平视频会议上表示“将向新冠疫情受害国提供20亿美元援助”进行批评。他指出：“这是为了从越来越多国家试图追究中国责任中分散注意力。”

　世界卫生大会将在19日的会议上表决有关新冠病毒展开立调查的决议。在194个世卫组织成员国中，如果得到三分之二的129个国家的支持，就可以通过该决议。澳大利亚、英国、法国、俄罗斯等122个国家已经表示支持，因此有观察人士认为通过的可能性很高。

　中国队也展开了反击。官方的《环球时报》19日称：“这项调查应该包括美国。中国不怕调查。”商务部也表示，“今后5年内将对澳大利亚产大麦征收反倾销税”。澳大利亚产大麦和牛肉的最大进口国中国12日还禁止了部分澳大利亚产牛肉的进口。有分析认为，澳大利亚一直站在美国一边敦促调查新冠病毒，中国因此进行了经济报复。愤怒的澳大利亚也表示将向世界贸易组织提起诉讼。

　处于中美矛盾中心的中国最大的通信设备制造商华为18日在声明中表示：“美国对华为的制裁是恣意的、致命的，最终也将损害美国的利益。”

　两国的矛盾也转移到了美国股市上。据路透社报道，美国第二大证券交易所纳斯达克近日将公布加强中国企业首次公开募股（IPO）资格及会计审计等方面的限制。报道称，中国金融当局也在鼓励中国企业在英国伦敦上市。

# **美驱逐舰接近上海附近海域**

　 不仅是经济方面，美国和中国之间在军事方面的紧张感也在加剧。美国军舰罕见地出现在中国近海，两国还展开了研发最新武器的竞争。

　香港《南华早报》16日援引北京大学下属研究机构“南海战略态势感知计划”发表的消息称，美国海军“阿利·伯克”级驱逐舰“拉斐尔·佩拉尔塔”号（DDG-115）最近出现在距离上海115海里（约213公里）的海上。美国海军太平洋舰队前一天在推特上宣布，“‘拉斐尔·佩拉尔塔’号本周航行在东中国海”，但没有公布具体的日期。

　“拉斐尔·佩拉尔塔”舰此次航行是在中国人民解放军14日开始在黄海的渤海湾进行实弹演习时进行的。预计将有航母参与的中国军队演习将持续两个半月。

　此前的上个月17日，另一艘美国海军驱逐舰“麦克坎贝尔”号（DDG-85）也曾接近距山东威海仅42海里的海域。一个月内，美国军舰两次逼近中国沿岸。

　美国一直在加强军事力量。美国总统特朗普当地时间15日出席太空军旗亮相活动时表示，“我们正在研制迄今为止从未有过的惊人军事装备”，“我听说，这种令人不可思议的导弹比我们现在拥有的快17倍。”他还表示，“因为有我们的敌国，所以这是必须要做的。”美国有线电视新闻网（CNN）报道说，在中国和俄罗斯推进开发高超音速武器的时刻，特朗普总统作出上述发言，可能是针对中国和俄罗斯。

　美国国防部没有就媒体提出的问题给出具体答复。美国防部发言人、空军中校罗伯特·卡佛对CNN表示，“高超音速武器的实战部睹，在技术研究及工程中是最优先事项”，并强调，“在高超音速武器系统开发方面，美国拥有稳定的项目”。

# **美中矛盾加剧，甚至出现“疯狂之举”**

美中矛盾正在越过界限。美国总统特朗普声称，“可能与中国断绝一切关系”，中国媒体则抨击特朗普“精神失常”。甚至有人声称，如果美国断绝关系，就会攻击台湾。美国则瞄准了在美国股市上市的中国企业。

　中国《人民日报》姐妹纸《环球时报》在15日的社论中批评特朗普前一天的发言是“疯狂之举”，并主张，“中国在科学技术以及与此相关的经济、人文、社会科学等美中关系的核心领域，应该做好‘实质性脱钩’的准备”。

　《环球时报》英文版则毫不掩饰地抨击特朗普是“就像被逼入绝境的野兽”“看起来像是疯了。”中国人民大学国际关系学院副院长金灿荣表示：“如果美国单方面断绝关系，中国可以立即（武力）统一台湾。为了维护中美关系，中国没有解决台湾问题。”

　特朗普在前一天接受福克斯商业频道采访时称，他正在“非常强烈”地审视一项方案，准备要求在美国股市上市的中国企业必须遵守美国的会计准则。分析认为，特朗普的意思是，可以考虑对在纽约证券交易所和纳斯达克上市但不遵守美国企业适用的一般会计标准（GAAP）的中国企业进行制裁。同时，美国还在积极诱导半导体、医药品等生产、供应设施前往美国，试图将中国排除在供应链之外。

　在台湾和中国南海，中美军事紧张加剧，偶发性冲突风险增加。据美国有线电视新闻网（CNN）报道，美国最近接连向中国南海派遣军舰和战略轰炸机，加大了对中国的军事压力。

# **在美中矛盾中执意寻求习近平访韩，将失去韩国外交道路**

青瓦台称，中国国家主席习近平前天在与文在寅总统通电话时表示：“今年访韩的坚定意志没有改变。”对此，文在寅表示，在韩中关系上，习主席的访韩比什么都重要。据悉，此次通话是应习近平主席的邀请进行的。

　两国首脑的通话是在美中矛盾再次激化的微妙时期进行的。最重要的是，中国想将韩国拉到自己一边的意图非常明显。韩国也希望习主席尽快访问韩国，以结束萨德（THAAD，末段高空区域防御系统）矛盾。但中国官方媒体却没有报道习主席访韩的相关内容，只是说两国在共同防控新冠疫情时“风雨同舟”。

　围绕新冠病毒起源的美中对立预示着在所有领域都将扩大战事。美国总统特朗普提出“新冠病毒中国责任论”，表示“不仅要加征关税，还要改变偏重中国的全球供应网”，并要求盟国予以协助。这等于是预告了不仅与中国打贸易战，还要重组国际贸易秩序。在这种矛盾局面下，韩国如果站在某一方一边，可能会遭到贸易报复，或陷入外交安保合作框架动摇的危机。

　美中早已开始在全球划拨分边。美国召集包括韩国在内的主要盟国的外交部长，在应对新冠疫情的国际合作中发出牵制中国、跟随美国领导的信息。中国也开始全面应对。中国通过禁止进口牛肉，对站在美国一边要求进行国际调查的澳大利亚进行了报复。可以说，去年美中在使用华为产品问题上强迫国际社会做出选择的事情正在以更大的幅度再次上演。

　此次美中矛盾将持续到年底美国总统选举为止，可能会在不同以往的层面上产生影响。如果美中矛盾只集中于贸易战，韩国可以靠经济体质的加强撑过去，但若出现超越这一层度导致整个国际秩序的变动，则选择余地不大。双方的橄榄枝将像是一把双刃剑，既能扩大韩国游泳的空间，但如果行动出错，韩国将失去立足之地。

　 特朗普只用金钱判断同盟，其提出的防卫费分摊额无理要求，令同盟感到疲劳，这也是现实。我们的选择更加困难。越是这样的时候，越需要有智慧的外交。首先，要明确韩国以韩美同盟为根本的外交政策坐标，在此基础上确定韩中合作的具体水平。只有这样，中美两国才不会以茫然的期待感来向韩国施压，并就真正可以合作的领域进行磋商。

# **《纽约时报》：“中国利用黑客窥伺美国新冠疫苗资料”**

《纽约时报》10日报道称，全世界都在埋头开发新型冠状病毒的治疗药物之际，中国等世界10多个国家正在政府层面动用黑客，展开有关疫苗的信息争夺战。

　美国联邦调查局和国土安全部决定近期发布警告，警告“中国试图窃取美国新冠病毒治疗药物及疫苗开发相关研究资料。企图通过非法手段获取美国的相关知识产权和公共卫生资料。”警告特别指出，中国不是利用情报人员，而是动员留学生、教授、研究员等，试图窃取美国主要大学和民间研究所的新冠病毒情报。

　美国一直怀疑，中国吸引海外人才计划“千人计划”是窃取美国尖端科技计划的一部分。据悉，联邦调查局将在近期访问主要大学，敦促加强保安。纳米技术的世界级学者——美国哈佛大学教授查尔斯·利伯（61岁）1月份也因涉嫌故意隐瞒参与"千人计划"的事实而被捕。2012年至2017年，他在新冠病毒爆发地湖北省武汉理工大学获得数十亿韩元资助，但他谎称“从未收到过参与邀请”。

　特朗普政府一直主张新冠病毒“源自中国”，与中国针锋相对。中国也对此表示强烈反对。因此有人担心，黑客风波会进一步恶化本来就不好的美中矛盾。

　《纽约时报》报道说，韩国、伊朗、越南等国也动员黑客，正在收集其他国家的新冠病毒情报。报道称，特别是“韩国黑客不仅攻击世界卫生组织，也攻击朝鲜、日本、美国政府相关人士的电子邮件，试图收集情报。这表明连美国的盟国也在怀疑美国的统计数据”。

# **美国与中国因新冠矛盾再起贸易战阴云……韩国应制定出口多元化等战略**

韩国综合股价指数（KOSPI）4日再次跌破1900点大关。因为此前一直呈现平稳状态的美中贸易矛盾有可能重新爆发的不安感，外国投资者在韩国股市抛售了1万亿韩元的股票。这给比其他国家更早平息了新冠疫情、带来经济活动也即将正式恢复的期待感的韩国经济泼了冷水。

　美中出现再次爆发贸易战的可能性，是因为美国总统特朗普提及中国对新冠疫情扩散的责任论，暗示可以追加征收1万亿美元（约1200万亿韩元）的关税。

　韩国出口方面，今年4月的业绩比去年4月锐减23%，贸易收支自2012年1月以后、时隔99个月首次出现逆差。如果特朗普总统在11月大选前夕为了形成有利的舆论形势而大举敲打中国，对于因新冠疫情已在出口市场和全球供应链上遭受重创的韩国来说，无疑是又一个超大型不利因素。

　多少有些希望的是，在防控新冠疫情的过程中，韩国的整体国格有所提高，经济潜力在世界市场上也得到高度评价。例如，由于期待今后非面对面活动增加，三星电子和SK海力士两家公司在世界市场上占据70%以上的存储器半导体DRAM，4月份平均价格创下了39个月以来的最大涨幅。与半导体一样，在另一个主要出口商品之一的汽车领域，也有消息称现代汽车在越南市场超过日本丰田，占据了销售第一位。

　即使如此，我们面前的不安因素也更多。世界经济衰退才刚刚开始，中美贸易矛盾至少将持续到美国大选结束的11月。越是这样，我们越要必须做可以自己做、必须做的事情。眼下的工作是积极促进消费，搞活内需经济，解除各种限制，鼓舞企业的士气。长期而言，为适应贸易保护色彩更加浓厚的“后新冠时代”的出口环境，韩国应努力提高内需比重，并加快加大努力，使出口市场从以美国和中国为中心向东南亚、印度、欧洲等多边化发展。

# **美国竭力宣称“新冠病毒武汉研究所起源说”，特朗普称“中国可怕的失误”**

围绕新冠病毒的发源地，美国和中国之间的矛盾正在走向爆发之前。美国总统特朗普和国务卿蓬佩奥以刺激性措辞提出“中国责任论”，并施压要求进行调查，中国对此强烈抗议，认为这是“政治秀”。

　据美国有线电视新闻网（CNN）等媒体报道，特朗普3日被记者问及“病毒是否出自中国研究所”时声称：“我认为中国犯下了可怕的错误。他们试图掩盖此事，但未能灭火，不承认失误。”他还强调：“会收到武汉研究所发生了什么事情的报告，那是决定性的。”他在4月30日也曾表示：“看到了病毒源自中国武汉研究所的证据”，提及了实施报复性关税等问题。

　同一天，蓬佩奥在接受美国广播公司（ABC）采访时指出，“有‘大量证据（enormous evidence）’可以证明，新冠病毒来自武汉研究所”，“中国研究所的失败导致全世界遭受新冠病毒，这不是第一次。”

　当记者问及中国是有意传播病毒还是偶发事故时，他说，“有很多事情需要了解。为了解开疑问，有必要进行现场调查。应该去那里”，向中方施压。特朗普总统最亲信的执政党共和党参议员林赛·格雷厄姆也表示：“制定了制裁中国的法案，直到中国协助武汉研究所调查为止。”

　美国国内就新冠病毒“中国起源说”有两种说法，一是武汉研究所出于使用生物武器的目的制造了病毒，二是武汉研究所因事故偶然泄露。政治媒体“Axios”报道说：“生物学武器说的可能性很低，事故说有一定可能性，但没有直接证据。”特朗普当天形容中国是“失误”，庞培也同意新冠病毒并非人工制造，“没有理由怀疑”。分析认为，他们是在强调，即便如此，如果是偶发性事故，即使是为了了解是以何种方式泄露的，对武汉研究所的调查也不可避免。

　据美联社3日报道，美国国土安全部3日制作了4页内部报告，内容为“中国领导层为储备医疗物品和设备，从1月初开始故意隐瞒了新冠病毒的严重性”。也就是说，中国故意推迟向世界卫生组织报告新冠病毒的危险性，从而从国外进口医疗设备，结果今年初中国口罩及防护手套进口量猛增。

　双方就台湾参加世界卫星组织会议一事也针锋相对。世卫组织最高决议机关世界卫生总会将于18日召开视频会议。美国国务院和美国驻联合国代表部2日在推特上上传了“支持台湾加入世界卫生组织”的推特话题。相当于美国驻台湾大使馆的美国在台协会也表示，“从当天开始，每天都会在脸书上传支持台湾参与世界卫生总会的文字”。

　中国则认为，“美国正在把新冠问题政治化”，表示强烈反对。中国驻日内瓦代表部批评称：“这违反了一个中国（台湾是中国的一部分））的原则，可能会给台独势力发出错误的信号。”

# **特朗普暗示要征收报复关税，称“已经看到新冠病毒在中国发生的证据”**

美国总统特朗普声称，“已经看到了新冠病毒源自中国武汉病毒研究所的证据”，表示正在研究对华关税。有分析认为，特朗普为了获得连任，亮出了强有力而具体的剑。不能排除今年1月通过第一阶段贸易协议好不容易找到出口的两国贸易战争再次演变成全面战争的可能性。

　特朗普当地时间4月30日在白宫举行的记者招待会上，当记者问及“是否看到了武汉研究所源头说的证据”时，他两次重复回答：“是的。看到了。”他虽然表示，“中国没能阻止扩散或放任其扩散”，但并未透露具体证据。他解释称，相关调查正在进行中，“不久的将来就会得到答案。其结果将决定对中国有何感受。”

　在回答“是否考虑停止履行债务以惩罚中国”的问题时，他说：“我可以做这样的事情，但只会通过征收关税获得更多的钱。”

　《华盛顿邮报》报道称，美国正在针对中国讨论剥夺国际法中“主权国家不得成为他国法庭被告”的“主权豁免”条款。其意图是将中国告上美国法庭，以获得损害赔偿。美国有线电视新闻网（CNN）也报道说，美国正在研究经济制裁，拒绝偿还债务和新贸易政策等。

　掌管美国17个情报机构的国家情报局当天说：“情报机构同意科学上达成的协议，即新冠病毒并非由人制造或遗传性变形。为了判断发病是武汉研究所事故导致的结果，还是始于与受感染动物的接触，将进行调查。”事实上，有观察人士认为，这是在支持“武汉起源说”。

　美国过敏症、传染病研究所所长安东尼·福奇曾多次表示，“疫苗的开发至少需要18个月”，如今改变态度说：“到明年1月为止，可以提供数亿支疫苗。”有分析认为，这也与大选不无关系。《纽约时报》报道说：“对福奇感到不满的特朗普总统亲自指示卫生部长亚历克斯·阿扎在年内开发。将加快疫苗的开发，即使疫苗引起疾病和死亡也不会追究其责任。”

　据分析，特朗普政府的总攻势是因为担心新冠疫情造成的人员伤亡以及经济停滞的长期化会对11月的总统选举产生负面影响。这意味着，他把反对派的矛头转向中国，为凝聚支持者而针对中国。特朗普总统上个月29日主张：“中国为了阻止我的胜利，将用尽一切办法。”

　中国对此表示强烈反对。中国国防部发言人吴谦4月30日在记者会上表示：“美国政客回避责任、指责中国，是自私和不负责任的行为。”前一天，中国外交部副部长乐玉成在接受美国全国广播公司（NBC）采访时表示：“不要把毫无根据的罪名转嫁给中国。没有向中国要求赔偿的法律依据，却提出荒唐的主张”，“这是荒唐的政治笑话。”

# **特朗普：“将向中国追究新冠疫情赔偿责任……目前正在认真进行调查”**

 美国总统特朗普表示，“将向中国追究新冠病毒扩散的巨额赔偿责任”，美中攻防战正在升温。

　据《政治报》等媒体报道，特朗普27日在华盛顿白宫举行的记者招待会上，当被问及美国是否会像德国那样向中国提出赔偿时说：“虽然金额还没有决定，但美国可能会要求支付比德国更多的赔偿金”，“新冠病毒的损失相当大。这一损失不仅仅局限于美国，而是全球性的。”最近德国最大日报《图片报》主张，应该向中国要求1490亿欧元（约合198万亿韩元）的新冠病毒造成的经济损失赔偿。

　因“把杀菌剂注入人体”的发言引发争议，特朗普上周后半期没有出席新冠疫情例行记者会，他当天大部分发言都对中国进行了攻击。他再次强调：“中国应该对新冠病毒的扩散负责。我们正在非常认真地进行调查。”

　以对华强硬派著称的白宫贸易制造业政策局局长彼得·纳瓦罗也参加福克斯新闻频道节目，指责“中国向美国出口劣质新冠病毒检察装备，并从中牟取暴利”。纳瓦罗说：“要解除移动管制，让美国人重返工作岗位，必须增加新冠病毒诊断检查。但是，如果用中国设备进行检查，就会出现错误的结果”，“中国生产的设备会影响经济正常化。”

　美国国立保健院（NIH）24日中断了对与中国携手进行新冠病毒研究的非营利研究所“回声健康联盟”的资金援助。该研究所一直在研究蝙蝠如何向人类传播冠状病毒。美国政府通报说，从2015年至今，美国共提供了370万美元（约合46亿韩元）的援助，但今年补贴中尚未使用的约37万美元将被停止使用。

　《政治报》推测，该研究所与中国武汉病毒研究所联手，可能违背了特朗普政府的心意。在美国，有人提出武汉病毒研究所故意制造或泄漏新冠病毒。“回声健康联盟”理事长彼得·达斯萨克称，“虽然与武汉病毒研究所合作过，但从未有过金钱往来”，表示冤枉。

　特朗普政府最近还加强了与台湾的合作。美国卫生部长亚历克斯·阿扎当天与台湾“卫福部长”陈时中通了电话。其目的是加强双方在新冠病毒疫苗及治疗药物开发等方面的合作。彭博社评价说：“两人进行了30分钟的交谈。这显示了美国对台湾的有力支持。”

　台湾当局期待美国增加台湾加入世界卫生组织及参与国际保健悬案的机会。由于中国政府主张的“一个中国”原则，台湾不能成为世卫组织的正式成员国，只有“观察员”地位。台湾声称，在以亲大陆倾向著称的世卫组织总干事谭德塞得到中国的支持在2017年7月就任后，“（台湾）受到世卫组织露骨的忽视。新冠疫情爆发初期也没能得到正常的信息共享”，一直表示不满。

# **哈尔滨71人集体感染，中国再次采取“准封锁措施”**

为防止新冠病毒疫情再次扩散，中国对拥有1085万人口的黑龙江省省会哈尔滨市采取了准封锁措施。哈尔滨市最近发生了1名从海外回来的感染者导致71人连锁、集体感染的事件。

　哈尔滨市政府22日表示，“管制全市所有居民小区和农村村镇的出入口，禁止外来人员和外部车辆进入。”婚礼和葬礼也被禁止。演出、比赛、论坛、展览等大型活动也被叫停。

　此前，中国一直表示“成功控制了新冠疫情”，放宽了移动管制措施。但是，随着继海外流入病例之后再次发生集体感染，担心第二次流行的可能性，受惊吓的中国当局再次拿出了强硬措施。

　哈尔滨市的连锁、集体感染始于上个月从美国经香港和北京回国的留学生韩某。其家人和邻居都受到了感染，其中一部分人访问的哈尔滨市内两家大型医院也发生了集体感染。由此，不仅是哈尔滨市，辽宁省抚顺市和内蒙古自治区也分别出现了1名患者。哈尔滨市政府正在对医院内集体感染当时在场的4106人进行是否感染的调查。

　据悉，韩某本月初还去过上海。有人担心，新冠病毒的再流行范围可能会在中国内扩散。

　中国外交部21日下午表示：“在国内的人不要去海外旅行。身在海外的人不要移动到其他国家。”这可以解释为，由于情况不妙，在5月1日劳动节长假之前，中国宣布了禁止中国人跨国移动的措施。

# **愈演愈烈的“中国新冠疫情责任论”……默克尔：“中国应透明地予以公开”**

对于新冠疫情的扩散，“中国责任论”正在愈演愈烈。继美国之后，欧洲各国首脑也敦促中国明确查清瞒报新冠疫情的疑惑。

　 据路透社等媒体报道，德国总理默克尔20日表示：“中国政府应该透明地公开新冠病毒的起源和初期扩散。只有这样，全世界才能吸取教训，情况才会好转。”最近在德国，“新冠疫情中国责任论”还通过主流媒体《图片报》等媒体扩散开来。

　 法国总统马克龙17日接受《金融时报》采访时指出：“认为中国妥善地应对了（新冠疫情），这是天真的想法”，“正在发生我们不知道的事情。”英国外交大臣拉布16日在记者会上也表示，“有必要对病毒在中国是如何传播的进行彻底的分析”。

　 欧洲与美国不同，在新冠疫情上一直克制对中国的批评。这是因为2017年特朗普总统就任后，随着与美国的关系逐渐疏远，欧洲对中国的依赖度也随之增加。对于欧洲基调的改变，美国外交专业媒体《外交政策》表示：“中国公布的有关新冠疫情的数据失去了信任。以此为基础制定的各国新冠疫情对策也是错误的。”中国没有明确地表明新冠疫情发病时间和累计死亡人数等，因此曾引起不信任。

　 新冠疫情在本国暴发后还宣传本国体制，贬低其他国家的做法也引起了反感。近日，中国驻法大使在其网站上发表文章《西欧应对新冠疫情行动迟缓》，遭到法国政府的抗议。也有人指出，中国打着人道主义的旗号支援医疗物品，有意挑起欧盟的分裂。部分人还怀疑中国在西方社会散布假新闻。

　 也有分析认为，为了唤起国内舆论，一些国家将矛头转向了中国。包括美国在内，法国、德国、英国等国的新冠疫情死亡人数达数万人，损失巨大。《金融时报》报道说：“西方政客攻击北京，避免自己的失败，同时分散批评对象。”

　 随着对中国的批评扩散到全世界，一直拥护中国的世界卫生组织陷入了尴尬的立场。世卫组织总干事谭德塞20日在瑞士日内瓦总部的新闻发布会上强调：“我们从未向美国隐瞒（任何信息）。”

# **中国第一季度经济增长率为-6.8%，历史上第一次负增长**

 受新冠疫情冲击，中国第一季度（1∼3月）的经济增长率自统计以来首次出现负增长。中国政府预告将采取高强度经济刺激措施。

　中国国家统计局发言人毛盛勇17日在记者会上表示：“第一季度国内生产总值（GDP）总额为20.6504万亿元，比去年同期减少了6.8%。”这是中国自1992年开始公布季度经济增长率以来，28年来的首次负增长和最低值。这与去年第四季度6%的经济增长率相比，下降了12.8个百分点。

　因文化大革命中国经济濒临崩溃的1976年（-1.6%）是最后一次出现负增长。如果今年整体经济增长率出现负增长，这将是44年来首次出现负增长。

　毛盛勇说，一季度第二产业（制造业）产值下降9.6%，第三产业（服务业）产值下降5.2%。一季度工业生产同比下降8.4%，反映消费需求的零售销售同比下降19%。

# **中国武汉市新冠肺炎死亡病例核增1290例**

中国缩小新型冠状病毒感染症(Covid19病毒)死亡人数的疑惑被证实。据中国《新华通讯社》报道称，17日湖北省武汉市政府对外表示，截至16日，武汉市的新冠肺炎累计死亡人数为3869人，比此前官方公布的死亡人数多出1290人。公告称，武汉市累计确诊病例50333例，比原来增加325例。

　中国保健当局国家卫生健康委员会(简称“卫建委”)在同一天公布，截至16日，武汉市的新冠肺炎累计死亡人数为2579人。这相当于现有官方累计死亡人数的一半，也就是说，到目前为止，这一数字还没有被列入统计数据。

　武汉市政府表示，市建委、疾病预防控制中心和公安等多部门对医院、养老院、殡葬信息系统等进行了调查。武汉市政府解释说，在新冠肺炎疫情初期，由于患者剧增，导致患者无法住院在家中死亡，而且由于医院超负荷运转，医疗人员忙于救治，客观上存在迟报、漏报、误报现象，导致死亡信息不准确。但是，有人批评当局回避责任，把漏报死亡人数的责任推给一线医护人员。

　此前，中国的新冠肺炎统计数据变化无常，被怀疑隐瞒事实，引起公众的不信任。中国媒体《财新》最近质疑称，运走的骨灰盒至少是官方死亡人数的两倍。

# **中国奇袭式禁止入境，导致企业人士等乱成一团**

中国以防控新冠肺炎疫情为由，事实上宣布了封锁国境的超强硬措施。不仅是在中国当地运营生产工厂及销售法人的国内企业，目前停留在中国以外的韩国侨民及留学生也因为无法进入中国而不可避免地出现混乱。

　当天，中国外交部和国家移民管理局决定，以28日零时为标准，禁止持中国居留签证和居留许可证的外国人进入中国。中国表示，如有经贸、科技活动及紧急人道主义原因，可向当地驻华使馆、领事馆例外申请签证。

　但韩国驻华大使馆表示：“已经要求中方说明在什么情况下可以作为例外措施申请签证，但中方没有给出明确的答复。”

　包括中国国家主席习近平在内的二十国集团领导人特别视频峰会结束后不久发表联合声明称，“为了促进国际贸易，将以避免在国家间移动和贸易方面造成不必要障碍的方式进行合作”。中国的措施是在上述声明发布一个多小时后发布的。

　对于中国突然采取的禁止入境措施，韩国政府也感到非常惊慌。韩国外交高层官员27日对记者说：“昨天（26日）突然接到了禁止入境的通知。我对事先没有接到通知感到遗憾。”

# **美国新冠肺炎确诊病例已超8.5万......超越中国成世界最大感染国**

26日(当地时间)，美国确诊的新型冠状病毒感染症(COVID19病毒)患者人数超过8.5万名，成为了世界最大的感染国。自1月21日美国出现首例患者后，已经过了65天。

　据全球统计网站“Worldometer”实时统计数据显示，截至当天美国确诊患者为8.5520万人，比前一日增加了1.6939万人，超过了中国(8.1340万人)。死亡人数增加了261人，达到1297人。自本月19日突破1万人大关后，短短一周时间，这一数字就超过了8万人，增速异常迅猛。

美国最大城市纽约所属的纽约州患者达3.7258万人。白宫新冠肺炎负责人戴伯拉•伯克斯说：“全美55%的感染者都在纽约和附近的新泽西州。芝加哥和底特律的患者剧增趋势也非同寻常。”由于医护人员及医疗用品严重短缺，纽约州长安德鲁•库奥莫当天批准了两名患者共同使用一个氧气呼吸器的方案。

　彭博社指责说，唐纳德•特朗普政府安逸的现实认识、松懈的初期应对方式导致了目前的状况，并称“新冠肺炎疫情是特朗普的越战”。但是，特朗普总统在当天举行的记者招待会上依旧坚持“在下月12日复活节前解除为防止新冠肺炎疫情扩散的社会距离”的原有立场，受到了批评。

　意大利(8.0589万名)感染者的增加速度也很快，预计不久后会超过中国。日本感染者在一天内增加了96名，新增确诊患者人数创历史新高，总患者人数达到了2099名(包括钻石公主号)。

# **全球化最大受益者中国遭遇的第二次危机**

最近在中国社交媒体微博上，在意大利留学的中国人瑶瑶（化名）的回国记成为了热门话题。意大利新冠肺炎疫情趋于严重后，瑶瑶决定返回家乡广东深圳。

　从阿布扎比、北京直到到达深圳，整整28个小时，瑶瑶不吃不喝，也没有摘过口罩，坚持了下来。对于瑶瑶令人哭笑不得的遭遇，中国网友拍手叫好：“教科书式回国！”但是，像他这样认为“中国最安全”并“逃离”欧美的中国人大幅增加后，由他们造成的新冠肺炎患者回流现象也呈上升趋势。这令宣传中国国内新冠肺炎新增病例为“零”的中国当局大吃一惊。

　对此，中国当局全面采取了入境管制措施。中国还开始实施限制措施，禁止外国飞往北京的飞机直接降落在北京。这与中国在形势严峻时对中国关上大门的世界各国进行批评的态度截然相反。空中通道被控制后，中国航空企业受到的打击成为现实。上月，中国国际航空公司等中国代表性航空公司的乘客数量比去年同期骤减八成以上。

　新冠肺炎疫情导致世界各国关闭或控制边境，全球供应链开始受到冲击。停止新冠肺炎疫情扩散趋势的中国重新启动工厂。但是，由于世界供应链的破坏，原材料不足，实际上相当多工厂无法正常运营。中国华南地区美国商会最近调查的237家中国南部地区企业中，32%面临原材料供应不足的问题。

　新冠肺炎疫情导致海外各国消费下降，也给中国经济带来不利影响。中国制造业中心杭州的一家汽车零部件相关企业对美国《华盛顿邮报》表示：“虽然从上个月开始启动工厂，但不仅中国国内需求减少，而且对韩国和日本的出口也受到影响，只有平时订单的三成。”据美国市场调查机构Coresight Research统计，美国鞋类制造商史蒂夫·麦登公司生产的产品的73%和美国电子产品企业百思买公司生产产品的60%，是在中国生产的。也就是说，如果新冠肺炎疫情在美国的需求减少，中国国内工厂生产也会减少，中国经济也会受到损失。

　中国政府想宣传“在阻击新冠肺炎疫情的战争中取得胜利”，但现在不得不担心全球供应链的受损和消费锐减对中国经济带来的冲击。因为作为全球化的最大受益者，中国可能成为全球化断裂的最大受害者。在新冠肺炎疫情大规模扩散的第一轮危机之后，中国面临的这场第二轮危机与第一次不同，中国政府无法独自解决。

　但是生产、消费、贸易相互依赖度最大的世界第一、第二经济大国美国和中国因为新冠肺炎疫情的对策和发源地问题，一再出现了回避责任和消耗性矛盾。人们普遍担心，如果美中继续以这种方式进行斗争，即使其他国家努力应对全球经济危机，效果也会下降。

# **中国驻韩国大使邢海明：“可以免除韩国企业人士的隔离”**

中国驻韩国大使邢海明（照片）就新型冠状病毒肺炎表示：“中国保障在对方国家工作的外国国民的必要往来，将为访问中国的韩国国民提供针对性的便利。”韩国政府正在与中国磋商，寻求允许持有新冠病毒检测阴性确认书或健康证明书的企业人士作为例外入境，中国高层官员对此表明肯定立场尚属首次。

　邢海明17日在中国驻韩国大使馆接受《东亚日报》独家采访时，就韩国企业人士入境时免除隔离措施的方案表示：“在韩国接受过检查，（到中国后）再检查一次，如果没有发热症状，可能就可以。”截至17日，中国的24个地方政府（省、市、自治区）对来自韩国的入境者实施14天隔离。邢海明表示：“韩国在韩中应对新冠肺炎合作体制第一次视频会议上提出如果能证明健康确认证没有问题就允许入境的原则，中国政府正在对此进行认真研究。”

　关于中国国家主席习近平的访韩，邢海明表示：“习近平主席已经接受了文在寅总统的访韩邀请，所以一定会访问韩国。”但他同时表示：“时间并没有决定。”当记者问及访韩时间是否与访日有关时，他表示：“不是。”

# **习近平致电文在寅：“中韩是同舟共济的友好邻邦……中国将提供力所能及的援助”**

中国国家主席习近平就新冠肺炎问题，致电文在寅总统表示感谢和慰问。

　据中国官方媒体中央电视台14日报道，习近平主席在慰问电中表示，“中韩是守望相助、同舟共济的友好邻邦”，“韩国政府和社会各界此前纷纷向中方抗击疫情表达关心慰问并提供大量帮助。”上个月20日，在韩中国家元首通话时，文在寅总统表示，“中国的困难是韩国的困难”，强调了两国为克服新冠肺炎疫情加强合作的重要性，习主席通过电文再次提及了这一点。

　习主席表示：“疫病没有国界，世界各国是休戚与共的命运共同体”，“中国政府和中国人民对韩方目前遭受的疫情和困难感同身受，将继续提供力所能及的援助。”他还表示：“我高度重视中韩关系发展，愿同文在寅总统一道努力，推动中韩战略合作伙伴关系迈向更高水平。”

　此前，两国于13日成立了“韩中联合新冠肺炎防疫体系”，并举行了由外交部和防疫当局等相关部门参加的“韩中应对新冠肺炎疫情合作对话（局长级）”视频会议。

# **中国外交部“对日本的入境限制表示理解”，不提出问题**

虽然日本对韩国和中国共同采取了事实上的禁止入境措施，但中国的反应与韩国政府截然不同。中国政府和媒体都表示“可以理解”，没有提出问题。有分析认为，这一场面是最近日益亲近的中日关系的象征。

　中国外交部发言人赵立坚5日就日本限制入境措施表示：“无论是中国还是日本，为保障本国和外国市民的健康和安全，维护地区和世界公共卫生安全，采取科学、专业的措施，大家都能理解。”他表示，“两国通过外交途径保持着密切的沟通”，暗示日本已经提前向中国通报了措施。

　具有排外倾向的《环球时报》也刊登了日本首相安倍晋三宣布的对从韩国、中国出发的乘客隔离14天的消息，并在社交媒体的官方账号上报道时加上了“可以理解”的题目。

　中国社交媒体微博上虽然接连出现了关于取消签证效力的担忧和咨询，但是很难找到露骨地指责日本措施的文章。一位中国网民在相关报道下写道：“中国首先在非常时期这样做。日本的措施不应该受到谴责。我建议韩国也这样做。”

　中国政府和媒体的反应，某种程度上是为了正当化中国多个地区以防止新冠病毒肺炎回流为由，对来自韩国和日本的乘客实施14天强制隔离的措施。

　还有分析认为，这与2018年10月安倍首相访华宣布“新中日关系”后两国关系不断改善的趋势不无关系。中国试图在美中矛盾中拉拢日本，日本也在美国忽视同盟的情况下为了经济、安全需要改善与中国的关系。实际上，新冠病毒肺炎在中国正式开始扩散的1月份，日本最先向中国表明了支援口罩等防疫物品的意向。当时中国政府对日本表示感谢，称“困难时的朋友才是真正的朋友”。

# **韩国政府高官：“习近平访韩不会改变，但如果新冠疫情不能克服，将受到影响”**

 韩国外交当局普遍认为，由于新型冠状病毒感染症事态长期化，中国国家主席习近平上半年访韩的计划可能会受到影响。

　外交当局高层官员3日会见记者时表示：“日本媒体上有很多报道称习主席4月份的访问计划已被推迟”，“我认为，如果（新冠病毒）事态不能尽快克服，（习主席访韩日程）也会推迟。”这虽然提到的是外电关于中日正在协调因新冠疫情而推迟习主席4月份访日日程的报道，但这是韩国政府高层官员首次提到习主席的访韩也会受到新冠疫情的影响。但是该官员补充说：“我们仍然在现有协议框架下推进（上半年访韩）日程。”

　有评价称，今年与俄罗斯迎来建交30周年，但对俄外交日程也很难在短时间内具体化。据悉，韩国政府原计划最早在本月内推进俄罗斯外长拉夫罗夫的访韩计划，但由于新冠疫情的影响而变得困难。据悉，除此之外，低级别的诸多外交日程已经延期或取消。

　在外交当局高度关注美国禁止韩国人入境等可能性的情况下，美国表明了暂时“加强检查”的立场。美国副总统迈克·彭斯当地时间2日在记者会上表示：“对意大利和韩国的所有直航航班，将在机场进行100%（发热）检查。” 但是，美国总统特朗普同一天在回答记者“是否考虑加强旅行限制”的提问时回答说：“对（新冠疫情）发病较多的特定国家会这样做”，暗示存在加强应对的可能性。

　日本外务省2日将庆尚北道庆山市、永川市、漆谷郡、义城郡、星州郡、军威郡等6个地区的感染症危险信息从原来的“等级1”提升至“等级3”。等级3建议暂停访问，是四个级别中第二个严重级别。

　据统计，截至3日下午，禁止韩国人入境或予以隔隔离的国家共有89个。占联合国会员国（193个国家）一半的46%。外交部表示，截至3日上午，在海外被隔离的韩国人超过1200人。据悉，中国和越南分别有960人和270多人被隔离，俄罗斯、吉尔吉斯斯坦、卡塔尔等地各有10多人被隔离。

# **习近平指示“查明COVID-19的发源地”**

中国国家主席习近平指示中国科学家尽快查明新型冠状病毒感染症(COVID-19)的发源地。这是最近以中国官方媒体和当局有关人士为中心，提出中国可能不是发源地的情况下做出的指示，其背景备受关注。

　2日，据中国中央电视台(CCTV)报道，习近平当天在视察正在进行新冠肺炎疫情相关研究的北京军事医学研究院和清华大学医学院时表示，“利用人工智能(AI)和大数据等新技术，追踪(新冠肺炎疫情的)根源。明确说明(新冠肺炎疫情)病源究竟来自何处，去往何处，提高检查的准确度和效率。”

　一些专家提出，“新冠肺炎疫情的发源地可能有多处，发源动物(宿主)也可能有好几种。”

　中国《环球时报》本月初甚至主张“美国可能是发源地”，因此有人分析说，新冠肺炎疫情扩散到全世界后，中国试图摆脱责任论。

# **中国国家卫健委：“COVID-19存在气溶胶传播可能性”**

在中国保健当局(卫生局)中的最上级机关首次正式承认了通过气溶胶(大气中漂浮的微小粒子)传播新型冠状病毒感染症(世界卫生组织将新冠病毒命名为COVID-19)的可能性。

　 中国国家卫生健康委员会(卫健委)在19日发布的《COVID-19诊疗方案(第六版)》中就传播途径表示，“在相对封闭的环境下，如果长时间暴露在高浓度的气溶胶中，病毒通过气溶胶传播的可能性较大。”

　 卫健委在4日发布的诊疗方案(第五版)中表示，“气溶胶传播途径尚不明确。”但此后，专家和一些地方政府提出了通过气溶胶传播的可能性。8日，上海市政府曾表示，“COVID-19主要传播途径确定为通过飞沫(唾液)的直接传播、气溶胶传播、接触传播。”但第二天的9日，卫健委反驳称“没有证据显示病毒可通过气溶胶进行传播”，引发了争议。

　 飞沫相对较重，通常无法超出2米范围，而气溶胶相对轻巧，传播距离较远。通过留在办公室等密闭室内空间的气溶胶，有可能发生集体感染。

　 卫健委还删除了只适用于湖北省的“有肺炎症状的临床诊断患者”分类条款。13日，湖北省将列入这一项目的患者包括在确诊患者中，仅一天时间，湖北省的患者就增加了14840人。 但有人指出，如果采用新诊疗方案，湖北省也没有必要将临床诊断患者包括在确诊患者中，因此，从统计上来看，此举是不是为了减少湖北省的患者规模。

# **患者暴增9倍，失去信任的中国“新冠肺炎”统计 /**

中国改变对新型冠状病毒感染症（COVID-19）确诊患者判定标准后，湖北省的感染者和死亡人数暴增。有人提出疑惑称，新冠病毒发生地武汉等湖北省的新冠病毒扩散趋势比人们所知的还要严重，一直以来是否隐瞒和缩小了事实真相。

　湖北省卫生健康委员会13日公布，12日一天内新增确诊病例14840例，死亡242例。与11日相比，确诊患者和死亡人数分别增加了约9倍和2.6倍。每天上午公布正式统计的国家卫生健康委员会直到当天下午，也没有公布统计结果。

　湖北省解释说：“为使患者得到及时确诊、提高治疗成功率，把临床（治疗）诊断作为确诊的标准。”“之前通过核酸检查确诊病例，现在改变标准，通过医疗团队的判断及计算机断层扫描（CT）影像，确诊病例大幅增加。”据湖北省统计，这种“临床诊断”导致的确诊病例和死亡病例分别约占当天新增病例的90%（10332例）和约56%（135例）。

　湖北省说，“为符合全国其他省份公布的确诊标准，湖北省决定从13日起将‘临床诊断’患者纳入确诊病例范围并予以公布。”即，此前已在其他省份适用的确诊标准现在才开始适用。值得一提的是，经确认，这一确诊分类的依据——中国国家卫生健康委员会《新冠病毒诊断方案》（第五版），早在一周多前的4日就已出台。香港《南华早报》当天指出，“在武汉等地发现疑似新冠病毒肺炎患者和死亡病例后，超负荷的医护人员一直将这些患者列为普通肺炎”。

　此外，日本厚生劳动省13日表示，“钻石公主”游轮号上新增感染者44人（乘客43人，乘务员1人）。至此，仅游轮上就有218名确诊患者，日本国内感染人数增至247人。

　韩国当天虽然没有确认的新增感染者，但是保健当局和医疗界对于中国的新冠病毒确诊患者和死亡者剧增非常紧张。疾病管理本部13日表示，已向中国保健当局要求确认患者人数剧增。

# **中国新型冠状病毒感染病例增幅创造单日新纪录，香港出现首例死亡病例**

中国因感染新型冠状病毒而死亡的人数已超过400人。香港出现首例因感染新型冠状病毒死亡的病例。这是继菲律宾之后，中国本土以外发生的第二个死亡病例。

　4日，中国国内确诊患者人数比前一天增加了3136人，共达20438人。一天里患者增加数首次超过了3000名。死亡人数为426人，比前一天增加了65人。一天内出现的确诊患者和死亡者数都呈现出最高的增加值，呈现出日益增加的趋势。中国政府正式公布的重症病例达2788人，而且新型冠状病毒感染症发病地湖北省武汉市死亡率超过5%，因此有人担心今后死亡人数还会继续增加。

　另据香港《明报》报道，一名曾到过武汉的39岁男子1月31日确诊后接受治疗，当天不治身亡。香港前一天宣布，除深圳湾检查站等两处外，所有与中国内地相连的检查站都将关闭，但香港境内要求完全封锁与中国内地接壤的边境的呼声日益高涨。

# **中国驻韩大使：“入境限制应根据世卫组织的规定”**

“对于韩国采取的（限制部分中国人入境）措施，我不会多加评价。”

　中国驻韩国大使邢海明4日在中国驻韩国大使馆举行的记者招待会上，就韩国政府从当天起实行限制访问过武汉的外国人入境的方针的提问做出了上述回答。由于新型冠状病毒感染症的扩散，韩国国内出现了批评中国的舆论，在这样的情况下，与其说积极的反对，不如说是邢海明以比较低的程度表示了不快。

　邢海明当天援引世界卫生组织的立场表示，希望韩国不要采取强硬的入境限制措施。他说：“（世界卫生组织）认为此次传染病没有理由不必要地妨碍国际旅行”，“世界性、科学性是世界卫生组织的根据，（限制入境等）根据世界卫生组织的规定就可以了吧？”他还表示：“（韩中）要以科学的态度换位思考，共同应对（传染病）。”

　对于韩国决定提供大规模人道主义援助，他表示：“对与传染病的斗争给予了大力支持，对此深表感谢，我们永远不会忘记这份温暖的礼物。”韩国外交部承诺向中国提供500万美元的援助，前一天租用货机将民间提供的300万个口罩等救援物资送往中国武汉。

　针对中国对韩国强化出入境政策表现出不快的态度，韩国政府仍然坚持立场称，“不排除追加措施”。外交部发言人金仁哲4日在例行记者会上表示：“关于（向上）调整旅游警报等问题，正在继续进行讨论。”

# **“史无前例的扩散”，WHO宣布将新型冠状病毒疫情列为“国际关注的突发公共卫生事件”**

世界卫生组织(WHO)宣布，将新型冠状病毒感染症(武汉肺炎)疫情列为“国际关注的突发公共卫生事件(英文简称PHEIC)”。世界卫生组织总干事谭德塞(Tedros Adhanom Ghebreyesus)当地时间30日就武汉肺炎表示，“过去几周以来，人们目睹了之前所不知道的病原体的出现，并扩大为史无前例的发病。”但他同时表示，宣布紧急状态“疫情不仅在中国国内大规模爆发，在其他国家也正发生疫情”。并强调称，“没有理由采取妨碍(与中国)贸易和旅行的措施。”

中国国内武汉肺炎确诊患者和死亡人数在1日均达到最高值，扩散速度正在加快。中国内地确诊病例9692例，较前一日增加1866例；死亡213例，增加43例。全球确诊病例9831例，超过2003年SARS全球病例数8098例。在中国甚至出现了武汉肺炎的“空气感染”可能性。中国疾病预防控制中心(CDC)首席专家吴尊友在接受中国中央电视台(CCTV)采访时表示，“武汉肺炎与非典有很大不同，与流行性感冒(流感)的传播模式更相似。在调查过程发现，在(特定的)封闭空间内，其他人吸入武汉肺炎感染者留在空气中的飞沫(分泌物)或浮质(微固体粒子或水滴)，引起无感染源的感染。”

# **中国出现无症状自发集体传染......第三次感染病例也出现**

随着中国连续出现包括“第3次感染”在内的新型冠状病毒感染症(以下武汉肺炎)集体传染事例，人们开始担心疫情是否超出了可控范围。

　29日，据中国河南省安阳市卫生健康委员会透露，父亲(45岁)和2个姑姑被从湖北武汉回家的鲁某传染。随后，鲁某父亲又传染给了鲁某的母亲周某和鲁某的另一个姑姑(第3次传染)。鲁女士是确诊患者，但她10日从武汉回来后，已过了潜伏期(最长14天)，症状仍未出现，无症状患者的集体感染已成现实。另外，安徽省合肥市参加同学聚会的6名20多岁学生集体感染，28日确诊。安徽省黄山市也有6名家属相继被感染。

截至28日，中国内地武汉肺炎确诊病例累计达到6018例，超过2003年非典流行时中国内地最终确诊病例5327例。武汉肺炎死亡人数为132人，比前一日增加26人。另外，在中东、阿联酋出现首例武汉肺炎确诊病例。

　世界卫生组织发言人克里斯蒂安•林德迈尔28日(当地时间)表示，“不能断定感染者在多大程度上出现症状才能传播武汉肺炎”，提到了无症状感染的可能性。但是疾病管理本部中央防疫对策本部总管组长朴惠京表示：“WHO文件中没有这样的文字，没有因无症状而感染的根据。”29日，韩国国内没有出现新增确诊患者。

# **“武汉咳嗽”动摇习近平，增长率和领导能力面临危机**

受“新型冠状病毒感染症（武汉肺炎）”事件的影响，中国国家主席习近平的领导力受到了不小的打击。中国政府在危机管理能力上暴露出了问题，经济也将蒙上阴影。美国《纽约时报》指出：“习主席遭遇了数年来最严重的政治危机。”

○对“习近平领导力”的批评加剧

　自去年12月30日武汉市政府公布“武汉肺炎”发病情况后，25天之后的本月25日，习主席才首次正式提及应对“武汉肺炎”。

　由于对政府的消极应对感到不满加剧，李克强总理27日在习近平主席的指示下访问了肺炎发源地湖北省武汉。他前往医院和超市等场所，鼓励医护人员和居民。在临时隔离病房建设现场，李克强向工人们说：“有困难就说吧。我会帮你解决的。”在场的人高喊：“没有（困难）！”不过据当地媒体报道，直到前一天，他们还是透露了口罩和医用手套等物资短缺的困难。社交媒体微博中不断出现嘲笑声，“一直向各地寻求帮助，突然改口了？”“真的是中国式回答。”

　政府的报告及决策体系也暴露出相当多的问题。武汉市市长周先旺对官方的中国中央电视台说：“我们也对信息公开不满。作为地方政府，我们只有在获得相关信息和权限后才能公开信息。”

　中国医疗体系的薄弱环节也暴露出来。据《纽约时报》报道，51岁的武汉居民肖西冰（音）虽然出现发烧和呼吸困难，但由于武汉市医院病床不足，一直辗转于医院，半个月后的26日才住进医院。妻子彭秀（音）不满地说：“（把病人）踢来踢去。”湖北省委副书记、武汉市委书记马国强26日晚在记者招待会上承认，武汉市病区处于超负荷运转状态。他说，“最近几天，每天都有15000名发烧患者涌进医院，出现排长队问题”。中国计划到下个月为止在武汉市建设1000个病床、1300个规模的两个临时隔离病区。

○“经济增长率可能降低到4%”

　如果“武汉肺炎”的扩散导致劳动天数减少、旅游萎缩等长期化，政府将很难达到6%的目标增长率。香港《南华早报》援引美国中国研究机构“plenum”的话预测说：“中国第一季度的增长率可能会达到4%左右。”

　中国把春节长假延长三天至下个月2日，但随着“武汉肺炎”的暴增，很有可能再次延长。金融城市上海通知企业，在下月9日之前不要恢复业务。据路透社报道，上海市此举将使在相关地区拥有工厂或在当地经营合资公司的特斯拉、通用汽车和大众汽车蒙受损失。

　制造业中心苏州也要求企业停止业务至下月8日中午。据悉，信息技术企业腾讯和中国最大的电子商务企业阿里巴巴等已通知职员在家工作14天。故宫、长城、上海迪斯尼乐园等主要旅游景点也暂时关闭。春节档期为高峰的电影上映也相继推迟。中国篮球联赛也无限期中断。北京还建议，即使春节假期结束，从湖北等地方回来的市民在家自行隔离14天。

　一些大企业为了支援医疗团队，开始建立基金。百度27日表示，将筹集3亿元人民币（约合506亿韩元）的基金，用于支援治疗“武汉肺炎”的研发等。送餐应用程序“美团”也将成立2亿元人民币（约合337亿韩元）的基金，支援医疗团队。美团还承诺向武汉市内医务人员提供每天1000次的免费外卖券以及湖北省内医务人员免费使用30万辆公用自行车的优惠券。

# **武汉肺炎警报**

 新型冠状病毒引发的“武汉肺炎”呈扩散趋势。去年12月31日，在中国湖北省省会武汉发生疫情后不到一个月，包括一名美国患者在内，全世界8个国家共确诊病例581例。其中死亡17人，均为中国人。一名中国人在韩国仁川机场被疑感染，隔离检查后已被确诊。作为交通要塞、有着“中国肚脐”之称的1100万人口的武汉，从23日上午10点开始，所有进城、出城的公共交通全部停运。这是中国历史上第一次封锁省会城市。

　据悉，武汉肺炎的宿主是蝙蝠或蛇。非典（SARS）的宿主是蝙蝠和麝香猫，中东呼吸综合征的宿主是骆驼。在武汉肺炎发源地水产市场，一直有各种野生动物的非法交易，第二名死亡者也是水产市场店主。潜伏期短则两三天，长则10-12天。症状与感冒和流感相似，没有治疗剂和疫苗。因为会以感染者的唾液或鼻涕传播，所以去人多的地方时要戴口罩。口罩可能会沾上病毒，最好戴上一次就扔掉。

　香港专家警告说，武汉肺炎有像2003年非典时期那样大流行的迹象。传染病的扩散会经历从动物传染到人类、人际传染、传染给患者家属和医疗团队、进入大规模发病阶段的不同阶段，而武汉肺炎已是进入最后阶段在即。有批评指出，中国的信息控制和马后炮应对加速了病毒的扩散。武汉虽被封锁，但据估计已有数百万武汉市民走出城市。《纽约时报》指出，中国控制媒体和市民社会，政府垄断信息，导致事态扩大。
　封锁武汉的消息导致中国股指暴跌。世界经济遭受的损失规模也将超过2003年非典时期。因为在世界经济中，预计中国所占的比重将从2003年的8.7%增加到今年的20%。呈现恢复迹象的韩国经济也从年初开始就遇到了不利因素。人们担心，像5年前的“中东呼吸综合征”时一样，消费冻结会影响增长率的反弹。

　今天开始的春节长假是第一个关口。虽然移动较多，人员聚集，感染的可能性较大，但大多数医院都关门。预计将吸引14万名中国游客。韩国政府相关部门应启动24小时应急防疫体系，个人应倾听相关信息，并遵循包括手消毒和戴口罩在内的传染病预防行为守则。

# **中国昨天才全面封锁肺炎发源地武汉市**

因新型冠状病毒导致的“武汉肺炎”确诊病例和死亡病例大幅增加，中国当局事后紧急封锁了病毒发源地湖北省武汉市。拥有1100万人口的武汉市是湖北省省会城市。这是历史上第一次封锁省级城市。

　当地时间23日凌晨2时，武汉市有关部门在一份紧急声明中说：“从当天上午10时起，将停止运营公交车、地铁、渡轮和长途汽车”，“市民如不是特殊情况，不要离开武汉。航班和列车也将暂时中断，届时将通知恢复时间。”

　位于中国中部的武汉也是连接周边9个省的交通要塞。分析认为，在看到传染病大流行的征兆后，着急的中国当局采取了前所未有的措施。

　但是据统计，当天中国国内武汉肺炎确诊患者为600人，比前一天增加了56人。中国31个省、区、市中有27个出现确诊或疑似患者。没有进入武汉肺炎影响圈的地区只剩下西部的甘肃省、青海省、新疆维吾尔自治区和西藏自治区等4个。拉美的墨西哥、巴西和哥伦比亚，北美的加拿大也出现了疑似患者等，全世界对武汉肺炎扩散的担忧正在加大。

　因此，韩国外交部当天向中国武汉发布了二级旅游警报（克制旅行）。这是政府首次就武汉肺炎问题发布旅游警报。目前，二级旅游警报地区目前包括与美国发生武力纠纷的伊朗、持续举行大规模示威的香港等地。外交部向湖北省全境发布了一级旅游警报（注意旅行）。外交部叮嘱道：“计划去该地区（武汉等）旅游的国民要慎重考虑旅行的必要性，滞留当地的国民要特别注意人身安全。”疾病管理本部为了迅速掌握当地情况，当天向中国北京韩国大使馆派遣了流行病学调查官。

# **“武汉肺炎”扩散到美国，出现大流行迹象**

美国出现首例因新型冠状病毒引起的“武汉肺炎”确诊病例，而在中国，患者人数持续暴增，全球出现大流行迹象。

　据路透社当地时间21日报道，美国疾病控制和预防中心表示，最近一名在中国湖北省武汉旅游过的30多岁男性被确诊为武汉肺炎。这名男子居住在美国华盛顿州西雅图附近，15日回国后正在接受治疗。这是亚洲以外大陆首次发现确诊病例。

　中国疾病预防控制中心22日宣布，武汉肺炎确诊病例增至440例（含台湾）。仅一天就增加了130多人。13个省、市出现了确诊病例。加上疑似患者，中国共有21个省、市受到影响，占中国31个省、市的68%。当天，澳门也出现首例确诊病例。中国国内死亡人数也从6人增至9人。医疗部门正在观察的密切接触者达1394人，预计患者人数将会持续激增。

　中国官方媒体新华社引述国家卫生与健康委员会专家组的报告指出，“病毒可能发生变异，传染情况有进一步扩散的危险”。专家们表示：“还有地区内传播。”等于是承认了一些地区存在集体感染病例。专家组组长钟南山警告说：“必须防止超级传播者出现。”香港《南华早报》预测说，将像2003年非典（SARS）时期一样进入全面扩散阶段。

　中国14亿人口中有超过4.5亿人以上移动的春节（25日）即将到来，预计将成为大流行的分水岭。中国政府明确表示将采取非典级紧急应对，下达了“不要去武汉或离开武汉”的武汉旅游克制呼吁令。

　韩国国内也新增了4名类似武汉肺炎症状的“有症状者”。至此，包括19日被确诊的中国女患者A某（35岁）在内，国内武汉肺炎患者总数增至16人。据悉，新增加的“有症状者”中有3人是与A某一起从武汉飞往仁川的乘客及机场负责人。

　青瓦台副发言人韩正宇表示，文在寅总统在22日听取情况报告后指示，“在做好检疫和预防措施的同时，还要综合检查对经济的影响”。据悉，文在寅总统前一天在政府世宗办公大楼举行的国务会议上，也下达了“请格外注意防疫”的指示。

# **春节在即，中国会否再现“非典恐慌”？**

 随着中国国内新型冠状病毒引发的“武汉肺炎”患者数量急剧增加，人们越来越担心“中国的防疫体系是否已被突破”。中国网民甚至怀疑当局隐瞒真相，应对迟缓。有人质疑，2002年至2003年非典疫情爆发初期，中国有关部门隐瞒疫情，导致疫情初期应对不力，仅中国内地和香港就有648人死亡，这一先例是否会重演？

　武汉肺炎的传染规模和范围都呈不可控制的扩大态势。发源地湖北武汉18日和19日两天新增确诊病例136例，是现有病例的3倍以上。另外，北京南部大兴区和广东省深圳市分别出现2名和1名确诊患者，深圳市出现8名疑似患者，浙江省出现5名疑似患者。这与中国有关部门强调“因为传染力很脆弱，人与人之间传染的可能性极低”的说法大相径庭。

　在大兴区，有一个去年投入运营的北京新机场。北京市有关部门表示，确诊患者曾到武汉旅游，但并未公开何时通过何种途径感染。上海市当局表示，“对部分疑似患者采取了防止传染措施”，但并未公开疑似患者人数。

　深圳市有关部门表示，一名确诊患者是曾去武汉探亲的66岁男子。但“印度报业托拉斯”通讯社报道说，深圳市国际学校教师、印度人普里蒂·马赫什瓦里（45岁，女）被确诊，目前正在深圳市医院隔离治疗。如果该报道属实，那么相当于中国有关部门隐瞒了印度患者发病的事实。更何况，印报托称，这名女子从未到过武汉。

　此前，中国一直只从地方当局层面进行应对，直到19日国家卫生健康委员会才公布预防对策，并向全中国派遣了工作组。中国当局表示：“没有找到病毒传染的源头，也没有完全掌握传播途径。”韩国政府高级官员表示：“据传，中国当局使用了新的检查方法，武汉的确诊患者人数大幅增加。”特别是中国春节前后将有30亿人大移动，不能排除因无法控制而发展成为大流行的可能性。

　中国网民的不信任也在扩散。某网民在中国社交媒体微博上留言说：“听说传染力很弱，患者如此激增？又要骗人、隐瞒吗？”还有人留言讽刺说：“不会再有人说爱国病毒了吧。”

# **威胁中国梦的新一代登场**

去年12月和本月，为采访台湾大选，记者访问了台北。记者在现场看到，一群20多岁的台湾年轻人形容自己是“天然独”。意思是说，从小开始就认为台湾是拥有独立主权的国家。大四学生程某（22岁）说：“所以我们不能接受中国提出的‘一国两制’统一方案。”

　在当地见到的台湾20岁至39岁的人，认为自己不是中国人，而是台湾人。他们的选票是反华倾向的蔡英文连任总统的原动力。让20世纪80年代出生的年轻人与老一辈变得不同的，是台湾的民主化。

　经过上世纪80年代的民主化运动，直到1996年实行总统直选制之前，台湾是一个以来自中国的外省人为主的国民党长期执政的权威主义社会。上世纪70年代以前出生的“老一代”有很多是“天然统”“当然统”，主张应当与中国统一。相反，从小开始经历民主化的年轻人作为台湾人的认同感很强。他们与搞不清自己到底是中国人还是台湾人、是应该与中国统一还是独立，头脑混乱的40岁以上台湾人不同。在台湾见到的专家说：“年轻人过去只关心就业等自身问题。但是，不能将自己的未来交付给希望与中国统一的旧世代身上的危机意识，导致了投票热潮。”

　在台湾大选以蔡英文的压倒性胜利结束后，台湾媒体分析说：“他们现在正从‘天然独’变成‘天然台’。”他们说，“台湾已经是一个主权国家，没有必要非要追求独立，而是希望维持目前台湾本身的现状”。这与主张如果统一台湾需要不排除武力的中国国家主席习近平站在了对立的位置上。

　去年在香港的示威现场，也目睹了与“天然独”几乎一模一样的新千年一代的出现。香港中文大学学生陈某（21岁，女）对记者说：“我们出生于香港回归中国后的90年代，是百分之百属于香港的第一代人。”是在既不是英国殖民地同时也是“在受保障与中国不同体制的自由社会香港接受教育长大的，我们不是中国人，而是香港人”。

　“直到中国建国100周年的2049年成为世界强国”的中国梦中，也包括“一国两制”的成功。以香港和澳门的“一国两制”为基础，台湾也要实现统一。但是，全世界都看到，与过去一代人有着完全不同价值观的台湾的“天然独”和香港的千禧一代，已经成为新的变数。在现场，记者了解到，他们拒绝中国人认同感并非一时现象。随着时间的推移，他们将成为台湾和香港的主轴一代。

　台湾和香港接受采访的专家表示：“中国未能直视新一代登场这一变化，仅认为这是对中国的对抗和挑战，予以强硬应对，这会带来相反的结果，这是台湾和香港的共同点。”这再次提醒我们，不管是什么样的政治势力，如果不能了解民心的变化，就只能经历危机。

# **中美达成第一阶段贸易协议，韩国对华出口进入紧急状态**

 在中美持续近两年的贸易战争中，双方达成第一阶段协议，开始停火。受世界前两大经济大国的争斗影响，一度萎缩不安的全球经济暂时一扫乌云。但是，包括制裁中国华为在内的敏感矛盾移交到了第二阶段，如果再有第一阶段协议的履行过程不顺利，关税战争随时都有可能重启，不确定性依然存在。最令人担忧的是，中国决定追加进口大量美国产品，这将对韩国的对华出口造成巨大打击。

　美中15日达成协议，中国将在两年内追加购买价值2000亿美元（约合232万亿韩元）的美国产品。相反，美国决定把对中国产品征收的15%关税下调至7.5%，不再对去年12月计划征收的其他产品征收追加关税。协议中还包含了中国保护知识产权、禁止强迫技术转让、停止人为压低人民币等承诺。

　中国决定从美国追加进口的2000亿美元商品，相当于韩国年总出口额的33%。不仅包括农产品，还包括工业产品、能源、服务等多种产品。如果中国的内需量没有大幅增加，那么从其他国家进口的量就会减少。国际货币基金组织去年底在题为《美中贸易协定的副作用》的报告中分析说，如果美中谈判达成妥协，韩国、日本、欧盟等其他国家将遭受打击。如果中国的进口额和以前一样，韩国最多将减少460亿美元（约合53万亿韩元）的出口，国内生产总值（GDP）将减少3%左右。

　韩国没有时间因美中贸易矛盾的中断而暗松一口气，对华出口直接受到冲击的可能性加大。强国之间签订的“私下协定”人为地重组了全球贸易体系，对今后经济产生的影响不确定性也增大了。韩国对华出口占总出口的27%。当务之急是实现出口多元化，减少对华贸易依存度，寻找中美贸易的缝隙市场。

# **中国试射把美全境纳入射程的潜射导弹，还与俄、伊举行联合军演**

在美国继续对伊朗实施空前严厉的制裁的情况下，中国和俄罗斯将与伊朗一起在印度洋北部和阿曼海举行历史上首次海上联合军事演习。中国还试射了可以打击美国全境的洲际弹道导弹级核弹头的潜射弹道导弹，展示了肌肉。中国与俄罗斯携手与美国对抗的态势仍在持续，引起了人们关注。

　据美联社和伊朗梅尔通讯社25日报道，三国海军参加的这次演习名为“海洋安全带”，在27日至30日期间举行。该地区是海湾的入口，且靠近世界最大的原油运输海域霍尔木兹海峡。伊朗军方说：“这次演习的目的是加强中东地区的国际贸易安全。伊朗、俄罗斯和中国交换安全经验、对抗恐怖主义和海盗行为，是最重要的目标。”

　但此次演习是为了支援因美国退出《中程核力量条约》及经济制裁而陷入困境的伊朗，很多人认为这是中国和俄罗斯在中东地区对抗美国的举动。

　为加大对伊朗的军事压力，美国与友好国家结成了在海湾海域活动的海军军事联盟“霍尔木兹护航联盟”，中国和俄罗斯对此表示，“威胁中东地区的稳定”。值得关注的是，韩国也有可能根据美国的要求，向霍尔木兹海峡派兵。

　美国表示，伊朗直接介入了也门胡塞反政府武装宣称是自己所为的今年9月针对沙特阿拉伯国营石油企业阿美石油公司的石油生产设施和油田的无人机及导弹攻击，正在加大对伊朗的压力。美国在阿美石油公司遇袭事件发生后，向沙特增派了美军，并增设了导弹防御系统。

　伊朗表示，将利用此次演习扩大与中国、俄罗斯的军事合作。据伊朗法尔斯通讯社报道，伊朗海军少将何塞因·汉扎迪说，“这次演习是伊朗与中国、俄罗斯海军广泛合作的一部分，其中包括潜艇和驱逐舰的生产”。也有预测称，像巴基斯坦这样反美倾向较强的国家今后有可能参加中国、俄罗斯、伊朗的联合军事演习。

　中国22日在渤海向西试射了射程为美国全境的洲际导弹级潜射导弹“巨浪-3”。中国官方媒体“观察者网”没有直接提及发射“巨浪-3”的事实，但表示：“在北京看到了奇怪的云层（导弹轨迹）”，“中国政府以20日至27日在渤海实行军事任务为由发布了禁航令。”

　据悉，“巨浪-3”是从中国的094型战略核动力潜艇上发射的。该导弹射程达1万公里，被认为可以搭载核弹头打击美国本土。

# **扩散至美国到处的“敲打中国”，复杂的全球外交方程式**

 在华盛顿一家中坚律师事务所工作的P律师，最近中国委托人大幅增加。具体情况是，在美国的中国科学家受到美国政府的监视，并因不可捉摸的原因活动受到限制。他负责提供法律应对，就此表示：“美国情报人员几乎是一对一地对中国科学家进行密切监视”，“美中关系恶化，业务就越来越多，真好。”

　“不会连民间领域的学者都如此严密地管理吧……”记者虽然很好奇，但看到P律师表情严肃，看来这句话没有错。从华为的事例中可以看出，特朗普政府想要牵制中国的技术霸权，已经出现了全方位扩散的趋势。不可否认，美国的“敲打中国（China bashing）”是华盛顿外交安全领域的一个明显趋势。

　事实上，将中国视为“敌对国家”的白宫和国务院高层官员的发言强度正在逐渐加强。最近，关于香港示威，指责中国人权状况的内容越来越多。美国国务卿迈克·蓬佩奥上个月在德国柏林墙30周年纪念仪式上发表演讲时甚至批评中国说：“中国共产党在压迫国民。”虽然他采取的是在继前东德之后连带指责中国存在的共产主义问题的方式，但看起来与题为《1989年的教训：自由和我们的未来》的演讲宗旨没有多大关联性。

　今年迎来70周年的北大西洋公约组织（北约）本月初在首脑会议声明中针对中国也不同寻常。中国攻略非洲、网络活动、增强军事能力等，虽然对于欧洲也可能成为新的挑战，但把地球对面的中国当作目标说事，多少有些莫名其妙。浮现在人们眼前的是北约大腕成员国美国的身影。让人觉得美国出口了其攻击性的对华战略。

　加大对中国研究力度的智库也越来越多。美国国际战略研究中心在不同领域专门成立了4个研究中国的小组。仅本月以来，就先后举行了“中国的崛起和全球秩序”“中国的人权问题和美国的应对”“中国的政治、宗教人权问题”等以中国为主题的研讨会。到现场一看，几乎没有中国人，倒是当地学界及媒体人士和香港、台湾、韩国等外国记者非常热闹。中国人似乎因为牵制氛围太强，很难露面。

　随着美国12日与中国达成第一阶段贸易协议，美中之间的紧张气氛似乎有所缓和。但是，双方还有第二阶段谈判，涉及知识产权等更加棘手的内容。这只是暂时的冻结，很难找到期待美中关系会好转的氛围。

　中国很久以前就不是美国外交安保政策的变数而是常数。在这种冲突中，北京的抗议更加强烈，由此引发的东北亚局势更加动荡。如果明年包括预告“新道路”的朝鲜问题在内，美中之间需要解决的外交函数也将变得更加复杂。现在，不仅是中国本身，对美国对华政策和战略也要进行更多的分析和研究。

# **中国出现27年来最低增长率，停滞阴影渐浓**

中国第三季度（7月至9月）的经济增长率仅为6.0%，为27年来的最低值。有分析认为，随着韩国企业出口和直接投资最多的中国市场萎缩，韩国经济的增长基础正在动摇。

　中国国家统计局18日表示：“第三季度国内生产总值（GDP）为24.6865万亿元（约4119万亿韩元），比去年同期增长6.0%。”这一增长率是自1992年开始统计季度增长率以来的最低值，比当初的市场预期下降了0.1个百分点。中国自2015年第二季度出现7.0%的增长势头后，4年来一直停留在6%多，但现在接近了跌破6%。从去年第一季度开始，季度增长率每次都呈下降趋势，因此很多人预测第四季度增长率有可能下降到不到6%。

　第三季度中国经济增长率低于市场预期，原因是中美贸易纠纷和非洲猪瘟导致出口和内需基础同时减弱。受中美贸易矛盾影响，中国9月份出口同比下降3.2%。显示制造业活力的生产价格指数（PPI）在今年7月至9月连续3个月减少，甚至产生了通货紧缩的担心。再加上非洲猪瘟，上个月猪肉价格上涨了70%，上个月消费者物价上升了3%。受此影响，随着消费减少，产生了降低增长率的效果。英国《金融时报》报道说：“地方政府通过道路、桥梁等基础设施建设，实现了增长目标，但这样的事业也日渐枯竭。”

　不少人担心，随着中国经济陷入低迷，在制造业和金融领域与中国密切相关的韩国经济将受到打击。韩国经济研究院等民间研究所认为，如果中国经济增长率下降1个百分点，韩国经济增长率将下降0.5个百分点。现代经济研究院经济研究室室长朱元（音译）表示：“随着世界经济长期低迷，中国政府的各种扶持政策也未能奏效，经济增长放缓现象可能会持续到明年，韩国经济可能会受到负面影响。”

# **香港示威者首次与中国军队对峙，刚刚接近部队就被警告“要为后果负责任”**

香港反中示威者和中国人民解放军驻港部队6日晚间发生短暂对峙。在始于6月至今已延续四个多月的这次示威中，这是双方首次直接对峙，令紧张感达到了高潮。

　当天，数百名示威者前往九龙地区的人民解放军部队营区附近，用激光灯光照射部队建筑物。中国军队立即在房屋顶上举起黄色旗帜，向示威者发出警告信号。旗帜上用中国本土使用的普通話和英语写着：“你们正在违反法律。可能会被起诉。”中国军队还用在香港使用的广东话发出语音警告：“以后发生的后果，你们自己要负责任。”

　这一过程中，中国军队使用摄像机拍摄示威者，密切监视他们的动态。但是，随着示威者不久即离开部队周边，再没有发生冲突。《南华早报》报道称，“中国军队对前所未有的行动发出了警告”。有人担心，如果中国军队向示威者射击或流血镇压，示威事态有可能朝着无法控制的方向发展。亲中派势力主张，示威者的此次行动可能是为了拉西方介入而实施的故意挑衅。

　随着5日开始施行《禁止蒙面规例》，对立也越来越激化。香港警方7日以涉嫌违反禁止蒙面法，首次起诉一名18岁的大学生和一名38岁的女子。她们5日凌晨戴着口罩展开示威，随后被捕。教育当局向初中和高中的校长们发出指示：“从8日起，要提交戴口罩上学的学生、拒绝上课的学生、展开手拉手示威或喊口号的学生的名单。”据称，警方6日在未经大学许可的情况下，进入香港中文大学和浸礼大学校内，将示威者和戴口罩市民全部逮捕。

　6日，在深水埗地区，一名60多岁的司机驾驶一辆出租车冲向示威人群，导致两人被车子轧倒，身受重伤。之后，人们看到，示威者们把司机拉出车外进行殴打，司机脸上流着血，失去意识。一名电视台记者被示威者投掷的火焰瓶砸中，脸部被烧伤。

　7日上午，香港地铁只运营了所有地铁站（94处）中的39处。当天下午6点起以内部修理为由关闭全部路线，令城市犹如一座幽灵城市。大型购物商场纷纷关门，主要超市也缩短了营业时间。去超市购买生活必需品的人群排起长队，还出现了东西被抢购一空的情景。部分市民认为“如同战争般的氛围”。

　此外，美国职业篮球（NBA）的著名球队休斯顿火箭队总经理达雷尔·莫雷因为支持示威者，导致赞助火箭队的中国企业纷纷中断赞助，遭受了屈辱。他在6日在推特上写道：“对于复杂的事件，仅支持了一方。”

# **爱国主义沸腾的建国70年，中国内部也有责任的声音**

10月1日，新中国成立70周年纪念阅兵式从上午10时（当地时间）开始在北京中心天安门一带举行。包括记者在内的外国记者在阅兵式开始前5个半小时，即上午4点半左右经过第一次安检后，聚集在北京西部的媒体中心。漆黑的清晨，记者驱车行驶在交通管制下空荡荡的马路上，早上6时左右到达天安门广场附近的前门。为了前往观看阅兵式场所天安门广场的最前方，必须通过第二次安检。

　《人民日报》、新华社等中国官方媒体的记者于前一天的30日晚11时在媒体中心集合。他们到达天安门一带的时间是1日凌晨1点50分左右。参加阅兵式的士兵们已经集结在长安大街上。他们在街上呆了整整一夜，但表情很明朗。

　阅兵式开始前在现场见到的中国人也掩饰不住激动的心情。3万多名观众从各地选拔，获得了参观阅兵的机会，从凌晨就到达了现场。在天安门广场亲眼看到“强大的祖国军队”是中国人的梦想。

　“任何势力也无法动摇我们伟大祖国的地位。”中国国家主席习近平的宣布，引起了中国人在欢呼。习主席说：“70年前，新中国成立以后，彻底改变了近代以来中国漫长的积贫积弱、惨遭欺负的悲惨命运。”此话意味深长。中国人民对“强大起来的祖国”的欢呼，展示了现在不仅是美国，世界上任何一个国家都无法撼动中国的自豪。这种自豪感有时通过对外部好战的语言表现出来。

　现在，全中国上下都在为“人民的团结”最为重要的爱国主义而沸腾。国庆节长假（1日至7日）上映的爱国主义电影《我和我的祖国》等票房火爆。“中华民族伟大复兴”的民族主义支撑着爱国主义。爱国主义本身不应该责怪。但是，中国内部也有人担心，中国是否意识到了“强国应承担的沉重责任”。

　曾任北京大学国际关系学院副院长的北京大学教授王逸舟在接受采访时警告说：“如果错误处理民族主义，可能会变成一种盲目的爱国主义。”他指出：“特别是一个国家，如果曾经遭受过欺负，或者存在主权争端，民族主义可能会产生意想不到地伤害他人的后果。”他一语道破，“好外交必须有好内治、好的社会基础”。他主张，走向“仁社会”的条件包括“人民更开放，没有好战情绪的社会”等。

　他担心的是，其他国家为什么对中国崛起感到担忧和威胁，而中国却认识不到这一点。他说，中国的崛起已经走到了十字路口，“越是高山之巅，风景越美，但高山症状越严重”。现在中国遇到了过去没有的问题，即世界中更大的责任和义务问题，需要回头看看是不是因为过分强调爱国主义而遮住了眼睛。

# **习近平：“任何力量也无法撼动中国”**

中国1日迎来建国70周年，在北京天安门广场举行了仿佛武器展示场的大规模阅兵式，展示了国力。国家主席习近平针对美国强调，“任何力量都撼动不了中国”。他一再呼吁香港要“一国两制”、台湾要“和平统一”以及“爱国、团结、民族主义”。

　当天的阅兵式以庆祝建国70周年的70次礼炮发射和五星红旗升旗仪式开始。身着中山装的习主席率领在两旁的江泽民、胡锦涛两名前任国家主席出现，并表示：“过去的70年里，中国取得了辉煌的成果。任何力量都不能动摇我们的地位。任何势力都不能阻止中国人民和中华民族的前进。”这可以解释为，尽管存在美中贸易战、香港反华示威、经济增速放缓等内忧外患，中国不会屈服于美国的压力。

　他针对香港和台湾说，“要恪守和平统一、一国两制的原则，保持香港和澳门的长期繁荣稳定”。他还表示，“中国的明天会更加美好”，希望大家团结起来，实现中华民族伟大复兴的“中国梦”。在演讲的最后，他大声疾呼：“中华人民共和国万岁，伟大的中国共产党万岁，伟大的中国人民万岁！”

　习主席检阅了事先在天安门广场前面长安街上列队的59个方队、15000名军人。如同最尖端武器展示场的阅兵式本身就是向美国传达的信息。最引人注目的武器是能够打击美国首都华盛顿的新一代洲际导弹“东风-41”。当天首次公开的该导弹射程为1.4万公里，全世界都在射程之内。据悉，该导弹最高可携带10枚核弹头，攻击目标误差范围也只有100米。

　此外，中国还展示了使用极超音速滑降技术、可以突破美国导弹防御系统的“东风-17”，以及被称为“航母杀手”的极超音速导弹“东风-100”等。与美国F-35相当的新型隐形战斗机“J-20”、与美国军用直升机“黑鹰”相匹敌的“Z-20”也加入其中。中国媒体报道说，当天参加阅兵式的武器中，40%是首次公开的。官方的《环球时报》报道说：“已告知中国拥有战略核武器，可以应对任何威胁。”有分析认为，这不仅对美国，对韩国、日本等周边国家也有威胁效果。

　当天，为迎接建国70周年，各国领导人也纷纷发来贺电。中国按照俄罗斯总统普京、美国总统特朗普、朝鲜国务委员会委员长金正恩的顺序介绍了贺电。

　据朝鲜中央通讯社1日报道，金正恩在贺电中表示：“（朝鲜）完全支持中国为维护国家稳定和核心利益、实现持续发展而进行的斗争”，“在坚持社会主义、为国争光的道路上，朝鲜将永远与中国站在一起。”金正恩还在贺电中谈到“中朝之间多次（首脑）会晤上取得的重要协议精神”，强调“深信（两国关系）将符合新时代的要求和两国人民共同的愿望，日益获得发展。”

# **中国再次下调人民币汇率，美国对中国输美家具征收惩罚性关税**

中国央行——中国人民银行9日再次下调人民币市场汇价。人民币汇率自2008年5月后时隔11年首次突破了“1美元兑7元人民币”大关（破七），当天又进一步下跌。

　今天上午，中国人民银行公布人民币对美元汇率中间价为7.0136：1。前一交易日，人民币对美元汇率中间价为7.0039，上升了0.14%（人民币贬值）。自上个月31日以来，人民币对美元汇率已连续7个交易日走低。

　有人指出，中国不顾美国的强烈反对，将人民币弱势作为关税及汇率战的“武器”，美国也对中国采取了“对攻”制裁。据美联社报道，美国商务部8日（当地时间）决定对中国产木制家具征收高额补偿性关税。补偿性关税是贸易对象国政府为提高出口竞争力而对提供补助金的商品适用的惩罚性税金。中国去年向美国出口的木橱和梳妆台规模达44.0117亿美元（约合53000亿韩元）。

　美国商务部当天宣布，对中国产橱柜和梳妆台的补偿性关税调查结果显示，中国企业获得的国家补贴最低为10.97%，最高为229.24%。商务部还表示，将按相关企业判定的不公平补贴支付额征收相应的税金。该措施将在明年1月30日经过美国贸易委员会的最终裁决后执行。

　彭博社等报道称，美国当天还保留了放宽对中国最大通信设备企业华为的出口限制。美国商务部长威尔伯·罗斯7月30日对美国企业提出的“请允许与华为进行交易”的要求称，“下周之内可以作出回应”，作出了正面的反应。但分析认为，随着两国对立的激化，这一措施已被无限期推迟。5月，商务部以与伊朗的交易情况为由，将华为及其子公司列入限制交易的黑名单。此后，希望与华为进行交易的美国企业必须事先得到政府的批准。

　另据《日本经济新闻》9日报道，金融市场人士预计，“人民币下跌”的下一个底线为1美元兑7.2至7.3元人民币。这是根据特朗普政府宣布从下个月1日起对中国价值3000亿美元的产品征收10%追加关税进行推算的结果。3000亿美元是中国对美国出口总额的约60%，如果在此基础上加征10%的关税，对美国出口整体影响为6%。据《日本经济新闻》分析，目前人民币汇率为7.0136元，若按6%的升值幅度计算，1美元将兑换7.3元人民币。也有人担心，如果人民币对美元汇率下跌到7.3：1，中国企业的美元负债将会剧增，从而引发人民币抛售及价值的进一步下跌。

# **中国认为香港示威是“颜色革命”**

中国当局首次把香港示威定性为指称政权更迭运动的“颜色革命”。对此，有分析认为，以大规模示威持续的本周末为转折点，中国可能针对香港开始投入军队等武力介入。　

　中国国务院港澳办公室主任张晓明7日在与香港接壤的广东省深圳市举行的非公开座谈会上表示：“‘罪犯引渡法’事件已经变质。明确地出现了颜色革命的特点。”550名全国人大代表、全国政协（国家咨询机构）委员参加了当天的座谈会。

　张晓明说：“目前形势是1997年香港回归以来最严重的一次”，“如果香港局势进一步恶化，香港特区政府不能控制动乱，中央（中国政府）绝不会坐视不管。”他提高声音表示：“根据香港基本法，中央有足够的办法和强大力量迅速镇压各种动乱。”

　一直解释中国共产党意图的中国共产党机关报《人民日报》的社交媒体官方账号“侠客岛”表示，“中国当局首次判断香港示威是颜色革命，”认为这是中国当局向香港示威队伍发出了明确的信号。“颜色革命”是社会主义国家阵营开始崩溃的1990年代前苏联国家和东欧、中亚、中东等地区发生的政权更迭运动。

　中国国家主席习近平领导的领导层今年年初流露出对颜色革命强烈的警惕。中国国务委员兼公安部部长赵克志今年1月召集全国公安干警后表示：“要聚集所有公安的智慧和力量，阻止颜色革命。要打击内外敌对势力渗透到各种破坏颠覆活动之中。”中国当局将香港示威规定为“颜色革命”后，有人指出这是动用武力的全面介入的信号，也是基于此的判断。碰巧在座谈会前一天的6日，深圳市举行了假定香港示威队伍的大规模镇压暴动演习。

　中国媒体透露，张晓明就邓小平在过去所说的“如果有必要，可以向香港投入中国军队”的说法表示：“邓小平有先见之明。”与张晓明一起主持座谈会的中国香港联络办公室主任王志民表示：“香港已经到了无路可退的地步”，“控制混乱局面、恢复秩序是最为紧迫的事情，这是关系到香港的未来和命运的战争和保卫战。”

　《环球时报》等中国媒体8日批评2014年香港民主化示威“雨伞革命”的领导人黄之锋、罗冠聪等人6日会见了美国驻香港总领事馆负责人，并抨击美国干涉香港问题。如果中国真的动员军队，美国等外部势力就会介入其中，进而在香港引发政权颠覆运动。此外，也有人谨慎地指出，在与美国展开贸易、关税、汇率等全面战争的情况下，如果再制造一个尖锐的矛盾战线，将会给中国领导层带来额外的负担。

# **中国国防白皮书首次明确提及萨德系统，猛批萨德“加剧紧张”**

 中国国防部24日（当地时间）发表的《2019国防白皮书》中提到了“萨德”，并向韩美两国亮出了兵刃。白皮书说，“美国在韩国部署萨德系统，严重损害了亚太地区的战略平衡和安全利益”。

　中国国防部当天发表了约90页的白皮书，题目是《新时代的中国国防》。中国自1988年发行第一本白皮书后，通常每两年发行一次。此次白皮书是2015年出版后时隔4年的第10次发表。特别是，4年前的白皮书长约20页，但此次白皮书中包含了萨德、韩朝问题、美国、日本、澳大利亚、台湾等多方面主题，露骨地强调了中国的主张。

　白皮书说：“世界经济和战略重心正在向亚太地区转移。在这个地区，大国之间博弈，给地区安全带来了不确定性。美国在加强亚太军事同盟、扩大军事部署及干涉的同时，给该地区增添了复杂因素。”

　这表明中国在韩朝问题上也将发挥主导作用。白皮书特别强调自我评价称：“虽然半岛取得了积极进展，但仍然存在不确定性。中国在半岛等纷争地区发挥着政治上的建设性作用。”

　中国对日本和澳大利亚也表现出了强烈的警惕。白皮书说：“日本为绕过战后体制而改变军事安全政策，军事活动十分活跃。出现了对外指向性的军事动向。”白皮书认为，澳大利亚也与美国加强军事同盟，正在跃升为亚太地区的安保玩家。特别是在台湾问题上，白皮书表示“坚持和平统一和一国两制的方针。反对分裂中国的企图和外国干涉中国内政”，把矛头指向美国。

　中国还向在南亚地区展开竞争的印度也亮出了兵刃。白皮书说：“在南亚，印度和巴基斯坦有冲突。一些国家的领土和海洋争端、民族和宗教矛盾不断发生。”

　白皮书以表格形式介绍了当前的情况，称“中国公开透明地公开国防开支及用途”。白皮书强调，在2012年至2017年国内生产总值（GDP）相比，中国的国防开支占比平均为1.3%，低于俄罗斯（4.4%）、美国（3.5%）、印度（2.5%）、和法国（2.3%）。

# **美中贸易战二次停火，保留华为火种**

美国总统唐纳德·特朗普和中国国家主席习近平6月29日在日本大阪的二十国集团（G20）首脑会议上举行80分钟的贸易谈判，就“第二次贸易战停火”达成协议，承诺暂停新增征收关税，并重启贸易谈判。

　特朗普总统当天在与习主席举行双边会谈后举行的记者招待会上表示：“我们同意继续进行协商，决定不对价值3250亿美元（约合375.5375万亿韩元）的中国进口商品加征关税。”他接着解释说：“我们暂缓征收关税，他们（中国）将购买（美国）农产品。”去年12月1日，两位首脑在阿根廷布宜诺斯艾利斯G20峰会上就保留追加关税、进行90天贸易谈判的“第一次停火”达成协议。

　特朗普总统6月29日表示，“美国的优秀企业将继续向华为销售产品”，暗示将放宽对中国最大的通信企业华为的制裁。但他同时表示，“将把华为留到最后”，“前提是只要（该交易）不引起国家安保担忧”，留下这一线索作为贸易谈判的最后筹码。同时他还表示：“希望中国学生能够前来美国，利用我们优秀的学校和大学。”

　两位首脑在前一天也进行了非正式会谈，但是当天的首脑会谈破例地持续了80分钟。特朗普说：“如果我们无法达成协议，就会重新回到（征收关税）上来，”“我不着急。希望达成正确的协议。”据中国外交部透露，习主席在会谈中强调：“在事关中国主权和尊严的问题上，中国必须维护核心利益。”

　大阪G20峰会当天在强调“公平贸易”重要性的《大阪宣言》后闭幕。G20领导人在宣言中表示，世界经济“下行风险很大”，将“采取各种行动应对风险”。以“反对贸易保护主义”为主旨的措辞，因为美国的反对，继去年阿根廷会议之后再次被排除在宣言内容之外。

# **传习近平将访问朝鲜，不要成为无核化的干扰者**

有分析认为，中国国家主席习近平将在本月底的二十国集团（G20）首脑会议之前访问朝鲜，与朝鲜国务委员会委员长金正恩举行首脑会谈。自2月28日朝美首脑河内会谈破裂之后，朝鲜拒绝一切对话，在这种情况下，如果习近平主席在G20峰会前后访朝，这一活动将给东北亚局势带来微妙的变化。

　由于把秘密视为对对方国家礼遇的朝中关系的特性，习主席访朝在正式宣布之前，确实难以得到确认。朝鲜国务委员会委员长金正恩已经四次访问中国，因此人们预计今年上半年内习主席会回访，但是随着河内会谈的破裂，习主席的访朝日程被推迟。

此次有关习主席访朝的说法，是在与美国的贸易、技术战以及围绕台湾、香港的内政干涉争议尖锐对立的情况下传出的。有分析认为，就像前年美中贸易矛盾初期一样，中国此次也将把朝核问题作为美中对抗的杠杆和反转筹码。习主席一方面可以确认作为金正恩监护人的影响力，另一方面也可以向将在G20峰会中见面的美国总统特朗普提出解决朝鲜问题的方案，以期实现美中矛盾的停战。

　最近，朝鲜对韩美有关恢复对话的一再敦促始终保持沉默。此前一直照顾朝鲜立场的文在寅总统，也在北欧巡访中多次敦促朝鲜首先表现出弃核的意志，这反映了对朝鲜的郁闷和失望。

　这种情况下，习主席访朝如果成行，将成为让朝鲜参加恢复对话的契机。但是，中国的对朝外交应该是对金正恩的无核化决断进行施压和支持，而不是展示朝中密切关系、让金正恩怀有异心。特别是中国如果出面摧毁国际社会的对朝制裁战线，只会造成难以挽回的后果。朝鲜在去年年初不得不参加协商，也是因为连中国也参与了对朝制裁。

# **如果中国统治世界**

应美国的要求，加拿大在温哥华机场逮捕了中国最大通信企业华为的副董事长、首席财务官孟晚舟（46岁），结果被中美贸易战争的激流所吞噬。中国扣留了两名加拿大人施压，孟晚舟则保释出狱。中国的后劲之大，导致加拿大高价羽绒服品牌“加拿大鹅”的股价也暴跌。

对绝对强者美国保持沉默，对帮助美国的盟国进行毫不留情的报复，这并不是中国第一次暴露出大国的真面目。美国为阻止北韩导弹而在韩国部署萨德系统时，中国对韩国进行了打击。当中国阻止团体游客访问韩国、压迫韩国经济时，认为他们是一生朋友的韩国人只能惊慌失措。

中国在联合国这样的国际舞台上强调对抗西方霸权的“水平多边主义”，但实际行动却非常混乱。就像封建时代对待诸侯国一样，中国强调以中国为中心的垂直等级秩序，一旦有人碰触其逆鳞，或多或少，总是显露出霸权国家的前近代性。

拥有2万多人口的南太平洋小岛国帕劳就曾受到中国的这种对待。只拥有中国一天新生儿人口的这个小岛国与台湾建交，对于一贯主张“一个中国”原则的中国来说，这无疑是眼中刺。

中国政府去年以没有外交关系为由，阻止了帕劳团体游。台湾航空公司运营的帕劳太平洋航空公司因中国游客减少一半，今年7月停飞了中国航线。在岛上建酒店、购买建筑的中国大腕投资者也放弃了投资。旅游等服务业比重占经济80%以上的帕劳受到了巨大的打击。

中国是占世界人口约20%的人口大国，因此与以少数人口掌握世界霸权的英国和美国有着本质上的区别。中国拥有着连旅游产业也能当作武器来使用的大规模经济和力量。而且，如果人民币能够进入美元等国际储备货币的行列，那么像美国的单方面制裁一样，只要驱离中国金融系统，就能威胁到一个国家的经济。在韩国、加拿大、帕劳发生的事情，也许只是中国支配世界后就会发生的预告而已。

无论喜欢还是讨厌，韩半岛的命运都是要与中国面对面生活下去。要想加强对华外交，比起倾向于一方的“亲中派”，更应该是更多的“乐观的现实主义者”，即积极看待同中国的关系，同时盘算潜在的危险因素。同时也应该启动检查政治、经济、社会、文化等方面是否过于集中于特定国家并采取应对措施的国家中长期战略系统。

帕劳在中国游客增加到接近总体的一半之后，才认识到了本国旅游产业过于依赖中国的弱点。为对抗中国的威胁，尽管为时略晚，帕劳开始集中培养环保高附加价值旅游产业，以吸引人均销售额远高于中国团体游客的欧洲或日本游客。这就是为了保护珊瑚礁，继美国夏威夷之后，帕劳率先禁止使用防晒霜的原因。最近帕劳当局表示，虽然中国游客有所减少，但随着人均销售额增加，旅游总销售额也相应有所增加。美中贸易战和在岛国帕劳发生的事件，让我们回味一下孙子所说的“战争的胜败，在战争之前就已见分晓”。

# **华为之争出现扩大征兆**

中国通信设备企业华为创始人的女儿、首席财务官（CFO）、副董事长孟晚舟因涉嫌违反（美国）对伊朗的制裁而在加拿大被捕，其后续影响正蔓延到美中两国之间的贸易谈判。美中两国1日在阿根廷举行首脑会谈，就贸易战停战达成协议并进入为期90天的谈判，但这一问题却成为可能破坏谈判的导火索。

美方谈判负责人、美国贸易代表办公室代表罗伯特·赖特希泽当地时间9日在接受CBS电视台采访时表示，“我认为（90天）是明确的最终时限，”“我和（特朗普）总统说话时，他说不要超过3月1日。”他同时强调：“90天之后，关税将提高。”

对于“美国总统特朗普在美中首脑会谈晚宴时是否知道逮捕孟晚舟一事？”的提问，他回答说，“不知道。这一点可以明确。”他强调：“贸易谈判不应受到孟晚舟被捕一事影响。”

但有不少分析认为，孟晚舟被捕会对美中贸易谈判产生负面影响。《纽约时报》报道称，“孟晚舟被扣押使美中经济关系变得相当复杂”，“中国人的自尊心——华为问题引发了愤怒和惊吓”。《华尔街日报》评论说：“孟晚舟被捕刺激了中国的民族主义，让习主席很难做出让步。”

前一天召见加拿大驻北京大使表示抗议的中国外交部9日召见了美国驻华大使特里·布兰斯泰德，要求美方撤回拘捕令。中国外交部表示，中国外交部副部长乐玉成对布兰斯泰德说：“美国侵害了中国市民的合法权益”，“中国将根据美国的行动采取追加措施。”

加拿大也取消了贸易使节团的访华计划，开始应对。加拿大西部不列颠哥伦比亚州在声明中表示：“贸易使节团取消访华计划是因为与孟晚舟相关的司法程序。”

结果，属于经济同盟体的美国和加拿大采取了一致行动，而自尊心强的中国也不甘示弱，因此有人提出，强强对峙局面有可能长期持续。美联社报道说，中国为了报复此次事件，有可能扣留加拿大人。

另外，在加拿大被捕的孟晚舟主张自己的清白，以健康问题为由，在审理中申请保释。据路透社等媒体报道，孟晚舟在9日公开的法庭陈述书中表示，“为了对抗引渡美国，将滞留在温哥华，如果被引渡至美国，将在美国与嫌疑作斗争”。她还表示：“考虑到严重的高血压和健康问题，希望在引渡至美国的过程中，交保释金后释放。”在7日举行的首次保释审理中，加拿大检察机关表示，孟晚舟涉嫌违反对伊朗制裁，要求法院禁止其保释。下一次审理将于10日举行。

# **“韩国人担心中国欺骗韩国”**

“韩国人担心中国会欺骗韩国。”

中国现代国际关系研究院韩半岛研究室副研究员陈向阳5日在中国江苏省盐城市举行的第6次韩中公共外交论坛上声称，“韩国对中国的自信似乎有所下降”。由韩中外交部共同主办、韩国国际交流财团和中国公共外交协会主管的此次论坛，以“构建韩中关系未来10年的支柱——全面信任”为主题，于4日和5日举行。从这次论坛可以看出，在中国对韩国部署萨德系统进行报复后，韩国对中国的负面舆论骤然高涨以及中国方面对韩国的态度。

陈向阳5日主张：“萨德给韩中相互信任带来了巨大的（负面）影响，”“双方不应越过对双方都造成损害的马其诺防线。”他补充说道：“中国需要尽全力强调韩中合作的重要性，韩国应该认识到韩中合作的价值。”中国前国务院新闻办公室主任赵启正表示：“民族主义情绪给（韩中）双边关系和外交产生了影响。”“中国学者们应向（中国）青年施加影响，克服极端民族主义。韩国也一样。”

中方人士还表现出了加强韩美同盟有损中国国家利益的认识。陈向阳认为：“并不是要求解散韩美同盟，而是担心韩美同盟的加强会对中国造成损害。”

仁川大学中国研究所所长李浩哲（音译）指出：“韩国不能集中于缓和、缩小或废除韩美同盟，只将外交政策转向韩中关系”，“应该在理解韩中两国所处的结构性条件的基础上，引领韩中关系的发展。”

# **美国“中国作出了让步”VS中国“捍卫了国家利益”，美中各说各话**

美国总统特朗普当地时间2日在推特上表示，“中国同意削减或撤销目前针对美国汽车的40%的关税。”这是在强调，在12月1日美中首脑会晤上，中美就“90日停战”达成协议后，中国立刻向美国作出了让步。

据英国《金融时报》3日透露，中国副总理刘鹤率领协商小组将于本月12日访问华盛顿，与美国进行后续贸易谈判。但是，中国政府和官方媒体与美国白宫不同，完全没有表明以下事实：“谈判时限为90天，如果不能在谈判期限内达成协议，美国将重新征收追加关税。”中国官方媒体反而强调，“中国在贸易谈判过程中坚决守护了核心利益”。

中国的这一态度表明，在特朗普总统要求的中国经济结构改革过程中，中国不会单方面作出让步。因此，有预测认为，美中今后的贸易谈判将走过荆棘田。《金融时报》也把美中首脑的协议称为“容易破裂的关税休战”，指出“后续谈判充满了困难”。

美中关系专家、北京大学教授王勇在香港《南华早报》上指出：“中国仍然必须作好在经济，尤其是尖端技术领域无法依赖美国的最坏准备。”他说：“中国可以调整包括扩大（美国企业的)市场准入性和知识产权保护在内的产业政策，但经济结构不可能发生根本性的变化。”

这是因为，作为美中后续谈判的领域，美国提出的中国调整经济结构的要求，与习近平政权维持权力及中国由国家主导的社会主义市场经济发展息息相关。美国要求中国解决强迫转让技术、侵犯知识产权、非关税壁垒等问题。这意味着，要让中国放弃在2025年之前在高科技制造业领域超越美国的“中国制造2025”。但是，中国认为美国的这种要求是不承认“中国特色社会主义制度”，是侵害中国主权的不平等谈判。因此，中国内部也担心今后的贸易谈判不能乐观。

对于美中首脑会晤达成的休战，有人评价称，“白宫内的稳健派得了分”。在特朗普总统身边主张强硬一边倒的贸易政策，主导对中国施压的白宫贸易、制造业政策局长皮特·纳瓦罗的影响力减少，而主张妥协的白宫国家经济委员会主席拉里•柯德和财政部长史蒂文·姆努钦占据了上风。

网络媒体Axios在题为《G20峰会上的外交胜利》的报道中称，“虽然此次谈判结果不是全国主义者对白宫内超强硬的贸易保护主义者的胜利，”“但谁也没有预料特朗普总统准备了与习主席的晚宴，超出预想，两国达成了就贸易战停战的协议。”

# **台湾民进党选举惨败，背后是中国大陆的经济施压**

“这不是赚了钱之后赞助台湾民进党吗?”

中国的一位专家表示，在中国大陆活动的台湾企业家中，经常会公开听到这样的话。2016年1月，具有独立倾向的民进党“总统”蔡英文当选后，出现了中国对民进党施压的氛围。

上个月24日台湾地方选举中民进党惨败于国民党，有分析认为，中国在经济施压也是主要原因之一。 

在此次选举中，广域地方自治团体长中，民进党所属人员从13人减少到6人，相反，在野党国民党所属当选人从6人增至15人。民进党还被国民党候选人韩国瑜夺走了守护了20年的第二大城市高雄的市长职位。

中国在蔡英文执政后，不仅缩减了前往台湾的游客配额，还采取了限制出口农产品和水产品的措施。大陆自行调配原本由台湾供应的零配件的所谓“红色供应网”的加强运转，也给以中小企业零配件产业为主力的台湾经济带来巨大打击。

韩国贸易投资振兴公社中国地区本部长朴汉镇30日接受《东亚日报》电话采访时说：“蔡英文执政后，中国对前往台湾的旅游团规模减少了近40%。在旅游领域尤其困难。”

中国大陆还加强了外交孤立行动，蔡英文就任时台湾的“建交国”只有22个，到今年已减少至17个。在此次选举中，民心背离民进党的主要原因是民生经济恶化。据分析，去年台湾对中国大陆的依赖度占总出口额的41.1%（含香港），台湾对中国大陆呈现绝对性依赖，在这种情况下，中国对民进党持续加压，台湾选民选择了“实利”“稳定”而不是“面子”，选择了“两岸稳定而不是矛盾”。

具有亲大陆倾向的国民党前领导人马英九执政10年后，台湾选民选择民进党“总统”蔡英文时，也预测到了两岸之间会出现一定程度的矛盾。蔡英文当局试图通过扩大与东南亚的交流等“新南方政策”来突破这一局面。但是，很多人分析认为，此次选举意味着“如果与中国背道而驰，经济将非常困难”。

在地方选举中，蔡英文的基础支持层——青年层的离脱现象非常严重，这充分说明了这一点。韩国外国语大学中国政治经济学教授姜俊英（音）解释说：“希望在中国就业的青年达69%。”“台湾内部青年失业问题非常严重，在这种情况下，民进党不断与中国步调不一致，导致青年支持层纷纷离开。”据悉，今年第3季度（7-9月）台湾的青年一代失业率为12.29%，大幅高于整体失业率(3.76%)。

据推算，在中国大陆从事经济活动的台湾人达150多万人。其中，电气、电子零部件工厂的员工也很多。有不少人建立皮包公司或以不包括在统计中的方式参与经济活动。进军中国的企业家们为了支持国民党和执政，在选举时会乘坐飞机去台湾投票，非常积极。

中国国务院台湾事务办公室发言人马晓光上个月28日在记者会上表示：“中国大陆和台湾城市之间的交流将进一步扩大。”“大陆旅游团正在前往高雄。”中国大陆在以在野党获胜的地区为中心扩大交流等向执政党民进党施压的同时，也暗示了对亲大陆的地方自治团体采取柔和措施的可能性。

# **中国科学家们发表声明“强烈谴责基因编辑婴儿”**

一名科学家声称成功完成世界上第一个使用基因编辑手段产生的婴儿，使中国卷入了争议。中国政府有关部门下令调查事实真相。与该科学家有关的大学彻底否认与实验有关。

中国科学家贺建奎26日表示：“通过基因编辑技术，产生了对艾滋病具有免疫力的基因，”“对接受不孕治疗的7对夫妇的胚胎进行基因编辑后，从中诞生了一对双胞胎女婴。”基因编辑是切除非正常基因或植入正常基因的手段，但编辑胚胎基因可能会导致变形的基因遗传到下一代，因此在世界上被禁止。

122名中国科学家当天通过微博发表了声明表示“强烈谴责”。他们表示：“基因编辑（在技术上）并不是新技术。（但是）它仍存在不确定性，在伦理问题上存在很大的危险，”“我们坚决反对在没有进行严格的伦理和安全性检查的情况下，编辑（会向下一代）遗传的胎儿基因的行为”。他们说，“潘多拉的盒子已被打开，”“在到达不能回头的阶段之前，还有机会盖上盒子。国家应该严格进行监督管理。”中国国家卫生健康委员会向广东省卫生健康委员会下达了调查实际情况的指示。

贺建奎供职的南方科学技术大学学术委员会表示：“从今年2月开始，贺建奎一直处于停薪留职状态，研究是在大学之外进行的，学校对实验内容一无所知。”

# **来自中国的沙尘暴从天而降，从微尘到黄沙“让人喘不过气来”**

27日，因大气凝滞积累的微尘(可吸入颗粒物)，加之源自中国的烟雾和沙尘暴赶在一块，整天都让人喘不过气来。黄沙袭击韩半岛是今年入秋以来的第一次。

　据韩国气象厅和国立环境科学院透露，首尔的雾霾浓度截止当天下午3时为每立方米48μg(微克，100万分之1克)，属于“恶劣”的水平。京畿和仁川分别在当天上午10时和中午12时发布雾霾注意警报。警报只有在每立方米75微克以上的雾霾持续2个小时以上时才会发布。从23日开始韩半岛上空大气凝滞，连续几天后在国内发生的微尘直接堆积，再加上当天从中国袭来的烟雾，浓度进一步上升。

　当天下午袭来的沙尘暴则更是雪上加霜。流入韩国的沙尘暴是自5月25日以后时隔6个月的首次。26日在中国内蒙古附近发生的沙尘暴中的一部分人乘着西北风降临在西海上，从27日下午开始对韩半岛产生了影响。从中国飞到韩国的黄沙大部分是与细颗粒物(PM2.5，直径2.5微克以下)相比粒子更大的微尘(PM10，直径在2.5微克至10微克中间)。

　受沙尘暴的影响，当天下午1时白翎岛的微尘(PM10)浓度飙升至每立方米471微克。这一数据是“非常恶劣”(每立方米151微克以上)的3倍以上。当天下午，首尔和仁川间的可视距离始终只有2 ~ 3公里，是晴天的五分之一水平。预计，截止到28日早晨为止，沙尘暴将对包括首尔在内的首都地区产生影响。

　到了28日白天，全国的微尘有望恢复至“一般”水平。国立环境科学院表示：“携带黄沙的西北风会将韩半岛上空的微尘推到外部，除了南方部分地区之外，微尘浓度将不会大幅上升。”

# **人工智能技术紧追美国的中国……如果“三星半导体”也被追上的那天到来**

“过去我一直认为（美中贸易战）只会在高层之间开战。但是最近确实感觉到对我们有影响。对我们企业来说压力很大。希望贸易战能尽快结束。”

三星电子15日在北京举行的“三星未来技术论坛”的小组讨论现场。上海燧原科技有限公司首席执行官赵立东比较坦率地吐露了受美中贸易战影响，中国的人工智能、半导体产业所处的困境。

19日，美国商务部产业安全局不顾赵立东的担忧，预告了将对人工智能、机器人工学等14个尖端技术项目限制出口的措施。虽然没有提到中国，但很明显这是为了阻止中国的人工智能和半导体崛起而舞刀动枪。中国也不会坐视不理。中国国家主席习近平本月初曾提出“要掌握人工智能的核心技术”。

15日作为嘉宾出席论坛的中国百度云副总经理谢广军表示，“现在美中两国的人工智能都处于起步阶段。我们期待中国能有一个美好的未来，”指出中国人工智能有政府支援等比较优势。这也透露出自信，因为中美差距不大，所以对美国的压力没有感到问题。

赵立东也表示：“电脑尖端半导体在一夜之间无法跟上，但人工智能距离（美国）起跑线并不遥远。”他说：“5年前在中国人工智能半导体是完全不切实际的说法，但现在由于有政府的支持、资本市场的狂热支持、国际上的技术连接等，发展变为可能。”

三星电子DS（设备解决方案）部门中国总管副总裁崔哲（音）当天在论坛开始前，就中国的半导体崛起表示：“我认为我们的竞争力非常强。”对于中国的存储器半导体生产计划，他充满自信地说：“第一的企业（三星电子）不是跟随市场，而是自己制造市场。”

但是第二天16日，中国国家市场监督管理总局就三星电子、SK海力士、美光垄断存储半导体市场的行为表示，“掌握了大量的证据资料”，暗示了制裁的可能性。

世界最大半导体市场中国露出了要牵制韩国半导体产业的爪子。中国试图用自己的力量对抗美国的尖端产业技术，在半导体产业上也会为了超越韩国而拼尽全力。

在15日论坛讨论最后阶段，小米的人工智能产品部总经理小米季旭等中国企业嘉宾们表示：“三星是重要的合作伙伴”，“希望在存储器半导体等领域得到三星更多的支持和合作”，“人工智能因为半导体依存度问，十分倚重三星”。

季旭甚至表示：“现在美中贸易摩擦对三星来说是一个更大的机会。”担任讨论主持人的三星半导体中国华北地区副代表欧阳基就赵立东对贸易战的担心表示：“不要担心，有三星在。”

但是，出席论坛的中国尖端技术企业和中国政府似乎都在期待中国早日赶超三星，“半导体领域超过了三星。现在不再需要三星了”。

**中国第三季度增长率为6.5%，创金融危机后最低纪录**

中国第三季度（7~9月）国内生产总值（GDP）增长率创下全球金融危机后的最低纪录。

中国国家统计局19日表示，第三季度GDP同比增长6。5%。这是经历全球金融危机的2009年第一季度增长率（6.4%）以来的最低值。中国季度GDP增长率从去年第一季度的6.9%起持续下跌。今年第一季度和第二季度分别为6.8%和6.7%。

中国当局认为，1~3季度平均GDP增长率为6.7%，今年的目标值为6.5%，综合来看，维持了稳定趋势。国家统计局当天在声明中说，“在严重复杂的国际形势下，在以习近平同志为核心的党中央的领导下，国民经济整体运营稳定，经济结构也正在不断优化。”在当天的记者会上，中国国家统计局发言人毛盛勇就明年经济增长的前景表示：“虽然外部不确定性增大，但我们认为完全能够应对外部压力。”

但是，随着中国GDP持续走下坡路，有分析认为，从美中贸易战争余波全面蔓延的第四季度（10~12月）开始，中国GDP增长率将遭受更大的打击。

另一方面，中国主管经济的副总理刘鹤当天在接受《人民日报》等官方媒体的采访时表示：“现在与美国进行接触”，“虽然中美贸易摩擦对市场产生了影响，但说实话，比起对市场产生的影响，心理上的影响更大。”

# **哼唱国歌获罪，中国“网红”铁窗行**

中国知名“网红”因在网络个人秀节目中开玩笑似地哼唱中国国歌，受到了拘留处分。有人认为，这从侧面体现了习近平时代中国对媒体网络等的社会管理正在向全方位扩大。

上海市公安局14日表示，网络直播主持人杨凯莉因违反《中国国歌法》，5日对她下达了行政拘留处分。上海公安部门表示，杨凯莉违反了“应严肃地起立唱国歌，不能对国歌不敬”的国歌法，并强调，因特网直播也不能成为法外之地。

网络直播主持人杨凯莉以“莉哥”的艺名广为人知。作为中国知名网红，她在人气视频应用软件“抖音”上有4400万名粉丝。“网红”在中国是网络明星的代称。

杨凯莉7日在中国版“优兔”——虎牙上进行介绍在线音乐庆典的网络直播时，坐在椅子上像哼唱一样唱起了“起来！不愿做奴隶的人们”开头的国歌。虽然只有3秒时间，但很多人指责她“侮辱了国歌的尊严”。

之后，杨凯莉两次在社交媒体上发文道歉，表示“没能真诚地演唱国歌，真心道歉。向祖国道歉”。虎牙方面停止了杨凯莉的直播账号。最终，杨凯莉在事件发生一周后，遭到了警方的拘留处分。上海市公安部门表示：“将依法坚决惩处挑战法律马其诺防线、违反公共秩序和良风美俗的行为。”

# **中国提出“连接丹东-平壤-首尔-釜山”，首次明确“一带一路”向韩半岛延伸**

中国首次公开了把中国-北韩交界地区辽宁省丹东市作为关门、把“一带一路”向韩半岛延伸的计划。

辽宁省政府在最近《辽宁日报》报道的《辽宁“一带一路”综合实验区建设总体方案》全文中明确指出，“以丹东为关门，连接韩半岛内陆，”明确表示“一带一路”向韩半岛延伸。辽宁省政府在该文件中表示，将用铁路、公路和通信网把丹东-平壤-首尔-釜山相互连接起来，并解释称，这一连接的性质是“直接通向南部港口”。分析认为，这是为了把“一带一路”向太平洋扩张而要延伸到釜山的意图。方案中还公布了包括新义州在内的丹东-平壤的公路建设计划。

文件还明确表示，将把位于新义州和丹东之间的鸭绿江岛屿黄金坪的北韩-中国经济区、丹东的北韩-中国互市贸易区，与丹东的重点开发开放实验区一起，打造对北韩经济合作的重要支持区。中央政府决定，努力在适当时期建设丹东特区，并加强辽宁省沈阳、大连、丹东机场和北韩、俄罗斯远东城市之间的航班。文件还公布了支援丹东互市贸易区成为国家之间网上电子商务平台的计划，中国与北韩之间电子商务成为现实的可能性也越来越大。辽宁省政府表示，“把中朝两国领导人的重要协议作为指导，巩固地规划了对北韩合作，”表明把“一带一路”向韩半岛延伸的计划是中国国家主席习近平和金正恩委员长的三次首脑会谈上达成协议的内容。

把“一带一路”延伸至韩半岛的计划，将以辽宁省为枢纽，与中国、韩国、北韩、日本、俄罗斯、蒙古国合作的东北亚经济走廊建设一起推进。辽宁省政府表示：“将结合中-俄-蒙经济走廊和韩中日+X模式，建立全面深化六国合作的东北亚命运共同体。”这体现了中国以辽宁省为中心，主导包括韩半岛在内的东北亚地区开发的意图。韩中日+X模式是今年5月中国总理李克强在韩中日首脑会议上提议的，内容包括推进韩中日自贸协定和与其他国家的自由贸易合作。

辽宁省政府表示，为此，将同时推进连接丹东-珲春-俄罗斯符拉迪沃斯托克的铁路建设和丹东港至符拉迪沃斯托克港的海上通道。横着要沿着北韩-中国边境地区，连接中国和俄罗斯，纵向要连接中国和韩半岛。文件明确指出，突显东北亚关口地位的时间点是2030年，表示目标是到2030年完成这一计划。习近平主席12日在符拉迪沃斯托克举行的东方经济论坛的演讲中倡导了与美国的单方主义形成对比的“东北亚经济圈”。也不能排除“一带一路”向韩半岛延伸是为了与美国争霸。

中国的“一带一路”向韩半岛延伸计划与文在寅政府的南北经济合作计划——韩半岛新经济地图的首尔-平壤-新义州-丹东高速铁路连接计划相重叠。外交消息灵通人士指出：“虽然这可能成为韩国和中国就北韩基础设施投资开发进行合作的契机，但中国也有可能试图将新经济地纳入‘一带一路’。”实际上，辽宁省铁路连接计划等向韩半岛延伸的计划中，就包括首尔和釜山在内，但只强调了与北韩的合作，丝毫没有提及与韩国之间的部分。

有人指出，在联合国安理会维持对北韩制裁的情况下，“一带一路”向韩半岛延伸有可能违反对北制裁。香港《南华早报》指出：“只要有制裁，中国就不会马上推进计划。”

# **习近平事实上不可能“9·9节”访问北韩，王沪宁有可能代替前往**

记者3日获悉，为纪念北韩政权成立（9·9节）70周年，中共中央政治局常委、书记处书记王沪宁将代替中国国家主席习近平访问北韩。

多名政府官员和中国消息人士表示：“习主席专心致志于3日-4日在北京举行的中非合作论坛峰会，事实上难以在9·9节访问北韩”，“考虑到中朝关系，要派有影响力的王（沪宁）书记，才够级别。”也有传言说，习主席北韩之行在上个月就开始变得前景不明，后来就发展为由王（沪宁）书记前往。

王沪宁负责北韩问题和思想、宣传，因此被认为是“代为访问北韩”的合适人选。北韩国务委员会委员长金正恩今年三次访问中国时，都是王沪宁出面迎接，并陪同出席北韩-中国首脑会谈。韩国国家安全战略研究院责任研究员朴炳光（音译）表示：“王（沪宁）书记是有访美经验的唯一一名常委，是可以在调整对美关系和为北韩改革开放建言献策的合适人选。”

外交界认为，习主席平壤之行事实上已经无望，这是美国总统特朗普对中国的谴责和警告起到了作用。韩国国立外交院金汉权（音译）教授分析称：“韩半岛问题比中美贸易问题和台湾问题在政策优先度上靠后，这种情况下习近平没有必要承担政治负担自找麻烦。”

据悉，北韩正在准备的9·9节阅兵规模大致与2月份建军节相仿，目前尚未捕捉到将公开洲际弹道导弹的征兆。韩国统一部高级官员表示：“似乎北韩也没有余力向阅兵式投入大量物资。”

# **中国军机时隔一个月再次进入韩国东海防空识别区**

一架中国军用飞机29日擅自进入南海和东海上空的韩国防空识别区，韩国空军战机升空应对。这是自上个月27日以来，中国军机时隔一个多月再次进入韩国防空识别区，也是今年以来的第五次。

据韩国军方透露，当天上午7时37分左右，一架据推测是Y-9侦察机的中国军用飞机进入离于岛西南方的韩国防空识别区，然后沿大韩海峡向东海方向移动，多次进出韩国防空识别区。随后，该机经过庆北浦项东北方向约74公里处，北上至江原道江陵东侧约96公里上空，然后调转机头向南，沿来路飞行，于上午11时50分左右完全脱离韩国防空识别区。

韩国军方表示，紧急出动了F-15K等10多架战斗机，对中国军机采取跟踪监视飞行和警告广播等战术措施。军方还通过韩中直通网，警告中方停止加剧局势紧张、引发偶然性冲突的行为。但中国军机置之不理，在韩国防空识别区连续飞行4个多小时。据悉，中方回应称，“在国际空域进行了正常的训练飞行，没有违反国际法”。离于岛周边区域，是韩国、日本和中国三国防空识别区重叠的地区。

在此之前，韩国国防部曾在上个月中国军机擅自闯入韩国防空识别区后，召见中国国防武官提出严正抗议，并敦促防止再次发生类似事件。军方高级官员表示：“中国军队为了侦察韩半岛周边地区，并展示侦察机的远程飞行能力，所以反复飞行进入韩国防空识别区。”

# **伊朗原油的最大进口国中国对美国的制裁战战兢兢**

中国是伊朗最大的贸易伙伴、伊朗原油的最大进口国，从7日起因美国恢复对伊朗实施经济和金融制裁而陷入了烦恼。中国虽然表示，尽管美国对伊朗进行经济制裁，仍将维持与伊朗的经贸关系，但在因与美国的贸易战争而受到不少打击的情况下，对与美国形成新的战线感到负担、战战兢兢。

中国外交部8日强调：“与伊朗的商业合作既开放又透明，是公正合法的。不违反任何联合国安理会决议，”“中国一向反对单方面制裁和美国在国际上适用国内法的方式。”去年中国和伊朗的贸易额达到了370亿美元（约413179亿韩元），比前年增加20%。中国还向伊朗提供大坝和发电厂、交通设施等基础设施建设的大规模贷款。

预计将于11月生效的第二阶段制裁措施——阻止伊朗原油交易，将会给中国带来更大负担。中国从伊朗进口的原油每天平均达65万桶，按目前市场价格计算相当于150亿美元（约合167745亿韩元）。中国的原油进口量中，7%来自伊朗。对于伊朗来说，对华出口超过了其总出口量的四分之一。据悉，中国国有石油企业向伊朗主要油田投资了数十亿美元。

美国对伊制裁侵害了中国的经济利益，中国不得不表示反对。但与在贸易战争过程中针对美国高额关税征收同等规模的报复关税不同，涉及制裁伊朗问题，中国的想法非常复杂。贸易战争以对抗美国贸易保护主义、维护自由贸易理念的名义，可以向欧盟和其他亚洲国家等国际社会呼吁共同应对。但是涉及制裁伊朗，中国很难要求国际社会发出一致声音。

况且，特朗普总统7日在推特上斩钉截铁地表示：“无论是谁，只要与伊朗进行交易，都无法与美国进行交易。”与伊朗进行交易的中国企业可能会成为美国制裁的目标。中国在对北韩制裁问题上也对上述二级抵制（制裁第三方）方式表现出敏感的反应。美国恢复对伊朗制裁之后，欧洲企业中断对伊朗的投资、从伊朗撤走，这种情况对中国而言也是负担。

中国共产党机关报《人民日报》的姐妹纸《环球时报》7日表示，“美国制裁伊朗，在（与中国）贸易战争以外，在（其他领域）点燃了新的火球，”把对伊朗制裁定义为美中矛盾的新因素。《环球时报》指出：“包括中国在内，向伊朗投资的国家面临了困境”，“中国很难抵制美国的方式。”该报表示，“中国反对美国的单方面制裁。（但）围绕伊朗问题，不能随意与美国对立”，“如何维持利益之间的平衡，是中国面临的外交挑战”。

# **中国与台湾就奥运会模式产生冲突**

“台湾的一些政治势力正在推动以台湾名义参加2020年东京奥运会的国民投票，对‘奥运会模式’发起挑战。因此，将在2019年在（台湾）台中举行的第一届东亚青少年运动会受到了政治性威胁和妨碍。”

由中国主导的东亚奥委会会议24日在北京召开。委员长刘鹏主张剥夺台中市的大会主办权，并提议通过委员们的举手表决决定是否剥夺举办权。刘鹏是担任中国全国人民政治协商会议外事委员会副主任的中方人士。

刘鹏和来自中国大陆、台湾、韩国、北韩、日本、蒙古、香港、澳门等地的9名委员出席了当天的会议。对于刘鹏的发言，委员们议论纷纷。台湾方面的委员抗辩称：“明年的大会是台湾首次适用奥运规则举办的大赛。不要夺走东亚青少年参加比赛的机会。”日本方委员提出异议称，“2014年确定由台中市举办，现在决定剥夺其主办权有点过分。”

但是，根据刘鹏的主张进行了投票表决，结果有7票赞成剥夺举办权。准备了4年的国际大赛瞬间变成了泡影。该大会原计划于明年8月24日至31日举行。只有台湾投票反对剥夺举办权，日本弃权。台湾总统蔡英文当天在脸书上表示反对称：“中国以政治力量粗暴剥夺了大会主办权。台湾国民绝对不接受这一决定。”台湾行政院长（总理）赖清德表示：“将正式向东亚奥委会提出抗议。”

最近在台湾出现了以台湾名义参加东京奥运会的国民投票请愿运动。目前，台湾只能以“中国台北（Chinese Taipei）”的名义参加奥运会。直到1968年墨西哥奥运会，台湾还可以以台湾名义参加奥运会，还拿着台湾国旗，高喊台湾为国家。

但是，随着从20世纪70年代起中国的国际社会影响力越来越大，国际奥委会在1981年决定，台湾只能以意为中国一部分的“中国台北”的名义参加奥运会。刘鹏提及“挑战奥运会模式”也是出于这个原因。2016年蔡英文执政以后，台湾政府拒绝接受意为台湾是中国一部分的“一个中国”原则，使得中国大陆和台湾之间的关系陷入僵局，这次再次爆发出一个不利消息。

打出“我们开始叫台湾”的标语、主导国民投票请愿运动的台湾田径国家队出身的纪政表示：“台湾是民主国家，法律允许使用（请愿国民投票）公民权利”，“不能接受剥夺举办权。”纪政曾在以台湾名义参加的1968年墨西哥奥运会上获得铜牌。

利用台湾问题向中国施压的美国和中国之间的矛盾也愈演愈烈。中国外交部24日向美国政府发出了最后通牒，要求美方“敦促把台湾和中国标注为不同国家的美国航空公司修正网站资料，如果25日之前不修改，将采取下一阶段措施”。路透社25日报道说，美洲航空、达美航空、夏威夷航空公司接受中国的要求，已经修改了标注。

# **“官僚腐败事件引发虚假疫苗事件”……愤怒的中国民心在社交媒体中扩散**

“（犯罪的人）自己调查自己?”“反应这样迟钝，领导人是怎么当的？”“贪官污吏继续留在位置上，商人继续赚钱。老百姓继续受苦。”

24日，在遭受造假婴幼儿疫苗事件打击的中国，中国共产党的腐败调查机构——中央纪律检查委员会表示，生产造假疫苗的长春长生生物技术公司的所在地吉林省的纪律检查委员会正在调查与该事件相关的责任人。但是报道这一消息的中国《新京报》微博账号上出现的回帖中，中国的民心却反应十分冷淡。该公司生产的被判定为不合格的DPT（百白破：百日咳、白喉、破伤风）疫苗接种了25万名婴儿，后来在重庆又发现了某公司的一种不合格疫苗在14万多名婴幼儿中接种。

中国网民说：“不想听官僚们的话。想看他们的行动。”“贪腐官员的职别比你们（调查人员）还要高！”“中央直接派人调查！省里的领导是腐败分子。”“事件发生都过了好几天了。舆论真假难辨。必须追究责任！”等，露骨地流露出对中国当局的不信任和敌对感。

重庆市的一名市民在得知子女接种问题疫苗的事实后立即在网上留言说：“要建立‘假疫苗受害者权利保护组织’。”不久后公安（警察）来到了他家，他被带到公安局接受调查后才获释。

不仅是总理李克强，连同正在非洲多国访问的国家主席习近平也公开指示调查真相，但是民心的愤怒直接针对中国共产党和政府。中国当局大举删除批评政府的网络留言的过程中，社交媒体成为直接表露对政府愤怒情绪的核心通道，这一点倍受关注。

在这种情况下，中国国家食品医药品监督管理总局副局长徐景合在接受官方媒体中央电视台“新闻联播”采访时，身穿英国名牌巴宝莉体恤，也遭到批评，认为很不合适。在22日播出的这一采访中，他穿了一件被推测为3200元人民币（约合53万韩元）的蓝色体恤。中国网民们愤怒地批评道：“从上到下都严重腐烂”，“这可能是他家里最便宜的衣服”。一名北京市民指出：“工资不高的公务员们居然穿着巴宝莉，堂而皇然地接受官方媒体的采访，这本身就令人寒心。”

# **中国与欧盟16日举行领导人会晤，会谈重组经济同盟**

第20届欧盟-中国领导人会晤16日在中国北京举行，预计将会比以往历届峰会演绎出更加沉闷的氛围。此次会晤的核心议题是经济。同样与美国进行贸易战、遭受同病相怜之苦的中国和欧盟，将针对美国特朗普政府的“孤立主义和保护贸易”，强调“多边主义和自由贸易”的共同分母。

更为急迫的是中国。美国对500亿美元的中国输美产品征收了25%的关税，接着又表示将对2000亿美元中国输美产品加征10%的关税，但中国没有合适的回应之策。因为去年美国的对华出口额只有1300亿美元。

中国强调今年是中欧战略合作伙伴关系建立15周年，努力营造气氛。不仅是欧盟委员会主席容克的对话伙伴李克强总理，中方还提前公布了中国国家主席习近平主席将会见容克和欧洲理事会主席唐纳德·图斯克的消息。

中国的算盘是把美中贸易战转变成美国对世界的格局，而不是美国对中国的格局。中国上周已将德国强烈要求的中国人权运动家刘晓波的夫人刘霞送往德国，向西方发出了强烈的求爱信。

中国的这种态度可能成为欧盟的绝佳机会。虽然欧盟也与美国在打贸易战，但与中国相比，现在还有很多应对措施。欧盟过去对中国限制市场准入、侵犯知识产权等问题提出过异议，正充分利用此次机会，争取中国的让步。

欧盟已经在汽车和化学等领域从中国得到了甜头。李克强总理上周访问欧盟最大国家德国，在柏林与德国总理默克尔举行会谈，双方达成了200亿欧元的经济协议。中国不仅拿出了德国汽车在中国设立生产工厂的许可，还决定对德国开发自动驾驶汽车提供帮助。在美国生产汽车出口中国的德国，如果在中国建厂，就可以以更低的费用向中国市场提供汽车。另外，德国企业巴斯夫与中国签订了协议，不与中国企业合资，将以100%控股的方式在广东省建立100亿美元规模的化学工厂。欧盟表示：“峰会上，双方将扩大战略关系，贸易和投资领域将成为最核心的议题。”

# **火热的贸易战争……看不见中方“消防员”王岐山**

中国国家副主席王岐山（照片）被认为是中美矛盾的“解决者”，但此次中美正式爆发贸易战争时却不见露面，其背景备受关注。

虽然有人分析王岐山可能赴美进行谈判，但多名中方消息人士10日表示：“目前王岐山副主席去美国的可能性很低。目前由国务院副总理刘鹤担任中美贸易摩擦中方谈判代表，这种情况下，由王岐山出面的理由很少。”

在今年3月的全国人民代表大会（相当于韩国的国会）上华丽回归的王岐山被寄予厚望，外界猜测他可能会担任调解外交、经济领域美中矛盾的“消防队长”。对于他在贸易战局面下不出面说话，有分析认为其中有难言之隐。

据悉，王岐山2009年至2012年担任副总理时主导中美战略经济对话，有着丰富的对美谈判经验，在美国拥有相当多的人脉。他在被任命副主席之前，曾接连秘密会见美国驻华大使特里·布兰斯塔德和前白宫首席秘书官史蒂夫·班农等核心人士。据悉，在出任副主席之后，王岐山还一直与美国企业主要负责人进行非公开会谈。

但正如“美国之音”中文版的描述，在中美贸易战箭在弦上一触即发的情况下，也没有看到王岐山露面。路透社8日在题为《消防队长副主席在避开美国的贸易战火》的报道中，援引一名西方高级外交官的话报道称：“中国似乎不愿意把王岐山投入贸易战。”

此前，在今年5月刘鹤参与的华盛顿第二次中美贸易协商中，中美双方达成协议，决定互不加征关税。当时，刘鹤在接受采访时曾自信地表示：“美中不会进行贸易战争，”但仅仅10多天后，白宫发表了对中国的加征关税计划。

刘鹤因此陷入了尴尬的处境。刘鹤虽然也是国家主席习近平的亲信，但王岐山被称为习近平的“左膀右臂”，是亲信中的亲信。如果王岐山参与对美磋商并像刘鹤一样失败，由此而引起的批评之声就会直接指向习近平，因此中国政府不愿投入王岐山。一名外交消息灵通人士表示：“虽然中美两国将会坐到谈判桌前，但此次贸易战争很有可能会长期化。如果受贸易战的影响，中国经济增速放缓、经济停滞，国民生活受到影响，中国政府将陷入烦恼的状况。”

# **中国进行回击……禁止美国半导体企业在华销售**

中国开始禁止美国芯片生产商美光科技公司在中国销售产品。有分析认为，在6日美国加征关税决定生效前夕突然出现的这一决定，是贸易战争的信号弹。

据彭博社当地时间3日报道，中国福州法院于本月2日发布了禁止美光在中国境内销售产品的预告。该决定适用于美光生产的固态盘、内存条、闪存等26种产品。总部设在美国的美光公司去年在中国的销售额占其总销售一半以上。

据悉，中国法院的这一命令是通过美光公司的竞争对手——台湾半导体企业UMC公布的。与中国国有企业福建晋华联手在当地建设DRAM生产工厂的UMC，从去年起与美光展开了专利侵权和商业机密的诉讼。去年12月，美光向美国加州法院提起诉讼，声称UMC侵犯DRAM半导体专利及商业机密，UMC则于今年1月向中国地方法院提起了针对美光的诉讼。

有分析认为，世界最大半导体市场的中国的这一决定，将使得美国和中国的贸易摩擦大举扩散到IT领域。彭博社报道说，美国此前对中国的IT企业华为、ZTE、中国移动通信等实施了制裁，美国芯片制造商高通也因中国的牵制而在收购荷兰芯片公司NXP遭遇困难。

# **动摇的对北韩制裁……美中会议检查会议已告中断**

据悉，负责检查中国是否如实执行联合国安理会对北韩制裁决议的美中联合工作会议，从今年起就宣告停顿。

美国特朗普政府的一名相关人士当地时间22日在接受本报的电话采访时表示：“由于中方的缘故，今年以来中美两国间的工作会议尚未举行。”另一名消息人士也表示：“自从金正恩在新年致辞中宣布参加平昌冬奥会之后，就没有召开过工作会议。”有消息称，特朗普总统21日在推特上表示：“最近，有传闻说，中国与北韩边境上出现了越来越多的漏洞，越来越多的东西流入北韩，”公开敦促中国履行对朝北韩制裁。有分析认为，此举也与中断美中工作会议有关。

这一会议负责集中检查对北韩制裁决议的执行情况，是因为特朗普政府坚持提出，尽管联合国通过了制裁决议，但通过中国国境仍有人暗中与北韩进行交易，制裁的实际效果低下，因此从去年年初起，根据中方的建议启动。此后中美双方往返于华盛顿和北京之间，每隔二三个月不定期地举行一次会议。去年9月27日的华盛顿会议是最后一次。

关于联合国安理会禁止或者限制对北韩进出口的提炼油、煤炭、铁和水产品，中国政府提交了具体的统计报告，美国也通过卫星观测等途径得到的情报进行确认，该会议则发挥了支持忠实执行的作用。美国评价称，占北韩对外贸易95%的中国积极参与制裁，最终使北韩走向了对话的舞台。特朗普总统也是每有机会就赞赏中国国家主席习近平的协助。但是有观测认为，受韩半岛解冻气氛的影响以及中国政府缺席工作会议，对北韩制裁可能会出现漏洞。

问题是，特朗普政府已把对中国施压的贸易筹码用尽，因此没有适合加强对北制裁的筹码。美中两国在经过两次磋商后，最近就推迟征收关税等问题达成协议，暂时搁置了争议。此前几乎每个月都要公布对北韩制裁对象的美国财政部，也从2月份起连续3个月没有公布制裁对象。

# **美与中国停止关税战争……贸易谈判达成一致** 中国最终向为减少美国对华贸易赤字全面施压的美国特朗普政府举起了白旗。中美在谈判中达成协议，中国将增加对美国产农产品的进口，并保护美国知识产权。 由美国财政部长姆努钦、中国国务院副总理刘鹤分别率领的美中经济贸易代表团，当地时间17日~18日在华盛顿举行第二轮贸易磋商，并于19日公布了协议内容。两国一致同意强化制度，把每年达到3750亿美元（约合406万亿韩元，去年美方统计基准）的美国对华贸易赤字减少一半，并防止中国侵犯知识产权。 两国代表团在共同声明中表示：“双方就为减少中国对美商品收支顺差达成共识，”“为此，中国将增加对美国商品和服务的购买。”协议表示，美国扩大出口的项目，主要是特朗普总统政治基础地区所生产的农产品以及汽车和能源产品。减少具体贸易顺差的目标值，并没有出现在共同声明中。据悉，美国提议写明每年减少2000亿美元，但遭到中国拒绝。 也有人指出，即使达成这一协议，但中国大幅减少对美贸易顺差并不容易。据分析，以消费大国美国和廉价劳动力为基础，发挥全世界生产基地作用的中国经济结构有差异，很难找到从根本上解决两国贸易不均衡的办法。特别是有评论认为，以增加低价农产品和能源产品对华出口的方式，作用有限。《纽约时报》分析说：“豆类大豆增加50亿美元，天然气、煤炭、原油等方面增加90亿美元的出口，是有可能的。即使包括高价的最尖端信息技术产品和飞机、军火，减少特朗普政府的目标值2000亿美元的逆差，是不现实的。” 刘鹤表示：“这次贸易磋商的最大成果，是双方达成协议，不打贸易战，并停止相互征收关税。”其意思是，虽然没有包括在共同声明内，但美中决定结束关税炸弹之战。中国媒体为了防止显得此次协议是中国向美国举白旗，强调了“贸易战结束了！”“共同胜利”等用词。

# **中国第一艘国产航母试航……摆脱沿岸前往远洋的“军事崛起”**

据悉，中国首艘国产航母、也是第二艘航母——“山东”号8日在辽宁省大连开始试航。人们在机场看到了中国国家主席习近平的专机和北韩国务委员会委员长金正恩的专机，有分析认为，两位领导人观看了中国国产航母的历史性试航。此前，辽宁省海事局发布公告说，从4日至11日将在渤海湾海域展开军事任务，禁止船舶进入。

有评价称，中国海军随着继辽宁舰之后拥有山东舰，在离本土海岸线数千公里的地方也可以展开军事行动，拥有了在海外对抗美国、寻求本国战略利益的能力。中国拥有两艘航母，预计将使原本致力于近海沿岸防御的中国海军走向远洋海军的重要里程碑。中国计划把航母的数量增至6艘。

○掀开面纱的山东舰的战斗能力

中国对从乌克兰引进的旧苏联航母进行改造，55000吨级的辽宁舰于2012年下水。通过以此积累的技术力量，中国生产了满载排水量更大的山东舰（7万吨级）。山东舰采用模块组建方式建造，从2013年11月着手建造，去年4月份下水。预计在完成试航后，将于明年投入实战部署。中国航母的名称以临海的省份命名，预计“山东”号之后的航母将被命名为“江苏”号。

“山东”号是柴油动力航母，长315m、宽75米，最大航速为31节。该舰可以搭载40架滑跃式起飞的歼-15舰载机。与搭载24架舰载机的辽宁舰相比，山东舰长度增加了10米左右，但舰载机数量增加了16架，可见中国在这段时间里研发出了能力最适化的航母设计技术。起飞甲板的倾斜度为12度，比最大14度的辽宁舰略有下降。据悉，与辽宁舰相比，山东舰舰载机在缩短起飞距离、节约燃料、增加武器装载量、强化航母结构等方面取得了诸多改善。

山东舰上安装了4个大型天线和可以探测周边360度的海上或空中目标的S波段雷达，装载了数十枚中国产短程导弹和中程导弹。

不过，在启动舰载机时，采用的并不是美国航母使用的电子式喷射方式，而是蒸汽喷射方式。相比电子式喷射方式，蒸汽喷射和滑跃方式的起飞，需要更长的距离。另外，由于需要减轻飞机重量，所以很难装载很多武器，最终导致其作战能力受到制约。因为不是核动力航母，所以每隔几天就要加一次油，这也是一大弱点。因为如果要开展远洋作战，就要率领多艘防御能力薄弱的大型供油船一同行动。

○远涉重洋，但仍不敌美国

中国海军此前从以沿海防御为主的“黄海海军”起步，后来发展成为守卫领海和领土的“绿海海军”，后来又寻求成为守护能源运输路线的“蓝海海军”。预计中国航母编队主要是以印度洋、西太平洋为中心进行活动，并发挥支援“一带一路”（丝绸之路经济带和21世纪海上丝绸之路）中“一路”的作用。但是，根据具体情况，也将起到牵制韩国和日本的作用，因此必然将导致东亚海洋秩序发生巨大变化。

如果中国今后再拥有三四艘航母，就将拥有名副其实的大洋海军的能力。预计，中国对各种国际悬案的介入也将有所增加。

尽管中国海军飞速成长，但与仅在亚太地区就拥有4个航母编队、在全球范围内拥有10个航母编队的美国海军相比，其数量和质量仍有很大差距。美国军舰总吨数达950万吨，但中国仅拥有不到50万吨的海军军力，不到美国的5%。军事专家们同时认为，中国运用的歼-15舰载机的战斗能力，也不如美国海军最新战机F-35。此外，美国拥有超过20万名的海军陆战队兵力，但中国仍在为从2万人增至10万人而努力。

# **王毅访问北韩，似乎将要求举行南北美中四方会谈**

中国国务委员兼外交部长王毅2日上午抵达平壤。据悉，王毅将在平壤逗留至3日，将与北韩外相李勇浩进行会谈，并会见北韩国务委员会委员长金正恩。

截至2日下午，中国和北韩都没有报道有关王毅在平壤境内行踪的消息。据观察，王毅此行是在北韩-美国首脑会谈之前，向北韩表明中方的具体立场，其中包括△中国国家主席习近平可能在北韩-美国首脑会谈前后访问北韩、△南北韩领导人在《板门店宣言》中阐明的和平机制磋商必须是有中国参与的南北美中四方会谈等。据悉，王毅还将听取有关南北韩首脑会谈结果的介绍。

南北首脑4月27日在《板门店宣言》中表示，为了构建和平机制，“将推进南韩、北韩、美国三方或南北美中四方会谈。”“美国之音”中文版分析称，“王毅为了阻止‘跳过’中国而急忙访问北韩。”这是中国积极介入韩半岛局势外交的一部分，其的的是要表明中方的立场，在驻韩美军撤离等直接关系到中国在韩半岛利害关系的和平协定谈判，中国不能被排在外。

这是中国外交部长自2007年7月以来时隔11年再次访问北韩。王毅和北韩方面如何协调习近平主席的访问时间备受瞩目。北京外交当局者表示：“从常识来看，（习主席访问北韩的时间）不会在北韩-美国首脑会谈之前。”也有观测称，习主席在访问北韩后可能会访问韩国。

# **中国北京大学的学生们，突破审查喊出“我也是”**

中国名牌大学的大学生突破政府部门的审查，正在扩散“我也是（#MeToo）”运动。在北京大学，有学生要求透明地公开过去性骚扰事件的相关资料，学校方面虽然堵住了他们的嘴，但是校园里贴出了支持学生的大字报，影响正在扩大。在人民大学，学生们针对怀疑性骚扰的教授展开了抗议示威。有人评价说，自1989年天安门事件之后，首次出现了有组织的学生运动。

北京大学学生岳昕23日在微信（相当于韩国的可可聊天）正式账户上以公开信方式，上传了批评学校措施的文章。据文章称，包括岳昕在内的8名北京大学学生，9日申请公开“1998年因遭受该校教授沈阳性暴力后不堪痛苦而自杀的高岩事件”的相关资料。但是学校方威胁称，“还想顺利毕业吗？”，20日回复学生称“没有可提供的资料”。

23日凌晨，校方相关人士和岳昕的母亲闯入岳昕正在睡觉的宿舍。有关人士要求岳昕“删除手机和电脑中有关要求公开信息的全部资料，并写下保证书不再介入此事。”据岳昕称，校方制造假消息并向其母亲施压，要求把岳昕带回家管教。《金融时报》报道称，要求公开信息的其他学生也因校方的恐吓和监视而痛苦不堪。

23日夜间，北京大学贴出了“声援勇士岳昕”为题的大字报。中国在法律上禁止贴大字报。大字报的作者在BBC的中文版上表示：“几个小时后，学校警卫们撕掉了大字报，‘北京大学’一度成为网络不能搜索的敏感词。”岳昕的公开信和大字报照片因中国当局的审查被删除后，仍在继续上传。

上周，在人民大学被疑对女学生进行性骚扰的经济学教授上课的教室前，有40多名学生展开示威，并要求“学校和教授就此事作出答复”。保安人员们阻止示威学生进入教室，并带着教授离开。据《金融时报》报道，据悉，问题教授最终已被解雇。听到示威消息的一名中国大学生对本报表示，“校园内的示威，我一次也没有见过，”感到十分惊讶。据悉，最近清华大学也有助教性侵学生的说法，但校方一直保持沉默。

# **中国媒体：“用打抗美援朝的意志来打对美贸易战”**

中国共产党机关报《人民日报》的姐妹刊《环球时报》在8日的社论中指出：“要以抗美援朝（中国在6·25战争时期为对抗美国帮助北韩而参加的战争）的意志，坚决粉碎美国特朗普政府的贸易攻击。”社论说：“抗美援朝的发生是因为美国军队打到了鸭绿江边，今天美国发起的贸易战也在冲击中国的核心利益线。”社论强调：“我们无处可退的危机感，和为维护国家根本利益决不可对美方让步的坚定意志正在中国全社会凝聚起来。”《环球时报》还主张，“中国打贸易战的武器和弹药十分充足。我们知道将付出一些牺牲。”

此外，随着美国海军“罗斯福”号航空母舰（CVN-71）起航，由“邦克山”号宙斯盾巡洋舰（CV-17）、“桑普森”号导弹驱逐舰（DDG-102）等组成战团的第九航母打击团，6日起在南中国海南部海域与新加坡海军展开了联合军事演习。中国唯一的实战部署航空母舰“辽宁”号从5日起在南中国海海南海域与40多艘军舰、潜艇以及12架轰-6K战略轰炸机一道，进行大规模演习。如果“罗斯福”号接近中国主张拥有主权的海域展开“航行自由”行动，人们担心会出现美中两国航空母舰之间的首次对峙。

# **中国下决心反击……对美国产大豆实施报复性关税**

美国瞄准中国的尖端产业，对1300种中国产进口产品引爆了25%的“关税炸弹”。不顾中国将对美国产猪肉和农产品实施报复性关税，按预告打出了强烈的一拳。

美国贸易代表部当地时间3日公布了征收25%关税、总额500亿美元（约合52.86万亿韩元）的1300项中国产进口产品。美国贸易代表部当天公布的58页征税目录中，包括半导体、通信设备、锂电池等尖端技术产品，以及钢铁、金属、铝制品、发电机、摩托车、航空器配件、武器、医疗器械等各种产品。特别是高关税产品中，包括了中国推出的尖端产业培养战略“中国制造2025”中相当数量的品种。

美国贸易代表部当天声称，“中国为获得美国公司的敏感商业情报和贸易机密，支持并进行电脑网络的无端入侵和技术窃取，”“（征收关税的措施）从对美国经济遭受的损失和为消除中国的有害政策、程序和行为这两个层面上，水平都是合适的。”美国总统特朗普批评中国是“贸易赤字的领袖”。

中国则立即反对美国贸易代表部公布征税品种。中国商务部在美国贸易代表部宣布后1个多小时，就以发言人名义发表声明警告说：“中国坚决反对美方的做法，近期将依法对美国产商品采取同等强度和规模的对等措施。”中国针对美国的钢铝报复关税，从2日开始对猪肉等30亿美元（约合31700亿韩元）美国产品征收高率进口关税。

# **北韩最高领导人参观“中国硅谷”中关村后回国**

疑似北韩劳动党委员长金正恩或者金正恩妹妹金与正的北韩最高层人士，27日接受中国当局最高级警卫的礼宾待遇，穿梭在北京西南部各处。特别是，北韩领导层人士访问了父亲金正日访华时必去的“北京硅谷”中关村，备受关注。

北韩最高领导层人士下榻的国宾馆——钓鱼台国宾馆当天也部署了大量武装警察，阻止记者们的采访，营造了森严的气氛。北京的一位消息人士透露说：“据我所知，北韩最高领导层人士住在（爷爷）金日成每次访华时下榻的钓鱼台国宾馆第18号楼。”

当天上午9时30分左右，最高领导层人士乘坐的两辆汽车在10多辆中国公安车辆的护卫下，驶出了钓鱼台正门。汽车驶向被称为北京硅谷的城西中关村。据悉，这位人士访问了中关村最大的电脑电子器械商街——海龙大厦。据悉，海龙大厦内的电脑电子器械商店已达到韩国首尔龙山电子商街的数倍。

金正日首次访问中国的2000年5月以及2010年5月、2011年5月都访问了中关村。访问中国的北韩最高领导层人士根据金正日访问中国的传统造访中关村，参观了电脑电子设备商业街。有分析认为，北韩领导层人士对科技非常感兴趣，与致力于发展该领域的国产化有关。

在当天北韩领导人的访问中，中方进行交通管制甚至禁止人们围观道路，引起中国国民和海外游客的抱怨和不满。

在钓鱼台的所有出入口都有公安，附近的200米区间被管制，记者被阻止接近。当天上午，在中关村一带，随着进行交通管制，市民们表达不满和交通管制、北韩领导人车队的照片接连不断出现在微博（中国的推特）上。一位市民甚至讽刺说：“应该是金三胖来了。”金三胖是中国网民贬低金正恩的典型称呼。

从当天上午11点开始，位于北京南部的北京火车站的贵宾出入口开始管制，从11点20分开始，北京火车站附近的道路也被部分管制。从中午12点30分开始，来自北韩的“1号”专列等候在站内。从上午11时开始，分割紫禁城和天安门广场的北京中心道路长安街和天安门广场西侧的道路不时被管制和解除。在这个过程中，公安们要求路过的市民和游客们不要站在人行道上，并且制止人们眺望被管制的道路。

还有消息说，当天中午12点30分左右，北京南部天坛公园周边的道路被管制。据观测，北韩领导人从中关村出发后，选择了经过人民大会堂北侧长安街到达北京火车站的路线。据悉，途中还在某处吃了午饭。

另外，北韩与中国接壤的辽宁省丹东，能看到鸭绿江铁桥的中联酒店，中断了截至27日能看到鸭绿江边的客房的预约。

# **中国应支持北韩与美国对话、成为“韩半岛和平”的襄助者**

中国国家主席习近平昨天会见了青瓦台国家安保室长郑义溶，对南北韩、北韩与美国的首脑会晤表示欢迎和支持，并表示“希望取得成果”。郑义溶与负责外交事务的国务委员杨洁篪和外交部长王毅分别会晤，并各自共进午餐和晚餐。在中国的重大年度政治活动“两会”（全国人民代表大会和全国人民政治协商会议）期间，包括习近平主席在内的中国外交最高层都抽出时间来，是相当罕见的。可以说，这反映出对韩半岛局势剧变的高度关注，同时也是因为担心出现“越过中国（排除中国）”。

中国对南北韩和北韩-美国首脑会晤表示欢迎和支持，但恐怕内心是复杂而不舒服的。一段时间以来，一直是中国牵线北韩与美国之间的对话、担任北韩核问题六方会谈主席国，发挥了积极的斡旋作用。但此次北韩和美国跳过中国、通过韩国的斡旋决定举行历史性的首次首脑会谈。而且，在金正恩执政后，北韩与中国之间还没有举行过首脑会谈。所谓“北韩-中国血盟”已成为过去之事，但中国却不能袖手旁观北韩和美国直接交易。

尤其是，中国一直提出的北韩核问题解决方案，即同时暂停北韩核导弹挑衅和韩美联合军事演习的“双暂停”主张也变得黯然失色。虽然中国表示奥运会期间暂停联合军演就是实际上的暂停，但北韩后来又声称谅解韩美举行联合军演，没有要求暂停就承诺暂停核导试验。相反，北韩直接同美国进行对话，是因为中国对对北韩制裁的协助。中国更为严格地实施对北韩的制裁后，北韩不是向中国、反而开始向美国“招手”。今后，如果北韩与美国关系迅速发展，在与美国的竞争中将北韩视为友好的缓冲地带的中国，可能将不可避免地全面修改其对北韩战略。

韩国总统文在寅昨天也表示：“我们想要实现的，是目前世界没有成功过的大转变之路。如果我们取得成功，将是世界史上戏剧性的变化，韩国将是其主角。”北韩与美国关系的变化必然会引发韩半岛乃至东北亚秩序的根本性改变。对于迅速的变化，中国也会起戒心。但是，美国不能突然代替中国的作用。北韩虽然与中国保持距离，但最终依赖生存的国家还是中国。

迄今为止的过程，是中国提出并坚持的韩半岛无核化、维护半岛和平与稳定、通过对话和协商解决等北韩核问题三原则的实现过程。中国刚刚通过了删除禁止国家主席三连任条款的修宪案，赋予了习近平主席强大的地位和权限。中国今后全面展开“大国外交”的第一个试验台，将是通过实现北韩无核化构建东北亚新的和平秩序。中国以朝鲜的“保护国”自居的时代已经过去。作为东北亚的领导国家，在制定新秩序的过程中，应当发挥积极的作用。

# **特朗普与金正恩5月举行元首会谈，周边各国怎么看？**

一直强调美国和北韩应尽早进行对话的中国外交部，9日在例行记者会上对美国总统特朗普表示要在5月之前与北韩劳动党委员长举行会谈表示欢迎。中国官方媒体当天也以快讯报道美国与北韩将举行首脑会谈，表示感到十分惊讶。

　平时不太使用刺激性表述的中国共产党机关报《人民日报》的官网人民网，也使用“重大事件！特朗普同意5月之前与金正恩会晤”的题目，播发了快讯。人民网还在另外一篇报道中指出，“虽然北韩核问题的外交解决前路漫漫而且艰难，但谈判可以消除爆发战争的忧虑。”正式代言中国政府立场的官方媒体新华通讯社也播发快讯“重大变化！特朗普与金正恩将在5月之内会面”。新华社的另一篇报道称，“特朗普总统表示，‘韩半岛问题正在出现重大进展’。他还表示在达成协议前美国将继续对北韩进行制裁。”

　《人民日报》的姊妹报纸《环球时报》的官网环球网也把韩国总统国家安保室长郑义溶在记者会上宣布美国总统特朗普愿意与金正恩会面的消息称为“重大声明”“突发新闻”。环球网报道称，“太过突然了。北韩与美国联手发动了突然袭击。”

　据外交消息人士透露，中国一方面欢迎北韩与美国对话，同时也颇为担心在今后的无核化谈判中会出现中国被排除在外的“越顶中国”局面。尽管中国因对北韩制裁付出了与北韩关系恶化的代价，但在真正的对话进程中却被排斥在外。因此有预测称，中国将视南北韩、北韩与美国首脑会晤的情况，着手改善与北韩的关系。

　中国外交部似乎意识到了“排斥论”，再次强调称，“中国在韩半岛问题上发挥了积极和建设性的作用，得到了国际社会的认可”。中国外交部长王毅在8日的记者会上也强调，“这也证明，中国提出的双暂停（北韩暂停核导试验和韩美暂停联合军演）是正确的处方。根据（同样由中方提出的）双轨并行（同时推进无核化与和平协定）方向，在无核化进程中应解决北韩的合理安全关切。”分析认为，中国是想强调目前的对话局面正是朝着中国所提出的解决方向前进，以此来驳斥“排斥论”。

# **中国国防开支增加8%，创历史纪录，加快与美国霸权竞争**

去年10月份中国共产党第十九次全国代表大会（十九大）上退下来之后一直没有公开露面的前中央纪律检查委员会书记王岐山，5日首次在公开场合出现在媒体面前。作为中国国家主席习近平的左膀右臂，王岐山很有可能出任国家副主席，当天出现在北京人民大会堂举行的全国人民代表大会（全人大、相当于韩国的国会）开幕式上，排在习近平主席、李克强总理等7名中共政治局常委（最高领导层）后面。

　在李克强朗读评价过去5年和2018年政府计划的政府工作报告的过程中，记者捕捉到了象征实质性二号人物不是李克强总理而是王岐山前书记的场面。李克强刚刚开始作政府工作报告，除习近平主席外，包括其他政治局常委、政治局委员、中央委员和2970名全国人大代表，都打开工作报告的小册子跟着阅读。只有习近平和王岐山没有打开工作报告小册子，连眼神也没有瞄一下。习近平在李克强开始作政府工作报告40多分钟后才打开了小册子，但也只扫了几眼自己想看的内容。有评论认为，这是预告“习王（习近平与王岐山）”体制上台的场面。

　李克强在当天长达1个小时50分钟的政府工作报告中，包括“坚决拥护以习近平总书记（习近平的党内职务）的核心地位”等在内，先后13次提到习近平的名字，不断赞扬习近平。李克强的政府工作报告结束后，关于取消习近平国家主席任期限制的宪法修正案草案被提交审议。提交审议并解释修宪理由的程序长达50多分钟，但中国官方的中央电视台只实况转播了李克强总理作政府工作报告，没有转播提交宪法修正案草案的过程。估计是考虑到舆论反对等敏感性。

　但是，人民大会堂现场，却充满了赞成取消任期限制的声音。提交宪法修正案草案时，出现的唯一一次掌声，就是在介绍“取消不得连任两届以上”的限制时。

　当天的全国人大会议上，中国提出了与去年持平的6.5%的经济增长率目标。政府工作报告中提出，国防预算将比去年增长8.1%。由此，中国今年的国防预算将达到创纪录的11069亿元人民币（约合1891249亿韩元）。继2016年和2017年先后缩减7.6%和7%之后，中国国防预算再次出现大幅增加。

　分析认为，虽然中国军费开支没有恢复到2016年之前的两位数增长率，但在去年的十九大上，习近平主席阐明了到2050年成为世界第一大军事强国的强军梦之后，中国启动了与美国进行军事霸权的竞争。

　中国以最近国家安全处于威胁之中为由，正在尝试建造核动力航母等各种军备扩张。

此外，李克强总统似乎是着眼于美中贸易战争，针对美国强调称，“中国主张通过平等协商来解决贸易争端。必须反对贸易保护主义，坚决维护合法权益。”

# **中国知识分子反对习近平长期执政，“要回到毛泽东时代吗？”**

中国共产党25日建议修改宪法，删除“国家主席连续任职不得超过两届（最长10年）”的内容，中国国内舆论的反对之声正在扩散。这是因为，中国人的担心正在加剧，“国家主席习近平想回到终身掌权的毛泽东时代吗？”

　中国著名女性社会学家、中国社会科学院教授李银河在博客上指出，“恢复终身制是历史的倒退。将会让中国回到毛（泽东）时代。”曾任中国共青团机关报下属《中国青年报》冰点栏目编辑的李大同也向出席将于下个月开幕的全国人民代表大会（相当于韩国的国会）的北京市市长陈吉宁等55名北京人大代表致公开信，呼吁“对修宪方案投反对票”。中国女性企业家王瑛发表声明说，“删除任期限制是背叛，是开时代倒车，”“即使要求我沉默，我也不会沉默。”天安门民主化运动时期的学生领袖、现流亡美国的王丹也和100余名中国学者一道发表声明称，“这暴露了习近平有皇帝野心。”

　日本《朝日新闻》27日报道称，习近平曾在去年10月的第十九次全国代表大会（党代会）后不久，在上海向共产党元老、前国家主席江泽民表示想废除任期限制，但江泽民表示“绝对不行”，遇到了强烈反对。

　微博（中国版推特）上，包括“我不同意”“移民”“登机”等词汇的文章成为审查对象。这是因为，在宣布建议删除任期限制后，有关移民的检索词使用迅速增加，而中文“登机”一词的发音与“（皇帝）登基”相同。终身制、登机、长期执政、反对修宪等词汇已经不能检索。英国《金融时报》援引中国主要门户网站百度新闻匿名职工的话报道称，“至少有13家互联网新闻企业接到了当局要求在显著位置刊登支持修宪报道的指示。”

# **香港媒体：“中国把南中国海上的人工岛用作‘军事指挥中心’”**

有消息称，中国在与东南亚国家存在领土主权争议的南中国海上建设人工岛，并企图把这些岛当作指挥周边军事设施的中心。随着美国持续实施“航行自由”行动、派遣军舰进入中国在南中国海上主张拥有主权的岛屿12海里内，南中国海上的军事紧张正在加剧。

　据香港《南华早报》18日报道，美国战略国际问题研究所通过航空照片，确认了中国最近在南中国海斯普拉特利群岛（中国称南沙群岛）的Fiery Cross Reef（中国称永暑礁）的西北部，集中部署了电信塔和高频率雷达设备等通信装备。在该暗礁上，中国在10万平方米的地面上不仅建造了长度达3000米的跑道，还为轰炸机、空中加油机、运输机等设立了机库。美国同时认为，中国不仅在Fiery环暗礁上，而且也还把斯普拉特斯群岛的苏比暗礁（中国名渚碧礁）、美斯济夫暗礁（中国名美济礁）等7个暗礁建设成了部署军事设施的人工岛。《南华早报》引用中华圈军事专家的话分析称，“Fiery环暗礁上的设施，是中国建设的军事设施中被用作通信中转的基地。”

　这一形势正在加剧美中之间的神经战。被提名担任美国驻澳大利亚大使的美军太平洋司令部司令哈里·哈利斯14日在美国国会众议院军事委员会的听证会上，对斯普拉特利群岛的军事基地化表示忧虑，并指出，“中国将在人工岛上部署高性能军事防御装备后，声称拥有其主权。”

# **为对抗美国，中国拟部署俄罗斯版“萨德”**

中国不久将部署被称为“俄罗斯版萨德”的S-400地对空导弹系统，其背景令人关注。分析认为，虽然部署地点尚不清楚，但如果选择在山东半岛部署，韩半岛将进入雷达的探测范围，一旦韩半岛发生战争，将成为牵制韩美军队动向的布子。

　据俄罗斯塔斯通讯社和香港《明报》19日报道，俄罗斯最近向中国提供了导弹控制中心、雷达基地、燃料供应设备等S-400地对空导弹系统。中国2014年签署了从俄罗斯引进3套S-400系统的协议，目前部署的是第一套。据悉，中国计划在2019年前结束S-400系统的引进。

　S-400的射程为400公里，最高飞行高速为185公里，雷达可以追踪700公里以内的目标，可以同时击落不同高度和不同射程的战斗机和导弹。在电磁波受到干扰的情况下，它也能继续工作。S-400被认为具有击落F-35隐形战斗机等美国尖端战斗机的能力。还有分析认为，它比射程200公里、最大高度150公里的萨德更具威胁。

　《明报》称，如果S-400部署在福建省等中国东南沿海，可以把台湾全境纳入射程，对台湾空军进行打击。最近中国出现了武力统一台湾的说法，引起了关注。

　但是，也有分析认为，中国为了应对美国的尖端战斗机和隐形巡航导弹，才推动S-400系统的引进。俄罗斯也在符拉迪沃斯托克等靠近北韩的远东地区实战部署了S-400系统。

# **中国改革开放第一号深圳，撤销经济特区管理线**

 广东省深圳市是中国改革开放的象征。曾经将中国这第一个经济特区和大陆其他地区分隔、在人员物资移动方面进行限制的特区管理线，在设立36年后彻底成为历史。分析认为，这一决定具有很大的象征意义，它向国内外宣告，中国经济已经发展到了不再需要隔离线的程度。

　据中国媒体16日报道，中国国务院正式决定，撤销深圳经济特区管理线（第二道关门）。广东省政府此前请求“取消这一市内的实质性边境，以促进相关地区的协调发展”，并得到了中央政府的批准。

　邓小平1978年宣布改革开放，并在两年后的1980年，将深圳市南部与香港相邻的部分地区（面积327平方公里），宣布为中国头一个经济特区。当时仅是一个3万人口小渔村的深圳，如今已成为中国的硅谷，集中了众多世界级信息技术企业。

　中国在宣布深圳成为特区后，为在特区内投资的外国人提供了自由投资等经济活动、设立工厂、免税等优惠条件。但是，特区外的中国人必须经过批准才能出入特区，为此还在1982年在特区周围设立了长达136公里的铁丝网，并设立了由武装警察值守的163个哨所和10处检查站。由于经济特区以南就是1997年才归还中国的香港，因而管制更加严格。

　人们把香港与深圳之间的边境线称为“一线关”，把深圳与内地之间的管理线称为“二线关”。二线关的存在，说明了深圳在中国国内独特的地位，同时也说明，中国并没有完全对外打开大门。二线关还发挥了阻止中国社会主义因迅速受西方经济和文化影响而陷入危机的作用。

　随着经济的迅速发展，深圳经济特区在2010年扩大到深圳全市。随着深圳市地铁贯通市内各地，原有的管理线变得有名无实。相反，管理线和检查站的运营每年还需要投入数千万元人民币，并成为交通等城市发展的障碍。市民们来来往往必须经过检查，也带来了不便。虽然取消管理线和检查站巡察路的工作早在2013年就已开始，目前也在进行之中，但在制度上，分割深圳特区与中国大陆其他地区的这一隔离线仍然存在。中国媒体称，“由于这一措施，深圳再次成为改革开放的最前线”，表现出期待。不过，国务院在相关决定中指示，“要加强深圳与香港、澳门之间边境上的一线关的管理管辖”。

# **美中贸易战一触即发**

中国最大的电子商务企业阿里巴巴的代表性卖场淘宝再次被列入美国政府的不良市场黑名单。有分析认为，美国和中国在贸易金融领域磕磕碰碰之际，中国去年对美贸易创下有史以来的最大顺差，美中已处于贸易战争的前夜。

　据BBC中文网站14日报道，美国贸易代表部声称淘宝上充斥着侵犯知识产权的商品，继2016年之后，去年再次把淘宝列入不良市场名单。美国贸易代表部把侵犯美国知识产权和给美国劳工带来严重损害的25家在线商家和18家线下市场列入黑名单。其中中国的在线和线下市场，包括淘宝在内共有9家，占总数的20%。美国贸易代表部虽然承认淘宝为驱逐假货作出了努力，但仍然以“没有公开显示假冒商品规模的客观指标，无法客观证明假货销售量的减少”为由，再次将其列入黑名单。

阿里巴巴立即表示反对。阿里巴巴指出，“在贸易保护主义抬头、高度政治化的环境中，阿里巴巴成了企图在特朗普政府拿到高分的美国贸易代表部的牺牲品。美国贸易代表部的措施不是为了保护知识产权，而是实现美国政府地缘目标的工具。”

　 根据中国海关总署12日公布的数字，中国对美国的贸易顺差为2758亿美元（约合293万亿韩元），是有史以来的最高纪录。“美国之音”中文版指出，“这一数字无助于缓和美中贸易关系的紧张。美中已处于贸易战争的前夜。”

　本月初，阿里巴巴的子公司、移动结算企业蚂蚁金服收购美国汇款公司“速汇金”的计划，也遭到了美国外资投资委员会（CFIUS）的拒绝。正因如此，有分析认为，美国制裁代表中国的阿里巴巴，标志着贸易战争已经打响。

　美国政府有关部门结束了就进口中国产钢铁产品等进对美国国家安全有多大损害进行的调查，11日向特朗普总统提交了报告。特朗普将在90天内决定采取什么政策。“美国之音”报道称，美国还将在本月内公布对各种中国商品的调查结果。

专家们认为，如果美国针对中国商品提高关税或采取事实上的大规模限制措施，中国很可能会以同样方式进行报复。美国国内担心，不仅波音飞机、汽车、集成电路、大豆等美国主要对华出口商品受到影响，中国还会对好莱坞电影进行限制。

# **中方：“不解决萨德问题，中韩关系就不可能恢复”**

“在韩半岛部署的萨德是中韩关系的绊脚石。萨德问题如果得不到彻底的解决，中韩关系就不可能恢复。”

　中国人民外交学会副会长魏苇11日在北京举行的“韩中未来发展智库高级别论坛”作出了上述表示。在文在寅总统访华两天之前举行的这一论坛，目的是提高两国间的相互理解和友好氛围、讨论改善关系的方向，但中方与会者集中谈到了萨德问题。

　魏苇谈到了中国官方媒体《环球时报》11月29日提出的“三不一限”问题。“三不一限”的主张，不仅包括两国协议中的三不（不追加部署萨德系统、不加入美国导弹防御系统、不发展韩美日三国同盟），还包括限制萨德的使用。魏苇表示，“中国当局虽然没有直接说出口，但对于三不一限的立场是一致的。”韩国某外交消息人士表示，“两国协议没有限制萨德的使用，即没有一限，这是（中方）在打舆论战。”

　中国全国政治协商会议外事委员会委员于洪君表示，“虽然有复杂的事由，但去年韩美关于部署萨德的决定是不合适的。应该想到，这是对邻国利益的侵害。”他表示，“中韩关系因为萨德问题降温，让人感到难过。责任在于韩国。”察哈尔学会国际顾问委员吴思科也表示，“引进萨德，对在半岛构建和平没有任何帮助。”

　清华大学社会科学院副院长赵可金表示，“韩国低估了中国对萨德的反对。韩国明知道部署萨德的原因，但没有讲真话。”分析认为，他是指韩国加入了美国的防导体系。赵可金还表示，“如果继续维持韩美同盟，它将成为韩中关系的绊脚石。”指称“韩美同盟”本身而不是“韩美日三国同盟”，是对韩中关系产生负面影响的因素，这是十分罕见的。

　韩国安保战略研究院顾问裴基灿（音译）反驳道：“在北韩宣称完成核武装的情况下，中国仍对韩国采取强硬的立场，难道中国希望韩国也走向核武装吗？”

　虽然双方攻防十分尖锐，但也就应把文在寅总统访华当作改善两国关系的机会进行了很多探讨。中国察哈尔学会会长（全国政协外事委员会副主任）韩方明表示，“最近的（萨德）矛盾不符合两国的利益，期待此次两国元首会谈成为改善这一重要关系的契机。”

　韩国外交部公共外交大使朴恩河（音译）表示，“论坛一致认为，某一领域发生的矛盾需要妥善管理，使它不扩散。”

# **中国生了气，超出联合国决议单方面制裁北韩**

 分析人士认为，中国政府超出执行联合国安理会决议的范围，下令实行禁止赴北韩旅游的单方面制裁，其背景是中国国家主席习近平对北韩感到愤怒。习近平不久前派中国共产党对外联络部长宋涛访问北韩，想向北韩劳动党委员长金正恩转达口信，但遭到了拒绝。

　在美国政府今年8月禁止本国公民前往北韩旅行后，中国政府也下令限制赴北韩旅行，将对北韩形成相当大的压力。据悉，北韩每年通过旅游项目赚取相当于4400万美元（约合478亿韩元）的外汇。其中，中国占80%。根据中国国家旅游局2012年最后一次公开的统计，有23.7万名中国人赴北韩旅游。

　但是，随着28日有消息说包括北韩游客集中的丹东和沈阳在内，辽宁省和吉林省仍在允许赴北韩旅游，限令的实效性有待观望。此外，由于需求减少，上述地区有不少旅行社已经有一段时间不再募集游客。

　据本报今天确认的结果，北京的A大型旅行社表示，“北韩游旅行商品还是没有，这一次没有接到（禁止）通知。”B旅行社回答，“下个月7日有从丹东出发的（北韩）5日游商品，下个月中旬有往返双飞的（北韩游）商品。但不清楚能不能预约。”山东地区的旅行社也都表示，没有赴北韩的旅行团。沈阳地区的旅行社表示，“下个月16日有4日游的旅游团。（新义州）一日游哪天都能出发。”吉林地区的旅行社表示，“冬天没有赴北韩的旅游团。”

# **中国学者：“中国不应犹豫，应与韩美磋商北韩剧变事态”**

在中国共产党对外联络部长宋涛作为中国国家主席习近平特使访问北韩的前一天，中国名门大学的一名教授在由韩国政府在首尔主办举行的国际论坛上主张，为防范北韩体制崩溃等剧变事态，韩美中三国应开展对话。这一事例说明，围绕北韩核武器和导弹挑衅，中国学界内部的反对之声正在扩散，引起了外界的关注。

　上海同济大学政治和国际关系学院院长夏立平（照片）16日在首尔大希尔顿饭店举行的“2017东北亚合作论坛”上表示，“中国不能再犹豫不决，现在是时候与美国和韩国就北韩的剧变事态进行磋商了。”他还提议三国举行“紧急计划对话”，提出了北韩体制崩溃时谁来掌控北韩的核武器、如何处理北韩难民问题、发生危机时谁来负责恢复北韩国内的秩序、危机后如何政治整顿韩半岛等议题。

　他表示，“由于中国也积极参与，对北韩的制裁正在得到加强，但在北韩弃核这一前景上，很多人持悲观论。考虑到北韩无视中国的外交努力，并继续进行核武器和导弹的挑衅等事态的比重和状况的危险性，现在中国应毫不迟疑地与美国和韩国进行真诚的对话。”

　夏立平表示，上述内容是中国感到感到非常头痛的议题，但美中就此展开对话和磋商，将有助于打开两国战略胶着状态。

# **习近平的特使宋涛访问北韩**

 “中国（赴北韩）特使希望目的取得进展。”

　北韩核问题六方会谈韩美两国的首席代表磋商17日在济州岛举行，美国国务院北韩政策特别代表尹汝尚在会谈结束后对记者作出了上述表示。韩方首席代表、外交部韩半岛和平交涉本部长李道勋也表示，“在当前时间点，应该赋予它相当大的意义，”期待当天赴北韩访问的中国共产党对外联络部长宋涛能够融化冰冻的涉北韩关系。

　事实上，韩美代表就北韩持续两个多月的“挑衅休眠期”中金正恩的意图进行了分析。据悉，他们尤其对以宋涛访问北韩为契机，能否把北韩拉进六方会谈等多边外交渠道进行了磋商。美国总统特朗普前一天也在推特上就中国派特使前往北韩一事，认为这是一个“大动作”。外交界普遍认为，特朗普前一天推翻人们的猜测、没有把北韩再次列为支持恐怖主义国家，也可以解读为观察中国今后举动的信号。

　但是，韩美代表明确表示，以制裁和施压为重点的对北韩政策基调仍然是优先课题。尹汝尚在磋商开场白中表示，“能否（在北韩核问题上）取得进展，（仍然）面临着诸多老难题。”只要北韩没有就无核化发出具体的信号，就不会轻易转入放宽制裁的局面。据某消息人士透露，韩美代表就提高韩美情报交流的水平、确保制裁的实效性等进行了磋商。尤其值得一提的是，尹汝尚表示，最近中国清退北韩劳工等，对北韩的制裁有效果，再次强调了中国的作用。

# **令人晕眩的旗袍**

“那个时代已经过去。现在什么也没有留下。”这是王家卫导演的电影《花样年华》中的一句台词。在这部含蓄描写无法实现的爱情的电影中，演员张曼玉换了几十身中国传统服装旗袍上场，散发出致命的魅力。

　▷旗袍的特征，是高领、高开衩和紧贴身材。旗袍来源于统治清朝的满族八旗军服装，后来由男女共用发展成为女性专用。1920年代以上海为中心，开始流行强调迷人氛围的改良型旗袍。1972年美中元首会晤时，尼克松总统的夫人帕特女士对旗袍的性感度曾有过这样的表述：“我明白了为什么中国的人口会这么多。”2008年北京奥运会以及其后的2010年广州亚运会上，颁装仪式上的服务员身穿的旗袍甚至暴露出内衣的线条，引起了一时的争议。

　▷继张曼玉之后，出现了旗袍的新强者。在9日中国国宾晚宴上，美中两国第一夫人都穿着旗袍登场。梅拉尼亚是袖子上带有毛皮的华丽刺绣旗袍，彭丽媛则是袖口看得出小臂的薄裙，展开了互不逊色的时装对抗。模特出身的梅拉尼亚身穿露到大腿中段的现代式旗袍，配以莫罗伯拉尼克高跟鞋，每一步都展现出曲线美。这身服装是意大利品牌古驰在2016年秋冬季的旗袍为基础推出的成衣。彭丽媛选择的则是开至膝盖、相对端庄的旗袍。

　▷两人的时装对抗中，当天最终的胜利者无疑是旗袍。中国方面宣称称，旗袍是具有端庄、优雅、知性美的女性外交服装。托美国第一夫人的福，中国没掏一分钱，却在全世界宣传了中国传统服装的风姿。时装不仅是一种文化，在产业领域也十分重要。世界级设计师们什么时候才会陷入韩服的美，设计出融韩国固有美学的服装呢？与旗袍的跃进相比，韩国服装的世界化还有很远的路要走。

# **习近平称“任何国家都不能成为孤岛”，暗指北韩**

 中国国家主席习近平18日在中国共产党第19次全国代表大会上作报告，阐述执政第二个任期的对内外政策。他表示，“任何国家都不能回到自我封闭的孤岛状态。”有分析认为，习近平虽然没有直接提及韩半岛和北韩，但针对的是自我孤立于国际社会、继续挑衅的北韩。  　习近平当天在北京人民大会堂举行的党代会开幕会上作工作报告，在公开对外政策时表示，“世界面临的不稳定性正在突显。地区争端问题不断发生。任何国家也无力独自应对人类所面临的各种挑战。”  　作为执政第二个任期的蓝图，他提出了两阶段的国家发展战略。第一个阶段是在到2020年全面建成小康社会的基础上，到2025年基本实现社会主义现代化；第二个阶段是从2035年起到21世纪中叶，把中国建设成为“富强、民主、文明、和谐、美丽的社会主义现代化强国”。他表示，“到2035年，将实现国防和军队的现代化，到21世纪中叶，将全面建设世界一流军队。”有分析认为，他对任期（2022年）以后的事情进行展望，是有长期执政的想法。习近平表示，“全党要服从党中央（习主席），集中统一到党的领导，”预告了权力集中。他把自己的思想称为“新时代中国特色社会主义”。

# **北京大学副院长：“中国应对北韩加大压力”**

“中国（在北韩问题上）没有责任，这种说法是不对的。”

　北京大学国际关系学院副院长王逸舟（照片）最近在接受《东亚日报》采访时表示：“中国政府有必要承认，我们（在北韩问题上）负有很大的责任，（在韩半岛）有着重要的利益。因此，只要不到引发战争的程度，中国应该加大对北韩的压力。”

　他表示，自己的这一看法，“和中国政府的立场不同，中国政府认为北韩核问题的主要责任在于美国和北韩，我们（中国）负有次要责任。”分析认为，王逸舟是在委婉地批评中国政府，因为中国政府认为自己不是北韩核问题的直接当事人，中国的作用有限，要求美国直接与北韩进行对话来解决。

　王逸舟与北京大学国际关系学院院长的贾庆国一样，是中国国内强调中国国际责任的自由主义学派的代表性人物。贾庆国9月份声称，“中国应承认韩半岛战争的可能性并与美国进行沟通，”与抨击这是“巅覆中 国在北韩核问题上外交原则的胡说八道”的主流学派公开发生意见冲突。王逸舟的表态再次显示，中国内部关于韩半岛政策路线的争议正在激化。

　王逸舟表示，“大部分支持”贾庆国的观点。他预测，“中国越是改革开放，赞成贾院长的声音会越来越多。”对于贾庆国言论引起的争议，他表示，“（中国）政府没有说谁对谁错，没有压制讨论。这是好事，比过去有了很多变化。”他还表示，“中国正在调整对北韩的政策。过去微弱的制裁强度正在越来越大，制裁的方向也在变得更为严厉。”

# **“中国的曼德拉”刘晓波**

据悉，中国代表性反体制人士刘晓波（62岁）被确诊肝癌晚期，上个月下旬获得保外就医，临终就在眼前。据说，中国政府允许一直以来阻止的家人探视，说明情况已十分严重。中国政府在二十国集团峰会前夕允许海外医疗人员接触刘晓波，但等到会谈一结束，就拒绝了让刘晓波到海外接受治疗的请求。虽然其理由是担心移送中出现安全问题，但实际上是担心此举给海外的反体制运动火上浇油。

　▷刘晓波曾主导1989年天安门事件时的学生运动，因反革命煽动罪被判有期徒刑3年；1995年因要求重新评价天安门事件，被判居家软禁9个月；第二年因主张与台湾和平统一，被判劳动教养3年；2008年因主导签名抨击共产党独裁的“08宪章”，被判有期徒刑11年，前后四次被软禁或判刑入狱。他在服刑中的2010年，作为首名中国人获得诺贝尔和平奖，在中国境外被称为“中国的曼德拉”。但是，在中国，没有人知道他是谁。中国的最大门户网站百度上，他被描述为接受美国团体不法资金的人。

　▷大部分反体制人士因为担心政府的镇压，选择了流亡海外，但刘晓波最终留在中国进行斗争。中国著名的天体物理学家方励之和患有视觉障碍的人权律师陈光诚也流亡或逃往美国。但是，刘晓波希望让他的夫人生活在自由的国度。据外媒报道，刘晓波希望到海外接受治疗，并不是为了自己，而是为了10年来处于居家软禁状态的夫人刘霞（55岁）。

　▷在中国，每年因社会不满而发生的集体示威事件超过18万起。在新疆维吾尔自治区，几乎每天都发生武装独立斗争。在人们不知情的情况下入狱关押的人不知其数。刘晓波平生呼吁的，是自由、人权、平等、民主和法制。中国连这样的人类普遍价值都不允许，离世界领导国家还有很远的路要走。

# **扛起反对特朗普贸易保护主义旗帜的韩中日，要将之扩大到G20**

韩国、中国和日本的经济首脑们昨天齐聚日本的横滨，举行了韩中日财长和央行行长会议，发表了名为《抵制所有形式的贸易保护主义》的共同宣言。韩中日3国表示：“我们一致认为，贸易是提高生产率、创造就业岗位等推动经济增长的最为重要的引擎”，决定进行更高水平的协作。在美国总统特朗普祭出贸易保护主义的大旗，追求本国利益优先的情况下，包括世界第二大和第三大经济体的东北亚三国的财长们发出一致的声音，令人瞩目。

在特朗普总统就任100天的时间里，各主要国家忙于看美国的脸色是事实。3月在德国举行的G20财长和央行行长会议上，由于美国的反对，共同宣言中没有写入过去3年都一直写入的“抵制贸易保护主义”的文句。一个月之前，国际货币基金组织总裁克里斯蒂·拉加德，世界银行总裁金庸，德国总理默克尔在德国就警惕贸易保护主义的扩散达成一致，但即便如此，在上个月举行的华盛顿G20财长和央行行长会议上，由于美国的反对，更加扩大了分歧。

拉加德总裁去年曾指出，“像第一次世界之前的贸易保护主义，将会带来灾难性的后果”，将会危及全球经济增长，对全球整合甚至对人也会带来危害。如她所指出的那样，由于贸易保护主义而获利的国家并不多。尤其像韩国这样靠贸易吃饭的国家，如果贸易保护主义的乌云不散，出口恢复的势头很可能遭到打击。中国国家主席习近平1月在瑞士达沃斯世界经济论坛上曾表示，“中国将成为自由贸易的守护者”，但之后表里行动不一，令人失望。抓住韩国部署萨德（THAAD，末端高空区域导弹防御系统）的小辫子，助长抵制韩货的运动，阻止游客访韩。力量逻辑第一，这是当今国际通商的现实。

尽管如此，韩中日此次共同发声，表示捍卫自由贸易，具有重大的意义。在没有国境的全球经济中，通商问题上升为和国家安全直接相关的问题。占世界经济和贸易量20%的韩中日3国如果能够合作，就可以在今后召开的G20财长会议上营造对抗特朗普贸易保护主义的氛围。韩中日共同宣言不应该停留在口头上，应该落实到实质性的政策协作中。自由贸易的精神是不可逆的世界贸易的潮流，应该努力创造契机，把这一事实扩散到G20中。
