# Supplementary material for: Bilingual translations of intensifiers in Dong-A Ilbo’s news about China: A corpus-based discourse analysis approach
Source: PLoS One. 2024 Feb 6;19(2):e0292603. doi: 10.1371/journal.pone.0292603 (PMC10846699; doi:10.1371/journal.pone.0292603)
Supplement: Attachment — Submitted filename: Response to Reviewers.docx [file pone.0292603.s002.docx]

1、In regards to matters concerning data and statistical analysis, this study is based on data compiled from all publicly available articles published on the website of Dong-A Ilbo. This study includes statistical analysis of intensifiers and their categories, collocation, frequency of variations within translated text, and statistical analysis of translation strategies.

To begin with, intensifiers were selected from the top 100 adverbs in the Sejong Corpus. The categorization of intensifiers was conducted strictly in accordance with the classification outlined by Quirk in "A Comprehensive Grammar of the English Language" from 1985. Marking the variations in intensifiers was determined rigorously based on whether corresponding intensifiers existed in the Korean-Chinese and Korean-English dictionaries of the Naver Dictionary (comprising a range of dictionaries including Oxford, Longman, and Goryeo Hanja). If there were no corresponding words, the classification was made based on Quirk's classification and the sentiment analysis data on adverbs carried out by Alqaryouti O, Siyam N, and Monem A in 2020. This determination identified whether the intensifiers in the translated text indicated an rise or down in intensity.

Furthermore, statistical analysis of translation strategies was conducted based on whether there were changes in intensifiers. If the corresponding words was present in dictionaries, it was categorized as a faithful translation; if not, it was categorized as a free translation. In cases of omission in translation, it was classified as a zero translation.

Additionally, the specific statistical analysis of intensifiers’ changes was conducted on the foundation of statistical data, with comprehensive explanations provided within the article. Throughout the statistical process, the author aimed to minimize subjective judgment and instead employed objective frameworks such as classic literature and dictionaries as standards for analysis.

2、Regarding the methodology, this study employed a 3x3 chi-square test to determine whether there were significant differences in the distribution of variations in the changes of intensifiers between English translations and Chinese translations. This was conducted to ascertain if distinct disparities existed. Furthermore, statistical data were employed to elucidate whether the variations in the translated text adhered to van Dijk's ideological square, thus delving into the systematic application of ideological frameworks in translation.
3、The author has already furnished comprehensive data and analysis processes; however, if further elaboration is required, the author could consider uploading the working corpus for this study.
4、In terms of the research methodology, this study employed a combination of corpus discourse analysis and van Dijk's ideological square. The application of corpus discourse analysis was evident in the establishment of the corpus, annotation and processing of linguistic data, statistical analysis, as well as the specific discourse analysis. The utilization of van Dijk's ideological square was manifested in both data analysis and corpus examination. The definitions of specialized terminology are available within the referenced literature, and any translation-specific terms not commonly known will be provided with definitions.
5、Concerning the translation subjects, the Dong-A Ilbo is chosen as the focal point in this study. This decision is rooted in the intention to investigate not the ideological stance of an individual translation editor, but rather the collective ideological perspective of a publishing media entity. In essence, the aim is to discern the shared ideological attributes among the editors of this publication.
6、The discourse on translation strategies represents a classic issue within the realm of translation studies, and its relevance extends beyond the confines of assignments, making it suitable for inclusion in academic journals. It is precisely by delving into translation strategies that we can uncover the underlying principles governing the essence of translation.
