# Supplementary material for: Bilingual translations of intensifiers in Dong-A Ilbo’s news about China: A corpus-based discourse analysis approach
Source: PLoS One. 2024 Feb 6;19(2):e0292603. doi: 10.1371/journal.pone.0292603 (PMC10846699; doi:10.1371/journal.pone.0292603)
Supplement: Attachment — Submitted filename: Response to reviwers.docx [file pone.0292603.s003.docx]

1、The calculation process for all statistics has been reexplained using the 3x3 chi-square equation to ensure transparency. The original text of my working corpus can also be found in the appendix.

2、The "selection of time frame" and "sample size rationale" in the corpus creation process have been reexplained.

3、Your review is greatly appreciated, and the issue of language consistency has been readdressed to ensure that proper nouns in the tables are consistent with the article.

4、It has been ensured that each abbreviation is accompanied by its full name when it first appears.

5、Regarding the issue in Example 6 that you mentioned, no changes have been made because the translation on the website is consistent with it. The image is attached below.

6、The decision to view the media as a cohesive entity rather than dissecting it into distinct roles of publishers, editors, and translators is rooted in the recognition of the complex and interconnected nature of news production and translation processes. Media organizations often operate as integrated systems where these roles collaborate closely to produce news content. This integrated approach acknowledges that translation decisions in news media are rarely made in isolation but rather result from dynamic interactions and negotiations among multiple stakeholders.By considering the media as a unified subject, our research aims to provide a more holistic perspective on translation strategies employed in news reporting. Understanding how translation choices are made within the broader context of media practices allows us to explore the multifaceted factors that influence these decisions. This approach enables a comprehensive examination of the interplay between ideology, policy, and media objectives in shaping translation strategies, which may not be fully captured by compartmentalizing the roles of publishers, editors, and translators.

Additionally, treating the media as a single entity simplifies the research process and analysis. Separating publishers, editors, and translators would require isolating their specific contributions, which can be challenging and may lead to oversimplification of the complex reality of news translation. Moreover, obtaining insights from these different roles might require access to internal processes and individuals within media organizations, which can be logistically challenging. I think future researchers can be inspired by this article to continue their research in this aspect.

7、I would like to extend my sincere gratitude to the reviewers for their valuable feedback and insightful comments on my manuscript. Your thoughtful reviews have significantly contributed to the improvement of this research.

I also want to express my apologies for not addressing some of the suggestions made in the previous review comprehensively. Your feedback is highly appreciated, and I have taken it to heart. I have now revisited the manuscript with great care, making the necessary revisions and enhancements to ensure its quality and clarity.

Your constructive input has been invaluable, and I am truly thankful for your dedication to the peer-review process. Your efforts have undoubtedly strengthened the overall quality of this work. Once again, I appreciate your time and expertise in reviewing my manuscript.
