# Supplementary material for: Bilingual translations of intensifiers in Dong-A Ilbo’s news about China: A corpus-based discourse analysis approach
Source: PLoS One. 2024 Feb 6;19(2):e0292603. doi: 10.1371/journal.pone.0292603 (PMC10846699; doi:10.1371/journal.pone.0292603)
Supplement: Attachment — Submitted filename: Response to Editors and Reviewers.docx [file pone.0292603.s004.docx]

**Response to editors：**

1. I have completed all the references that appear in the paper. In order to ensure the integrity and accuracy of the paper, I have taken the time to thoroughly search for and obtain the necessary cited references, and I have correctly cited them in the paper.
2. I have updated all the documents that have been withdrawn or cannot be found in electronic versions to the latest available versions. It was essential to ensure that the information presented in the paper is based on the most current and reliable sources.

The following is the literature that I deleted.

Putri, V. M And Hamzah. 2021 Type of Translation Shifts From Indonesian to English In Antara News. E-Journal Of English Language & Literature. Jell Vol. 10 No. 4. ISSN: 2302-3546(Electronic version not found, deleted)

The following is my updated literature.

Lefevere A. Translation, Rewriting and the Manipulation of Literary Fame. London, New York, Routledge Publ[J]. 1992. (Update to the latest version-Venuti, Lawrence. 1998. The Scandals of Translation. London and New York: Routledge.)

Van Dijk T A. Politics, ideology, and discourse[J]. 2006.(Update to the latest version-Van Dijk T A. Ideology and discourse[J]. The Oxford handbook of political ideologies, 2013: 175-196.)

Bolinger D. Degree words/By Dw[J]. Bolinger. The Hague. Paris. Mouton, 1972.(Update to the latest version-Bolinger D. Degree words[M]. Walter de Gruyter, 2013.)

3. Additionally, I have incorporated some recent and the missing literature in the article into the paper. This new literature provides valuable insights and enhances the overall quality of the paper.

Fernández Costales A. Exploring translation strategies in video game localization[J]. 2012.

Venuti L. Translation, history, narrative[J]. Meta, 2005, 50(3): 800-816.

Vandepitte S, Lefever E. Translation as a multilingual activity in the digital era[J]. Revue francaise de linguistique appliquee, 2018, 23(2): 59-72.

Chiaro D, De Bonis G. Multilingualism and translation on screen[J]. The Palgrave handbook of audiovisual translation and media accessibility, 2020: 687-711.

Prayitno D, Muchtar M, Mono U. Translation Shift of Google Translate in Translating Sports News on CNN[J]. 2021.

Lefevere A. Translation, rewriting, and the manipulation of literary fame[M]. Taylor & Francis, 2016.

Venuti L. The scandals of translation: Towards an ethics of difference[M]. Routledge, 2002.

Al-Hamadi H M. Investigating Ideologies in EM Forster’s A PASSAGE TO INDIA in Terms of Van Dijk’s Model: A Study in Literary Discourse[J]. Journal of Basra researches for Human Sciences, 2014, 39(1).

Ahlstrand J L. Strategies of ideological polarisation in the online news media: A social actor analysis of Megawati Soekarnoputri[J]. Discourse & Society, 2021, 32(1): 64-80.

Van Dijk T A. Discourse, ideology and context[J]. 2001.

Friginal E., Hardy JA. The Routledge handbook of corpus approaches to discourse analysis[M]. Routledge, 2020.

Laviosa S. Corpus-based Translation Studies: the theory[M]//Corpus-based Translation Studies. Brill, 2002: 23-31.

Olohan M. Introducing corpora in translation studies[M]. Routledge, 2004.

Hu K. Introducing corpus-based translation studies[M]. Berlin: Springer, 2016.

Munday J, Pinto S R, Blakesley J. Introducing translation studies: Theories and applications[M]. Routledge, 2022.

Jurko P. Semantic Prosody in Translation: Slovene and English ADV-V combinations[J]. ELOPE: English Language Overseas Perspectives and Enquiries, 2021, 18(1): 187-209

Volanschi A, Kübler N. Semantic prosody and specialised translation, or how a lexico-grammatical theory of language can help with specialised translation[C]//25th European Systemic Functional Linguistics Conference:“Change, Mutation, Transformation”. 2014.

Peters H. Degree adverbs in early modern English[J]. Studies in early modern English, 1994, 13: 269-288.

Partington A. Corpus evidence of language change[J]. Text and technology. In honour of John Sinclair, 1993: 177-192.

Quirk R, Greenbaum S, Leech G, et al. A COMPREHENSIVE GRAMMAR OF THE ENGLISH LANGUAGE[J]. 1987.

Biber D, Johansson S, Leech G N, et al. Grammar of spoken and written English[J]. (No Title), 1999.

Bolinger D. Degree words[M]. Walter de Gruyter, 2013.

Quirk R, Greenbaum S, Leech G N, et al. A grammar of contemporary English[J]. 1972.

Alqaryouti O, Siyam N, Abdel Monem A, et al. Aspect-based sentiment analysis using smart government review data[J]. Applied Computing and Informatics, 2020.

Straubhaar J, LaRose R, Davenport L. Media now: Understanding media, culture, and technology[M]. Cengage Learning, 2015.

4. For the sake of clarity in citing references, I have added the abbreviations of the authors' surnames at the relevant places in the text, making it easier for readers to identify and locate the sources.
5. I reference and provide explanations for Table 4 and Table 5 within the main text.

**Response to reviewers:**

I have incorporated explanations regarding the translator's definition and potential misunderstandings that could arise in Example 6.
